# A plant‐centric view of class B flavin‐dependent monooxygenase evolution and diversity

**DOI:** 10.1111/tpj.71106

**Published:** 2026-09-09

**Authors:** Joachim Møller Christensen, Elizabeth H. J. Neilson

**Affiliations:** ^1^ Plant Biochemistry Section, Department of Plant and Environmental Sciences University of Copenhagen 1871 Frederiksberg C Denmark

**Keywords:** evolution, FMO, flavin‐containing monooxygenase, phylogenetic analysis, YUCCA

## Abstract

Flavin‐dependent monooxygenases (FMOs) are ancient enzymes present throughout all kingdoms of life. FMOs utilize flavin‐based cofactors to incorporate an oxygen atom into their substrate, altering its chemical properties. Class B FMOs are enriched throughout the plant kingdom, catalyzing essential reactions for plant development and defense, including auxin biosynthesis and systemic acquired resistance. Despite these essential metabolic roles, class B FMO functional characterization remains relatively limited, with no common evolutionary framework for FMO diversity across the plant kingdom currently available. By mining genomes representing 78 major Viridiplantae lineages, we present a curated dataset and comprehensive plant‐centric phylogenetic analysis of class B FMOs, classifying eight distinct families: BVMO, N‐Ox, N‐Ox like 1, SeedlessFMO, S‐Ox, S‐Ox like 1, S‐Ox like 2, and YUCCA. Most families evidently originate from an algal progenitor, although disparate degrees of FMO prevalence within and across families suggest multiple acquisition events in plants via horizontal gene transfer. Structural modeling and domain architecture analysis provide a refined framework for class B FMO diversity in plants delivering new insights into protein diversity and evolution. This plant‐centric focus on class B FMOs provides an important resource that will facilitate further biochemical and functional characterization within plant development and response to environmental change.

## Significance Statement

Class B flavin‐dependent monooxygenases (FMOs) are critically important enzymes for plant development, defense, and signaling. This study provides a comprehensive phylogenetic and structural analysis of FMOs across the plant lineage, establishing a framework to guide further characterization of their role in plant metabolism.

## INTRODUCTION

**Figure 1:**
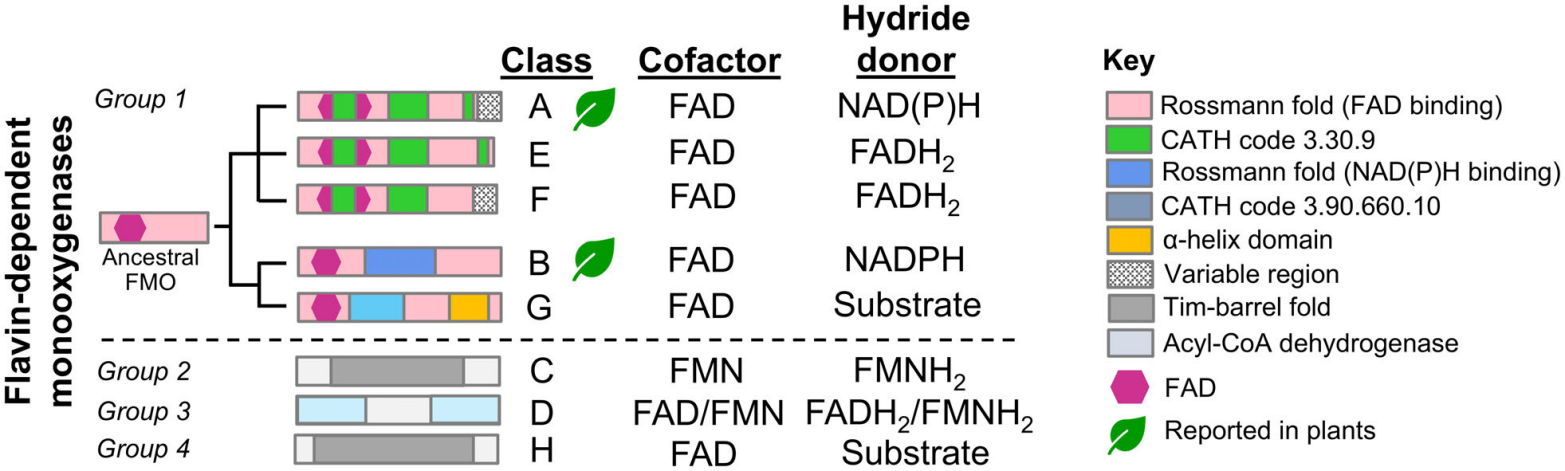
Schematic overview of flavin‐dependent monooxygenase (FMO) classes with different flavin co‐factors and hydride donors. FMOs are separated into eight classes, where classes A, B, E, F, and G share a common evolutionary ancestor and FAD‐binding structural similarities. Plants possess FMOs belonging to classes A and B. Figure adapted from Mascotti et al. (2016).

Flavin‐dependent monooxygenases (FMOs) are ancient enzymes widespread through different kingdoms of life. FMOs utilize flavin‐based coenzymes to incorporate an oxygen atom into their substrate altering its chemical properties such as bioactivity and solubility and are therefore highly valuable proteins for different applications including xenobiotic detoxification (Bailleul et al., 2023) and as industrial biocatalysts (Paul et al., 2021). FMOs have been classified into eight different classes (A–H) based on structural and functional characteristics (Huijbers et al., 2014; Mascotti et al., 2016) (Figure 1). Classes A, B, E, F, and G are proposed to share a common evolutionary ancestor, with evolutionary divergence resulting from recruitment of different domains (Mascotti et al., 2016). Some classes (A, B, and G) function as single‐component enzymes, while Classes C–F require a reduction partner for activity (Mascotti et al., 2016).

Among the plant kingdom, class B FMOs are the most prevalent, abundant, and diverse, present in all plant species, and in high copy numbers (Thodberg & Neilson, 2020). In fact, outside class B, only a single FMO has been characterized: a class A FMO involved in nitrogenous volatile compound biosynthesis in tomato (*Solanum lycopersicum*; Liscombe et al., 2022). Class B FMOs possess a distinct and characteristic domain architecture, characterized by two dinucleotide‐binding domains. This structure arises from a duplication and subsequent functionalization of the FAD‐binding domain into an NADPH‐binding domain, which results in the FAD‐binding domain being split into two noncontiguous segments (Figure 1; Mascotti et al., 2016). Correspondingly, class B FMOs possess two conserved Rossmann fold motifs important for the binding of the two di‐nucleotides: FAD (GxGxxG) and NADPH (GxGxxG/A). Class Bs also possess a so‐called ‘FMO‐identifying’ motif (FxGxxxHxxxY/W), where the tryptophan is unique to a family of class B FMOs known as Baeyer–Villiger monooxygenases (BVMOs) that catalyze the insertion of an oxygen atom in a C–C bond (Fraaije et al., 2002). A fourth, less conserved motif common to class B FMOs is the ‘FATGY’ motif but can also be referred to as the ‘ATG‐containing’ or ‘TGY’ motif (Stehr et al., 1998).

Functional characterization of plant class B FMOs (herein referred to as FMOs) is relatively limited, and predominantly described within model plant species and crops. The best known characterized FMOs include the highly conserved YUCCAs, which perform an oxidative carboxylation of indole‐pyruvic acid (IPA) to form the essential hormone auxin (indole acetic acid; IAA; Dai et al., 2013; Mashiguchi et al., 2011; Zhao et al., 2001), and FMO1 that performs an *N‐*hydroxylation of pipecolic acid to form the mobile immune signal, *N‐*hydroxy pipecolic acid (Hartmann et al., 2018; Holmes et al., 2019). Beyond these well‐known FMOs, other characterized members are generally found to perform C‐, N‐, and S‐oxidizing reactions within plant specialized metabolism (Christensen et al., 2026; Florean et al., 2023, 2025; Holmes et al., 2019; Jiang et al., 2025; Kong et al., 2016; Thodberg et al., 2020; Valentino et al., 2020; Won et al., 2011). Given the diverse and essential catalytic roles these characterized FMOs possess, it is likely that other FMOs carry out key roles in general and specialized metabolism important for plant performance and survival. Further functional analysis of plant FMOs would be greatly aided by a detailed phylogenetic analysis to determine their evolutionary nature and diversity. Mapping the evolutionary landscape of FMOs will aid in placing newly identified members into clades of shared homology and can provide clues of shared catalytic functionality. Historically, an ad hoc naming system of different FMOs has been employed and may offer some confusion. For example, FMO1 from *Arabidopsis thaliana* and garlic (*Allium sativum*) are evolutionary distinct and catalyze respective *N*‐ and *S*‐hydroxylation reactions. Furthermore, these plant FMO1s are evolutionary distant from the similarly named mammalian FMO1. At present, phylogenetic analysis of plant FMOs contains a small number of plant species, with a bias toward model angiosperms (Carrillo‐Carrasco et al., 2023; Thodberg & Neilson, 2020), have focused on individual FMO families (Jiang et al., 2025; Vijayanathan et al., 2025) or have performed detailed analysis within a restricted taxonomic group (Gaba et al., 2023; Sun & Bakkeren, 2024).

The most comprehensive phylogenetic analysis of class B FMOs includes a diverse array of FMOs representing different kingdoms of life (Nicoll & Mascotti, 2023). Six major clades were identified, four of which contained FMOs from plants: Clade II that contains the plant S‐Ox FMO family; Clade III contains the YUCCAs; and Clade IV contains the plant N‐Ox family and the BVMOs (historical name preserved). Although the phylogenetic analysis conducted by Nicoll and Mascotti (2023) provides valuable insights into the evolutionary history of class B FMOs across life, the plant lineage is only represented by three plant species: a eudicot (*A. thaliana*), monocot (*Oryza sativa*), and bryophyte (*Physcomitrium patens*). A comprehensive, plant‐centric phylogenetic investigation encompassing a broad range of species is necessary to uncover lineage‐specific adaptations and functional diversification within this enzyme family in the plant kingdom. This study therefore presents a robust, curated dataset and phylogenetic analysis of class B FMOs in plants, providing a nuanced perspective on their evolutionary history and functional specialization. Furthermore, this study aimed to map the structural diversity and placement of FMO‐specific motifs. By refining the definition and structural context of these conserved motifs, this study provides a valuable framework and resource to accurately identify and compare class B FMOs. Understanding the evolutionary and structural diversification of class B FMOs in plants is crucial for elucidating their roles in specialized metabolic pathways, where monooxygenases frequently catalyze key regio‐ and stereo‐specific oxidation reactions. As central enzymes in hormone biosynthesis, defense compound production, and specialized metabolism, class B FMOs represent an underexplored reservoir of enzymatic functions with potential applications in both plant biology and synthetic biochemistry.

## RESULTS

### A wide net catches many: navigating the phylogenetic BLAST analysis for class B FMOs

**Figure 2:**
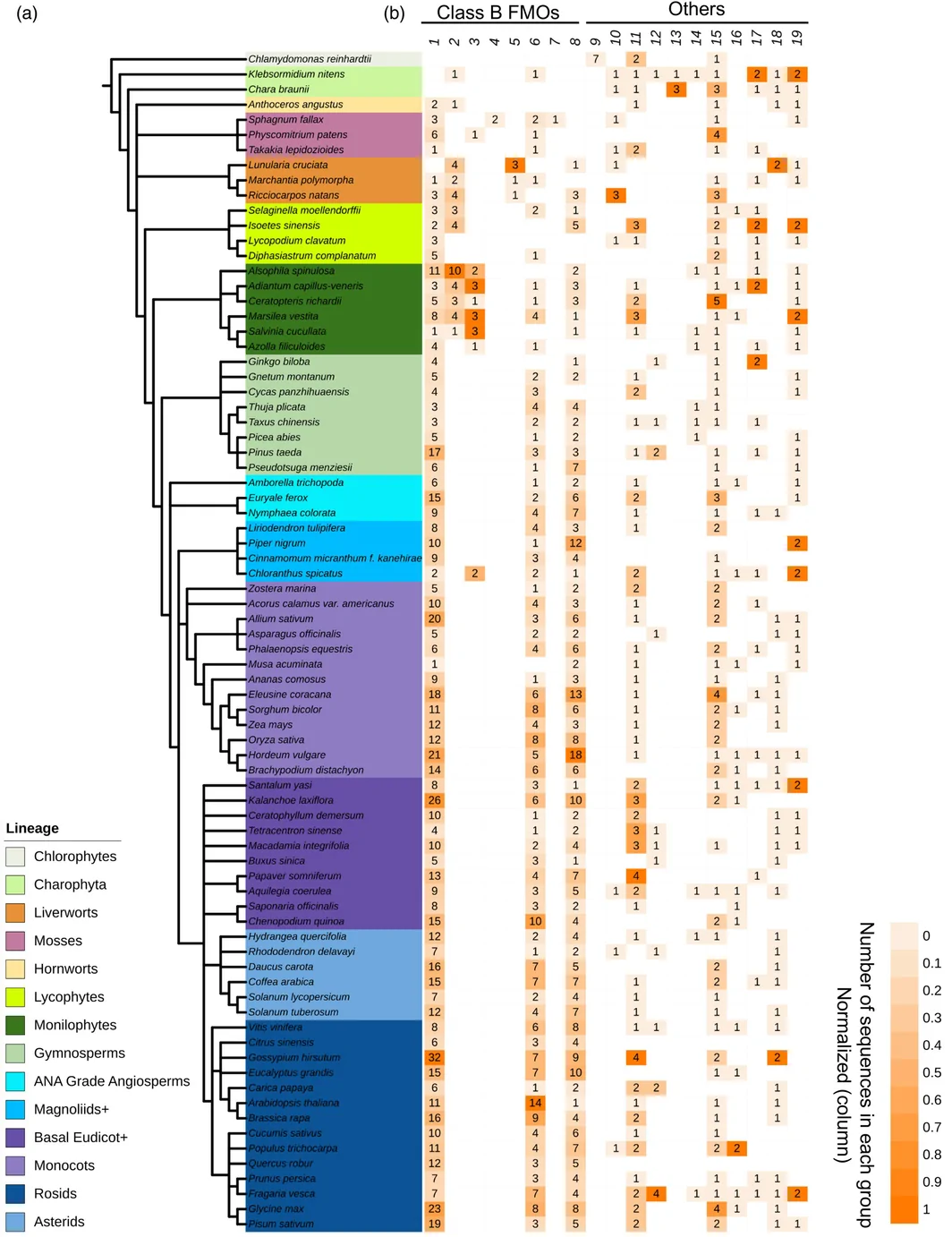
Overview of species and sequence distribution curated in this study. (a) The taxonomic tree displays the evolutionary relationships among the 78 species genomes included in this study, representing a broad diversity within the plant kingdom. (b) Heatmap illustrates the distribution of identified and curated sequences following a low cutoff BLAST analysis of class B flavin‐dependent monooxygenases (FMOs) from each species. Heatmap columns represent 19 different phylogenetic groups, with corresponding group names annotated in Figure 3. Values are normalized per column to account for differences in total sequence counts. Taxonomic overview and sequence distribution can be accessed at: https://itol.embl.de/shared/PlantEcoPhys.

**Figure 3:**
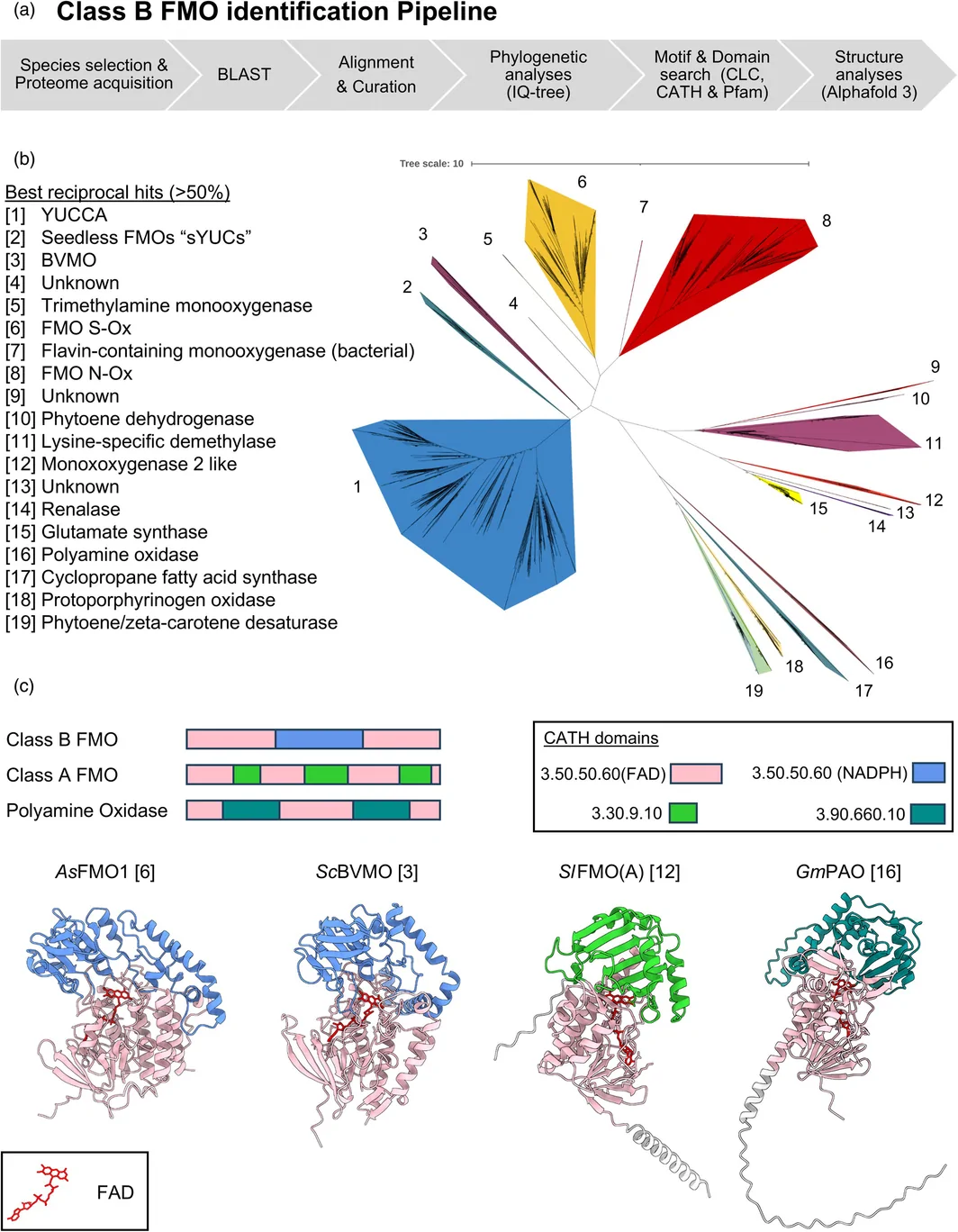
Methodological process for class B flavin‐dependent monooxygenases (FMOs) identification in plants. (a) Pipeline for phylogenetic and structural analysis. The workflow illustrates the process from species selection to sequence analysis, including BLAST searches, multiple sequence alignment (MAFFT), phylogenetic inference (IQ‐TREE with ModelFinder [JTT+R10]), a motif search and structural analysis of domain architecture. (b) Initial phylogenetic tree of sequences acquired by BLAST with class B FMO queries, which was used to stratify returned sequences into 19 different evolutionary groups for further analyses. The tree was constructed using maximum‐likelihood inference in IQ‐TREE based on a MAFFT alignment. Best reciprocal BLAST hits from NCBI indicate potential enzyme functions. Phylogenetic tree (including all bootstrap values) is available at https://itol.embl.de/shared/PlantEcoPhys. (c) Structural validation of class B FMO domain architecture. AlphaFold 3 models of four representative enzymes are shown with FAD as a ligand (red), each belonging to distinct phylogenetic clades (shown in squared brackets). CATH domains are color‐coded: the first Rossmann fold in pink and the second in cornflower blue. CATH domain distinctive of class A FMOs (3.30.9.10) is colored in green while 3.90.660.10 from PAOs is colored in dark cyan.

To ensure a comprehensive and balanced phylogenetic analysis of FMOs across embryophyte, the Lifemap server (De Vienne, 2016) was used to select a broad representation of plant species across the plant kingdom (Figure S1). A total of 78 green algae and plant species were selected, spanning Chlorophyta, Charophyta, Liverworts, Mosses, Hornworts, Lycophytes, Monilophytes (ferns), Gymnosperms, and a diverse range of Angiosperms (Figure 2a). To specifically identify class B FMOs across the plant kingdom, a dedicated pipeline was designed to capture FMO diversity, including potentially undescribed FMO families (Figure 3a). Representative FMOs from the canonical YUCCA, BVMO, S‐Ox, and N‐Ox families were used as BLASTP queries against collected genomes (Tables S1 and S2), resulting in an initial dataset of 2339 sequences. A low E‐value cutoff was deliberately assigned (i.e., *E*‐value <10^−3^) to ensure a wide capture of FMO diversity, and potentially the capture of distantly related protein classes. Publicly available genomic resources frequently contain incorrectly predicted gene models (Thomson et al., 2022), so rigorous manual curation was performed to identify and remove misannotated exons and introns, frameshifts, insertions, and deletions. The full dataset was too divergent to reliably curate, so the dataset was subdivided into 16 smaller, less diverse alignments (Figure S2A). Each subset was then manually inspected and curated (Figure S2B), ultimately resulting in a dataset of 1642 high‐confidence sequences and a robust alignment (Data S1 and S2). To better classify and identify the output of this alignment, a maximum‐likelihood (ML) phylogenetic analysis was then performed on the curated dataset, resulting in ‘navigational tree’ and the identification of 19 distinct phylogenetic groups (Figure 3b). The identity of each group was then assigned by first delineating each group based on tree topology, and then annotated according to best reciprocal BLAST hit (≥50% identity). The output resulted in a mixture of characterized enzyme families (e.g., YUCCAs and glutamate synthases), putative protein homologs (e.g., ‘monooxygenase 2‐like proteins’), and several uncharacterized sequences (Figure 3b). Several putatively identified non‐FMO enzyme hits are known to contain FAD and are classified as flavoproteins, including lysine‐specific demethylase, glutamate synthase, and protoporphyrinogen oxidase (Brzezowski et al., 2019; Perillo et al., 2020; Van Den Heuvel et al., 2004).

An examination of the resultant navigational phylogenetic tree revealed several emerging patterns. First, the three well‐characterized plant class B FMO families (i.e., YUCCAs, N‐Ox, and S‐Ox) showed broad representation across most plant lineages (Figures 2b and 3b). YUCCAs were identified in all plant species analyzed (note: no green algae), while N‐Ox and S‐Ox FMOs were generally present in vascular plants and sporadically found in nonvascular species. Within these families, angiosperms displayed an expansion in sequence number, suggesting lineage‐specific diversification. Beyond these canonical FMO families, several clades with the best reciprocal BLAST hits to non–class B FMO enzymes also exhibited broad phylogenetic distribution. Notably, Clades 11 and 15, corresponding to lysine‐specific demethylases and glutamate synthases, respectively, were represented across nearly all major plant lineages; from chlorophytes to angiosperms (Figure 2b). In contrast, other groups such as Clade 12 (monooxygenase 2‐like) and Clade 14 (renalase) showed more limited representation, appearing only in a restricted subset of taxa.

To assess whether any of the retrieved sequences represented novel class B FMO families, an unbiased analysis of all 19 phylogenetic groups was conducted to assess the prevalence of canonical class B FMO motifs. Sequences were selected for further investigation based on the presence of at least two Rossmann fold motifs (GxGxxG/A) and a conserved FMO‐identifying motif, characterized by a highly conserved histidine residue. Structural analysis of AlphaFold 3 (Abramson et al., 2024) predicted models for representative sequences revealed that eight phylogenetic groups exhibited the distinctive class B FMO architecture: two FAD/NAD(P)‐binding domains (CATH 3.50.50.60), with the NADPH‐binding domain splitting the FAD‐binding domain (Figure 2c). Interestingly, phylogenetic groups 12 and 16 (Figure 2b) also possess the FAD‐binding CATH domain 3.50.50.60 and share sequence similarities with class B FMOs, possessing two Rossmann fold motifs (GxGxxG/A) and an FMO‐identifying motif (FXGXXXHXXXY/F). Structural comparison using the Foldseek server (Van Kempen et al., 2024), however, classified representatives from these groups—*Sl*FMO(A) (Group 12; XP_069144694.1) and *Gm*PAO (Group 16; Glyma.15G276500)—as a class A FMO and a polyamine oxidase (PAO), respectively. These findings were further validated through domain architecture analysis using the CATH server (Greene et al., 2007; Figure 3b). *Sl*FMO(A) from tomato was identified as a class A FMO and appeared in the initial phylogenetic analysis within Clade 12, which had monooxygenase 2‐like proteins as its best reciprocal BLAST hit (Figure 3b). This family is not broadly represented across the plant lineages used in this study, with 18 sequences retrieved from Charophyta and 11 taxonomically diverse seed plant species (Figure 2b).

### Plant‐centric phylogenetic analysis of class B FMOs

**Figure 4:**
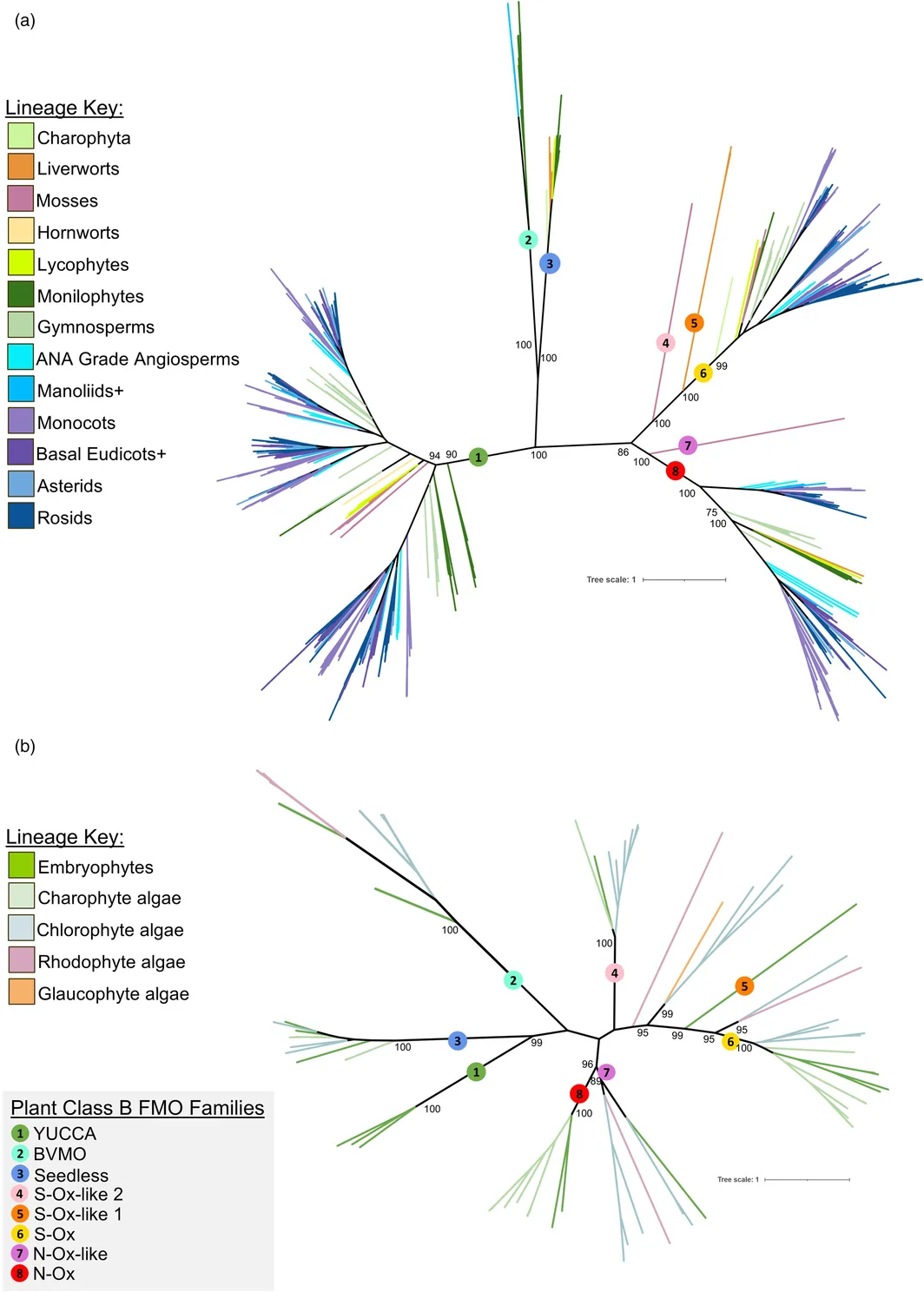
Plant‐centric evolutionary view on class B flavin‐dependent monooxygenases (FMOs). (a) Unrooted maximum‐likelihood (ML) tree of 1273 class B FMOs curated from 78 Viridiplantae genomes identifying eight major families (highlighted by colored dots). (b) Evolutionary relationship of plant class B FMOs incorporating greater representation of algal species. The unrooted ML tree of was generated by combining using Embryophyte FMOs baits representing the eight identified plant FMO families. All top BLAST hits in the Phycocosm database were included, except for S‐Ox representatives where sequence number was limited to the top 10 hits (i.e., 74 sequences in total). No close algal hits were identified for the YUCCAs or S‐Ox‐like 1 families. Phylogenetic relationships were inferred using ML analysis of MAFFT v7–aligned sequences in IQ‐TREE applying the JTT+F+R10 substitution model for the embryophyte‐focused tree, and the Q.pfam+F+R6 substitution model for the algal tree. Branch support was assessed with 1000 ultrafast bootstrap replicates (UFBoot2). Bootstrap values (>75) for major branches are presented. Online version of the tree is available at: https://itol.embl.de/shared/PlantEcoPhys.

We used a navigational phylogenetic output and structural comparisons to identify and assign class B FMOs in plants. Based on the output of this methodological pipeline, a second phylogenetic analysis was performed using only the determined plant class B FMO members (Figure 4; Data S3 and S4). This analysis shows that plant FMOs are polyphyletic, with eight distinct phylogenetic clades identified, each supported by bootstrap values of 100. As expected, the large canonical YUCCA, N‐Ox, and S‐Ox families form major branches in the tree. The other well‐recognized BVMO family, previously represented by only a single characterized member from the moss *P. patens* (Beneventi et al., 2013), was expanded to include new BVMO members from the angiosperm species *Chloranthus spicatus*, and 13 monilophyte members (Figure 2b). In addition, based on their evolutionary position, divergence from these aforementioned families (<35% amino acid similarity; Table S3), four additional FMO families are classified, each with relatively narrow representation across the plant kingdom (Figure 2b). One clade was previously suggested to be a sister group to the YUCCA family (Carrillo‐Carrasco et al., 2023). This family consists of FMO members from non‐seed plants (algae, liverworts, hornworts, lycophytes, and monilophytes) (Figure 2b), so we therefore suggest naming this family ‘Seedless FMOs’ [3]. Of the three remaining novel phylogenetic FMO families, two consist exclusively of members from the moss *Sphagnum fallax* (S‐Ox‐like2 [4] and N‐Ox‐like [7]), and the third family only consists of members from liverwort species *Marchantia polymorpha*, *Ricciocarpos natans*, and *Lunularia cruciata* (S‐Ox‐like 1 [5]). No additional FMOs were captured when the pipeline was rerun with the newly identified groups.

Of the three chlorophyte and charaphyte species selected and curated, class B FMOs were only detected in the S‐Ox and seedless FMO families, limiting the resolution of each family's origin and general prevalence across the Streptophyte lineage. To provide a deeper assessment of each family's evolutionary origin and position, we performed two additional analyses. First, a BLASTP search was performed in the PhycoCosm algal genomics resource (Grigoriev et al., 2021a) using selected members from the eight plant class B FMO families. The resultant best hits for each queried family were collected, aligned, and phylogenetically analyzed (Figure 4b; Data S5 and S6), with a limit of 10 best hits set for S‐Ox FMO members. Second, we collated the identified plant FMO families together with the cross‐kingdom class B FMO phylogeny previously published by Nicoll and Mascotti (2023), consisting of 234 class B FMOs from diverse species including metazoan, fungi, haptophytes, bacteria, archaea, and algae (Figure S3).

These analyses support the proposed classification and paraphyletic nature of the different class B FMO families in plants. Algal FMO representatives position at the base of six plant FMO families (BVMO, N‐Ox, N‐Ox‐like, Seedless, S‐Ox, S‐Ox‐like 2), with no close algal hits identified in PhycoCosm for the YUCCAs or S‐Ox‐like 1 families. The N‐Ox, N‐Ox‐like, S‐Ox, S‐Ox‐like 2, and Seedless FMO families are represented in Streptophyte algae. N‐Ox FMOs are present in charophyte species, while S‐Ox and S‐Ox‐like 2 FMOs are present in both charophytes and chlorophytes. The N‐Ox‐like family contains homologous chlorophyte, rhodophyte, and haptophyte FMO members, while the Seedless FMO family contains members from charophyte, chlorophyte, and haptophyte algae, with rhodophyte algae members reported in the literature (Carrillo‐Carrasco et al., 2023). The evolutionary positioning of the N‐Ox and N‐Ox‐like families, and apparent lack of FMOs from other kingdoms (Figure S3), suggests these families are a plant‐specific innovation arising following the Archaeplastid endosymbiosis event. Seedless FMOs BVMOs are present also chlorophyte and rhodophyte algae, but evolutionary positioning suggests that BVMO presence in Embrophyta follows multiple acquisition events. A single chlorophyte BVMO is positioned next to fern BVMOs (Figure S3), while the *P. patens* and *C. spicatus* BVMOs lie in a clade together with BVMO members from an archaea, fungi, rhodophyte, and hydrozoa species (Figure S3).

### Sequence diversity across key plant class B FMO motifs

**Figure 5:**
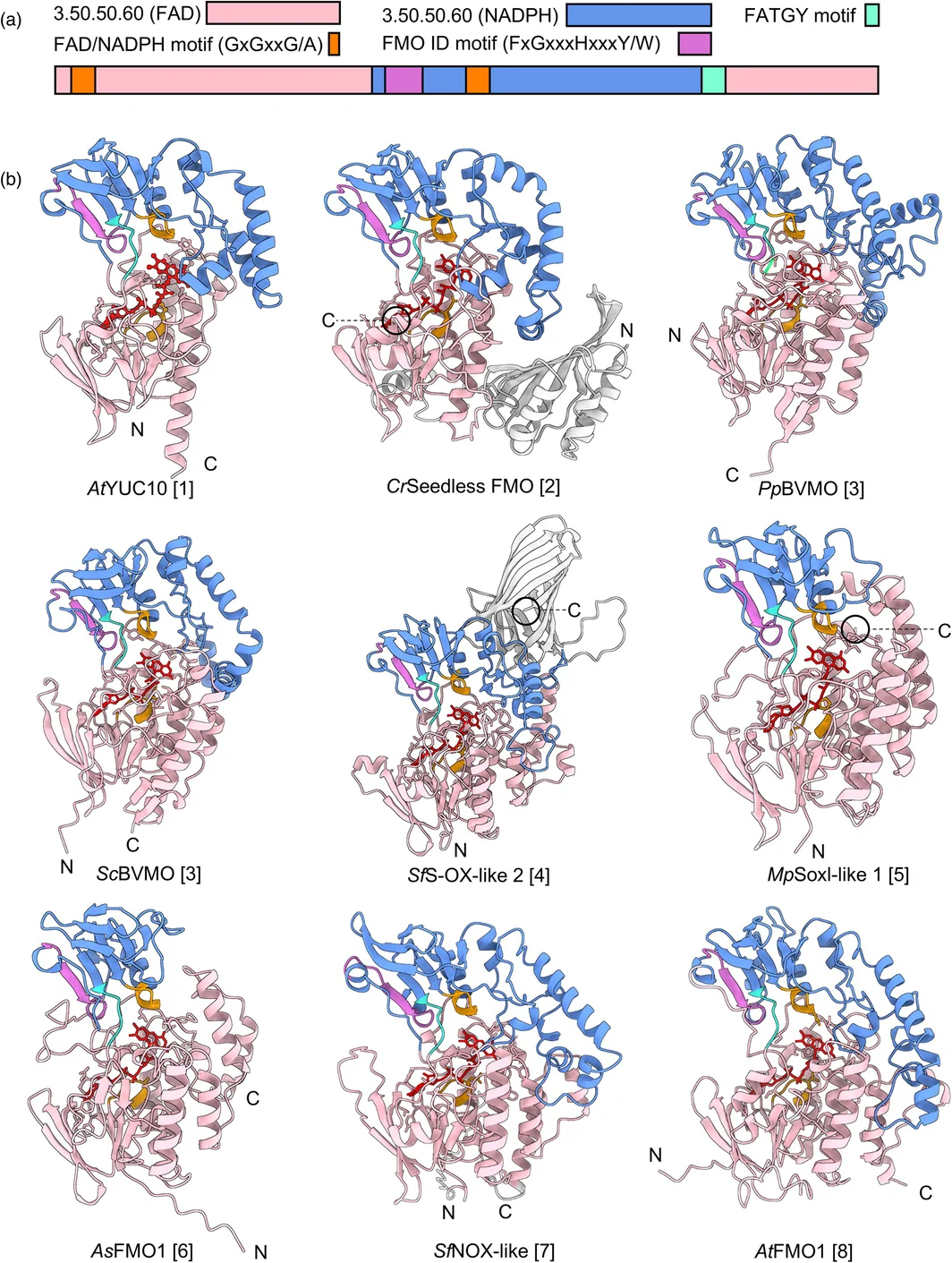
Structures of identified class B flavin‐dependent monooxygenases (FMOs). (a) Class B FMO schematic of characteristic dinucleotide domain architecture and canonical motifs/folds. (b) AlphaFold 3 predicted structures of representatives from eight identified phylogenetic families of class B FMOs. The FAD‐ and NADPH‐binding domains are colored in pink and cornflower blue, respectively. Structural folds belonging to canonical class B FMO motifs are colored in orange (GxGxxG/A), purple (FMO ID motif; FxGxxxHxxxY/W), and aquamarine (FATGY). FAD molecule is colored red.

To investigate the structural diversity within class B FMOs, eight representative enzymes were selected from each family. Structural models were predicted using AlphaFold 3 with FAD as a ligand (Figure 5). The plant class B FMOs all adopt a two‐domain architecture, comprising both FAD‐binding and NADPH‐binding domains (Figure 5a), and the four canonical FMO motifs—FAD‐binding (GxGxxG), NADPH‐binding (GxGxxG/A), FMO‐identifying (FxGxxxHxxxY/W), and F/LATGY (or ATG)—were generally conserved across the different plant FMO families, although some family‐specific substitutions were observed (Table 1; elaborated below). The most pronounced structural differences between the plant FMO families were observed in the NADPH‐binding domain, particularly in the region between the NADPH‐binding motif and the FATGY motif, where size and structural embellishments varied significantly. Notably, despite these variations, the FATGY motif was consistently positioned in the same overall location within the protein, effectively facilitating the transition from the NADPH‐binding domain to the second part of the FAD‐binding domain. S‐Ox and S‐Ox‐like 1 representatives exhibited the simplest NADPH‐binding domain, whereas other groups displayed additional α‐helical insertions. Notably, the Seedless FMO family possesses a conserved N‐terminal domain in addition to the distinct class B FMO domain architecture, denoted as a catalytically inactive SnoaL‐like fold (Chánique et al., 2023). Additionally, both S‐Ox‐like 2 FMOs from *S. fallax* are distinguished by a C‐terminal β‐barrel (Figure 5b).

**Table 1:**
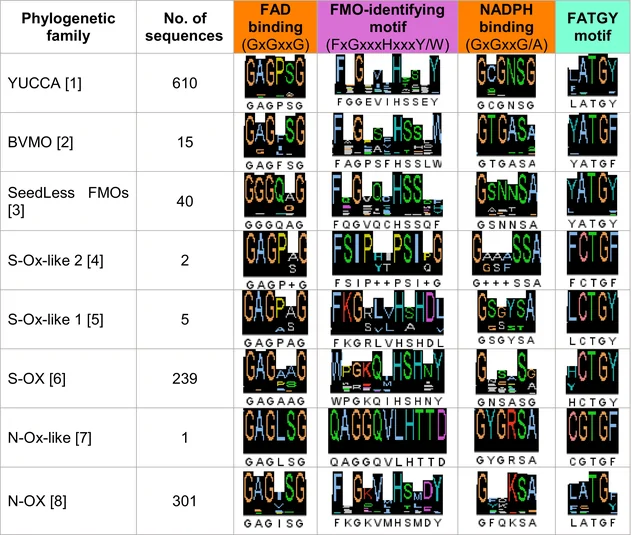
Evaluation of the conserved class B FMO motifs represented by logos of different plant families.

Alignment and full conservation logos for each phylogenetic family can be found in Figures S6–S13. Numbers in square brackets refer to the families assigned in Figure 4.

To further compare the structural relationships between different plant FMO families and systematically assign canonical motifs across all phylogenetic clades, *At*YUC10 was structurally aligned to the other representative FMO families. The structural folds corresponding to the conserved FMO motifs were well‐preserved across all analyzed FMOs, even when sequence conservation was not immediately evident (i.e., the NADPH‐binding GxxxxxG/A motif from AtFMO1; N‐Ox family; Figure S4; Table 1). Accordingly, these conserved folds were used to compare sequence‐level divergence and motif variation. While the FAD‐binding motif was consistently conserved across all families, the NADPH‐binding motif displayed greater variability. Here, YUCCAs and BVMOs possess the central glycine, while the other families have a conserved serine residue, and a NADPH‐binding motif of GxxxSG/A (Table 1). The FMO‐identifying motif (FxGxxxHxxxY) also exhibits family‐specific substitutions: a tryptophan (W) replaces phenylalanine (F) in the S‐Ox family (WxGxxxHxxxY), and the S‐Ox‐like 2 and N‐Ox‐like families lack the central histidine. The FATGY motif, considered less conserved, showed a more consistent pattern with a conserved (A/C)TG core: ATG in YUCCAs, BVMOs, FMO N‐Ox and Seedless FMOs, and CTG in S‐Ox and S‐Ox‐like families (Table 1). Further family‐specific motifs were assessed by comparing each major family (i.e. families with >15 sequences; Data S7) against all others using STREME (Bailey, 2021). Unique motifs were identified for the BVMO, N‐Ox, Seedless, S‐Ox, and YUCCA families, with several highly conserved residues (Data S8–S12). By mapping motifs with >95% conservation within a family to representative alpha fold predicted models (i.e., models from Figure 5), we identified two family‐specific motifs that may interact with FAD (Figure S5). For the S‐Ox family, we identified a FPFLDT motif (Data S11) where the conserved central phenylalanine lies in the FAD‐binding cavity, with a 3.83 Å distance to the exocyclic amino nitrogen of the adenine ring (Figure S5A). Similarly, the GDSWRNRY motif in Seedless FMOs (Data S10) contains conserved tryptophan and arginine residues that are predicted to form hydrogen bonds with the diphosphate bridge of FAD, and a conserved glycine of the FAD‐binding motif, respectively (Figure S5B).

Structural alignments were also used to specifically investigate the hypothesis that Seedless FMOs may be sister to the YUCCA family. Structural comparisons of the Seedless FMO from *Ceratopteris richardii* (Ceric.34g041100) suggest it is most similar to *At*YUC10 (YUCCA) and *At*FMO1 (FMO N‐Ox), with RMSD values of 5.3 and 6.8, respectively, while showing lower similarity to the BVMO representative (*Pp*BVMO; RMSD = 9.3) (Table S4). These values suggest that while the Seedless FMO shares general FMO fold characteristics, the family does not exhibit greater structural similarity to YUCCAs compared with other class B FMOs, supporting its position as a distinct family.

## DISCUSSION

Here, we present a comprehensive phylogenetic and structural analysis of class B FMOs across the plant kingdom for the first time. To identify and place these enzymes in a broader evolutionary context, we used a methodological pipeline that not only identified new FMO families but also examined class B FMO diversity alongside other plant flavin‐dependent proteins with shared partial sequence similarity. Rigorous phylogenetic analysis is dependent on high‐quality sequence input (Thomson et al., 2022; Vaattovaara et al., 2019). The sequence quality from publicly available genomic resources is challenged by automated annotation pipelines, where misannotated exons, introns, insertions, and deletions frequently occurs (Chen et al., 2026; Tello‐Ruiz et al., 2019; Vaattovaara et al., 2019). The curated FMO datasets and alignments presented here provide a robust framework for further analyses, such as profile HMM analysis and ancestral sequence reconstruction (Bailleul et al., 2021; Thomson et al., 2022).

A large‐scale BLAST search using characterized plant FMO sequences returned a wide range of flavin‐related proteins, many of which shared conserved regions corresponding to the FAD‐binding domain. For example, our search identified PAOs and class A FMOs (Figure 3c) that possess two Rossmann folds and an FMO‐like identifying motif. These protein families share approximately 30–50% sequence identity across 30–60 aligned amino acids relative to class B FMO queries and represent a deep evolutionary relationship with class B FMOs. Class A FMOs are specifically hypothesized to derive from an ancestral FMO shared with class B FMOs (Mascotti et al., 2016), which was followed by the recruitment, duplication, or insertion of additional domains to form the different classes. On the other hand, PAOs share the monoamine oxidase (MAO) 3.90.660.10 CATH domain with class G FMOs (i.e., the class most structurally similar to class B FMOs; Figure 1). In the same way that class B and class G FMOs derive from an ancestral flavo protein, class G and PAO proteins are also reported to share a common ancestor (Mascotti et al., 2016; Salvi & Tavladoraki, 2020). Our search for class B FMOs also identified the glutamate synthase family in the initial sequence acquisition. This family was captured due to common features in the FAD‐binding region, with approximately 40–50% sequence identity over alignment lengths of 40–70 amino acids. In contrast to the class A and PAO families, the glutamate synthase family hits reflect general sequence conservation involved in FAD binding rather than evidence of a shared evolutionary origin with class B FMOs, although deep homology among FAD‐, NAD‐, and other nucleotide‐binding Rossmann fold domains may reflect inheritance from an ancient pre‐LUCA ancestor (Laurino et al., 2016). Importantly, this workflow and analysis establishes a clear framework to distinguish class B FMOs from other flavin‐containing protein families in plants.

Interestingly, the tomato class A FMO identified in this study (*Sl*FMO(A); Figure 3c) is distinct from the previously reported tomato class A FMO, *Sl*TNH1, described by Liscombe et al. (2022). *Sl*FMO(A) has been shown to negatively regulate drought tolerance in tomato through modulation of reactive oxygen species (ROS) homeostasis (Wang et al., 2023). In the present study, this FMO belongs to a clade comprising 18 sequences from 12 species, suggesting the presence of additional class A FMOs across the plant kingdom. It is noted, however, that as the BLAST did not specifically target class A FMOs, nor did it retrieve the characterized *Sl*TNH1. It is therefore highly likely that class A FMOs have a much wider representation in plants than what is reported here. While *Sl*FMO(A) and *Sl*TNH1 share only 37% sequence identity, structural alignment of their AlphaFold‐predicted models revealed a high degree of similarity, with an RMSD of 0.930 Å across 295 atom pairs. This structural conservation despite low sequence similarity highlights the underlying diversity within the class A FMO family and underscores the need for further research to map this family across plant lineages.

**Figure 6:**
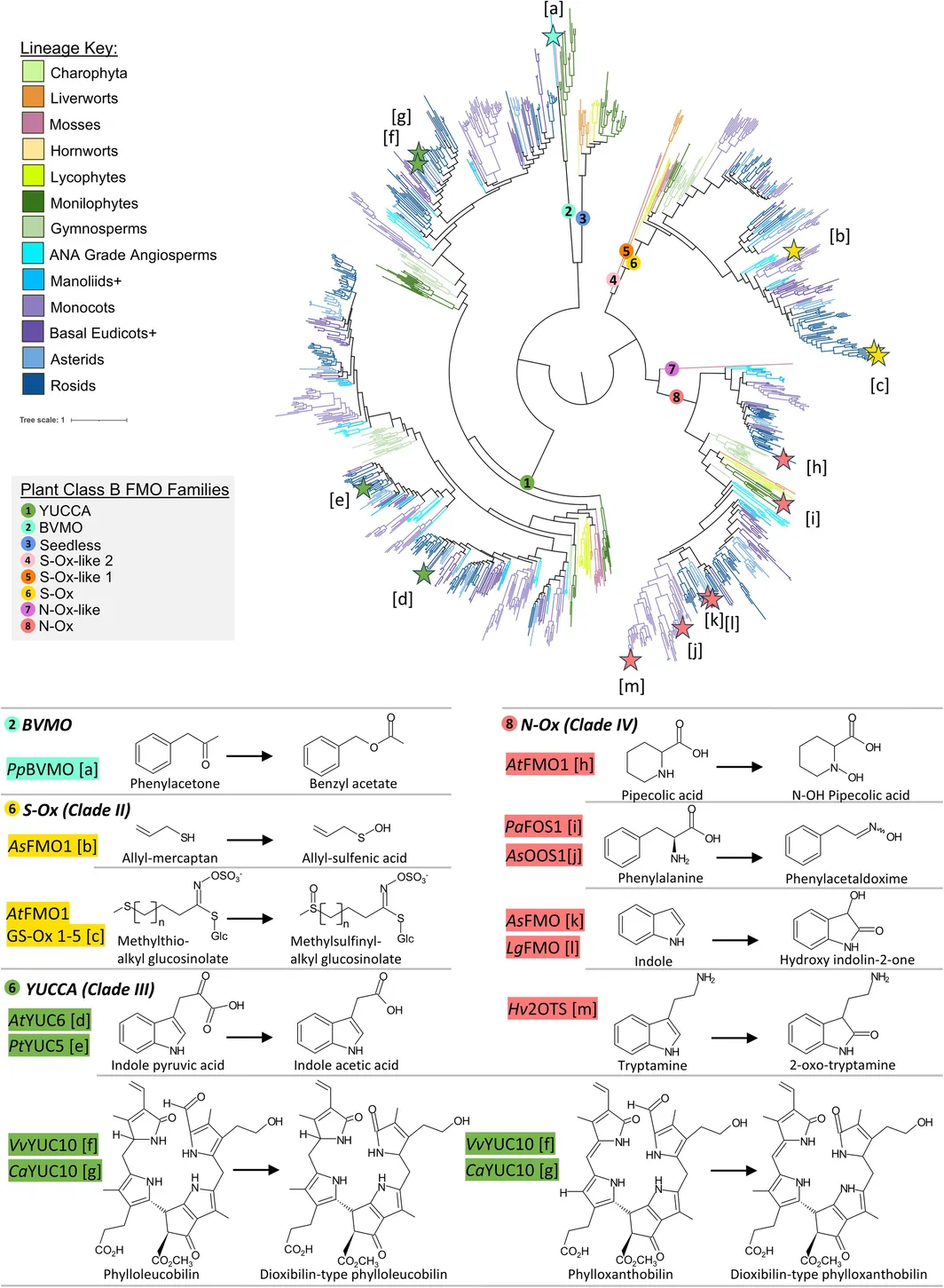
Plant‐centric overview of characterized plant class B flavin‐dependent monooxygenases (FMOs). Radial visualization of a maximum‐likelihood phylogenic tree rooted at the midpoint for balanced visualization, with eight distinct phylogenetic families denoted by numbered colored circles. Characterized and representative plant class B FMOs highlighted with letters a‐m, with their corresponding catalytic functions listed below. Due to unavailability of the *As*OOS1 data at time of tree generation, its positioning is based on the closest homolog from the orchid *Phalaenopsis equestris*. Characterized members are also denoted by the Nicoll and Mascotti (2023) clades, listed in brackets. Online version of the tree is available at: https://itol.embl.de/shared/PlantEcoPhys.

Using the genomes of 78 different plant and algae species, eight distinct FMO families were identified, including three previously unrecognized clades composed of sequences from non‐seed plant species (Figures 4 and 6). The families were classified according to phylogenetic position with bootstrap support of 100 (Figure 4a). Deeper evolutionary analysis shows that plant FMO families map into clades II, III, and IV of the class B FMO phylogeny reported by Nicoll and Mascotti (2023). Clade II includes the *S*‐oxygenating FMOs such as *As*FMO1 and *At*FMO GS‐OX1–7 and likely also includes the newly identified S‐Ox‐like 1 and 2 families (Figure S3). Clade III contains the YUCCA and Seedless families, while Clade IV is composed of plant sequences and includes the catalytically diverse N‐Ox family, with the N‐Ox‐like group also likely belonging to this clade (Nicoll & Mascotti, 2023; Figure S3).

The plant BVMO family, previously represented by a single characterized member from moss (Beneventi et al., 2013), has been expanded to include BVMOs from ferns (monilophytes) and a single angiosperm. The sporadic distribution of BVMOs among land plants, encompassing a single bryophyte, several ferns, and one angiosperm suggests that these enzymes may have arisen from three independent horizontal gene transfer (HGT) events. It is noted, however, that while high‐quality genomic resources were used as the starting input, the anomalous BVMO representative from *C. spicatus* may result from possible contamination. The disparate origins and sequence diversity of the plant BVMOs also complicates a classical sequence identity cutoff criterion for FMO families, such as the criteria set for cytochromes P450 (Nelson, 2005) and UDP glucosyltransferase (Ross et al., 2001) families. Previous YUCCA and N‐Ox family‐specific analyses suggest a plant FMO family sequence identify cutoff criteria of approximately > 40% (Jiang et al., 2025; Vijayanathan et al., 2025). However, we do not set this as an absolute criteria for plant FMOs, as the fern BVMOs share only 25–35% amino acid sequences similarity to the BVMOs from *P. patens* and *C. spicatus*.

Some FMO families, namely the YUCCA, N‐Ox, and S‐Ox families, are highly prevalent and diverse across the plant kingdom, particularly within angiosperms (Figure 2b). The YUCCA family is especially large, with many YUCCA genes retained following many whole gene duplication events (Vijayanathan et al., 2025). All characterized YUCCAs from different plant species catalyze a conserved reaction: the oxidative decarboxylation of IPA to form IAA, an essential auxin hormone (Figure 6). Accordingly, the high copy number of YUCCAs is largely attributed to the fine‐tuned regulation of auxin across plant development and in response to a wide range of environmental factors (Vijayanathan et al., 2025). Some YUCCAs—specifically Arabidopsis YUCCA6 and *Populus trichocarpa* YUCCA4/5—have also been reported to possess thiol reductase activity, which aids the mediation of ROS upon environmental stress (Cha et al., 2015; Wang et al., 2022), but it is currently unknown how conserved this role is across the YUCCA family. Most recently, *Vitis vinifera* and *Coffea arabica* YUCCA10 members were shown to possess dual functionality. In addition to auxin capabilities, these YUCCAs are proposed to catalyze Baeyer–Villiger oxygenation of the chlorophyll catabolites phylloleucobilin and phylloxanthobilin and subsequent hydrolytic deformylation and tautomerization, contributing to chlorophyll degradation and leaf senescence (Figure 6; Rütschlin et al., 2026).

In contrast to YUCCAs, the family member expansion of N‐Ox and S‐Ox FMOs in plants may be due to species‐specific adaptations and unique responses to environmental factors. Characterized N‐Ox family members perform *N‐* or *C‐* hydroxylations, with their catalytic products important for plant defense and immunity. *At*FMO1 is a key regulator of systemic acquired resistance by catalyzing the *N‐*hydroxylation of pipecolic acid to produce *N‐*hydroxy‐pipecolic acid (NHP), a crucial immune signaling molecule (Hartmann et al., 2018; Holmes et al., 2019). FOS1 from the fern species *Phlebodium aureum* and *Pteridium aquilinum*, and OOS1 from Darwin's orchid (*Angraecum sesquipedale*) catalyze successive *N‐*hydroxylations of an amino acid to form its corresponding aldoxime. Oxime synthase functionality of these two N‐Ox FMOs represents an example of convergent evolution as the trait evolved independently in ferns and Darwin's orchid, and for different purposes (Sánchez‐Pérez & Neilson, 2024). In ferns, the oxime is formed as an intermediate for cyanogenic glycoside production (Thodberg et al., 2020), while the oximes are emitted from Darwin's orchid to attract a specific pollinating moth (Jiang et al., 2025). N‐Ox *C‐*hydroxylation capacity is represented by characterized FMOs from different eudicot species involved in the biosynthesis of benzoxazinoids. These FMOs catalyze two sequential hydroxylations of indole to form hydroxy indolin‐2‐one (Florean et al., 2023, 2025). In a similar catalytic role, a recently characterized FMO from barley (*Hordeum vulgare*) hydroxylates tryptamine to produce 2‐oxo‐tryptamine, produced in response to pathogen infection (Christensen et al., 2026). Fewer S‐Ox FMOs have been characterized, but all are involved in sulfur oxidation. *At*GS‐OXs are involved in glucosinolate biosynthesis by catalyzing the oxygenation of sulfur atoms (Hansen et al., 2007; Kong et al., 2016), while *As*FMO1 catalyzes a sulfur oxidation in the biosynthesis of allicin (Valentino et al., 2020).

In contrast to the large YUCCA, N‐Ox, and S‐Ox families, the other families show a highly restricted distribution (Figure 2b). Notably, the novel families FMO N‐Ox‐like [7] and FMO S‐Ox‐like 2 [4] are comprised exclusively of sequences from the moss *S. fallax*. This highly restricted taxonomic distribution raises intriguing questions about their evolutionary origin. One potential explanation lies in the unique genome architecture of the *Sphagnum* genus, where recent comparative studies have shown that *Sphagnum* genomes exhibit no gene collinearity with any other reference genome to date (Healey et al., 2023). This suggests that *Sphagnum* genomic architecture may reflect a more ancient and independently evolving lineage with unique FMO representatives. The absence of collinearity could be the result of retained ancestral genome structures or extensive rearrangements accumulated over deep evolutionary time. An alternative hypothesis is that these gene families arose via HGT from microbial sources. Reciprocal BLAST analyses for the N‐Ox‐like family returned highest similarity scores to bacterial sequences, including *Geodermatophilaceae bacterium* (56% identity) and *Actinomycetospora soli* (53%), which could suggest potential bacterial origin. In contrast, the S‐Ox‐like 2 family yielded its top BLAST hit to a fungal sequence from *Botryobasidium botryosum* (42.42%), raising the possibility of a distinct fungal contribution. This interpretation is further supported by phylogenetic context: *Sphagnum* sequences are found within the broader moss clades of the S‐Ox and YUCCA families, whereas no moss species possess members of the N‐Ox family.

The prevalence of BVMOs in plants may also be attributed to HGT given that the members of this family are present in distinct but restricted plant lineages of moss, monilophytes and a single angiosperm. A reciprocal BLAST using the BVMO sequence from *P. patens* returned approximately 64% identity to a homolog from the moss *Ceratodon purpureus* indicating that BVMOs are present in additional moss species beyond *P. patens*. Interestingly, the *Pp*BVMO has approximately 62% identity to a bacterial sequence from *Blastococcus deserti* supporting a bacterial origin. Similarly, a BVMO sequence from the monilophyte *Salvinia cucullata* yielded top hits to a bacterial sequence from an *Acidobacteriota* species (approximately 45% identity). In contrast, the angiosperm BVMO from *C. spicatus* showed the highest similarity (approximately 78% identity) to a fungal sequence from *Zymoseptoria tritici*. These findings support the hypothesis that BVMO genes were acquired independently in different plant lineages through multiple HGT events from diverse microbial sources. As the large plant YUCCA family is also widely accepted to arise from HGT (Bowman et al., 2021; Vijayanathan et al., 2025), this phenomenon has been an important evolutionary innovation for class FMO recruitment within plant metabolism.

The phylogenetic analysis of plant‐specific FMOs presented here has enabled the reclassification of a lesser‐known family previously referred to as ‘sister YUCCAs’ or ‘sYUCs’. This family was initially identified as putative YUCCA‐like enzymes and proposed to position evolutionarily to the important auxin‐biosynthesizing YUCCA family (Carrillo‐Carrasco et al., 2023). Phylogenetic and structural analyses, however, show that this family is an evolutionarily independent lineage within the class B FMO superfamily, and we therefore designate it a new name of ‘Seedless FMOs’ as it apparently lacks related FMO members from Gymnosperms and Angiosperms. To date, no members of the Seedless FMO family have been functionally characterized. Their original identification was based on transcriptomic data from the 1KP project (One Thousand Plant Transcriptomes Initiative, 2019), providing strong evidence that these genes are actively transcribed across a wide range of non‐seed plant species. This widespread expression implies that Seedless FMOs may carry out conserved and potentially important biological roles in early‐diverging plant lineages. To investigate their expression dynamics in more detail, we analyzed the two Seedless FMO genes from *M. polymorpha* (Mapoly0122s0026 & Mapoly0052s0062) using the Marchantia Atlas eFP Browser (Tan et al., 2023). Both genes are broadly co‐expressed across a variety of tissues, with notably higher expression levels in the archegoniophore and antheridiophore, the female and male reproductive structures, respectively. Furthermore, both genes show clear induction in response to abiotic stresses, with particularly strong upregulation of Mapoly0052s0062 under specific environmental conditions (Figure S14). These patterns suggest potential roles in reproduction and stress adaptation, although functional studies will be required to validate these hypotheses. Interestingly, a FoldSeek search (Van Kempen et al., 2024) of a representative Seedless FMO from *C. richardii* against crystallized proteins identified an FMO from the bacteria *Janthinobacterium svalbardensis* (*Js*FMO), which contains the same SnoaL‐like N‐terminal fold common to the Seedless FMO family. *Js*FMO possesses the ability to perform BV oxidations (Chánique et al., 2023), and given the structural similarity, raises the intriguing possibility that Seedless FMOs may share a comparable catalytic mechanism. While functional data are currently lacking, the closest phylogenetic neighbors to Seedless FMOs — the BVMOs and YUCCAs — are also associated with BV‐type oxidations. Taken together, these observations suggest that Seedless FMOs could represent an additional, uncharacterized branch of BV‐oxidizing enzymes in early‐diverging land plants and warrants further biochemical investigation.

Predicted structural modeling suggests that the overall domain architecture is conserved across plant FMOs, particularly in the core orientation of the FAD‐ and NADPH‐binding domains (Figure 5). This core structure facilitates the fundamental catalytic mechanism of these enzymes, whereby the bound FAD is reduced by NADPH and reacts with dioxygen to form a moderately stable C4a‐(hydro)peroxy FAD intermediate. This intermediate reacts with the substrate to perform the catalytic reaction. In the absence of a substrate the C4a‐(hydro)peroxy intermediate decays into oxidized FAD and hydrogen peroxide; a process known as uncoupling (Ziegler, 1988). Some family‐specific substitutions were observed in the NADPH‐binding and FMO‐identifying motifs (Table 1) and may indicate unique reactions. For example, the tryptophan substitution in the FMO‐identifying motif of BVMOs, together with a conserved arginine (also present in all plant BVMOs), is critical for binding the peroxyflavin Crigee intermediate during a confirmation shift in catalysis and insertion of oxygen into a carbon–carbon bond (Fraaije et al., 2002; Malito et al., 2004; Mirza et al., 2009). Here, we also report an S‐Ox family‐specific diversification of the FMO‐identifying motif of ‘WxGxxxHxxxY’ where this initial tryptophan residue replaces the canonical phenylalanine (F) (Table 1), inviting further analyses to investigate whether this motif is linked to the *S*‐oxidation catalytic reactions considered unique to the FMO S‐Ox family in plants (Figure 4).

Beyond clear family‐specific structural novelties (i.e., the beta barrel of S‐Ox‐like 2; Figure 5), the most notable structural variability was observed within the NADPH‐binding domain. The structural diversifications between the different plant FMO families likely contribute to the functional diversification reported for characterized members. For example, variable portions could influence substrate specificity by acting as a gatekeeping module that regulates access and binding to the active site (Valentino et al., 2020), affect sub‐functionalization (i.e., cellular/tissue localization; Vijayanathan et al., 2025) or be involved in determining the proteins oligomeric state. Most purified or structurally characterized class B FMOs has been shown to form homodimers (Alfieri et al., 2008; Cho et al., 2011; Eswaramoorthy et al., 2006; Thodberg et al., 2020). One exception is the S‐Ox FMO — *As*FMO1 — from garlic (*A. sativum*), which stands as the only plant FMO to be successfully crystalized, occurs in a mono‐ and trimeric form (Valentino et al., 2020). Interestingly, *As*FMO1 also possesses the smallest and least elaborated NADPH‐binding domain among the predicted structures and may suggest a potential link between domain truncation and oligomeric form. These findings raise the possibility that the structurally variable portion of the NADPH domain may influence dimerization or mediate protein–protein interactions. To date, very few biochemical studies on plant FMOs have been reported. Aside from the aforementioned *As*FMO1, only *At*YUC6 has been biochemically characterized in detail (Dai et al., 2013). This lack is likely due to issues relating to FMO expression and purification. Plant FMOs are challenging to work with (Thodberg & Neilson, 2020) issues relating to poor soluble protein recovery, inefficient folding and FAD incorporation, and poor heterologous expression reported (Chen et al., 2018; Dai et al., 2013; Krönauer & Lahaye, 2021; Thodberg et al., 2020). Nonetheless, biochemical studies focusing on the dimeric interfaces and overall quaternary structure of plant class B FMOs will be necessary to determine whether structural variation in different regions contributes to functional specialization.

## CONCLUSIONS

This study presents a comprehensive phylogenetic and structural analysis of class B FMOs in plants, identifying eight distinct families, including three newly recognized groups found exclusively in non‐seed plants. Structural modeling showed that all plant class B FMOs share a conserved domain architecture composed of two dinucleotide‐binding domains. However, considerable structural diversification was observed across families, particularly within the NADPH‐binding domain. In addition, we define a refined set of conserved FMO‐specific motifs by anchoring the analysis in structurally conserved folds rather than amino acid sequence alone. These motifs offer a tool for phylogenetic annotation and discovery of uncharacterized plant class B FMOs in the future studies. Altogether, the expanded phylogenetic analyses and insights into structural variation provide a foundation for more precise characterization of class B FMOs in plants.

## METHODS

### Dataset assembly and assessment

A total of 78 species were selected to ensure broad and comprehensive representation across the Streptophyta lineage (Figure S1; Table S2), with a prerequisite of a fully sequenced genome. Genomes were retrieved from publicly available repositories, including Phytozome (Goodstein et al., 2012), PlantGenie (Sundell et al., 2015), Phycocosm (Grigoriev et al., 2021), and FernBase (Li et al., 2018). If a genome was unavailable in these databases, predicted peptide FASTA files were obtained from other sources (Table S1). Representative sequences from each class B FMO family were selected, including those from key model organisms: *M. polymorpha* (liverwort), *P. patens* (moss), *Selaginella moellendorffii* (lycophyte), *C. richardii* (monilophyte), *Picea abies* (gymnosperm), *A. thaliana* (dicot), and *H. vulgare* (monocot) (Table S2). These sequences were used as BLAST queries at the amino acid level against each of the 78 genomes individually. Local BLAST searches were performed against retrieved proteomes, and hits with an *e*‐value ≤10^−3^ were retained. Sequences shorter than 100 amino acids were removed. Sequences were aligned using MAFFT v7 (Katoh & Standley, 2013). Initial alignments were visually inspected to identify clusters of closely related sequences, which were subsequently realigned separately to improve alignment quality and facilitate the identification of sequences containing large insertions, deletions, or frameshift mutations. Sequences that failed to align with any other members of the dataset were excluded from further analyses. Manual curation of each subsequent alignment was conducted to remove misannotated proteins and pseudogenes exhibiting large insertions, deletions, or frameshift mutations as previously described (Figure S2) (Koleva et al., 2024; Thomson et al., 2022). Additional algal class B FMO members were identified by utilizing BLASTP in the PhycoCosm algal genomics resource (Grigoriev et al., 2021) querying with representative members from each identified plant class B FMO family and collecting the best scoring hits from each, with S‐OX representatives limited to the top 10 hits. Further manual assessment and visualization of sequences was performed in CLC Main Workbench 20.0.4 (QIAGEN, Aarhus, Denmark). The presence of canonical class B FMO motifs (i.e., FAD‐binding motif (GxGxxG), NADPH‐binding motif (Gx(x)GxxG/A), FMO‐identifying motif (FxGxxxHxxxY/F), BVMO‐identifying motif (FxGxxxHxxxW), and FATGY motif (F/L/YATGY)) in phylogenetic groups 1–19 was conducted using the motif search tool with an 80% accuracy threshold.

### Class B FMO motif analysis

To generate motif logos for class B FMO families (Groups 1–8), multiple sequence alignments (MSAs) were performed using MAFFT and visualized in Jalview. Sequences lacking information at the motif sites were excluded from conservation analyses. Family‐specific motifs were identified using STREME (v 5.5.8) from the MEME Suite (Bailey, 2021; Bailey et al., 2015). For each analysis, protein sequences belonging to a given FMO family (Data S7) were used as the primary dataset, whereas the complete class B FMO sequence dataset was used as the control dataset to identify motifs enriched within the target family. Motif discovery was performed using the protein alphabet with motif widths restricted to 5–8 amino acid residues. Motifs were considered significant at a threshold of *P* < 0.05. Other parameters were left at their default setting. A complete list of family‐specific motifs identified by STREME analysis is provided in Data S8–S12.

### Phylogenetic analysis

Phylogenetic relationships were inferred using ML analysis based on MSAs generated with MAFFT v7 employing the FFT‐NS‐2 alignment strategy (Katoh et al., 2019). The initial phylogenetic analysis included 1642 sequences, while the class B FMO phylogenetic analysis incorporated 1273 sequences. ML phylogenetic inference was conducted using IQ‐TREE with ModelFinder selecting the optimal substitution models: JTT+R10 for the initial analysis, JTT+F+R10 for the class B FMO analysis, Q.pfam+F+R6 for the algal class B FMO analysis, and Q.pfam+R10 for the combined phylogenetic analysis on identified class B FMOs within this study and in Nicoll and Mascotti (2023). All phylogenetic trees were inferred with 1000 bootstrap replicates utilizing the UFBoot2 algorithm (Hoang et al., 2018) to assess branch support.

### Structural analysis

All structural models were predicted using AlphaFold 3 (Abramson et al., 2024), incorporating FAD as a ligand. Structural analyses and visualizations were conducted with ChimeraX 1.9 (Meng et al., 2023). To supplement manual domain architecture determination, the CATH domain server (Greene et al., 2007; Orengo et al., 1997) was used. Accession of predicted structures (AtYUC10[1]; AT1G48910, CrSeedless FMO[2]; Ceric.34G041100, PpBVMO[3]; Pp6c18_10300, ScBVMO[3]; Sacu_v1.1_s0081.g018001, SfS‐OX‐like 2[4]; Sphfalx02G048800, MpS‐Ox‐like 1[5]; Mapoly0020s0006, AsFMO1[6]; Asa8G05386.1, SfN‐Ox‐like[7]; Sphfalx14G087800, AtFMO1[8]; AT1G19250). Structural alignments and root mean square deviation (RMSD) calculations (Table S3) were performed using the Matchmaker tool (Meng et al., 2006) in ChimeraX, with no pruning of long atom pairs.

## AUTHOR CONTRIBUTIONS

JMC: Conceptualization; data curation; investigation; methodology; writing—original draft; writing—review and editing. EHJN: Conceptualization; investigation; supervision; funding acquisition; project administration; writing—review and editing.

## CONFLICT OF INTEREST

None of the authors have a conflict of interest to disclose.

## Supporting information


**Figure S1.** Subtree of streptophyte species included this study overlaid the entire taxonomy of streptophyta.
**Figure S2.** Curation process to generate a robust alignment of highly divergent sequences.
**Figure S3.** Class B FMOs across different kingdoms of life, combining identified plant class B FMOs with the FMO gene sequences from Nicoll and Mascotti (2023).
**Figure S4.** AlphaFold 3 predicted structure of AtFMO1 superimposed on AtYUC10 to assign class B FMO motif; FMO‐ID (purple), NADPH‐binding (orange), and ATG (aquamarine) motifs of AtFMO1 (colored in white).
**Figure S5.** Identification of a family‐specific STREME motifs.
**Figure S6.** Full conservation logos of YUCCA alignment created in Jalview.
**Figure S7.** Full conservation logos of BVMO alignment created in Jalview.
**Figure S8.** Full conservation logos of seedless FMO alignment created in Jalview.
**Figure S9.** Full conservation logos of S‐Ox‐like 2 alignment created in Jalview.
**Figure S10.** Full conservation logos of S‐Ox‐like 1 alignment created in Jalview.
**Figure S11.** Full conservation logos of S‐Ox FMO alignment created in Jalview.
**Figure S12.** Full conservation logos of N‐Ox like FMO created in Jalview.
**Figure S13.** Full conservation logos of N‐Ox FMO alignment created in Jalview.
**Figure S14.** Gene expression of two Marchantia SeedLess FMOs visualized in the Marchantia Atlas eFP Browser (Tan et al., 2023).
**Table S1.** BLASTP queries for sequence acquisition.
**Table S2.** Species list with data source.
**Table S3.** BLAST analysis of small class B FMO families.
**Table S4.** RMSD values for comparison between predicted protein structures.

**Data S1.** Navigational sequences.

**Data S2.** Navigational sequence alignment.

**Data S3.** Class B FMO sequences.

**Data S4.** Class B FMO alignment.

**Data S5.** Class B FMO and algal hits.

**Data S6.** Class B FMO and algal hits alignment.

**Data S7.** Individual family sequences.

**Data S8.** YUCCA STREME analysis.

**Data S9.** BVMO STREME analysis.

**Data S10.** Seedless STREME analysis.

**Data S11.** S‐OX STREME analysis.

**Data S12.** N‐OX STREME analysis.

## Data Availability

All relevant data can be found within the manuscript and its Supporting Information.
