# Supplementary material for: A plant‐centric view of class B flavin‐dependent monooxygenase evolution and diversity
Source: Plant J. 2026 Sep 9;127(5):e71106. doi: 10.1111/tpj.71106 (PMC13557801; doi:10.1111/tpj.71106)
Supplement: Supplementary file 1 [file TPJ-127-0-s004.docx]

>Aspi01Gene36448|Alsophila_spinulosa/1-417

MMGMAHMAEVMCKESNDSNGGGAIVRVEDGVIIVGAGPAGLATAACLKQKGIASVVLEKSDCIASLWRKRTYDRLCLHLPKQFCELPCMRFSSSVPKFPTRQNFVAYLEEYASKCGERPRFNETVSCASFDTSTSMWHVLSHEGARGRRDVVYCAPWLVVASGENAEPLLPQGLPGLHNFNGKSLHTSHYKNGLEFSGQRVLVIGCGNSGMEVSLDLANFGAKPAVSVRSPVHVLPREIYGISTFSIAMTLTKLLPLRWTDRLLLLLSRLVLGDPSRHGFKKPTFGPLELKETMGKTPLLDIGTLTKIKDGSIRVVPAVQKLTHSGCFFKDGTYSEFDAVILATGYQSNMPSWLKVEPNCFNQACTGHWKGKDGVFFAGFSRKGILGISMDAQRIAADISKHYNSLKDAKRKLHLHV

>Pp6c3_9570|Ppatens_v6.1/1-434

MYPRKDSWKSGDLPVCPSVQLGSIAGESLRNRSISSKVNSKEAPQTVLIIGAGPAGLATAACLRSKYWIPSIILERANCSAPLWRYMTYDRLRMHLPKQFCQLPLRPFPAVYPKYPTKNQFIAYLEDYQRHFGISPVYNATVTSAEFSTALGLWVVIAEQKLEDNCETVTYTTRSLVVATGENAEPYMPDLFGSHKFHGVISHGSTYRNGVKYKDMKVLVVGAGNTGMEISLDLAKFGAKPTLVARSKFHVMPRDLFGLNISAFQVMLMLLKVLPVSFVDKLLVIFSRLTLGDTDHLNLVRPKEGPLKMKARTGHTPVLDVGTVAEVRNGFIKVAPAIDQLTKSGARFVNGVEEEFDAVIMATGYTSNVYEWLKIDGMSGINGFPKRPFRNGWKGGRGLYAVGFGRKGLMGCAHDAELVADDIGAHHRDNEKTY

>AT4G32540|Athaliana_Araport11/1-414

MESHPHNKTDQTQHIILVHGPIIIGAGPSGLATSACLSSRGVPSLILERSDSIASLWKSKTYDRLRLHLPKHFCRLPLLDFPEYYPKYPSKNEFLAYLESYASHFRIAPRFNKNVQNAAYDSSSGFWRVKTHDNTEYLSKWLIVATGENADPYFPEIPGRKKFSGGKIVHASEYKSGEEFRRQKVLVVGCGNSGMEISLDLVRHNASPHLVVRNTVHVLPREILGVSTFGVGMTLLKCLPLRLVDKFLLLMANLSFGNTDRLGLRRPKTGPLELKNVTGKSPVLDVGAMSLIRSGMIQIMEGVKEITKKGAKFMDGQEKDFDSIIFATGYKSNVPTWLQGGDFFTDDGMPKTPFPNGWRGGKGLYTVGFTRRGLLGTASDAVKIAGEIGDQWRDEIKGSTRNMCSSRFVFTSKS

>Mapoly0020s0006|Mpolymorpha_v3.1/1-460

MKKRVAVIGAGPSGLSLLRAFDSAAEKGAEIPEIVCFEKQGEVGGLWKFEEGKGVDEHGEIVHGSMYRDLYINGPKEVNEYVDYSFEEHYGKVIGSYPPRPVLLSYIRGRAEKYNLCAKFSVRFNSSVSKISYSEDTAKFTVTVKDRSTARNGTEAKVYSEDFDHVVVAVGHFSTPNVPQFPGLEAFKGSVLHAHDVRDLSEFRGMDVLLVGSGYTAEDIACQCFKLGAASVTITFRSNPTGCCNWPESIKEVPLLERVDSNGRTCHFKDGSSKDVDAIILCTGYLHDFPFMVGDELRLVAGNRMWPLGLYQGVVLETHPKVFYLGMQAQFYSFTMFDAQAWYVRDVIMGRLTLPSSTEEMVAHSRKWREAELAALAKLDVKPFQGDYVKELIRHTDCPITPEFVDKTQQMGVDWDKHKALDIMSYRDHAFVSAVTGNLARTHHTPWMQNFDDSLESYVN

>413958|Smoellendorffii_v1.0/1-446

MEKRVAVIGAGASGLVAARELLREGHSVVIFEQARRIGGTWVYDPPPQESHSRSSNVARNHSCHSSIYQSLRTNLPREVMGFLDYPFVPRRSSRDARRFPGHEEVLDYLESFTVEFGLHGYVRFNSKVKYVGLVESNWIVKSSSSAGKEEKESEEVFDAVVVCNGHYFQPRVAQIPGIEKWPGKQYHSHTYRTPDLFKDQVVAVIGNGPSGQDLCSDIAADCKKVHWCAKLWNSPVESLQQGKIQRHLMISKADKDGRLHFMDGTSAVVDAILHCTGYLYDFSFLDTKNYVKVEDNRVGPLFKHVFPPALAPSLSFIGLPWKTIPFPLSELQAKWISAVLKGRASLPPKGEMANDVVGFYRTLEAQRVPNRHTHRLDLEMFDYAEWLAKQCGGTCHGFEPWRKELFLSTRDNRKLNSDSYRDEWSDNDLHEKVVGVLATSFFMREQ

>jgi|Selmo1|175034|estExt_Genewise1Plus.C_280315

MEKRVAVIGAGASGLVAARELLREGHSVVIFEQARRIGGTWVYEENHSGSSNAARNYSCHSSMYESLRTNLPREVMGFLDYPFVPRRSSRDARRFPGHEEVLDYRESFAVEFGLHGYVRFNSKVKYVGLVESNWIVKSSSSAGKEEKESEEVFDAVVVCNGHYFQPRVAQIPGIERWPGKHYHSHTYRTPDPFKDQVVAVIGNGPSGEDLCSDIAAACKKVHWCAKSWNSLSEPLQQGKIQRHLMISRADEDGRLHFMDGMSAVVDVILHCTGYLYNFSFLDTKSYIKVEDNRVGPLFKHVFPPALAPSLSFVGLPCKTIAFPLSELQAKWIAAVLKGRASLPSKGEMANEVVDFYRTLEAQGVPKRYTNHLYLETFDYAEWIAKQCGGTCHGFEPWRKELCLSTLDKKKLNPGIYRDEWNDNDFHEKVVAILAKKQQQ

>AT1G12160|Athaliana_Araport11/1-468

MTSVITSRARHVAVIGLGAAGLVAVRELRREGHTVIGFEREKHVGGLWVYTDRVDSDSVSVDPDRTIVHSSIYQSLRTNLPRECMGYSDFPFVTRSSDGDPRRYPDHREVLMYLQDFAKEFKIEDMIRFETEVLCVEPSPENNRKWRVQFKSSNGVSGEEIFDAVVVCNGHFTEPRLAHIPGIESWPGKQIHSHNYRIPDPFKDEVVIVIGSQASGNDISTDIATIAKEVHISSKMVASDSYGCYDNLRIHPTIYRAREDGSVVFRNGKVVFADAIVHCTGYKYHFPFLKTSGYVTVEDNRVGPLYKHVFPPALAPGISFIGLPFMGLQFFMFEIQSKWVASVLSGRVKLPAEDKMMEEAVAFYSKLEDLGIPKRYTHFLTDPRGNPMLGTFKPEDAVVISQSDYFNWIAKQCGCTSIERWRERLYNVAIKKVFFGGDSYRDRWDDDQLIEEVYREFAKLKPNQDCSS

>Poptr1_1|eugene3.00130182

MQASPNLLSSHHVAVIGAGAAGLVAARELHREGHKVVVFEKDDQVGGLWMYDPRVEPDPLGLDLTRPVVHSSLYESLRTNLPRETMGFMDYPFVTREGEGRDPRRFPGHREVLMYLQDYAREFGIEEMVRFGCEVVNVEMIDSGKWKVKSKRKRLDDNDRGDDFADHEDFDAVVVCVGHYTQPRIAEIPGINLWPGKQIHSHNYRIPEPFRDQIIILIGASASAADISVEIAGLAKEVHIARRSAVDDDTYEKKPGYDNIWLHSTIERACEDGTVIFRDGSVILADVILHCTGYKYGFPFLKTDGIVTVDDNRVGPLYKHVFPPILAPWLSFVGIPYWTFPFPTFEVQSKWIAGVLSGRIALPSQEDMVEDVKIYYSELEASGVPKHHTHNLAHSTNDYNMWLASQCQCSCFEEWRIEMSHEILKNWRARPNMYRDEWDDDHLILQAHEDFNRRISNKASNGHI

>jgi|Meskra658_1|1799451|fgenesh1_kg.Chr_24_#_1028_#_TRINITY_DN72_c2_g1_i3

MHSLSTYLHSPSHLAFTVSSSSKVKGHLTKRSKSGVKGSLPHSQRGLRLPKFRPGQVFCKPYVSKSKCRCRCGIGCVESSWSMEVSTEISTRCQSNFVKVPGDFAEAAKVDKGGNSTCRKIISTAAFNHETSPVPSEGQQPMELEQNRKPGLRVAVIGAGAAGLAAARELLREGHQVEVFEQGSEVGGVWVYDDLTESDPLGLSSSREAVHSSMYASLRTNLPRECMSFLDFPFTAPPGSSDSSRYCTHQTVCLYLQHFAEVFGLLPHVRFRSRVEKVTPEHPVSRQFPAESRPGEAQALSSSCNGVGLANQDAGKAAEDFIGRARDSIEEGELLSASPTAKRNHWIQWRVETSRVTSSESNIDDANGGENKGLSLNSQACREGVTSSERGRMDKTEVYDAVVICNGHYSQPKIASVPGIETWPGRQIHSHNYRRPEPFTGLVVVILGAASSGEDIARETAGTAREVHLCSRGWTAEEVPGSPEGKHRNLWRHPMVVKAHSDGRVDFGDGSNVHADVILHCTGYVYHFPFLRGVEPWFGVEDARVGPLYEHVFPLHLAPTLSLVGIPWQVAPFPLMEIQAKWIAKVLAGKITLPSPQEMEAEVQSFYGQMEAAGVPQRYSHAMGSTAVVGQFEYLDGILERCGLPPLDRWRRGMYAAASLNKRSNPDNYRETWTDYDILAEAVASQAKILEANPHW

>jgi|Meskra657_3|3031339|fgenesh1_kg.23_#_1670_#_TRINITY_DN5806_c0_g1_i8

MCPCLMHSLSTYLHSPSHLAFTVSSSSKVKGHLTKLSKSGVKGSLPHSQRGLRLPKFSPGQVFCKPYVSKSKCRCRCGVGCVESSWSMEVSTEISTRCQSVFVKVPGDFAEAAKVDKGGNSTCRKIFSTAAFNHETSPVPSEGQQPMELEQNRKPGLRVAVIGAGAAGLAAARELLREGHQVEVFEQGSEVGGVWVYDDLTESDPLGLSSSREAVHSSMYASLRTNLPRECMSFLDFPFTAPPGSSDSSRYCTHQTVCLYLQHFAEVFGLLPHVRFRSRVEKVTPEHPVSRQFPAESRPGEAQALSSSCNGVGLANQDAGKAAQDFIGRARDSIEEGELLSASPTPKRNHWIRWRVETSRVTSSESNIDDANGGENKGLSLNSQACREGVTSSERGRMDKTEVYDAVVICNGHYSQPKIASIPGIETWPGRQIHSHNYRRPEPFTGLVVVIVGAASSGEDIARETAGTAREVHLCSRGWTAEEVPGGPEGKHRNLWRHPMVVKAHSDGRVDFGDGSNVHADVILHCTGYVYHFPFLRGVEPWFGVEDARVGPLYEHVFPLHLAPTLSLVGIPWQVAPFPLMEIQAKWIAKVLAGKVTLPSPQEMEAEVQSFYGQMEAAGVPQRYSHAMGSTAVVGQFEYLDGILERCGLPPLDSWRRGMYAAASLNKRSNPDNYRETWTDYDILAEAVASQAKILEAHPHWLKEASLPL

>jgi|Zygcyl6981a_1|43428|Zci1a_00166.1

MSSVAIAPLLVARVRSPLPLSSFQLSSSNVRGAMAVSPHASVMTSAGSHGKTLKVAVVGAGAAGLVAARELLREGHHVAVFEQSSEVGGTWNYSEAIDSDPISQDSSRTRVHSSMYASLRTNLPRECMGFLDFPLVPALTPGSEDSRRYCSHEAVALYLQHFARVFEIVPHIRFRTQVDFVEPLPMKSSHNSLQLRNATTDKNGEEESKEEWLKWRVGSSSASILDEHDSGLNSSVDKDEVEEKSEEEIYDAVFVCNGHYSEPKVAAIPGLEEWPGRQIHSHNYRRPEPFSGLTVVVVGAGSSGEDLAREVAMVSREVHMCARSWSPSENGSPSRPDGPRGNLWRHPMAVRAFPDGRIEFADGFSVVADVVLHCTGYTFSFPFLRNVESFLEVSDARIHPLYEHVFPPSLAPSLSFVGIPWQVAPFPLMELQSTWIAKILSGKVELPSKEAMDSDVQAFYERLEEAKIPKRYTHAMGKTAIIQQFEYLDGLLSRCGLPPVENWRKEMYSLGGFNKQSNPDSYRDTWQEADVALEAATAQEAQLKALEKR

>jgi|Zygcir6981b_2|15954|Zci_06976.1

HGSHSLKVAVIGAGAAGLVAARQLLREGHHVAVLEQGSRVGGTWQYEERAESDPLSRAPSRERVHGSMYASLRTNLPREVMAFRDFPLSVSVPVPVAVAVDGRRYCSHGTVARYLERFAEVFGVLPHVRFGRRVEWVKWRVASREVTFGSAVEEVYDAVVVCNGHFSEPKIADIAGLSEWPGRQMHSHNYRRPEPFAGLVVVVVGAGSSGEDIAREAAMAAREVHMCARSWPGAGGGGSPSPTPSPSPSPSPGGKYKNLWRHPMAVRAHADGKVEFADGVSVVADVILHCTGYMYHFPFLRGIDHCVKVSDSRVGPLFEHVFPPTLAPTLSFVGLPWQVAPFPLMEIQSTWIAKVLSGSVGLPSREEMESEIETFYKRMEEAGLPQRFTHSMGKTAVVGQFEYLDGICARCGILPVEQWRREMYALGSLNKQNNPDEYRDMWPESSLVEGEVAAAQAAQLEALRALTDTVSISGL

>jgi|Myrbis2043_1|636|KAK9830413.1

MQRPGVPALHKPRLLVAWARVRRVMAPSAALQSASLAPQGPGQKSVAVIGGGAAGLVAARELLRESHCVTVFEQGPGLGGVWKYDPQTEQDDPLGLRPDRQRVHSSMYARLRTNLPREVMAFLDFPFDALTMQGRSSDPRRFCGHEEVQRYLEAFAEAFALEQHMRFNMRVQQLTPLPHSQLDSANGLDSTEQGCPRGQAAIWNGASNETSSNGASTGREYSQEFDAVVVCNGHYSQPRLPDVAGAAAFPGRQLHSHNYRENSAFAGQAVVVVGASASGEDICREVAEVADKVYLCARSWQNPAWTHETTPIGQRANIWRRPMVQALGANGSVTFTDGQTLERVDCILYCTGYQYAFPFLEDAGVVSIEDNRVSPLFQHIFPPTAPSLAFVGLPWRVVPFPQMELQSKWVARVLSGRVSLPAAVDMQRWVDDRYRELDAKQVPVRYTHYQGDAQWAYNDWLAEACGPDVPKLPSWREAMYKATGNNKRDFPEKYRDVWRDEGLVEQAAVDARKSRSRPQHAVVEGVAAA

>jgi|Cosub3|1196263|fgenesh1_kg.4_#_571_#_HTPTB_transcript/17940

MHVAAQRSPRFFLSKVLGPRIVPTEYRSLVIFGAQKLTTRAATMNGRDHTTESLKVAVIGAGAAGLVTARELRREGHEPVIFEQGSKVGGVWVYTDKVEEPHGASSRIGAAEERVHSSMYAGLRTNLPREVMSYTDFPFTRSWGDTRRFCGHAEVEAYLEAFAAAFDLEKYIRFNTPVLSLTPCEAGSPPRARLGSDTAPTNGHQAGHQREAMPGTEEDGKGLVGLPNGHAAVNGTSNSRELPWPRWRMTTAPTGDQGDVSTSGDEVFDAVIVCNGHYSDPRRPDIPGMAEFPGRLLHSHSYRRNEPFEGMTVVVIGASASGEDISREIAHVADKVYLCARSWQNPAWAAETVEPFGARRNIWRRGVPSRLHPDGGVTFQGGKRVDAVDVVMFATGYCYSFPFLADTRIDGAEIVTVEDNRVSPLYKHIFPPSTAPTLSFVGLPWKVVPFAQYELQAKWIARVLSARVSLPGQRHMLADISAFYADLDKEGVPKRHTHMLGDKQWEYNDWLAAACGPDVTPLPQWRPKMYKVAGDTKRAVPETYRDVWPEAELLAEAAAEASRLGAPVEIQNQIAKTTVV

>jgi|Klenit1_1|1302|kfl00117_0190_v1.1

MTVDVAANGKEGQKTRVSRSVCVIGAGAAGLVALKELREEGHQVTAFEQNEDIGGVWKYDPDTDDDPLGIREDRKKVHSSMYASLRTNLPREIMSFAAYPFVPSGEPGRDDRRFCSHQEVQAYLRDYARHFDLHRHIRFGTQVLRVSRLPEKTNQYGSVQQPLWEVVTRTASSSENWACDTTEFDAVVVCNGHYSQPKLATIEGIASWPGRQLHSHNYRTPGPFAGQTVVLIGAFASGEDISRELAEHCSEVYLSARSWKDEPRQTPPNLHRKLVVTRAYPDGSLEFADGSRVVADVIIHCTGYVYEFSFLESGLVSTEDQRVHPVYEQVFHVGTAPTLSFIGLPFKVSPLPMFELTTRWVAQCLSGRAVLPSEAAMHKWIDGFYEGLKAGGVPVRYTHEMSRTGFDYLARLCEWTGTPMFESWREDLFKDFSYRRHNYPANYRDEWPEDLELKRKVIWEDLRKEAEKLSLGGSEKSFGS

>jgi|Coprin2167_1|3429|KAK9903581.1

MEIVDVRPISSDSSRSLSARKVAVIGAGSAGLVAARELSKEGHQVQVFEQGQSIGGIWCYQDEVEDDLLGKCPGRRKVHSSLYSSLRVNLPREIMSFSDFPFTPEVMGGRSQDRRRYPHHTEVLGYLEAFVQEFDLRPLIRFGTRVVDVSPVTGLEGRPEAPNGLPSNGREICSANHWIVKSELLPTADLQVPEVHAEVFDAVIVAVGNYHEPNLPDVEGMEDFPGLQMHCHNFRHNEVFTNKTVVVVGASFSGEEIARQIADVALHVYHSARTWSKQLTEPKGRANLTRMPMLTRLGSNGRADFFGGVSADSVDAVVYCTGYQYSYPFLKRTGLICTDDNRVTPLYRHIFVPSVAPTLAFIGLLWKSLRNSQFELQAKWVAQVLSGRVALPTQQEMEADMEAFYELLKEKGIPVRYTHCQNDAMPVSQWEYNNLLRESCSPVPPPAEAWRARLHDAISTKILESPDTFRDEHSAEEQALFDDAAQACAEMARPFTGMPIMDRGLLQSGGLSFNTQDGSAVNDLLQLLLNNDPRAAQLIKQDAQQGRTGAIATALATASATGNGQAAATAVSQAVAQGAPASAVAKAVAAATSNAPVGAPSQALPPASPGLENAAASGNTQAIASALAVSTGGKVESVASANAQTFTQAENAISQAVTTAVAQVKGGGNATAAALSAATAIGNATAEAFASASAKTTVQGKGLGAADASATAAATSTAVEHAIATAIAAATNNNAQALSIARAEDIQKAVARAIATATASLVSTGGTAQVDTQAVARDVQDAVASTLAAAFAVVKGSQNGSSAPSSGRHKN

>jgi|Cosub3|1238953|fgenesh1_kg.8_#_2755_#_HTPTB_transcript/21239

METLDLKSGGSDRCQSLSASKVAVIGAGSAGLVAARELSREGHHVQVFEQGQTFGGIWNYQDEVEDDLLGRCPERRKVHSSLYSSLRVNLPREIMSYSDFPFTPDAMRGKSQDSRRYPHHTEVQHFLEAFVEEFGLRDLIQFSTRVVEVSPLDYTVGQQACSNGLCSNGEEPLSISPQWVITTELLPGSDLQVPERHTDVFDAVVVAVGNYHEPNLPDVEGLDDFPGLQMHCHNFRHAERFRDQTVVVVGASFSGEEIARQIADVALHVYHSARTWGKQLNDSQTRPNLQRVPMLARLGGNGSAEFSGGITAEGVDAVVYCTGYRYSYPFLERTGLISTDDNRVTPLYRHIFVPSVAPTLAFIGLLWKSLRNSQFELQAKWVAQVLSGRVALPSREEMEADMEAFYRLLEKNSIPVRYTHCQNDAMPVSQWEYNDMLRRSCSPVPLPAEAWRVKLHDAISTKILQQPDTFRDVHSAEEQALFEDAAKGCAATWAYLKERHAA

>jgi|Spipra1|3415|Sp_v1_0120200.1

MPSSIEPLRVAVIGAGAAGLVSARELLREGHDVTIFEQSEQVGGVWVYDERTEEDLTIHPNSRRKLHCETETKTKTKTEAEAETSASNGVFCSLYASLRTNLPRELMSFTDFPFVTELSSESSLFPSLSNNRSSNHHPSSSSSSSSSSSSSTLSSNSDINTSIQTLRVDSNDERRFPSHRTVLNYLRRFAEAYNLLPRVKFSTRVISIHPISDASASHVTSNTSDTDTWIKWQVATCQTVTKTKDEINGLDLNSNLNRNSYKYEKKIEKENTNVIDTYDAVIICNGHYSIPKWTDIEGIDNWNGKQMHSHNYRSPNMFKNKVVGIIGFGNSGDDIAREIADVADKVFVFSRSWNHENVPIGSFGRRKNMFCYPMIQKAHANGSVELEKGEKLFMDNLIHCTGYFYSFPFLESLKSQFKVNEQRVEPLYMHMFHPSFSPSLSFIGLPWKVVPFPLFELQSRWVSKILSKKVVLPEKSKMEKEIKKFYADLAKRNVPIRYTHMLGDCQREYFDFICDELGDEKLEGWRNEMYESVSKQKKTKPDDYRDRWQDVELHDMAMMCQCRQYQTIKERLDQGLFLI

>jgi|GsuMS1_1_1|5328|GsulpMS1_G1757.1

MEQNLQLGNNAKIAIIGAGAAGLIAAKNLRDIGLFQVEVFEETDTVGGTWVYQEEVATSYYFQSSMYRDLHTNLPKEIMGFLHFPFDDTFGVSSFPSHQVVLKYLVSFCKTFQLYELIRFHCSVKQISRFEYFGGFHWDLVLYNHGTQQVEQRRYDAVVVCNGHYTKPYIPEIPGADLFLRPIIHSHFYKSPEPFCKLRIAVLGAGNSGIDISYELSRMASKVSLCHRKCQIRKTIGKNLEECPTIESLEADGKILLADKSSLQVDILILCTGYEYDFPFLDSSCEVFVQDRVVLPLYRHLIHAKYPTMSFVGLPLRVLPFPLFDYQTRYLASIYSGKCTLPSCERMLVEQQEHLVDLDSKGCRKYYHLFAEKQWNTAENLQI

>jgi|GsuRT22_1_1|3935|GsulpRT22_G1455.1

MEQNLQLGNNAKIAIIGAGAAGLIAAKNLRDIGLFQVEVFEETDTVGGTWVYQEEVATSYYFQSSMYRDLHTNLPKEIMGFLHFPFDDTFGVSSFPSHQVVLKYLVSFCKTFQLYELIRFHCSVKQISRFEYFGGFHWDLVLYNHGTQQVEQRRYDAVVVCNGHYTKPYIPEIPGADLFLRPIIHSHFYKSPEPFCKLRIAVLGAGNSGIDISYELSRMASKVSLCHRKCQIRKTIGKNLEECPTIESLEADGKILLADKSSLQVDILILCTGYEYDFPFLDSSCEVFVQDRVVLPLYRHLIHAKYPTMSFVGLPLRVLPFPLFDYQTRYLASIYSGKCTLPSCERMLVEQQEHLVDLDSKGCRKYYHLFAEKQWEYCRELADLANGPRLSVAVQRVYEDVSQFRKKEPEKYRRRKYIIFGKGPNDWRVFENDSDVTL

>jgi|Caulen1|93398|g8005.t1

MAQKRVAIIGAGAAGLVCAKECIQEGLNVQMFEKYPYSGGLWVVDEVPLDLQELNVHEDGYTDMARKAYSSVHDTLQTNLSRFLMGFSDFPFDTNFPGSKDPRTFPGNDEVLRYLDAFKKEFELDQFISYETEVINVDRTDDSEFEWQVKINCKGEIKTLFFDSVAVCSGHHTVPQLPELKNIMGFGRPCLHSHLYRNPNRYKDKVVLILGSGGSGRDTALEISKVSKKVYFCAREWKAPYDMSTPFEVIENIECCDSISHCPAKGTVQLEDGTILTDVDEMLLCTGYIYDYPFLKDSGLISTAESFVYPVYKHIFHTKYPTLGFIGLIDFANVSFLTFECQSKYFARVILGRCQLPDYEIMMKDIDQHRQRLEENNQPIKHFHALSYIENPFIYKNEVLSLCDDCSALPSWRKELFDSNLYLLLTEPLTFRAIEIPSDIQAIIGAGAAGLICAKECIQEGLNVQIFEKHPYSGGMWVVDEVPLDLQELNVHEDGYKDKAKKVHSSIYDEMQTNLSRFVMGFSDFPFDTEFPGSKDPRTFPGTDEVLRYLDAFKKEFELDQFISYETEVIDIDRTEDSECEWQVKINCKGKIKTLFLDSVAVCSGHYTVPQLPELKNIVGFGRPCLHSHLYRNPNRYKDKVVLILGNGSSGRDIVIEIAEVAKKVYFCARKWKSTLDLSKAFEGIKNICCCKSISHCPAEGTVQLEDGTILTDVDEMLLCTGYSYDYPFLKNSGLISTAESFVYPIYKHIFHTKYPTLGFIGQSSQLSVINRGIRLPPGIHLVTYFLQVAWQMLSLIPHCFILITILSLGAIPCIDAEYKDEGTDIGTAVFISRLAFLHIVQNLRSHVCFSKGEAALRIGRDETATSLQDRNPQFVRPFGAPSVPNAYRSGPTRTLKYSGCSETHNTISAIVECSNAVRQNPDLFVNDYPCSYASWRNEVSGKSLGPLKTGPTIMRLAQLHTDDQARMNTMSHQGSDGSDLQTRADRVDLPWGTVGENVAVGFSSAKAVVMAWMCSPPHRKNLMSCDFSTIGVGAGVSSAGRNFYTQVFSCGVSEECSCGPQGGALKRRKEKKSSTEQFQLPLEKTHSGPLCGWVDGEWKCDQHFGASTQPYNPFGY

>jgi|Cosub3|1167623|fgenesh1_kg.1_#_10111_#_TRINITY_DN7923_c0_g1_i1

MSDEERRNGPTPSCSDTKLPSKDHKITIEITTRAGNDALKQRACVIGAGKSGLIACKVLHERNIPFDCFEASSQVGGLWVLNSDSGLSASYESLRINTSKQMTSFHDFPMPKHYPTYPTRKEILEYLESYADHFGFRSHITFRTEVKKVEQLPDGSFRVFAEDKATGHTLARLYTSVLVANGHHWHAAWPELPGSFTGTLMHSHEYRTPKVMEGKRVMVIGAGNSGMDIASEASQCGAAAVFLSCRRRVHVVPRYIFGAPSDSILPAWLGVTAPRRLMEKGVTCLIHISRGSQTSFKFPPPDFGLLRVHPTVSPGTGDILQLIKDGKVTVRPGIERIEDRTVHFTDGTKEDIDIIVCATGYNVSCPFLPPKVEVLKGDKPQLLLNVAHPRAPGIFFLGFVQAHGPMIPCVEGQMPWVADLIQGITELPGEAEMAAKIAEQHEFNRRNFTQVKRHALMVEFHRYLLQLRMARCPKGKGWLGRKLWKAKTFLVFCTGKTC

>jgi|Coprin2167_1|5401|KAK9917333.1

MSDEERRNGPIPSCSDKKVPTKGHTIAIQITTRAGNDAPEQRVCVIGAGKSGLIACKVLHERRVPFDCFEASSQVGGLWVLNSDSGLSASYESLRINTSKQMTSFHDFPMPQHYPTYPTRKEILEYLEAYADHFGFRGCITFRTEVTRVEPLPDSSFRVFTEDKATGHTVARLYTSVVVANGHHWNAAWPELPGTFTGTLMHSHEYRTPKSMEGKRLMVIGAGNSGMDIASEASQLSRGSQASFKFPPPKFGLLRVHPTVSPGTGDILQLIKDGKVAVRPGIERIEDHTVHFPDGTKEDIDIIVCATGYNVCCPFLPSEVEVLKEDKPQLLLNVAHPHAPGIFFLGFVQAHGPMIPCVEGQMPWVADLIQGIAELPEKAQMEAKIAKQHDFNRRNFTQVKRHALMVEFHRYLLQLRAAKMQVKAVC

>jgi|Botrbrau1_1|11255|Bobra.0173s0079.1

MAALPCVPQSLDEHEGSLKHAMQYGLCGMEHSLRNTKRQLRGHPAACFAAYRCPRIVPESKSRTSSNHKTVCVVGAGCAGLSACNALRERGIPYVCYEKGSKIGGLWRIENDNGLSGAYESLSINTSKSTMEFAGFPFPEEWTPFLSHKQVLEYIEQYAAHFDLKKNIQFETTVTEVAPHNNGGYTVSIQRKIESKPKSKHFATVIVANGHHWDPLFPTLPGKFAGQLLHAHQYRNHSILDDKRVVVLGMGNSGVDIASEASKYARSVIISSRESAYVVPRWIKGKPMDTINTSAIALLPWRWRQWLFSQQLKPFIQEQIAAGWGVPKHGLMQAHFTIHKDIVEQCKAKKVTPKSGIKQISGNLITFMDGTSVEVDVIVCATGYNVTFPFFKGNRIRVVDEDGPRLYKNMIHPEHPNLFFVGLLQPTGPMIPLCEQQTALIGDILQGKVVPPCPEKQLRAIQLQKQRVKDRWISAQRHLLEVDFHEYLMELKCERCPSKISFWERILYTIKVYLEALFPRTGCLGATRYEKSPEHHQSIEK

>jgi|Botrbrau1_1|17708|Bobra.0352s0002.1

MDTNSICIIGGGAAGIALARVLNDRGIQFTWLEKGTRLGGLWVYNNDSGMSAIYQSLTTNTSRTTMGFPGFPLPEQLPDFPPHHEVLKYLDAYIERYNLQPLATLGCEVEKLEEAPDGAFLVSSLVLATGQRECRRYESVILATGMNWDPALPSLEGSFSGIVMHSVQYREPSILKGQRVLVVGAGPSGADIATEAAREAGSATLSLRHGMYAFHRYGKDGRPSDTKNAAWLGLLPWKLRQYIMTRAVQPKALPGFPQPAYQLLQRTGHLKTKPGIASLEDRTVQFVDGTREDFDVIIFATGFRESTPFLSSELEGRLRSADGQLSLYLNIVPPDVPGLYFTGFVRQLGPIFPSFHEQALFLADLLQGKCKLPAPESMRQSLTIRHANGEKRYSKPYKSLEVDLHIYWLQLRIARCLRGLPVWRRWIWTIQSFFSILFRPIEES

>jgi|Cyapar1_1|14550|g14034.t1

MRIAVIGAGVSGLTAAKCCADEGLTPVVFEQSRQIGGLWRFTEEESHSSIMRTTVINTSKEMMCFSDFPMPESYPTFLPHRLVLQYLEDYAEHFKLLQYVRFGCRIVSVEPERVGEERPESAGGPGGRWRIRWESTHDESGTGASAAAEELFDAVMVCTGHHWKPRTPAFPGADTFKGVQVHSHAYKDDLPYRGKRVVVVGIGNSGADIAVELSRAAAQVELSTRSGTWVFPRYLLNTPFDHMLSRLVSTICPLWVRTLFMEKAISLTHGSVKDFGLRPKHRIFQAHPTIHSELLGRIGTGTIAVRPDVAELHPDAVSFADGTRVKADAIIYCTGYEIEVPFVKNRPGGNPLSVGIGPSNSVRLYKHMLPLEGPPTLAFIGLVQPLGAIMPISEIQARWFARLLKGLCRLPPREEMARDVERREAAVRARYKPSPRHTVQVDYLDYMEEIAGLVGCRPRLSNPLVWLRSPRVALATLVGPASPVHFRLRGPGAWAGAPDAVLRMAGWDKGPSKAALGLGALLLAAAAAALAFALYAGAGRASLSFLPVR

>Sphfalx14G087800|Sfallax_v1.1/1-543

MLKGQAVKETAILFSSTLDITLPPPPNSMKVAIVGAGLSGLVTAKVLAQFGHKVTVYEKCPDVGGTWSRTRWYPGLTTQNGKDSYCFSDFPMPDSYPEWPSGEQVHQYLDEYVKHFGLDALLQLNTEVVSIELDEVAGNIWTIGTRQSDNNKDSSMPILATQSETFDHVVIANGIFSDPYVPTFPGLEEFQQAGGQVLHTTDVHQLLGDATGKHFIIVGYGRSACDVACALSDISSIKVVVVARELIWKIPKWIGGVLNFKYLLLTRMGEALFQYIHPHGFEQFLHNGLGKPLRDLMMSSLQAIITCQLKLRKLGLLPDGPLEDIARTTLSLVTDGFYEKVEMGAIEVHGGTHIVKLCVIDDKPSTLLSDGTIVPTDFVICGTGFHQSVPFFSMNIIKQLTDDQGNFLLYRHIHPINVPQLSFNGYNSSLFCPLSSEVAALWIGNLLAGGMKLPSPNKQQLEVIEQLAWMEKRTKGKHACGGSIIPFTFHYIDELLKDMNLTLNAHTHIREWLLPINFGAYVGLIDELTKRLASEGKLTFPLC

>jgi|Chrzof2|4671200|fgenesh1_kg.chr_08_#_4392_#_transcript/24020

MRIAVIGSGVSGIVVAKTLLQAGHAVTVYEKSRNLGGVWNPDVVYPGAHTQNIGHMYRFTDFAPPKTWDKYPTATQVYDYLRDYATHNELWGSIRLSTNVIRIMALTTDDGRAGWFVTVHSSGQRLEEVFDFVVISTGTYCQPSIPSFTGQEEFVEAGGKVFHTSQLSDVDISSKIAGKSVAVVGFGKSALDVATYCTNCATAKSVTILYRQARWCVPKKLAHLIPYEYVLSCRAGMLLMPSPYEGHLVQFIRKLLKPFTWLAFRSMEMLISMQQGLRKIGMVPCEQLEKALTCEGSHLETDGFYDAIHQGKLKPKQGEIQSLSPGQLTLKDGSKLSADVLILGTGYIWHHLPFLDPNTRKQLIDEDKNTCQLYRRILPLNIPNLAFIGYQSSLTSVLTFEMGAKWLTAYLEGRIKPTHDMMQDTLLKERSYALRNFTGKHVRTVSGPCIAPYAMPYVDSLLSDMNQKPVKGLINKLKDFLLPWDASLYKDCR

>jgi|Chrzof2|4671163|fgenesh1_kg.chr_08_#_4355_#_transcript/69809

MQIAVIGGGISGLVVAKTLSKAGNVVTVFERSRNLGGVWNPDVIYPGAHTQNVGSMYRFTDFAPPSTWETFPTSEQVYDYVVSYVKHNDLLGSIQLNTTVTSIKPITGPTACKGWSVTVEHNGQSSTEEFEFVVVCTGTYREAKIPIYPGQDEFVKAGGKVVHTSQLSDVDISGTITGKSVAVVGFGKSALDMATYCTNCTTAKSVTILYRHLRWCLPKVLGGLLPYQYALLCRAGMLLLPSPYASPLVQFIRMILLPVFWVAFRSLELLITLQQGLHKLGIVPTVQLNRSMTCEGAQIEPDGMFQLMHDGKLKAKQGEIQSLSPGQLTLKDGSKVSADVLILGTGYIWHHLPFLDPNTRKQIIDEDKNTCQLYKKILPLNVPNLAFIGYQSSLTSVLTFEMGAKWLTAYMEGRIKPTHEMMQDTMLKERSYAVEHFTGHNVNSLFGPCIAPYAMPYVDSLLSDMKQEPVKGLINKLKDFLLPWDASLYKDCR

>AT1G19250|Athaliana_Araport11/1-530

MASNYDKLTSSRVAIIGAGVSGLAAAKNLVHHNPTVFEASDSVGGVWRSCTYETTKLQSARVDYEFSDFPWPNNRDDTTFPPYLEILDYLESYAKHFDLLKFMKFGSKVIEVRFIGDGETPQMVDLGAYGNLLPGKPVWEVAVQIGDSGDIQWHAFEFVVVCTGKYGDVPRIPAFPAKKGPEMFQGKVMHSMDYCKLEKEEASTLLSGKKVAVIGFKKSAIDLALESALANQGEGGKACTMVVRTTHWGIPHYWVWGLPFFLFYSSRASQFLHDRPNQSFLRTLFCLLFSLLRAVVSKFIESYVLWKLPLEKYGLKPNHSFEEDYASCQMAIIPENFFEEADKGMIRFKKSSKWWFYEEGIVFEDGTTLEADVVILATGYDGKKKLKAIVPEPFRTWLEFPSGVMPLYRGTIHPLIPNMGFVGYVQSSSNLHTSELRSMWLSRLVDEKFRLPSKEKMLDQFLKEMEVTRNSSRFYKRHCISTFSIQHADDMCNDMGLNPWRKSNFLLEAFSPYGSQDYRLGQEEKEDMTA

>Mvestita_S18g14860-RA|Marsilea_vestita/1-572

MDHATTKSSIAIHPYILRSQSSETYSERLSKEEEQGSFVLLHELMESSANLPVSQSPDAPSVCIVGAGMTGICASKYLLQHGIQPVIFEAQPGIGGVWRHTFASTKLQTPRDAYQFSDYTWPPGTPDYPSHDHVMKYLHSYASEFHVLERIRFNCKVVEARQVGCFDVADDNGNVDEGARKGHQWAVCVQKISDASSASTEWFYFDFLLLCIGRYGDVPNLPIYPPSRGPEIFKGKVLHSMEYAMLDDKTANEFLANKRVVVVGMMKSALDVAVEASNANLGLHGHPCTVVFRSAHWMLPHHKPFGLPLSYLYSTRFSELMIPKPDQGHFLSIISTLLKPMRWTISKFVELYLLLKLPLRQFDLVPKQTFYQQFASCQVPLLPDLFFQRVQAGHIQLKRTNDWSFSENGLVLDDGTNIEADVVILATGYKGEMKLKSILQGSCAQGLKSSGDIIPLYRGIIPPRIPHLAVIGYHENLSNLHSSEMGARWLAHLLSSKIHLPSIVKMEEDVQKWTDFMKNSTPFYQKSCIGAVSIWHSDQMCRDLGWNPKRKNNCIQELFTPYSNMDYQDCST

>jgi|Spimu1_1|10495|SM000022S07132

MLGPGMVAAPTDGDGCAATAAVLATPPGSAWLDPRGARGSASRHAAAAVCLSSDERMASQAAHSGREPGDVSLRDERRWLLPASSAERNSVLFLPSEPTNERGGSLPRQQAGSDEAAQDACSRVSSDQWRPPSADRTSSAKRVAVIGGGVSGIAACKVLSEDGHNVTVFEARGSIGGIWSHTYPSTALQTPKEAYRFSDWPWPESVPTFPSHEQVMEYLSTYVRHFNLGARFRLNTRVMKMTRLRVAPGGQAGWSLILEEQKPGSLQPQETVQEFDFAVMCCGCFGDVPKLPCFEPGKGPEVFQGTTLHSAQYSELDKAGTEKLLHGKKVVVVGYQKTSIDVAIEAAEVNRDVAHASCTLLFRKPHWLLPRSQLLYGLPLSYLILSRFAEFWVPKPGQSRLETLKQTLLSPLRSLYFGLIEMYLLYNFPLRKYGLVPEHSFFQDLSACILPQLPDSFFALIEQGSIQLQQSTQWHFTRKGVVLMGEKEIEADVVLLCTGYDGASKLKSVLPCEYRDCLSTPGECLYLYRGMLHPEIPDVAFLGYHEGLSAVQSAEMGSRWLGKVISGTLVLPAGEVMERERQHWRAYKQRQTPFFRRVSCIAPVHIWHYSTLCQDMGWNPRRKPGFLANIFAPHSSMDYCSPAVVADEHVEVSFSSPACKEQALVQEPSARQCLHHWAAEKLLQVCGWWLVLEAVVTGALRGKGSRQAGPPVCGAKED

>jgi|Spimu1_1|21718|SM000006S19534

TRGATEHRNRAWPDWHGLQAEEKQSTDCQTDPTRHKAWGRAETRPGVQICPPPPPPPPPPPSPPPTARAAAPARPDEAPPSSSDRRRCHGIAVASLRRLPAWPLLRALRACRGALAGRGNKLGLRMVAAPADGEGCAAAAAVLATPAGSVWPDSGGAQGSASRHAAAAVCSSSDDERMAFQTAHSGREPGDVPLRDEHRWILPASSAERNSELRLPSEPANERGGSLPCQQAVSDEAAQDACCRGSSEQWRTPSADSTLSAKRVAVIGGGVSGIAACKVLTEDGHNVTVFEARGSIGGIWSHTYPSTALQTPKEGYRFSDWPWPDSVPTFPTHEQVMEYLSTYVRHFKLGARFRLNTRVMKMTRLPVAPGGQAGWSLMLEERRPGSLQPQEIVQEFDFVVMCCGCFGDVPKLPYFEPGKGPEVFQGTTLHSAQYSELDKVGTEKLLHGKKVVVVGYQKTSIDVAIEAAEVNRDVAHASCTLLFRKPHWLLPRSQLLYGLPLSYLILSRFAEFWVSKPDQSQLQTLKQTLLSPLRSLYFGLIEMYLLYNFPLRKYGLVPEHSFFQDLSACILPQLPDSFFSLIEQGSIQLQQSRLWHFTRKGVALMGEKEIEADVVLLCTGYDGASKLKSVLPCVYRDCLSTPGECLYLYRGMLHPEIPDVAFLGYHEGLSAIQSAEMGSRWLGKVTSGTLVLPAGEVMERERQHWRAYKQRQTPFFRRVSCIAPVHIWHYSTLCQDMGWNPRRKPGFLANIFVPHSSVDYCSPAVVADDEHVAVSFNSPACKEQALVQEPSAVQCLHHWATERLLQVCGWWLVLEATFTGALRGKGSRQAGIPVCGAKED

>jgi|Spimu1_1|2272|SM000116S24242

MLGAGMIDAPADGDSCAAAAAVLATPAGSAWPDRGGAGGSASRLAAAAVCLSSDDGRMAFQTAHFGREPGDVPLRDEHRWLLSASSAERDSVLRLPSERRGGSLPCQQAVSDEAAQDACGRVSSDQWRTPSADSTPPAKRVAVVGGGVSGIAACKVLSEDGHDVTVFEARGSIGGIWSHTYPSTALQTPKEAYRFSDWPWPESVPTFPSHEQVMDYLSTYVRHFNIRARFRLNTRVMKMTRLPGWSLMLEERIPVSLQPQETVQEFDFVVMCCGCFGDVPKLPSFEPGKGPQVFQGTTLHSAQYSELDKAGTEKLLHGKKVVVVGYQKTSIDVAIEAAEINRDVAQASCTLLFRKPHWLLPRSQLLYGLPLSYLILSRFAEFWVPKPDQSGLETLKQTVLSPLRSLYFGLIEMYLLYNFPLRKYGLVPEHSFFQDLSACILPQLPDSFFSLIEQGSIQLRQSTQWHFTRKGVALMGEKEIEADVVLLCTGYNGASKLKSVLPCEYRDCLSTPGECLYLYRGMLHPEIPDVAFLGYHEGLSAVQSAEMGSRWLGKVISGTLVLPADEVMERERQHWRAYKKRQTPFFRRVSCIAPVHIWHYSTLCQDMGWNPRRKPGFLANIFVPHSSVDYCSPAVVADDEHVGVSFSSPACKEQALVQEPSAMQCLHHWATVKLLQVCGWWLVLEAIVAGALRGKGSRQAGPPVCGAKEH

>jgi|Meskra658_1|1272703|estExt_Genewise1.C_Chr_12_t20322

MALANLCMELATPKICTNFYDEAFSTCRRSPHIAAASLQAATLQFPQFGTGSEPLAPSNSALQRNGRSFKRPHRVFIHCTLAGDFTQDYSSPANQQKIDNQELTGVNGIANAGHAAVPHFHSSGNKVATYADSAANGQGIHHSAGRAGLDASSMNTGLSSGAVTRVSATSSIGLSEAWPGIGGLDERIDRRGHPLGRTEELNGVDSGRPRFGTPGDVSAKATKLDPDSKVAIIGSGVSGIAACKALTEAGIDVTVFEARERLGGIWSRTYPTTSLQTPRTAYRFSDWDWPVDAPMNPRHDQVVEYLQGYIEHFQLGDKFKFNSRIAEMQRPEGGRRGWRLRIQRKRTNEDGSLGEVEEQWLQFDFVVNCCGCFGDVPNIPQFAAGEGPDVFGGTVMHSAQYSGLGPDGCEALLRGKRVVVVGFQKTAIDVAIEAAEANKGSDGKPCTLLFRKPHWLVPRNQLIFGVPLKYLLLTRFSELFHRTPNQSPLLTALQTVLPLRALVGKLVEAYLCGKYPLQRHGLVPSHSLVDDVAACILPQMPPAFFPLVEAGRVQLARAPRWRFTPAGVELDDGRQLAADVVLLCTGYDALAKLRGLLPAPYAGQLSSNGRSLYLYRGVIHPRVPNMAFLGFHEGASSVQTSEMGARWLAKLLQGAFALPDEGAMERDRREWWEFKRAQSPFAGRVSCVAPIHSWHFDLLCRDMGWSPWRKKGWVANLFSPHTSADYVEREADRRATAEEKRAAHGGPSSEVKVV

>jgi|Mesen1_1|1395|ME000013S00885

MPEVAIIGAGVSGLAVCKTLTEAGVPVVVYEARGSIGGIWSNTYASTTLQTPRSSYCFSDWPWPDDAPMHPSHDQVIHYLQGYIDHFRLRSKISLSSKVLEMKRATAGDSWLVDGGGYAPLVVHNTGKGTETRGGPGGVACTLLFRRAHWIVPRSGRVFGIPLPFFFLTRLSELGLPKPGQHPLVALLQRLSPMRWVTSKLVELYMVRTYPLRKHGLVPSHSFSQDLAACILPQLPDKFFPLIEQGRILLKRSASWHFTPAGMRLENGEELAADVVLLCTGYNGLAKVKAMLPEPYSALLEEEGESLHLYRGLVHPRIPDVAFLGFHEGLSSMHVAEMGARWLTALLRGGFSLPSAAAMDAERAEWRRYKRAQTPFSQGRVSCIAPLHVWHFDALCRDMGWPLMRKRGLLQNLLLPYGSVDYRAPAPPSASPAPGPALTPVPVVVTLATGAADAAAAKADKEARSVHAHYQEQRTARGAAAALASATASLTAAAAMTPATATE

>jgi|Zygcir1559_1|6120|UTEX1559_15026.1

MHRRVRGSPCRQAEGCVCAFCSDGALPASHVATRSVSIAATAAAVGVGGGRGGEASCAEDGGRRKMAAVQREGGNSRGPPSLLSLCVAFLGCHLDELIDEAHHFVPAFPTPVRQALAGIARRRGLMCDELLRALIDDSWRVLDVSGSAVTDDGIRCAADTCSRLEAVDLSGCDDVTVEALQRLVEANPQLHTIRFGGTPASDAVARGSLRHIIPGISVMEAEREEGSWEDVDVEHVGRGAQALRWLVWPTIDEKSLRRIESKCPRIRINPKKPEYWTSIVRTPPEALQQFDLDAPFVEGIDPITWSDGKDWRRLNENCDENGDLGCDVSSFSDLSLAERFKLAYKLRDERLAPKRAKNLRQKLRRAERAWLDENGEARARVLAGTICKFQKVQKLGILTKVDDREETIVGLNIGDAKDSTLFTGGPLSSSSRSQEERPIQQLADETRMKIFRFAKRSGTEHCNSAMNALFTLLHTLESLERGSRRESQQVMREAIDRAVAAEQEGANTPLNLQCTILLLGKSGAGKSTVVNIFLGGPIADASSNKGTKAVREYRQRVHGVDFTLIDTPGLQPASSDRKMNEKMLSSLSKYLQRKKKKIDLILYCERLDTKVEEISELQILLTISSILGSRSWLRTSILFTHALTPIGANFNEQDLHPEYAAERKSHIFRRLISEAAGPSLDMGLNPFALIDANDKMEESPLAGKWLPNIFSMCCALKLLNEVEEKATTHHSYLAWDGEQREWESLFSGEGEGEGEGEGEREDFWKGSLACLLDEELSSIRDSVVGRKEATLRNGASEGFFPHTENLRRPNTSGEEGSKDRQLGFEEGDDLQSQQSPYSQPVKIQRSFRILSDKIAVLLKGAFSANKTKGIEAEFAAHGAGMYGPRECQGSLQLQKLPWIDCYGCAARAAIRAKPTAWNCVGVGATALFADDGTGQTEKRERTAMGLKIEDSLQLSENIFISAAAGVVGARREIAKGAQIEADICNGARSGRDNVPFKEREMLQRGRVGLGIMLLKAAGRVVTSGNLRTDITCEGGLRVQAGACMTSSGYGHIAASVLSHGLPEAVPLLLLPAGYGALWASRFFSGTGGERREEGGEREQEGVRGWLREASDRPERRVADLQPATLLACFKMGHGLSLRSDSLLGFLERKGRVGPGGDPMWPGVSPNGRVAGNGAQCSDSSSSPASHVPSAAISGPFTDSESTQLEQKSFLKSLSSAEYWPSHLKEFPGAAHAREAARRGKLKDAPSTHFSGRNALEKGSRVAVVGAGVSGMVSAKLLMEAGYEVTVFEAREKIGGIWARTYASTSLQTPRIAYRFSDWEWPDSAPMNPSHREVVEYLQGYANHFGVYNKIRFNTRIVEMQEAEGSERGWRLRVKGGESGGRDGETELWLHYDFVVMCCGCFSGVPHIPKFESGKGLEAFKGTTMHSAQYSELDEAASRSLLHGKKVVVVGFQKTAIDVVLESAEINRGSESAQSTLLFRKTHWLVPRDQMVCGVHLKYILLTRFVELFFPYPQQGILRTILQKLVPVRQVLNSMVERYLKQRYPLEKYNLIPSHSFHDDVSACILPQLPPTFFPLVEEGSIRLQKSATWHFTSAGIALEDGRQLEADVVVLCTGYDGLGKLRGLLPPHYGSKLASPGQSLSLYRGVLHPRIPNMAFIGFHEGLSSMHSAEMGARWLSKLLQGAFSLPEAVAMEAECSKWWTFKNSQTPFAGRVSCVAPIHMWHFDALCRDMGWDPMRKKGFVANWFTPHGSVDYVETAPSVRDSPEEEKLREREELLPLTAVPSSRDVAKPNFEGGSFAEEFYNMDKLAYTYSGQGEPAGKQGLQGPLPLQPSGKARLAAGAGAGAGAGAGAGAASAVAALCCILLLLHLSGAAADVYVANMTGLDVVGYPAPVPETYEGTAVLRIQDTGRIDYVFTQTDLDEIQEVWLKEPVPGDPINGQTIFNFNLTRVQLAAFTENGSLSGFIDVDPYLAIGVTQRPYQFYISVKTSKYSQGAIKGFLRDTTPSPTTGLEPNWLIDTNMTAEEVVPKAGPENGYTTGLFSVFIQSNGSLYVEFDFMDLNFTGLTSVGLYTGKKGAKGTLVYDMTPHVNLPAFASNEFTAGAFVTTKEVLFPILSNFNQSYCQIASSGYPSGASRGQLHLVKVNLQSPPSDAPVGYLQDSPPPSGACRRREWEEWRLTLSVLSSCLLCLLGAPWLLG

>jgi|Zygcir1560_1|6463|UTEX1560_14885.1

MHRRVRGSPCRQAEGCVCAFCSDGALPASHVATRSVRIAATAAAVGVGGGRGGEASCAEDGGRRKMAAVQREGGNSRGPPSLLSLCVAFLGCHLDELIDEAHHFVPAFPTPVRQALAGIARRRGLMCDELLRALIDDSWRVLDVSGSAVTDDGIRCAADTCSRLEAVDLSGCDDVTVEALQRLVEANPQLHTIRFGGTPASDAVARGSLRQIIPGISVMEAEREEGSWEDVDVEHVGRGAQALRWLVWPTIDEKSLRRIESKCPRIRINPKKPEYWTSIVRTPPEALQQFDLDAPFVEGIDPITWSDGKDWRRLNENCDENGDLGCDVSSFSDLSLAERFKLAYKLRDERLAPKRAKNLRQKLRRAERAWLDENGEARARVLAGTICKFQKVQKLGILTKVDDREETIVGLNIGDAKDSTLFTGGPLSSSSRSQEERPIQQLADETRMKIFRFAKRSGTEHCNSAMNALFTLLHTLESLERGSRRESQQVMREAIDRAVAAEQEGANTPLNLQCTILLLGKIGAGKSTVVNMFLGGPIADASSNKGTKAVREYRQRVHGVDFTLIDTPGLQPASSDRKMNEKMLSSLSKYLQRKKKKIDLILYCERLDTKVEEISELQILLTISSILGSRSWLRTSILFTHALTPIGANFNEQDLHPEYAAERKSHIFRRLISEAAGPSLDMGLNPFALIDANDKMEESPLAGKWLPNIFSMCCALKLLNEVEEKATTHHSYLAWDGEQREWESLFSGEGEGEGEGEGEREDFWKGSLACLLDEELSSIRDSVVGRKEATLRNGASEGFFPHTENLRRPNTSGEEGSKDRQLGFEEGDDLQSQQSPYSQPVKIQRSFRILSDKIAVLLKGAFSANKTKGIEAEFAAHGAGMYGPRECQGSLQLQKLPWIDCYGCAARAAIRAKPTAWNCVGVGATALFADDGTGQTEKRERTAMGLKIEDSLQLSENIFISAAAGVVGARREIAKGAQIEADICNGARSGRDNVPFKEREMLQRGRVGLGIMLLKAAGRVVTSGNLRTDITCEGGLRVQAGACMTSSGYGHIAASVLSHGLPEAVPLLLLPAGYGALTLLACFKMGHGLSLRSDSLLGFLERKGRVGPGGDPMWAHHFTGPGVSPNGRVAGNGAQCSDSSSSPASHVPSAAISGPFTDSESTQLEQKSFLKSLSSAEYWPSHLKEFPGAAHAREAARRGKLKDAPSTHFSGRNALEKGSRVAVVGAGVSGMVSAKLLMEAGYEVTVFEAREKIGGIWARTYASTSLQTPRIAYRFSDWEWPDSAPMNPSHREVVEYLQGYANHFGVYNKIRFNTRIVEMQEAEGSERGWRLRVKGGESGGRDGETELWLHYDFVVMCCGCFSGVPHIPKFESGKGLEAFKGTTMHSAQYSELDEAASRSLLHGKKVVVVGFQKTAIDVVLESAEINRGSESAQSTLLFRKTHWLVPRDQMVCGVHLKYILLTRFVELFFPYPQQGILRTILQKLVPVRQVLNSMVERYLKQRYPLEKYNLIPSHSFHDDVSACILPQLPPTFFPLVEEGSIRLQKSATWHFTSAGIALEDGRQLEADVVVLCTGYDGLGKLRGLLPPHYGSKLASPGQSLSLYRGVLHPRIPNMAFIGFHEGLSSMHSAEMGARWLSKLLQGAFSLPEAVAMEAECSKWWTFKNSQTPFAGRVSCVAPIHMWHFDVLCRDMGWDPMRKKGFVANWFTPHGSVDYVETAPSVRDSPEEEKLREREELLPLTAVPSSRDVAKPNFEGGSFAEEFYNMDKLAYTYSGQGEPAGKQGLQGPLPLQPSGKARLAAGAGAGGGAASAVAALCCILLLLHLSGAAADVYVANMTGLDVVGYPAPVPETYEGTAVLRIQDTGRIDYVFTQTDLDEIQEVWLKEPVPGDPINGQTIFNFNLTRVQLAAFTENGSLSGFIDVDPYLAIGVTQRPYQFYISVKTSKYSQGAIKGFLRDTTPSPTTGLEPNWLIDTNMTAEEVVPKAGPENGYTTGLFSVFIQSNGSLYVEFDFMDLNFTGLTSVGLYTGKKGAKGTLVYDMTPHVNLPAFASNEFTAGAFVTTKEVLFPILSNFNQSYCQIASSGYPSGASRGQLHLVKVNLQSPPSDAPVGYLQDSPPPSGA

>jgi|Zygcir6981b_2|7494|Zci_13903.1

MAQAVIWVNSALVASIASSASVYTSSCNRNPKAVALASDFLQPLHLLSPEARSGSSERQRERQRLRQREQQRERHRERQKQGGSHSPCILAAAREGGASTDGPGGAHHFTGPGVSPNGRVAGNGAQCSDSSSSPASHVPSAAISGPFTDSESTQLEQKSFLKSLSSAEYWPSHLKEFPGAAHAREAARRGKLKDAPSTHFSGRNALEKGSRVAVVGAGVSGMVSAKLLMEAGYEVTVFEAREKIGGIWARTYASTSLQTPRIAYRFSDWEWPDSAPMNPSHREVVEYLQGYANHFGVYNKIRFNTRIVEMQEAEGSERGWRLRVKGGESGGRDGETELWLHYDFVVMCCGCFSGVPHIPKFESGKGLEAFKGTTMHSAQYSELDEAASRSLLHGKKVVVVGFQKTAIDVVLESAEINRGSESAQSTLLFRKTHWLVPRDQMVCGVHLKYILLTRFVELFFPYPQQGILRTILQKLVPVRQVLNSMVERYLKQRYPLEKYNLIPSHSFHDDVSACILPQLPPTFFPLVEEGSIRLQKSATWHFTSAGIALEDGRQLEADVVVLCTGYDGLGKLRGLLPPHYGSKLASPGQSLSLYRGVLHPRIPNMAFIGFHEGLSSMHSAEMGARWLSKLLQGAFSLPEAVAMEAECSKWWTFKNSQTPFAGRVSCVAPIHMWHFDALCRDMGWDPMRKKGFVANWFTPHGSVDYVETAPSVRDSPEEEKLREREELLPLTAVPSSRDVAKPNFGELVET

>jgi|Rhomari1|33|NDN08_005046.1

MIVREAKRGFRVAVIGAGTAGVATTRVLSEQGFDCVVFEKHSKSGGLWADNYYGASVQMPKSLYRFPDEVHAAEANEFLGKDEVCDYLERIIDKHEIRKCFRFNTLVTEMKQLEGGIWRLTVKDLKTDVSNDETFDYLVMCNGTFSSKPHRPNFPGQEKFQGKIMHSSEWRSPDEFAGKKIVIVGMGKSAVDVSMVASEVGESAVQLVRSMHWFTTSHVGFIPTDMVTFTRLFQMMLPPFYSSSSMYKNLHKLAAPVRWLYFRTIEALMLTNYRLPRNLWPKSRFEFDVLHPALFNGPAEYLADVRSGRINLKVTAVNEIKEKTVVLSTGEEVEADLIVLATGWDKTIGLFNDEVLNRLEVEEDGIWLYKNILPPGIDRMAFIGSNAFTAINPMTHMVQAKWLAALLTNAMPRPSHKEMVLDIWDMRQSRRAITPMNAMRASCIQLHMQSYHDDLLKDMNVNIRKRSGLMGWIEHYFYRITPAGENEVVKRCPPTA

>jgi|Plesta4479_1|7928|GLC49954.1

MPTTVIIGAGISGLQCASAFLRLGHTVTILEREEDVGGVWLRYTGFNAHVPREFFCFPDFAPPPDPDPDLDPEEYPTGRSLLRHVIAYAHRNGLYRHIIFRATVTRLHRLGGQWQCFFDLATGGRGAGGGAGGGGGGGGGGGARGGGGGLGEVQQCGVPQRHQQQQQQQQRISADYVVVATGLHSSPNVPVLDGQHLFRGRLLHVQDLPPNDLELGQQFGGRRVVVVGGTKTAVDTATRLVEAGAEDTFVVFRQAHWLIPRFPLRGMHFTQLLHSRLTAAMLNPYYDAGLWGRVKGGISRPLRRLFWRTVERSLVRQQHLYGDLLPSMPLDKDLLSGGVVHDGRWRQYLEEGAVHAVRGEPLRLTPGGLILRSGEFIAADVVIMATGHLKDYSFIDSDILSKLGVQRDGLYLYRHVLPPLVRNIAFVGAANSTNNPLTSYLQTKWLVACLQNRVRLPEPHDMIRDLQRQTRWQRRKLPPQADRASMIGIYAHHYHDRLLKDMGLPIRRKKNLVRDCFEPYTAADFADIADVLAGDRRRQQSKDVAAAAAEVTAEVTATAGPAAAAGPPPPPLTTSMSMGLGSSFKLSAASAATPSEVYDVTAAEHSVGFGLMPSMRRTAMAAPSRGSTAAETAAAAAAAVAAAAGADGEGEGDARWAYPPPQESSGPGSYPGGYVAAVGSLPGPGPGPGPGPAAGVGAAGQAAGAGAGPATAPSMLYGARPGTAGAAYSNRLLGTLYEQEGGGAPGLHGRGALATRSGPGQGPGRLGLAPAPALSSSLTPPTPPTPPDTPSQLMSPTSYGAGFRSVGRPAIYVPPSSGRSCLMDAAALALMDGVVGGALLDRPPKPGAAAAAAAEQQQQQQRATSTSGELPTAAAGTGGAADGGGGPAGNRSETAAVAVTREEVAAGAVYGTGRAHTAPAAAQMTQVTSPAAAAAAAAAAAAAAAAAAAAAAVMSRSRRQGSGDTGGEPAASEAAAAAAGAGDGRESSSSEALRRRRQGSLAAEAEAEAEEVMSSRNDAAAASTAASTAGGGGGGGDGGGGDARRPSRRPASARSVSGAPSFVTPALAAALAAGATAAAAGPPPWIESPEISEADGGGGDGGGGGGAACAARAEAAGASDTVRFGPSTEGGSGGGGAAAAAAPPLLRYASAGFVANADDEAASCPSAASSESNNVVATCTRASSSSPHSRSHSASRAGGGGGAAAAGVAGKAASRNLVRLVTTRHAAAAAASGADGASGPHSGGSVGAAASGSIAVRAGSSTAARSAGRVSARRTSDFLDESEDFIGTPPGCFEGSLDAMMSDRGGREGGGGGGARSRIAAASSATASSRRRRRRLTADRSTLDGDDVDEYDDEDAEYDEYDDDDDEYGYMPGGGKGREGQASDGGGGGDGGGAAGASGPDGKVAAVAGDHAGGGTTGAGRIGAATASAAGYKSPFALQPSASFGPGTTAAPALTRSASSPRSNGSPPHRSGTGTRFAAAADGGGGGGARRTREVHPTRMSGDSVADAGNRPGGGGGGGGGSQLRSYGTAPAAAPPGGGGGGGGGGGGGGGSVNNASSALERYAARMTAAPTFRMSLTNMADMKPANLNHAARASAAAAAMAAAMAAAGGASSGGGGGAAHHPAPLRPFFLQPSAGGAGGAGGGGGSAAAAAAAAAAASGYVSHHHHTTSLHPHFAATSRLGAMRPMRVSEEEEGDGCGELPPGVVVVGGSTDNGGGGGSGGEADDDDGGGGGGGRLSMSALMVVGGGDDDGGVNQSCASNASFTAGGGGGGGGGGAAAAAPRRSNTTAGYGSSMQSEGYGRTPNVASVSPAFGRGGGGAAAAAAGGGMLSSSPFGTHVSATNALPHTAGGGGGGGGFDGGGSWDMTLRQLSRAVVAPPQRAAGVLRAALISRRNLASGGDRPSESGLSNPNVGGNAGGSFFNARRAGFADDGSSGPVSRGSAAAAAAAAGGGGGGGIPDAQLMSVLLAPGGGGGGGGPPTGTGSGSGPVLLGAGVQPSGPAMAPVREPRDASRFVAAMSTAAAAAAQCPPPPSDAAAPLAKSPRGSGDGGGGAATAASQNGAGGAADAADADAGGAFRRPATTDGGGSSSGGGGSGFVDDIGEIGGGGGGGARMHWSAAVAAAKELRLQSESARSAFAALAMAARARRSCTMYGAPNPSPAHESPMLAKLIAASQSHNGVASDGAAAAVPPHAAVHAAASSGGGGGAAAAAAAATVPRRATSPNVVVAGGRLSLLGNNSGGGGGGGDSRTSWDSGGGVAKSLKRLGGLLMAVPSSPPPSAVQLAAATTTGNKSQTGSVADGGGGGGGSRLPSGSSEGGGVTPARLLRRMMSSSKRRSNEGAYAGGYIAMVEEDNEAVALPPPPPPVAVTVAAFAGGGGGGGGSGGGSCASTAVVSPLEAPPAELAAALFGRTTSRRSVVLYNPPVAAPLELYASAPAAPAAPPTG

>jgi|Plesta1363_1|12791|GLC39407.1

MPTTVIIGAGISGLQCASAFLRLGHTVTILEREEDVGGVWLRYTGFNAHVPREFFCFPDFAPSPDPDPDLDPEEYPTGRSLLRHVIAYAHRNGLYRHIIFRATVTRLHRLGGQWQCFFDLATRGRGAGGGAGGGGGGGGGGGGGARGGGGGGLGEVQQCGVPQRQHQQQQQQRISADYVVVATGLHSSPNVPVLDGQHLFRGRLLHVQDLPPNDLELGQQFGGRRVVVVGGTKTAVDTATRLVEAGAEDTFVVFRQAHWLIPRFPLRGMHFTQLLHSRLTAAMLNPYYDAGLWGRVKGGISRPLRRLFWRTVERSLVRQQHLYGDLLPSMPLDKDLLSGGVVHDGRWRQYLEEGAVHAVRGEPLRLTPGGLILRSGEFIAADVVIMATGHLKDYSFIDSDILSKLGVQRDGLYLYRHVLPPLVRNIAFVGAANSTNNPLTSYLQTKWLVACLQNRVRLPEPHDMIRDLQRQTRWQRRKLPPQADRASMIGIYAHHYHDRLLKDMGLPIRRKKNLVRDCFEPYTAADFADIADVLAGDRRRQQSKDVAAAAAEVTAEVTATAGPAAAAGPPPPPLTTSMSMGLGSSFKLSAASAATPSEVYDVTAAEHSVGFGLMPSMRRTAMAAPSRGSTAAETAAAAAAAVAAAAAAGADGEGEGDARWAYPPPQESSGPGSYPGGYVAAVGSLPGPGPGPGPGPAAGVGAAGQAAGAGAGPATAPSMLYGARPGTAGAAYGNRLLGTLYEQEGGGAPGLHGRGALAIRSGPGQGPGRLGLAPAPALSSSLTPPTPPTPPDTPSQLMSPTSYGAGFRSVGRPAIYVPPSSGRSCLMDAAALALMDGVVGGALLDRPPKPGAAAAAEQQQQQQRATSTSGELPTAAAGTGGAADGGGGPAGNRSETAAVAVTREEVAAGAVYGTGRAHTAPAAAQMTQVTSPAAAAAAAAAAAAAAAAAVMSRSRRQGSGDTGGKPAASEAAAAAAGAGDGRESSSSEALRRRRQGSLAAEAEAEAEEVMSSRNDAAAASTAASTAGGGGGGGGDGGGGDARRPSRRPASARSVSGAPSFVTPALAAALAAGATAAAAGPPPWIESPEISEADGGGGDGGGGGGAACAARAEAAGASDTVRFGPSTEGGSGGGGAAAAAAPPLLRCASAGFVANADDEAASCPSAASSESNNVVATCTRASSSSPHSRSHSASRAGGGGGAAAAGVAGKAASRNLVRLVTTRHAAAAAASGADGASEPHSGGSVGAAASGSIAVRAGSSTAARSAGRVSARRTSDFLDESEDFIGTPPGCFEGSLDAMMSDRGGREGGGGGGARSRIAAASSATASSRRRRRRLTADRSTLDGDDVDEYDDEDAEYDEYDDDDDEYGYMPGGGKGREGQASDGGGGGDGGGAAGASGPDGKVAAVAGDHAGGGTTGAGRIGAATASAAGYKSPFALQPSASFGPGTTAAPALTRSASSPRSNGSPPHRSGTGTRFAAAADGGGGGGARRTREVHPTRMSGDSVADAGNRPGGGGGGGGGSQLRSYGTAPAAAPPGGGGGGGGGGGGGGGGGSVNNASSALERYAARMTAAPTFRMSLTNMADMKPANLKHAARASAAAAAMAAAMAAAGGAGSGGGGGASIHSAPLRPFFLQPSAGGAGGGGGSAAAAAAASGYVSHHHHTTSLHPHFAATSRLGAMRPMRVSEEEEGDGCGELPPGVVVVGGSTDNGGGGGSGGEADDDDGGGGGGGRLSMSALMVVGGGDDDGGVNQSCASNASFTAAPRRSNTTAGYGSSMQSEGYGRTPNVASVSPAFGRGGGGAAAAAAGGGMLSSSPFGTHVSATNALPHTAGGGGGGGGFDGGGSWDMTLRQLSRAVVAPPQRAAGVLRAALISRRNLASGGDRPSESGLSNPNVGGNAGGSFFNARRAGFADDGSSGPVSRGSAAAAAAGGGGGGGGIPDAQLMSVLLAPGGGGGGGGPPTGTGSGSGPVLLGAGVQPSGPAMAPVREPRDASRFVAAMSTAAAAAAQCPPPPSDAAAPLAKSPRGSGDGGGGAATAASQNGAGGAADAADADAGGAFRRPASTDGGGSSSGGGGSGFVDDIGEIGGGGGGGARMHWSAAVAAAKELRLQSESARSAFAALAMAARARRSCTMYGAPNPSPAHESPMLAKLIAASQSHNGVASDGAAAAVPPHAAVHAAASSGGGGGAAAAAAAATVPRRATSPNVVVAGGRLSLLGNNSGGGGGGGDSRTSWDSGGGVAKSLKRLGGLLMAVPSSPPPSAVQLAAATTTGNKSQTGSVADGGGGGGGSRLPSGSSEGGGVTPARLLRRMMSSSKRRSNEGAYAGGYIAMVEEDNEAVALPPPPPPGGGSVAAFAGGGGGGGSGGGSCASTAVVSPLEAPPAELAAALFGRTTSRRSVVLYNPPVAAPLELYASAPAAPAAPPTG

>jgi|Plesta4481_1|6463|GLC75079.1

MPTTVIIGAGISGLQCASAFLRLGHTVTILEREEDVGGVWLRYTGFNAHVPREFFCFPDFAPPPDPDPDLDPEEYPTGRSLLRHVIAYAHRNGLYRHIIFRATVTRLHRLGGQWQCFFDLATGGRGAGGGAGGGGGGGGGGGARGGGGGLGEVQQCGVPQRHQQQQQQQQRISADYVVVATGLHSSPNVPVLDGQHLFRGRLLHVQDLPPNDLELGQQFGGRRVVVVGGTKTAVDTATRLVEAGAEDTFVVFRQAHWLIPRFPLRGMHFTQLLHSRLTAAMLNPYYDAGLWGRVKGGISRPLRRLFWRTVERSLVRQQHLYGDLLPSMPLDKDLLSGGVVHDGRWRQYLEEGAVHAVRGEPLRLTPGGLILRSGEFIAADVVIMATGHLKDYSFIDSDILSKLGVQRDGLYLYRHVLPPLVRNIAFVGAANSTNNPLTSYLQTKWLVACLQNRVRLPEPHDMIRDLQRQTRWQRRKLPPQADRASMIGIYAHHYHDRLLKDMGLPIRRKKNLVRDCFEPYTAADFADIADVLAGDRRRQQSKDVAAAAAEHSVGFGLMPSMRRTAMAAPSRGSTAAETAAAAAAAVAAAAGADGEGEGDARWAYPPPQESSGPGSYPGGYVAAVGSLPGPGPGPGPGPAAGVGAAGQAAGAGAGPATAPSMLYGARPGTAGAAYSNRLLGTLYEQEGGGAPGLHGRGALATRSGPGQGPGRLGLAPAPALSSSLTPPTPPTPPDTPSQLMSPTSYGAGFRSVGRPAIYVPPSSGRSCLMDAAALALMDGVVGGALLDRPPKPGAAAAAAAEQQQQQQRATSTSGELPTAAAGTGGAADGGGGPAGNRSETAAVAVTREEVAAGAVYGTGRAHTAPAAAQMTQVTSPAAAAAAAAAAAAAAAAAAAAAAVMSRSRRQGSGDTGGEPAASEAAAAAAGAGDGRESSSSEALRRRRQGSLAAEAEAEAEEVMSSRNDAAAASTAASTAGGGGGGGDGGGGDARRPSRRPASARSVSGAPSFVTPALAAALAAGATAAAAGPPPWIESPEISEADGGGGDGGGGGGAACAARAEAAGASDTVRFGPSTEGGSGGGGAAAAAAPPLLRYASAGFVANADDEAASCPSAASSESNNVVATCTRASSSSPHSRSHSASRAGGGGGAAAAGVAGKAASRNLVRLVTTRHAAAAAASGADGASGPHSGGSVGAAASGSIAVRAGSSTAARSAGRVSARRTSDFLDESEDFIGTPPGCFEGSLDAMMSDRGGREGGGGGGARSRIAAASSATASSRRRRRRLTADRSTLDGDDVDEYDDEDAEYDEYDDDDDEYGYMPGGGKGREGQASDGGGGGDGGGAAGASGPDGKVAAVAGDHAGGGTTGAGRIGAATASAAGYKSPFALQPSASFGPGTTAAPALTRSASSPRSNGSPPHRSGTGTRFAAAADGGGGGGARRTREVHPTRMSGDSVADAGNRPGGGGGGGGGSQLRSYGTAPAAAPPGGGGGGGGGGGGGGGSVNNASSALERYAARMTAAPTFRPPTSNTQRAPPPPPPPWRRPWPPPVAPAAAAAAAPPSTRRRCVRSFCSRPPAEPAEPAAPAAAAAAASGYVSHHHHTTSLHPHFAATSRLGAMRPMRVSEEEEGDGCGELPPGVVVVGGSTDNGGGGGSGGEADDDDGGGGGGGRLSMSALMVVGGGDDDGGVNQSCASNASFTAGGGGGGGGGGAAAAAPRRSNTTAGYGSSMQSEGYGRTPNVASVSPAFGRGGGGAAAAAAGGGMLSSSPFGTHVSATNALPHTAGGGGGGGGFDGGGSWDMTLRQLSRAVVAPPQRAAGVLRAALISRRNLASGGDRPSESGLSNPNVGGNAGGSFFNARRAGFADDGSSGPVSRGSAAAAAAAAGGGGGGGIPDAQLMSVLLAPGGGGGGGGPPTGTGSGSGPVLLGAGVQPSGPAMAPVREPRDASRFVAAMSTAAAAAAQCPPPPSDAAAPLAKSPRGSGDGGGGAATAASQNGAGGAADAADADAGGAFRRPATTDGGGSSSGGGGSGFVDDIGEIGGGGGGGARMHWSAAVAAAKELRLQSESARSAFAALAMAARARRSCTMYGAPNPSPAHESPMLAKLIAASQSHNGVASDGAAAAVPPHAAVHAAASSGGGGGAAAAAAAATVPRRATSPNVVVAGGRLSLLGNNSGGGGGGGDSRTSWDSGGGVAKSLKRLGGLLMAVPSSPPPSAVQLAAATTTGNKSQTGSVADGGGGGGGSRLPSGSSEGGGVTPARLLRRMMSSSKRRSNEGAYAGGYIAMVEEDNEAVALPPPPPPVAVTVAAFAGGGGGGGGSGGGSCASTAVVSPLEAPPAELAAALFGRTTSRRSVVLYNPPVAAPLELYASAPAAPAAPPTG

>jgi|Volre3785_1|12283|GIL87019.1

MPTTVIIGAGISGLQCASAFLRLGHTVTVLEREEDVGGVWLRYTAFEVQVPREFFSFPDFACPPELEPEEYPNGRSLLRYIIAYAHKNQLYRHIVFRATVTRLHRLSGQWQCFFDVRTGGPGREAGPGGSAGANGGGGGGGGVVVQQHRITADFVVVATGLHSSPHMPLFDGHHLFRGQVLHAQDLPDSAAGLSQLVSGRRVLLVGASKTAVDTATRVSAAGAEDTVVVYRQAHWPISRYPFRGVHFTQLLHSRLAASMLDPYYDAGLCARAKGWLTRPLRRLFWRTLERSLIRQQHLSGDLLPTLPLDKDLLTGGLVHDGKWLQCLQDGTVRAVRGEPQRFTADGLVLRSGDFIAADVVVMATGHVKDYSFIDADILSKLGVQRDGLYLYRHVLPPLVRNIAFVGTANSLNNPLTSYLQIKWLVACLQQRVRLPEPHEMLRDMQRQTRWQRRKLPPQADRASMIGIYSHYYHDRLLKDMGLRTHRKRNLLRECFEPYTTADYQDLAESAESEGQWQPTPASAPMLAPEMSMALGSSFKLSAEPSAASPSELYDVTPAEHSVGFGFMPSMRRGAAPSRGSTATDTAAAAAAAVAAAGLTENEIGPAEAEARWVTMSGAAVPDHGPAGSTSGPSTAPPILMPSLSYRSGAGQVVSTRLLTLYEQEALNGRVASALRSGPALARVGVSSAALTPPETPMNLMSPVSYGAGFRSVGRPALYVPPSSGRSCLMDAAALAMMDGVIGGALLDNPPNLSPQMAAAAAAAGTATATATVTVTAAGQHPKPLVGSGSSGDVPASTTAPSTASGMRSSTAAAAAAAVVTERALGEMRFGVTRARTAPAATTMSPTMMQGSGSGREPNAGGVTASGGGGGPGATSTSVVAVSAAAAAAAVEIDRGESESTSDEDPTTRSGPLVTEDESSSAEVIAARVLPFDFEASAASGCNGGNADGGGVSHMGGPRGLSAPFMTPVLAAALAAAAGSANMVNHNSVREVSMSTLEVANGWGDGGSDGCGGGRAPHDETEAAATPAGLLAVPPNENVFLPCRLGEEEEEEAPGPSSAGGEAPASNAESNPEFQCRTRSNSRTNSTADGHGAQGGNVGRSTPPPLAPRSLVRLAMAHHQQATTGAAATDAPSTLTADSTISRPSSNGSVHAIAGGAVRIGSSSVGPLRASRRISDFFDESDEFVGTPPSCFDGSMEALAVDRDRDRDRDRDGRGSRSCTVMSLRQRRSQRYPYNGDDTDDYDNEDDDFDEEYVYIGGMDNVNAKLPVSAPQGR

>jgi|Gonpec1|3698|rna5993

MERAAGTDAALIPDPNVQHGKPRTCLVVGAGVAGLQAARHLQRIGYQVLILEASKDIGGVWASNYVGYGIQVPWRTYQFPDFLWPEELRPLSDYPTGQQVQQYIRAYAEHFGLMRHIRLNSKVLRLRWHPGSCKWEAVYCDSAREKFYKVRVDYAVICTGMYSSPFIPRYTGADAFAGKQLHAKDFVDMSIVRGRRVLIVGAGKTALDCVCSLVASRTAASVTMLYRQAHWPMPRSILGIGIRSLLFNRATPAMLPPYYTAGRGSRALDVVAQPLKRLFWKTMQGFVGCKFRITKEMRPRVGLPADLFYGGQVLEDSYTRFLSSDGFTSLRGEINSFVRNGVILQDGNFLPADVVLFCTGYEKTYEYFDGDIRSRLGLQKDGLYLYRNCIPPGVPHLAFVGSEVSTYSNILTHGLQALWLSHVLSGRVTLPCPAEMAADVRAQQHWRRQVMPAQRGRGAVLMLYGMQYHDQLLRDMGAQPRRKGVNLLAECFGSYSAVDYSPLMPQDGPDAELEAAWTASQPSKSQWSHLARMLAD

>Mapoly0122s0026|Mpolymorpha_v3.1/1-622

MGSEELSAGQKITSWLSEFDTVLQDEDVDQILELFDDQECFWRDLLPFTWNIETFESKDEILKMLTENLARVKPRAFVIDGDVIDRPNGLMGSAFKFETSLAWCRGFVWLKGGKCWTITTSMWDLKGYEEKISRSRPFGPTLGQIKGRKTAYQEKQQEQHDMGRSKQPYCLVVGAGQGGVMLGARLRMLGVPAIIVEKNERLGDNWRNRYKSLALHTPCFSDHYPYLPFPSNWPVLAPKEKLADFIECYGKLMDLNVWCSTECKSASYDEKEGHWTVVLERKCGSEVSEVVVHPSHVVLACGLAGYPKMPSFPGKENFQGVQIHSSKFVQGEDYTGKKCIIIGAGTSAHDICLDLWEQGASEITLVQKSHTHIVTMKDFLESRLDPQLEKAELDTNFDTDAFDLKSASLPYKVLPRLNVPIYARIAQKEAELYDKLHKAGFKTFMGEDESGLILFLLRRAAGYYIDMGASELIADGEVRVKNGVTVQAMKENSVVFDDGSEVSADVIIYATGYGNMQEFVEKLISKEAAEKVGLCWGLGSGFKGDPGPWVGELKNMYRPLPVPALWILSGGLSHCRFYSRHVALQLKARMEGIPTPVYKLVRHKEPDSPVLASKMSSIQMQD

>178716|Smoellendorffii_v1.0/1-611

MATMNGSPKIDPDALVDAWLARLDSSLASGDASAAATLFEESTSFWRDLIAFTWNIVTVEGRSQIHAMLQSTLESVAPKGWVRNGTASYSADSGVIEAWIKFETRDAVCTGHLRLMATTSLCRTLLTAMEGLKDFPEKKGHTRPKGVTHGVIRHRASWLDGRKEEERTLGSTVQPYCVIVGGGQAGIGLAARLRQLGVPCIVVEKNPRPGDSWRSRYKSLCLHDPVWYDHLPYLPFPENWPIFTPKDKMGDWLEAYTKIMEINYWTSSECLGARFDPQSGEWEVKILRDRSKEVTLRPKQLILATGMSGFPNVPRIPGQEEFVGDLHHSSKHPGGKAYKGKRAVILGSNNSAHDIAADLWENGAAEVTMIQRSSTHVVRSESLLQFVMKNTYSESAVESGLTTDKADMIFASVPYKIMGDVNRPTYDAIRAHDKDFYAALTKTGFMLDFGDDDSGLFMKYLRRGSGYYIDVGASQLLIDGEIKLRSGVTIASLKPSSVVLSDGTEIAADVVILATGYGSMNQWAAKLISQEVADRVGKCWGLGSNTRKDPGPWEGELRNMWKPTAQEGLWFHGGNLHQARHYSLYLALQLKARMEGITTPVYGIPKVRHLS

>Poptr1_1|eugene3.32340002

MPFPDNWPVFAPKDKIGDWLEMYTKVMEVNYWPSTDCLNARFDEDRQEWEVTVERNGETQVLRPKHVVFATGMSGKANVPKIKGQDVFKGEQQHSSQHHGPDAYAGKKVVVIGANNSAHDICAALWEHGADVTMVQRSSTHIVRSDSLMDIGLGDLYSERAVASGVTTRKADLIFASLPYRIMADFQVPVYDRIRERDADFYRQLEATGFMLDFGDDDSGLFMKYLRRASGYYIDVGACDLVIDGSIKLQAGKQISHLSENAVVLADGTELPADLVVYATGYGSMNGWVADLISQEVADKVGKVWGLGSDTTKDPGPWEGEQRNMWKPTQQEALWFHGGNLHQSRHYSLYLALQLKARQEGLPVRVYDLQQVHHLXX

>jgi|Mesvir1_1|21105|Mv12382-RA.1

MAKEWIAKFGRALESGDTSAASSMFADDCYWRDLVCWTWNILTCEGKGEIKAMLDARLKATGPSNWEISSESTGADGVTEAWFTFETILARCNGVLRLKEGRCWIMLTTMQELKGYEEKRGPTRPAGVVHGAIKGRRSWLELKAEEESKLGHSDSSQPYVLIVGGGQCGIALAARLKMLNVPAIIIERNARPGDSWRRRYRTLCLHDPIWFDHLPYLPFPDHWPVFIPKDKMGDWLEAYTSIMELNYWGSTACTHAAFDDSNKEWVITVERHVGPASSSNSGTGHPGTGSTPPGDASPPAKLVLRPKHLVIATGMSGFPNIPSFPGKETFLGEQCHSSQFTGEGEYAGKKCVVLGANTSAHDICAALWEQGAASVTMVQRSPSLVAKSETLFDVTMSPLYSEKALAAGISVDKADMILGSLPYRVMPFVHMPLYEEIQKRDAEFYKQLAGAGFLLDWGEDASGLFMKYIRRASGYYIDVGASEMIIDGRIGLKSGVNIAAVREKSVVLTDGTELPADILIYATGYSSMNDWVAQLISPQVADKVGKCWGVGSGTAKDPGPWEGELRNMWKPTAQEGLWFQGGNLYQSRQYSLYLALQLKARHEGLPDVEVYGMKPVHHLR

>jgi|Mesovir1_1|1813|Mesvi1423S02809

MAKEWIAEFGRMLDSGDTSAASSMFADDCYWRDLVCWTWNILTCEGKGEIQAMLDARLKATAPSKWEITSESTGADAVTEAWFTFETGLARCNGVLRLKEGKCWTMLTTMQELKGYEEKRGPTRPAGVVHGAIKGRRSWLELKAEEESKLGHSDSSQPYVVIVGGGQCGIALAARLKMLDVPAIIIERNARPGDSWRKRYRTLCLHDPIWFDHMPYIPFPDHWPVFIPKDKMGDWLEAYTSIMELNFWGGTRCIGAAFDEGKEEWAVTVELDAKKAPSTAHSITGHVAAGGSHFAAPDSTLTTLVLRPKHLVIATGMSGFPEMPLFPGQESFHGKQWHSSQFNGEGDFAGKNCVVLGANTSAHDICAALWEQGADSVTMVQRSPSLVAKTETLFDVTMSGLYSEKALAAGISVDKADMILASLPYRVMPFVHMPMYEEIQRRDAEFYKQLAGAGFLLDWGEDASGLFMKYIRRASGYYIDVGASEMIIDGRIGLKSGANVATVKEKSVVLTDGTELPADILVYATGYSSMNDWVAQLISKQVADKVGKCWGVGSGTAKDPGPWEGELRNMWKPTAQEGLWFQGGNLYQSRQYSLYLALQLKARFEGLPGVDVYGMKPVHHLR

>jgi|Auxpr25_1_1|888|APUTEX2500004442-RA

EGVLSGTSPSDTVRAWLSSLDSALRARDAVTAASLFEEEGLWRDLVCMTWNLKTCEGRGEIAAMLGACLDQASPRDWTLEGEATAASSPVTGDTVEGWIRFRTAALECKGYLRLRAGRAWTLLTAAQGLVDFPEPSGANRPMGAEHGARRNRTTWLDERQREARELGRSVQPYAVIVGGGQGALALAARLRQARVPTIVLERNARPGDSWRRRYKPLCLHDPVWYDHLPYLPFPDTWPVFTPKDKIGDWLEMYAKVMEINYWASTTCKKAEWDEAAQEWAVHVTRVGEDGAAEDITLRPKQLIMATGMSGRPSMPIIPGAETFQGDYHHSSQHPGAEPYCGKDCVILGSNNSAHDIAADLWENGAKSVTIVQRSSTHVIRSQTFTDLITGPLYSEEAMKAGITTEKADMLFASIPYKVLPAIHRAVNEVVKERDADFYAALRAKGFLLDFGDDDSGLFLKYLRRGSGYYIDVGASGLVLSGEVSLRSGVSIARVNPRSVTLTDGSELPADLVVLATGFGSMNGWAEELISKDVADRVGRCWGLGSGTTKDPGPWEGELRNMWKPTAVPALWFHGGNLHQSRHYSKYLALQIKARYEGLPTPVYGQAKVPDWAHSKLERRANKAVLFLQQLTTIQQTGEGLKVPTMPKSAVVSAEGIAIKYKSKYGALLTRTTERGFVIRKLTGPGGEIRWSAPVFLQGSAMGAGLSGGYRKSRTCIALANDQAVQSALRSQSEFGPCVHFLLDMNGTRIKRAHFDSSDQASQTAVSQDRHGGMTAKYYQVDAAMVDISVEGGSTYVDKDFNAAVYGQGVTAEGILSGEVPPPAEFQLLYTVLQQFVDETPSPVVKRELGASGHGARSIRSVRSINMKSSPEEGTALPGVALQRRTSATPSVAGIKE

>jgi|Ostque1|9343|CAD7697881.1

MIGARLARLGVPAIILERRARPGDSWRHRYASLATHDPVWMNHLPYIPFPSHWPTYFPKDKLADWLEMYVKVMELNYWGGSACTGAAYDEGEGVWRVTVDKGGAEVTLRPRQLVLATGVHGAPFVPDFPGAASFKGPQVHSGNYKGDEEWEGLRCVVIGSNNSAHDICHDLWERGALPTMIQRTSTHVTRSAVWQDMYLKPLYSEEALASGMTTDKADMIFASIPYRVHAVLDVPTYTAMAKRDAPFYKRLTDVGFLLDFGEDGSGLFMKWLRRGAGFYIDTGASDLISSGDIGLKSSVGVDSIGEASVKLTDGTVLPADVIVYATGWASLSDSLGKMTSPEVAGKVGRCWGLGSNTTGDPGPWEGELRNMYKPTAQRGLWIFGGSFMQCRFYSQFLALQVKARMEGLHTPVYGGPEVATAN

>jgi|Ostque1|10765|CAD7699277.1

MSAPTHEEQRRAWLDAFASALERRDVDAAASLFADECYWRDLVAFTWNLVTLEGRDAVAAMLTATLPAASPRGWAAEGDAIESAEGVVEGRFNFETGAARCKGHVRLRGGRCWTLTTTMDELIGHEERRGRRRETGRCTAAGTKSVGEVGDPGWQPYCLVVGGGQGGITVGARLGVLGVPVLVVDRLERPGDAWRRRYACLVTHDPVHADHLPYMPFPDHWPVFSTKDRMGDWLEAYATVMGLNYWGSTECRNAVYDEEGQRWTVTLNREGKEVKVYPTHVVLATGLSGAPKVPEVPGAATFVGLQQHSSEFSSGKGWEGKRCVVVGAGNSALDICGDLWAHGAQVTMVQRSATLVVQSQTLTGRMLGKWYSEEAVEAGVTHEMADFAQSTVPYRLMPARSKVLFDEIRAANAEFYDGLSKAGFLLTFGPDGSGMPMRYLRRAAGSLIDTGNSELIASGKVRLKSGVEISNAEADALVLNDGTRLPADLVVYATGYGNMSEVVAKLLGQGVADRIGRVWGMGSGLDGDPGPWEGELRNMWKPTRQPGLWIHAGMLALSRTYSRYLALQIKARFEGIPTPVYGLAEVHHKR

>jgi|Klenit1_1|804|kfl00109_0340_v1.1

MATQTLTEDVRAWLERFSAALEESDLEKVLALFGDQCFWRDMVSFTWNVKTAEGKDEIRAMLEATLPSAAPASWRVEGEAMEMFGAVTAFVAFETATGRGKGHLRLVDGKCFTLLTTLYELKGFEEKIGASRLPGRNLAPEPNRQSWAAKRELERGELGYGKQPFVVIVGAGHSGVMLAARLRALNVPTIVVDKGSPGDSWRNRYGSLHLHTPSYMDNFPYLDYPAGWPTYPSKDEMGDWIEAYVKIMNVNVWSHSSVESSTFDDESKEWAVAVTRPDGKTLTLRPKHLVLCTGMYGDPRVPDFSGAEIFGGSQHHSSRHGCGGADFAGKKCVVIGTGTSGHDVAQDLWEKGADVTMVQRSPVFVIRSETLAAVYVDPLFHDSSPMSQQHADLLMVSTPYRLVAPFQAPMVEEIKRRDGDLHERLRERGFRVHFAEDGSGLAGLLNRTATGYYYNMGASYLIASGEIKVRSGGVEKVTERTVVLTDGSQVPADVIVYATGSRPVSEVVARVTSPEVAAKVGPLWGYGSGLDGDPGPWEGELRNIYKPTAQEGLWFHIGGLVQTRFGSLLVALQLKARFEGLPTPVYR

>Sphfalx02G048800|Sfallax_v1.1/1-665

MEPMIGRKMVAIIGAGPAGLVAAKSMLEVGLFPTLFDKLPDLGGVWNPQSSSCWFSLRTNDSRYSCCFSDFPWDSDSPIFPSQHDVYGYLQAYANKFLQPVHFCLGSEVTNVSRSNNDKWLVQWSSLEQKYQGSKAGSQIFDFVVFASGFFSIPYTPSIQGLDGFPGEVMHSSEYRSPERFRGKRVVILGASFSSAEVAADLAPLATEVINIVSRPFWVLPRYIALDPDSASTPFFPLDLVLYRRSTRQSQGEEVFANEEENRKANQYFRKLCGDQGPLNLQQDTDPPIIAISDSYLELVRSNHIMVRHGKVQEVQGQTVILEDQTTIPEVDCIIFCTGFQSSMPYLSSNVLDTLSFDPADRFVPFLLHRSTFHPSLPGSAFVGMYRGTFFGVMELQARWAAAIFSGDLPPIPESVQQEGIAFEKWIKSQTPRPQFPHGDYVGFADTFAADLNVLPSKDWYQKHDISIPAHFRRGSVAEEAVEVLEKACKEFLDGKMVPHAVFRSLAGSWTLHRHIENRIDFANSGEVKGTATFNVTELSHEYLYTEVGELSMQRGQHFRVHRKYVYVYDTHTNQIDVFFENQGSRGSPFHSLQFLSPKPSSATEKGGIEMSGGWRAAGQHLCGEDLYNASYRFSFSGNSIAQMEISYSVTGPKKDYTSTATYFR

>jgi|Gonpec1|8977|rna9192

MWGDPSATYESLQTNLSRYTCSFSDFPWPPTSPDFPSAVDVSNYLASYRDAFLAGDNCAMALGCEVLQVLRSPQGPGWVVEWRYAGPSSGGGEPATPQSREFDFVIVASGYAAAPFSPQIPGLETFPGLVIHSKDYRGPSSLTGRRRVAVVGGGHSAVDIAADLAAGVSQGTAAPPSAQAGDTAVHAAAAAASTSVVHVLPRPFWVIPRYLPSDPQSPHPAFLPLDLQLYRRSRRQGSGERLFPTAEHHRQANTYFHSVCGDQGARLSPCLAIPPDCSEPAIVAVSDTYGVLAASGRMQLRRASLQRIHGASLELSDGGPPLDGVDAIVLCTGFRPRMDFLPADVLATLSYTPDDRYVPLALHRGVLHPDVPGLAFVGMFRGPYFGTAELQARLAAAALSGALPAEADAAQREGVAVELRLRAQSPRPVLPHGDYVGHADSLAAALGVLPPPEWRAEHDVVVPAHFALVPPPRPAPPPHAQREQEQQSGEVPPGADQGADGAWEAEAARRAVANVESALRAYRGGRMVAGAVFRALHGEWALHRRIDSRMASSPSGTVTGRASFVMTDAPGGGADTAQYLYQESGQFTIDGGGSMRVRREYVYSYDKAVDRIDVHFAEGGSRGGFFHSLRFLPPASAGEQPGGLQCEAAAAPPGADTDTSAGAGVGPCRTGGPAGCGTGVCGAGSTGCSPAIGALETAVVEQGEGGAGASISGDGWRAVGDHLCIRDMYRASYRFAFQGIQLREFEIQFRVSGPQKDYTATALYTRRK

>jgi|Plesta1363_1|1829|GLC41090.1

MPHARREPSTTTPQPKSVAVIGGGLAGLSAAKAAVQAGLLPTIFDRRPSIGGMWGDAAGATYASLHTNVSRFTCSFSDFPWPPGSPDFPTAAQVGDYLSAYQRAFLPPDKCRLRLGCEHAGDPPRTEQTRTEQQQQLPWVVEWHEAQPSPGASAPTHGSSHTQHFDFVVVASGFCAAPRIPSIPGLDSFPGLLLHSAAYRGPHSLRLAAAAAAGAGRRCRRVAVVGAGHSAADIAADIAAAGGGTGAGGGTAAAAAPEPASDPDASVMPMEVIHVTPRPFWVLPRYLPLSLDAAAPPFAPLDVMCHRWSARPGCEGAENDKGRERLFASREERVKDNAFWATVCGDQGALLSPALAISPGSSEPMVGSITDCYGPLVASGRLKVLRGRLHRVEGSTLELSGGPPGGGGGGGAAAADSAVAAAAPPPAPLRDVDAIVLCTGMRPSLSFLPAEVLETLSYTPYDDYVPLALHRQVLHPDVPGLAFVGVFRSPFFAVVELQARLAAAAAAGTLPAAEADPAAQRAGVDLELRVRQYEPRTPYPRVDFVGHADGLAAALGVRPPAEWRAAHDVIVAAHYGVFPGPSAAANSTTTAETQGGEAAAAARTTAAVDDDDGDASAAAAAAMGDAVAAAPEGVRRALDDVAAAMRSYRGGHRVASAVFHDLAGEWALHRRIVSRLPSSPSGEVTGRATFTATAADTSGVYSPAAGGAGGLSYLYEEQGQFAMLGGPVMRVRREYVYAYDSTADRIDVFFAEGGSRRGAFFHSLRFLPPEEQSSSAAAAAAPAAAAAAQPAAATSAEAAAATAAADVAATDAGGASAATASSPASPAAGAAAAAAAAAGWRAVGEHLCVRDMYDASYRFSFQGLRLRQFEIEFRVKGPNKDYTATATYTRQPGGGRIPYEGC

>jgi|Plesta4481_1|2164|GLC71143.1

MLLSSFQRPLHPQTVSTKNNNFAPIHFQHQGRQLSFILQRARSSHSRATRASAMPHARREPSTTTPQPKSVAVIGGGLAGLSAAKAAVQAGLLPTIFDRRPSIGGMWGDAAGATYASLHTNVSRFTCSFSDFPWPPGSPDFPTAAQVGDYLSAYQRAFLPPDKCRLRLGCEVLRVLRPSPPPAPPPPPPPPLPPRTPETQHAGDPPRTEQTRTEQQQQLPWVVEWHEAQPSPGASAPTHGSSHTQHFDFVVVASGFCAAPRIPSIPGLDSFPGLLLHSAAYRGPHSLRLAAAAAAGAGRRCRRVAVVGAGHSAADIAADIAAAGGGTGAGGGTAAAAAPEPASDPDASVMPMEVIHVTPRPFWVLPRYLPLSLDAAAPPFAPLDVMCHRWSARPGCEGAENDKGRERLFASREERVKDNAFWATVCGDQGALLSPALAISPGSSEPMVGSITDCYGPLVASGRLKVLRGRLHRVEGSTLELSGGPPGGGGGGGAAAADSAVAAAAPPPAPLRDVDAIVLCTGMRPSLSFLPAEVLETLSYTPYDDYVPLALHRQVLHPDVPGLAFVGVFRSPFFAVVELQARLAAAAAAGTLPAAEADPAAQRAGVDLELRVRQYEPRTPYPRVDFVGHADGLAAALGVRPPAEWRAAHDVIVAAHYGVFPGPSAAANSTTTAETQGGEAAAAARTTAAVDDDDGDASAAAAAAMGDAVAAAPEGVRRALDDVAAAMRSYRGGHRVASAVFHDLAGEWALHRRIVSRLPSSPSGEVTGRATFTATAADTSGVYSPAAGGAGGLSYLYEEQGQFAMLGGPVMRVRREYVYAYDSTADRIDVFFAEGGSRRGAFFHSLRFLPPEEQSSSAAAAAAPAAAAAAQPAAATSAEAAAATAAADVAATDAGGASAATASSPASPAAGAAAAAAAAAGWRAVGEHLCVRDMYDASYRFSFQGLRLRQFEIEFRVKGPNKDYTATATYTRQPGGGRIPYEGC

>jgi|Volca2_1|5804|Vocar.0002s0558.1

MPRARVDPTPQAHAEPDPQHRKTVAIIGGGLSGIAAAKAAVQAGLFPTIFDRRPALGGMWTDGAATYDSLHTNVSHFTCSFSDFPWPSSSPDFPSAREMGQYLTAYQHAFLTQDNCLLQLGCEVLRVEPNAPLLKCLGSGGDGAGQAGPSWSVHWRHVAHPRPEAAVSPSLGAVATRRFDFVVVATGFCAVPYVPDIPGLATFPGLVLHSADYTGPSCLPGSCRRVAVIGSGHSAADIAADLAAAAAAGRSAAAATVDSSLKVVHVTPRPFWVIPRFLPLEPTAAAAAAADGGGGGSPFVPLDLFFHRRAARAAGGLGPEELVFPRGHDNAGVNSFFASLCGDQAKLLSPVLAVSPSSAEPAVSSISDSYGPLLVSGRLQLRRARLHRVAGSTLEFTAAAAAAGDEQPDSGGGGGVAVAAEAAEAAPQPLHDVDAIVLCTGLRPALPFLSTDLLSTLSYTPNDNYVPLALHRRVLHPDAPGLAFVGVFRSPFFAVVELQARLAAAAFTGTLSAAEADPETQHAGVALELRVRQHRPRPPYPHQDFVGHADGLAEALGALPPAAWRAEHEVVVSAHYAVHMFQRPTQPPPPAPPPSSSRTASAFTAATAAAAVAAAAAANGHGDMAAAATASEGLELDAIPTSAPPASADGETAAAAAAVRQALTDVAAAMRAYRSGRRVASAVFYALAGEWALRRSIISRLDSSPSGEVTGRASFAAFADADFTPCAGGAGGLSYLYDEQGQFAIHGGAVMRVRREYVYAYDSTADRIDVYFAAEGSGSPRGNFFHSLKFLPPCDGADGGGGHHLASTAGIADTADGAGCWRAVGQHLCGRDMYDASYKFYFQGIQLRQFEIVFRVTGPSKDYTATAVYTRPYV

>jgi|Volre3785_1|1640|GIL77707.1

MAVAMPFVPAEPTPQQRKTVAIIGCGLSGLAAAKAAIQAGLFPTIFDRRRAAGGMWTDGSATYDSLHTNVSRFTCSFSDFPWPASAPDFPSALEVGQYLSAYQRTFLTEENCRLQLGYEVLRVAPSSPDPSLSLSHPAADSSQSPPWRVEWRKVHEPTGLREDCAASADADDSEVDLLQNPESRPTQEQQTSATPASSHTLQAPSRDTAGGSMVSQASTASLGSSDQFDYVVVATGFCAVPHLPDIPGLDTFPGLVLHSAAYRGAHLLRGRRRVAVIGSGHSAADIAADLAAAALRTAAVAVRSPGGSCSSGDSTAAAAATDVPPPQTEVNSGMEVIHATPRPFWLIPRYLPLAAAAATDANGASGAAPPFVPLDVFFHRWSGRVASGRGPEEQVFPTSEDHIKSNAFFSSLCGDQGKLLSPVLAVPRVTSEPVGVAISDSYGPLVASGRLKLRRGRITRVSGCSLELSDENAAYVASASSGSGSGGSDGGNNLSQPLHDVDAIVLCTGLRPALKFLPDELLSKLSYTPYDNYVPLALHRRVLHPDAPGLAFVGVFRSPFFAVVELQARLAAAAFTGALPAEEADPATQYAGVAMELRVRNHTPRTPYPHQDFVGHADGLAAAMGLLPPPGWRTKHDVVMAAHYAGAVLGPKPQPAVAALPRGHGPAANVPGGDAPTAAAATVDGQQGGEAKAAAITEEEEPRTEVPPAVAAVDAVQRALDDVEAAMGEYRHGRRVASAVFYALAGEWALRRRIVSRFPSSPSGEVTGRARFTTIADADFAPAAGGAGGLSYLYDEQGQFAVQGGPVMRVRREYVYTYDNTADRIDVYFADASPRRGGFFHSLRFLSPEGPQNDEDGSSSSSSSGSGGDGCWRAVGDHLCVRDMYDASYRFSFQGIQLREFEIEFRVKGPNKDYTATATYTRP

>jgi|Volafr1|21367|GIL49855.1

MPCSNGVRQRPDPGRTILLGNVSRNYKHYCSYIFLRSRSVHSPPLMKWAAPMAVAMPFVPAEPSPQQRKTVAIIGGGLSGLVAAKTAIQAGLFPTIFDRRRTVGGMWADGSTIYDSLHTNVSRFTCSFSDFPWPSSSPDFPSALEVGRYLSAYQRAFLTEANCRLQLGCEVLRVAPSYPDQQLPVSQPAVAISQSPPWNVEWRKAHEPAGIAALADKDDPVSGLLEDFELGPKNQRQAPNTPDSLHRLEAPYECTAGRAMGSQASAASRSCSDQFDFVVVATGFCAVPHLPDIPGLDTFRGLVLHSAAYRGAHLLSGRRRVAVIGGGHSAADIAADLAAAALRAAAVEARSPGWGGPVAAAACTAAPAAAAMTDVASGGALAEIDPGMEVFHVTPGPFWVIPRYLPIAAGNASCAAPPFVPLDVFFYRWSVRMASGLGPEEQTFPTGEDHAKSNAFFATLCGDQGKLLSPVLTVPRVTSDPIATAISDSYGPLVASGRLKLRRGRLTRVSDSSLELSDENAASVASAGNGSGSGSGNGSGNSPSQSLHDIDAIVLCTGLRPSLTFLSDGLLDKLSYTPYDNYVPLALHHRVLHPDAPGLAFVGLNRSPFFAVVELQGRLAAAAFTGALPAAEADLAAQRAGVAMELRVRQHTPRTPYPHQDYVGHADGLATAMGLLPAPGWRTEHDVVMAAHYAGAILAPEPPPPPAAALRGGLGPDADIAARAALTAAVATVDGQQHGDVAKAAAIAEVPTAAVVTVAVADAIRQALDDVKAAMGEYRLGRRVASAVFYALAGEWALRRRIVSRLPSSPSGEVTGRARFTTIADAHFAPAVGGAGGLSYLYDEQGQFTIQGGAVMRVRREYVYTYDSTADRIDVYFVDAGMRRGGFFHSLRFLPPEPPQNDDDDGGGGGGDGCWRAVGDHLCVRDTYDASYRFSFEGIQLREFEIEFQVKGPEKDYTATATYTRSLR

>jgi|Chlre5_6|5911|Cre13.g603176.t1.1

MTLLSARTFGPAKQTGAELARHPAPQLVHGPCARHRPWALGARLSLARPAVASPHAAADAYIDEYKLAGLDLADEAQQQLRSWYLQELLRQEEQRQQQLLAAMTPPATAAATAAAAAAAEGVTGPRMKELPAAPVSSRTTNAKVTLLYDLEAIKPFTQQAPSAAPSLSDTASHADTPASASAAAAASSRGGGAGSARGGGQSRLAAAGLVPSSSPDLVAALDQLVRTLAEYGAVHAVHLFLRESTLAKAPALRKQVQQLCTWVEPEELLAGAADPPAAARGAASRRPPSTSPSSSSSSSSLRTSTTSTSSSGSTSSSSGGAAAAAAPATPHDCPLCRTCCATRLDLLSHFATTHQAPVLAATAAADPRARIMWTRGRPLSLTLPQPMEAAEAGAGQAGAGQAGAAAGQQLQVSPQDFVFAREALQRGRYLDPLLAAAPPTTLSIAADQLQPPTQAQHGPLQPQHGPLQPSPQHRLQQHRPQQRPQQEQQQQAVSPDEALGGPGLFLHVLPAGQPGSALPGALMRVAQRLAQQAEAEAETLLPAAAAAGQAPRRGRGALLRGRGAGRRLAMAEAAAAGAGPPRYLCVVSDGEELDQAVASLKRPGVGVIAVTRTTRVPSATATLRWWAVQGGEYRLRRKQLDNSSKAGKARQFYMIAPKLLKPSPPRLHARSARVCVTAQMRKYELKPVAAAKSVAIIGAGLSGLVAAKSALEAGMLPTLFDRRGSIGGMWSTERSGMYDSLHTNVSRYSCVFSDFPWPEGAPDFPTPGELGAYLSAYRSTFLPPDKCPAQVGVEVLRVEPAGSPGGDGGGTGAKTGWRVQWRPLSPDADGSPSPSTCGHDTHDKQEQPPIQSAEFDAVIIASGYAAVPAVPDIPGLEAFAGKVVHSLHYKNPHEFAGLRVAVIGAGHSALDIASDLALASAACAPPSAAAEAEAAPAAAVGPAGVVHVFPRPMWVTPRYLPDSSAAAAAAASGTAAAAVAPPPAFLPLDMQFYRRSRRAGPEEVAVPNAAQRQKGNGFWASMCGDQGALISPLHAIAADTTEPPVIGICDSYVPLAATGAMHLRCARLLRITGPSTLELSDGGPPLSDIDALVLATGIKPDLSYLPHDVLEKLSYTPDDNYLPVAAHRGVTHPDVPGLACVGMYRGTYFGAVELQARLAAATLSGRAPPEPEEVVRAGVAAELAIREQTPRPVLPHGDYVGHTGGMAAALGVLPDAAWRAAHDQVVPGDFGLVPPSWEQQEQQAGTQAAMVKDAAAAAEAAMVAAVKEAPAGGEQGGGEPAGHVVVAGRPAAAYAAAAAAVAESHASLARFAGGRMVARAVFYALAGNWRLRRTLVSRMESSPSGVVTGAASFLPKRPDADAAANGSGSSNGSSGGSSSGGGSSRVRLGSMPFQYLYREQGQFAIQGSGSSSGSSSGSGGISSGGAVMRVSKEYVYRYDSPSDRLDVFFADAHGVPGGFFHSLRFLAPAEGAPGPQHFQREEEPAADDAADDSGCWRAVGEHLCVRDMYYSSYRFWFQGVHLKKFQIQFVVQGPNKDYTSTATYTRQ

>jgi|Mesvir1_1|12953|Mv17927-RA.1

MLSTARLLSGSVSSPYATVQLTPRQCPTALWESRQQGKYHLHWQRQVGKNLCPAWASRKFGAQTSLHLQSRDGPLCSPATPRRIVCRAEAADAVDMAPSKSIAVVGAGLSGLVAAKSLLERGLLPTVFDAAEKVGGMWNPEAHPGCQRWPSMRTNVSRFTCSFSDLLWPPGSPTFPSQPEVGRYLVRYAARFLSQPGVTFSLGSRVTRVAPRPDGLWDVHWQVTDASGNREQQRAVFAGVVVASGFFAAPRLPPLPPPSRGAATFQGTLLHANDYRGATGVHAAPFAGKRVAVVGASLSAVEIAADIAVSGRAASVTNVVPHHAYVLPRLLPDDTGSAAPAFLPLDLLLYQRKEPGKGGAQLGKGVAGQGGGEEGAEEGAEAAHAYFGRILPREEDVLAALKHKEGDVPPPPYVGICDHYAGLVRGGAITLLPGRMVGVAEDGRALLVDATPAPAAANSTTATSPPSPPVHLDVDTVIFCTGFGPSLDFLQPAPSHADKPGEPTASAADAVDPLVPLAYDPDDPLLPLLLDADSVFHPTLHGLAFVGMYRGVFPGVVELQARLVADVFAGAIPHPYANDKGAAAASLSRAQALRDTHPRPQFPFKYVEHADVLAARIGALPPASWPLATNYPVTPLQYRMAVGDAGAQPRREMAFAGLGEARTADVPFLATQPTRAPSPLSLGEAFRDLEDELAAYRAGKGVARAVFRGLGGKWCFRRRLVSRLDASPSGVVVGEASFKPQPWPLMAEAWVVEPPAETASGQGDAPVAGQGRAWDVLKPSWSAHEDPVVAREQAAMPACAATGAGTGGVSGRGATAANPVLDYLYQETGVFRTSSGLELNVKSSYVYTQEGEGDTLELFFAAPPGAQATNRDPRIVQGDQGPIMVRGRPFLSLRPSRADPGSGWEADATHLCGADTYVARFRFAFEGARLREMEIHFHVSGPHKDYDSVTLMRRGPVSELAFQQGISFADALE

>jgi|Grachil1|4236|Gchil4728.t1

MMSSDGLLGKPPTTRICVVGGGISGICAARACLLSGLNVTLFEKRSKLGGIWSLENSPVFDSLSSNTATFEAGLSDIPPLRVFPSSSLGKYDDLCCTSQEMADYIQLIVKKTPRFFESTKLKTNVLSVTQTPSKTYCVSFLSDGTRAQESEFDKVIISTGYFNTPFVPPASELPGIDKYRGKVLHVSMYKNPSQLKGKHVLIVGGSVSGCEAAGDMLAFASSEGPTSVTLNTRSMRFLLLKQHKGEVLLSKLGTRFQRFRDLAGRFENEQYSQTMSSFIPQFLSNSDYPAPEPDGPIKIPYVPSWVPVSGKLYEACQKKKLQWKVSSLECITENGVRFRDGTEGEYDVIVFATGYRLHLPFLEKDVQQTICAGNGVNLLDLYDYTFHPEVPNMAFVGMFSPATSIIPIVDNQSRWVARVFNGDVQLPSDEELNHGVQLYRQTRSSPNSRFVVHGFDVLDVFAKHGDFEVNLAKFPQLCRALLFGPMIPAQFRLFGSEHSEEALEETMKQFEAAGFKIGDNVVSSEIYEELRTICHILDEKGICPSSLPVALGHLVVSKT

>Pp6c18_10300|Ppatens_v6.1/1-540

MSNLMTEFDAVIVGAGIAGLYQLYRLRNLGLRTHVLEAEKEVGGTWYLNKYPGARCDIKSIMYSYSFSPELEQEWNWSENYSNQPEILRYINHVADRFDLRKDITFNTRVTGALYNESSKRWEVHTDQGKTIRTQFLIMATGCLSTPKQPEIPGIEKFQGSSYYTGRWPDKPVDFTGKRVAVIGTGSSAVQSIPIIAQQASQLTVFQRSAACCVPGLNYKLTADEIAASKASYSTLRKAQRESPGGFVDERGSKSALEDSEEDRQARYEMLYQRGTLDAIIVCAYNDLVINKEANDTFSEFIRQKIRSIVKDPVVADSLSPRGYAVGTKRICLDYGYYETFNLDHVHLVDLTKTPILEMTEKGIRTSEKDHVFDAIVYATGFDAITGTLTRIGIQGKNKLLLKDKWAKGPRTYLGIAIAEFPNLFTITGPFSPSVFTIMTVAIEQHVDWITDCLKYLREHGVEEIEATVEAEDAWVEHSLAVANMTLFPKTASYYTGGNVEGKPFMLYPYVGGLANYRQVCDGVAAKGYEGFKLKTKGIV

>jgi|Cyacal1|1570|CDCA_CDCA15G3951.1

MGTQPAKEAHSASTNGAVEVDALIIGAGVAGLYQLYQLRNEGLRVLAVDTAADVGGTWYWNCYPGAKFDSESYVYQYLFDERLYKDWTWSERYPSQPEIERWMHYVTERLELRRDIRFGTTVTSAVYDEARGRWMVSTDRGGTFDTRFLISCGGMLSAPLEKRFPGQERFSGRIFHTARWPREPLDLAGKRVGVVGVGATGIQVIQTIASEVAHLKVFVRTPQYVVPMKNPKYGPREEKAYKARFDELRDLLPRTHTGFEYEIKHRWADLTPEQRRAVLEETYGDGSLRAWLGGFLEIFTEPEVSEQVSEFVREKMRARLKDPKLIEKLVPTDYGFGCRRVPLETRFLEVFHRDNVEAVSVRDNPIVEVVPEGIRLDDGTVHPLDVIVLATGFDAGSGALTRIDIRGRDGRSLRDDWSRDIRTALGMMVHGYPNMFMTGAPLAPSAALCNMTTCLQQQIEWITDCIRYMRGNGYTVIEPTASFEEEWVRHHDEMANTTLIAKTNSWYTGTNVEGKPRRVLSYTAGVGTYRQKCTKVAEGGYQGFQMV

>jgi|CyamerSoos_1_1|3320|CmerSOOS_G3306.1

MRTELAQSPDSVTRNAVIELDALIIGAGVAGLYQLHQLRSLGLRAIAVDTAGDIGGTWYWNCYPGAKFDSECYVYQYLFDKKLYKDWTWGERFPSQPEIERWMHYVTDRLGLWHDIRLGTTITLAVYDDERDRWMVETNRGDSFDTRFLIGCCGMLSAPLERRFPGQDRFCGRIFHTSRWPREPLSLAGKRVGVVGVGATGIQVIQTIASEVGHLKVFIRTPQYVVPMKNTKYGPEEVHAYKERFDELRDLLPRTFSGFEYEIKHRWAELTPEQRLAVLEESYGDGSLKAWLGGFNEIFFDPEVSEQVSEFVREKMRARLKDPKLIEILIPKDYGFGTRRVPLESGFLEVFHRDNVEAVSVRNNPIVEVVPEGIRLADGTVHELDVIILATGFDASSGALTRIDIRGRGGRSLREEWSRDIRSSLGLMVHGYPNLFLAGAPLAPSAALCNMTTCLQQQTEWITDCIRYMQANGFTVIEPTAAFEEAWVRHHDETANATLIAKTDSWYVGSNVDGKPRRMLSYTGGVGTYRQKCAEIAKDNYRGFKMA

>jgi|Cyamer1|57806|XM_005536881.1

MGAELNQFTNHVQNKSVVELDALIIGAGVAGLYQLHKLRSAGLNALAVDTAEDVGGTWYWNCYPGAKFDSECYVYQYLFDEKLYKDWSWSERFPGQPEIERWLHYVTDRLGLRPYLRFCTTVTAAEYDEKRGRWIIKTDRGQTYDARFLIGCCGMLSAPLENRFPGQERFRGRIFHTARWPREPIDLNGKRVGVVGVGATGIQVIQTIADKVGQLKVFVRTPQYVVPMKNPKYNPDDVNSYKKRFEELRELLPTTASGFEYVIKHRWADLTPEQRLAVLEETYNDGSLRAWLGGFDEIFTDPQISEEVSEFVREKMRARLKDPKLIEILVPTDYGFGSRRVPLESGYLEVYHRDNVEAICVRNNPIVEVVPEGIRLADGTVHELDIIILATGFDASSGAFIRIDIRGRDGRSLKEEWSRDICSALGLMVHGYPNMFLTGAPLSPSAALCNMTTCLQQQVEWITDCIQYIQAKGHNVIEPTASFQEAWVRHHDDLANSTLFSKTNSWYTGTNVEGKPRRMLSYTGGVGKYRQKCAEVAAGGYEGFAVA

>Mvestita_C1185g21950-RA|Marsilea_vestita/1-509

MHRVETLIVGAGFSGICMGIKLQQAGRNSFLIVEKLGDLGGTWTVNRYPGCECDVPSHLYSYSFYPNPDWTTAFASRGEIHNYLQSCAIHYGILPDHIRFHTRATTATWDNDAMLWRVELVSNNNEPYIVFSRFLVSAVGMLSVPTLPSVPGIQNFRGVSVHSHEWMPSVDELLSGKRVAVVGTGASAVQIVPEVARVAEKLYVVQRTPAWILPKPGFCVPKWCREAFRCLPLLMWLWRVLILCFMEFFGSGVYGGAVFPLLVIKKTIQKVAKHNMEVHIKDVELRKRLTPTYHLGCKRLLLSNHFYPALARPNVELISGSLVEVRENNIVVKPLEDGSDEMVEIPLDTIIYATGFDVSQPKLHVVGSNPDSPLPGSSSPSFLGVTKHGFPNFFTIMGPHSYVGHTSLLLIIEAQARYILSCVDLVLKQNAQSLEVKLCSEDRFQEFLARRFSRTVWQNGGCANSWYVDSMTGQNSIIWPGSTIEYIWHTSSASLDDYTICYAQENVLM

>jgi|Chrzof2|4539484|fgenesh1_kg.chr_02_#_14091_#_transcript/62828

MAPNPIGLPIAIIGAGFSGLGMGIQLKKAGIDSFTIFEAADDVGGTWRDNTYPGCACDIKSYLYCYSFEPNPTWSKPYPSQPEILQYLHNCVDKHQLKPHIQLNAAIVEARFDETSGIWHLSTHDGRKFQARVVIVAKGALTKPKVPALPGIDTFQGTILHSARWDPSIDLSGKRVAVVGTGASSVQMIPAIQPLVKQLVVFQRSAPWVFPRPNKPFSAQQRMLAAVFPLFMKARRFLIYLQNELIGRAFYAKPERLRDAEGLAKAHMESQVSDPQLREKLTPQYRLGCKRVCVSDDYYPALCQSNVNLVASPVVEMRSGSVVAADGSEHEVDAIVFGTGFDVARFLEGTAIHGLGGRELNQQWREKGAQAYYGLAVSGYPNMFMLLGPNSGLGHNSVVVMIECQVKYVVNCIQQLLQRKLKWLDVKPGCQQKFKDEVDAMMVGTVWQSGCSSWYLTPEGKSQTLWPRSTWEYWWRLWSVKLSDWQLGRW

>jgi|Sceob152z_1|12062|Sobl_g21218.t1

MAAAAAPAAGQELSVAIVGAGFSGLCMAIQLKRAGIQDFTIFEAADAVGGTWRDNTFPGCACDIPSVLYCFSFEPYPFTRHYSGQPEIVRYQQHCVTKYGLAPHIRLSTRVTAAAFDAGSGTWAVTTAAGDTVTARVLVLARGGLAVPSVPSFPGMDTFPGAAFHTARWDHSVELTGKRVAVVGTGASAIQIVPAIQPHVASLTLFQRTPPWVMPRKARLPGWAAPAEGCSSWYNKVQRRRWFLGMELLLGGALTRNKQGILKRVDKVGLSHLQAQVPDHQLRAALAPSYSVGCKRILASDEYYPALCQPNVQVVTSAVKEAHGSVIIAEDGTEAEVDVIIYATGFDLTATHHGIDITAPAAGAGVAAAAAGSHAQQTLNFADVMGDKPRAFYGLGVAGFPNLFMINGPNTGLGHNSMIYMAECGVNFIVKLLRRMRRQRLKSIQVTAAAQQQFNKELQARLRSSVWLAGGCGSWYVDRDGEGSSVLWPGLCLEYWWRTCVAGPRAGDWAGAKMDKPVPAAPGASAAAAAAAAVTADVRRKESWGSERSDKKLL

>jgi|Sceobl393_2|1990655|estExt_Genemark1.C_8_t10164

MAAAAAPAAGQELSVAIVGAGFSGLCMAIQLKRAGIQDFTIFEAADAVGGTWRDNTFPGCACDIPSVLYCFSFEPYPFTRHYSGQPEILRYQQHCVTKYGLAPHIRLSTRVTAAAFDAGSGTWAVTTAAGDTVTARVLVLARGGLAVPSVPSFPGMDTFPGAAFHTARWDHSVELTDKRVAVVGTGASAIQIVPAIQPHVASLTLFQRTPPWVMPRKARLPGWAAPAEGCSSWYNKVQRRRWFLGMELLLGGALTRNKQGILKRVEKVGLAHLQEQVPDHQLRAALAPSYSVGCKRILASDEYYPALCQPNVQVVTSAVKEVHGIVIIAEDGTEAEVDVIIYATGFDLTATHHGIDITAPAAGAGVAAAAAGSHAQQTLNFADVMGDKPRAFYGLGVAGFPNLFMINGPNTGLGHNSMIYMAECGVKFIVKLLRRMRRQRLKSIQVTAAAQQQFNKELQARLRSSVWLAGGCGSWYVDRDGEGSSVLWPGLCLEYWWRTCVAGPRAGDWAGAKLDKPVPAAPGASAAAAAAAAVTADVRRKESWGSERSDKKLL

>jgi|Tetrobl72_1|508778|CE508777_788

MAAAAPAAGQELSVAIVGAGFSGLCMALQLKRAGIQDFTIFEAADAVGGTWRDNTFPGCACDIPSVLYCFSFEPYPFTRHYSGQPEILRYQQHCVTKYGLAPHIRLSTRVTAAAFDAGSGTWAVTTAAGDTVTARVLVLARGGLAVPSVPSFPGMDTFPGAAFHTACWDHSVELTGKRVAVVGTGASAIQIVPAIQPHVASLTLFQRTPPWVMPRKARLPGWAAPAEGCSSWYNKVQRRRWFLGMELLLGGALTRNKQGILKRVEKVGLAHLQAQVPDHQLRAALAPSYSVGCKRILASDEYYPALCQPNVQVVTSAVKEVHGIVIIAEDGTEAEVDVIIYATGFDLTATHHGIDITAPAAGAGVAAAAAGSHAQQTLNFADVMGDKPRAFYGLGVAGFPNLFMINGPNTGLGHNSMIYMAECGVNFIVKLLRRMRRQRLKSIQVTAAAQQQFNKELQARLRSSVWLAGGCGSWYVDRDGEGSSVLWPGLCLEYWWRTCVAGPRAGDWEAAKMGESGPAAAGASAAAAAAAVTADVRRKESWGSERSDKKLL

>jgi|Acuobl2326_1|2595238|estExt_Genewise1Plus.C_2_t10261

MGAAAPAAAAAPAELSVAIVGAGFSGLCMAIQLKRAGIHDFTIFEAADAVGGTWQQNTYPGCACDIPSVLYCFSFEPYPFTRHYSGQPEILRYQQHCVNKYGLAPHIRLSTRVTAAAFDSASGTWAVTTAAGDIVTARVLVLARGGLAVPSVPSFPGMQAFPGPSFHTARWDHSVDLKSKRVAVVGTGASAIQLVPAIQPRVALLTLFQRTPPWVMPRKARLPGWAAPAEECSSWYNKVQRRRWFLGMELLLGGALTRNKQGILKRVEKVGLSHLQAQVPDPQLRAALAPSYSVGCKRILASDEYYPALCQPNVQVVTSGVKEVRGSVIIAHDGSAAEVDVIIYATGFDLTATHHGIDITVAPAAAAAAAGTSPDAPQTLSFSEVMGDKPRAFYGIAVAGFPNLLMVNGPNTGLGHNSMIYMAECGVNSIVKLLRRMRRQRLKSIQVTAAAQEQFNKELQARLARSVWLAGGCGSWYVNKDGEGSSVLWPGLCLEYWWRTCVAGPRAGDWVGVKINKQAPAAASGATAAAAAAAAEVATDVRRKESWGSERSDKKLL
