# Supplementary material for: A plant‐centric view of class B flavin‐dependent monooxygenase evolution and diversity
Source: Plant J. 2026 Sep 9;127(5):e71106. doi: 10.1111/tpj.71106 (PMC13557801; doi:10.1111/tpj.71106)
Supplement: Supplementary file 2 [file TPJ-127-0-s008.docx]

>Adc26099|Adiantum_capillus-veneris/21-526

MADVEVLIIGAGFSGLCMAIKLLQAGYTSFVLIEKHADVGGTWLLNRYPGCRCDVPSHLYSFSFDRNPRWTSMYADGDEIHRYIKDLASRHGILPHVHFNSVVLSAVWDDAAALWSVEIEDQHLPVVVSDHSQPLHRPVVNPRQTLTISARFVVRAIGGLHIPNYPAIKGMEKFAGPAFHSSLWDSSINLEGKTVAVIGTGASSIQIVPEIAPIVKKLYVFQRSPPWILPKRNSTIPQKCRQLFKQFPFLMFLFSWLLFLMQETVGCLLLASKGMRRVAQKAALRHMHGIIEDRDMRRKLEPKYELGCKRILLSSSFYPALTRPNVEIIADHILEMHENSILVGAMGGQKTLSLRTDIIVYATGFDLVPPLRVVGTSGLEFNKEGYSSLLGTTKQGFPNFFTLLGFNTGAAYTSQLLGIEAQVRYTMSCLTLMRGLARSIEPHGASQRRYQDFLARRVPATVWTSKGCNSWYLDPNTGHLCSLWPGSTLEFIYRLWSAKPSDYCIK

>Adc26100|Adiantum_capillus-veneris/34-536

MADVEVLIIGAGFSGLCMAIKLLQAGHTSFVLIDKHADVGGTWLLNRYPGCQCDVPSHLYSFSFDRNPCWTSMYADGREIHRYIKDSASRHGVLPHVRFNSVVVSTVWDDAAALWHVEIEQPPAVVSDSQPPAVNPPQMSGTSTITARFVVRAIGGLHIPNLPVIKGMEKFSGPAFHSSLWDSSISLEGKTVAVIGTGASSIQIVPEIAPIVKKLYVFQRSPPWIFPKLNSTIPLWCRQLFMQFPFLMCLFSWLLFLMQEVLGYLLLISKGTRKVAQRAALTHMHGIIEDPDMRQKLEPKYEIGCKRVLLSSSFYPALTRPNVELIADHILEMHENSILIGATGGQKMRSLHIDIIIYATGFDLFSPFRVVGTSGLEFNQEGYSSLLGTTKQGFPNFFTLLGYNTGAAYTSQLIGIEAQVRHTMSCLNLMRGPARSIEPHGASERRYQDFLARRIPGTVWTSKGCNSWYLDPNTGHLFSLWPGSTLEFIYMLWSAKPSDYCIK

>Adc12496|Adiantum_capillus-veneris/1-499

MADVKVLIIGGGFSGLCMAVKLLHAGISSFVLIEKYAELGGTWFLNRYPGCRCDIPSHFYSYSFDRNPHWSSMYADRSEIHQYIKDCAIRHGVLPHVRFNTVVTAAIWDAAESVWHVKMEESVADKSHDVDHPPSSVTSTIKAQFVVRAVGPFHAPQYPEIKGMEKFKGPAFHSSYWDASVDFAGKTVAVMGTGASSIQIVPSIAPIVKKLYVFQRTPAWISPKPTNWSFSKRCRQLFAHFPLLMCLFSWFIFWMHEIIIWGVLRRKWIRRVAEWVVMRNIQGAIKDPALIEKVKPKYEMGCNRILLSNDFYPALARPNVEVMTHPILEVRENALLVSDGPAGVRPLPVDAIVYATGFQLVTKLPVVGANGLEFTQAGYSSILGITKQGFPNFFTLFGYNTGVAQTSQILTIEAQARYVVSCIGLVGGPSRSLEPLEVSERRHQEFLARRFPKLVWSFGGCSSFYLDKSTGHNFTLWPASTLEYIYRTWSANRRDYHIR

>Ceric.10G019200.1.v2.1|Ceratopteris_richardii/1-510

MSSMADVEVLIVGAGFSGICMAIKLLEASLSSFVILERCSDIGGSWFFNRYPGCRSDIPGHFYCYSFARNPSWTTMYPSRSELHQYLKDCASLHGVLPKIRFNTVATSATWNAVDSLWRVEVKETLPYPSSEWSAEPPQVVRTSVILARYVIRAVGGLHLPNYPDIPGVENFTGMSLHSSLWPDNGINLKGKTVAIMGTGASGIQLVPALAPDVEKLYVLQRSGAWVVPKPPNCTFSKRTRQCFAQFPFLMFLFYWILFLLHELIIWWVLEWRYIRRIGEWLVRWNMRRTIKCPDLLRKLEPTYEMGCKRILLSNDFYPAFARPNVNLIRESVLEIREKSLILSSDDGAPEKKTREIPIDVLIYATGFKLTSDLQVVGRCGIDFNSAGFSTLLGITKHGFPNFFMLFGFNTGVAQTSQILTIEAQVRYIVQCLGLMKQRGARSMEARTASEKRHQDFISQQLPKVVWTTGGCSSWYNAPGTHHNFAMWPASTMEYLRRSRNVDPQHYLIQ

>Aspi01Gene61719|Alsophila_spinulosa/1-497

MASVETLIVGAGLSGICLAIKLLEAGRTSFLLVEKSEHIGGTWSVNRYPGCCCDVPSHLYSFSFDRNPAWSKMYADRCEIHQYLTSCAQRHGVVPHVRFNTVARAATWDEAAALWRVEVSPPPTDDVDEGASTTSSIIMARFLVQAVGALHVPKYPDVKGIERFAGPSFHSSLWDAGVDLHDKVVAVIGTGASAVQIVPEIAPITKKLYVCQRTPAWILPKPDFVIGHGWRRLFLQVPVLMWVFRWVLYCLHEVLAWAFLGGRSLRKVGEMLAKMHMNSAISDPVMRASLVPSYELGCKRVLLSSTFYSALARPNVELVTEGIVEVRERSIVFRSLCTQSHVEKDVDFIIYATGFDPLNPKFRVVGTAGVDLKEAGYAAMLGVTKQGFPNFFMLLGPNSGIGHTSQILMVEAQVHYTISCIKLVTPPTRSMELRGSSQCRFQDLLARRFPSTVWRHGGCNSWYLDPHNGHNPTIWPASTVEYIFRTRSVDPCDYHFR

>Aspi01Gene61726|Alsophila_spinulosa/1-497

MACVETLIVGAGLSGICLAIKLLEAGRTSFLLVEKSEHIGGTWSVNRYPGCCCDVPSHLYSFSFDPNPAWSKMYADRCEIYHYLTSCAQRHGLLPHMRFNTVARAATWDEAAALWRVEVSSPPTDDVDDGASTTSSIIIARFLVRAVGTLHVPKYPDLKGIEKFASPSFHSSLWDAGVDLHDKVVAVIGTGASAVQIVPEIAPITKKLYVCQRTPAWILPKPDFGIGHAWRRLFLQVPVLVWVFRWVLYCLHEVLAWAFFGGWSFRKMGEMLAKMHMHSAISNPVMRASLVPSYELGCKRVLLSSTFYSALARPNVELVTERIVEVRERSIVFRSPCTGSHVEKDVDVIIYATGFDPLNSKFRVAGTAGVDLHEAGSATMLGVTKQGFPNFFMLLGPNSAIGHTSQILMVEAQVHYTISCMKLITPPARCMELRASSQCHFQDLLARRFPSTVWRHGGCNSWYSDPHNDHNPTIWPASTLEYIFLTRFVVPSHYHFR

>Mvestita_C1185g21950-RA|Marsilea_vestita/1-509

MHRVETLIVGAGFSGICMGIKLQQAGRNSFLIVEKLGDLGGTWTVNRYPGCECDVPSHLYSYSFYPNPDWTTAFASRGEIHNYLQSCAIHYGILPDHIRFHTRATTATWDNDAMLWRVELVSNNNEPYIVFSRFLVSAVGMLSVPTLPSVPGIQNFRGVSVHSHEWMPSVDELLSGKRVAVVGTGASAVQIVPEVARVAEKLYVVQRTPAWILPKPGFCVPKWCREAFRCLPLLMWLWRVLILCFMEFFGSGVYGGAVFPLLVIKKTIQKVAKHNMEVHIKDVELRKRLTPTYHLGCKRLLLSNHFYPALARPNVELISGSLVEVRENNIVVKPLEDGSDEMVEIPLDTIIYATGFDVSQPKLHVVGSNPDSPLPGSSSPSFLGVTKHGFPNFFTIMGPHSYVGHTSLLLIIEAQARYILSCVDLVLKQNAQSLEVKLCSEDRFQEFLARRFSRTVWQNGGCANSWYVDSMTGQNSIIWPGSTIEYIWHTSSASLDDYTICYAQENVLM

>Mvestita_C716g21983-RA|Marsilea_vestita/1-509

MHRVETLIVGAGFSGICMGIKLQQAGRNSFLIVEKLGDLGGTWTVNRYPGCECDVPSHLYSYSFYPNPDWTTAFASRGEIHNYLQSCAIHYGILPDHIRFHTRATTATWDNDAMLWRVELVSNNNEPYIVFSRFLVSAVGMLSVPTLPSVPGIQNFRGVSVHSHEWMPSVDELLSGKRVAVVGTGASAVQIVPEVARVAEKLYVVQRTPAWILPKPGFCVPKWCREAFRCLPLLMWLWRVLILCFMEFFGSGVYGGAVFPLLVIKKTIQKVAKHNMEVHIKDVELRKRLTPTYHLGCKRLLLSNHFYPALARPNVELISGSLVEVRENNIVVKPLEDGSDEMVEIPLDTIIYATGFDVSQPKLHVVGSNPDSPLPGSSSPSFLGVTKHGFPNFFTIMGPHSYVGHTSLLLIIEAQARYILSCVDLVLKQNAQSLEVKLCSEDRFQEFLARRFSRTVWQNGGCANSWYVDSMTGQNSIIWPGSTIEYIWHTSSASLDDYTICYAQENVLM

>Mvestita_S4g09217-RA|Marsilea_vestita/1-514

MDKNRDEEEVVETLIVGAGFSGLCMAIKLKQAGRASFLIVEKTGELGGTWAENQYPGCACDVPSHLYEFSFYQNPEWSRLFAPRSEILDYINNCAAHFGILPAHVRFHMRAASALWDRTASLWRVDLITGDGKMSTVRSRFLVGAVGPLNVPRFPSVPGIERFRGLSVHSNQWLGRSIDELRGKRVAVVGTGASAIQIIPEVARVAEKLYVCQRTPPWILPKRDREIATWVRWIFRHVPLVLWLWRLWLFCLLELMGSAYWGLMKPASLLRRFGEAMVTKHMEEHIKDPNLRDRMTPSYQIGCKRVLMSSDFYPTLARPNVEVISGSLIEVHEHGLVLKPHPSYSCHKTHVVIDDLDVIIYATGFDLFEPKIRIVGLDCDVAFFDKLWPTFLGVARHGYPNFFVILGANSGLGHSSQLLVIEAQSSYIAKCIQLTLGNHVSHMDVKLKSQEQFEEFLASRFPKTVWRPGGCLSWYVHAESGHNPIIWPASTLEYMYHTRKVHPSDFDFYLEGCS

>Sacu_v1.1_s0019.g007861|Salvinia_cucullata/1-492

MDEADGDEIQAFETLIVGAGISGICIAVRLLQSGRSSFVILEKSDAIGGTWSQNCYPGCRCDVPSHLYCFSFARNPAWETTFADRSQIHAYLCQTAAAYRLNAFIRLRTAATSACWDQCSATWLVRAAASSGSRCAFRARFLVRATGAFQVPRFPDVPGRGNFGGLALHSSQWPEGAGAELARGKAVAVVGTGASAIQIVPAIAGLAGKVYVCQRTPAWVLPKPEFGVPAAVRRLMERFPTLLWLLWWAIFLVQELLLGLALFGVKPLHALATYLAERHLRHSVVDPALRAALQPQYELGCKRVLLSNDFYRSLTQPNVELLCGAVDKVQADGVVVGGVKKKVDMIVWATGFDIWAPTLRIEGRGGKVLEASMGMYYGMTVEGFPNMFILLGPKTGVGHTSQILMIEAQARYVVSCVEMAEGAAVEVRRSSLQKFQKLVARRLSGTVWTRSGGCRSWYVNPKTGTVDAIWPSSIVEYMVRTRRASRCDYHFL

>Sacu_v1.1_s0081.g017998|Salvinia_cucullata/1-511

MAPEEEVDVECVIVGGGISGICMAAQLLSAGRKSFLVLEKSGDVGGTWASNRYPGCRCDVPSHLYCYSFHPSADWTQSYAPRAEIHSYLRHCAKDRHLLPHFRFHSTLISAVWEDANAVWKLALLTAKNGGATNSGDVSTLRCRFLIRATGPGLHIPRLPSVKGVAEGTFAGLSVHSSQWDPDIDVRGKVVATIGTGASAVQIVPAIAPIAKQVIVCQRTPAWVVPVLQIEVPPAVRHFFHYFPVLVLLLRYLLYWIHEVLFASQFIGVLLPYVGVVFSWASRRMLHRSVSDPNIRAALTPSYTFGCKRALLSNTFYETFSKPNVRLLSGSLTEVRRHSLVVKEHATQKEVEAEAEVIIYATGFDACAQPFEVVGRGGVRLSDAGHETMLGIVKHGFPNFFMLLGPNTSLGYSSVVLMAEAQARYIVKCVQMTAAAGGGCWRAMEVTAVAQRRFQKLLDRMFPNTVWKTGNCHSWYVHPITGKNHVIWPGSTLDYFRRTESVSLSDFHVSS

>Sacu_v1.1_s0081.g018001|Salvinia_cucullata/1-508

MAPEEAVDVECVIVGGGISGICMAAHLLSAGRKSFLVLEKYGDVGGTWAYNRYPGCRCDVPSHLYCYSFHPSADWTESYAPRAQIHSYLRHCVKDHNLLPHFRFRTTLVSAVWEDANALWKLALRTAENGGEADSGVLSTLRSRFLIRATGPGLHIPRLPSAKGAADGTFAGTSLHSSQWDPDLDLRGKVVAVVGTGASAVQIIPAIAPIAKKLIVCQRTPAWVVPVLHIGVPPAVRRVFHYFPVLVLLLRYVEYCIQELMFSSQFFGIYLRYFGAVFPWALRQLIRRSVADPNISAALTPTYTFGCKRALMSNTFYKTFARPNVRLLAGSLTEVRRHSLVVKENATQKEVEAEVEVIIYATGFDACAEAFEVVGRGGVRLSDAGHETMLGIVKHGFPNFFMLLGPNTTLGHNSLILMAEAQARYIVKCVQMAAGRGLRAMEVTAEAQRRFQKLLDRMFPNTVWKAGNCLSWYVHPITGKNHVIWPGSTLDYFRRTGSVSLTDFHITS

>Azfi_s0073.g037082|Azolla_filiculoides/1-461

MPSHLYSFSFHRDPSAWSSLYATRAEIHSYLADCARVRGVLPHVRFHTEALSATWDDRAAMWRIRAAHRRHDQDQDKDHDHEQDQDHDHDQDQDQDHGFFEIHARFLVRAIGPWHMPKFPDKALVPGIELFAGPSFHSSAWDPSIDLRGRVVAVVGTGASAVQIAPEVAPIARKLYICQRTPAWVLPKLEARIPTAAQRVLRRFPTLLCGLLWWTFLFVVELYLWALLGGPRRRRMGAMLAKRHLYRSIRDPTLRAALLPTYEFGNFGCRRFLTSSTFYPTLSRPNVELLPGGLREVRPHSIVVKDQSQGVDVEKDVDVIIYATGFDIHYPNFDIVGLNGIHLKHASSDSLLGVTKHGFPNLFMILNPYNGLVLTSLTLVAEIQASYILSCMSLLNPTDKFQPRSLEPRETSQQQFHDRLTKKTWKTCHTTPLNKPIFWTFSTIDYIFDACFASPSDYIIK

>GWHPBFSI003790|Chloranthus_spicatus/1-580

MAGDMPWPEVAVGNKNHANAAVVIIGGGISGMCVAIDLLKRNKCRNFIILEKSAGLGGTWLDNKYPGCCCDVWSALYSYSFAKNPNWTREYPGQEEILAYLQDVAQDYKLLPHFRFNTMVKDATWDETEKKWKVSVETSKGSKEAEYSEGYEITADFVVSAVGQLNVPQWPKIDGIDSFEGKKMHSARWDWSYGLEGKKIAMIGNGCTSVQILPEIAKVAKQVTVFQRTANWVIPRLDAPVSEVWRTIYKYVPGVMNRKRAGQMDFRESSHGFISDSNSPIAAMFTDASKQLMEAQLPNQPELWKKLTPTYNLGCKRIIISDDYFPAIARDNVSLETRPIDSISGNSIKVKDDNGSTVDLSDFDLLVCATGFKTVDFMHPVQLYGKNGHPIADVWKSGAKALYGMTVEDMPNFAMLYGPNTNLGHNSIILMIESQSRYINGLIKPVLDARKQGQALSVRPKKDVLEAFNEEIQAELRSSAFNDPNCNSWYKNDAGLITNNWSRTVVEYQKLVEDVKFDDYVVEGSGKNMIGEKKTAHVGRVREESLISDTALTVLGAVSTAAVVGGWLMRSGYLQRIMAR

>PaFOS1|Phlebodium_aureum

MAPNVPSAMYTTCLPERTALQEKLSGDRAALAARVCIVGAGVTGLCACKHLLQHGVESVVLEMQTGLGGVWRNTFSSTKLQTPRDAFQYSDFLWPPGTPRLPSHAQVLDYLESYARKFQVLERIVFNCRVLEVRRLDLEGPPPVSAFQKLPANAKWAVRVQQGSADLTQLDAEWLYFEYVLVCVGHYGDIAKTPKFAVPSKGPQAFHGKVLHSLDYSKLDEAATKKLLTGKRVIVVGSRKSALDAVMEASNSNRGPDGKPVTLLFRTPHWVIAHHFFYGIPMSYMTSRFSQLLVPQPGGGSTLHDIISAALSPLRWALSKALEFYLLSTLPLAEHGFVPNQNIFEEFKSCKLTLLPEHFFDYVKEGHIKLQKTSTWCFSSNGVTLPNGDELEADVIILCTGYEGERKIKSLLGTSFEDILQENDSSIPLYRGVLPPRIPNMAILGFQESYSNLFSAEMSARWVAYWIAGKIALPNKEIMEMEAYKWLRNTKPFMTINDKTCVNTTALQLADQMCRDMGWNSRRKKSLLQEIFAPYGNMDYKDE

>PaqFOS1|Pteridium_aquilinum

MAPNVPSAMYTTCLPERTALQEKLSGDRAALAARVCIVGAGVTGLCACKHLLQHGVESVVLEMQTGVGGVWRNTFSSTKLQTPRDAFQYSDFLWPPGTPRLPSHAQVLDYLESYARKFQVLERIVFNCRVLEVRRLDLEGPPPVSAFQKLPANAKWAVRVQQGSADLTQLDAEWLYFEYVLVCVGHYGDIAKTPKFAVPSKGPQAFHGKVLHSLDYSKLDEAATKKLLTGKRVIVVGSRKSALDAVMEASNSNRGPDGKPVTLLFRTPHWVIAHHFFYGIPMSYMTSRFSQLLVPQPGGGSTLHDIISAALSPLRWALSKALEFYLLSTLPLAEHGFVPNQNIFEEFKSCKLTLLPEHFFDYVKEGHIKLQKTSTWCFSSNGVTLPNGDELEADVIILCTGYEGERKIKSLLGTSFEDILQENDSSIPLYRGVLPPRIPNMAILGFQESYSNLFSAEMSARWVAYWIAGKIALPNKEIMEMEAYKWLRNTKPFMTINDKTCVNTTALQLADQMCRDMGWNSRRKKSLLQEIFAPYGNMDYKDE

>Adc12184|Adiantum_capillus-veneris/1-508

MAASVCIVGAGVTGLCACKHLVQHGVECVVLEAQEGVGGVWRNTFASTKLQTPRDAFQFSDYLWPKGTPRLPTHFQVMDYLTAYARHFRVLERILFGCTVVKVCCLDRWPPTPASPKIRANANWAVCVRRGDQSDAEWLFFEYLVMSVGHYGDVPKFPSFPESKGPAAFGGKVLHSLDYSKLDEGASKELLSAKRVVVVGSRKSALDLVMEAANCNRGDDGQPAMLLYRTPHWVIANHNFYGVPMAYMTSRVSQLLVPQPGTDKNTLHGLISAIILSPLRWAISKAMEHYLLTTLPLVEHDFVPTQKVFEELKSCKLTLLPERFFERAKAGHITLQRTSDHWGFVEDGIVLPSGDKLKADVVVLCTGYEGEEKLKSLLGMSFHDLLQQRDGSIPLYRGVLPPRVPHLAVLGFQESYSNLFTAEMGARWTAHWIAGKVAVPSVEKMEKEAQRWLLNTKPFVTSNDKTCVNSTILGLADEMCKDMGWNPRRKKSLLQELFAPYGNMDYKD

>MA_10430096g0010|Picea_abies/1-526

MLWVRLHLNEFGKRRVCIIGAGMSGLLATKYLLSLNLNIIVFEAKPGIGGVWRETFASTKLQTPRSDFEFTDFPWPSYVTTQFPSHSEVMDYFHSYAKHFGLLDHIHFNSKVVEIRYIEKCCEKSPGHAGLWGKNGSPYADSAVWELGVQKRNAESIEWHRFDFLVLCPGRYGDVPKIPSFPPNKGPEVFQGKVFHTMDYSVLDERSAYELIKGKRVIVIGFQKSAMDFAAECAEANQGGGYPCTMVFRTVHWTIPSDKVWGVPLSFIYGTRFAQWLLAKPGQSFLESVILRLFAPMRWAMSKFVEFYLLWKLPLRKYGLVPDRSFLQEISSCQVSSVPDNLFPKAEEGFILFRKSSNCSFSSKGIVLDDGTEVGADLVVLGTGYDGEKKLKSILPTRFVEVLQKSADLLPLYRGLIHPHIPHMTILGYQESLSNLHSSELHTRWLAHFLSGKFVLPSIKEMEADTEKHSKHIKKITPFYWKSCLATFQIWDNDNLCRDIGWNPMRKKNWFCELFSPYSNIDYREM

>PSME_00024572-RA|Pseudotsuga_menziesii/1-535

MAHCNGNLFLEGEVENHNGFWQRRVCIIGAGMSGLLATKYLLNLNLNTTVFEAKQGIGGVWRETFASTKLQTPRPAFQFTDFPWPKHVTTQFPTKSEVMDYIHSYAEHFGLLDYIQFNSKVVEIRYSEKYSEKSLEDAGLWGKNGGPYADGEVWEVGVQKSHAESIEWHRFDFLVLCVGRYGDLPKIPSFPPNKGPEVFQGKVLHTMEYSVLDERSAYELIKGKRIVVIGFQKSAMDLAAECAEANQGGRHPCTMVVRRVHWTIPSYNVWGVPLSFLYGTRFSQLLLWKPGQNFLQSVILRLLAPMRWAMSKFVELYLLWKLPLRKHGLVPDHSFLQEISSCQIASLPDNLFPKAEEGCILFRKSSNCSFSSKGIVLDDGTEVEADLVILGTGYDGEKKLKSLLPTRFADVLQKSADPLLLYRGLIHAQIPHMAILGYPESLSNLHSSEIRTQWLAHFLSGKFVLPSIKEMELDTENYFKHIKRITPFYWKSCLATFQIWDNDNLCRDMGWNPMRKKNLFCELFSPYSNLDYREM

>PSME_00044684-RA|Pseudotsuga_menziesii/1-506

MANWNGNLFVEGEVENENGFRQRRVCIIGAGMSGLLATKYLLSLNLNTTVFEAKQGLGGVWRETFASTKLHTPRPAFEFTDFPWPKHVTTQYPTASDVMDYFHSYAQHFGLLDHIQFNSQVVEVRYSEKYCEKSLEDAGLWCKNGGGPYADGALWEVGVQKSHAESIEWHRFEFLVMCLGRYGEVPKTPSFPPNKGPEVFQGKVLHAMEYSVLDERSAYELIKGKRNVVIGFQKSAMDLAAECAEANQGGGHPCTMIVRRAHWAIPTFNIWGIPLSYLCGTRFSQFLIGNPGQSFLQSVILRLLAPVKWALSKFVELYLLWKLPLRKHDLVPDHSFLQEISSCQVAALPDNFFLKAEEGCILFRKSSNWSFYSKGIVLDDGTEVEADLVLLGTGYDDEKKLKSLLPTRIGDVLKKSADPLQLYRGLIHPRIPHMAMLGYPGSLSYLHNSEIRTQWLAHFLSGKFVLPSIKEMEADIGNYFKHIKRITPFHSKYCLATFQIWDNDNL

>MA_10240160g0010|Picea_abies/1-335

MDYSALDEKSAAELIKGKKIVVIGIRKSAMDFAAECAEANQEGGHPCTLVFRAAHWTIPGYNVWGVPLSLFYGTRFAQLLLEKPGQGFLYSLISRLFAPMRWAMFKLVELYLIWKLPLRKYGLVPDHSFLQELSSCQISSLPDSLFPKAEEGCILFRKSYNWGFCSKGIVLDDQTVVEADLVVLGTGYDSEKKLKSVLPKIFTDALDRSAVVLLYRGLIHPHIPHLAILGYPECLSNLHSTEIRSQWLAHFLSGKFVVPSIKEMEADTDNHSKYMKKITPFYSKTCLDAFQIWDNDTLYRDMGRNPMRKKNWFCELFSPYGNLDYQESAEIVCVY

>Thupl.29381623s0008|Tplicata_v3.1/1-530

MLNSNENVCPENERKDGCRKVCIIGAGMSGLLALKYILNIDVNLTVFEAKEGIGGVWRETFASTTLQTPRPAYQFTDFPWPSHVTDRLPTQSQVMDYLNRYAENFGLLDRIQFNSKVVGIRYIGEKSMECSGLWSKNGGPYAEGRAWEVGVQKRNGGSIEWHHFDFVVMCIGRYGDVPNIPSFPPNKGPEVFEGKVLHTMDYSALNKTEAYELLKEKRVLVIGFQKSALDFAVECAEANQGKGGKSCTMIFRTVHWTVPHDHKFWGVPLVYLYGTRFTQFLLDKPNRGFLLRSISHLFAPLRWAVSKFVEFYLLWKFPLRKYGLVPCHSFLQEISSCQIATLPEKLFTKAEEGLIQFRKASTWSFSRKGILIDDVTEVEAEVVVLGTGYDGEKKLKTLLPKNFGDVLEKPKGVVPLYRGLIHPKIPQMAIMGYPESISNLHSSEIRSQWLAYFLSGKFVLPSIKDMEAYTERQEEFMKRITPFYWKTCHATFQISDNDNLCRDMGWKPLRKKNWFQELFSPYSNLDYMQV

>KAH9305699.1|Taxus_chinensis/1-503

MSGILAAKYLLHIGLNPTVFEAKQGIGGVWRETFASTKLQTPRPAFQFTDFPWPSHVTTQLPTQSQVMDYFHSYANHFRLLDHIQFNSKVVGIRYLGKESMECLGLWGKNRGPYAEGTVWEVGVQKSNGDSLEWHHFDFLVMCIGRYGDVPKIPSFPTSKGPEVFQGKVIHTMDYSALNKTQADELLKGKRVVVIGFQKSALDFAVECAEANQGDGGKSCTMVFRTVHWTVPHYYKCWGVPLPFLYGTRFAQFLLEKPDRSFLLTSIFRLFAPLRWAMSKFVELYLLWKFPLKKYGLVPDHSFLKEISSCQIATLPDKLFPKAEEGLIRFSKASTWSFSSKGIMIDDVTEVEADVVILGTGYDGETKLKTLLPKQFADVLENPEGVVRLYRGLIHPNIPQMAILGYPESLSTLHSSEIRCQWLAYFLSGKFVLPSIKDMEEYTDRMEKYMKRITPFYWKSCYATFQIWDNDNLCTDMGWNPMRKRNCFQELFSPYSNLDYRQV

>KAH9305700.1|Taxus_chinensis/1-449

MLPTQLPTQSQVMDYFHSYANHFGLLDCIQFNTKVVDIQHTGKETVASLGLWGKNGGSYADGRVWQVGVQKSNGESIEWHHFDFLVLCIGKYGDLPKIPSFPPNKGPEVFQGKVLHTMDYSALEEKQAYKLLKGKRVVIIGYQKSAMDLSVECAEANQGENGHPCTMVFRRTHWILSEDYMCSGVHLAYLYGTRFAQFLLGKPDQGFMLNSISYLFAPLRWAVSKFVEFSLLWKFPLRKYGLIPDHSFLQEISSCYTGLFPNNFFSKAEEGLIRFRKSSSWSFSCKGIILDDGTELEADIVVLGTGFDGVKKLKSLLPNKFGDVLEKSAGVVPLYRGRVHPRISQMAILGYSESLSNLHSSELRSQWLAHFISGKIVLPSIKEMEAYTDMYENYVKRITPFYWKSCHATFQIADNDHLCRDMGRNPLRKNSWFDELFSPYSNLDYAQDL

>Thupl.29379188s0006|Tplicata_v3.1/1-531

MASSKWGAMEEGENCNGYRVKKVGIVGAGLSGILATKYLLSIGLKPTVFEAKEGIGGVWRETFASTKLQTPRPAYQFTDFLWPSHVTTLLPNHSQAMDYFNSYAIHFGLLDCIQFNTKVVEIRYSGKQSGASAGLWGKNGGPYDDGEVWEVGVQKSNGEFIEWYHFDFLILCIGKYGGMPKIPSFPPNKGSEVFQGKVLHTMDYSALNEKEAYEIVKGKKIVIIGYQKSAIDFAVECAEANQGENCPPCTMVFRKPYWLVPEDYLVGGVHLAYLYGTRFSQFLLGKPAQGFLLNSLSSMFAPLRWAISKFVELSLLSKFPLKKYGLVPDMSFSQTLASCNTGSFPNNFFPKVEKGLICFQKSSGWSFYSKGVVLDDGTSVEADVVVLATGYDGDKKLKTFLPKEFGDAFEKSGVALSLYRGLIHPSIPQMAILGYSESLSNLHSAEIRSQWLAHFLSNKITMPSVKEVEENAKDLQKYLKKLTPCFWKACIFQIADNDNLCKDMGRNPMRKGNWYDDIFAPYSNMDYGMDN

>Thupl.29381994s0001|Tplicata_v3.1/1-331

MDYSALNEKEANEIVKGKRIVIIGYQKSAVDFALECAEANEAGENCPPCTMVFRKAYWFIPEDYVIGGVHLAYLYGTRFSQFLLGKPAQGFLLNSMSCTFAPLRWAISKFIELSLLWKFPLKKYGLVPDMTFLQTITSCTVGAFPNSFFQKVEEDLIRFRKSSSWSFSNKGIVLDDGTSVEADVVVLGTGYDGDQKLKTFLPKEFGDAFEKSGAALSLYRGLIHPHIPQMAILGYSEGLSNLHTSEIHAQWLAHFLSNKIMMPSVKEMEENAKDLKKYLKRLTPCFWKTCIFQISDNDNLCKDMGRNPMRKGNWYADFFSPYSNMDYGMDN

>Thupl.29380306s0012|Tplicata_v3.1/1-534

MASYGGGLLQEAERSSTDYKQKKVGVIGAGISGLLATKYLVSIGLKPTVFEAKEGLGGVWRQTFASTKLQTPRPAYQFTDFPWPSHVTTLLPNHTQVMDYLNCYADHFGLQDYIQFNTKVVEIRYSGNESSESAGLWGKNGGPYDVGEVWEVGVQKSNGESIQWHHFDFLVLCIGKYEGLPNIPSFPLNKGPEVFQGKVLHTIDYAALDEQESYKTIKGQRVVIIGYQKSAIDFAVECAEANQGDNDPPCTMVFRRTHWMIPEDYLFWGVHLQYLYGTRFTQFLLGKPNQGFLLIILAFLFSPLRWTISKFVELYVLWKFPLKKYGLVPDMNFIQAITSCNTGSFPSKFFPKVEKGSIRFQKSSSWSFYNKGIVLDDGTKIEADVVVLGTGFDGDKKLKSLLPKKKGLILEKIGIALSLYRGLIHPHIPQMAILGYVDSLSLLHSSEIHSQWLVHFLSNKLLLPSVKEMEENTKYLEKYLEIMTPFFWRTCHATFQITDNDNLCRDMRRNPMRKENWIDELFSPYSNMDYGLDN

>PITA_32379|Pinus_taeda/1-532

MATSIANMSVECETENGFRKRRVCIIGAGVSGLVATKYLLKLNMDVTVFDLKQGVGGLWRETFGSTILQTPRHEYQFTDFPWPAHVTAKYPTHSEVLDYFESYAMHFGLLDHIHFNTKVVEIRYMGEDRERSLDHAGLWSNNGGPYADGAVWDVGVQKRNEESIEWYRFDFLVLCIGRYGNLPYIPSLPPNKGPEVFQGKVLHTMDYSGLDEKDAYELIKGKRVVVIGLQKSALDFAAECAEANRDGHPCTVVFRRAHWGIITYELFGLPLQLFYNTRFAQFLLERPAQGFLLTVLSRLLRPLRWIIFKLLEFYLCWKLPLQKHGLVPDHSFVEELASCNTSTYPANLFPRAEEGCVHFRKSSNWSFSSKGIVLDDGTEVKADIVVFATGYDAEKKLKTLLPEPFAEVLEKTAGVVPLYRGTIHPHIPHMAIIGYSESLANLYSSEIRAQWLSHFLSGKFVLPSLKEMETDAENYYNHWMRVSPFYWKPCHAAFQIWDNDNLCRDMGWNPMRKKNWFYELFAPYSNLDYREM

>PSME_00044768-RA|Pseudotsuga_menziesii/1-527

MATTETENGFRKRRVCIIGAGMSGLVATKYLLNLNMDPIVFEVKQGIGGVWRETFASTRLQTPRHEYEFSDFPWPNHVTHKHPTHSEVMDYFQSYAIHFGLLDHIHFNSKVVEIRYNGQESCESSLEHAGLWSKNGGPYADSAVWDVAVQKRNGESIEWYRFDFLVLCIGKYGDLPNIPSFPPNKGPEVFQGKVLHTRDYSVLDENSAYELIKGKKVVVIGSQKSAMDFAVECADANRGEDGHPCTVVFRRAHWAIISYELFGLPLQLFYNSRFAQFLLERPAQGFLHSVLSRLFRPLRWTIFKFMELYLCWKLSLRKYGLVPDHSFVEELSSCNTSTYPDNLFSRAEEGCILFRKSPNWSFSSKGVVLDDGTEVETDLVVYATGYDAEKKLKTLLPESFADVLEKSAGIVPLYRGLIHPHIPHMAILGYSESLSNVYSSEMRSRWLSHFLSGKFVVPSMKEMEIDIDNHFKHWKRITPFYWKSCHGAFHIWDNDNLCRDMGWNPMRKKNWFSELFSPYSNLDYREM

>PSME_00045029-RA|Pseudotsuga_menziesii/1-525

MATSESENGFRKRRVCIIGAGMSGLVATKYLLNLNMDPIVFEVKQGIGGVWRETFASTRLQTPRHEYEFSDFPWSNHVTHKHPTHSEVMDYFQSYAMHFVLLHHIHFNSKVVEIRYMGQEYCESSLENAGLWSKNGGPYADSAVWDVAVQKSNGESIEWHRFDFLVLCIGKYGDLPNIPSFPPNKGPEVFQGKVLHTRDYSVLDENSAYELIKEKRVVVIGSQKSAMDFAVECADANRDGHPCTLVFRRAHWAIISYELFGFPLQLVYNSRFAQFLLERPAQGFLHSVLSRLFRPLRWAIFKFMELYLCWKLSLRKYGLVPDHSFVEELSSCNISIYPDNLFSRAEESSILFRKSPNWSFSCKGVVLDDGTEVETDLVVYATGYDAEKKLKTLLPEPFADVLEKSAGIVPLYRGLIHPHIPHMAILGYSESLSNLYSSEMRSRWLSHFLSGKFVLPSIKEMEIDIGNHFKHWKRITPFYWKSCHGAFHIWDNDNLCRDMGWNPMRKKNWFSELFSPYSNLDYREM

>PSME_00024745-RA|Pseudotsuga_menziesii/1-237

MATSETENGFRKRRVCIIGAGMSGLVATKYLLNLNMDPIVFEVKQGIGGVWRETFSSTRVQTPRHEYEFSDFPWPNHVTHKHSTHSEVMDYFQSYAMQFGLLDHIHFNSKVVEIRYIGKEFCESSLKHAGLWSKNGGPYADSAVWDVAVQKSNGESIEWHRFDFLVLCIDKYGDLPNIPSFPPNKGPEVFQGKVLHTRDYSILDENSAYELIKGKRVVVIGSQKSTMDFAVECADAN

>PITA_13475|Pinus_taeda/1-535

MATSSANMSMDCKTENGFRKRRVCIIGAGMSGLVATKYLLNLNMNPVVFEVKPGIGGVWRETFGCTRVQTLRHEYQFTDFPWPSHVTNQFPTHYEVLDYFQSYAMHFGLLDHIHFNTKVVEIRYIGHEYCESSLEHAGLWSKNGGPYAHSIVWDVGVHKVNGESIEWHRFDFLVLCIGKYGDLPHIPSFPPNKGPEVFQGKVLHTMDYARLDEKSAYELIKGKSVVVIGCQKSALDFAVECAEVNQEKDGHPCTVVFRRAHWALISFELYGLPIQLFYNTRFAQFLLERPAQGFLHGVLSRLFRPLRWAIFKFFELYLCRNLPLRKYGLVPDQSFVEELSACTVSVYHGDLFSRADEGSILFRKSPNWSFSSKGVVLDDGTEVEADLVVFATGFDAEKKLKNLLPGPFAGVLEDSTGIIPLYRGLIHPHIPHMAILGYSESLSNLYSSEMRSRWLSHFLSGKFVLPSMKEMLTDIDNHYKHWKRISPFYWKSCHGAFHIWDNDNLCRDMGWNPMRKKNWFYELFAPYSNLDYREM

>PITA_44980|Pinus_taeda/1-535

MATSSANMSMECETENGFRKRRVCIIGAGMSGLVATKYLLNLNMDPVVFEVKQGIGGVWRETFGCTRVQTPRHEYQFTDFQWPDHITAQHPTHTEVMDYFQSYAMHFGLLDHIHFNTKVVEIQYIGQEYCESSLEHAGLWSQNGGPYGDSVVWDVGVQKINGESIEWHRFDFLVLCIGKYGDLPHIPSFPPNKGPEVFQGKVLHTMDYARLDEKSAYDLIKGKRVVVIGYQKSALDFAVECAEANRGEDGHPCTVVFRRAHWGIISYELYGLPIQHCYNTRFAQFLLERPAQGFLQSALSRIFRPLRWAIFKFFELYLCSNLPLRKYGLVPDQSFVEELSACTVSTYHGDLFSRADEGSILFRKSPNWSFSSKGIALDDGTEVEADLVVFGTGFDAEKKLKTLLPGPFAGVLEDSTGIIPLYRGLIHPHIPHMAILGYSESLSNVYSSEMRSRWLSHFLSGKFVLPSMKEMLTDIDNQYKHWKRVTPFYWKSCHAAFHIGDNDNLCRDMGWNPMRKKNWFYELFAPYSNLDYREM

>PSME_00042948-RA|Pseudotsuga_menziesii/1-229

RWAMFKFLELYLCWKLPLRKHGYVPDHSFVEELISCNTSTYPGNLFPRVEEGCILLRKSPNWIFSSKGVVLDDGTEVEADLVIFATGCAAEKKLKTLLPETFADVLDKSAGVVPLYRGLIHPHIPHLAILGYSESLSNVYSSEMRSRWLSHFLSCKFVLPNVKEMEADIDSDFKHWKRCTPFYWKSCHAAFHIWDNDNLCRDIGWSPMRKKNWFCELFSPYSNRDYREM

>PSME_00056060-RA|Pseudotsuga_menziesii/1-226

MFKFLELYLCWKLPLRKHGYVPDHSFVEELISCNTSTYPGNLFPRVEEGCILLRKSPNWIFSSKGVVLDDGTEVEADLVIFATGCAAEKKLKTLLPETFADVLDKSAGVVPLYRGLIHPHIPHLAILGYSESLSNVYSSEMRSRWLSHFLSCKFVLPNVREMEADIDSDFKHWKRCTPFYWKSCHAAFHIWDNDNLCRDIGWSPMRKKNWFCELFSPYSNRDYREM

>Gb_40713|Ginkgo_biloba/1-518

MGKKVGIVGAGVSGLVACKHLLQRGFEPVVFEAQESIGGQWRQTCASTKLQTPRAAFQFSDFPWPPQVTAEYPTHLQVMDYFHAYAIHFNLLPSIRFNSRVVEIKYLMTEGLHNLSSAGLWGHTGEPFNERESVWEIRVQNINNLNSIEVHRFDFLILCIGRFGEEPRLHTFPADKGPQIFQGKVVHSMDYSALDDQSAYDLVKGKRVVVIGFQKSALDIAAECANANFGENGIPCTMIYRSVHWAVPNYVPWGVHIAYLYCTRFSEMLVHKPGEGFLLSALATLLAPMRWAVSKFVESYLLWKLPLRKYGLIPEHSFLQQISSCQLSTLPDDFYSKVEEGAIVLRKSVEWNFFRKGIVVDDGTEIEADVIILGTGYDGDKKLKDLLPKSFSDLLLDSTGLVPLYRGCIHPRIPHMAILGYPESLSNLHSSEIRSQWLAYLLAGKFGLPSINEMRRDISRWVKYMKNSTPFYWRACIGALHIWHNDQLCIDMGWNPKRKKNWFQELFSPYSNMDYREE

>Adc07644|Adiantum_capillus-veneris/1-523

MGSCPPPQRAKSPLVCIVGAGMTGICACKHLLQHGIQPVVLEAQPGVGGVWRHTFASTKLQTPRDAYQFSDYTWPPGTPDYPSHTDVKEYLRSYACNFDVMDCIRFNCKVVQIRQLNEPLDELHSSRADDARGLHWAVCVQSSSSDKHMNAEGDLEWLFFDFLVLCIGRYGDIPKLPAYPASKGPEIFQGKVLHSMEYAKLDDCAAKHLLQDKRVVVIGMMKSALDVAVEASNANQGIRGHPCTVIFRAAHWMLPHHEPFGLPLSYLYSTRLSELMIPKPDQGFLLSVISTLLTPARWAISKLVEKYLLHTLPLRELGLVPNQTFFEQFASCQVPLLPDAFFQRVKDGQINLLKAESWSFSKSGVVLGDRTSIEADVVILCTGYDGEKKLRSLLQPVTRELQNFGSVIPLYRGVIHPRIPHLAIMGFHENLSNLHSAEMGARWLAHLLSQKISLPSIGTMETVTQKWIEYMRNATPFYQKSCIGAVSIWHSDQVCQDLGWNPRRKRNFFQELLAPYSNMDYKD

>Aspi01Gene40270|Alsophila_spinulosa/1-544

MGNNHTESPTPVFELGSHEKRRSPSSVCIVGAGMTGICACKYLLQHGMEPTVLEAQPGVGGVWRHTFASTKLQTPRDAYQFSDYTWPSGTPDYPSHTHVMDYLESYARKFHVLERIRFNCKVVEIRQIRDGGLDLSVADLKEEEKKKKKLAHWAVRIQNGSPEPETKPSDDGSEWIYFDFLVLCIGRYGDIPKLPTYPPSKGPELFQGKVLHSMQYAMLDDTVATELLKDKRVVVVGMMKSALDVAMEASNANQGIHGHPCTVIFRTPHWMLPHHKPFGLPLSYLYSTRLSELMIPKPDQGLLLSVISTLLTPARWAISKFVELYLLFNLPLREFGLIPNQTFFEQFASCQVPLLPDTFFQRVKDGHINLQRATDWSFSKKGVVLADGTNIEADVIILGTGYEGEKKLRALLQRECGELREPGNIIPLYRGIIHPRIPHLAIMGFHENLSNLHSSEMGARWLGHLLSHKITLPSIDKMENVAQKWIEYMKNSTPFYQKSCIGAVSIWHSDQVCHDLGWNPRRKRNVLEEFLAPYSNMDYKDEVQ

>Ceric.23G070500.1.v2.1|Ceratopteris_richardii/1-537

MVTNHTGSTNMGSSQKSRSPLVCIVGAGMTGICACKHLLQHGIRPVVLEAQSEIGGVWRQTFASTKLQTPRDSFQFSDYLWPPGTPVYPTHVDVKQYLHSYASKFGVLDCIRFKCKVLQIAQISGPRDELRPHLGNCLDAESVNKDCGWAICVQVDSSGNHLDADDNVNWLYFDFLILCIGRYGDIPKLPTYPPSKGPDVFRGKVLHSIEYANLDDNSVRSLVHNKRVVVVGMMKSALDLAMEASNANQGKNGHPCTMIFRSAHWMRPDNRTFSLIMSYLYATRFSELMIPKPEQGLLLSIISTFLTPMRWGISKFVEMFLLYSLPLRQFGLVPEETFFEQYASCQLPAIPESFFERVQEGHIKLCKTRNWGFFENGIVLEDGRQIEADVVILGTGYDGEKKLMSLLQKTEYTKLQNSEGGIPLYRGLIHPHIQNLAIMGFNEALSNLYASEMGARWLAHLLSGKFTLPSIEIMEKMSQKWNEYIKNATPFHQRSCIGAISIWYSDQVCHDLGWNPRRKRNIFQELFAPYSNMDYRD

>Ceric.23G070500.3.v2.1|Ceratopteris_richardii/1-305

MMKSALDLAMEASNANQGKNGHPCTMIFRSAHWMRPDNRTFSLIMSYLYATRFSELMIPKPEQGLLLSIISTFLTPMRWGISKFVEMFLLYSLPLRQFGLVPEETFFEQYASCQLPAIPESFFERVQEGHIKLCKTRNWGFFENGIVLEDGRQIEADVVILGTGYDGEKKLMSLLQKTEYTKLQNSEGGIPLYRGLIHPHIQNLAIMGFNEALSNLYASEMGARWLAHLLSGKFTLPSIEIMEKMSQKWNEYIKNATPFHQRSCIGAISIWYSDQVCHDLGWNPRRKRNIFQELFAPYSNMDYRD

>Ceric.23G070500.2.v2.1|Ceratopteris_richardii/1-280

MIFRSAHWMRPDNRTFSLIMSYLYATRFSELMIPKPEQGLLLSIISTFLTPMRWGISKFVEMFLLYSLPLRQFGLVPEETFFEQYASCQLPAIPESFFERVQEGHIKLCKTRNWGFFENGIVLEDGRQIEADVVILGTGYDGEKKLMSLLQKTEYTKLQNSEGGIPLYRGLIHPHIQNLAIMGFNEALSNLYASEMGARWLAHLLSGKFTLPSIEIMEKMSQKWNEYIKNATPFHQRSCIGAISIWYSDQVCHDLGWNPRRKRNIFQELFAPYSNMDYRD

>Mvestita_S18g14860-RA|Marsilea_vestita/1-572

MDHATTKSSIAIHPYILRSQSSETYSERLSKEEEQGSFVLLHELMESSANLPVSQSPDAPSVCIVGAGMTGICASKYLLQHGIQPVIFEAQPGIGGVWRHTFASTKLQTPRDAYQFSDYTWPPGTPDYPSHDHVMKYLHSYASEFHVLERIRFNCKVVEARQVGCFDVADDNGNVDEGARKGHQWAVCVQKISDASSASTEWFYFDFLLLCIGRYGDVPNLPIYPPSRGPEIFKGKVLHSMEYAMLDDKTANEFLANKRVVVVGMMKSALDVAVEASNANLGLHGHPCTVVFRSAHWMLPHHKPFGLPLSYLYSTRFSELMIPKPDQGHFLSIISTLLKPMRWTISKFVELYLLLKLPLRQFDLVPKQTFYQQFASCQVPLLPDLFFQRVQAGHIQLKRTNDWSFSENGLVLDDGTNIEADVVILATGYKGEMKLKSILQGSCAQGLKSSGDIIPLYRGIIPPRIPHLAVIGYHENLSNLHSSEMGARWLAHLLSSKIHLPSIVKMEEDVQKWTDFMKNSTPFYQKSCIGAVSIWHSDQMCRDLGWNPKRKNNCIQELFTPYSNMDYQDCST

>Sacu_v1.1_s0103.g019960|Salvinia_cucullata/1-558

MMKKMEAEVRSLNSRNGGGTEDVGALGGEGEEGKRSSNRRRVVGIIGAGMTGVCVCKYMVQQGMEAVVMEAQPGMGGVWRHNFANTRLQTPRDAYQFSDFLWPHGTPLYPSHVHVLEYLESYARKFHVLPRILFNTKVVAIRQMHPGSSSSSPRWAVLIRRSSSMAQSDVKQVPHDAAAEHEERWLYFDYLVSCVGRYGDVPKLPILPKSHGFEIFRGKILHTMEYAMLDDRAACELVQSKRVVVVGMMKSALDAAAQIAIANKGTTNGQQSCTMVFRTPHWMLPHHKPFGLPLSYLYSTRLSELMIAKPDQGPFLDVVSTLLRPVCGALFNSVSAHMPAEMGDLKARGVIPPAQFTPVRFASCQVPLLPDNFFEDVKNGHIQLRRTSQWGFTERGVMLEDGTHIEADVVILGTGYDGEKKLRSLLEDVTSKDGFPPEPGNVVPLYRGIIPPEIPHLAIMGFYENFSNLHSSEMGARWLSHLVSGKISLPPLPSMVRAVQQWIQYMKSSTPFYQKSCVGAVSIWHSDQMCCDLGWNPKRKTNFLRDLLVPYSNMDYRD

>TnS000517369t01|Gnetum_montanum/1-532

MASGSGDNIFLNRRACIVGAGLSGLLAAKYLLQHNIKPVVFEAKENVGGVWRQTFSSTKLQTPRLGFQFSDFPWPQDVTADFPGHDEVVDYLERYAQHFGVLDCVRFNSKVVKVQQSNKPGLASVGIWGKNGGPFPDDAVWEVGVQNVQTASYEFENQIEWQYFDFVVLCIGKYGDLPKLPSFSGRKGPEIFQGTVLHALDYSALNDKAAFDLIKGKRIAIIGCQRSAVELAMECAEANPDGPPCTLVFSRAHWKFAEILVWGVPVSYFIDSRIAQFIIAKPGKGFLHELQWKLLTPLRWAVSKIVELYLLWTLPLKKYGLDPQHSFIEDFSSSNICMLPDGFFAMAEKGCLRFQRAPSWTFVEKGILVGDGSEVEADIVVMCTGYDADTKLKRILSQPLVNILEQSDDLIPLYRGIIHPRIPHMAILGYSESISNLFSTEMRSRWLACFLSEKITLPNVQEMEDDIEKYNLLMKRLSRFYAKSSHAAFHIWQNGDICRDMGWNPYRKQNWWSELFTPYSNYDYQIPPMEAN

>TnS000202953t01|Gnetum_montanum/1-526

MEEKPFSERKICVVGAGVTGLATTKYLLEHGFKPVVFETQDGVGGVWQQTFSSTRLQSPRMIYEFHDFPWPSSVTAMDPTSAEVLNYLESYAKNFGLLDYIHFNTKVATIQHLNGGTAFGIESSGLWGRNGGAFAEGAVWEVGVQKVGNLAEKSENIEWHCFDFVVLCIGKYGDLPKTLEFPPNKGPEMFQGTLIHSMGYSTLKKSDQYDLIHGKKVVIIGIMKSAMDLAVECAEINPDGLPCTMVFNRTQWNASTFFAWGVPFACLIDTRLAQIIIGKPGKGFWQQLVWHLLTPMRWAISKFVELYLLWTHPLHKYGLVPESSFLESISSCHFAIIPKEFFPKTVADLIQFRKTEKWYFTPRGISLNDGTELEADVVITCTGFDVHQKMASMLSQPLVNILKKSTDLLPLYRGVIHPQIPHMAILGYSESLSNLASSEMRARWLAYFLSGKFMLPSIKEMEDDIDKYNHHMKRISRFYWKCSHSVYHIWQNDDICRDMGWNPHRKSNWLTELLSPYTIHDYQNKK

>266739|Smoellendorffii_v1.0/1-494

MEKLGVCVVGAGVSGLCACRHLLKRGIRPTVLEGSSHIGGVWRSNSVAITRLQTPSVAYQFSDFPWPPGTPAFATGGQVLDYLHGYAAKFGVLECVRFDSRVVGIRRDGDRGPWQLLVVRGDGDGAARETLEFDFVILCIGRFGDVPRLPSFSPGQGADVFKGRVLHSTEFSALDYENASKLLRGNSVVVVGFSKSALDVAAEAVEANQGKPCTLLFRRAHWIVQHWDVFGVNIAYLFGTRLSEMYYHKPGQGFFSSLLTSLLWPLKWATSKVVEVYLRWTLSLSKYGILPSIPFYKQLSSCKISVIPDNFMDYVKEGRLKLCKSPKWHFTATGVMLDNKRELKADVVLLCTGFEGEVKLKAILPERYAEVLQGPDGVVPLYRGMIHPRIPGLAILGYQEDVSNVRASEMGARWLSYYLTGKVKLPDSQTMSKSANEWVRYMKWSTPYFDRACIALARTWHSDELCRDIGWNPRRKKNWLQELLSPYSNMDYID

>evm.model.Chr10.1479|Isoetes_sinensis/1-519

MGAKKICIIGAGMSGLCACKHLLCHGLDPVVLEAEDEIGGVWRHTFSTTKLQTPSLAYQFSDFPWPRNTPECPSRDQVMDYFRNYAVQFDVLPRIRFGCKVTKISFLGSLKSSNLNIWGYNGSALHPHDDDVWQVPFQEKKGQETTEKVLYFDFVILCVGRFGDVPNLPAFNPPEDYKGKLMHSMEYSTLGAEEAKDLVSGKRVVVVGFQKSALDLAAEVADANQGVLGHPCTLVFRKPHWIMPHLHPCGINAMYLCGTRLWELANDKSNQGWPLDMLSLLLFPWKWAISKFVELYPKVNFPLSKYNIVPSHSFHEQLSSCLISILPDSFFKKIEQGQIRLRKSPHWRYSNKGILLDDGTEVEADVIMLCTGYNGESKLRSILDEQFDENLQDASGVVPLYRGIIHPRIPHMAFIGYQEDVSNVRASDMAARWVAYFLKGKFYLPNIPTMEKDAQYWIHYMKQSTPFYRRACVSAIRIWHTDQLCRDIGWNPYRKGSWLQELLSPYSNMDYQDQPNSNM

>evm.model.Chr6.2528|Isoetes_sinensis/1-513

MGPKKICIVGAGMSGLCACKHLLGHGLDPVVLEAQDEIGGVWRRTFSTTKLQTPSLAYQFSDFPWPQNTPEFASRDQVMDYFYNYAVKFDVLPRIRFGCKVSKISFLGSVNSSNSGIWGYNSGAFHPHDDDIWQVIVQEKKGQEITEKVLYFDFVVMCVGRFGDVPNLPAFNPPEDYKGKVMHSMEYSALGAEEAKELVSGKRVVVVGFQKSALDLAAEVADANEGVLGHPCTLVLRNPHWIMPHFHPCGINLMLLCGTRFWELANHKPNQGWPLDMLSILLFPWKWAVSKFVELYLKLNFPLSKYNIVPTHSFHEQLSSCRTSVLPNNFFEKIEHGQIRLRKSPHWKYSNKGILLDDGTEIEADVIMLCTGYNGESKLRSIFHEEFAQNLQDASGVVPLYRGIIHPRIPHMAFIGYQEDVSNVRASDMAARWVAYFLNGSFHLPNIAKMEKDAQCWIHYMKQSTPFYRRACVAAIRIWHNDQLCRDIGWSPYRKGSWLQELFSPYSNMDYQD

>evm.model.Chr5.2478|Isoetes_sinensis/1-523

MGPKKICIVGAGMSGLCACKHLLGHGLDPVVLEAQDEIGGVWRRTFSTTKLQTPSLAYQFSDFPWPQNTPECASRDQVMDYFYNYAVKFDVLPRIRFGCKVSKISFLGSVNSSNPGIWGYNSGAFHPHDDDIWQVIVQEKKGQEITEKVLYFDFVVMCVGRFGDVPNLPAFNPPEDYKGKVMHSMEYSALGAEEAKELVSGKRVVVVGFQKSALDLAAEVADANEGVLGHPCTLVLRNPHWIMPHAHPCGINLMLLCGTRFWELANHKPNQGWPLDMLSILLFPWKWAVSKFVELYLKLNFPLSKYNIVPTHSFHEQLSSCLISILPNNFFEKIEHGQIRLRKSPHWKYSNKGILLDDGTEIEADVIMLCTGYNGESKLRSIFHEEFAHNLQDASGVVPLYRGIIHPRIPHMAFIGYQEDISNVRASDMAARWVAYFLNGSFHLPNIAKMEKDVQCWIRYMKQSTPFYRRACVGAIRIWHTDQLCRDIGWNPYRKGSWLQELFSPYSNMDYQEEKTHHLILIS

>AsFMO-BX|Aphelandra_squarrosa

MQMKKTVAIVGAGISGLLACKYALSIGLNPVVFEAESCVGGLWNHTIECTRLQSLKDYFQFSDFPWPSTIKTMFPKNEQVLEYVNSYADHFQLRQYVRFNSQVMSIGYEGESKEEMGSWKSWGGTGMAFGSKGKWRLQVEAGGGMQEYHVADFVIVCIGKFSGLPAVPDFPPGSGPEVFSGKVLHSMEYSDMDNASAAQFIKGKRIAVIGSGKSAIDIAFECGRANGRDTPCTVIHRNAHWMLPYEKPWLLAFALLCFTRFSELLVHKPGEGFFASVLATLLSPLRWLVSKFLEISLRWRLPLKKYDMIPSENFLEEANSCQVIMLQDNFFDKVVDGSIVFKKSEHFSFCKEGLIVEGEDSPVEVDIVIFATGYKGDEKLKNIFASPAFQNHIFGSRNSTVPLFRQMIHPRIPQLAVVGYSESLSNLYTFEMRCRWLAFFLDRAFALPSIKEMEDDIKMWERYMKKYAGNGEFKRGCIAGIHIWYNDQLCKDIGCSPRRKKGISSELFQPYGLEDYDGITPAQINPSYFAK

>LgFMO-BX|Lamium_galeobdolon

MLMEKRVAIIGAGISGLLACKYTASIGLKPIVFEEQDNVGGLWNHTIEITRLQQVKQSFQFSDFPWPSSADDNPKSAQLLEYLQSYAHNFDLLPFIKFNSKVMNIDYVGESKEEMQSWDLWGGSGKAFGSKGRWILKVLHTKDDSIKVYESEFVVVCTGRFSGLPNIPEFAPGYGPEIFSGQALHSMDYSNMDNASAAQFIKGKRIAIIGSGKSAFDIAFECANANGNDNPCTLVQRTIHWTFPSTKTWGLNFAFLIFTRFSELMVRKPGQGFVSSALTTLLTPLRWGMSKYVESYLRWKLPLKKYGMVPKESFAAESSACQIFYLPRGNFYGKVEDGSIVLKKSQEFRFCKHGLIIDGDDKNPINADIVIFATGYKGDEKLKSIFASPTFQNYIVGSPTSTVPLYRQMIQPRIPQLAVIGYSESFSNLFTFEMRCKWLAFFLDQAFELPSIRAMKKEVEAWETYMKRYAGNNKFRRACIGRVHVWYNDQLCRDIGCNPRRKKGFLSELFEPYGSADYVGL

>evm.TU.supercontig_20.191|Cpapaya_ASGPBv0.4/1-521

MEKQIAIIGAGLSGLLACKYVLSKGLQPIVFESRTDVGGVWIKTIQTTKLQTPKSIYQFSDFPWPSSVPEDFPDHDKVLDYIKSYAEHFDLIKHIKFNSRVVGIEYEGASGSEEIEKWSLWSGNGERFGSGGKWKVVVEHNHGVADETKIYKVDFVILCVGRFSDVPNIPEYPPGKGPETFHGKVIHSIDYTKMEYETAGKFVEGKRVAIVGFQKSALDIAMEVAAVNGKQNPCTVLYRSEQWGVPDYLPWGLPLAYLYLTRFSELLLHKPGEGLLLSLLASILSPVRWAFSKFVETHIKRKLRLEKFGMVPEHSFFNQINTCLVSTVPENFYEKVEEGSIILKKAPSFSFCKEGIMVDGETKPIETDLVIFATGFSGDKKLKDIFMSKTFQDSILGSTNAAVPLYRECIHPRIPQLAVIGYSESVSNLYTSEIRSKWLAELLGGTFKLPSIKDMEEDVAKWDKHWKRYSGKYYRRSCIAAIHIWYNDQLCKDMGWNPKRKKGFFADLFEPYGPTDYLVSS

>orange1.1g009917m.g|Csinensis_v1.1/1-522

MEKKQIAIVGAGVSGLLACKYLLLKGFHPIVFEARSDIGGAWIKTVETTMLQTPKQLYQFSDYPWPDSVTTDFPDHNQVLDYIQSYASHFDLRKHIKFNRKVVGIEFEGLSGEDEKSWSFWNGNGQPFGSRGKWTVAVEDAKNHSTEVHQVDFVILCVGRFSDVPNIPEFPPKKGPEAFHGKVIHSMDYSDMDYEAAANLVKGKRVTVVGLQKSALDIAMECTTANGLENPCTVLYRTEHWNIPDYFPWGFPLAYLYLNRFAELLVHKPGEGFLLSLLATMLSPLRWAISKFVEADIKKKHRLAKFGMVPKHSFLQELSSCLTITVPEKFYDKVEEGSIILKKSQDFSFCEDGIVVDGQTTPLKTDLVILATGFKGDVKLKNIFLSQTFQDYLAGSPTEKLPLYREIIHPQIPQLAIIGFSESISNLYTSEMRCRWLAELLDGAVKLPSIKEMEEDMRKWDEYMKRYSGKYYRRSCIGALHIWYNDQLCKDMGWNPKRKKGWFAELFEPYGPVDYAPPSTSN

>Eucgr.G01845|Egrandis_v2.0/1-518

METKKIAIVGAGISGLLACKNTLSKGLHPIVFESEAGIGGVWTKSVETTKIQTPKPAYQFSDFPWPDSVAEDFPDAHQVLGYIESYARHFDLLKHIKFGTRVLSIGYEGPPVEAMQSWSLWGGDGRPFSSGGKWKIRVQDCLSLETETYEVDFVILCAGRFSGIPNIPEFPPNRGPEAFHSKVIHSMDYAAMDYQAAKEFVSDKKVVVVGLQKSALDIAMECSAVNGVERPCTVLYKTEHWNLPDYLPWGVPLGLLYLNRFSELLVHKPREGLLLSLLATLLSPLRWAISKFVESHIKRKLQLKKFGMVPKHSFLREISSCLISTVPEKFYDRVEEGSIILKKAPAFGFCKNGISIDGEVEPLNSDLVILATGFRGEKKLLDLFESQLFQDCLSGSPNSIVPLYRECIHPRIPQLAVIGFSESIANLYTSEIRCRWLAELLAGTFKLPSVKEMEEDIEEWEKYMKRSSGPYYRRSCIAALHIWYNDQLCRDMGWNPKRKKGFFAELFEPYGPTDYVSP

>Eucgr.G01843|Egrandis_v2.0/1-518

METRKIAIVGAGISGLLACKYTLSKGLHPIVFESETSIGGVWTKTVETTKLQTPKPAYQFSDFPWPDSVTEDFPNGHQVLEYVESYACHFNLLKHVKFGTRVLSIGYDGPPAEAMQSWSLWGGDGMPFRSGGKWKIRVQDCLSLETETYEVDFVILCVGRFSGIPNIPEFPPNRGPEAFHGKVIHSMDYAAMDYQAAKEFVSGKRVVVVGLQKSALDIAMECSTVNGVERPCTVLYKTEHWNVPDYLPWGVPLALLYLTRFSELLVHKPREGLLLSLLATLLSPLRWAVSKFVESDIKRKLPLKKFGMVPKHSFLQEISSCAISTVPEKFYDRVEEGSIILKKALAFRFCEDGISINGEVEPLNSDLVILATGFRGEKKLVDVFESQLFKDCLSGSPNSIVPLYRECIHPRIPQLAVIGFSESVANLYTSEIRCRWLAELLAGTFKLPSIKKMEEDIEEWNKYMKRSSGQYYRRSCIGALHIWYNDQLCRDIGWNPKRKGGFFAELFEPYGPTDYVSP

>Prupe.8G177000|Ppersica_v2.1/1-514

MGKQVAIIGAGISGLLACKYTLSKGFQPIVFEASSSIGGVWTKTVETTRIQSPKDYYQFSDFPWPSSVTEGFPTQNQVLDYVKSYAQHFDLLKHIKFNTKVCGIEYEGPSSEDEMQAWSLWGGNGEPFSSEGKWKVVAEDKHSLSTDIHLVDFVILCLGRFSDVPNKGPEAFHGEVIHSMNYADMDYISAANLVKGKQVTVVGFQKFAMDIAMECSNANGVELPCTVIYKTEHWNLPDYLPWGLPLAYLYFSRFSELLVHKPGEGLLLSLLATILSPLRWAFSKFLESHVNKKLGLAKYGMVPMHSFLQEISSCMIATVPEKFFDRVQEGSIILKKSPSSTFSFCQEGILVEGESSPVKTDLVIMATGFRGEKKLKDVFVSPTLQDQIVGSPETILPLYRECIHGRIPQLAVIGFSESISNLFTSEMRCRWLSELFVGTFKLPSIKEMEKDVEKWDKYAKQYASGQYYRRSCIGALHLWYNDQLCKDMGWNHKRKKGVFAELFEPYGPMDYVTS

>Prupe.8G177100|Ppersica_v2.1/1-528

MGKKVAIIGAGTSGLLACKYTLSKGFQPIVFESASTIGGVWTKTIETTKLQTPREAYRFSDFPWPSSVTEDFPNQRQVLDYIQSYAHHFDLLKHIKFNTKVCGIEYEGSSEDEMQAWSLWGGSGEAFSSKGKWKFVVEDKQSLSTEIHVVDFVILCIGRFSDVPNIPEFPSNKGPEVFDGEVIHSMDYAAMDYESARRFVRGKQVTVVGFQKSALDIAMECSNTNGLEHPCTVLYKTEHWNVPDYLPWGVPLAYLYLNRFSELLVHKPGEGILLSLLATILSPLRWAFSKFVESYITKKLGLAKYGMVPKHSFLQAISSCLISTVPEKFYDHVQEGSIILKKSPSSFSFCREGILVEGESSPVKTDLIILATGYRGDKKLKDIFVSPTFQDYIAGSPNATLPLYRECIHGRIPQLAVIGFSESVSNLYTSEMRCRWLAELLGGTFKLPSIKEMEKDVEKWDEYAKRYSGKYYRRSCIGALHIWYNDQLCKDMGWNPKRKKGLLAELFEPYGPTDYVSLKMGSSDIKDD

>FvH4_2g16450|Fvesca_v4.0.a2/1-475

MGKKVAIIGAGISGLLACKYTLSKGFQPIVFEASSSIGGVWTETIESTKLQTPKALYEFSDFPWPSSVTEDFPNQHQVMDYIQSYAQHFDLLKHIKFNTKVCGLEFEGPSEDELQSWSLWGGTGEPFNTKGKWKVTVEDKKSPSTEIYLVDFVVLCIGRFSGVPNIPQFPLNKGPEEFHGEVIHSMEYAAKDYESATEFVKGKQVTVVGFQKSALDIAMECSNANGIENPCTVLYKTEHWHVPDYLPWGVPMALLYLNRFSELLVHKPGEGLLLCILATILSPLRWAFSKFVETYIDKKLGLSKYGMVPKHSFLQEISSCLISTVPEKFYDRVQEGSIVLKKSPKSFSFCQEGILVEGDNSGPVKTDLIILATGYRGDEKLKDIFVSPTFQYYVAGSPKSTLPLYRECIHARIPQLAVIGFSESISNLYTSEMRCRWLAELLGGTFKVPSIKEMEKDVKRWDEYAKRYTGKYYRR

>FvH4_2g16430|Fvesca_v4.0.a2/1-519

MDKKVAIVGAGISGLLACKYTLSKGFRPIVFESTSTIGGVWTKTVETTKLQTPKAFYQFSDFPWPSSVEEDHPNHNQVLEYIESYARHFDLVRHIKFNTKVCGMEFEGPSEDEMQSWSLWGGTGEPFSSEGKWKVIVEEKQSLSSKTYLVDFVILCIGRFSDVPNIPEFPLNKGPESFHGKVIHSMDYANMDYERAAKFVTGKQITVVGSQKFAIDIAMECSNANGIENPCTLLYRTEHWNIPDYFPWGMSLALLYFNRFSELLVHKPGEGFLLSLLATLLSPLRWMFSKFVETYASKKLGLYKYGMIPKQSFLQDISSCVVAIVPEKFFDRVQEGSIVLKKSSASFGFVEEGILVEGDASTLIKTELVILATGFRGEKKLKDIFVSPTFQDYITGPKDAILPLYRQCIHGRIPQLAVIGFSESISNLYTSEMRCRWLAELLAGTFKVPSIKEMEKDVKKWDKYAKQYAGEYYRRSCIGALHIWYNDQLCKDMGWSHKRKKGMFAELFEPYTPMDYVSP

>Qrob_P0638570.2|Quercus_robur/1-517

MERKVAIIGAGISGLLACKYTLSKGFHPIVFEAKSNIGGVWTKTIETTRLQTPKVLYQFSDFPWPSSVTEDFPKQQEVFDYIQSYAHHFDLLRHIKFNTKVVSIEYESLSEVEMQSWTMWSGTDAFLSSKGKWKLMVEDTSSLSTEEYLVDFVILCIGRFSDIPNILGFPPNQGPEVFHGKVIHSMEYAAMDYESAAKLIKGKQVTVVGFQKSALDIAMECSMANGIEHPCTVLYKTEHWIVPDFFPWGVPLPLLYLNRFAELLIHKPGEGFLLSLLATILSPVRWAFSKFVESYIRKKLRLAKLGMVPKHSFLQQLSSCLICIMPEKFYDKVEEGSIILKKGTNFSFCKGGVMINGEEQLLKTDLVILATGFRGDRKLKDIFVSPTFKDHMAGSPNTTVPLYRECIHPRIPQLAVIGFSESVSNLYTSEIRCRWLAELLDGTFKLPSIKDMEKNVEIWDKYMKTYSGQYYRRSCIGAIQIWYNDQLCKDMGWNPKRKKGLIAELLEPYGPLDYVSS

>VIT_203s0038g03130|Vvinifera_v2.1/1-517

MERKVAIIGAGISGLLACKYTLSKGFTPVVFESRSSIGGVWRKTLETTKLQTPKPFYQFSDFPWPSSVKEEFPNQFQLFDYLQSYARHFDLLRHIKFNSKVVSIEYEGASDEEMQAWELWGGNGEAFSSKGKWNLTVTDTQILSTEVYQVDFVILCIGRFTDVPNIPEFPPGKGPEAFHGKVIHSMEYSAMDFASAAEFIEGKRTAVVGFQKHALDISMECSAANGEQNPCRLLYKTEHWNVPDYQPWGVPLAYLYFSRFSELLVHKPGEGFFLSLLATILSPVRWGFSKWVESHIKKKLSLEKFGMVPKHSFLKEISACLISTVPEGFYDRVEKGSIILKKAPKFSFCEQGIVVDGETTPIETDLVILATGFKGEKKLKDIFVSPAFQDCILGSPTASVPFYRECINPRIPQLAVIGFSESISNLYTSEMRCRWLAELLDSTFKLPSIKEMEKDAARWDKYMKQSSGEYYRRSCIGALHIWYNDQLCKDMGWNPKRKKGFFAELFEPYVPSDYCPS

>VIT_203s0038g03170|Vvinifera_v2.1/1-517

MERKVGIIGAGISGLLACKYTLSKGYTPVVFESRSSIGGVWTKTLETTKLQTPKPIFQFSDFPWPSSVVEDFPSHSQVLDYLQSYVHHFGLLRHIKFNSKVLSIAYEGPPEEEMQAWELWGGAGEPFGSKGKWNLTVNDTHNLSTEVYQVDFLILCIGRFSDVPNIPEFPAGKGPEMFHGKVIHSMEYAAMDFERAAEFIKGKRTTVVGFQKHALDIAMECSTANGGEHPCRVLYKTEHWTVTDYQPWGVPLAYLYLNRFSELLIHKPGEGFLLSLLATILSPVRWGFSKFVESYIKRKLSLEKFGMVPKHSFLNEISSCLISTLPEGFYDRVEEGSILLKKAPKFGFCKEGIVVDGESTPLETDLVILATGFKGDEKLKDIFVSPTFRDYIIGSPTASLPLYRECIHPRIPQLAVIGFSESVSNLYTSEMRCRWVAELLDGKFKLPSIKEMEKDAERWDKYMKQYSGEYYRRSCIGSVHIWYNDQLCKDMGWNPKRKKGFIPELFQPYGPMDYCPS

>VIT_203s0038g03150|Vvinifera_v2.1/1-517

MERKVGIIGAGISGLLACKYTLSKGYTPVVFESRSSIGGVWTKTLETTKLQTPKPIFQFSDFPWPSSVVEDFPSHSQVLDYLQSYAHHFGLLRHIKFNSKVLSISYEGPPEEEMQAWELWGGAGEPFGSKGKWNLTVNDTHNLSTEVYQVDFLILCIGRFSDVPNIPEFPAGKGPEMFHGKVIHSMEYAAMDFERAAEFIKGKRTTVVGFQKHALDIAMECSTANGGEHPCRVLYKTEHWTVTDYQPWGVPLAYLYLNRFSELLIHKPGEGFLLSLLATILSPVRWGFSKFVESYIKRKLSLEKFGMVPKHSFLNEISSCLISTLPEGFYDRVEGGSILLKKAPKFGFCKEGIVVDGESTPLETDLVILATGFKGDEKLKDIFVSPTFRDYIIGSPTASLPLYRECIHPRIPQLAVIGFSESVSNLYTSEMRCRWVAELLDGKFKLPSIKEMEKDAERWDKYMKQYSGEYYRRSCIGAVHIWYNDQLCKDMGWNPKRKKGFIPELFQPYGPMDYCPS

>VIT_203s0038g03140|Vvinifera_v2.1/1-517

MERKVAIIGAGISGLLACKYTLSKGYTPVVFESRSSIGGVWTQTFETTKLQTPRSVYQFSDFPWPSSVPEFPNHSQVLDYIVSYATHFNLLPHIKFNSEVLGIEYEGPSDEEVQAWELWSGTGEPFNSKGKWSITVKDTNSLSTEVYQVGFVILCVGQYSDVPKIPEFPAGKGPEAFRGKVIHSMEYAAMDFERAAEFIKGKRTTVVGFQKSALDIAMECSAANGALNPCRLLYRTEHWNIPDYYPWGVPLAYLYFNRFAELLVHKPGEGFFLSLLATILSPLKWGFSKFVETYVNQKLSLAKFGMVPEQSFHKDISSCLISSMPEEFYDRVEKGSIILKKAPKFSFCKEGIVVDGEASPLESDLVILATGFKGDEKLKDIFVSPTFQNHIIGSPNASIPLFRQCIHPRVPQLAVIGFSESVSNLYTSEMRSRWLAEFLDGTFKLPSIKEMEKDAERWDQYLKRCSEKHYRRSCIAALHIWYNDQLCKDMGWNPKRKKGFFAELFEPYGPLDYRPIS

>VIT_203s0038g03160|Vvinifera_v2.1/1-517

MERKVAIIGAGISGLLACKYTLSKGYTPVVFESRSSIGGVWTQTFETTKLQTPRSIYQFSDFPWPSSVPEFPNHFQVLDYIVSYATHFNLLPHIKFNSEVLGIEYEGPSDEEVQAWELWSGTGEPFNSKGKWSITVKDTNSLSTEVYQVGFVILCVGQYSDVPKIPEFPAGKGPEAFRGKVIHSMEYAAMDFERAAEFIKGKRTTVVGFQKSALDIAMECSAANGALNPCRLLYRTEHWNIPDSYPWGVPLAYLYFNRFAELRVHKPGEGFFLSLLATILSPLKWGFSKFVETYVNQKLSLAKFGMVPEQSFHTDISSCLISSMPEEFYDRVEKGSIILKKAPKFSFCKEGIVVDGEASPLESDLVILATGFKGDEKLKDIFVSPTFQNHIIGSPDASIPLFRQCIHPRVPQLAVIGFSESVSNLYTSEMRSRWLAEFLDGTFKLPSIKEMEKDAERWDQYLKRYSEKHYRRSCIAALHIWYNDQLCKDMGWNPKRKKGFFAELFEPYGPLDYRPIS

>Potri.009G143500|Ptrichocarpa_v4.1/1-518

MEKQVAIIGAGVSGLLACKYTVSKGFNPIVFEARNSIGGAWINTVETTKLQTPKPAYQFSDFPWPDSVTELFPNQYQVLDYLQSYAHHFDLLKHIKFSTKVLGIKYQGASDEEMQSWSLWGGNGEPFSSRGKWIVEAQDTQNQLSTEAYQVDFVILCIGRFSDVPNIPEFPPDKGPEAFHGDVIHSMDYANMDCESATDFVRGKRVTVVGSQKSAMDIAMECSIANGVEHPCRVLYRTEHWNIPDYNPWGVPLPYLYLNRFSELMVHKPGEGFLLSLLATILAPLRLAFSKFVESDIEKKLRLAKFGMVPKHSFLQELNSCLIATVPQGFYDKVEEGSIILKKAPSFSFCKEGIKVQGEDTTLETDLVILATGFKGEKKLKDIFESKMFQDCILGSPDSAVPLCRECIHPRIPQLAVIGFSESVANLYTSEMRCRWIAELLDSTFKLPSIKEMEKDAEKWDQYRKQYSGRYYRRSCIGALHIWYNDQLCKDMGWNPKRKKGFFAELFEPYGPADYVSP

>Hyque.03G206000|Hquercifolia_v1.1/1-518

MERKVGIIGAGISGLLACKYSLSKGYHPIVFESQSSSGGVWTKTFETTKLQIPKSLYQFSDFLWPSTVEEDFPNQHQVFDYIQSYATHFDLLRHIKFNSKVISISYEGPSDEEMQAWSLWGGTGEAFSSKGKWNISVQDTLTLSTDQVYQVDFVILCVGRFSGLPNIPEFPPNRGPEAFQGKVIHSMDYAAMDYASAADFVKGKQVAVVGLQKSALDIAMECSSANGVEHPCTVIYKTEHWNMPDWLPWGVNVGYLYLNRFAELLFHKPNEGFLLRLLATILSPLTWGFSKFVESYIKHKLQLAKYGMVPKHSFFKELSSCSISIVPEDFYNRVDKGSIKLKKAPSFSFCKDGILVDGEVEPLKTDMVILATGFKGVQKLKDVFESPTFQDYIVGSTDTTVPLYRECIHPRIPQLAIIGYSESVVNLFTSEMRCRWLTELLDGIFKLPSIKDMEKDVAKWDEYMKLYSGQYYRRSCIGALHIWYCDQLCKDMGWNPKRKKGLFAELFEPYGPLDYAHP

>Hyque.03G206100|Hquercifolia_v1.1/1-517

MERKVGIIGAGISGLLACKYSLSKGYHPIVFESQSSPGGVWTKTFETTKLQSSKFLYQFSDFLWPSSVEEEFPNQHQVFDYIQSYATHFDLLRHIKFNSKVISISFEGPSNEEMQAWSLWGGTGEAFSSKGKWNISTQDTQSLSTEVYQVDFVILCVGRFSGLPNIPEFPPDKGPEAFQGKVIHSMDYAAMDYASAADSVKGKQVAVVGLQKSALDIAIECSIANGVKHPCTVIYKTEHWNVPDYLPWGFNLGYLYLNRFAELLIHKPGEGFLLSLLATILSPLRWGFSKFVESYIKHKLQLAKYGMVPKHSFLKEVSSCLISTVPPDFYNRVDQGSIKLKKAPNFSFCKEGILVNGEAEPLKTDMVILATGFKSVQKLKDIFVSPTFQDYIAGSTNTTVPLYRECIHPRIPQLAIIGYSESVANLFTSEMRCKWLTELLDGTFKLPSIKDMEKDVAKWDENMKLYSGQYYRRSCIGVVHIWYCDLLCKDMGWNPKRKKGLFAELFEPYGPLDYAQP

>Rhdel05G0054700.1|Rhododendron_delavayi/1-518

MERKVAIIGAGISGLLACKYALSKGHRPIVFEAQSGLGGVWTKTVEITKLQIPKPFYQFSDFPWPDSVTEVFPDQNQVLEYIESYARQFDLLHHIRLDTKVVSIGYEGASSEVQQAWALWGGTGEPFDSKGGKWYITIQDTKSLSTEVCQFDFVIICVGRFSGIPNIPEFPQGKGPEVFQGKVIHSMDYAAMDYATAKELVKGKRVAVVGLQKSALDIAMECSTANGMDLPCTVIYRTEHWNIPDFQPWGVPLAYLYLNRFSELSIHKPGEGPLLSLLATLLSPVRWAFSKFVESHVKHKLKLVKFGMVPKHSFLQELSSCLTSTVPDDFYNRVEKGSIRLKKAPSFSFCKEGILIDGKTETLKADLVILATGFNGVKKLQDIFASSSFQDLIAGSPDKTVPLYRECIHPRIPQLAVIGFSESFANLYTSEMRCRWLAELLDGTFKLPSIKEMEKDIAEWDEYMKQYSDKNYRRSCIGALHIWYNDQLCKDMGWNPKRKKGIVAELFEPYSPMDYACP

>Gohir.A01G173900|Ghirsutum_v3.1/1-517

MERKIGIVGAGVSGLLACKYILSKGFHPIVFESQSSIGGVWTKALETTKLQTPKPFYQFSDFAWPLSVTEYFPDKHVVLDYIEAYAKHFHLLNHIKFNTKVAGIEYEGFQDEEIQSWSLWGGTGEPFSSRGKWKLIVEDLNTLSTEIYQVDFVIFCVGRFSGLPNIPEFPPNKGPEAFHGKVIHSMEYAAMDDEKAAEFIKGKRVIVVGFQKSALDIAMECCAANGVENPCTVLYRTAHWHLPDYFKRGFSLAYMYFNRFSELMVHKPGEGFLLGLLATILTPLRWAYSKFVESDIKRKFHLAKHGMIPTHSFLHELSSCLTSTVPEKFYDKVEGGEIKLKKAPSFSFCDNGVMVEDETAPIEAEVVILATGFKGEKKLKDMFVSQTFQNFIAGSPDTATPLYRECIQPRIPQLAVIGYSESNANLYTSEIRCRWLAELLDGTFKLPSIEEMEKDVRKWDEYLKQYSGGYYRRKCIGALHVWYNDQLCKDMGWNPRRKKGLFAEFFEPYGPLDYVPS

>Gohir.A01G174101|Ghirsutum_v3.1/1-332

MEYAAMDDEKAAEFIEGKRVIVVGFQKSALDIAMECCSANGVENPCTFLHRTAHWHLPDYLPWGFSLAYMYLNRFSELMVHKPGEGFLLGLLATILTPLRWAYSKFVESDIKRKLHLAKHGMVPTHSFLHEISSCLTSTVPEKFYDKVEDGEIKLKKAPSFSFCDNGVMVEDETAPIEAEVVILATGFKGEKKLKDMFVSQTFQNFIVGSPDAATPLYRECIQPRIPQLAVIGYSESIANLYTSEIRCRWLAELLDGTFKRPSIEEMEKDVRKWDEYLKQYSGGYYRRKCIGALHVWYNDQLCKDMGWNPRRKKGLLAEFFEPYGPLDYVPS

>Gohir.D01G165300|Ghirsutum_v3.1/1-332

MDYAAMDDEKAAEFIKGKRVIVVGFQKSALHIAMECCSANGVENPCTVLYRTAHWHLPDYLPWGFSLAYMYLNRFSELMVHKPSEGFLLGLLATILTPLRWAYSKFVESDIKRKLHLAKHGMVPTHSFLHEISSCLTSTVPEKFYDKVEDGEIKLKKAPSFSFCDNGVMVEDETVPIEADVVILATGFKGEKKLKDMFVSQTFQNFIVGSPDAATPLYRECIQPRIPQLAVIGYSESIANLYTSEIRCRWLAELLDGTFKLPSIEETEKDVRKWDEYLKQYSGGYYRRKCIGALHVWYNDQLCKDMGWNPRRKKGLFAEFFEPYGPLDYVPS

>Gohir.D04G170200|Ghirsutum_v3.1/1-517

MDRKVAIIGAGISGLLACKYTLSKGFHPIVFESQSTVGGVWTKTVETTKLQTPKPVYQFSDFPWPESVSEDFPDGQKVFDYVNGYAKHFDLVKHIKFNTKVVGIEFEGPNDEEIQAWSLWGCNGEPFGSKGKWKVVVEDLVTLSTEIYNVDFVIVCVGRFSGLPNIPEFPPKKGPEAFDGKVIHSMEYAAMDHKQAAEFVKGKRVIVVGFQKSALDIAVECSAANGKEDPCTVLYRTAHWNVPDYLPWGFSLAHMYLSRFSELMVHKPGEGLLLSLLATILTPLRYAYSKFVESDIKKKLRLEKHGMVPTHSFLKEISSCLISTVPEKFYDKVENGEIKLKKAPSFSFCNNGVLVEGETSPIEADLVILATGFKGEKKLKHIFMSRTFQDYITGSPDAALPLYRECIQPRIPQLAVIGFSESISNIFTSEMRCRWVAELLDGTFKLPSIKDMEINIKEWDEYLKQYSGGYYRRKCIGALHIWYNDQLCKDMAWKPRRKKGFFAELFEPYGPMDYVSN

>TESI_007740-RA|Tetracentron_sinense/1-523

MERRVAIIGAGISGLLACKYSLEKGLRPIVLEAKSSIGGVWTKTVETTKLQTSKEYFRFSDFAWPSSVEQEFPKQSEVLEYLESYARHFDLLRHIKFDSKVIGIDHEGPSDEEMLPWTLWGGTGEPFSPTTSGKWNVSVQDTHGPSTQVYQVEFVILCIGRFSDVPNIPKFLPDKGPEVFDGKVIHSMDYSAMDYASAAKFIKGKRVTVVGFQKSALDIAMECSTANGLDHPCTVLYKTNHWNVPNYLPWGVPLALLYLNRFAELMIHKPGEGLLLSLLATMLSPVRWAFSKFVESYIRWKLPLKKFGALPEHSFFKEITSCLTSTVPEDFYDRAQKGSIVLKKSPSFSFCKGGIIIDGEVAPLETDIVILATGFRGDKKLKNLFMSPTFQDYVMGSPTTTVPLYRSILECIHPRIPQLAIIGFSESLSNIYTSEVRCRWLAELLDGTFKVPSIREMEEDVTKWDENMKRYSGQYYRRSCVAALHIWYNDQLCKDMGWKPKRKNGFLAELFQPYGPTDYTTLD

>Aqcoe6G186400|Acoerulea_v3.1/1-519

MEKRVAIIGAGISGLIACKYMSAKGFNPVVFEVKDSIGGVWTRTVETTKLQTPKDAYQFSDFPWPSSVTEEFPDHNQVLEYIESYARHFDVLQYIKFQTKVLAVDYEGPSDEEMVAWDLWGGTGEPFSSKGKWNITVENTQNKSTEIYQVEFVILCIGRFSDIPNIPDFPPNKGPEVFGGKVIHSMDYSDMDDASAADFVKDKRVTVVGFQKSALDIAFECAVVNGVKNPCTLIYRTEHWIVPNYLPWGVPLAYLYVNRFSELMVHKPGEGFLLSLLATLLSPVRWGFSKFVESYIKWKLPMKKNKMIPKHSFSQEISSCLISTIPRNFYDKVEEGSLILKKSIAFSFNKDGLMIDGETAPLETDMVIFATGYRGDVKLKSIFTSSAFQKYIMGSSTTTIPLYRECIHPRIPQLAVIGYSESLSNLYTSEMRCRWLAQLLEGTFKLPSIKVMEDDALKWEKYMKRYSGKNYRRSCIGVLHIWYNDQLCKDMGFNPKRKKGIIAELFEPYGALDYGNLSE

>Litul.02G098100|Ltulipifera_YP108A_v1.1/1-521

MDRRVGIVGAGISGLLACKYMLEKGIRPIVFEARSSIGGVWRQTVESTKLQTPRDAYQFSDFPWPSNVEEDFPDGDQVMEYIQSYADHFDIVRYIKFNSRVVAIDYVGRPDEEMATCDQWAGTGEAFNSGGKWHISVQEGDQQSTEVYQVEFVILCIGRFSDVPNIPEFPPGKGPDAFNGQVIHSKDYSAMDNASAAILVKGKRVTVVGLQKSAYDIAAECADANGKELPCTVICRTVHWAVPHYLPWGVPLGLLYVNRFAELLVHKPGEGLLLSLLATLLSPVRWGISKFVESYLKWKLPLKKYGMVPKHSFHRQISSCQVTTIPNNFYDKVEEGSIILKKSQSFNFYKDGLIIDGGDTLLETDMVILATGYRGDQKLKDVFTSPFFRKHIMGSSTSTVPLYRECIHPQIPQLAIIGYSESLANLYTSEMRCRWLAHFLDGDFKFPSIRNMEEDVLRWERYMKRYSGEYYRRSCIGVLHIWYNDQLCKDMGINPRRKKGFFAELFSPYGPLDYKEISMVK

>Litul.02G098200|Ltulipifera_YP108A_v1.1/1-521

MDRRVGIIGAGISGLLACKYMLEKSIHPIVFEAQSSIGGVWRETVESTKLQTPRDAYQFSDFPWPSNVEEDFPNSDQVKGYIQSYADHFDIVRYIKFNSRVVAIDYVGPSDEEMATWDRWAGTSEAFNSGGKWHISVQEGDQQSTEVYQVEFVILCIGRFSDVPNIPEFPPGKGPDAFNGQVIHSMDYSAMDNASAAEMVKGKRVTVIGFQKSALDIAAECADANGREIPCTVICRTVHWTVPHYLPWGVPLGLLYVNRFSELLIHKPGEGLLLSLLATLLSPVRWGISKFVESYLKWKLPLKKYGMVPKHSFHMQISTCQLATLPNNFYNKVEERSIILKKSQSFNFYKDGLIIDNGDSNLETDMVILATGYKGDRKLKDIFTSPIFQKHVMGSLTSTVPLYRECIHPRIPQLAIIGYSESLSNLYTSEMRCRWLAHFLDGGFNLPSIRNMEEDIIRWERYMKRYAGEYYRRSCIGIVNIWYNDQLCKDMGINPRRKKGFFAELFSPYGPLDYKEISMVK

>GWHPBFSI011146|Chloranthus_spicatus/1-522

MERRVGIIGAGVSGLLACKYVLEKGFHPIVFEARSSIGGVWTQTVESTKLQTPKDDYRFSDFEWPSHVKEEFPRNEQVLEYLQSYARNFDLLRRINFNSKVISVDFVGTSDEEQKSWNLWAGNGEAFNPRGKWHITVEDIIRHSSEVHQVEFLILCIGRFTDVPNIPIFPPNKGPESFDGKVMHSMGYSAMDDADAADLVKGKKVTVVGFQKSAVDVAAECAIANGVEKPCTVICRTVHWTVPNYLPWGVPIGNLYCTRFSELLLHKPGEGLLLSLVATMLSPLRWAISKFVESYLRWKLPLKKYGMIPKNSFFQQISTCLLTTLPQNFYDRVGEGSIILKKSQTLSFCKSGLIIDGQDAPIETDLVIMATGYKGDQKLKNIFTSPTFQSYIMGSLDGTVPLYRECIHPRIPQLAIIGYSESLSNPYTSEMRCRWIAHFLDGIFRLPSIKEMEDNVVEWEKYMKRYTGSYYRRSCLGLVNIWYNDLLCKDMGYNPKRKKGFLAEWFEPYGPLDYSDFSQPLN

>CKAN_00702900|Ckanehirae_v3/1-521

MDRRVGIVGAGIGGLLACKYMLEKGFHPIVFEAQSGVGGVWTHTIETTKLQTPKELYQFSDFPWPSSVKEDFPNSKKVLEYLESYAQHFDLLPHIKFNSKVVGVEYVGFSDEEMSSWDLWTGTGEAFNKGKWHISVQQANHESVEVYHVEFMILCIGRFSGFPNIPEFPPGKGPEVFDGKVIHSMEYAAMDNAIAAESVKGKRVAVVGFQKSAMDIAAECAQANGVEFPCTLICRTPHWNVPDYLPWGVPLALLYLNRFAELLFHKPGEGVLLGLFATLLTPVRWAFSKFVESYIRSKYPLKKYGMIPKHSFFQEISSCLTSTVPEDFYDNVEKGSIVLKKSQTLSFCKKGLIIEGEASPIETDMVILGTGFKGDQKLIDIFKSPAFQKYIKGSSTTTVPLYRECIHPRIPQLAVIGYSESLANLYTFEMRCRWLAYFLEGGFKLPSIKNMEEDVLKWEKYMKQYSHNYYRRSCVGAIHIWYNDQLCRDMGCNPKRKNGYFAELFSPYGPMDYCNLSPPKK

>GWHPBAUK007395.1|Macadamia_integrifolia/1-521

MAKRVAIVGAGISGLIACKYAVEKGFRLIVFEAGSSVGGVWTRTLQTTKLQTPKELFQFSDFPWPPNVLTSFPDHNQVLGYIESYANHFDLLRHVKFNSKVISIDYEGASEEEMQAWDLWGCTGEAFGQGGRWIVTVQDTTIQTTQVYQVEFVILCVGRFSDVPNIPEFPQNEGPEVFDGKVLHVMDYSDMEDASAAELIKGKRVTVVGFSKSALDIAAECADANGAEHPCTVVYRTEHWNVPHYLPWGVPLALLYLNRFSELMIHKPGEDLLLGLLVTLLSPLRWVFSKFVESYLRWKLPLKKYGMIPKHSFFQQIATCVVSTLPDNFYDKVTEGSIILKKSESISFCKDGLVINKDSMSTPLQSDIVILATGYRGDQKIKHIFTSPSFRKQVTGSSTTTVPLYRECIHPRIPQLAIIGYSESISNLYTSEMRCRWLVNLFDGGFKLPSIREMEKDVMRWENYMKRYTGKYFRGSCIGIVHIWYNDQLCRDMGYNPRRKNGFLAGWFEPYGPLDYTELSP

>AUR62012936|Cquinoa_v1.0/1-488

MERKVGIIGAGISGLLACKYALAKGYHPLVFESRNTIGGVWTQTVETTKLQTPKPLYQFTDFAWPSSVETVFPDQNQVFDYLQSYANHFDLLKHIKFNTKVVSIKYEGPSDEEIQSWALWGGNNDPFGRFSDLPNIPEFPSDKGPEVFDGKVMHSMDYSALDYQSARNLIKGKQVTVVGFQKSALDIAMECSVANGVEKPCTVICRTPHWNVPDYFPWGFPLAYLYLNRFSELLVHKPGEGLLLSLLASLLLPLRWSISKFVESDIKKKHRLKKFDMVPDHSFLQEMNSCLISTVPEGFYDNAEKGSIVLKRSQTFSFCKDGVLLDNHAKTKEPIKCDLVILATGYKGDQKLKDIFSSPKFQDYIIGSSNKTVPLYRECIHPRIPQLAVIGFSESIANLYTSELRCKWLFELLDSKFKLPNIQKMEKDVLEWDKFKKRSSGKYYRRSCIGALHIWYNDQLCKDMGLNPKRKKGAWAEMFEPYGPNDYA

>AUR62012935|Cquinoa_v1.0/1-177

RSPAFSFFEKGVELDDSIKSTEPITSDLVIFATGYKGDQKLKDIFASSEFKDYMFGSSNKTLSLYRECIHPRIPQLGVIGFSESLANLYTSEIRCRWLFELLDGKFKLPSIEEMEKDVIEWEKFMKRYSGKYYRGSCLGALHIYYNDQLCIDMGFNPKRKDGYWAELFEPYGPMDYA

>AUR62027133|Cquinoa_v1.0/1-518

MERKVCIVGAGISGLLACKYALSKGYHPIVFESRSAVGGVWTQTVETTKLQTPKPLFQFSDYPWPSSLETLFPDKNQVIDYLRSYAKHFDLLKHVRFNTKVVSIKYEGPSHEEIQSWALWSGNGDPFGNMGKWTVNTQDLLNQSTQVMEVDFVILCVGRFSDLPNIPQFPSGEGPEVFQGKVMHSMDYSALDYGSARNLVGGKRVTVVGFQKSALDIAMECSIVNGVEHPCTVICRTPHWNVPDLFPWGIPITSLYLNRFSEFLLHKPGEGPLLSLLATLLSPLRWGISKFVEFDIMYRHPLKKFDMVPDHSFLQEVSSCKLSIVPEGFYDNVEKGSIILKRSPAFSFYKEGVKLDYSIKSTKPIKSDLVIFATGYKGDQKLKDIFESSEFKDYMFGSSNETLSLYRKCIHPRIPQLGVIGFSESLANLYTSEIRCRWLFELLDGKFKLPSIEEMEKDIIEWEKFMKRYSGKYYRGSCIGALHICYNDQLCIDMGLNPKRKDGFWAELFEPYGPMDYA

>Sapof.11G145700|Sofficinalis_v1.1/1-522

MERRIGIVGAGISGLLACKYALAKGYNPILFESRNTIGGVWTKTVETTKLQTPKPFYQFSDFPWPTEIDTFFPNQTQVLNYLQSYANHFDLVKHIRFNTKVVSIKYDGPTDDEIESWALWGGNGDPFGNKGTWSITIEDVITHSTEVKQVDFVILCLGKYSDIPNIPEFAEGKGPEAFQGKVMHSMEYANMENDQAKALIMGKRVTVVGFQKSALDIAMECSTANGVEHPCTVICRTPHWNLPDYFPWGVPLANLYLSRFAELLLHKPGEGPLLAAVAALLSPVRWAISKFVESDIKKKHRLKKHGMVPDHSFTQELNTCLTSSIPEGFYDNADKGTLIFKRSPTFHFCKEGVQLDPNVKGTDLVVKSDLVILATGFKGDHKLKDVFVSPMFKAYIMGSPDKIVPLYRQCIHPRIPQVAAIGFSESVTNLYTFEMRARWLMELLDGKFKLPSIKNMEKDINQWDKFMKKYGGKYYRKSCIAGVHIWYNDQLCKDMGLNPRRKNGLWAELFEPYGPMDYAQKS

>GWHPBAUK025024.1|Macadamia_integrifolia/1-520

MERRVAIVGAGISGLLACKYVLEKGFRPVIFEAESEIGGLWNHTVETTKLQTAKQLYQFSDFPWPSSVKEDFPSANQVFEYVKSYATQFDLLRHIKFSSKVTGIDYQGASDEEIEAWDLWGGNGEAFGLGGKWNVTVLDLNSGSTEVYQVDFAILCLGRFSGVPNIPEFPTTKGPQVFQGNVIHSMDYSLMDNTTAIEFVKGKHVTVVGFQKSGLDIAAECAKANGVDRPCTVLYRNVHWYVTDYLPWGVSLGFLYLNRFSELLVHKPGEGLFLSIVATLLTPVRWAFSKFAESYYRSKIPMKKYGMIPEHNFFEQIVSCQLSTLPNNFYNKVEEGSIILKKSKSFSFCKDGLMVDEDDAPLKSDIIIFATGYKGDQKIKNIFTSPAFQDRAIGSPTTTVPLYRECIHPRIPQLAIIGYSESISNLYTSEMRCRWLAHYLDGVFKLPSIREMEKDILRWEKYMKRYTSKYFRGSCIGLTHIWYNDQLCKDMKCNPRRKKGFLAEWFEPYGPMDYANLSPY

>Aqcoe6G194500|Acoerulea_v3.1/1-523

MDRQVGIIGAGVSGLLACKYVLKAGYNPIVFEAKTSIGGVWAHTSETTKLQNHKQQYEFLDFPWPSSVKEDFPDHNQVMDYLESYARQFDLQQYIRFNCRVMAIDYEGVSDEEMVTWDLWGGTGDPIIPKGKWNITVEDTQIESTELEQVYQVEFVILCIGKFSDLPNIPDFPPNKGPESFNGKVIHSMDYSAMDDASAAKFIKGKRVAVVGIQKSGLDIATECAMANGIEHPCTVIYKRAHWNFPDFFPWGVPLAFLYFNRLAEFMIHKPGEGLILSLLATLLSPLRWLISKFVESYIRWKLPLKKYNMMPKHSWDKDFSAGTISITPEKFYHRVDEGSIILKKSTTFSFWKDGVMISDNNERIETDLVILATGYKGDQKLKNIFASPTFQKYIMGSSKSTVQLYRECIHPRIPQLAIIGYLESLMNLFTSEIRCIWLTHLLKGTFKLPRIIEMEKDVLRWERYMTQNTSEYYRRSCQSVMHIWYNDQLCRDMRFNPKRKKGFFVELLQPYGSKDYEDLSTH

>Aqcoe6G186200|Acoerulea_v3.1/1-518

MERKVGIIGAGISGLLACKYVLQMGFNPIVFEAQPGVGGLWNHTNKTTKLQTPKQAYQFSDFPWPSSVKEDFPDHNQVMEYIESYAHHFDLLQYIKFNSRVIGIEYKGASDEDMASWDLWGGTGASFSPLGKWNITVLKDIHRKSTQVYQVEFAILCIGRFSEVPNIPEFPPNQGPEAFDGKVIHSMHYAYMDDADAAKFVKGKRVTVVGIQKSALDIANECAMVNGVENPCMVLYRNAHWSFPHYFPWGVPFALLYFNRFAELMTHKPDEGFMLSLLATLLSPLRWLQSKFVESYLRWKLQLKKYHMIPNHSFNKDFSAGTIAVTPENFFQRVEEGSIILKKSATFSFCKNGLMIDGNTAPLETDVAILATGYKGDEKLRNIFVSSKIQKNIFGASNTTVQLYRECIHPRIPQLAIIGYSESFSNLFTFEMRCKWLAHLLRGTFKLPQIKEMEVDALRWEKYMKDHSGQYYRRSCNSSLDIWYNDQLCKDMKCNPRRKKGFFKELFEPYGPMDYANL

>Aqcoe6G186100|Acoerulea_v3.1/1-517

MERKVGIIGAGISGLLACKYVLEMGFIPIVFEAKPGIGGLWNHTIKTTKLQTPKQAYQFSDFPWPSSVKEDFPDHNQVMEYIESYARHFDLLQYIKFNSRVTGIEYEGASDEDMASWDLWGGNGDPFSLQGKWNITVEDIHRKSTQVYQVEFVILCIGRFSEVPNIPEFPPNQGPEAFDGKVIHSMHYSSMDDVDAAKFIEGKRVTVVGIQKSALDIASECAMVNGVENPCTLLYRNAHWQLPHYFPWGVPLALLYFNRFAEFMIHKPGEGFMLSLLATLLSPLRWLQSKFIGSYIRWNLQLKKYNMIPKQSFNKDFSGCTLAITPENFFYRVEEGSIILKKSATFSFCKNGLMIDGDTAPLETDVVILATGYKGDEKLKNLFVSSTFQKYIFGSSNTTVPLYRECINPRIPQLAIIGYSESLANLYSFEMRCKWLAHLLKGTFKLPRIKEMEVDVLSWEKYMKDHSSEYYRRSCNSLLQIWYNDRLCKDMKCNPRRKKGFFKELFEPYGPMDYANL

>BUSI_005279-RA|Buxus_sinica/1-462

MERRVAIIGAGISGLLACKYALQKGIQPIVFEAQSGIGGVWIKTLESTKLQSPKDAYCFSDFPWPSSVKEEFPDHTQVMEYLHSYASHFKILPCIKFNSKVIHIDYVGVSDEEILSWDQWGGTGEGFNPRGKWHLTVQHMEDHSVSTVVYQVEFVVLCIGRFTDAPNIPEFPPGKGPEVFDGKVIHSMEYSAMDFASATESIKGKCVTVVGFQKSALDIAVECSKANGLKHPCTVLYKTKHWNVPNYLPWGVPLGFLYLNRFAELMIHKPGEGLLLSLVATLLSPMGWVFSKFVESYITWKLSLRKFGMVPEHSFVQEMSSCLISTVPEDFYDRVEKGSIILKKAPRFGFCNEGVLIEGEVEPLKTDMVILATGFRGDKKLKEIFKSTTFQGCVMGSPDKTVPLYRQCIHPRIPQLAVIGYSESLANLYTSDIRCRWVIELLDGTFKLPRIKDMEEDVDNWD

>DCAR_728970|Dcarota_subsp_sativus_v3.0/1-520

MVKQVAIVGAGISGLLACKYTLSKGFHPIVFEARSSIGGVWINTLETTKLQTPKSYYQFTDFPWPSSVVEDFPNQQQVHDYVSSYATQFGLLEYIKFSSKVKSLDYEGPSDEEMQAWSLWGGTGEPFSSKGKWNLSVQDTQTLSTKVHQVDFVILCVGRFSDVPNIPKFPHDMGPEQFHGKVIHSMDYAEMDNESAAQFVKGKEVTVVGLQKFALDIAMECSSANGSAHPCTVVYRTEHWNVPDYLPWGVPLAYLYFNRFSELLVHKPGEGFILALLAYILSPMRWGISKFVESYIKWKLKLGKFGMVPKHSFLTEMNSCLISTVPEDFYKRVEGGSINLKKAQSFCFEKEGVLLDNDKAEVVKADLVILATGFRGVDKLKDIFLSPTLQTHISGRPENAVPLYRECIQPRVPQLAIIGFSESISNLYTSELRCQWLVELLDGRFKLPNISEMEKDIAKWDEYLKEYSGNKYYRRSCIGALHIWYNDQLCKDMGRNPKRKDGVIAELFQPYGPMDYACLC

>AUR62021441|Cquinoa_v1.0/1-518

MIDLTMERKVGIIGAGISGLLACKYAISKGYNPIVFESRSTIGGVWIKTLEATKLQTPKQLYQFSDFPWPSSVETTFPNQNQLFSYIQSYAKHFDLLKHIKFNTKVVSIKYDCFNNEDILSWPLWGSNEGNKWVITMQDLTQPTRVNQVMQVDFVILCLGRFSDLPNIPKFPLGKGPEVFEGKVMHSMDYSNMENEDARNLIKGKRVTVAGFKKSALDIAMECSVANGVEFPCTMICRTPHWNVDESFTWKNLYLNRFSELLVHTPGEGPLINLLASLLSPLRWGLSKLVEREIKKKQPLEKFDMVPDYNFLQEMNSCMIVRTPKGFYNNVEKGSIDLKRSTNFSFYKEGVVLDDSSVGHPTQLIKSDLVIFANGFKGDQKLKDIFASPKFQECITDSFNETAPLYRECIHPRIPQLAVIGSTESIANLYTSELRCRWLFELLDGKFKLPSIEKLEKEVLKCEKVMKRYSGKYYKRSCIGAVHIWYNDQLCKDMGLNHKRKTGLLAEWFEPYGPMDYA

>Kalax.0210s0063|Klaxiflora_v1.1/1-516

MERKVAVVGAGLSGLIACKYIVSKGYKPIVFEAKSSLGGVWTKTVETTKLQTAKQLYHFTDFPWPESVPDFPDQDQVLAYIQSYARHFDLQKHIRFNTKVVSISYEGVDEEEIKAWGMWGGTGEAFSKKGKWNIAVENSDSSSEVHQVDFVILCLGRFSEVPNIPEFPPGNGPEAFQGEVIHANEYVAMDYRKAADFVKGKRVTIVGFQKFALDIAAECAAVNGKENPCTVLYKTPHWFMPDFMPWGVPLALLYLNRFAELLVQKPGQGFLLSVLSTLLSPVRWSFCKFVESHIKRKLPLEKYGIVPKHRFLEEISSCTIATMPENFFDLAEKGSIVLKKADGFRFCKEGVVANGEAEPAKSDLVLFATGFRGEQKLRDIFASRSFGELITGSPTAIVPLYRQCVHPRIPQLAVIGFSESVANLYTSEMRCKWVAEFLDGTFKLPSIPEMEKEIKEWDAYMKRYSGVNYRRSCIGAVHIWYNDQLCKDMGWNPKRKKGFLAELFEPYGPLDYTQSS

>Kalax.0267s0001|Klaxiflora_v1.1/1-516

MERKVAVVGAGLSGLIACKYVVSKGYKPIVFEAKSSLGGVWTKTVETTKLQTAKQLYHFTDFPWPESVPDFPDQDQVLAYIQSYARHFDLQKHIRFNTRVVSISYEGVDEEEIKAWGMWGGTGEAFSKKGKWNIAVENSDSSSEVHQVGFVILCLGRFSEVPNIPEFPPGNGPEAFQGEVIHANEYVAMDYRKAADFVKGKRVTIVGFQKFALDIAAECAAVNGKENPCTVLYKTPHWFMPDFMPWGVPLALLYLNRFAELLVQKPGQGFLLSVLSTLLSPVRWSFCKFVESHIKRKLPLEKYGIMPKHSFLEEISSCSIVTMPENFFDLADKGSIVLKKADGFRFCKEGVVANGEAEPAKSDLVLFATGFRGEQKLRDIFGSRSFGELITGSLSAIVPLYRQCVHPRIPQLAVIGFSESVANLYTSEMRCKWVAEFLDGTFKLPSIPEMEKEIKEWDAYMKRYSGVNHRRSCIGAVHIWYNDQLCKDMGWNPKRKKGFLAELFEPYGPLDYTQSS

>Kalax.0646s0022|Klaxiflora_v1.1/1-522

MERKVAIVGAGLSGLIACKYVLSKGYKPIVFESKSKLGGVWTKTVETTKLQSEKDSYQFSDFPWPESLETVHPDKDQVLAYIESYAQHFDLARHIRFSTKVVSIAYDGADEEEMNAWSMWGGTGEGFSNKGKWNINAENASTSASEVHRVDFLILCLGRFSEVPNIPDFPPNKGPEVFQGQVIHAKDYIDMDYDKAANFVKGKRVTIVGSMKFAMDIAAECSAVNGKDNPCTMLRRTCHWILPDLHPWGVPFALLYFNRFSELLLRKPGQGFLHNILSTALSPVSWSISKFVESYVKWKLPLAKYGLVPEHSFLDDISSCTFAIWPDNFFDRVDSGSIVLKKADGFGFCKEGVTVNGELEPVKSDLVILATGFNGEQTLREIFASSRLQGLITGSTSSIVPLYRQCVHPRIPQLAVIGFSESASNLFTSEMRSKWVAELLDGSFSLPSVAEMEKDIREWDGYIKRYSGSNYRRSCIAALHIWYSDQLCKDMGLNPKRKNGFLAELFQPYGPLDYQPTNSKSQ

>Pso06G20070.0|Papaver_somniferum/1-523

MERRVGIIGAGISGLLACKYVLEKGFQPIVFESQPGLGGVWTHTVETTKLQTPKQAFEFSDFPWPSSVKEDFPDHNQVMEYIESYALHFNLHPYIKFNTKVINIDYLVQEGQDMVSWSHWGGSDQAFGNPKGKWEIATVAGQEHQVHEVEFVILCIGRFSGVPNIPDFPPNKGPDAFLNGKVIHSMDYSAMDDSDAAKFVKGKRVVVVGLQKSALDIANECAIANGVENPCTLIYRNAHWNVPDYLPYGVPLGYLYLNRFSELLLHKPGEGLFLSLLATLFSPLRWAASKFVESYIKSKFPLKKYNMVPEHSFLQEISSCSISTAPEKFYDRVEEGSIIFKKSRGFGFYKDGLIFDDDVRAPLETDVVIFATGYKGDEKLKNIFKSPTFQNYIMGSSNSTVALYRECIHPRIPQLAVIGYSESVANLYTSEMRSRWLAHLLGDDFKLPSIKKMEKDALEWEKFFKRYSGQYYRHSCIGVLHIWYNDQLCEDMGCNPRRKKGFYAEMFEPYGPMDYANLTPGNN

>Pso01G37410.0|Papaver_somniferum/618-1136

MERRVGIIGAGISGLLACKYVLEKGFQPIVFESQPGLGGVWTQTVETTKLQTARQAFVFSDFPWSTSVEEDYPDHNQVVEYLESYALHFGLLPYIKFNTKVVNIDYLVQEGQDMLSWSHWGGSDQAFSNPKGKWEITTDHQAYEVEFVILCIGRFCGVPNIPDFLPNKGPDVFTKGKVMHSMDYSAMDDADAAEFIKGKRVTVVGLQKSALDIATECAIANGVENPCTLIFRSGHWNVPDYLPWGVSLASLYLNRFSELMFHKPGEGLFLSLLATVLSPLRWAISKFVESYIKSKFPLKKYNMVPDNSFSQEISSCLLALVPEKFFDRVEEGSIILKKSKGFSFYKDGLVFDDDAGATLETDAVILATGYKSDENLKRIFKSPTFQNYIMGSSNSTIPLYRECIHPRIPQLAVIAFSESLVNLYTSEMRCRWLAHLLDDGFRLPSIIEMEKDILKWEKYMKRYCGQYYRRSCIGAVHIWYNDQLCKDMGFNPKRKKGFYAELFEPYGTMDYANLTHSKD

>Pso06G20060.0|Papaver_somniferum/89-605

MERRVGIIGAGISGRLACKYVLEKGFQPIVFESQPGLGGVWTQTVETTKLQSPRQSFVFSDFPWSVEEEYPDHNQVVEYLESYALHFGLLPYIKFNTKVINIDYLVQEGQDMLSWSHWGGSDQAFSNPKGKWEITTEHQAYEVEFVILCIGRFCGVPNIPDFPPNKGPDVFTKGKVMHSMDYSAMDDADAAKFVKGKRVTVVGLQKSALDIANECAIANGVENPCTLIFRSGHWNVPDYLPWGVSLASLYLNRFSELMFHKPGEGLFLSLLATLLSPLRWAISKFVESYIKFKFPLKKYNMVPDNSFSQEISSCLLAMVPEKFFDRVDEGSIILKKSKGFSFYKDGLIFDDDAGATLETDAVILATGYKGDENLKRIFKSLTFQNYIMGSPNSTVPLFRECIHPRIPQLAIIGFSEGLSNLYTSEMRCRWLAHLLDDGFRLPGIIEMEKDILKWEKYMKRYCGQYYRRSCIAVVHIWYNDQLCKDMGFNPKRKKGFYAELFEPYRTMDYANLTQKKD

>Pso01G56390.0|Papaver_somniferum/1-531

MRGRRVGIIGAGISGLLACKYVLEKGFEPIVFESQPGVGGVWTRTPKTTKLQSSKQAYEFSDFPWPCSVQEEYPNHNQVMEYLESYALHFGLLPYIKFNTKVIDINYCVQQGQDMRSWSHWGGTATDNCFTNPKGKWEVTTTCAAAVQGHLGQDPRVYEVEFVILCIGRFSGTPNIPDFPPKKGPEGFVNGKVIHSMHYSAMEDSEATKFVKGKRVTVVGFQKSALDIANECATVNGVENPCTLLYRNLHWNIPADYNPYTVLASLYLNRFSELMLYKPGEGRFLSLLVTLLSPLRWAISKCVESYIKFKLPLKKYDLVPEHRFSADVTSCSISIAPKDFYDRVEEGSILLKKSKGFGFYTNGLTFDDDATAALESDIVILGTGYKGDLKLKNIFKSPTFQKHITDVLNSTVPLYRESIHPRIPQLAVIGYSESYANLYTSEMRCRWLAHFLEDGFKLPSIKEMEKDMLEWEKYMKRYSSRGACIGALHTWYNDQLCKDMGCNPKRKKRFFAELFEPYGPTDYANLTPGKK

>Pso01G56430.0|Papaver_somniferum/1-452

MEYLESYALHFGLLPYIKFNTKVIDINYCVQQGQDMRSWSHWGGTATDNCFTNPKGKWEVTTTCAAAVQGHLGQDPRVYEVEFVILCIGRFRGTPNIPDFPPKKGPEGFVNGKVIHSMHYSAMEDSEATKFVKGKRVTVVGFQKSALDIANECATVNGVENPCTLLYRNLHWNIPADYNPYTVLASLYLNRFSELMLYKPGEGRFLSLLVTLLSPLRWAISKCVESYIKFKLPLKKYDLVPEHRFSADVTSCSISIAPKDFYDRVEEGSILLKKSKGFGFYTNGLTFDDDATAALESDIVILGTGYKGDLKLKNIFKSPTFQKHITDVLNSTVPLYRESIHPRIPQLAVIGYSESYANLYTSEMRCRWLAHFLEDGFKLPSIKEMEKDMLEWEKYMKRYSSRGACIGALHTWYNDQLCKDMGCNPKRKKRFFAELFEPYGPTDYANLTPGKK

>evm.TU.Scaffold_671.1153|Carabica_v0.5/1-523

MEKRIAIIGAGISGLLACKYALAKGYHPIVFEAQDCVGGLWNHTIESTKLQNSTEVFLFSDFPWPSSVKETFPGNTQVLKYVQSYAQHFGLLPYIKFNSKVIGIDYVGVSREEMQAWDLWGGTGQPFGSKGKWNMKVQHTTEDKSIKEYEVEFVILCIGRFSGVPNIPEFPPNHGPEIFGGKVLHSMDYSAMDNASAAALVKGKNIAIIGSQKSAVDMAAQCANVNGIDNPCTMVQRTAHWVLPSYYVWGGVSLHYLYYNRFAEILVHKPGEGFLLSALAFLLSPLRWGIAKLVESYLRWKLPLKKYNMVPPHGFLQEMSSCQILVLPENFYDKVVDGSIVLKKSKNISFCREGLMIDGETQPLTADVIILATGYRGDQKLGNIFTCPSFQECILGSASSTISLYRQIIHPRIPQLAVIGYSESLNNLYTLEMKCRWLGHFLDQTFQLPSIKEMEKDVRMWDGYMKRYAGKYYRRSCLGASHIWYNDQLCKDIGCNPRRKKGFFSELIEPYGPADYAELNPQS

>evm.TU.Scaffold_2241.6|Carabica_v0.5/1-523

MEKRIAIIGAGISGLLACKYALAKGYHPIVFEAQDCVGGLWNHTIESTKLQNSTEVFLFSDFPWPSSVKETFPGNTQVLKYVQSYAQHFGLLPYIKFNSKVIGIDYVGVSREEMQAWDLWGGTGQPFGSKGKWNMKVQHTTEDKSIKEYEVEFVILCIGRFSGVPNIPEFPPNHGPEIFGGKVLHSMDYSAMDNASAAALVKGKNIAIIGSQKSAVDMAAQCANVNGIDNPCTMVQRTAHWVLPSYYVWGGVSLHYLYYNRFAEILVHKPGEGFLLSALAFLLSPLRWGIAKLVESYLRWKLPLKKYNMVPPHGFLQEMSSCQILVLPENFYDKVVDGSIVLKKSKNISFCREGLMIDGETQPLTADVIILATGYRGDQKLGNIFTCPSFQECILGSASSTISLYRQIIHPRIPQLAVIGYSESLNNLYTLEMKCRWLGHFLDQTFQLPSIKEMEKDVRMWDGYMKRYAGKYYRRSCLGASHIWYNDQLCKDIGCNPRRKKGFFSELIEPYGPADYAELNPQS

>evm.TU.Scaffold_576.532|Carabica_v0.5/1-168

MIDGETQPLKADVIILATGYRGDQKLGNIFTCPSFQEYILGSTSSTIALYRQIIHPRIPQLAVIGYSESLNNLYTLEMKCRWLGHFLDQTFQLPSIKEMEKDVRMWDKYMKRYAGKYYRRSCLGASHIWYNDQLCKDIGCNPRRKKGFFSELIEPYGPADYAELNPRS

>Eucgr.C00445|Egrandis_v2.0/1-522

MERRVVIIGAGISGLLACKYSVEKGFDPVVFEAEDRIGGVWNHTIQSTKLQNAKDGFRFSDFDWPASVQEIFPSHTEVLDYVESYARHFNLYPYIKFHSRVINIDFVGESFEEMKTWDLWGGTGMAFGSKGKWHITVLDTETNATEVHQTEFVILCIGRFSGLPNILELPPAYCSKVFDGKVMHSMEYSDMEKSAAAELIKGKRITIVGSQKSAVDLAIECAEANGVKYPCVMIQRNPHWLLPGNGFLISHFGHLYFNRFAELRLHKPGQNILYWLLATVLSPLRWGISKFSEIYLQWKLPLKKYGVVPRHSFLEDLSSCQIGMLPDNFYDKVEEGSILIKRSQSFRFCREGLIVGGEDKPISSDLVILATGYKGDQKLKNIFRSSVFQKYLVPSPTSTIPLYRQVIHPRIPQLAVIGYTEGLANLPSFEIRCQWLTHLLDGNIKLPSIGAMEKDTARWESHMKRYGRRNFWRSCVGTTNIWYNDQLCKDMNCNPRRKKGFLAEWFQPYGPADYAGLADQKR

>Eucgr.L01044|Egrandis_v2.0/1-522

MERRVVIIGAGISGLLACKYSVEKGFDPVVFEAEDRIGGVWNHTIRSTKLQNVKDGFRFSDFDWPASVQEIFPSHTEVLDYVESYAQHFNLYPYIKFHSRVINIDFVGESFEEMKTWDLWGGTGMAFGSKGKWHITVLDTETNATEIHQAEFVILCIGRFSGLPNIPELPPTYCSKVFDGKVMHSMEYSDMDKSAAAELIKGKRITIVGSQKSAVDLAVECAEANGVKYPCVMIQRNPHWLLPGNGFLISHFGHLYFNRFAELRLHKPGQNILYWLLATVLSPLRWGISKFSEIYLQWKLPLKKYGIVPRHSFLEDMSSCQIGMLPDNFYDKVEEGSILIKRSQSFRFCREGLIVGGEDKPISSDLVILATGYKGDQKLKNIFRSSVFQKYLVPSPTSTIPLYRQVIHPRIPQLAVIGYTEGLANLPSFEIRCQWLTNLLDGNIKLPSIGAMEKDTARWESHMKQYGRRNFWRSCVGTTNIWYNDQLCKDMNCNPRRKKGCLAEWFQPYGPADYAGLADQKR

>Eucgr.C00446|Egrandis_v2.0/1-522

MERRVVIIGAGISGLLACKYSVEKGFDPVVFEAEDRIGGVWNRTIQSTKLQNVKDGFRFSDFDWPASVQEIFPSHTEVLDYVESYARHFNLYPYIKFHSRVINIDFVGESFEEMKTWDLWGGTGMAFGSKGKWHITVLDTETNATEVHQAEFVILCIGRFSGLPNIPELPPTYCSKVFDGKVMHSMEYSDMEKSAAVELIKGKRITIVGSQKSAVDLAVECAEANGVKYPCVMIQRNPHWLLPGNGFLISHVGHLYFNRFAELCLHKPGQNVLYWLLATVLSPLRWGISKFSEIYLQWKLPLKKYGLVPRHSFLEDVSSCQTCMLPDNFYDKVEGGSIIIKRSQSFRFCKEGLIVGGEDKPISSDLVILATGYKGDQKLKNIFRSSVFQKYLVPSPASTIPLYRQVIHPRIPQLAVIGYTEGFANLPSFEIRCHWLTHLLDGNIKLPSIGAMEKDTARWESHMKRYGRRNFRRSCVGTSNIWYNDQLCKDMNCNPRRKTGFLAEWFQPYGPADYAGLADQKR

>Eucgr.L01045|Egrandis_v2.0/1-522

MERRVVIIGAGISGLLACKYSVEKGFDPMVFEAEDRIGGVWNHTIQSTKLQNVKESYQFSDFDWPAFVQEMFPGHTEVLDYVKSYAQHFNLYPYIKFHSRVISIDYVGESFEEMKTWDLWGGTGMAFGSKGKWHITVLDTETNATEVHQAEFVILCIGRYSGLPNIPELPPSSCSKVSDGKVMHSMEYSDMEKSAAAELIKGKRITIVGSQKSAVDLAVECAEANGVKYPCVMIQRNPHWLLPSDSFLISHLGHLYFSRFAELLLHKPGQNILYWLLATMLSPLRWGISKFSEIYLQWKLPLTKYGLLPRHSFLEDMSSCLIGMLPDKFYDKVEEGSILIKRSQSLRFCKEGLIVDGEDKPISSDLVILATGYKGNQKLKNIFRSSVFQKYLVPSPTSIIPLYRRVIHPRIPQLAVIGYTGSLANLPSFEIRCQWLMHLLDGNFKLPSIGAMEKDTARWEIHMKRYSGRNFWRSCIGTTSIWYNDQLCKDMNCNPRRKKGFLAEWFQPYGPADYASLADQKH

>Glyma.09G094000|Gmax_Wm82.a6.v1/1-522

MERRVVIIGAGISGLGACKYLLEFGFNPIVFEVDDGVGGLWRHTMDSTKLQNNKQMFQFMDFPWPSSVKEDNPSHKQVLDYVNSYAEHFSLIPYIRFNFKVIDIDYVGGESSEEMKSWELWGGNGRPFCSKGTWHIAVQHTKNLSIEMHEAEFVVLCIGKYSGFPNIPEFPPGKGPEVFNGKVMHSMDYSNLDNETAAELIKGKRVTIIGSQKSGLDLAAECANANGVKHPCTVIQRTAHWFLPDFNFWGVIAGFLYFNRFAELLVHKPGESFLLGLVATLLSPWRWGISKLVETTLKWKLPLKKYGMAPNHSFLQDLSTCLFAVYPDNFFDKLKEGSIIMKGSQNFSFCREGVIIDGEAKPLESDIVFFATGYKGDQKIKNIFKSPLFQKYIIGQATSTVPLYRQIIHPQIPQLAIIGYAESPSNIFASEMKSLWLSHFLDGNIELPSIREMEKDVKLWEDNLKQYGGKYYWKTCIAHCGIWYHDQLCKDMKHDPRRKNGLFSELFEPYGHADYAGLTRKR

>Glyma.15G201100|Gmax_Wm82.a6.v1/1-521

MERRVVIIGAGISGLVACKYVIEFGFNPIVFEVDDGVGGLWRHTMESTKLQNNKQMFQFMDFPWPPSVKEDNPSHKQVLDYVNSYAEHFSLIPYIRFNSKVIDIDYVGGESSEEMKSWELWGGNGRPFCSKGTWHIAVQHTKNLSIEVHEAEFVVLCIGKYSGFPNIPEFPPGKGPEVFNGKVMHSMDYSNLDNDTAAELVKGKRVTIIGSLKSGLDLAAECANVNGMKHPCTIIQRTAHWFLPDFNFWGIIAGFLYFNRFAELLVHKPGESFLLGLVATLLSPLRWGISRLVETTLKWKLPLKKYGMVPNHSFLEDLSTCLFGVYPDNFFDKLKEGSILIKESQSFGFCKEGVIIDGEAKPLESDIVFFATGYKGDQKIKNIFKSPVFQKYIIGHATSTVPLYRQIIHPRIPQLAIIGYAEGPSNIFASEMKSLWLAHFLDGNIELPSIREMEKDVKLWEDNLKLYGGRYYWKICIAHCAIWYHDQLCKDMKHNPRRKKGLFSELFEPYGHTDYASLTRK

>Glyma.09G093800|Gmax_Wm82.a6.v1/1-520

MERRVAIIGAGTSGLVACKYLIEFGFNPIVFEVDDGVGGLWRHTIESTKLQNTKQTYQFIDFPWPSSVKEDNPSHNQVLDYLKSYAEHFSLISYIRFNSKVINIDYVGESSEEMKSWELWGCNGRPFCSKGTWHIVVQDTKNLSIEVHKADFVILCIGKYSGFPNIPEFPPGKGPEVFNGRVMHSMDYSNLDNETAAEFIKRKKITIIGSQKSGLDLAAECANVNGVMYPCTIIQRTAHWILPDFDLWGVIVGFFFLNRFAELLIHKPGEPFLLGLVATLLSPLRWGISKFVETILKWKLPLKKYGLVPNHSFLQDLSTCLLGVFPDNFFDKLKEGSILMKKSQSFSFCREGVIIDGEAKPLETNIVIFATGYKGDQKIKNIFKSPIFQNYIIGPATSTLPLYRQIIHPRIPQLAMVGYAEGISNIFASEMKSLWLVHFLDGNIELPSIREMEKDVKLWEDNMKQYGGKYYSKSCIANCGIWYHDQLCKDMKRNPRRKKGLFAELFEPYGHADYMDLTRK

>Glyma.15G200600|Gmax_Wm82.a6.v1/1-521

MERRVAIIGAGTSGLVACKYLLEFGFNPIVFEVEDGVGGLWRHTIESTKLQNKKQMYQFLDFAWPSSVKEDNPSHEQVLDYLNSYAEHFSLIPYIRFNSKVIDIDYVGGESSEEMKSWELWGGNGRPFCSKGTWHIALQHTKNLSIEVYKAEFVILCVGKYSGFPNIPEFPPGKGPEVFNGRVMHYMDYSNLDNETAAELIKGKRVTIIGSQKSGLDLAAQCSNANGVTYPCTIIQRTAHWFLPDFDTWGVIVGFLFLNRFAELLIHKPGEPFLLGLVATLLSPLRWGISKLVETILKWKLPLKKYGLVPNHNFLQDLSTCLLSLLPDNFFDKLKEGSILIKKSQSFSFSREGVIIDGEAKVLETDVVIFATGYKGDQKIKNMFKSPIFQNYIIGPTISTVPLYRQVIHPRIPQLAMVGYAEGISNIFASEMKSMWLVHFLDGNIGLPSIREMEKDVKLWEDNMKQYGGRYYWKSCIANCCIWYQDQLCKDMKHNPRRKKSLFAELFEPYSLADYVGLTRN

>KAI5441074.1|Pisum_sativum/1-518

MEKKVAIIGAGLSGLIACKYVLETGFQPVVFEADEGLGGQWRHTIESTKIQNPKQDFQFVDFPWDSSVKEDNPNNQQVQAYFNAYAQRFGIVPCIRFNSKVIDIDYVGESNEDIKSWELWNGDGTPFGSKGTWHITLQDTKTFSTEVYKAEFVILCIGKYSGLPNFPEFPHGKGPEIFKGKAMHSMDYAALDNKTAAELIKNKRVTVVGSGKSALDVATEIADKNGVNYPCTMIQRTVHWFLLDFKIWGFSLAYLYFNRFAELLVHKPGENLLLSLVATLLSPFRWGFGRLIEIYLKSKLPLKKFGLVPKHSFHHDMYKCTFGVLPDLYFDKLKERSILIKKSQTVSFCNEGLIIDGEAKPIESDLVIFATGFKGDQKLKSIFKSPTFQSYIKTPTNVTVPLYRQVLHPQIPQLAIMGYAEALSNIYSNEMRCQWLAQFLDGNIKLPSVKEMEKDVKVWEETIKQYDRKWNSCIVTCGIWYNDQLCKDMKCNHLRKKSAFAELFEPYGPSDYNGLVQK

>KAI5441078.1|Pisum_sativum/1-520

MERRVAIVGAGISGLLACKYVLQIGFHPVVFEADDGIGGLWRHTIQSTKLQNPKETFQFSDFPWDSSVKQDCPSSEQVLDYLNSYAQQFSIVPYIRFNSKVIDIDYVGESDEEMNSWELWNGDGTPFGSKGTWHITLQDTKSLSKEVHKAEFVILCIGKYSGLPNIPEFSPGKGPEIFEGKVMHSMDYSALDNDIAAELIKNKRVTIIGSEKSALDIASECANANGVTHPCTIIQRTSHWFLQDFNIGGINLAFLYFNRFAELLVHKPGESFLLSILATLLSPLRWGISKFVETYLRWKFPLKMYGVLPNHNFLQDISSCKTGVLPEFFFDKVKEGSIVIKKSQSFSFCREGLIIEGDAKPIETDLVILATGYKGEEKLRSIFKSPMFQSYINGSVNSAVPLYRQIIHPRVPQLAIIGYAESLSNIFSNEMRCQWLAHLLDGNIELPSIREMKKDVKMWEDTMKQHTKNLNFRSCIVTCGIWYNDQLCKDMKCNHLRKKSVFAELFEPYGPSDYNGLVRK

>KAI5441087.1|Pisum_sativum/1-338

MHSMDYSSLDNKIASELIKNKSVTVVGSGKSALDIASECADANGVTHPCTIIHRNAHWFLLDFNIWIIGLGYFYFNRFAELLVHKPGENSILFLLAILLSPLRWGISKLVETYLKWKMPLKEYGLVPNHSFLQEISACQIGVLPEFFFDKVKEGSIVIKKSQNFSFCKEGLIVNEEPKPLETDLVILATGYKGDQKLRSIFKSSTFQNYISSSEKSSVPLYRQIIHPRIPQLAMIGYSEGISNIVSNEMRCQWLAYFLDGNIELPSIKEMEKDVKVWEENMKQYAGKFYWRSCIISCGIWYNDQLCKDMKCNHLRKKSTFAELFEPYGPEDYNGLVRK

>Qrob_P0740610.2|Quercus_robur/1-529

MEKRVAIIGAGTSGLLACKYILEKGFNPIVFEAKEGVGGLWNHTIESTKLQNSKETCQFTDFPWPASVQDMYPTHTQVLVYLKSYAQHFGIIPYIKFNSKVTYIDYVGEPFEEMESWDLWGVTSKPFGSKGKWHIVVQDTKCCTIEVHQVEFVVLCIGRFSGVPNIPKFPPDQGPEMFDGKVIHSMDYSEMGRASAAELIRRKRVVIVGSLKSAVDIAAECANANGVQYPCTMIQRTAHWLIPSFSFWGVNLGFLFFNRFSELLVHKPGEPFLLKLLATILSPLRWGISKFAESYLRWKLPLKKYGTIPKHSFLLDISSCQIGLLPENFYDKVEEGSIVLKKSQSFSFCKEGLIINGETQPLKADLVILATGYKGDQKLMKMFKSQIFHKYIVGSASSIVPLYRQIIHPRIPQLAIVGYAEGFSNLSTSEIRCQWLAHLLDGNFELPSIREMEKDVILWENNMKQYAGRYFQRLCIGNVNIWYNDQLCKDMGTKHKRKKGIFAEYFQPYGPTDYAGLTRLDQQRGMKIN

>Potri.006G060300|Ptrichocarpa_v4.1/1-404

MGSWGGTGKPFGTEAKWHVKVQDTKNGSIQVFHTEFVVLCVGQFSGLPNIPEFLPNKGPEVFKGKVMHSEDFSALNNSTAAELIKTKRVTIVGSHKTAADIAAECANANGVKYPCTMIQRNAHWFLPSDKLSGLLLGFLYFNRFSEFLVHKPGETFLLSFVATMLSPLRWGISKLIETYLRWNLPLKKYGMLPKISFLEDMSACQTVMLPDKFYDRVEEGSIIIKNSQILSFCEEGLIIDGENQPIETDVVIFATGFKGDEKLRNIFESPVFQNNIMGSPTSTVSLYRQIIHPRIPRLAIIGYNENFSNLGRSEIKSVWLSQFLDGNLELPSIRDMEKEANMWADHIKQVTGRYFRRACISNSSIWYTDQLCRDMGCNPRRKKGFLVDLFIPYAPTDYAGLTSK

>Potri.006G060200|Ptrichocarpa_v4.1/1-520

MEKQIVIIGAGASGLLACKYALNIGLNPIVFEAEDRIGGVWSHTLESTRLQNTKEIFQFSDFPWPPSITDTYPTHTQVMEYFESYAQHFNLFPCIKFNSKVIGIDYVGVSVEEMESWALWGGTGKPFGTEAKWHVEVQDTKNGSIQVFHTEFVVLCVGQFSGLPNIPEFLPNKGPEVFKGKVMHSEDFSALNNSTAAELIKTKRVTIVGSHKTAADIAAECANANGVKYPCTMIQRNAHWFLPSDKLSGLLLGFLYFNRFSEFLVHKPGETFLLSFVATMLSPLRWGISKLIETYLRWNLPLKKYGMLPKISFLEDVSACQIAMLPDKFYDRVEEGSIIIKNSQILSFCEEGLIIDGENQPIETDVVIFATGFKGDEKLRNIFESPVFQNNIMGSPTSTVSLYRQIIHPRVPRLAIIGYNENFSNLGRSEIKSVWLSQFLDGNLELPSIRDMEKEANMWTDHIKQVTGRYFRRACISNSSIWYTDQLCRDMGCNPRRKKGFLVDLFIPYAPTDYAGLTSK

>Potri.018G115800|Ptrichocarpa_v4.1/1-520

MEKRVVIIGSGVSGLLACKNTLKIGLNPIVFEAEERIGGVWSHTLESTRLQNSKEIYQFSDFPWPSSIKNTYPTHNQVMEYLESYAQHFNIFACIKFNSKVIGIEYFGESNEEMDSWALWSGTGKPFGSKGKWHVKVQDTKNCSIQVYHAEFVVLCIGQFSGLPNIPEFLPNQGPEVFKGKVMHSEDFSALDNSTASELIRTKRITVVGSHKSAVDIAAECANANGVMYPCTMIQRNAHWFFPRDNLSGLLLAFLCYNRFSELLVYKPGETFLLSFLATLLAPLRWGISKFIEIYLRWNIPLKKYGMLPKSSFLEDISSCQIAMLPDKFYDRVEEGSIIIKNSQSLSFFPEGLIINGENRPFETDLVIFATGFKGDEKLKNIFESPVFQNYIIGSPTTTVSLYRQIIHPRIPQLAIIGYSENFSSLGYSEIESVWLSHFLDGNLELPSIRDMEEEANMWADHIKQVTGRYFRRACISNFNIYYNDQLCKDMGYNPRRKKGLLADLFIPYTPTDYSGLTSK

>Aco013039|Acomosus_v3/1-526

MEGKRVAIVGAGISGLAACKQAVDRGFDPTVFESERGVGGVWARTLDSTRLQSPRTAFQFSDFPWPPSVAEVFPDHLQVMDYLRAYAAEFGLLRRVKFDHRVLGIDYVGAGEAEIAAWEMWAGNGEAFGGGGKSRGEWHLTVQHGYETEIYHADFVILCIGRFSGVPNIPEFPAKKGPENFDGKIIHSMDYSNMGTAKASELIRDKLVTVVGYLKSAIDIASECADVNGTKYPCTMICRTKRWIIPDYYAWGVPLAFFYLNRFSELLIHKPGEGFLLCLLATILSPLKWLLSKFTESYYKWAIPMKKHGMVPDHSFFQAITSCLIAILPENFYSRVDEGSIVLKKAKEFSFCKDGVMVDGEDTPIKSDLVIFATGFKGDQKLKDIFKSPFFRNIVAGSSSTTVPLYRECIHPRIPQLAIIGYSESLANLYTSELRSKWLAHFLDGGFKLPSIKNMEKSIMEWEKFMKKYSQDYFRRSCIGTLHIWYNDQLCRDMGCNPRRKKGFFAEWFEPYGPQDYVDVEPKKKK

>evm.TU.AsparagusV1_07.2361|Aofficinalis_V1.1/1-462

MYRFSDYPWPPEVTEKYPDHAQVMEYLRSYAAHFHVLDHVRFSTRVVGVEYVGPSEEEMMAWDTWGGNGEAFGSGGVWHVRVQRGHDCQIEVHETDFLILCVGRFSGVPNIPNFPVNKGPEVFDGKVIHSMDYSDMGSARAQELIKGKRVTVVGYLKSAIDIAAECSNVNGVEYPCTMLYRTKRWIVPDYIAWGIPLAFFYLNRFSELLFHKPGEGFLLSLLATLLSPLAWVFSKFAESYYKWALPMKKYGMVPEHSLSHAISSCLFALLPDKFYDKIEEGSIILRKSQSFVFCKKGVMIDGETSPLETDIVILATGYKSDQKISDIFVSPLFQKVVAGSSTTTVPLYRECIHPKIPQLAIIGYSESIANLYTSELRSKWLAHFLDGGFRLPSISKMEENVKEWEKYMKRYNHQSFRRSCIGTTHIWYNDQLCRDMGCEPRRKKGFLADWFLPYGPEDYADL

>Bradi4g37120|Bdistachyon_v3.2/1-524

MEKKRVAIVGAGVSGLAACKHLLARGFLPVVFEASEEAIGGVWARALASTRLQTPRPFYEFSDFPWPPDVAEVFPDHAQVTAYLRSYARRFGVLERVRFGCRVAGMEYDGVGEEEMMGAWDRWAGCGEAFGDGRGEWRLTVQQGSDDVETHIADFVILCTGKFSGVPNILTFPPDKGPDNFDGTVIHSMDYSDMGSAKAAQLIRSKLVTVVGYQKSALDIAAECANINGAEHPCTIICRTEKWTIPDSYAWGVNFAFFCLNRFSELLVHKPGEGLLLSILATILSPLRWVFSKFVESYYRWAVPMDKHGMVPDHSFFQAISSCLIAILPDKFYDMVDKGSIILKKAKSFSFCKQGVTVEGESVPIKSDIVIFATGYKGDQKLREIFTSSLFRDIVTGSPSNVIPLYRQCIHPRIPQLAIIGYSESIANLYTFEMRSKWLACFLDGVFQLPSIQSMEANIKEWDEYMKRYSREYFRRSCIGALHIWYNDQLCQDMGCEPRRKKGFLADWLMPYLPADYKDIDLKK

>HORVU.MOREX.r3.5HG0502830|Hvulgare_Morex_V3/1-525

MDEKQKRVAIVGAGASGLAACKHALERGLQPVVFESSGAIGGVWARALASTRLQTPRTLYEFSDFPWPPEVAEVFPDHAQAAAYLRSYAARFGVLERVRFGCRVTAMEYAGVVEEEVQAWDRWAGDGSAFGDGRGEWRLTVQQQGGGDVETHVADFVILCTGRFSGIPNIPTFPPNKGPEKFDGTVIHSMDYSDMGTAKATELIRGKLVTVVGYQKSAIDIAAECANANGAKYPCTIICRTKRWIIPDYYAWGVPIAFFYLNRFSELLVHKPGEGLLLSLLATFLSPLRWLFSKFVESYYRWVVPMDKHGMVPDHSFFQAISSCLVAILPDKFYDMVDQGSIVLKKAKSFSFCNQGVIVEGESMPIKSDVVIFATGYKGDQKLKEMFTSSLFRDNVTGSPSNIIPLYRQCVHPRIPQLAIIGYSESIANLHTFDMRSKWLVHFLDGVFQLPSIKSMEMDIKKWDEYMKRYSREYFRRSCVGALHIWYNDQLCQDMGCEARRKKGFFADWMLPYLPSDYKDINLKK

>LOC_Os09g37650|Osativa_v7.0/1-528

MEKIKKRVAIVGAGVSGLAACKHALERGFRPVVFEADGAGAVGGVWARTIASTRLQTPRPYFEYSDFPWPPGVTDLYPDHDQVTAYLRSYAERFGVLECVRFGCRVAGMEYAAAGGEEEVMAWEHWAGNGEAFGTGRGEWRLTVQSGHNIELHVADFVVLCIGRFSGFPNMPRFPPDKGPEAFAGTAIHSMDYSNMGAAKAAQLVKGKRVAVVGYQKSAVDIAAECADANGASHPCTIVLRTKRWIVPDLYAWGVPVPVFYINRLSQILLHKPGDGLILSFLAILLSPLRWLFAKFVESYYKWALPMEKHGMVPDEDFMEAMCSCSVMKLPDKFYDKVEEGSIVLKKSKRFSFCKEGLVVEGDSSSETIKSDVVIFATGFNGDQKIREMFKSPLFREIVAGPPSSIVPHFRQCIHPRIPQLAIIGYAESWSNLCVSELLSKWLAHFLHGSFRLPSVKEMEEDIDEWDKYMKRYSPGRFRRSCIGPVSVLCSDRLCQDMGVQRRRKKWLLADWLVPYGPADYADINLNS

>HORVU.MOREX.r3.5HG0473870|Hvulgare_Morex_V3/1-520

MEKKRVAIIGAGVSGLTACKHALEHGFRPVLFEAEADAIGGVWAHTLASTRLQTPRPYYQFTDFPWPPGVSDLYPDHKQVTEYLRSYARRFGVLECIRFGSRVAALEYSGAVNEEEMMAWDQWAGTGEAFSSGRGEWRLTVQRGDDVEIHVADFVVLCVGRFSGMPNIPNFPPGKGAEAFDGTVIHSMDYSNMDDAKATQLIKGKLVTVIGYQKSALDIATECANVNGPKYPCTMICRTKRWIIPGYNAWGVPIGFFYLNRFSQLLIHKPGEGLLLGLLATLLSPLRWLFSKFVESYYRWAVPMKKHGMVPDHSFFDAMSSCLVASLPDKFYSNVDQGSIVLKKAKQVNFCRQGLVVQGESSPVIKSDVVIFATGYRGDEKLREMFASHMFRDIVAGSPSRIVPHFRQCVHPRIPQLAIIGYCENVSNLHAFELQSKWLAHFLRGTFRLPSIRCMEENVDEWDKYMKRYASDHFRRSCVAPVHIMCNDQLCQDMGIDHKRKNGFLADWVLPYGPADYGGI

>LOC_Os04g14710|Osativa_v7.0/1-521

MEKKRVVIVGAGVSGLAACKQLLERGCRPVVFEADTGVGGVWAWTLEGTALQTPRPMYQYSDFPWPETVTEVFPDHRQVMDYLGAYARRFGVLDCVRFGHRVAGMEYVGVGKEDVAAWDEWAGNGDAFGSGSGEWRLEVADGEGHLETHTADFVILCVGKYSGVPNIPTFPACKGPEAFDGQVIHSMDYSKMGTKNCKEMIKGKRVTIVGYLKSALDIAAECAEVNGTEIPCTMVVRTKHWIIPDYYAWGVPISKLYLNRFSELIIHKPGEGLLLSILATLLTPLRWIFSKFAESYYSIPMKKYGMVPDHSLFEALITCLIAITPKDHYKRLEEGSIVLKKSKTFSFCNEGVLVEGESSPIKSDIVIFGTGFRGGQKIKEMFTSEYFQSIAVGSASTTIPLYREIIHPKIPQLAVIGYSESLANLYTSELRAKWLAHFMDGGFRLPSISVMQNDVQEWEKFMKRYSRAYFRRSCIGFLHIWYNDKLCRDMGCNPRRKNGFWADLFDVHGPVDYIDLHPKKE

>LOC_Os04g14690|Osativa_v7.0/1-521

MEKKRVVIVGAGVSGLAACKQLLERGCRPVVFEADTGLGGVWARTPECTALQTPRPMYQYSDFPWPETVTEVFPDHRQVMDYLGAYARRFGVLDCIRFGHRVAGMEYVGVGEEAVAAWDEWAGNGDAFGSGSGEWRLEVVDGEGHIETHKADFVILCIGRFSGVPNIPTFPPGKGPEAFDGQVIHSMDYSKMGTKNSKEMIKGKRVTIVGYLKSALDIAAECAEVNGTEIPCTMVVRTKHWIIPDYFAWGVHISKLYLNRFSELLIHKPGEGLLLSILATLLTPLRWIFSKFAESYYSIPMKKYDMVPDHSLFEALITCLVAITPKDHYKRLEEGSIVLKKSKTFSFCKEGVLVEGQSSPIKSDIVIFGTGFRGDQKIKEMFTSEYFQSIAVGSASTTVPLYREIIHPKIPQLAVIGYSESLANLYTSELRAKWLAHFMDGGFRLPSISVMQNDVLEWEKFMKRYSRGYFRRSCIGILHIWYNDQLCRDMGCNPRRKNGFWADLFDVYGPSDYIDLFPKKE

>Bradi3g20960|Bdistachyon_v3.2/1-519

MENRRVAIVGAGVSGLAACKHLLERGCRPIVFEADTGLGGVWARAPECTSLQTPRPMYQYSDFPWPESVTEVFPDHRQVMDYLGAYARHFGVLDCVRFGHRVLGMEYVGVSEEKLAGFQEWGGSGEAFGYGDGEWRLEVADADGHVETHDVDFVVLCIGRFSGVPNIPTFPAGRGPEAFDGQVIHSMDYSKMGSEKAKEMIKGKRVIVVGYLKSALDVAAECAETNGTEQPCTMIVRTKHWILPDYYAWGVHISKLYLNRFSELLIHKPGEGMLLSLLATILTPLRWMFSKFAESYYAIPMKKHDMVPDHSLFEALVTCLIAITPKDHYKRLEEGSIVLEKSKTFSFCKEGVLLEGQSSPIKSDIVIFGTGFKGDQKIKDMFKSEYFQSIAVGSTSTTVPLYRECIHPKIPQLAVIGYSESLANLYTTELRVKWLTHFLDGGFRLPSIEAMQKDVLEWEKFMKRYSSQYFRRSCIGILNIWYNDQLCKDMGCNPRRKSGFFAELTEVYGPGDYANLHPK

>HORVU.MOREX.r3.1HG0024380|Hvulgare_Morex_V3/1-519

METKRVAIVGAGVSGLAACKHLLERGYRPVVFEADTVLGGVWAHPPECTALQTPRPMYQYSDFPWPETVTEVFPDHRQVMDYLDAYARHYGVLDCVRFGHRVLGMEYVGVSEEKLAAWEEWGGSGEAFGSGDGEWRLKVASGDDHVETHIVDFVVLCIGRFSGVPNIPEFPPGRGPEAFDGQVIHSMDYSKMGSTKAKEMIAGKRVTVVGYLKSALDIAAECAEVNGTEEPCTMIVRTKHWIIPDYYAWGVHISKLYLNRFSELLIHKPGEGFLLSLLATTLTPLRWIFSKFAESYYSIPMKKYDMVPDHSLFEALVTCLIAITPKDHYKRLEEGSIVLKKSKTFSFCKEGVLLQGESSPTKSDIVIFGTGFKGDQKIKDMFTSEYFQKIAVGSTSTTVPLYRECIHPKIPQLAVIGYSESLANLYTTELRVKWLTHFMDGGFRLPSVEAMQRDVLEWDKFMKRYSRVYFRRSCIGILNIWYNDQLCKDMGCNPRRKKGFFAELTEVYGPGDYVNLHPK

>Bradi5g03710|Bdistachyon_v3.2/1-521

MRKEEKRVAIVGAGVSGLTACKHALERGFRPTVYEADTVLGGVWARVPDCTSLQTERPMYQYSDFPWPDTVTEVFPDHQQVADYLAAYARRFCVLDCIRFGQRVVGMEYEGVEEEEVAAWEEWAGTGEAFGSGHGQWRLAVADAEGNIQTENADFVILCIGRFSGVPNIPTFPQGKGPEEFDGQVMHTMDYSKMGTKKANEMLKGKRVTVIGYLKSALDVAAECAQVNGIEQPCTMVVRTKHWIIPDYYAWGFPISKLYLTRFSELLIHKPGEGLLLGLLATILTPLRWLFSKFAESYYSIPMKKHDMVPEHSLFEALVTCLIAITPKDHYKRLDEGSIILKKSKTFSFCKDGVLVEGEPSPIKSDIVIFGTGFKGDQKIKDMFMSDYFQSIAVGQIFSTVPLYRECIHPKIPQLAVLGYSESLANLYTAEIRAKWLAHFMDGGFRLPSVNAMQSDILEWEKFMKRYSRGYFRRSCIGILHIWYNDQLCRDMGCNPRRKSGFFAELFEVYGPADYINLRPK

>HORVU.MOREX.r3.2HG0103940|Hvulgare_Morex_V3/1-519

MERKRVAIIGSGVNGLTACKHLLERGCRPAVFEADTLLGGVWARAPDCTSLQSERPMYQFSDFPWPESVTEVFPDRRQVADYLDAYARRFGVLDCVRFGRRVVGMEYVGVGEEEVAAWEEWAGCGEAFGSGEGEWRLTVADAEGNMETHVADFVVLCLGRFSGVPNIPTFPPGKGPEAFDGEVIHTMDYAKMGTKKANEMLKGKRVTIIGYLKSALDVAAECADVNGIEHPCTMVVRTKHWIIPSYYAWGFPISNLYLNRFSEFLVHKPGEGFLLCLLATILTPLRWLFSIFAESYYSIPMKKHDMVPDHSLFEALATCLIAITPKDHYKRLDEGSIVLKKSKTFSFCKEGVLVECESSPIKSDVVIFGTGFKGDQKIKNMFTSEYFQSIAVGPTSSTVPLYRECIHPKIPQLAVLGYSESLSNLYTAELRAKWLAHFIDGGFRLPSVKAMQSDILEWEKFMKRYSRVYFRRSCIGLLHIWYNDQLCQDMGCNPRRKNGIFAELFDVYGPHDYVNLHPK

>ELECO.r07.4BG0358370|Ecoracana_v1.1/1-288

MLVRTKHWIIPDYVAWGVHISKLYLNRFAELLIHKPGEGFLLWILATVLTPLMWLFSKFAESYYSIPTKKYDMVPDHSLFEALATCLIAITPKDHYKRLEEGSIVLKKPKTFSFCKDGVLVEDESSRTKSDIVIFGTGFKGDDKIKNVFTSEYFRIIAIGSTSSTVPLYRQCINPKIPQLAVIGYSESIANLYTSELRAKWLAHFLYGGFKLPNVAAMQKNIFEWEKCMKRYSGRYFRRSSIGLLHIWYNDQLCRDMGCNPRRKKGFFAELFDIYSPGDYIDLHPKKQ

>ELECO.r07.4BG0358400|Ecoracana_v1.1/1-522

MAMEKKQVVIVGAGVSGLAACKQLLDRGFRPVIFEADTVLGGVWAHVPDCTSLQTPRPLYQYSDFPWPETVTEVFPDHRQVKTYLDAYARHFGVLDCIRFGHRVVGMEYDGVGEEAMAAWEEWAGNGKAFGTGAGEWHLNVADAQGRVEIHKADFVILCIGRFSGVPNIPTFPEGKGPEAFDGLVIHSMDYAKMGTKKGQEMMKGKPVTVIGSLKSALDVAAECAEVNGTDHPCTMVVRTKHWIIPDYVAWGVHISKLYLNRFAELLIHKPGEGFLLWILATVLTPLRWLFSKFAESYYSIPMKKYDMVPDHSLFEALATCLIAITPKDHYKRLEEGSIVLKKSKTFSFCKDGVLVEGESSPIKSDIVIFGTGFKGDDKIKNVFTSEYFRSIAIGSTSSTVPLYRQCINPKIPQLAVIGYSESIANLYTSELRAKWLAHFLYGGFKLPNVAAMQKNIFEWEKCMKRYSGRYFRRSSIGLLHIWYNDQLCRDMGCNPRRKKGFFAELFDIYGPGDYIDLHPKK

>ELECO.r07.4AG0326400|Ecoracana_v1.1/1-523

MAMEKKHVVIVGAGVSGLAACKQLLDRGFRPVIFEADTVLGGVWAHVPDCTSLQTPRPLYQYSDFPWPETVTEVFPDHRQVKTYLDAYARHFGVLDCIRFGHRVIGMEYDGVGEETMAAWEEWAGNGKAFGTGAGEWRLNVADAQGRVEIHKADFVILCIGRFSGVPNIPTFPEGKGPEAFDGLVIHSMDYAKMGTKKGQEKMKGKRVTVIGSLKSALDVAAECAEVNGTDHTCTMVVRTKHWIIPDYVAWGVHISKLYLNRFAELLIHKPGEGFLLWILATVLTPLRWLFSKFAESYYSIPMKKYDMVPDHSLFEALATCLIAITPKDHYKRLEEGSIVLKKSKTFSFCKDGVLVEGESSPIKSDIVIFGTGFKGDDKIKNVFTSEYFRSIAIGSTSSTVPLYRQCINPKIPQLAVIGYSESIANLYTSELRAKWLAHFLYGGFKLPNVAAMQKDIFEWEKCMKRYSGRYFRRSSIGLLHIWYNDQLCRDMGCNPRRKKGFFAELFDIYGPGDYIDLHPKKQ

>Zm00001d004389|Zmays_RefGen_V4/1-342

MDYAKMGTKKAREMIKGKRVTVVGYLKSAVDIAAECAEVNGADHPCTMVVRTKHWIIPDYFAWGVHISLLFLNRFAELLIHKPGEGFLLCLLATLLAPLRWLFSKFAESYYAIPMRKHGMVPDHSLFQALATCLIAIQPKGFYERLEEGSIVLKRSKTFSFCREGVVVEGEVSSSSLVESDVVIYATGFRGDLKIKDMFASEYFRSVAVGSACTTVPLYRECIHPKIPQLAVLGYSESIANLYTSEIRAKWLAHFMDGGFRMPSVAAMQKDVLEWEKCMKRYAGGRYFRRSCIGLLHIWYNDQLCRDMGCDPRRKKGFLEELFGVYGPADYADLHPDRKKDD

>Sobic.006G021000|Sbicolor_v5.1/1-527

METNSKRVAIVGAGVSGLAACKHLLERGFNSPVPVVFESGAVVGGVWARVPDSTALQTPRPMYQYSDFPWPDTVTDVFPGHRQVMAYLDAYARRFGVMDCVRFRRRVLGMEYHGVDEDDVAKWEEWSGNGEAFGSGAGEWHLKVADAEGRVETHKADFVILCIGRFSGVPNTPTFPPGKGPEAFDGQVIHSMDYAKMGTKKTKEMIKGKRVTVVGYLKSAIDIAAECAEVNGTDHPCTMVVRTKHWIIQDYFAWGVHISLLYLNRFAELLIHKPGEGFLLWLLATLLAPLRWLFSKFAESYYSIPMKKYDMVPDHSLFQALATCLVAIEPKGFYKRLEEGSIVLKKSKTFTFCKDGVVVEGEELSPIKSDIVIYGTGFRGDLKIKDMFTSEYFRSIAVGSTCTTVPLYRECIHPKIPQLAVLGYSESIANLYTSELRAKWLAHFMDGGFRLPNVTAMQKDILEWEKCMKRYAGRYFRRSCIGLLHIWYNDQLCRDMGCDPRRKKGFLQELFGIYGPGDYADLHPKKD

>Sobic.002G051300|Sbicolor_v5.1/1-530

MEKKRVVIVGAGVSGLTVCKHLLELGCRPVVFEGDTVLGGVWARAPACTELQTPRPMYQYSDFPWPETVTEVFPNHRQVVAYLDAYARHFGVLGCIRFAHRVVGMEYHGGGGGGVSEQDMDACEEWAGNGQAFGSGAGEWRLSVADADGHVETHKADFVVLCMGRFSGIPNIPTFPAGKGPEVFDGQVIHSMDYAKMGASKAKEMIRGKRVTVVGYLKSALDIAAECAEVNGTAHPCTMVVRTKHWILPDYFAWGFHISKLYLNRFSELLIHKPGEGLLLSILATLLTPLRSVFSKFAESYYSIPMKKHDMVPDHSLFEALAAATIAITPKDHYKRLDEGSIVLKKSKTFSFCKEGVVVEGETSPVKSDVVIFGTGFKGDDKIKNMFTSEYFRNIAVGSESTTVPLYRECIHPKIPQLAVIGYSESIANMYTSDIRAKWLARFLDGGFRLPNVAAMQKDVLEWEKYMKRYSGRYFRRSSILILHTWYNDQLCRDMGCNPRRKKGFFSELFEVYGPGDYANLLSNSKSKEQ

>Sobic.002G051400|Sbicolor_v5.1/1-527

METKRVAVVGAGVSGLTVCKHLQELGCRPVVFEGDTVLGGVWARAPACTELQTPRPMYQYSDFPWPETVTEVFPTHRQVVAYLEAYARHFGVFGCIRFAHRVLGMEYHGGVGEQAMAAWEEWAGNGQAFGSGAGEWRLSVVDADGHVETHKADFVVLCMGRFSGVPNIPTFPAGKGPEVFDGQVIHSMDYAKMGTSNAKDMIRGKRVTVVGYLKSALDIAAECAEVNGTAHPCTMVVRTKHWILPDYSAWGFHISKLYLNRFAELLIHKPGEGLLLSILATLLTPLRWAFSKFAESYYSIPMKKHGMVPDHSMFDAWAATTFAIAPKDHYKRLEEGSIVLKKSKTFTFCKEGVIVEGEPSSPVKSDVVIFGTGFKGDDKIKDMFTSEYFRNIAVGSESTTVPLYRECIHPKIPQLAVIGYSESIANIYTSDIRAKWLARFLDGGFTLPNVAAMQKDVLEWEKYMKRYCGRYFRRSSILILHTWYNDQLCRDMGCNPRRKKGFFSELFEVYGPGDYANLLSNSKSKEQ

>ELECO.r07.4BG0358390|Ecoracana_v1.1/1-519

MEKKRVAIVGAGVSGLAACKHLLERGCRPVVFEADTVIGGVWAHVPDSTKLQTPRPMYQYSDFPWPESVTEVFPDHHQVTAYLDAYARHFGVLECIRFGHRVTGMEYDGVDEEAVVMWEEWAGNGEAFGSGTGEWCLAVADAQGRVETYKADFVILCIGKFSGVPNIPTFSAGNGPEAFDGQVIHSMDYAKMGTKKAREMLKGKSVTIIGYLKSAIDIAAECAEANGTAHPCTLVVRTKHWIIPDYSAVAISRLFLNRFAELLIHKPKEGFLLWLLATLLAPLRWLFSKFAESYYSIPMKKYDMVPDHSLFEALAMCLVAFEPKDYYKRLEEGSIILKKSKTFSFCKEGLLVEGQSSPIKSDAVIFGTGFRGDQKIKDMFISEYFRGVSVGSASTTVPLYRECIHPKIPQLAILGYSESTANLYTAELGAKWLAHFLDDGFRLPDIASMEKDVLEWEKYMKRYAGPYFRRSCIGLLPIWYNDQLCKDMGCNPRRKKCFIEELFGVYSPSDYADLQPKKE

>ELECO.r07.4AG0326390|Ecoracana_v1.1/1-545

MNLLTLQSPRPTVSYLLGILHKKSSSMEKKRVAIVGAGVSGLAACKHLLERGCRPVAFEADTVIGGVWAHVPDSTKLQTQRPMYQYSDFPWPETVTEVFPDHHQVTAYLDAYARHFGVLECIRFGHRVTGMEYDGVDEKVVAAWDEWAGNGEAFGSGTGEWCLAVADAEGRVETYKADFVILCIGRFSGVPHIPTFSAGNGPEAFDGQVIHSMDYAKMGTKKAREMLKGKSVTVIGYLKSAIDIAAECAEANGTAHPCTLVVRTKHWIIPDYSVVHISLLFFNRFAELLIHKPKEGFLLWLLATLLAPLRWLFSKFAESYYSIPMKKYDMVPDHSLFEALAACLVAIEPKDYYKRLEEGSIILKKSKTFSFCKEGLLVENQSSPIKSDVVIFGTGFRGDQKIKDMFTSEYFRGISVGSASTTVALYRECIHPKIPQLAVLGYSESTANLYTAELRAKWLAHFLDDGFRLPDIASMEKDVLASEKYMKRYAGPYFRRSCIGVLHIWYNDQLCKDMGCNPRRKKGFIEELFGVYSPADYADLQPKKE

>HORVU.MOREX.r3.2HG0103930|Hvulgare_Morex_V3/1-549

MIMPPAHHTAACHSHRESVEHRRTHATRSTMDKKRVAIVGAGLSGLAACKHLLERGCRPAVFEADTVLGGVWAHAPECTRLQTPRPMFQYSDFPWPESVTEVFPDHRQVADYLDAYARRFGVLDCIRFGRRVVGMEYVGVDEEEMAAWGDWAGCGEAFGSGDGEWLLTVADADGLVEVHKADFAILCVGRFSGVPNIPTFPVGKGPEVFDGKVVHSTEYSQMGGKNARDMIKDKRVTVIGYLRSAVDIANECANVNGTEKPCTMIVRTKSWIIPDYYAWGVHISNFYLTRFAELLIHKPGEGFLLNMLATVLTPLRFIFSKFVESYYSIPMRKHDMVPDHSFFEGLVACVAAIAPKDHYKNIEEGSIILKKSKTFSFCEEGVHVEGECTPVKSDIVIFGTGFKGDQKIKDMFTSKYFQSRVVGSTSMTVPLYRECIHPKIPQLAVIGYSESLTNIYTTELMAKWISHFMDGGFRLPSVREMQRDILEWEKFMKRYSRDYFRRSCIGIVHIWYNDQLCQDMGCNPKRKNGFFSELFEPYGPCDYVNLHPK

>HORVU.MOREX.r3.7HG0751200|Hvulgare_Morex_V3/1-521

MGMEKKSVVIIGAGVSGLAACKHLLERGCRPVVLEADSVVGGVWAHTPDLTRLQTSRTMYQYTDFPWPDSVTEEFPNNRQVADYLNAYARHFGVLECIRFGHRVAGMEYVGVAEEEVAAWDDWAGCGDAFGSGNGKWRLTVTDAQGLVQVHMADFVILCIGRFSSVPNIPKLPPGKGPDAFDGKVIHSLDYSKMGSHKAKEMVKGKRVTVIGYGHSALDIANECASLNGTERPCTMVVRTKQWVLPDFYAWGIDISNFYLTRFGELLIHKPGEGLFLSLLATTLTPLKLMFSKFAESYYSITMKQHDMVPDHSFFEGIVGGWVELAPKDHYKNLEEGSILVKKSKTFSFCKEGVMVEGESTLVKSDIVILGTGFKGDQNIKSMFASKYFQRILIGSISMDVSLYRDCVHPKIPQLAVIGYSETYANLHTSELRAKWLAHFMDGGFRLPSVKAMHRDALEWEKFLKRYSHGEFRAASIGLLNNWYKDNLCRDMGCNPRRKNGLLAELFEVSGPSDYIGLHPM

>HORVU.MOREX.r3.7HG0751170|Hvulgare_Morex_V3/1-521

MGMEKKSVVIIGAGVSGLAACKHLLERGCRPVVLEADSVVGGVWAHTPDLTRLQTSRTMYQYTDFPWPDSVTEEFPNNRQVADYLNAYARHFGVLECIRFGHRVAGMEYVGVAEEEVAAWDDWAGCGDAFGSGNGKWRLTVTDAQGLVQVHMADFVILCIGRFSSVPNIPKLPPGKGPDAFDGKVIHSLDYSKMGSHKAKEMVKGKRVTVIGYGHSALDIANECASLNGTERPCTMVVRTKQWVLPDFYAWGIDISNFYLTRFGELLIHKPGEGLFLSLLATTLTPLKLMFSKFAESYYSITMKKHDMVPDHSFFEGIVGGWVELAPKDHYKNLEEGSILVKKSKTFSFCKEGVMVEGESTLVKSDIVILGTGFKGDQNIKSMFASKYFQRILIGSISMDVSLYRDCVHPKIPQLAVIGYSETYANLHTSELRAKWLAHFMDGGFRLPSVNAMHRDALEWEKFLKRYSHGEFRAASIGLLNNWYKDNLCRDMGCNPRRKNGLLAELFEVSGPSDYIGLHPM

>HORVU.MOREX.r3.7HG0751160|Hvulgare_Morex_V3/1-521

MGMEKKSVVIIGAGVSGLAACKHLLERGCRPVVLEADSVVGGVWAHTPDLTRLQTSRTMYQYTDFPWPDSVTEEFPNNRQVADYLNAYARHFGVLECIRFGHRVAGMEYVGVAEEEVAAWDDWAGCGDAFGSGNGKWRLTVTDAQGLVQVHMADFVILCIGRFSSVPNIPKLPPGKGPDAFDGKVIHSLDYSKMGSHKAKEMVKGKRVTVIGYGHSALDIANECASLNGTERPCTMVVRTKQWVLPDFYAWGIDISNFYLTRFGELLIHKPGEGLFLSLLATTLTPLKLMFSKFAESYYSITMKKHDMVPDHSFFEGIVGGWVELAPKDHYKNLEEGSILVKKSKTFSFCKEGVMVEGESTLVKSDIVILGTGFKGDQNIKSMFASKYFQRILIGSISMDVSLYRDCVHPKIPQLAVIGYSETYANLHTSELRAKWLAHFMDGGFRLPSVNAMHRDALEWEKFLKRYSHGEFRAASIGLLNNWYKDNLCRDMGCNPRRKNGLLAELFEVSGPSDYIGLHPM

>HORVU.MOREX.r3.7HG0751320|Hvulgare_Morex_V3/1-372

MGLDTKSVAVIGAGVSGLAACKHLLERGCRPVVFDAGDAVGGVWPNAMEGTRLQAPRHMYRYSDFPWPDSVTEMFPNQRQVADYLHAYARRFGVLDCVRLGHRVTNMEYVGVAEEEVVAWEEWAGCGDAFGSGDGEWRLTVADAQGHTEVHMTDFVILCTGRFSNYPNIPKFPPGRGPEAFDGKVIHSMDYSKLGSEKAKEMIKDKCVTVVGYGNSALDIANECAHVNGTEKPCTMVVRTKQWIIPDFYAWGINISNFYLTRFAELLIHKPGEGLLLSILATVLTPLRLMISMFAESYYSIPMKKHDMVPDHSFFEGMVGCMLSTTPKDHYKNLEEGIIVIKKSKTFGFCKEGVLVEGESTFVKSDIVIFGT

>Soltu.DM.01G051730|Stuberosum_v6.1/1-506

MADFNERESRKKEVVAIIGAGISGLLACKYCLSKGFDPIVFESESSIGGVWTKTIGSTKLQTPKPFYQFSDFPWPNSVTEMFPDHQTVLEYIESYARHFDLVRCIQFNSKVLSLSYEDGKLSGDNWNVVWGEVYQVDFVIVCVGRFSQVPNIPEFPPKKGAEAFEGQVIHSMEYSNMDPETAANFVKGKHVAIVGFQKSGMDIAMECSTINAGTERPCTVVIRTPHWNLPDFFPWGLNLGYLYLTRFSELTVHKPGEGLALSLLATTLSPLRWALSKFVEIYIRRKHRLAKHGMVPDHSFLNEFSSCSLSIVPEGFYDRVEEGSIKLVKRAESVGFSKEGMMLKGQAEPLKSDLVILSTGFKGIDKLKHIFESKKYQDFIAGLDDSAAVPLYRECIHPRIPQLAIIGFSESITNLYTSEIRCRWLAELLDGKFKVPSIKVMEKDIAEWDKYKRRYSYNKYYRNSCIAALHVWHNDQLCKDMGWNPKRKKGFWDEWFQPYGPMDYRG

>Soltu.DM.01G051770|Stuberosum_v6.1/1-522

MAHFEGRKKEVIAIIGGGISGLLACKYCISKGFDPIVFESESSIGGVWTKTIGSTKLQTPKPVYQFSDFPWPDSVTQVFPDQQMVLEYIESYARHFDLFTHIHFNSKVLSLNFEDSSISGDGEWNLWGGKGKWNVTVQDTRTISPQIQIYQVDFVVVCVGRFSQVPNMPEFPQNKGPEAFEGEVIHSMDYSKMDSSTATNFVKGKQVAVVGFQKSGMDIAMECSTVNGVERPCTVLIRTPHWNLPDYFPWGLNLGYLFLNRFSELMVHKPGEGFLQSLLATILSPLRWAFSKYIGSYIKHKHQLAKHGMVPEHSFLNELSSCSVSLVPEGFYERVEEGSIKLIKKVESFGFSKEGIVLEGHATEPIKSDLVILATGFNGLDYLKHIFQSKKYQEFIAGSDDSAAAPLYRECIHPRIPQLAIIGFSESISNLHTSEIRCRWLGELLDGKFMLPSIKVMEKDIAEWDKYKKRYSYRKYYRRSCIGALHIWYNDQLCKDMGWNPKRKKGLWAEWFEPYGPMDYIG

>Solyc01G004383|Slycopersicum_ITAG5.0/1-549

MKLELFASLSKKRTKETSVAVAMANFEGRKKEVIAIIGGGISGLLACKYCISKGFDPILFESKSSIGGVWTKTIGSTKLQTPKPVYQFSDFPWPDSVNQVFPDQQMVLEYIESYARHFDLFSHIHFNSKVLSLNFEDGGSGDGEWNLWGEAYSYSNKGKWKVTVQDTRAISPQIQIYQVDFVVVCVGRFSQVPNMPEFPQNKGPEVFEGEVVHSMDYSMMDSSTATNFVKGKQVAVVGFQKSGMDIAMECSTVNGVERPCTVLIRTPHWNLPDYFPWGLNLGYLFLNRFSELMVHKPGEGFLLSLLATILSPLRWAFTKYIESYIKHKHQLAKHGMVPEHSFLNELSSCSVSLVPEGFYERVEEGSIKLIKKAESFGFSKEGIVLDGHATEPIKADVVILATGFNGLDYLKHIFESTKYQEFIAGSDDSAAVPLYRECIHPRIQQLAIIGFSESVSNLHTSEIRCRWLGELLDGKFKLPSIKVMEKEIAEWDKYKKRYSYNKYYRRSCIGALHIWYNDQLCKDMGWNPKRKNGFWAEWFQPYGPMDYIG

>Soltu.DM.01G051750|Stuberosum_v6.1/1-516

MANFNDRKKEVIAIIGAGISGLLACKYCISKGFDPIVFESESSIGGVWTKTIESTKLQTPKPVYQFSDFPWPDSVTEMCPDHKTVLEYIESYATHFDLVRHIKFNTKVLSLSYEDDSSSSINGAGEWNLCKGKWNVTVQDTPTLSTQVYQVDFVVVCVGRFSQVANMPEFRPSNGPEAFEGQVIHSMDYSKMDSKTATNFVKGKQVAVVGFQRSGMDIAMECSTVNGVERPCTVLIRTPHWNLPADFSPWGLKLGYLYSSRFSELMVHKPGEGLLLSLLATTLSPLRWALSKYVESYIKHKNRLAKYGMVPEHSILNECSVAVVPEGFYDRVEEGSIKLIKKAECFGFSKEGIVLEGEAETIKSELVILATGFKGIDKLKHIFESTKYQEFIAGSDDSATVPLYRECIYPQIPQVAIIGFSESIATLYTSEIRCRWLAELLDGKFKVPSIKVMEKDIAEWDKYQKRYSYNKYYRKSCIAALHVWHNDQLCKDMGWNPKRKNGFWAEWFQPYGPMDY

>Soltu.DM.01G051740|Stuberosum_v6.1/1-524

MESFNERESRKKEVIAIIGAGISGLLACKYAITKGFDPIVFESESSIGGVWSKTIESTKLQTPKPLYQFSDFPWPDSVTEMFPDHQTVLEYIESYARHFDLLRHIHFNSKVLSLSYEDGEPSLSGDWNDAFSSKGKWNVNVQDTLTLSTQVYQVDFVVVCVGRFSQVPNIPEFPPNKGPEAFEGQVIHSIDYSKMDSTTATNFVKGKQVAVVGFQKSGMDIAMESSTVNGIERPCTVVIRTPHWNVPDYFPWGFPMAKLYLNRFSELTVHKPGEGLLLYLIATILSPLRWGFSKFVESHIKHKLKLSKHGMVPEHSFLNELSTCLISTVPEGFYDRVEEGSIKLVKKAKSFGFTKQGIVVEGQAEPIKSDLVILATGFKGIDKLKHIFESPKYQEFIAGSDDSSVPLYRECIHPRIPQLAIIGFSESIANLFTSEIRCRWLAEFLDGRFKLPNIKVKEEEIAEWDKYKKRYSYNKYYKRSCIGALHIWHNDQLCKDMGWNPKRKNSFWAEWFQPYGPMDYLFQQ

>Soltu.DM.01G051720|Stuberosum_v6.1/1-516

MANCDGRRKEVIAIIGGGISGLLACKYAISKGFDPIVFESESSIGGVWSKTIESTKLQTPKPLYQFSDFPWPDSVTEVFPDQHTVLEYIESYARHFDLVRHIQFNNKVLSLSYEGDGDSSTGEWMSKGKWNVTVQHTPTLSIKVYQVDYVVVCVGKFSQVPNIPEFPENKGPEAFEGKVIHSMDYSKMDSTTATDFVKGKHVAVVGFQKSGLDIAMESSTVNGIERPCTVIIRTPHWNVPDYFPWGFPMAKLYLNRFSELTVHKPGEGLLLYLIATILSPLRWGFSKFVESYIKHKLRLSKHRMVPDHSFLNELSACLISTVPQGFYDRVEEGRLKLKKAKSFGFTKQGIVLEGQAEPIKSDLVILATGFKGIDKLKHIFESPKYQQLIAGSDDSAAVPLYRECIHPQIPQLAIIGFSESIANLYTSEIRCRWLTELLDGKFKLPSIKMMEKDIAEWDKYKKRYSKKYYRKSCIGALHIWHNDQLCKDMGWNPKRKKGLWAEWFEPYGPMDYTGPI

>Solyc01G004381|Slycopersicum_ITAG5.0/1-510

MANCDGRRIAIIGGGISGLLACKYAISKGFDPIVFESESSIGGVWSKTIESTKLQTPKPLYQFSDFPWPDSVTQVFPDQHTVLEYIKSYARHFDLVRHIQFNNKVLSLSYEDEDDREWMSKGKWNVTVQQTQTLSTKVYQVDYVVVCVGRFSQVPNIPEFPENKGPEAFEGKVIHSMDYSKMDSTTATEFVKGKHVAVVGFQKSGLDIAMESSTVNGVERPCTVVVRTPHWNVPDYFPWGFPMAKLYLNRFSELTVHKPGEGLLLYLIATILSPLRWGFSKFVESYIKHKLRLSKHGMVPDHSFLNELSACLISTVPQGFYDRVEEGRLKLKKAKSFGFTKQGIVLEGQAEPIKSDLVILATGFRGIDKLKHIFESPKYQQLIAGSDDSAAVPLYRECIHPQIPQLAIIGFSESIANLYTSEIRCRWLAELLDGKFKLPSVKMMEKDIAEWDKYKKRYSKKYYRRSCIGALHIWHNDQLCKDMGWNPKRKKGLWAEWFEPYGPMDYTGPI

>evm_27.TU.AmTr_v1.0_scaffold00001.33|Atrichopoda_v1.0/1-517

MEKRVGIVGAGSSGLIACKHLMQRGFDPIIFEAKGRIGGIWTKTIESTKLQTPKDAYQFSDFPWPESVTEEFPNHDQVLQYIESYAKHFGVVERVRFNNKVLGLEFVGAREEDQLSWGLWGGNGEAFGSKGRWDITVEDTNFGSTEVHQVEFVVLCIGRFGDVPKLPLLPQNNGPQVFKGEVMHAKDYSELSSSEAAYAVRGKRVAVVGFQKSALDIAAECARVNGKQIPCNVILREPHWNVPHYLPWGVHLAYLYCTRFSELLVHKPQEGFLLSLLATLLSPVRWGISKFAESYLRWKLPLKKYGMVPNHSFLLQISSCQLSTLPPDFYDKVEEGSILLKQAPKWGFCKEGLILNDKSEPLDMDLVILATGYDGDQKLKGIFQSPTFQKYIMRSETNTLPLYREIIHPRIPQLAVIGYSESLSNLFTSEMRSQWLCHFLDGGFRLPSIKEMEDDLKKWEAYMKSYAGKYYWRSCIGVLHIWYNDQLCKDMGYNPKRKKGWYDELFSPYGVMDYCKL

>Pn21.263|Piper_nigrum/1-518

MDKKVGIVGAGVSGLLACKYVLEKGLRPVVFEEQGCTGGVWTRTIESTSLQTFRDYYQISDFPWPSSVKEAFPSSQQVMEYLSSYAYHFDLYRYIKFNSRVVGVEFVGVSDGELQSWDLWGGTGEAFSSNGRWQIKVEDVTNQSQEIYEVEFVILCVGRFGGIPNIPEFPAGGGGPEAFDGEVMHAMDYAALDNVSAAKLIKGKRVTVVGFQKSAVDIAAECANANGVEHPCTVVCRTVQWHLPDFLPWGVSLVYLYLTRVSELLAHKPGEGLLLSLIASILAPIRWGMSKFVESYLKWKLPLKKYGMVPKHSFHQQIFSCKAVLLPDKFYDKVEEGSIILKKMKDLSFCRNGLNLDNQQLPVESDLVIFATGYKSDQKLKNIFTSITFQKRLSDSFNSTIPLYRECIPPQIPQLAIIGHSESLSNLFTSEMRCRWLAHFLAGGLHLPSIRDMEEDASRWQSYYRKYSGNSYNTSCVGALNVWYCDQLCKDIGCNPRRKSGLYSEWFTPYLAMDYAQL

>Acora.04G033200|Aamericanus_v1.1/1-520

MEKTVGIVGAGMSGLLACKYALEKGYRPIVFEADRAIGGIWAHTIESTRLQTPREGYSFSDFPWPPDVTDEFPDHNQVMDYLEAYARRFDLKRHIKFNKRVLDIDYVGVSEDKILSWDLWSGTGEALNPSGWWQIRVQDTNLGSIEIYEVGFVIVCVGHFSGVPNVPDFPVKEGPEMFGGDVIHAMDYSNMDDASAARFVKGKRVTVVGFQKSALDIAAECARANGVEKPCTMICRTPSWFVPNYTAMGVSVKKFYLSRFSELFVHKPGEGTMLSLLATVASPLKWMFSKVMESHLRWKFPLKKYDMVPDQSFSEAASACRTPMLPNNFYNMVEEGSIVLKRSKGFKFCEKGVIVDGDEAPIEADLVIFATGYKGDQKLRDIFKSTTFQKALMGTSTAVTPLYRECIHPRIPQLAIIGFLESPSSLFPAEMRCKWLSHLLGGGFKLPSVKSMEAEVAKWERYIRRYSGEGCRWSCVGTLHIWFNDKLCKDMGWSHNRKKGFLSDWLSPYGPADYAEPSTV

>Acora.08G207100|Aamericanus_v1.1/1-523

MERRVGIVGAGVSGLLACKYALEKGLNPIVFEADRTIGGVWAHTVESTRLQTPKAFYSFSDFPWPAHVKDDFPTTHQVVDYLDSYARRFDLIRHIKFNTRVLQIEYLGVGDDEILSWDLWSGTGGAFHPGGKWQITVQDTTDLQSVQTYEVEFVVLSVGRFSGLPNVPSFPPNGGPDAFRGEVIHAMEYSNMDNDAAARFVKGKRVTIVGFLKSALDIAAECARANRVDRPCTMICRTPRWLIPDYNAWGVPFVMFYLSRFSELLVHKPGEGVLLALLASFLSPLRWFFSKFVESYLLWRFPLKKYGVIPNHRFAEAFTSCQLAILPMNFYEMVEEGSIVLKKSESFRFCEKGVIVDGDGEDALLETDLVILATGYKGDQKLKDIFKSAAFGEAVVGTSDSTVPLYRECIPPRIPQLAIIGYSESLSNMYTSEMRCRWLVHFLDGEFKLPSIRAMEEDIKGWEQYMKRYAGDGYRRSCVGALHVWYNDQLCKDMGWNHRRKNGFLADWLVPYGPIDYDGPSPK

>XP_020583434.1|Phalaenopsis_equestris/1-527

MEFCLIGAGISGLTTCKYLLQRGFRPVVFEAQPSIGGLWRTTPASTRLQSLRWDYQFSDFPWPEKVTEVCPDNRQVMETEYIESYALQFDLIRHVRFGERVEGIEYVGVGEEEMEAWDLWGGDGELFGGGRQGRWHVTVRREDDGAIEVHVMDFVILCIGRFSGVPNIPTFPKGKGPEAFKGEVIHSMDYSNMHNAAAAELVKGKRVTVVGFLKSALDIAAECANINGPEIPCTMIVRTKRWNISQLTAWGIPLPYMYLNRFSELLLHKPGEGLMLSLLATLLSPLRWIFSKFTESYLIKTIPMKKYGMVPEHSFFQDMAAGLFCILPKNFYGKVEEGSIIMKPSKTFEFCNNGVLIEDGTTSIESDLVIYATGYKGDQKLRNIFISPWFQKIVAGTEDTIVPLYRECIHPHIPQMAIIGYSENLSNLFTSEMRARWLACFLDGGFKLPSKRNMEKNIKEWDLFYKTYLGSSSKCYRRNCITVVNTWYYDQLCIDMGCNPRRKKRFFTDLFIPYCPTDYAKLESKLQ

>XP_020585319.1|Phalaenopsis_equestris/1-522

MGEKRVCVVGAGISGLVTCKYLLNKGFHPVIFEADDSIGGVWAKNTLDSTRLQSPTSAYCFSDFPWPETVNGMPHHTEVMRYIGDYARQFDLLRYVRLREKVVGLDYVGVAEEEMVGWKQWGGIGQAFAGASGGPGVWRVTVQRAEDGSVEVHMMDFVVLCLGRFCDLPNIPSFPNNQGPEVFDGMVIHSMDIARMGRAAATELVEGKRVVIIGCLKSALDVAAECSDINGTEHPCTMLFRTKRWIIPDFTAWGFSTSYFYENRFSELLLHKPNEGIMLYLLAIFLSPLRWIFSKFVEVYYKRILPMKKYGMVPNHSFFQAMTTCLVAILPDKFYDKVEDGSIVLRQSKNFVFCKEGIMVDGDSMPIKADLVIFCTGFKGDQKLINIFESPMFQNLMAGCLDSTVPLYRECIHPRIPQLAVIGYSESLANLYTSEMRARWLANLLGGFQLPNIENMEKDIIEWDKFMKRYSHKYYRRSSIGTIHICYNDQLCKDIGCNPKRKKGFFANLFLPYGPLDYVGVG

>XP_020585627.1|Phalaenopsis_equestris/1-523

METKRVCVVGAGISGLVTCKYLLHKGFRPVVYETGRSIGGVWASTLGSTRLQTSTQSYRFTDFPWPESVAGAPNHEQVMEYLEAYARRFDLMRHVRLGEKVVGLDYVGSNREQMAVWDLWGGTGGAFAEAGCGAWKVTVEKEDGVVVEHVMDFVVLCLGRFSGLPNIPNFPKNKGPEVFNGKVIHSMDLANMGSTAAAKFVNGKRIVIVGFLKSALDVAAECANINGAELPCTMIFRTKRWNVLDFSAWGVPFSYIYFTRFAELLFHKPGEGIILSILATMLSPLRWVISKFVESYIKKTTPIEKYGMVPDHSFFEAMSTTLFSTLPENFYERVKNDSIILKHTKSFEFCQDGVILEGESTPIKADLVIFATGFKGDQKLQNIFHSPSLQEIVIGSSENTVPLYRECIHPRIPQMAVIGYSESLSNLYTSEIRAMWLASFLDGGFRLPSIKLMEEDVKKWDKYMKEYSHEHYRRSSIGTIHIWYNDQLCKDMGCNPKRKKWFLSELFVPYGPNDYVGLDSKVE

>XP_020598528.1|Phalaenopsis_equestris/1-406

MESKRVCVVGAGISGLVTCKYLLEKGFRPAVFEALGASRSIGGVWASPLGSTRLQTPTDFYRFTDFPWQESITGAPNHEQVMEYLEAYARRFDLMKHIRFGEKVVGLDYVGSDWEQMAFWDLWGRTGDAFAEDGRGVWNVTVEKEDGAVVHVMDFVVLCLGRFSGLPNIPNFANNKGPEAFNGKVIHSMDLANMGSTAAAKLVNGKHVVIVGFLKSALDVAAECANINGPELPCTMIVRTKRWNVLDFSAWGVPFSYLYFNRFSELLFHKPGEGIILSFLATMLSPFRWVISKLVESYIKKTTPIQKYGMVPDHSFFEAMSAALFGIIPEKFYDRVKNNSIILKHSKSFEFCEDGVILEGESTPIKADLVIFATGFKGDQKVRNIFNSPSLQEIVTGSSENTVPLY

>XP_020585633.1|Phalaenopsis_equestris/1-539

MALTETPSDQLMESKRVCVVGAGISGLVTCKYLLEKGFRPVVFESSRSIGGVWASPLGSTRLQTPTDYYRFTDFPWPESVTGVPNHRQVMEYLEAYARRFDLIKHVRFGEKVVGLDYVGSDLEEMAVWDLWGRSGDAFAGDGRGVWNITVEKDDGATAEHVMDFVIICLGRFSGLPNIPDFPKNRKNKGPEVFVFNGKVIHSMDLANMGSTAAMEFVNGKRIVVIGFLKSALDIAAECADINGVDLPCTMIVRTKRWNVLDFSVCGIPFSYYCCTRFSELLFHKPGEGTILSLLATMLTPLRWVISKIVESYIKKITPIEKYGMVPDHSFFEAASSILVAILPEKFYERVNNKSILLKHSKKFEFCQDGVILEGESVPIKADLVIFATGYKGDQKLRNIFKSPSIQEIVTGSSENTVPLYRECIHPRIPQMAIIGYSESYSNLYTSEIRAMWLSYFLKGGFRLPRIKLMEEDVKEWDKYMKKYSREHYRRSSIGVIHIWYNDQLCKDMGYNPRRKKWFLPELFMPYGPNDYVGLGSKME

>Nycol.I00670|Ncolorata_v1.2b/1-521

MAAVKKVIGIIGAGVSGLIACKYVLQKGFHPVVFEASDAIGGVWTQTIASTRLQTPKFVYEFSDFPWPPHVNDAFPDHDQVLHYLQSYAQHFDLLKHVRFNCRVEGVEYVAASGDGQVVSCDRWGGTGDAFGSEGCWHITFHDALLKSNHVMEVDFIILCIGRFSDFPNLPEFPPNKGPEIFRGQVMHSMDYSRLSDSDAAVLVSGKRVVVVGFQKSAVDIAAECAKANGLKSPCTLLFRHPQWTIPSFNAWGVNLAFLYLSRLSEFLVHKPNEGALLSLLATCLSPLRWMRSKFVESYIKWKLPLKKYNIVPNHSFDEQISSCRIALLPDRFYSCVEEGSIMLKRAANWSFCEKGVVCEQDASPVEADLVILATGYRGDKKLMNIFKSPLFQKIVSGSESNTLGLYRECIHPRIPQAAIIGYSESLSNLYTSEMRIRWVVEFMDGGFKLPSTRQMEEDAKKWEKYMKQYSGSHFRRSCVGAVHIWYNDQLCEDMRRNPRRKGSWFEELFIPYFSKDYANL

>Nycol.I00669|Ncolorata_v1.2b/1-520

MAAVKKVGIIGAGVSGLIACKYVLQKGFHPVVFEASDSIGGVWTQTVASTRLQTPRFLYEFIDFPWPPHVKEVYPDHNQVLHYLQSYADHFDLLKHIRFNCRVEGVEYVDASGDGQLMSWEEWGGTGEDFGSKGCWHITFHDKLLRSDHVMEVDFIIMCIGRFSDFPNLPDFPPNKGPEVFRGQVMHSMDYSRMSDSDAAALVSGKRVIVVGFQKSALDIAAECAEANGVKTPCTVLFRDPQWNLPGFSVWGVNLGFLYLNRVSELLVHKPGEGALLSLLATCLSPLRWMRAKFVESYIKWKVPLKKYNVVPSHSFEEQISSCRIVILPDKFYSNVEEGSIILKRATNWSFCEKGVVCESDDSPVEADIVILATGYRGDKKLKNIFKSPLFQKIISRSEFDTLGLYRECIHPRIPQAAIIGYSESLSNLYTSEMRTRWVVEFMDGGFKLPSIRQMEEDAKKWEKYMKQHAGKHFRRSCVGALHIWYNDQLCKEMGRNPRRKGSWFEELFIPYLSRDYANL

>g10929.t1_1|Euryale_ferox/1-520

MADVKKVGIIGAGVSGLIACKYVLQKGFHPVVFEASDSIGGVWTQTVASTRLQTPRFLYEFSDFPWPPHVKDVFPDHDQVLCYLQSYAQHFDLLKHIRFNCRVEGVEYVAASGDGELISWDKWGGTGEAFGSKRCWHITFHNTLLQSNQVMEVDFIILCIGRFSDFPNLPEFPPKRGPEIFQGQVLHSMDYSRMSYADAAALVAGKRVIVVGFQKSAVDIAAECAEANGVRTPCTVLLRDPQWNLPGFSAWGVSLAFLYLNRLSEFLVHKPGEGALLGLLATCLSPLRWMRTKFVESYIKWKLPLKKYNIVPSHSFDEQLSSCRVVLLPERFYSNVEEGSIILKKAANWSFCEKGVVCDGDASPMEADIVILATGYRGDKKLKSIFKSPLFQNIISRSESSTVGMYRECIHPLIPHAAIIGYSESLSNLYTSEMRIRWVVEFMDGGLKLPSIKEMEEDAMKWEKYMKQYAGSYFRRSCVGALHIWYNDQLCKEMGRNPRRKGSWFEELFIPYLAKDYTNL

>g12536.t1_1|Euryale_ferox/1-520

MAGVKKVGIIGAGVSGLIACKYVLQKGFHPIVLEASDSIGGVWTQTVASTKLQTPRFLYEFSDFPWPPHVKDDFPDHDQVLCYLQSYAQHFDLLKHIRFNCQVEGVEYVAASGDGELMSWDKWGGTGEAFAYAGCWHVIFHDKLLQSNQVMEVDFIILCIGRFSDFPNLPEFPPNKGPEIFRGQVLHSMDYSRMADADADALVSGKRVIVVGFQKSAIDIAAECAEANGTKIPCTVLLKDPQWNFPSFNAWGVNLGFLYLNRLSEFLVHKPGEAALFSLLATCLSPLRWIRAKFVESYLRWKLPLKKYNIVPSHGVDEQLISCRVAFLPKGFYSNVEKGSIILKKAANWSFCDKGVVCDGDTSPMEADLVILATGYRGDKKLKNIFKSPLFQNIISRAASSTPGMYRECIHPRIPQAAIIGYSESWSNLHTSEMIMRWVVEFMDGGLKLPCIKEMEEDAMKWEKYMKQYAGKHFRRSCIGTLHIWYNDQLCKEIGRNPRRKGNWFEELFIPYLPKDYANL

>Cucsa.118920|Csativus_v1.0/1-520

MMEQKKVAIIGAGVSGLAACKFTLSKGLIPMVFEARGDIGGVWTETLETTALQTPKEMFQFSDFPWPKSVTEEFPRYDQVLDYLRSYAQHFGLLNHIRFNSRIVGIDYEGCSDEEMKGWTLWGGSGEAFDERRKWRLNVVDARTNIPLQAIVVDFVVLCTGRFCDIPNIPEFPPNEGPEAFKAGQVLHSLQYSAMDFHTASNLIKDKRVTVVGFQKSALDLAMECANANGPNKPCTVLYKTEHWNLPNHHPWGIPLSLLYMNRFAELLIHKPGEGFFLYLLAVLLSPIRWLFSKIVETYVIRKNRLAKYGMVPTHSFLQSISACVIATLPEKFYDKVDEGSIILKKSQSFSFCEEGIMIEGETEPICSDLVILATGYRGDQKLKQIFTSSTLRDYMTFHNSTISLYRLCVHPRIPQLAVVGFTESSSNLFTSEMRCRWLAEFMDGTFKLPSIKEMENDIAKWEKTFKHYSGPFVKRACIAILHIWYCDQLCKDIGWNPRRKKGFFADLFLPYGPLDYVSP

>Cucsa.119370|Csativus_v1.0/1-522

MEPKKVGIIGAGISGLAACKFVLSKGFIPIVLEARGDIGGVWTETLQTTALQTPKEMFQFSDFPWPKSVTEELPRYDQVLDYINSYAQHFGLLKHIRFNTRVVSIQYEGCSDEEIGGWNLWGGSGDAFAEGRKWRLNVVDARTDVPVEETGEFVVDFVVLCIGKFSDVPNIPEFPPNGGPEAFKAGKVLHSSEFSAMDFDNASNLIKNKLVTVVGFQKSGIDLAMECANANGPNKPCTVLCRTKHWSLTHYYPWGIPLAFLYMNRFAELLIHKPGEGFLLYLLALLLSPIRWLFSKIVETYMMKKLGLAKYGMVPTQSFLQDISSCLFAIIPENFYDKVEEGSIIFKQSQSFSFCEEGIMIDGETEPIRSDLVILATGFRGDLKLKEIFASSMFRDYMTFHDLAAPMYRHCIHPRIPQLAVIGYSESASNLYTSEIRCRWLAEFLDGTFKLPSIKEMDKDIANWEKCMRLYSGPFYKRASIAILHIWYNDQLCKDMGWNPKRKKGFLADLFLPYGPSDYATA

>Cucsa.119710|Csativus_v1.0/1-523

MSKMMEQKKVAIIGAGISGLAACKFIISKGMVPIVLDARGVIGGVWNETLKSTVLQTPTHMFRFSDFPWSKSVTAEFPMHNQVLDYIKSYAEHFGLLKYIRLNSKVVSIEYEGFSDEEIEGWTHWGHSGNAFAKGSKWRLNVVDAQTNAPLQEVVADFVVLCIGRFSDVPNIPNFPPNKGPEAFKAGKVLHSLQYSAMDFDSAANLIKDKRVTVVGFHKSALDLAMECANTNGPKNPCTLLYRTKHWNPPDTTPWGISLGFLYMNRFAELMIHKPGEGLLLYLLVILFSPIRWLISKLVETHIKRKVKLAKYGMIPKQSFLQDISSCVYAGLPNNFYDKVDEGSIILKKAPSFSFCEEGIMIKGETKPVHSDLVVLATGYRGDLKLKDIFASSTFRDYMFFGDSVVPMYRQCIHPRVPQLAVIGFSESNSNLYTSEIRSRWLAEFLDGTFKLPSVKEMEKDIANWEKCLKLYSGPFYKRGCIATLHNWYNDQLCKDMGWNPKRKKGFFADLFLPYGPTDYAFP

>Cucsa.119550|Csativus_v1.0/1-522

METKKVAVVGAGLSGLIACKLLLSKGLTPIVFEAKDVIGGLWNDTIKSTLLQTRRQMFELSDFPWPKSVTEEYPRYDQVLDYLRSYAEHFGLFKYIRLNTKVLSIEYEGFSEEEIDSWTHWGGSGNAFSEQGKWKLNLVDARTNLPLQEEVVDFVVLCTGKFGDIPNIPKFPPGGGPKAFKNGKVLHYIEYAALDFDTATKHVKDKKIAIVGFQKSALELIRECTNLIGTTKPCTLIYKTEYWNPPDAQPWGIHIDYLFASRFAELLIHKPGEGFLLYLLAMLLAPIRWLITKLAEFHVRRKTKMDKYGMVPKHSILQDVTSCRYAVLPERFYERVEEGSIVLKKAPSFSFCEEGIMIQGETKPIHLDLVILATGYRGDLKYRNIFASSTFRDYMSFGDAALPLYRLCIHPRIPQVAVIGLTESISNLYTSEIRCRWLVEFLAGTFKLPSIKKMEKDIENWERCLKLYSGESYWRGCIGMLHHHYNDQVCKDIGWNPRRKKGFFANLFVPHGPRDYASPDNW

>LOC_Os09g37620|Osativa_v7.0/1-519

MDRKRVGIIGAGVSGLAACKHSLDKGFNPIVFEADDTIGGVWAHTLESTRLQAPTTAFRFSDLAWPATVTEKYPSHRKVMEYLRSYASEFDLLKCIRFNSQVLGVEYLGATEGEIMQWEHWSGNGEAFGAQKDGVWRLTVKDLKIGNIEVFLVDFLIVCIGRHSGSPNIPEFPANSGLELFKGKILHSIDYSYMDNAAEFVKGKKVTIIGSGKSAFDIAAEVAKVNGETQPCTMIYRTRHWLVHKSSICGVDLSYFYLNRISQLLVHKPGEGFLYYVLATALSPLRWAISKVIETYFKQSIPLQKHGMVPDYSFSFAMSSCLIAMLPEGFYDKVDEGSIILKKSKRFSFFNDGIILEDGNEHIKSDIVILATGFRGDQKLRDIFTANWCKEKVAGSSATAVPLYRECIHPRIPQLAIVGYSESLTNIYASERMANWVTHFLGGRFKLPSIRCMEESVAEWAKYKDLYNGKYFRRSCISTVNIWFNDILCQDIGCNPKRKKGVLAEWFQPYGPADYASLY

>Bradi4g37110|Bdistachyon_v3.2/1-519

MESKRVGIVGAGVSGLAACKHALDKGFSPVVFEADDTVGGVWAHTLQSTRLQAPTSSFRFSDLAWPASVTATYPSHHEVMEYLRSYACQFDLLKCIKFNSQVLGIDYLGATEEEIMGWEHWSGNGKALGAGKDGGWRLTVKDLKIGNVEVFEVDFLILCIGRHSGTPNIPEFPENKGFELFKGKILHSVDYSYMDDVAPFVKGKSVTIIGSGKSAFDIAAEVAKVNGTAQPCTMIYRTKHWLVHKSSIWGINLSYFYLNRISQLLVHKPGEGFLHYILASALAPLRWAITKVIETYFKQSIPLQKHGMVPDYSFSFAMSSCLIAMLPEGFYDRVDEGSIVLKKSKRFSFYSDGIILEDRNECIKSDIIILATGFRGDQKLRDIFMENWCRKIVVGSSDTPVPLYRECIHPRIPQLAIVGYSESLTNIYASERMAHWVIHFLAGGFQLPSIRCMEESVAEWGKYKDIYNGKYFRRSCISTVNIWFNDLLCKDIGCTPRRKKGFLAEWFQPYGPADYAGLC

>HORVU.MOREX.r3.5HG0502780|Hvulgare_Morex_V3/1-519

MDRKRVGIVGAGVSGLAACKHALDKGFSPVVFEADDTIGGVWAHTLESTRLQAPTTSFRFSDLAWPASVTATYPGHREVMEYIRSYVCEFDLLKCIKFNSQVLGVEYLGATEEEIMEWEHWSGNGTAFGAGKDGGWSITVKDLKIGNVEVFQVDFLILCIGRHSGTPNIPEFPANKGPELFKGKILHSIDYSYMDNVAEFVKGKSVTIIGSGKSAFDIAAEVAKVNDVDQPCTMICRTRHWLVDKSSIWGINLSYFYLNRISQLLVHKPGEGFLHYLLASALSPLRWAITKVIETYFKQRIPLEKHGMVPDYSYSFAMSSCLIALLPEGFYDRVDEGSIILKKSKRFSFCSNGIILEDGNECIKSDIVILATGFRGDQKLRDIFTANWCRNIVVGSSDTSVPLYRECIHPRIPQLAIVGYSESLTNIYASERMANWVIHFLAGGFQLPSIRCMEENVAEWAKYKDLYNGKYFRRSCISTVNIWFNDLLCQDIGCNPRRKKGFLAEWFQPYGPADYAGIC

>HORVU.MOREX.r3.5HG0502770|Hvulgare_Morex_V3/1-520

MDRKRVGIVGAGVSGLAACKHALDKGFSPVVFEADDTIGGVWAHTLESTRLQAPTTSFRFSDLAWPAGVTATYPGHRQVMEYLRLYAREFDLLKCIKFNSQVLGVEYFGANEEEIMGWEHWSGDDGKAFRVAKDRGWNLTVKDLKNGNIEVFQVDFLILCIGRHSGTPNIPEFPANKGPEFFKGKILHSMDYSYMDNAAEFVKGKSVTIIGSGKSAFDIAAEVAKVNGAAQPCTMVHRTIHWRVDKFSIWGVNLNYFYLNRISQLFVHKPGEGFLHYMLASALSPLRWASTKVIETYLKQSIPLQKHGMVPDYSFSFAMSSCLIAMLPKGFYDRVEDGSIILKKSKRFSFCSDGIILEDGEESIKSDIVILATGFKGDQKLRDIFTANWCRNIVAGSSDTSVPLYRECIHPRIPQLAIVGYSESLTDIYVSERIANWVIHFLAGGFQLPSIRRMEGSVAEWAKYKNLYNGKYFRRSCLSTVNIWFNDLLCQDIGCNPRRKKGFLAEWFQPYGPADYAGLS

>Zm00001d021444|Zmays_RefGen_V4/1-521

MDSKSVGIVGAGVSGLAACKHALDKGFRPVVFEADGSVGGVWAHTLESTRLQAPTTAFRFSDLAWPPESAREAYPGHRRVMEYIRSYARAFDLLKHIRFNSQVLGVEYLGATEEEVMGWDQWSGDGTAFGAVKGGGWRLTVQDLKVGSTEVFVVDFLILCIGRHSGTPNIPEFPANGPELFKGKILHSLDYSYMDDGDAAEFVRGKRVTVIGSGKSAFDIAAEVAKVNGAAQPCTIVYRTRHWLVHKSSVWGVDLSYFYLNRISQLLVHKPGEGFLRCMLAAALSPLRWAISKAIETYYKWSVPLRKHGMVPDYSFSFAMSSCLIAMLPEGFYDRVDEGSIVLRKSKAVGFSSDGVVLRDRNESVRSDIVILATGFRGDQKLRDIFTANWCREIVAGSPDTAAPLYRECIHPRIPQLAIVGYSESLTNIYASERMSNWVAQFLAGGFQLPSVRCMEESVAEWAEYKNRYNGRYFRRSCVSTANIWLNDLLCRDMGCNPRRKKGFLAEWFQPYGPADYAGLC

>Sobic.002G287000|Sbicolor_v5.1/1-520

MDSNSKRVGIVGAGVSGLAACKHALDKGFRPVVFEADGTIGGVWAHTLESTRLQAPTTAFRFSDLAWPESVREAYPNHRRVMEYIRSYACAFDLLKHIRFNSQVLGVEYLGAAEEEIMSWEQWSGNGTAFGAVKGGGWRLTVQDLKVGSTEVFMVDFLILCIGRHSGTPNIPEFPGNGPELFKGKILHSLDYSYMDDVAQFVKGKCVTIIGSGKSAFDIAAEVAKVNGATQPCTIIYRTKHWLVHKSSIWGVDLSYFYLNRISQLLVHKPGEGFLRYMLATALSPLRWAISKVIETYYKWSIPLQKHGMVPDYSFSFAMSSCLIAMLPEGFYDRVDKGSIILKKSKAFNFSSDGIILQDRNESIKCDIVILATGFRGDQKLRDIFTANWCRNIVAGSPDTAAPLYRECIHPRIPQLAIVGYAESLTNIYASERMSNWVAHFLAGSFQLPSIRCMEKSVAEWAEYKNLYNGKYFRRSCISSTNIWLNDLLCQDMGCNPKRKKGFLAEWFQPYGPADYAGLC

>ELECO.r07.6BG0481180|Ecoracana_v1.1/1-518

MERKRVGIVGAGVSGLAACKHALDKGFSPVVFEADESIGGVWTHTLESTRLQATTRAYRFSDLAWPANVTEKYPSHCKVMDYIKSYACKFDLLRYIRFNSQVLGVEYFGATEEDIMSWELWSGNGTAFGTGKDGVWRITVKDSKVGSTEVFQMDFLILCIGRHSGNPNIPGFKANGPELFRGKILHSLDYSYMDNVAEFVKGKHVTIVGSGKSAFDIAAEVARVNGTSQPCTMIYRTKHWLVHKASVWGVELGYFYLNRISQLLVHKPGEGFLHYILATAFSPLRWAISKVIETYFKWNIPLQKHGMVPDYSFSYAMSACLIAMLPEGFYDMVDEGSIILKKSKTFSFCDKGIILQDGNECIKSDIVILATGFRGDQKLRDIFTANWCKRIVTGSPDTAVPLYRECIHPRIPQLAIVGYSESLTDLYASERMANWVAHFLGGSFQLPSIRCMDKSIAEWAKYKNRYNGEYYRRSCISTINIWFNDLLCQDIGCNPKRKNGFLAEWFQPYGPADYAGLY

>ELECO.r07.6AG0527240|Ecoracana_v1.1/1-518

MERKRVGIVGAGVSGLAACKHALDKGFSPVVFEADESIGGVWTHTLESTRLQATTRAYRFSDLAWPASVTEKYPSHCKVMDYIKSYACKFDLLRYIRFNSQVLGVEYFGATEEDIMSWELWSGNGTAFGTGKDGVWRITVKDSKVGSTEVFQMDFLILCIGRHSGNPNIPGFKANGPELFRGKILHSLDYSYMDNVAEFVKGKHVTIIGSGKSAFDIAAEVARVNGTSQPCTMIYRTKHWLVHKASVWGVELGYFYLNRISQLLVHKPGEGFLHYILATAFSPLRWAISKVIETYFKRNIPLQKHGMVPDYSFSYAMSACLIAMLPEGFYDMVDEGSIILKKSKTFSFCDKGIILQDGNECIKSDIVILATGFRGDLKLRDIFTANWCKRIVTGSPDTAVPLYRECIHPRIPQLAIVGYSESLTDLYASERMANWVAHFLGGSFQLPSIRCMEKSIAEWTKYKNRYNGEYYRRSCISTINIWFNDLLCQDIGCNPKRKNGFLAEWFQPYGPADYAGLY

>Aco013043|Acomosus_v3/1-219

MEKHGMVPEYSFFRAMSSCLIAMLPEGFYSRVEEGSIVLKKSKTIRFSDQRGVLLDGEDEPTIEADVVIFATGFSGDQKLRDIFVSPWLQKIVVGPSNRTVPLYRECIHPRIPQLAIIGYSESLTNIYAAETMSRWVSHFLGGRFRLPSVRCMEKDVAEWEKYKKRYNMKYFRRSCVSRVNIWYNDLLCRDMGCDRKRKKGWFAEWFEPYGPADYAGLH

>Soltu.DM.07G002700|Stuberosum_v6.1/1-523

MEEKRVGIIGGGISGLLACKYTLEKGLIPIVFEASNAIGGVWTQTIESTRLQSPKRTFEFTDFPWPKSVRGRYPHNKEVLKYVEDYAKHFGLMGYIQLNTKVIGINYVGVSGYDMDSWEFWSGNGNAFGSEGKWQILVQHEDFTTQEYEVEFLILCIGRYSGLANMPEFPLGEGPDIFAGKVIHSMDFSAMDNESARELIKGKRVAVIGSQKSAIDIAAECANVNGPEIPCTLVQRTIPWALPSGCFWWGATLGNLYGSRFSELLVHKPGQNIIYSVVASLLAPMRWGFSKFVESYITWKLPLKKYNMVPKQSFLQDMSSCKSFLLPDNFYGKVEEGSIVLKKIQHFSFIKQGLILDGEVGKPIKADLVIFATGYKGQEKLKNMFSSKKFQNHIVGSPNSIIPLYRHIIHPRIPNLAIIGYSGSITNLHTSEMRCQWLAHFLDQTFKLPNIKEMENEIQKWEDYMKTYGGKEYKRSCIAALHIWYNDQLCKDIGCKNRRKKSFYEEWFQPYLQSDYVRLSPNN

>Solyc07G000240|Slycopersicum_ITAG5.0/1-523

MEEKRVGIIGGGISGLLACKYTLEKGLIPLVFEASNAIGGVWTQTIESTRLQSPKRTFEFTDFPWPKSVRGRYPHNKEVLKYVEDYAKHFGLMRYIQLNTKVIGIDYVGVCGDEMDSWEFWSGNGNAFGSKGKWQILVQHEDFTTQEYEVEFLILCIGRYSGLANMPEFPLGEGPDIFAGKVIHSMDFSAMDNESARELIKGKRVAVIGSQKSAIDIAAECANVNGHEFPCTLVQRTIPWALPSGCFWWGVTLGNLYGSRFSELLVHKPGQNIIYSVVASLLAPLRWGFSKFVESYITWTLPLKKYNMVPKQSFLQDMSSCKSFLLPDNFYGKVEEGSIVLKKIQHFSFIKQGLILDGEVDKPIKADLVIFATGYKGQEKLKNMFSSKKFQNHIVGSPNSVIPLYRHIIHPRIPNLAIIGYSGSITNLHTSEMRCQWLAHFLDGTFKLPSIKEMENDIQEWEEYMKTYAGKEYKRTCIAALHIWYNDQLCKDIGCKNKRKKTFYEEWFQPYLQSDYVRLSPNN

>Asa1G06364.1|Allium_sativum/1-471

TPKRAYRFFDFPWPDSVTDDCPTHSHLMDYLYSYARRFDLIPNIRFNTEVLGVDYVGASEDEIMAWDLWGGNGDAFGGSGEWHVTVKQGSSTTVLQFDFLVLSIGLFCNIPRIPIFPQGKGPEVLQGQVLHSKDCGLMDSESMLKLVKGKHLTVVGCLKSALDIAAECANANGRSEYPCTLLFRNKKWNIPQISAFGIPLGFFCGNRFSELLVHKPGEGIVHSMLATLLSPMACAISKLIETYYKWATPMKKYGMVPEYSFFEAVSSCTFAVLQEKFYERVEEGSIVLKNSKSFEFTKDGVILEDETSSLESDVGINCYWAGPAGYKGDENLKGMFMSPFYKTLAVRSQTTVTCPLYRECIHPRIPQLAVIGNSDSIANIHTSEMKAKWLAHLLDRKIQLPRIRDMEKDVIKWENFTKRYASGYYKRMSFGVCHIWFNDQLCNDMGCNPERKKGLISELFMPYLPTDYAEI

>Asa1G06366.1|Allium_sativum/1-517

MNKQRICIVGAGISGLTTCKHLLAKGFSPTVFEAGPTIGGVWAHTLSITRLQTPRIAYQFSDFPWSDSVTDYSPTHSQVMDYLYSYARHFDLIPNIRFNTEVLGVDYVGAPEDEIMAWDLWGGNGDAFGGAGEWHVTIKQGSSTTVLQFEFLVLCIGLFSNKPRIPTFPQGKGPEVFQGQVLHSKDCGLMDSESMLKLVKGKRVTVVGCLKSALDIAAECANANGSEYPCTLLFRNKKWNIPQLSAFGIPLGFFYGNRFSELLVHKPGEGIVHSMLATLLSPMAWAISKLIETYYKWTTPMKKYGMVPEYSFFEAISSCTFSILPEKFYERVEEGSIILKNSKSFEFTKDGVILEDATSPLDSDVVIFATGYKGDENLKGIFMSPFYKNLAVRSQATVTCPLYRECIHPRIPQLAVIGNSDSVANLHTSEMKAKWLAHLLDRKIQLPRIMDMEKDVIRWEIFMKRYTLGYYKRMSFGICHIWFNDQLCKDMGCNPKRKKGLISELFMPYLPTDYAEI

>Asa8G05210.1|Allium_sativum/1-518

MNKRRICIVGAGISGLITCKYLLAKGFHPTVFEAGPTIGGVWAHTLSITRLQSPRIAYQFSDFPWSDSVTDDCPTHSQVMNYLYSYARRYDLIPNIRFNTEVLGVDYVGAPEDEIMAWDLWGGNGDAFGGAGEWHVTVKHGSSTTVLQFDFLVLCIGLFSNKPRIPTFPQGKGPEVFQGQVLHSKDCGLMDNESMLKLVKGKRVTVVGCSKSALDIAAECANANGRTEYPCTLLFRNKKWNIPQLSAFGIPLAFFYGNRFSELLVHKPGEGIVHSMLATLLSPMAWAISKLIETYYKWTTPMKKYGMVPEYSFFEAISSCTISLLPEKFYERVKEGSIILKNSKSFEFTKDGVILEDETSPLESDVVIFATGYKGDENLKGIFMSPFYKNLAVRSQATVTCPLYRECIHPRIPQLAVIGNSESVANLHTSEMKAKWLAHLLDRKIQLPRIRDMEKDVIKWENFMKRYTLGYYKRMSFGICHIWFNDQLCKDMGCNPKRKKGLISELFMPYLPTDYAEI

>DCAR_415050|Dcarota_subsp_sativus_v3.0/1-513

MERQVAIIGAGISGLAACRYCKAKGLNPIVFESESSVGGVWRKTIRTTKLQTPKHFYQFSDFPWPASVTEDFPAQSQVFEYLQSYASHFDLLRHIRFRSQVVSIRYDGPPDAEMQAWSLWGGTNDHSNTKGKWHVTVKDQQALLTEVYQVSFVILCFGKFSNLPNIPEFPNDKGPEVFHGKVIHSAEYASMDHTAAANLIRDKKVAVVGFQKSALDIAMECSSVNGPEHPCTVVYRTKHWGIPYFLPFGIPLLYPYLNRFSELMVHKPGESLALSLLVSILTPLRWGISKYIERYMTSKFGLAKFDMVPEHSFLKEINSCSIAILPEDFYNRVEKGGIKLKMSQAFCFSEKGILFDDEVESEDVDMVILATGFKYFEKLKDIFVSPTFQSHIANASGLYRNCLHPRVPQLAVIGFSESISNLHTSEMSCRWLAELLDGTFSLPSIKEMEDDVKEWDKYMKKYCGSYSTKWCIGGLKIWYNDQLCKDMGWISKRKGGFFAELFEPYSPLDYIRP

>DCAR_415051|Dcarota_subsp_sativus_v3.0/1-513

METQVAIIGAGISGLAACKHCKSKGLNPIVFESEASVGGVWRKTIGITRLQTPKKIYQFSDFPWPPSVTDEFPPQSQVIQYLQSYAQHFDLLQHIRFGCQVVSLRYDGPSDEEMRAWSLWGGKGDPFDTKGKWHVTVQDQNTLSTKVYSVSFVILCFGKYNGLPDIPEFPKDKGPEVFQGKVMHSADYSAMDSTDAANLVRGKRVAVVGFRKSAVDIALECSTVNGPEHPCTVVYRTGHWGIPSVLPMGISIPFPYGTRFSELMVHKPDESFALSLLASILTPLSWGISKSIEIYISSYFRLGKFGTVPKHRLSKEIMSCHSVKLSEEFYDMVEKGSIKLKKSKAFCFSKKGILFDSEVESADVDLVILATGFKYFEKLKDIFVSSTFQSDITTAPGLYRNCIHPRVPQLAIIGFSESISNLHNAEMNCLWLSELLDGTFNLPSIKEMDDDIAKWDKYMKEYSGEYHYKRCIAGLQIWFNDQLCKDIGWFPKRKKGFMAELFEPYGPTDYVQS

>Kalax.0474s0015|Klaxiflora_v1.1/1-512

MERKVAIVGAGIAGLIACKYFLSKGYKPIVFESRSRLGGVWCKTIETTKLQTVKQFYEFMDFPWPESEETYPNHEEVLAYIESYAQHFDLIRHIAFDTEVVSIDYQGVDEEEIMAWGMWGGTGEVFSNKGRWNLKVENTRTRSSEVHQVDFVILALGLVRDVPYVPEFPTGEGPEVFQGDTIHAQDYADMDYDKARNFIRGKRVAVVGFGKFAVDITAECAAVNGKDRPCTLLFRRKHWFMLETKNWRNLLGYLYTSRFAELLADRPGRGSLHNLLATTLSPLRRLFSFLSESYLKWKFPYKKYGLVPKYHLSEAVTTCKVNIVPEGFFDRVDEGSIKLQKAEEFSFCKEGILVKREPEPVEVDLVIFATGYRGERKVQDIFVSKTFRNLLTGLDSKIAPLYRQCIHPRIPQLAIVGLAGSFSMLFSSEMMCKWVVELLDGTFKLPSVPEMEKCTKEWSEYTKRHGGKTCLPAIHIWYNDQLFKDMGLNPRRKNGFLAELFEPYSPSDYKIS

>Kalax.0210s0064|Klaxiflora_v1.1/1-512

MERKVAIVGAGIAGLIACKYFLSKGYKPIVFESRSRLGGVWCKTIETTKLQTIKQFYEFMDFPWPESEETYPNHEEVLAYIESYAQHFDLIRHITFDTEVVSIDYQGVDEEEIMAWGMWGGTGEVFSNKGKWNLKVENTRTRSSEVHQVDFVILALGLVRDVPYVPEFPTGKGPEVFQGDTIHAQDYADMDYDKARNFIRGKRVAVVGFGKFAVDITAECAAVNGKNRPCTLLFRRKHWFMPETKNWRNLLGYLYTSRFAELLADRPGRGCLHNLLATALSPLRWLFSFLSESYLKWKFPYKRYGLVPKYHLSEAVTTCKVNIVPEGFFDRVDEGSIKLQKAEEFSFCKEGILVKGEAEPVEVDLVIFATGYRGERKVQDIFVSKTFQNLLTGPDSKIAPLYRQCIHPRIPQLAIVGLAGSFSILFSSEMMCKWVVELLDRTFKLPSVPEMEKSTKEWSEYTKRHGGKTCLPAIHIWYNDQLFKDMGLNPRRKNGFLAELFEPYSPSDYKLS

>ELECO.r07.8BG0659700|Ecoracana_v1.1/1-524

MDSSNKKRVAIIGAGPSGLAACKHALAKGFVPVIFDSGASVGGVWTRTLASTKLQTPAAAFQFSDFPWPAGTSEFPSHDQVLGYLTAYARKFGVLECVKLGCKVVGAEYSGASEKEVTAWDRWSGNGEAFGDGSGEWHLTVEHIESGSIQKYEFDFLIVCVGRYGVTKIPTFPPNKGPEVFHGKVLHAQDYSHLEDHDAAELIRGKRVVVVGSGKSGLDTAAECAEANGSKYPCTLIYRTANWMVDHKLTWGLNVSKRTTTRLAELMVHKPEEGFALSLLATLLTPLRWLLSTVTETYYKMHIPMRKNGMVPDCTISQSLLGWKLGLLPDRFYDLVDEGSLVLNKCDSFSFCADGLVLDSGKRVEADVVILATGFNPDQLLRSVFTSPWVRDIVSGASSDTMLPLYRNIVHPRIPQMAIVGYLESRATIYPYEMMSKWVAHLLDGTVRLPSVKDMEKSVGEWALWGQGARRRSGSLFLKSCIATVTTWYHDQLCRDMGYSARRKKGFVAELMEPYGPTDYADIQ

>ELECO.r07.8BG0659710|Ecoracana_v1.1/1-524

MDSSNKKRVAIIGAGPSGLAACKHALAKGFVPVIFDSGASVGGVWTRTLASTKLQTPAAAFQFSDFPWPAGTSEFPSHDQVLGYLTAYARKFGVLECVKLGCKVVGAEYSGASEKEVTAWDRWSGNGEAFGDGSGEWHLTVEHIESGSIQKYEFDFLIVCVGRYGVTKIPTFPPNKGPEVFHGKVLHAQDYSHLEDHDAAELIRGKRVVVVGSGKSGLDTAAECAEANGSKYPCTLIYRTANWMVDHKLTWGLNVSKRTTTRLAELMVHKPEEGFALSLLATLLTPLRWLLSTVTETYYKMHIPMRKNGMVPDCTISQSLLGWKLGLLPDRFYDLVDEGSLVLNKCDSFSFCADGLVLDSGKRVEADVVILATGFNPDQLLRSVFTSPWVRDIVSGASSDTMLPLYRNIVHPRIPQMAIVGYLESRATIYPYEMMSKWVAHLLDGTVRLPSVKDMEKSVGEWALWGQGARRRSGSLFLKSCIATVTTWYHDQLCRDMGYSARRKKGFVAELMEPYGPTDYADIQ

>ELECO.r07.8AG0630890|Ecoracana_v1.1/1-524

MDSSNKKRVAIIGAGPSGLAACKHALAKGFVPVIFDSGASVGGVWTRTLASTKLQTPAAAFQFSDFPWPAGTSEFPSHDQVLAYLTAYARKFGVLECVKLGSKVVGAEYSGASEKEVTAWDRWSGNGEAFGDGSGEWHLTVQHIESGSIQKYEFDFLIVCVGRYGVTKIPTFPPNKGPEVFHGKVLHAQDYSHMEDRDAAELIRGKRVVVVGSGKSGLDTAAECAEANGSKYPCTLIYRTANWMVDHKLTWGLNVSKRTTTRLAELMVHKPEEGFALSLLATLLTPLRWLLSTVTETYYKMHIPMRKNGMVPDCTISQSLLGWKLGLLPDRFYDLVDEGSLVLNKCDSFSFCADGLVLDTGKRVEADVVILATGFNPDQLLRSVFTSPWVRDIVSGASSDTMLPLYRNIVHPRIPQMAIVGYLESRATIYPYEMMSKWVAHLLDGTVRLPCVKDMEKSVGEWALWGQGARQRSGSLFLKSCIATVTTWYHDQLCRDMGYSARRKKGFVAELMEPYGPTDYADIQ

>LOC_Os06g10180|Osativa_v7.0/1-291

MVYRSPQWMVDAGLVWGVNLQNLTTSRLAEVMVHKPGEGLLLSLLATMLTPLRWLLSKLTEMYYKRHTPMQRHGMVPGYSFSQSILACRLGILPKRFYDRVDDGSIVLRRCDPSFSFCAGGLVLDVATGDHIVDADVVILATGFQADRQLRDIFVSPWFSKIVAESSDATVPLYRRCVHPRIPQMAIIVYAESAANIYPYEMMAKWVAHLLDGAFQLPSIARMEKSVVEWVCWAHDMRRCSGNYFRKSCIGTITTWDNDQLCRNMGYNPRRKKGIVAEWIEPYGAADYADI

>LOC_Os09g37690|Osativa_v7.0/1-527

MDHRTKRVAIVGAGTSGVAACKHLLARGFRPVVFDAGASVGGQWTRTLASTRLQSPHVAYRFSDFPWPDSVDWYPRHDQVVDYLAAYARRFAVDERVRFRSTVLAAEFVGDDAADGWERWNGNGEAFGDGSGAWRLTVHHDDTDTTQVYEFDFLILCIGRFSGVPNIPAFPPAGGPDVFRGRVIHSMEFSDMDDADAAALVKGKRVVVVGSGKSAFDIAAECAEANGVEQPCTMICRSPRWLLHDTNVWGKVDIGYIYFTRFAELMVRKPGAGVASNLLATFLSPLGWLISKLTEAYYKKEIPMEEHGMSPEYGLSKSLSSCLIGMLPERFYDKVKEGSVVIKRSAKSFTFRDDGLVLDDDGGGGGERVVQADLVILATGFRGDEKLRRMFASRRVRDIVAGSPETAAPLYRECVHPRVPQMAVIGYSESATNIQTCEMLTKWVARLLDGAFRLPPVRRMEASVAEWGRYMRRSAGEEHFRRSCLGGVGIWYSDELCRDMGCDPRRKKGLLAEWFQPYGAVDYADIQ

>Sobic.002G287100|Sbicolor_v5.1/1-527

MDHKAAKRVAIVGAGTSGLAACKHLLARGFRPVVFEAGASVGGLWTRTLASTRLQSPTAGYRFSDFPWPEGADTYPRHDQVVEYLAAYARRFGVHECIRFRSQVVAAEYVGEDDDACVAEQWAGNGEAFGGDGAGVWRLTVRHGDSDSDTTQVYEFDFLILCIGRFSGVPNIPAFPPGGGPDAFRGRVLHSMDLSDMDDADAAALVKGKRVAVVGSGKSAYDVAAECADANGVERPCTVICRSPRWLLHDVTVWGKLHLGYLYMNRFAELMVRKPGAGLASTLLATLLTPLAWLISKVTGAYYKKAIPMWEYGMEPEFGFAGSISSCKIGMLPEAFYDKVRDGSVVIRRSRSFAFCEDGLVLDGGAVVVPADVVILATGFRGDQKLRDMFVAPRVKAIVAGSSSDTAAVPLYRECVHPRIPQMAVVGYSESLTNIYSVEMMAKWVARFLDGAFRLPSVPRMERSAAEWGRYMRRSNGESFRGACLGAVNIWYNDQLCRDMGCHPRRKKGFLAEWFQPYGAVDYADIQ

>ELECO.r07.6AG0527230|Ecoracana_v1.1/1-533

MASSTDRNAAKRVAIVGAGTSGLAACKHLLARGFRPVVFEAGASVGGLWTRTLASTRLQSPHVAYRFSDFPWPEGVDWFPSHDQVVDYLAAYARRFGVDECVRFRSRVVAAEHVGEDDDAADGEMWAGNGEAFGIDGAGVWRLTVLHGDSDATQMYEFDFLILCIGRFSGVPNIPAFPPNGGPDAFRGQVLHSMDLSDMDDADAAALVKDKRVAVVGSGKSAYDIAAECAEANGVEHPCTMVCRSPQWIVHDTEVWGKVNLGYLFVNRFAELMIRKPEAGIVSSLLATLLTPLAWLISKVTEAYYKKAIPMREYGMEPEYGFAGSISACKTGMLPDGLAFYDRVKDGSIVFRRSRSFSFCEDGLVLDGADDHQRVVVPADLVILATGFRGDQKLRDMFVSPRVKEIVAGSSDTTVPLYRECVHPRIPQMAVIGYSESLTNIYTSEMMAKWVARFLDGAFRLPSVRRMEQIVAEWGRYMKRSSGDRFRRSCLGAVNIWYNDQLCRDMGCKQRRKKGLVAEWFQPYGPADYADIQ

>Bradi4g37130|Bdistachyon_v3.2/1-542

MDQRAKRVAIVGAGTSGLAVCKHLLARGFRPVVFEAGAAVGGLWTRTLASTRLQSPAAVYRFSDFPWPESTDAYPRSAQVAAYLAVYARRFGVLERVRFRSTVVSAEHVGDGGCEDAADGWDRWNGNGEAFGDGTGVWRLTVRHCHGEPEPEKTTTEVCEFDFLILCVGRFSGVPNVPAFPPGRGPEAFKGRVLHSMELSDMGHADAAALLKGKRVAVVGSGKSAFDIAADCADANGAERPCTMVCRNPQWLLHDTQIWGKLNIGYLYMNRFAELMVAKPGAGVISRLLATLLTPLGWLVSAVTGAYYRSAIPMREHGMEPGHGFASCVSSCLIAMLPDGFYDKVKQGSIVFRKLPTASPFTFSRDGLEFLDNSGESESTVKKIPADVVILATGYRGDQKLRDMFASPRLKAIVAGSPDAAVPLYRNCVHPRVPQMAVVGYAEGLNNIYVCEMMSKWVARLLDGAFRLPGVRRMEGSVAEWGRYYRRSGGGGEHFRRSCLGAVNVWYNDELCRDMGCDPRRKKGLLAEWFQPYGAVDYAGIQ

>HORVU.MOREX.r3.5HG0503010|Hvulgare_Morex_V3/1-538

MDHRAKRVAIVGAGTSGLAACKHLLARGFRPVVFEAGESVGGQWTRTLASTRLQSPAAVYRYSDFPWPDSAGAFPLHDQVADYLASYARRFGVDACVRFRSRVVAAEYVGAEPEGAADRWQRWNGNGEAFGDGTGAWRLTVRHGGIGEQEAETHEFDFLILCIGRFSGVPNIPAAFPGPEAFRGRVLHSMELSDMADADAAALVKGKRVAVVGAGKSAFEIAAECAEANGAGTPCTMVCRNPQWLLHRADVWGGVSIGYLYMNRFAELMVPRPGAGAASRILAALLSPLSWLISAATGAYYRKAIPMREHGMEPGHGFARSVSSCILSMLPDGFYDKVREGSVVFARSRSFGFCHDGLVLDGAGAGDKRVVPADVVVLATGFRGDEKLKDMFASPRVKGIIAGTGSPDAAVPLYRECVHPRIPQMAVVGHAEGLNNIYSSEMTAKWVARLLDGAFRLPVVRRMEESCAEWGRYYLRRSGGGGEGQRPWRPCLGAVNVWYNDELCRDMGCDPRRKRAKGQGILAEWFQPYGAVDYADIH

>LOC_Os06g10170|Osativa_v7.0/1-517

MDATKKRVAIVGAGASGLTACKHALAKGFRPVVFEAAGDGVGGVWRRTLASTRLQTPAFAYRFSDFPWPADVSGAEVFPRHDQVVEYLAAYARRHGVTECVRFGCKVLAAEYAGVPDEEAAAWERWSGNGEAFGDGSGEWLLTVQHPGSEATQIHRVDFLILCTGRFSGVAHTPTFPPNRGPEVFHGQVLHSMDYSNMGHAAADELIRGKRVAVVGSGKSAFDTVAECAAANGGRYPCAMICRSGRWMVNGGFVWGVSLGHLFCNRLAELTVHKPGEGLALALLAILLTPLRWLLSKLAETYFKMQIPMEKHGMVPEESFAGSMSGCRLGVLPDKFYDRVEEGSILIKRARSFSFCTDGLVLDDDDTGERVDADVVVLATGFRGDQKLTDMFVSATFKQQIVAAPLYRQCVHPRIPQMAVIGYTENLTSIYTFEMMAKWVAHLLAGAFRLPSVVRMEASAAEWDEHLAMRRHGEGGGGKPCLGGVSTWYNDEMCRDMGYEPRRKKGILAATLVSSEV

>orange1.1g009897m.g|Csinensis_v1.1/1-523

MGKGEGCQITLDQQLSLSCFTVFSSGPKMERKIAIIGAGVSGLLACKYTLENGFKPIVFEARSGIGGVWSQTIESTKLQTPKSFYQFSDFAWPNSVTETFPDHNKVMEYLQAYAAHFNLFPSIKFDTKVTSIDRLVPSDEDEHSWDLWGGTGKPFSSSGKWNVTVQEARNVSSATEAYQVDFVILCIGRYSDLPNTPDFPMNKGPEVFDGKVLHSMNDDLAAELINGKRVTVIGFQKSAVDVAAEVANRNGVRYPCTLLFKTVHWMVPDYFLWSTFRSLNRWTELIIHNPGEGFFSWLLALSLSPLLWLSSKVVESCLKWTFPLKKYNMIPGHSFLNQISSCMFTVLPRNFYDRVQGGGLSLMKSRSFTFCKNGLVIDGETTPLVTDIVIFATGYKSDEKLKNIFKSTYFQKQITGSSAPLYREGIHPQIPQLAILGYADSPSILRTTEMRSKCLAHFLAGNSNLPTIKEMEHNVMNWEKSMRLYAGESYRRSCVSVLLQKYSNDQLCKDMGCNSKKEKMVFG

>VIT_218s0122g01430|Vvinifera_v2.1/1-515

MEKRIAIVGAGISGLLACKYAMEKGFNPIVFEARSSIGGVWSQTIESTKLQTPISYYRFSDFAWASSVTETFPNHNQVMEYLKSYALHFNILPQIRFSSRVISIDYFTPRSEDFPSWDLWGGTGKPFSPTGKWNITVKVTRHPLMVYEVDFVILCIGKYSDLPNIPDFPINRGPDIFDGKVIHSMDYAAMGGDLASEFIKEKRVTVVGLQKSAVDVAAEVATRNGEGHPCTLLFRRVHWLVPEHFNTVSFQNLNRFSELMVHRPSEGFFLWLLAFLLSPLFWMFTKLVEIYLKWTYPLKKYNMVPEHPFLKQIVSCMSMVIPANFYDRVREGSLILKKAQSFSFCRNGLVLDGEAAVLETDIVIFATGYKSDEKLSNIFTSTFFKNCITGSSAPFYRECIHPRIPQLAILGYSESPSVLYTMEVKSMWLAHFLAGNFKLPPVKEMEDDVMKWEKCNERYAGDGYKRSCVSVLLQIHVNDQLCRDMGCNPRRKRWFLPEMFAAYGPTDYANLTWPG

>Potri.005G250600|Ptrichocarpa_v4.1/1-518

MEKKIAIIGAGISGLLACKHILEKGFSPIVFEARSGIGGVWSQTIESTKLQIPKKMYQFSDFAWPPSVTETFPDDEQVLEYIKAYAVHFNILPRIRFNCKVTCIDYVVPGNEDFPSWDLWGGTGWPFSPTGRWNVTVQDARDPTAPVEVYQLDFVILCIGKYSDLPNIPDFPLNSGPEVFNGKVLHSMDYAAMANDCAAELVTNKRVTIIGFQKSAVDIATEVADRNGVDHPCTLIFRTVHWIVPDYFIALTFKSLNRFTEFMVHKPDQGFFIWLLVILLSPLLWIFSKLVEAYLKRKQPLKKYNMVPEHGFLKQISSCMFTVLPANFYDKVEEGSLVLKKSQSFNFCKNGLVIDSEETPIATDIVIFATGYKSDEKLKNIFKSSYFQKCITESLAPFYRECIHPQIPQLAILGYADSPAFLYTTEMKSKWLAHFLAGKFKLPTIGEMEADMKKWEKCMKYYANESYKRSCISVLLQIYCNDQICKDMGFNPRRKKWLLAELFAPYCPDDYKYLCWQM

>Gohir.D09G013400|Ghirsutum_v3.1/1-521

MESDKKRIAIIGAGISGLMACKYTLEKGFNPIVFEARSGIGGVWSQTIESTKLQTPKDFYQFSDFSWPREVKETYPDHTQVLEYLHAYALHFNILPRIKFNSKVTCIDYVTSSSADLHSWDLWGGSGEPFSPWGKWRLTVRNGQNPSASAPTDQVYQVDFVILCMGRYSDLPNIPDFPLSKGPEVFNGKVLHSMDYAAMDDDVAADLIKEKRVTVIGFQKSAVDIAAEVANKNGATHPCTLLFKTAHWIVPEYLLPLVFQGLNRFTELMIHKPGEGFFIWLLAMLLSPLLWIFSMSIECYLKYMYPLKKYNMVPTHRFLKQISSCMFTVLPANFYDRIKDGSLFLKKSQSFSFCRNGLIVQGESKPLLSDIVILATGYKSEEKLKNMFSSTLFQKCIAESSAPFYRECIHPQIPQLAILGYADSPAILYSTEMRSKWLAHFLAGNFRLPTISKMEEDVMEWEKCKRRDEKESYRRSCVSTLLQIYCNDQLCKDVGCNSRRKNWFLAELFAAYGPKDYKDLS

>Gohir.A09G014800|Ghirsutum_v3.1/1-521

MESEKKRIAIIGAGISGLMACKYTLEKGFNPIVFEARSGIGGVWSQTIESTKLQTPKDFYQFSDFSWPREVKETYPDHTQVLEYLHAYALHFNIFPRVKFNSKVTCIDYVTSSAADLHSWDLWGGSGEPFSPWGKWRLTVRNGQNPSASAPTDQVYQVDFVILCIGRYSDLPNIPDFPLNKGPEVFNGKVLHSMDYAAMDDDVAADLIKEKRVTVIGFQKSAVDIAAEVANKNGATHPCTLLFKTAHWIVPEYLLPLVFQGLNRFTELMIHKPGEGFFIWLLAMLLSPLLWIFSMSIECYLKYMYPLKKYNMVPTHRFLKQISSCMFTVLPANFYDRIKDGSLFLKKSQSFSFCRNGLIVQGESKPLLSDIVILATGYKSEEKLKNMFSSTLFQKCIAVSSTPFYRECIHPQIPQLAILGYADSPAILYSTEMRSKWLAHFLAGNFTLPTISKMEENVMEWEKCKRRDEKESYGRSCVSTLLQIYCNDQLCKDMGCNSRRKNWFLAELFAAYGPKDYKDLS

>Hyque.06G091600|Hquercifolia_v1.1/1-523

MAVDSPKLEKQIAIIGAGISGLLACKHTLEKGFNPIVFEARTSIGGVWAQTVESTKLQTPKNYYQFSNFPWPSSITESFPDHNQVIDYIHSYAVRYHLLPWIKFNSRVIAIDYYYSPSDEDMLSWDLWCGSGGPFSPTGKWNVTVQDTRRPSSAPQVYQMDFVILCIGKFSDLPNIPNFPESEGPEVFDGKVIHSMDYGAMGDNEAAEFTRGKLVTVIGFQKSAVDIAAEVAKRNGIRHRCTLLFKTVHWTVPEQLLTLIFQNLNRFSEFMIHKPGEGFFSWLLVVLLSPLLCIFSKLVESYLRWIYPLKKYNMVPDHGFLKQISSCMFTVLPSYFYKRVGEGSLVLKRSQRFRFCNKGLIVDGEITPLVTDIVIFATGYRGDEKLKNIFTSTYFQRCITGSSAPLYRECIHPRIPQLAILGYSESPTILYTTEMRSKWLAHFLAGNFDLPTIRDMEDDVKKWENCTRHYACESYKRSCVSVLLQIHCNDQLCMDIGCNPRRKKWFLSELFAPYGPMDYAELT

>evm.TU.Scaffold_214.995|Carabica_v0.5/1-535

MAMDIHMPKSRAPNQENRTIAIIGAGVSGLLACKYALQKGFSPTVFEARSSIGGVWSRTCDSTKLQTPKNHYRFSDFAWSDSVKEAFPDHNQVREYIYSYALHFNILPHIKFNCKVVSVDYLCTSSEEDMSSWELWAGTGEAFSPKGKWHVSVQDLQTSADTTQVYQFDFVILCLGKFSGLANIPQFSMNKGPEVFDGEVMHSMDYAAMDNAAEFIKNKRVTIVGFQKSAIDIGTEIAKVNGAKHPCTLLFRTAHWSVPENLVEFIFRNLNRVSELTVHKPDEAFFLWLLAALLSPLLWIYSKVIENYLKWLYPLAKYDIIPEHGFLGQIRSCMLTVLPADFYEIVREGCLILKKAQTFSFCQTGVVVDDEDVPLETDIVIFATGYKSDQKFASVFKSIDFQKYLLGSSAPFYRECIHPKIPQLAIVGYSESHATIYTTEIRAKWLAHFLEGNFRLPTIREMEHNVMRWEKCMRRYTDPNFKRGCVSVMLQIYCNDEICRDMNCNPRRKKSILSELFAPYGPSDYADLSSLGREE

>evm.TU.Scaffold_214.1132|Carabica_v0.5/1-535

MAMGIHMPKSRAPNQENRTIAIIGAGVSGLLACKYALQKGFSPTVFEARSSIGGVWSRTCDSTKLQTPKNHYRFSDFAWSDSVKEAFPDHNQVREYIYSYALHFNILPHIKFNCKVVSIDYLCTSSEEHMSSWELWAGIGEAFSPKGKWHVSVQDLQTSTDTNQVYQFDFVILCLGKFSGLANIPQFAMNKGPEVFDGEVMHSMDYAAMDNAAEFIKNKRVTIVGFQKSAIDIGAEIAKVNGAKHPCTLLFRTAHWSVPENLVEFIFRNLNRVSELTVHKPDEAFFLWLLAALLSPLLWIYSKVIENYLKWLYPLAKYDIIPEHGFLGQIRSCMLTVLPADFYEIVREGCLILKKAQTFSFCQTGVVVGDEDVPLETDIVIFATGYKSDQKFASVFKSIDFQKYLLGSSAPFYRECIHPKIPQLAIVGYSESHATIYTTEIRAKWLAHFLEGNFRLPTIREMEHNVMRWEKCMRRYTDPNFKRGCVSVMLQIYCNDEICRDMNCNPRRKKSILSELFAPHGPSDYADLSSLGREE

>Kalax.0208s0009|Klaxiflora_v1.1/1-515

MSKQIAIIGAGLSGLLACKYAMEKGFSPIVFEAKNGLGGVWSQTLKSTKLQTPRDYYQFSDFPWPDSVTGDFPHSNQVKDYLHSYAFHFNILPQIKFNSRVVDIDYVTPYEDHLHSWTCWGGSGEAFSAYGKWVVTVENHSAGPLPKTETYQVDFVVLCLGNFSDLPHIPDFPVNKGPEVFHGKVLHSMDYSAMDNDKAAEFINCKRVTVVGFQKSAIDIAAEVSTQNGVKHPCTLLFRRAHWVVPELLLKLSFKYQNRFTEFMLHKPHEGFFLWLLAFLLYPLIWLYAVVFETVVKLKYPLRRYKTIPDQNFIKQVSSCKAKVMPANFYKNANEGSLIFKKAENFHFCKNGLKVEGEKEPVETDIVIFATGYRTDDKLKSIFKSTYFQKRFVDSSAPFYRECIHPRIPQLAILGYSATPATLYTCEMNARWLSHFLAGRFKLPAIKEMQTDMMEWKKSTYTFAKEDYKRACVTALLQIHNNDQLCKDMGVNPKRKSWFMPELFAPYTPADYVNI

>Kalax.0183s0055|Klaxiflora_v1.1/1-515

MSKQIAIIGAGLSGLQACKYAMEKGFSPIVFEAKNGLGGVWSQTLKSTKLQTPRDYYQFSDFPWPDSVTGDFPHSNQVRDYLHSYAFHFNILPQIKFNSQVVDIDYVTPYEDHLHSWTCWGGSGEAFSAYGKWVVTVENHSTGPSPKTETYQVDFVVLCLGNFSDLPHIPDFPVNKGPEVFRGKVLHSMDYSAMDNDKAAEFINCKRVTVVGFQKSAIDIAAEVSTQNGVKHPCTLLFRRAHWVVPELLLKLSFKYQNRFTEFMLHKPHEGFFLWLLAFLLSPLIWLYAVLFETMVKLKYPLRRYKTIPDQNFIKQVSSCKAKVMPANFYKNADEGSLIFKKAENFHFCKNGLKVEGEKEPIETDIVIFATGYRTDDKLKSIFKSTYFQKRFVDSSAPFYRECIHPRIPQLAILGYSATPATLYTCEMNARWLSHFLAGRFKLPTIKEMQADMMEWKKSTYTFAKEDYKRACVTALLQIHNNDQLCKDMRVNPKRKSWFMPELFAPYTPADYANI

>Kalax.0183s0056|Klaxiflora_v1.1/1-523

MEKNLPAEKMSKQIGIIGAGVSGLLACKYVKAKGFHPIVFEAKNGFGGIWSRTLKSTKLQTPRYYYQFSDFPWPESVAEKFPDHDQVMDYLQSYVLHFNVLPHIKFNSKVVCIEYAIPSDDELDSWSLWGDSGEAFSSHGSWSVTVENNQGPSPKIEIYQVDFVILCLGNYSDLPYIPEFPVGKGPEVFGGKVIHSMDYAAMENDQAAEFISGKRVTVVGFQKSAIDTAVEVSAKNGVGYPCTILFRRAHWVVPEFLLLLVFSKLNRFSELMVHKPDEGLFTWLVAALLSPLLWVSGKIIENYLKFMYPLHKYNTVPDQNFLKQVSSCKLMAMPADFYDRSEEGSLIFRKSESFCFCGNGIMLENEKQVLETDIVIFGTGYRSVDKLINIFKSAYFQKQISESSAPLYRECIHPRIPQLAVLGYSESPANLYATEMKARWLSHFLAGKFKLPAIEQMEDDMMAWKGSACRSAKEGYKRTCITSLLQIHSNDQLCKDMGVNPRRKKWFLRELFTPYIPSDYADI

>Eucgr.F01767|Egrandis_v2.0/1-525

MQPKQRVAIVGAGISGLVACKHLIEKGFDPIVFEAHGTVGGVWSRTIESTKLQTPKHLYQFSDFPWPASVTETFPNHEQVMEYLHSYVTHFGILPKIKFNCKVTGIDFIVELGNEEDMTDWETWSGSGGGRPFSSGGKWNVAVQDTVAGCAGVEVYQVDFVILSIGMFSDLPRTPEFPLKEGPDVFKGKVLHSMDYAMMSQGLAAALTREKRVTVVGSQKSALDIAVEVATRNGSRYPCTLLFKTAHWTLPDYLVAFIFRHLNRFAEFMVHKPGEGFFLWLLAVLLWPLRATFLKLVETYLTWIYPLKKYNMVPSHSLIEQLASRMTPTLPSNFYNAVEGGSLILKRSPSFRFCHTGLILDGGANSYHLTDVVIFATGYESEEKLKNIFSSNYFQKCLARSSYPLYRECIHPRIPQLAILGYAESPSTLYTTEMRSKWLTQFLAGQFELPTIREMEDEARKWETCTRRYVQDKYKRYRVSVMLQIYCNDQLCRDMRCNPRRKNSLLAELFASYGPSDYKNLTPES

>Nycol.F00211|Ncolorata_v1.2b/1-522

MEGKKIAIVGAGISGLIACKYVKTKGFDPIVFEMKDNVGGVWLRTLDSTLLQTPKLAFQFSDFPWPASITNSRPTHGEVLEYIQSYARHFQLLQYIEFNSKVVGMDFVKNSESQENGIISCNGTAWSGEAFSSSGSWHITVHNAQQRCLKVHIVDFVIVCTGRYGDIPKMPTFEAGKGPEVFKGKVVHAMELYSMDHNQVDDLISEKKIVVVGFQKSAFDITAKCASINGKEFPCTMICREPKWHCPQTFGWSLALAFLYGTRFSELLDHKPGESLIHGILATLLSPLRWGVSKMVEICLQWQLPMKKYDMLPKENLFHQIASCHFLSLPENFYGCLEEGSIVIKKSSGWGFCEHGLTLGSNEDAKLEADLVILATGYESNQKLKDLFDSPLHQQLLSGFESGIVPLYRECIQPQIPEMAIIGYTEGVSNLFTSEMEIRWLVSFLSGGFRLPSTKEMEEDMKKWDDYKRKHSGYKYKKSSIAAIHIWYNDMLCKDMGCIPKRKKNWLSELFSPYGPADYSNL

>g14619.t3_1|Euryale_ferox/1-534

MLAKFLIFSLSTMESKKIAIIGAGISGLIACKYVKKKGFIPIVFEMKDNVGGVWLRTLDSTLLQTPKLAFQFSDFPWPTSVTNSRPTHGEVLEYIRSYARHFQLLQYIEFNSKVIGMDFVKNSDSQENGFISRTDTAWSGEAFGSGGSWHIIVQNAQQGCTKLHVVDFVIVCTGRYGDIPKMPTFEAGKGPKVFKGKVMHAMELYSMDKKQVDDLISGKKIVVVGFQKSAFDIAAKCAGANGREFPCTMVCRGPMWHLPHTFGWSMALGFLYGTRFSELLDHKPGESLIHEILAALLSPLRWGVSKMVEICLQWQLPMKKYVMLPKENLFHQIASCHFLFLPENFYGYLQEGSIVIKKSTGWGFCEKGLTLESNEDAVLEANLVILATGYESNQKLKDLFSSPAHQQLLSGFESGIVPLYRECIHPQIPEMAIIGYTEGLSNLFTSEMEIRWLVCFLSGGFRLPSTLEMEEDMRKWDDYKREHSGYKYKKSSIAAVHIWYNDKLCEDMGCNPKRKKNWLSDLFLPYGPADYSNL

>Nycol.F00213|Ncolorata_v1.2b/1-531

MESRTVAIIGAGISGLIASKYVKEKGFTPIVFEMKDNIGGVWLRTLDSTLVQTPRLAFQFSDFPWPSAVTGDLPTHSEFLEYLQSYARHFHLLQYIQFNSKVVGMDFVENLDSRDLGVLASMGDAWSGEAFNSGGKWHISVLNANEGCSKVHIVDFVMVCTGRYGDIPNMPDFEAGKRPEVFKGKVVHAMELYSMDHEQVDDLISGKKIVVVGFQKTAFDVADKCAGANGVELPCTMICREPRWHLPPSLAWTMTLGFLFATRFSELMDHKPGESFLLALLATLLSPMRWGLCKLVEFCLRWQLPLKKYGILPEGSFFQEMASCNIFTLPQDFYDHVEAGRIIIKKSARWGFCEKGLVLESESHDQKKQELVEADLVILATGYRGNQKLKSLFEPSSPYHRYLLPGLECGVLPLYRECIHPRIPQLAIIGYTESASNLFTAEMEVRWLVCFLAGGFRLPSTKEMEDDIKRWDEYRRKHSAYIYKKPSVTVIHIWYNDMLCKDMGCNPRRKSNWLSELFLPYGPADYSNLKI

>Nycol.F00214|Ncolorata_v1.2b/1-525

MDAKKIGIVGAGISGLIACKYVREKGFIPIVFEMKDMIGGVWLRTLDYTLLQTPKIAFQFSDFPWPSSVTSDHPTHGEVLEYIQSYARQFQLLQYIQFNSQVVGVEFVKDSCSSKDGVLPSMSDDAWSGEPFSSGGTWQITVHNVLHGCSQVHTVDFVIICTGRYGDIPKWPLFVKGRGPEVFKGKVIHAVDLYSMEPKEVDNLITGKRIVVVGFLKSAIDIAAKCANINGMEFPCTLICRKPLWHFPGTLGWTIALAFLYGTRFSELLDHKPGESLFFSLLATVLSPLRWGLNKLVELCLQWQFPLKKFGILPEEGVFQQIASCHVAALPENLFTCLEEGSIVIKKSESWAFYDSGLMLENENEPVEADIVIFATGCRATQKLKNIFKSSIYQQFLSGSESSIIPLYRECIHPWIPRMAVIGHTEAVSNLFMSEMEVRWLVCLLTGGFRLPNTKEMEEDMRKWYQYKENHGGCKYNKSSISAIQIWYNDKLCEDMGCNPRRKKNWFLEFFSPYGPADYSNLKIL

>Adc00270|Adiantum_capillus-veneris/1-500

MHHGFTPTLMEAGDSLGGIWRRTCSITKLQSTSARYQFSDFPWPNGTPPHPHHQQVLDYFDSYAAHFHIKDKILFNCKVVEIKCTQVQGPSMLWGGKGSAFANINGNRENKVWDVRVERRDFQLDDKVHHEWLQFDFLVLCVGRYGDPSYPTFPLNRGPDSFSGKVMHAMEYAMLDNASAQQMLKGKRVIVVGYMKSAIDITMEATTANQGAQGHPCHLIFRTTNWITVDRNLFGKFLSLFYLTHFAELMHPKPGQGWLLSILSVCLCPLKWLSSKVIELYLLWHLPLLKYGLVPKHSFWTHASACKTFLVPDEFFPRVEEGEIVLHKTGEWSFCKDGVVLHDGTFVPADVVILGTGFDSHKKILSLLPIEYSSLLLDPSGVIPLYRGTIHPRIPHLAILGYQLKSSYMLASEMSARWLAHFLQGSFSLPSVLDMEKNAQQWIRHMSAVSPFFHQKVCNASAPIWHCDEMCRDFGWNPWRKTSWFQELFSPYSNLDYKEP

>Aspi01Gene12654|Alsophila_spinulosa/1-529

MADILPVTEKDVCIIGAGMTGLSACKHLMQQGFKPTVLEARDCVGGIWRHTCSITKLQTSRDGYQFSDFIWPEGTPPNPHNEQVVDYLESYATQFHLKERIIFNCKVIEIESRLAGDAHVTGLWGGNGSAFAGDDEDNGGKVWQVLVQRRKSILHDEIEEEWLHFDFLVVCVGRFGDSPKFPIFPSGKGPEIFSGKVLHAMQYGMLDNEAARKLINGKRVIVVGYMKSAIDISMEASDANQGVHGYPCHLIYRTTNWVYVNKKPFGKLVALLYFTRLSELLNYKPGQGWALSILSMCLSPLKWACSKVVELYLLLHLPLRKHGLVPNHSFSKHASSCKLFVLPELFFPRVEKGEIVLHKTSEWSFCQNGVILHDGTEIPADVVILGTGYNSHKKLLSMLPKDHSSLLVDPAGVIPLYRGSLHPRIPHLAILGYQLKFSHLQSSEMSARWLAYLLSGKFRLPRVVDMEKDAQGWIQHMQKISPFPEQACIGAAGIWHSDQMCRDMGWNPWRKRSLVQEFLSPYSNMDYKD

>evm.model.Chr3.4383|Isoetes_sinensis/1-498

MAMKQKSVCIVGAGASGLCACKHLLQHGFKPTVLEARDKVGGVWRNTLASTELQSPILHYQFSDYPWPAGTPEYPCAQQVRDYLHGYATNFNVMDCIQFGCKVTLISKSEELSFTIADTWGTGSAFPPNCHDPNGHWQVVVEENHGGETLKKVLHFDFVIICMGLYGDVPRLPPCSPPKDFKGKVMHSMDYSSLAAKQAKELVSQKRVIVVGFYKSALDIAAEVAVANQGTFGYPCTILYRTLRWFRPTASGPLVPFLGTRFWELCNRKPNQGLALNILSSLLFPTKWLLSKFYELYLVLKFPFGKYGMVPSHSFYEQLDGCRAATLPANFFSMLDQGLIRLRRSLKWKYSSKGILLDDGTEVEADLVILCTGYDGGAKLKSIISKQYREHILRPRYRKMINPQIPHMAFLGYDTDISYLRAIDIGAKWLAHFLSRKFNLPDDISMQEEAIIWSPHKPCVTRIWHNDQLCKDLAWNPYRKGSWFSELFCSYSNSDYHA

>evm.model.Chr3.4375|Isoetes_sinensis/1-498

MAMKQKSVCIVGAGASGLCACKHLLQHGFKPTVLEARDKVGGVWRNTLASTELQSPILHYQFSDYPWPAGTPEYPCAQQVRDYLHGYATNFNVMDCIQFGCKVTLISKSEELSFTIADTWGTGSAFPPNCHDPNGHWQVVVEENHGGETLKKVLHFDFVIICMGLYGDVPRLPPCSPPKDFKGKVMHSMDYSSLAAKQAKELVSQKRVIVVGFYKSALDIAAEVAVANQGTFGYPCTILYRTLRWFRPTASGPLVPFLGTRFWELCNRKPNQGLALNILSSLLFPTKWLLSKFYELYLVLKFPFGKYGMVPSHSFYEQLDGCRAATLPANFFSMLDQGLIRLRRSLKWKYSSKGILLDDGTEVEADLVILCTGYDGGAKLKSIISEQYREHILRPRYRKMINPQIPHMAFLGYDTDISYLRAIDIGAKWLAHFLSRKFNLPDDISMQEEAIIWSPHKPCVTRIWHNDQLCKDLAWNPYRKGSWFSELFCSYSNSDYHA

>Rn_04803.1_1|Ricciocarpos_natans/1-579

MAPSAESVPSWGGAQEEHMMGQGRRGGDIRVGIIGAGVSGLTTCKHFLEQGLNVTVFEGKSGLGGVWRSTYATTKLQTPKQGYQFSDFAWPDHVPQFPTHHQVVDYLESYASRFGFFERIHFNSKVLSLARISRTTFERLDWIEGKERGVARLRTIEEDDETRSNKSERNYRRKLEENTDDYVWEVCVRRNLSTPNGFADYVDEFYEFDYLVMCCGRFGDRPKVPAFPPLEGPEIFDGRVLHALEYSSLDANSAHEFVAGKKVVIVGFQKTSLDIAVEVAEANQGIDGPPCTMVFRRTHWMYPRHRQAFGYIPFGFFWSTRFASFWTSKPDQGLVQRVLQSLLKPLRWAVWKMIEYYLRFTLPLSRFGLVPEHSLFDDLSACVLPQVPVTLFGHLEAGRILTRKTPSMSFYTKGLRLEDGTEIEADSVILCTGYDGDAKLKSILPKMYREVLLEPGNVLTLYRGIVHPHIPNMGFIGYFEGLSSVHSSEIGAQWLAQLLRKRFTLPSVADMETERSKWLQHAKKTTPFFNRPCLAPWQIWHTDKLCRDMGQDPRRKRSWWQEMFSLYSNNDYDFSNSLL

>Rn_04803.1_2|Ricciocarpos_natans/1-579

MAPSAESVPSWGGAQEEHMMGQGRRGGDIRVGIIGAGVSGLTTCKHFLEQGLNVTVFEGKSGLGGVWRSTYATTKLQTPKQGYQFSDFAWPDHVPQFPTHHQVVDYLESYASRFGFFERIHFNSKVLSLARISRTTFERLDWIEGKERGVARLRTIEEDDETRSNKSERNYRRKLEENTDDYVWEVCVRRNLSTPNGFADYVDEFYEFDYLVMCCGRFGDRPKVPAFPPLEGPEIFDGRVLHALEYSSLDANSAHEFVAGKKVVIVGFQKTSLDIAVEVAEANQGIDGPPCTMVFRRTHWMYPRHRQAFGYIPFGFFWSTRFASFWTSKPDQGLVQRVLQSLLKPLRWAVWKMIEYYLRFTLPLSRFGLVPEHSLFDDLSACVLPQVPVTLFGHLEAGRILTRKTPSMSFYTKGLRLEDGTEIEADSVILCTGYDGDAKLKSILPKMYREVLLEPGNVLTLYRGIVHPHIPNMGFIGYFEGLSSVHSSEIGAQWLAQLLRKRFTLPSVADMETERSKWLQHAKKTTPFFNRPCLAPWQIWHTDKLCRDMGQDPRRKRSWWQEMFSLYSNNDYDFSNSLL

>Rn_04803.1_3|Ricciocarpos_natans/1-579

MAPSAESVPSWGGAQEEHMMGQGRRGGDIRVGIIGAGVSGLTTCKHFLEQGLNVTVFEGKSGLGGVWRSTYATTKLQTPKQGYQFSDFAWPDHVPQFPTHHQVVDYLESYASRFGFFERIHFNSKVLSLARISRTTFERLDWIEGKERGVARLRTIEEDDETRSNKSERNYRRKLEENTDDYVWEVCVRRNLSTPNGFADYVDEFYEFDYLVMCCGRFGDRPKVPAFPPLEGPEIFDGRVLHALEYSSLDANSAHEFVAGKKVVIVGFQKTSLDIAVEVAEANQGIDGPPCTMVFRRTHWMYPRHRQAFGYIPFGFFWSTRFASFWTSKPDQGLVQRVLQSLLKPLRWAVWKMIEYYLRFTLPLSRFGLVPEHSLFDDLSACVLPQVPVTLFGHLEAGRILTRKTPSMSFYTKGLRLEDGTEIEADSVILCTGYDGDAKLKSILPKMYREVLLEPGNVLTLYRGIVHPHIPNMGFIGYFEGLSSVHSSEIGAQWLAQLLRKRFTLPSVADMETERSKWLQHAKKTTPFFNRPCLAPWQIWHTDKLCRDMGQDPRRKRSWWQEMFSLYSNNDYDFSNSLL

>Lc_composite_07994.1_1|Lunularia_cruciata/1-374

CHEFDYLVMCCGRFGDVPRVPSFPLSQGPDVFEGQVLHALEYSALDSQGAHDFVAGKKVIVVGFQKTSLDIAVEIAEANQGMDSPPCTMVFRRTHWMYPRHRQAFGVIPFAFFWSTRLSGFFTPKPGQGLARRLLQSFFNPLRLGLWRMIEFYLRLTLPLSRFGLIPEHSLVDDLTSCVLPQVPIPLFRHLEAGRILTKKSPYLSFYSKGLRLADGTSIEADAVILCTGYNGEAKLKSMLPKMYRDVLFEPENVLTLYRGIIHPQIPNMGFIGFFEGLSSVHSSELGAAWLARLLRKKFTLPPVADMETDRWEWLQHFKTVTPFFNRPCVAPLQIWQSDKLCQDMGMKHLRKKSWWQEMFSLYGNSDYDLSQSS

>evm.TU.supercontig_82.77|Cpapaya_ASGPBv0.4/1-521

MACNKKNPLIVSKVGIIGAGVSGIAAAKQLSHHKPIVFEASDSIGGVWKSCSYYSTKLQSYRSDYEFSDFSWQNRDDTTFPSYLEILDYLKCYAYYFDVFKYVKFNSKVVELRFIGDRDLVNTSGKYNSPLSGHPIWKVAVQTHNSDDIQWYTFEFVVVCTGKYGDIPKIPKFPQNKGPEIFKGQVLHSIDYCKLDKNDASQLLKDKKVTVIGYKKSAIDLAVECAEANQGPEGQPCTMVVRTLHWTVPHYWIWGLPFFLFYSTRFSQFFHERPNQSFLKTLFCLLLSPRHIVSKFIESYLLWKLPLQKYGLKPDHPFLEDYASCQMAIMPDNFFAEADKGKIVIKRASKWWFWTGGIEFEDGSKLDTDVVILATGYDGKKKLKAILPEPFCSLLEYPSGMMPLYRGTIHPLIPNMAFVGYVESISNLHTAELHSIWLARLIDEKFKLPSIEKMLQQTSKEIEVTKKTTRFYKRHCISTFSINHSDEICQEMGWNTWRKKNWLLEAFSPYGSQDYLQVQGK

>Prupe.7G193500|Ppersica_v2.1/1-521

MAPIVSKLGIIGAGVSGLAAAKQLAHHNPIVLEASDSIGGVWRHCSYSSTKLQSHRRDYEFSDFPWPDRDNADFPSHLEILDYLDSYAQHFDVLKFVRFNSKVVEVRFVGDRETVDFSVKPVEYGGLSPGRGQPVWEVSVQTNDSEAIQWYAFEFIVVCIGKYGDIPKLPQFPQNKGPEVFEGQTLHALDYCKLDKDAASDLLKDKKVVVVGYKKSAIDLAIECAEANQGPEGKPCTMVVRTLHWTVPHYWVWGLPFFLFYSTRSSQFLHQRPNQSFLRALLCSLVMSPMRHAVSKFIESYILWKLPLEKYGLKPDHPFEEDYASCQMAIMPENFFSEADKGKILFKRSESKWWFCKDGIEFDDNTKIKADVVVFATGYDGKKKVKSILPEPFRSLLEYPSGLIPLYRGTIHPLIPNMAFVGYLESVSNLHSSELRSIWLARLLDNKFKLPSVEQMLEQTRKELEVSKKTTRFYKRHCISTFSINHSDEICEEMGWTSWRKNTWLAEAFSPYGSQDYLKKQ

>FvH4_1g09240|Fvesca_v4.0.a2/1-513

MAPVVSKVGIIGAGVSGIAAAKHLSHHNPIVLEASDSIGGVWRHCSYNSTKLQSHRRDYEFSDFPWFDRDKPEYPSHLEILDYLNSYAEHFDVLKFVRFNSKVVEVRFIGDRETTDAGFKPPAGQPAWEVAVQTSGEDTIQWYAFEFLVVCIGKYGDIPRIPEFPHNKGPQAFQGQVLHSLDYCKLDKEAASELLKDKKVVVVGYKKSALDLAVECAEANQGPKGQPCTMVVRTLHWTVPHYWVWGLPFFLFYSTRASQFLHPRPNQSFIRTITCLLALSPLRHAISKFIESYILWKLPLEKYGLKPDHSFEEDYASCQMAITPENLFSEADKGRIMFKRSQGNWWFNNDGIEFDDGSKIDADVVVLATGYDGKKKLKAILPDPFRSLLEYPSGIMPLYRGTIHPLIPNMAFVGYLESVSNLHSSELRSIWLARLIDNKFKLPSVEKMLEQTTKELEVMKDTTRFYKRFCISTFSINHSDEICEEMGWTSWRKNTWLAEAFSPYGGQDYLQKY

>Qrob_P0388020.2|Quercus_robur/1-514

MAPVSSKIGIIGAGVSGLAAAKQLAHHNPLVFEASDSIGGVWRHCSYSSTKLQSLRCDYEFSDFPWSDRDSTSFPSYTEILEYLTSYANHFDVLKFVRFNSKVVEIRFVGDREATDLTGKPGDYPTILPGHPVWEVAVQTNHSDAIEWYAFEFIVVCIGKYGDVPKIPEFPRNKGPEIFQGKVLHTIDYCKLQKEETSQLLKGKKVAVIGFKKSAIDLALECAEENQGPEGQPCTMVVRTLHWTVPHYWIWGLPFYMFYSTRSSQFLHEKPNQSFLRTLLCLLLSPMRRGASKFIESYLLWKLPLQKYGLKPDHPFEEDYASCQMAIMPENFFSEADKGKILFKRASNWWFSKEGIEFDDDTKLEADVVVLATGYDGKKKLKAILPEPFRTLLEYPSGIMPLYRGTIHPLVPNMAFVGYLESVTNLHTSELRSIWLARLVDEKFKLPSVEKMIEQTTKEMAVSKKTTRFYKRHCISTFSINHSDEICEEMGWNSWRKKNWISEAFSPYGSQDYI

>Qrob_P0388040.2|Quercus_robur/1-514

MAPVSSKIGIIGAGVSGLAAAKQLAHHNPLVFEASDSIGGVWRHCSYSSTKLQSLRCDYEFSDFPWPDRDSTSFPSYTEILEYLTSYANHFDVLKFVRFNSKVVEIRFFGDREATDLTGKPGDYPTILPGHPVWEVAVQSNHSNTIEWYAFEFLVVCIGKYGDVPKIPEFPRNKGPEIFQGQVLHTIDYCKLQKEETSQLLKGKKVAVIGFKKSAIDLALECAEENQGPEGQPCTMVVRTLHWTVPHYWIWGLPFYMFYSTRSSQFLHEKPNQSFLRTLLCLLLSPMRRGASKIIESYLLWKLPLQKYGLKPDHPFEEDYASCQMAIMPENFFSEADKGKILFKRASNWWFSKEGIEFDDDTKLEADVVVLATGYDGKKKLKAILPEPFRTLLEYPSGIMPLYRGTIHPLVPNMAFVGYLESVTNLHTSELRSIWLARLVDEKFKLPSVEKMIEQTTKEMAVSKKTTRFYKRHCISTFSINHSDEICEEMGWNSWRKKNWISEAFSPYGSQDYI

>Gohir.A05G035500|Ghirsutum_v3.1/1-526

MAENHYENPPLTISKIGIIGAGVSGIAAAKQLYHHNPVVFEASDSIGGVWRTCAYNSTKLQSAPKDYEFSDFPWPNRDDPSFPTHVEIVDYLESYAKHFDVLKFVKFNSKVMELRFVGQQGTTELGGNGGAFGSPLPGHPVWQIAVQTNGSQNLQWYAFEFVVVCIGKYGDIPKIPSFPHNKGPEMFQGEVLHSIDYCKLDKDAASQLLKGKKVAVIGYKKSAIDLAVECAEANQGPEGQPCTMVVRTLHWTVPHYSVWGLPFFLFYSTRASQFLHEKPNQSFLRTMLCYLLSPLRRGVSKFIESYLLWKLPLRKYGLKPDHPFEEDYASCQMAIMPENFFTEADKGNIVFKRSSNWWFWKEGIEFDDNTKMKADVVILATGYDGKKKLKTILPDPFRSLIEYHPSGIMPLYRGTIHPLIPNIAFVGYIESVANLHTSELRSMWLARYVDEKFKLPSAENMLEQIYKEMEVSRRITRFYKRHCISTFSINHGDDICEETGWNSWRKRSWFAEAFSPYGSQDYRLKN

>Gohir.D05G037000|Ghirsutum_v3.1/1-526

MAENHYENPPLTISKIGIIGAGVSGIAAAKQLYHHNPVVFEASDSIGGVWRTCAYNSTKLQSARKDYEFSDFPWPNRDDPSFPTHVEVVDYLESYAKHFDVLKFVKFNSKVMELRFVGQQGTTQLGGNGGAYGSPLPGHPVWQIAVQTNGSQNLQWYAFEFVVVCIGKYGDIPKIPSFPHNKGPEIFQGEVLHSIDYCKLDKDAASQLLKGKKVAVIGYKKSAIDLAVECAEANQGPEGQPCTMVVRTLHWTVPHYSVWGLPFFLFYSTRASQLLHEKPNQSFLRTMLCYLLSPLRRGVSKFIESYLLWKLPLLKYSLKPDHPFEEDYASCQMAIMPENFFTEADKGNIFFKRSSNWWFWKEGIEFDDNTKMKADVVILATGYDGKKKLKTILPDPFRSLIECHPSGIMPLYRGTIHPLIPNMAFVGYIESVANLHTSELRSMWLARYVDEKFKLPSAENMLEQIYKEMEVSRRITRFYKRHCISTFSINHGDEICEEMGWNSWRKRNWFAEAFSPYGSQDYRLKN

>VIT_211s0016g00570|Vvinifera_v2.1/1-522

MAGQQNHVISSRIGIIGGGISGIAAAKQLSSYNPVVFEATDSIGGVWKHCSYNSTKLQSLRCDYEFSDYPWPERDNSSFPSHTQILDYLHSYATYFDLLKFVRFNSKVVELRFVGDRDATDADGRPGEYGSLLAGRPVWEVAVQTNQSQDVQWYSFEFVVVCIGKYGDIPKMPAFPRNKGPEIFQGQVLHSLDYSKLDQEGVTNLLKGKRVAVVGYKKSAIDVAAECAEANQGPEGQPCTMVIRTLHWTVPHYWVWGLPFSWFYSTRFSQFFHERPDQGMLRSLFCHLLSPMRQAVSKFIESYLAWKLPLRKYGLKPDHPFEEDYASCQMAILPDNFFSEADKGNIMFKRASKWWFWNGGIQFEDDTKLEADVVVLATGFDGKKKLKDILPEPFRSVIESPSGVMPLYRSTIHPLIPNMAFVGYVESVANLHTAELRSKWLARLVDDHFKLPSVEKMIEKTNNETEVMKKTTRFYKRGCISTYSINHSDEICEEMGWTSWRKKSWLSEAFSPYNSEDYGEEK

>GWHPBAUK010121.1|Macadamia_integrifolia/1-529

MASEKNNCFKVSRVGIIGAGISGIAAAKQLSGYKPVVFESTDSIGGVWKHCSYKSTKLQTPRCDYEFSDFPWPERDNSTFPTYSEILVYLHDYATHFDVLKYVKFNSKVVEIRFTGDRETTNFGGNPGHYGNLLSGRPVWEVAVQTKHSEILQWYAFEFLVVCSGKYGDVPMMPRFPHNKGPDVFHGKVLHSLDYSKLDEEAASELLKGKKVVIVGYKKSAIDLAVECGEANQGPEGQPCTMVIRTLHWTVPSYWIWGLPFFLFYSTRSSQFLHERPNQSLLKTLLGLLVSPLRRGISKFIETYLVWKLPLIKYGLKPDHPFEEDYASCQMAILPDNFFSLADRGLISFKRTSSSSPSNKWWFYNGGLQFEDGTKLDADVVLLATGFDGKSKLKSLLPEPFRSLVEDSSGIMPLYKGTIHPLIPHMAFVGFVESVSNLHTAEMRCKWMARLVENQFKLPSVEKMLEQTTQEIEVMKRTTRFYKRHCISTFSINHSDEICEEMGWRSWRKKNWFLEAFSPYNSQDYEEEK

>GWHPBAUK035386.1|Macadamia_integrifolia/1-532

MASENYCHRRVSRIGIIGAGISGIAAAKQLSGYEPVIFEATDSIGGVWKHCSYRSTKLQTPRCDYEFSDFPWPERDNSTFPSYAEILDYLHDYANHFDVLKYVKFNSKVVEIRFIDHRETTNFGGEPGDYGSLLSGRPVWEVAVQTNHSETLQWYAFEFLAVCIGKYGDVPLMPRFPHNKGPEVFHGKVLHSLDYCKLDKEAATELVEGKKTVVIGYKKSAIDLAVECAEANQGPDGQPCTMVIRTLHWTVPSYWVWGLPFFLFYSTRSSQFLHKRPNLSSLRALLCSFLSPLRKGISKFIESYLVWKLPLVKYGLKPDHPFEEDYASCQMAILPENFFSLADQGMINFKRTSSSPSNKWWFWNGGIEFEDGTKLGADVVFLATGFDGKRKLKALLPEPFRSLVEDSSGIMPLYRGTIHPLIPHMAFVGYIESVSNLHTAEIRCKWLARLVEDRFKLPSVEKMLDQTTKEIEIMKKTTRFYKRHCISTFSINHSDEICEEMGWSSWRKKNWFLEAFSPYNSQDYEEGKEKMI

>TESI_030623-RA|Tetracentron_sinense/1-523

MANEQNPIHVSKVGIIGAGISGIAAAKQLSGHNPVVFEASDSIGGVWKHCSYKSTKLQTPRCDYEFSDYPWPERDNSSFPSHVEILEYLHAYATHFDVLKCVKFNSKVVEVRFIGDRETVPYLGREPGEYGSLLAGQPVWEVAVQTNQSQTVQWYAFEFLVVCIGKYGDLPKMPVYPHNKGPEIFQGKVLHSLDYCKLDKESANQLLKGKKVVVIGYKKSAIDLAVECAEANQGPDGEPCTMVVRTLHWTVPSYWIWGLPFFLFYSTRSSQFLHERPNQHLLRALFCLLLSPMRRAVSKFIESYLTWKLPLVKYGLKPDHPFEEDYASCQMAILPENFFSEADKGKILFKRASKWWFWNGGIEFEDNTKLEADVVVLATGFDGKKKLKALLPEPFRSLVEDYSSIMPLYRGTIHPLIPNMAFVGYLKSVSNLHSAELRCIWLARLVDNQFKLPGVEKMLEQTTKEMEVMKKTTRFYKRHCISTYSINHSDEICQEMGWNSWRKKNWLLEAFSPYNSQDYEQEK

>Qrob_P0299710.2|Quercus_robur/1-532

MASEQNQSHILLASKVGIIGAGISGIAAAKQLSGYSPVVFEATDSIGGVWKHCSFTSTKLQTPRCDYEFSDYPWPERDNSTFPSHVEILEYLHGYATHFDVLKFIKFNSRVIEIRYVGDRSTDNIQHKLSSKTSTEYGNLLKGHPVWEVAIENNQSQTVQWYAFEFLVVCIGKYGDVPKMPSFPPNKGQEVFQGKVLHSLDYSKLDRETSRALLKGKKVAIVGYKKSAIDLAVECAQANQGPDGQSCTMVIRTLHWTVPSYRIWGLPFFLFYSTRSSQFLHERPNQNLFRTLFLPVLSPMQRAISKFIESYLVWKLPLVKYGLKPDHPFVEDYASCQMAILPENFFAEADKGKIEFKKTSKWWFWNGGIEFEDNTRLEADVVLLATGYDGKKKLQAVLPEPFRSLIVDDSGIMPLYRGTIHPLIPNMAFVGYIESVSNLHTAELRCTWLARLADNVFKLPSVEKMLTQVTEEMQVMKRTTRFYKRHCISTFSINHSDEICEEMGWNAWRKKNWLSEAFSPYNSQDYIDEEEN

>Hyque.01G297700|Hquercifolia_v1.1/1-513

MAIFSKIGIIGGGISGIAAAKQLSEYNPVVFEATESLGGVWRHSSYKSTKLQTPRCDYEFSDYPWPQRDNSSFPTHLEILDYLYSYANYFDLLKLVRFNSKVVQIRFVGDRETNDLGEYGTLLTGGKPVWEVAVQTNHSDTLQWYSFEFLVICSGKYGDIPKIPAFPQKRGPEVFKGQVMHCLDYSKLDNEAATQLLKGKKVVVIGYKKSAIDLAVECAEANQGEEGQACTMVVRTLHWTVPHYWIWGMPFYLFYSTRFSQFLHERPNQGFIRTLLCYLLSPARRAVSKMIESYLVWKLPLHKYGLKPEHPFEEDYASCQMAILPENFFSEAEKGNIVFKRASKWWFWEGGVQFDDNTRLEADVVVFATGYDGKKKLKSILPEPFSSFIEFPSGVMPLYRGTIHPLIPNMAFVGYIESVSNLQTAELRCKWLARLVNDHFKLPSVEKMLEQTTTEMDIMKRTTRFYKRHCISTFSINHSDEICEEMGWPSWRKNSWFAEAFAPYTSQDYVHQH

>Rhdel05G0003200.1|Rhododendron_delavayi/1-508

MAPVFSKIGIIGGGISGLAAAKQLSHHHPVVFEATDSLGGVWKHCSFKSTKLQTPRCDYEFSDYPWPERDNSSFPSYVEILEYLYSYATHFDLLRFVKFNSKVVEIRFVGDRKTAYYGSLLPGQPMWEVAVQSEEGVQWYAFEFLVICTGKYGDVPKLPEFPHDKGPEIFRGKVLHTLDYSKLDDEGTTQLLKGKKVAVIGYKKSAIDLATECAEANQGPEGQTCTMVIRTLHWTVPHYWIWGLPFYLFYSTRSSQFLHERPNYGFLRNSLCHLLSPARQAVSKIIESYLAWKLPLDKYGLRPDHPFEEDYASCQMAILPENFFAEADKGNIAFKRASRWWFWEGGVEFEDNTKLKADVVILATGFDGKKKLKSIIPEPFRSLIEFPSGMMPLYRGTIHPLIPSMAFVGYIESVSNLHTAELRCKWLARLVDDQFKLPSVEKMLQQTTIEMEIMKRTTRFYKRSCISTFSINHSDEICEEMGWSSWRKKNWLAEAFSPYNSQDYEEEK

>DCAR_728458|Dcarota_subsp_sativus_v3.0/1-515

MPIFSKIGIIGAGISGIAAAKQLSQFHPMVFESSDSLGGVWKHCSFRTTKLQTPRCDYEFSDFPWTKRDNSSFPSYQEVLDYLYSYATSFDLLKFVKFNSKVVEIRFVGDRETTAEVGSFIGTGKPVWEVAVQTNQSEIVEWYSFEFLVVCTGKYGDEPKVPLFPQNKGPEVFRGKVMHSMEYSKLDEEGSVELVKDKKVAVVGYKKSAIDMAVECAEANQGPEGQACTMIIRTLHWTVPHYSIWGLPFYWFYSTRFSQFLHQRPNQGLLRTILCHFASPMRQGISKFIESYLAWKLPLRKYGLKPDHPFEEDYASCQMAILPESFFSEADKGKIMFKRASRWWFWEGGIEFEDNTRLEADVVLLSTGFDGKKKVRSILPDPFRSLLESPSGAMPLYRGTIHPFIPNMAFVGYVESVSNLHTAEIRCIWLARLLGGQFKLPSIEKMLEQTTTEMEIMRNTTRFYKRNCISTFSINHSDEICEEMGWSSWRKKNWFSEAFSAYNSRDYEQHKEDKA

>DCAR_313469|Dcarota_subsp_sativus_v3.0/1-518

MAIFSKIGIIGAGISGIAAAKQLAEHNPVVFEATDSLGGVWKHCSFRSTKLQTPRCDYEFSDFPWTERNNTSFPTYQEILDYLYSYANNFDVLKFVKFNSKVVEIRFVGDRETADYGGKMGKYGRLLSTGKPFWEVAVQTSDSDTVQMYAFEFLVICTGKYGDIPKIPAFPQNKGPEVFKGKVMHSIEYSKLDEEASVDLLKDKKVVVVGYKKSAIDLAVECAEANQGPEGKPCTMIVRTLHWTVPHYSIWGLPFYMFYSTRFSQFFHERPNQSLLKTLLSHLLSPVRRAISKTIESYLAWKLPLDKYGLRPDHPFEEDYASCQMAILPENFFSEADKGKILFERASRWWFWEGGVEFEDGTRLEADVVFLATGFDGRKKLKAILPDPFRGLMEFPSGMMPLYRGTIHPFIPNMAFVGYVESVSNLHTAEIRCKWLARLVGGHFKLPSVEKMLEQVTTENEIMKKTTRFYKRNCISTFSINHSDEICEEMGWSSWRKKNWLAEAFSPYNSQDYEEEHK

>Soltu.DM.07G013270|Stuberosum_v6.1/1-513

MAIIRSKIGIIGGGISGIAAAKQLAKYDPIVFEATDCIGGVWKHCSYRSTKLQTPRCDYEFSDFPWTQRDNTNFPTYEEILDYLYSYAKHFDVLKFFNFNSKVVEIKFVGDREMNYFGEYGSLLTGNPVWEVAVRNNQSQTIQWYAFDFVVMCTGKYGDIPNIPNFPEKKGPQVFNGQVLHTLDYSKLDQEASTKLMKGKKVVVVGYKKSAIDLAVECAEANQGPEGQPCTMVVRTLHWTVPHYSIWGLPFYLFYSTRSSQFLHERPNQGLFRTFLCHMLSPMRRAASKLIESYLEWKLPLEKYGLKPEHPFEEDYASCQMAILPENFFTEADKGKIMFKRTSKWWFWEGGVEFEDNTKLEADVVILATGFDGKKKIKAILPDPFRSLVELPSGMIPLYRGTIHPLIPNMAFVGYLESVSNLHSAEIRCIWLSRLVDDLFKLPSVEKMLEQITQEMEIMKRTTRFYKRNCISTFSINHSDEICQEMGWRFWRKTNWLAEAFTPYNSQDYAEEK

>Solyc07G001530|Slycopersicum_ITAG5.0/1-513

MAIIRSKIGIIGGGISGIAAAKQLAKYDPMVFEATECVGGVWKHCSYRSTKLQTPRCDYEFSDFPWTQRDNTNFPTYEEILDYLYSYAKHFDVLKFFNFNSKVVEIKFVGDREMNYFGEYGSLLTGNPVWEVAVRNNQSQTLQWYAFDFVVMCTGKYGDIPNIPNFPPKKGPKEFNGQVLHTLDYSKLDQEASTKLMKGKKVVVVGYKKSAIDLAVECAEANQGPEGQPCTMVVRTLHWTVPHYSIWGLPFYLFYSTRSSQFLHERPNQGLFRTLLCHMLSPMRKAASKLIESYLEWKLPLEKYGLKPEHPFEEDYASCQMAILPENFFTEADKGKIMFKRTSKWWFWEGGVEFEDNTKLEADVVILATGFDGKKKIKAILPDPFRSLVEFPSGMIPLYRGTIHPLIPNMAFVGYLESVSNLHSAEIRCIWLSRLVDDLFKLPSVEKMLEQITQEMEIMKRTTRFYKRNCISTFSINHSDEICQEMGWQVWRKTNWLAEAFSPYNSQDYAEEK

>Prupe.1G401400|Ppersica_v2.1/1-532

MAAADNQTRLIHSVSKVGIIGAGISGIAAAKQLSGHSPVVFEATDSIGGVWKHCSYNCTKLQTPRCDFQFSDYPWAEGDNSSFPSHVEVLDYLYGYATHFDVLKYVKFESRVVEIRYVGNDQATQLPVSLKSGQYGSLLTGHPVWEVAVQTSGDPNTIQWYAFEFIVICIGKYGDIPRMPSFPRNKGQEVFQGKVLHSMDYSKLDNEAARELLKGKKVAIIGYKKSGIDLAMECAEANQGPDGQACTMVIRNLHWTVPSYWIWGLPFFLFYSTRSSQFLHERPNQSLFRALLCLLSSPMRMAISKFIESYLVWKLPLVKYGLKPDHPFVEDYASCQMAILPESFFAEADKGKILFKRSPKWWFWNGGIEFEDHTKLEVDVVLLATGYEGKKKLQSVLPEPFRSLVVDSSGTMPLYRGTIHPLIPNMAFVGFIESVSNLHTAELRSKWLARLVENEFKLPTVQEMLDQISREMEVMKKTTRFYKRHCISTFSINHSDEICEEMGWKYWRKDNWFSEAFSPYNSRDYCEEENDN

>FvH4_2g37000|Fvesca_v4.0.a2/1-536

MAAQHLNQFQFHSVSKIGIIGAGISGIAAAKQLSGHSPVVFEATDSIGGVWKHCSYASTKLQTPRCDFEFSDYPWSERDNANFPSHVEVLEYLHGYATHFDLLKFVKFGSKVVEIRYVGDDHSTTTSTHSSENYTSGEYGNLLAGHPVWVVGVQSSNNPDSTIEWYGFEFIVVCIGKYGDIPRIPTFPRNKGQEVFKGKVMHSMDYSKLDQEAARELVKGKKVAVIGYKKSAIDLALECAEANQGPDGQPCTMVVRTLHWTVPSYWIWGLPFFLFYSTRSSQFVHERPNQSFLKALFCLFSSPMRKVVSKFIESYLLWKLPLVKYGLKPEHPFLEDYASCQMAILPDNFFPEADKGNLKFKRASKWWFYDGGVEFEDNTRLEADVVVLATGYDGKKKLQSVLPEPFRSLMLDSSGTMPLYRGTIHPLIPNMAFVGFIESVSNLHTAELRCKWLSRVVDNKFKLPSVKEMLDQISREMAVMKRTTRFYKRHCISTFSINHSDEICEEMGWKSWRKSNWLSEAFSPYNSRDYNEEKED

>orange1.1g041145m.g|Csinensis_v1.1/1-522

MASAQNHVQYSSKIGIIGAGISGIATAKQLRHYDPLVFEATNSIGGVWKHCSFNSTKLQTPRCDFEFSDYPWPERDDASFPSHVELLDYLHGYAVHFDVLKYIKFNSKVVEIRHLGDRDTARVSDTAGEYGSLLKGHPVWEVAVETNQAIQWYGFELLVMCIGKFGDIPRMPAFPANKGEEIFGGKVLHSMDYSKLDKEAATELLEGKKVAIIGYRKSAIDLAVECAEANQGPNGQPCTMVIRTLHWTLPSYRIWGLPFFLFYSTRSSQFLHPRPNLGFLRTFLCSILSPMRKAISKFIESYLVWKLPLVKFGLKPDHPFEEDYASCQMAILPENFFSEAEKGNILFKRASKWWFWSGGIEFEDKSKLEADVVLLATGYDGKKKLQSILPKPFSSLLADSSGIMPLYRGTIHPLIPNMAFVGYIETVSNLQIAEIRCKWLARLADQKFKLPAVEKMLEQTTKEVEIMKQTTRFFKRHCISTFSINHSDEICEEMGWKSWRKNNWISEAFMPYSSQDYQEAKY

>Potri.001G335900|Ptrichocarpa_v4.1/1-542

MPTQAIKPSSSYISSYTKMACEKNHIQCSSKVGIIGAGISGIAAAKQLRHYSPLVFEASNSIGGVWKHCSFNSTKLQTPRCDFEFSDYPWPERDNASFPSHVQVLEYLHNYATHFDVLKYVKFNSKVVEIRYVGDRTSQLDMKPKEYGSLLNGNPAWEVAVETNQSTKVQWYAFEFLVVCIGKYGDLPRMPVFPPHQGEEAFLGKVLHALDYAKLDKDAAHKLLKGKKVAVVGYKKSALDLAVECAAANQGPEGQPCTMVIRTLHWTVPSYWIWGLPFFLFFSTRSSQFLHERPYQTLFKTLLCLLLSPMRRGISKFIESYLAWKLPLAKYGLKPDHPFVEGYASCQMAILPENFFEEADKGNILFKRASKWWFWSRGIEFEDKTRLEADVVLLATGYEGKKKLQDLLPEPFSSLVVDSSGIMPLYRGTIHPLIPNMAFVGYIESVSNLHTAELRCIWLARLADEKFKLPGIEKMLEQTNEEIQVMKRTTRFYKRHCISTFSINHSDDICEEMGWNSWRKKNWFSEAFSPYNSQDYDENKEN

>orange1.1g010421m.g|Csinensis_v1.1/1-511

MANYSKIAIIGAGVSGLAAVKQLRHHNPVVFEASDSIGGIWKSCSYNSTKLQSHRSDYEFTDFPWPNRDDPGFPSYTEILDYLESYAKHFDVFKCVRFNSKVVEVRFTGSLETTDSGGNLLPAGHPVWEVAVQTHNSNSIQRYGFEFLVVCTGKYGDVPIIPAFPNNKGPEVFEGQVLHSIDYCKLDKEAASQLLKDKKVAVVGFKKSAIDLAKECAESNQGPEGQPCTMIVRTTHWTVPHYRIWGLPFFMFYSTRFSQFLHGSPNQSLLRTLLCLLLSPLRRGVSKFIESYLLWKLPLLKYGLKPDHPFEEDYASCQMAIMPEGFFSEAEKGKIVFKRASKWWFWKGGLEFEDNTKLEADVVILCTGYDGKKKLKAFLPEPFQSLLEHPSGLLPLYRGTIHPLIPNMAFVGYIESVSNLHTAELRSIWLSRLIDDKFKLPSAEKMLEQTSKEMEVMKQSTRFYKRHCISTFSINHSDEICEEMGWNAWRKRNWLLEAFSPYGSKDYEDEK

>Potri.006G137600|Ptrichocarpa_v4.1/1-520

MARQSNLMVSKIGIIGAGVSGIAVAKQLSNYNPVVFEASDSIGGVWKSCSYSSTKLQSPRGDYEFTDFPWPNRDDPSFPSYIEILDYLKSYAEHFDVMKYVMFNTKVVEVRFVGGREPADSGEPGEYGSLLPGLPVWEVAVQSKQSDTIQWYAFEFLVVCTGKYGDIPKIPEFPLKKGPEVFKGKVMHSLDYCKLDKEAATQLHKGKKVAVVGFKKSAIDLALECAEANQGPEGQPCTMVVRTLHWTVPHYWVWGLPFFLFFSTRSSQFIHERPNQSLLRTMLCLLLSPMRHVVSKFIESYLLHKLPLQKYGLKPDHPFIEDYASCQMAIMPENFFSEADKGKIVFKKASKWWFNKEGLEFEDNTKVEADVVIFATGFDGKRKLKDILPDPFCSLLEDARSGVMPLYRGTVHPLIPNMAFLGFIESVANLHTSELRSIWLARLIDNKFKLPTIDKMLDQVSKEIEIAKRTTRFYKRHCISTFSINHSDEICEEMGWSSWRKNNMLSEAFSPYGSRDYELK

>Glyma.15G181900|Gmax_Wm82.a6.v1/1-527

MATTLDNPPISVSKIAIIGAGVSGIAASKQLSHHNPLVFEASDSIGGVWRHCSYNSTKLQSHRRDYEFSDFPWPQRESSEFPTHLEILDYLHSYAEHFDVLKNIRFNSKVVEIRYVGDQEDLSSSFGGLPSDHRTPLPGHPVWEIGVQKNNQSDSIQWYAFEFVVVCIGKYGDIPKIPEFACNKGPDVFKGRVMHTLDYCKLDQEAATKLLEGKKVVVVGFKKSGIDLAMECAKANQGPQGQSCTMVVRTPHWIVPHYRIWGLPFSMFYSTRSSQFLHERPNQGVLKALLCLLLSPVRSGISKFIESYLLRKLPLEKYELKPEHPFEEDYASCQMAIVPENFFSEADKGKIVFKRASNWSFWSGGIEFEDNSKLEADVVVFATGFDGKKKLKSILPRPFCSLLDYPSGLMPLYRGTIHPLIPNMAFVGYIESVSNLATSEIRSMWLSGLLDKKFELPSVEKMLSQTLHEMDVMKRSTRFYKRHCNSTHSINHNDEMCEDMGWSSWRKKNWITEGFSPYSSEDYRKKD

>Glyma.15G181901|Gmax_Wm82.a6.v1/1-527

MATTLDNPPISVSKIAIIGAGVSGIAASKQLSHHNPLVFEASDSIGGVWRHCSYNSTKLQSHRRDYEFSDFPWPQRESSEFPTHLEILDYLHSYAEHFDVLKNIRFNSKVVEIRYVGDQEDLSSSFGGLPSDHRTPLPGHPVWEIGVQKNNQSDSIQWYAFEFVVVCIGKYGDIPKIPEFACNKGPDVFKGRVMHTLDYCKLDQEAATKLLEGKKVVVVGFKKSGIDLAMECAKANQGPQGQSCTMVVRTPHWIVPHYRIWGLPFSMFYSTRSSQFLHERPNQGVLKALLCLLLSPVRSGISKFIESYLLRKLPLEKYELKPEHPFEEDYASCQMAIVPENFFSEADKGKIVFKRASNWSFWSGGIEFEDNSKLEADVVVFATGFDGKKKLKSILPRPFCSLLDYPSGLMPLYRGTIHPLIPNMAFVGYIESVSNLATSEIRSMWLSGLLDKKFELPSVEKMLSQTLHEMDVMKRSTRFYKRHCNSTHSINHNDEMCEDMGWSSWRKKNWITEGFSPYSSEDYRKKD

>Glyma.13G113100|Gmax_Wm82.a6.v1/1-517

MANRAENHPILVSKIGILGAGVSGIAAAKQLSHHNPIVFEASDSIGGVWSHCSYNSTKLQSHRRDYEFTDFPWPQRDNPDFPTHLEILNYLHSYAKHFDVLKNIRFNSKVVEIRYTGNREVTCSGSLLPGLPVWEVAVQTNHSDTIQWYGFELVVVCVGKYGDIARIPTFPQKKGAEVFKGKVMHTLDYCKLDQEAATQLLKGKKVVVVGFKKSGLDLAMECAQANQGPEGQPCTMVVRTLHWTVPHYWIWGLPFFLFFSTRSSQFIHERPNQGLLRTLLCLMCSPLRRGISKFIESYLLWKLPLEKYGLKPEHPFVEDYASCQMAIMPENFFSEAEKGQIVFKKASKWWFWNGGIEFEDNTKLNADVVVLATGFDGKKKLKTILPEPFSSLLEYPSGIMPLYRGTIHPLIPNMAFVGFVESVSNLHSSELRSMWLSGLVDDKFKLPSVESMLSQTHKEIEVMKRSTRFYKRHCISTYSINHSDEICKDLGWSSWRKKNWISEVFGPYSIEEYAKED

>Glyma.17G046600|Gmax_Wm82.a6.v1/1-517

MANRAKNHPILVSKIGIIGAGVSGIAAAKQLSHHNPIVFEASDSIGGVWSHCSYNSTKLQSHRRDYEFTDFPWPQRDNPDFPTYLEILNYLHSYAEHFDVLKNIRFNSKVVEIRYTGNREVTGFGSLLPGLPMWEVAVQTNHLDTIQWYGFELVVVCVGKYGDIARIPTFPQKKGAEVFKGKVMHTLDYCKLDQEAATQLLKGKKVVVVGFKKSGLDLAMECAEANQGPEGQPCTMVVRTLHWTVPHYWIWGLPFFLFFSTRSSQFIHERPNQGLLRTLLCLMCSPLRRGISKFIESYLLWKLPLDKYGLKPEHPFVEDYASCQMAIMPENFFSEAEKGKIVFKKASKWWFWNGGIELEDNTKLNADVVVLATGFDGKKKLKTILPEPFCSLLEYPSGIMPLYRGTIHPLIPNMAFVGFVESVSNLHSSELRSMWLSGLVDEKFKLPSIETMLSQTLKEIEVMKRSTRFYKRHCISTYSINHSDEICKDLGWSSWRKKDWISEVFGPYNIEEYAKED

>KAI5415326.1|Pisum_sativum/1-531

MSMINENNENNHPKIVSKIGIIGGGVSGIAIAKQLSHFNPIVFEASDSIGGVWKHCTYNSTKLQSHRRDYEFTDFPWPNRDNNDFPTHVQVLDYLHSYAKYFDVVKNIRFNSKVVELKFVGNPNSRFGGIGLTCDLEYGSLLSGQSMWDVAVQINNSDIIQWYSFEFVVICIGKYGDIPKIPMFPLKRGPEIFNGKVLHTLDYCNLHQQAANQLLSNKKVVVVGFKKSALDLAMECAQANQGVEGKPCTMVIRTLHWTVPHYWIWGLPFFLFYSTRSSQLLHQRPNQGLLRALLCFMLSPMRLGISKFIESYLLWKAPLEKYGLKPEHPFEEDYASCQMAIMPENFFSEVEKGKILFKKASKWWFDNEGIEFEDGTKVEADVVILATGFEGKKKLKAILPEPFCSLLEYPSGLMPLYRGTIHPLIPNMAFVGYVESVSNLHSSELRSMWLSGLVDEKFKLPKVERMLSQTLEEMEVMKKSTRFYKRHCISTYSINHSDEICNDLGWSSWRKKNWISEAFGPYNSQDYKKEN

>Gohir.D06G080600|Ghirsutum_v3.1/1-528

MACEQISYRKSTGVGIIGAGISGIAAAKQLSHHNPIVFEATDSIGGVWKHCSFASTKLQTPPCDFEFSDYPWPQRDNSTFPSYLDILHYLHAYATHFDVLKFVRFNSRVVEIRYIGGGDGRETTNLDVAAGPGEFGPLLSGHPVWEVAVETHPPKTIERYGFELLVVCIGKYGDIPKMPVFPPNKGQEVYGGKVMHTLDYSKLDKEAARELLKGKKVAVVGYKKSAIDLATECAQANQGADGQPCTMVIRTLHWTVPAYWIWGLPFFLFYSTRSSQFLHQRPNQGLLRNLLFPLLSPVRKAVSKFIESYLVWKLPLVKYGLKPDHPFEEDYASCQMAILPENFFSEADNGKIMFKKTPRWWFWNGGLEFEDNTKLEADVVLLATGFEGKKKIQDLLPQPFSSLIVDSSGIMPLYRGTIHPLIPNMAFVGYIESVSNLQTAELRCKWLSRLADGLFKLPSIKKMLDQTSEEIEIMKKTTRFYKRHCISTFSINHSDEICEEMGWKCWRKNNWVLEAFAPYSSQDYGEED

>VIT_204s0008g02260|Vvinifera_v2.1/1-523

MAGDQNLHHVSKIGIIGAGISGIAAAKQLSGHSPVVFEATDSIGGVWKHCTYTSTKLQTPRCDFEFTDYPWPQRDNSSFPSHIEILEYLHNYASHFDVLKYVKLNSKVVEMRYIGDESESYFDQLETEPREYGRLLSGRPVWEVAVQTNQSPTLQWYAFECLVMCLGKYGDIPKMPSFPPNKGREIFQGKVLHSLDYSKLDEAAAHRLLKGKKVVVIGYKKSAIDLAMEAAEANQDPDGQPCTMVIRTLHWTVPSYWIWGLPFFLFYSTRFSQFLHERPNQSFLRTLLCFLLSPMRKAISKFIESYLVWKLPLVKYGLKPDHPFEEDYASCQMAILPENFFAEADNGRILFKRSSKWWFWSGGIELEDNTKLEADVVALATGFDGNKKLKALLPQPFCRLIQHSTGNLPLYRGTIHPLIPNMAFVGYVESVSNLHTSELRSKWLAQLVDEQFKLPTIEKMLEKTKKEMQVMKRSTRFYKRNCISTFSINHSDELCEDMGWKSWRKNTWFSEAFGPYTCQDYQY

>CKAN_02518500|Ckanehirae_v3/1-521

MASYKNNTTESKVAIIGAGISGIAAAKQLSHLKPVVFEASGSIGGVWKHCSYRSTRLQTPRCDYEFSDYLWPERDNSTFPTSTEILEYLHGYATHFDVLKCVQFNSKVVEMRFIGEPDASCPGGTWGADGRPLAGRPVWEVAIKTNGSDTVQWYAFEFVVMCIGKYGDIPKMPVFPVEKGPEVFTGKVMHSLDYCKLNKEEASRLMKGKKVVVIGYKKSAIDLAVECAEANQGEDGQPCTMVIRTLHWTVPSYWIWGLPFFLFYSTRSSQFLHERPNQSFMRRLFCLLASPMKTAVSRFIESYLAWKLPLKKYGLLPDHPFDEDYASCQMAILPENFFSEADEGRILFKKSPKWCFWEGGIELEDKTKLEADVVFLCTGFDGKSKLKSILPEPFRDYIENSSGVMPLYRGTIHPMVPHMAFVGYLESVSNLHSAELRCKWLARLIDGHFTLPTVDRILQQTNEEVEVMKRTTRFYKRHCISTFSINHSDEICEEMGWSAWRKTNWLLEAFSAYNNQDYKEN

>CKAN_02030000|Ckanehirae_v3/1-519

MATFPIHPGESKVAIIGAGISGLAAAKQLSHYNPVVFEATSSIGGVWEHCSYQSTRLQTPRCDYEFSDYHWPERDNPTFPTYAEILEYLDGYVVKFNLLNFIRFNTMVVEVRFIGEPEFGSDGKWGTCGQPLAGRPVWEVAVRTDGSDTVEWYEFEFVVMCIGKYGDVPKLPDFPSNEGPEIFNGKVMHSLDYCKLNKEETTQLMKGKKVAVIGYKKSAIDLAVECAQANHGPDGQPCTMVIRTLHWTVPSYWIWGLPFFMLFSTRSSQFLYEPNQSFMRTISCLLSSPMKKPVSKFIESYLAWKLPLEKYGLKPDHPFVEDYAACQMTILPENFFSEADEGRIEFKRSSKWWFWEGGIELEDKTKLEADVVFLCTGFDGKAKLESILPVPFCNLIENSSGIMPLYRGTIHPLIPHMAFVGYLESVSNLHSAELTCKWLARLIDGRFTLPTVEKMIERTNEEEEVMKRSTRFYKSLCISTFSINHGDDFCEEMGWKSWRKNNWFSESFSAYNYQDNKED

>CKAN_02029900|Ckanehirae_v3/1-520

MATFPNHPSESKVAIIGAGISGLAAAKQLAHYNPVVFEASGSIGGVWKHSSYLSTRLQTPRCDYEFSDYLWPERDNSTFPTYAEILEYLDGYVEKFNLLNFIRFSTKVVEVRFIGEPELGSDGKWGTSGQPLAGRPVWEVAVITDGSDNVQRYEFEFVVMCIGKYGDVPKLPDFPSNKGPEVFNGKVMHSLEYCKLNKEEATQLMKGKKVVVIGYKKSAIDLAVECAEANQGPDGQPCTMVIRTLHWTVPSYWIWGLPFFMFFSTRSSQFLHERPNQSFMRNLCCVLSSPMKKAVSKFIESYLAWKLPLGKYGLKPDHPFVEDYASCQMAILPENFFSEADEGRIEFKRSSKWWFWEGGIELEDNTKLEADVVFLCTGYDGKAKLKTILPGPFCDLLENSAGIIPLYRGTIHPLIPHMAFGGYLESVSNLHSAELRSKWLARLIDGHFTLPTVEKMIEQTNEEVEVMKRTTRFYKRHCISTFSINDSDEICKEMGWKSWRKNNWFSEAFSAYNNQDYKED

>Litul.09G113600|Ltulipifera_YP108A_v1.1/1-522

MSTMQNRRLDSRVAIIGAGISGIAAAKQLAAHKPVVFEASDSIGGVWKHCSYQSTKLQTPRRDYEFSDYPWPDRDNSTFPTNIEILEYLCGYATQFDLMKYVQFNSKVVEVRFIGEPESAGSDGRWGLNGRPLAGQPVWEVAVRTNGSETVQWYAFEFVVVCIGKYGDLPKMPVFPHNKGPEAFRGTVMHSLDYCKLDKESANQLVKGKKAAVIGYKKSAIDLALECAEANQGEGGQPCTMVVRSLHWIVPSYSIWGLPFSLLYSTRSSQFLHQRPNQSFLRDLFCLLSSPMRRAVSRFVESYLAWKLPLKKYGLLPDHPFEEDYASCQMAILPENFFPEADKGRILFKRASKWWFWEGGIEFEDNTKLEADVVFLATGFDGKRKLKSILPEPFSDLMENSSGIMPLYRGTIHPLIPHMAFVGYLESVSNLQTAELRCIWLGRLLDGQFMLPNVEKMLDQTNEEVDVMKRTTRFYKRHCISTYSINHSDEICEEMGWKSWRKNSWFSEVFSAYTSQDYKEEK

>Pn6.2904|Piper_nigrum/1-522

MAADKGYAWDNKVAIVGAGISGIAAAKHLAGFRPVVFEASDSIGGVWRHCSYLSTRLQTSWRDYEFSDYPWPDNRSVFPTYSEVLEYLESYATHFDVLKFVQFNCKVVEVQYVAELLEDDGSAGKRWSVGGQPLVGQPVWKIGVQRNGSDAIEWHCFEFVVICMGKYGDMPRLPLFHHEEGPEIFHGKVMHTLDYCKLDREACDELMRGKKVVIVGFKKSAIDLACECATANLGPEGQPCTMVVRTLHWTIPNSYRIWGVPFFLFYSTRSAQFLQRRPFPSFLQSLCYLFSSPLRKGVSKFIESYLKWKLPLERYGLVPDHPFEEDYASLQINILQDNFFAEADEGRIQFKKSLNWRFWEGGVILDDGTKLEADVVILSTGYDGELKLKSILPERYRDLIDNASGVMPLYRGAIHPQIPNMAFIGYLGNVSFLHSSDLVCKWLSRLLVNQFALPSVEKMLEQTNEEERVMRETSRFYKNHCIATFSINHDDEICEDMGWSSWRKESWLDEAFSPYNNQDYRE

>Pn11.2187|Piper_nigrum/1-522

MAADKGYAWDNKVAIVGAGISGLAAAKHLAGFRPVVFEASDSIGGVWRHCSYLSTRLQTSWRDYEFSDYPWPDNRSVFPTYSEVLEYLESYATHFDVLKFVQFNCKVVEVQYVAELLEDDGSAGKRWSVGGQPLVGQPVWKIGVRRNGSDAIEWHCFEFVVICMGKYGDMPRLPLFHHEEGPEIFHGKVMHTLDYCKLDREACDELMRGKKVVIVGFKKSAIDLACECAKANLGPEGQPCTMVVRTLHWTIPNSYRIWGVPFFLFYSTRSAQFLQRRPFPSFLQSLCYLFSSPLRKGVSKFIESYLKWKLPLERYGLVPDHPFEEDYASLQINILQDNFFAEADEGRIQFKKSLNWRFWEGGVILDDGTKLEADVVILSTGYDGELKLKSILPERYRDLIDNASGVMPLYRGAIHPQIPNMAFIGYLGNVSFLHSSDLVCKWLSRLLVNQFALPSVEKMLEQTNEEERVMRETSRFYKNHCIATFSINHDDEICEDMGWSSWRKESWLDEAFSPYNNQDYRE

>Pn11.2190|Piper_nigrum/1-522

MAADKGYAWDNKVAIVGAGISGIAAAKHLAGFRPVVFEASDSIGGVWRHCSYLSTRLQTSWRDYEFSDYPWLDNRSVFPTYSEVLEYLESYATHFDVLKFVQFNCKVVEVQYVAELLEDDGSAGKRWSVGGQPLVGQPVWKIGVQRNGSDAIEWHCFEFVVICMGKYGDMPRLPLFHHEEGPEIFHGKVMHTLDYCKLDREACDELMRGKKVVIVGFKKSAIDLACECAKANLGTEGQPCTMVVRTLHWTIPNSYRIWGVPFFLFYSTRSAQFLQRRPFPSFLQSLCYLFSSPLRKGVSKFIESYLKWKLPLERYGLVPDHPFEEDYASLQINILQDNFFAEADEGRIQFKKSLNWRFWEGGVILDDGTKLEADVVILSTGYDGELKLKSILPERYRDLIDNASGVMPLYRGAIHPQIPNMAFIGYLGNVSFLHSSDLVCKWLSRLLVNQFALPSVEKMLEQTNEEERVMRETSRFYKNHCIATFSINHDDEICEDMGWSSWRKESWLDEAFSPYNNQDYRE

>Pn6.2910|Piper_nigrum/1-522

MAAEKGYAWDNKVAIVGAGISGIAAAKHLAGFRPVVFEASDSIGGVWRHCSYRSTRLQTSWRDYEFSDYPWPDNRSVFPTYSEVLEYLESYATHFDVLKFVQFNCKVVEVQYVAELLEDDGSAGKRWSVGGQPLVGQPVWKIGVQRNGSDAIEWHCFEFVVICMGKYGDMPRLPLFRHGEGPEIFHGKVMHTLDYCKLDREACDELMRGKKVVIVGFKKSAIDLACECAKANLGTEGQPCTMVVRTLHWTIPNSYRIWGVPFFLFYSTRSAQFLQRRPFPSFLQSLCYLFSSPLRKGVSKFIESYLKLKLPLERYGLVPDHPFEEDYASLQISILQDNFFAEADEGRIQFQKSLIWRFWEGGVILDDGTKLEADVVILSTGYDGELKLKSILPERYRDLIDNASGVMPLYRGGIHPQIPNMAFIGYLGNVSFLHSSDLVCKWLSRLLVNQFALPSVEKMLEQTNEEERVMRETSRFYKKHCIATFSINHDDELCEDMGWSSWRKESWLDEAFSPYNNQDYRE

>Pn6.2912|Piper_nigrum/1-522

MAADKGYACDNKVAIVGAGISGIAAAKHLAGFRPVVFEASDSIGGVWRRCSYRSTRLQTSWRDYEFSDYPWPDNRSVFPTYSEVLEYLGSYATHFDVLKFVQFNCKVVEVQYVAELPEDDGSARKRWSVGGQPLVGQPVWKIGVQRNGSDAIEWHCFEFVVICMGKYGDMPRLPLFRHGEGPEIFHGKVMHTLDYCKLDREACDELMRGKKVVIVGFKKSAIDLACECAKANLGPEGQPCTMVVRTLHWTIPNSYRIWGVPFFLFYSTRSAQFLQRRPFPSFLQSLCYLFSSPLRKGVSKFIESYLKWKLPLERYGLVPDHPFEEDYASLQISILQDNFFAEADEGRIQFQKSLNWRFWEGGVILDNGTKLEADVVILSTGYDGELKLKSILPERYRDLIDNASGVMPLYRGAMHPQIPNMAFIGYLGNVSFLHSSDLVCKWLSRLLDNQFALPSVEKMLEQTNEEERVMRETSRFYKKHCIATFSINHDDEICEDMGWSSWRKESWLDEAFSPYNNQDYRE

>Pn6.2915|Piper_nigrum/1-523

MAADKGYAWDNKVAIVGAGISGLAAAKHLAGLRPVVFEASNSIGGVWRHCSYRSTRLQTSSRDYEFSDYPWPDNRSVFPTYSEVLEYLARYAIHFDVLKFVQFNCKVVEVQYVAELPEDDGSAGKRWSVGGRPLVGQPVWKIGVERNGSDAIEWYCFEFVVICMGKYGDMPRLPLFHHEEGPEIFHGKVMHTLDYCKLDREACDELMRGKKVVIVGFKKSAIDLACECAKANLGPEGQPCTMVVRTLHWTVPNSYRIWGVPFFLFYSTRSAQFLQRRPFPSFLQSLYCLFSSPLRKGVSKFIESYLKWKLPLERYGLVPDHPFEEDYASWQISILPDNFFAEADEGRIQFKKSLNWRFWEGGVVLDDGTKVEADVIILSTGYDGELKLKSILPERYRDLIDNASGVMPLYRGAIHPQIPNMAFIGYLGNVSFLHSSDLVCKWLSRLLYNQFALPSVEKMLEQTNEEEKVMRETSRFYKRHCIATFSINHDDEICEDMGWSSWRKESWLDEAFSPYNNQDYREK

>Pn11.2184|Piper_nigrum/1-524

MAADKGYAWDNKVAIVGAGISGLAAAKHLAGLRPVVFEASDSIGGVWRHCSYRSTRLQTSSRDYEFSDYPWPDNRSVFPTYSEVLEYLERYAAHFDVLKFVQFNCKVVEVQYVAELPEEDGSAGKRWSVGGRPLVGQPVWKIGVERNGSDAIEWHCFEFVVICMGKYGDMPRLPLFHHEEGPEIFHGKVMHTLDYCKLDREACDELMRGKKVVIVGFKKSAIDLACECAKANLGPEGQPCTMVVRTLHWTVPNSYRIWGVPFFLFYSTRSAQFLQRRPFPSFLQSLYCLFSSPLRKGVSKFIESYLKWKLPLERYGLVPDHPFEEDYASWQISILPDNFFAEADEGRIQFKKSLNWRFWEGGVVLDDGTKLEADVVILSTGYDGELKLKSILPERYRDFIDNASGVMPLYRGAIHPQIPNMAFIGYLGNVSFLHSSDLVCKWLSRLLYNQFALPSVEKMLEQTNEEEKVMSETSRFYKRHCIATFSINHDDEICEDMGWSSWRKESWLDEAFSPYNNQDYREEK

>Pn6.2914|Piper_nigrum/1-525

MAVDKGYVWDNKVAIIGAGISGIAAAKQLTGLRPVVFEASDSIGGVWRHCSYRSTRLQTPHRDYEFSDYPWPESDDESIFPTYSEILEYLESYATRFDVLKFVQFNCKVVEVQHVAELPEDDEGTGEMWGVGGRPLVGRPVWKIGVKRNGSDAIEWYCFEFLVMCIGKYGDIPRLPSFHFEEGRKIFDGKVMHTLDYCKLDRDACDKLMRGKKVVVVGYKKSAIDLACECAEANQGPEGQPCTMVIRTLHWTVPSYRIWGLPFFLFYSTRISQFLHKRPFPSFLHNLSCLLSSPIRKGVSKFIESYLKWKLPLEKYGLVPDHPFEEDYASCQMAILPENFFAEADQGRIQFKKSLKWRFWEKGIELEDGTKLEADVVILATGFNGKLKLKSILPEQYRDLIEFASGIMPLYRGAIHPLIPNMAFVGYLESVSNLHSSELVCKWLSRLLDNQFMLPSAEKMLEQTNEEVKVMKKTSRFYKRHCISTFSIKHSDEICEEMGWKSWRKESWLAEAFSPYNNQDYRELK

>Pn11.2185|Piper_nigrum/1-525

MAVDKGYVWDNKVAIIGAGISGIAAAKQLTGLRPVVFENSDSIGGVWRHCSYRSTRLQTPRRDYEFSDYPWPESDDESIFPTYSEILEYLESYATRFDVLKFVQFNCKVVEVQHVAELPEDDEGTGEMWGVGGRPLVGRPVWKIGVKRNGSDAIEWYCFEFLVMCIGKYGDIPRLPSFHLEEGRKIFDGKVMHTLDYCKLDRDACDELMRGKKVVVVGYKKSAIDLACECAEANQGPEGQPCTMVIRTLHWTVPSYRIWGLPFFLFYSTRISQFLHKRPFPSFLHNLSCLLSSPIRKGVSKFIESYLKWKLPLEKYGLVPDHPFEEDYASCQMAILPENFFAEADQGRIQFKKSLKWRFWEGGIELDDGTKLEADVVILATGFNGKLKLKSILPEQYRDLIEFASGIMPLYRGAIHPLIPNMAFVGYLESVSNLHSSELVCKWLSRLLDNQFMLPSAEKMLEQTNEEVKVMKKTTRFYKRHCISTFSIKHSDEICEEMGWKSWRKESWLAEAFSPYNNQDYRELK

>Pn6.2911|Piper_nigrum/1-525

MAVDKGYVWDNKVAIIGAGISGIAAAKQLTGLRPVVFEASDSIGGVWRHCSYRSTRLQTPRRDYEFSDYPWPESDDESIFPTYSEILEYLENYATHFDVLKFVQFNCKVVEVQHVAQLPEDDEGTGEMWGVGGRLLVGRPVWKIGVKRNGSDAIEWYCFEFLVMCIGKYGDIPRLPSFHLEEGRKIFDGKVMHTLDYCKLDRDACDKLMRGKKVVVVGYKKSAIDLACECAEANQGPEGQPCTMVIRTLHWTVPSYRIWGLPFFLFYSTRISQFLHKRPFPSFLHNLSCLLSSPIRKGVSKFIESYLKWKLPLEKYGLVPDHPFEEDYASCQMAILPENFFAEADQGRIQFKKSLKWRFWEGGIELDDGTKLEADVVILATGFNGKLKLKSILPEQYRDLIEFASGIMPLYRGAIHPLIPNMAFVGYLESVSNLHSSELVCKWLSRLLDNQFMQPSAEKMLEQTNEEVKVMKKTSRFYKRHCISTFSIKHSDEICEEMGWKSWRKESWLAEAFSPYNNQDYRELK

>Pn44.73|Piper_nigrum/1-362

MCIGKYGDIPKLPSFPLEEGPEIFRGKVMHSLDYCKLDQEASDQLMKGKKVVVVGYKKSAIDLALECAEANQGPDGQPCTMLIRTLHWVVPSYRIWGLPFFLFYSTRSSQFLHKRPYPSFLRDLFCLLSSPMRKAVSKFIESYLTWKLPLKKYGLVPDHPFEEDYASCQMAILPGSFFPQADQGRIRFKRSSKWRFWEGGIELDDGTKLEAGVVILATGFDGKLKLKSILPEPYRNLIENKSGIMPLYRGTIHPLIPNMAFVGYLESVSNLHSAELRCMWLSRLLDHHFYLPSVEKMMEQIDEEMDVMKNTTRFYKRHCISTFSINHSDEICKEMGWKSWRKGSWLAEAFSPYNNQDYREGQ

>Eucgr.C00748|Egrandis_v2.0/1-537

MSSDHEQTPVRASRIGIIGAGISGIAAAKELSSYAPIVFEATGSIGGVWKHCSFASTKLQTPRCDFEFSDYPWPERDNESFPSHVEILDYLHGYAAHFDVLKYVQYNSRVVEIRYVGEHATSSMPRRDDGLLNSSEYGSLLKGQPVWEVAVETGPSKTIQWYAFEFILMCIGKYGDVPRMPVFPRNRGREEFQGEVLHTMDYARLDEDGAKNLLRGKKVAVVGYKKSAIDLAVECAEANQGPEGQACTMVIRTLHWTVPSYWIWGLPFYLFYSTRSSQFLHEKPDQNLLWTLLCPLLSPMRRAISKFIESYLVWKLPLVKYGLKPDHPFEEDYASCQMAILPENFFSEADKGKIQFKRAPSRWWFWSRGIEFEDGSKLEADVVILATGYDGKQKLQSLLAEPFRSLVVDSSGVMPLYRGTIHPMIPNMAFVGYVESVSNLHTAELRCKWLAQLVNDKFKLPPVEAMIRQVTQEMEVMKRTTRFYKRHCISTFSINHSDELCREMGWTPWRKSNWLSEAFSPYSSQDYADQKADKKDD

>Eucgr.C03288|Egrandis_v2.0/1-518

MASADRNVQISKIGIIGAGVSGLAAAKQLARHEPIVFEATDSIGGVWKHCSYSSTKLQSLRCDYEFSDFPWPDRDNSSFPSHAEIVDYVEAYAKHFDLLKCVRFNSKVVGVRFMGDQKITDNGGSYGSLLPGEPVWEVAVQTGSDTIQLYAFEFLVMCVGKYGDIPKMPVFPKGKGPEVFQGKVMHSMDYSKLDQEATTQLLRGKKVVVVGYKKSAIDLAMECAEANQGPEGQPCTMIVRTLHWIVPHYWVWGLPFFLFYSTRFSQFLHERPSQSLLKTLLCLLFTPLRRGVSKFIESYLLWKMPLAKYGLKPEHPFEEDYASCQMAIVPENFFAEAEKGKIVFRRPSKWWFWEGGIEFDDKTRVEADVVALATGFEGKQKIKSILPEPFRSLVEFPSGVMPLYRGMIHPLIPNAAFVGYLESVSNLHSAELGCKWLSRLAAGEFKLPSVEGMLEQTAREVEVSKRTTRFYKRHCISTYSINHDDEICEEMGWSAWRKANWFFEAFSPYSSQDYEKEK

>Eucgr.C00775|Egrandis_v2.0/1-520

MDPLQNPTVMNPRIAILGAGVSGLATAKQLSHYNPIVFEASDSIGGVWKHCSYNSTKLQSLRCDYEFSDFPWPDRDNSSFPSHAQILDYLHAYATRFDLMKFIRFNSKVVEVRFVGNQKPTDNLADYGSLLPGQPVWEVAVRTHESESVQWHSFEFLVICIGKYGDIPKIPSFPHDKGPEIFHGQVLHNIDYSKLDHEAATELLKGKKVVIVGYKKSGIDLAMECAEANQGPEGQTCTMIVRTLHWTVPHYWVWGLPFFLFYSTRSSQLLHEKPNQTFLRACLCFLLSPLRWAISKFIESYLVWKLPLKKYGLKPDHPFEEDYASCQMAIMPENFFSEADKGRIAFKRPNKWWFWEGGIEFDAGNRLEANVVIFATGYDGKKKLKSIMPEPFRSLIEFPSGVMPLYRATIHPLIPNMAFLGYVESVANLHSADLQSKWLAHLLDEKFNLPSVEDMLEQTTKEIQMMKRTTRFYKRHCISVFSINHDDEICRDMGWNPLRKKNWLAEAFSPYTSQDYEKEE

>GSMUA_Achr3G01990_001|Macuminata_v1/1-521

MEKSGTPRVAIIGGGISGLAAAKELRWLEPVLFEATNSIGGVWRHCSFRSTRLQTPRPDYEFSDYQWADRNDPTFPTHSEILEYLHGYATHFDLWRFIELETKVVEIRFVGHGETKTGFTDLWGDKGRPIADGPVWEVGVVTPRSNTVQWCKFEFVVMCIGKYGDVPNMPTFPRGKGPETFLGKVLHSLDYCKLDDEAAKSLMKGKKVVIIGYKKSAIDLAVECAEANHGTDGQPCTMVIRTLHWTVPSYSISGLPFFLFFSTRLSQFLHERPNQGLFRSLACHLLSPLRRGVSRFIESSLTWKLPLEKYGLKPNHPFVEDYASCQMAILPENFFAEADEGRIAFKRSSRWCFWEGGVVLDDNTKLEADVVLLATGFDGKQKLKSVLPEPFRGLIVDSSGVMPLYRGTIHPLIPHMAFVGYVESVSNLHTSELRCKWLGRLLRGHFKLPGTGAMFRQTGAEIDVMKRTTRFYRRHCISTFSINHSDDMCEEMGWSSWRKRSWLAELFSAYNNQDYKEDESQ

>GSMUA_Achr6G11710_001|Macuminata_v1/1-526

MEKSGKPRVAIIGGGISGLAAAKELRWLEPTLFEATDSIGGVWKHCSFRSTRLQTPRPDYEFSDYEWRDRTDPSFPTHAEILEYLHGYATRFDLWRLIRLESKVVEVRFLGDGGNAGFPQLWGGKRQAMGDRPLWEVGVVSGPSGTVQWYEFEFVVMCIGKYGDVPNMPEFPRGKGPEVFQGKVMHSLDYCKLDEEAAETLMKDKKVVVIGYKKSAIDLAVECAAVNRGPGGEACTMVIRTLHWTVPSYSIWGLPFFLFFSTRFSQFLHERPNQGLLRSLACHLLSPVRRGVSKFIESYLTWKLPLEKYGLKPDHPFVEDYASCQMAILPDNFFAEADQGRILFKKSSRWCFWEGGVVLDDGTRLEADVVLLATGFDGKKKLRSVLPEPYRGLIVDSAGVMPLYRGTIHPLIPHMAFVGYIESVSNLHTSELRCKWLGGLLKGHFELPSVEEMFRQSSQETEVMKRTTRFYRRHCISTFSINHSDEMCEEMGWSSWRKRNWMSEAFGAYNNQDYKEDKSEQDDVLD

>evm.TU.AsparagusV1_03.1372|Aofficinalis_V1.1/1-511

MERQRAPKVVIIGGGISGLAAANDPVVFEATNSIGGVWKHCSYRSTRLQTPRPDYEFSDYPWKNREDPSFPTHNEIVEYLHGYATHFNLWRLIRLESKVTEVRFLGDRQKAGFTELWGEEGRAVPSQPIWEIGVVTGTLETVQWYKCDFVVMCIGKYGDIAKMPVFPYGKGPEIFQGKVMHSMDYCKLDENSASKLMTGKKVVVVGYKKSAIDLALECAEANKGPDGQPCTMVIRTLHWTVPSYSIWGLPFFLFYSTRFSQFLHPRPNQGLMRSLTCHLLTPMKRGVSKFIESYLAWKLPLDKYGLRPDHPFDEDYASCQMAILPDNFFTEADQGRIRFEKSSKWWFWEKGVVLDDGTKLEADVVFLATGFDGKKKLKSVLPEPFCGLLEDLNGVMPLYRGTIHPLIPHMAFVGYIESVSNLHSAELRSKWLARLLEEKFKLPSVEDMYGQANREIEVMKQTTRFYRRHCISTFSINHSDEICEEMGWESWRKNNWISEAFSPYNNQDYKE

>XP_020574259.1|Phalaenopsis_equestris/1-523

MTMNLNSRIAIIGGGISGLAAAKQLLPFEPVLFEATDSIGGVWKHCSYRSTRLQTPRPDYEFSDYPWNNRADPSFPTHIEIMDYLHGYATAFGLWPLIRLKSKVVEIRYLGDREKAGFTAQWGEDGRPLASQPVWELGVSADDSNTIEWCNFEFVVMCIGKYGDTPKLPVFPNGKGEDVFKGRVMHSLDYCKLDEEEATKLMKGKKVVVVGYKKSAIDLATECAQVNKGAGGQPCTMVIRTLHWTVPSYSIWGLPFFLFYSTRLSQFLHPRPNQGLLRSLLCHISTPLRKGVSKFIESYLTWKLPLNKYGLRPNHPFEEDYASCQMAILPDNFFPEAEEGRIKFEKSEKWWFWEGGVVLEDGRKLEADIVFLATGFDGKKKLRLVLPEPFSGLIEDSSGVMPLYRGSINPLIPHMAFVGYIESVSNLHTAELRCKWLARLVEGRFKLPSVEQMFRQVHQETEIMRKTTRFYRRHCISTFSINHSDEICEEMGWNSWRKNSWFAEVFSPYGNEDYKKDEGDHDE

>Aco003989|Acomosus_v3/1-494

LKPTVFEATESIGGVWRHCAYRTTRLQTPRPDYEFSDHPWADRADPTFPTSTEILDYLDGYADRFRLRDFIKLNSKVVEIRYLGSPEKDAFAAQWGGDCTAPLPGQPVWEIGVVAGESGTVQWYKFEFIVMCIGKYGDLPKMPVFPWGGGPEIFQGKVMHTLDYCKLDEEATKELMRGKKVVILGYKKSAIDLAVECAEANQGPDGEPCTMVIRTLHWTVPSYSIWGLPFFLFYSTRLSQFLHERPNQGLLRSLLCLFLAPLRRGVSKFIESYLTWKLPLEKYGLKPDHPFDEDYASCQMAILPENFFAEADRGLIRFEKSSRWCFWEKGVVLDDGTKLEADIVFLATGFDGKKKLRSVLPEPFRGLIEDSNGVMPLYRGTIHPLIPHMAFIGYIESVSNLHTSELRCKWLARLLGGRFELPTVEGMVRQTNEETEVMKRTTRFYRRHCISTFSIDHSDEICAEMGWKPWRKGNWLSEIFSPYSNQDYKADKSD

>Asa3G00011.1|Allium_sativum/1-521

MVEKKQYVPKIAIIGGGISGLATAKQLSAFNPILFEATNSVGGVWKHCSYRTTKLQTPRRDYEFSDFPWKNRDDPSFPSSKEIVEYLEDYATHFNLWRFIRLESKVVEVKYLGDRQAAAFDEFWGNGGRPIAGQPIWEIGVINDHSQVTQWYKFDFVVMCIGKYGDFRKIPSFPKGKGPEIFHGQVMHSLDYCKLEEDETKELMKGKNCVVVGYKKSAIDLAVECAEANKGKLYTCTMVIRTLHWTVPSYSIWGLPFFLFYSTRLAQFLHESPNQGYFKSLLCHLLSPMKKCVTKFIESYLAWKSPLKKYGLKPDHPFHEDYASCQMAILPENFFEEAEQGRIKFKKSNKWWFYENGVVLEDGTKLEADVVFLATGFDGKKKLKSVLPGPFSDLIEDRFGIMPLYRGTVHPLIPHMAFVGYIESVSNLHTSELRARWLSRLLEGKFKLPSVELMLNQTNRETEIMQRTTRFYRRHCISTFSINHSDEICEEMGWKPLRKNNWLSEIFCPYNNQDYKEEKVR

>Asa3G05256.1|Allium_sativum/1-523

MADQMKQYVPKVAVIGGGISGLATAKQLSAFNPVLFEATNSIGGVWKHCSYRTTKLQTPRRDFEFSDFPWKNRDDPSFPSSNEIVEYLEDYATHFSLWKFIRFDSKVVAIKYIGDRQVAGFDQFWGNGGRPIASRPVWEIGVVTGNSQLTEWYKFDFVVMCIGKYGDFRKIPSFPKGKGPEAFQGKVMHSLDYCKLEEDETKELMKGKNCVVVGYKKSAIDLAVECAEANKGVDGKACTMVIRTLHWTVPSYSIWGLPFFLFYSTRLAQFLHESPNQGYFKSLLCHLLSPMKKCVTKFIESYLAWKSPLEKYGLKPDHPFHEDYASCQMAILPENFFEEADQGRIKFKKSNKWWFYENGVVLEDGTKLEADVVFLATGFDGKKKLKSVLPGPFSDLIEDRFGIMPLYRGTVHPLIPHMAFVGYIESVSNLHTSELRARWLSRLLEGKFKLPGVEHMLNQTNRETEIMQRTTRFYRRHCISTFSINHSDEICEEMGWKPFRKNNWLTEIFCPYNNQDYKEEKVE

>Asa3G05260.1|Allium_sativum/1-522

MAEKKQYIPKVAVIGGGISGLATAKQLSAFNPALFEATNSIGGVWKHCSYRTTKLQTPRSDYEFSDFPWKNRDDPSFPSSNEIVEYLEDYATHFSLWKFIRLDSKVVAIKYIGDRQVAGFDQFWGNGGRPVASRPVWEIGVVTGNSQVTRWYKFDFVVMCIGKYGDFRKIPSFPKGKGPEAFQGKVMHSLDYCKLEEDETKELMKGKNCVVVGYKKSAIDLAVECAEANKGVDGKACTMVIRTLHWTVPSYSIWGLPFFLFYSTRLAQFLHESPNQGYFKSLLCHLLSPMKTCVTKFIESYLAWKSPLEKYGLKPNHPFHEDYASCQMAILPENFFEEADQGRVKFKKSNKWWFYENGVVLEDGTKLEADVVFLATGFDGKKKLKSVLPGPFNDLIEDRFGIMPLYRGTIHPLIPHMAFVGYIESVSNLHTSELRARWLSRLLEGKFKLPGVELMLNQTNRETEIMQRTTRFYRRHCISTFSINHSDEICEEMGWKPLRKNNWLSEIFCPYNNQDYKEEKVE

>Acora.02G187600|Aamericanus_v1.1/1-539

MASEGKLIMNRSKVGIIGAGISGLAAAKQMKQLDPVVFEATDSIGGVWKHCSFDSTRLQTPRKDYEFSDYHWKDREDPTFPTHTEIVEYLDGYATHFDLYKYIRFDSKVVEVCYKGDKDKAGFTELWGDNGKPLPGHAVWQVGVRSGDSQTIQWYEFEFLVMCIGKYGDLPRMPVYPYKKGPEVFKGKVMHSMDYCKLSKEETQNMMRGKKVVVIGYKKSAIDLAVECAEANQGPDGQPCTMVIRTLHWTVPSYSIWGLPFFLFFSTRSSQFLHERPNQGTLRSLLCNLSSPVRSGVSKFIESYLSWKLPLDKYGLRPEHPFVEDYASCQMAILPDNFFKEADKGMIKFKKSSNWWFYENGIELDDHTRLEADVVLLATGFEGKKKLRSVLPEPFRGLVEDTSGLMPLYRGTIHPLIPHMAFVGYIESVSNLHSSELRSKWLARLLHGHFNLPSVEGMFKQTSEELEVMKRTTRFYKRHCISTFSINHSDEICEEMGWTSWRKGNWLSEIFSPYNNQDYNNEEEVEEEGGGDVDYEKES

>gene_prefix12408.t1_1|Ceratophyllum_demersum/1-517

MESNKNRQGFYKVGIIGGGISGIAAAKQLSHYNPLVFEASDSIGGVWKHCSYRSTRLQTPRRDYEFSDYHWPNRNDDSFPTHFEIVEYLENYAKHFDVMKFVKFNSKVVEVKYIGSFEEKNSWFNGRAMGGSPAWEVAVKMPESEHVEWYSFEFIVVCIGKYGDIPKMPVFARNKGPEVFQGKVMHSLDYCKLDTESTKELMKGKKVVVVGYKKSAIDLAVECAEANQGDDGKPCTMIIRTLHWTVPSYWIWGLPFFLFYSTRGAQSLHERPNQSLLRDIYCLLASPLRRGISRFIESYLKWKLPLEKYGLVPDHPFEEDYASCQMAILPENFFAEADHGRILFKKTTDWCFYSNGIEVGDGTKIEADIVFLATGFDGKKKLKLMLPQPFREFIEDSSGVMPLYSGTIHPLIPHMAFVGYIESVSNLHTSELRCKWLSGLLDEKFKLPSTEDMFQQITQEIEVAKRTTRFYRRHCISTYSINHSDHLCDQMGWSSWRKKNWLAEAFSPYNNQDYKEE

>gene_prefix16024.t1_1|Ceratophyllum_demersum/1-518

MEGNKNRQGFYKVGIIGGGISGIAAAKQLSHYNPLVLEASDSIGGVWKHCSYRSTRLQTPRRDYEFSDYHWPNRNDDSFPTHFEIVEYLENYAKHFDVMKFVKFNSKVVEVKYVGGGFEEKSSWFNGRVMGGSPVWEVAVKTPESEHVEWYSFEFVVMCIGKYGDIPKMPVFPRNKGPEVFKGKVMHSLDYCKLDTESTKELMKGKKVVVVGYKKSAIDLAVECAEANQGDDGKPCTMIIRTLHWTVPSYWIWGLPFFLFYSTRSSQSLHERPNQSLLRDIYCVLASPLRRWISRFIESYLKWKLPLEKYGLVPGHPFEEDYASCQMAILPENFFAEADHGRILFKKTTDWCFYSNGIEVGDGTKIEADIVFLATGFDGKKKLKLILPQPFREFIEDSSGVMPLYSGTIHPLIPHMAFVGYIESVSNLHTSELRCKWLSGLLDENFKLPSTEHMFQQITQEIEVAKRTTRFYKRHCISTYSINHSDHLCDQMGWSSWRKKSWLAEAFSPYNNQDYKEE

>Aqcoe7G436500|Acoerulea_v3.1/1-525

MASKKNCSVVYSRIGIVGAGISGLAAAKHLSHHHPIVFEASDTIGGVWKHCSYKSTRLQTPRSDFEFSDYPWPERELSDFPTHNEVLDYLHGYATNFGLLKYVKLNSKVIEIRFTGELETGKFGQKHIDYGTLLTGEPVWEVAVKNNQSESLQWYAFEFVVICIGKYGDIPHIPAFPGHKGPEIFNGKVLHSLDYCKLNNEAAYQLLKGKKVVIVGYKKSAIDLAVECAEANKGPDGQPCTMVIRTVHWTVPSYDIWGLPFFLFYSTRLSQFIHERPNQTLWRTFICYLLSPLRKIISKIIESYLLWKLPLRKYGLVPSHPFLEDYASCQMAILPKTFFQEANEGRILFKKSSKWWFWDGGVEFDDKTKLEADVVVLATGFEGKKKLKSILPEPFSSLIADASSIIPLYRGTIHPLIPNIAFVGYVESVSNLHSSEIRCKWLTRLLDDHFKLPTVEKMLDQTTKEEDIVKRTTRFYKRHCISTFSINHSDEICEEMGWNSWRKKNWFLEAFSPYSNHDYRETLGK

>evm.TU.Scaffold_631.43|Carabica_v0.5/1-519

MAIFSKIGIVGAGISGIAAAKQLRKYDPIIFEATDSLGGVWKHCSYRSTKLQTPRCDYEFSDFPWTQRDNSSFPTYLEVLEYLHSYATHFGVVELIKFNSKVVEIRFVGNHANDPDDQVNSNGYGNLLNGQPVWEVAVQTSDSDTAEWYAFELLVICTGKYGDVPIIPQFPHNKGPEVFKGQVLHSLDYCKLDEENSVQLLKDKKVVVVGYKKSAIDLAVECAKANQGPEGQSCTIVVRTLHWTIPHYSIWGLPFYLFYSTRASQFLHERPNQGILRNILCKLLSPVRKAMSKIIESYLVWRLPLEKYGLKPDHPFVEDYGSCQMAILPEELFEEAEKGMIDFKKASKWCFWEGGVEFDDNTKLEADVVLLATGFDGKRKLRNILPEPFRSLLETEGMMPLYRGTIHPLIPNMAFVGYIESVSNLHTAEIRCKWLSRLADNYFKLPSVGQMLEQTQKEMGIMRKTTRFYKRSCISTFSINHTDEICEEMGWKSWRKNNWLAEAFSPYCSQDYQEQKHID

>evm.TU.Scaffold_671.607|Carabica_v0.5/1-519

MAIFSKIGIVGAGISGIAAAKQLRKYDPIIFEATGSLGGVWKHCSYRSTKLQTPRCDYEFSDFPWTQRDNSSFPTHLEVLEYLHSYATHFGVVELIKFSSKVVEIRFVGNHANDPDDQVNSNGYGNLLNGQPVWEVAVQTSESDTVEWYAFELLVICTGKYGDVPIIPQFPHNKGPEVFKGQVLHSLDYCKLDEENSVQLLKDKKVVVVGYKKSAIDLAVECAKANQGPDGQPCTIVVRTLHWTIPHYSIWGLPFYLFYSTRASQFLHERPNQGILRNIFCKLLSPVRNAMSKIIESYLVWRLPLEKYGLKPDHPFVEDYGSCQMAILPEELFEEAEKGMIDFKKASKWCFWEGGVEFDDNTKLEADVVLLATGFDGKRKLRNILPEPFRSLLETEGMMPLYRGTIHPLIPNMAFVGYIESVSNLHTAEIRCKWLSRLADNYFKLPSVGQMLEQTQKEMGIMRKTTRFYKRSCISTFSINHTDEICEEMGWKSWRKNNWLAEAFSPYCSQDYQEQKHID

>Pso02G24860.0|Papaver_somniferum/1-529

MAENMQKNTSTTRRVSRVGIIGAGISGIAAAKQLKRYNPIVFEGSDSLGGVWQHCSYRSTNLQTPRYDYEFSDYPWPERDNHNFPSSTEIVDYAYNYAKHFDVLKYVEFNTKVVELRYVGSRDTMTTDLLVEDDVSTLCTGKPLWEVAVKSKYSETVEWYSFEFLVVCIGKYGDVPKIPVFPKNKGQEDFNGKVLHTLDYCKLNKDETTELLQGKKVVVIGYKKSAIDLANECAEANQGPDGKPCTMVIRTLHWTVPSYSIWGLPFFLFYSTRFSQFLHERPNQSLLKDLFCLFASPMRKGVSKFIESYLEWKLPLAKYGLKPSHPFEEDYASCQMAILPENFFKQADEGRILFKKSPKWSFWSGGIELEDKTKLEADVVVLATGFDGKGKLKSLFPEPFRSLLEDSTCIMPLYRGTIHPLIPGVAFVGYTESVSNLHSSEIRCKWLSRAMDGHFKLPTIENMLEQISKEIEVSKRSTRFYKKKPCISTFSINHTDEICEEMGLKTWRKKNWLLEAFSPYNNRDYEEEK

>Pso02G69530.0|Papaver_somniferum/1-529

MAENMQKNTSTTRRVSRVGIIGAGISGIAAAKQLKRYNPIVFEGSDSLGGVWQHCSYRSTKLQTPRYDYEFSDYPWPERDNHNFPSSTEIVDYVYNYAKHFDVLKYVEFNTKVVELRYVGGRDTMTTDLLAEDDVSTLCPGKPLWEVAVKSKYSETIEWYSFEFLVVCIGKYGDVPKIPEFPKNKGQEDFNGKVLHTLDYCKLNKDETTELLQGKKVVVIGYKKSAIDLANECAEANQGPDGKPCTMVIRTLHWTVPSYSIWGLPFFLFYSTRFSQFLHERPNQSLLKDLFCLFASPMRKGVSKFIESYLEWKLPLAKYGLKPSHPFEEDYASCQMAILPENFFEQADEGRILFKKSPKWSFWSGGIELEDKTKLEADVVVLATGFDGKGKLKSLFPEPFRSLLEDSTGIMPLYRGTIHPLIPGVAFVGYTESVSNLHSSEIRCKWLSRAMDGHFKLPTIQNMREQISKEIEVSKRSTRFYKKKPCISTFSINHTDEICEEMGLKSWRKKNWFLEAFSPYNNRDYEEEK

>Kalax.0777s0007|Klaxiflora_v1.1/1-521

MALEIDHRQTQSRDAKIAIIGAGVSGIAAAKQLSTHNPVVFEASDSIGGVWRSCAYRSTKLQSPRTDYEFSDFPWHDRDNASFPSNIEILDYLESYAKHFDLIKYVRFNCKVVELRFLGQSSEKKGEYGQLLTGKPMWEVAVQTIDSTNIERHTFEFVVMCLGKYGDIPKLPEFPINRGPEVFKGQVLHSLDYSKLDHHASTQLLQGKKVAVIGYKKSAIDMAVECAEANQGADGQPCTMVIRTLHWTVPHYQVWGLPFFLFYSTRISQFLHRRPNQGRLRTAFSPLMSPMRRGVSKIIESYLRWKLPYEKHGLAPDHPFEEDYASCQMAILPDNFFEEADKGMIRFKKASKWWFTEDGIEFDDNTKLEADVVLLATGYDGKKKVKDVLPEPFRCLVDPPTGVMPLYRGTIHPLIPNMAFVGYLESVSNLHTAELRSIWLSRLADYMFKLPSVEKMLEQTAKELEVSKSSTRFYKRYCISTYSINHSDEICEEMGWKAWRKSSWLEEAFSPYTSRDYEDNN

>Kalax.0590s0012|Klaxiflora_v1.1/1-521

MAFEIDHRQTQSRDAKIAIIGAGVSGIAAAKQLSSHNPVVFEASDSIGGVWRSCAYRSTKLQSPRTDYEFSDFPWHDRDNASFPSNIEILDYLESYAKHFDLIKYVRFNCKVVELRFLGQSSEKKGEYGQLLTGKPMWEVAVQTIDSTNIERHTFEFVVMCLGKYGDIPKLPEFPINRGPEEFKGQVLHSLDYSKLDHHASTQLLQGKKVAIIGYKKSAIDMAVECAEANQGADGQPCTMVIRTLHWTVPHYQVWGLPFFLFYSTRISQFLHRRPNQGLLRTAFSPLMSPMRRGVSKIIESYLRWKLPYEKHGLAPDHPFEEDYASCQMAILPDNFFEEADKGMIRFKKASKWWFTEDGIEFDDNTKLEADVVLLATGYDGKKKVKDVLPEPFRCLVDPPTGVMPLYRGTIHPLIPNMAFVGYLESVSNLHTAELRSIWLSRLADNMFKLPSVEKMLEQTAKELEVSKSSTRFYKRYCISTYSINHSDEICEEMGWKAWRKSSWLEEAFSPYTSRDYEDNN

>GWHPCBIR011320|Santalum_yasi/1-536

MASCVENQNVSKIAIIGAGVSGIAAAKQLSQHNPIVFEATDAIGGVWRHCSYRSTRLQSLRCDYEFSDFPWPPNPPNHDSYSHDFPSHVEVLDYLHSYATHFDLLKFVQFNSKVVELRFVGDQRRVHDLNPTDENSYGSLLTGGDPVWEVAVKTTLSDTVQWYRFEFIVVCIGKYGDVPKEPIFPRNEGPEVFEGKVMHSIDYCKLNKEEATRLLQDRKVVVIGFKKSAIDLAAECAQANQGPNGKPCTMVIRTLHWTVPHYWIWGLPFSLFYSTRSSQFLHPNPNQGLLRAFLCLLLSPLRRAVSKFVESYLLWKLPLNKYKLIPDHPFEEDYASCQMAILPDDFFAEADKGKIVFRRAPSKWGFWKGGIQFEEEGEEKTRLEADLVFLATGYDGKKKLRAILPEPFCSLLEYPSGNMPLYRGTIHPLIPNMAFVGYLESVANLFTAEIRSKWLARLVDEQFKLPSIEKMLQQTTKDVEIMKRTTRFYSRHCLSTSSINHNDEICEELGWKSWRKKNWFSEAFSPYTSQDYEDEK

>Cucsa.142050|Csativus_v1.0/1-517

MAPILSKIAIVGAGVSGIAAAKQLAHHEPIVFEASDYIGGVWKHCSYDSTKLQSFRSDYEFADFPWPNRDRVDFPSHLEILEYLNSYARHFDLRRFIQFNSKVVGVRFIGNGNSGKFGPLLSGRPAWEISVMDTLSETIQLYEVEMVVMCIGKYGDIPKLPEFPSGKGPEIFAGKVMHSLDYCKLGKEETNELLKGKRVAIIGYKKSAIDLAVECAQANQGEEGKGCTMVVRGLHWTVPHYWVWGLPFFFFFSTRSSQFLHERPNQTFLKTLIGLFLSPMRHGVSKFIESYLTWKMPLEKYGLKPDHPFLEDYASCQMAIMPENFFRQADEGKIVIKRASKWWFWNGGIEFDDGSRSEFDVVIMATGFDGKKKLTDIFPQPFRTFLQYPSGMIPLYRGTINPMIPNMAFVGYLESVANLHTSELRSIWLARLVDEKFKLPETQKMVEQINKEMEVMKRSTRFYKRHCISTYSINHSDQICEEMGWNPWRKNSWFSEAFSPYGSQDYNNINNANNKIN

>KAI5445722.1|Pisum_sativum/1-523

MASQDNPPVMVSKIGIIGAGVSGLAVAKQLSNYNPVVFEATDSIGGVWRHCSYRCTKLQSQTWNYEFSDFPWPKRESSDYPTYAEILEYLHTYAVHFDLFKYVKFNSKVVEIKFIGDKEGFDFGRLHRDSGNPLSGRPMWEFSVQSNESDTIQLYHFEFVVICTGKYGDIPLMPTFPRNKGPEVFNGKVLHSIDYCKLDKEATSDLVKGKKVVVVGYKKSAIDLALECAQANQGPEGQPCTIIIRSLHWTLPHYQMWGVPFFLFYATRSAQFLHQTPNQGLLKSLLCLLLSPMRRGISKFIESYLLWKQPMEKYGLKPDHPFEEDFASCQIAITPESFFHEADKGKIIFKRASKWWFSNDGVEFDDNTKTEADVVILATGYDGKKKLQAILPEPFSSLLEYPSGMMSLYRGTVHPLIPNMAFVGYVESVSNLYTSEIRSMWLSGLLANKFKLPTADKMLSQTIKDMETMKNSTRFYKRNCITTFGINHNDEICEDLGWNTWRKKSLIKEAFTPYTAFDYKKED

>Brara.F01358|BrapaFPsc_v1.3/1-525

MTSNYDKLTSSRVAIIGAGVSGLAAAKHLAHHNPTVFEASDSVGGVWKSCSYETTKLQSTRVDYEFSDFPWPNRDETTFPSYVEIIDYLEAYAKHFDLLKFMNFGSKVIEVRYTGDGETPLMVDLGAYGNLLPGKPVWEVAVQSGDAGDIQWHAFEFVVVCTGKFGDVPRIPTFPANKGPELFKGKVMHSMDYCKLNKEEASHLLRGKKVAVIGYKKSAIDLALESALANHGEGGQACTMVVRTTHWVFPHYWIWGLPFFLFYSTRASQFLHDRPNQSFLKTLFCLIFSLLRAVVSKFIESYVTWKLPLEKYGLKPDHSFEEDYASCQMALIPKNFFEEADKGMIRFKKTSKWCFYDQGIEFEDGTTLEADVVILATGYDGKKKLKAIVPEPFRTWLEFPYGVMPLYRGTIHPLIPNMGFVGYVQSNSNLHTSELRSLWLSRLVDGKFKLPSKEKMLEQFSKEMEVMRRSSRFYKRHCISTFSIQHADDLCNDMGLNPRRKSNLFLEAFSPYGSQDYRLDQEERK

>Brara.H02378|BrapaFPsc_v1.3/1-524

MASNDKLTSSRVAIIGAGVSGLAAAKHLAHHNPIVFEASDSIGGVWNSCTYETTKLQSTRVDYEFSDFPWPNRDDTTFPSYVEILDYLESYAKHFDLLKFMKFGSKVIEVRYTGDGGTPQMADLGAYSNSFPEKPVWEVAVQNGDAGEIQWYAFEFVVVCTGKYGDVPRIPTFPEKKGPEIFKGKVMHSMDYCKLEKEEAYRLLRGKKVAVIGFKKSAIDLALESALANQGEGGQACTMVVRTTHWVVPHYWVWGLPFFLFYSTRASQFLHDRPNQSFLKTFFCLLFSLLRAVVSKFIESYVTWKLPLEKYGLKPDHSFEEDYASCQMAIMPENFFEEADKGMIRFKKTSKWCFYDQGIEFEDGTMLEADVVILATGYDGKKKLKAIVPEPFRSWLEFPCGVMPLYRGTIHPLIPNMGFVGYVQSNSNLHTSELRSLWLSRLVDGKFKLPSKEKMLDQFSKEMDVMRKSSRFYKRHCISTFSIQHADDLCNDMGLDPRRKSNLFLEAFSPYGSQDYRLNQKETN

>Brara.I04744|BrapaFPsc_v1.3/1-526

MASNHTKLTSSRVAIIGAGVSGLSAAKHLAHHNPIVFEASDSVGGVWKSCTYETTKLQSARVDYEFSDFPWPNRDDTTFPSYIEILEYLESYAKHFDLLKFMKFGSKVIEVKYTGEGDALQMVDLGGSGSLLPGKPVWEVAVQNLDARDIQWHAFEFVVVCTGKFGDVPRIPTFPAKKGPEIFKGIVMHSMDYCKLEKEEASYLLRGKKVAVIGFKKSAIDLALESAIANQGEGGQACTMVVRTTHWVFPHYWVWGLPFFLFYSTRASQFLHDRPDQSFLRTLFCFIFSLLRAVVSKFIESYVTWKLPLEKYGLKPDHSFEEDYASCQMAIIPENFFEEADKGMIRFKKTSKWCFYDEGIEFEDGTTLEADVVILATGYDGKKKLKAIVPEPFRTWLEFPCGVMPLYRGTIHPLIPNMGFVGYVQSNSNLHTSELRSMWLSLLVDGKFELPSQEQMLDQFSKEMEVMRRSSRFYKRHCISTFSIQHADDLCNDMGLNPWRKSNLFLEAFSPYGSQDYRLAQEETNI

>AT1G19250|Athaliana_Araport11/1-530

MASNYDKLTSSRVAIIGAGVSGLAAAKNLVHHNPTVFEASDSVGGVWRSCTYETTKLQSARVDYEFSDFPWPNNRDDTTFPPYLEILDYLESYAKHFDLLKFMKFGSKVIEVRFIGDGETPQMVDLGAYGNLLPGKPVWEVAVQIGDSGDIQWHAFEFVVVCTGKYGDVPRIPAFPAKKGPEMFQGKVMHSMDYCKLEKEEASTLLSGKKVAVIGFKKSAIDLALESALANQGEGGKACTMVVRTTHWGIPHYWVWGLPFFLFYSSRASQFLHDRPNQSFLRTLFCLLFSLLRAVVSKFIESYVLWKLPLEKYGLKPNHSFEEDYASCQMAIIPENFFEEADKGMIRFKKSSKWWFYEEGIVFEDGTTLEADVVILATGYDGKKKLKAIVPEPFRTWLEFPSGVMPLYRGTIHPLIPNMGFVGYVQSSSNLHTSELRSMWLSRLVDEKFRLPSKEKMLDQFLKEMEVTRNSSRFYKRHCISTFSIQHADDMCNDMGLNPWRKSNFLLEAFSPYGSQDYRLGQEEKEDMTA

>Brara.I04746|BrapaFPsc_v1.3/1-526

MASNHNTLNSSRVAIIGAGVSGLAAAKHLARHNPTVFEASDSVGGVWRNCTYGTTKLQSPRASYEFSDFLWHDRNNTAFPTYVEILDYLESYAKHFDLFKYIKFGSKVTEVRFNGEVEAPQMVGLGDYGNLLPGKPVWEVAVQTRDRNPEDIQWHAFEFVVICTGKYGDVPRIPTFPLKKGPEIFNGTVMHSLDYCKLEKEEASHLLRGKKVAVVGFKKSAIDLALESALANQGEGGQACTMVVRTPHWVIPHYWIWGLPFFLFYSTRASQFIHDRPNQSFLRTLFYLLFSLLRTVVSKFIESYVMWKLPLKKYGLKPDHPFEEDYASCQMALVPDNFFEAADKGMIRFRKTSKWCFYDKGIEFEDGTTLEADVVILATGYDGDKKLKAIIPEAFRTWLEFPCGLMPLYRATIHPLIPNMGFVGYVQSNWNVRTAELSSMWLSRLMDEKFKLPSKEKMLDQFFKEVEVMKRSSRFYTNHCFSTFIIQHADDLCNDMGLNPWRKSNFFRDVFCPYGSQDYRFDQEET

>Nycol.K00968|Ncolorata_v1.2b/1-535

MANLHDSGTGTPEISKVGIIGAGVSGIAAAKQLRNFSPIVFEATDSVGGVWKHCAFHSTRLQTPRNDYEFSDYPWTPLPDYPPPENDSATFPTHLEIVEYLENYARHFDVLKFVKLNHRVVEIKYLSDRKPTYYDGMWGDFGKPLSGDPAWEVGVRTPLSEDIQWFKFEFLVVCLGKYGDVAKMPEFPTGKGPEIFQGTVLHSLDYSKLGRQEAERLVKGKKVVVVGYKKSAIDFATECAEVNQGPDRQPCTMLIRTLHWMMLPSYSVWGISFLLLYATRFSQFLHERPSMSLLRALLCLLLSPLRKAISKFIELYLIWKLPLRKFGLVPEHAYEEDYASCQMAILPEKFFPAAEDGRICFRRSSKWCFWSGGVELEDGSRLEADVVMLATGFDGLKKLKWIFPEPFRHYIQDSSGIVPLYRGTVHPLIPHMAFVGYIESVSNLHTSEIRCRWLSSLLQGAFVLPTVDKMVYEISKQMEVMKRTTRFYKRHCISTFSINHTDEMYEDMGLSPWRKRNWLQEAFSPYSNRDYKDDN

>g02733.t1_1|Euryale_ferox/1-535

MANVQDSGTGTIEISKVGIIGAGVSGISAAKQLRHFSPIVFEATNSIGGVWKHCAFHSTRLQTPRNDYEFSDYPWPPMPEYPPPENDSATFPTHLEIIEYLKNYARHFDVLKFVKLNHRVVEIKYLSERKPTYYDGMWGDFGKPLSGELMWEVGVRTPLSEDIQWFKFEFLVVCLGKYGDVAKMPEFPTGKGPEIFQGTVLHSLDYSKLDRQEAERLVKGKKVVVVGYKKSAIDFATECAEANQGPDGQPCTMLIRTLHWIMLPSYSVWGISFLLLYATRFSQFLHERPSMSLFRALLCLLLSPLRKAISMFIELYLIWKLPLRKFGLVPDHAYEEDYASCQMAILPEKFFPAAEDGRIRFRRSSKWRFWSDGVELEDGSRLEADVVMLTTGFDGLKKLKWIFPEPFRHYIQDSSGIMPLYRGTIHPLVPHMAFVGYIESVSNLHTSEVRCRWLARLLQGAFVLPTVDKMVFEISKQMEVMKRTTRFYKRHCISTFSINHTDEMYEDMGLSPWRKRNWFLEAFSPYSNRDYKDDN

>g20115.t1_1|Euryale_ferox/1-535

MANLQDSGTGTLDISKVGIIGAGVSGIAAAKQLRHFTPIVFEATDSIGGVWKHCSFHSTRLQTPRNDYEFSDYPWPPMPEYPPPENDSATFPTHLEIIEYLENYARQFDVLKFVKLNHRVVEIKYLSERKPTYYDGVWGDFGKPLSGVPVWEVGVRTPLSEDIQWFKFEFLVVCLGKYGDVAKMPEFPTGKGPEIFQGTVLHSLDYSKLDPQETERLVKGKKVVVVGYKKSAIDFATECAEVNQGPDGQPCTMLIRTLHWIMLPSYSVWGISFLLLYATRFSQFLHERPSMSLLRALLCLLLSPLRKAISKFIELYLIWKLPLRKFGLVPDHAYEEDYASCQMAILPERFFPAAEDGRIRFRRSSKWCFWSGGVELEDGSRLEADVVMLATGFDGLKKLKWIFPEPFRFYIQDSSGIMPLYRGTIHPLVPHMAFVGYIESVSNLHTSEVRCRWLARLLQGAFVLPTVDKMVFEISKQMEVMKRTTRFYKRHCISTFSINHTDEMYEDMGLSPWRKRNWLQEAFSPYSNRDYKDDN

>Zosma04g19510|Zmarina_v3.1/1-522

MAMHRQGRFDPKVAIIGAGISGMVAAKELARFNPMVFEATDSIGGVWKHCSYRSTKLQTPRIDYEFSDYQWKDRRNPSFPTHDEILDYLAGYATHFDLWKNIYFRSKVVEIKFIGANVTSADRYSLSGHPVWEIAVQDHLSETIKWYKFEFVIMCIGKYGDVANIPVFPEGNGPEVFAGTVMHSMDYCKLDGEEIRRLVKGKKIIVVGYKKSAIDLAIECAAAAAKHCTMIVRGLHWVVPSYSIWGLPFFLFYSTRFSQFFHRTPNQGFLRSVLSYLSSPFRRGVSKFIESYLAWKLPLEKYGLKPDHPFVEDYASCQMAILPTDFFREADAGRIRFQKLPMTGGRNWCFYENGVLLNDKDDNVTKLEADVVFLATGFNGKGKLKSILPEPFRDIILDPSSGLIPLYRGTIHPLISQMAFVGYIESVSNLHTSELRCKWLSRLLDGRFELPTVEEMFANTREETEVMKKTTRFYKRHCISTYSINHSDEMCRDMGWRTRRKKGWFSDVFSPYGNQDYKEDEK

>Zosma06g17370|Zmarina_v3.1/1-527

MDGGKVNPGRYDPKVAIIGCGISGLAAAKELARFNPVVFEATDSIGGVWKHCSYHSTKLQTPRMDYEFSDYQWKDRKNSSFPSHIEILDYLDGYATHFDLWKFIRFQNKVVEIKHLLLNDDVNHSDQYWRRPIGQPIWKIGVEDSSSGGVKWQEFEFVVMCIGKYGDIANIPVFPPEKGPGVFNGKVMHSMDYCKLKKEETVELLKGKKVVVVGYKKSAIDLALECAQANAGEEGEPCTMIVRSLHWIVPSYSIWGLPFFLFFSTRFSQFLYERPNQGFLRSVICNLSDPLRKFVSKFIESYLTWKLPLRKYGLKPDHPFLEDYASCQMAILPDTFFDEADRGMIRFERPASPDWCFWEGGVLLDDGSTKLEADLVLLATGYDGKAKLKLVLPEPYRGLIDDPSGVLPLYRGMIHPEIPHMAFVGYIESVSNLHTSELRCKWLSRLLNEQIELPSVKTMLERTKEETEIMRRTTRFYKRHCISTYSINHSDEMCEEMGWGSWRKKGWLSEAFSPYNNQDYIEKQPED

>Sapof.06G177600|Sofficinalis_v1.1/1-517

MEKSSKIAILGGGISGIAAAKQLAKYEPVVFEATGSIGGVWKHASFRSTKLQTPRCDYEFSDFPWPDRNNATFPTNDEIIDYLHAYATHFDVLKFVRFNSKVVELRYIGGGPGGGRGGGNGQEYGELLCGHPAWEVAVQTGDDFEDIKRYTFELVVVCIGKYGDVPKMPTFAPKKGPEVFKGQVIHTLDYCKLNQEEASKLVKGKRVAIVGYKKSAIDLALECALANQGVDGKPCTMVIRTLHWTVPHYSIWGLPFYLFYSTRFSQFIHERPNESYFKALTSLLLSPMKRVISKIIESYLLWKLPYEKYGLKPEHPFHEDYASCQMAILPENFFSEADNGRIKFEKVTSWSFYENGLQFDNTKKVEFDVVICATGYDGKKKLEAILPQPFNNFLDYNGVMPLYRSTINPMIPNMTFVGYIETVSNLQAAELRCKWLARLVDDRFKLPSIDNMLGQIVKEVEVMKRTTRFYKRQCISTYSINHNDEICEEMGWTSWRKKSLIYEAFSPYTSQDYDGED

>LOC_Os03g08410|Osativa_v7.0/1-539

MAAAQQQQQKEGGARRTREEVPAVGRVAIIGGGISGLAAAKQLAAHDPVVFEATPHIGGVWKHCAYRSTRLQTPRPDYEFSDMAWPNRDDPSFPTHVEIVDYLEDYADRFGLWRYIALRSKVVGVKFLGGPSAGFTELWSGSGEPLQGKPMWELAVSSTDDPDSDDVQLYKFEFVVMCTGKYGDVARMPVFPPGKGPEVFKGKVMHSLDYCKLNEQETVELMRGKKVVVVGYKKSAIDLALECAEANQGEGGQPCTMLVRTLHWVVPSYSIWGLPFFLFYSTRFSQLFYERPNQGIFRSLLCRLMTPLQKAGVSKFIESYLSWKLPLSRYGLRPDHPFVEDYASCQMAILPDGFFDMADRDLIRFRRSAGGWCFSENGVVLDDGTHVDADLVFLATGFEGKDKLRSVLPEPFRGLVVNKSSMMPLYRGTIHPLIPNMAFVGYVESVSNLHTSELRCRWLAGLLGGRFALPAVEEMVRHVDGETEAMRRTTRFYRRHCISTYSIHDSDAMCADLGSRVHRKSNFLAELFAPYNNQDYKEE

>Zm00001d027908|Zmays_RefGen_V4/1-530

MAAAQQQSRSTRQAVPASSRVAIIGGGISGLAAAKQLAAHDPVVFEATPSIGGVWKHCVYRSTRLQTPRPDYEFSDYSWRNRDDPTFPTHGEIVEYLEGYADTFGLWRYIMLGAKVVDVKFLGGAAAGFTERWSGTGEALQGKPMWEVGVATAGSHTVQYYQFEFVVVCAGKYGDVPRMPVSFPEGKGPEVFRGQVMHSLDYCKLSEEETVELMRGKKVVVVGYKKSAIDLALECAQANQGEGGEACTMLVRTLHWVVPSYSIWGLPFFLFYSTRLAQFLYERPNQGILRSMLCRLMTPLRAGVSKFIESYLAWKLPLDKYGLRPDHPFVEDYASCQMAILPDGFFDMADRDMIRFKRASGGWCFSRNGVLLDDGTEVEADLVFLATGFEGKDKVRAVLPDPFRGLVVDRSGMMPLYRGTIHPLIPNMAFVGYVESVSNLHTSELRCRWLAGLLGGRFALPSVEDMVRHVEGEAEAMRRTTRFYRRHCISTYSIHDSDAMCADLGSRVLRKGNWLAELFAPYNNQDYKED

>Sobic.001G482000|Sbicolor_v5.1/1-529

MAAQQEQARSTRQAVPASSRVAIIGGGISGLAAAKQLAAHDPVVFEATPSIGGVWKHCVYRSTRLQTPRPDYEFSDYSWRNRDDPTFPTHAEIIEYLEGYADTFGLWRYIMFGAKVVDIKFLGGGGGFTELWSGTGGEPLQGKPMWEVGVATAGSDTVQYYQFEFVVMCAGKYGDVPRMPVFPPGKGPEVFRGQVMHSLDYCKLSEEETVELMRGKKVVVVGYKKSAIDLALECAQANQGEGGKACTMLVRTLHWVVPSYSIWGLPFFLFYSTRLAQFLYERPNQGLLRSMLCTLMKPLRAGVSKFIESYLAWKLPLDKYGLRPDHPFVEDYASCQMAILPDGFFDMADRDMIRFKRASGGWCFSRNGVLLDDGTELEADLVFLATGFEGKDKVRSVLPDPFRGLVVDRSGMMPLYRGTIHPLIPNMAFVGYVESVSNLHTSELRCRWLAGLLGGRFALPSVEDMVRHVDGEAEAMRRTTRFYRRHCISTYSIHDSDAMCADLGSRVLRKGNWLAELFAPYNNQDYKED

>ELECO.r07.3BG0291970|Ecoracana_v1.1/1-531

MAAEQQQQQARCTRSVVPPSSRVAIIGGGISGLAAAKQLAAHDPVVFEATPSVGGVWKHCVYRSTRLQTPRPDYEFSDYSWANRDDPTFPTHAEIVDYLEGYADRFDLWRYIMFGAKVVGVKFLGGAAAGFTELWSGTGEPLQGRPMWEIGVATGGPDDDNNWHKFEFVVMCTGKYGDVPRMPVFPQGKGLDVFKGQVMHSLDYCKLTEEETVKLMKGKKVVVVGYKKSAIDLALECAQANQGEGGEACTMLVRTLHWVVPSYSIWGLPFFLFYSTRLAQFLYERPNQGMLRSMLCRLMTPLRSGVSKFIESYLAWKLPLDKYGLRPDHPFVEDYASCQMAILPDGFFDMADQDMIRFKRASGWCFSEKGVVLDDGTEVEADLVFLATGFEGKDKLRAVLPDPFRGLVQDKSGMMPLYRGTIHPLIPNMAFVGFVESVSNLHTSELRCRWLSGLLGGRFALPSVEAMVRHVQGETAAMQRTTRFYRRHCISTYSIHDSDAMCADLGSRVHRKPNWLAELFAPYNNQDYKEE

>ELECO.r07.3AG0236120|Ecoracana_v1.1/1-532

MASEQQQQARCTRTVVPPSSRVAIIGGGISGLAAAKQLAAHDPVVFEATPSVGGVWKHCVYRSTRLQTPRPDYEFSDYSWRNRDDPTFPTHAEIVDYLQGYADRFDLWRYIMFGAKVVGVKFLGGAAAGFTELWSGTGEPLQGRPMWEIGVATGGPAGDDNVQWYKFEFVVMCTGKYGDVPRMPVFPQGKGLDVFKGQVMHSLDYCKLSEEETVELMKGKKVVVVGYKKSAIDLALECAQANQGEGGEACTMLVRTLHWVVPSYSIWGLPFFLFYSTRLAQFLYERPNQGMLRSMLCRLMTPLRSGVSKFIESYLAWKLPLDKYGLRPDHPFVEDYASCQMAILPDGFFDMADQDMIRFKRASGWCFSENGVVLDDGTEVQADLVFLATGFEGKDKLRAVLPDPFRGLVQDNSGMMPLYRGTIHPLIPNMAFVGFVESVSNLHTSELRCRWLAGLLGGRFALPSVEAMVRHVQGETAAMQRTTRFYRRHCISTYSIHDSDAMCADLGSRVHRKPNWLAELFAPYNNQDYKEE

>HORVU.MOREX.r3.4HG0405420|Hvulgare_Morex_V3/1-531

MAMAQGQAARATREAVPLVSRVAIIGGGISGLAAAKQMSAYDPVVFEATPSVGGVWKHCVYRTTRLQTPRPDYEFSDYSWKNRDDPSFPTHTEIVDYLEGYADEFDLWRYISFGSKVVDIKFLGGAEAGFTELWSGTGKAPLRGKPMWEVGVVTGDSNIVQYYKFEFVVMCTGKYGDVPRMPVFPPGKGPEVFKGTVMHSLDYCKLSEEEAVELMRGKKAVVVGYKKSAIDLANECAQANQGEGGQPCTMLVRTLHWVVPSYSIWGLPFSMFYSTRLSQLFYERPNQGFFRSLLCRLMSPLRTGVSKFIESYLSWKLPLGKYGLTPDHPFVEDYASCQMAILPEGFFDMADRGLVRFKRASAGWCFSENGVVLDDGTKVEADLVFLATGFEGKDKLREVLPKPFRDLVVGKSSMMPLYRGTIHPLIPNMAFVGFVESVSNLHTSELRCRWLSGLLEGRFELPTVKAMMGHVAGEADAMRRTTRFYRRHCISTYSIHDSDGMCADLGSATLRKANWIAELFAPYNNKDYKEQ

>HORVU.MOREX.r3.4HG0405470|Hvulgare_Morex_V3/1-531

MAMAQGQAARATREAVPLVSRVAIIGGGISGLAAAKQMSAYDPVVFEATPSVGGVWKHCVYRTTRLQTPRPDYEFSDYSWKNRDDPSFPTHTEIVDYLEGYADEFDLWRYISFGSKVVDIKFLGGAEAGFTELWSGTGKAPLRGKPMWEVGVVTGDSNIVQYYKFEFVVMCTGKYGDVPRMPVFPPGKGPEVFKGTVMHSLDYCKLSEEEAVELMRGKKAVVVGYKKSAIDLANECAQANQGEGGQPCTMLVRTLHWVVPSYSIWGLPFSMFYSTRLSQLFYERPNQGFFRSLLCRLMSPLRTGVSKFIESYLSWKLPLGKYGLTPDHPFVEDYASCQMAILPEGFFDMADRGLVRFKRASAGWCFSENGVVLDDGTKVEADLVFLATGFEGKDKLREVLPKPFRDLVVGKSSMMPLYRGTIHPLIPNMAFVGFVESVSNLHTSELRCRWLSGLLEGRFELPTVKAMMGHVAGEADAMRRTTRFYRRHCISTYSIHDSDGMCADLGSATLRKANWIAELFAPYNNKDYKEQ

>HORVU.MOREX.r3.4HG0405450|Hvulgare_Morex_V3/1-529

MAQGQEARATREAVPPVSRVAIIGGGISGLAAAKQMAAYDPVVFESTPSVGGVWKHCVYRTTRLQTPRPDYEFSDYSWKNRDDPSFPTHTEIVDYLEGYADEFDLWRYISFGSKVVDIKFLGGAEAGFTELWSGTGKAPLRGKPMWEVGVVTGDSNIVQYYKFEFVVMCTGKYGDVPRMPVFPPGKGPEVFKGTVMHSLDYCKLSEEETVELMRGKKAVVVGYKKSAIDLANECAQANQGEGGQACTMLVRTLHWVVPSYSIWGLPFFMFYSTRFSQLFYERPNQGFFRSLLCRLMSPLRAGVSKFIESYLSWKLPLGKYGLTPDHPFVEDYASCQMAILPEGFFDMADRGLVRFKRASAGWCFSENGVVLDDGTKVEADLVFLATGFEGKDKLREVLPKPFRDLVVGKSSMMPLYRGTIHPLIPNMAFVGFVESVSNLHTSELRCRWLSGLLEGRFELPTVKAMMGHVAGEADAMRRTTRFYRRHCISTYSIHDSDGMCADLGSATLRKANWIAELFAPYNNKDYKEQ

>HORVU.MOREX.r3.4HG0405500|Hvulgare_Morex_V3/1-529

MAQGQEARATREAVPPVSRVAIIGGGISGLAAAKQMAAYDPVVFESTPSVGGVWKHCVYRTTRLQTPRPDYEFSDYSWKNRDDPSFPTHTEIVDYLEGYADEFDLWRYISFGSKVVDIKFLGGAEAGFTELWSGTGKAPLRGKPMWEVGVVTGDSNIVQYYKFEFVVMCTGKYGDVPRMPVFPPGKGPEVFKGTVMHSLDYCKLSEEETVELMRGKKAVVVGYKKSAIDLANECAQANQGEGGQACTMLVRTLHWVVPSYSIWGLPFFMFYSTRFSQLFYERPNQGFFRSLLCRLMSPLRAGVSKFIESYLSWKLPLGKYGLTPDHPFVEDYASCQMAILPEGFFDMADRGLVRFKRASAGWCFSENGVVLDDGTKVEADLVFLATGFEGKDKLREVLPKPFRDLVVGKSSMMPLYRGTIHPLIPNMAFVGFVESVSNLHTSELRCRWLSGLLEGRFELPTVKAMMGHVAGEADAMRRTTRFYRRHCISTYSIHDSDGMCADLGSATLRKANWIAELFAPYNNKDYKEQ

>HORVU.MOREX.r3.4HG0405530|Hvulgare_Morex_V3/1-529

MEQGQAARATREAVPTVSRVAIIGSGVSGLAAAKQMAAYDPVVFEATPSVGGVWKHCVYRTTRLQTPRPSYEFSDYSWPNRDDPAFPTHTEVVDYLEDYADEFDLWRYISFGSKVVDIKFLGGAKAGFTELWSGTGKAPLQGKPTWEVGVATGGSDIVQYYKFEFVVMCTGKYGDVPRMPVFPLGKGPEVFKGRVMHSLDYCKLSEEETVELMRGKKVVVVGYKKSAIDLANECVQANQGEGGQACTMLVRTLHWMVPSYSIWGLPFFMFYSTRFSQLLYERPNQGFFRSLLCRLMSPLRAGVSKFIESYLSWKLPLAKYGLTPDHPFVEDYASCQLAFVPEGFFDMADRGLVRFQRALNGWWLSENGVVLEDGTEVEADLVFLATGFEGKDKLREVLPKPFRGVLVNNSSMMPLYHGTIHPLIPNMAFVGFVESASNLRTSELRCRWLAGLLEGRFELPSVEAMMGHVAGEADAMRRTTRFYQRHCISTYSIHDSDAMSADLSSATHHKSNWMAELFAPYNKEDYKKQ

>HORVU.MOREX.r3.4HG0406250|Hvulgare_Morex_V3/1-529

MEQGQAARATREAVPTVSRVAIIGSGVSGLAAAKQMAAYDPVVFEATPSVGGVWKHCVYRTTRLQTPRPSYEFSDYSWPNRDDPAFPTHTEVVDYLEDYADEFDLWRYISFGSKVVDIKFLGGAKAGFTELWSGTGKAPLQGKPTWEVGVATGGSDIVQYYKFEFVVMCTGKYGDVPRMPVFPLGKGPEVFKGRVMHSLDYCKLSEEETVELMRGKKVVVVGYKKSAIDLANECVQANQGEGGQACTMLVRTLHWMVPSYSIWGLPFFMFYSTRFSQLLYERPNQGFFRSLLCRLMSPLRAGVSKFIESYLSWKLPLAKYGLTPDHPFVEDYASCQLAFVPEGFFDMADRGLVRFQRALNGWWLSENGVVLEDGTEVEADLVFLATGFEGKDKLREVLPKPFRGVLVNNSSMMPLYHGTIHPLIPNMAFVGFVESASNLRTSELRCRWLAGLLEGRFELPSVEAMMGHVAGEADAMRRTTRFYQRHCISTYSIHDSDAMSADLSSATHHKSNWMAELFAPYNKEDYKKQ

>Bradi1g72500|Bdistachyon_v3.2/1-535

MAHHQQAAARSTRQWAPASSRVAIIGGGISGLAAARKLAAHDPVLFEATPSIGGVWKNCSYRTTRLQTPRPDYEFTDYSWSNRDDPSFPTHAEIVDYLEGYADAFGLWRYIMLGARVVGVRFLGAPDAAFTDLWSGSGKAQLDGKPMWEVGVETGTGDSATVQWYKFEFVVMCTGKYGDVPRMPVFPKGKGPEVFKGQVMHSLDYCKLSEPETVELMRGKKVVVVGFKKSAIDLANECAQANQGEGGQPCTMLVRTLHWVVPSYSIWGLPFFLFYSTRFSQLFYERPNQGFFRSLVCGLMSPLQRAGVSKFIESYLSFKLPLGRYGLRPDHPFVEDYASCQMAILPEAFFDMADRGLVRFQRAPDGWCFSETGVVLDDGTRVDADLVFLATGFEGKDKLRSVLPDPFRSLLVNKESSMMPLYRGTIHPRIPNMAFVGYVESVSNLHTSELRCRWLAGLLEGRFALPSVGEMMAHVEDEAEAMKRTTRFYRRHCISVYSIHDSDGMCADLGSATLRKRNCFAELFAPYNNQDYKEQ

>evm_27.TU.AmTr_v1.0_scaffold00007.327|Atrichopoda_v1.0/1-528

MENHDFSPKSGFIETKVAIVGAGISGIAAAKQLRNLKPVVFEVSDSTGGVWKHCTFHSTKLQTPRLDFQFSDYPWPKSVTSLFPSHSEFLDYLDGYARHFDVLKFIKFNSKVVHIRFSGDCKSTEFISNQWGEKGRPIFSRPIWELAVKTNDSDDLQWYRAEFVVMCTGKYGNIPRIPVFPHGKGPEKFNGKVMHTLDYSKLDKESAHQLLKGKKVVVVGYKKSAIDLATECAEANQGPDGKPCTVVIRTLHWIVPPTDYIWGVPFHLLYGARSAQFLHEKPSQTFFREVLCRLFAPVRKAVSKFVEAYLIWNLPIKKYGLVPDHPFEEDYASCQMALVPENFFPAADDGRIIFRRAEKWWFWSEGIELDDRSKVEADIVMLATGFEGSKKLKSLFPQPLTGMLEHPSGVMPLYRGTIHPLIPNMAFVGYIQGVSGVHAAELRCKWLAHLIEGGFVLPSVEEMMEHTNKEIKVMTKSTRFYKRQCTSTFDISQYDEICEDMGWKAWRKKNWVSEAFSAYTNLDYGDEN

>Cucsa.055910|Csativus_v1.0/1-513

MAAAAVTTTDQSRHRSVSKVGIIGAGISGIAAAKHLSSHHPVVFEATDSIGGVWKHCAYRTTRLQTPRRDFEFSDYPWPSQTTESDEFPTYLEILDYLHGYATHFDVLKFVKFNSKVVQLRYIGGPEAEDSTAAAGVYGSLLNGRPAWEVAVQDTHLNTIQWYEFELVVICVGRYGDIPNIPTFPHNKGQEVFKGKVLHSLDYAKLDHDDARALLQNKRVVIVGYRKSAIDLAVECAEANQGPEGKACTMLIRSLHWTVPSYRIWGLPFFLFYSTRFSQFLQETPNQGLLKSIFCSLFSPMIRKVFSKFIESYLTWKMPLEKYGLKPEHSFEEDYASCQMAIMPNTFFEEADKGKIVFKKTTKWWFWSGGVVFDEQDNNNTTTKLEADVVIFNTGFQGKLKLQSLLPHPFNTLLVDSSDIVPLYRGVIHPLIPNMAFVGYLESVSNLRTGELRCKWLAKLADDGFKLPSIHQMLQQISDEIQVMKNTTRFYKRQCVSTFSISYTDEISQDIYG

>Nycol.I00674|Ncolorata_v1.2b/1-417

MGKRVGIIGAGISGLLSCKYMLQRGYEPIVFEFQSSIGGVWTSPAPPTPPSPVAPPPSSDPAAFMPQSTPPRPAYQFSDFPWPPKSNEEYPNRSKVHRYIESYARHFNLIDCIRFNSKVLSLEYLGATDEEILSWSLWANTGHAFSSEGAWKITVQDTEHETTHVYEVEFVVVCIGKFNSAPKMPALPSDKGPEVFKGKVMHAKEYLMMDELDAVELIEGKKVVIVGTHKSAFDIATQCARENGLESPCTMVITSPNKNSNDAIPNAAGRSNGTIISKLPFALPDDFHKRVEEGSIILEQSTEWLFCEAGLTLGSEDSQVNADIVIIATGCNGDHRLMSLFKSPAIQKLILSSSSGEAPLYRNCIHPRIPQMAIMATSDGVFSINGSETRVKWLVEFFEGGFRLPTVKEMEDDSGCE

>g08439.t1_1|Euryale_ferox/1-418

MGKKVGIIGAGISGLLSCKYMLQEGYEPIVFEFQSSIGGVWTSPAPPTPPSPVAAPPPSDQTAATPLSTLPRPAYQFSDFPWPPKSNEEYPNRSKVHRYIESYARHFNLIDCIRFNSKVLSLEYVGATDEDILSWSLWGNTGDAFSSERAWKITVLDTELETIHVYEVEFVVVCIGKFDSAPKMPALPSDKGPEVFKGKVMHAKEYLMMDEIDAVDLIEGKKVVIVGVHKTAFDIATQCARENGLENPCTMVITSPNKNSTDAIPKAFGRSNGTIISKFPFALSDDFHKRVEEGSIILEQSTEWQFCESGLTLGSEDSRMNADIVIIATGYNGDYRLMNLFKSSAIQKLILSSSAGETPLYRNCIHPRIPQMAVLATTDGVFSILGSETRVQWLVRFFHGGFRLPTVKEMEEDKIECK

>PITA_02600|Pinus_taeda/1-484

MMVLVPKKIPLQSSTVPSLLINHKFNHCCQRPPRKPLIAMAHDEPNKVCRVAVIGAGAAGLVAARELNREGHQIVVFEQTDHVGGTWVYDPYVETDPLGLDTARTVVHSSMYLSLRTNIPREVMGFMDYPFKVVEGRDCRRFPGHKEVALYLQDFAAEFGLLRSIRFRTEVEYVGRDPKNENQWIVRSNAEKRDVVEEVYDAVVVCNGHYTQPRVAEIPGIDKWPGKQIHSHNYRVPAPFQDQVVIVIGNAASGEDISKELMSVAKEVHLSARSITSEVIMREQLAAPGNLSLHPMIEYVCEDGTVRFQDGCSIIADTIIHCTGYYYHFPFLETEGIVSVNDNCVGPLYQHVFPPVLAPSLSFVGLPWKVVPFPLCELQSRWIAKTLSRKAHLPSKHDMIEAVRNFYVQNEAAGRPKHYTHNIGHCQFEYDDWLADQCGCAHVEDWRKNIYAASGKNKVSHPRTYRDEWEDHYWLEIALLDLLK

>PSME_00001261-RA|Pseudotsuga_menziesii/1-507

MISILPKHVTQQSAILPSLLLINHKFNHYRNIFNHFCQRPPRKLLKAMAHNEPNKACRVAVIGAGAAGLVAARELNREGHQIVVYEQTDHVGGTWVYDPHVETDPLGLDPARTVVHSSMYSSLRTNIPRDVMGFMDYPFKVVEGRDCRRFPGHKEVALYLEDFAAEFGLLSFIRFRTEVEYVCRDPNNENQWIVRSNAEKRDVTEEVYDAVVVCNGHYSEPKVAEIPDINKWPGKQIHCHNYRVPAPFQDQVVIVIGNGASGEDISRELASTAKEVHLSARSFTSEVIMKEYLAVSGNISLHPMIEYVCKDGTVRFRDGHSIVADTIIHCTGYSYHFPFLETGGIVSVNDNCVEPLYEHVFPPALAPSLSFVGLPWKVVPFPLCELQSRWIAKTLSGKAHLPSKNDMIEAVRNFYVQNEAAGRPKHYTHNIGHCQFEYDDWLADQCGCSHVEDWRRNIYRATSINRVSHPQTYRDEWEDHYWLEIALSDLPNSTLTLFFICLPLFLL

>MA_809037g0010|Picea_abies/1-187

SGCDRSRSSWAGSCPGIRVAVIGAGAAGLVAARELNREGHQIVVFEQTDHVGGTWVYDPHVETDPLGLDPARTVVHSSMYLSLRTNLPREVMGFMDYPFRVVEGRDCRRFPGHKEVALYLQDFASEFGLLRFIRFRTEVEYVGRDPKNENQWIVRSNADKRDVMEEVYDAVVVCNGHYTQPRVAEIP

>Thupl.29379979s0007|Tplicata_v3.1/1-448

MRETTKFSKVAVIGAGAAGLVAARELHREGLEVVVFEQSDNVGGTWVYNPHVESDPLGLDPNRRVVHSSMYLSLRTNLPRDLMGFLDYPFGMVEGRDCRRFPGHREVALYLEDFAAEFDVLRFIRFRTEVEYVGMVTKNQWIVRSCSDRADTREDFYDAVVVCNGHHTQPRIAQIPGIEIWPGKQIHSHNYRVPDSFRDEVVVIIGNSYSGEDIALELVGVTKQVHVSTRSRKSDAPVTNPLDGLYKIFLHPEIKSLCQDGTVNFEDGASIVADSIIHCTGYSYYFPFLDTKGIVTVNDNCVGPLYKHVFPPLLAPSLSFVGLPWQAIPFVVCELQSKWIAHTLSGKAILPSKNDMIEAVKDFYARNETAGQPRHYIHSLGPHQSEYMDRLADQCGCVHVEAWRKEMYEETLRNRVKHPQTYRDEWEDHFWVEKALLSQSDGKNILIV

>KAH9313936.1|Taxus_chinensis/1-241

VVVIIGNSYSGEDIALELAGVSKEVHISARSRKSEVTLTNPVDGPGNIFLHPVIESLCRDGTINFHDGASIVADSIIHCTGYSYHFPFLDTKGIVTVNDNRVGPLYEHVFPPLLAPSLSFVGLPWMTVPFVLCELQSKWIACILSGKTLLPSENDMMEAVKDFYARNEAFGRPNHYTHCLGTYQFGYLDWLSDQNGCAHVEAWRKEMSEETVRNRVKRPHTYRDKWEDLYWLQMALSPESN

>KAH9327748.1|Taxus_chinensis/1-131

IESLCQDGTINFHDGASIVADSIIHCTGYSYHFPFLDTKGIVTVNDNRVGPLYEHVFPPFLAPSLSFVGLPWMTVPFVLCELQSKWIACILSGKTLLPSENNMMEAVKDFYARNEAVGRPNHYTHCLGTYQ

>Thupl.29379979s0009|Tplicata_v3.1/1-521

MQLSCFVLNIKKNSIYMPRCHLSDAMSLVFRTVPLRSAIISVSVSPIQYKYKSNYNTTIHYTNIPKTTSPEHHSTMKGIPKTCKVAVIGAGAAGLVAARELDRENHQIVVFEQSDNVGGTWVYDPQVESDPLGLDPNCTVVHSSMYLSLRTNLPRELMGFLDYPFRVVEGRDCRRFPGHREVALYLEDFAAEFDLLRFIRFRTKVEYVGMGNNNQWIVRSLADSTDLKEEFYDAVVVCNGHYTQPRIAEISGIDRWPGKQMHSHNYRVPDPFQDEVVVIIGNSASGEDIAKELAGVAKEVHISVRSLNSPVNMTKPADGPGKTFLHPLIESLCQDGTVSFQDGASIVADSIIHCTGYSYHFPFLDSKGVVTVNDNCVGPLYEHVFPLLLAPSLSFIGLPWKVVPFPLCELQSKWVACTLSGKAIIPSENHMTEAIKDFYAQNEAAGRPKHYTHNIGNNQFEYCDWLADQCGCAHLDTWRKKMYESTSRNKVKRPQTYHDEWEDHYWREIALSSQSKRKEHT

>PITA_43622|Pinus_taeda/1-223

IEYVCEDGTVMFRDGYSIVADTIIHCTGYSYHFPFLETEGIVTMNDNCVGPLYEHVFPPVLAPSLSFVGLPWQVAPLSVCELQSRWIAKTLSGKARLPSENDMIEAVRNLYAQNEAAGRPKHYTHNIGHCQSEYVNWLADQCGCAHVEDWWENIYALTFKYRASHPQTYRDEWEYHYSLEIALKGLSNVNYVLQYHIRGLFFLVGTDLFRSRLRAFTVKEYHS

>PITA_33587|Pinus_taeda/1-277

QVVIVIGNSASGEDISRELASVAKEVHVSDRSITSEVIMRENLAGPGNISLHPMIEYVCEDGTVMFRDGYSIVADTIIHCTGYSYHFPFLETEGIVTMNDNCVGPLYEHVFPPVLAPSLSFVGLPWQVAPLSVCELQSRWIAKTLSGKARLPSENDMIEAVRNLYAQNEAAGRPKHYTHNIGHCQSEYVNWLADQCGCAHVEDWWENIYALTFKYRASHPQTYRDEWEYHYSLEIALKGLSNVNYVLQYHIRGLFFLVGTDLFRSRLRAFTVKEYHS

>CYCAS_017148|Cycas_panzhihuaensis/1-478

MASEENSNECNKYKKVGVIGAGASGLVTARELKREGFEVTVYERNDDVGGVWLYDPRIEEDDLLGSNPNRKVVSSSLYASLRANLSREIMGFMDYPFTVKDGRDGRRFPGHREIQLYLRDFATDFNLMELIQFSTEVVYVGMMPKRQRRVSDESDSDCKIDSNHSEGLRHNIRWIVRSFRRGLSGASVVEEVFDAVVVCSGHYSQPKIPQIPGMNTWPGKQMHSHNYRVPDPFRGQVVVIIGNSSSGHDISVELASVAKEVHLSARSASFADTTQVLAKHPNLQHHSMVESLEESGTVIFQDGSSVVADSIIHCTGYSYSFPFLDTEGKVTVDDNHVGPLYEHIFPPLLAPSLSFVGIPQKVITFPFFELQSKWVARILSGKAELPSRKDMMKAVEDLYNCLDIAGLPMNSTHFIDNFQYYDWLADQCGSARIEEWRKEICLAILNNIKSNIETFREVWYDQHWLEIALLDKCKGPIS

>CYCAS_017149|Cycas_panzhihuaensis/1-486

MGSEGSSHRSNRFRKVGVIGCGASGLVAARELKREGFEVVAYERNDGIGGIWVYDPRIEEDDLLGLNPNRKVVTSSLYASLRSNLSREIMGFTDYPLIVKDGRDGRRFPGYQELQLYLTDFATDFNLMELIKLSTEVVYVGPVPKHPFITNDNGLSGGNKSNYNCDIYSTDSEQLDHDIQWLVRSCNRTVNGTSVVEEEVFDAVVVCSGHHSQPRVPHIPGMHVWPGRQIHSHNYRDPDPYRDQVVIIIGNSSSGIDISLEVAEIAKEVHVSARSAGFADITKILVDHPKLQLHSMVESLEENGTVIFQDGCSVVADSIIYCTGYSYSFPFLDTKGKVTVDDNRVGPLYQHIFPPLLAPSLSFVGIPWKVISFPFFELQSKWIACILSGKAELPSEKDMMRAVEDLYNHQEMAGLPKHYTHCIEGFQIYDWLADQCGSARIEEWRKEIFLATLSNIRSNAKTFRYSWDDQHLLEIALLDKPIGPFV

>CYCAS_017264|Cycas_panzhihuaensis/1-489

MVSERWCSNRTGIGNGNSQYRSWKVCVIGAGPAGLVTARELRREGHQVIVYEQNEDVGGTWLYEPRIEGDDPQGLKVHSSVYASLRLNSSRETMGFTDYPFVVKEGRDCRRFPGHRDLLLYLKDFTRHFGILDLINFNTKVEYVGMRSRADPDKKLYVDDLLDASKCGSMDKPVKGSDWSNIEWVVRSRRRTRGCSVEEEEEEDAVEEIFDGVVVCNGHYSYPRMAQIPGMQRWPGKQIHSHSYRIPDPFCGEIVVVIGNSQSGQDISLELLNVAKEVHLSARSVNITGALSNIHANHLNFQMHPVVESLHENGTVLFQDGASVVADSIIYCTGYSYTFPFLDTKGIVTVDDDRVGPLYEHIFPPLLAPSLSFVGIPRKIIAFPFFESQSKWIAHILSGKAVLPSRKDMMKAVEVFYGLKEAAGLPKHSTHDIADFEYCDWLADQAGCPRLEEWRKEITLATLRNAESNLQTFRDSWDDQYWLELALLS

>Aqcoe3G055400|Acoerulea_v3.1/1-458

MVSERNLYITKHVCVIGAGPSGLVAARELRKEGHTVVVLEQNHEVGGQWLYEPNIEGEDPLGKASFLKVHSSIYASLRLTSPREIMGFTDFPFLAKKGRDMRRFPGHSELLQYLKDFCDWFGLREMIRFNTRVEYVSMFDFAKFGRDKWIVRSKEILSEKVNEEVFDAVVVAIGHYSHPRLPNIKGMDTWKRKQMHSHIYRVPQPFCNEVVVVVGNSLSGTDISMELVDVAKEIHLSAKSLEISEGLSKVISKNRSLHLHPQIESLYEDGRVQFVDGSWVIADSIIYCTGYTYSFPFLDTKGIVVVDDDRVGPLYEHTFPPSLAPSLSFVGIPRKLIGFPFFESQAKWIAQLLSGKRTLPSWDDMMKSIKEFYNSRDEAGIPKHNTHDIASFEYCDKYGEHCGFPHLEDWRKELCVSALINADKDLEAYRDSYDDHELLQVAHESPHYTQLGPEAFDV

>Hyque.15G033900|Hquercifolia_v1.1/1-458

MVSERVRLSKHMCVIGAGPSGLVAARELRKEGHNVVVFEQSHDVGGQWLYEPKVEGEDPLGKRGVLKVHSSIYASLRLTSPREIMGFTDFPFLVKKGRDMRRFPGHMELLLYLKDFCEVFGLREMIRFNTRVEYVGMLDYGVFGKDVKWFVRSKEKKGEKSVEEIFDAVVVATGHYSQPRLPSIKGMDSWRRKQMHSHIYRVPEPFRNEVVVVVGNSLSGQDISMELVDVAKEIHLSAKSLDVSEGLSKVLSKHENLHLQPEIDSLNEDGKVLFVDGSWVIADTIIYCTGYSYSFPFLDTKGIVVIDDDRVGPLYEHTFPPSLAPSLSFVGIPRKLIGFPFFESQAKWIAELLSGKRTLPSWDDMMLSIKDFYHSRDAAGIPKHNTHDLASFEYCDKYGDHIGFPHLEEWRKVLCLSALKNADVNLDTYRDSYDDLEMLQVAHQSPHFTQLGIEAFHL

>Soltu.DM.03G029680|Stuberosum_v6.1/1-455

MVSERNLSKNVCIIGAGPSGLVAARELRKEGHNVVVFEQNHEVGGQWLYQPKVEHEDPLGSSNVLKVHSSIYASLRLTSPREIMGFTDFPFVVKKGRDPRRFPGHKELLLYLQDFCEYFGLREMIRFNTTVEYVGMLDYPEFGKDLRWIVKSKEKEAEERVEEVFDAVVVATGHYSQPRLPSIKGMDAWRRKQMHSHIYRVPEPFRNEVVVVVGNSLSGQDIGMELVDVAKEIHLSAKSLDVSEGLSKVISKHQNLHLHPQIDSLHEDGRVLFVDGTSIVADTIIYCTGYSYSFPFLDTKGMVIVDDDRVGPLYEHTFPPSLAPSLSFVGIPRKLIGFPFFESQAKWIAQLLSGKRTLPSWDEMMQSIKEFYRSREVDGIPKHNTHDLANFEYCDKYADYIGFPHLEEWRKELCLSVLRNADINLDTYRDSYDDSEMLQEAYQSPHFAHLGPETF

>Solyc03G002827|Slycopersicum_ITAG5.0/78-532

MVSERNLSKNVCIIGTGPSGLVAARELRNEGHNVVVFEQNHDVGGQWLYQPKVEHEDPLGRNNILIVHSSIYASLRLTSPREIMGFTDFPFVVKKGRDPRRFPGHKELLLYLQDFCEYFGLREMIRFNTRVEYVGMLNYPEFGKDLRWIVKSKEKEAEEGVEEVFDAVVVATGHYSQPRLPNIKGMDAWRRKQMHSHIYRVPEPFRNEVVVVVGNSLSGQDIGMELVDVAKEIHLSAKSLDVSEGLSKVISKHQNLHLCPQIDSLHEDGRVLFVDGTSIDADTIIYCTGYSYSFPFLDTKGMVIVDDDRVGPLYEHTFPPSLAPSLSFVGIPRKLIGFPFFESQAKWIAQLLSGKRTLPSWDEMMQSIKEFYRSREIDGIPKHNTHDLANFEYCDKYADYIGFPHLEEWRKELCLSVLRNADKNLDTYRDSYDDSEMLQEAYQSPHFAHLGPETF

>TESI_021357-RA|Tetracentron_sinense/1-457

MVCEKNHPKFVCVIGAGPSGLVAARELRKEGHSVIVMEQNHDIGGQWLYDPRVENEDPLGKGTTLKVHGSIYASLRLTSPREIMGFTDFPFLVKKGRDMRRFPGHEELLLYLKDFSEWFGLREMIRFNTRVEYVGMVDYGKFFKDMKWVVRSKDKKSEKVIEEVFDAVVVATGHYSQPRLPSIKGMDAWRRKQMHSHIYRVPEPFRNEVVVVVGNSLSGQDISMELVGVAKEIHLSSKSLEVSEGLSKIISKYENLHIHPQIDSLREDGRVLFMDGSWVIADTIIYCTGYSYSFPFLDTKGIVVVDDDRVGPLYEHTFPPSLAPSLSFVGIPRKLIGFPFFESQAKWIAQLLSGKRTLPSWDDMMQSIKEFYHSRDVAGIPKHNTHDIANFEYCDKYGDQIGFPRLEKWRKELCLSALRNADADLETYRDSWDDLEQLQIARQSPHFTEFGPEAFSM

>GWHPBAUK022935.1|Macadamia_integrifolia/1-457

MVSMGSNSKHVCVIGAGPSGLVAARELRKEGHSVVVLEQNHDIGGQWLYEPKIENQDPLGRGSTLEVHGSVYASLRLASPREIMGYTDFPFLAKKGSDMRRFPGHREVFMYLKDFCDWFGLREMIKFNTRVQYVGMVDSSMFGKDIKWFVRSKDKKSEKVMEEEFDAVVVATGHYSQPRLPTIKGMDTWKRKQMHSHIYRVPEPFRNEVVVVVGSSMSGQDISIELVDIATEIHLSAKSLEISQGLAIVISKYDNLHLHPQIDSLWEDGRVQFVDGSWVIADSIIYCTGYSYSFPFLDTKGIVVVDDDRVGPLYEHTFPPSLAPSLSFVGIPRKIIGFPFFESQAKWIAQLLSGKRTLPSWDDMMQSIKEFYHSRDVKGIPKHNTHDIADFEYCDKYGEYCGFPHLEDWRKQLCISAIRNADANLETYRDFWDDHQLLDVAHQSPHFTQFGPDAFGQ

>BUSI_018021-RA|Buxus_sinica/32-489

MVLETKNSKNVCVIGAGPSGLVAARELRKEGHSVVVIEQNHDIGGQWLYDPNVELENPSGRGNILKVHSSLYASLRLLSPREIMGFTDFPFLVKKGRDMRRFPGHKELFLYLRDFCEWFGLREMIRFNTRVDYVGMVDSAINSKDMKWVVRSKVKKTDKVIEETFDAVVVAVGHYSQPKLPSIKGMDTWRRKQMHSHIYRVPESFSNEVVVVVGNSLSGQDISMELVDIAKEIHLSSKSLEVSEGLSKVLSKHTTLHLHPQIDSLWGDGRVLFVDGSSVIADTIIYCTGYSYSFPFLDTKGIVVVDDNRVGPLYEHTFPPSLAPSLSFIGIPRKIIGFPFFESQAKWIAQLLSGKRTLPSWEDMMQSIQEFYHSRDVAGIPQRNTHDIASFEYCDKYGDYIGFPHTEEWKKELCLSALRNAETNLENFRDSWDDDLELLQVAHESPHFTQLEPEAFDL

>Qrob_P0688630.2|Quercus_robur/1-458

MVSVRTQSKHVCVIGAGPSGLVAARELSKEGHSVMVLEQNHDVGGQWLYDPNVEVEDPLGRNTFLKVHSSIYNSLRLTSPREIMGFTDFPFLVKKGRDMRRFPGHKELFLYLKDFCDWFGLREMIRFNTRVEYVGLLDSEAIGKDLRWVVRSKEKKTEKVVEEVFDAVVVATGHYSQPRLPSIKGMDAWKRKQMHSHIYRVAEPFHNEVVVVVGNSLSGQDISIELVEVAKEVYLSSKSLNISEGLSKILSKHDTLHLRPQIESLHEDGRVLFVDGTWVIADTIIYCTGYSYTFPFLDTKGIVAVDDDRVGPLYEHTFPPSLAPSLSFMGIPRKIIGFPFFESQAIWIAQLLSGKRTLPSWDDMMQSIKDFYHSRDLAGVPKHYTHDIAEFEYCDRYGDHVEFPHLEEWRKKLCVSVLVNADVNLETYRDSWDNDHELLQEALQSPHFTQLGTEAFTL

>Prupe.5G199900|Ppersica_v2.1/1-464

MVSETYQSKKVCVIGAGPSGLVAARELRKEGHRVVVLEQNHDVGGQWLYDPNVEGEDPLGRRPSLKVHSSLYTSLRLISPREIMGFTDFPFSVKKGRDMRRFPGHRELLLYLKDFCDWFGLRDLIRFNTRVAYVGMLDYYATSTDDVVGCEDLKWVVRSVDKKTQIVMEEVFDAVIVATGHYSQPRLPSIKGMDAWKRKQMHSHIYRVPEPFADEVVVVVGTSLSGQDISMELVEEAKAVYLSGKSLDISEGLSKVISKHENLHLRPQIDCLQEDGKVLFVDGSWVIADTIMYCTGYSYTFPFLDTKGIVAVDDDRVGPLYEHTFPPSLAPSLSFIGIPRKIIGFPFFESQAKWIAQLLSGKRTLPSRDDMMQSIKELYHSRDVAGIPKHNTHDIAEFEYCDRYGDHIGFPRLEEWRKELCLSALRNADTDLETYRDSWDDHELLQEALQSPHFTQHGAQDFPL

>VIT_217s0000g06790|Vvinifera_v2.1/1-454

MVSERNQSKHVCVIGAGPSGLVAARELRKEGHSVVVIEQNHDIGGQWLYEPKVEGEEALGKSTFLKVHSSIYDSLRLFSPREIMGFSDFPFVVKKGRDMRRFPGHRELLLYLQDFCEWFGLREMIRFKTRVEYVGMLDSDQVGRDLKWVVKSRDMESEKVVEEVFDAVVVATGHYSKPRLPSIKGMDVWKRNQMHSHIYRDPEPFRKEVVVVVGNSLSGQDISMELVDVAKEIHLSAKSLEISEGLAKVISKHDSLHLHLEIESLHEDGRVVFVDGSWVMADTIIYCTGYSYAFPFLDTKGIVGVDDDRVGPLYEHTFPPSLAPSLSFVGIPRKLIGFPFFESQAIWIAQLLSGKRTLPSFHDMMQSIKDFYHSREIAGVPKHNTHDLANFEYCDKYGDNVGFPHLEEWRKELCISAIVKAEVDLEGYRDAWDDVELLQVALQSPHFTQFPRST

>Potri.012G067500|Ptrichocarpa_v4.1/1-458

MVSPLSLSKKVCVIGAGPSGLVAARELRKEGHNVVVLEQRYDVGGQWLYEPNVESEDPLGKKKFLQVHSSIYASLRLISPREIMGFTDFPFLVKKGRDTRRFPGHRELWLYLKDFCEHFGLREMIRFNTSVEYVRMLEYDEIGKDLKWMVKSRDKSSEEMAVEEVFDAVVVATGHYSHPRLPTIKGMDTWKRKQMHSHIYRVPEPFRNEVVVVVGNSLSGQDISMELVEVAKEVHLSAKSLNITEGLSKVISKHENLHLHPQIETLQEDGRVVFVDGSWLIADTILHCTGYSYTFPFLDTKGMVAVDDDRVGPLYEHTFPPALAPSLSFVGIPRKIIGFPFFEAQAKWIAQLLSGKRTLPSREEMMHSIEEFYRSRDAAAIPKHYTHDIGDFEYCDRYGDHIGFPHLEEWRKQLCLSALVNADANLEAYRDSWEDHELLQEALQSPHFTQLGAEDLTF

>Glyma.06G198100|Gmax_Wm82.a6.v1/37-495

MVSETLQSQTKNVCVVGAGPSGLVAARELRKEGHSVALLEQNHDVGGQWLYELNVEGEDPLGKKPFLKVHSSIYESLRLTSPREIMGFTDFPFLVKKGRDMRRFPSHTELLMYLKDFCDHFGLREMIRFNTRVDYVGMLDYGVCSNDLKWVVRSVDKKSEKVVEEVFDAVVVATGHYSQPRLPSIQGMDTWKRKQMHSHIYRTPEPFRNEIVVVVGNSLSGQDISIELVDVAKEVHMSSRSLNISEGLSKVISKHANFHLHPQIETLQEDGRVTFVDGSSIFADSILYCTGYSYAFPFLDTKGMVVVDDDRVGPLYEHTFPPSLAPSLSFIGIPRKIIGFPFFESQAIWIAQLLSGKRVLPSWEEMMKSIKEFYHSREAAGIPKHCTHEIANFEYCDKYGDNVGFPRIEEWRKQLCLSALINSDANLETYRDSWDDHELLKEALQSPHFTQLGLEDSPM

>Glyma.04G165000|Gmax_Wm82.a6.v1/1-459

MVSETLQSQTKNVCVIGAGPSGLVAARELRKEGHRVVLLEQNHDVGGQWLYESNVEGEDPLGKKPFLKVHSSIYESLRLTSPREIMGFTDFPFLVKKGRDMRRFPSHTELLMYLRDFCEHFGLGEMIRFNTRVDYVGMLDYGVCSNDLKWVVRSEDKKSEKTVEEVFDAVVVATGHYSQPRLPSIQGMDTWKRKQMHSHIYRTPEPFRNEIVVVVGNSLSGQDISIELVDVAKEVHMSSRSLNISEGLSKVISKHANFHLHPQIETLQEDGRVTFVDGSSILADSILYCTGYSYAFPFLDTKEMVVVDDDRVGPLYEHTFPPSLAPSLSFIGIPRKIIGFPFFESQAIWIAQLLSGKKVLPSWEEMMKSIKEFYQSREAAGIPKHCTHEIANFGYCDKYGDNVGFPRIEEWRKQLCLSALINSDANLETYRDSWDDHELLQEALQSPHFTQLGLEDSPM

>Pso04G22690.0|Papaver_somniferum/1-458

MVSERNPCSNHVCVIGAGPSGLVAARELRKEGHTPIVLEQNYDIGGQWLYEPRVENEHPLGKNDTLKVHSSMYASLRLTSPREIMGFSDFPFLIKKGRDMRRFPGHKELLLYLRDFVEWFKLRDMIRFNTKVEYVGMLDFVKFGKEKWIVRSKDMRDEKSIEEVFDAVIVATGHYSQPRLPNIKGMDSWRRKQMHSHIYRIPDPFRNEVVVVVGNSLSGTDISMELVGVAKEIHLSAKSLEITEGLSKVLSKHKKHLLLHPQVESLREDGRVLFVDGSWVMADSIIYCTGYSYSFPFLDTKGMVVIDDDRVGPLFEHTFPPSLAPSLSFVGIPRKIIGFPFFESQAKWIAQVLSGKRTLPSWEDMMESIKVFYKSRDIEGIPKHNTHDICSFEYCEKYGDYCDFPHLEEWRKELCLAALKNSFANLETYRDSWNDDELVQEAHHSPHFTQLGPEAFDM

>Pso05G13710.0|Papaver_somniferum/1-304

MKRDEKSIEEVFDAVIVATGHYSQPRLPNIKGMDSWRRKQMHSHIYRIPDPFHNEVVVVVGNSLSGTDISMELVGVAKEIHLGAKSLEITEGLSKVLSKHKKTLTPPSTESLREDGRVSFVDGSWVTADSIIYCTGYSYSFPFLDTKGMVVIDDDKVGPLFEHTFPPSLAPSLSFVGIPRKIIGFPFFESQAKWIAQVLSGKRTLPSWEDMMESIKVFYKSRDIEGIPKHNTHDICSFEYCEKYGDYCDFPHLEEWRKELCLAALKNSFANLETYRDSWDDDELVQEAHHSPHFTQLGPEAFDM

>orange1.1g044575m.g|Csinensis_v1.1/1-454

MVSDDHQQCAQSKNVCVIGAGPSGLVAARELRKEGHRVVVLEQNHDVGGQWLYDPNTDQTEVHSSVYASLRLTSPREIMGYTDFPFVLKKGRDVRRFPGHKELWLYLKDFCQRFGLREMIRFNTRVEYVGMLDCGELIIGNDLIKWVVKSKEKKADKVVEEVFDAVVVATGHYSYPRLPSIKGMDKWKRKQMHSHIYRVPEPFRNEVVVVVGNSLSGQDISMELVEVAKEVHLSAKSLNISEGLSKVISKHNNLHLHPQIDCLREDGRVTFVDGCWVTADTILYCTGYSYSFPFLDTKGIVVVDDDRVGPLYEHTFPPSLAPSLSFVGIPRKLIGFPFFESQAKWIAQLLSGKRTLPSWDQMMQSVKEFYHSRDVAGIPKHNTHDIANFEYCDRYADQIGFPHLEEWRKGLCISALVNSDANLETYRDSWDDHELLQEALQSPHFTQLNAQAFD

>CKAN_02499200|Ckanehirae_v3/1-453

MVSVKSHSKHVCVIGGGPSGLVSARELKKEGHSVVVIEQNHDVGGQWLYEPCVESEDALGKEKFLKVHSSIYASLRLTSSREIMGFTDFPFSVKKGRDMRRFPSHRELHLYLKDFCDWFGLREMIRFHTRVEYVGMLNYGVFGEDLKWVVRSVEKNTGKVMEEVFDAVVVATGHYSHPRLPTIKGMDAWKRKQMHSHVYRVPEPFRNEVVVVIGNSLSGQDISMELVDVAKEVHLSAKSLDISNGLSKVVSKTSTLHIHPQIDSLHEDGRVSFVDGSWVTADSIIYCTGYSYSFPFLDTKGIVVVDDDRVGPLFEHTFPPSLAPSLSFVGIPRKIIGFPFFESQARWIAQLLSGKKTLPSWDDMMKSIKDFYHSLDEAGVPKHKTHEIANFEYCDRYGDYCDFPYLEQWRRELVLATLKNAEENLETYRDSWYDEEQLHEALQSPHFSQLSHI

>Litul.19G028900|Ltulipifera_YP108A_v1.1/1-460

MVSEKIHTKHVCVIGGGPSGLVAARELRKEGHSVVVIEQNHDIGGQWLYDPSVVSGSSEDALGKGSFLKVHSSIYASLRLTSSREIMGFTDFPFLIKKGRDMRRFPGHQELYSYLKDFCEHFGLRDLIRFNTRVQYVGMLNYAEFGEDLKWVVRSIEKKGEKVSEEVFDAVVVATGHYSQPRLPTIKGMDAWKRKQIHSHVYRVPQPFHNEVVVVIGNSLSGQDISMELVDVAKEVHLSAKSLEISEGLSKVISKYHTLHIHPQVDSLHEDGRVLFVDGSCVVADSIIYCTGYSYSLPFLDTKGILVVDDDRVGPLYEHTFPPSLAPSLSFVGIPRKIIGFPFFESQARWIAQLLSRKRTLPSWDDMMSSIKEFYHALDVAGIPKHKTHEIANFAYCDRYGEHCDFPHVEQWRKDIVLATLKNAEENLETYRDSWDDEELLQEALHSPHFRQLASEASSL

>KAI5410180.1|Pisum_sativum/1-455

MVSETTNQSKNVCVIGAGPSGLVAARELRKEGHKAIVLEQNHDIGGQWLYDDQNVEGEDPLGRNPLLKLHSSVYSSLRVASPREVMGFTDFPFLIKKGRDMRRFPSHVEVLMYLKDFCEWFGLREMIRFNTRVEHVGMLDENGVCRNDLKWIVRSKEKNSDKALEEVFDAVVVATGHYSQPKLPSIKGMDTWKRKQMHSHIYRTPEPFHNEVVVLVGNSFSGQDIAIELVGVAKEIHISSRSLSSITEGLSKVISKHDTLHLHPQIETLEEDGRVIFKDGSWLKADSILYCTGYSYSFPFLDTKGMVVVDDDRVGPLYEHTFPPSLAPSLSFIGIPKKILGFPFFESQAMWVAQLLSGKKALPSWDEMMKSIKEFYHSREVACIPTHDIADFEYCDKYGENVGLPPLQEWRKVLCIKSIVNSIVNLETYRDSWDDDELLQEALKSPHFTQLGIED

>Gohir.D12G182900|Ghirsutum_v3.1/1-458

MVSEQGQSTKNVCVIGAGPSGLVAARELRKEGHTAVVLEQNHDIGGQWLYEPNVEKEDPLGKNKFLDVHSSVYDSLRIVSPREIMGFTDFPFVSKKNRDTRRFPGHKELWLYLKDFCEYFGLKEMIRFNTRVEYVGMLDYGVFGKDLKWVVKSKEKKGEKRVEEVFDAVIVATGHYSQPRLPSIKGMDAWKRKQIHSHVYRVPEPFRNEVVVIVGNSLSGQDISMEIVKVAKEVYLSAKSLEVITEGLSKVISKHRNLHLYPQIQSLHEDGRVEFEDGSCVIADTIIYCTGYSYAFPFLDTKGIVGVDDNRVGPLFEHTFPPSLAPFLSFIGIPRKLIGFPFFESQAKWIAQVLSGKRELPSYDDMMLSIKEFYRSRELAGIPVSDTHDIANFEYCDKYADYSESPHLEEWRKQLCLSAIVNSFTNLETYRDSWDDGDDELLQQALQSPHFTQLGVQP

>Gohir.A12G180200|Ghirsutum_v3.1/1-457

MVSEQGQATKNVCVIGAGPSGLVAARELRKEGHTAVVLEQNHDIGGQWLYEPNVEKEDPLGKNKFLNVHSSVYDSLRIVSPREIMGFTDFPFVSKKNRDTRRFPGHKELWLYLKDFCEYFGLKEMVRFNTRVEYVGMLDYGVFGKDLKWVVKSKEKKGEKRVEEVFDAVIVATGHYSQPRLPSIKGMDAWKRKQIHSHVYRVPEPFRNEVVVIVGNSLSGQDISMEIVKVAKEVYLSAKSLEVTEGLSKVISKHRNLHLYPQIQSLHEDGRVEFEDGSCVIADTIIYCTGYSYAFPFLDTKGIVGVDDNRVGPLFEHTFPPSLAPSLSFIGIPRKLIGFPFFESQAKWIAQVLSEKRNLPSYDDMMLSIKEFYRSRELAGIPVSDTHDIANFEYCDKYADYSESPHLEEWRKQLCLSAIVNSFTNLETYRDSWDDGDDELLQQALQSPHFTQLGVQP

>Acora.06G081600|Aamericanus_v1.1/1-455

MVSENAYSKHICVIGAGPSGLVAARELRKEGHTVVVLEQSHDIGGQWLYDSRIENEDALGKAIPLKVHSSVYASLRLVSPREIMGFTDFPFLVKKGRDVRSFPGHKELYLYLRDFCDCFGLRELVRFNTRVEYVGMVDVSEFGKNMKWVVKSSERGGKVVVEEIYDAVVVATGHYSQPRLPTIKGMDEWRRRQIHSHVYRVPEPFANEVVIIVGSSLSGQDISLELVGVSKEVHLSAKSLEVPEGLSKVISKYDNLNFHPQIQSLNEDGRVTFVDGTCVVADSIIYCTGYSYSFPFLDTKGIVMVDDDRVGPLYEHTFPPSLAPSLSFIGIPRKIIGFPFFESQAKWIAQVLSGKRTLPPWDEMMKAIKEFYQSRNAAGIPKHNTHDICDFEYCDKYGENCGFPHLEDWRKELCLSALRSALINLETYRDTWDEQELLQMAHQSNHFTQLGPENI

>Eucgr.B00559|Egrandis_v2.0/1-465

MACNEPAVQSKHVCVIGAGPSGLVAARELRKEGHSVVVLEQNHDIGGQWLYEPNVEGEDPLGREPFLRVHSSMYKSQRFVWPREIMGFTDFPFLVKKDRDMRRFPGHRELLLYLQDFVDWFGLRELIRFNTRVEHVGMVNYGEYGKDLRWVVKSKEITTKGDDDNDEKMVEEVFDAVVVATGHYSRPRMPSIKGMGTWKKKQFHSHIYRVPEPCRDEVVVVVGNFVSGQEISMELAEVAREVHLSAKSLDITGGQSKVLDKHRNLHLRPRIDSLEEDGTVSFIDGSSVIADTILYCTGYSYAFPFLDTKGIVVVDDDRVGPLYEHTFPPSLAPSLSFIGIPRKIISFPFFESQAKWIAQLLSGKRTLPTWNEMTQSINDFYSSIDAAGVPKRHTHDIDNFEYCERYGDNVGFPHLEEWRKGICTSTIKNIEANLETFRDSWDEADEKLLQEALQSLTSLNSDHKS

>Eucgr.B00562|Egrandis_v2.0/1-474

MVSDKPGIRSKHVCVIGAGPSGLVVARELRKEGHSVVVLEQNHDIGGQWLYEPDAEGEDPLGRDPFLKVHSSMYESLRLLSAREIMGFSDFPFLVKRDRDMRRFPGHREVLLYLQDFVEWFGLRELIRFNIRVEHVGMVDYGEFGKDLRWVVKSKEIITKSDDDDEKMVEEVYDAVVVATGHYSHPRMPSIKGMGTWKRKQFHSHIYRVPEPYRNEVVVVVGNSFSGQDISMEIAGVAREVHLSAKSLDITEGLSKVLDKHHNLYLRPQIDSLEEDGTVLFVDGSSVIADTILYCTGYSYSFPFLDTKGIVVVDDDRVGPLYEHTFPPSLAPSLSFVGIPRKIIGFPFFESQAIWIAQLLSGKRTLPPWNEMMQSIKDFYNSVDAAGLPKHNTHDIANFEYCERYGDHVDFPHLEEWRKELCISTLKNLVVNLETFRDSWDEGDEKLLQEAFQSPHFTQLGSQVLTFQSQSERP

>Aco004870|Acomosus_v3/1-459

MVSEKRQPKHVCVIGAGPAGLVSARELRKEGHSVVVLEQKHDIGGQWLYDPIVEIEDALGRITPLKVHSSMYASLRLMSPREVMGFTDFPFFTKKGRDMRRFPGHREVYLYLRDFCQWFELRELIKFNTRVEYVGMVYNIEFGSDMKWVVRSKEEKCETVNEEIFDAVVVASGHYSQPTMPTIKGIDTWKRNQIHSHVYRVPEPFRNEVVVIVGGSFSGQDISLELVDVAKEVHLSVKSLELSEGLTKVITKYQNLHLHPQIDNLFEDGRVLFIDGSCVVADSIIYCTGYSYSFPFLDTKGIVVVDDGRVGPLYEHTFPPSLAPSLSFVGIPKKILGFPFFESQAKWISQVLSGRRTLLSRDDMMKSLEEFYHSREIAGIPKHCTHDIADFDYCDKYGDHCDFPHLEDWRKVLCITALRNSIMNLETYRDQWDDHIDELLQETLESPHFTQLDPEAIPS

>evm.TU.AsparagusV1_03.2031|Aofficinalis_V1.1/1-457

MVSEKTNSKHVCVIGAGPSGLVSARELRNEGHFVVVLEQNHDVGGQWLYDHRVEHEDALGRLNPLRVHSSIYNSLRLTSPREIMGFTDFPFVAKKGRDMRRFPGHNELLLYLKDFCQRFGLRELIRFNTRVEHVGMVDIPEFRKGMKWVVRFKNTKSEKAKEEIFDAVVVATGHYSQPRLPSIRGMDRWTRKQIHSHVYRVPKPFHNEVVVVVGNSYSGRDISLELVDVAKEVHLSAKSLQISEGLSKVISKYQNLHIHLQIDSLCEDGRVLFADGSWVIADSIIYCTGYSYSFPFLDTNGIVTIDDNRVGPLYEHTFPPSLAPSLSFVGVPRKILGLPFFESQAKWIAQVLSGRRKLPSSDAMMRAVEEFYHSREVAGIPKHNTHDIADFEYCDKYGDNCGFSHLEDWRKELCTAAIRRSIVNLETYRDTCDDHELIQLAHQSPHFTQLDPDVLAL

>Asa4G04549.1|Allium_sativum/1-456

MVSEYINSKHVCVIGAGPSGLVSARELRKEGHSVVVLEQNFDVGGQWLYEPNIEKEDTLGRKTPLKVHSSVYASLRLNSPREIMGYTDFPFHSKKGRDTRRFPGHAELLSYLRDFCLRFRLREMIRFNTRVEYVGMTNTRDFRNLKWIVRSKELERGVVKEEIFDAVVVANGHYSKPRLPSIKGMDTWTRKQIHSHVYRVPGPFRNEVVVVIGNSMSGQDISLELVGVAKEVHISTKSLDISEGLSKIIDKYQNLHIHLQVDCLYGDGKVLFVDGTWVIANSIIYCTGYSYSFSFLDTKGIVSIDDDRVGPLYEHTFPPSLAPSLSFVGIPRKLIGFPFFESQAIWIAQVLSDKRKLPSWDTMMRAITEFYRLKDDAGIPKHNTHDLADFEYCDRYGDNCGFPHLEEWRKELCLSALKNAEMNLESFRDIFEDLELLQEAHKSPHFMQLDPDVIKI

>Asa4G04544.1|Allium_sativum/1-465

MVSEYINSKHVCVIGAGPSGLVSARELRKEGHSVVVLEQNFDVGGQWLYEPNIEKEDALGRKTPLKVHSSVYASLRLNSPREIMGYTDFPFHSKKGRDMRRFPGHAELLSYLRDFCLRFRLREMIRFNTRVEYVGMTNTRDFRNLKWIVRSKELERGVVKEEIFDAVVVANGHYSKPRLPSIKGMDTWTRKQIHSHVYRVPGPFRNEVVVVIGNSMSGQDISLELVGVAKEVHISTKSLDISEGLSKIIDKYQNLHIHLQVDCLYGDGKVLFVDGTWVIANSIIYCTGYSYSFSFLDTKGIVSIDDDRVGPLYEHTFPPSLAPSLSFVGIPRKLIGFPFFESQAIWIAQVLSDKRKLPSWDTMMRAITEFYRLKDDAGIPKHNTHDLADFEYCDRYGDNCGFPHLEEWRKELCLSALKNAEMNLESFRDIFEDLELLQEAHKSPHFMQLDPDVIKYEDKQIQALL

>AUR62008483|Cquinoa_v1.0/1-457

MVVESYQSKSVCVIGAGPSGLVVARELMKEGHNVAVIEQNHDIGGQWLYEPKVEGEDPLGKANKFLKVHSSMYESLRLASPREIMGYTDFPFVEKKGRDMRRFPSHREVLLYLNDFCEYFGLRGMIRFNTRVEYVGMAEFAEVAKELKWMVKSKEKKSEKLVEEVFDAVVVASGHYSHPRLPIIKGMNSWKRRQMHSHIYRIPDPFRDEVVVIVGSSMSGQELSMELVEVAKEIYLSAKSPISEGFSKVFANHKNLHLCPKIDCLEEDGRVLFEDGSCITADTIIYCTGYSYTFPFLDTKGIIEVDDDRVGPLYEHTFPPSLAPSLSFVGIPRKIIGFPFFESQAKWIAQLLSGKKTLPSWEEMMKEIKDFYQSREAAAIPKHNTHDIANFEYCDLYADKVGFPRLEEWRKELCVSALMNAEANLEVYRDVYDDFELLQVARQSPHFTQLGAHTFEV

>AUR62021610|Cquinoa_v1.0/1-457

MVVESYQSKSVCVIGAGPSGLVAARELMKEGHNVVVIEQNHDIGGQWLYERKIEGEDPLGRANKFLKVHSSMYESLRLASPREIMGYTDFPFVEKKGRDMRRFPSHREVLLYLNDFCEYFGLRGMIRFNTRVEYVGMAEFAEVAKELKWMVKSKEKKSEKLVEEVFDAVVVASGHYSHPRLPIIKGMNSWKRRQIHSHIYRVPDPFRDEVVVIVGSSMSGQELSMELVGVAKEIYLSAKSPVSEGLSKVFAKHKNLHLCPKIDCLEEDGRVLFVDGSCVTADTIIYCTGYSYTFPFLDTKGIIEVDDDRVGPLYEHTFPPSLAPSLSFVGIPRKIIGFPFFESQAKWIAQLLSGKKTLPSWEDMMKEIKEFYQSREAAAIPKHNTHDIADFEYCDLYADKVGFPRLEEWRKELCVSALMNAEANLEVYRDVYDDFELLQVARQSPHFTQLGAHTFEV

>Sapof.10G215600|Sofficinalis_v1.1/1-460

MVTTSMSQGKKVCVIGAGPSGLVTARELRKEGHNVVVMEQNHDIGGQWLYESKTEGEDPLGRASTFLRVHSSMYESLRLQSPREIMGYTDFPFVEKKGRDMRRFPSHREVYLYLKDFCEFFELRGLIRFNTRVEYVGILDDFRWVVKTKEVVKGGGCCGGDKVVEEVFDAVVVASGHYSHPRLPTIKGMDKWKRRQMHSHVYRVPEPFKNEVVVIVGCSLSGQDISMELAGVATHVYVSVKLGHVTPGFAKIVAKHHNLHLRPQIECVEEDGRILFVDGSWIIADTIIYCTGYSYTFPFLDTKGIVNVDDDRVGPLYEHTFPPSLAPSLSFVGIPRKLIGFPFFEAQGKWVAQLLSGKRRLPTWEEMMKSINEFYQSKDDAGIPKHNTHDLASFEYCDKYGDNIGFPHLEEWKKQLCLSALQNAEDNLETFRDVYDDFELLQIAHQSPHFTQLGPEAFES

>Glyma.05G023800|Gmax_Wm82.a6.v1/1-457

MASETNQSKNVCVIGAGPSGLLAARELRKEGHKVVVLEQNHDIGGQWLYDPNVQEEDPLGRDPWLKVHSSIYESLRFMSPREIMGSTDFPFLVKKGRDTRRFPSHTEFLLYLKDFCEWFKLSEMIKLNTKVHYVGPLNYGVPSEDLKWVVRSKENKSEEEVEQVFDAVVVATGHFSNPRLPCIQGMDTWKRKQMHSHIYRSSEPFRGEIVVVVGNFLSGQEISMELVKVAKEVHLSSKSLIIFEGLSKVISKNENFHLRPQIDTLQEDGTVIFTDGSNIIADTILYCTGYNYSYPFLDTKGIVVVDDNRVGPLYEHTFPPSLAPSLSFVGIPRKILGITFFESQGKWIAQLLSGKKVLPSYEEMMESIEEFYHSNEVAGIPKRYTHVVDAFDYCDKYGENVGFPKTEEWRKELCISAGMNAFANLETYRDSWNDDIDEKLQEALRSPYFTQLEQLSL

>Glyma.05G023700|Gmax_Wm82.a6.v1/1-461

MVSETNQSKNVCVIGAGPSGLVAARELKREGHKVVVLEQNHDIGGQWLYNPNVQEEDPLGRDPWLKVHSSIYESLRLMSPREVMGFTDFPFLVKKGRDPRRFPSHRELLLYLKDFCEWFELRDMIKFNTKVHYVGPLNYGVPSEDLKWVVRSKDKNSEEEVEQVFDAVVVATGHYSNPRLPCIQGMAIWKRKQMHSHIYRSPEPFRGEIVVVVGNSFSGQEISMELVKVVKELHLSSKSLDIYEGLSKVISKHENFHLRPQIETLREDGTVIFNDGSSIIADTILYCTGYTYSFPFLDTKGMVVVDDNRVGPLYEHTFPPALAPSLSLVGIPRRILGLPFFESQGKWVAQLLSGKKALPSYEEMMKSIKEFYHSKEEAGIPKHYTHEIEGFDYCDKYAENVGLPKLQEWRKDLCVSAVVNLFINLETYRDSWNDDIDEKLQEALRSPYFTQLGVEPEDPSL

>Glyma.05G023601|Gmax_Wm82.a6.v1/1-164

MVVVDDNRVGPLYEHTLPPLLAPPLSFIGIPKKILGLPFFESQGKWIAQLLSGKKALPSCEEMMKSIEEFYHSKEVVGIPKRFTHEIGAIGFEYCDKSAKYVGLPKLQEWRKELCILSLVNSCVNLETYRDSWNDDEKLQEALQSPYFTQLGVELEDPSLQNSN

>Brara.B00261|BrapaFPsc_v1.3/1-459

MVSVTLSEASRSRSKKVCVIGAGPSGLVSARELRKEGHKVVVMEQNNDVGGQWLYQPNVKEEDPLGSVPTNGALKVHSSIYSSLRLTSPREIMGYSDFPFLAKKGRDMRRFPGHKELWLYLKDFSEAFGVREMIRFNVRVEFVGEEDKNEDVKRWIVRSKEKLSGKVTEEIFDAVVVATGHYSHPRLPSIKGMDSWKRKQVHSHVYRVPDPFRNEVVVVVGNSMSGQDISIELVEEAKEVHLSSKSLDITSGLSKVISKYPNLHLQPQIECLKDDGRVIFVDGSWVVADTILYCTGYSYKFPFLESKGRVEVDDDRVGPLFEHTFPPPLSPSLSFVGIPRKLIGFPFFEAQAKWIAQVLSGKLSLPSSDQMLQSVADFYRSRDLAGVPKHNTHDIADFTYCDKYADYVGFPHLEEWRKLLCLSALDNSQENLETYRDCWDDHELLQEAMQSSHFTNFNS

>AT5G07800|Athaliana_Araport11/1-460

MVTFTSEASRSRSKKVCVIGAGPAGLVSARELRKEGHKVVVLEQNEDVGGQWFYQPNVEEEDPLGRSSGSINGELKVHSSIYSSLRLTSPREIMGYSDFPFLAKKGRDMRRFPGHKELWLYLKDFSEAFGLREMIRFNVRVEFVGEKEEEDDVKKWIVRSREKFSGKVMEEIFDAVVVATGHYSHPRLPSIKGMDSWKRKQIHSHVYRVPDPFRNEVVVVVGNSMSGQDISMELVEVAKEVHLSAKTLDISSGLSKVISKHPNLLIHPQIESLEDDGKVIFVDGSWVVADTILYCTGYSYKFPFLESKGRIEVDDDRVGPLFEHTFPPCLSPSLSFVGIPRKLIGFPFFEAQAKWIAQVLSGKSSLPSPDQMLQSVDEFYRSRDLAGVPKHNTHDIADFTYCDKYADYVGFPHLEDWRKLLCLSALNNSQENLETYRDSWDDHELLQEALQSSHFTNFNS

>Brara.C04244|BrapaFPsc_v1.3/1-460

MVLVISEPIKSQSKTVCVIGAGPSGLVSARELKKEGHKVVVMEQNDDVGGQWLYQPNVEEEDPLGKTKALKVHSSIYSSLRLISPREVMGFSDFPFTVKEGRDSRRFPGHEELWLYLKDFCEVFGLREMIRFNVRVEFVGMVDDDKDVDDNMKRWMVKSVAKKTGEVIEEVFDAVVVASGHYSYPRLPSIKGMDSWKRKQLHSHIYRVPDPFRDEVVVVVGCSMSGQDISIELVEVAKEVHLSAKSLDVPPGLSKVISKHHNFHLHLEIESLEEDGRVIFVDGSCIVADTILYCTGYWYKFPFLESKGRVEVDDNRVGPLFEHTFSPFLSPSLSFVGIPRKLIGFPFFESQAKWIAKLLSGKTSLPSFDEMMESISEFYLAREAAGIPKRNTHDICDFNYSDKYADYIGFPHLEEWRKELCMSALLNSIENLDTYRDSWDDDDLLQETLQNPYFTQFTSP

>AT5G61290|Athaliana_Araport11/1-461

MVLVITEPIKSQSKTVCVIGAGPSGLVSARELKKEGHKVVVMEQNHDVGGQWLYQPNVDEEDTLGKTKTLKVHSSVYSSLRLASPREVMGFSDFPFIAKEGRDSRRFPGHEELLLYLKDFCQVFGLREMIRFNVRVEFVGMVNEDDDDDDDVKKWMVKSVKKSGEVMEEVFDAVVVASGHYSYPRLPTIKGMDLWKRKQLHSHIYRVPEPFCDEVVVVVGCSMSGQDISIELVEVAKEVHLSTKSLDIPPGLSKVIEKHQNLHLHPQIESLEEDGRVIFEDGSCIVADTILYCTGYEYKFPFLESKGRVEIDDNRVGPLFEHTFSPSLSPFLSFVGIPRKLIGFPFFESQAKWIAKLLSGKTSLPSSDQMMQSISDFYLAREADGIPKRNTHDIADFNYSDKYADYIGFPHLEEWRKVLCLSAILNSIENLETYRDSWDDDDLLQETLQDPYFTQLSIPSV

>Brara.C04245|BrapaFPsc_v1.3/1-462

MVLETTKSEEPIMSQSKTVCVIGAGPSGLVSARELKKEGHKVVVMEQNDDVGGQWLYQPNVEEEDSLGKTKILKVHSSIYSSLRLISPREIMGFSDFPFTVKEGRDTRRFPGHEELWLYLKDFCEVFGLREMIRFNVRVEFVGMVNDDDITKRWRVKSVEKKSGEVIEEVFDAVVVATGHYSYPRLPSIKGMDLWKGKQVHSHVYRVPDPFRDEVVVVVGGFISGPDISIELVKVAKEVHLSVKSLDVSPGLSKAITKHQSLHLQPQIESLEEDGRVIFVDGSCIVADTILYCTGYRYKFPFLESQGRVEVDDNRVGPLFEHTFSPSLSPSLSFVGIPQKLIGFPFFESQAKWIAMILSGKTSLPSYDEMMQSVAEFYRAKEANGVPKRNTHDIGRDFVNYCDKYADYTGYPHLEEWRKKLCVTTILRSLDNLETYRDSWDDDHELLQEALQDPYFAQRTTP

>XP_020587142.1|Phalaenopsis_equestris/1-461

MVSENLSSKRVCVIGAGPSGLVSARELRKEGHCVSVLEQNHDIGGQWLYQPEVEDEDSMGKKKPLKIHSSVYSSLRLASPREIMGFTDFPFTVRKGRDMRRFPGHEELFLYLHDFCNWFQLREMIRFNTRVEYVGIAEGSAFEFGDQGIRWKVRSREMENGAMIEEIFDAVVVANGHYSVPRLPVIRGMDEWRRRQLHSHVYRVPQPFSNEVVVMVGNSMSGQDISMELVNFAKEVHLSAKSLEIFQGLCKVIAKYQNFHLKPQIDTLYEDGRVMFVDGTVITADTIIYCTGYSYYLEFLDTKGIVDVDDDRVGPLYEHTFPPSLAPSLSFVGIPRKIIGFPFFESQAKWIAQVLSRRRTLPSCEEMMKAIKEYYNSRDMDGIPKHNTHDIANFEYCDKYGDKCDFPHLEGWRKELCLSSLLNAQMNLETYRDVWDDDLQLLQEAHQSPHFSHLDYENVVV

>GWHPCBIR021229|Santalum_yasi/1-461

MNLTTNIIPHHKKKRVCVIGAGPSGLVAARELHRVGHTAVVLEQNEDVGGQWLYQTTVAEEVHSSMYASLRLASPREIMGFTDMPFAVKRGGEGRDPRRFPGHQELLLYLREFCDRFGIREVIRLNTRVEYVGMVGYGGAPDDLRWVVRSRDRRSQAVEAAEVVEEVFDAVVVATGHYSHPRLPAIKGMDAWRRKQLHSHVYRVPDPFRDQVVVVVGNSLSGQDISMELVEIAKEVHLSSKSLSVSEGLSKVISKHHNLHLRPQIECLHEDGCVSFLDGTWIIADTIIYCTGYSYSFPFLDTKGIVVVDDDRVGPLYEHTFPPSLAPSLSFIGIPRKIIGFPFFEAQAKWIAQLLSGEKTLPSWDEMMQSIKEFYHSRDVAGIPKHNTHDIANFEYCDKYADLVGFPRLEEWRKELCVSSLRNAESNLETYRDSWDEDIELLQVAHQSPHFTQLEPQTIPK

>FvH4_2g15410|Fvesca_v4.0.a2/1-474

METTTMCESYQSKSVCVIGAGPSGLVAARELKKEGHRVVVLEQNHDVGGQWLYDPNVEGEDPLGRNTTSLTTTHNSHHKVHSSVYTSLRLGSPREIMAFTDFPFSVKKGRDSRRFPGHRELLLYLQDFCDWFGLRDFIRFNNKVAYVGMLDHVEGCQDLRWLVKSVEKNTQIVMEEVFNSVVVATGHQYQPRLPSIKGMDSWKRKQLHSHIYRVPEPFRDEVVVVVGTAQSGQDISVELVQEAKAVVYLSARSLSNIFEGLSKVISKHDNLHLRPQIECLQQDGKVLFVDGSWVIADTIIYCTGYSYTFPFLDTKGIVAIDDDRVGPLFEHTFPPSLAPSLSFIGIPRKVIAFHFFESQAKWIAQVLSGKRKLPSRDEMMQSIKDFYHSRDVAGIPKYQTHDIGEFEYCDKYADYTGFPHLEEWKKKIFFIILNNYFTNLETYRDSLDDDDLIKEALQSPHFIEQEQDAQVSPL

>Kalax.0013s0042|Klaxiflora_v1.1/1-465

MVSDQRSQSKTVCVIGAGPSGLVAARELRRELHKVVVMEQSHDIGGQWLYSSDTRDEVPLARYFSTDSVVVPRVHSSVYASLRLTSPREIMGYTDFPFVAKKGRDMRRFPGHRELFLYLKDFCEWFGLRELIRFDTKVEYVGMADNGCRIGEDLKWVVRSRAVAAPDATVVEEIFDAVVVATGHYTQPRLPKIKGMDSWRRRQIHSHTYRVPIPFRDEVVVIVGNSLSGQDISMELVEVAKQVHLSSKTLDVTEGLSRVISKHKTLHLHPQIDRLCDDGRVVFVDGSCVEADTIIYCTGYSYSFPFLDAQGIITVDDDRVGPLYEHTFPPSLAPSLSFIGIPRKLIGFPFFEAQAKWVAQLLSGKKTLPSYEDMMVSIKEYYHSRDAAGIPQHYTHDIANFEYCDRYCDLAGFPRLEQWRKDLCISALENAAANLETYRDLHDDAEVLQKAMRSAHFTQIIETDL

>Kalax.0120s0024|Klaxiflora_v1.1/1-465

MVSDQRSQSKTVCVIGAGPSGLVAARELRRELHKVVVMEQSHDIGGQWLYSSDTHDEVPLARYFSTDGVVVPRVHSSVYASLRLTSPREIMGYTDFPFVAKKGRDMRRFPGHRELFLYLKDFCEWFGLRELIRFDTKVEYVGMADNGCRIGEDLKWIVRSRAVAAPDATVVEEIFDAVVVATGHYSQPRLPKIKGMDSWRRRQIHSHTYRVSIPFRDEVVVIVGNSLSGQDISMELVEVAKQVHLSSKTLDVTEGLSRVISKHKTLHLHPQIDRLCDDGRVVFVDGSCVEADTIIYCTGYSYSFPFLDAQGIITVDDDRVGPLYEHTFPPSLAPSLSFIGIPRKLIGFPFFEAQAKWVAQLLSGKKTLPSYEDMMVSIKEYYHSRDAAGIPQHYTHDIANFEYCDRYCDLAGFPRLEQWRKDLCISALENAAANLETYRDLHDDAEVLQKAMRSAHFTQIIETDL

>DCAR_729889|Dcarota_subsp_sativus_v3.0/1-448

MVTQSYLSRNVCVIGAGPSGLVAARELRKEGHKVVVIEQNNDVGGQWLYDPNVEAEDPLGKCTTLKVHSSVYDSLRLQSPRETMGFSDFPVLVKDGRDTRSFPGHRELLLYLQDFCERFELREMIRFSTRVENVRMLNYGDQFGKDLIWVVKSIDTKNEDVLEEEFDAVVVATGHYSHPRLPPIKGIDGWTRKHLHSHVYRVPEPFRNEVVVVVGNSFSGQDIAMELLQVAKEVHLSSKSLDICEGLSKVISKHDSLHLHLEIESLHEDGRVVFVDGSCLTADTIIYCTGYRYTLPFLDSKGIITVDDGRVGPLYEHTFPPSLAPSLSFIGIPKKIIGFPFFESQAKWIAQLLSGKITLPSRDDMMQSIKEFYHEKDLAGIPKHNTHDIADFEYCDKYADFAGSPHVEEWKKELCIAALVRAEVDLEMYRDFCYEDYELLHQNSTLAN

>DCAR_729890|Dcarota_subsp_sativus_v3.0/1-448

MVAESYLTKNVCVIGAGPSGLVAARELRKEGHKVVVIEQNHDVGGQWLYDPHVEAEDPLGKCTTLKVHSSVYDSLRLQSPRETMGFSDFPVLVKDGRDMRSFPGHRELLLYLQDFCECFELREFIRFSTRVENVRMLNYGDQFGKDLIWVVKSIDIKNEEVLEEEFDAVVVATGHYSHPRLPPIKGIDGWTRKHLHSHVYRVPEPFRDEVVVVVGNSFSGQDIAMELLQVAKEVHLSSKSLDICEGLSKVISKHDSLHLHPEIDSLHEDGRVVFVDGSCLTADTIIYCTGYQYTLPFLDSKEIITIDDGRVGPLYEHTFPPSVAPSLSFMGIPTRIIGFPFFESQARWIAQLLSGKLTLPSQDDMMQSIKEFYREMDVAGIPKHSTHDIGDFEYCDKYADFAGSPHVEEWKKELCLAAVMRAQVDLEMYRDSSYEDYGLPHQNSKLAN

>DCAR_417675|Dcarota_subsp_sativus_v3.0/1-451

MGTESYQSKNVCVIGAGPSGLVAARELQKEGHDVVVLEQKHDVGGQWLYEPNVEGEDPLGKCTTLKVHSSVYNSLRINLARETMGFTDFPFFVKEGRDARRFPGHRELLLYLQDFCECFGLREMIRFYTRVENVKFFESTDPFSEDLRWVVRSIDYKNEKVVEEVFDAVVVATGHYSHLRLPTIQGKDGWRKKHLHSHIYRVPEPFRNEVVVVVGIANSGQDIAMELVEVAKEVHLSAKSVDICEGLSKVISKHDNLYLHLEIDSLHEDGRVVFVDGSCIAADTIIYCTGYTCSFPFLDIGGIVAVDDGRVGPLYEHTFPPLLAPSLSFVGIPKKVIAFPFFEWQAKWIAQLLSGKKSLPSRADMMRSVEEFYELKDHAGVPKRNTHDIGNFEYCEKYADLVGVPHLEEWRKELCMAAIKKSEVDLETYREYSYDRELLQVPHHYLSPKPL

>VIT_213s0019g00620|Vvinifera_v2.1/1-456

MAQSARVAVIGAGVAGLAAARELHREGHHVVVLEKRHSLGGTWLYDSRVDSDPLSLDPARDVVGTSLYHSLRTNLPRQLMGFLDYPFAKRPHEDQRTFPGHEEVLRFLNEFADEFRLRELIRFCTEVVRVERAERRNDAWVVESRNHGSDSAVVVREVYDAVVVCNGHFTEPQLAEVPGIEKWCEYQIHSHNYCTPKSFMGQVVVLIGLGPSAFDISREVATVAKEVHITTRAPNVTVGKSDNHENIWLHKMIEFVYEDGKVVFQDGSSVHADTIFYCTGYKYHFPFIETNGIVTIDDDNRVGPLYKHVFPPHLAPWLSFIGMPKQDTPFLTTELQSKWLAHVLSGKVLLPTEEEMMSDVENYYHHMEETGVPKSFTHVLPPNEIEYRNWLLAQVEMPPLKEWRGRMYRECVKFAKAKLDGYRDQWDDDYWDAVIASQEVNHLANGQDVGVAEDVA

>VIT_213s0019g00660|Vvinifera_v2.1/1-459

MALLARVAVIGAGVAGLAAARELHREGHRVVVLEKRHSLGGTWLYNSRVDSDPLSLDPARAVVGTSLYHSLRTNLPRQIMGFLDYPFAKRIHGDQRTFPGHEEVLWFLNEFADEFQLRELIRFCTEVVRAERAEGRNDEWVVESRSRGSESAKVVREVYDAVVVCNGHYTEPHLVEVPGIEKWRGYQIHSHNYRIPKPFTGQVVVLIGFGPSAFDISRDVATVAKEVHIATRAPNVTVGKLDNHKNIWQHEMIEFVDEDGKVVFQDGSSVHADTIFYCTGYKYHFPFIETNGIVTIDDNRVGPLYKHVFPPRLAPWLSFIGMPKQGTPFMTAELQSKWLAHVLSGKALLPTKEEMMSDIEKYYHHMEETGVPKRFTHVLPPNEIEYMNWLLAQVGMPPLKEWTGRMYKECAMLAKAKRDGYKDQWDDDYWDAVIASQVDSIIFTMIKREYKRILKPFTL

>Litul.05G117300|Ltulipifera_YP108A_v1.1/1-440

MARSCMVAVIGAGASGLVAARELLREGHRVVVFEKSDRIGGTWVYDPRVESDPLGLDPSREIVHGSLYLSLRTNLPRQIMSFLDFPFTATRNGDSRLFPRHEEVLAYIEDFTAEFGLLGLIRFGAEVVRVSSVEMRNDEWVVEWRSQGSGSVPESEIFKAVVICTGIYTQPRVAEIPGIDKWPGKQIHSHNYRIPDPFLDQIVVLIGNGPSGQDICRDISKLAKEVHLTSRSIDVEVAKLEGHDNIWQHPMIKSVHEDGTVLFHDGLSFFVDVIFHCTGYNYHFPFLETNGIVNVDDNRVGPLYKHVFPPQLAPWISFVGITRRAIPFLTFELQSKWVAHVLSGKVVLPTEEEMLGSVEEFYMRMEEAGKPKHHTHILHPFEFEYKNWLIAQLGLPPIDESIEQMYIATITSLLSHFDGYRDQWDDGFQTQQSHCALERE

>Litul.05G117200|Ltulipifera_YP108A_v1.1/1-440

MASSCKVAVIGAGVSGLVAARELVREGHRVVVFEKSDRIGGTWVYDPRVESDPLGLDPSREIVHGSLYLSLRTNLPRQIMGFLDFPFTATRNGDSRTFPRHEEVLAFIEDFAEEFRLLELIRFGAEVVRVSSVEMRKDEWVVEWRTQGSDSIPESETFEAVVVCTGHHTEPRVAEIPGIDKWPGKQIHSHNYRIPDLFLNQIVVLIGKGPSALDISRDISKLAKEVHLTSRSMDVEVVKLEGNDNIWQHSMIKSVHKDGLVLFHDGSSVFADVIFHCTGYNYHFPFLETNGIVHIDDNRVGPLYKHVFPPRLAPWLSFVGIPNKVIPFLTSELESKWVAHVLSGKVVLPTEEEMLASVEELYVRMEAAGRPKHYTHFLYPFESEYKNWLVDQLSLPPLQESIEQMYVASVTSLTSHSDGFRDKWDDGNWTQQSHSAPESE

>Glyma.13G200400|Gmax_Wm82.a6.v1/1-439

MHRSLKVAVIGAGVSGLAAARELRHEGHNVVVLEKSNHVGGMWAYDPKTDSDPLGLDPTRETVHSSLYLSLRTNLPRQLMGFSDFPFVKNESGDPRTFPGHEEVLRFLEGFAGEFGINELTQFETEVVRVERKGNEWVVESRTSRDGDSVSREGFDAVVVCSGHFVEPKLAEVPGIDTWRGFQMHSHNYRVPQPFHNQVVILIGLGPSAFDISREIAQVAREVHVATRLNPDLAGMKLGDYGKIMFRTAVKCVFEDGLVAFEDGFSVYADAIIHCTGYKYHFPFLETNGLVTVDDNRVGPLYKHVFPPALSPWLSFIGLTFKNSVYQIIELQCKWVAKVLSGKVLLPTEKEMMESVKEYYQLMEENGLPKRYTHSLYPLQADYKHWLVAEIGLPPLEEWKENMLKQCFKNFVEMNEKYRDEWDDTYWDAIIHGAPASEK

>KAI5421251.1|Pisum_sativum/1-448

MATRSVKVAVIGAGVSGLIAAKELHQQGHTVVVFEKNNRIGGTWIYDPKTDSDPLSIDPTREIVHSSVYLSLRTNLPRPVMGFLDFPFAKRESGDPRTFPGHEEVLRFLEDFAGEFGIYGLTRFETEIVKVERKGKGNEWVVESRMTRGSDSVSREVFEAVVVCSGHFVEPKLGVVPGIENWRRFQMHSHNYRVPHSFKDQVVILIGLGPSSVDISRDIADVAKEVHVATKPNPQLKDIKMENVRNISFHTLIECVHEDGLVTFEDGFSIYADAIIHCTGYKSHIPFLETNGIVTIEDNRVGPLYKHVFPPSLAPWLSFIGLTFKEIIFNVIELQAKWVARVLSGKILLPTEEEMTESVKKLYQFMEENALPKHFTHSLRPFQAEYKEWLVAQIGLPPLEDWRDNMLMECYKNFATKEMYRDEWDDNYWDSIIHQSGLAFEKAKILNV

>Glyma.11G153100|Gmax_Wm82.a6.v1/1-443

MTHPLRVAVIGAGVAGLAAARSLRREGLDVVVFEKSNHLGGTWSYDPRTDSDPVGLDPNREVVHTSLYRSLRTNLPRQLMGFLDYPFPNRNNGDPRTFPGHEEVLWFLNRFSDEFGLRGLTRFGSEVVRVELVSEKSDSWVVESRRNRDSVLTREVFGAVVVCTGHFTQPRLPTIPGIEKWPGYQIHSHNYRVPEPFQGQIVVVIGFASSAFDISREIAKVAKEVHIATRSPDVKVMKLANHDNMWQHKMVKCVSEDRLVAFDDGSSVYADVLFYCTGYKYHFPFLETNGIVTIEDNRVGPLYKHVFPPALAPWLSFIGIPEKEVIFQMTELQCKWVARVLSGKVLLPTEKEMMAYVEEYYQQMEKDGFPKHMTHYLHFKEIGYCNWLAAKAGLPPIEHWRDAMYLESIKPVLLGLQDNYRDQWDDAHWKAIIKDASLPQNVN

>XP_020593959.1|Phalaenopsis_equestris/1-445

MPKSCSVAVIGGGVSVSGLCAARELLREGHRVVVFEKTNRIGGTWVYNPYTDSDPSGLDPARRVAQGNLYRSLRTNIPRPLMGFSDYPFPDPEASFGDPRAFPGHGEVLAFVETFAREEGLLDVVRLGVEVVRVEQVGERKDEWEVEWRQTTEATEARETELFEAVVVCNGHNSELRFADIPGIQKWPGKQIHSHNYRIPEPFLDQVVVIIGKGASAFDISKEISKLAKEVHVASRTTEVKLGKLDNFDNIWQHLMIKCVHEDGNVVFEDGFEVIADVILHCTGYIFSFPFLKENKVVCVDDSRVGPLFKHVFPPHLAPWISFIGISSKSITFMMIEFQSKWVAKVLSGKVKLPSEEEMVASVNEVYAKMEELKKPKRHTHFLSVLEPGYLNWIAAEIGLPLVEEWKLQMYSLLVKCVVSYDDGYRDRWSLEQWKAQSEPSNQGT

>Eucgr.D02486|Egrandis_v2.0/1-447

MAKRSVKAAVIGAGMSGLIAARELRREGHRVVVFEKGSEVGGTWVYDPRVESDPLGLDPGREVIHSSMYKSLRTNLPRPIMGFLDYPCDAKPGVWDDPRPFPGHKEVLRFLQGFAADSGVVELVRFGHEVVRVEQVAEGRNDEWVVEWRTRDGEASAEAFEAVVVCNGKYTEPRLAEFPGRSTWRGEQIHSHNYRIPEPYKDKIVVIIGNGPSGIDIAKDILHVAKEVHLASRETTKLEILEGLFQHSPINHAKEDGKIVFQDGSSIYADAIIHCSGYKYHFPFLRMNGIVNVEDNCVAPLYQHVFPPALAPWLSFIGVPYRTIAFVVSELQSRWVAKILSGKILLPSEEEMTSSVHEYYHRIEAAGWPKRHTHRLQLEKFDYENWLAGELGLTPLEKWRQNMYFTSVSLPMTLGENWRDTWDAEKWMKGEAGGEEESDKINCTSEC

>Eucgr.D02484|Egrandis_v2.0/1-447

MAKRSVKAAVIGAGMSGLIAARELRREGHRVVVFEKGTEVGGTWVYDPRVESDPLGLDPGREVIHSSMYKSLRVNLPRPIMGFLDYPLDAKPGVRDDPRPFPGHEEVLRFLQGFAADSGVVELVRFGHEVVRVEQVAEGRNDEWVVEWRTRDGEASAETFEAVVVCNGKFTEPRLAEFPGRSTWRGEQIHSHNYRIPEPYKDKIVVIIGNGPSGIDIAKDILHVAKEVHLASRETTKLEILEGLFQHSPIDHAKEDGKIVFQDGSSIYADAIIHCSGYKYLFPFLRMNGIVNVEDNCVAPLYQHVFPPALAPWLSFIGVPYRAIAFLLSELQSRWVAKILSGKILLPSEEEMMSSVHEYYQRIEAAGWPKRHTHRLQLEKFDYENWLVGELGLPPLEKWRQNMYFTSLSLLMTLGENWRDTWDVEKWMKGEAGGEEEPDKINCTLEC

>Kalax.0079s0077|Klaxiflora_v1.1/1-436

MTPRSLNVAVIGAGMAGLEAARELRREGHRVTIYEQSCQLGGTWRYDPRTESDPLGLDPDRAVVHSSAYESLRVNLPRSLMGFLDYPFEGVGDGRWFPWHGEVLRFLEEFAAEFGLVELIRFGREVVGVGRMGGGGWFVESRGGGGDLEVETEVFEAVVVCNGKHTEPRIAEFEGREKWVGVQLHSHSYRTPEPFQNQIVVVIGAGSSALDISEDISSVAREVHLATRSPIPQFAGIEDLDCYHQMIKCAYEDGKVAFEDGSYVYADAIIHCTGYKFHYPFLKTDGIVSVEDNRVGPLYQHVFPPSAAPWLSFIGLPYRGIVSLIIELQSRWVAKVLSGEVELPSEEDMLSSVENHYAQMKRTSWPVRHSHNLKQQIHKFGYENWLAAQVGLSPIEKWREELYFYMYILYPEGSKYRDELDVDEWMQERGYNEGEL

>Kalax.1302s0009|Klaxiflora_v1.1/1-442

MTPRYLNVAVIGAGMAGLEAARELRREGHRVTIYERSRDLGGTWRYDPRTESDPLGLDPDRAVVHCSTYESLRVNLPRSLMGFLDYPFEGVGDGRWFPGHGEVLRFLEEFAAEFGLVELIRFGREVVGVGRAGGGGWFVESRGGGGDLEVEVEVEVETEVFEAVVVCNGKHTEPKIAEFEGREKWVGVQLHSHNYRTPEPFQNQIVVVIGDGPSALDISEDISSVAREVHLATRSPIPQFSGIEDPDCYHQMIKCAYEDGKVEFEDGSYVYADAIIHCTGYKFHYPFLKTDGIVSVEDNRVGPLYQHVFPPSAAPWLSFIGLPYRGLFSLIIELQSRWVAKVLSGEVELPSEEDMLSSVENHYAQMKRTSWPVRHSHNLKQQIHKFGYENWLAAQVGLSPIEKWREELYFYMFSLYPEGSKYRDELDVDEWMQERGYNEGEL

>Gohir.D11G083800|Ghirsutum_v3.1/1-484

MLLFPWINKHFNSGADLKESKSRYPNGETQLLNRSGKPSIVIVLKMRQSYKVAVVGGGFAGLVTARELKREGLLVTVFEKADHVGGIWLYNSGVETDLLGLDPNREIVHSSLYRSLRVNLPRQIMGFTDYPFMKKEGGDPRTFPGHEEVLKFLEDFVRDFRLMELIRFGHEVVRVELTDEARHKWVVESRTRETESRWESKEELFEAVVICNGKHTEPKIAEFPGRDAWPGLQMHSHSYRTPEQFENKIVVLIGNGSSAKDILKEISPLASQVHQAIRGPDSQLKRLENHDNAWQHSMIECARKDGKVVFQDGSIVDADVIIHCTGYKFHFPFLRSNGTVTVDDNRVGPLYKHVFPPSLAPWLSFVALPYKAVPSIVMESQAKWVAKVLSGKVKLPTQAEMADSVEELYRLMEKSGRPKHLTHTLQQDKFEYENWLATQLNIRPPERWKEIMFFSMEKIKSYYGDKYRDAWDVDKWIQEVDCSN

>Gohir.A11G079200|Ghirsutum_v3.1/1-439

MGQSYNIAVIGAGAAGLVTARELQREGHRVTVFEKANKVGGTWLYDSRVESDLLGLDPNREIVHSSLYKSLRTNLPRQVMSFMDYPFVKRESGDRRPFPGHEEVLRFLQDFARDFGLMELIRFGHEVIRVELVDEVSHEWVVESRTRETESRWESKEEVFEAVVICNGKHAEPKVAEFPGRDTWPGLQMHSHNYRTPEQFENKIVVLIGNGPSATDILKDISPLAKQVHLAFRGSDIMLINLKNYDNAWPHSPIECAHEDGKVVFQDGSIVEAEVIIHCTGYKLHLPFLKSNGIVTVDDNRVGPLYKHIFPPSLAPGLSFVGINYRALVFRVIELQAIWVAKVLSGKVKLPTQEAMAASVEEFYEQMEKAGLPKYHTHSLQNDEGEYVSWLAAQLDIRPPKSWEELRFFSFVKGIFAHGENFRDTWDVDKWIQEIESSD

>Gohir.D11G083900|Ghirsutum_v3.1/1-439

MGQSYNVAVIGAGTAGLVTARELQREGHRVTVFEKANQVGGTWLYDSLVESDLLGLDPNREIVHSSLYKSLRTNLPRQVMSFLDYPFVKRESGDTRPFPGHEEVLRFLQDFARAFGLMELIRFGHEVIRVELVDVVSHDWVVESRTRETESRWESKEEVFEAVVICNGKNTEPKVAEFPGRDTWPGLQMHSHNYRTPEQFENKIVVLIGNGPSATDILREISPLAKQVHLAFRGSDIKLINLKNYDNAWPHSPIECAHEDGKVVFQDGSIVEADVIIHCTGYKFHLPFLKSNGIVTVDDNRVGPLYKHIFPPSLAPWLSFVGLNYRALVFRVIELQAIWVAKVLSGKVKLPTQEAMAASVEEFYEQMEKAGWPKYHTHSLQNDEGEYASWLAAQSDIRPPKSWEEITFFSFVKGIFGHGENFRDTWDVDKWIQEIESSD

>Qrob_P0578460.2|Quercus_robur/1-257

MDSWPGVQIHSHNYRTPEPFQNLIVVVIGNGSSAVDILTEISSVAKEVHQALRASDIHFKKLESHNNIWQHSMIECTKEDGKVVFQDGSFVYADVIMHCTGYKYHFPFLRTNGIVSVDDNRVGPLYKHVFPPQLAPWLSFVGLPYRGVTTLIIELQSRWVARVLSGKVALPSEEEMASSVEELYQHMEESGWPKQHTHQLQNKFDYENWLVTQILDLPPLEKWREEMYFSALKKIISIDDDEFRDTWDINKWMQEIA

>evm.TU.supercontig_29.133|Cpapaya_ASGPBv0.4/1-445

MPSAFFPISRHVAVIGAGAAGIVAARELRREGHSVVVFERGNQVGGIWVYTPKVESDPMSLDPARNVVHSSLYSSLRTNLPRECMGYQDYPFVPRPGDSRDPRMYPCHREVLVYLQEFAKEFEIEEMIRFETEVELVGLVEEGKWRVRSKEKGGGGGDEIYDAVVVCNGHYTEPRVADIPGIKSWPGKQMHSHNYRVPDPFQDQVVVLIGSSASAVDISRDIAGVAKEVHVASRSVADETYEKLTGHANLWLHSMIETTCEDGSVVFRNGRAVFADVILHCTGYKYHFPFLETSGYVTVDDNRVGPLYKHIFPPALAPWLSFVGLPWKVAPFPMFEFQSKWIAGILSDRIKLPSQEEMMEDVKAFYLALEADGIPKRYTHCMADAQFEYNNWLAAQCGCRGIEEWRKEMYRASSRNKRLLPEIYRDDWEDHHLVSQAYDDFHKCT

>Gohir.D03G161300|Ghirsutum_v3.1/1-483

MAINYFSRCAKMNISAINLAMPPTSLSVKSRHVAVIGAGAAGLVAARELRREGHSVVVFERGHEVGGTWVYTPQVEPDPLGLDPNRPIIHSSLYSSLRTNLPREVMGYIDFPFVTRPGENRDPRRYPGHREVLLYLKDFAREFGVEEMVRFETDVIKVGILGDEKWKVRSKKSSFNDNNEIISKSNAEFDDEIYDAVVVCNGHYTEPRIADIPGINLWPGKQMHSHNYRIPEPFRDQVVIVIGSSASAVDICRDIAPVAKEVHVASRSVADETYTKQPGYNNLWFHSMIDHAHEDGAVVFRNGKTVLADLIMHCTGYKYHFPFLDTKGIVTVDDNCLGPLYKHVFPPALAPYLSFIGIPWKIVPFPLFEFQSKWIASILSGRITLPSQKEMMEDIQAFYSTLEVSSIPKRYTHCIGQSQFEYNNWLATQCGCQGVEKWREAMYSMASVNRSLRPEMYRDEWDDHHLVSEAYEDFMKYLSASNL

>Gohir.A03G009500|Ghirsutum_v3.1/1-483

MAINYFSRCAKMNISAINPAMPPTTLSVKSRHVAVIGAGAAGLVAARELRREGHSVVVFERGHEVGGTWVYTPQVEPDPLGLDPNRPIIHSSLYSSLRTNLPREAMGYMDFPFVTRPGENRDPRRYPGHREVLLYLKDFAREFGVEEMVRFETDVIKVGILGDGKWKVRSKKSSFNDNNEIISKSNAEFDDEIYDAVVVCNGHYTEPRIADIPGINLWPGKQMHSHNYRIPEPFRDQVVIVIGSSASAVDICRDIAPVAKEVHVASRSVADETYMKQPGYDNLWFHSMIDHAHEDGMVVFRNGKTVLADLIMHCTGYKYHFPFLDTKGIVTVDDNRLGPLYKHVFPPALAPYLSFIGIPWKIVPFPLFEFQSKWIAGILSGRITLPSQKEMMEDIQAFYSALEDSSIPKRYTHCIGQSQVEYNNWLATQCGCQGVEKWREAMYSMASENRRLLPEMYRDEWDDHHLVSEAYEDFIKYPSASNL

>orange1.1g037525m.g|Csinensis_v1.1/1-182

VIILVGSSASAVDICRDLAGVAKEVHLVSRSVADGTYEKQPGFDNMWLHSMIESAHDNGAVVFRNGHTVHADVILHCTGYKYHFPFLETNGIVTMDDNRVGPLYKHVFPPVLAPWLSFVGLPWKVIPFPLCEYQSKWIAGVLSGQIVLPSQEEMMEDTKAFYSTLEASGTPKRYTHNMGDYQ

>Potri.001G121100|Ptrichocarpa_v4.1/1-459

MPPPQLPPPISRHVAVIGAGAAGLVSARELRREGHDVVVFERDNQVGGTWVYNPRVEPDPLSLDPNRRIIHSSLYSSLRTNLPREVMGFKDYPFIAKNDKKRDQRRFPGHREVLLYLQDFASEFGIEEMVRFDTEVVHVGPVEDNIGKWIVRSKRKISDDDREVSFGFDVDEEIYDAVVICNGHYTEPRIAQIPGISSWPGKQMHSHNYRTPEGFQDQVAILIGSSASSDDISREIAGVAKEVHVASRSVADETYQEQPGYDNMWLHSMIESVHDDGSVIFRNGRVVVADIILHCTGYKYHFPFLDTNGIVTMDENRVAPLYKQVFPPVLAPWLSFVGLPWKVVPFPLVELQTKWIAGVLSGHIALPSPEEMMEDVKAFYETLESSNKPKHYTHNLGGCQFEYDNWLASQCGCPGIEEWRRQMYDAASKSKRLRPEIYRDEWDDDDLVLEAYGDFTKYT

>Prupe.5G074900|Ppersica_v2.1/1-455

MQLALNPLASRQVAVIGAGAGGLVAARELWREGHKVVVFERGEQVGGTWVYTPKVESDPLGLHPDRTTVHSSMYQSLRTNLPRESMGFRDYPFVAKEEDEKRDPRRFPGHREVLRYLKDFASEFGISEIVRFETEVMVVDLVEGGKWKVKSKSKGGDGVHDEIYDAVVVCNGHYTEPRIAEIPGINTWKGKQFHSHNYRNPEPFRDQVVILIGGAASSADISRELAGVAKEVHIASRSVADEAIGKQPGYDNMWLHSMIKSAHDDGSVAFQDGSVVIADIILHCTGYKYHFPFLETNGIVTVDDNRVGPLYKHVFPPALAPSLSFVGLPWKVVPFPQFELQSKWIAGLLSNRIALPSKEEMMEDIKAFYSLLEASGLPKRYTHNLGDCQFEYNDWLAALCGCPVSEEWRKKMYLEVSKNRHARPETYRDEWEDDHLILQAHEDFKKYTLNGVTKK

>Prupe.5G074800|Ppersica_v2.1/1-467

MQPTHHETASRHVAVIGAGAGGLVAARELRREGHKVVVFERGDQVGGTWVYTPNVESDPIGLDPNRTRVHSSMYQSLRTNLPREAMGFRDYPFVAIEEDEERDPRRYPGHREVLMYLKDFAIEFGVSEIVRFETEVVFVGLVEGGKWKVKSRSKRGEVVEDEIFDAVVVCNGHHTEPCVAQIPGINTWKGKQIHSHNYRTPEPFRDQVVILIGSATSALDISRDIVGVAKEVHIASRSVADETLEKQPGYNNMWLHSMIKSVQEDGSVVFRNGSVVLADVILHCTGYKFHFPFLETNGIVTVDDNRVGPLYKHVFPPDLAPSLSFVGLPWKVAPFPMFEFQSKWIAGILSNRIALPSREEMMEDVKAFYSLLEASGIPKRYTHNIADYQFGYDDWLAAECGCPAFEEWRKQMYFVAIENLFARPETYKDEWEDHHLVLQAHEDFRKYTLNGVENGYVTSNDVPPSSR

>FvH4_5g00220|Fvesca_v4.0.a2/1-365

MGFRDFPFVAKEGDEDGDPRRFPGHREVLKYLNDFAREFGICEMVRFESEVVFVGLVEGGKWKVKSKNKRSEVVEEIYDAVVVCNGHHTDPRVAQISGMHTYKGKQIHSHNYRTPEPFRDQVVILIGSATSAVDISRDIAGVAKEVHIASRSVADETIEKHPGYDNLWLHSMIQTVHEDGGVAFQDGSVVCADIILHCTGYKYHFPFLEMDGIVIVDDNRVGPLYKHVFPPVFAPSLSFVGIPWKDALFFMFELQSKWVAGILSNRIALPSQEDMMEDVKAFYSSLEASGTPKRYTHNIADHQSGYDDWVATECGCPVSEEWRKQMYYAAIENMLTKPETYRDEWGEYEHLVLQAHEDFKKYALN

>FvH4_5g00230|Fvesca_v4.0.a2/1-485

MTIPSLIQRMTTQSLLSFTAKPTTPTRRVFLSAMQPPTTRNVAVIGAGAAGLVAARELRREGHKVVVFERGDQVGGTWVYTPEVESDPIGLDPDRATVHSSMYQSLRTNLPREVMGFRDYPFVAKAKEGDGERDPRRFPCHREVLMYLKEFADEFEICESVRFEREVVFVGLVEGGKWEVKTRSKRGENFDDVYDAVVVCNGHYSQPRIAHIPGIDTWKGKQIHSHNYRTPEPYRDQVVILIGSSASAVDISRDMVGVAKEVHIASRSLPDETFEKMSGYDNMWLHSMVKSINEDGVAVFPDGSAVRADIILHCTGYIYDFPFLETNGTVTVDDNRVAPLYKHVFPPVLAPSLSFVGLPWKVVPFPLFELQSKWIAGILSNRITLPSEEEMMEDVRAFYSLLESSGIPKRYTHNMGDYQFEYDDWLAAQCGCPVSEEWRKPMYLAASKNRRARPETYRDEWEDDHLVLQAHEDFKKCTSHAVSRG

>FvH4_5g00210|Fvesca_v4.0.a2/1-457

MQPLCRHVAVIGAGAAGLVAARELRREGHNVVILERGDQIGGTWVYTEEVESDPIGLDPDRTRVHSSMYQSLRTNLPREIMGFRDYPFVAKEEGDEDRDPRRFPGHREVFKYLNDFAKEFGICEMVRFESEVVFVGLVEGGKWKVKSKSKRGEVSEEVYDAVVVCNGHYTEPRVAQIPGISSWQGKQFHSHNYRTPEPFRDQVVILIGSSVSAIEISRDIAGVAKEVHIASRSVADETMEKKPGYDNMWLHSMIKSVHEDGSVVFPDGSVVLADSILHCTGYKYHFPFLEANGIVIVDDNRVGPLYKHVFPPALAPSLSFVGIPFKVVPFALYEFQSEWIAGILSNRIALPPEEEMMEDVQAFYSLQKASNISKRFTHMMDNYQFEYDDWLAAQCGCPASEEWRQQMWLATFKNARLRGMLYREEWNDHHLILQAHEDFRKYTFNGVSMDNWISDVK

>GWHPBFSI015759|Chloranthus_spicatus/1-476

MAAQTPKPLTLLSQTLSSNLRPSKTSRKVAVIGAGPAGLVAARELRSEGHRVVVFECGDKVGGTWNYNPDVESDPLGLDPSRRIVHSSMYDSLRTNLPREVMGFHDFPFVVRDGRDSRRFPGHQEVFLYLDDFARAFDLKELIRFDTEVFGVRLVGEGRWMVRSRRVLMDGEDQMVDVEEVYDGVVVCNGHYREPRIADIPGIHTWPGKQMHSHNYRVPEPFCDQVVVLIGSSASAVDISRDISKVAKEVHLASRSAPDGTPTKQPGYDNMWLHSMIRRANEDGMVVFQDGSSLLVDVIVHCTGYKYHFPFLETDDIVTVDDNRVGPLYKHVFPPLLAPWLSFIGLPWKVAPFLLYELQSKWVAGVLSGCVPLSSQEEMMEDVKAFYSELEAKGYPKRYTHNMSDYQFEYGDWLATECGYPKMEEWRKEMFAATRKNRLAYPETYRDEWEDHRWVIQAHEEFSNAEKTVNSQNHLH

>GWHPBFSI015761|Chloranthus_spicatus/1-476

MAAQTPKPLTLLSQTLSSNLRPSKSSRKVAVIGAGAAGLVAALELRREGHRVVVFECGDKVGGTWNYNPDVESDSLGLDPSRRIVHSSMYDSLRTNLPREVMGFHDFPFVVRDGRDTRRFPCHQEVFLYLDDFARAFDLKVLIRFDTEVFGVRLVGEGRWMVRSRRVLMDGEDEMVDVEEVYDGVVVCNGHHTEPRIADIPGIHTWPGKQMHSHNYRVPEPFCDQVVVLIGSSASAVDISRDISKVAKEVHLSSRSAPDGTPTKQPGYDNMWLHSMIRCANDDGMVVFQDGSSLLVDVIVHCTGYKYHFPFLETDDIVTVDDNRVGPLYKHVFPPLLAPWLSFIGLPWKVVPFLLCELQSKWVAGVLSGRVPLSSQEEMMEDVKAFYSELEAKGYPKRYTHNMSDYQFEYDDWLAAECGYPKMEEWRKEMDAVTRKMALAHPETYRDEWEDHRWVIQAHEEFSNAEKTVDSQNHLL

>Hyque.11G102700|Hquercifolia_v1.1/1-458

MTIPTPTLHFSPMRPLTSRNVAVIGAGAGGITAARELRREGHAVVVFEREDRIGGTWIYNPNTDSDPLGLDPLRTVVQTSIYASLRTNLPREAMGFREYPFIASNKTHRDPRRFPGHGEVLEYLGDFASEFGLSELVRFGTEVSHVGLAESGKWEVRSTIGGDQVDEIYDAVVVCNGHYTEPRIAEIPGIEAWPGKQIHSHNYRVPEPYRDQVVIVIGSAASAVDISRDIAGVAKEVHIASRSGTDGNLGKLPGYDNMFLHSMIENSHEDGTVLFQDRSSVCADVILHCTGYKYHFPFLDTNGIVAVDDNRVGPLYQHIFPPVLAPWLSFVGLPWKVAPFPLCEFQCKWIAGVLSGRLALPSPEEMMAEVEAFYSKLEASGVPKRYTHNLTGYQFEYNDWIAAQCGCPPVEEWRKQMYSATSKNRIARPETYRDEWDDQHLVQDAHEDFLNYLYKRSY

>Rhdel07G0121900.1|Rhododendron_delavayi/1-449

MTTVLPKSVAVIGAGAAGLVAARELRREGHEVVVFERGNKVGGTWVYNPTTESDPLGIDPARTVVHSSLYASLRTNLPREVMGFREYPFVAKNGAHRDPRRFPGHGEVLEYLNDYTTEFGLGELVRFGAEVRRVGLEGGKWKVRSTACGEPLDEVYDAVVVCNGHYTEPRIAEIPGLETWPGLQIHSHNYRVPEPFRDQVVILIGSSASAVDICRDIAGVAKEVHIASRSVTDGTMGKLPGHDNMWLHSMIESAHGDGTVVFRDGSAICADVILHCTGYKYHFAFLDVNDTVTVDDNRVGPLYKQVFPPLLAPLLSFVGLPWKVGPFPLCELQSKWIAGVLSGRVSLPSSDEMMADVEAFYRTLDASGIPKHYTHNIDDSQFEYNDWLATECGCPPSEEWRKQMYSETNKNRKVRPETYRDEWDDELLVAQAHEDFANCSSEGNGNLSQ

>Litul.16G098000|Ltulipifera_YP108A_v1.1/1-460

MAASPTLHHVPSPNISRAVAVIGAGAAGLVAARELRREGHKVVVFERGERVGGTWIYDPSVESDPLGSDPSRKIVHGSLYASLRTNLPREAMGFRDYPFVARDGRDSRRFPGHQEVLLYLEDFSRDFGLEGSIRFETEVFEVGLDGGRWRVRSRRIGMDGCDGASEVYDGVVVCNGHFTEPRIAEIPGIDVWPGKQIHSHNYRVPELFRDQVVVLIGSSASAADISRDIAKVAKEVHISSRSAPDGPTAKQPGYDNMWLHSMIQSAHEDGTVVFKDGSSVLAEIILHCTGYKYHFPFLKTNDIVNVDDNCVGPLYKHVFPPSVAPCLSFIGLPWKVVPFPLCELQSKWVAGTLSGRVVLPSQEEMMEDVKAFYLKLDAAGYPKRYTHNLSDCQFDYNDWLAAECGYPAVEEWRKQMYAATSKNRVLRPETYRDEWDDDHLVLQAQADFQQYCKRPCSQQP

>Qrob_P0629240.2|Quercus_robur/1-471

MPTTTIQMQCHLPIPQPPAPHPLTSRHVAVIGAGAAGLVTAIELRREGHSVVVFERGDQVGGTWVYTPKIESDPIGFDPARTKIHSSLYKSLRTNLPRECMGFRCYPFVAKDDPDRDPRRYPGHQEVLWYLQDFTREFRIDELVRFETEVVRVRLVEEEKWKIKSKQRSGKDETEIFDAVVICNGHYTEPRVAQIPGINEWPGKQMHSHNYRVPEPFRDQVVVLIGSSASAVDISRDIAGVAKEVHVAARSVANETSGEQSGYDNMWLHPMIESVRKDGTVFFQDGSFVHADIILHCTGYKYHFPFLETNGIVNVDDNRVGPLYKHVFPPALAPCLSFVGLPWKVVPFPMFELQSKWIACVLSNQFALPTQEKMMEDVEAFYSSLEASGMPKRYTHNMGSSQFEYNNWLAAQCGCPAIEDWRVQMYNTSSKNKRAQPESYRDDWEDHHLVLQACEDFMKYTSHKVSDRSAS

>BUSI_026619-RA|Buxus_sinica/1-473

IPKVVHAPHKDPENSWKVAVIGAGAAGLVAARELRKEGHKVVVFEQSDRIGGTWVYNPNVESDLLGLDSSRTIVHSSLYESLRTNLPREVMGFRDYPFVAKDGRDRDSRRFPGHRETSLATSKLTELVRFETQVFHVGLVEEGKWVVRYRKKYQNDKEHVRIDEDEFYDSIVVCNGHFTEPRIAEIPGIDSWPGKQMHSHNYRVPDPFQEKIVVLIGSSASAVDISRDIAEVAKEVHIATRSFGDGATKQPGYNNMWLHCMVKGALEDGTVVFQDGTSIFADVVLHCTGYKYHFPFLKTNNIVTVDDNCVGPLYKHIFPPVLAPCLSFIGLPWKVIPFPMLEFQVKWVAGVLSGSILLPSQEEMMGDVEAFYSKLESAGIPKRYAHNMSDYQFEYNDWLAAECGCPASEKWRKEMYYSTGKNRVSRPNTYRDEWEDQDLVSEAQTDFVQYLSCYNRCEVEHKQSLPAETPPKV

>BUSI_015850-RA|Buxus_sinica/1-465

MAASIPASQNLSMPPIKTPENSCKVAVIGAGAAGLVAARELRKEGHRVVVFEQSDRIGGTWVYNPNVESDLLGLDSSRTIVHSSLYESLRTNLPREVMGFRDYPFVAKDGRDRDSRRFPGHREVLHYLEDFARDFKLTELVRFETQVFHVGLVEEGKWVVRYRKKHQNDKEHVRIDEDEVYDSIVVCNGHFTEPRIAEIPGIDSWPGKQMHSHNYRVPDPFQEKIVVLIGSSASAVDISRDIAEVAKEVHIATRSFGDGATKQPGYNNMWLHCMVKGALEDGTVVFQDGTSIFADVVLHCTGYKYHFPFLKTNNIVTVDDNCVGPLYKHIFPPVLAPCLSFIGLPWKVIPFPMLEFQVKWVAGVLSGSILLPSQEEMMGDVEAFYSKLESAGIPKRYAHNMSDYQFEYNDWLATECGCPASEEWRKEMYYSTGKNRVCRPNTYRDEWEDQDLVSEAQTDFVQYLS

>GWHPBAUK014006.1|Macadamia_integrifolia/1-464

MDMTISTLQRLAMTPMMNSKSSHKVAVIGAGVAGLVAARELRREGHKVVVFERGQKVGGTWIYDPNVESDHLGLEASRAIVHTSLYDSLRTNLPREVMGFRDYPFVAKEGKDRDTRRFPGHREVLLYLDDFARDFELTELVRFETEVFHIRMEERKWIVRSKKRAPTDREDSQIDMDEVYDGVVICNGHYTEPRIAEIPGIDSWPGKQMHSHNYRVPDPFRDQVVVLIGSSSSASDISRDIARVAKEVHIAARSVTDETMVKQPGYENIWLHPMIKSAHGDGTMLFKDGSSVLPDVVLHCTGYKYHFPFLDTRDIVTVEDNCVGPLYKHVFPPSLAPWLSFIGLPWKIIPFPLFELQSKWVAGILSGRIVLPSQEEMMEDVNNFYSKIEAAGWPKRYTHNMGDYQFEYDDWLATECGCSVTEEWRKKMYDANGRNKHARPETYRDEWEDQHLILQANEDFLQYK

>Acora.08G045700|Aamericanus_v1.1/1-471

MAAPTRPNHCAAPHVRPPRTARRIAVIGAGAAGLVAARELRREGHSVVVFERAGGVGGTWIYDAAVESDPLSLSPRRRVVHTSLYESLRTNLPRESMGFLDYPFLAREAEDGRDRRRFPGHGEVLRYLEDFARDFDLGRMIRFETEVSHVGMANDDSGGGGWTVRSRRADGDGEGEEETSEVYDGVVVCNGHYTTPRVAEIPGIDTWPGKQMHSHNYRVPEPFRNLVVVLIGGSASAVDISRDIAKVAKAVHVASRSAPPGTELQQPGYDNMWLRSMIESAHEDGTVVFEGGGSMLADVILHCTGYTYHFPFLETNGIVTVDDNRVGPLYKHIFPPLLAPWLSFIGLPWKVIPFPLFELQSKWVAGVLSGRIALPTQQEMMDDVKAFYSELESSYCPQRYTHNVSNYQFDYNDWLSAACGCEFAEDWRRKMYVATGKNRVARPETYRDEWEDDHLVIQAHEDFQRQSKVHT

>DCAR_831668|Dcarota_subsp_sativus_v3.0/1-454

MPIPIPNPTRLRHVAVIGAGAGGLAAARELRREGHTVVVFERENNIGGTWVYDPRTESDPLGPDPTRNIVHGSLYASLRTNLPREVMGYRDYPFVASTRPGRDPRRYPGHKEVMEYLNDYATEFGLGELVRFETEVLEVGIVENGKWKVKYGKNGKVDDEVYDAVVVCNGHYTEPRIADIPGIDSWPGKQIHSHNYRVPDPYRNQVVIMIGSAASAVDISRDVSTVAKEVHIASRSYNNGASKLPGHNNIWLHPMIESVREEGSVTFQDGSEVRASIILHCTGYKYHFPFLKTNNIVTVDDNRVGPLYKHIFPPTLAPWLSFVGLPWKVVPFPLFEYQSKWIAGALSGKFSLPSPEEMMKDVENFYSSMETAGIPKRYTHNLASCQYEYNDWVAAECKSAPSEEWRKEMYVSASKSKIVRPETYRDEWDDEHLIMQAHEDFNKNFKVAKNLIKG

>CKAN_01643800|Ckanehirae_v3/1-484

MQSSTSFHSLSMAAPPIRDPAAMYPHLPSSNGPQIRSCHVAVIGAGAAGLVAARELRREGHKVVVFESGDSVGGTWVYSPSVDSDPLGLDPYRKKVQTSLYQSLRTNLPRESMGFRDYPFVAKPTNKERDSRRFPGHGEVLLYLQDFAVDFGLMGLVRFKTEVFDVHLVDEGRWMVRSRTVRMDGYDDDDENEVYDGVVVCNGHYMEPRIAEIPGIDVWPGKQIHSHNYRVPEPFQDQVVILIGSSASAVDISRDICAFAKEVHVSSRSAPDKQPASKQPGYDNMWLHSMIESAHDDGTVVFRDGSSVLADVILHCTGYKYDFPFLKVNGVVTVDDNRVGPLYKHIFPPLLAPSLSFIGLPWKVVPFPLFEMQSKWVASVLSGRLALPSQEEMMMDVEAFYSKLDAAGMPKRYTHSMSDYQFDYTDELAAECGYPAVEEWRKQMYAMSWENKVARPESYRDEWDDHSLVLQAHEYFLRYCKASI

>CKAN_01643700|Ckanehirae_v3/1-454

MGHSRSCEVAVIGAGAAGLVAGRELRREGHKVVVFEKGESVGGTWVYSSSVESDQLGLDPCRKIVHSSLYQSLRVNTPRECMGFFDYPFVPRPSNKERDSRRFPEHTEVLLYLQDFAREFELMGLLRFGTEVFDVHLVDEGRWMVRSRKVRMDGCDDSVADESEVFDAIVVCNGHYTEPRIAEIPGIERWPGKQIHSHNYRVPEPFQDQVVVLIGSSASALDISRDICAFAKEVHLSSRSAPDKQPMSKQPGYDNMWLHSMIERAHADGTVAFRDGSTVRADIILHCTGYKYNFPFLKVNGVVTVDDNRVGPLYKHIFPPLLAPSLSFIGLPCRVLPFPLFEMQSKWVASVLSGRLALPSQEEMMMDVESFYSKLDAAGLPKRYTHAMLDYQSDYLDELATVCGHPAMEEWRKQMSDMTLKNIRSRPDSYRDEWDDHSLVLQAYEDFHRYCKSL

>evm.TU.Scaffold_465.563|Carabica_v0.5/1-534

MGVSKYPESEGGINFDLPFVIKNAPPCAGVEHVEGDVFQCVPRGEAILLKQTVTYDELTTPHDCSSPVPIVRFSNPPTMYPIASKNVAVIGAGAAGLIAAHELRQEGHKVVVFERENQVGGIWVYNPATESDPLGVDPTRNLIHSSLYASLRTNLPREVMGFRSYPFLYKRGSRRDPRRFPGHREVLEYLKDFAVDFKLCGLVRFGTEVWHVGLIENGKWKVTSRKREGNAYNDRNQEKLDEVYDAVVVCNGHYTEPRPAEIPGVEGWPGKQIHSHNYRDPEPFRDQVVVLIGSAASADDISREIAKVSKEVHIASRAVQNGIMGKLAGYDNIYHHSMIESTHGDGSVAFQEGSVVYADIILHCTGYKYHFPFLETNGMVTVDDNRVGPLYKHIFPPALAPWLSFVGLPWKVVPFPLFEFQSKWIAGTLSGRLSLPSPKEMMADIQAFYSSMEASGTPKRYTHNMAGYQFEYDDWLAAQCGCLPTEEWRKQMYVETSMRKRSQPETYRDQWEDEHLVRQALADFSQYFSKVPSS

>evm.TU.Scaffold_629.313|Carabica_v0.5/1-456

MYPIASKNVAVIGAGAAGLIAAHELRQEGHKVVVFERENQVGGIWVYNPATESDSLGVDPTRNLIHSSLYASLRTNLPREVMGFRSYPFLYKRGSHRDPRRFPGHREVWEYLKDFAVDFKLCGLVRFGTEVWHVGLMENGKWKVTSRKREGNAYNDRNQEKLDEVYDAVVVCNGHYTEPRPAEIPGVEGWPGKQIHSHNYRDPEPFRDQVVVLIGSAASADDISREIAKVSKEVHIASRSVQNGIIGKLAGYDNIYHHSMIESTQGDGSVAFQEGSVVYADIILHCTGYKYHFPFLETDGIVTVDDNRVGPLYKHIFPPALAPWLSFVGLPWKVVPFPLFEFQSKWIAGTLSGRLSLPSPKEMMADIQAFYSSMEASGTPKRYTHNIAGYQFEYDDWLAAQCGCLPTEEWRKQMYVETSMRKRSQPETYRDQWEDEHLVRQAEADFSQYFSKVPSS

>Eucgr.E00454|Egrandis_v2.0/1-451

MVSQHVAVIGAGAAGLVAARELHHEGHRVVVFERGSCVGGTWVYTPVTEPDPLGIDPSRPVVHSSLYKSLRTNLPREVMGFGDYPFLPREDGSGDPRRYPGHEEVLAYLEDFTREFGLEEVVKFEKEIAGVRKAEEEEEGGKWKVAWTMRNDGGGGGGREEEVFDAVVVCNGHYTEPRIAEIPGIDLWPGKQMHSHNYRTPEQFRDLVVVLIGSAASAVDVSRDIAKVAREVHVASRSYEYDTLGMQSGHDNMWLHPMIEKAHEDGKVTFLDGSYVGADIILHCTGYKYHFPFLETNGIVSVDDNRVGPLYKHVFPPHLAPWLSFVGLPWKAVPFPQFEHQSKWIAGVLSGRISLPSEEEMMEDVKDLYSELKALGIPKRYTHNLANSQFEYNDWLAVQSGSQPSEEWRKEMFLESTRKMQARPEVYRDEEIDETLISQAHEDFAKFPSHR

>Eucgr.E00455|Egrandis_v2.0/1-424

MVSQHVAVIGAGAAGLVAARELRRKGHRVVVFEREPRAGGTWVYTPATKPDPLGFDPSQPVVHSSLYKSLRTNLPREVMGFGDYPFLPRADGSGNPRRYPDHKEALAYLEDFAREFGLEEVVRFEEEIVGVGKAEEGGKWKVSSRMRSGGGGGGGGGEEEVFDAAVVCNGHYTEPRIAEIPGIDLWPGQQIHSHNYRTPEPFQDLVVVLIGSAASAVDVSRDIAKVAKEVHVASRSYEYDTLGMQSGHDNMRLHPMIEKAHEDGKVTFLDGSSVDANVILHCTGYKYHFPFLETNGIVSVDDNRVGPLYKHVFPPHLAPWLSFVGLPWKVVPFPQFEYQSRWIAGALSGRIALPSEEEMMEDVEDLYSELEVLGIPKRYTHNLASSQFEYNDWLAIQSGSRPSEKWRKEMYSESMRKMRARPEV

>Eucgr.E00456|Egrandis_v2.0/1-458

MHMAASMISRHVAVIGAGAAGLVAARELRREGHRVAVFEREPRAGGTWVYTRATEPDPLGIDPSRPVVQSSLYKSLRTNLPREVMGFGDYPFLPRADGSGDPRRYPGHEEVLAYLEDFAREFGLEEMVRFGEEIVGVGMAEEGGKWKVTCRMSNGGGGGGGGGGGGEEEVFDAVVVCNGHYTEPRIAEIPGIDLWPGKQMHSHNYRTPEPFQDLVVVLIGSAASAVDISRDIAQVAKEVHVTSRSYEVETLGMQSGYDNLWLHPMIDKAHEDGKVTFLDGSSIGADIILHCTGYKYHFPFLETNGIVSVDDNRVGPLYKHVFPPHLAPWLSFVGLPWKVVPFPQFEYQSKWIAGALSGRVALPSEEEMMEDVKDLYSKLEALGVPKRYTHNLGNSQFEYNDWLAAQSGSQPSEEWRKKMYSESSMKRRARPEAYRDEEVDETLISQAHEDFAKLPSHR

>Pso09G24940.0|Papaver_somniferum/1-485

MSSPAYGGGDIRGFSNSLISTMNSRIPSSLTSRNVAVIGAGAAGLVAARELRREGHKTVVFERSNQIGGTWVYDPNIESDDPLGLNPSRNIIHTSLYKSLRTNLPRESMGFRDYPFVTKDGNDQRDNRRYPGHKEVLNYLEDFANDFKLTELIRFETEVFHVGLLKDDDKDGKKWVVRSAQKMRGGGGDGEAVDEVFDGVVVCNGHYTEPNIAEIPGIDEWPGKQMHSHNYRVPEPFRDQVVVVIGTSASGQDISRDIAEVAKEVHIAGRSLTEGVPTKLPGHENMWLHSMIESAHDDGRVVFQDGSSVTAGVILHCTGYKYHFPFLETNNIVIVDENRVGPLYKHVFPPLLAPGLSFIGLPWKVIPFPLSELQSKWVAGVLSGRISLPSQEEMMADVEAFYLELEAAGLPKRFTHNMSDYQFEYGDWLAAQCGCTPSEKWRKLMYYEASKKKRSQPETYRDEWEANHLILEAYEYFRQCIPTSV

>Kalax.0273s0014|Klaxiflora_v1.1/1-473

MNIPCDEIISIIHSSSMPIPTRLVSRHVAVVGAGAGGLVAARELRNEGHSVVVYEKLSRLGGTWVYDPNVESDPICQDPSRTIIHSSLYDSLRTNLPREAMGFRDFPFVAKDGVGRDARRFPGHKEVLMYLEDFADEFRIGEMIRFDSEVRYVGLVDGGKWRLRSWEKRDGEFEDVYDAVVVCNGHYAEPKIADIPGIGCWPGKQMHSHNYRVPEPFRDQVVVLIGSAASAVDISIDIAAVAKEVHIASRSAKEVALGKQPGYKNMWLHSMITSAHEDGTVVFQDGSKILANVILHCTGYKYHFPFLDTSGMVTVDDSRVGPLYKHVFPPALAPGLSFVGLPWKVVPFPLCELQSKWIAGVLSGRISLPSQEEMMEDIEAFYMKLEAAGIPKRYTHNCADCQFEYGDWLATKSGCHGSEEWRKEMYSQSSKTRHEKPDSYRDEWSNNQLFLQALEDFRKFLPPALIDILCNSL

>Kalax.0088s0090|Klaxiflora_v1.1/1-473

MNIPCDEIISIIHSSSMPIPTRLVSRHVAVVGAGAGGLVAARELRSEGHSVVVYENQSRLGGTWVYNPDVESDPICQDPSRTMIHSSLYDSLRTNLPREAMGFRDFPFVAKDGVGRDARRFPGHKEVLMYLEDFADEFRIGEMIRFDSEVRYVGLVDGGKWRLRSWEKRGGEFEDVYDAVVVCNGHYAEPKIADIPGIGCWPGKQIHSHNYRVPEPFRDQVVVLIGSAASAVDISIDIAAVAKEVHIASRSAKEVALGKQPGYKNMWLHSMITSAHEDGTVVFQDGSKILANVILHCTGYKYHFPFLDTSGMVTVDDSRVGPLYKHVFPPALAPGLSFVGVPWKVVPFPLCELQSKWIAGVLSGRISLPSQEEMMEDIEAFYTKLEAAGIPKRYTHNCADCQFEYGDWLATKSGCHGSEEWRKEMYSQSSKTRHEKPDSYRDEWSNNQLFLQALEDFRKFLPPALIDILCNSL

>Aqcoe7G079700|Acoerulea_v3.1/1-475

MSSIIYPSPKIPPPSTISHLTMQSQIPQSKNSYKVAVIGAGAAGLVAARELRREGHQVVVFERNSTIGGTWVYEPAIESDPLGVNPSRTIVHTSLYDSLRTNLPREVMGFLDYPFVAKQGKQRDNRRFPGHPEVLHYLEDFMSEFKLSELIRFETEVIHVGLFKEEETQGKWLVKSRRKDRNVEDDEVYNAVVVCNGHYTVPRVAEIPGIDLWPGKQIHSHNYRVPDPFCKKVVVLIGSSASAYDISRDIARVAKEVHVASRSITTETPTKQVGYNNLWMHSMIKCAYEDGTLVFQDGSSVLVDVILHCTGYKYDFPFLETTNIVTVDDNRVGPLYQHVFPPPLAPWLSFIGLPWKVVPFPLCELQSKWVAGILSGRVSLPSQEEMMSDVEAFYANLEAAGMPKRYTHNMASYQFEYDDWLAAQCRRAPTEEWRKQMYAATSKNKVIQPETYRDEWEDHNWLLQAHQDFHQYISN

>Aqcoe7G079500|Acoerulea_v3.1/1-456

MQPQIAPSKNSYKVAVIGAGASGLVAARELRREDHQVVVFERNNRIGGVWVYDRTIESDPLSIDPSRTIVHTSLYDSLRTNLPREAMGFRDYPFVAKDGKQRDTRRFPGHKEVLHYLEDFTSEFKLSELIRFETEVVHVGLVEEEAEGKWIVRSRGKNIIVEDEEVYDAVVVCNGHNTKPCVAEIPGIDVWPGKQLHSHNYRVPELFSNKVVVLIGSSMSAVDISREIARVAKEVHISSRSVTNKLLTNHFDFNYLWLHSMVKSAHEDGSVVFEDGSSVTADVILHCTGYTYDFPFLDSCSIVNVDDNRVGPLYKHVFPPQLAPSISFIGIPWKVVPFPLYELQSKWVAHILSGKVSLPSQENMMSDVQAFYSKFEADGGPKRYTHNVVDYQFEYYDCLAAQCGVPLPEEWRNQMLFATMMRLVAQPETYRDEWEDENLILQAHEDFAQYIPQANK

>FvH4_5g36500|Fvesca_v4.0.a2/1-468

MADASSAHSAEMHTLSRHVAVIGAGASGLVAARELRREGHSVVVFEQGDQLGGNWVYTPEVESDPIGLDPNRTIVHSSIYQSLRTNLPRELMGFRDYPFAAKEEEGRDPRRFPGHREVLMYLNDFASEFGICEMVRFETEVVFVRLVEGGKWMVKSKRNTSDEGLEEIYDAVVVCCGHYTKPRVAEIPGITTWKGKQIHSHNYRTPEPFRDQVVILIGRAASALDISRDIAGVAKEVHISSRSVASGTTEKKPDFATMRLNGMINGYDNMWLHSQIKSVHEDGSVVFTDGSVVVADTILHCTGYKYHFPFLDTNGIVTVDDNRVGPLFKHVFPPALAPSLSFVGVPWRTISFPNFELQSKWIAGVLSNRIALPSKQEMIENVRAFYSSLEAAGKPKRHTHDISDSLLILQGEYAGWLAAQCGCPISEWREQMILTVAKNWIVRPRTYQDEWDDNHLVLQAYEAFRNGV

>FvH4_5g36530|Fvesca_v4.0.a2/1-477

MADASSAESAATHTLSRHVAVIGAGASGLVAARELRREGHSIVVFERGDQLGGTWVYTPEVESDPIGLHPNRTIVHSSIYQSLRTNLPREIMGFRDYPFAVKEEDEGRDPRRFPGHREVLMYLDDFAGEFGISEMVRFETEVVFVGLVEGGKWMVRSKRKTSDEGLEEIYDAVVVCCGHYTKPRVAEIPGITTWKGKQIHSHNYRTPEPFRDQVIILIGRAASALDISRDIAGVAKEVHISSRSVASGTTEKKPDFATMRLNGMINGYDNMWLHSQIKSVHEDGRVVFTDGSVVVADTILHCTGYKYHFPFLDTNGIVTVDDNRVGPLFKHVFPPALAPSLSFVGVPWRTIPFPNFELQSKWIAGVLSNRIALPSKQEMIEDVRAFYSSVEAAGKPKRHTHDISDSQLILQGEYAGWLAAQCGCRIAEEWREQMILTVAKNWIVRPRTYQDEWDDNHLVLQAYEAFRNEVSLQKKKI

>FvH4_5g36860|Fvesca_v4.0.a2/1-477

MADPSSAHSDAMHTLSRHVAVIGAGASGLVAARELRREGHSVVVFERGDQLGGTWVYTPEVESDPIGLHPNRTIVHSSIYQSLRTNLPREIMGFRDYPFAVKEEDEGRDPRRFPGHREVLMYLNDFAGEYGISEMVRFETEVVFVGLVEGGKWMVKSKRKTSDEGLEEIYDAVVVCCGHYTKPRVAEIPGITTWKGKQIHSHNYRTPEPFRDQVVILIGSSASAIDISRGIAGVAKEVHISSRSVASETTEKKPDLATLCLTAMIDGHDNMWLHSQIKSVHGDGSSVVFEDGSVVVADTILHCTGYKYHFPFLETNGIVTVDEDDNRVGPLFKHVFPPALAPSLSFVGVPWRAIPFPCFELQSKWIAGVLSNRIALPSKEEMIEDVRAFYSSLEASGKPRRHTHDISKSSGEYSGWLAAQCGCPILSEEWREHMILAVAKNWIVRPKTYQDEWDDNHLVLQAYESFRTGVITSLQLM

>Nycol.D01468|Ncolorata_v1.2b/1-451

MGSTGTASDGLEAKEVAVIGAGAAGLVAARELKREGHRVVVFERGDTVGGTWVYTSEVDSGDLLGRDPSRMVVQSSLYKSLRTNLPRESMGFLDYPFTAVAGRDDRRFPSHEEVLAYLSDFALDFDLLKLIRFQTDVFHVTRVADGRWLVRSRKVLSGEEAVDASEVFDAVVVCSGHHTEPRIAEIPGVDRWPGKQIHSHNYRVPEPFEDKVVVLIGDAASGSDISRDIAPMAKEVHISSRSALIETTKRQPGSDNMWLHPTIKSAHEDGKVVFTDGSSVEADVILHCTGYQYHFSFLETNGFVTVDDNRVGPLYKHVFPPPLAPSLSFVGIPWKMLPFPMFELQCKWVAGILSEKISLPTEKEMMEDVEAFYSQIESVGYPKRYTHNMSECQFEYDDWLAMQCGCPMVESWRKGMYEAASVNRGARPETYRDEWDDHDLVLQAQECFLSL

>Nycol.D01469|Ncolorata_v1.2b/1-226

MAKEVHISSRSALIETTKIQPGFDNMWLHPTIKSAHEDGKVVFTDGSSVVADVILHCTGYQYHFPFLETNGVVTVDDNRLGPVYKHVFPPPLAPSLSFIGIPWKMLPFPMFELQSKWVAGILSEKVSLPTEKEMMEDVEAFYSQIEDVGYPQRYTHNMSEYQFEYDDWLATQCGCPMVESWRKGMYEAASVNRGARPETYRDEWDDHDLVLQAQESFLSLEKLKIR

>g12783.t2_1|Euryale_ferox/1-473

MGSKACDDGLEAKEVAVIGAGAAGLVAARELKREGHRVVVFERGDTAGGTWVYTSEVDYGDLLGRDPNRTVAQSSLYKFLRTILPRESMGFLDYPFTAVAGRDDRRFPGHEEVLAYLSDFSRDIELMELIRFRTDVFHLTRAADGRWLVRSWKVDAEEAVEVNEVFDAIVVCSGHNTEPRIAEIPGIDKWPGEKIHSHNCRVPEPFEGKVAVLIGDAASGSDISRDIAPMAKEVHISSRSTLIESTKRQPGFDNIWLHPTIKSAHEDGKVIFSDGSSVVADVVLHCTGYKYHFPFLETNGLVTVDDNRVGPLYKHVFPPSLAPSLSFIGLPWKMLPFPLFELQCKWVAGILSGKVSIPTEKEMMADVEAFYSQIEAAGYPKRYTHNMAEYQFEYDDWLAKQCVCPVVEGWRREMYGVASQNRGARPETYRDEWDDHDLVLQAQEHFLSLEKLKIRRWIPSRIWLAGVRMPEDE

>Nycol.D01470|Ncolorata_v1.2b/1-468

MAMPTTRTALTVAGSRSTVPRRTAAKEVAVIGAGAAGLVAAREMRREGHRVVVFELGDSVGGTWVYTPEVESDDLLGVDPKRMVVQTSLYRSLRTNLPREVMGFFDYPFKEVPGRDGRRFPGHEEVLAYLSDFARDFNLLKCIRFKTDVFHVSRVPDGKWLVQSRKVFDDDVSLDTNELFDGVLVCNGHYTEPRIADIPGVDRWPGKQIHSHNYRVPESFQGKVVVLIGSAASATDISRDIAPVAKEVHVSSRSASDETTKSQTGYNNMWLHPMIQGTLEDGTVIFNDGTSVIADVILHCTGYKYYFPFLDTNVIVTVDDNRVGPLYKHIFPPSLAPSLSFIGIPWKVVPFPLFELQSKWVAGILSGRIALPTEQEMMDDVEAFYTQLKATGYPKRYTHNMDGYQFEYDDWLATQCGYPKVENWRRKMYAEVSKRRRAQPETYRDEWDDHDLVLQAQEHFLSLKTLKI

>g08277.t1_1|Euryale_ferox/1-468

MAMPTARTAMTVAGSCSTVPRRAAAKEVAVIGAGAAGLAAAREMRREGHRVVVFERGDTVGGIWVYTPEVEFDDLLGVNSNRRVVQSSLYRSLRTNLPREVMGFFDYPFKEVPGRDVRRFPGHEEVLAYLSDFARDFNLVKCIRFKTDVFHVSKVPDGKWLVQSRKIFDDDVCLDVNELFDGVLVCNGHYAEPRIADIPGVDKWPGKQIHSHNYRVPEPFEGKVVVLIGSAASATDISRDIASVAKEVHVSSRSASDETTKRQPGFKNMWRHPLIQGTLDDGTVIFNDGTSVVADIILHCTGYKYYFPFLDTNGVVTVDDNRVGPLYKHVFPPSLAPSFSFIGIPWKVVPFPLFELQSKWVAGILSGRIALPTEQEMMEDVEAFYAQLEATGYPKRYTHNMDGYQFDYDDWLATQCGYPKVENWRRKMYAATSKRKRAQPETYRDEWDDHDLVAHAQEHFLSLETLKI

>Nycol.L00801|Ncolorata_v1.2b/1-459

MVIEALVPAARKRAGKEVAVIGAGAAGLAAGRELRREGHRVVVFERGNTVGGTWVYTPEVESPDLLGVDPNRTLVHSSVYESLRTNLPREVMGFLDYPFSVRPGCDGRRFPSHQEVAAYLADFAADFDLVRWIRLETEVVNVVQHPDGRWLVRSRRALLNRECTPPSAAVIEEVFDAVVVCNGHYTEPRLAVIPGIEKWRGKQMHSHNYRVPGPFADKVVVLIGGYASAKDISRDIAPVAKEVHVSSRTESGELPRMQPGYANMWLHSLIKSTHEDGGVVFSDGSSVIADIIIHCTGYKFRFPFLDSNGAVTVDDNRVGPLYKHVFPPSLAPSLSFIGIPWKVYPFPLFELQSKWVAGVLSGRISLPSEREMLEDVEAFYLQIEAAGYPKRYTHNISSYQSEYDDWLSMQCGCMKVEEWRKEMFAATYKNRIARPENYRDEWDDHDLVLQAQEHFLTIE

>gene_prefix21531.t1_1|Ceratophyllum_demersum/1-472

MMSMAISSAPNVSLPVHANTIERSSRRSKQLAVIGAGAAGLVAARELRREGHEVVVFERLYNVGGIWNYDPNIESDLLGIDSSRDVVHGSLYKSLRTNLPREVMGFRDYPFIATENGLDSRRFPTHREVCRYLHDFSRDFNLMDLIRLGTEVFDIGLLDDGGWMVKSRSVEKDSDVITEVYDGVVVCNGHYTEPRIAEIPGVDKWPGKQMHSHNYRVPDQFQGQVAVLIGNSASAVDISKDIYEVTKEVHIASRSTTVATPIKQPGYENMWLHSMIESAHEDGTIVFQDGSSVVADVILHCTGYKYHFPFLKTNGIVTVDDNCVGPLYKHVFPPSLAPWLSFIGLPWKVAPFPLFELQSKWLAGILSGWITLPSEQEMMDDVKAFYLKLESIGYPKRYTHNLTEYQFDYDDWLASKSGCPEIEEWRKQMYMSTSQNRKAHPETYRDEWDDHYWISHAHEDFSKVSCSNNSSQ

>Brara.I01444|BrapaFPsc_v1.3/1-449

MAPSLSPLRSHHVAVIGAGAAGLVAARELRREGHSVVVFERQKQVGGTWIYTDHVEPDPLSVDPTRPVVHSSVYATLRTNLPRECMGYRDFPFVVRSGGDQRRFPSHGEVLAYLQEFAKEFKIEEVIRFETAVERVAPATEGGSGKWRVESTETEKRVRRDEVYDAVVVCNGHYIEPRLAEIPGISSWPGKEMHSHNYRTPEPFKDQVVVLIGNSASAVDISRDIAGHAKEVHVAGRSNPADTYIKQPGYINLWMHSMIERVNKDGSVVFQNGQTILVDVIMHCTGYKYHFPFLETNGSVTVDDNRVGPLYKHVFPPELAPWLSFIGIPWQVVPFPLFELQSKWIAGVLSGRIMLPSKDDMMEDIKSLYATLEAQGIQKRYTHRMGIAQFEYNDWLATQCGCPGTEEWRKEMYLTTGVRKRTTPETYRDEWEDHHLISQASQDFSLYTH

>Brara.I01443|BrapaFPsc_v1.3/1-449

MAPSLSPTRSHHVAVIGAGAAGLVAARELRREGHSVVVFERQKQVGGTWIYTDHVETDPLSVDPNRTVVHSSVYASLRTNLPRECMGYLEFPLVVRSGDPRRFPSHGEVLAYLQDFAKEFAIEEVIRFETAVERVSPAKEGGNGKWRVESTETEKRVRRDEVYDAVVVCSGHYIEPRLADVPGISSWPGKEMHSHNYRIPEPFKHQVVVLIGNSASAVDISRDIAGHAKEVHVAGRSNAADTFIKQPGYSNLWMHSMIDRVHEDGSVDFQNGKTILVDVIMHCTGYKYHYPFLETNGSVTVVDNRVGPLYKDVFPPALAPYLSFIGIPSQVSPFRLFELQSKWIAGVLSGRIILPSKEDMMKDIKSFYATLEAQGIPKRYTHRTQFEYNDWLASQCGCSEKEQWRKEMYLTTGVRKKAHPETYRDEWEDHHLVSQAYQDFSLYTLTCNM

>AT1G62620|Athaliana_Araport11/1-450

MAPALSPTRSHHVAVIGAGPAGLVAARELRREGHSVVVFEKQKQVGGTWIYTDEVESDPLSVDPTRSVVHSSVYRSLRINGTRECTGYRDFPFVVRSGVSRDPRRFPSHGEVLAYLKDFAKEFGIEEMVRFETEVVKVSPAAEEGIGKWRIESTEKEKKVRRDEIYDAVVVCNGHYVEPRLAQIPGISSWPGKEMHSHNYRIPEPFRDKVVVLIGNSSSAEDISRDIARVAKEVHVACRSNPADTFIKQTGYNNLWTHSMIESVHEDGSVVYQNGKTISVDIIMHCTGYKYHFPFLDTNGIVTVDDNRVGPLYKDVFPPAFAPWLSFIGIPWQVLPFPMFELQSKWIAGVLSGRIPLPSKEDMMIEIKTFYSTLEVQGIPKRYTHRMGNTQFEYYNWLASQCGCSETEEWRKEMCLANGVRKEAHPETYRDEWDDHHLVSEAYQDFSLYS

>AT1G63370|Athaliana_Araport11/1-450

MAPALSPTRSHHVAVIGAGPAGLVAARELRREGHSVVVFEKQKQVGGTWIYTDEVESDPLSVDPTRSVVHSSVYRSLRINGTRECTGYRDFPFVVRSGVSRDRRRFPSHGEVLAYLKDFAKEFGIEEMVRFETEVVKVSPAAEEGIGKWRIESTEKEKKVRRDEIYDAVVVCNGHYVEPRLAQIPGISSWPGKEMHSHNYRIPEPFRDKVAVLIGNSSSAEDISRDIARVAKEVHVACRSNPADTFIKQTGYNNLWTHSMIESVHEDGSVVYQNGKTISVDIIMHCTGYKYHFPFLDTNGIVTVDDNRVGPLYKDVFPPAFAPWLSFIGIPWQVLPFPMFELQSKWIAGVLSGRIPLPSKEDMMIEIKTFYSTLEVQGIPKRYTHRMGNTQFEYDNWLASQCGCSETEEWRKEMCLANGVRKEAHPETYRDEWDDHHLVSEAYQDFSLYS

>AT1G62600|Athaliana_Araport11/1-452

MAPSLSPIRSHHVAVIGAGAAGLVAARELRREGHSVVVFERQKQVGGTWIYTDHIEPDPLSVDPTRSVVHSSVYGSLRTNLPRECMGYRDFPFVIRSDVSESRDPRRFPSHGEVLAYLQDFAKEFAIEEMIRFDTAVVKVAPAAEEGSGKWRIESTEKEKKVLRDEIYDAVVVCNGHYIEPRHAEIPGISSWPGKEMHSHNYRIPEPFRDQVVVLIGNSASADDISRDIARVAKEVHVACRSNAADTYIERPGYSNLWMHSMIESVHEDGSVVFQNGKTISVDVIMHCTGYKYHFPFLETNGNVTVDDNRVGPLYKDVFSPAFAPWLSFVGIPWKVVPFPMFELQSKWIAGVLSGRIPLPSKEDMMMEIKTLYSTLDAQGIAKRYTHQMGISQFEYNSWLASQCGCSETEEWRKEMYFATGVKKRAHPETYRDEWDDHHLVSQAYQDFSLYT

>GWHPCBIR019223|Santalum_yasi/1-491

MAAPLQPERSVPYPKRARLCRPPMMASWGLRTPLPVLPTISQPIRSGHVAVIGAGAGGLVAARELRREGLGVAVFERGDRIGGTWVYDPITESDPLGIDPGREIIHSSLYASLRTNLPRESMGFRDFPFVAVESDRRDPRRFPGHAEVLCYLEDFAREFGLGDSIRFGTEVLSVVLTEEGKWKVRFRRRIGGDEEDEVFDAVMVCSGHYTEPRIAEISGINTWPGKQMHSHNYRIPEPFQDQVVILIGSSASAVDISSEIAGVAKEVHIATRSHGITGMQPCYENMRFHSMIESACTDGSVTFKDGSVVVADVILHCTGYKYHFPFLETNGVVAVDDNRVGPLYKHAFPPSLAPWLSFVGLPWKVIPFPLIELQSKWVAGILSGRVALPPEKEMMKDVESFYSKLEASGTPKRYTHNISDYQFEYDDWLAAECGVAPSEQWRKQMYVAVSKNRLLRPDTYRDEWEDEHLVAEALEDFANHCSNGNCRRLGS

>Glyma.11G031100|Gmax_Wm82.a6.v1/1-457

MACEVPIKSMMSSAVTLLTPRHVAVIGAGAAGLVAARELRREGHRVVVFEKGEEVGGMWVYSPEVDSDPLGLEAKRRLVHSSLYDSLRTNLSRESMSFRDYPFRRREGKGRDSRRFPGHREVLLYLQDFAAEFEIGELVRFGTEVLFAGLDQCGKWRLTSTSPHTHPVDEIYDALIICNGHYVQPRLPHIPGINAWPGKQMHSHNYRTPEPFQDQVVVLIGSSASAVDISRDIATVAKEVHIAARSVEEDKLGKVPGHENMWLHSMIDSVHEDGTVVFQDGNAVGADFIIHCTGYKYDFPFLETNGEVTVDDNRVGPLYKHVFPPALAPWLSFVGLPWKVAPFSLFELQSKWIAGILSNRIALPSKEEMAKDVDAFYSSLEASGTPKRYTHNMGILQWDYNNWIADQCGVPSIEEWRRQMYIATSKNRVLRPESYRDEWDDDDLVLQAQQDFANYLT

>KAI5439775.1|Pisum_sativum/1-455

MPIKFIMSTATPLLTPRHVAVIGAGAGGLVAARELRREGHQVVVFDRGDQLGGSWVYTAEVESDPIGKDPKRRIVHSSLYNSLRTNLPRESMGFRDYPFRRKEGKERDSRRFPSHIEVLKYLQDFAADFEINDSVRLQTEVVYAGLDEGGKWTVRSRSIENDCVDEIYDAVVVCNGHYFQPRLPHITGISSWPGKQMHSHNYRTPEPFQDQVVVLIGGAASGVDICRDVATVAKEVHVAARSVEEDKLGKLPGHDNIWLHSMIDRVHEDGTVVFLDGNALIADFIVHCTGYKYEFPFLETRGVVTVDDNRVGPLYKHVFPPALAPWLSFVGLPWKVAPFPLFELQSKWIAGVLSNRIALPSEEEMVKDIEDFYMSLEASGTPKSHTHNMSKNLAQWDYNNWIADQCGVPAMEEWRKQMYIATSKNRLTRPETYRDEWDDDDLVQQAEHEFANYVI

>Soltu.DM.06G017750|Stuberosum_v6.1/1-480

MILRSVISSNQPYTRILLSHFPILSMAQNSSKNVAVIGAGSAGLVTARELKREGHSVVVFEQENQLGGTWVYTPATESDPVGIDPNREIVHSSLYSSLRVNLPREVMGFWDYPFVAKKKPGRDPRRYPGHGEVLNYLNDFAVDFGIIGVVRFGMEVGFVGKMENGKWKVSCRKRENDVMFANEEYDAVVICNGHYTEPRIADIPGIEVWPGKQIHSHNYRVPDPFRDQVVVLIGGAASATDISREIAEVAKEVHISSRSATSGVPMKLPGYDNIWLHNMIEAVGSDGGVNFQDGSKILADIILHCTGYKYHFPFLETNGIVTVDDNRVGPLYKHVFPPTFAPSLSFVGLPWKVIPFFLCELQSKWIAGVLSGRISLPSKEDMNADIEAFYSSMAASCIPKRYTHNMDDSQFDYDDWLAAECGSPPSEEWRKQMYFISRKNKKTLPETYRDEWDDDDLIIQAHEDFVKYIPELSQEQKLSR

>Solyc06G001318|Slycopersicum_ITAG5.0/1-455

MSQNSSKNVAVIGAGSAGLVAARELQREGHRVVVFERENQLGGTWVYTPDTESDPVGIDPNREIVHSSLYSSLRVNLPREVMGFGDYPFVAKKKPGRDPRRYPSHGEVLEYLNDFAVDFGIIGVVRFGMEVGFVGKMENGKWKVSCRKRENDDLFANEEYDAVVICNGHYTEPRIADIPGIEVWPGKQIHSHNYRVPDPFRDQVVVLIGGAASATDISREIAEVAKEVHISSRSATSGVPMKLPGYDNIWLHNMIEAVGSDGGVNFQDGSKILADIILHCTGYKYHFPFLETNGIVTVDDNRVGPLYKHVFPPAFAPSLSFVGLPWKVIPFFLCELQSKWIAGVLSGRISLPSKEDMNADIEAFYSSMAASCIPKRYTHNMDDSQFDYDDWLAAQCGSTPFEEWRKQMYLISRKNKRTLPETYRDEWDDDDLIIQAHEDFVKYIPELAQEQKLSR

>Soltu.DM.06G018770|Stuberosum_v6.1/1-456

MSQNSPKNVAVIGAGPAGLVAARELKREGHIVVVFERENQLGGTWIYTPHTDSDPIGIDPNRRIVHSSLYSSLRVNLPREVMGFRDYPFVATKKPNRDPRRYPGHREVLDYLNDFASDFGLIELVRFGTEVGYVGLLENGKWKVSSRKSENDDHVVFVNEEYDAVVICNGRFTEPQIADIPGIEVWPGKQIHSHNYRVPEPFRDQVIVVIGAATSASDISREIAEVAKEVHISSRSALSGTPKKVHGYENLWLHSMIEAVGIDGGVNFHDGSKVHADIIIYCTGYKYHFPFLETNGIVTVDDNRVGPLYKHIFPPAFAPSLSFVGMPWKLIPFPRCELQSKWIAGVLSGRISLLSKEDMIADIDAFYSSLDASCIPKRYTHNMDFQLDYEDWLAAECGSPPPEKWRKEMFFIAREKIKTQTERYRDQWDDDDLIIQAHQDFVQFIPELPQVQKLST

>Asa8G05386.1|Allium_sativum/1-446

MPVTPLSLVTRHVAIIGAGAAGLVTARELRREGHTTTIFERGSSIGGTWIYTPDTEPDPMSQDSSRPIVHSSLYKSLRTNLPREVMGFLDYPFVEKTNGGDRRRFPGHEEVLDYLERFGREFGVSREVGMEKEVVRVDMEQGGKWTVKWKGKDGGGGEEGFDAVVVCNGHYTEPRFAEIPGIDVWPGKQMHSHNYRIPEPFHDQVVVIIGSSASAVDISRDVARFAKEVHIANRSITEGTPAKQPGYDNMWLHSMIKITHNDGSVVFHDGCSVHVDVIMHCTGYVYNFPFLNTNGIVTVDDNRVGPLYKHVFPPLLAPSLSFVGIPWKIVPFPLCELQSKWIAAVLSGRISLPTKKEMMEDVEAYYKQMEAAGIPKRYTHNIGHNQFDYDDWLANECGYSCIEEWRRLMYKEVSKNRKERPESYRDEWDDDHLVAQARETFSKFLS

>LOC_Os10g40570|Osativa_v7.0/1-482

MKTPQNDKLQYHTPPLPSKSQQKSEAKSRTHRLAMPSPSLRLAVVGAGAAGLVAARELRREGHSPVVFERAASVGGTWLYDAAPATSDPLAAGAAHSSLYASLRTNLPREVMGFLDFPFASSAAEAGGGGDTRRFPGHDEVLRYLEEFARRFDLYGLVRFGTEVVRVRRDGGGGGGRWAVTSRKIGEKGRREEEEEVYDAIVVCNGHYTEPRVAHIPGVEAWPGKQMHSHNYRVPEPFHDQVVIIIGASASAVDISRDLAGVAEEVHVADRSAPACTCKRQPGYDNMWLHSMIDHAQEDGCVVFQDGSSIKADVIMHCTGYLYDFPFLEDDSAITVDDNCVDPLYKHVFPPEVAPHLSFIGLPWKVIPFPLFELQSKWVAGVLSGRVKLPSSEEMMEDVKAFHSKMEARGWPKRYAHNFSDCQFEYDDWLAEQCGHPPIEQWRKLMYAANSENKAARPESYRDEWDDDHLVAEAAEDFKKYL

>Zm00001d029865|Zmays_RefGen_V4/1-440

MPSASLRLAVVGAGAAGLVAARELRREGHAPVVFERAAAVGGTWLYTPPATSSDPLGAAATHSSLYASLRTNLPRETMGFLDFPFAAGAAGSRDPRRFPGHEEVLRYLEAFARRFDLLRLVRFETEVLSVRREDGGRWAVTSRKLGDKGSGEEEFYDAVVVCNGHYTEPRLAVIPGVDAWPGKQMHSHNYRVPEPFLDQVVIIIGASASAVDISRDIASMAEEVHIADRSAPASTCNKQPGYDNLWLHSMIDHAQEDGTVVFQDGSSIKADVIMHCTGYLYDFPFLGDDSTITVDDNRVDPLYKHIFPPEVAPHLSFIGLPWKVIPFPLFELQSKWVARVLSGRINLPSEDRMMEDVKAFYLKLEAHGWPKRYTHNFANYQFEYDDWLAEQCGHPPVEEWRKQMYAVTSMNKAARPESYRDEWDDEHLVAEANEYFKKFL

>Sobic.001G303200|Sbicolor_v5.1/1-445

MPSASLRLAVVGAGAAGLVAARELRREGHAPVVFERAAAVGGTWLYTPPATSSDPLGAAATHSSLYASLRTNLPRETMGFLDFPFAAGAAAAGSPRDPRRFPGHEEVLRYLEAFARRFDLLRLVRFETEVLSVRREDGGGGRWAVTSRKLGEKGSGQEEFYDAVVVCNGHYTEPRIAVIPGVDAWPGKQMHSHNYRVPEPFLDQVVIVIGASASAFDISRDIASMAEEVHIADRSAPASTCKKEPGYDNLWLHSMIDHAQEDGTVVFQDGSSIKADVIMHCTGYLYDFPFLGDDSTITVDDNRVDPLYKHIFPPEVAPHLSFIGLPWKVIPFPLFELQSKWVARVLSGRVKLPSKDKMMEDVKAFYLKLEALGWPKRYTHNFSNHQFEYDDWLAEQCGYPPIEEWRKQMYAVNAMNKAARPESYRDEWDDEHLVAEANEYFKKFL

>ELECO.r07.2AG0116030|Ecoracana_v1.1/1-441

MPSASLRLAVVGAGAAGLAAARELRREGHAPVVFERAAAVGGTWLYTPPATAADPLGAAATHSSLYASLRTNLPREVMGFLDFPFVASAATGSADPRRFPGHEEVLRYLEAFARRFDLLPLVRFETEVLRVRREPGVGWAVASRKLGEKGSGEEEVYDAVVVCNGHYTEPRVATIPGVHTWPGKQMHSHNYREPEPFLDQVVIIIGASASAVDISRDVASVAKEVHIADRSAPASTNQKQPGYDNLWLHSMIDRAQDDGSVVFRDGSLIKADVIMHCTGYLYDFPFLGDDSTINVDDNRVDPLYKHVFPPEVAPQLSFIGLPWKVIPFPLFELQSKWVAGVLSGRIELPSKEEMMEDVKAFYLNLEALGWPKRYTHNFSNYQFEYDDWLAEQCGYPPIEEWRKLMYAATTKNKAARPESYRDEWDDDHLAIEANEDFKKYL

>ELECO.r07.2BG0169860|Ecoracana_v1.1/1-440

MPSASLRLAVVGAGAAGLAAARELRREGHAPVVFERAAAVGGTWLYTPPATATDPLGAAATHSSLYASLRTNLPREVMGFLDFPFVASAATSSADPRRFPGHEEVLRYLEAFARRFDLLSLIRFETEVLRIRREPGVGWAVASKKLGEKGSGEEEVYDAVVVCNGHYTEPRVATIPGVDAWPGKQMHSHNYREPEPFLDQIVIIIGASASAVDISRDIASVAKEVHIADRSAPASTNQKQPGYNLWLHSMIDRTQEDGSVVFRDGSSVKADVIMHCTGYLYDFPFLGDDSTITVDDNRVDPLYKHVFPAEVAPQLSFIGLPWKVIPFPLFELQSKWVAGVLSGRIDLPSKEEMMEDVKAFYLNLEALGWPKRYTHNFSNYQFEYDDWLAEQCSYPPIEEWRKLMYAASTKNKAARPESYRDEWDDDHLVVEASEDFKKYL

>HORVU.MOREX.r3.1HG0056290|Hvulgare_Morex_V3/1-482

MNELNQQRRDRTRLLDEKKQGRHGQFQLRKPLCSLLPASMPSPSLRLAVVGAGAAGLAAARELRREGHAPVVFERAAAVGGTWLYASPAPAAATDPLGAAATHSSLYASLRTNLPREVMGFLDFPFTAARGSVVDARRFPGHEEVLRYLEDFAQRFDLYGLVRFQTEVVGVRRVAGGRWAVTSRKLGEKGEQEEEVYDAVVVCNGHYSEPRIASIPGADAWPGKQMHSHNYRVPEPFLDQVVIVIGASASAVDISRDIASVAKEVHIADRSAPTSTCEQQPEYDNMWLHSMIDHAQGDGSVVFQDGSSIKADVIMHCTGYLYDFPFLGDDSTITVDDNCVDPLYKHVFPIEVAPDLSFIGLPWKVIPFPLFELQSKWVAGILSGRIKLPTKDEMMEDVKAMYSRRETRGWPKRYTHNFSGGYQFEYDDWLAEQCGHPPIEEWRKLMYAANAKNKAARPERYRDEWDDDDLVAQASEDFKKYL

>Bradi3g32990|Bdistachyon_v3.2/1-481

MSSKPTELQSYTTPRREKPPDPTANPNHSASQLHKPTMPSPSLRLAVVGAGAAGLVAARELRREGHAPVVFERAAAVGGTWLYASPSHADPLGAAATHSSLYASLRTNLPREVMGFLDFPFTAPKLSSPAAAIDARRFPGHREVLRYLEDFARRFDLCDLVRFETEVVGVRREASGRWAVTSKKLGEKGELDEEIYDAVVVCNGHYTEPRVASIPGADTWPGKQLHSHSYRVPEPFVDQVVIVIGASASAVDISRDIASVAKEVHIADRSAPASTCELQPGYNNMWLHSMTDRAQEDGCVVFRDGSTIKVDVIMHCTGYLYDFPFLGDDSTITVEDNCVDPLYKHVFPIEVAPDLSFIGLPWKIIPFPLFELQSKWVAGVLSGRINLPSKSEMMEDVKAIYLRREAHGWPKRYTHNFSGDYQFEYDDWLAEQCGHPPIEEWRKMMYAANARNKAARPENYRDEWDDDHLVAQANEDFRKHL

>evm.TU.AsparagusV1_10.11|Aofficinalis_V1.1/1-381

MGFFDYPFVAVEDGREGDSRRFPRHQEVLRYLEDFARDFEVLEVVRFRTEVVRVEMEEEGKWAVRWRRVAAGEDGGGEITGGGEDQVEEEEEEEIYDGVVVCNGHYTEPRLAAIPGIDIWPGKQMHSHNYRVPEPFRDQVVVIIGSAASAVDISNEIAQFAKEVHVASRSDTEGTPKRQPGYGNLWLHSMIESTHEDGTVVFQDGSSTHVDVIMHCTGYKYHFPFLQTHGIVTVDDNRVGPLYKHVFPPALVPYLSFIGIPWKVAPFPLVELQSKWVAAVLSGRVSLPTRKVMMEDVDAFYKELETAGYPKRYTHIMTNYQYEYIDWLANECGYPPTEEWRKLMYKATSKNRKERPGSYRDEWDDDHLVAQAHEYFKRFFK

>VIT_202s0154g00170|Vvinifera_v2.1/1-451

MSPTISKIKSLNVAIIGAGAAGLVSARELRREGHQVVVFERQAQLGGTWVYNPGVEADPLGSDPSRAIVHSSLYASLRTNLPREAMGFRAYPFVSTGQPHRDSRRFPGHQEVLLYLNDYATEFGLTELVRFETEVVYAGLFEDGKWKVRSRQENGVAVDVDEIFDALVVCNGHYTEPRTAEIPGIDAWPGKQMHSHNYRIPEPYRDLVVILIGSGPSALDISIDIAQVAKEVHVASRSDEAEVLRKQFGYHHIQLHPMIESVQKDGTVIFYDGSMVLADVILHCTGYKYHLPFLDTHGIVTVDDNCVGPLYKHIFPPVLAPRLSFVGLPWMVLPFPMFEFQSKWIAGVLSDRIRLPSQEEMMENVSAFYLSLEASGMPKRHTHKMGAHLFEYNGWLAAECGVPVTEEWRKKMLYATFQNYLAHPGTYRDAWEDDDLVLEALMEMATVATLD

>VIT_202s0154g00190|Vvinifera_v2.1/1-468

MPPPTLLYYLPMAPSISLFKSRDVAVIGAGAAGLVAARELRREGHKVVVFERERQVGGTWVYTPTVETDPLGSDPSRHIVHSSLYASLRTNLPREVMGFLDYPFVSTGEPHRDPRRFPGHREVSLYLKDFAVGFGLNELIRFETEVVYAGLVEDEKWRVKSRSGNDAAIDVEEIFDAVVVCNGHYTEPRLAEIPGIDAWPGKHMHSHNYRIPEPFRDQVVVLIGGAASAVDISMDIAQVAKAVHIASRSVEAGILKKLSGNAIDNMWLHPMIESVQKDGTVIFYDGSVVLADVILHCTGYKYHFPFLDTSGIVTVDDNRVGPLYKHIFPPHLAPGLSFVGLPWKVLPFPMFEFQSKWIAGALSGRIGLPSQEEMMADVSAFYLSLEASDTPKHYTHNLADSQFEYDDWLALECGIPGVEEWRKKMYEATAKNKKVRPDKYRDKWEDEDLMLEAQKDFAGCRLNGAGDN

>VIT_200s0233g00180|Vvinifera_v2.1/1-468

MPPPTLLHFLPMAPSISLFKSRDVAVIGAGAAGLVAARELRREGHKVVVFERERQVGGTWVYTPTVETDPLGADPSRHIVHSSLYASLRTNLPREVMGFLDYPFVSTGEPHRDPRRFPGHREVSLYLKDFVVGFGLNELIRFETEVVYAGLVEDEKWGVKSRSGNDAAIDVEEIFDAVVVCNGHYTEPRLAEIPGIDAWPGKHMHSHNYRTPEPFRDQVVVLIGSAASAVDISMDIAQVAKAVHIASRSVEAGILEKLSGNAVDNMWLHPMIESVQKDGTVIFYDGSVVLADVILHCTGYKYHFPFLDTSGIVTVDDNRVGPLYKHIFPPHLAPGLSFVGLLWKVLPFPMFEFQSKWIAGALSGRIGLPSQEEMMADVSAFYLSREASDTPKHYTHNLADSQFEYDDWLALECGIPGVEEWRKKMYQATAKNKKVRPDKYRDEWEDEDLTLEAQKDFARCRPNGGGDN

>orange1.1g013303m.g|Csinensis_v1.1/1-446

MFRHVAVIGAGAAGLVVGHELLREGHTVVVYEKGEQVGGSWIYTSETESDPLGVDPNRYPVHSSLYKSLRVNLPRELMGFQAYPFVARNYEGSVDLRRYPGHEEVLRYLQNFAREFGVDQVVRLHTEVLNARLVESNKWKVKSRKKDDVVEEETFDAVVVCNGHFSVPRLAQVPGIDSWPGKQMHSHNYRIPNPFQDQVVILIGHYASGLDIKRDLAGFAKEVHIASRSVADETHEKQPGYDNMWLHSMVERANEDGTVVFRNGRVVSADVIMHCTGYKYNYPFLETNGIVTVDDNRVGPLYKHVFPPVLAPGLSFVGIPQKVIPFPFFELQSKWIASVLSGRIVLPSQDEMMEDVKAFYSKLEASGKPKRYTHIMDYPQLIEYTDWLAAQCNCQGYEEWRKQMAYSAFKNAFITRPGTYRDEWDDEHLVAEANKDFIKYTSNKSK

>Potri.001G121000|Ptrichocarpa_v4.1/1-452

MQTSNATSLTSRHVAVIGAGAAGLVAARELRREGHQVVVFEKDSQIGGTWVYTPQVETDPLGLDPTRHIVHTSLYKSLRTNLPRESMGFMDYPFVTRAGEGSDPRRFPGHAEVLKYLQDFAREFGIEEMVRFECEVVSVEMVDNEKLKVKCKRMRPDGGDDDLLDEVFDAVVVCNGHFTYPRIAEIPGINLWPGKQIHSHNYRTPELFKDKVVILIGSSASAIDLSLEIGGIAKEVHIASRSVANDTYEKRAECDNIWLHSMIKSAHKDGSVAFRDGNTIVADIILHCTGYKYYFPFLKTNGIVTVDDNRVGPLYKHVFPPIFAPQLSFVGLPYRSLPFPIFEIQSKWISGVLSDRIVLPSQEDMMEDVNTFYSTLEDSGVPKHHTHSMGDTMIDYNAWVASLCQCPCFEEWRVQMFYETAKRLNANPKTFRDEWEDDNLVLQACEDFSKYI

>Potri.013G019900|Ptrichocarpa_v4.1/1-464

MQASPNSLSSHHVAVIGAGAAGLVAARELHREGHKVVVFEKDDQVGGLWMYDPRVEPDPLGLDLTRPVVHSSLYESLRTNLPRETMGFMDYPFVTREGEGRDPRRFPGHREVLMYLQDYAREFGIEEMVRFGCEVVNVEMIDSGKWKVKSKRKRLDDNDRGDDFADHEDFDAVVVCVGHYTQPRIAEIPGINLWPGKQIHSHNYRIPEPFRDQIIILIGASASAADISVEIAGLAKEVHIARRSAVDDDTYEKKPGYDNIWLHSTIERACEDGTVIFRDGSVILADVILHCTGYKYGFPFLKTDGIVTVDDNRVGPLYKHVFPPILAPWLSFVGIPYWTFPFPTFEVQSKWIAGVLSGRIALPSQEDMVEDVKIYYSELEASGVPKHHTHNLAHSTNDYNMWLASQCQCSCFEEWRIEMSHEILKNWRARPNMYRDEWDDDHLILQAQEDFNRRISNKASNGHI

>AUR62002282|Cquinoa_v1.0/1-467

MPLPSTSKKVAVIGAGAAGLVAARELKKEGHNVEVFERSAQLGGCWVYSPEVESDPLGLDPTRKVVGSSLYASLRTNIPREAMGFRDFPFFPTGKVDRDSRRFPGHQEVLRYLEDFAFEFGLNELISYQTEVRHVGLETDGKWMVKYRRGGGDESSDVAKTYDAVVVCNGHYTEPRVADNIPGIDLWPGRQIHSHNYRIPEPYQDQVVVLIGFSASAIDICRDIAVSAKQVHVATRSEAAGKFHKFGKQPVYDNLWFHPMIEKAHEDGRVSFQDGSVVLADVFLHCTGYKQHFPFLHTSGAVNVDDNRVGPLYKHVFPPALDPGISFIGVPSLVIPFFLFELQSKWVAGILSGRISLPSKEKMMKDVEAFYSELEAKEVPKRYTHRIHDIKRFFGSFEYEDWLAAECGCPPAEEWRKGMFFGTFVRVIKQIETYRDEWDDEDLVLQAYEDFMQYLSAESRAAIAATL

>AUR62015414|Cquinoa_v1.0/1-467

MPLPSTPKKVAVIGAGAAGLVAARELKKEGHNVEVFERNTRLGGCWVYSPEVESDPLGLDPTRKIVESSLYASLRTNLPREVMGFRDFPFIPTGKVNRDSRRFPVHQEVLRYLEDFAFQFGLNELIRYQTEVRHVGLETDGKWMVKYRRVGGDEGSDVAKTYDAVVVCNGHYTEPRVADNIPGIDLWPGRQIHSHNYRTPEPYQDQVVVLIGLSASAIDICRDIAVSAKEVHVASRSEAAGILQKLGKHPVYDNLWFHPMIEKAHEDGRVSFQDGSVVLADVILHCTGYKQHFPFLHTNGSVNVDDNRVEPLYKHVFPPALAPGISFIGLPLLVLPFFLFELQSKWVAGVLSGRISLPSKEKMMEDVEALYSELEAKGVPKRHTHRIHDAKRLFGPFEYEDWLAAECGCPPAEEWRKGMFYGTFVRLIKQTETYRDEWDDEDLVLQAYEDFMQYLSAEGRAAIAAAL

>AUR62002280|Cquinoa_v1.0/1-287

IDLWPGRQIHSHNYRTPELYQDQVVVLIGLASSVFDISRDIAVSAKEVHVTTRSEAAGKLAKFGKHPGYDNLWLHPMIEQAHEDGRISFQDGSVVLADAIIHCTGYKQHFPFLHTNGVVNVDDNRVGPLYKHVFPPALAPGISFIGVPFNVDLFFLFELQSKWVGGILSGRLSLPSEEKMMEDVEAFYSELEAKGVPKRYTHRIHDFKQLCGWSYEYEDWLASESGCPPSEEWRRGMHIATVGRAFKQTETFRDEWDDEDLVLQAYKDFMQDSSAEGRVAVAPSTQR

>AUR62015416|Cquinoa_v1.0/1-470

MTSPLTFKKVAVIGAGPAGLMAAHMLRNEGLDVVVFERAAQLGGCWVYSAEVESDLLGHDPTRKIVPSSVYASLRTNSPRESMGFRIFPFVPTGKVGRDSRRFPGHQEVLRYLEDFAYEFGLNKLICYQTDVRHVGLETDGKWMVKYRRVGGDKGSDMAETYDAVVVCNGHYTEPQLADNIPGIDLWPGRQIHSHNYRVPELYQDQVVVLIGHASSAVDISRDIAGSAKEVHVTTRSEAAGKLAKFGKHPVYDNLWLHPMIEQAHEDGRISFQDGSVALADVIIHCTGYKQHFPFLHTNGVVNVDDNRVGPLYKHVFPPALAPGISFIGVPFYVDPFFLFELQSKWVAGVLSGRLSLPSEEKMMEDVEAFYSELEAKGIPKRFTHRIHDFKQLSGWSYEYEDWLASESGCPPAEEWRKGMYIASVDRAFKQTETFRDEWDDEDLVLQAYKDFMQYLSAEGRVTVAASTLR

>AUR62002281|Cquinoa_v1.0/1-472

MTVPLSTKKVVVIGAGAAGLVAARELIKEGHDVVVFDRGAQLGGNWVYSDEVECDPLGFDPTRKIVDSSLYASLRTNTPREAMGFRDFPFVPTGKADRDSRRFPSHHEVRKYLEDFASEFGLNELISYETEVRYVGLETDGKWMVKSTSVGGDESSEVAQTYDAVVVCNGHFTQPQVADNIAGFDLWPGRQIHSHNYRIPEPYKDQVVVVIGLSSSAVDISREIALSAKEVHVTTRSKAAGKLGKFGKHHIFDNLWLHPMIERAHEDGRVSFQDGSSVLADVILHCTGYKQHFPFLHTNGVVNVDDNRVGPLYKHVFPPSLAPGISFIGVPSLILSFFVYELQSKWVAGVLSGRISLPSEEKMMEDVEAFYSKLEAKGVPKRYTHKIHDVKQLSWSLEYEDWLAAQCGCPLAEEWRKAIFFGRLVHAHKEPETYRDEWDDQDLVFQAYEDFMQYLNAEGRAALAACASTPRI

>AUR62015415|Cquinoa_v1.0/1-472

MTVPLSTKKVAVIGAGASGLVAARELMKEGHDVVVFDRGAQLGGNWVYLDEVESDPLGLDPTRKIIDSSLYASLRTNTPREAMGFRDFPFAPTGKVDRDSRRFPGHHEVRRYLEDFASEFGLNELISFRTEVRHVGLETDGKWMVKYASVGGDESTEVAQTYDAVVVCTGHFTEPQVADNIPGLDLWPGRQIHSHNYRIPEPYQDKVVVVIGLSSSATDISREIARSAKEVHVTTRSKAAGKLGKFGKHDIFHNLWLHPMIERAHEDGKVSFQDGSTVFADIILHCTGYKQHFPFLDTNGVVSVDDNRVGPLYKHVFPPSLAPGISFIGVPSLILSFFVYELQSKWVAGVLSGRISLPSEEKMMEDVEAFYSKLDAKGVPKRYTHKIHDVKQLSWSLEYEDWLAAQCGCPLAEEWRKAIFLGRLVHAHKEPETYRDEWDDDDLVLQAYEDFMQYLNAEGRAALAACASTPRV

>AUR62039939|Cquinoa_v1.0/1-476

MLKLNTVSFHQGSTIPMTPIQLTPKKVAVVGGGAAGLVAARELRREGHKVVVFERGPQLGGTWVYSPEVESDPLGLDPTRKIVHSSLYSSLRTNLPREVMGFRDFPFVASGKADRDSRRFPGHQEVLRYLEDFANEFRLHELVRYQTEVCHVGLETEGIWKVKSRMQVGNDGSEFDETYDAVVVCNGHYTEPRVAEIPGIEHWPGKQIHSHNYRLPEPYRDQVIVLIGSSASAVDICRDLAKFAKEVHVVSRSKPEGSFGKQPGYDNLWLHPMIERAHEDGRVTFQDGSVVLAQVILHCTGYKYHFPFLHNIGAVNVDDNRVGPLYKHVFPPALAPGISFVGLPWKVVPFILCELQSRWIAGALSGRISLPSEEKMMEDIESYYSKLDAAGVPKHYTHNIGDHQFDYDDWLATETASPLIEEWRKGMYRAASLRRRAKPETYRDEWDDEDWKSQAYEDFKQYFPVESSTVIASSAL

>AUR62007483|Cquinoa_v1.0/1-476

MIKLNSVSFHQGSTIPMTPIQLTPKKVAVVGGGAAGLVAARELRREGHKVVVFERGPQLGGTWVYSPEVESDPLGLDPTRKIVHSSLYSSLRTNLPREVMGFRDFPFVASGKADRDSRRFPGHQEVLRYLEDFANEFRLHELVRYQTEVCHVGLETEGNWKVKSRMQVGNDGSEFDETYDAVVVCNGHYTEPRIAEIPGIEQWPEKQIHSHNYRLPESYRDQVIVLIGSSASAVDICRDLAKFAKEVHVVSRSKPEGSFGKQPGYDNLWLHPMIERAHEDGRVTFQDGSAVLAQVILHCTGYKYHFPFLHNIGAVNVDDNRVGPLYKHVFPPALAPGISFVGLPWKVIPFILCELQSRWIAGALSGRISLPSEEKMMEDIESYYSKLEAAGVSKHYTHNIGDHQFDYDDWLATETASPLIEEWRKGMYRAASLRKRAKPETYRDEWDDEDWKLQAYEDFKQYFPVESSTVIASSAL

>Sapof.08G127800|Sofficinalis_v1.1/1-457

MTISSLQVAVVGGGAAGLVAARELRREGHKVVVFERGSRSGGIWVYTPETESDSLGLDPTRKIVHSSVYSSLRTNLPREVMGFFDFPFVSSVKPGRDSRRFPSHQEVMWYLEDFVHEFQLNELIRYQTEVWNVELETGGKWNVKSKTNDDNYVKEFDETYDAIVVCNGHYTEPRIAVIPGIERWPGRQIHSHNYRTPEPYQDQVVVLIGSAASAVDICRDIAPFAREVHVASRSEPAGEIRKQPGYDNMWLHSMIERVHEDGGVSFPDGSTVLSDVILHCTGYKYHFPFLHTNGVLNVDDNRVGPLYKHVFPPALAPGLSFVGLPWKVIPFILCELQSKWIAGILSGRISLPSKETMMEDIEAFYSKMDAMGLPKRYTHNIGSSQFEYDDWLAIECGFPPTEEWRKAMYSAVSRQRRTHPETYRDEWDDDDLILEAHHHFKQYLPTETRAVLTSSAL

>Brara.F00871|BrapaFPsc_v1.3/1-464

MAHSNRDPTTSRHVAVVGAGAAGLVTARELRREGHSVVVLERGSQIGGVWTCTSQVEPDPLSLDPTRPVVHSSVYKSLRTNIPRECMGFTDFPFAARPHDGSRDPRRHPGHSEVLAYLRDFAKEFDVEEMIRFETEVVRAEPAADEENRGKWRVESRSSDGAADEIYDAVVVCNGHYTEPRHAHIAGIDSWPGKQIHSHNYRVPDPFKDQIVIVIGSSASGVDISRDIAKVAKEVHVSSRSTSPDTYEMLPGYDNLWLHPVIETARKDGSVVFQNGKTVYADTIMHCTGYKYYFPFLDTKDEVTVDDNRVGPLYKHVFPPALAPGLSFIGLPWQITPFPMFELQSKWVAAVLSGRVSLPSQDEMMEDTKAFYDKLEASGIPKRYTHLMPDDSQFEYDNWLADQCDYPRIEKWREEMFYIGFKRIYAQSATYRDNWDDDHLVVEAYDDFVKFMSSYPELLPLLKT

>Brara.I05131|BrapaFPsc_v1.3/1-464

MAPGSNHPTGSHHVAVIGAGAAGLVAARELRREGHSVVVLERGSQIGGVWAYTSQVEPDPLSLDPARPVVHSSLYKSLRTNIPRECMGFTDFPFSTRPHDGSRDPRRHPGHSEVLAYLRDFAKEFDIEEMIRFETEVVRAEPVADEENSGRWRVESRSSDAVADEIYDAVVVCNGHYTEPRHALVSGIDSWPGKQIHSHNYRVPDPFKDQVVIVIGSSASGVDICRDIAKVAKEVHVSSRSTSLETYEKLPGYDNLWLHPTIETAREDGSVVFKNGKTVYADTIMHCTGYKYYFPFLDTKGEVTVDDNRVGPLYKHVFPPALAPGLSFIGLPWQITPFPMFELQSKWVAAVLSGRVSLPSQDEMMEDTKAFYDKLEASGIPKRYTHLMPDDSQFEYDNWLADQCDYPRIEKWREEMFYIGFKRIYAQSATYRDNWDDDHLIVEAYDDFVKFMSGYAELLPMLKT

>AT1G12200|Athaliana_Araport11/1-465

MATSHPDPTTSRHVAVIGAGAAGLVAARELRREGHSVVVLERGSQIGGVWAYTSQVEPDPLSLDPTRPVVHSSLYRSLRTNIPRECMGFTDFPFATRPHDGSRDPRRHPAHTEVLAYLRDFAKEFDIEEMVRFETEVVKAEQVAAEGEERGKWRVESRSSDGVVDEIYDAVVVCNGHYTEPRHALITGIDSWPGKQIHSHNYRVPDQFKDQVVIVIGSSASGVDICRDIAQVAKEVHVSSRSTSPDTYEKLTGYENLWLHSTIQIAREDGSVVFENGKTIYADTIMHCTGYKYYFPFLDTKGEVTVDDNRVGPLYKHVFPPALAPSLSFIGLPWQITPFPMFELQSKWVAAVLSGRVSLPSQDEMMEDTKAFYDKLEASGIPKRYTHLMPDDSQFEYDNWLADQCEYPRIEKWREQMFYIGFKRIYAQSSTYRDNWDDDHLIVEAYDDFVKFMSSYQELLPMLKT

>AT1G62580|Athaliana_Araport11/1-464

MVPAVNPPTTSNHVAVIGAGAAGLVAARELRREGHSVVVFERGNHIGGVWAYTPNVEPDPLSIDPTRPVIHSSLYSSLRTIIPQECMGFTDFPFSTRLENGSRDPRRHPGHSEVLAYLRDFVREFKIEEMIRFETEVVRVEQAGENPKKWRVKSRNFGDISDEIYDAVVVCNGHYTEPRHALIPGIDTWPGKQIHSHNYRVPEQVKDQVVVVIGSSVSGVDISRDIANVTKEVHISSRSTKPETYEKLPGYDNLWLHSNIETVREDGSVVFKNGKTVYADTIMHCTGYKYYFPFLDTKGEVTVEDNRVGPLYKHVFPPALSPGLSFIGLPWQVIPFPMFELQSKWVAAVLAGRVSLPSQEEMEDTKMFYLKLEASCIPKRYTHLMAELDSQFVYNNWLADQCDYPRIEKWREQMFYKVFKRIQSQASTYKDDWDDDHLIAEAYEDFVKFPSNYPSSLIEREYTS

>Brara.I01446|BrapaFPsc_v1.3/1-457

MAPAQNLVSSKHVAVIGAGASGLIAARELHREGHTVVVFEREKQVGGLWIYSPKSESDPLGLDPTRPIVHSSVYESLRTNLPRECMGFRDFPFVPCVDDFSRDSRRYPSHREVLAYLQDFAREFKIEEMVRFETEVVRVEPVDGKWRVRSKNSEDEIFDAVVVCSGHYTEPYVAHIPGIKSWPGKQIHSHNYRVPGPFKNEVVVVIGNFASGADISRDVAKVAKEVHVASRGSEASTYEKLSVPTNNLWIHSEIETACDDGSIVFKNGKAVHADTVVYCTGYKYKFPFLETNGYMSIDDNRVEPLYKHVFPPALAPGLSFVGLPGMGIQFVMFEIQSKWVAAVLSGRVTLPAPEKMMEDLIASYAMLEALGIPKRYTHKLGKIQSNYLDWVAEECGCQPVEPWRTQQVDRGYGRLVSNPENYRDEWDDDDLIKEAYEDFASKNLISFLPSYFPKSGR

>AT1G65860|Athaliana_Araport11/1-459

MAPTQNTICSKHVAVIGAGAAGLVTARELRREGHTVVVFDREKQVGGLWNYSSKADSDPLSLDTTRTIVHTSIYESLRTNLPRECMGFTDFPFVPRIHDISRDSRRYPSHREVLAYLQDFAREFKIEEMVRFETEVVCVEPVNGKWSVRSKNSVGFAAHEIFDAVVVCSGHFTEPNVAHIPGIKSWPGKQIHSHNYRVPGPFNNEVVVVIGNYASGADISRDIAKVAKEVHIASRASESDTYQKLPVPQNNLWVHSEIDFAHQDGSILFKNGKVVYADTIVHCTGYKYYFPFLETNGYININENRVEPLYKHVFLPALAPSLSFIGLPGMAIQFVMFEIQSKWVAAVLSGRVILPSQDKMMEDIIEWYATLDVLGIPKRHTHKLGKISCEYLNWIAEECHCSPVENWRIQEVERGFQRMVSHPEIYRDEWDDDDLMEEAYKDFARKKLISSHPSYFLES

>AT1G62540|Athaliana_Araport11/1-457

MAPAQNPISSQHVVVIGAGAAGLVAARELSREGHTVVVLEREKEVGGLWIYSPKAESDPLSLDPTRSIVHSSVYESLRTNLPRECMGFTDFPFVPRFDDESRDSRRYPSHMEVLAYLQDFAREFNLEEMVRFEIEVVRVEPVNGKWRVWSKTSGGVSHDEIFDAVVVCSGHYTEPNVAHIPGIKSWPGKQIHSHNYRVPGPFENEVVVVIGNFASGADISRDIAKVAKEVHIASRASEFDTYEKLPVPRNNLWIHSEIDTAYEDGSIVFKNGKVVYADSIVYCTGYKYRFTFLETNGYMNIDENRVEHLYKHVFPPALSPGLSFVGLPSMGIQFVMFEIQSKWVAAVLSRRVTLPTEDKMMEDISAWYASLDAVGIPKRYTHKLGKIQSEYLNWVAEECGCPLVEHWRNQQIVRGYQRLVSHPETYRDEWDDNDLMEEAYEDFARKKLISFHPSHIL

>AT1G62560|Athaliana_Araport11/1-462

MAPAQNQITSKHVAVIGAGPAGLITSRELRREGHSVVVFEREKQVGGLWVYTPKSDSDPLSLDPTRSKVHSSIYESLRTNVPRESMGVRDFPFLPRFDDESRDARRYPNHREVLAYIQDFAREFKIEEMIRFETEVVRVEPVDNGNWRVQSKNSGGFLEDEIYDAVVVCNGHYTEPNIAHIPGIKSWPGKQIHSHNYRVPDPFENEVVVVIGNFASGADISRDIAKVAKEVHIASRAREPHTYEKISVPQNNLWMHSEIDTTHEDGSIVFKNGKVIFADSIVYCTGYKYNFPFLETNGYLRIDEKRVEPLYKHVFPPALAPGLAFVGLPAMGIVFVMFEIQSKWVAAVLSGRVTLPSTDKMMEDINAWYASLDALGIPKRHTHTIGRIQSEYLNWVAKESGCELVERWRGQEVDGGYLRLVAHPETYRDEWDDDELIEEAYNDFSRKKLISVDPSYYLENGR

>AT1G62570|Athaliana_Araport11/1-461

MAPAPSPINSQHVAVIGAGAAGLVAARELRREGHTVVVLDREKQVGGLWVYTPETESDELGLDPTRPIVHSSVYKSLRTNLPRECMGYKDFPFVPRGDDPSRDSRRYPSHREVLAYLQDFATEFNIEEMIRFETEVLRVEPVNGKWRVQSKTGGGFSNDEIYDAVVMCCGHFAEPNIAQIPGIESWPGRQTHSHSYRVPDPFKDEVVVVIGNFASGADISRDISKVAKEVHIASRASKSNTFEKRPVPNNNLWMHSEIDTAHEDGTIVFKNGKVVHADTIVHCTGYKYYFPFLETNNYMRVDDNRVEPLYKHIFPPALAPGLSFIGLPAMGLQFYMFEVQSKWVAAVLSGRVTLPSVDEMMDDLKLSYETQEALGIPKRYTHKLGKSQCEYLDWIADLCGFPHVEHWRDQEVTRGYQRLGNQPETFRDEWDDDDLMEEAYEDFARLNLINFHPSRFLESGR

>Brara.I05135|BrapaFPsc_v1.3/1-476

MIAPSDDWSPTISKPTKMAPSCSPINSLHVAVIGAGAAGLVAARELRRESHSVVVFERNTEVGGLWVYTPQSEPDPLSLDPNRTVVHSSVYDSLRTNLPRECMGYSDFPFVPRPEHDESRDPRRYPTHREVLAYLRDFAREFKLVEMVRFGTEVVRVEQDGRKWKIRSRNSDGVSRDEIFDSVVVCNGHYTEPRVAQIPGIDLWPGKQLHSHNYRVPDPFKDQVVVVIGNFASGSDISRDLTGVAKEVHIAARSKPSETYEKLPGADNLWLHPMIETARKDGSIVFKNGKVVQADTIVHCTGYIYHFPFLNTNGYITVEDNCVGPLYKHVFPPALAPGLSFIGLPWMTLLFTLFELQSKWVAAVLSGRVTLPSEDKMMEDTNALYAKRDANGFPKRYTHRLGVVGQAEYLNWIADQIGEPPVEQWRNKELEDGYVRLATQADTFRDKWDDDHLIAEAYEDFLRQKLISAIPSQIFG

>AT1G12140|Athaliana_Araport11/1-459

MAPARTRVNSLNVAVIGAGAAGLVAARELRRENHTVVVFERDSKVGGLWVYTPNSEPDPLSLDPNRTIVHSSVYDSLRTNLPRECMGYRDFPFVPRPEDDESRDSRRYPSHREVLAYLEDFAREFKLVEMVRFKTEVVLVEPEDKKWRVQSKNSDGISKDEIFDAVVVCNGHYTEPRVAHVPGIDSWPGKQIHSHNYRVPDQFKDQVVVVIGNFASGADISRDITGVAKEVHIASRSNPSKTYSKLPGSNNLWLHSMIESVHEDGTIVFQNGKVVQADTIVHCTGYKYHFPFLNTNGYITVEDNCVGPLYEHVFPPALAPGLSFIGLPWMTLQFFMFELQSKWVAAALSGRVTLPSEEKMMEDVTAYYAKREAFGQPKRYTHRLGGGQVDYLNWIAEQIGAPPGEQWRYQEINGGYYRLATQSDTFRDKWDDDHLIVEAYEDFLRQKLISSLPSQLLES

>AT1G12130|Athaliana_Araport11/1-470

MTPPPNSISSRNVAVIGAGAAGLVAARELRREGHTVTIFERQKQVGGLWVCTPNVEPDLLSIDPDRTVVHSSVYQSLRTNLPRECMGYSDFPFVTRPDDESRDPRRYPDHREVMRYLQDFAKEFKIEEMIRFETEVFRVEPTAENSCKWRVQFRSSSGVSGEDIFDAVVICNGHFTEPRLAHIPGIESWPGKQIHSHNYRVSDPFKGQVVIVIGYQSSGSDISRDIAILAKEVHIAAKSDAYAKESSIYSNLHFHPTIDRVYEDGSVVFQDGKLIFADAIVHCTGYKYCFPFLETKGYVNVEDNRVGPLYKHVFPPALAPGLSFIGLPSMALQFFMFEIQSRWVASVLSGRVKLPSEEQMMEDVIAFYAKLKSLGIPKRFTHFLTDPQWTPMFEKLKPHEAVLISQSEYFNWIAEQCGCSSIERWREEQYNIAIKKDDDNFRDEWDDDDQIIQEAYRDFAKFKPSKVWST

>AT1G12160|Athaliana_Araport11/1-468

MTSVITSRARHVAVIGLGAAGLVAVRELRREGHTVIGFEREKHVGGLWVYTDRVDSDSVSVDPDRTIVHSSIYQSLRTNLPRECMGYSDFPFVTRSSDGDPRRYPDHREVLMYLQDFAKEFKIEDMIRFETEVLCVEPSPENNRKWRVQFKSSNGVSGEEIFDAVVVCNGHFTEPRLAHIPGIESWPGKQIHSHNYRIPDPFKDEVVIVIGSQASGNDISTDIATIAKEVHISSKMVASDSYGCYDNLRIHPTIYRAREDGSVVFRNGKVVFADAIVHCTGYKYHFPFLKTSGYVTVEDNRVGPLYKHVFPPALAPGISFIGLPFMGLQFFMFEIQSKWVASVLSGRVKLPAEDKMMEEAVAFYSKLEDLGIPKRYTHFLTDPRGNPMLGTFKPEDAVVISQSDYFNWIAKQCGCTSIERWRERLYNVAIKKVFFGGDSYRDRWDDDQLIEEVYREFAKLKPNQDCSS

>evm_27.TU.AmTr_v1.0_scaffold00007.125|Atrichopoda_v1.0/1-487

MASSSVCNPSLPIHHHHNPSAANTTASVFAGTGGGSMVTAKKKVAVIGAGAAGLVAARELEREGHRVVVFERGEKAGGTWVYDPSTETDPLGIDPCRKVVHSSLYRSLRTNLPREIMGFLDFPFSIKEGRDLRRFPGYQEVELYLDDFAAESDVLKAIRFRTEVFHVGMMEGGKWVVKSRRDFGEDVAVDVNEIYDGVVVCSGHYAEPRIAEVPGVERWPGEQIHSHNYRIPEPFQDKVVIVIGSSESAADISREISKVAKEVHVSSRSALDRFGKKFYGHENMWLHSMIQGTHEDGTVVFQDGTSVIANVILHCTGYKYHFPFLETNGAVTVDDNRVGPLYKHVFPPSLGPWLSFVGLPWKTIPFPMMELQCKWVAGALSERIALPSEEEMMKDVEAFYSRIAAMGYPKHYTHNISGCQFEYFDWLATECGYPKGEEWRKEMYFANTRNKTENPDTYREEWEDHDLISQAHEEFSRLKQQHSVKNM

>GWHPCBIR007652|Santalum_yasi/1-467

MAAQPPQTPLPAVPALSRHVAVIGAGPGGLVAAAELRRFGHRVVVFERENQVGGTWVYNPLTDSDPLGVDPALKSVHSSLYASLRTNLPRESMGFRVFPFVVPVDSAHGDPRRFPGHGEVLQYLRDFAGRFEVCELIRFETEVFFVGLVEEGKWKVGFRGRGGGSDEFEVFDAVVVCCGHHSEPRIAEIPGIETWPGKQLHSHNYRVPELFQDQVVIVIGSSASAVDISSEIAGFAKEVHIAARLTGMTGFKPCHENMRFHSMIEKACEDGSVVFQSGGVVFADAILHCTGYKYHFPFLDTNGTVAVDDNRVGPLYKHVFPPTLAPWLSFVGLPWKITPFPMLELQSKWIAGILSGQITLPSQEDMMRDVESFYSKLEASGIPKRYTHNIIDFQQFEYLDWLAAECGCAAPEEWRKQTYLAVSKNWQLRPGMETHYRDEWEDEHLIMEAYEDFAKFSSNGNCNKYGS

>XP_020583148.1|Phalaenopsis_equestris/1-449

MPSTRRVAVIGAGAAGLSTSRELRREGHTVAVFERSANVGGAWIYSAAVESDPLSLDPDREIVHSSLYDSLRTNIPRESMGFLDYPFISVGGEEDCRRFPGHREVLRYLKAFARDSGLCGLVRFGSEVMRVEMEGEGRWEVVSRTVAGAVGDEREVFDAVVVCVGHYSEPRVAEIPGIDSWPGKQMHSHNYRVPEPFLGLVVVIIGSSASAADISKDIAGFAKEVHIASRSASGGTPIMDPAHHNIIIHSMIVKAHGDGTVLFQDGSSANVDVIMHCTGYKYHFPFLKTSGIVNVDDNRVGPLYKHVFPPLLAPYLSFVGLTTKIIPFPLFQLQGKWIAGVLSGRIVLPTQEEMMEDVKKLYFEMQIAGRPKRYTHNIENYHFEYVNWLAGECGHPQVEDWRKLLYHANRKNRAAKPDRYRDEWDYDDLVMEAYEHLRNIFISSSRLQI

>XP_020592568.1|Phalaenopsis_equestris/1-486

MATEDISGHLGLRRKNYSDASGSPMPTLRALLPTTTALFPDSQLMFRRRIAVIGAGAAGLVAARELRREGHSVVVFERGHGVGGTWVYTPAIESDPLGLDPNRKIVHSSLYDSLRTNLPRECMGFSDYPFVAITENSDNDRRRFPGHREVLRYLEDFSRDTDLNGMIRFGTEVISVEMEGEGRWAVRSLKIDDGRADDETEVYEGVVVCNGHHTEPRIAEIPGIETWPGKQIHSHNYRMPQPFTGQVVVIIGSATSSADISRELAGFAKEVHVASRTSRVKIPMKQPGYDNLWLHSMIDKTFSDGTVAFQDGCSVQVDVIMHCTGYKYRFPFLRSDDIIDVDDNRVVPLYKHVFPPFAAPYISFIGLPWKVVPFPLCELQSRWVAGVLSGRIALPAPEEMMGDVKAFYSDMEQAKRPKRYTHNIADYQFEYDDWLASQCGCPPVEEWRKQMYEETAKNRIERPESYRDEWEDDDLVAAAYHDFKNS

>Zosma02g02230|Zmarina_v3.1/1-461

MGSNSFATTSRHIAVIGAGAAGLVAARELRRQTHTVVVFERSHSIGGTWIYTPDTESDQLGGVDRNCNILHGSLYDSLRTNLPRECMGFLDYPFVGIEDHRRFPHHKEVLRYLQAFGRDFGIEKLVRFGTEVVGVEMGFHDKWIVKTRCVDDVIVGKQDPLPPHVAASEVYDGVVICNGHYTEPRIAEIQGIMAWPGKQLHSHNYRVPKPFADQVMVLIGASASSFDISKELARFAKEVHIASRSEDPIGTPKKLSGYDNMWIHPMIERANEDGTILFQGGYSFRVDTIMHCTGYKYHFPFLKTDDIVTVEDNRVGSLYKHIFPPSLAPGLSFIGLPWKVIPFPMFELQSKWVAMILSGQVTLPSVEDMANDVNEFYEELESIGYPKRYTHNLAGQQFKYNDFLATQCGYTAIEEWRKGMYEATSKNRIVRPETYRDVWDDGCLVKKAQNDFNLWRINFKA

>Cucsa.320630|Csativus_v1.0/1-439

MQHSRRVAVIGAGGAGLISARQLSREGHQVVVFERNNQIGGVWVYSPEIESDPLGVHPKRTRIHSSLYKSLRTNIPREVMGVRDFPFVPREGEDRDPRRFPSHTEVLKYLEDFANEFGICKLVRFRTEVVFAGLEKLGKWRVEFRCENGDVHYDIFDAVVVCVGNFSQPRVAEIPGIDGWPGEQVHSHNYRDPEPFRGKVVVLIGYSSSGTDISQELIGVAKEIHIACRPAKTESSDEKSIISNVSFHPMIESVHKDGTVVFQDGSVVSADVILHCTGYKYHFPFLETNGTVTVDDNRVGPLFKHVFPPALAPGLSFVGLPFKVVPFVIFELQSNWIAGVLSNRIALPSKEEMLADVKAFYEELEARGKPKHRTHKLGGYTPAYCNWLAATCGCPPYEEWRKEMFVATDINKVANLESYRDDWHDDELIHQAYEEFGKL

>Cucsa.320650|Csativus_v1.0/1-442

MQQSRRVAVIGAGAGGLVSARELSREGHHVVVFERNTQIGGTWVYSPEIESDPLGIDPNRTRMHSSLYKSLRTNLPRELMGVRDYPFVPREGEDRDPRRFPSHPEVLKYLEDFANEFGICKLVRFRTEVVFAGLEEVGKWRVEFRCENGDVDYDIFDAVVVCVGNYSEPHVAEIPGIDGWPGEQVHSHNYRDSKPFRGKVVVLIGYSFSGMDISQELNGVAKEIHIACRSAKTELLDTQSIISNASFHPLIKSLHEDGTVVFQDGCVISADVILHCTGYKYHFPFLETNGIVTVDDNRVGPLYQHVFPPALAPGLSFVGLPFKAVPLPIFELQSNWIAGVLSNRIALPSKEEMLADVKAFYEDLEAFGKPKHRTHELGDDMPVYCNWLATTCGCPAFEEWRKKMYIAIGIYKKANLETYRDDWQDNELIRQAYEEFSKYKYK

>Cucsa.320640|Csativus_v1.0/1-441

MPHSSRVAVIGAGAGGLVSARELSREGHHVVVFERNTQIGGAWVYSPEIESDPLGVDPDRTRIHSSLFKSLRTNIPRELMGVRDFPFVPREGEDRDPRRFPSHQEVRKYLEDFANEFGVYKFVRFGTEVVFAGLEELGKWRIEFRCENGDVDYEIFDAVVVCVGNYSQPRVAEIPGIDGWPGEQVHSHNYRDPEPFRGKVVVLIGYSSSGTDISQELIGVAKEIHIVWRSPKRELLDRESIISNVSFHPMIESVCKDGTVVFQDGCVVSADVILHCTGYNYHFPFLETNGNVTVDDNRVGPLYKHVFPPALAPGLSFVGLPFKVIPFPLFELQSNWVAGVLSKRIALPSKEEMLADVKAFYEDLEALGKPKHRTHLLGDYMMPAYCNWVATTCGCPPYEEWRKEMNISVHLYRLPNLKTYRDDWHDDELIRQAYEEFKHPL

>Cucsa.320660|Csativus_v1.0/1-374

MGVRDYPFVPREGEDRDPRRFPSHREVLKYLEDFANEFGICKLVRFGTEVVFAGLEEVGKWRIEFRCENGDVEEDLFDALVVCVGNYSQPRVAEIPGIDGWPGEQLHSHNYRDPEPFRGKVVVLIGYSSSGTDISQELIGVAKEIHIAWRSTKTELLNTESINSNVSFHPMIESVHKDGAVVFQDGCVVLADIIMHCTGYKYHFPFLETNGIVTVDNNRVGPLYKHVFPPALAPGLSFVGLPFKVVPFPLFELQSNWIAGVLSNRIALPSKEEMLADVKAFYENLEAFGKPKHRTHELGDDMPAYLDWLAAVCGCSAYEEWRKEMYIAAHMNKVANLRSYRDDWHDNELIRQAYEEFSKYATNEGSGNHSKLSV

>Bradi1g78290|Bdistachyon_v3.2/1-437

MGSLSVAVIGAGAAGLAAARELRREGHGPVVVFERCAGVGGTWLYDDKVSTNHHSSLYASLRTNLPRETMGFLDFPLVSSDSGDSRRFPGHAEVLRYLQEFARRFDLHGMVKLQTEVLSVRREASSSWKVTYSRKQEEKEVIVEEEVFDAVVVCNGHFTHPRLADIAGIDGWPGKQMHSHSYRVADPFHGQVVVVIGYKPSGMDISRDIAGVAKEVHVAIRSPAAAAANISQTQQMPLPANQAHPNLWLHSCTVERAEGDGSVVFQDGSRVKADAIVHCTGYKYSFPFLDEDDAGINVDDNRVGPLYKHVFPPRLAPHISFIGLPFRAMLFPVFQLQSNWVAGVLSGRIELPSQEEMMKDVAAFYSDLEARGCPKRYTHDLGTSFEYEDWLAERSGLDKIEEWRKAVYLAARKNVTDRPEAYRDDDRSLRYDATGCV

>HORVU.MOREX.r3.4HG0415610|Hvulgare_Morex_V3/1-447

MPSSRVAVIGAGAAGLVAARELRREGHTPVVFERAAGVGGSWRYQDAASADPLGAGGVHGSLYASLRTNLPRECMGFLDFPFVADEGSVDPRRFPGHQEVLRYLEEFARRFHLLGLIRLETDVVSVRRDTGGRGRWRVSSSSPTKLAGEPEEARSELFDAVVVCNGHFAEPRLAEHIAGMDGWPGKQTHSHSYRLPDPFHGQVVVIIGYNPSGMDISRDIAGVAKEVHVAIRAAPAEMQSATAHANLWLHPMVDRAEEDGGVVFQDGSRVKADAIVHCTGYKYNFPFLDGDQDGDPVIAVDDNRVGPLYKHVFPPHLAPHISFIGLPFKAILFPVFQLQSSWVAGVLSGRIELPSPEEMTQEVAAFYSDMEARGCPKRFTHDLGASTFEYEDWLAERSGLERMEEWRRAIYVAARGRLRGRPESYRDEWDDDAHLLDQAHRDFAKYL

>Bradi3g05860|Bdistachyon_v3.2/1-456

MRPRRSVRVAVIGAGAAGLVAARELRREGHSPVVFERADAVGGVWVYDDAVAGGSEQRPSSSCLYASLRTNLPRESMGFLDFPFHAAGDGDARRFPGHEEVRRYLEDFARRFDLLGLVRLQTEVVRVTREAAGASESWRVSYHTSERREAEEEEVFDAMAVCNGHYGEPRFADIPGMDAWPGKQVHSNNYRVPEPFRQQVVVVIGCGPSGTDIARDMAGVAKEVHLANRSAPASTCERLPLPGNANLWLHSMVDRAEEDGTVVFQDGSRVKADVIMHCTGYKYSFPFLGEDSTISVDDNRVGPLYKHVFPPQLAPRLSFIGLPVKVIPFPLFELQSSWVAGVLSGRIELPSEEEMTRDVTAFYSALSSRGCPRRYTHNMRNHEFEHEDWLASQCRRERIAEWRRGMFAAATGKNALGRMGSYRDEWDAGDGDQLDLLLARANREFAQYFLPAAPPP

>Bradi3g05867|Bdistachyon_v3.2/1-496

MSGRKTDTRGGEQNRGDPSISLPFPPRRSLILSESSMPRRSVRVAVIGAGAAGLVAARELRREGHSPVVFERADAVGGVWVYDDAGGGSEQRPSSSCLYASLRTNLPRESMGFLDFPFHAAGDGDARRFPGHEEVRRYLEDFARRFDLLGLVRLQTEVVRVTREAAGASESWRVSYHTRTKLERREAEEEVFDAVVVCSGHYREPRFADDIAGIDAWPGKQLHSNSYRVPEPFQNQVVVVIGCGPSGTDIARDIAGVAKEVHLTNRSAPAATCDRLPLPGHDNLWLHFHSMVDRAEEDGTVVFQDGSRVKADVIMHCTGYKYSFTFLSEDSTISVDDNRVGPLYKHVFPPQLAPRLSFIGLPHKVAPFPLFELQSNWVAGVLSGRIELPSEEEMTQDVTAFYSELSARGWPRRYTHSMQSHEFEYEDWLAGQCRRERVTEWRKELFAAALRINTLGRMGSYRDEWDAGDGGGKLDQLLAQANREFARYSCPATPPP

>HORVU.MOREX.r3.6HG0569600|Hvulgare_Morex_V3/1-454

MPSRSLRVAVIGAGAAGLVAARELRREGHAPVVFERTDGVGGTWVYEADADASASPEPPGGPRRRSNLYASLRTNLPRESMGFLDFPFVAGADCDIRRFPGHAEVLRYLQSFARRFDLHGLVRLETEVVRVSREASGASWRVRYTRKASGGEKREAEEEVFDAVVVCNGHYSQPHFADVAGVDAWPGKQLHSTSYRVPEPFHGQVVVVIGCGPSGTDISRDIAGVAKEVHLASRSAFAATSDKLPGHANMWLHSEIECVQEDGSVVFHDGSRVKADVIMHCTGYKYNIPFLNSDATVSVDGNCVEPLYKHVFPPKAAPQLSFIGLPLKVIPFPLFELQSHWVAGILSGRFQLPSEEEMMRDVTAFYSRLGARGWPRRYTHRLRDREFENEDWLAEQCRRDGPEEWRREMFAAAIEVMGERPETYRDEWDGGDYDHLLAQANRDFAAHLPAPLWP

>Sapof.06G231800|Sofficinalis_v1.1/1-442

MRSKKVAVVGGGFSGLVAIRELCREGHEVVAFERGAQLGGTWVYSPETESDPLGIDPTRTVVHSSLYDSLRTNLPRHIMGFREFPFVDSGKPGRDSRRYPKHEEVLRYLEDFACEYGLKELIRFQTEVLFVGLEKDGKWTVKSRRKNTDELEETFDAVVICCGHNTEPRIADIPDVEVWPGWQCHSHSYRTPEAYRDQVVIVIGASASAIDISRDIAGVAKEIHVVRQSEGRFKFWDKFRLTNMFFHPMIDKAYADGRVGFQDGTEVIANVILHCTGYKHHYPFLHTDGAVNVDDNRVGPLYKHVFPPALAPGIAFVGIPNFVITSLHFELQSKWIAGILSERISLPSEEEMMQDVETLYSKLEASNVPKRHTHMLGDFLFELEDWLAKECGCPAIEEWRKNMFKSCFASFIAFPKTYRDEPDCSEELYEQAEQEFRQVLGT

>Dicom.23G067300|Dcomplanatum_v3.1/1-554

MVEAVSSSTRNRGSSRSSSANTGHNDYGRKKCEALAGNSNLNGEDELQMAIGSDKNATKQSCKVAVIGAGAAGLVAARELQREGHEVVVYEEGEKVGGVWLYNSDVEDDPLGLDPNRCSVHSSMYASLRTNLPRELMGYLDYPFLPREGRDVRRFPRHEEVASYLQDFAEEFNLLLLVKFCTKVEYVGIYRGDGNTEADIVNDSSFKQQLLREGCEDLREDSANLETRWVVRTRERRQAVEKERDVLNFATGCEDQVTDVIFDAVVICNGHYSQPRVAWIPGLEKWPGKQMHSHNYRIAEPFRDKVVVVIGNGPSGIDISLELISVAKEVHFCAKKWDLSKDLFWPLHEGKLQCHLLVVELVTEDGTVHFQDGISCHVDVILHCTGYLYSFPFLDTHGIVTVENSAVGPLFEHVFPPLLAPSLSFVGLPFKVVPFPLFELQSKWIACILSKSSSLPSTKDMMADVQSFEVRLAEEGIPKHYTHNLDSCQFEYDNWLAAQCGSEPIESWRIKMYKVAQKIRKLYPDSYRDIWDDYDLLDEAQSYLLKLKTEKHWK

>Acora.08G044000|Aamericanus_v1.1/1-431

MSRKVAVVGAGVAGLTAARSLLSEGHQVTVFEKSRHVGGTWVYDERVESDPTGLDPARSSVHGSIYHSLRTNLPRPLMGFSDYPLPAGPPSAAFPSHEEVRSFIEGFARDFGLSERVRFGSEVVSVGPGLRADEWVVEALEAEAVVVETFEAVVVCNGHHSVSRVAHVPGIDKWPGKQIHSHNYRVPEPFRDQIVVLIGGATSAHDISREISVVAKEVHLSSRSVDVEFSKLEGQSNIWQHSSISCVYEDGMVAFHDGSSIHADVIFYCTGYKYSFPFLKTNGIISIDENRVGPLYQHVFPPKLAPSISFIGLPNKVIPFLMIDLQSKWIAQVLSSKVMLPPEEEMMASIKALYMQMEATGKPKHHTHMLHPYEIDYMNWLAAQVGVPPLDKWKEQMYLAVFECYSSQSDGYRDNWDDEYWNRLRLSETGL

>Acora.08G043900|Aamericanus_v1.1/1-435

MVKRRSCKVAVIGAGVAGLAAARSLLNEGHHVTVFEKSRHVGGTWVYDERVESDPIGLDPARSRVHSSLYQSLHANLPRPLMGFSDYPFPSGPPSSAFPSHEEVRSFIEGFARDFGLSERVRFGSEVVSVGLGPRADEWVVEALEAKAVVVETFEAVVVCNGHNSVSRVADVPGINKWPGKQIHSHNYRVPEPFRDQTVVLIGSSASAHDISREISVVAKEVHLSSRSTDVEFSKLKGQSNIWQHSSISHVYEDGMVAFHDGSSIHADVIFYCTGYKYSFPFLKTNGIISVEDNRVGPLYQHVFPPKLSPSISFVGLPERAISFHMMDLQSKWIAQVLSSKVMLPPEEEMMASVKALYAQMEATGQPMHYTHMLHSYEIDYMNWLAAQVGVPPLGKWKEEMYNAIFESYRSQNDAFRDNWDDEYWNRLMLSEAGL

>Sphfalx04G133500|Sfallax_v1.1/1-459

MANCQIVDQVYKCCKVGVIGAGAAGLATAKELIHEGHKVTVFEQAMQVGGLWVYDPEVDSDLLGVEPHRKRVHSSMYASLRTNLPREVMSFIDFPFLTQGRDERCYPSHKEVALYLENFAKKFDLLHVIQFGTLVEHVELVTMDSTGKKRWKVITTRKIDGVVDEVKEELFDAVVVCSGHYFEPKIANIPGINNWPGKQMHSHNYRLSQPFTNQCVVIIGTQPSGMDISRDVANVAKEVHLSGRSWNSSIDFAKPMGQHQNIWPHSTVIRTYEDGTVKFKEGSSTMADVIMHCTGYRYYYPFLDTKGLVTAADIVKPVYQHVFPPSLAPSLSFVGLPYKVLPFPLFQFQAKWIAMVLSGKVKLPPTNEMTKSIEAFYDQCEASGKPMHQWHDLVDIEFKYLDWLADQSGSEHLESWRSKMKNIAFQHLFNNPDTYQDQWPDEDLHQEALLCLQQLEITR

>Sphfalx04G135100|Sfallax_v1.1/1-558

MATLSAKCMSKFHFNLLPCMWVGQTRSSLGYCLGEARIMVTKKIASELVDERQRKVKCNSSCTLEENQMGNCGADDRVAGAAAAAAAAAGDGNDDDDGVYKRCCKVGVIGAGAGGLVTARELIREGHSVVVFEKSTTVGGVWVYEPEVDSELLGVKSDRKRVHSSMYASLRTNLPRELMSYVDYPFLARNGRRRDGRRYPGHQEVAFYLQDFAQECELLDFIRFGVSVEHVELEMDRPDGEISWKVTTRRRVVDGFEEVVEEEVFDAVVVCSGHFSEPRIAEIPGIDTWPGKQMHSHNYRDPQSFMDKSVVVIGTGASSDDISRELAEVAKEVHVSGRSWKSSVEFAKPVGRHQNIWLHSMVKCAHEDGTVEFEEGGSTVADVILHCTGYYYYYPFLDTKGLVTADNKVGPLYEHVFPPTLAPSLSFVGLPQKVIPFPLSQLQARWIAKVLSGKVQLPSQSEMMKSVQAFYAQFEASGSPMPHLHYLQGDSQFQYDDWLADQTGSEHLETWRLKMYATAGQNRSDNPETYRDIWPDDDLREEALSSLRKLDTNRTLAD

>Tle1c01776.1|Takakia_lepidozioides/1-622

MEHRSHWGCVAEVRLREGIGLVNKFRAPSSDKNFNGSKLSLRIASLVHLLRGSQRAGELARDEKSRCSGAQLSTDNCDSQLPSTNFFGQLRPSGIFLCSASGCDFGGGRVRVMGLHSNDSVPAHASPPSNSFKSESGQINETGEEACPDLSTLHNLSTSEANKASNVSGASCIQKSCKVAVIGAGAAGLVAARELIREGHRVTVYEQATSVGGVWVYDPNVESDLLGVSPSRSRVHSSMYASLRTNLPREIMGFTDFPFLPRDGRDRRRFPGHKEVALYLQDFTEEYGIAQFIEFGVVVEYAGMHKEGGIIKFWKVRTRRLDESEGVKEEMFDAVVVCNGHYSEPKIAHIAGIDMWPGKQMHSHNYRCPQPFTDKTVVVIGNSASGADISKEIGEVAKEVHVSGRTWGSSVDFLKPVGRHQNIWQHSTVECALEDGTVKFTDGASTVADVILHCTGYFYSFPFLDTEGIVTEDDNCVGPLYKHVFPPILAPSLSFVGLPFKVVPFQLCELQSRWIARVLSGKVQLLSSSEMMKEVGGFYVQLQASGKPKCFTHNLATFQQYEYDNWLADQSGSDHVETWRKEIYVTTSKNKRENPTTYRDVWPDEEFHKAAMLCLLNQQMMR

>Pp6c11_7940|Ppatens_v6.1/1-588

MWKPSTGAAMLTLCPGILCSLNGIHVTSSSSGLRVRGRAHCHVASASVSTEIAKNSAGLRGRTIGCVRGWSSAGRSQSFSSATCFGAMKSRSGFTSSGNGVDIRWIKTNGDDTSNQTAGVVYPSKSCKSKSCKVAVIGAGAAGLVTARELLREGHDVIVFEQTKSVGGVWVYDPEIEGDDLLGLSQDRKRVHSSMYASLRTNLPREIMGYTDFPFLPRDGRDGRRFPGHAEVAAYLQDFAEFYHLLDCVQFSTSVDYVGSCKDQNKISWKVRTRRRHQTDDVKEEQFDAVVVCNGHYSQPKLAEFPGSSSWPGVQMHSHNYREPSSFTDQTVVVIGNAASGEDISREIADVAKEVHISGRTWSASVDFSEPIGQHGNIWRHSTIECACEDGTVLFAEGGCVSADIILHCTGYFYHYPFLDTKGEVAVDENCVGPLYEHVFPPSLAPSLSFVGLPWKVVPFQLCELQSRWIAMALSRKIDLPSTQEMMDSVESFYAELEASGKPKRLAHNMATTQYDYDNWLADQTGSAPVETWRIQIFEATSKNKRANPETYRDEWPDEELHQEAFARLEELDLSCRLTVECIQQAKL

>Pso06G22590.0|Papaver_somniferum/1-418

MARVSPSAFRHDFQIGAGAAGLVASRELSREGHEVVVFERSDRIGGTWVYDPNIDSDPLGLDPSRTIIHSSLYESLRTNLPRETMGFRDYPFAAQVGLLEEEEKLRNNWVVKSRKKGEVGNSSVAHEVFDAVVVCSGHYTQPNIAEIPGIGAWPGKQLHSHSYRVPEPSPSVVRASAADISKEILTVAKEVHVASRSTMNVSPTLRFGNNNVPHHPLVKRAHEDGTVVFQDGSLVRVDVIVHCKGYKYYFPFLEINDIVTVDDNRGGPLYKHIFPPVLAPGLSFIGIPWMALPFPVFESKSKWVAGILSGRISLPSKSKMIEDVEAFYFQLKVAAIPNRYTHFLGENQFDYTDWLASESGCQPWEEWRKQMYKARQENATARPETYRDEWEDEDLVVQAHEDFRQYLPAELCVHGKQT

>evm.TU.Scaffold_521.187|Carabica_v0.5/1-326

MGRQLLKVAVIGAGVSGLLTARELQKEGQQIVVYEKSNQIGGLWVYDPEVETDPLGLDPNRRIVHSSIYDSLRTNLPTQLMEFTDYSFTIEKNGKYLCFPDYKEVLKFLNDFARDFGLDDLIQFNTEVVSVKQKNGMWVVESKTNGDDQFKKEEHFDMVVVCNGHNTQPKLVNAPGIKKWPGKQIHSHNYRVPEPYKDQVVVVIGHGPSGFDIAFDIAKVAKEVHVSSRCPQVEVRKLETYKNVWQHPKIEYCHENGEVAFQDGALIAADVIIHCTRYKYDFPFLETNGIVMVDENRVGPLYKHVFPPQLAPTLSFVGIPSQVLRF

>evm.TU.Scaffold_2157.112|Carabica_v0.5/1-459

MAAACSGGDEIQTASVTTYLDELQDCTIHMASQLHKVAVIGAGVSGLVTARELQREGQQIVVYEKSNQIGGVWVYDPEVETDRLGLDANRRIIHSSMYDSLRTNLTKQLMEFTDYSFTIEKNGKYLYFPDRKEVLKFLNDFGRDFGLDHLIRFNTEVVSVEQKNGKWVLESKTSDVDQINQKELFDAVVICNGHHTQPKLADTPGIKKWPGKQIHSHNYRVSDPYKDQVVVVIGNGPSGYGIAFDIAKVAKEVHVSSRFPQVEVRKLETYDNVWQHSKIEYCHENGAVAFEDGALVAADIIIHCTGYKYDFPFLKTNGIVTVDDNRVGPLYKHVFPPRLAPALSFVGIPKQTLNFRVAELQAKWVAQVLSGKVTLPSRQKMVADVEEHYRLMEETGVPKHHTHTFLSCNDTFIYLDWLAAQVGLPPTDEQLKRIFDKLVELATISRVGFRERLVESWPF

>evm.TU.Scaffold_521.188|Carabica_v0.5/1-430

MAPQLHKVAVIGAGVSGLLTARELQREGQQIVVYEKSNQIGGVWVYDPEVETDPLGLDPNRRIIHSSMYDSLRTNFPKQLMEFTDYSFTIEKNGKYLYFPDRKEVLKFLNDFARDFGLDHLIRFNTEVVSVEQKNGKWVLESKTSDVDQTNQKELFDAVVICNGHHTQPKLADTPGIKKWPGKQIHSHNYRVPEPYKDQVVVVIGHGPSGFDIAFDIAKVAKEVHVSSRFPQVEVRKLETYENIWQHSKIEYCHENGAVAFEDGALVAADIIIHCTGYKYDFPFLNTNGIVTVDDNRVGPLYKHVFPPRLAPTLSFVGIPKQTANFRVAELQAKWVARALSGKVTLPSREKMVADVEEHYRLMEETGVPKHHTHTFLSCNDTFIYLDWLAAQVRLPRTDEQLKRILDKLVELVRISRVGFRERLVESWPF

>evm.TU.Scaffold_263.397|Carabica_v0.5/1-428

MARSLKVAVVGAGVSGLVTARELQREGQQVVVYEKSNQIGGTWVYSPQVESDPLSLDPKREIVHSSLYYSLETNLPRRLMGFSDYPFTIRKNGELRTFPGHQEVLQFLNNFVQDFGLLDFIRFNTEVVRVEQQNDQWVVESRRRTCDELSSSEEIFEAVVVCNGHYTIPKVADLPGIKSWPGKQIHSHNYRVPEPFRDQVVIVIGAGPSAMDIGQEITKVAKEVHLCSRSPEIKVSKLEMFNNLWQHLKIEYCYENGLVAFQDGTSVAADIILHCTGYKYDFNFLRTNGTVTIDDNRVGPLYKHVFPPELAPGLSFVGLPYRAVIFFMIEVQAKWVASVLSGKVILPPKEEMLHDVEQHYRLMEENRIPKHHTHRLPLDPFEYLNWVAAQVGFPVDTELLEIYHKFFEFICGASWIKFREQDVESWNN

>evm.TU.Scaffold_634.59|Carabica_v0.5/1-433

MARPLKVAVIGAGVAGLTTARELKSEGHQVVVYEKADQIGGMWVYNPEVETDPLGLDPGRKIVHSSLYHSLKTNLPRPLMRFWDYPFTVKNKDGVLMDFPGHEEVLQFLNNFAEDFGLVEFIRLNTEVVRVEQGNDDQWVVESRSNGGLISEEPFEAVVVCNGHNTQPRVAELPGINNWKGKQIHCHNYRVPEPFRDQVVVIIGGAASANDISLEIVEVAKEVHLSSRSQEIEVKKWDMYDNLWQHSKVTNCYENGEIAFEDGALVTADTILHCTGYKYNLPFLKTNGVITIDDDNRVGPLYKHVFPPQLAPRLSFVGIPRVAINFVMIDLQAKWVASVLSGKVSLPSEEDMSADLEQCYRLLEEKGIPKHHTHSLDLNMHEYLDWVAAQVGLPPVDKRQKEIFYKIREILPTSWIGFREMLLKQLPWISRIS

>Soltu.DM.05G020690|Stuberosum_v6.1/1-433

MVESRRVAVIGAGPSGLVTARELQREGHRVVVFERSNQLGGLWDYNPQVETDLLSLDPNRKIIHSSLYKSLRTNLPRQLMSFSDYSFDCAENGNRLNFPEHKEVLKFLNEFAKDFGINELIRFNTEVVRVAPVEFGGNRWGVESKSEELSSEEVFDSVVICNGHYTVPRVANIPGINNWPGKQIHSHNYRVPEPFKDQIVVVIGDGPSGLDICLEIATVAEQVHLSTRSSEIEVPKLDNYENLWNHSKIDHVDESGEVTFLDGSSIHADIILHCTGYKYDFPFLETNGIVSVDDKGRVVGPLYKHVFPPKLSPCLSFVGIPYQGIVFLMFELQAKWIAQVLSGKVLLPSEEEMLADVEDHNRQLEEAGIPKRHTHRLHPQMEYMDWIAAQMGIPSVDAGLKEMYWSIYRCAGEVGYANYRDLWVFDNLAKLSL

>DCAR_728804|Dcarota_subsp_sativus_v3.0/1-466

MILSLKIAVIGAGVAGLIAARELKRSGHQVTIFEKSGQIGGTWAYNPQTESDPLSLDPNREIIHSSLYSSLRTNHPRHLMSFSDYPFAKFYGDPRNFPGHEEVLKFLNEFWVEFGLTELTRLGTEVVRVEAVDDSGYCEWIVESRKGELSEVEVFDGVVICNGHHTVPRVAAIRGIGQWPRKQIHSHNYRVPEPFHNQIVVMIGAGPSAYDISRDIAEVAKEVHLSSRNSTCGGFYKLVNLSNVRQHAEIDYVDESGRVAFVDGSSVCADIIFHCTGYKFSFPFLRTNGIVTVDDNRVGPLYKHVFSPEIGSRLSFVGIPCNITLFPMMELQSKWISCVLSSKVVLPSKEAMLADTNEHYRSLEERGIPKHSTHSLATTGFQYLDCLASQVKVQPVEERLKNIYKQLVSVFFSSEGDQFREWDVDSWVNGNHLKLLAASEKRSGSKLLAAFEENVGHDNSKVVESS

>DCAR_728802|Dcarota_subsp_sativus_v3.0/1-425

MPSLNVAVIGAGVAGLLAGRELQRAGHRVTIFEKQNQLGGTWVYDPRIESDPVGLDPSREIIHCSLYSSLRTNFPRHLMGFSDFPFTKIYDDSRTFPSHEEVLKFLNDFALHFGVDKLIRLSAEIVGVELRNDEWILESRTGGLIREEVFDAVVVCNGQYTEPRVANFPGIEKWPGKQIHSHNYRVPDPFRDQIVVVIGAGPSAMDISGEIAAVAKEVHISTRPSSLLSKSVIFEKFKQHSEIDYVDESGTVAFEDGSSVQADIIFHCTGYKYNFPFLKTNNIVTVEDNRVGPLYKHVFPPEIAPRLSFIGIPNAITVFPMMELQAKWISLVLSGQVLLPSKESMLADTEAYYKSMEQFGSPIRFTHFLFPTKFEYLDWLADQAGAPRIDELTRRIFAQVFTNIFSSEADKFREWDVDSWIDTVM

>DCAR_728803|Dcarota_subsp_sativus_v3.0/1-421

MPCLNVAVIGAGVGGLCAGRELQRAGHRVTIFEKQNQLGGTWVYDPRVESDPLSLDPKREIIHSSLYASLRTNFPRHVMGFSDFPFTKIYEDSRAFPSHEEVLRFLNDFAEQFGLVELTRLNTEVVRVELKEDEWVVESRMGELTREEAFDAVVVCVGHHTEPRVANFPGIEKWPGKQIHSHNYRNNKPFRDQIVVVIGAGPSAMDISIEIAKVAREVHLSTRSSSMLSKLAIFAKLNKHPEIDYVDESGRVVFEDGSSVEADILFHCTGYRYNFPFLKTNNIVTVEDNRVGPLYKHVFPPELAPRLSFIGIPYATTGIPLMELQAKWLSLVLSGQRLLPSKETMMADTEDFYKSLELRGIPIRHTHSLFEYPNWLADQLGLPGIDEQIKKIQGQVFKILLSSEGSDLREWDVDSWLDSIN

>Pn6.527|Piper_nigrum/1-424

MPMPTKVAVIGAGVSGLLTARELNREGHPVTVFEQGDRVAGLWVYDPRTETDLLGMDPKREIVHSSLYKGLRTNSPRKLMEFLDYPFVVREDGDTREYPCHEEVLAFVENFAKDFGLMELIRFAARVVRVEVVEKGKRLFVVWKNLVASTAEEEVFDAVVVCNGHYTQPLLAEIPGIEKWPGRQIHSHNYRTPEPFRDQILVVVGNGPSAVDISRELASVAKEIHLASRPPDIKLGKLANHANIWQHPMVIDAQEDGSLLFQDGTSLVVDIILYCTGYRYSFPFDLKTNGYIKVENNRIHPLYKHIFPPRFAPSLSFIGIPFKAIPNFLVKELQAKWLASVLSGKVSLPSEADMTASIENHYLKLKEEGKSEHYTHCLFPHEFDYITWIIDQLGLPALDPGIEETYRSIIKRILSKNDEEYRDS

>TnS000637613t01|Gnetum_montanum/1-511

MISYSPWLQRLTISRRGICHRKQQPVSIILSSTASRLQLRIMSCKKPSESCSVDAYSSQNFRRKKVAVIGAGAAGLVAARELDREGHDVTVFERRSCVAGLWTYDPSTEEDDPLGLDPNRKVVSSSMYQSLRTNLPREIMGFHQYAFGYFPGRDPRRFPGHREVALYLQSFSDHFKLHRFIRFETEVVHVTMERDYHNKVVGWNVKYKAADDDNVVGIQEERYDAVVLCNGHHTQPRVAPIMGLSKWPGIQIHSHNYRVPEPFKDQVVIVVGNASSGTDISLELAMVAKEVHLSARSIASTDVKNVQGSWKIIMHPMIESLFEDGRVRFQDGNTVVADAIVHCTGYCYEFSFLEIDGIVTVDDNCVGPLYQHVFPPALAPSLSFVGLPWKVLPFIILEMQSRWIAKLLSEKISLPSESDMMEAVTIFYDEFMANGGRKHQIHMLGDNQFKYDDWLCDQCGCVRVEQWRKEIYLATAENRTAQLETYRDRWKDHYWHEMALLDLSSRHGYGS

>175034|Smoellendorffii_v1.0/1-439

MEKRVAVIGAGASGLVAARELLREGHSVVIFEQARRIGGTWVYEENHSGSSNAARNYSCHSSMYESLRTNLPREVMGFLDYPFVPRRSSRDARRFPGHEEVLDYRESFAVEFGLHGYVRFNSKVKYVGLVESNWIVKSSSSAGKEEKESEEVFDAVVVCNGHYFQPRVAQIPGIERWPGKHYHSHTYRTPDPFKDQVVAVIGNGPSGEDLCSDIAAACKKVHWCAKSWNSLSEPLQQGKIQRHLMISRADEDGRLHFMDGMSAVVDVILHCTGYLYNFSFLDTKSYIKVEDNRVGPLFKHVFPPALAPSLSFVGLPCKTIAFPLSELQAKWIAAVLKGRASLPSKGEMANEVVDFYRTLEAQGVPKRYTNHLYLETFDYAEWIAKQCGGTCHGFEPWRKELCLSTLDKKKLNPGIYRDEWNDNDFHEKVVAILAKKQQQ

>413958|Smoellendorffii_v1.0/1-446

MEKRVAVIGAGASGLVAARELLREGHSVVIFEQARRIGGTWVYDPPPQESHSRSSNVARNHSCHSSIYQSLRTNLPREVMGFLDYPFVPRRSSRDARRFPGHEEVLDYLESFTVEFGLHGYVRFNSKVKYVGLVESNWIVKSSSSAGKEEKESEEVFDAVVVCNGHYFQPRVAQIPGIEKWPGKQYHSHTYRTPDLFKDQVVAVIGNGPSGQDLCSDIAADCKKVHWCAKLWNSPVESLQQGKIQRHLMISKADKDGRLHFMDGTSAVVDAILHCTGYLYDFSFLDTKNYVKVEDNRVGPLFKHVFPPALAPSLSFIGLPWKTIPFPLSELQAKWISAVLKGRASLPPKGEMANDVVGFYRTLEAQRVPNRHTHRLDLEMFDYAEWLAKQCGGTCHGFEPWRKELFLSTRDNRKLNSDSYRDEWSDNDLHEKVVGVLATSFFMREQ

>Mapoly0002s0275|Mpolymorpha_v3.1/1-549

MIIQAAFRPPFSSARRTACTSALNRSGAKSQLVDRSRRLFDEDFVPTLSSRGGRRELLRNEGVSDRKPSILVRFKGVDEMTREEDGRSGAAEIKRVLVIGAGAAGLAAAVELAREGHDVVVYEKSSEIGGVWNYTPDADSDPLSIDPQRKRVHSSMYASLRTNLPRELMGFLDFPFLAKEGRDKRRYPGYEEVNSYLRDFAEHHDLLSLIEFGVEVENVRLKKAGNGNSASESRDLKWIVRSNRFGKFNHEPQTKEDVFDAVVICNGHYAEPKLINIPGVESWPGFQMHSHNYRVPESFQDKIIVIIGNANSGGDISKELAEVAKEVHVCGRRWPETAQIKQQGNIWLHPLVERACEDGTVKFQDGKSTRADVILHCTGYLYSFPFLDTEGVVTVKDGRIDPLYKHVFVPAFGPTLSFVGLPQKIVPFPLFQAQSKWISKLLSGKVKLPSESTMMSDLKQFYDELEANGITTYNTHTMFKRDQFVYCDWILEQCGAQPLDSWREAIYFSAHANKGTSTNYRDDWKDQDLLDTATKSLVELSERLSLQVK

>TnS000194937t05|Gnetum_montanum/1-533

MERIMLVLQQRHSALIDIGKEEEEMKKEKDNVNVNVNVEEEKEEMAEDLRQRLRQQQQQQQWRMMTMKTGGANGNGNGNENGKMTTTMKVCVVGAGPAGLVCARELLREGHHVQIYEQNQDVGGQWLYQAQPPPPPPGHDHDQHSQHSHSSIYASLRLRSGRETMGLLHYPFKIVPGRDPRRFPTHRELFLFIKDFAKDFGLLDIVKFNSRVEYIGMLGQPARNVKWLVRSSSTICGSVSENVYDAVVVCTGHYSKPKFPSIPGLKEWPGKQMHSHYYRVPDPFKNKIVLIIGNSHSGQDLSMELLAVAKEVHLASRPHHVTKAVKKLEYAHQNFHVKSEVKSVHKSGQVKFKDGTFIFPDFLFYCTGYSYSLPFLDTKGIVTIEDDRVGPLYEHIFPPMLAPSLSFVGIPRKVIALSFFESQAKWVAQVLSGKIKLPSVEEMMRSVEELYKTMDALGLPKHATHDIANFEYCDKLSDYSGYPHLEEWKKQLCLAALVRSEEDLETFRDSTDDEHLLDTAVASNPEVALCLQT

>ChrUn.fgenesh.gene.86|Osativa_v7.0/1-466

MAGLAAARELRREGLDVTVLEQSAGVGGQWLYDAATDGGDPLGMAGVHSSIYSSLRLNSPREVCGFSDFPFRPTNGGGGDARRYPVHGELLRYIREFCDVFGLMDAVRLDTTVVRVAMAPPRRDGSLRWTVRSKHNGDAETEEVFDAVVVATGHYSQPRLPSIDGMDKWRRRQLHSHSYRVPDSFAGEVVVIVGCNLSGKEVALELLRVAKEVHLSSKSTKEAMTPSMSKMLARYDNLHLQPLVEHLCEDGRVVFDDGSFVVADAVIYCTGYNYSFPFLETEGKVTVDDNRVGPLYEHVFPLALAPSLSFVGIPVKVILPRFAEVQARWVAQVLSGRRTLPSPGEMLRAVEEYNRAKEAAGLPKRQTHDLFLDLEYCDEYGERHCGFPRTEQWKKELIWSSISDMCDDIENFRDDYHDSDIVRDGRRLHGWASSPPLAPRPQEKDDDHDKEEDKHLGFGPSKFSLR

>LOC_Os07g02100|Osativa_v7.0/1-463

MLNPIMSSDRSSKKVCVVGAGMAGLAAARELRREGLDVTVLEQLAGVGGQWLYDAATDAGDPLGMAGVHSSVFASLRINGPRESIGFSDFPFRPTNDAGGDARRYPVHGELLRYIRDFCDAFGLMDAVRLNTTVTRVAMAPPRRDGSLRWAVRSKRHGEAETEEVFDAVVVAIGHYSQPRLPTIDGMDRWRRKQLHSHSYRVPDSFAGEVVVIVGCSVSGAELALELRRVAKEVHLSTKSTEETITSAMSKSVARYENLHLRPQVEHLREDGTVVFDDGSFVVADAVIYCTGYNYSFPFLDTNGKVTVDDNRVGPLYEHVFPPELAPSLSFVGIPGKVVLPVFIEVQARWVAQVLSGRRTLPSPEEMQRAVEEHTRGMEAAGLPKRWTHDMFLNLERCDDYGERICGFPRMEQWKKEIFVSSLSDMVDDIENFRDGYHDSDLVRDALRRHGWTPAEEDDDAHK

>LOC_Os07g02120|Osativa_v7.0/1-476

MVSDHDDGKLPAQWKKVCVVGGGMAGLAAARELRREGLDVTVLEQRGGVGGQWLYDAATDAGDPLGMAGVHSSVFASLRLNSPRESIGFSDFPFRPTNDAGGDARRYPVHGELLRYIRDFCDAFGLMDAVRLNTTVTRVAMAPPRRDGSLRWAVRSRRHGEAETEEVFDAVVVAIGHYSQPRLPTVDGMDRWRRKQLHSHSYRVPDSFAGEVVVIVGCSVSGAELALELRRVAKEVHLSTKSTEETITSAMSKSVARYENLHLRPQVEHLREDGTVVFDDGSFVVADAIIYCTGYNYSFPFLDTNGKVTVDDNRVGPLYEHVFPPELAPSLSFVGIPAKVLLPVFIEVQARWVAQVLSGRRTLPSQEEMQRAVEEHSRGMEAAGLPKRWTHDMFLDLERCDDSGERTCGFPRMEQWKKEIFFSSLSDMVDDMESFRDGYHDSDLVRDGLRRHGWTPVAPRPQEEDDDAKAIGVANI

>LOC_Os07g02130|Osativa_v7.0/1-468

MASCKKVCVIGAGVSGLAAARELRREGLDVTVLEQRGGVGGQWLYDTATDAGDPLGVAGVHSSMYASLRLITPREVMGFSDFPFRPGKDGDSGAGEVDARRFPGHAEFLRYIRELCDVFGLMDAVRLNTAVTRVAMAPPRRDGSLRWAVRSKHHGEAETEEVFDAVVVASGHFCQPRLPTIDGMDRWRRRQLHSHSYRVPDAFHGEVVVIVGCGISSKDIGLELRRVAKEVHLSAKSPEEAMTPAMSKILARYDNLHLHPQIEHLREDGTVVFVDGTCVVADAVVYCTGNTYSYPFLDTDGKVTVDDNRVGPLFDHVFPPALAPSLSFVGIPAMVVVPLFNEVQARWVAQVLSGRRALPSPEEMARAAEEYNRGREAAGVAKRRTHDILDLEYCDDYGERNCGFPRLEAWKKELMWSSYLTMCDNLETFRDDYHDSDLVADGLRLHGWISPATTLTQQGDDDKRPHLL

>LOC_Os06g33720|Osativa_v7.0/1-485

MKKKVCVVGGGLVGLAAARELQREGHDVSVLEQRGGVGGQWLYDHTAAIDGADPLGVAGVQSSVYASLRLITPREVTGFSDFPFSPTTVAGGGDARRFPSHAEFLRYLRDFCDAFGLMDVVRLNTRVLRVAADRDGWAVRSRRGEVETEEVFDAVVVAVGSYTQPRLPSIDGMEAWPGRQLHSHSYRVPDSFRGEVVVVVGCGFSGKDIALELRRVAREVHLSVRSTEEAMASPAMSKMLARYGNLHLRPQIARLCEEEDGAVVAFADGSRVAADTVVYCTGYSYSYPFLDTGGKVTVDDDNRVGPLFEHVFPPELAPSLSFLGIPNMVTTRFFEAQARWVAQVLSGRRALPPAGDMLRAAEEHARAMDAAGVARRRAHDVPDLGEEFCERSCGFPRLEEWEKELIWTSITAMRDDLESFRDDFRVTDLVADGLRRHGWILAPAPEPEPLRQEDVVAGDGEGEGVQPVQANQAAVASPLGFINSA

>Zm00001d018652|Zmays_RefGen_V4/1-458

MVSSKKVCVVGAGVSGLACARELLREGHDVTVMEQSGGVGGQWLYDPRADGGDPLGAAGAHSSMYASVRLISPRELTGFSDFPFFPRDDGTGDSRRYPGHAEFLRYIRDFCDAFGLMDVVRLNTKVLRVGLAAPRAADDGDGIKRWTVSWSRHRGCDGEVVTTEEEVFDAVVVAVGQYTQPRLPTIRGMDKWSRRQLHSHSYRVPDSFHGEVVVIVGFRHSGVDIALELSKLAREVHVSVKSMEALTPAVSKAVARHSDLHLHLQIECLREDGQVTFADGSRVAADSIIYCTGYDCSFPFLDTGGLVTVDDSRVGPLYEHTFPPALAPSLSFVGLPRMVLVPRFYEAQARWVAQALSGWRPLPPSEEMARSAEEYHRAREAAGVPRRLSHALFFDMDYCDEFGAKHCGFPPVEGWKRDLLSLSLASASDGAVESYRDSYHDSDLVREGLRSEGWWPWL

>Sobic.002G008332|Sbicolor_v5.1/1-233

MASSSKKVCVVGAGVSGLVSARELRREGHEVTVMEQSSGVGGQWLYDARTDSGDPLGVAGVPSSIYASLRLNTPRESMGFSDYPFVYPSIDDDDGDARRYPGHAEFLRYIRGFCDAFGLMDAVRLNTKVLHVGLLAPPGHDDDGGVTRWTVRCSSRRGDCEGEVVTTEETFDAVVVAVGQFTHPRLPTINGMDKWSRRQLHSHSYRTPDSFQDQVVVVVGCQSSGVDIALELS

>Sobic.002G008200|Sbicolor_v5.1/1-473

MASSSSKKVCVVGAGVSGLVSARELRREGHDVTVMEQSGGIGGQWLYDPRTDAGDPLGVAGAQSSIYASLRLNTPRETTSLSDFPFFPTNDGTGGDARRYPLHGEFLSYIRDFCGAFGLMDAVRLNTKVLHVGPLAPCGRGRGHDGAVTRWMVRWSRHGDCEGQVVTAEEVFDAVVVAVGQNTQPRLPTINGMDKWSRRQLHSHSYRSPDSFDDQVVVVVGCHPSGTDIALELCTVAREVHISVKSMDAAAVVPGMRRAVSRHDNLHLHLQIDCLCEDGQVMFADGSCVVADSIIYCTGYDFSFPFLDTGGLVTVDDNRVGPLFEHTFPPSLSFVGVPRMVVVPRFYEAQARWVAQVLSGRRPPLPPEEEMLRAAEYHHRAREEAGVPRRQSHNIFFDVDYMDEFGAKHCGFPRLPEWKKELLRSSVARLRDATESYRDDYRDSGLVREGLQAQGWLTGRPPPPPDTRVENES

>Zm00001d018648|Zmays_RefGen_V4/1-498

MHVAVKMMASSTEEELEMNKKKVCVVGAGVSGLVSARELRREGHDVTVMEQSGGVGGQWLYDPRTDASDPLGAAGVHSSVYASLRLTSPRDIMGFSDFPFFPRSNDDGDSRRYPGHAEFLRYIRDYCDTFGLMDAVRLNTKVLHVGRPLLDDDGVVTRWTVRYCSSGHGVSEHEAVAEEEFDAVVVASGHYSQPRLPAINGMDKWTRRQLHSHSYRVPDSFHAEVVVLVGFHQSGVDIALELCKVARDVHVVSVKSLEGLTPGVRKAVPRHHNLHLHLQIECLCEDGKVMFADGSCVVADSIIYCTGYDFSFPFLDTGGLVTVDDNRVGPLFEHTFPPALAPSLSFVGVPSQVQTPLFYEVQARWVAQALSGRRLLPPAEEMLRAAEEYHRAREKAGVPRRMSHAIFFDFDYCDEFGEKHVGLPRPPEWKKELMRAAVARLLQDTETFRDDYRDGDLVLESLRLEGWSSAPSPGTLNDKENGIAMHDMMHDRDGETLE

>Sobic.002G008266|Sbicolor_v5.1/1-213

MRKAVSRHHNLHLHLQIDCLCEDGQVVFADGSSVIADAVVYCTGYDLSFPFLDTGGVVTVDDNRVGPLYEHTFPPALAPSLSFVGVPKRVVVPRFYETQARWVAQVLSGRRMLPSSEEMMAWAEEHHRAREMAGMPKRHAHEFFDDYNCCDEFGEKHCGFPRLEDWKKELRWASVTRQRGDGSETFRDVYHDSELVRQGLLAQGWPAGSQQHK

>ELECO.r07.7AG0552100|Ecoracana_v1.1/1-394

MLCYPHAAAHKTERPDEHHGLCTHFRARVCLLAVGEMLSKKKKVCVVGAGVPGLVSVRELRREGHDVTVMEQSGGVGGQWLYDPKVDAGDPLSLAGVQSSIYASLRLIIPREVMSFSDFPFYPRDGGDGLRYPGHGEFLRYVRDYCDAFGIIDAVRLNTTVLRVAADGNSRWTVRSCTRQRGEDDAVVTTEEEEEEEVFDAVVVAVGQYTQPRLPTINGMDKWRRKQLHSHSYRVPDTFRGEVVVVVGCHESGKDIALDLCKVAMDVHISVKSVVDVAAGVPRAVSRHHNLHLHPQIKCLREDGTVVFADGSHLAADAIIYCTGYDYSFPFLDIGGAVTVDDDGGRVGPLFEHTFSPALAPELSFVGVPKKVVVPWFYELQARWVAQVLSGRRR

>ELECO.r07.7BG0583070|Ecoracana_v1.1/1-461

MGSIMVSTKKVCVVGAGVSGLVAARELHREGHAVTVLEQNNGVGGQWLYDTKVDAGDPLGLAGVHSGVYASLRLIVPREVMSFLDFPFYPRHGGDGRRYPGHGEFLRYIRDYCDVFGIMDAVRLNTTVLRVAAIVGDDGNTWWTVRSCTRRRGEDDDDDVVTTTEEEVFDAVVVAVGQYAHPRLPTINGMENWRRTHLHSHSYRVPDSFRGEVVVVVGCHESGKDIAVDISKVAMEVHISVKSVDDVTAGVHKTLSRHHNLHLRPQIECLCEDGTVVFVDGTRVVADSVIYCTGYDYSYPFLDTGGVVTVDENRVGPLFEHTFPPALAPELSFVGVPKKVVVPRFYEVQAKWLAQVLSGRRRLPPAEEMLSSVEEYNSAQEAAGVPKQSSHDLWDFEYCDEFGEKHCGFPRLPEWKKELMLSSIRNMYEDHENFRDNNYEHSELVKEGLLSEGWLPDAERP

>LOC_Os07g02140|Osativa_v7.0/1-510

MVSNHDDGKPPAKWKKVCVVGAGMAGLAAARELRREGHAVTVLEQAGDVGGQWLYDPRTDDPLGASPAPVRVHSSMYASLRLISPREAMGFTDFPFLPVDGAGGGGRDPRRFPGHREVLLYLKDFCDAFGLMDAVRLSLNTRVLRVAMAPPQCRAPAVAGGERKWVVRSVRVGERDDTGVQEEVFDAVVVATGHYSQPSVPTIKGMEAWRRRQLHSHSYRLPEPFRDEVVVMVGCGDSGKDIALDLISVAKEVHLTDKSTEEATTPAMSKLLAKYANLHLRPRVEHLCEDGTVVFVDGSRVVADTVMYCTGYVYSFPFLDTDGVVTVDDNRVGPLFEHVFPPALAPSLSFVGVPRKVPAPWFFEAQGKWVAQVLSGRRTLPPVEEMLRAVDEHYRARAAAGVPVKYTHELGGIEPQKYIEFGEKYCGFPRYEDWKREMIVSTISRRDDDDMETFRDRVDDDSDHVRLCLKSWHCSSPARHQASLAVSAADGHPPPPRLAQTAKAIAADAL

>LOC_Os02g37010|Osativa_v7.0/1-516

MAACVDDVGKPRASRKVCVIGAGMAGLAAARELRREGHAVTVLEQAGDVGGQWLYDDPRADDDDEDPLAAAAAAAKPVRVHSSMYASLRLLGPREVMGFSDFQFVPARKSRCGGGDNPGGRDPRRFPGHREVYLYLRDFYRAAGLTDSVRFNTRVVRVAVAPPPCRGGPGDALRWVVRSMDAGLWKRCTDDQMAEAHCVEEVFDAVVVATGHYSQPKLPSIQGMGDWKRRQMHSHWYRVPDSFRDEVVVLVGCGDSGMDIALDLLAVAREVHLSAKSVEAAATPAMSKMLARHANLHLHPQIERLCDDGRVVFADGGGGVVAADTVMYCTGYRYSFPFLDTEGKVAVDDDDNRIGPLFEHTFPPSLAPSLSFVGIPRKVMVPWFFEAQGRWIAGVLSGRRALPSEEEMTRSVEEFYRARELAGVPKAHTHDVEPHKMYELGEKYCDFPRTEEWKRELMAIISRNTSDDMETFRDRDDDSDNVRRCLQEWYALAEHQAQDEEDPAAAAAQAPVHSSL

>Bradi3g10560|Bdistachyon_v3.2/1-494

MKGLRTDGEWLQQKMKKKVCAIGAGMAGLAAARELRREGHEVTVLEQSGDVGGQWLYDPATDEADPLGALGPVKVHSSMYASVRLISPRETTGFTDFPFATMDGRDNRRFPGHREVYLYLKDFCDAFGLMDAVRLNTKVLRVAMTPSRRQWTVRSVGLTDADDKKEEVFDAVVVATGHYSQPRFPTIQVGMEKWRGRQMHGHSYREAEPFRGQVVVVVGTGESGKDITMELRDVAKEVHIVARSMEDVTPGLSKVLAKYSTNLHLKLNLERLCEEGRVVFGDGSVVVADAVIYCTGYNYSFPFLDTAGAVTIDDNRVGPLFEHVFPPSLAPSLSFVGIPRKVFAPRFFETQGRWVAQVLSGKRTLPTEEEMLRSVEEFYRARESAGVPKKYTHEIGGHDRTYMDEFGEKYCDFPRLERWKYELLVSSVTNMIDNFETFRDDYQDSDSILKGVQEWQLSARHRAAARTSTAVTGLLEESRTVITDMQQLSVISPG

>HORVU.MOREX.r3.4HG0400910|Hvulgare_Morex_V3/1-502

MDTACGGEPPQPKAVCVIGAGMAGLAAARELRREGHDVTVLEQSGDVGGQWLYDPRTDGADPLGAAAPVKVHSGVYASLRLISPRQSTGFSDFPFCPKTGRDNRRFPGHREVHLYLKDFCAAFGLMDAVRLNTRVLRAAMTPAGRWAVRSMDLGLGERDGDADGKALDTYVDEVFDAVVVATGHYSQPRLPGIKGMKTWRGRQMHSHSYRVPEPFRGEVVVVIGCGESGKDIAMEIRSVAKEVYLVAGSMEAVTPGLSKVLAKHSANLHLRLDVERLCEDGRVEFKDGGGSSVAADTVIYCTGYIYSFPFLETGGAVAVDDNRVGPLFEHVFPPSLAPSLSFVGVPRKIFAPRFFEMQARWVAQVLSGRRVLPAEEEMLLSVEEFYRAREAAGVPRKYTHEIGGRDRMYMDDFGEKCCDFPRLEGWKQELLTSSVRDMIEDLETFRDEHQDSDSIREAMQEWQLSCLHTKPPTTTSCCWLAATQAETVVPAKLLSINKQNDD

>HORVU.MOREX.r3.4HG0417160|Hvulgare_Morex_V3/1-489

MAAGDGEPALQSKSVCVVGGGVAGLAAARELRREGHAVTVMEQSGDVGGQWLYDPLTDGEDPLGAAAPVRVPSSIYACLRLISPREAMGFSDFQFLPREGAGRDPRRFPAHRELHCYLRDFCDAFGLMDVVRLNTRVLHVAPATTATRQWTVRSVRLLGSTEDDESREKEEVFDAVVVANGHHSQPMLPTDIQGMGEWTRRQLHSHSYRTPEPFRGEAVVVAGCGDSGKDIALDLCRVAREVHLAASSEATAATPDVSRMLANHGDVLRLHTRIRRLHADGRVEFADGSSVVADTVIYCTGYTYSFPFLDTGGAVTVDSDGYVVGPLFEHVFPPSLSFVGVVRKVLIPWFFEVQARWVAQVLSGRKTLPAEEEMVRSVEEHLRAREAAGVPWKHTHNIGGIDPQKMYELGEKYSDLAPVEEWKKELVMSSIASKMADVETFRDRADDSENVRKGVQAWLDLKLAAQPRAKSMSTVADDVEADARAMPHA

>ELECO.r07.2BG0183820|Ecoracana_v1.1/1-487

METVSDDVPPATESKREKKKVCVVGAGMAGLTAARELRREGHAVTVMEQSGDVGGQWLYDPRTDAGDPAAPVRVHSSMYASVRLLSPRECMGVSDFQFLPKHGVPGRDPRRFPGHREVFFYLKDFCHEFGIMEMVRLNTKVVRVAMASEVAARWQVRSVRVDPESGEEDEEEEEEVFDAVVVANGHYSQPRLPTITGMGEWRRRQMHSHSYRVPDTFRGQAVVLVGSGDSAMDIALDLICGSAKSVHISANTPVVDEKIMTPAMRKMLANHADLHLHQQIHRLHEDGTVAFADGTSIMADCIIYCTGYRYSFPFLDTGGAVAVDEDGWRVGPLFEHTFPPALAPGLSFVGVQKKLFVPWFFEAQGRWIARVLSGRTKLPPVEEMMTAVEQYRRDREVAGVPAKYAHDIGGVNPSETYEFWDKYSELPRREEWKRELTRAVLRDINDDRETFRDLDNDSDSVREGLQTWLGLSSDDALHQAAGAYAQV

>Adc18396|Adiantum_capillus-veneris/1-499

MRTALFRMAPSGAVAVGAASQAPKISSKLPQRRKLSVAVIGAGAGGLTATRELLKEGHQVVVFEQDSYGVGGVWRYLPQTEAEDPLGISPDCTRIHSSMYLSLRTNLPREIMGFLDFPFLPKYGDECRDARRYPDHAEVLAYLQDYALHFNLLPYVRFGRRVTQVAPIHKPTVHPESLAHPLPSKSEFRLPHQWSVTSEAEAGRMTMTDIFDAVVICNGHYFQPKLPLIPGIEKWPGTQLHSHNYRVPQPFADQIVVVIGNSSSGSDISREIATVAKEVHLVGRTWDSGVDLKKPAGPSQNIFLHPMVKLAQANGELLFEGGDTVVANAILYCTGYAFSMPFLETVGVINVDSDFVGRLYQHIFPPQLAPTLSFVGLPFKVIPFPLMQLQSKWIAKALSGKVLLPSTEQMSEDVEEFYKNLEAKGIPKSKVHDLGSCQFEYDNWLADQCGCQAIEDWRIQIYINSSKRKRALPDSYRDEWTDQELHELAIADLNQLQIA

>Ceric.34G032600.1.v2.1|Ceratopteris_richardii/1-502

MTMSSVCGAMSRWPITADHSARGEARDKQQIASAKLNKSLSVAVVGAGAGGIAATRELLKEGHRVVVFEQEIDGLGGVWRFSHRTEDEDPLGLSSDRSAIHSSMYLGLRTNLPREIMGFLDFPFLPKYGDSSRDARRYPGHVEVLDYLEDYAVHFGLLPCIRFGRRVTRVCMHARPKTRDDALIAPDCSNSGGCQWSVTTVAERDCKDCTTDMFDAVVVCNGHYFQPKLAPIPGIEEWPGIRLHSHNYRVPDPFADQVVVVIGNSSSGSDISREIATVAKAVHLVGRTWDSDVDLQKPAGPKQNISLHPMVKSAHEDGKLVFENGDSVIADVILYCTGYAYSMPFLQTEEIVNVNADYVGPLYQHVFPPRLAPTLSFVGLPFKVIPFPLMQLQSRWIAQSLSGKVTLPSEEQMLKDSLEFYERLDARGIPTNKIHDMGSYQFEYDNWLAAACDSSPIEPWRIQIYNNSSKRKRASPDTYRDEWSDQELHDLAIAELQQLQIV

>Mvestita_C2120g21607-RA|Marsilea_vestita/1-522

MATLCTLQGVTKLPMTTSPVMQLQSTANSKIKKPCSVAVVGAGAAGLVAARELQREGHRVVVYEQNREGVGGVWRYCPEIEEDDLLGVSPNRKSVHSSMYLSLRTNLPREIMGFLDFPFIPKEDNPSRDPRRYPGHEEVLRYLEDFALHYELLPLILFGRSVTHIGIKGIVVQDGASYHTIPVDGLDIPGLRSLLQLHLGVDASSKLHQIQWSVTSKAVGDDVDVITEDFDAVVVCNGHYFQPRISKIPGIEKWPGIQMHSHNYRVPDSFTDQVVVVIGNSSSGMDISREIAAVAKEVHWVARNWESNVDMKKPVGPFGNILLHPMVKSAHDDGSLTFDDGVSVVADIILYCTGYGFSLPCLQLEGIVNVDEDCVWPLYQHVFPPLLAPFLSFVGLPYKVVPFPLMELQTKWIAMILSGKVVLPSSEEMNKRLEEFYKGIEAKGLPRIKVHDMSSYQFEHDDWLANQCNHPPLESWRSEIYRDSSIRKRSIPDTYRDEWTDQNLHEIAMADLYKLGRSINYI

>Mvestita_S2g11791-RA|Marsilea_vestita/1-522

MATLCTLQGVTKLPMTTSPVMQLQSTANSKIKKPCSVAVVGAGAAGLVAARELQREGHRVVVYEQNREGVGGVWRYCPEIEEDDLLGVSPNRKSVHSSMYLSLRTNLPREIMGFLDFPFIPKEDNPSRDPRRYPGHEEVLRYLEDFALHYELLPLILFGRSVTHIGIKGIVVQDGASYHTIPVDGLDIPGLRSLLQLHLGVDASSKLHQIQWSVTSKAVGDDVDVITEDFDAVVVCNGHYFQPRISKIPGIEKWPGIQMHSHNYRVPDSFTDQVVVVIGNSSSGMDISREIAAVAKEVHWVARNWESNVDMKKPVGPFGNILLHPMVKSAHDDGSLTFDDGVSVVADIILYCTGYGFSLPCLQLEGIVNVDEDCVWPLYQHVFPPLLAPFLSFVGLPYKVVPFPLMELQTKWIAMILSGKVVLPSSEEMNKRLEEFYKGIEAKGLPRIKVHDMSSYQFEHDDWLANQCNHPPLESWRSEIYRDSSIRKRSIPDTYRDEWTDQNLHEIAMADLYKLGRSINYI

>Mvestita_C2120g21605-RA|Marsilea_vestita/1-495

MGILLDNKCSVAVIGAGAAGLVAARELHRQGHTVTVYEQNPHGIGGVWRYTPETEEDDPLGISPHRKSVHTSMYLSLRTNIPREIMGYLDFPFLPKEDDPIRDPRRFPVHQEVLHYLEDFAMHFDLLRFVQFGRRVKDVSIHRIVTGDKGSYNYHSVVADDLKNIHSVLELYSQRDQVHQIEWNVVSENVKDASDVSTDTFDAVVVCNGHYFQPKLVHVPGIDKWPGLQIHSHNYRVPDPFTDKIVVVIGNSFSGADISREIASVCKEVHWAGRKWDSCVNIGKPLNPYTNIIPHPMVISADQDGSIQFDDGASVNADVVLYCTGYGFSLPFLRIQSIVNVDPDHIWPLYQHIFPPALAPFLSFVGCISKAIPFPIMELQSKWIAMILAGKVVMPSRKEIKESIDKFYLDLKIKGLRSEDCHNLNSNQFEYANWLANLCQCSSLESWRSEIYLSCSIDRQLLPESFRDDWTNIESYTLAMTELQAFDIHTPCTLT

>Mvestita_S2g11793-RA|Marsilea_vestita/1-495

MGILLDNKCSVAVIGAGAAGLVAARELHRQGHTVTVYEQNPHGIGGVWRYTPETEEDDPLGISPHRKSVHTSMYLSLRTNIPREIMGYLDFPFLPKEDDPIRDPRRFPVHQEVLHYLEDFAMHFDLLRFVQFGRRVKDVSIHRIVTGDKGSYNYHSVVADDLKNIHSVLELYSQRDQVHQIEWNVVSENVKDASDVSTDTFDAVVVCNGHYFQPKLVHVPGIDKWPGLQIHSHNYRVPDPFTDKIVVVIGNSFSGADISREIASVCKEVHWAGRKWDSCVNIGKPLNPYTNIIPHPMVISADQDGSIQFDDGASVNADVVLYCTGYGFSLPFLRIQSIVNVDPDHIWPLYQHIFPPALAPFLSFVGCISKAIPFPIMELQSKWIAMILAGKVVMPSRKEIKESIDKFYLDLKIKGLRSEDCHNLNSNQFEYANWLANLCQCSSLESWRSEIYLSCSIDRQLLPESFRDDWTNIESYTLAMTELQAFDIHTPCTLT

>Azfi_s0032.g024784|Azolla_filiculoides/1-517

MAGALCSSANPPSPPPPLPTRLWQRQCSVGVIGAGAAGLVAARELLRQGHSVVIYEQSIDSIGGTWCYYSHSSSTPHHHQQHHVHSSMYLSLRTNLPREIMGFLDFPFLPCHRNTMVDEEEKEKPFLKSRDPRRYPHHREVLSYLQDFAHHFDLLRFIRFGRRVTHVHIKNITDRTSGVTATSTSSASTSDLLSILNSQLHLGGIQWCVESEAVNNHSDVTTDILDAVVVCNGHYFQPKFASIPGIEKWPGKQSHSHNYRVPDSFSDQVVVVIGNSSSGMDISRELALVAKEVHWAARTWPANVDLQKRVGASNNINLHPMVISAQEDGHLGFEDGTSILADTILYCTGYGYSLPFLHLEGIVNVDDDHIGPLYQHVYPPLLAPFLSFVGIPFKVIPFPLMELQSKWIAMALSGKLALPSSEEMKRWVDDFYDSIQAKGLPKSKIHDLSSCQFEYDDWLANECGCETLEIWRRKIYANSSYNKRASPDTYRDEWDDQDVYEAAMADLLQLQTIISRI

>Thupl.29379979s0003|Tplicata_v3.1/1-470

MSNKEVLNGISKVGVVGAGAAGLVAARELKREGFEVVVYEQNADVGGVWFYNPNAGGDPLGLELTGPNQGIAYQSLRSNITREVMGYMDYPFLVKEGRDPRRFPGHHEVFLYLKDFVRDFNLVEFIRFNTKVEYVGMVGESQGCVMTGEEGLDMDGDPMDERVQWVVRARSSGIVEEEVFDAVIVCNGHYRQPIIADIPGAQKWPGKQIHSINYRVPKPFSNQVVVIIGCSSSAFDISSELKMVAKEVHMSIRSKYSKYEKGASVHIDLHRLNVHLHPKVTYLDEDGTVVFEDGSTIIADSIIHCTGYSYSIPFLDTKGIVIVENKHVGPVYEQIFPPLLAPSLSFVGIPNKIICFPFFELQSKWISSILARKVKLPSRKDMVKLVKDLYDYHLEMYDLSKCLLHDIGSFEYCDQLADRSGTAHFEEWRKEIERETLKLILSNPGTFRDMWDENYLRELLEKSPSVMSDS

>Thupl.29379979s0004|Tplicata_v3.1/1-460

MANKDVFKGIRKVGVIGAGAAGLVAARELKREGFEVVVYEQNVDVGGIWLYNPNVGDDPLGLELTGPNQGTIYQSLRTNTARELTCFMDYPFLVKEGRDPRRFPGHHELFLYLKDFAREFNLVELIRFNTKVEYVGMVGEDQECVRNGDMDGDPMDDRVQWAVRARNSGIVEEEVFDAVVVCNGHYRQPIIAEIPGAQKWPGKQLHSFNYRFPEPFSNQVVVIIGKSESARDISLDLKTVAKEVHLSIRSTKLEKDTKFHIDLHRFNVHLHPMITCLEDDGTVMFEDGSTIIADSIIYCTGYSYSIPFLSTKGIVTVENYCVGPLYEHIFPPLLAPSLSFVGIPQMVLTFPFFEFQSKWIASVLARKVKLPSRRDMIKLVKDLYDHHMEISRLSRHRVHDIGNFEYYDQLADWSRASHFEEWKKDMATQNIKLIKSYPEMFRDLWNENYPHELMDKLPKP

>Zm00001d029924|Zmays_RefGen_V4/1-434

MRRRAEVAVVGAGAAGLVAARELLREGHTVSVFEKSGRVGGTWAYSPRADADPLGSDPGAPGAVHSSLYASLRTNLPRELMGFSGFPLAGRAFAGDPRTFPGHREVLAYLDAFAADSGVAPHVRLRAQVLRVRPLCPGRGQGEKWAVAWRGEDGDVAEEAFDAVVVCNGHCSVPLVPKIRGINKWRGKQMHSHNYRTPEPFQDQSVVVVGLGASGIDIAREISHVAKEVHISARYSEGRLGKIELYHNAWMHSEIECIQDDGQVRFAEGSSVAADTILYCTGYRYHFPFLDLDGFTVDDNRVGPLYKHVFPPKHAPNLCFVGLPYKSIIFQALELESKWVAAVLSGRAELPVEEDMTADVEEEYQRMEDAGKPKRHTHTLWPRWVEYLNWVADQVGEAHLEPRKCEMYEKALKCVWSLDEGYRDRWEEESIGSG

>Sobic.001G302732|Sbicolor_v5.1/1-435

MRRRAEVAVVGAGPAGLVAARELLREGHAVAVFEKSGRVGGTWAYDPRADADPLGRDPGAPGAVHSSLYASLRTNLPRELMGFSGFPLAGRVFAGDPRTFPGHREVLAFLDAFAVDSGVAAHVRLGAEVLRVRPLCRGQQGEQWAVAWRGEDGGVAEEAFDAVVVCSGHCSVPLLPKIRGINEWQGKQMHSHNYRTPEPFQDQSVVIVGLGASGIDIAREISHVAKEVHFAARYSEDRLGKIELYHNAWMHGEIECIQDDGLVRFAEGSSVAADTILYCTGYRYHFPFLDLDGFTVDDNRVGPLYKHVFPPKYAPNLSFVGLPYKSIIFSALELESKWVAAVLSGRATLPSEEDMMANVQEDYQRMEDARKPKRHTHTLWPRWVEYLNWLADQVGETHVEPRRSEMYEKALRCVWSLDEGYRDRWEEENIGCGSP

>Sobic.001G302500|Sbicolor_v5.1/1-436

MPRTVAVVGAGPAGLVAARELLRDDHAVAVFEKSGRVGGTWAYDPRADADPLGRDPGAPGAVHGSLYASLRTNLPREIMGFSGFPLEGRVFAGDPRTFPGHREMLAFLDTFAVDSGVAPHVRLGAEVLRVRRPLCRGQGEQWAVAWRGEDGEVTEEAFDAVVVCSGHCSVPLVLEIRGINKWQGKQMHSHNYRTPEPFQDQSVVVVGLGASGIDIASEISHVAKEVHIAARYSEDRLGKIELYHNAWMHGEIECIQDDGQVRFAEGSSVAADTILYCTGYRYHFPFLDLDGFTVDDNRVGPLYKHVFPPKYAPNLSFVGLPYKSIIFQSLELESKWVAALLSGRAALPSEEDMMADVQAEYQRMEDAGKPKRHTHTLWPRWVEYLNWLADQVGEPHVEPRRSEMYEKALRCVWSLDVGYRDRWEEENIDSGSPPWF

>Sobic.001G302666|Sbicolor_v5.1/1-437

MPRTVAVVGAGPAGLVAARELLREDHAVAVFEKSGRVGGTWAYDPRADADPLGRDPGGPGAVHSSVYASLRTNLPRELMGFSDFPLAGRVFAGDPRTFPGHREMLAFLDAFAVDSGVAAHVRLGGEVLRVRPLCRGQQGEQWAVAWRGEDGGVAEEPFDAVVVCSGHCSVPLVPKIRGINKWQGKQMHSHNYHTPEPFQDQSVVVVGLGASGIDIASEISHVAKEVHIAARYSKDRLGKIELYHNAWMHGEIDCIQDDGLVRFAEGSSVAADTILYCTGYRYHFPFLDLDGFTVDDNRVGPLYKHVFPPKYAPNLSFVGLPYKSIIFQSLELESKWVAALLSGRTAALPSEEDMMADVQEEYQRMEDAGKPKRHTHTLWPRWVEYLNWLADQVGEPHVELWRYEMHEKALRCVWSLDVGYRDRWEEENIDSGSPPWF

>ELECO.r07.2BG0169920|Ecoracana_v1.1/1-447

MPGARRDGGPTPMPHAARRVAVVGAGAAGLAAARELLRDGHAVRVFEKSSRAGGTWAYDPRADDDAGPLLGRDPGAVHSSMYASLRTNLPRELMGFSGFPLAGRAFAGDPRTFPGHREILAFLHAFAEESGVAKHVRFRTEVIRVAPLLRQGTSEQWTVEWRGEDGAVAEEVFDAIVVCTGHCTVPLVPKIRGIDKWQGKQVHSHNYRVPEPFRDQSVVVVGLGASGIDIAREISRVAKEVHIASRYCQDRLGKVELYPNTWMHAEIDCVQDDGQVRFAEGPAVPADTILYCTGYRYYFPFLDLDGFTVDDNRVGPLYKHVFPPKYAPNLSFIGLPSKSIVFQSFELEAKWVVAVLSGRVTLPSEEDMLAAVLKDYRRMEEAGTPKRHTHVLWPEWVEYFNWLADQVGEPHLEARRYEMYEKAIKCILSLDESYRDRWDEQEKQWAS

>Sobic.001G302400|Sbicolor_v5.1/1-435

MPRMVAVVGAGPAGLVAARQLLREGLHVAVFEKSGRAGGTWAYDPRADADDPLSRDPGAPGAVHGSLYASLRTNLPRDLMGFSDFPMAGRVFAGDARAFPGHREVLAFLDAFAEESGVAARVRLRSEVVRVAPALGHGERWVVAWRREDGEVVEEVFDAVVVCNGHWTVPMVPKIRGIDNWSGKQIHSHNYRTPEPFRDQNVVVVGFSASGIDIALEISHFAKEVHVADRYSKDRLGKIALYKNVWIHAEVDCIQDDGKVRFAEGSVVAADIILYCTGYRYHFPFLDLDGLTVDDNRVGPLYKHVFPPKYAPNLSFVGLAYKTIVFQTLEQESKWVAAVLSGRATLPSEEDMMAAVREHYQLMEEAGRPKRHTHTLSPQWAEHMNWLAGQVGEPPLEPWKREMFDRVLGSILTLDEAYRDRWEQEEDRDSGTGAH

>Bradi3g33047|Bdistachyon_v3.2/1-435

MLQRRARAVAVVGAGAAGLVAGRELLLEGHAVAVFERSARLGGTWAYDDEPPARARSSSMYASLRTNLPRELMGFSGHDLADGVFAGDPRVFPGHREVLAFLRAFADESGVASRVRLRAEVLRVAPVGGGEEEVEEEKRWSVAWRDLEKGEVAVEVFDAVVVCTGHCSVPLVPKLPGIENWQGKQMHSHRYRIPEPFRDEVVVVVGLGASGVDIAREISHVAKEVHIASRHDEHRLGKIDIYRNVWMHTEVNCIQDDGQVRFGEGTTMAADIILYCTGYRYHFPFLDHLEELTVDEDDNYVSPLYKHVFPPKYAPGLSFVGLPSKTIIFQTLELVSRWVARALSGSVALPDDEGMLAAVREDCRRMEESGKPRRHTHVLVPDWVEYMDWLAEQVGEPQLEARRRELYERALRCIWSLDDAYRDRWEEGGSGGGAL

>Klenit1_1_1302_kfl00117_0190_v1.1|Klebsormidium_nitens/1-480

MTVDVAANGKEGQKTRVSRSVCVIGAGAAGLVALKELREEGHQVTAFEQNEDIGGVWKYDPDTDDDPLGIREDRKKVHSSMYASLRTNLPREIMSFAAYPFVPSGEPGRDDRRFCSHQEVQAYLRDYARHFDLHRHIRFGTQVLRVSRLPEKTNQYGSVQQPLWEVVTRTASSSENWACDTTEFDAVVVCNGHYSQPKLATIEGIASWPGRQLHSHNYRTPGPFAGQTVVLIGAFASGEDISRELAEHCSEVYLSARSWKDEPRQTPPNLHRKLVVTRAYPDGSLEFADGSRVVADVIIHCTGYVYEFSFLESGLVSTEDQRVHPVYEQVFHVGTAPTLSFIGLPFKVSPLPMFELTTRWVAQCLSGRAVLPSEAAMHKWIDGFYEGLKAGGVPVRYTHEMSRTGFDYLARLCEWTGTPMFESWREDLFKDFSYRRHNYPANYRDEWPEDLELKRKVIWEDLRKEAEKLSLGGSEKSFGS

>Mapoly0020s0006|Mpolymorpha_v3.1/1-460

MKKRVAVIGAGPSGLSLLRAFDSAAEKGAEIPEIVCFEKQGEVGGLWKFEEGKGVDEHGEIVHGSMYRDLYINGPKEVNEYVDYSFEEHYGKVIGSYPPRPVLLSYIRGRAEKYNLCAKFSVRFNSSVSKISYSEDTAKFTVTVKDRSTARNGTEAKVYSEDFDHVVVAVGHFSTPNVPQFPGLEAFKGSVLHAHDVRDLSEFRGMDVLLVGSGYTAEDIACQCFKLGAASVTITFRSNPTGCCNWPESIKEVPLLERVDSNGRTCHFKDGSSKDVDAIILCTGYLHDFPFMVGDELRLVAGNRMWPLGLYQGVVLETHPKVFYLGMQAQFYSFTMFDAQAWYVRDVIMGRLTLPSSTEEMVAHSRKWREAELAALAKLDVKPFQGDYVKELIRHTDCPITPEFVDKTQQMGVDWDKHKALDIMSYRDHAFVSAVTGNLARTHHTPWMQNFDDSLESYVN

>Rn_03022.1_1|Ricciocarpos_natans/1-470

MGKKRVAIIGAGPSGLSLLRAFQSAEEKGAEIPDIVCFEKQRECGGLWNFSERTGVDEHNDVVHSSMYRDLYINGPKEVNEYVDYSFEEHFGKPISSYPPRPVLLDYIKGRAKKFKLCERFVRCHTKVQSVTYSEETDKFTVTVTDYSTSDYREWKVYSEQVDHVVVATGHFSTPNVAHFEGLSAFKGSLLHAHDLRDVTDFKGKDVLLVGSGYSAEDIACQCYKFGARSVTISYRSYPTGCCNWPDSIKEVPLLQRVDPNGRTCHFKDGSTKDVDAIILCTGYLHDFPFMPESLRLVTGNRMWPMGLYEGVVLESEPIVFYLGMQAQFYSFIMFDAQGWYVRDVIMGRLTLPSIQEMKAHSERWRVAEEEALAKRDVKTFQGDYVKELISQTDCPLTPEFIDETLQMGLDWDKHKAQDIMSYRDHPFRSVVTGNVARKHHTPWIQEALSSRTSECRNCASGSSQKLCEH

>Lc_composite_21270.1_1|Lunularia_cruciata/231-691

MAERRKRVAIIGAGPAGLSQLKAFQSAGEKGAEIPDIVCFEKQSDCGGQWIYSWRTGIDENGEAVHSSMYRDLWINGPVEINEYADYPIETHFGRAVPSYAPRPAILDYVRGRAEKAGLRKWVRFNSAVRMVTFNEESNIFTVSVLDRARDIMYSEDFDHIVVATGHFSTPNVPDFEGIGNFKGRLVHSHDLRDVFHVKEKDVLIVGGSYSAEDVACQCYKFGAKSITISYRSKPTGCIWPTGVMEIPQLEKVDRNGRTCHFKDGSSRDVDAIFLCTGYVHDFPFLAEGLRLRAKNRMWPLGLYEGVVLESNPRVFYLGMQAQFHTFNLFEVQAYYARDVIMKRLSLPSAEEMAGHSQEWRKREEEAIGKNSVVAMVHFQSDYVTEVVSLTDCPLTVEAIDGMRQFGIDWEQHRAQGVMSYRDHAFRSTVTGKVAPMHKTPWMTESAWSYRDHEIRCTVTE

>Lc_composite_16566.1_1|Lunularia_cruciata/1-468

MAERRNRVAVIGAGPAGLAQLKSFQSAGEKGAEIPDIVCFEKQSDCGGLWIYNWRTCIDENGEAVHSSMYRDLWTNGPVNEYADYSIETHFGKPVPSYAPRPAILDYIRGRAEKAGLRKWVRFNSAVRMVTFNEESSAFTVTVLDRARDIMYSEEFDHIVVATGHFSTPNVPEFEGIGNFKGRLVHSHDLRNVFDVKKKDVLIVGSSYSAEDVASQCYKFGAKSVTISYRSRPTGCIWPKGVVEIPQLEKVDRNGRTCHFKDGSSRDVDAIFLCTGYLHDFPFMTEGLRLRAKNRMWPLGLYQGVVLESNPRVFYLGMQAQFHTFSLFDVQAYYARDVIMNGLSIQSAEEMVGHSQEWRRREEEPLWKSNVIAMVHFQSDYVTEVGGLTDCPLTVEAIDAIRQAGIDWYQHKIQDLMSYRDHGFRSTVTGKVAPISKTPWMKEFDDSLENYLTSSTATSYVITHLHEL

>Lc_composite_26202.1_1|Lunularia_cruciata/54-515

MTETRVRVAIIGAGAAGLSQLSAFQSALEKGAGIPDIVCFEKQSDCGGLWIYNWRTGTDENGETVHTSMYRDLWTNGPKECDEYADYSYEEHFGKAVPSYAPRSAILDYIKGRATKAGLLKWVRFNSVVRMVTFNEENSTFSVTIFDRAEDKMYSEEFDYIVVASGHFSTPNVPEFEGIGNFKGRLVHSHDLRNVIDLKDKDVLLVGSGSSAEDIACQCCKFGAKSITISYRSRPTGCIWPSHVMEVPMLERVDINGRSCQFKDGSTKDVDAIILCTGYLHDFPFMAEGLRLRAKNRLWPLGLFEGVVWESNPKVFYLGMQAQLFSFNMFEAQGYYVRDVIMKRLTLPSTEEMVAHSQVWRRVEEEAIARKSIRELIIFQTNYVKELVDQTDCPFTVKMIEAMKPIGMHWENHRKQDMRSYRDHEFRSIVTGNMAPVHHTPWMKEFDDSLANYLDPFRATAY

>Sphfalx14G087800|Sfallax_v1.1/1-543

MLKGQAVKETAILFSSTLDITLPPPPNSMKVAIVGAGLSGLVTAKVLAQFGHKVTVYEKCPDVGGTWSRTRWYPGLTTQNGKDSYCFSDFPMPDSYPEWPSGEQVHQYLDEYVKHFGLDALLQLNTEVVSIELDEVAGNIWTIGTRQSDNNKDSSMPILATQSETFDHVVIANGIFSDPYVPTFPGLEEFQQAGGQVLHTTDVHQLLGDATGKHFIIVGYGRSACDVACALSDISSIKVVVVARELIWKIPKWIGGVLNFKYLLLTRMGEALFQYIHPHGFEQFLHNGLGKPLRDLMMSSLQAIITCQLKLRKLGLLPDGPLEDIARTTLSLVTDGFYEKVEMGAIEVHGGTHIVKLCVIDDKPSTLLSDGTIVPTDFVICGTGFHQSVPFFSMNIIKQLTDDQGNFLLYRHIHPINVPQLSFNGYNSSLFCPLSSEVAALWIGNLLAGGMKLPSPNKQQLEVIEQLAWMEKRTKGKHACGGSIIPFTFHYIDELLKDMNLTLNAHTHIREWLLPINFGAYVGLIDELTKRLASEGKLTFPLC

>MA_218308g0010|Picea_abies/1-407

MDSSCGQLSGELARNEDCSTVKQQQQQSCVWVNGPIIVGSGPSGLATAACLQEQGIPCVILEKANCIASLWQQRTYDRLKLHLPKRFCELPLMAFPEEFPTYPTKNQFIHYLESYAHRFNLNPRFNECVESAHYDKICGMWRIRTENRCGEEAAEYMCRSLIVATGENAEPVIPYIKGMENFNGQIIHASNYKSGADFHSKNVLVVGCANSGMEVSLDLSNHNANPFIVARSSVHVLPREMFGISTFGVAIWLMKWLPVWLVDWFLLCVAWLMFGSTDRYGIKRPLMGPLELKNKAGKTPVLDVGAMDKIRNGRIKILPGLDCLTQHGAKFVNGEEMEFDSVILATGYRSNVPSWLKDCSFFSEDGIPNTPFPNGWKGENGLFAVGFTKRGLLGASIDAMHIAEDMG

>PITA_06172|Pinus_taeda/1-428

MDSFRRLLSGKLACNEDNSTVKQQQRCVWVNGPIIIGSGPSGLATAACLQEQGIPCVILEKANCIASLWQQRTYDRLKLHLPKRFCELPLMPFPEEFPTYPTKNQFIDYLESYARRFNLKPRFNECVESAHYDKMCGMWRIRTDNSCGEKMEYTCSWLVVATGENAEPNIPDVKGMENFKGQIIHASNYKSGASFHGKNVLVVGCANSGMEVSFDLSNHNANPSMVVRSAVHILPREMFGRSTFGIAVWLMKWLPLWLVDMFLLCVAWLMFGSTERYGIKRPLMGPLELKNKAGKTPVLDVGAMDKIRNGQIKILPGLDCLTHHGAKFVNGEEMEFDSVILATGYRSNVPSWLKDCSFFSEDGIPNTPFPNGWKGENGLFAVGFTKRGLLGASIDAMHVAEDIGRLRKTLNRSFSFCKSFSFRKRQST

>PITA_03225|Pinus_taeda/1-428

MDSFRRLLSGKLACNEDNPTVEQQQRCVWVNGPIIIGSGPSGLATAACLQEQEIPCVILEKENCIASLWQQRTYDRLKLHLPKRFCELPLMPFPEEFPTYPTKNQFIDYLESYARRFNLKPRFNECVESAHYDQICGMWRIRTENSCGEKVEYTCSWLVVATGENAEPIIADIKGMENFKGQIIHASNYKSGAAFHGKNVLVVGCANSGMEVSLDLSNHNANPSMVVRSAVHVLPREMFGRSTFGVAIWLMKWLPLWLVDMFLLCAAWFMFGSTERYGIKRPLMGPLELKNKAGKTPVLDVGAMDKIRNGQIKILPGLDCLTHPGAKFVNGEEMEFDSVILATGYKSNVPSWLKDCNFFSEEGIPNTPFPNGWKGENGLFAVGFTKRGLLGASIDAMHVAEDIGRLRKKMNRRFSFCKSFSFRKRESA

>PSME_00049131-RA|Pseudotsuga_menziesii/1-427

MDSSCEQLNREYGHDTDQDCFIVKQQQQQQRCVWVNGPIIVGSGPSGLATAACLQEEGIPCVILEKEDCIASLWQQRTYDRLQLHLPKRFCELPLMPFPEEFPSYPTKNQFIHYLESYAHRFNLKPRFNECVESAQYDKVCGMWRISTENRCGEKSEYICRWLIVATGENAEPIIPYIKGMENFNGQIIHASNYKNGAPFHGKKVLVVGCANSGMEISLDLSNHNANPSMVVRSAVHVLPREMFGRSTFGIAIWLMKWLPVWLVDCLLLCVTWLMFGNTDRYGIKRPPMGPLELKNKAGKTPVLDMGAMDRIRNGQIKILPALDCLTPHSAKFVNNEEMKFDSVILATGYKSNVPSWLKDCRFFSEDGIPNTSFPNGWKGENGLFAVGFTKRGLLGASIDAMHIAQHIGRLWKKLTERFSFCSREST

>PSME_00029739-RA|Pseudotsuga_menziesii/1-424

MDSSCEQLNREYGHDTEDCFTVTQQQQRCVWENGPIIVGSGPSGLATAACLQEEGIPCVILEKEDCIASLWQQRTYDRLQLHLPKPFCELPLMPFPEEFPSYPTKNQFIHYLESYAHRFNLKPRFNECVGSAQYDKVCGMWRISTENRCGEKAEYICRWLIVATGENAEPVIPYIKGMENFNGQIIHASNYKNGAAFHGKKVLVVGCANSGMEISLDLSNHNANPSMVVRSAVHVLPREMFGRSTFGIAIWLMKWLPVWLVDCLLLCVTWFMFGNTDRYGIKRPPMGPLELKNKAGKTPVLDMGAMDRIRNGQIKILPALDCLTPHSAKFINDEEMKFDSVILATGYKSNVPSWLKDCRFFSEDGIPNTPFPNGWKGENGLFAVGFTKRGLLGASIDAMHIAQHIGRLWKKLTERFSFGSREST

>Gb_39502|Ginkgo_biloba/1-428

MDHCLSEQWGKEVHDIYIKKEQQQRSVWVNGPIIVGGGPSGLATAACLRERGVPCVILERADCIASLWQRRTYDRLKLHLPKHFCELPHMPFPQEFPTYPTKNQFIQYLESYADHFKLNPRFNERVESAWFDETCGLWRVKTVNSSTSGEEAEYICRWLIAATGENAEPVIPDINGVESFNGGIIHASTYQSGADFQGQNVLVVGCANSGMEVSLDLCDHNARPYMVVRSTVHVLPREMFGTSTFGVAMRLMKWFPVWLVDALLLLVAWWMFGSTDRYGIKRPSMGPLELKNKAGKSPVLDVGAVAKIKRGQIKVLPGIDYLTSHGAKFVNGQHMDFDSIILATGYKSNVPSWLKECNFFSADGMPNTPFPNGWKGQNGLYAVGFTRRGLLGASIDAMHVAEDVGRLWKEDTKQFTLYHSELRSDQNP

>PSME_00033487-RA|Pseudotsuga_menziesii/240-659

MDAACCLSEQWGKEAHDCLLEKQRIVYVDGPIIVGAGPSGLATAACLTEEGVSCLILEKADCVASLWQHRTYGRLKLHIPKQFCELPLMPFPDDFPMYPTKEDFIKYIESYVTKFNLRPKFNECVQSASYDRTLGLWRVRTVSTIGYDNKNKNIEYICPWLVVASGENAEPIVPSIVGMRSFSGSLIHSHEYKTGADYEGKNVLVVGCGNSGMELCLDLFHHKAKPSMVVRNTVHVLPREMFGKSTFGLAMALMKRLPIWWVDRLLLLFASLKFGNTERYGLRRPVLGPMELKSQTGKTPVLDVGTLAQIRNGNIKVLPAISCLTPCGAKFVDGQKLEFDSVILSTGYKSNVPSWLQDSDFFSKDGMPKTPFPNGWKGENGLYSVGFTRRGLLGASLDAQRIAQDIGELWRSGRKQQPTY

>PITA_32685|Pinus_taeda/1-420

MDACCFSERWGKEAHDGLLEKQRCVWVNGPIIVGAGPSGLATAACLREQGVSCVILEKADCLVSLWQQRTYNRLKLHLPKQFCELPLMPFPDRFPTYPTKEQFIEYIESYAKEFKLQPRFNQRVESASYDRTCGHWRVRTVNTAACDNMNKNTDYISRWLVVASGENAEPIVPDIAGVKSFRGSVIHASRYRTGADYEGKKVLVVGCGNSGMELCLDLFNHNAKPFMVVRNTVHVLPREMFGRSTFGVAMALMKRLPLWMVDRFLLAVAYFKFGNTECYGLKRPALGPMELKDKTGKTPVLDVGTLAQIKNGNIKVLPAISCLTPCGAKFLDGQKLEFDSVILCTGYRSNVPNWLKDSDFFSDDGMPKTPFPDGWKGENGLYSVGFTRRGLLGASLDARRIAQDIGGLLRAGRKQQFVLA

>PITA_21706|Pinus_taeda/1-420

MDACCFSERWGKEAHDGLLEKQRCVWVNGPIIVGAGPSGLATAACLREQGVSCVILEKADCLVSLWQQRTYNRLKLHLPKQFCELPLMPFPDRFPTYPTKEQFIEYIESYAKEFKLQPRFNQRVESASYDRTCGHWRVRTVNTAACDNMNKNTDYISRWLVVASGENAEPIVPDIAGVKSFRGSVIHASRYRTGADYEGKKVLVVGCGNSGMELCLDLFNHNAKPFMVVRNTVHVLPREMFGRSTFGVAMALMKRLPLWMVDRFLLAVAYFKFGNTECYGLKRPALGPMELKDKTGKTPVLDVGTLAQIKNGNIKVLPAISCLTPCGAKFLDGQKLEFDSVILCTGYRSNVPNWLKDSDFFSDDGMPKTPFPDGWKGENGLYSVGFTRRGLLGASLDARRIAQDIGGLLRAGRKQQFVLA

>Gb_32507|Ginkgo_biloba/1-425

MDFSAAEQMGKEAHDGYIVQQANEQRRMWVNGPIVVGAGPSGLATAACLRQEGVPCIILEKADCIASLWQHRTYDRLKLHLPKQFCELPLLPFPRDFPTYPDKDQFVEYLESYSKRFCLQPRFNECVQSAFYDQTDHLWRVRTVNTRAESATNVETDYICDWLIVATGENAEPVIPDVAGMDNFNGTIIHASAYKTGAGYRDKNVLVVGCGNSGMEMSLDLCNHNAKPSMVVRNTVHVLPREMFGKSTFGIAMALMKWLPLWVVDRLLLAVAGIMFGNTDRYGLRRPETGPIELKNKAGKTPVLDMGALAHIRSGHIKVLPAIDCLTPRGAKFVNGQEMEFDSVILATGYKSNVPSWLKDCDFFSQDGMPKTPFPNGWKGENGLYSVGFTRRGLLGAAVDARRIAEHIGGLWKAGTKQLILCRRP

>CYCAS_029312|Cycas_panzhihuaensis/1-435

MDCFTEQWGKIAHDSFNTNNNDDSDKQAQPQRCVLVNGPIIVGAGPSGLATAACLREEGVPCIILEKADCIASLWQHRTYDRLKLHLPKEFCELPLMPFPDDFPAYPNKVQFIEYLQSYSDRFDLHPRFNECVQSASYDHTCGLWKVRTTTASAKNGGSRLNRVEYISRWLIVATGENAEPIIPDILGMDSFHGTVIHARDYKTGADYGGKNVLVVGCGNSGMEVSLDLSNHNARPSMVVRSTVHVLPREMFGRSTFGIAMKLMKWFPLWVVDRLLLVVACIMFGNTDRYGLRRPAAGPLELKSKAGKTPVLDVGALAHIRRGHIKVLPAVERLTSRGAKFVNGEETEFDSVILATGYKSNVPSWLKECDFFSEDGMPKSPFPNGWKGENGLYSVGFTRRGLLGASADAKRIAQDIGGQWKAGTYKHFILCRTRP

>KAH9298013.1|Taxus_chinensis/1-190

VHVLPREMFGISTFGIAMALGKWLPLWIVDRLLLTVARFMLGSTERYGLRRPAVGPIGLKNNAGKTPVLDVGAFARIKSGRIKVLPAVHCFTSCGAKFVDGQEIEFDSVILATGYTSNVPSWLKKCDFFTEDGMPKTPFPNGWKGENGLYSAGFTRRGLRGVALDARRIAGDIGALWTGVRKQLVLCRRH

>CYCAS_004167|Cycas_panzhihuaensis/1-425

MESFFSEQSGKEAHDDYYAKKKSKQRCVWVNGPIIVGAGPSGLATAACLQEKGVASLILEKADCIASLWQHRTYDRLKLHLPKQFCELPLMPFPQHFPKYPDKLQFIAYLESYAQRFNIHPEFNRCVQSAFFDQTCGLWRVRTRGNAKELGGGETEYFCRWLVVATGENAEPIIPELCGIEKFDGQILHTSKYTSGEDYQGQNVLVVGCGNSGMEVSLDLSNHNAKPSICARSTVHVLPREMFGISTFGLGMWLLKWFPLWLVDRLLLLAAWIILGNTDRYGLSRPKMGPLELKNKAGKTPVLDVGTLAKIRKGEIKVLPALERLTSRGVKFVNGEEMEFDSVILATGYRSNVPLWLQERDLFSEDGMPKKPFPNGWKGESGLYAVGFTKKGLLGASMDAKRIAEDIATRRKEEAKQFTFCHRSL

>Thupl.29377126s0001|Tplicata_v3.1/1-430

MDRFCEQVGKEANDDYLNRKRCVWVNGPVIVGAGPSGLATGACLKEQGVPSIILEKADCIASLWQRRTYNRLKLHLPKQFCELPRKPFPEDFPTYPSKSQFIDYLESYAKEFELNPRFNECVQSAKYDETCGLWRVSTKSCPKGGLAVREFEYICRWLIVATGENAEPVIPDIAGMECFRGQIIHACEYKNGASYQGKKVLVVGCGNSGMEVSLDLANHNANPSMVVRSQVHVLPREMFGKSTFGVAMAMMRWLPLWLVDRLLLFIAWLMLGNTGRLGLPRPKAGPIELKNVAGKTPVLDVGALKKIRSGHIKVVPAVECFTAHGAKFADGTEQNFDAVVWSTGYKSNVPYWLEKCDFFSKSGMPKTPFPNGWKGGSGLYAVGFTRRGLLGTSIDARRIAKDIGTTWKEDNKQTKTNQFKSCHRRCISQF

>PSME_00051847-RA|Pseudotsuga_menziesii/1-450

MVMERFSEQYGKEANDLIIKSSSSEQHGKEANDDYLNRKRCVWVNGPVIVGAGPSGLATAACLKEQGVPSIILEKADCIASLWQRRTYNRLKLHLPKQFCELPRMPFPADFPTYPSKKQFIEYLERYAREFELSPSFNVRVQSAKYDETCGLWRVSTVNTGYPKGLGVPEAEYICRWLIVATGENAVPVIPDIAGKEKFNGKIIHACSYKNGAGYEGKNVLVVGCGNSGMEVSLDLANHNANPSMVVRSDVHVLPREMLGKSTFGVAMALMKWFPLWLVDSFLLFIAWIMLGNTHQYGLRRPKMGPLEMKNVAGKTPVLDVGALAKIRSCDIKVVPAVECLTPHGAKLVNGKEIEFDAVIWATGYKSNVPSWLEKCDFFSKSGLPRQPFPNGWKGESGLYAVGFTRRGLLGSSVDARRIAQDIGNVWKAETKQTKQQTFKSCHRRCISQF

>PITA_05784|Pinus_taeda/1-448

MERFSEQYVKEANDLNINSSSSEQYGKEANDDYLNRKRRVWVNGPVIVGAGPSGLATAACLKEQGVPCIILEKADCIASLWQRRTYNRLKLHLPKQFCELPRMPFPDDFPTYPSKKQFIEYLESYATKFALSPRFNVRVQSAKYDETCGLWRVSTVNTGYPKGLGVPEAEYICRWLIVATGENAVPVNPDISGKESFNGEIIHACSYKNGARYEGKNVLVVGCGNSGMEVSLDLANHNAKPCMVVRSNVHVLPREILGKSTFGVAIALMKWFPLWLVDRFLLFIAWIMFGDTYQYGLTRPEIGPLEMKNVAGKTPVLDVGALAKIRSGHIKVVPAVEYLTTHGAKLVNGKEMEFDAIIWATGYRSNVPSWLEKCDFFTKSGLPKQPFPNGWKGESGLYAVGFTRRGLLGSSVDARRISQDIGNVWKAETKHTKQQMFKSCHRRCSSQF

>Gb_40961|Ginkgo_biloba/1-455

MDRFCEQYGKEANDDYLNRKRCVWVNGPIIVGAGPSGLATGACLREQGVPCIILEKADCIASLWQRRTYNRLKLHLPKQFCELPRMPFPEDFPTYPCKKQFIEYLECYAKRFELNPKFNVCVQSAKYDETCGLWRVATVNTSYPKGLADREAEYICRWLIVATGENAEPVIPDISGMDRFRGKIVHASSYKNGAEYQGQNVLVVGCGNSGMEVSLDLANHKANPSMVVRSNVHVLPREMFGKSTFGVAMAMMKWIPLWLVDRLLLFIAWLMLGSTEKYGLRRPEMGPLELKNKAGKTPVLDVGALAKIRSGHIKVVPTVECFTPHGAKLVNGKEMEFDSVILSTGYKSNVPSWLEKCDFFSKSGMPKRPFPNGWKGESGLYAVGFTRRGLLGTSVDARRIAKDIGNLLVPQQVEKNKHPTGTQQGQKAVHNRKEEGLKSLLLSGGLCSDKEFCKA

>TnS000225445t01|Gnetum_montanum/1-447

MARRYSYRKEGMALASIAESEQIGKEANDDYVNSMKRCVWVNGPVIVGGGPSGLATAACLKEQGVPCIILEKADCIASLWQRRTYNRLCLHLPKQFCQLPRMPFPEHFPTYPSKNEFIRYLESYAQRFELSLRFNVRVLAAKYDDTCELWRVSTVSTGPGNSVGLREAEYICRWLIVATGENAVPVIPDIPGLDAFSGDIVHASKYKNADTYCAKDVLVVGCGNSGMEVSLDLANHGARPSMVVRSQVHVLPREMLAMSTFGVAMALMRWFPLWLVDKFLLFMAWIMLGNTARYGLQRPGMGPLQLKNEAGKTPVLDVGALAKIRNGDIKVVPAVHFFTAHGAVFTDGSRCKFDAVILATGYRSNVPSWLEKCDLFSKTGMPKQSFPNNWKGESGLYAVGFTRRGLLGASMDATRIAKDIGAVWKAETKQTRQHSFKSCHRRCISQF

>CYCAS_023462|Cycas_panzhihuaensis/1-415

MSVNMDEKWIQEIQNSYSEKQQRCVWINGPIIVGAGPSGLATASCLQELHGIPCVILEKADCIASLWQHRTYDHLKLHLPKHFCELPLMPFPAEFPTYPTKHHFIQYLESYAKKFELNPRFNECVHSASYNNTSGLWKIVSVKYSYYSNLPVNQEDQTEYICRWLIVATGQNAEPMLPDINGMEDFNGPIFHASTYKSGENFQGKNVLVVGCANSGMEVSLDLSDHNAKTAMVVRSAVHVLPREIFGRSTFEVAMWLTKWFPLWIVDGMLLFVTWLMFGNTNKYGLRRPSMGPLELKNKQGKTPVLDVGAMARIKSGKIKILPGLDHLTSHGAKFLTGQHLDFDSVILATGYKSSVPLWLKECSLFADNGMATGWKGENGLYAVGFTGRGLLGASADALLVAEDILPSWETENST

>Thupl.29380002s0001|Tplicata_v3.1/1-449

MDSSCCLSEAMGKEAQDSYMERHRGVEVDDISWVMGKEEQSCVRVKKKMDGCVMVKGPIIVGAGPSGLATAACLKEEGVPSLILEKADCVASLWQRRSYDRLKLHLPKQFCELPLMPFPSDFPRYPTKEQFVHYLESYARHFDLQPRFKESVLSASFDKECGVWRVRAMQSGGGRIEYVSRWLVVATGENAEAVIPDIVGMGSFKGSVIHASEYKNGAAFKGKSVLVVGCGNSAMELSLDLCDYNANPSMVVRNTVHVLPREILGMSTFGIAVTLGKWLPLWAVDRLLLTVAGVIFGRTERYGLTRPAVGPIELKSKSGKTPVLDVGALALIREKRIKVLPAVKCFTSHGAKFVDGQEIQFDSVILATGYTSNVPYWLKDSDFFTKEGMPKKPFPNGWKGENGLYTVGFTRRGLLGAALDARSIARDIGGLTKDLVSCHTQHSYTHKQD

>TnS000183447t02|Gnetum_montanum/1-453

MESKSEWVKGAVIVGGGPSGLATAACLQQHGVPCVVLERAECVASLWQQRTYDRLRLHLPKHFCELPLMPIPDHFPTYPSKDQFVAYLKAYATRFGIQPLFRRRVESARYDRKCGMWRVTTVATGEEKEEEEVIEYLCRWLVVATGENAEPFVPEFPGSNEFRGSVIHSSSFKSGAEFSAKRVLVVGCANSGMEISLDLCNHNAFPSMVVRSSVHVLPREIMGMFSTFGVAIWLMKWIPVWVVDMLLIMLAWLMLGSTKRYGITRPSMGPLQLKSLAGRTPVLDVGAMALIRKGRIKVMPALECLTRTGARFVNGQEQDFDAVILATGYRSNVPSWLKDCTFFSEEGIPNTPFPNGWKGENGLYAVGFTGKGILGASVDAVHIADDIQGLWNKFNRHISFCNKLTRHLSFRLIRTSSSSSLSSSTPSTPQSLSLSPRFRSSAPRSAPSSSLFS

>evm.TU.supercontig_91.28|Cpapaya_ASGPBv0.4/1-310

MESYADHFCIHPRFNQTVQNAEFHPTSGLWTVRTQDRRYISRWLVVATGENAEPVVPEIPGINKFGGAIVHTSLYKSGSDFKNQRVLVVGCGNSGMEVCLDLCRYNAIPHMVVRNTVHVLPREMLGFSTFGIAMALLKWFPIKLVDKLLLIMANLTLGNTDKLGLRRPKTGPIELKNITGKTPVLDVGALSQIKSGKIKVMEGVKEITRNGARFMDGQEREFDSIILATGYTSNVPNWLEGCDFFTKDGMPKTPFPNGWKGENGLYTVGFTRRGLLGTASDAVKIAQDIDRQWRTVKDYKNICNSQINLI

>Gohir.A05G009900|Ghirsutum_v3.1/1-421

MGSYKGEDQHRHHHHRNESNCVLVHGPIIVGAGPSGLATSACLKQQSVPSLILEKSDCIASLWQHRTYDRLKLHLPKQFCELPLLGFPHNFPKYPTKHQFISYMETYASHFSIKPRFNQAVIKAEFDHVLGFWRVKTQDFEYLSRWLIVATGENAEPVIPDIPGIDRFKGRVVHTSLYKSGSVFKNQRVLVIGCGNSGMEVCLDLCRFNVIPHMVVRNTVHVLPREMFGFSTFGIAMALVKWFPLKLVDKLLLLLANFTLGNTDQMGLRRPKTGPIELKNVTGKTPVLDVGALSQIKSGKIKVIEGVKEITRNGAKFVDGQEKEIDSIILATGYKSNVPTWLKGCEFFTKDGMPKSPFPNGWKAETGLYTVGFTRRGLLGTASDAVNIAKDIGEQWRTIKECNDSNFNYLPQRNVVQKVKK

>Gohir.D05G010600|Ghirsutum_v3.1/1-422

MGSYKGEEQHHHHHHRNESNCVLVHGPIIVGAGPSGLATSACLKQQSVPSLILEKSDCIASLWQHRTYDRLKLHLPKQFCELPLLGFPHNFPKYPTKHQFISYMETYASHFSIKPRFNQAVIKAEFDHVLGFWRVKTQDFEYISRWLIVATGENAEPVIPDIPGIDRFKGRVVHTSLYKSGSVFKNQRVLVIGCGNSGMEVCLDLCRFNVIPHMVVRNTVHVLPREMFGFSTFGIAMALVKWFPLKLVDKLLLLLANFTLGNTDQIGLRRPKTGPIELKNVTGKTPVLDVGALSQIKSGKIKVVEGVKEITRNGAKFVDGQEKEIDSIILATGYKSSVPTWLKGCEFFTKDGMPKSPFPNGWKAEKGLYTVGFTRRGLLGTASDAVSIAKDIGEQWRTIKECNDSNFNYLPQRNVVQNIVKK

>Cucsa.162080|Csativus_v1.0/1-399

PPPKCIWVHGPIIVGAGPSGLAAAACLSHNQIPSLILEKSDCIASLWQYRTYDRLKLHLPKQFCELPLMGFPENFPKYPSKDQFISYMESYASHFSIHPRFNQTVLAAEFDSVSGFWKVSTQDSQYISRWIIVATGENAEPVIPEIVGIERFARTVVHTSMYKSGSEFKNQRVLVVGCGNSGMEVSLDLCRQNAIPHMVVRNTVHVLPREMFGFSTFGIAMGLMKWLPLRLVDKILLLVANLTLGNTDHLGLRRPKTGPIELKNATGKTPVLDVGALSQIRSGKIKVMEGVKEITRNGAKFIDGQEKEFDSIILATGYRSNVPSWLKQGCDFFTKDGMPKTPFPNGWKGERGLYTVGFTRRGLLGTASDAMKIANDVAEQWRMAGNKDGKHCSTSYALL

>evm.TU.Scaffold_1082.314|Carabica_v0.5/1-398

MGSCKEEDGNQPNCLWVDGPIIVGAGPSGLAVSACLKENGVPSVILEGSDCIASLWQQKTYDRLKLHLPKQFCELPLLGFPENFPKYPTKHQFISYMQYYAIHFSIEPKFKQVVQSAEYDVVCGVWRVRTQDCEYVSRWIIVATGENAEPFIPDIQGIEKFQGHVRHTSVYKCGSEFQDQRVLVIGCGNSGMEVSLDLCRYNATPHIVVRNTVHILPREIFGFSTFAVAMALLKWLPLWLVDKFLLMVTNFTIGNTDTLGLKRPKTGPIQLKNATGKTPVLDVGALSQIKAGKIKVAEGVREITRKGAKFVDGQEREFDSIILATGYKSNVPAWLKGCDFFTKEGMPKTPFPNGWKGDNGLYTVGFTRRGLLGTASDAVKIAKDIADQWRLNKGCKKH

>evm.TU.Scaffold_2631.192|Carabica_v0.5/1-398

MGSCKEEDGNQPNCLWVDGPIIVGAGPSGLAVSACLKENGVPSVILERSDCIASLWQQKTYDRLKLHLPKQFCELPLLGFPENFPKYPTKHQFISYMQYYAIHFSIEPKFKQVVQSAEYDVVCGVWRVRTQDCEYVSRWIIVATGENAEPFIPDIQGIEKFQGHVRHTSVYKSGSEFQDQRVLVIGCGNSGMEVSLDLCRYNATPHIVVRNTVHILPREIFGISTFAVAMALLKWLPLWLVDKFLLMVANFSIGNTDTLGLKRPKTGPIQLKNATGKTPVLDVGALSQIKAGKIKVAEGVREITRRGAKFVDGQEREFDSIILATGYKSNVPAWLKGCDFFTKEGMPKTPFPNGWKGDNGLYTVGFTRRGLLGTASDAVKIAKDIAGQWTLMKGCKKH

>Hyque.11G050400|Hquercifolia_v1.1/1-406

MGSCKQEENQSNCLWVQGPIIVGAGPSGLAVSACLKDQNVPSLILERSDCIASLWQKRSYDRLKLHLPKQFCELPLFGFPENFPKYPSKHQFISYMESYASHFSINPRFNQVVQRAEFDHVSGLWRVQTQDSDYVSKWLIVATGENAEPVIPEISGIERFQGSVLHTSLYKSGSDFRNQRVLVVGSGNSGMEVSLDLSRHSSTPHLVVRNSVHILPREMFGFSTFGIAMALLKWLPLRLVDKFLLLMASLTLGNTDKLGLRRPKTGPIELKNATGKTPVLDVGALSQIKSGKIKVMEGVREITRNGAKFVDGQDREFDSIILATGYKSNVPFWLKGSDFFTEEGMPKTEFPNSWKGENGLYTVGFTRRGLLGAASDSAKIATDIADQWRTIKNFNNCSYSQNILKR

>Hyque.01G076800|Hquercifolia_v1.1/1-394

MWVKGPIIVGAGPSGLAVSACLKENGVPSLILERSDCIASLWRQRTYDRLKLHLPKQFCQLPLFGFPENFPKYPSKQQFISYMESYAAHFSIEPRFKQVVQRAEFDEIKGFWRIQTQDCDYGTRWLIVASGENAESLIPEIQGIDRFQGHVVHTSGYKCGSDFRNQSVLVVGCGNSGMEVSLDLCRYSATPHMVVRNSVHILPREMFGFSTFGVAMALQKWLPLSMVDKLLLIMANFTLGNTDRLGLRRPKTGPIELKNATGKTPVLDVGALSQIKSGKIKVMEGVREITRNGAKFVDGQEREFDSIILATGYKSNVPCWLKGSDFFTKEGLPKTPFPNGWKGENGLYTVGFTRRGLLGTVSDAYNIAKDIADHWRTIKDCNKTSNSHVVPERQ

>Rhdel02G0266400.1|Rhododendron_delavayi/1-407

MGFWTNQVGNQPKCLKIEGPIIVGAGPSGLAVSACLKENGVPSLILERSNCLASLWQQRSYNRLKLHLPKQFCELPLFGFPENFPKYPTKHQFISYVESYAEHFNIQPLFNQGVEKAEYDHSAGIWRVRTQDCEYVCRWLIVATGENAEPFTPEIAGIERFDGAVVHTSGYKSGSDFRNQRVLVVGCGNSGMEVSLDLCRYNASPHLVVRNTVHILPREMFGFSTFGIAMALLKWLPLRLVDKLLLLAANFTLGNTDSLGLRRPKTGPIELKNATGKTPVLDVGAISQIKSGKIKVMEGVREITRHGAKFIDGQEKEFDSIILATGYKSNVPCWLKGSDFFTEDGLPKTPFPNSWKGEKGLYTVGFTRRGLLGTASDSLNVARDIAEQWKTIKGSNIIWNSHIVLQV

>Prupe.8G020600|Ppersica_v2.1/1-189

MFGFSTFGIAMALLKWLPLKLVDKVLLLLATIILGNTDQLGLRRPKTGPIELKNATGKTPVLDVGALSQIKSGKIKVVVEGVKEITRNGARFMNGQEKEFDSIILATGYKSNVPTWLKGCDFFTKDGMPKTPFPDCWRGSEGLYIVGFTRRGLLGTTSDAVEIANDVAQLWKTNKDCRSSCNSHIILLK

>Prupe.1G468500|Ppersica_v2.1/1-409

MGSCKENQQENPQQPNCGFVQGPIIVGAGPSGLAAAACLSNHGVPSLILEKSDCIASLWQHKTYDRLKLHLPKQFCELPLMGFPEDFPRYPTKFQFISYMETYASHFSIKPKFNQAVQSAEFDSGFWRVKTQDFEYISKWLIVATGENAEPAIPEIGGMEKFQGPILHTSLYKSGSDFKNQRVLVVGCGNSGMEVSLDLCRHNANPHMVVRNTVHVLPREMFGFSTFGIAMALLKWLPLKLVDKVLLLLATIILGNTDQLGLRRPKTGPIELKNATGKTPVLDVGALSQIKSGKIKVVVEGVKEITRNGARFMNGQEKEFDSIILATGYKSNVPTWLKGCDFFTKDGMPKTPFPDCWRGSEGLYTVGFTRRGLLGTASDAVEIANDVAQLWKTNKDCRSSCNSHIILLK

>FvH4_2g29930|Fvesca_v4.0.a2/1-407

MGSCKDPKPNCEFVEGPIIVGAGPSGLAVAACLSNHGVPSLILEKSNCIASLWQHKTYDRLKLHLPKQFCQLPLMGFPEDFPKYPTKQQFINYMESYASHFSIKPSFNQAVQNAAFDSASGHWRVQTQDFEYISRWLIVATGENAEPVIPDIHGIDKFKGLIRHTSLYKTGKDFRNQRVLVIGCGNSGMEVSLDLCRNNATPHMVVRNTVHVLPREMFGFSTFQIAMTLLKWIPVKLVDRILLLVASLTLGNTDQLGLRRPKTGPIELKAATGKTPVLDVGALSLIKSGKIKVVENVKEITINGAKFMNGQEKEFDSIILATGYKSNVPTWLKGCDFFTKEGMPRAPCPSCWKGSDGLYTVGFSRKGLSGTAYDAVEIAKDIADHWKTSKDCKGSVASCDSHVILLK

>Potri.006G248200|Ptrichocarpa_v4.1/1-411

MDSCKEQEQQGFNQAKFVKVLGPIIVGAGPSGLAVAACLSQQGVPSLILEKNDCIASLWQQKTYDRLKLHLPKQFCELPLRGFPDDFPKYPTKGQFISYMESYASHFRIQPKFNQAVKTTEFDHGVWRVQTEDLEYHSRWLIVATGENAEPVIPDIVGYDKFKGNILHTSEYKSGSKFKNQRVLVVGCGNSGMEVSLDLCRHNAIPHMVVRNTVHVLPREMFGMSTFGIAMALLKWLPLRLVDKFLLLVANLILGNTEQLGLKRPKTGPIELKNVTGKTPVLDVGALSQIKSGKIKVMEGVKEVTKNGVKFMNGQEKKFESIILATGYKSNVPTWLKGCDFFTKDGMPKTPFPNGWKGENGLYTVGFTRRGLLGTASDAVKIAQDIGDQWKTIKGNDKSCNSHVIILGKPW

>Potri.018G033200|Ptrichocarpa_v4.1/1-416

MASGKEQEQQGFTQAKCVKVHGPIIVGAGPSGLAAAACLSQQGVPSLILEKSDCIASLWQQRTYDRLKLHLPKQFCELPLLGFPANFPKYPTKSQFISYMESYASHFNIHPKFNQAVKKAEYDRVKGIWRVQTEHLEYHSRWLIAATGENAEPVIPEIAGCGKFKGAILHTSEYNSGSKFKNQRVLVIGCGNSGMEVSLDLCRHNAIPHMVVRNTVHVLPREMFGMSTFGVAMALLKWLPLRLVDKLLLLGANLTLGNTDQLGLKRPKTGPIELKNVTGKTPVLDVGALSQIKSGKIKVMEGVKEITKNGVKFMDGQEREFESIILATGYKSNVPTWLKGCDFFTKDGMPKTPFPKGWKGGNGLYTVGFTRRGLLGTASDAVKIAHDIAGEWKAAKDSIRSCNSHVIIHLDHDGAK

>AUR62024856|Cquinoa_v1.0/1-396

MNCKSEEDETTKLATKMVEFHGPIIVGAGPSGLAVAACLRKQGIPSLILERSDCIASLWQQKTYDRLKLHLPRKFCELPLMGFPKDYPKYPSKKQFISYMEAYAAFFKISPVFNQEVESAGFDTDTRVWRLKIKDIEYISKWVIIATGENAEPLIPEIQGIEKFQGKFLHTSLYKSGIEFKNQRVLVIGCGNSGMEVSMDLCRYNAMPFMVVRNSVHVLPREMFGFSTFGVAMALLKWLPLRLVDKFLLLVANLTLGNTDRLGLRRPKTGPIELKNATGKTPVLDVGALSLIKSGNIEVMEGVKEITKNGAKFIDGQEREFDSIILATGYKSNVPFWLKDCEFFSDDGMPKVAFPNGWKGGKGLYTVGFTRRGLLGTASDAVKVAQDIAHQWNQRS

>AUR62032364|Cquinoa_v1.0/1-380

MVEFHDPIIVGAGPSGLAVAACLRKQGIPSLILERSDCIAPLWQQKTYDRLKLHLPRKFCELPLMGFPKDYPKYPSKKQFISYMESYAAFFNISPVFNQEVESAEFDTETRVWRLKIKEIEYISKWVIIATGENAEPLIPEIQGIEKFQGKFLHTSLYKSGIEFKNQRVLVIGCGNSGMEVSKDLCRYNAMPFMVVRNSVHVLPREMFGFSTFGVAMALLKWLPLRLVDKFLLLVANLTLGNTDRLGLRRPKTGPIELKNATGKTPVLDVGALSLIKSGNIKVMEGVKEITKNGAKFIDGHEREFDSIILATGYKSNVPFWLKDCEFFSDDGMPKVPFPNGWKGGKGLYTVGFTRRGLLGTASDAVKVAQDIADQWNQRS

>Sapof.03G127300|Sofficinalis_v1.1/1-414

MSCCKEEKEGKKSQKLIYFEGPIIVGAGPSGLATSACLKKQGISSLIIERSNCIASLWQQRTYDRLKLHLPNKFCQLPLMGFPKNFPKYPSKQQFIAYMENYASHFGINPLFNKEVKKAEFDEVLGGWRVTTVGICPMSITVNSEVEYFSKWLIVATGENAEPLIPEIQGIEKFSGKLVHTSLFKSGVEFCNQKVLVVGCGNSGMEVCLDLCAHLAFPHMVVRNSVHVLPREMFGFSTFGVAMALSKWFSLRLVDKFLLLVANLTLGDADRLGLRRPKTGPMELKNVAGKTPVLDMGAFSLIKSGKIKVMEGVREITKNGAKFVDGQEKDFDSIILATGYKSNVPFWLKDCEFFSIDGMPKIPFPNGWKGGKGLYTVGFTKRGLLGTSSDAVNVARDIANEWRRTMQNTNNDQK

>orange1.1g037065m.g|Csinensis_v1.1/1-412

MGSCKVQNDKQTKSVLVHGPIIVGAGPSGLAVSACLSQQGLPSLILERSDCLASLWKHRTYDRLKLHLPKQFCELPLFGFPENFPKYPTKRQFIAYIESYASHFKIQPKFKQAVQTALFDHASGFWRVQTQDSEYISKWLVVATGENAEPVFPDVVGLDKFNGHVLHTSKYKSGSEFKNQKVLVIGCGNSGMEVSLDLCRHNAIPHMVARNSVHVLPREIFGFSTFGIAMALLRWFPLRLVDKILLLMANITLGNTDQLGLRRPKTGPIELKNITGKTPVLDVGALSQIKSGKIKVVGGVKEITKNGARFTDGQEKEIDAIILATGYKSNVPTWLKECDFFTKDGMPKTPFPNGWKGENGLYTVGFTRRGLQGTALDADKIAQDISEQWRKIKDLNNNNNNNYTSNSPSYPT

>Soltu.DM.06G019760|Stuberosum_v6.1/1-410

MGSCKEEEPKWLWVNGPIIVGAGPSGLAVSACLKENGVPSLILERSDCIASLWQQKTYDRLKLHLPKQFCQLPLFDFPENFPKYPTKHQFISYLESYAKHFSITPKFKQSVKNAEFDEVCGFWKVQTQDFQYLSKWLIVATGENAEAVIPEISGIDKFKGRLMHTSVYKSGTEFINQRVLVIGCGNSGMEVSLDLCRHNAIPHMVVRNSVHILPREMLGISTFSIAMALLKWMPLRIVDKFLLLVANLTLGSTDKLGLRRPKTGPIELKNATGKTPVLDVGALSQIKTGKIQIMPGVKEITKIGARFLDGKEGEFDSIILATGYKSNVPSWFKGSDFFTEQGMPKTPFPNGWKGENGLYTVGFTRRGILGTANDAKNIARDISEQWREFKGFCKNFCTTRNLSDSQGICF

>Solyc06G001591|Slycopersicum_ITAG5.0/1-410

MGCCKEEEPKWLWVNGPIIVGAGPSGLAVSACLKENGVPSLILERSDCIASLWQHKTYDRLKLHLPKQFCQLPLFDFPENFPKYPTKHQFISYLESYAKHFSITPKFKQSVKVAEFDGVCGFWKVQTQDFQYLSKWLIVATGENAEAVIPEIPGIDKFKGRVMHTSVYKSGTEFINQRVLVIGCGNSGMEVSLDLCRHNAIPHMVVRNSVHILPREMLGISTFSIAMALLKWMPLRIVDKLLLLVANLTLGSTDKLGLRRPKTGPLELKNATGKTPVLDVGALSQIKTGKIQIMPGVKEITKIGAKFLDGKEGEFDSIILATGYKSNVPSWFKGSDFFTEQGMPKTPFPNGWKGENGLYTVGFTRRGILGTANDAKNIARDISEQWREFKGFCKNFCTTKNLSDNQGICF

>DCAR_103210|Dcarota_subsp_sativus_v3.0/1-405

MGSCKEEERKQQKSVWVHGPIIVGAGPSGLAVAACLKQSQIPSLVLERSDCIASLWQQRTYDRLKLHLPKQFCQLPLLDYPKHFPKYPTKHQFISYMESYAKHFCIEPKFKQLVEKAEFVNGIWRVKSQDCEYISKWLVVATGENAEPVIPQIQGLDRFNGNVVHTSGYKSGYEFRKQKVLVVGCGNSGMEVSLDLCRHNASPHMVVRNSVHVLPREIFGCSTFGIAMALHKWLPLRLVDKFILLMSNLILGSTENLGLRRPETGPIELKNITGKTPVLDGGALNQIKSGNIKVMEEVKEITRNGAKFMDGQEKEFDSIVLATGYKSNVPFWLKGSDFFTEQGMPRRPFPNGWKGENGLYTVGFTRRGLLGTASDAIKIANDIADQWKINKEWDNICSSYVILQI

>Aqcoe7G200400|Acoerulea_v3.1/1-409

MGSCKEDEKQMIKCAWVHGPIIVGAGPSGLAVAACLADNGVPSVVLEKNNCIASLWQYKTYDRLNLHLPKQFCQLPLMGFPENFPKYPSKHQFITYMESYAKHFSIEPRFGQAVETAEFDVECELWRVRTKDTDYMSRWLIVATGENAEPVIPEISGMDKFHGPVIHTSVYKSGADFKNQNVLVVGCGNSGMEVSLDLCSHNASPHMVVRNAVHVLPREMLGLSTFGIGMALLKWIPLRLVDKILLLMAHFTLGNTDRLGLRRPKTGPLELKNVTGKTPVLDVGALSQIQSGNIKVMEGVKEITRRGAKFVNGQEKEFDSIILATGYKSNVPCWLKTCDFFTAEGMPKTPFPNGWKGDKGLYCVGFTRRGILGTASDAGKIAKDISDQWTIKDPMNCCNAHIILRREIQ

>BUSI_024597-RA|Buxus_sinica/1-408

MGSRREEQTELKCVWVHGPIVVGAGPSGLAVAACLVENGVPVVVLEKSNFIASLWQCRTYDRLKLHLPKHFCELPLMGFPENFPEYPSKQQFISYMESYAEKFSIKPRFQQSVNSAEFDSSCGFWRVRTQNMEYISKWLIVATGENAEPVIPEITGLGSFHGPVIHTSAYKSGADYKNQKVLIVGCGNSGMEVSLDLCRHNANPHMVVRNTVHVLPREMFGVSTFGIAMALLKWLPLRLVDRLLLLVTSFILGNTDRLGLRRPKTGPIELKNATGKTPVLDVGALSLIKSGKIKVMEGVRKITRRGAKFMDGQEKEFDSIILATGYKSNVPSWLKSCDFFTENGMPKTPFPNGWKGGDGLYSVGFTGRGLLGTASDAVKIASDVADQWRAMKNTNNVCNAHNIILRLK

>GWHPBAUK022099.1|Macadamia_integrifolia/1-415

MGTCREEETPLKCAWVHGPIIVGAGPSGLATAACLAMKDVPSLLLEKSNCIASLWQYKTYDRLTLHLPREFCELPLMGFPEDFPKYPSKQQFVSYMESYASKFKIKPRFGQSVESAEFNRSCGFWRVRTQDKEYLSRWVIVATGENAEPVVPEIPGMDKFNGPVIHTSVYKSGSDFRNQKVLVVGCGNSGMEVCLDLCSHNAAPHMVVRNTVHVLPREMFGLSTFGVAMALIKWFPLRLVDQFLLLVANFTMGNTERLGLRRPKTGPIELKNVTGKTPVLDVGTLSQIKTGKIKVMEGVREITRSGAKFVDGQEKGFDSIILATGYKSNVPSWLKSDFFTEQGMPRTPFPDGWKGEKGLYSVGFTRRGLLGTASDANKIAKDIADQYTAFKEAKITTCNTHIVLHKKSLKKTKKI

>Pso03G21920.0|Papaver_somniferum/1-410

MGNQEDEAVGVMKCSWVHGPIIVGGGPSGLAVAACLSDKGIPSLVLEKSDRIASLWQYRSYDRLNLHLPKQFCELPLMSFPKHFPKYPTKHQFISYLESYASNFGIKPKFNQSVVSAEFDSGSGVWRVRTQDTEYISKWLISATGENAEPVIPEISGVEKFHGAVIHTSVYKSGSDFKNQKVLVVGCGNSGMEVSLDLCSHNASPHMVVRNTVHVLPREMCGISTFGIAMGLMKWLPLRIVDKVLLLAAYFSLGNTEQLGLHRPKIGPIELKNATGKTPVLDVGALSRIKSGHIKVMAGVREITRKGAKFIDGQEKEFDSIILATGYKSNVPSWLKSCDLFTEEGMPRTPFPNGWKLGANGLYTVGFTRRGLLGTASDAVKIASDIGEQWRTMKDPRNSCNSQIILRRAC

>Pso05G25630.0|Papaver_somniferum/1-409

MGKEDEAVGVMKCSWVHGPIIVGGGPSGLAVAACLSDKGIPSLVLEKSDRIASLWQYRSYDRLNLHLPKQFCELPLMSFPKHFPKYPTKHQFISYLESYASNFEIKPKFNQSVLSAEFDSGSGVWRVRTTDTEYISKWLISATGENAEPVIPEISGVEKFHGAVIHTSVYKSGSDFKNQKVLVVGCGNSGMEVSLDLCSHNASPHMVVRNTVHVLPREMCGISTFGIAMSLMKWLPLRIVDKVLLLAAYFSLGNTEQLGLHRPKIGPIELKNATGKTPVLDVGALSRIKSGHIKVMAGVREITRKGAKFIDGQEKEFDSIILATGYKSNVPSWLKNCDLFTEEGMPRTPFPNGWKLGANGLYTVGFTRRGLLGTASDAVKIASDIGEKWRTMKDPRNSCNSQIILRRAC

>Litul.01G149400|Ltulipifera_YP108A_v1.1/1-412

MGSFSESENLLKRVLVNGPVIVGAGPSGLAVAACLDKNGVPSVVLEKTNCIASLWQQRTYDRLNLHLPKQFCELPLMGFPKDFPKYPSKRQFISYMESYASEFSIKPKFEQSVQSAEFDSACGWWRIRTQDVDYMSRWLIVATGENAQPVIPEITGIEKFHGPVLHTSVYKSGADFSQQKVLVVGCGNSGMEVSLDLCGHNASPHMVVRNSVHILPRDMFGISTFGIAMALLKWFPLRVVDQFLLLVAHFTLGNTDGFGLRRPKTGPIELKNTTGKTPVLDVGALAKIKSGKIKVMEGVKEITRRGAKFVDGQEKEFDSIILATGYKSNVPSWLKGTDFFTQQGMPKTPYPNGWKGENGLYSVGFTMRGILGASKDAMKIAEDIAQQWKTSKDTHNLNNAHFVLCTNSHRRS

>GWHPBFSI001488|Chloranthus_spicatus/1-413

MCSCKGRTMVKCSWVNGPIIVGAGPSGLAVAACLADQGVPSVVLEKSQCIASLWQHRTYDRLNLHLPKQFCELPLMGFPIGFPKYPSKHQFISYLEDYASKFCIEPRFGQSVMSAEFDSVFGVWRVRTEGVEYMSKWLIVATGENGEPVIPHITGIESFCGPVLHTSVYKSGSDFKQQKVLVVGCGNSGMEVSLDLCSHNATPYMVIRNTVHVLPREMFGISTFGIAMALLKWFPLKHVDRFLLLVAYLTLGNTDKLGLRRPCAGPIELKNSTGKTPVLDVGALSHIKSGKIKVMEGVQEITRKGAKFVDGQEKEFDSIVLATGYKSNVPSWLKGCDLFTNEGMPKTPFPNGWKGDNGLYSVGFTRRGLLGASSDATKVAKDIAQQWRTTKDPSNSTITHFVLSKTSLHCRTT

>CKAN_00594900|Ckanehirae_v3/1-450

MGSCKEDVSELKCELVHGPIVVGAGPSGLAAAACLTANGVPSLVIEKSDCIASLWQNRTYDRLKLHLPKQFCELPLMGFPPNFPKYPSKHQFVSYMESYAANFSIEPRFGQEVQSAEFDSACGFWRVRTEDSMYMTRWLIVATGENADPVIPAISGIDRFEGPVIHTSSYKSGAAFRHKKVLVMGCGNSGMEVSLDLCSHNATPYMVVRNSVHVLPREMFGISTFGMAMALLKYLPLRLVDKFLLLVARFTLGNTDRLGLRRPKTGPIELKNSTGKTPVLDVGALAQIKSGNIKVMGGVKEITKRGAKFIDGQEKEFDLIIFATGYRSNVPSWLKGSDFFTPQGMPKVPFPSGWKGENGLYSVGFTMRGLMGASLDAMKIAGDIAQQWTTEQWTTEDTKNISNTQFVLSKQSSQKSMKNTLFVLCHRKLTQSNTIILSPLLQRRSCAVCK

>Qrob_P0436830.2|Quercus_robur/1-414

MGSCKEKEESTPMCAWVQGPIIIGAGPSGLAVSACLSEHGIPFVILERSNCIASLWQQRTYDRLKLHLPKRFCALPLLGFPLNFPKYPTKEQFISYMEDYATHFSIKPKFNQNVQSAEFDSSCGYWRVKTLDMEYNSRWLIVATGENAEPIIPEIVGIERFHGPVVHTSEYRSGSYFKNRKVLVIGCGNSGMEISLDLCRHKAVPHLVVRNTVHVLPREMFGISSFGLAMALLKLFPLSVVDKFLLLMSYFTLGNTEKLGLHRPKTGPMEHKNVTGKTPVLDVGALSKIKSGKIKVMVGVKEITGNGAKFMDGQEKEFDSIILATGYKSNVPSWLKGCDFFTKDGMPRTPFPNGWKGENGLYTVGFTRRGLLGTASDANKIAKDIADQWRPSEDWKSISNSQVILLKNCNKNKF

>gene_prefix14565.t1_1|Ceratophyllum_demersum/1-411

MGSFKEHEEQKIKCVWVHGPIIVGGGPSGLAVAACLSKEGVPSLILEKSNCIASLWQHSTYDRLNLHLPKQFCELPLMGFPKDFPKYPTKYQFIYYIESYAKNFSLEPKLDQNVQNTEYDSASGFWRVGTQDAEYMSWWLVVATGENAAPIIPEISGIEKFQGSVIHSSDYKTGSDYENQNVLVIGCGNSGMEISLDLCSHNATPYMVVRNTVHVLPREILGISTFGIAVFLLKWLPARTVDKFLLLAARVMLGNTGNFGLKRPKTGPLELKNATGKTPVLDVGALAKIRTGKIKVTEGVKEITGRGAKFVDGQEEEFDSIILATGYKSNVPSWLKGCDFFTKEGMPRKPFPNGWKGKNGLYTVGFTRSGILGSSSDAMKIALDISVQWNAIKDHNNSCNTHLVLSKSLPS

>gene_prefix16546.t1_1|Ceratophyllum_demersum/1-411

MDSFKEHEEQKIKCVWVHGPIIVGGGPSGLAVAACLSKHGVPSLILEKSNCIASLWQHRTYDRLNLHLPKQFCELPLMGFPKDFPKYPTKYQFICYIESYAKNFSLEPKLDQNVQNTEYDSASGFWRIRTQDAEYMSWWLVVATGENAAPIIPEISGIEKFQGSVIHSSDYKTGSDYENQNVLVIGCGNSGMEISLDLCSHNATPYMVVRNTVHVLPREILGISTFGIAVFLLKWLPVRTVDKFLLLAAHVMLGNTGNFGLKRPKTGPLELKNITGKTPVLDVGALAKIRAGKIKVTEGVKEITRRGAKFADGQEEEFDSIILATGYKSNAPSWLKGCDFFTKGGMPRTPFPYGWKGENGLYTVGFTRRGILGSSSDAMKIALDISLQWNAIKDHNNSCNTHLVLSKSLPS

>GWHPCBIR018864|Santalum_yasi/1-407

MGRSNCNQEGDEETEHVWVEGPIIVGAGPSGLAAAACLLEKGVPSLILEKCGCIASLWQERTYDRLKLHLPRNFCELPLMGFPSNFPKYPTKHQFVSYMKSYASHFKIEPEFNQAVEKAEFDPRHGGFWRVQTRDRVYLSRWLIAGTGENAEPRVPEIPGIVGFQGRVLHTSGYKSGLEFQNQRVLVVGCGNSGMEVSLDLCNHNAEPHMIVRNTVHVLPREMFGFSTFGVAMALLKWLPLRVVDKFLLVVANFTLGRTDGLGLRRPKTGPLELKNATGKTPVLDVGAVSQIKAGKIKLMKGVKEITRRGARFVDGQESEFDAIIFATGYKSNVPSWLKGSDFFTEEGMPKAPFPKNWKGENGLYTVGFSRRGLLGTASDALNIARDIGDQWRTIKGSQSQCDSHFF

>Glyma.04G070100|Gmax_Wm82.a6.v1/1-405

MESLKGEEVKCVWVQGPIIVGAGPSGLAVAACLSHHGVPYVILERSHCITSLWQHRTYDRLKLHLPKHFCELPLMPFPLHFPKYPSKNQFISYLNSYASRFNIRPRFNQSVQTAEFDPSSQLWLVRTNGFQYISPWLVVATGENAEPVVPSISGMDMFHGPIVHTSVYKSGSDYNNQRVLVIGCGNSGMEVSLDLCRHNANPYMVARNTVHVLPREMFGFSTFGIAMALLKWLPIKVVDKLVLAAARLMLGDTARYGVRRPKTGPIELKLVTGKTPVLDVGQVAQIRSGNIKVMEGVKEITRNGAKFMDGQEKEFSAIILATGYKSNVPTWLKSCESFTKDGMPKTPFPMGWKGENGLYTVGFTRRGLLGTASDAVKIAKDIADQWMTVKDKSYRNSHIILLKST

>Glyma.14G128200|Gmax_Wm82.a6.v1/1-404

MGRSCNKSQQQHVEGPIIIGAGPSGLAVAACLSEDKVPFVILERHNCIASLWQNKTYDRLKLHLPKQFCELPLKGFPHTFPKYPTKYQFISYMESYASHFNIHPIFNQTVKSAEFDKGSNVWVVRTEEFEYSSRWLVVATGENAEPVVPRIHGMELFGGAVAHTSVYKSGSEYRNKKVLVIGCGNSGMEVCLDLCRHNAKPYMVARNTVHVLPREMLGFSTFGIAMALYKWFPIKLVDKIILLATNLILGNTNHYGIKRPKTGPIELKLATGKTPVLDVGQVAQIKCGNIKVMEGVKEITRNGAKFMDGKEKEFDAIILATGYKSNVPTWLKGCDFFTKDGMPKTPFPHGWKGEQGMYTVGFTRRGLHGTSCDAIKIAEDIAEQWRTVEDKSHCDSHIILLNNS

>Glyma.17G205800|Gmax_Wm82.a6.v1/1-406

MGSCNKPQQQQQQHVQGPIIIGAGPSGLAVAACLSEHKVPFVILERHNCIASLWQNKTYDRLKLHLPKQFCELPLKGFPHTFPKYPTKYQFISYMESYASHFNIHPIFNQTVESADFDKGSKVWVVKTQEVDYSSRWLVVATGENAEPVVPRIHGMELFNGDVAHTSVYKSGSEYRNKKVLVIGCGNSGMEVCLDLCRHNAKPYMVARNTVHVLPREMFGFSTFGVAMALYKWFPIKLVDKIILLATNFILGNTNHYGIKRPKTGPIELKLATGKTPVLDVGQVAQIKCGNIKVMEGVKEITRNGAKFMDGQEKEFDAIILATGYKSNVPTWLKGCDFFTEDGMPKTPFPHGWKGEQGLYTVGFTRRGLQGTSCDAIKIAEDIAEQWRTVEDKSHCDSHIILLNNS

>KAI5393867.1|Pisum_sativum/1-411

MDPFKQKPKSLFIHGPIIVGAGPSGIAVAACLSEQGVPSLILERSDCIASLWQNRTYDRLKLHLPKHFCELPLMSFPQDFPMYPTKHQFISYMESYADQFGIRPRFNQTVVTAEFDPSSEIWNVKTLDGFQYSSPWLVVATGENAEPVIPKIHGMEHFHGPVVHTCDYKSGSQYKNKKVLVIGCGNSGMEVSLDLCRHNALPHLVARNTVHMLPRDIFGFSTFGVAMTLNKWLPLKLVDKFLLLVSSFFLGNTNHYGIKRPKTGPIELKLATGKTPVLDVGQIAQIKSGNIKVMEGVKEITRSGAKFMDGQEKEFEAIILATGYKSNVPSWLKGSDFFTKDGMPKTPFPHGWKGEQGLYTVGFTRRGLHGTYFDAIKISEDITSQWKTIKSKSCSDSHIINLISTTTLNNV

>Kalax.0552s0003|Klaxiflora_v1.1/1-409

MGTAAECVHVHGPIIVGAGPSGLAAAACLFQKGVPSVVLERAGCIASLWQNRTYDRLKLHLPKHLCKLPLMNFPPHFPKYPAKQQFISYMETYASRFNIHPQFNQPVTEAAFDRFAGLWRVTTESSVFISRWLIVASGENAEPLIPQIVGMERFHGPVNHTSVYKSGLDFEKRKVLVIGCGNSGMEVSLDLCRHNATPYMVARNNVHVLPREMFGFSTFAIAICLLKWLPLRLVDRLLLFIASCKLGQTDKYGLRRPKTGPIELKNATGKTPVLDVGALAQIKAGKIKVMEGVKEITRNGAKFVNGVEEGFDAIILATGYKSNVPTWLKGCDFFTEDGMPRTPFPNGWKGQNGLYTVGFTRRGLLGTASDAFKIAADIAEDWGTMKDKTNNCCNAQTVPRKIPLHHQLE

>Kalax.0311s0021|Klaxiflora_v1.1/1-409

MGTAAECVHVHGPIIVGAGPSGLAAAACLFQKGVPSVVLERAGCIASLWQNRTYDRLKLHLPKHLCELPLMNFPPHFPKYPAKQQFISYMETYASRFNIHPLFNQPVTEAAFDRFAGLWRVTTESSVFISRWLIVASGENAEPLIPEIVGMERFHGPVNHTSVYKSGLDFEKRKVLVIGCGNSGMEVSLDLCRHNATPYMVARNNVHVLPREMFGFSTFAIAICLLKWLPLRLVDRLLLFIASCKLGQTDMYGLRRPKSGPIELKNATGKTPVLDVGALAQIKAGKIKVMEGVKEITRNGAKFVNGVEEEFDAIILATGYKSNVPTWLKGCDFFTEDGMPRTPFPNGWKGQNGLYTVGFTRRGLLGTASDAFKIAADIAEDWGTMKDKTNNCCNAQTVPRKIPLHHQLE

>Kalax.0067s0126|Klaxiflora_v1.1/1-417

METSRKCVYAHGPIIVGAGPSGLAAAACLSQQGVPSIVLERADCVASLWQNRTYDRLKLHLPKHLCELPLMKFPAHFPKYPSKQQFVSYMEAYASHFGISPKFNQAVAEAAFDHSSGFWKVTTKDCVYISKWLIVASGENAEPLIPEIPGMEKFHGPVKHTSVYKSGLDFEKRKVLVIGCGNSGMEVSLDLCRHNATPYLVARNNVHVLPREMFGFSTFGIAMCLLKWLPLRLVDRLLLFTANCTLGKTDKYGLRRPKTGPIELKNATGKTPVLDVGALAQIKSGKIKVMEGVKEITRNGAKFVNGAEEEFDAIILATGYKSNVPTWLKGCDFFTEDGMPRTPFPNGWKGQNGLYTVGFTRRGLLGTASDAFKIASDIGEDWRTMKDKNDCCNAQIVLRRRTSPSPSPSTVVVAKQT

>Kalax.0046s0049|Klaxiflora_v1.1/1-425

METSRKCVYAHGPIIVGAGPSGLAAAACLSQQGVPSIVLERADCVASLWQNRTYDRLKLHLPKHLCELPLMKFPAHFPKYPTKQQFVSYMEAYASHFGISPEFNQAVAEAAFDHSSGFWKVTTKDCVYISKWLIVASGENAEPLIPEIPGMEKFHGPVKHTSVYKSGLDFEKRKVLVIGCGNSGMEVSLDLCRHNATPYLVARNNVHVLPREMFGFSTFGIAMCLLKWLPLRSVDRLLLFTANCTLGKTDKYGLRRPKTGPIELKNATGKTPVLDVGALAQIKSGKIKVMEGVKEITRNGAKFVNGAEEEFDAIILATGYKSNVPTWLKGCDFFTEDGMPRTPFPNGWKGQNGLYTVGFTRRGLLGTASDAFKIASDIGEDWRTMKDKNDCCNAQIVLRRRTSPSPSSSPSPSPSPSTVVVAKQT

>DCAR_104882|Dcarota_subsp_sativus_v3.0/1-407

MRGFKEDIKDETKGVWVDGAIIVGAGPSGLAVSACLKKNNITSLVLERNDCLASLWQKRTYDFLKLHLPQNFCELPFFGFPRNFPKYPTKHHFISYIEAYAQHFGIQPRFKQVVERAEFDSLSGFWRVFSQDCEYVSRWLVVATGENAEPFIPEIGGIERFDGPVVHTSEYRSGGEFKKQRVLVVGCGNSGMEVSLDLCKHNAIPHLVVRNSVHVLPREMFGCSTFGIAMALLKWFPLRLVDKFLILMCNSILGKTEKLGLRRPKTGPIELKNATGKTPILDGGALSHIKSGEIKVMVGVKEITSNGAKFMDGQEREFDAIVLATGYKSNVPFWLKGNELITEDGLQKASNGNGWKGKNGLYTVGFSKKGLLGTASDALNIARDIADHLKSCKDCNNSCRSIAVLQT

>Eucgr.C01808|Egrandis_v2.0/1-420

MGSVSCGDQELEDNQRTDQPLFVEVEGPIIVGAGPSGLAVSACLSEHGVPSLILEKSDCIASLWQKWTYDRLKLHLPKRFCELPLLRFPKNFPKYPTKQQFVSYLESYASHFKIEPIFNQTVQSAEFDESCGLWRVRTREGLERRSRWLVVATGENAEPATPDVDGLERFQGRVVHTSRYKSGLEFEGERVLVVGCGNSGMEVSLDLCRSNAIPHMVVRSTVHVLPREMLGLSTFGVAMALLKWLPLRLVDKILLLIANITLGNTDRLGLRRPKTGPLELKNVTGKTPVLDVGALAQIKSGKIKVVKEVKKITKNGAKFLDGQEEKFDSIILATGYKSNVSSWLKGGDFFTKEGMPKTPFPNGWKGDDGLYTVGFTRRGILGTGCDAIKIAGDIADQWRSSVKESNQDLHNSHIIHLQKS

>Brara.B00402|BrapaFPsc_v1.3/1-411

MNSCLEAEHSPIFVNGPIIVGAGPSGLSVAACLSNRGVPSVILERTDCLASLWQKRTYDRLKLHLPKHFCELPLMKFPKNFPRYPSKQQFISYIESYAARFNIKPVFNQTVEKAGFDVVSCLWKVKTQDAVYTSKWLVVATGENAEPVVPDIPGLKNFTGPVVHTSAYKSGSEFANRKVLVVGCGNSGMEVCLDLCRYNALPHMVVRNPLHVLPRDFFGLSSFGIAMTLLKWFPLKLVDNFLLLLANSYLGNTDRLGLRRPKTGPIELKNATGKTPVLDVGAISLIQSGQIRVRHAVKELTKKGAKFVDGQEMEFESIILATGYKSNVPDWLKENSFFTKEGMPKTPFPNGWKGENGLYTVGFTKRGLLGTAFDAVKIAENITDQWMKSNGPLSASNICSSCIIHFHFNKS

>Brara.J02239|BrapaFPsc_v1.3/1-411

MSSCLEEKHSRIFVTGPIIVGAGPSGLSVAACLSNRGVPSVILERTDCLASLWQKRTYDRLKLHLPKHFCELPLMKFPRNFPRYPSKQQFISYVESYAARFNIKPVFNQTVEKAEFDVAPGLWTVKTQDAVYSSTWLVVATGENAEPVIPDIPGLKKFTGPVVHTSAYKSGSEFANRKVLVVGCGNSGMEVCLDLCRYNSLPHMVVRNSVHVLPRDFFGLSSFGIAMTLLKWFPLKLVDNLLLLLAYLSLGNTDRLGLRRPKTGPIELKNVTGKTPVLDVGAISLIQSGQIRVMQAVKEITKKGAKFVNGQEMEFDSIILATGYKSNVPYWLKDNSFFTKEGMPKTPFPNGWKGENGLYTVGFTRRGLLGTAFDAVKIAEDITDHWMKMNGPLSSSNVCSSHIIHFDFNKS

>AT5G11320|Athaliana_Araport11/1-411

MGTCRESEPTQIFVPGPIIVGAGPSGLAVAACLSNRGVPSVILERTDCLASLWQKRTYDRLKLHLPKHFCELPLMPFPKNFPKYPSKQLFISYVESYAARFNIKPVFNQTVEKAEFDDASGLWNVKTQDGVYTSTWLVVATGENAEPVFPNIPGLKKFTGPVVHTSAYKSGSAFANRKVLVVGCGNSGMEVSLDLCRYNALPHMVVRNSVHVLPRDFFGLSTFGIAMTLLKWFPLKLVDKFLLLLANSTLGNTDLLGLRRPKTGPIELKNVTGKTPVLDVGAISLIRSGQIKVTQAVKEITRNGAKFLNGKEIEFDSIILATGYKSNVPDWLKENSFFTKEGMPKTPFPNGWKGEKGLYTVGFTRRGLSGTAYDAVKIAEDITDQWMKFNGPLSCRNICSSHIIHLHFNKS

>Eucgr.C02837|Egrandis_v2.0/1-413

MRSCKDREQDPQTLLEDVHKPIIVGAGPSGLAASACLSKLGVPSLILEKSDCIASLWQYRTYDRLKLHLPKLYCELPLWGFPEHFPKYPTKHQFILYMEAYASHFSIRPKFNQNVAKAEFDPIHGFWRVQTQEFQYISRWLIVATGENAESFVPDIVGLDKFKGSKLHSSDYKSGSDFRNQRVLVVGGGNSGMELSLDLCRHNATPCMVVRNTVHVLPREIFGFSTFGIAMSLLKWLPLRVVDKLLLLMANLLLGNTNHLGLRRPKTGPIELKNITGKTPVLDVGALSHIKSGNIKVMASVKEITKTGAKFVDGQEKEFDSIILATGYRSSVPSWLKSEFFTKKGIPKTPFPDGWKGGNGLYTVGFTKRGLLGTASEAIKIAKDIADQWRAINDLSEISVIPISSHLEKSQAT

>Brara.A00540|BrapaFPsc_v1.3/1-418

MDSPPLPRHNQTDHQTKQIILVHGPIIIGAGPSGLATSACLSNRGVPSLILERSDSIASLWKTKTYDRLKLHLPKHFCRLPLLDFPENFPKYPSKNEFLDYLESYASHFGIAPRFNENVVNAAFDTSSGLWRVKTLNKTEYLSKWLIVATGENADAYVPETPGIVKFSGGKIIHASDYRSGEEFRQQRVLVVGCGNSGMEISLDLVRHNASPHLVVRNTVHVLPREILGLSTFGVGMTLLKCLPLRLVDKFLLLMANLSFGNTDRLGIHRPKTGPLELKNVTGKSPVLDVGAMSLIRSGKIKIMEGVKEITKNGAKFMDGQEKEFDSIIFATGYKSNVPTWLQGSDFFTKEGMPKTPFPNGWRGGKGLYTVGLTRRGLLGTASDAVNIAGEIADQWRDEIKGPIKNMCSSRFVLISKS

>Brara.H01304|BrapaFPsc_v1.3/1-410

MDSHSCHQTKQIILVHGPIIIGAGPSGLATSACLSSRGVPSLILERSDSIASLWKSKTYDRLKLHLPKHYCRLPLLDFPENFPKYPSKNEFLDYLESYASHFGIVPRFNENVQNASYDSSSGLWRVKTLSGAEYLSKWLVVATGENADAYVPEVPGILKFSGGRIIHASEYKSGEEFRQQKVLVVGCGNSGMEISLDLVRHNASPHLVVRNTVHVLPREILGLSTFGIGMTLLKCLPLRFVDKFLLLMANLSFGNTDRLGLRRPKTGPLELKNLTGKTPVLDVGAMTLIRSGKIQIMEGVKEITKKGAKFMDGQEKEFDSIIFATGYKSNVPTWLQGSDFFTKEGMPKTPFPNSWRGGKGLYTVGFTRRGLLGTASDAVKIAGEIADQWRDIKGATKNLCSSRFVIISKS

>AT4G32540|Athaliana_Araport11/1-414

MESHPHNKTDQTQHIILVHGPIIIGAGPSGLATSACLSSRGVPSLILERSDSIASLWKSKTYDRLRLHLPKHFCRLPLLDFPEYYPKYPSKNEFLAYLESYASHFRIAPRFNKNVQNAAYDSSSGFWRVKTHDNTEYLSKWLIVATGENADPYFPEIPGRKKFSGGKIVHASEYKSGEEFRRQKVLVVGCGNSGMEISLDLVRHNASPHLVVRNTVHVLPREILGVSTFGVGMTLLKCLPLRLVDKFLLLMANLSFGNTDRLGLRRPKTGPLELKNVTGKSPVLDVGAMSLIRSGMIQIMEGVKEITKKGAKFMDGQEKDFDSIIFATGYKSNVPTWLQGGDFFTDDGMPKTPFPNGWRGGKGLYTVGFTRRGLLGTASDAVKIAGEIGDQWRDEIKGSTRNMCSSRFVFTSKS

>Pn14.797|Piper_nigrum/1-410

MGLCREGEEIRREWSPGPIIVGSGPSGLAVATLLAKSGVPSLVLERAHCIASLWQNRTYHRLKLHLPKQFCELPLMGFPSDFPKYPSKQQFVSYMESYAAHFDIRPRFGNPVLNAQFHPTAAGDGVWRVRTPVAEYVSRWLVVATGENAEPVVPEIPGLERFHGPVLHTSVYMTGADFKNQRVLVVGCGNSGMEVSLDLSTHNATPHMVVRNTVHILPREMLGFSTFGIAMALLKWLPLKLVDKLLLLVAEAKLGSTDRLGLRRPSTGPIELKNATGKTPVLDVGALSAIKAGKIKVMKEVKEITRRGAKFVDGLEIEFDSIILATGYKSNVPSWLKGSDFFTEEGMPQTPFPNGWGGPSGLYAVGFTRRGLLGACSDAVNIARDISKRWWTVDDVDNLQNSEPVLSPSQ

>Pn17.33|Piper_nigrum/1-396

MDSCQQWSPGPIIVGAGPSGLAVAALLAEEGIPSLLFERSHCIASLWQNKTYNRLKLHLPKQFCELPLMPFPSHFPKYPSRQQFVDYLESYAEKFSLRPRFGQLVVSAEFEGGLWRVRTEDGAEYVSRWIVVATGENADPVVPEIHGLDRFGGRILHASLYKLGADFEGEKVLVVGCGNTGMEVSLDLSMHNATPFMVVRDTVHVLPRDIFGFSTFGVAMALLKWLPLKVVDGILILAANSTIGSTDRLGIRRPATGPLELKDTTGKTPVLDVGALSAIKAGKIKVMKGVTEITRRGAKFEDGAEEEIDSIVLATGYKSNVPSWLKGTDFFTAEGMPRTPFPNGWNGGNGLYTVGLTGRGLLGASLDAVNVARDVVKQWRTKEDADKPNFHLALSS

>Asa4G04252.1|Allium_sativum/1-397

MEMGSMPASPHIHKNGTLIHGPLIIGAGPSGLAVAACLSNLHIPSLILEKSNCLASLWQNKTYNRLKLHLPKQFCELPMMPFPSHFPKYPSKEQFISYLESYALKFGIEPKFCCAVKSTEFEEKIGFWKVVTENGNEYLSKWVVVATGENADAFVPEIHGMEKFCKRAVHTCGYKSGKIFKGEKVLVVGCGNSGMEVSLDLCRHGAKPYLVVRNSVHVLPREMLGISTFGVAMALLKWLPLKFVDKFLLTMANLILGDTDKLGLKRPKVGPIELKNLTGKTPVLDVGTLAQIKSGNITVMVGVKEMTREGAKFVDGKEENFDSVIFATGYKSNVPAWLKGGDDLFTKEGIPKEEFPNGWKGENGLYCVGFTRRGLLGTSCDALNIARDISMKWCSET

>Acora.12G097100|Aamericanus_v1.1/1-418

MGSCGEEETELKCTWVHGPVIVGAGPSGLAVAASLSESGVPSLVLEKSDCVASLWQHRTYDRLKLHLPRQFCELPMMGFPESFPKYPSKLQFIQYMESYASRFGIRPLFRCAVSEARYDEGLGGWRVAVEGEGEVVYVSRWLVVATGENSEAAVPELQGLERFCGGSVLHTSDYKTGANFEAKKVMVVGCGNSGMEVSLDLCRHNARPYMVVRNSVHVLPREIFGMSTFGVAMALLKWLPLKAVDKFLLAMAQLILGDTNRYGLRRPKTGPLELKNSTGKTPVLDIGALAQIKSGKIKVVEGVKEITKRGAKFVDGKEEDFDAIILATGYKSNVPSWLKGSELFTKEGMPKTQFPNGWKGEKGLYSVGFTRRGLLGTCSDALNIAKDISQQYLKMTTPTTMDPKCQNQLSHLVLHLRP

>Zosma01g06000|Zmarina_v3.1/1-402

MDWVMKLKHDRKNCTWVHGPIIVGAGPSGMAVAACLSNLGIPSTILEKSNCVASMWQYKTYDRLKLHLPKQFCQLPLLGFPPNFPKYPSKDQFIAYMESYARAFDIKPVFGCTVNLAEYDAANSCWKLTTSEEGVFFSRWLVVASGENADPVLPEMVGIEKFGGRVIHSCCYKSGKEFQGKNVLVIGCGNSGMEVSLDLCRHNAKPFMVARNSVHVLPREMLGVSTFSITMALLKWLPVRLVDRFLLLGARLSLGNTDRVGLHRPKAGPIELKNTTGKTPVLDVGSLSLIKSGKIMVTKGVREVTKRGARFVDGKYQDFDSIILATGYKSNVPSWLKACCICLTDCGMFTEEGKSRKLFPNGWKGEKGLYSVGFTLRGILGTCSDSKNIATDIAILWEKEAS

>evm_27.TU.AmTr_v1.0_scaffold00122.41|Atrichopoda_v1.0/1-416

MGLLDTDLQGQNHRCVLVNGPIIIGAGPSGLAASACLRSHGVPSIILEKTDCIASLWQYRAYHRLKLHLPKHFCELPLMDFPPNFPLYPTKQQFISYIESYAEKFEIRPEFNRLVKSASYDPVCGFWRVKTADLSNGFLGNFVEYIGKWLVVATGENAEPLIPEIDGLNKFLGPVLHTSVYKTGEEFKEQKVLVVGCGNSGMEVCLDLCSHGAEAYMVARNSVHILPRDMFGISTFGVAMSLLNFFPLKLVDKFLLLMANLMLGRTEKYGLRRPSIGPIELKNGSGKTPVLDVGALSKIKSGGIKVMGGVKEVTHRGAKFVNGEEKEFDALILATGYKSNVPTWLKECDFFSEDGMPKMPFPNGWKGEKGLYSVGFTNRGILGAAQDARKVASDIALEWRKFSQFKRFIVIKPSLK

>evm_27.TU.AmTr_v1.0_scaffold00002.564|Atrichopoda_v1.0/1-424

MDCVCKQRGKGSQDQHQHLISFWVSGPVIVGAGPSGLATAACLKQQSVPSIILEKEDCMASMWQYKTYGRLKLHLPKYFCELPLMEFPQTFPTYPTKEQFISYINAYAERFNIRPRFNRRVQSACYDSGLKRWRVRTEMVRERESILVEYICRWLVVATGENAEPMVPDLPGLSSFKGRVVHTSDYKTGSEMEGQKVLVIGSGNSGMEVCLDLCNHDAKPFMVVRNSVHVLPREMFGLSTFGVAMGLLKWLPLRMVDKFLLMASHVILGSTEKQGLKRPEMGPIELKNTTGKTPVLDVGALAKIKSGKIKIVPAVKSLTLNGAKFVNGQEEAFDSVILATGYKSNVPSWLKERELFGEEGLPRKSFPNGWKGEKGLYSVGFTRRGLLGVGSDARNVAQDIGAQWRNLGERPHHHKHFILYTKPG

>Nycol.F00334|Ncolorata_v1.2b/1-419

MDRLASSETEARCVWVNGPVIVGAGPSGLATAAWLGRLGVPCVVLDRADRIASLWHYRTYDRLKLHLPKQFCQLPFLPFPPHFPEYPSKHQFLSYLHSYAAKFGIRPRFNQSVLSTQFDPLCGLWRVRTLDLAADSGRTTEYICKWVVVASGENAQPVLPDIPGLDKFKGRVLHTCAYKSGEDFRGQRVLVVGCGNSGMEVSLDLCNQGAKPYMVVRNSVHVLPREILGFSTFGVAMGLLKCFPLKLVDKFLLTCALVAFGDTKKHGIPRPKVGPLELKNVAGKTPVLDVGALSKIRDGKIKVMRGVKEVTCCGAKFVDGQEVEFDAIILATGYKSNVPSWLKGTESTVHFSEDGMPRLPFPNGWKGENGLYTVGFTRRGILGACADATKIAHDIAEQWRTPATTETTRFIVSKRSSTQ

>g27490.t1_1|Euryale_ferox/1-419

MDQLASSETEARCVWVNGPVIVGAGPSGLATAAWLGRLGVPYVVLDRADRIASLWHYRTYDRLKLHLPRQFCQLPFLPFPPHFPEYPSKHQFLSYLHSYATKFGIRPRFNQSVLSAHFDPLCGLWRVRALDLADGSGRSTTEYICRWVVVASGENAESVLPDIPGLDSFKGRVLHTCAYKSGEDFRGQRVLVVGCGNSGMEVSLDLCNQGAKPYMVVRNSVHVLPREILGFSTFGVAMGLLKCFSLRLVDRFLLMCALLAFGDTKKHGIPRPKVGPLELKNVAGKTPVLDVGALSKIREGKIKVMRGVKEVTCCGAKFVDGQEVGFDAIILATGYKSNVPSWLKGTESTAHFSEDGMPRLPFPNGWKGENGLYTVGFTRRGILGACADATKIAHDITEQWRTPATETTRFIVSNRSSIQ

>g36230.t1_1|Euryale_ferox/1-418

MDQLLSSETEVKCVWVNGPVIVGAGPSGLATAACLGRLGVPCVVLDRADRIASLWQYRTYDRLKLHLPKQFCQLPFLPFPPHFPEYPSKHQFLSYLHSYATKFGIRPRFNQSVLSAHFDPLCGLWRLRTLDLAAGSGTTTEYICRWVVVASGENAEPVLPHIPGLDKFKGRVLHTCSYKSGEDFRGQKVIVVGCGNSGMEVSLDLCNQGAKPYMVVRNSVHVLPREILGFSTFGISMGLLKCFPLRLVDKFLLMCALVAFGDTKKHGIPRPKVGPLELKNVAGKTPVLDVGALSKIKEGKIKVMRGVKEVTCCGAKFVDGQEVEFDAIILATGYKSNVPSWLKGSESTAHFSVDGMPRLPFPNGWKGENGLYTVGFTRRGILGACADATNIAHDIAEQWRTPATETTRFIVSKSSSVQ

>Nycol.A02388|Ncolorata_v1.2b/1-420

MESKEAELLRCVWVNGPIIVGAGPSGLATAASLGQLGVPCVVLDRSDRIASLWHYRTYDRLKLHLPKQFCQLPFLPFPPHFPDYPSKHQFLSYVNSYASRFNIRPRFNQSVQSARFDPLCSLWRLRTLDLSSPDSKSTVEYISRWLIVASGENAEPLLPDIPGLEQFAGPVLHTSEYKSGEDFRGQRVLVVGCGNSGMEVSLDLCNQAAVPYMVVRNSVHVLPREILGFSTFGISMALLKWLPLRVVDKFLLLCALATFGSTKKHGIPRPKIGPLELKNVAGKTPVLDVGALAKIREGKIKVMPGVKEVTCHGAKFVDGQEEEFDSVVLATGYKSNVPSWLKGTDHFSEDGMPKMPFPNGWKGEHGLYSVGFTRRGILGACADASSIARDIALQWRTPATEPTRFIVSKGSPSPPPHSRN

>g21205.t1_1|Euryale_ferox/1-420

MESKEAELLRCVWVNGPIIVGAGPSGLATAASLGQLGVPCVVLDRSDCIASLWHYRTYDRLKLHLPKQFCQLPFLPFPSDFPDYPSKHQFLSYVNSYASRFNIRPRFNQSVQSARFDPLCGLWRLRTLDLSSPDSKRTNEYISRWLIVASGENAEPVFPDIPGLEQFAGPVLHTSQYKSGEHFRGQRVLVVGCGNSGMEVSLDLCNQGAVPYMVVRNSVHVLPREILGFSTFGISMALLKWLPLRVVDKFLLLCALATFGRTKKHGIPRPKIGPLELKNVSGKTPVLDVGALAKIREGKIKVMPGVKEVTCHGAKFVDGQEMEFDSVVMATGYKSNVPSWLKGTNHFSEDGMPNMPFPNGWKGGHGLYTVGFTRRGILGACADASRIACDVALQWRTPATEPTRFIVSKGSPPPPPHIRN

>g24781.t1_1|Euryale_ferox/1-419

MESKEAELLRCVWVNGPIVVGAGPSGLATAVSLGQLGVPCVVLDRSDCIASLWHYRTYDRLKLHLPKQFCQLPFLPFPPHFPEYPSKHQFLSYVNSYASRFDIRPRFNQSVHSARFDPLCGLWRLRTIDLSSPDSKRTVEYFSRWLIVASGENAEPMLPDIPGLEQFAGPVLHTSKYKSGEHFRGQRVLVVGCGNSGMEVSLDLCNQGAVPYMVVRNSVHVLPREILGFSTFGISMALLKWLPLRVVDKFLLLCALATFGFTKKHGIPRPKIGPLELKNVAGKTPVLDVGAVAKIREGKIMVMPGVKEVTCHGAKFVDGQEMEFDSVVLATGYKSNVPSWLKGTDHFSEDGMPKMPFPNGWKGEHGLYTVGFTRRGILGACADASRIARDVALQWRTPATEPTRFIVSKCSPTPPHIRN

>Aco005446|Acomosus_v3/1-415

MGSSQKRVWVPGPIIIGAGPSGLAAAACLAELGVPSTVLEKSSCVASLWRHRTYDRLKLHLPKRFCELPLMGFPAGFPKYPTKHQFVAYMESYAAAFGIAPRFGAEVVRAERDAAAGAWRVTTRLAAAGPGPGAEEEEEELVSRWVVVATGENAEPVGAEVISGIERFAGRVVHTSAYKTGAEFSGQKVLVVGCGNSGMEVSLDLCRHNAKPSMVVRNSVHVLPREMLGMSTFGVAMALLKWLPVRVVDRLLLAAAHMILGDTGRLGLRRPKTGPIELKNLTGKTPVLDVGALDQIKSGNITVVGGVKEIMKGGAKFVDGREEKYDAIILATGYKSNVPSWLKDADEHFTKEGIPKTPFPSGWKGDKGLYCVGFTRRGLLGTSSDAVNIANDIYRQWTELSDHSTPLHHLFDFKV

>LOC_Os01g45760|Osativa_v7.0/1-406

MDNKPAQERRETWVPGAVIVGAGPSGLAAAACLAARGVPATVLERSDSLASTWRHRMYDRLALHLPKRFCELPLLPFPEEYPTYPSKDQFVAYMEAYAAAAGVAPRFGATVEEAAFDAAVGAWRVRLDGGEVLMARWLVVATGENAEPRVPDFPGMQKFAGCAMHTSEYKSGEQFAGKKVLVVGCGNSGMEVSLDLCRHGAKPSMVVRNTVHVLPREMFGLSTFGIAMALLRWLPVQLVDRFLLTAAHLILGNTGQFGLRRPKTGPIELKNLTGRTPVLDVGTLDHIKSGKIKVVGAVKEMTRQGVRFTDGKEEQFDTIILATGYRSNVPSWLKDAGDLFTREGISKVPFPNSWRGRNGLYTVGFTQRGLLGTSSDALNVAKDIHCQWRERDRSAINVLEISNSSF

>Bradi2g45105|Bdistachyon_v3.2/1-406

MDKEQAEGRRATWVPGAVIVGAGPSGLAAAACLKARGVPATVLERSDSLAFTWRHRMYDRLALHLPKRFCELPLLPFSDKYPTYPSKKQFLFYMEEYAAKAGITPRFGASVEEAAFDASVGAWIVRLAGGEVLMAKWLVVATGENAEPHIPEFPGMSQFGGRVMHTCDYKSGEEFAGKKVLVVGCGNSGMEVSLDLCRYGAKPSMVVRNTVHVLPREMLRLSTFGIAMALLKWFPVQLVDRLLLAAAHLVLGDTGQLGLRRPKTGPIELKNLTGKTPVLDVGTLDHIKSGKIKVVGAVKEVTRRGARLADGKEEQQFDAIILATGYRSNVPSWLKDGGDVFTREGTPKARFPNCWKGRNGLYTVGFSQRGLLGASSDALSVAIDIHCQWRERERPTKNVFQVNNSV

>HORVU.MOREX.r3.3HG0275500|Hvulgare_Morex_V3/1-406

MDKEQEEEGRATWVSGALIVGAGPSGLAAAACLAARGVPATVLERSDSLAFTWRHRMYDRLALHLPKHFCELPLLPFPAEYPTYPCKDQFVAYMEAYAAAAGVAPRFGASVEEAAFDASVGAWAVRLAGGEVLMTRWLVVATGENAEPHVPDFPGMRQFAGRVLHTCDYKCGEEFAGEKVLVVGCGNSGMEVSLDLCRHGAKPSMVVRSTVHVLPKEMLGVSTFGIAMALLKWLPVQLVDRFILAAARLTLGNTSQLGLRRPKTGPIELKNLTGRTPVLDVGTLDHIKSGKIKVVGAVKEVTRRGARLADGKEEEQFDAIILATGYRSNVPSWLKDGGDVFTREGTPKTPFPDGWKGRNGLYTVGFSQRGLLGASSDALCVARDIHCQWRERDKPTSDVHQADSSV

>Zm00001d044069|Zmays_RefGen_V4/1-418

MDDDKQQLARQEDQAGRASWVPGAVIVGAGPSGLAVAACLAARGVPATVLEMSDSLASTWRHRTYDRLTLHLPKRFCELPLLPFPRGYPAYPSKGQFVAYLEAYAAAAGVAPRFGARVEEAAFDAGAGAWALRLAGAGGGGAGDLLLARWLVVATGENAVPRLPDLPGAARFAGRVLHTCDYRSGEEFAGRKVLVVGCGNSGMEVSLDLCRHGAAPSMVVRNTVHVLPREMLGLSTFGIAMALLKLLPVRVVDRILLAAARLALGDTGKLGLRRPKTGPIELKNLTGRTPVLDVGTLAHIKTGKIKVVGAVKEVTQRGVRFADGKEEQFDAIIQATGYRSNVPSWLKDGGDVFTSEGMPRIPFPNGWKGKNGLYAVGFSQRGLLGASADALNIARDIHRQWTDTATRPVVLRCNSAVV

>Sobic.003G236900|Sbicolor_v5.1/1-422

MDMDDDMARQQEEEQGRARAAWVPGAVIVGAGPSGLAAAACLAARGVPATVLEMSDSLASTWRHRTYDRLTLHLPKRFCELPLLPFPPGYPAYPSKDQFVAYLESYAAAAGVAPRFGCRVEEAAFDAGAGAWAVRVAGEGEGSGGELLLLARWLVVATGENAVPRTPDLPGAPERFAGRVLHTCDYKSGEEFAGKKVLVVGCGNSGMEVSLDLCRHGATPSMVVRNTVHVLPREMLGVSTFGIAMALLKLLPVQVVDRILLAAARLALGDTGKLGLRRPKTGPIELKNLTGRTPVLDVGTLAHIKTGKIKVVGAVKEVTQRGVRFADGKEEQFDAIIQATGYRSNVPSWLKDGGDVFTREGMPRIPFPNGWKGKNGLYTVGFSQRGLLGASADALNIARDIHSQWKQDMMSRPAGNKYCASM

>ELECO.r07.1AG0026300|Ecoracana_v1.1/1-413

MAMDEASSQEATWVPGAIIVGAGPSGLAAAACLAARGVPSTVLEMSDSLASTWRHRTYDRLTLHLPRRFCELPFLPFPEDYPPYPTKDQFVAYMESYAAASGVAPRFGARVEEAAFDAAAGAWRVRLAGAGGDDETQTLVARWLVVATGENAVPRVPEFPGGAGRFAGRVLHTCDYKSGEAFAGEKVLVVGCGNSGMEVSLDLCRHGAKPSLVVRNTVHVLPREMLGLSTFGIAMALLKWLPVRLVDRILLAAAHLALGDTGQLGLRRPKTGPIELKNLTGRTPVLDVGTLHHIKTGKIKVVGAVKEVTRRGVRFADGKEEQFDAIILATGYRSNVPTWLKDGGDVFTKEGMPRIPFPNGWKGRNGLYTVGFSQRGLLGTSSDALNVARDIHCQWNDTGRHPANNVLKSNDSV

>ELECO.r07.1BG0076760|Ecoracana_v1.1/1-414

MAMDEASSQEAATWVPGAIIVGAGPSGLAAAACLAARGVPSTVLEMSDSLASTWRHRTYDRLTLHLPRRFCELPFLPFPEDYPPYPTKDQFVAYMESYAAASGVAPRFGARVEEAAFDAAAGAWRVRLAGAGGDETQTLVARWLVVATGENAVPRVPELFPGGAGRFAGRVLHTCDYNSGEAFAGEKVLVVGCGNSGMEVRLDLCRHGAKPSLVVRNTVHVLPREMLGLSTFGIAMALLKWLPVRLVDRILLAAAHLALGDTGQLGLRRPKTGPIELKNLTGRTPVLDVGTLHHIKTGKIKVVGAVKEVTRRGVRFADGKEEQFDAIILATGYRSNVPTWLKDGGDVFTREGMPRIPFPNGWKGRNGLYTVGFSQRGLLGTSSDALNVARDIYCQWNDTGRHPANNVLESNDSV

>evm.TU.supercontig_59.54|Cpapaya_ASGPBv0.4/1-420

MTNPCSMFDEPDVFSRRSIWVNGPVIVGAGPSGLAVAAGLKTQGVPFIILERANCIASLWQHRTYNRLRLHLPKQFCQLPYFPFPDHFPQYPTTAQFIQYLESYANHFHITPQFNETVQSAKYDETFGLWLVKTIRGSGGEVEYICRWLVVATGENAEKVVPEFEGLGDFGGDVSHACQYKSGESYCGKKVLVVGCGNSGMEVSLDLCNHNASPSMVVRNSVHVLPREILGRSTFELAVMLMKWIPIRVVDKILLILARVILGNVEKYGVKRPPMGPLELKNTVGKTPVLDIGALQKIKSGEIKIAPGIKKFYRDKVEFINGESLEIDSVVLATGYRSNVPSWLKENEFFTEDGIPKNPFPNGWKGKAGLYAVGFTRRGLSGASLDAISVVHDIAKSWKEETKQKKKSIAARHRRCISHF

>Cucsa.255980|Csativus_v1.0/1-384

MDGDHFFDHRCIWVNGPVIVGAGPSGLSVAAALKQQGVPFVVVDRADCIASLWQHRTYDRLKLHLPKQFCQLPNFPFPEEFPAYPSKFQFISYLESYANHFDITPCFNETVQSAKYDETFGLWRVKTLSLSPKPTQVEYICRWLVVATGENAEKVVPEFEGMEEFGGHVMHACEYKSGEAYRGKRVLVVGCGNSGMEISLDLCNHDAKPSMVVRSSVHVLPREILGKSTFEVAISLMKWLPLHMVDKMLLIIARLVLGNVEKYGLKRPSVGPLQLKNAAGKTPVLDIGALQKIRAGEIKVVAGIKKFRRGAVELVNGEVIEIDSVILATGYRSNVPSWLKENEFFSSDGIPKSPFPNGWKGKAGLYAVGFTRKGLSGASLDAIN

>Eucgr.H02874|Egrandis_v2.0/1-422

MLQSLDHGTFFSRRCIWVNGPVIVGAGPSGLAVGAGLKQQGVPFIILERANCIASLWQNRTYDRLKLHLPKQFCQLPDFPFPEDFPEYPTKHQFITYLDSYARHFDISPNFNETVQSAKYDETFGLWRVRTVLAGGPGLGQLEVEYICRWLVVASGENAEKVVPEFEGLESFGGDVTHACDYKSGENFRGKRVLVVGCGNSGMEVSLDLCNHNARPSMVVRSSVHVLPKEVLGKSTFELAVFMMKWLPLWLIDKILMFLAWLILGNVEKYGLKRPKIGPLQLKNAAGKTPVLDIGALSKIRSGQIKVVVGIKKFSSGRVELLDGEVLDIDSVVLATGYRSNVPSWLKENEFFSEDGILKNPFPNGWKGRAGLYAVGFTRKGLSGASLDAISVAHDIGRSWKEDMKQRKHSVTARHRRCISHF

>Prupe.1G054300|Ppersica_v2.1/1-424

MVPHSFDQDDLFARRCIWVNGPVIVGAGPSGLAVGAGLKEQGVPFIILERANCIASLWQKRTYDRLKLHLPKQFCQLPNFPFPEDFPEYPTKNQFINYLESYAQHFDISPQFNETVQSAKYDETFGLWRVKTIVSTSNSNPAAAEVEYICRWLVVATGENAEKVVPEFEGLEEFGGNVMHVCDYKSGVAYRGKQVLVVGCGNSGMEVSLDLCNHNASPSMVVRSSVHVLPREICGKSTFGLAVLLMKWLPLWLADKILLILAWLIFGNLEKYGLRRPSLGPLELKNISGKTPVLDIGALQKIRSGEIKVVPGIKRFSHGRVELVDGQNLEIDSVILATGYRSNVPSWLKENEFFSREGIPKNSFPNGWKGKGGLYAVGFTRRGLSGASLDAMGVSQDIAKSWKEETKQKRKSVAARHRRCISHF

>FvH4_4g05280|Fvesca_v4.0.a2/1-428

MMFDSACHSQSPDQNELFSRRCVWVNGPVIVGAGPSGLAVGAGLKDQGVPFIILERADCIASLWQKRTYDRLKLHLPKQFCQLPNFPFPEDFPEYPSKNQFIKYLESYAKHFDINPNFNETVQSAKYDETFGLWRVKTIAQSGTVFAVEVEYICRWLVVATGENSEKVVPEFEGLENFGGHVMHACDYRSGAAYGGKNVLVVGCGNSGMEVSLDLCNHDASPSMVVRSSVHVLPREILGKSTFELAVFLMKWVPLWLADKILLILAWIFLGNLQKYGIKRPSTGPLQLKHNSGKTPVLDIGALQKIRSGEIKVVPGIKRFSHGSVELVNGQNLMIDSVILATGYRSNVPSWLKENEFFSGDGIPRNPFPNGWKGKAGLYAVGFTRRGLSGASLDAIGVSQDISKSWKEETKQKRKTVAARHRRCISHF

>Potri.008G174600|Ptrichocarpa_v4.1/1-431

MDNTCLNTPNMFQTSGPDHDFFARRCIWVNGPVIVGAGPSGLAVGSGLRGQGVPFVMLERANCIASLWQNRTYDRLKLHIPKQFCQLPSFPFPEDFPEYPTKYQFINYLESYAKHFNINPHFNETVQSAKYDETFGLWRVKTVSTSSSNPSEVEYICRWLVVATGENAEKFLPEFEGLQDFGGHVMHACDYKSGESYHGERVLVVGCGNSGMEVSLDLCNHNASPSMVVRSSVHVLPREILGRSTFELAVTMMKWLPLWMVDKILLLFAWLILGNLEKYGLTRPCLGPLQLKNTQGKTPVLDIGALEKIRSGKIKVVPGIKRFSSGKAELVNGEILQIDSVILATGYRSNVPSWLKENEFFSEDGIPKNQFPNGWKGNAGLYAVGFTKRGLSGASLDAISVAFDIAKSWKEETKQKKITVAARHRRCISHF

>Gohir.A05G316900|Ghirsutum_v3.1/1-411

MLSRRCIWVNGPVIVGAGPSGLAVGAGLKNQGVPFIILERADCIASLWQKRTYDRLKLHLPKQYCQLPYFPFPDDFPEYPTKHQFIKYLESYAKHFDINPNFNETVQSAKYDETFGLWRVKTISTGGPIPVEVEYICRWLAVATGENAEKVVPEFEGLEDFGGHVTHACDYKSGKSYFGERVLVVGCGNSGMEVSLDLCNHNANPSMVVRSSVHVLPREVFGTSTFEFAVSLMKWLPLWLVDKILIILAYLILGNIEKYGLKRPCLGPLELKNTAGKTPVLDIGALQKIKSGKIKIVPGIKKFSRGKVELVNGQTLDIDSVILATGYRSNVPSWLKENELFSNEGVPKNPFPNGWKSKGGLYAVGFTRRGLSGASLDAISVALDIAKTWKDETKQKKKSVAARHRRCISHF

>Gohir.D05G318300|Ghirsutum_v3.1/1-411

MFSRRCIWVNGPVIVGAGPSGLAVGAGLKNQGVPFIILERADCIASLWQKRTYDRLKLHLPKQYCQLPYFPFPDDFPEYPTKHQFIKYLESYAKHFDINPNFNETVQSAKYDETFGLWRVKTISTGGPIPVEVEYICRWLAVATGENAEKVVPEFEGLEDFGGHVTHACDYKSGKSYFGERVLVVGCGNSGMEVSLDLCNHNANPSMVVRSSVHVLPREVFSSSTFEFAVSLMKWLPLWLVDKILIILAYLILGNIEKYGLKRPCLGPLELKNTAGKTPVLDIGALQKIKSGKIKIVPGIKKFSRGKVELVNGHTLDIDSVILATGYRSNVPSWLKENELFSSDGVPKNPFPNGWKSKGGLYAVGFTRRGLSGASLDAISVALDIAKTWKDETKQKKKSVATRHRRCISHF

>Gohir.D10G146900|Ghirsutum_v3.1/1-430

MSSCCLNMPNMFPTLASEAFLSSRCIWVNGPVIVGAGPSGLAVGAGLKNQGVPFIILERANCIASLWQNRTYDRLKLHLPKQFCQLPNFPFPEDFPEYPTKYQFISYLESYTKHFDINPHFNETVQSANYNATFGLWRVKTIATGGPNPIGVEYICRWLVVATGENAEKVVPEFEGLQDFGGHVTHACDYKSGQSYSGERVLVVGCGNSGMEVSLDLCNHNANPSMVVRSSVHVLPREVLGKSTFELAVSMMKWLPLWLVDKILLILTHLILGNTEKYGLKRPRVGPLELKNSAGKTPVLDIGAFQKIRSGEIKIVPGIKKFSRGRVELVNGETLEIESVILATGYRSNVPSWLKENELFSSEGVPKNPFPNGWKGKGGLYAVGFTRRGLSGASLDAISVAHDIAKSWKEETKQKKKSMAARHRRCISHF

>Gohir.A10G119700|Ghirsutum_v3.1/1-430

MSSCCLNMPNMFPTLASEDFLSSRCIWVNGPVIVGAGPSGLAVGAGLKNQGVPFIILERANCIASLWQNRTYDRLKLHLPKQFCQLPNFPFPEDFPEYPTKYQFISYLESYAKHFDINPHFNETVQSANYDETFGLWRVKTIATGGPNPIGVECICRWLVVATGENAEKVVPEFEGLQDFGDHVTHACDYKSGQSYSGERVLVVGCGNSGMEVSLDLCNHNANPSMVVRSSVHVLPREVLGKSTFELAVSMMKWLPLWLVDKILLILAHLILGNTEKYGLKKPCVGPLELRNTAGKTPVLDIGAFQKIRSGEIKIVPGIKKFSRGRVELVNGETLEIESVILATGYRSNVPSWLKENEFFSSEGVPKNPFPNGWKGKGGLYAVGFTRRGLSGASLDAISVAHDIAKSWKEETKQKKKSMAARHRRCISHF

>Glyma.10G128700|Gmax_Wm82.a6.v1/1-441

MHDICLNMPPMVQTFDPEDLFKRRCIWVNGPVIVGAGPSGLAVAACLKEQGVPFLILERANCIASLWQNRTYDRLKLHLPKQFCQLPNFPFPEDFPEYPTKFQFISYLESYAKHFNISPQFNETVQSAKYDETFGLWRVKTIRKIKKLGEASSGCCGAVECEVEYICRWLVVATGENSEKVVPEFEGLGEFGGHVMHACDYKSGEGYGGQKVLVVGCGNSGMEVSLDLCNHNANPSMVVRSSVHVLPREAFGKSTFELAVMLMKRFPLWMVDKILLVLARLILGNVEKYGLKRPSVGPLELKHTAGKTPVLDIGALEKIRSGKIKVVPGIRRFFPGKVELVDGQVLQIDSVVLATGYHSNVPSWLKENDFFTSDGTPRNPFPNGWRGKGGLYAVGFTRKGLSGASLDAINVAHDIAKNWKEENKQKRKTVAARHRRCISHF

>Glyma.20G080000|Gmax_Wm82.a6.v1/1-439

MHDICLNMPPMVQTFDPEDLFTRRCIWVNGPVIVGAGPSGLAVAACLKDQGVPFIILERANCIASLWQNRTYDRLKLHLPKQFCQLPNVPFPEDFPEYPTKFQFISYLESYAKHFSIAPQFNETVQSAKYDETFGLWRIKTIRKIKKLGGLSSGGCAECEVEYICRSLVVATGENSEKVVPEFEGLGEFGGHVMHACDYKSGEGYGGQKVLVVGCGNSGMEVSLDLCNHNANPSLVVRSSVHVLPREVFGKSTFELAVTLMKRFPLWIVDKILLILARLILGNVEKYGLKRPSVGPLELKHTAGKTPVLDIGALEKIRSGKIKVVPGIRRFLPGKVELVDGQVLQIDSVVLATGYHSNVPSWLKEDDFFTNDGTPRNPFPNGWRGKGGLYAVGFTRRGLSGASLDAINVAHDIVKSWKEETKQKRKTVAARHRRCISHF

>KAI5397693.1|Pisum_sativum/1-465

QTTKSHLILILFSSHNSQFISQNLKIFNHKTTKMHNISLNMPPITQTFNPEHIFKNRCIWVNGPVIVGAGPSGLAVAACLKDQNVPFVIVERANCIASLWQNRTYDRLKLHLPKQLCQLPNFPFPDDFPEYPSKFQFINYLDSYAKHFNITPNFNETVQSAKYDETFGLWRIKTIRKKSDEFEFVEVEYICKWLVVATGENAEKVVPEFEGLNDFGGHVMHACDYKSGESYSGKKVLVVGCGNSGMEVSLDLCNHNANPSMVIRSSVHVLPREVFGKSTYELAAMLMKKLPIWMVDKILLVLTRLILGNVEKYGVKRPCVGPLELKNISGRTPVLDIGALEKIRSGKINVVPGIKRFSQGKVELVDGSFLDIDSVVLATGYRSNVPSWLKENEFFSEDGIPKDPFPNGWKGKAGLYAVGFTRKGLSGASLDAISVSHDIAKSLKEETKQRKKTVAARHRRCISHF

>Brara.J00274|BrapaFPsc_v1.3/1-436

MHRNNNKKSLNISSMFQTFIPDSNIFSRRCIWVNGPVIVGAGPSGLAVAAGLKREGVPFIILERANCIASLWQNRTYDRLKLHLPKQFCQLPNFPFPEDYPEYPTKFQFIQYLEEYATHFDINPKYNETVQSAKYDETFGLWRVKTISKSGQLGSCEFEYICRWLVVATGENAEKVVPDFEGLEDFGGDVLHAGDYKSGGRYQGKKVLVVGCGNSGMEVSLDLYNHGANPSMVVRSSVHVLPREILGKSTFELGVTMMKWMPVWLADKTLLLLARIVLGNTDKYGLKRPTIGPLELKNKEGKTPVLDIGALPKIRAGKIKIVPGIIKFGRGMVELVDGRVLDIDSVILATGYRSNVPSWLKDNDFFSDDGIPKNPFPNGWKGEAGLYAVGFTRKGLFGASLDAMSVAHDIANRWKEESKQQKKTAAARHRRCISHF

>Brara.I05483|BrapaFPsc_v1.3/1-436

MYRNNNKKSLNISSMFGNFIPDSDIFSRRCIWVNGPVIVGAGPSGLAVAAGLKREGVPFIILERANCIASLWQNRTYDRLKLHLPKQFCQLPNFPFPEDYPEYPTKFQFIQYLEDYATHFDINPKYNETVQSAKYDETFGLWRVKTVSKSGPLGSCEFEYICRTLVVATGENAEKVVPDFEGLEDFGGDVLHAGDYKSGGRYQGKKVLVVGCGNSGMEVSLDLYNHGANPSMVVRSSVHVLPREILGKSTFELGVTMMKWMPVWLADKTLLFLARIVLGDTDKYGLKRPKIGPLELKNLEGKTPVLDIGALPKIRSGNIKIVPGIIKFGRGKVELVDGRVLEIDSVILATGYRSNVPSWLKDNDFFSDDGIPKNPFPNGWKGEAGLYAVGFTRKGLFGASLDAMSVAHDIACRWKEESKQHKKTAAARHRRCISHF

>AT1G04610|Athaliana_Araport11/1-437

MYGNNNKKSINITSMFQNLIPEGSDIFSRRCIWVNGPVIVGAGPSGLAVAAGLKREGVPFIILERANCIASLWQNRTYDRLKLHLPKQFCQLPNYPFPDEFPEYPTKFQFIQYLESYAANFDINPKFNETVQSAKYDETFGLWRVKTISNMGQLGSCEFEYICRWIVVATGENAEKVVPDFEGLEDFGGDVLHAGDYKSGGRYQGKKVLVVGCGNSGMEVSLDLYNHGANPSMVVRSAVHVLPREIFGKSTFELGVTMMKYMPVWLADKTILFLARIILGNTDKYGLKRPKIGPLELKNKEGKTPVLDIGALPKIRSGKIKIVPGIIKFGKGKVELIDGRVLEIDSVILATGYRSNVPSWLKDNDFFSDDGIPKNPFPNGWKGEAGLYAVGFTRKGLFGASLDAMSVAHDIANRWKEESKQQKKTAAARHRRCISHF

>Brara.E01069|BrapaFPsc_v1.3/1-436

MCTNQNTNCVSISSMLPNFTPKEDDMFSRRCIWVNGPVIVGAGPSGLAVAASLKRQEVPYIILERANCIASLWQNRTYDRLKLHLPKQFCQLPNLPFPDDFPEYPTKYQFIQYLESYATHFDIQPKFNETVQSAKYDKRFGLWRVKTVSRKGLLSSCEFEYICRWLVVATGENAEKVVPEFEGLEDFCGDVLHASDYKSGEMYRGKRVLVVGCGNSGMEVSLDLCNHDASPSMVVRSSVHVLPREILGKSTFEIGVTMMKWMPVWLVDKTLLLLTRLSLGNTDKYGLKRPELGPLELKNTAGKTPVLDVGAFSKIKSGKIKIVPGIVKFGPGKAELVDGRVLEIDSVVLATGYRSNVPSWLKENDLAEIGIEKNPFPKGWKGEAGLYAVGFTRRGLSGASLDAISVAHDIANAWKEETKQQIKSVAARHRRCISQF

>AT2G33230|Athaliana_Araport11/1-431

MCNNNNTSCVNISSMLQPEDIFSRRCIWVNGPVIVGAGPSGLAVAADLKRQEVPFVILERANCIASLWQNRTYDRLKLHLPKQFCQLPNLPFPEDIPEYPTKYQFIEYLESYATHFDLRPKFNETVQSAKYDKRFGLWRVQTVLRSELLGYCEFEYICRWLVVATGENAEKVVPEFEGLEDFGGDVLHAGDYKSGERYRGKRVLVVGCGNSGMEVSLDLCNHDASPSMVVRSSVHVLPREVLGKSTFELSVTMMKWMPVWLVDKTLLVLTRLLLGNTDKYGLKRPEIGPLELKNTAGKTPVLDIGAISMIKSGKIKIVAGIAKFGPGKVELVDGRVLQIDSVILATGYRSNVPSWLKENDLGEIGIEKNPFPKGWKGKAGLYAVGFTGRGLSGASFDAMSVAHDIANSWKEETKQQIKTVATRHRRCISHF

>Hyque.10G114700|Hquercifolia_v1.1/1-420

MSHAFDFENFFARRCIWVNGPVIVGAGPSGLAVGAGLKQQGVPFVILERANCIASLWQNRTYDRLKLHLPKQFCELPYLKFPQNFSEYPTKYQFIDYLESYAKNFDINPRFNESVQFAKYDETCGLWRVKTVNTSGSRGEVEYICRWLVVATGENAEKVVPEFEGLQDFGGHVIHSCDYKSGETYEGKSVLVVGCGNSGMEVSLDLCHHKAIPSMVVRSPVHVLPREVIGKSTFELAVSISKWLPIWVVDKILLVIARSVLGNIEKYGLERPKIGPLELKNTEGKTPVLDIGALQKIRSGEIKLVPGIKRFTRGRVQLVNGEILEIDCVILATGYCSNVPSWLEESHFFSRNGFPRNPFPNGWKGKPGLYAVGFTRRGLSGASLDAIRVAQDIGKIWKEETKHKKHSVAVACHRRSKSHC

>Rhdel09G0061800.1|Rhododendron_delavayi/1-436

MMHYSTDHLPMPTMIQNPDHENFFPGRCILINGPVIVGAGPSGLAVGAGLKQQGVPFVILERANCIASLWQNHTYDRLKLHLPKQFCQLPYFPFPDNFPEYPTKYQFLNYLESYARNFDINPQFNENVQSATYDETCGLWRVKTIGSTDNDFSRGEVEYICRWLVVATGENAEKVVPEFKGLEEFGGQVMHVCDYKSGETYRGKRVLVVGCGNSGMEVSLDLCHHNAIPSMVVRSSVHVLPREILGKSTFDIAVSMMKFLPLWVVDKILLAVARVVLGNIEKYGLKRPSKGPLQLKNTEGKTPVLDIGALQKIRSGEIKVVPGIKRFSPGRVELVNGENLEINSVILATGYCSNVPSWLKENEFFCRNGIPKSSFPNGWKGKAGLYAVGFTRRGLSGASLDAIKIANDIGRIWKEETKHKKHSVNVGCHRRCKSHC

>Soltu.DM.09G027910|Stuberosum_v6.1/1-431

MNQYCNSPCSPLMVHELEQEDLFAGRCILVNGPVIIGAGPSGLAVGAGLKQQGVPFVILDRANCIASLWQNRTYDRLKLHLPRQFCELPYFPFPQNFPEYPTKYQFISYLESYAKNFEISPRFNESVHSAKYDETCGLWRLKTVCRNGSVIEYICRWLVVATGENAEKVVPEFEGLEDFGGHIMHACDYKTGEVYEGKNVLVVGCGNSGMEVSLDLCHHNASPSMVVRSSVHVLPREILGKSTFELGVSMMKWLSIDVVDKILLVAARLLLGNIEKYGLKRPSIGPLQLKNTEGKTPVLDIGALQKIKTGDIKIVPAIKKFSQGKVEFVNGEIQEIDCIILATGYCSNVPSWLKESEFFSREGFPRSPFPNGWKGKAGLYAVGFTRRGLSGASLDAIKVSQDIGKIWKEEIKQKNQSVAVACHRRSKLPSF

>Solyc09G002635|Slycopersicum_ITAG5.0/1-431

MNQYCNSPCSPLMVHELEQEDLFAGRCILVNGPVIIGAGPSGLAVGAGLKQQGVPFVILDRANCIASLWQNRTYDRLKLHLPRQFCELRYFPFPQNFPEYPTKYQFISYLESYAKNFEISPRFNESVHSAKYDETCGLWRVKTVCRNGSVIEYICRWLVVATGENAERVVPEFEGLEDFGGHIMHACDYKTGEVYEGKNVLVVGCGNSGMEVSLDLCHHNASPFMVVRSSVHVLPREILGKSTFELGVSMMKWLSIDVVDKILLVAARLLLGNIEKYGLKRPSIGPLQLKNTEGKTPVLDIGALQKIKTGDIKIVPAIKKFSQGKVEFVNGEIQEIDCIILATGYCSNVPSWLKESEFFSREGFPRSPFPNGWKGKAGLYAVGFTKRGLSGASLDAIKVSQDIGKIWKEEIKQKNQSVAVACHRRNKLSSF

>evm.TU.supercontig_87.71|Cpapaya_ASGPBv0.4/1-425

MDALLRLVDHEDFFSKRCIWVNGPVIVGAGPSGLAAAACLREQGVPFEVLEKAECIASLWQKRTYDRLKLHLPKQFCQLPKMPFPEDFPEYPTKRQFIEYLESYARRFEIKPKFNECVQSARYDETSGLWRVKTVNASGSTRAEVEYICRWLVVATGENAEPVKPEELEGLAEFRGEVIHACEYKSGEKFKGQEVLVVGCGNSGMELSLDLCNHNASPSMVVRSSVHVLPREVFGKSVFELAVMLMKWFPLWLVDKLLLILAWLVLGNVEKYGLKKPSMGPMELKNTKGKTPVLDIGALEKIRSGEIKVVPGIKRFTRDQVEFVNGEKLYIDTVVLATGYRSNVPSWLQESEFFSKNGFPKASFPNGWKGNAGLYAVGFTRRGLSGASSDAMRIAQDIGKVWKDETKQHKKRTTACHRRCISQQF

>Cucsa.348750|Csativus_v1.0/1-423

MENLFRLADHDDFFSRRCIWVNGPVIVGAGPSGLATAACLREQGVPFVVLERAECIASLWQKRTYDRLKLHLPKQFCQLPKLPFPEDFPEYPTKRQFIEYLESYAKHFEINPQFNECVQSARYDETSGLWRVKTVSTAGSARNEVEYICRWLVVATGENAERVMPEIEGLSEFCGDVSHACEYKSGEKFTGKKVVVVGCGNSGMEVSLDLCNHNASPSMVVRSSVHVLPREIFGKSTFELAITMMKWLPLWLVDKLLLVLAWLILGNIEKYGLKRPSMGPLELKNEIGKTPVLDIGALEKIKSGDIKVVPGIKRFTRNQVELVNGQKLDVDSVVLATGYRSNVPSWLQEGEFFSKNGFPKAASPHSWKGNAGLYAVGFSRRGLSGASSDAMKIAQDIGNVWKAETKQQKKRTTACHRRCISQF

>Glyma.19G170800|Gmax_Wm82.a6.v1/1-424

MENLLRLVDHEDFFSRRCIWVNGPIIIGAGPSGLATAACLREQGVPFMVLERADCIASLWQKRTYDRLKLHLPKQFCQLPKLPFPEDFPEYPTKKQFIEYLESYAKHFEINPQFNECVQSARYDETSGLWRVKTVSSSGAAARGEIEYICRWLVVATGENAECVMPDIEGLSEFKGDVIHACDYKSGESFRGKKVLVVGCGNSGMELSLDLCNHHASPSMVVRSSVHVLPREVFGISTFELAVMLLQWLPLWLVDKILLILAWFVLGNIEKLGLKRPSMGPLELKNTKGKTPVLDIGALEKIRSGDIEVVPGIKRFNNGEVEFVNGEKLDIDAIVLATGYRSNVPSWLQEGEFFSKNGYPKMPFPHSWKGNAGLYAVGFTKRGLSGASSDAVKIAQDIGQVWKNETKQKKQRTTACHRRCISQF

>Glyma.03G169600|Gmax_Wm82.a6.v1/1-424

MENLFRLVDNEDLFSRRCIWVNGPIIVGAGPSGLATAACLREQGVPFMVLERADCIASLWQKRTYDRLKLHLPKQFCQLPKLPFPEDFPEYPTKKQFIEYLESYAKHFEINPQFNECVQSARYDETSGLWRVKTVSSSSGAARGEVEYICRWLVVATGENAECVMPEIEGLSEFKGDVIHACDYKSGERFRGKKVLVVGCGNSGMELSLDLCNHHSSPSMVVRSSVHVLPREVFGISTFELAVMLLQWLPLWLVDKILLILAWFVLGNIEKLGLKRPSKGPLEMKNRKGKTPVLDIGALERIRSGDIEVVPGIKRFNNGEVEFINGEKLDIDAIVLATGYRSNVPSWLQEGEFFSKNGYPKMPFPHGWKGNAGLYAVGFTKRGLSGASSDAVKIAQDIGQVWKNETKQKKQRTTACHRRCISQF

>KAI5426184.1|Pisum_sativum/1-416

MENLFRLADHEDFISRRCIWVNGPVIIGAGPSGLATAACLREQGVPFLVLERADCIASLWQRRTYDRLKLHLPKQFCQLPNLPFPDDFPEYPSKKQFINYLETYANKFEINPQFNQCVQSAKYDETSGLWRVKTNEVEYICRWLVVATGENAECVTPEIEGLSEFKGEVVHACDYKSGENFKGKKVLVVGCGNSGMELSLDLSNHHALPSMVVRSSVHVLPREIFGISTFELAVMMLKWLPLWIVDKLLLILTWFILGDMEKFGIKRPSMGPLQLKNTEGKTPVLNIGTLEKIRSGNIKVVPGIKKFNKNGEVELVNGEKIDVDAVVLATGYRSNVPSWLQEGEFFSKNGYPKMPFPHGWKGNSGLYAVGFTKRGLSGASYDAVKIAQDIGNVWKHETKQKKQRNTACHRRCISQF

>Potri.002G254200|Ptrichocarpa_v4.1/1-422

MENMFRLADHEDFFSRRCIWVNGPVIVGAGPSGLATAACLRDQGVPFAVLEKEDCIASLWQKRTYDRLKLHLPKQFCQLPKLPFPEDFPEYPTKKQFVEYLESYAKHFEINPKFNEYVQSARYDETSGLWRVKTVSTSGSNRTEVEYICRWLVVATGENAECVMPEIEGLAEFGGEVMHACQYKSGEKFSGKNVLVVGCGNSGMEVSLDLCNYNASPSMVVRSSVHVLPREIMGKSTFELAVLLMSWLPLWLVDKLMLIMAWLVLGNTDKYGLKRPSMGPLTLKNTMGKTPVLDIGALEKIRSGDINVVPGIKRFSRGQVELVNGEILDIDSVILATGYRSNVPYWLQEGEFFSKNGFPKAPFPNGWKGNAGLYAVGFTRKGLSGASSDAIRIAQDIGKVWKEETKQTKRATACHRRCISQF

>orange1.1g014522m.g|Csinensis_v1.1/1-423

MENLFRLVNHEDFLSRRCIWVNGPVIVGAGPSGLATAACLRDQGVPFVMLERAECIASLWQKRTYDRLNLHLPKQFCQLPKLQFPEDFPEYPTKRQFIQYLESYAEKFEINPRFNECVQSARYDETSGLWRVKTASSAGSTKTEFEYICRWLVVATGENAERVMPDIEGLADFGGEVIHACDYKSGEKYKGKKVLVVGCGNSGMELSLDLSNHNASPSMVVRSSVHVLPREILGKSTFELATLMMKWLPLWLVDKILLILAWFILGNTESYGLKRPSMGPLALKNTMGKTPVLDIGALKKIRSGHINVVPGIKRISCGQAELINGEKLDIDAIVLATGYRSNVPSWLQESEFFSENGFPKAPFPHGWKGNAGLYAVGFTRRGLSGASSDAMRIAQDIGKVWKEETKQQKKRTTACHRRCISQF

>Glyma.10G041800|Gmax_Wm82.a6.v1/1-423

MENLFRLVDYEDMFSKRCIWVNGPVIVGAGPSGLATAACLKQQGVPFMVLERAECIASLWQKRAYDRLKLHLPKQFCQLPNLPFPKDFPEYPTKKHFIDYLESYAQKFEINPRFNECVQCARYDETSGLWRVKTVATCGAAKSEFEYICRWLVVATGENAECVIPEIEGLGEFKGDVIHACEYKSGESFKGKKVVVVGCGNSGMELSLDLCNHNASPSMVVRSSVHVLPREVFGKSTFELAVLMLQWVPLWLVDKILLVLAWLVLGNMERFGLKRPSEGPLLLKNTKGKTPVLDIGTLEKIRSGDIKVVPEIKRFTNGCVEFVNGEKQHVDAVVLATGYRSNVPSWLQEGEFFSKNGFPKSPFPNGWKGNGGLYAVGFTRRGLSGASSDAMKIAQDIGQVWKQETRQKKQRTNACHRRCISQF

>Glyma.13G128800|Gmax_Wm82.a6.v1/1-423

MENLFRLVDYEDMFSKRCIWVNGPVIVGAGPSGLATAACLKQQGVPFMVLERAECIASLWQKRTYDRLKLHLPKQFCQLPNLPFPKDFPEYPTKKHFIDYLESYAQKFEINPRFNECVQCARYDETSGLWRVKTVATCGSAKSEFEYICRWLVVATGENAECVIPDIEGLGEFKGDVIHACEYKSGESFKGKKVVVVGCGNSGMELSLDLCNHNASPSMVVRSSVHVLPREVFGKSTFELAVLMLQWLPLWLVDKILLVLTWLVLGNMERFGLKRPSEGPLLLKNTKGKTPVLDIGTLEKIRSGDIKVVPEIKRFSNGYVEFVNGEKQGVDAVVLATGYRSNVPSWLQEGEFFSKNGFPKSPFPNGWKGNAGLYAVGFTRRGLSGASSDAMNIAQDIDQVWKQETRQKKQRTTACHRRCISQF

>Gohir.A11G134600|Ghirsutum_v3.1/1-424

MENLFRLADQDQGFFQRKWTLVNGPVIVGAGPSGLATAACLREQGVPFVVLERAECIASLWQKRTYDRLKLHLPKQYCQLPKMPFPEDFPEYPTKRQFIEYLESYAKHFDINPKFNECVQSARYDETSGFWRVKTVVMSGSNRTEFEYICRWLVVATGENAERVVPDIEGLAEFGGEIIHACDYKSGEKFQGKKVLVIGCGNSGMEVSLDLCNHNASPSMVVRSSVHVLPREIFGKSTYELAFFMLKWLPIWLVDKLMLILTWLVLGNVEKYGLKRPSIGPLELKNTQGKTPVLDIGALEKIKSGDINVVPGIKRFSRGEVELLDGKKLDIDSVILATGYRSNVPSWLQEGEFFSKNGFPKAPFPNGWKGNGGLYAVGFTRRGLSGASSDAISIAKDIGMVWKQETKQHKKRTIACHRRCISQF

>Gohir.D11G140800|Ghirsutum_v3.1/1-424

MENMFRLADQDQGFFQRKWTLVNGPVIVGAGPSGLATAACLREQGVPFVVLERAECIASLWQKRTYDRLKLHLPKQFCQLPKMPFPEDFPEYPTKRQFIEYLESYAKHFDINPKFNECVQSARYDETSGFWRVKTVVTSGSNRTEFEYICRWLVVATGENAERVVPDIEGLAEFGGEIIHACDYKSGEKFQGKKVLVVGSGNSGMEVSLDLCNHNASPSMVVRSSVHVLPREIFGKSTYELAFFMLKWLPIWLVDKLMLILTWLVLGNVEKYGLKRPSIGPLELKNTQGKTPVLDIGALEKIKSGDINVVPGIKRFSRGEVELLNGEKLDIDSVILATGYRSNVPSWLQEGEFFSKNGFPKAPFPNGWKGNGGLYAVGFTRRGLSGASSDAMSIAKDIGMVWKQETKQHKKRTIACHRRCISQF

>Gohir.D08G210500|Ghirsutum_v3.1/1-424

MENLFRLVDQDQGFFQRKWTLVNGPLIVGAGPSGLATAACLREQGVPSVVLERAECIASLWQKRAYDRLKLHLPKQFCQLPKVPFPEDFPEYPTKKQFIEYLESYAKRFDINPKFNECVQSARYDETSGFWRVKTVRGSNKAEFEYICRWLVVATGENAESVVPDIDGLADFGGEVIHASEYKSGEKFKGQNVLVVGCGNSGMEVSLDLANHNASPSMVVRSSVHVLPREVLGKSTFELAVLMMKWLPLWLVDMILLILAWLVLGNIEKYGLKRPLMGPLELKNSKGKTPVLDIGALEKIKSGDIQVVPGIKRFSRGRVELVNGEKLDVDSVVLATGYRSNVPAWLQEGEFFSKNGFPKAPFPNGWKGKGGLYAVGFTRRGLSGASSDAINIAQDIGKAWKQENKQPKKRTIACHRRCISLSQF

>Gohir.A08G192500|Ghirsutum_v3.1/1-424

MENLFRLVDQDQGFFQRKWTLVNGPLIVGAGPSGLATAACLREQGVPFVVLERAECIASLWQKRAYDRLKLHLPKQFCQLPKVPFPEDFPEYPTKKQFIEYLESYAKRFDINPKFNECVQSARYDETSGFWRVKTVRGSNKAEFEYICRWLVVATGENAESVVPDIDGLADFGGEVIHASEYKSGEKFKGQNVLVVGCGNSGMEVSLDLANHNASPSMVVRSSVHVLPREVLGKSTFELAVLMMKWLPLWLVDMILLILAWLVLGNIEKYGLKRPLMGPLELKNSKGKTPVLDIGALEKIKSGDIQVVPGIKRFSRGRLELVNGEKLDVNSVVLATGYRSNVPSWLQEGEFFSKNGFPKAPFPNGWKGKGGLYAVGFTRRGLSGASSDAINIAQDIGKAWKQENKQPKKRTIACHRRCISLSQF

>Gohir.D07G209200|Ghirsutum_v3.1/1-425

MDQSLFRLVDQEQDSFQRKWTLVNGPIIIGAGPSGLATAACLREQGVPFVVLERAECIASLWQKRTYDRLKLHLPKQFCQLPKLPFPEDFPEYPTRKQFIEYLESYAKHFDINPKFNECVQSARYDETSGFWRVKTIVTSGSNKIEFEYICRWLVVATGENAERVVPDIQGLAEFGGEVIHACEYKSGEKFQGQKVLVVGCGNSGMEVSLDLCNYNASPSMVVRSSVHVLPREVYGKSTFELVVLMMKWLPLWLVDKLMLVLAWLVLGNVEKYGLKRPSMGPLELKNTKGKTPVLDIGALKKIKSGDINVVPAIKRFSYRQVELVNGEKLDIDSVVLATGYRSNVPSWLQEGEFFGENGYPKAPFPHGWKGNGGLYAVGFTRRGLSGASSDAMRIAQDIGKLWKDETKQHKKITIAGHRQCISQF

>Gohir.A07G203000|Ghirsutum_v3.1/1-425

MDQSLFRLVDQELDSFQRKWTLVNGPVIIGAGPSGLATAACLREQGVPFVVLERAECIASLWQKRTYDRLKLHLPKQFCQLSKLPFPEDFPEYPTRKQFIEYLESYAKHFDINPKFNECVQSARYDETSGFWSVKTIVTSGSNKTEFEYICRWLVVATGENAERVVPDIQGLAEFGGEVIHACEYKSGEKFQGQKVLVVGCGNSGMEVSLDLCNYNASPSMVVRSSVHVLPKEVYGKSTFELAVLMMKWLPLWLVDKLMLILAWLVLGNVEKYGLKRPSMGPLELKNTKGKTPVLDIGALKKIKSGDINVVPGIKRFSYRQVELVNGEKLDIDSVVLATGYRSNVPSWLQEGEFFGENGYPKAPFPHGWKGHGGLYAVGFTRRGLSGASSDAMRIAQDIGKLWKDETKQHKKITIACHRQFISQF

>Gohir.D07G160100|Ghirsutum_v3.1/1-428

MTMENLFRLVDQDQDFFQRKWTLVNGPVIVGAGPSGLATAACLKEQGVPFVVLERADCIASLWQKRTYDRLKLHLPKQFCQLPKLPFPEDFPEYPTKRQFIDYLESYAKHFDINPKFNECVQSARYDETSGFWRVKTIVTSDSNKTEFEYVCRSLVVATGENAECVLPDIEGLSEFGGEVIHACDYKSGEKFKGQKVLVVGCGNSGMEVSLDLCNHNASPSMVVRSSVHVLPREIFGKSTFELAVLMMKWLPLWLVDKLMLILAWLVLGNIEKYGLKRPSTGPLELKNTKGKTPVLDIGALEKIRSGDINVVPGIKRFSRGQVELVNGEKLDIDSVVLATGYRSNVPSWLQEGEFFSKNGYPKASFPHEWKGKAGLYAAGFTRRGLSGASSDAMRIAQDIAQVFKDETKQLRMRTVARHRRCISLSQF

>Gohir.A07G153900|Ghirsutum_v3.1/1-428

MTMENLFRLVDQDQDFFQRKWTLVNGPVIVGAGPSGLATAACLKEQGVPFVVLERTDCIASLWQKRTYDRLKLHLPKQFCQLPKLPFPEDFPEYPTKRQFIDYLESYAKHFDINPKFNECVQSARYDETSGFWRVKTIVTSGSNKTEFEYVCRSLVVATGENAECVLPDIEGLSEFGGEVIHACDYKSGEKFKGQKVLVVGCGNSGMEVSFDLCNHNASPSMVVRSSVHVLPREIFGKSTFELAVLMMKWLPLWLVDKLMLILAWLMLGNIEKYGLKRPSTGPLELKNTKGKTPVLDIGALEKIRSGDINVVPGIKRFSRGQVELVNGEKLDIDSVVLATGYRSNVPSWLQEGEFFTKNGYPKASFPHGWKGKAGLYAAGFTRRGLSGASSDAMRIAQDIAQVFKDETKQLRMRTVARHRRCISLSQF

>Prupe.8G211000|Ppersica_v2.1/1-423

MEYMFRLVDYKDSFARRCIMVNGPVIVGAGPSGLATAACLRDQGVPFVVLERAECIASLWQKRTYDRLKLHLPKAFCQLPKLPFPEDFPEYPTKKQFIDYLESYAKHFEINPKFNSCVQSARYDETSGFWRVKTVTSTESTRSEVEYICRWLIVATGENAECVVPEFDGLSDFGGKVVHACEYKSGESFRGKKVLVVGCGNSGMELSLDLCNHNALPSMVVRSSVHVLPREMFGKSTFELAVLLLKWLPIWLADKLLLLFSWLILGSIEKYGLNRPSVGPMELKNMEGKTPVLDIGALDKIKSGGIKVVPGIKRFSSGQVELVNGERLDIDSVVLATGYRSNVPSWLQEGDFFSKNGFPKQPFPHGWKGNAGLYAVGFTRRGLSGASSDAMRIAQDIGKVWKGETKQIKKRTTACHRRCISQF

>FvH4_2g20150|Fvesca_v4.0.a2/1-424

MENLFRLVDHEDFFARRCIMVNGPVIVGAGPSGLATAACLRDQGVPFEVLERADCVASLWQKRTYDRLKLHLPKAFCQLPKLPFPEDFPEYPTKRQFIDYLESYAKHFEINPKFNSCVQSARYDETSGFWRVNTVSTTGSTRSEVEYICRWLIVATGENAECVVPDIDGLCEFGGEVVHASEYKSGEKYKGKKVLVVGCGNSGMELSLDLCNHNASPSIVVRSSVHVLPREIYGKSTFELAVFLLKWLPVWLADKLLLLFSWLILGSIEKYGLNRPSVGPMELKNTQGKTPVLDIGALDKIKSGGIKVVHGIKRFSYGRVELVGGEVLDIDAVVLATGYRSNVPSWLQEGDFFSKNGFPKQPFPQGWKGNAGLYAVGFTRRGLSGASCDAVRIAQDIGNVWKCETKQNKKRTTAGHRRCISQTF

>GWHPBAUK019847.1|Macadamia_integrifolia/1-420

MLCVSDHKDFFARRTVWVNGPVIVGAGPSGLAVGACLKEQGVPFVVLERADCIASLWQKRTYDRLKLHLPKQFCQLPKLPFPKDFPEYPTKNQFINYLESYAKKFEINPRFNECVQSAKYDETSGLWRVKTVSTSHSSGRNEVEYICRWLVVATGENAECVMPEIEGMSEFGGEIVHACHYKSGEDYRGKRVLVVGCGNSGMEVSLDLCNHNALPSMVVRSSVHVLPREFLGKSTFGLAAMMMKRFPLWLADKMLLIMAWLVLGNIEKYGLKRPAMGPLELKTAQGKTPVLDIGALEKIKSGDIKVVPGIKRFSYGGVELVNGQNIEVDAVVFATGYRSNVPSWLQDNEFFSKNGFPKAPFPNGWKGKAGLYAVGFTKRGLSGASFDAMRIAQDIGKAWKEETKQTKKTIACHRRCSSQF

>GWHPBAUK036404.1|Macadamia_integrifolia/1-420

MFCLADHEDIFARRNVWVNGPVIVGAGPSGLAVGACLKEQGVPFVVLERADCIASLWQKRTYDRLKLHLPKSFCQLPKLPFPEDFPEYPTKKQFIHYLELYAKKFEINPRFNECVQSAKYDETSGLWRVKTVSTSPISGRIEVEYICRWLVVATGENAECVVPEIEGLSEFGGDIVHVCNYKSGEDYRGKKVLVVGCGNSGMEVSLDLCNHNAQPSMVIRSSVHVLPREILGKSTFELAVLLLKWLPLWLVDKLLLIMAWFVFGNIEKYGLKRPEMGPLLLKNTEGKTPVLDVGALQKIKSGDIKVVPGIKRFSSGRVELVNGENLEIDSVVLATGYRSNVPSWLEESEFFSKNGFPKASFPNGWKGNTGLYAVGFTRRGLSGASSDAMRIAQDIGKVWKEETKQTKKRIACHRRCTSQF

>TESI_003198-RA|Tetracentron_sinense/1-422

NSTMFRISDHDDFFARRCVWVNGPVIVGAGPSGLAVAACLKDQGVPFVVLERANCIASLWQKRTYNRLKLHLPKQFCQLPKLPFPEDFPEYPTKKQFIDYLESYANHFDINPQFNECVQSAKYDETCGLWRVKTVSTSGTSRAEVEYICRWLVVATGENAECVVPEIEGLSEFGGHVMHACEYKSGEDYRGKRVLVVGCGNSGMEVSLDLSNHNALPSVVVRSSVHVLPREILGKSTFELGVLMMKWLPLWLVDKLLLIMAWLLLGNVEKFGLKRPSIGPLELKNTEGKSPVLDIGALQKIKSGEIKVVPGIKRFSCGRVELVNGENLEIDSVVLATGYRSNVPYWLQETEFFSKNGFPKAPFPNGWKGNAGLYAVGFTRRGLSGASSDAMRIAQDICKVWKEETKQKKQSIACHRRCISQF

>TESI_005202-RA|Tetracentron_sinense/1-416

MFRVLDHEEFFARRCVWVNGPVIVGAGPSGLAVGACLKEQGVPFVVLERADCIASLWQKRTYDRLKLHLPKQFCQLPNLPFPEDFPEYPTKKQFIDYLESYAKQFEINPRFNECVQSAKYDETCGLWRVKTVSSSGTSRAEVEYICRWLVVATGENAESVVPEVEGLADFGGQVMHVCDYKSGEDFRAKRVLVVGCGNSGMEVSLDLCNYNALPSMVVRSSVHVLPREILGKSAFELAITMMKWLPLWLVDKLLLIIARLVLGNIEKYGLKRPSMGPLELKNSEGKTPVLDIGALKKIRYGEIKVVPGIKKFSRGRVELVNGEILEIDSIILATGYRSNVPSWLQETEFFSQNGFPKAPFPNGWKGNAGLYAVGFTRRGLSGASFDAMRIAQDIGEVWKKETKQSIACHRRCISQF

>Eucgr.K03581|Egrandis_v2.0/1-423

MENLFRLVDHEDFFARRCVWVSGPVIIGAGPSGLATAACLREQGVPFVVLERADCIASLWQKRTYDRLKLHLPKQFCQLPKLPFPEDFPEYPNRKQFIDYLESYATRFEISPNFNECVQSARYDETSGLWRIRTVQAKGSSRSEIEYICQWLVVATGEHAERVVPDIEGLAEFKGEVIHACDYKSGEKFAGKKVLVVGCGNSGMEVSLDLCNRNAKPSMVARSTVHVLPREVMGKSTFELAVLLMKWLPLWLVDKILLILTWLVLGDTGKYGLKRPSMGPMELKNERGKTPVLDIGALEKIRSGEIKIVPGIKRFSRGHVVELENGDELDVDSVVLATGYKSNVPSWLQESEFFTKNGYPKSQFPNGWKGNAGIYAVGFTRRGLSGVSSDAMRIAQDIGKVWKVETRQKQRTTACHRRCISQF

>VIT_207s0005g04800|Vvinifera_v2.1/1-424

MEKTLRISDHDDFFSRRCVWVNGPVIVGAGPSGLAVGACLKEQGVPFVVLERAECIASLWQKRTYDRLKLHLPKQFCQLPKMPFPEAFPEYPTKKQFIEYLESYAKRFEVNPRFNECVQSAKYDETCGLWRVRTVSTNAAAGAHSEVEYICRWLVVATGENAERVVPDIEGLGAFGGNVMHACEYKSGETFRGKRVLVVGCGNSGMEVSLDLCNHNATPAMVVRSSVHVLPREVFRKSIFELATLMIKWLPLWLVDKLMLILAWLVLGDVEKYGLKRPSMGPLELKNTQGKTPVLDIGALEKIRSGDIKVVPGIKRFFPDSVELVNGEKLDIDSVVLATGYRSNVPFWLKESQFFCKNGFPKASFPNGWKGKAGLYAVGFSRRGLSGASLDAIRIAQDIGKVWKEETAQKPKTTACHRRCISQF

>evm.TU.Scaffold_2016.447|Carabica_v0.5/1-418

MFNFMDPDFLARRCVWVNGPVIVGAGPSGLAVGACLKEQGIPFVILEKTDCIASLWQKRTYDRLKLHLPKQFCQLPKFPFPDHYPEYPNKKQFIDYLESYATHFDINPQFNECVQSAKYDEACRLWRVKTVSTNGSTRSDVEYFCQWLVVATGENAERMVPDIDGLKEFGGEVLHACDYKSGEKFRGKKVLVVGCGNSGMEVSLDLCNHEAQPSMVVRSSVHVLPREICGRPTFELAMFMLKWLPLWLVDKLLLVLAWFILGNIEKYGLKRPSLGPLQLKNTKGKTPVLDIGALEKIRSGEINVVPGIRRFACGMVELVDGEMLNIDAVVLATGYSSNVPYWLQEAEFFSRDGFPKSPFPNGWKGKDGLYAVGFTRRGLSGASADATKIAQDIGKVWKEDLKQKKQKVPTHRRCISQF

>evm.TU.Scaffold_635.212|Carabica_v0.5/1-418

MFNFMDPDFLARRCVWVNGPVIVGAGPSGLAVGACLKEQGIPFVILEKTDCIASLWQKRTYDRLKLHLPKQFCQLPKFPFPDHYPEYPNKKQFIDYLESYATHFDINPQFNECVQSAKYDEACRLWRVKTVSTNGSTRSEVEYFCQWLVVATGENAERMVPDIDGLKEFGGEVLHACDYKSGEKFRGKKVLVVGCGNSGMEVSLDLCNHEAQPSMVVRSSVHVLPREICGRSTFELAMFMLKWLPLWLVDKLLLVLAWFILGNIEKYGLKRPSLGPLQLKNTKGKTPVLDIGALEKIRSGEINVVPGIRRFSCDMVELVDGEMLNIDAVVLATGYSSNVPYWLQEAEFFSKDGFPKSSFPNGWKGKDGLYAVGFTRRGLSGASADATKIAQDIGKVWKEDLKQKKQKVPTHRRCISQF

>Hyque.04G157000|Hquercifolia_v1.1/1-419

MFSVSDYNDLFSRRCVWVNGPVIVGAGPSGLAVGACLKEQGVPFVVLERADCIASLWQQRTYDRLKLHLPKQFCQLPNLPFPEHFPEYPSKKQFIDYLESYANHFEINPQFNECVESAKYDQACKLWRVKTVSSSGSTRTEVEYICQWLVVATGENAERLVPEIDGLNEFGGEVLHACDYKSGEKFSGKKVLVVGCGNSGMEVSLDLCNHNAKPSMVVRSSVHVLPREIFGKSTFELAIMMMKWLPLWLVDKVMLIMAWFILGNIENYGLKRPAMGPLELKNTQGKTPVLDIGALERIRSGDIKVVPGIKKFSCGMVELANGEKLEIDSVVLATGYRSNVPYWLQETDFFCKNGFPKAPFPNGWKGKAGLYAVGFTRRGLSGASSDAMRIAQDIGKVWKEDLKQKKQKVPCHRRCISQF

>DCAR_521208|Dcarota_subsp_sativus_v3.0/1-419

MFTFTDQNDFLDRRCVWVNGPVIVGAGPSGLAVGACLKEQGIPFVVLERADCIASLWQKRTYDRLKLHLPKQFCQLPNFPFPEHFPEYPSKKQFIDYLESYARHFDINPQFNECVQTAKYDEACQLWRIKTVSTCGSVQSEVEYICQWLVVATGENAERVVPDIEGLQEFGGEVIHACEYKSGEKYSGKKVLVVGCGNSGMEVSLDLCNHNAKPSMVVRSSVHVLPREICGKSTFELAMIMMRWLPLWLVDKLMLVLAWFILGNTESYGLKRPSVGPLELKNTRGKTPVLDIGALAKIRSRDINVVPGIKRFSKGLVELLDGEKLEIDSVVLATGYCSNVPYWLQETDFFSKNGLPKTPFPNGWKGNAGLYAVGFTRRGLSGASLDATRIAQDISKVWKEDLTHKKQKVPTHRRCISTF

>DCAR_314301|Dcarota_subsp_sativus_v3.0/1-419

MFTAMDQTDFLARRCVWVNGPVIVGAGPSGLAVAACLKDQGIPCVVLERADCIASLWQRRTYDRLKLHLPKQFCQLPKLPFPDHYPEYPSKKQFIDYLESYARKFDIRPQFNESVLSAKYDKACRLWRIKSVSTCGSVSSEVEYICQWLVVATGENAERVVPEIEGLDEFKGEVIHACEYKSGEKYSGKKVLVVGCGNSGMEVSLDLCNHNAKPSMVVRSSVHVLPREICGKSTFELAMVLMRWLPLWLVDKLVLLLSWFILGNTESYGIKRPSIGPLELKNIHGKTPVLDIGALEKIRSRDIDVVPGIKKFSKGLVQLVNGQELEIDSVVLATGYCSNVPYWLQETDFFSKNGLPKTPFPNGWKGNAGLYAVGFTRRGLSGASLDATRIAQDISQVWKEDLKQKKQKVPTLRRCISTF

>DCAR_105111|Dcarota_subsp_sativus_v3.0/1-419

MFSFSDQNDLFARRCVWVNGPVIVGAGPSGLAVGACLKEQGIPCVVLERADCIASLWQKRTYDRLKLHLPKQFCQLPKLPFPQHFPEYPTKKQFIDYLESYANHFGIKPQFNESVESAKYDEACRLWRIKTVSVQGSVRSEIEYICQWIVVATGENAERVVPEIDGLGDFHGEIIHACEYKSGEKYSGKKVLVVGCGNSGMEVSLDLVNHNAKPSMVSRSSVHVLPREIFGRSTFELAMMMMKWLPVWIVDKILLLLTWFILGNTESYGLKRPSIGPLELKNTYGKTPVLDVGALEKIRAGDIEVVPGIKKFSKNMVELVNGEELAIDSVVLATGYCSNVPIWLKEAEFFSKAGYPKTPFPNGWKGNTGLYAVGFTKRGLSGASSDAMKIAQDISKVWKEDLKQKKQKVPTHRRCISTF

>Soltu.DM.09G018700|Stuberosum_v6.1/1-415

MFNFSDNDMFARRCVWVNGPVIVGAGPSGLAVGACLKEQGVPCVILERSDCIASLWQKRTYDRLKLHLPKQFCQLPKFPFPEHYPEYPSKRQFIEYLESYARKFDLSPQFNECVQSAKYDEFCRLWRVKTVSADGVEVEYICQWLVVATGENAERVVPEIDGLKEFGGEVIHACDYKSGEKFRGKKVVVVGCGNSGMEVSLDLCNHDAQPSMVCRSAVHILPREIFGKSTFELAMLLMTWLPLWLVDKILLILTWFILGDIEKYGLKRPTTGPLQLKNSQGKTPVLDIGALEKIRAGKVKVVAGIKKFSCGTVELVTGEKLEVDSVVLATGYCSNVPSWLQESEFFSKNGYPKAQFPNNWKGKSGLYAVGFTKRGLAGASADAIRIAKDIGKVHKEDLKQKKQKVPTHRRCISTF

>Solyc09G001845|Slycopersicum_ITAG5.0/1-415

MFSFSDNDMFSRRCVWVNGPVIVGAGPSGLAVGACLKEEGVPCVILERSDCIASLWQKRTYDRLKLHLPKQFCQLPKFPFPEHYPEYPSKRQFIEYLESYAKQFDLTPQFNECVQSAKYDEFCRLWRVKTVSADGAEVEYICQWLVVATGENAERVVPEIDGLKEFGGEVVHACDYKSGEKFRGKKVVVVGCGNSGMEVSLDLCNHDAQPSMVCRSAVHVLPREIFGKSTFELAMLLMTWLPLWLVDKILLILTWFILGDIEKYGLKRPSIGPLQLKNSQGKTPVLDIGALEKIRAGKVKVIPGIKKFSCGTVELVTGEKLEVDSVVLATGYCSNVPYWLQETEFFSKNGYPKAQFPNNWKGKSGLYAVGFTKRGLAGASADAIRIAKDIGKVHKEDLKQKKQKVPTHRRCISTF

>Soltu.DM.06G034100|Stuberosum_v6.1/1-416

MFTFSSEQDSVSRRCVWVNGPVIVGAGPSGLAVGACLREQGVPFVVIERSDCIASLWQKRTYDRLKLHLPKKFCQLPKFPFPNHFPEYPTKRQFIEYLELYAKHFDINPQFNECVQSAKYDETCRVWRVKTSSPNGFEVEYISQWLVVATGENAERVVPDIEGLKEFGGEVIHACDYKSGEKYQGKKVVVVGCGNSGMEVSLDLSNHGAQPSIVCRSSVHVLPREIFGKSTFELAMFMMKWVPLWLVDKILLILAWFILGNIENYGLKRPSIGPLELKNKHGKTPVLDIGALEKIRSGKVKVVPGIKKFSCGTVELVTGEKLEIDSVVLATGYCSNVPYWLQESEFFSNNGFPKAQFPNNWKGKSGLYAIGFTRKGLAGASADAINIAQDIGKVYKEDLKQKKQKVPTHRRCISTF

>Solyc06G002693|Slycopersicum_ITAG5.0/1-416

MFTFSSEQDSVSRRCVWVNGPVIVGAGPSGLAVGACLREQGVPFVVIERSDCIASLWQKRTYDRLKLHLPKKFCQLPKFPFPNHFPEYPTKRQFIEYLELYAKHFDINPQFNECVQSAKYDETCGVWRVKTSSPNGFEVEYISQWLVVATGENAERVVPEIEGLKEFGGEVIHACDYKSGKNYKGKKVVVVGCGNSGMEVSLDLSNHGAQPSIVCRSSVHVLPREICGKSTFELAMLMMKWLPLWLVDKILLILAWFILGNIENYGLKRPSIGPLELKNKHGKTPVLDIGALEKIRSGKVKVVPGIKKFSCGTVELVTGEKLEIDSVVLATGYCSNVPYWLQESEFFSNNGFPKAQIPNNWKGKSGLYAIGFTRKGLAGASADAINIAQDIGKVYKEDLKQKKQKVPTHRRCISTF

>Soltu.DM.06G002120|Stuberosum_v6.1/1-408

MVSFNGQDLFSSRCVWVNGPVIVGAGPSGLAVGACLKEQGIPFVILEKSDCIASLWQKKTYNRLKLHLPKQFCQLPKFPFPQHYPEYPTKKQFIDYLESYARKFDINPMFNECVQFAKYDQICKLWRVKTISPNGLEVEYICQWLVVATGENAEKVVPNIEGLKEFGGEVIHACDYKSGEKFSGKKVLVVGCGNSGMEVSLDLSNHNAHPSLVCRSSVHVLPREIFGKSIFELAMFMMKWLPLWLVDKILLILTWFILGNIEKYGLKRPKIGPLELKNTQGKTPVLDIGALEKIRSRKINVVPGIKKFSCGTVELVNGEKLEIDSVVLATGYCSNVPFWLKESEFFSKNGFPKAPFPNSWKGKSGLYAVGFTRRGLSGASADAIQIAQDITKAYNEDIMQKKQKISTL

>Solyc06G000189|Slycopersicum_ITAG5.0/1-411

MASFNDHDLFSSTSRCVWVNGPVIVGAGPSGLAVGACLKEQGIPFVILEKSDCIASLWQKKTYNRLKLHLPKQFCQLPKFPFPQHYPEYPTKKQFIDYLESYAKRFDINPMFNECVQLAKYDKICKLWRVKTISPNGLEVEYICQWLVVATGENAEKVVPNIEGLKEFGGEVIHACDYKSGEKFSGKKVVVVGCGNSGMEVSLDLCNHNAQTSLVCRSSVHVLPREIFGKSIFELAMLMMKWLPLWLVDKILLILTWFILGNIEKYGLKRPKIGPLELKNTQGKTPVLDIGALEKIRSRKINVVPGIKRFSCGTVELVNGEKLEIDSVLLATGYCSNVPFWLKESEFFSKNGFPKAPFPNSWKGKSGLYAVGFTRRGLSGASADAIKIAQDITIAYNEDIMQKKAKKISTL

>Rhdel13G0046700.1|Rhododendron_delavayi/1-419

MCNFSGRDDFFQRRCVLVHGPVIIGAGPSGLAVGACLREQGVPFVVLEKSDCIASLWQKRTYDRLKLHLPKQFCQLPNLPFPEDFPEYPTKKQFISYLETYANRFGIKPQFNECVQSARYDEACRSWRVKTVSTSGCCRTEVEYICQWLVVATGENAECVKPEIDGLKEFKGEVLHACEYKSGEKFAGKRVLVVGCGNSGMEVSLDLSNHNAKPSMVVRNSTHVLPREVFGKSTFEFAMLMMKWLPLWLVDKILLILAWFILGNTEKWGLKRPSVGPLELKNTQGKTPVLDVGALQKIRSGDIKVVPGIKRFSSGAVELVNGEKIEIDSVVLATGYRSNVPYWLQESEFFSENGFPKAPFPNGWKGKAGLYAVGFTRKGLSGASFDAKRIAQDIGNVYKTDLNQKKRKVSGHRRCISQF

>CKAN_01805100|Ckanehirae_v3/1-423

MVFAPDHNDFSARRCVWVNGPVIVGAGPSGLAVAACLKEQGVPFVILERADCIASLWQRRTYNRLKLHLPKQFCQLPKLPFPVDFPEYPSKKQFIDYLESYAQHFEINPQFNECVQSAKYDETCGLWRVKTCSTKGAVARGAASEVEYICRWLVVATGENAECVVPDIEGLNEFGGKMLHACDYKSGEGFKGKRVLVVGCGNSGMEVSLDLCNHNSFPSMVVRSSVHVLPREIFGKSTFETAVFLMKLLPLWLVDKLLLILAWLVLGNTESYGLKRPSTGPMQQKNTQGKTPVLDIGALEKIKSGDIKVVPGIKRFSHDRVELVDGENLYVDSVILATGYRSNVPSWLQESDFFTKNGFPKQPFPNGWKGKAGLYAVGFTRRGLSGASVDAMRIANDIGQVWKEETKPTKRFTACHRRCISQF

>CKAN_02673000|Ckanehirae_v3/1-422

MEIGSDHKDFFSRRCVWVNGPIIVGAGPSGLAVGACLKEQGVPFIVLERADCIASLWKNRTYDRLKLHLPKQFCQLPKLPFPDDYPEYPTKNQFIDYLESYAQHFEINPRFNECVQSAKYDETCGLWRVRSFSTKGSGVKGGSEVEYICRWLVVATGENAERVIPEIEGLAEFMGQVIHACDYKSGKDFKGKQVLVVGCGNSGMEVCLDLCNHSAIPYMVVRSSVHVLPREVFGKSTFETAVFLMKWLPLWLVDKLLLFLAWLVLGGTEKYGLKRPSVGPFELKNTQGKTPVLDIGALEKIKSGEINVVPGIKRISLGRVELDDGQNLEVDSIVLATGYRSNVPSWLQGSDFFSKNGFPKQPFPNGWKGKSGLYAAGFTRRGLSGASLDAMRIAQDIGTVWKEETILTKQFIACHRRCISQF

>CKAN_02545400|Ckanehirae_v3/1-421

MVMGSEHEEFFSSRCMWVNGPIIVGAGPSGLAVGACLKEQGVPFIMLERADCIASLWQKRTYDRLKLHLPKQFCQLPKLPFPDEFPEYPTKNQFINYLESYAKHFKISPRFNECVQSAKFDETSGLWRVKSICTKGGTKGLGEVEYICRWLVVATGENAERVVPEIEGRAEFMGQVIHACDYKSGEDFRGKQVLVVGCGNSGMEVSLDLCNHNAFPSMVVRSSVHVLPREICGKSTFETAVFLMKWLPIWLVDKLLLVLAWLVLGSTEKYGLKRPSVGPLELKNAQGKTPVLDIGALNKIKSGEIKVVPGVKKFSSGSVELVDGQNLEVDSVILATGYRSNVPSWLKETDFFSKNGFPKQPFPNGWKGKSGLYAVGFTRRGLAGASVDATRIAEDIGRVWKEETKQTKQFIACHRRCTSQF

>Litul.08G085500|Ltulipifera_YP108A_v1.1/1-420

MVSASDHEDFLTRRCVWVNGPIIVGAGPSGLAVGACLKEHGVPFIILEKEDCIASLWQKRSYNRLKLHLPKQFCQLPKLPFPEDFPKYPSKKQFIDYLESYAQHFEINPRFNECVQSAKYDETCGLWRVKSFCMREGARGAEVEYICRWLVVATGENAVRAVPEIEGLSEFGGRVMHVCDYKSGENFKGEQVLVVGCGNSGMEVSLDLCNYNAFPSMVIRSSVHVLPREVLGKSTFELAVSLMKWLPLWLVDKLLLVLAWLVLGNTEKYGLKRPSIGPMQLKNAQGKTPVLDVGALRKIKSGKIKVVPGIKRFSPGKVELVSGQNLEVDSVIFATGYRSNVPSWLQESDFFSRNGFPNQPFPNGWKGNAGLYAVGFTRRGLSGASVDAVRIAEDIGRVWKEETKQAKQFIACHRRCISQF

>Litul.03G153400|Ltulipifera_YP108A_v1.1/1-416

MFNSSGHNDLFSRRCVWVNGPIIVGAGPSGLATGACLKEQGVPFIILEKADCIASLWQKRTYDRLKLHLPKQFCQLPKLPFPEEFPEYPTKNQFIEYLESYAKHFDIEPQFNECVQSAKYEETCGWWRVRSSGTAGSGEVEYICRWLVVATGENAECVVPEIEGLGEFQGQVMHACHYKSGEDFKGKQVLVVGCGNSGMEVSLDLCNHNAFPSMVVRSSVHVLPREILGKSTFEMAVLLMKWLPLWWVDKLLLFLAWLMLGNTAKYGLKRPSAGPLQLKNTEGKTPVLDIGALEKIRSGEIKVVPGIKSFSNGGVELVNGQKLEVDSVILATGYRSNVPSWLQENDFFSKQGFPRQPFPNGWKGNSGLYAVGFTRRGLSGASIDAMRIAQDIGKVWKEETKQTKQFASHRRCISMF

>GWHPCBIR011954|Santalum_yasi/1-420

MDKMFGICDQEGLFSRRCVWVNGPVVIGAGPSGLAVGACLRQQGVPFVVLERADCIASLWQSRTYDRLKLHLPKRFCQLPNLPFPESFPEYPTKKQFIDYLESYASRFDIAPQFNECVQSAKYDEACGLWRVRTLSGSTRSEIEYICQWLVVATGENAEPVVPEIEGLKEFQGEVVRACDYKSGEKFQGRNVLVVGCGNSGMEISLDLCNHNANPSMVVRSSVHVLPREVMGKSTFELASFMMKWLPIWMVDKILLILAWLVFGNVENFGLTRPSLGPLELKNTQGKTPVLDIGALAKIRSGDIQVVPGIKKFSRHGVEFINGKKMDIDSVVLATGYKSNVPSWLREDEFFSKNGFPKALFPNGWKGKTGLYAVGFTRKGLSGASSEAMSIAQDVGKVWRDDIKQKKQTVPRHRRCISQF

>GWHPCBIR013400|Santalum_yasi/1-419

MDKMYDISDQGGLFSGRCVWVNGPVVVGAGPSGLAVAACLRQQGVPFVVLERANCIASLWKNRTYDRLKLHLPKRFCELPNLKFPESFPEYPTKNQFIDYLESYANKFEIFPQFNQCVRSAKYDEAWRLWRVRSCSGAGVEVEYICQWLIVATGENAERVVPEIEGLKEFDGEVVHACDYKSGEKFQGKNVLVVGCGNSGMEVSLDLCNHNANPTMVVRSSVHVLPREVMGKSTFELAVYLMKWLPIWLVDKILLILAWLILGNIKKFGLNRPSIGPLQLKNSQGKTPVLDIGALEKIRSGEIKVVPGIKKFSTRGVELVNGEKLDLDSVVLATGYRSNVPSWLKEDGFFSGNGFPKAPFPNGWKGKSGLYAVGFTRRGLSGASSDAMSIAQDIGKVSRDDIKQKKRTVARHRRCISQF

>KAI5395306.1|Pisum_sativum/1-426

MENLFRLVDNQQDSISPRCIWVNGPIIVGAGPSGLATAACLKQQGVPFMILERSNCIASLWQNRTYDRLKLHLPKQFCQLPNFPFPQDFPEYPSKKQFINYLESYAKHFEINPQFDECVSSAKYNENTGLWRVKTKTKTKTVSNQNEIEYICRWLVVATGENAERVVPDFEGLSEFNGEVIHACDYKSGEGFRGKKVLVVGCGNSGMELALDLCNHNASPSIVLRSSVHVLPREMLGRSTFELAVMMMKWLPVWFVDKILLMLAWLVFGNIEKYGIKRPLEGPLELKNTKGKTPVLDIGTLEKIRSGNIKVVPGIKRFCDGFVELVNGEKNEVDAVVLATGYRSNVPFWLKEGEFFSKNGFPKSPFPNGWKGNVGLYAVGFTKRGLSGSSYDAMKIAQDIGQVWKQETKQKKQRISACYRRCISQF

>CKAN_01519000|Ckanehirae_v3/1-422

MALPSDHDDFFTPRCIWVNGPVIVGAGPSGLAVAACLKEQSVPFVILEKADCIATLWQRRTYDRLKLHLPKQFCQLPNYPFPEDYPEYPSKKQFIDYLESYAQHFDIKPRFNECVQSAKYDETCGLWCVKSCSIKGVERGVNSEVEYISQWLVVATGENAESVVPEIEGLNEFGGRVIHACDYKSGKDFKGKRVLVVGCGNSGMEVSLDLCNHNAFPFMVVRSSVHVLPREVFGKSTFRIATFLMKWLPLWLVDKLLLILTWLVLGNTERFGLRRPSMGPLHFKNTNGKTPVLDIGALQKIKTGGIQVVPGIKRFSHGRVELVDGNHLYFDSVILATGYRSNVPSWLQESDLFSNNGLPNQPFPNGWKGKSGLYAAGFTRKGLSGASLDATRIADDIGQVWKEETKLKKRFIACNGRFISQF

>Kalax.1550s0003|Klaxiflora_v1.1/1-419

MENLFRISDHEDLSTRRCVWVNGPVIVGAGPSGLAVGACLKEQGVPFVVLERADCIASLWQKRTYDRLKLHLPKQFCQLPKLPFPDHFPEYPTKRQFIDYLESYARQFDITPHFNECVQSAKYDETCGLWRVRTISSARSEVEYICRWLVVATGENAEAVVPEIDGLAGFDGQVLHACEYKSGESYTRKRVLVVGCGNSGMEVSLDLCNHKALPSMVVRSSVHILPREFLGKSIFELAMLMMAWLPLWLVDKLLLVMAWLTLGSLEKVGINRPSMGPLELKNKAGKTPVLDIGALAKIRAGGISVVPGIKKFSRNGVQLVNGESLELDAVVLATGYKSNVPFWLKEKEFFAENGMPNAPFPHAWKGKSGLYAVGFTRRGLSGASSDAISIASDIGKVWKQETRQIKKTVARHRRCISTF

>Kalax.0045s0091|Klaxiflora_v1.1/1-419

MENLFRISDHEDLSTRRCVWVNGPVIVGAGPSGLAVGACLKEQGVPFVVLERADCIASLWQKRTYDRLKLHLPKQFCQLPKLPFPDHFPEYPTKRQFIDYLESYARQFDISPHFNECVQSAKYDETCGLWRVRTISSARSEVEYICRWLVVATGENAEAVVPEIDGLAGFDGQVLHACEYKSGESYTGKRVLVVGCGNSGMEVSLDLCNHKALPSMVVRSSVHILPREFLGKSIFELAMLMMAWLPLWLVDKLLLIMAWLTLGSLEKVGINRPSMGPLELKNKAGKTPVLDIGALAKIRAGGISVVPGIKKFSRNGVELVNGECLELDAVVLATGYKSNVPCWLKEKEFFAENGMPNAPFPHAWKGKSGLYAVGFTRRGLSGASSDAISIASDIGKVWKQETRQIKKTVARHRRCISTF

>Pso10G12300.0|Papaver_somniferum/1-425

MFRVVSDHEDFSSKRCVWVNGPVIVGAGPSGLAVGACLKEQGVPFVVLERADCIASLWQKRTYDRLKLHLPKKFCQLPNLAFPEDYPEYPSKKQFISYLENYAKKFDITPQFNESVQSAKYDETCGLWRVKTVSTDPFGTGNRAEVEYICRWLVVATGENAENVVPEIEGLSHEFAGEIVHACDYKSGEDYRGKRVLVVGCGNSGMELSLDLCNHNAFPSMVVRSSVHILPREILGISTYELAGMLMAWLPLWVVDILILIMAWLKFGSMEKYGLKRPAMGPLTLKGAIGKTPVLDIGALERIKSGDIEVVPAIHKFSKSSVQLVNGEILDIDAVILATGYRSNVPSWLQESEFFSKNGFPKPSFPNAWKGQSGLYAVGFTRRGLSGASFDATKIAQDIGQVWKEETKQKKTTKAACHRRCISQF

>Pso02G13730.0|Papaver_somniferum/1-425

MFRVVSDHEDFSSKRCVWVNGPVIVGAGPSGLAVGACLKEQGVPFVVLERADCIASLWQKRTYDRLKLHLPKKFCQLPNLPFPEDYPEYPSKKQFISYLENYAKKFDITPQFNESVQSAKYDETCGLWRVKTVSTDPFGTGNRAEVEYICRWLVVATGENAENVVPEIEGLSHEFAGEIVHACDYKSGEDYHGKRVLVVGCGNSGMELSLDLCNHNAFPSMVVRSSVHILPREILGISTYELAAMLMAWLPLWVVDILILIMAWLKFGSMEKYGLKRPAMGPLTLKGAIGKTPVLDIGALERIKSGDIEVVPAIHKFSKSSVQLVNGEILDIDAVILATGYRSNVPTWLQESEFFSKNGFPKASFPNAWKGQSGLYAVGFTRRGLSGASFDATKIAQDIGQVWKEETKQKKTTKAACHRRCISQF

>Aqcoe5G009000|Acoerulea_v3.1/1-420

MFHVSENDDSSSRRCVWVNGPVIVGAGPSGLAVGACLKEQGVPFVVLERADCIASLWQKRTYDRLKLHLPKQFCQLPKMPFPEEYPEYPTKKQFITYLENYANHFDIKPRFNECVQSAKYDETCGLWRVKTVSTSGTTRTEVEYIGRWLVVATGENAESVMPEIEGLSEFKGHITHASEYKSGEDYQGKRVLVVGCGNSGMEVCLDLCNHNAIPSMVVRSSVHVLPREFLGKSTFELAMTLMRWLPLWLVDKFMLLMAWLVLGNMEKYGIKRPDMGPLALKNMAGKTPVLDIGALKKIKSGDITVVPGIKRFFNHSVELVNGEIIDVDAVVLATGYRSNVPSWLKENDFFSNNGYPKAQFPHGWKGQSGLYAVGFTKRGLSGASADATRISQDMSKVWKEETKLVQQKTKSGHRRCISQF

>Kalax.1398s0005|Klaxiflora_v1.1/52-475

MEHLFKTSDHDTNADSTSCRCIWVNGPVIVGAGPSGLAVAACLRQQGVPFLVLERAPCIASLWQNRTYDRLKLHLPKQFCQLPNMPFPQHCPEYPTRKQFIQYLESYAQIFEISPRFNESVVSAKYDETYGLWRVKTCSSAGNGVEYICRWLVAATGENAECVVPEIDGLAGFDGQVKHACEYKSGRGFEGKRVLVVGCGNSGMEVSLDLCNHGAFPSMVVRSSVHVLPREFNGRSTFQLACRMLKWLPLWLVDKILLIMAWIVFGSVEKYGLRRPSTGPLELKYKAGKTPVLDIGALAKIKAGGINVVPGIKKFSRHGVELVNGECLSLDAVILATGYRSNVPSWLHESGFFCKNGFPKTAQPNCWKGQSGLYAVGFSRKGLAGASMDATKVALDIGEVWRKETRRSCKTTAALGRCISQFRR

>Kalax.0318s0027|Klaxiflora_v1.1/52-474

MEHLFKTSDHDTNVDSTCRCIWVNGPVIVGAGPSGLAVAACLRQQGVPFLVLERAPCIASLWQNRTYDRLKLHLPKQFCQLPNMPFPQHFPEYPTRKQFIQYLESCAQIFEISPRFNESVVSAKYDETCGLWRVKTCSSAGNGVEYICRWLVAATGENAECVVPEIDGLAGFDGQVKHACEYKSGRGFEGKRVLVVGCGNSGMEVSLDLCNHGAFPSMVVRSSVHVLPREFNGRSTFELACRMLKCLPLWLVDKILLIMAWIVFGSVEKYGLRRPSTGPLELKCKAGKTPVLDIGALAKIKAGGINVVPGIKKFSRHGVELVNGECLSLDAVILATGYRSNVPSWLHESDFFCKNGFPKTAQPNCWKGQSGLYAVGFSRKGLAGASMDATKVALDIGEVWRKETRRSCKTTAALGRCISQFRR

>Kalax.0275s0002|Klaxiflora_v1.1/1-423

MEHLFKTTDHDQSKNPTSARRCIWVNGPLIVGAGPSGLAVAACLKQQGVPFLVIDRASCIASLWQNRTYDRLKLHLPKQFCQLPNMPFPDHFPEYPTKNQFIDYLESYARKFEINPQFNESVASAKYDETCGLWRVKTCGPARSDVEYICRWLVVATGENAECVVPEIDGLAGFDGQVQHACEYKSGDGFKGKRVLVVGCGNSGMEVSLDLCNHGAFPSMVVRSSVHVLPREFNGRSTFALASRMLKWLPLWLVDRILLVMAWIVLGSLEKYGLKRPATGPFELKNKAGKTPVLDIGALAKIKAGEIKVVPGIKEFSSDGVELVNGEYLRLDAVILATGYRSNVPSWLHESDFFCKNGFPKTALPNSWKGQSGLYAVGFSRKGLAGASMDAMKIAQDIGEVWRKETRRSCKTTACLRRCISQF

>Kalax.0122s0027|Klaxiflora_v1.1/1-423

MEHLFKTTDHDQSKNPTSARRCIWVNGPLIVGAGPSGLAVAACLKQQGVPFLVIDRASCIASLWQNRTYDRLKLHLPKQFCQLPNMPFPDHFPEYPTKNQFIDYLESYARKFEINPQFNESVASAKYDETCGLWRVKTCGPARSDVEYICRWLVVATGENAECVVPEIDGLAGFDGQVQHACEYKSGDGFKGKRVLVVGCGNSGMEVSLDLCNHGAFPSMVVRSSVHVLPREFNGRSTFALASRMLKWLPLWLVDRILLVMAWIVLGSLEKYGLKRPATGPFELKNKAGKTPVLDIGALAKIKAGEIKVVPGIKELSSNGVELVNGEYLHLDAVILATGYRSNVPSWLHESDFFCKNGFPKTALPNSWKGQSGLYAVGFSRKGLAGASMDAMKIAQDIGEVWRKETRRSCKTTACLRRCISQF

>Eucgr.H00831|Egrandis_v2.0/1-422

MENLFRLADHDESSARRCVVVNGPVIVGAGPSGLATAALLRDQGVPFVVLEKTDCIASLWQKRTYDRLKLHLPKQFCQLPKLPFPEDFPEYPTKSQFIGYLEAYARRFDINPQFNECVQSARYDETSGWWHVQTVSTAGGSRGEVEYLCRWLVVATGENAECVTPDFEGLAEFGGDVMHACEYKSGEKFAEKKVLVVGCGNSGMELSLDLCNHDALPAMVIRDSVHVLPREVMGISTFGLAVLLMKRLPLWLVDKLLLILAWLVLGNVEKYGLKRPLVGPMELKNTKGKTPVLDIGALAKIRSGEIKVVPGIKKFSPGQVELVNGEKHDVDSVILATGYRSNVPYWLKESEFFSKEGFPKSSFPNGWKGNSGLYAVGFTRRGLCGASSDAINIAQDIGKEYKEKMGQKKTSTPTHRRCISQY

>Brara.A00847|BrapaFPsc_v1.3/1-426

MENMFRLIDNDQEAATNRCIWVNGPVIVGAGPSGLATAACLREQNVPFVVLERADCIASLWQKRTYDRLKLHLPKQFCQLPKMPFPESFPEYPTKRQFIDYLESYASRFDISPKFNECVQTARFDETSGLWRVKTVSDAESTRTEVEYICRWLVVATGENAERVMPEIEGLSEFTGEVVHACDYKSGEKFAGKKVLVVGCGNSGMEVSLDLANHFAKPSMVVRSSVHVMPREIMGKSTFELAMKMLKWFPLWLVDKILLVLCWFVLGNIEKYGLKRPKMGPMELKSAKGKTPVLDIGALEKIRSGKIDVVPGVKRFNGNRVELVNGEELDVDSVVLATGYRSNVPYWLQESEFFAKNGFPKTAIGNSGWKGRTGLYAVGFTRRGLSGASMDAVNIAQDIGSVWKLETKQPTKRSTASHRRCISQQF

>Brara.H01467|BrapaFPsc_v1.3/1-426

MDSMFRLMDQEQEVTTTTNRCIWVNGPVIVGAGPSGLATAACLREQNVPFVVLERADCIASLWQNRTYDRLKLHLPKKFCQLPKMPFPESFPEYPTKRQFIDYLESYASRFEINPKFNECVQTARFDETSGLWRVKTVSNAESTRTEVEYICRWLVVATGENAERVVPEIDGLNEFTGEVIHACDYKSGEKFAGKKVLVVGCGNSGMEVSLDLANHFAKPSMVVRSSVHVMPREVMGKSTFELAMKMLAWLPLWLVDKILLVLCWLVLGNIEKYGLKRPEMGPMELKSVKGKTPVLDIGALEKIRSGKIDVVPGVKRFDGNRVELVNGEQLEIDSVVLATGYRSNVPYWLQESEFFAKNGFPKAANSGWKGRTGLYAVGFTRRGLSGVSMDAVKIAQDIGSVWKLETKQPTKRSTASHRRCISQQF

>AT4G28720|Athaliana_Araport11/1-426

MENMFRLMDQDQDLTNNRCIWVNGPVIVGAGPSGLATAACLHEQNVPFVVLERADCIASLWQKRTYDRLKLHLPKQFCQLPKMPFPEDFPEYPTKRQFIDYLESYATRFEINPKFNECVQTARFDETSGLWRVKTVSKSESTQTEVEYICRWLVVATGENAERVMPEIDGLSEFSGEVIHACDYKSGEKFAGKKVLVVGCGNSGMEVSLDLANHFAKPSMVVRSSLHVMPREVMGKSTFELAMKMLRWFPLWLVDKILLVLSWMVLGNIEKYGLKRPEMGPMELKSVKGKTPVLDIGAIEKIRLGKINVVPGIKRFNGNKVELVNGEQLDVDSVVLATGYRSNVPYWLQENEFFAKNGFPKTVADNNGWKGRTGLYAVGFTRKGLSGASMDAVKIAQDIGSVWQLETKQPTKRSRGSLRRCISQQF

>Brara.B02765|BrapaFPsc_v1.3/1-430

MENMFRLMGSEDFSDRQRCIWVNGPVIVGSGPSGLATAACLREEGVPFVVLERAECIASLWQKRTYDRLKLHLPKKVCQLPKMPFPENYPDYPTKRQFIDYLESYATRFDISPQFNECVQSARYEKTSGLWRIKTVKSSPSPSSAVSGSEMEYICRWLVVATGENAERVVPEIDGLTTEFDGEVIHSCEYKSGEKYRGKSVLVVGCGNSGMEVSLDLANHSANPSMVVRSSVHVLPREILGKSTFEISMILMKWLPLWLVDKILLILAWLVLGNLTKYGLKRPKMGPMELKVLTGKTPVLDIGAMEKIKSSQVDIVPGIKRFSRSHVELVDGQILDLDAVVLATGYRSNVPSWLQENDLFSKNGFPKSPFPNAWKGKSGLYAAGFTRKGLAGASADAISIAQDIGNVWREETKRRKMRTRVGHRRCISVA

>AT5G43890|Athaliana_Araport11/1-424

MENMFRLMGSEDSSDRRRCIWVNGPVIVGAGPSGLATAACLREEGVPFVVLERADCIASLWQKRTYDRIKLHLPKKVCQLPKMPFPEDYPEYPTKRQFIEYLESYANKFEITPQFNECVQSARYDETSGLWRIKTTSSSSSGSEMEYICRWLVVATGENAEKVVPEIDGLTTEFEGEVIHSCEYKSGEKYRGKSVLVVGCGNSGMEVSLDLANHNANASMVVRSSVHVLPREILGKSSFEISMMLMKWFPLWLVDKILLILAWLILGNLTKYGLKRPTMGPMELKIVSGKTPVLDIGAMEKIKSGEVEIVPGIKRFSRSHVELVDGQRLDLDAVVLATGYRSNVPSWLQENDLFSKNGFPKSPFPNAWKGKSGLYAAGFTRKGLAGASADAVNIAQDIGNVWREETKRQKMRTRVGHRRCISVA

>Brara.J00235|BrapaFPsc_v1.3/1-422

MENMFRLMASEAYLSDRRCIWVNGPVIVGAGPSGLATAACLRDQGVPFVVVERSDCIASLWQKRTYDRLKLHLPKKFCQLPKMPFPDHYPEYPTKRQFIDYLESYANKFEIKPEFSKSVVSARFDETSGLWRVKTTTTAGEEMEYICRWLVVATGENAERVVPEINGLKTEFDGEVVHACEYKSGEKYRGKRVLVVGCGNSGMEVSLDLANHNAIASMVLRSSVHVLPREIMGKSTFGISLMLMKWLPLWLADKLLLILSWLVLGSLSKYGLKRPSIGPMELKSKTGKTPVLDIGALEKIKSGDVEIVPAIKRFSRCHVELVDGQKLDIDAVVLATGYRSNVPSWLQESEFFSKNGVPKSPFPNAWKGKSGLYAAGFTRKGLAGASADAVNIAQDIGNVWREETKRQKVRRNVGHRRCISVA

>AT1G04180|Athaliana_Araport11/1-421

MENMFRLMASEEYFSERRCVWVNGPVIVGAGPSGLATAACLHDQGVPFVVVERSDCIASLWQKRTYDRLKLHLPKKFCQLPKMPFPDHYPEYPTKRQFIDYLESYANRFDIKPEFNKSVESARFDETSGLWRVRTTSDGEEMEYICRWLVVATGENAERVVPEINGLMTEFDGEVIHACEYKSGEKFRGKRVLVVGCGNSGMEVSLDLANHNAITSMVVRSSVHVLPREIMGKSTFGISVMMMKWLPLWLVDKLLLILSWLVLGSLSNYGLKRPDIGPMELKSMTGKTPVLDIGALEKIKSGDVEIVPAIKQFSRHHVELVDGQKLDIDAVVLATGYRSNVPSWLQESEFFSKNGFPKSPFPNAWKGKSGLYAAGFTRKGLAGASVDAVNIAQDIGNVWREETKRQKMRRNVGHRRCISVA

>Brara.I05505|BrapaFPsc_v1.3/1-422

MENNMFILMASEEYSSDRRCIWVNGPVIVGAGPSGLATAACLRDEGVPFVVVERSDCIASLWQKRTYDRLKLHLPKKFCQLPKMPFPDHYPEYPTKRQFIDYLESYASHFKIKPEFNKSVESARFDETSGLWQVRTTSAGEEMEYISRWLVVATGENAEQIVPEINGLKTEFNGEVIHACEYKSGEKFRGKRVLVVGCGNSGMEVSLDLANHNAITSMVVRSSVHVLPREIMGKSTFGISVMLMKWLPLWLVDKLILILSWLFLGSLSNYGLKRPNIGPMELKSKTGKTPVLDIGALEKIKSGDVEIVPAIKRFSRSHVELVDGRKLDVDAVVLATGYRSNVPSWLHESEFFSKNGFPKSPFPNAWKGKSGLYAAGFTRKGLAGASADAVNIAQDIGNVWREETKRQKMRRNVGHRRCISVA

>GWHPCBIR017244|Santalum_yasi/1-421

MAQTLDRREIFARRCTWVNGPIIVGAGPSGLAVGAGLKEQGVPFVILERANCIASLWQNRTYDRLKLHLPSQFCQLPNFPFPRHFPEYPSKDQFIAYLESYAKRFEIRPRFKESVQSAKYDETCGLWRVRTISSCGSFRYEFEYICRWLVVATGENAEKVVPEFQGLKQFGGQVMHAREYKSGKKFEGKRVLVVGCRNSGMEVALDLCNHNAIPSIVVRSSVHVLPREIMGRSTFDWAISMMKWLPLWAVDKILLAMALVVLGDTGKHGLKRPSMGPLQLKNTEGKTPVLDTGALKKIKCGEIRVVPGIKRFWKGKVELVNGECLEIDSVVLATGYCSNVPSWLEEKEFFSRNGIPKTPFPNGWKGNVGLYAVGFTRRGLSGAFSDAIRVAADIGTTWKQDTKHKKNSTAAACHRRCVSQF

>Pn20.572|Piper_nigrum/1-418

MAFMGEREESMYLRRCVWVNGPVIVGAGPSGLAVGACLKEQGVPFVMLERADCIASLWQKRTYDRLKLHLPKQFCQLPRMPFPDHFPPYPTKNQFIEYLESYARHFQLSPRFSQCVLSAKHDHTSGLWRVRAADRGKDEVEYICRWLVVATGENAERVVPEIEGMGEFCGGVSHACEYKSGESYRGKRVLVVGCGNSGMELSLDLSNHNAFPSMVVRSSVHVLPREIFGKSTFETAVLLMKWLPPWLVDKLLLVLAWLVLGNIDKYGLRRPGMGPFEFKNTFGKTPVLDIGALEKIRSGHIKVVPGIRRFFPRCVELVDGRIIDIDAIVLATGYSSNVPSWLQESDFFAKNGFPKQPFPNGWKGNAGLYAVGFARKGLSGTSMDATRIAQDIGREWKKETRQTKQFIACHRRCTSQQF

>Pn20.549|Piper_nigrum/1-418

MALMGEREESMYLRRCVWVNGPVIVGAGPSGLAVGACLKEHGVPFVMLERADCIASLWQKGTYDRLKLHLPKQFCQLPRMPFPDHFPPYPTKNQFIEYLESYARHFQLSPRFSQCVLSAKHDHTSGLWRVRAADRGKDEVEYICRWLVVATGENAERVVPEIEGMGEFCGRVSHACEYKSGESYRGKRVLVVGCGNSGMELSLDLSNHNAFPSMVVRSSVHVLPREIFGKSTFETAVLLMKWLPPWLVDKLLLVLAWLVLGNIDKYGLRRPGMGPFEFKNTFGKTPVLDIGALEKIRSGHIKVVPGIRRFFPRCVELVDGRIIDIDAVVLATGYSSNVPSWLQESDFFAKNGFPKQPFPNGWKGNAGLYAVGFARKGLSGTSMDATRIAQDIGREWKKETRQTKQFIAYHRRCTSQQF

>Acora.10G153700|Aamericanus_v1.1/1-416

MLSNVDFFARRCVWVNGPIIVGAGPSGLAVGACLREQGVPFVILERADCIASLWQRRTYDRLKLHLPKQFCQLPRLPFPDDFPDYPSKNQFIEYLERYARAFELKPSFNERVKSAKFDETCGLWRVHTAGGDADEEEEVEYICRWLVVATGENAERVVPDVEGVADFGGRILHVSEYRSGECFKGENVLVVGCGNSGMEVCLDLCDHDAHPHMVVRDSVHVLPREVFGKSTFELAVFMMKWLPLSVVDKLMLLLAWLVVGDVEKLGLKRPSIGPLELKNTHGKTPVLDLGAVAKIRSGELKVVPGIRSFFPGRVELVDGRVLDVDSVIFATGYRSNVPSWLQDTDFFCKNGFPKEPFPNGWKGKSGLYAVGFTKRGLSGASVDAKRIAGDIGRVWKEETKQPKQFIACHRRCISQI

>Acora.01G200900|Aamericanus_v1.1/1-418

MLSEDFFMRRCVWVNGPIIVGAGPSGLAVGACLKEQGVPFVILERADCIASLWQKRTYDRLKLHLPKQFCQLPRLPFPADFPEYPSKNQFIDYLENYATTFELNPRFNQCVQSAKYDETSGLWRVRSASTTATAAGGAAEVEYICRWLVVATGENAESVTPDVEGIREFGGHVSHASEYRSGDSFRGKKVLVVGCGNSGMEVCLDLCHYNAYPSMVVRDSVHVLPREVLGKSTFELAVFLMKWLPLWLVDKLMLLMAWVVLGNIEKLGLKRPSIGPLELKNTQGKTPVLDLGALAKIRSGEIKVVPGIQRFFPGRVELVDGQVLAVDSVIFATGYRSNVPSWLQDTEFFCKNGFPKQPFPQGWKGKSGLYAVGFTRRGLSGASLDARRIAGDIGKVWKQETKQPKQFIACHRRCISQI

>Aco014634|Acomosus_v3/1-421

MGEWDELMLSSPSRRCVWVNGPIIVGAGPSGLAVAACLKEQGVPFVILERADCVASLWQNRTYDRLKLHLPKQFCQLPGLPFPDHYPDYPTKQQFVDYVVSYTVFNDIHPRFNQSVQSARFDRTSGLWRVRAAETGTRGAEVEYIGRWLVVATGENAERVVPEIAGLDAEFGGRVAHVADYKSGEKYAGKRVLVVGCGNSGMEVCLDLCDHNAFPAMVVRDSQVHVLPREVLGKSTFEMAVFLLKWLPLWLVDKILLLLAWLVLGNVEKFGLKRPSAGPLELKNTQGRTPVLDTGALGKIRSGKIKVVPAIKRFSPGKAEFVDGRELEIDSVIFATGYRSNVPQWLQESELFGKDGFPKMPFPNGWKGKSGLYAVGFTRRGLSGASSDAVRIAKDIGRVWKEETRQTKRLVACHRRCISQI

>Aco008902|Acomosus_v3/1-432

MVRRAPETEELFGRRCVWVNGPIIVGAGPSGLAVAACLRERGVPFEVLERSDCIASLWQRRTYDRLKLHLPKQFCQLPMMPFPADYPDYPTKRQFVDYLQAYAARFAIAPRFGQAVQSARFDDTSGLWRVRTTPAAAAPDDDDDDRRSSGEVEYIGRWVVVATGENAERVAPEIEGLAECFGAAAAVAHVADYKRGEAYRGKRVLVVGCGNSGMEVCLDLCEHNAFPAMVVRDSIHVLPREVLGKSTFEMAVLLMKWLPLWLVDKILLLLARIVLGNVEKLGLKRPKNGPLELKNTQGRTPVLDIGALEKIKKAEIKVVPAIKRFLPGKVELVDGQVLSIDSVILATGYRSNVPQWLQESEFFSKDGFPKEPFPNSWKGRSGLYAVGFTRRGLSGTSSDAIKIAADISQVWKEETKQTKRLVACHRRCISQI

>Asa5G03538.1|Allium_sativum/1-211

MEVCLDLCDHEAHPSMVVRDSVHILPREVLGKSTFELAVLLMKWLPLWIVDKILLVLAWLILGNIGKYGLKRPKRGPLELKNTQGRTPVLDIGTLGKIKSGKISIVPGIKRFLQGKVELVNGQILNVDSVILATGYKSNVPFWLKSNSFFNEEGYPNEQFPNSWKGKNGLYVVGFTKRGLSGASFDAVRIARDLSEVWKEELKNGRCISQF

>evm.TU.AsparagusV1_07.1910|Aofficinalis_V1.1/1-415

MACASDTDELFSGRCIWVNGPLIIGAGPSGLAVGACLKEHGVPFVILERSDCIASLWQKRTYDRLKLHLPRKFCQLPKLPFPENYPEYPSKDQFIEYLESYATKFDLNPQFNETVISAKYDEVGGIWRVRTVSKKTEVEYICQWLVVATGENAESLIPEFEGIRDFGGLKMHACDYKSGKDFEGKKVLVVGCGNSGMELCLDLCLHNSRPSMVVRDSVHVLPREILGKSTFELAVTLMKWLPVKFADKIVLALARIVLGNVEKYGLKRPDIGPLELKNTQGKTPVLDIGALKRIRSEEIKIVPGIKKFSPGKVELVDGRVLDVDSVIFATGYSSNVPSWLQGSDFFNKDGFPKEQFPNGWKGKSGLYAVGFTRRGLSGASLDAVRIAEDIGKVWKEETRQAKHIIACHRRCMSQM

>Asa3G01793.1|Allium_sativum/1-419

MSSMSSISNPERCIWVNGPLIVGAGPSGLAVGACLKDHGVPFIILERSNCIASLWQNRTYDRLKLHLPKQFCQLPNFPFPKDYPTYPTKHQFIKYLESYVEHFDLQPQFNETVMSAKYDEANCIWRVRSLSRNVEVEYISQWLVVASGENAESFVPEIEGSSDFSGEVLHASDYKSGKDFEGKRVLVVGCGNSGMELCLDLCLHNAIPSLVVRDSVHVLPRKILGKSTFELAVTLMKWLPVWLADRVILALARMVIGNVEKYGLKRPKMGPMEMKNKQGKTPVLDIGTVQRIRSGDIRVVPGIKKFMHEKVELVDGETLDVDSVIYATGYRSNVPSWLQGFDFFSKEGLPNEDFTEGWKGKLGIYAAGFTRKGLSGTSSDAVKIAEDVAEVWRKERKEAKHTICHRCCLPQYRDDIVEN

>Cucsa.140470|Csativus_v1.0/1-418

MEATTNHEAFLLSQRCVWVNGPVIVGAGPAGLATAACLREQGVPFIVIERADCIASLWQKRTYDRLKLHLPKQFCQLPRLPFPQEFPEYPSKKQFIQYLQSYTQKFDINPQFNETVHSARFDHTSALWRLKTESSVSGQVVEYVCQWLVVATGENAECVMPEIDGLNEFSGEVLHVSDYKSGERFKGKNVLVVGCGNSGMEVSLDLCNHQASPSMVVRSSVHVLPREIMGKSTFELAVLLMKWLPIWIVDKLMLILSWLVLGSIEKYGLKRPLMGPLELKNTTGKTPVLDIGALSKIKSGEIKVVPGIKKLNKHEVEFINGEKMGIDSVLLATGYRSNVPFWLEEKEFFGKNGFPKTPFPNGWKGKSGLYAVGFTRRGLSGVTSDAIKIAQDIGNVWRQETKQKKKPIACLRRCISHF

>evm.TU.AsparagusV1_04.275|Aofficinalis_V1.1/1-418

MGPQLPDSVDFLSRKCMWVNGPIIVGAGPSGLAVAASLKERGVPYVILERSDCIASLWQKRTYDRLKLHLPKQFCQLPGLLFPDSYPEYPCKKQFVDYLESYAKHFDISPKFNQSVQSAKYNETSGLWHVRAIETQHEEVEYIGKWLVVATGENTERVVPDMDGLVSFGSEVTHASDYKSGEAYRGKKVLVVGCGNSGMEVCLDLCDHDAFPSMVVRDSVHVLPREVYGKSTFELSVFLMKWLPLWLVDKIMLVVAWFVLGSLERYGLKRPSVGPLELKTTHGRTPVLDYGALSKIKSGEIEVVPGVKRFMLGRVEFVDGRVLEFDAVVMATGYRSNVPYWLQGTDFFSKDGMPKAQFPNAWKGKSGLYTAGFTRRGLAGASSDAVKVAKDLSKAWKEETQQAKKLVSCHRRCISQIY

>Asa5G02846.1|Allium_sativum/1-418

MCSTEIDSYDFISRKCMWVNGPIIVGAGPSGLAVAASLKDHGVPYVILERSDCIASLWQKHTYDRLKLHLPKKFCQLPGLPFPDSYPEYPCKKQFVDYLESYAKHFDICPKYGQTVQSAKYDETSGLWRVRTVGYNNEEIEYIGKWLVVATGENAESVMPDMEGLDSFGNPITHVCNYKSGAAYQGKKILVVGCGNSGMEVCVDLCDHDAFPSMVVRDSVHVLPREVYGKSTFELAVFLMKWLPIWCADKIMLVFAWLVLGNIENYGLKRPSIGPLELKNTQGRTPVLDTGALNKIKNGQIEVVPGVRRFMPGQVEFCDGRVVEFDAVIMATGYRSNVPFWLQGTDFFTKNGIPKTQFPNSWKGKCGLYAVGFTQRGLAGASYDAVKVAKDLSNAWKEETKQAKRLVSCHRRCISQIY

>Asa1G02429.1|Allium_sativum/1-410

MDFFSRKCMWVNGPIIVGAGPSGLAVAAQLKERGVPYVMLERSDCIASLWQKKTYDRLKLHLPKKFCQLPGLPFPDTYPEYPCKKQFIDYLESYAKHFEICPKFNQSVQSAKYEETSGLWRVKTVGSNKEEVEYLGRWIVVATGENAESVMPEIDGSDSFGSQITHVSDYKSGEVYKGKNVLVVGCGNSGMEVCLDLCDHGAMPSMVVRDSVHVLPREIYGKSTFEIACFLMRWLPLWFVDTIILLLSWFALGNIENYGLKRPTTGPLELKSTLGRTPVLDYGALGKIKSGEIEVVPGVKRFLPGRVEFFDGKILEFDSIIMATGYRSSVSYWLQGTDFFSKNGLPKSQFPNSWKGKSGLYAVGFTKRGLAGASSDAVKVAKALGEAWKEETRQIKKLVPNHRRCISQIY

>Asa1G03675.1|Allium_sativum/1-410

MDFFSRKCMWVNGPIIVGAGPSGLAVAAQLKERGVPYVMLERSDCIASLWQKKTYDRLKLHLPKKFCQLPGLPFPDTYPEYPCKKQFIDYLESYAKHFEICPKFNQSVQSAKYEETSGLWRVKTVGSNKEEVEYLGRWIVVATGENAESVMPEIDGSDSFGSQITHVSDYKSGEVYKGKNVLVVGCGNSGMEVCLDLCDHGAMPSMVVRDSVHVLPREIYGKSTFEIACFLMRWLPLWFVDTIILLLSWFALGNIENYGLKRPTTGPLELKSTLGRTPVLDYGALGKIKSGEIEVVPGVKRFLPGRVEFFDGKILEFDSIIMATGYRSSVSYWLQGTDFFSKNGLPKSQFPNSWKGKSGLYAVGFTKRGLAGASSDAVKVAKALGEAWKEETRQIKKLVPNHRRCISQIY

>AUR62034131|Cquinoa_v1.0/1-422

MFKLTSSHNEEDLVARRCEWVNGPVIVGAGPSGLAVGACLKEQGVPFVVLDRANCIASLWQKRTYDRVKLHLPKNFCQLPKMPFPESYPEYPTKSQFITYLESYAKHFGIEPEFNTSVQSARYENAFGLWRVKTTGHAEGPDGPEVEYLCRWLVVATGENAECVVPDIDGLAHFGGKVIHSSEYKTGESFRGQKVLVVGCGNSGMEVSLDLANHNAQPSMVCRNSVHILPREIMRKSTFELAMFLMRWIPLWLVDKILIFFAWLILGNVEKYGLKRPKTGPLELKNEHGKSPVLDIGALAKIRSGDIKVVPGIKSFYANSVELVNGVAMEVDSVILATGYHSNVPSWLQDGEFFNKNGMPKSPFPNAWKGKSGLYAVGFTRRGLAGASLDAVKIAEDICRVWKKETKQKKLNAPRFRRCFTT

>AUR62008842|Cquinoa_v1.0/1-425

MDQMFNLASIGDNGDDLLSCRFVWVNGPVIVGAGPSGLAVGACLKEQGVPFVVLERADCIASLWQKRTYDRLKLHLPKQFCQLPKMPFPESFPEYPTKRQFIDYLESYANHFDINPHFNECVQSARFDETFGLWRVKTVGQGPTKPEVEYVCRWLVVATGENAECVVPDIDGLAHFGGKVIHSSEYKSGDSYRGQKVLVVGCGNSGMEVSLDLSNHNAEPSVVCRSSVHILPREIMGRSTFELAMLLMKWIPLWLVDKIMLFFAWFMLGDMRKFGLKRPNIGPLELKNKHGKTPVLDIGALAKIRSGDIKVVPGIKSFYTNGVELVDGQTLVVDSVILATGYRSNVPSWLQEGEFFNKNGFPKTPFPSAWKGKSGLYAVGFTRRGLAGASFDAIKIAQDIGKVWKEETIQKKLTAPRHRRCISTF

>AUR62025403|Cquinoa_v1.0/1-425

MDQMFNLAYIGDNGDDLLSRRFVWVNGPVIVGAGPSGLAVGACLKEQGVPFVVLERADCIASLWQKRTYDRLKLHLPKQFCQLPKMPFPESFPEYPTKRQFIDYLESYANHFDINPHFNECVQSAKFDETFGLWRVKTVGQGPTEPEVEYACRWLVVATGENAECVVPDIDGLANFGGKVIHSSEYKSGDSYCGQKVLVVGCGNSGMEVSLDLSNHNAEPSVVCRSSVHILPREIMGRSTFELAMLLMKWIPLWLVDKIMLFFAWFMLGDMRKFGLKRPNIGPLELKNKHGKTPVLDIGALAKIRSGDIKVVPGIKSFYANGVQLVDGQTLDVDSVILATGYRSNVPSWLQEGEFFNKNGFPKTPFPSAWKGKSGLYAVGFTRRGLAGASFDAIKIAQDIGKVWKEETRQKKLTAPRHRRCISTF

>Sapof.05G217400|Sofficinalis_v1.1/1-428

MDQISTDMNKSCSAAPAAEQDLFSRRIVWVNGPVIVGAGPSGLAVGACLRDQGVPFVVLERADCIASLWQKRTYDRLKLHLPKQFCQLPKMPFPKSFPEYPTKRQFIDYLESYANHFEINPRFSECVESAKYDETFGLWRVTTSGLDRSEVEYVCRWLVVATGENAECVVPDIEGLANFNGKVIHSSEYKSGDEYRGQNVLVVGCGNSGMEVSLDLSNHNAVPSMVCRSSVHVLPREIMGRSTFELAMVLMKWLPLWIVDKILLFFAWFIQGDIEKYGIKRPKMGPLELKNKHGKTPVLDIGALDKIKSGDIKIVSEIKGFTSNGVVLDNGDHMTVDSVILATGYRSNVPSWLKEGEFFNKNGFPKAPFPSGWKGKSGLYAVGFTRRGLAGAAFDATKIAEDISQVWKEETTHKKLIAPRHRRCISTF

>Sapof.04G095300|Sofficinalis_v1.1/1-424

MEQIFNLKLSQESPSPKRCVWVNGPVIVGAGPSGLAVGACLREKGVPFVVLERANCIASLWQKRTYDRLKLHIPKQFCELPKMPFPKEFPEYPSKKQFIDYLESYAAHFDISPHFSECVKSAAYQETLGLWRVSTSRTNPDGSVSEVEYLCRWLVVATGENAECVVPEIDGLSDYNGKVVHSSEYKSGDTYRDQRVLVVGCGNSGMEVSLDLSNHNAFPSMVCRSSVHVLPREIMGKSTFELGMFLMKWLPIWVVDKILLFFAWILLGDIEKHGLKRPKIGPLELKNKQGKTPVLDIGALAQIRSKNITVVPGIKRFSSNGVEFENGEKMEVDSVILATGYKSNVPSWLKEGEFFNKNGFPKTTLASTWKGKSGLYAIGFTRKGLAGSSFDATNIAQDIALIWKNQTIRKNPTTPRLRRCISTF

>gene_prefix24410.t1_1|Ceratophyllum_demersum/1-418

MTVDSSKPSLHTSKNEEFPNRCVWVNGPIIVGGGPSGLAVGACLKEYGVPFVILERSNCVASLWQNRTYDRLKLHLPKQFCQLPMFPFPEDFPQYPTRNQFLDYIESYRSHFDITPRFNETVLSAKYDKTSGLWRVKSTEAEYIGRWLVVATGENAESVVPEFEGIEDFGGHVIHACDYKSGEEYVGKKVLVVGCGNSGMEVSLDLCNFNASPIMVVRSSVHVLPREILGKSTFEIAVLMMKWLPLRVVDKILVTSALLLLGNTEKYGLKRPSIGPLQLKNSQGKTPVLDLGALEKIKTGQVKIVPGIAKFSPGKVELVNGDILDVDSVILATGYQSNVPTWLEECDFFSQDGFPKAPFPNSWKGSRGLYAVGFTRRGISGASLDAIRIAQDIAKVWKEETKQKKQFMAPSRRCISQI

>AUR62035901|Cquinoa_v1.0/1-432

MNPVSNAAFIAKTTSANHEEFFAKRCVWVNGPVVVGAGPSGLAVAAGLKEQGVPFIVLERANCIASLWQIRTYNRLKLHLPKQFCQLPNFPFPDDYPEYPSKDQFINYLESYAKKFDIQPQFNESVQSAEYDETCGLWRIKTALGADNCTNKREVEYICRWIVVATGENAEKVVPKFFGFHEFGGDIIHACEYKSGEQYRGKKVMVVGCGNSGMEVSLDLCNHDVQPLLCVRSSVHVLPKDVLGKSTFELAMTLLRWFSLRTVDKILLFLASLLLGNTDKYGLKRPEVGPMELKNTEGKTPVLDLGTFGKIRSGQIKVVPGIKRFARGKVAFVDGEIREVDSVILATGYRSNVPLWLKESDFFTKNGFPKAPFPNGWKGNAGLYAAGFTKRGLSGASEDAIRVAEDIGKIWKEETKQKKPPAACHRRCISQF

>AUR62043861|Cquinoa_v1.0/1-432

MNPVSNAAFIAKTTSANHEEFFAKRCVWVNGPVVVGAGPSGLAVAAGLKEQGVPFIVLERANCIASLWQIRTYNRLKLHLPKQFCQLPNFPFPDDYPEYPSKDQFINYLESYAKKFDIQPQFNESVQSAEYDETCGLWRIKTALGADNCTNKREVEYICRWIVVATGENAEKVVPKFFGFHEFGGDIIHACEYKSGEQYRGKKVMVVGCGNSGMEVSLDLCNHDVQPLLCVRSSVHVLPKDVLGKSTFELAMTLLRWFSLRTVDKILLFLASLLLGNTDKYGLKRPEVGPMELKNTEGKTPVLDLGTFGKIRSGQIKVVPGIKRFARGKVAFVDGEIREVDSVILATGYRSNVPLWLKESDFFTKNGFPKAPFPNGWKGNAGLYAAGFTKRGLSGASEDAIRVAEDIGKIWKEETKQKKPPAACHRRCISQF

>Sapof.14G064100|Sofficinalis_v1.1/1-422

MSNKIKSGSIGNQEEFLGRRCIWVNGPVIVGGGPSGLAVAGGLKEKGVPFIMLERANCIASLWQTRTYDRLKLHLPKQFCQLPNYPMPETYPDYPTKAQFIRYLESYATHFGIQPQFNESVQSAKYDDTCGLWRIKTTTKDEREVEYICRWIVVATGENAEKVVPKFFGFHEFGGEIIHACDYKSGESYRGKKVLVVGCGNSGMEVSLDLTHFHAQPLLCVRSSVHVLPKDVLGKSTFQLATTLLKWFELKTVDKILLFLAKLKLGETDKYGLKRPEVGPLELKNTEGKTPVLDLGTFGKIKSGQIKVVPGIKRFGRGKVAFVDGQILDIDSVVLATGYRSNVPSWLKESEFFSKNGFPKAPFPNGWKGNAGLYAAGFTRRGLSGASEDAIRVADDIGKIWKEDTKQRRTPAACHRRCISQF

>evm.TU.Scaffold_315.833|Carabica_v0.5/1-428

MNNQCNLCLPPMADASEHDELFAGRRILVNGPVIVGAGPSGLAVAAGLQQQGVPFIILERASCIASLWQNRTYDRLKLHLPRQFCELPYFPFPKEFPEYPTKNQFIDYLESYAKHFKISPRFNESVQSAKYDQKCGSWRIKTVVASDNSEIEYICSWLVAATGENAEKVVPEFEGLQDFGEVMHVCDYKSGEAYEGKRVLVVGCGNSGMEVSLDLCDYNAMPSMVVRSSVHVLPREILGLSTFQLAVSFMKWLPVWLVDKILVTATRLILGNLEKYGIKRPSAGPLELKNAEGKTPVLDIGTLDKIRSGKISIVPGIKKFFRGGVELENGEFLEIDSVILATGYRSNVPSWLKENEFFSVDGFPKTPFPNGWKGKAGLYAVGFTRRGLSGASFDAIRVSEDIGQIWKQELKQKNQGALSLANRRTCKS

>DCAR_100332|Dcarota_subsp_sativus_v3.0/1-414

MAGGTEQNGMFSRRCVWVNGPLIIGAGPSGLAVGACLKEQCVPFVVVDRADCLASLWQNRTYDRLKLHLPKQFCQLPKLPFPEDYPEYPTRKQFIQYIESYADHFGIKPQFNESVESAKYDEAARAWRVTTVSTRGSVRSEVDYICQWIVVATGENAEPVIPEIAGLGDFGGEVIHGREYKSGKDYSGKKVLVVGCGNTGMEVSLDLCNHNAKPSMVSRSPVHVLPREIFGKSTFDWAMMMMKWLPIWLVDKILLTLTSFILGDLEKYGIKKPSIGPMELKIKDDGKTPVLDIGALEKIRSGGIKVVPGIKKFSKTMVELVNGQELEIDSVILATGYRSNVLSWLQETEFFSKSGYPKTPFPNGWKGKNGLYTVGFTQRGLLGASADAMKVAQDIAELWKEDWNKELKQKKPNV

>DCAR_100331|Dcarota_subsp_sativus_v3.0/1-414

MAGGPEQKGMFSRRCVWVNGPLIIGAGPSGLAVGACLKEQCVPFVVVERADCLASLWQNRTYDRLKLHLPKQFCQLPKLPFPQDYPEYPTRKQFIQYIESYADHFGIKPQFNESVESAKYDEAARAWRVTTVSTKGSVRSEVDYICQWIVVATGENAEPVIPEIAGLGDFGGEVIHGREYKSGKDYSGKKVLVVGCGNTGMEVSLDLCNHNAKPSMVSRSPVHVLPREIFGKSTFDWAMMMMKWLPIWLVDKILLTLTWFILGDLEKYGIKKPSIGPMELKIKDDGKTPVLDIGALEKIRSGGIKVVPGIKKFSKTMVELVNGQELEIDSVILATGYRSNVLSWLQETEFFSKSGYPKTPFPNGWKGKKGLYAVGFTQRGLLGASADAMKVAQDIAELWKEDWNKELKQKKPNV

>DCAR_100333|Dcarota_subsp_sativus_v3.0/1-410

MAGGTEQNGMFSRRCVWVNGPLIIGAGPSGLAVGACLKEQCVPFVVVDRADCLASLWQNRTYDRLKLHLPKQFCQLPKLPFPEDYPEYPTRKQFIQYIESYADHFGIKPQFNESVESAKYDEAARAWRVTTVSTKGSVRSEVDYICQWIVVATGENAEPVIPEFAGLGDFGGDVIHGREYKSGKDYSGKKVLVVGCGNTGMEVSLDLCNHNAKPSMVSRSPVHVLPREIFGRSTFDWAMMMMKWLPIWLVDKILLALTWFILGDLEKYGIKKPSIGPMELKIKDDGKTPVLDIGALEKIRSGGIKVVPGIKKFSKTMVELVNGQELEIDSVILATGYRSNVLSWLQETEFFSKSGYPKTPFPNGWKGKNGLYTVGFTQRGLLGASADAMKVAQDIAELWKKELKQKKPNV

>DCAR_100330|Dcarota_subsp_sativus_v3.0/1-410

MAGGPEQNGMFSRRCVWVNGPLIIGAGPSGLAVGACLKEQCVPFVVVERADCLASLWQNRTYDRLKLHLPKQFCQLPKLPFPEDYPEYPTRKQFIQYIESYADHFGIKPQFNESVESAKYDEAARAWRVTTVSTKGSVRSEVDYICQWIVVATGENAEPVIPEIAGLGDFGGEVIHGREYKSGKDYSGKKVLVVGCGNTGMEVSLDLCNHNAKPSMVSRSPVHVLPREIFGKSTFDWAMMMMKWLPVWLVDKILLTLTWSILGDLEKYGIKKPSIGPMELKIKDDGKTPVLDIGALEKIRSGGIKVVPGIKKFSKTMVELVNGQQLEIDSVILATGYRSNVLSWLQETEFFSKSGYPKTPFPNSWKGKNGLYAVGFTKRGLLGASADAMRVAQDIAELWKKGLKQKKPNV

>Pn1.2583|Piper_nigrum/1-409

MGMTRSDDQQEDGKWVWVHGPVIVGAGPSGLAVGACLKEHDVPFVILERADCIASLWQKRTYDRLKLHLPKQFCQLPGMAFPLDFPEYPSKLQFIQYLESYSAHFQLSPRFNECVSSAKHDPVTGLWQVRTAAGAEYICRWLVVATGENAEMVVPEFDGSELFAGRVSHACEYKSGEDFRGKKVLVVGCGNSGMEVSLDLCNHNAFPSMVVRSKVHVLPREVLGRSTFEIAVLLLKWLPLWLVDKLLLVVAWLVLGNFEKYGLHRPEVGPLQLKNSVGKTPVLDIGTLEKIRSGGMKVVPGIKRFHPRKVELVNGECLDIDAVVMATGYRSNVPSWLQENHFFSANGFPRQPFPDGWKGKDGLYAVGFTRRGLSGATADAVRIAQDIGQVWKEETRQTKQVVIACNRRR

>Pn1.2591|Piper_nigrum/1-409

MGMTRSDDQQEDGKWVWVHGPVIVGAGPSGLAVGACLKEHDVPFVILERADCIASLWQKRTYDRLKLHLPKQFCQLPGMAFPLDFPEYPSKLQFIQYLESYSAHFQLSPRFNECVSSAKHDPVTGLWQVRTAAGAEYICRWLVVATGENAEMVVPEFDGSELFAGRVSHACEYKSGEDFRGKKVLVVGCGNSGMEVSLDLCNHNAFPSMVVRSKVHVLPRKVLGRSTFEIAVLLLKWLPLWLVDKLLLVVAWLVLGNFEKYGLHRPKVGPLQLKNSVGKTPVLDIGTLEKIRSGGIKVVPGIKRFHPRKVELVNGECLDIDAVVMATGYRSNVPSWLQENHFFSANGFPRQPFPDGWKGKDGLYAVGFTRRGLSGATADAVRIAQDIGQVWKEETRQTKQVVIACNRRR

>Pn3.1551|Piper_nigrum/1-406

MTRSDDQQEDGKWVWVNGPVIVGAGPSGLAVGACLKEQGVPFVILEKADCIASLWQKRTYDRLKLHLPKQFCQLPSMAFPLDFPEYPSKLQFIQYLESYSTHFQLSPRFNECVSSAKHDPVTGQWHIRTAAGAQYICRWLVVATGENAEMVVPEFDGRELFAGGVSHACEYKSGEDFRGKKVLVVGCGNSGMEVSLDLCNHNAFPSMVVRSKVHVLPREVLGRSTFEIAVLLLKWLPLWLVDKLLLVVAWLVLGNLEKYGLHRPEVGPLQLKNSVGKTPVLDIGTLEKIRSGGIKVVSGIKRFHPRKVELVNGECLDIDAVVMATGYRSNVPSWLRESHFFSENGFARQPFPNGWKGKDGLYAVGFTRRGLSGATADAVRIAQDIGQLWKEEARQAKQVIAFNRLR

>Pn4.2696|Piper_nigrum/1-420

MGTTSFDDEHKDESRVVWVNGAVIVGAGPSGLAVAAGLQEQGVPFVIIERADCIASLWQKRAYDRLRLHLPKQFCQLPGLPFPPHYPSYPSKDQFLDFLHSYAHHFRLRPRFNETVASARHDAVSGLWLVRTVAVGGGGGAEYLCRSLVVAAGENAEMVVPEVEGVQEFGGRVLHASEYKSGEDYRGKKVLVVGCGNSGMEVSLDLSNHNAFPSMVVRNSVHVLPREVMGRSTFKMAVFLLKWLPLWVVDKVLLVTAWLVLGDLEKLGIRRPAVGPLQLKNSVGKTPVLNNGTVEKIRSGRIKVVPGIKRFHPRKVELVDGRFLDVDAVVFATGYRSNVPSWLQENDFFSENGFPKQPFPNGWKGKDGLYAVGFTRRGLSGASADAVRVAQDIGLAWKEETRCYGLESGMRRELAFGGCP

>Nycol.D01636|Ncolorata_v1.2b/1-425

MASLVPDNVPLFAGRSVWVNGPVIVGAGPSGLATGACLRQQGVPFVILEKADCIASLWQRRTYDRLKLHLPKRFCQLPRMPFPDHYPQYPSKKQFVDYLERYADHFHLEPTFSTCVVSAIHDDTCGFWRVRTSRVQDGSAADAGSEMEYICRWLVVATGENAEKVVPEIEGMEEQFAGKVIHASEYKCGEEYRGKKVLVVGCGNSGMEVSLDLSDFKAEPAMVVRDSVHVLPREIFGKSTYEVAATLLKWLPLWMVDKLLLICARLELGNIQKFGLKRPAMGPLQLKNTFGRTPVLDIGALKKIRSGDIKVVPGIKKFLSGKVELINGEILDIDAVILATGYKSNVPSWLKECDFFARDGFPRQPFPNGWKGKSGLYAVGFTRRGLSGVSMDAMKIAEDIGKIWKEETKQAKQFVASSRRRISQM

>g08385.t1_1|Euryale_ferox/1-432

MASLVPENGLLFAGRSVWVNGPVIVGAGPSGLATGACLRQQGVPFVILEKADCIASLWQRRTYDRMKLHLPKRFCQLPRMPFPDHYPQYPSKKQFVDYMERYAEHFHLDPTFNTCVVSAVYDDTCGFWRVRTLCVQDGGAADPGSEMEYISRWLVVATGENAEKVVPEIEGMEEQFAGKVIHASEYKCGEEYRGKKVLVVGCGNSGMEVSLDLSDFKAEPAMVVRDSVHVLPREIFGKSTYEVAAILLKWLPLWMVDKLLLICARLELGSIQKFGLKRPSMGPLQLKNTFGRTSVLDIGALKKIRCGDIKVVPGIKKFLPGKVELVNGEILDIDAVILATGYKSNVPFWLKECDFFARDGFPRQPFPNGWKGKSGLYAVGFTRRGLSGVSMDAVKIAEDIGKIWKEETKQAKQFVASNRRCISQILNSPSKM

>g39611.t1_1|Euryale_ferox/1-445

MTSLVPENWPLFAGRSVWVNGPVIVGAGPSGLATGACLRQQDVPFVILEKADCIASLWQHRTYDRLKLHLPKRFCQLPRMSFPDHYPQYPSKKQFVNYLERYTEHFHLDPTFNTCVVSAVYDDTCGFWRVRTLRVQNSDAADPGSEMEYICRWLVVATGENAEKVVPEIEGMEEQFAGKVIHASEYKCGEEYRGKKVLVVGCGNSGMEVSLDLSDFKAEPAMVVRDSVHVLPREIFGKSTYEVAAILLKWLPLWMVDKLLLICARLELGTIQKFGLKRPSMGPLQLKNTFGRTPVLDIGALKKIRSGDIKVVPGIKKFLPGKVELVNGQILDIDAVILATGYKSNVPFWLKEGDFFARDGFPKQPFPNGWKGKSGLYAVGFTRRGLSGVSMDAVKIAEDIGKIWKEETKQRNQFVAFNRRCIPHMHHLRCNWGTDYTQSTEIRRK

>g12633.t1_1|Euryale_ferox/1-424

MASLVPENGPLFAGRSRWVNGPVIVGAGPSGLATGACLRQQGVPFVILEKAECIASLWQRRTYDRLKLHLPKRFCQLPCMPFPDHYPQYPSKKQFVNYLENYADHFHLNPTFSTCVVSAVFDDTCGFWRVSTMRIQDGGAADPDSEMKYFCRWLVVATGENAEKVVPEIEGMEEQFAGKVIHASEYKSGEEYRGKKVLVVGCGNSGMEVSLDLSDFKAEPVMVVRDSVHVLPRDIFGKSTYEVAAILLKWLPLWMVDKLLLICARLEFGNMEKFGLMRPSKGPLELKNTCGRTPVLDVGALKKIRSGDIKVVPGIRKFLPGKVELVNGEVLDIDAVVMATGYTSNVPFWLKERDLFARDGFPRQPFPNGWKGESGLYAVGFTRRGLSGVSMDAVKIAEDIGKIWKDETKQANQFVATDVKFTIY

>Nycol.L00992|Ncolorata_v1.2b/1-424

MVRMPDHFQPLFARRSVWVNGPVIVGAGPAGLATGACLKQQGVPFVMLEKADCIASLWQRRTYDRLKLHLPKRFCELPRMPFPPHYPQYPSKRQFVEYLENYANEFGITPNFNTWVQSAVYDATCGIWRVRTCHVQGGAANGGGEVEYICKWLVVATGENAEKVVPEIDGLNENFGGKVIHACEYKSGEEYRGKKVLVVGCGNSGMEMSLDLCDFDAKPSMVVRNSVHVLPKEIFGISTYVVAAFLLRWLPVWLVDKLLLICAWLELGSIQKYGIKRPAMGPLYLKNTLGRTPVLDIGALEKIRSGDIRVVPGIKRFLPGKVEFVNGETLDIDAVILATGYKSNVPYWLRESDFFSKTGFPKTPFPNGWKGKSGLYAVGFTKRGLSGASIDAVKTAQDIAKMWKEETKQTKQFMPRHRRCISQF

>g18930.t1_1|Euryale_ferox/1-424

MVRMPDDFQPLFARRSVWVNGPVIVGAGPAGLATGACLKEQGVPFVMLEKADCIASLWQHRTYDRLKLHLPKRFCELPRMPFPPHYPQYPSKRQFVEYLENYANEFGITPNFNTCVQSAVYEATCGIWRVRTCHVQGGAANGGGEVEYICKWLVVATGENAEKVVPEIDGMNEHFGGKVMHACEYKSGEEYRGKKVLVVGCGNSGMEVSLDLCDHDAKPWMVVRNSVHVLPREIFGISTYEVAAFLLRWLPVWLVDKLLLICAWWELGRIQKYGIKRPVMGPLYLKNTLGRTPVLDIGALKKIRSGDIEIVPGIKRFLPGKVEFVNGETLDIDAVILATGYKSNVPYWLKESDFFSKTGFPKTPFPNGWKGKSGLYAVGFTRRGLSGASMDAVKTAQDISNMWKKETKQTKQFMPRHRRCISQF

>g20060.t1_1|Euryale_ferox/1-424

MVRMPDEFQPLFARRSVWVNGPVIVGAGPAGLATGACLKQQGVPFVMLEKADCIASLWQRRTYDRLKLHLPKRFCELPRMPFPPHYPQYPSKRQFVEYLENYSNEFGITPNFNTCVQSAVYETTCGIWRVKTCHVQGGAASGGGEVEYICKWLVVATGENAEKVVPEIDGMNEHFGGKVMHACEYKSGEDYRGKKVLVVGCGNSGMEVSLDLCDHDAKPWMVVRNSVHVLPREIFGISTYEVAAFLLRWLPVWLVDKLLLICTWWELGSIQKYGIKRPAVGPLYLKNTLGRTPVLDIGALEKIRSGDIKIVPGIKRFLPGKVEFVDGETLDIDAVILATGYKSNVPYWLKESDFFSKTGFPKTPFPNGWKGKSGLYAVGFTRRGLSGASMDAVKTAQDIAKMWKEETKQTKQFMPRHRRCISQF

>gene_prefix13884.t1_1|Ceratophyllum_demersum/1-419

MMFSSNEEDILTRRVVWVNGPVIVGAGPSGLAVGACLHEQGIPYIILEKSDCIASLWQKRTYDRLKLHLSKQFCQLPFLPFPKDYPQYPTKKQFIDYLESYAKKFDISPNFNECVLSARYDESSEVWKIRSISSNNNEEKEYICKWIVVATGENAESAIPEIDGLREFSGNIIHSSQYKSGENYRGKKVLVVGCGNSGMELGLDLCNHNASPLIVVRSSVHVLPREVLGKSTYELAVFLMKLLPLWLIDKTLLVLAWLVLGNIEQYGLKRPSIGPLEYKNSHGKTPVLDIGALEKIKSGEIKIVPGIKRFTENTVEIVNGEEMEIDSVILATGYRSNVPSWLQETDFFCKNGFPKTQFPNGWKGTSGLYAVGFTRRGLSASSDEAIRISEDISKLWKEEIKPKKTFVPCKRICKILQIR

>XP_020576473.1|Phalaenopsis_equestris/1-415

MPQSPTNSHFLPIRCVWVNGPIIVGAGPSGLATAACLKHKGVPFVILERDDCIASLWQRRTYDRLNLHLPRKFCELPRFPFPKHYPKYPNKKQFIDYLESYARYFDICPRFNEEVTSARYDETCGLWRVRTACAHGEVEYMGRWLVASSGENAEKVIPAIEGILEFGDGVSHVCDYKSGKEYAGKRVLVVGCGNSGMEVSLDLCDHNAFPSIVVRDSVHVLPREVFGKSIFEVAVCLMKWLPLWLVDRIVLIISCLVLGNVEKYGLKRPSTGPLELKNTRGKTPVLDIGALEKIKSGEIKVVPGVRRFLPGKAQLVDDRILDVDSVILATGYSSNVPSWLQGTDFFSNGGFPKNPFPNSWRGESGLYAVGFTKRGISGASFDAVRVAEEIGKVWKEETGQQKMQGSCYRRSISQS

>Zosma04g25860|Zmarina_v3.1/1-412

MSFDLFSAPNSSFVTGPIIIGAGPSGLAVAATLRESGVPFLILERADCIASLWQNRTYDRLKLHLPKKFCELPKLPFPEDYPEYPNRKQFISYLESYARRFEINPNFNESVVSARYDETCDLWRVETVKKGGVKMNYICQSIVVATGENAESVMPEMEGIEDFDGDMVHVSEYKSGKTYKGKKVLVVGCGNSGMEVSLDLCNHGASPSMVVKDSVHVLPREVLGKSTFEIAVMMMGWLPLWLVDKILVILAWAVLGSTDKYGIPRPSIGPMALKNIDGKTPVLDIGALEMIRSGDIKVVPAVKRLLPYGVELVDGLIFEVDSVIFATGYQSNVPSWLQEAEFFSKDGFPRKPFPNCWKGKSGLYACGFTRRGLSGLSMDAVKIANDIGKVWREETRHAKRQTACHRRCISQF

>LOC_Os04g03980|Osativa_v7.0/1-419

MVLQHSDRMDSLFSPQTSWVSGPIIVGAGPSGLAVAASLREQGVPFTMLERADCIASLWQKRTYDRLKLHLPKQFCELPRMAFPAHYPEYPTRRQFIDYLEDYAAAFDINPLFGHTVLSARYDETSGLWRVRASSSAGAEMEYIGSWLVVATGENAESVVPDIPGIDGFGGEVVHVADYKSGEAYRGKRVLVVGCGNSGMEVSLDLCDHGARPAMVVRDAVHVLPREVLGKSTFELAVLLMAWLPLWLVDKILVLLAWLVLGNLAKLGIRRPATGPLELKNTTGRTPVLDYGALARIRSGEITVVPGVARFGRGFAELADGRVIALDAVVLATGYRSNVPQWLQGNDFFNKDGYPKTAFPNGWKGESGLYAVGFTRRGLSGASADAMRAAKDLARVWKEATKPTKKSTACHRRCISVIF

>Zm00001d025005|Zmays_RefGen_V4/1-422

MGLLPTDRMDSLFSPRCQWVNGPIIVGAGPSGLAVAACLREQGVPYVMLERADCIASLWQRRTYDRLKLHLPKQFCELPRMPFPDHYPEYPTRRQFIDYLEDYAARFEIRPEFSTTVVLARYDETSGLWRVTTSAPANGGDVEYIGRWLVVATGENAEAVVPDIPGLGGFHGKVTHVSDYKSGEAYAGKRVLVVGCGNSGMEVSLDLSDHGARPAMVVRDAVHVLPREVLGKSTFQLAVLLRRWLPLWLVDKIMVILAWLVLGNLAKLGLRRPAAGPLELKETHGRTPVLDTGALARIRAGDIAVVPAVTRFGKGGQVELADGRTLDFDAVILATGYRSNVPQWLESSNDSFNKEGYPKTAFPHGWKGQSGLYAVGFTRRGLLGASTDAVRIAKDLGNVWREETKPTKRAGACHRRCISVVF

>Sobic.006G012200|Sbicolor_v5.1/1-422

MGLLPTDRMDSLFSPRCVWVNGPIIVGAGPSGLAVAACLREQGVPYVILERADCIAALWQKRTYNRLKLHLPKRFCELPRMPFPDHYPEYPTRRQFIDYLQDYAAKFEIKPEFSTTVLSARYDETSGLWRVVTSAPNNGGDMEYIGRWLVVATGENAEAVVPDVPGLDGFDGKVTHVSEYKSGECYAGKRVLVVGCGNSGMEVSLDLSDHGARPAMVVRDAVHVLPREVLGKSTFELAVLLMRWLPLWIVDKIMVLLAWLVLGNLAKLGLRRPAAGGPLELKEKYGRTPVLDYGALARIRAGDIAVVPAVTRFGKGGQVELADGRTLNFDAVILATGYRSNVPQWLQGNDFFNKDGYPKTAFPHGWKGESGLYAVGFTRRGLSGASADAVRIAKDLGNVWREETKPTKRAGACHRRCISVVF

>Zm00001d004467|Zmays_RefGen_V4/1-421

MGLLPTDRMDSLFSPRCVWVTGPIIVGAGPSGLAVAACLREQGVPFVVLERADCIASLWQRRTYNRLKLHLPKQFCQLPRMPFPEDYPEYPTRRQFVDYLERYAAEFEIKPEFGTTVLSARYDETSGLWRVVTNGGAGGDMEYIGRWLVVATGENAEAVVPDIPGLAGFDGEVTHVSEYKSGEAYAGKRVLVVGCGNSGMEVSLDLAEHGARPAMVVRDAVHVLPREVLGTSTFGLAVLLMRWLPLWLVDWLMVLLAWLVLGNLARLGLRRPAAGPLQLKETHGRTPVLDYGALARIRAGDITVVPAVTRFAGKGGQVEVADGRTLGFDAVILATGYRSNVPQWLQGNDFFNKDGYPKTAFPHGWKGESGLYAVGFTRRGLSGASADAVRIAKDLGNVWREETKPTKRAGACHRRCISVVF

>ELECO.r07.4BG0359200|Ecoracana_v1.1/1-419

MVLLPTDRMDSLFSPRCVWVNGPIIVGAGPSGLAVAACLREQGVPFVVLEREDCIASLWQKRTYNRLKLHLPKQFCELPRMHFPEDYPEYPTRRQFIDYLQDYAARFEIKPEFNSTVLSARYDETSGLWRVRTSVPDAPEMEYIGRWLVVATGENAENVKPDIPGLEDFAGEVTHVSDYKSGAKYRGKRVLVVGCGNSGMEVSLDLSDYGAHPAMVVRDAVHVLPREVLGKSTFELAVLLMRWLPLWVVDKIMVLVAWLVLGDLAKLGLRRPATGPLELKETHGRTPVLDYGALARIREGEIDVVPAVTRFNKSHVQLADGRVLNFDAVILATGYRSNVPKWLQDNGLFNKDGYPNTAFPHGWKGQSGLYAVGFTKRGLSGASADAVRIAKDLGNVWREETKPTKRTGACHRRCISVVF

>ELECO.r07.4AG0327230|Ecoracana_v1.1/1-419

MVLLPTDRMDSLFSPRCVWVNGPIIVGAGPSGLAVAACLREQGVPFVVLERQDCIASLWQKRTYNRLKLHLPKQFCELPRMHFPEDYPEYPTRRQFIDYLEDYAAKFEIKPEFNTTVLSARYDETSGLWRVRTSVPDAPEMEYIGRWLVVATGENAENVKPNIPGLEDFAGEVTHVSDYKSGAKYRGKRVLVVGCGNSGMEVSLDLSDYGAHPSMVVRDAVHVLPREVLGKSTFELAVLLMRWLPLWIVDKIMVLLAWLVLGDLAKLGLRRPATGPLELKETHGRTPVLDYGALARIREGEINVVPAVTRFNKSHVQLADGRVLNFDAVILATGYRSNVPKWLQDNGLFNKDGYPKTAFPHGWKGQSGLYAVGFTKRGLSGASADAVRIAKDLGNVWREETKPTKRTGACHRRCISVVF

>HORVU.MOREX.r3.2HG0096800|Hvulgare_Morex_V3/1-421

MVLLSSDRMDSLFSPRCVWVNGPIIIGAGPSGLAVGASLREQGVPYVMLEREDCIASLWQKRTYDRLKLHLPKQFCQLPRMPFPADYPEYPTRRQFIDYLEDYAAAFHVKPEFGSTVQSARYDETSGLWRVHSSSAKSGEMEYIGRWLVVATGENAENVVPDIPGLDGFAGEVAHVSEYKSGDKYKGKRVLVVGCGNSGMEVSLDLCDHGALPSMVVRDAVHVLPREVMGKSTFELATLLMAWLPLWFVDKVMVFLSWLILGNLAGFGIRRPAIGPLTLKNKYGKTPVLDTGALAKIRSGDITVVPGVSRFTKSRAELSDGTALDLDAVVMATGYRSNVPQWLQGTDFFGKDGYPTTGFPNGWKGKSGLYSVGFTRRGLSGASADAVRIAKDLGQVWREETKPTTKRAAGACHRRCISVIF

>Bradi5g01327|Bdistachyon_v3.2/1-422

MGLLRSDRTDSLFTPRAVWVTGPIIIGAGPSGLAVAASLREQGVPFVVLERENCIASLWQNRTYDRLKLHLPKRFCQLPRMPFPDNYPEYPDRAQFISYLETYAATFDIKPEFGVTVQSAKYDEDTGVWRVLASPASGKSTVEYLGRWLVVATGENAENVVPDIPGLDSFSGEITHVSDYKSGATYRGKKVLVVGCGNSGMEVSLDLCDHGASPSMVVRDAVHVLPREFLGKSTFELATFLMAWCVPLWFVDKVMVFLSWLVLGNLARFGIRRPAVGPLTLKNTHGRTPVLDTGAMARIKSGDITVVPGVARFTKAGAELSNGTEIDVDAVVMATGYKSNVPRWLESGLFGKDGYPTTAFPNGWKLGESGLYSVGFTRRGLSGASADAVRIAGDIGKVWKEENKPTQRAAGAGHRRSISVIF

>HORVU.MOREX.r3.2HG0207720|Hvulgare_Morex_V3/1-451

MQNISAPAVEQTQQSSLTLVITFTWQPRKRSREAAMMLRMQSLCPPRRVSVDGPIIVGAGPSGLAVAASLGEEGMPFLMLEREDCIASLWQKRTYDRVKLHLPKHFCELPRMPFPDSYPEYPTRRQFIDYLETYAARFDIKPEFNTTVLSAHYDNTSGLWHAQATRAGGAQVEYIGRWLVVATGENAVNVVPDIPGLGGFCGEVTHVSHYKSGEPYNGKRVLVVGCGNSGMEVSLDLCDHGAHPFMVVRDAMHVIPREVLGKSTFELAMLLMAWLPLWFVDKIMIFLAWLVLGDLDKLGIHRPAVGPLTLKHTQGRAPVLDTGALARIRSGQITVVPGITRFTNSDAVLSDGTAVHVDAVILATGYRSNVPQWLRATDFFGKDGYPKTEFPNGWKGQFGLYSVGFARRGISGASADAVRIAKDLCQLWKEETTPTKKAGRPSYRRSISVVL

>LOC_Os07g25540|Osativa_v7.0/1-398

MAARVVWVNGPIVVGAGPAGLSVAACLRERGVPSVLLERADCIASLWQRRTYDRLRLHLPKHFCELPGMPFPDGYPEYPDRRQFVDYLQAYAARAGVEPRFNQSVTSARYDDAAGLWRVRAEDVSVDAAGDVTEYIGRWLVVATGENAERVVPEIDGADDFEGPVSHVAEYKSGAAYRGKRVLVVGCGNSGMEVCLDLCHHNALPAMVVRDSKVHVLPREMLGVATFSVAVFLLRFLPLWVVDRILVVLAWLFLGDLAKIGITRPSRGPLELKNTRGRTPVLDIGALARIRSGDIEVVPGIRRLLRGGAELVDGRRVPADAVILATGYQSNVPQWLKGSDFFTQEGYPRVPFPDGWKGESGLYSVGFTRRGLSGVSSDAVKVAQDIAMAWNHQTATTR

>Bradi1g28967|Bdistachyon_v3.2/1-400

MAASGGGRVVWVNGPIVIGAGPAGLSVAACLNERGVPSVVLDRADCVASLWQHRTYDRLRLHLPRRFCELPGMPFPDHYPEYPDKAQFVAYLEAYAARFGVRPRFNQSVTSARYDSAAGLWRVHAQAHDEDGVVTEYISRWLVVATGENAERVLPEIHGMEDFDGPVSHVSEYKSGAPYRGKRVLVVGCGNSGMEVSLDLCHHNALPAMVVRDSKVHVLPREMLGVATFSVAVFLLRFLPLWLVDRILVVLAWMFLGNLEKLGIRRPSGAGPLELKNARGRTPVLDIGALARIRSGDIEVVPGIRRLFRGGAELVDGCRVAADAVIMATGYHSNVPQWLKGSDFFTQEGYPRVPFPDGWKGESGLYSVGFTRRGLSGVASDAVKVAQDIARAWKHQTATR

>Zm00001d019527|Zmays_RefGen_V4/1-412

MEAADASSAPPPAEGRVVWVNGPIVVGAGPGGLSVAACLRARGVPCVVLDRADCIASLWQRRTYDRLRLHLPRQFCELPGMPFPDHYPEYPTKRQFVDYLQAYAERAGVQPRFNQAVTSARYDRAAGLWRVRAADALADDDVASAASTEYIGRWLVVATGENAERIVPEFDGAQDFAGPVSHVSEYKCGEAYRGKRVLVVGCGNSGMEVCLDLCDHNALPSMVVRDAKVHVLPREMFGVATFSVAVFLLRFLPLWLVDAVLVLLARLFLGDLEKLGIRRPAGGPLELKNARGRTPVLDIGALARIRSGHIQVVPGIKRFFRGGAELVDGRRVAADAVILATGYHSNVPQWLKGSDFFTQEGYPRVPFPHGWKGESGLYSVGFTRRGLSGVSSDAVKVAQDIAVEWEKQTSTR

>Sobic.002G120300|Sbicolor_v5.1/1-425

MAAAAAAAADASSAEAPPSPPAASRVVWVNGPIVVGAGPAGLSVAACLRARGVPCVVLDRADCIASLWQRRTYDRLRLHLPRHFCELPGLPFPDNYPEYPTKRQFVDYLNAYAEQAGVQPRFNQAVTSARYDAAAGFWRVRADDVVLAEDAAAVAAGATTTEYIGRWLVVATGENAERIVPEFEGAEDFAGPVSHVSEYKSGEAYRGKRVLVVGCGNSGMEVCLDLCDHNALPSMVVRDAKVHVLPREMFGVATFSVAVFLLRFLPLWLVDAILVLLARLFLGDLDKLGIRRPAGGPLELKNTRGRTPVLDIGALARIRSGHIQIVPGIKRLFRGGAELVDGRRVAADAVILATGYQSNVPQWLKGCDFFTQEGYPRVPFPHGWKGESGLYSVGFTRRGLSGVSSDAVKVAQDIAVEWEKQTSTI

>Zm00001d005439|Zmays_RefGen_V4/1-398

MAAAASRTVWVNGPIVVGAGPAGLSVAACLRERGVPSVVLDRADCIASLWQRRTYDRLRLHLPRRFCELPGMPFPAHYPEYPTKRQFVEYLQAYADRAGVEPRFNQAVTSARYDEAAGLWRVRAEDVLASTSTTEYIGRWLVVATGENAERVVPEFEGARDFAGPVSHVAEYKCGEAYRGKRVLVVGCGNSGMEVCLDLCDHGALPSMVVRGSVHVLPREMLGVATFSVAVFLLRFLPLRLVDAVLVLLARLFLGDLERLGIRRPACGGPLQLKNARGRTPVLDIGALARIRSGHVRVVPGIRRLFRGGAELQDGRRVAADAVILATGYHSNVPQWLKGSDFFTEEGYPRVAFPHGWKGESGLYSVGFTRRGLSGVSSDAVKVAQDIAVEWEKQTSKR

>ELECO.r07.7AG0564960|Ecoracana_v1.1/1-402

MASAASKVVWVNGPIVVGAGPAGLSVAACLKARGVPSVVLDRADCVASLWQRRTYDRLRLHLPRHFCELPGMPFPDDYPEYPTKRQFVDYLEAYAAQSGVEPRFNQAVTSARYDKAAGLWRVCAEDVSATSSAGEDVVTEYIGRWLVVATGENAERIVPDFQGMEEFAGPVSHVSEYKSGEAYRGKRVLVVGCGNSGMEVCLDLCDHNALPAMVVRDSVHVLPREMLGVATFSVAVFLLRFLPQWLVDRILVLLARVFLGDLEKIGIRRPSRGPLELKNSRGRTPVLDIGALARIRSGDIEVVPGIKRFFRGGAELVDGRRVAADAVIMATGYHSNVPQWLKGSDFFTQEGYPRVPFPDGWKGESGLYSAGFTRRGLSGVSSDAIKVAQDIAMAWEQQTSSR

>ELECO.r07.UnG0722520|Ecoracana_v1.1/1-406

MASAAISKVVWVNGPIVVGAGPAGLSVAACLKARGVPSVVLDRADCVASLWQRRTYDRLRLHLPRHFCELPGMPFPDDYPEYPTKRQFVDYLEAYAAQSGVEPRFNQAVTSARYDKAAGLWRVCAEDVSATSTASAGDEDVVTEYIGRWLVVATGENAERIVPDFEGMDEFAGPVSHVSEYKSGEAYRGKRVLVVGCGNSGMEVCLDLCDHNALPAMVVRDSVHVLPREMLGVATFSVAVFLLRFLPQWLVDRILVLLARVFLGDLEKIGIRRPSRGPLELKNSRGRTPVLDIGALARIRSGDIEVVPGIKRFFRGGAELVDGRRVAADAVIMATGYHSNVPQWLKGNDFFTQEGYPRVPFPDGWKGESGLYSAGFTRRGLSGVSSDAVKVAQDIAMAWEQQTSSR

>HORVU.MOREX.r3.5HG0537690|Hvulgare_Morex_V3/1-413

MMASRTMWVNGPIIVGAGPAGIAAAACLHTRGVPSVVLDRDDCLASLWQRRTYDRLRLHLPKHFCQLPGMPFPDHYPEYPTKHQFVAYLQSYAAAFHVHPRFRQSVVSARFDHAAGLWRVQASDQDTGITTEYIGRWLVVATGENAERVIPDLDGADSFKGPVTHVSEYKSGEPYKGKRVLVVGCGNSGMEVCLDLCDHGARPSMVVRDGVHVLPREMLGVATFSVAVFLLRFLPLRVVDRLLVLLAGLFLGGDLARLGLRRPSHGGPLELKNSKGRTPVLDIGALEKIRAGDIKIVPGVKRLEAGGAELVDGRFVAADAVILATGYHSNVPQWLKGSDFFSGEGYPKVDFPEGWKGESGLYSVGFTRRGLAGLSLDAVRVAGDIAAEYHTTSPSIVPQPTQLVPSSQSQAET

>Bradi1g00587|Bdistachyon_v3.2/1-410

MTWVNGPIVVGAGPAGLAVAACLDTHGVPSVVLERDDCIASLWQRRTYDRLRLHLPKQFCELPGMPFPADFPEYPSKHQFLSYLHSYAARFHVAPRFNRAVVSAQFDHAAGLWRVHTETSSSSSPATAEEEYIGRWLVVATGENAERIIPPEYSSSGSSGFSGPVSHVSEYKSGAPYAGKKVLVVGCGNSGMEVSLDLCDHGARPAMIVRGAVHVLPRDVLGVATFSLASLLLRFLPLRLVDGLLVFLAGVFLGADLPRLGLRRPTGAGPLEMKNSKGRTPVLDVGALDKIRKGDIEVVNAGVKRLVAGGAELVDGRFVAADAVVLATGYHSNVPQWLKGSECLFSGEGYPKVGFPEGWKLGESGLYSVGFTRRGLAGVSLDAVRVAADIATAYHNNNTNTNTSSSSASV

>DCAR_935750|Dcarota_subsp_sativus_v3.0/1-424

MVYNLEYNHPFFSRRCVLVTGPVIVGAGPSGLAVAAGLKQQGVPFVILEQADCIASLWQNRTYNRLKLHLPKQFCQLPLFPFPKHYPEYPTKFQFLEYLESYARKFEITPRFNESVQSAKYDETCGMWRLRVVTTSGGVVVSVMNYISRWLVVATGENAEKIVPDIEGLKEFGGSVMHACDYKSGKDFQYQNVLVVGCGNSGMEVSYDLCHHKANPLMVVRSSVHVLPRNIFGASTYGLAATMLNWMPVWMVDIVLVALSRLILGNLEKYGLKRPSIGPLQYKNTEGKTPVLDVGALHKIRSGEIKIVPGIKKLSRRRVEFVDGNVLDIDAVIFATGYCSNVPSWLKENEFFSREGTPVGKYPNGWKGKDGLYAVGFTNRGLAGASSDATRVSIDIGKLWKEETKKMKHSSVVLAGHRRCKSHV

>KAH9300426.1|Taxus_chinensis/1-191

VHVLPREMLGRSTFGLAMWLLKWLPLRVVDFLLLLVAWVMLGDTSRYGLRRPAMGPLELKNKCGKTPVLDVGTLARIREGKIKVFPAIERFTSGGAKFVDEQVKDFDSVILATGYKSNVPTWLKECDFFSEDGFPKTPFPNGWKGESGLYTVGFTRKGLLGASMDASRIAEDISRCWKAEAKQFEGPALLK

>MA_58284g0010|Picea_abies/1-442

MDCFSEQSGKEAHDHYLSCFSEQSGKEAHDHYLRKASVSVNDPRIFNGPIIVGAGPSGLATAACLHEKGVPSLIFEKADCIASLWQRRAYDRLKLHLPKQFCELPLMPFPEDFPTYPDKQQFVDYLESYAQRFEIHPRFNVSVESASYDDTCGFWRVRTKCEKEGMHARETEYLSRWLIVATGENAEAIHPHFSGKEKFPENRIIHARDYKSGREFKGKNVLVVGCGNSGMEMSLDLSDNNANAFIVARNNVHLLPREMLGTSTFGLGMWLSKWLPLRLVDRLLLFVAYFMLGNTSRYGLRRPEMGPLELKNKFGKTPVLDVGALAKIKEGKIKVVPEIESLTSGGARFVDGQEKEFDAVILATGYRSNVPSWLKERQFFGEDGFPRNPFPNGWKGETGLYAVGFTRKGLLGASKDATRIAQDIAMCWKAESVASKGSQTNN

>PITA_40117|Pinus_taeda/1-422

MDCFSEQSGKEAHDRYLSWSGECVDGPIIVGAGPSGLATAACLHEKGVSSLLLEKSDCIASLWQRRAYDRLKLHLPKQFCELPLMPFPEHFPAYPDKQQFVEYLESYAQRFNIHPRFNVCVESASYDDNCGFWRVRTRSQEEGMDSTREKEYCSRWLIVATGENAEAIYPHFPGLEHFQKPIIHASAYKSGVHYKGQNVLIVGCGNSGMEMSLDLSDHNANAFIVARNNVHLLPREMLGMSTFGLGMLLSKWLPLWLVDRLLLLVAYFMLGNTSRYGLRRPEMGPLELKNKFGKTPVLDVGTLAKIRQGKIKVVPAIESLTSGGARFVDGQEKEFDAMILATGYRSNVPLWLKERQFFAEDGFPRRPFPNGWKGENGLYAVGFTRKGLLGASKDATRIAQDIAMYWKAEVVSKSASTSAAAN

>PITA_02278|Pinus_taeda/1-422

MDCLSEQSGKEAHDRYLSWSGECVDGPIIVGAGPSGLATAACLHEKGVSSLLLEKSDCIASLWQRRAYDRLKLHLPKQFCELPLMPFPEHFPAYPDKQQFVEYLESYAQRFNIHPRFNVCVESASYDDNCGFWRVRTRSQEEGMDSTREKEYCSRWLIVATGENAEAIYPHFPGLEHFQKPIIHASAYKSGVHYKGQNVLIVGCGNSGMEMSLDLSDHNANAFIVARNNVHLLPREMLGMSTFGLGMLLSKWLPLWLVDRLLLLVAYFMLGNTSRYGLRRPEMGPLELKNKFGKTPVLDVGTLAKIRQGKIKVVPAIESLTSGGARFVDGQEKEFDAMILATGYRSNVPLWLKERQFFAEDGFPRRPFPNGWKGENGLYAVGFTRKGLLGASKDATRIAQDIAMYWKAEVVSKSASTSAAAN

>PSME_00043651-RA|Pseudotsuga_menziesii/38-464

MACFSEQSGKEAHDYYLKTEWAVQSESVDGPIIVGAGPSGLATAACLHNEGVPSLILEKSDCIASLWQRRAYKRLKLHLPKQFCELPLKPFPEHFPTYPSKDQFVDYLESYAKHFNIHPRFNVCVDSASYDNTSGLWRVSTECREEGTNVRDRKEYLSRWLIVATGENAEAIDPRFSGSEKFQGRTIHASNYKSGEEFEGQNVLVVGCGNSGMEMSLDLSMNNATAFIVVRQNVHLLPREMLGRSTFEVGMWLSKRLPLWLVDRLLLVAAYFMLGNTSRYGLRRPQMGPLELKDKFGKTPVLDVGTLAKIREGKIKVVPAVESLTSRGARFVDGQEKDFDAVILATGYRSNVPSWLKERQFFAEDGFPRTPFPNGWKGENGLYAVGFTRKGLMGASMDATRIAEDIAMCWKAEAASKSKLHVAAVKY

>evm.TU.supercontig_78.87|Cpapaya_ASGPBv0.4/1-421

MDCLREIQGKEAHDPMSTSCNCNNKSVRRVWVPGPVIVGAGPSGLAVAACLKERGVPCEILERSNCIASLWQLKTYDRLRLHLPKQFCELPLMGFPADFPTYPTKQQFIYYLEAYARRFDVRPRFNETVAGAEYDGTVGFWRVRSVGGKGGEMTEFVCRWLVVATGENAEAVKPKIEGMEEFGGDIRHTSSYKSGDVFRGKRVLVVGCGNSGMEVCLDLCNHSATPWLVVRDSVHVLPREMLGRSTFDLSMWLLKWLPMGIVDSLLLILSRLMLGDTSRFGLHRPATGPLHLKNLSGKTPVLDVGTLAKIKSGHIKVCPGIKRLKRRAVEFVNGKIENFDAIILATGYKSNVPSWLKEREMFSEKDGLPRKPFPNGWKGENGLYAVGFTKRGLLGASMDAKRIAEDIERSWKLSIAFGSQS

>Potri.006G243400|Ptrichocarpa_v4.1/1-434

MDCLREIEGKQAHDPLFDKIMNKSSRRVFVPGPVIVGAGPSGLAVAACLKEKGFPSMVLERSSCIASLWQLKTYDRLRLHLPKQFCELPLMGFPSEFPTYPTKQQFIHYLETYARKFEIRPRFNETVSHAEYDKAIGFWRVKTVGKKLEETEYMCRWLVAATGENAEAVVPEIDGMGEFGGDIRHTSHYKSGEEFKSKKVLVVGCGNSGMEVCLDLCNYSAKPSLVVRDTVHVLPREMLGKSTFGLSMWLLKWLPMRLVDRFLLIVSRLMLGDTARLGLDRPELGPLELKNLSGKTPVLDVGTLAKIKSGDVKVCPGIKKLKRHTVEFLDGKMENFDAIILATGYKSNVPSWLKEGDMFEKDGFPKRPFPNGWRGECGLYAVGFTKRGILGASMDAKRIAEDIERYCRNEEAAPYDHHHRSVLLLKSSSSSPSP

>Potri.018G036800|Ptrichocarpa_v4.1/1-437

MDCLRELEGKHAHDPLFEKIMNKPSRVFVPGPVIVGAGPSGLAVAACLKEKGVLSMVLERSSCIASLWQLKTYDRLRLHLPKQFCELPLMGFPSEFPTYPTKQQFLHYLESYAQKFEIGPRFNETVSHAEYDKVNGFWRVKTVGKKLEETEYVCRWLVVATGENAEAVVPEIDGMGEFGGDIKHTSYYKSGEEFTGKKVLVVGCGNSGMEVCLDLCDHSAKPSLVVRDTVHVLPREMLGQSTFGLSMWLLKWLPVRLVDRFLLIVSRLMLGDTARFGLERPELGPLELKNLSGKTPVLDVGTLAKIKSGDIKICPEIKKLKRHTVEFIEGKTENFDAIILATGYKSNVPSWLKEGDMFSEKDGFPRRPFPNGWKGECGLYAVGFTKRGILGASVDAIRIAEDIERCRNEEAAPCDRSVLMKSPPPPPPSFSCLEFDL

>orange1.1g013943m.g|Csinensis_v1.1/1-433

MFMDCLREIEGKQAHDPIFIEKMNKSSPRCICVPGPVIVGAGPSGLATAACLKERGIPSILLERSNCIASLWQLKTYDRLRLHLPKQFCELPLMGFPSEFPTYPSKQQFVDYLEAYAKRFEIRPRFNETVSQAEYDATIRFWRVKTTVGGQKCGVEEMEYRCRWLVVATGENAEAVVPEIEGSDEFGGDIRHTSLYKSGEDFRGKRVLVVGCGNSGMEVCLDLCNHDAMPSLVVRDTVHVLPQEMLGKSTFGLSMWLLKWLPMRLVDKLLLVVSWLMLGDTARFGLDRPLLGPLQLKNLSGKTPVLDAGTLAKIKSGHIRVFPGIKRLKRYAVEFVNGRCENFDAIILATGYRSNVPSWLKESEMFSRKDGLPRRPFPNGWKGESGLYSVGFTKRGLLGVAMDAKRIAQDIESCWKAKAPFARSLLLPHSSSP

>Gohir.A05G048600|Ghirsutum_v3.1/1-434

MDCLTEIQGKQAHDPIFIEKMNTSASWRCICVPGPLIVGAGPSGLATAACLKEKGVPSVVLERSNCIASLWQLKTYDRLRLHLPKQFCELPLMGFPTDFPTYPTKQQFVDYLEAYAREFDIKPRFNETVSQAEYDATLGFWRVKSVGVNGKGTEYVCRWLVVATGENAEAVMPEMEGMGEFGGDIWHTSLYKSGEEFRGKRVLVVGCGNSGMEVCLDLCNHNARPSLVVRDTVHVLPREMLGRSTFGLSMWLLKWLPIRFVDRLLLIVSWLMLGDTARIGLDRPRLGPLELKNLSGKTPVLDVGTLANIKSGDIKVCPSIKRLKRYAVEFVNGRTENFDAIILATGYKSNVPSWLKEREMFSEKDGYPRRPFPNGWKGESGLYAVGFTKRGLLGTSMDARRIAEDIERCWKHEAKHRMAFTRSLLQQPSSDSSS

>Gohir.D05G050500|Ghirsutum_v3.1/1-434

MDCLREIQGKQAHDPIFIEKMNTSASWRCICVPGPLIVGAGPSGLATAACLKEKGVPSVVLERSNCIASLWQLKTYDRLRLHLPKQFCELPLMGFPTDFPTYPIKQQFVDYLEAYAREFDIKPRFNETVSQAEYDATLGFWRVKSVGVNGKGTEYVCRWLVVATGENAEAVMPEMEGMGEFGGDIRHTSLYKSGEEFRGKRVLVVGCGNSGMEVCLDLCNHNAKPSLVVRDTVHVLPREMLGRSTFGLSMWLLKWLPIRFVDRLLLIVTWLMLGDTARIGLDRPRLGPLELKNLSGKTPVLDVGTLANIKSGDIKVCPSIKRLKRHAVEFVNGKTENFDAIILATGYKSNVPSWLKEREMFSEKDGYPRRPFPNGWKGESGLYAVGFTKRGLLGTSMDARRIAEDIERCWKHEAKHRMAFTRSLLQQPSSDSSS

>Gohir.D07G027700|Ghirsutum_v3.1/1-433

MDYLSEIEGKQAHDPMFLEKMKESSSSSSSCLCVPGPVIVGAGPSGLATAACLKEKGVPSVILERSNCIASLWQLKTYDRLRLHLPKQFCELPLMGFPTGFPTYPSKQQFVDYLEAYARKFDIKPRFNETVSQAEYDPTLGFWRVTSVGVKGKEMEYVCRWLVVATGENAEAMMPEMEGMGEFSGDIRHTSLYKSGEEFRGKRVLVVGCGNSGMEVCLDLCNHNARPSLVVRDTVHVLPQEMLGTSTFGLSMWLLKWFPLRLVDRFLLIVSWLMLGDTARFGLARPHLGPLQLNNLSGKTPVLDVGTLAKIKSGDIKVRPSIKSLKRHAVEFVNGTTENFDAIILATGYKSNVPSWLKEKVMFSEKDGYPRRPFPNGWKGEYGLYAVGFTKRGLLGTSMDAIRIAEDIQRCWKEEAKHRLAFTNSLLHQSSSL

>Gohir.A07G024100|Ghirsutum_v3.1/1-433

MDYLSEIEGKQAHDPMFLEKMKESSSSSSSCLCVPGPVIVGAGPSGLATAACLKEKGVPSVILERSNCMASLWQLKTYDRLRLHLPKQFCELPLMGFPTGFPTYPSKQQFVDYLEAYARKFDIKPRFNETVSQAEYDPTLGFWRVTSVGVKGKEMEYVCRWLVVATGENAEAMMPEMEGMGEFSGDIRHTSLYKSGEEFRGKRVLVVGCGNSGMEVCLDLCNHNARPSLVVRDTVHVLPQEMLGTSTFGLSMWLLKWFPLRLVDRFLLIVSWLMLGDTSRFGLARPHLGPLQLKNLSGKTPVLDVGTLAKIKSGDIKVRPSIKSLKRHAVEFVNGTTENFDAIILATGYKSNVPSWLKEKVMFSEKDGYPRRPFPNGWKGEYGLYAVGFTKRGLLGTSMDAIRIAEDIQRCWKEEAKHRLAFTNSLLHQSSSL

>Gohir.D06G095000|Ghirsutum_v3.1/1-430

MDSLREIEGKQAHDPIFIEKMNKPCSRSSRCVYVPGPLIVGAGPSGLAAAACLKAKGVPSVILERSNCIASLWQFKTYDRLHLHLPKQFCELPLMGFPTDFLTYPTKQQFVDYLESYAKKFDIRPRFNETVSRAEYDPTVGFWRVRSVRSKGKETEYVCRWLVVATGENAEEVVPEIEGMGEFGGDIRHTSSYKSGEEFRGKRVLVVGCGNSGMEVCLDLYNHNAKPSVVVRDTVHILPREMLGRSTFGLSMWLLKWLPMRLVDRFLLIVSWLMLGDTARFGLDRPRLGPLQLKNLSGKTPVLDVGTLAKIKSGDIKVCPSIKCLNGHAVEFVNGKTESFDAIIFATGYKSNVPSWLKERKMFSEKDGYPRRPFPNGWKGESGLYAVGFTKRGLLGTSMDAKRIAEDIERCWKQEAKHHFCTHHHHHHHD

>Gohir.A06G138000|Ghirsutum_v3.1/1-434

MDSLREIEGKQAHDPIFIEKMNKSCSRSSRCVYVPGPLIVGAGPSGLAAAACLKAKGVPSVILERSNCIASLWQFKTYDRLHLHLPKQFCELPLMGFPTDFLTYPTKQQFVDYLESYAKKFDIRPRFNETVSRAEYDPTVGFWRVRSVRSKGKETEYVCRWLVVATGENAEEVVPEIEGMGEFGGDIRHTSSYKSGEEFRGKRVLVVGCGNSGMEVCLDLYNHNAKPSVVVRDTVHILPREMLGRSTFGLSMWLLKWLPMRLVDRFLLIVSWLMLGDTARFGLDRPRLGPLQLKNLSGKTPVLDVGTLAKIKSGDIKVCPSIKCLKGHVVDFVNGKTENFDAIIFATGYKSNVPYWLKERKMFSEKDGYPRRPFPNGWKGESGLYAVGFTKRGLLGTSMDAKRIAEDIEWCWKREAKHHFCTHHNHHHHHHHHG

>Glyma.06G081300|Gmax_Wm82.a6.v1/1-440

MEYFLREIEGKQAHDPLFMNNYNKSSSSSSSFSSSSSSTVWVHGPVIVGAGPSGLAAAACLRDKSVPSVILERSNCIASPWQLKTYDRLRLHLPKQFCELPFMGFPSHFPNYPSKQQFVQYLENYAESFGIRPRFNETVQHAEFDGKLGLWRVKSVDKVGKTTEYMCRWLIVATGENAEAVVPDIEGVEEFGAPIKHTSLYKSGEEFRGKRVLVVGCGNSGMEVCLDLCNHNATPSLVVRDTVHVLPREMLGKSTFGLSMWLLKWLPMRLVDRFLLMVSWLLLGDTSHLGLDRPRLGPLELKNLSGKTPVLDVGTLAKIKGGDIKVRPAIKRLKRHTVEFVDGRTENYDAIILATGYKSNVPYWLKEEDMFSKKDGYPRRPFPNGRKGRNGLYAVGFTKKGLLGASMDAKRIAEDIEQCWETGANHRTTLAPSHLPQPNS

>Glyma.04G079700|Gmax_Wm82.a6.v1/1-437

MEYFLREIEGKQAHDPLFMNNNKSSLSSSSSSSFSTVWVHGPVIVGAGPSGLAAAACLREKSVPSVILERSNCIASLWQLKTYDRLRLHLPKQFCELPFMGFPSHFPTYPSKQQFVQYLENYAERFGIRPRFNETVQHAEFDAKLGLWRVKSVDKAEKTTEYVCRWLIVATGENAEAVVPDIEGVEEFGAPIKHTSLYKSGEEFRGKRVLVVGCGNSGMEVCLDLCNHNATPSLVVRDTVHVLPREMLGKSTFGLSMWLLKWLPIRLVDRFLLMVSWLLLGDTSKLGLDRPRLGPLELKNLSGKTPVLDVGTLAKIKGGDIKVRPGIKRLKRQTVEFVDGRTENFDAIILATGYKSNVPYWLKEEDMFSKKDGYPRRPFPNGWKGRNGLYAVGFTKKGLLGASMDAKRIAEDIEQSWKAGANHRTTFARSHLPQPNS

>Glyma.14G141200|Gmax_Wm82.a6.v1/1-436

MDYCLRELEGKQAHDSLFLEKMKNSLSQRTTTSERCVWVPGPVIVGAGPSGLATAAYLKEKGVPSLILERSNCIASLWQLKTYDRLHLHLPKNFCELPLMGFPCDFPTYPTKQQFIEYLESYAERFHIRPRFNETVQHAEFDATLGFWRVKSLNKREVATEFVCRWLIVATGENAEAVVPGIEGMGEFGGTIKHTSLYKSGEEFRGKRVLVVGCGNSGMEVCLDLCNHNATPSLVVRDTVHILPREMLGKSTFGLSMWLLKWLPIRFVDRFLLIVSWLMLGDTARFGLDRPKLGPLQLKNLSGKTPVLDVGTLAKIKSGHIKVRPGIKRLKRYTVEFVDGRTENFDALILATGYKSNVPYWLKEEDMFSKEDGFPTKPFPNGWKGENGLYAVGFTKRGLLGASMDAKRIAEDIERCWKAKHSTSFSLSLNVPQSNS

>Glyma.17G189700|Gmax_Wm82.a6.v1/1-438

MDYCLRELEGKQAHDPLFLQKMKNSSSQSTTTSSERCVWVPGPVIVGAGPSGLATAAYLKEKGLPSLILERSNCIASLWQLKTYDRLHLHLPKNFCQLPLMGFPCDFPTYPTKQQFIEYLESYAESFDIRPRFNETVRRAEFDATLGFWRVKSFNKKEVATEFVCRWLIVATGENAEAEVPEIEGMGEFGGAIKHTSFYKSGEEFRGKRVLVVGCGNSGMEVCLDLCNHNATPSLVVRDTVHILPREMLGKSTFGLSMWLLKWLPIRFVDWFLLIVSWLMLGDTARFGLDRPKLGPLQLKNLSGKTPVLDVGTLAKIKSGHIKVRPGIKRLKRYTVEFVGGRTENFDAIILATGYKSNVPYWLKEDDMFSKEDGFPTKPFPDGWKGENGLYAVGFTKRGLLGASMDAERIGEDIERCWKEKHSSTSFSLSLNVPQSNS

>KAI5394071.1|Pisum_sativum/1-433

MDSYLRELEGKQANDPLFIENMNKNNSTSSNERCLWIPGPLIVGAGPSGLAVAAYLKQKGVPSLILERSNCIASLWKLKTYDRLRLHLPKQVCELPLMKFPSGFPTYPTKQQFIEYLESYSEKFDIRPWFNETVKHAEFDATLGFWRVRSEGKGGMITEFVCRWLIVATGENAEAVVPEMEGVGEFGGSIKHTSLYKSGEEFRGKKVLVVGCGNSGMEICLDLCNHDAAPSIVVRDSVHILPRDMLGKSTFGLSMWLLKWLPMQLVDCILLIMSWLLLGDTARFGLVRPREGPLELKKLSGKTPVLDVGALAKIKRGDIKVCSGIKRLKRYTVEFVDGKIENFDAIVLATGYKSNVPYWLKDKDMFSKKDGYPMKPFPNGWKGENGLYAVGFTKRGLLGASMDAKNIAQDIEWCWKSEAKHTLAFALLPQSKS

>KAI5394072.1|Pisum_sativum/1-238

MDSYLRELEGKQANDPLFIENMNKNNSTSSNERCLWIPGPLIVGAGPSGLAVAAYLKQKGVPSLILERSNCIASLWKLKTYDRLRLHLPKQVCELPLMKFPSGFPTYPTKQQFIEYLESYSEKFDIRPWFNETVKHAEFDATLGFWRVRSEGKGGMITEFVCRWLIVATGENAEAVVPEMEGVGEFGGSIKHTSLYKSGEEFRGKKVLVVGCGNSGMEICLDLCNHDAAPSIVVRDSV

>KAI5403377.1|Pisum_sativum/1-430

MDYCIRELEGKQAHDPLFMKKMNNHKSPSSPSSTDPHRRVFVNGPVIVGAGPSGLAAAACLKQKNIPSIILERSNCIASLWQLKTYDRLRLHLPKQFCELPFMEFPSNFPTYPSKQQFIKYLENYAETFHIRPKFNETVKNAEFDSKIGCWKLKCQSSFKGDVTTEYVCRWLIVATGENAEAVVPDIEGADEFEGVIRHTSLYKSGEEFRGKRVLVVGCGNSGMEVCLDLCNHDATPSLVVRDSVHVLPREMLGKSTFGLSMWLLKWFPLRLVDRFLLLVSWLLLGDTSQLGLDRPTLGPLQLKNLTGKTPVLDVGTLAKIKAGHIKVRPSIKKLKRHTVEFVDGRSENFDGIILATGYKSNVPYWLKEEDMFSMKDGFPMKPFPNGWKGKNGLYAIGFTKRGLLGASMDAKRIANDIEQCLKAEAKHGS

>VIT_204s0008g03920|Vvinifera_v2.1/1-415

MDFLREIEGKRAHDPIFNDKAMSCGRSLILGPVIVGAGPSGLATAACLKEKGVPSVILERSNRIASLWQLNTYDRLCLHLPKQFCELPLMPFPENFPTYPTKQQFIEYLEAYAERFDIQPRFNESVARAEYDHTLGFWRVKTETTEYLCRWLIVATGENAEAVVPEIEGRRKFGGPIVHTSSYKSGDVYRGKRVLVVGCGNSGMEVCLDLCNHNARPSLVVRDSVHILPRQMLGKSTFGLSMWLLKWLPMRLVDHLLLIVSRFMLGDTAKFGLDRPKLGPLELKNLSGKTPVLDVGTLAKIKSGDIKICPGIRQLKCHAVEFVDGRTENFDAIIFATGYKSNVPFWLKERDLFSEKDGLPRRPFPNGWKGNCGLYAVGFTKRGLLGASMDAKRISEDIERCWKADAKRLTVKSHT

>Hyque.11G054300|Hquercifolia_v1.1/1-423

MFMDYLREIEGKRAHDDNTTNMINSPKRVWVPGPVIVGAGPSGLAAAACLRERGVPSVILERSNCIASLWQLKTYDRLRLHLPKKFCELPLMPFPEEFPTYPSKQQFINYLEGYANIFDIRPVFNQTVAKAEFDKSLGFWRVRSVGLKGEEREYVCRWLVVATGENAEAVVPEIEGVEEFGGDIVHTSVYKSGGVFRGKRVLVVGCGNSGMEVCLDLCNSNAFPSLVVRDTVHVLPREMLGRSTFGLSMWLLKWLPMRLVDRLLLIASWLMLGDTARFGLERPQVGPLELKNVSGKTPVLDVGTLAKIKSGDIKVCPGIRRLMPHTVEFVDGRKENFDGIILSTGYKSNVPSWLKETEMFSEKDGLPRMPFPNGWKGKCGLYAVGFTKRGLLGASMDAKRIAEDIEKCWKDELKHLTAFAHSQ

>Hyque.01G074400|Hquercifolia_v1.1/1-424

MDYLREIEGKRGHDHHFPMGSSPRCVWVPGPVIVGAGPSGLAAAACLKQKGVPSVVLERSNCIASLWQLKTYDRLRLHLPKQFCQLPLMSFPEEYPTYPSKQQFVDYLEGYAREFGINPVFNRSVESAAYDESIGFWRVKSNGGGVVGEEEREYVCRWLVVATGENAEPVVPEIDGVEEFGGPIVHTSCYKSGDVFNGKRVLVVGCGNSGMEVCLDLCNYNASPSLVVRDTVHVLPQEMLGRSTFGLSMWLLKWLPMRLVDRLLLIASWLMLGDTTRFGLDRPHVGPLELKNLSGKTPVLDVGTLAKIKSGDIKVCPGIQRLTRHTVEFVNGRKENFDGIILATGYKSIVPSWLKEREMFSEKDGLPRRPFPNGWKGECGLYAVGFTKRGLLGASMDARKLAEDIENCWRMESKHLSRFASSYY

>Rhdel02G0259500.1|Rhododendron_delavayi/1-432

MDFLREIEGKKSHDPLLSCEDKATAPDRRCRFVPGPVIVGAGPSGLAAAACLKQKGIESLVLERSNCIASLWQLKTYDRLRLHLPKKFCELPLMPFPQDFPNYPSKQQFVDYLEDYAREFDIRPVFNETVARAEYDSGAGYWRVRCTGGGGGEEREYVSRWLVVATGENAEEVVPEVEGREEFGGSVVHTSGYKSGEVFRGKRVLVVGCGNSGMEVCLDLCNHSASPSLVVKDTVHVLPREMLGRSTFGLSMWLLKWLPIRLVDRFLLILSWLVLGDTARLGLHRPSVGPLQLKNSAGKTPVLDTGTLAKIKSGDIKVCPAIRKLTRHSAEFINGEKENFDAIILATGYKSNVPSWLKESDMFSEKDGLPRRPFPNGWKGECGLYAVGFTKRGLLGASIDAKRIAEDIEQCWRHEAKYLTAIAQSLSPELRN

>Litul.01G162300|Ltulipifera_YP108A_v1.1/1-424

MDYLRETEGKRAHDPFDHGRTMKPDRCLCVPGPVIVGAGPSGLATAACLKERGIPSVILERANCIASLWQLKTYDRLRLHLPKQFCELPLMPFPTSFPTYPKKQQFIAYLEAYARRFELQPTFNEAVVSAEYDRKFGFWRVRTVGPKEETEYVCRWLVVATGENAEAVLPKINGMEDFIGPVVHTSMYKSGDDFQGKRVLVVGCGNSGMEVCLDLCNYNARPSLVVRDTVHVLPREMLGRSTFGLSMWLLKWLPVHMVDRFLLLVSWLMLGDTAQLGLDRPQIGPLELKNLSGKTPVLDVGTLAKIRDGDIKVCPAIKRLTVHGAEFVDGRMEDFDAVILATGYRSNVPSWLKERDFFSEKDGLPRKPFPNGWKGECGLYAVGFTKRGILGASMDARRIAQDIERYWKAETKQFTAFYRPPTAS

>Pn9.681|Piper_nigrum/1-405

MDYLREVEGKRVHDPLEGGRQVYVLGAVIVGAGPSGLAVAACLKERGVPSMILERNDCIASLWQLKTYDRLRLHLPKSFCELPLMPFPSSFPTYPTKQRFIDYLESYARQFDLRPMFRQAVESAEYDDSVGLWRVRAVEGKKGEAVEYVCRWLVVATGENAEPVVPEMDGKDQFLGNIVHTSGYKSGDAFGGKKVLVVGCGNSGMEVCLDLCNFNASPSLVVRETVHVLPREMLGSSTFGLSMWLLKWFPIHIVDHLLLLASRIILGDTSSLGLNRPQLGPLQLKNITGKTPVLDVGTLSKIRSGEIKVFPGVKRLTRRGAEFVDGREEEFDAVILATGYRSNVPSWLKENKFFSKKDGYPRSPFPNGWRGENGLYAVGFTKRGLLGASMDARRIAQDVELSRKA

>evm.TU.Scaffold_465.92|Carabica_v0.5/1-427

MDYLREIEGKRLHDRNPNEKMMMKSSRCVCVSGPIIVGAGPSGLAAAACLKVKGVRSVVLERSHCIASLWQLKTYDRLRLHLPKQFCELPLMPFPEGFPTYPTKQQFIQYLEAYARKFDINPCFNQSVVSAEYDQVLGLWRVRTVGLKKEETEYVCPGLIVATGENAEPVVPDIEGMKDFGGDILHTSLYKKGGDFGGKRVLVVGCGNSGMEVCLDLCNHNASPTLVVRDTVHVLPREMLGKSTFGLSMWLLKWLPMRLVDGFLLIVSRLLLGDTARYGLHRPQMGPLQLKNLSGKTPVLDVGTLAKIKAGDIKVSPGIQRLRPLSAEFVNGKTEKFDAIILATGYKSNVPSWLKEKEMFSVKDGLPKRPFPNGWKGESGLYAVGFTKRGLLGASMDAKRIADDIEMCWKAESKHFSYFARPSSLQS

>evm.TU.Scaffold_597.40|Carabica_v0.5/1-427

MDYLREIEGKRLHDRNPNEKMMMKSSRCVCVSGPIIVGAGPSGLAAAACLKVKGVRSVVLERSHCIASLWQLKTYDRLRLHLPKQFCELPLMPFPEGFPTYPTKQQFIQYLEAYARKFDINPSFNQSVVSAEYDQVLGLWRVRTVGLKKEETEYVCPGLIVATGENAEPVVPDIEGMKDFGGDILHTSLYKKGGDFGGKRVLVVGCGNSGMEVCLDLCNHNASPTLVVRDTVHVLPREMLGKSTFGLSMWLLKWLPMRLVDGFLLIVSRLLLGDTARYGLHRPQMGPLQLKNLSGKTPVLDVGTLAKIKAGDIKVSPGIQRLRPLSAEFVNGKTEKFDAIILATGYKSNVPSWLKEKEMFSVKDGLPKRPFPNGWKGESGLYAVGFTKRGLLGASMDAKSIADDIEMCWKAESKHFSYFARPSSLQS

>DCAR_104873|Dcarota_subsp_sativus_v3.0/1-413

MGYLREMDHRRCVFVPGPVIVGAGPSGLAVAACLKQRGIPSVVVERSNCVASLWQLKSYDRLRLHLPKQFCELPLMPFPQSFPTYPTKQQFVRYLEDYSTKFEISPLFNECVEGAEFDPSIGFWRVRSVGRRGDGEERRSEFVCRWLVVATGENAEAVVPEIRGMEEFSGEIVHTSCYKSGDAFGGKKVLVVGCGNSGMEVCLDLCNHNATPSLVVRDAVHVLPREVLGKSTFGLSMWLLKWFPVRLVDTFLLIVCRILLGDTSKLGLVRPSIGPLELKNSAGKTPVLDVGTLDRIKKGDIKVVPGIQRLTKKTVEFVDGEKENFDAIILATGYKSNVPSWLKEREMFSEKDGLPRRPFPNGWKGESGLYAVGFTKRGLLGASMDARQIAEDIERCWIAEAKNLPLLSRSLTS

>DCAR_100533|Dcarota_subsp_sativus_v3.0/1-428

MDYLQERAWKESNDHHVNNNNNNNNNLRRVFVPGPVIVGAGPSGLAVAACLKQRGIPSVIIERTNCVASLWQLKSYDRLRLHLPKQFCELPLMPFPQSFPTYPTKQQFITYLEAYATKFDINPLFDESVESATFDSGIGFWRVRSVGKGKSGEEGGGHEFVCRWLVVATGENAEEVVPEIRGMKEFKGQIVHTSCYKSGGAFQGKKVLVVGCGNSGMEVCFDLCLHNASPSLVVRDAVHVLPREMLGRSTFGLSMWLLKWFPVRLVDAFLLIVCRILLGDTSKLGLVRPRIGPLELKSSAGKTPVLDVGTLDRIKKGDIKVVPGIKRLTRQTVEFVNGETEDFDAIILATGYKSNVPSWLKEKGMFSEKDGFPRQPFPNGWKGDNGLYAVGFTKRGLLGASMDARRIAEDIEGCWKAEVKNLTPLSRS

>Aqcoe7G179100|Acoerulea_v3.1/1-424

MDYLKEMEGKSLHDRFDMKNIKIIRSVWVPGPVIVGAGPSGLAVAACLKQRGVPSMILERANCIASLWQNKTYDRLRLHLPKEFCELPLKPFPQHFPTYPTKQQFVAYLESYAKEFGIKPQFNATVKSAEYDQNLGFWRVRTLGLKEEIEYICQWLIVATGENAEPIVPGIEGSNDFVGTMVHTSSYKSGEAFRGKRVLVVGCGNSGMEVCLDLCNHDARPSLVVRDTVHVLPREMLGKSTFGLSMWLLKWLPIRLVDRFLLLVSRALLGDTTHLGLTRPTLGPLQLKNMSGKTPVLDVGTLAKIRTGEIKVCPGLKRLTRNTAEFTDGSAGDFDAVILATGYKSNVPSWLKDNELFSEKNGLPRREFPHGWKGECGLYSVGFTKRGLLGTSMDARKISEDIDRSWKAEAKHLKDLYSLPTSSL

>GWHPBAUK021886.1|Macadamia_integrifolia/1-426

MESWREIEGKMVHDHLGKKKKKNRRVWVPGPVIVGAGPSGLATAACLKERGVPSLILERERCIASLWQLKTYDRLCLHLPKQFCELPLMPFPAGFATYPTKEQFLDYLESYAKVFDLKPVFNETVKNAEYDHSLGFWRVKTMGLKEEETEYVCRWLVVATGENAEAVVPEIEGITQFNGPKVHTSQYKNGQTFDGKRILVVGSGNSGMEVCFDLCNHNARPSLVVRDAVHVLPREMLGRSTFGLSMWLLKWFPVRIVDRFLLFVSRLILGDTSRLGLYRPSIGPIELKNKSGKTPVLDVGTLSKIKSGEIKICRAIKRLTRYSAEFVDGKVEDFDAIIFATGYKSNVPTWLKERDLFSEEDGYPKKPFPNGWKGECGLYTVGFTKRGLLGASMDARKIAQDIECCWKAESKHLTAVAPSLRPSPQS

>GWHPBAUK034810.1|Macadamia_integrifolia/1-435

MDSWREIGGKMVHDQLENKMDRGSAPRRVWVPGPVIVGAGPSGLATAACLKEGGVPSLILERANCIASLWQLKTYDRLRLHLPKQFCELPLMPFPAGFPTYPTKQQFMEYLESYAKVFDISPAFNQTVKSAKYDHSLGLWKVMTIGLKEETEYVCRWLVVATGENAEQVLPEIEGIHDFKGSKVHTSQYKNGQNFDSKKVLVVGCGNSGMEVCLDLCNHNARPSLVVRDTVHVLPREMLGRSTFGLSMWLLKWFPMCLVDRFLLLVSRLMLGDTSRLGLCRPPLGPLELKSMSGKTPVLDAGTLAKIQSGQIKVCRGIKRLTDHAVEFVDGKVENFDAIIFATGYKSNVPSWLKERDLFSEEDGYPRKPFPNGWKGECGLYAVGFTKRGLLGASMDARRIAQDIERCWKAESKHLTAVASPRRPPPPPPPTPLLS

>Pso10G21760.0|Papaver_somniferum/1-433

MEFLKELEGKRFHDRLNEIKMTTTTTTATPMKKRRSVWVSGPVIVGAGPSGLAVGACLQEKGISSVILERANCIASLWQLKTYDRLRLHLPKKFCELPLMPFPAHFPTYPMKQQFVSYLEDYAAKFNLNPQFNETVVSAEFDKTVGFWRVKTNGLKEDFEYICRWLIVATGENAEEVVPEIEGSDEFVGPIVHTSLYKSGKIYSGKKVLVVGCGNSGMEVCLDLCNYNAMPSLVVRDTVHVLPRELMGKSTFGLSMWLLKWLPVRIVDRLLLVLTWLILGNTSQIGLSRPDLGPLELKGITGKTPVLDVGTLAKIRAGDIKVYPSVKRLTRTSVEFIDGRMEDFDAIIMATGYKSNVPCWLKDKDLFSEKNGLPWKPFPNGWKGENGLYSVGFTKKGLLGSSMDARKIAQDIEEYWKADELKHLCTFTRHSQL

>Pso08G40300.0|Papaver_somniferum/1-436

MEFLKELEGKRFHDSLNEIKMTTTTTATQMMMTIKKRRSVWVSGPVIVGAGPSGLAVGACLQEKGISSVILERANCIASLWQLKTYDRLRLHLPKKFCELPLMPFPAHFPTYPMKQQFVSYLEDYAAKFNLNPQFNETVVIAEFDKTVGFWRVKTNGLKEDFEYICRWLIVATGENAEEVVPEIEGSDEFVGPIVHTSLYKSGKVYSGKKVLVVGCGNSGMEVCLDLCNYNAMPSLVVRDTVHVLPRELLGKSTFGLSMWLLKWLPVRIVDRLLLVLTWLILGNTSQIGLSRPDLGPLELKGITGKTPVLDVGTLAKIRAGDIKVYPSVKRLTRTSVEFIDGRMEDFDAIIMATGYKSNVPSWLKDKDLFSEKNGLPWKPFPNGWKGENGLYSVGFTKKGLLGSSMDARKIAQDIEEYWKADELKHLCTFTRHSQL

>Gohir.A05G023200|Ghirsutum_v3.1/1-426

MDYYKEVEGKRVHDHRFGKNTMSTLARCIWVPCPVIIGAGPSGLAVAACLKERGIPSLILEKANCIASLWKLKTYDRLRLHLPKQFCELPFMPFPANFPTYPTKTQFVAYLESYKNRFGLDPVFNKTVVSAEFDDQCGFWRIKTLGLKQEETEYVSQWLIVATGENADEVVPHIDGMDDFGGPILHTSSYKSGQLFRGKDVLVVGCGNSGMEVSLDLCNSNARPSLVVRDSVHVLPQEMLGKSTFGLSMWLLKWFPMRLVDRFLLLVSRFMLGDTEKFGLHRPEFGPLELKSRSGKTPVLDVGTLAKIKSGNIKVCLGIKRITHRAVEFVDGKKENFDAIILATGYKSNVPQWLKEADMFSEEDGMPKEAFPRGWKGERGLYAVGFTKRGLLGASFDAKKIAEDIERQWKAEATNFMPYIHPLTSS

>Gohir.D05G024700|Ghirsutum_v3.1/1-426

MDNYKEVEGKRVHDHRFGKNTMSTLARCIWVPCPVIIGAGPSGLAVAACLKERGIPSLILEKANCIASLWKLKTYDRLRLHLPKQFCELPFMPFPENFPTYPTKTQFVAYLELYKNSFGLVPVFNKTVVSAEFDDQCGFWRIKTLGLKQEETEYVSQWLIVATGENADEVVPRIDGMDDFGGPILHTSSYKSGLLFLGKDVLVVGCENSGMEVSLDLCNSNARPSLVVRDSVHVLPQEMLGKSTFGLSMWLLKWFPVRLVDRFLLLVSRFMLGDTEKFGLHRPEFGPLELKNRSGKTPVLDVGTLAKIKSGNIKVCLGIKRITHRAVEFVDGKKENFDAIILATGYKSNVPQWLKEADMFSEEDGMPKEAFPRGWKGERGLYAVGFTKRGLLGASFDAKKIAEDIERQWKAEATNFMPYIHPLTSS

>Gohir.D06G061000|Ghirsutum_v3.1/1-409

MDYCKEVEGKGVHDPFNKNMTTLSRCVWVPYPVIIGAGPSGLATAACLKQRGISSLILERENCIASLWKLKTYDRLRLHLPKHFCQLPFMPFPESFPTYPTKKQFLSYLESYKNHFGLDPVFNKTVVSAEFDLRCGFWRIKTLGLKHEETEYVSRWLIVATGENAEEVVPKIEGMDDFSGPILHTSSYKSGRLFRGKDVLVVGCGNSGMEVCLDLCNCEARPSLVVRASVHVLPQEMLGRSTFGLSMWLLKWFPVRIVDRLLLLLSRFTIGDTGKFGLHRPQLGPLELKSRSGKTPVLDVGTLDKIKSGNIKVYPGIRRITYNAAEFVDGTKENFDAIILATGYKSNVPQWLKDRDLFSEKDGLPREPFPNGWKGECGLYAVGFTKRGLLGSSLDAKRIAEDIALQWKA

>Gohir.A06G062700|Ghirsutum_v3.1/1-409

MDYCKEVEGKGVHDPFNKNMTTLSRCVWVPYPVIIGAGPSGLATAACLKQRGISSLILERENCIASLWKLKTYDRLRLHLPKHFCQLPFMPFPESFPTYPTKKQFLSYLESYKKHFGLDPVFNKTVVSAEFDHRCGFWRIKTLGLKHEETEYVSRWLIVATGENAEEVVPKIEGMDDFGGPILHTSSYKSGRLFRGKDVLVVGCGNSGMEVCLDLCNCEARPSLVVRASVHVLPQEMLGRSTFGLSMWLLKWFPVRIVDRLLLLLSRFTIGDTGKFGLHRPQLGPLELKSRSGKTPVLDVGTLDKIKSGNIKVYPGIKRITYNAAEFVDGTKENFDAVILATGYKSNVPQWLKDRDLFSEKDGLPREPFPNGWKGECGLYAVGFTKRGLLGSSLDATRIAEDIALQWKA

>Prupe.7G231200|Ppersica_v2.1/1-432

MDYLKELEGKRLHDHFEAKMAAKTTRRRIFVPGPVIVGAGPSGLAAAACLKEKGIPSLILERANCIASLWQLKTYDRLRLHLPKHFCELILMPFPSDFPTYPTKQQFLAYLNAYAEHFDLKPVFNMTVVSARFDSRCGLWFVKTLGLKNEETEYVCQWLIVATGENAEEVVPEFEGVDEFGGPILHTSSYKSGELFRGKKVLVVGCGNSGMEVCLDLCNYNASPSLVVKDSVHILPQEILGISTFGLSIWLLKWFPMRLVDHFLLLVSRLMFGDTAQFGINRPERGPLELKSMTGKTPVLDIGTLAKIKTGHIRVCKAIKQLKHQAVEFIDGKVEHFDAIIFATGYKSNVTSWLKETNMFSEKNGLPRKPFPNGWKGECGLYSVGLTQRGLLGASLDARRIAEDIEHSWKAEATHFMAFTACALPPTPPPQP

>FvH4_1g04800|Fvesca_v4.0.a2/1-437

MDSLKELEGKRVHDPLIHFQSKTDMAAAKTTRRILVPGPVIVGAGPSGLAAAACLKEKGIPSLILERANCIASLWQLKTYDRLRLHLPKKFCELILMPFPADFPTYPTKQQFLGYLKAYAERFDLKPAFNTTVVSAKYDTCSGLWLVKTLGLKNEEEIEYVCQWLIVATGENAEEVVPEFEGTKDFGGPIVHTSSYKSGEMFREKKVLVVGCGNSGMEVCLDLCNYNAKPSLVVKDSVHILPQEILGISTFGLSMWLLKWFPMRLVDRFLLLVSRFLLGDTARFGIHRPRRGPLELKSVTGKTPVLDIGTLAKIRSGHIKVCKAIKQVKHQSVEFIDGKVESFDAIILATGYRSNVMSWLKETSMFSEKNGLPRKAFPNGWKGESGLYSVGFTQRGLLGASMDAKRIAEDIEHRRKAEKATHIMPRLVTCASPLAQP

>Hyque.01G272500|Hquercifolia_v1.1/1-419

MDYLKEMEGKRAHDHFQETRTESPTRVRVVRVPGPVIVGAGPSGLAVAACLKEKGVESLILERANCIASLWQLKTYDRLRLHLPKKFCELPLMSFPTNFPTYPTKEQFVGYLEAYAERLGLDPVFNKTVVSAEFDNGYGVWRVRTLGPKMEEIEYVCQWLIVATGENAEEVVPKFEGMSEFVGPIVHTSSYKSGDLFCGKSVLVVGCGNSGMEVCLDLCNYNARPSLVVRDSVHVLPQEMLGRSTFGLSMLLLKWFPIRVVDQFLLLMSCLMLGDTARYGLTRPKLGPLELKSISGKTPVLDVGTLAKIKAGDIKVCHGIKRLTCHAVEFSDGRVENFDAIILATGYKSNVPTWLKESNFFSEKDGLPRKPFPNGWKGENGLYAVGFTKRGLLGTSMDARRIAEDIDHRWNSEGNEFNA

>Hyque.18G093800|Hquercifolia_v1.1/1-418

MVLMDYLNEIEGKRVHDHFEDKMIKSAATVWVAGPIIVGGGPSGLATAACLKEKGVPTLIIERTNCIASLWQLKTYDRLRLHLPKKFCELPLLPFPSDFPTYPTKQQFVSYLEAYMARFDLRPVFNTTVVSAEYDGRYGGVWRVKTVGPKMEGTTTEYVCKWLVVATGENAEEVVPEFEGVSEFGGPIVHTSLYKSGDSFRGKRVLVVGCGNSGMEVCLDLCNYDAKPSLVVRDSVHVLPQEMLGRSTFGLSMWLLKWLPIWVVDQFLLVMSRIMLGDTAQIGLTRPKLGPLELKSISGKTPVLDVGTLAKIKAGDIKVCQGIKRLTHQAVEFVDGSVENFDAIILATGYKSNVLTWLKENNFFWEKDGLPRKPFTNGWKGENGLYAVGFTKHGLLGTSMDARRIAEDIEHQWKSEKV

>VIT_211s0016g03930|Vvinifera_v2.1/1-422

MDYLKEVEGKRVHDPLGRKMSKSERRILVPGPVIVGAGPSGLAVAACLKERGISSLILERENCIASLWNLKTYDRLHLHLPKEHCELPLMPFPPDFPRYPTKHQFLNYLEAYAKRFDIRPFFNKTVVSAEFDPRSRLWQVKTRGFKKEEEIVYQCQWLIVATGENAEEVVPEIQGMNEFAGPIIHTSSYKSGDSYRGKRVLVVGCGNSGMEVCLDLCNHNAFPSLAVRDSVHVLPQEMLGRSTFGLSMWLVKWFPVRLVDQFLLLVSRMMLGDTARLGLNRPKLGPLQLKSLSGKTPVLDVGTLAKIKTGNIKVCRGIKQLSCHTAEFVDGRVENFDAIILATGYKSNVTSWLKEGNMFSEKDGLPRKPFPNGWKGECGLYAVGFTKRGLLGASLDARRIAEELSVHSSWPFQHFRHDNHDL

>orange1.1g013435m.g|Csinensis_v1.1/1-443

MDNNNHLKEVEGKRVHDHFNNKKAAISAARRIMVPGPVIVGAGPSGLATAACLTEKGVPSLILERANCIASLWQLKTYDRLRLHLPKQFCQLPLMPFPSNFPTYPTKQQFLTYLETYTNHFGLDPVFNTTVVNAEYDHLSRLWRVKTQQGLKQEETVYLCQWLIVATGENAEEVVPYIEGMDGFRGPIFHSSSYKTGELFRDKNVLVVGCGNSGMEVSLDLCNYNARPSLVVRDTVHVLPQEMIGRSTFGLSMCLLKWFPVRLVDQFLLLMSWLMLGDTSQFGLIRPKLGPLELKNVSGKTPVLDVGTLAKIRSGNIKVCRAIKRLTHHAAEFIDGSIENYDAIILATGYKSNVPYWLKDTEMFSEKDGFPRMEFPNGWKGAHGLYAVGFNKRGLLGASIDARRISEDIEHQWNSEAKKLMAFSRSLPLPPNQDLEFNSMVVN

>Glyma.07G086200|Gmax_Wm82.a6.v1/1-427

MDYLKELEGKSVHDCYHQHQIKMSKMASPIFVPGPVIVGAGPSGLAAAACLKQKGIPSLVLERAQCLASMWQFKTYDRLRLHLPKQFCQLPLMPFPKNLPSYPTKQQFLAYLKAYADHFDIKPVFSQTVVSAEFDHVCHHWRVKTQGVLKKEDTAEYVCQWLIVATGECAEEVVPQIEGMGEFEGQIVHTCKYKSGNKFCGKNVLVVGCGNSGMEVCLDLCNHNARPSLVVRDTVHILPQQMLGKSTFGLSMFLLKWFPIRFVDQFLLLMSHLMLGDTDQFGLRRPKLGPLELKNLYGKTPVLDVGTLTQIKNGKIKVCRGIKRLARNAVEFVDGKVENFDAIILATGYKSNVPSWLKGSDMFSEKDGFPRKPFPNGWKGENGLYAVGFTKRGLLGASIDAKRIAEDIEHSWKAEATHVLEFPCPLA

>Glyma.09G190700|Gmax_Wm82.a6.v1/1-431

MDYLKELEGKSVHDCYHHHQQQQIKMSKMASPIFVPGPVIVGAGPSGLAAAACLKQKGIIPSLILERAQCLASMWQFKTYDRLRLHLPKQFCQLPLMPFPKNLPSYPTKQQFLAYLKAYADHFDIKPVFSQTVVSAEFDHVCQLWRVKTRGVIKKEDTAEYVCQWLIVATGECAEEVVPQIEGMGEFEGQIVHTSKYKSGSMFCGKNVLVVGCGNSGMEVCLDLCNHNARPSLVVRDTVHILPQQMLGKSTFGLSMFLLKWFPIRFVDQFLLLMSHLMLGDTAQFGLRRPKLGPLELKNLYGKTPVLDVGTLTQIKNGKIKVCRGIKRLARNAVEFVDGKVENFDAMVLATGYKSNVPSWLKGSDMFSEKDGFPRKPFPNGWKGENGLYAVGFTKRGLLGASIDAKRIAEDIEHSWKAEAKHVLEFPRPLA

>KAI5441892.1|Pisum_sativum/1-419

MLMNYLKELECKMVHDHQKKDKMVTSILVQGPIIVGAGPSGLAAAACLKQKGIQSLILERANCLASMWQLKTYDRLKLHLPKQFCQLPLMPFPKGLPSYPTKQQFLSYLKAYANHFDINPIFGKQVVNAEFDLTCGVWRVKTQEIIMKKCIVIEYVCQWLIVASGENAEEVMPSIEGMEQFQGPILHTSLYKSGSMFCGKNVLVVGCGNSGMEVCLDLCNHNAHPSLVVRDTVHILPQQIFGKSTFGLSMWLLKWFSVHFVDQFLLLMSYFILGDTSQYGIQRPKIGPLELKNLYGKTPVLDVGTVAQIKTGKIKVCKGIKRLAHNAVEFVDGKVENFDAIILATGYKSNVPSWLKGSDMFSEKDGFPRKPFPNGWKGEKGLYAVGFTKRGLLGSSIDAKRIAEDIEHSWKALKAKPLA

>Glyma.08G038600|Gmax_Wm82.a6.v1/1-418

MLNMEYLKEVEGKSVHDYVEVKMGKMTKPISVAGPVIVGAGPSGLAAAACLKQKGIPSLILERADCLASMWQLKTYDRLCLHLPKQFCQLPLMPFPQNFPSYPTKQQFLAYLKAYADHFDIKPALSKTVISANFDHGCGYWRVKTQGLKKEETEYVCQWLIVATGENAEEVVPQIEGMSEFEGPILHTSSYKSGSMFGGKNVLVVGCGNSGMEVCLDLCNHDARPSLVVRDTVHILPQQMFGKSTFGLSMSLLKWFPMRLVDKFLLLMSHLILGDTAQFGLNRPKIGPLELKNLCGKTPVLDVGTLAHIKSGKIKVCRGIKQLAKHKVEFVDGKTENFDVIIMATGYKSNVPTWLKGSNMFCEKDGLPRKDFPNGWKGENGLYAVGFSKRGLLGASIHSKRTAEDIEHCWKAANTTRV

>Glyma.05G231100|Gmax_Wm82.a6.v1/1-414

MLYMEYLKEVEGKTVHDYLEVKMGKMTKPIGVEGPVIVGAGPSGLAAAACLKQKGIPSLILERDDCLASMWQLKTYDRLCLHLPKQFCQLPLMPFPQNFPSYPTKQQFLAYLKAYADHFDIKPALSKTVISANFDHRCGYWRVKTQGVKKEETEYVCQWLIVATGENAEEVVPQIEGMSEFEGPILHTSSYKSGSMFCGKNVLVVGCGNSGMEVCLDLCNHHARPSLVVRDTVHILPQQMFGKSTFGLSLSLLNWFPMRLVDKFLLLMSHLILGDTAQFGLNRPKIGPLELKNLCGKTPVLDFGTLAHIKSGKIKVCRGIKQLAQHKAEFVDGKIEDFDVIILATGYKSNVPTWLKGSDMFCEKDGLPRKPFPNGWRGENGLYAVGFTKRGLLGASFDAKRIAGDIEHCWKAAN

>Qrob_P0219550.2|Quercus_robur/1-425

MAYMKEVEGKRVHDPLHEKMAKTRCKLVRGPVIVGAGPSGLAAAACLKQREVPSLILERADCIASTWQHRAYDRLRLHLPKQFCELPLMKFPKNFPTYPTKEDFVSYLQDYADHFDIQPVFNKTVVSAEFDQICGFWRVTTTGSEKEVTEYVCQWLIVATGENAEEVVPQFEGMNQFDGTIAHTSVYKSGEKYSGKSVLVVGCGNSGMEVCLDLCGNNARPSLVVRDSVHVLPQQMLGTSTFGLSMWLLNWLPTRLVDHFLLLMSRLMLGDTSRFGLNRPEIGPLELKSKSGKTPVLDVGTLAKIRTGNIKVCKAVKRLTRHAVEFVDGKVENFDAIILATGYKSNVPSWLKESDMFSNKDGLPRKPFPNGWKGEGGLYSVGFTKRGLLGTSLDARKIAEDIEPLWKRSLINSKQIVFVSVTDCL

>GWHPBFSI005308|Chloranthus_spicatus/1-428

MACWSELEGKRVHDRYLDDVEEKMKPSRRVYVVSGPIIVGAGPSGLAVAACLRQRGIESLILERANCIASLWQLKTYDRLCLHLPKKFCELPLMPFPSNLPKYPTKEHFIGYLESYAKKFDIQPRFDESVVSAEYDKSIGYWRVKTTAGWKEEEIEYLCRWLVVATGENSDAVVPEIDGIGDFKGKIVHTSLYKSGQKFQGKRVLVVGCGNSGMEVCLDLCNYNASPSLVVRDKVHVLPREMLGKSTFDLAMWFLKWFPVHVVDRFLLLLAWIMLGDTAQLGLDRPFLGPLELKNLSGKTPVLDVGTLDKIRAGEVKVCPAIRRFTDHGAEFIDNRVEDYDAVILATGYKSNVPSWLKERNFFSEKDGLPRNPFPNGWKGECGLYAVGFTKRGLMGASMDATRISQDIERCKQAEASLMAFECLKSSL

>FvH4_2g36220|Fvesca_v4.0.a2/1-435

MDYTTIQGKQSLDDPSLIHKMNNLSSPPRCVVCVAGPVIVGAGPSGLATAACLKNKGVPSVILERSNCLASLWQLKTYDRLRLHLPKQFCELPFVPFPSDFPTYPTKQQFIRYLDDYATKFDIQPRFNETVLTAQYDPAVGFWRVRTAGSENGVETEYVTRWLIAATGENAEALVPKLEGIMEFGGPIRHTSLYKTGEEFRGKKVLVVGCGNSGMEVCLDLCNHNARPSLVVRDTVHVLPREMLGKSTFGLSMLLLKWLPIRLVDRLLLVASRLLLGNTSQLGLVRPKLGPLELKNLSGKTPVLDVGTLAKIRTGDIQVRPAIKRLKRHRAVEFVDGRTENFDAIVLATGYKSNVPSWLKEGEMFSKEDGLPKLPFPNGWKGERGLYAVGFTKRGLLGASMDAKRIAEDIERCWKAEAKHSTPFTRSHLPLSSSP

>Cucsa.161450|Csativus_v1.0/1-434

MSTYLRELEGKLSNDPFFTEKMNGGGGVDGNVGMRRVWIPGPVIVGAGPSGLAAAACLKQRGVPSVILERSNCIGSLWQLKTYDRLRLHLPKQFCELPFMGFPVEFPTYPSKQQFVKYLEDYAERFDIRPRFNETVIEAEYDRTLGFWRVKSKRGRSEETEYVSRWLIVATGENAEAVVPELDGMDVFGGSITHTSLYRSGEEFRGKKVLVVGCGNSGMEVCLDLCEHSATTYLVVRDTVHVLPREMLGRSTFGLSMWLLKWFPIRLVDAFLLMVSRFILGDTARFGLDRPIMGPLRLKNSCGKTPVLDVGTLAKIRSGHIKVRPSIKRLKRQAVEFVDGKTERFDSIILATGYRSNVPSWLKEGEMFGKEDGMPRMPFPKGWKGESGLYAVGFTKRGLLGTSMDAKRIAEDIERCWKADAKLCTPTMQSPPST

>Kalax.0478s0031|Klaxiflora_v1.1/1-444

MELAWTEIQGKIAHDPVIIDKMICSSASRRVYVPGPVIVGAGPSGLAMSACLREKGVPSIILERSNCIASLWQLKTYDRLRLHLPKQFCELPLMSFPKNFPTYPSKQQFIKYLENYAEAFGIKPLFNETVKLAEFDKVLGMWRVRSESKSSGSGFDGELMVTEYVCRWLVVATGENAEVMIPEIEGMEEFGSAGGKIVHTSSYKSGEIFKGKKVLVVGCGNSGMEVCLDLCHHSARPYLVVRDTVHVLPQEMLGKSTFGLSMCLLKWLPVRLVDQFLLIMSWLVFGDTSRLGLVRPKLGPLELKNVAGKTPVLDVGTLAKIKSGDIKVCPAIRRLKPHAVEFIDGRTEKLDSIILATGYKSNVPSWLKEGNMFSDKDGFPKKPFPNGWKGDNWLYAVGFTKRGLLGASMDARRIAEDIAQCWKAESKHHLTSYPSSVVLLPQTT

>Kalax.0046s0062|Klaxiflora_v1.1/1-444

MELAWTEIQGKIAHDPVIIDKMICSSASRRVYVPGPVIVGAGPSGLAMSACLREKGVPSIILERSNCIASLWQLKTYDRLRLHLPKQFCELPLMSFPKNFPTYPSKQQFIKYLENYAEAFGIKPLFNETVKLAEFDKVLGMWRVRSESKSSGSGFDGELMVTEYVCRWLVVATGENAEVMIPEIEGMEEFGSAGGKIVHTSSYKSGEIFKGKKVLVVGCGNSGMEVCLDLCHHSARPYLVVRDTVHVLPQEMLGKSTFGLSMCLLKWLPVRLVDQFLLIMSWLVFGDTSRLGLVRPKLGPLELKNVAGKTPVLDVGTLAKIKSGDIKVCPAIRRLKPHAVEFIDGRTEKLDSIILATGYKSNVPSWLKEGNMFSDKDGFPKKPFPNGWKGDNGLYAVGFTKRGLLGASMDARRIAEDIAQCWKAESKHHLTSYPSSVVLLPQTT

>Kalax.0419s0014|Klaxiflora_v1.1/1-441

MEWACTEIQGKGAHDPVIVDKMICSSARRVYVPGPVIVGAGPSGLAMSACLREKGLPSIILERSNCIASLWQLKTYDRLCLHLPKQFCELPLMSFPKNFPIYPSKQQFVKYLENYAEAFDVKPLFNETVKLAEFDTVLGMWRVRSESKSSGSGFTNELMVTEYVCRWLVVATGENAEVMIPDIEGMGEFASAGGKVVHTGSYKSGEMFKGRKVLVVGCGNSGMEVCLDLCHHGARPYLVVRDAVHVLPREMLGKSTFGLSMWLLKWLPMPLVDRFLLFMSWLVLGDTSKLGLVRPKLGPLELKNVAGKTPVLDVGTLTKIKSGDIKVCPAIRRLKPHAVEFIDGRREKLDVIILATGYRSNVPSWLKEGNMFSDKDGFPKKPFPDGWKGDNGLYAVGFTNRGLLGASMDARRIAEDIAQCWKAESKHHLTSYSSSILLPHT

>Kalax.0112s0041|Klaxiflora_v1.1/1-441

MEWAWTEIQGKGAHDAVIVDKMICSSARRVYVPGPVIVGAGPSGLAMSACLREKGVPSIILERSNCIASLWQLKTYDRLCLHLPKQFCELPLMSFPKNFPIYPSKQQFVKYLENYAEAFGVKPLFNETVKLAEFDTVLGMWRVRSESKSSGSGFTNELMVTEYVCRWLVVATGENAEVMIPDIEGMGEFASAGGKVVHTGSYKSGEMFKGRKVLVVGCGNSGMEVCLDLCHHGARPYLVVRDAVHVLPREMLGKSTFGLSMWLLKWLPMPLVDRFLLFMSWLVLGDTSKLGLVRPKLGPLELKNVAGKTPVLDVGTLTKIKSGDIKVCPAIRRLKPHAVEFIDGRREKLDVIILATGYRSNVPSWLKEGNMFSDKDGFPKKPFPDGWKGDNGLYAVGFTNRGLLGASMDARRIAEDIAQCWKAESKHHLTSYSSSILLPHT

>AUR62043360|Cquinoa_v1.0/1-443

MEYFYNNLREMEGKLANDPQRNTKTTTNTRCILVSGPVIVGAGPSGLAVAACLKEKGVPSLILERSNCIASLWQLKTYDRLRLHLPKQFCELPLMPFPQDFPMYPTKRQFIEYLEDYATKFDIKPLFNQTVEKAEFDQTLGFWRVRTSTTCSTSGRGTIEKGGGDEEIVFTTEYVCRWLVVATGENAEVVMPELEGLKEFGKQVVHTSSYKSGETFRGKRVLVVGSGNSGMEVCLDLCHHNAMPSIVVRDTVHLLPREMFGRSTFGLSMWLLKWLPITLVDRLMLIIAWLIFGDTSKLGLARPKTGPLELKNLTGKTPVLDVGTFDKIKRGDIKVCPGIRRLMHHAVEFVDGSVENFDAIILATGYKSNVPHWLKEREMFSEQDGLPRRPFPNGWKGESGLYAVGFTKRGLLGASMDAKRIAADIGQCWNSESRHLTTFDRSH

>AUR62023030|Cquinoa_v1.0/1-443

MEYFYNNLREMEGKLANDPQRNTKTTTNTRCILVSGPVIVGAGPSGLAVAACLKEKGVPSLILERSNCIASLWQLKTYDRLRLHLPKQFCELPLMPFPQDFPMYPTKRQFIEYLEDYATKFDIKPLFNQTVEKAEFDQTLGFWRVRTSTTCSTSGRGTIEKGGGDEEIVFTTEYVCRWLVVATGENAEVVMPELEGLKEFGKQVVHTSSYKSGETFRGKRVLVVGSGNSGMEVCLDLCHHNAMPSIVVRDTVHLLPREMFGRSTFGLSMWLLKWLPITLVDRLMLIIAWLIFGDTSKLGLARPKTGPLELKNLTGKTPVLDVGTFDKIKRGDIKVCPGIRRLMHHAVEFVDGSVENFDAIILATGYKSNVPHWLKEREMFSEQDGLPRRPFPNGWKGESGLYAVGFTKRGLLGASMDAKRIAADIGQCWNSESRHLTTFDRSH

>Sapof.08G200800|Sofficinalis_v1.1/1-431

MVSHSYNHMREIEGKLANDPHQLDKNTSKTTRYVLVSGPVIVGAGPSGLAVAACLKEKGVPSIVLERASCIASLWQLKTYDRLRLHLPKKFCELPLLPFPTHYPRYPNKGQFVSYLEGYSRKFDINPNFNETVDRAEFDQTLGFWRVKTSKKDETAPVGVVMTEYVTRWLIVATGENAEVAVPEFDGLEEYEGIIVHTSLYKSGEVFRDKKVLVVGSGNSGMEVCLDLCHHNAMPSIVVRDKVHVLPREMFGKSTFGLSMWMLKWLPINLVDKLLLVIAWFILGDTSKLGLTRPLMGPLQLKNLSGKTPVLDVGTLDKIKCGDIKVCPGIKRLMTHKNVEFVDGRVENFDAIILATGYKSNVPYWLKEREMFSREDGLPRKPFPNGWKGDNGLYAVGFTKRGLLGASMDAMKIAEDIEQCWISTSKPSYYL

>Sapof.03G151300|Sofficinalis_v1.1/1-433

MVVYYNNLSEMEGKLANDPHHMINKKSSKTTRYVLVSGPVIVGAGPSGLAVAACLKDKGVPSLVLERSNCIASLWKLKTYDRLRLHLPKSFCELPLMPFPKDFPTYPTKGQFIGYLEDYARKFDINPIFNESVDQAEFDQTLGFWRIRTSKKGGVKGDEGVVVMTEYVCRWVIVATGENAEVVEPEFDGMGDFKGSIVHTSMYKSGEDFRGKKVLVVGSGNSGMEVCLDLCHHNALPSIVVRDRVHVLPREMFGRSTFGLSMWLLKWLPISVVDKLLLIIAWFKLGDTSKLGLIRPHLGPLQLKKLSGKTPVLDVGTLDKIKRGHIKVCPGIKRLLMNNKVEFINGRIENFDAIILATGYKSNVPYWLKEREMFSKEDGLPRRPFPNGWKGDHGLYAVGFTKHGLLGASMDAKRIAEDIGQCWFSESKPSYYL

>Aqcoe7G399500|Acoerulea_v3.1/1-422

MDYLRELESKRVHDSFDETMKKVGRCVCVPGPIIVGAGPSGLAVAACLKEKGVPCVIIERANCIASLWKLKTYNRLHLHLPKKYCELPFMPFPAKFPTYPTKEQFVEYLEDYVKKFDLEPQFNQTVESANYDHSLGLWRVKTVGVKEFRYSRWLVVATGENSEAVVPEIEGINEFEGPVVHTSSYKSGETFSGKKVLVVGCGNSGMEICLDLCNNNAQPSIVVRDTLHILPREMLGRSTFGVSMWLLKLFPICLVDRILLFISWFILGDTSRIGLDRPNIGPLQLKCTSGKTPVLDVGTLAKIKTGEIKVRPGVKRTTCRTVEFTDGSAEEYDAIVLATGYRSNVPSWLKDKEMFSEEDGLPRRSFPHGWKGEYGLYAVGFTKRGLLGSSMDAKRIAQDIEQSWKAEVKPFTAFTRTPAPAS

>Pso02G37200.0|Papaver_somniferum/1-436

MDFLKEMEGKRVHDSFGKNTTKNVRHVCVPGPVIVGAGPSGLAAAACLSDRGVPSVIIERSNCIASLWQLKSYTRLRLHLPKEFCELPLMPFPAEFPTYPSKQQFVDYLETYTKKFDLKPHFNESVDCAEYDHNLGLWRVKTTGLHNEEMEYVTRWLIVATGENAEEIVPVIEGMDEFEGPIVHTSVYKSGNVFRGKRVLVVGCGNSGMEVCLDLCNSDASPSLVVRNKAHILPREMLGVSTFGLSMWLLKWLHVRYVDQLLLFLSWFFLGDTSRLGLDRPKIGPIELKMATGKTPVLDVGTLAEIRSGSIKVCPGIKQISTHKVHFVNGRAEDFDAIILATGYKSNVPSWLKDTNLFSEEDGLPKKPFPNGWKGEYGLYSVGFTKRGLLGTSMDAKKIAQDIELLWKADSKHYLSALFYSPSSSPPPPPPPSLGH

>Pso02G55950.0|Papaver_somniferum/1-434

MDFLKEMEGKRVHDPFGKNTIKNVRHVCVPGPVIVGAGPSGLAAAACLSDRGVPSVIIERSNCIASLWQLKSYTRLRLHLPKEFCELPLMPFPAEFPTYPSKQQFVDYLETYTKKFDLKPHFNESVDCAEYDHSLGLWRVKTKGSHNEEMEYVTRWLIVATGENAEEIVPVIAGMDEFEGPIVHTSVYKSGNVFRGKRVLVVGCGNSGMEVCLDLCNSDASPSLVVRNKAHILPREMLGVSTFGLSMWLLKWLHVRYVDQLLLFLSWFFLGDTSRLGLDRPKIGPIELKMATGKTPVLDVGTLAEIRSGSIKVCPGINQISTHKVHFVNGRAEDFDAIILATGYKSNVPSWLKDTNLFSEKDGLPKKPFPNGWKGEYGLYSVGFTKRGLLGTSMDAKKIAQDIELLWKADSKHYLSALFYSPSLSPPPPPSLGH

>Qrob_P0044800.2|Quercus_robur/1-431

MGSYYRTEIEGKQAHDTLFKEKTNSNSNKSSPFEYVDEPVIIGAGPSGLAVAASLKKKGVPSVVLERSDCIASLWQRKTYDRLRVHLPKQFCELPFMGFPNDFPTYPTKEQFIEYLEDYANEFEIKPRFNKKVAQAEYDCTLGLWRVKSEGLNGEMTEIACRWLVAATGENAEPVTPKIEGEDEFGGSIKHTSLYKCGGEFSSQKVLVVGCGNSGMEVCLDLCNHGAKPSIVVRDSVHILPREMLGKSTFGLSMWLLKWLPMRLVDRLLLIVSWLILGDTSRFGLDRPSLGPLELKNLSGKTPVLDVGTLAKIKSGDIKVLPSIKRLKRHAVEFVDGKMENFEAIILATGYRSNVPSWLKEGQMFSEKDGLPRRPFPNGWKGECGLYAVGFTKRGILGASMDAKRIAEDIERYWKVEAKHKSKTFDSSPLS

>CKAN_00588900|Ckanehirae_v3/1-418

MEHSRDRELEGKQAHDRLEMKGARGVPERVHGPVIVGAGPSGLASAACLKVRGISSVILERANCIASLWQLKTYDRLSLHLPKQFCELPLMPFPESFPTYPTKQQFLAYLVEYAKRFDLKPVFNETVECAEYDAKFMCWRVRTEKTEYLCSWLVVATGENAEEVIPKIDGMVDFAGPVIHTSKYRSGDAFQCMKVLVVGCGNSGMEVCLDLCNNGANPSIVVRDTVHVLPREILGRSTFGLSMWLLKWFPVQMVDRFLLLVARVMLGDTAQVGLDRPHLGPLQLKNMSGKTPVLDVGTLAKIRAGEIKVYPAIKRLTRRGAVFVDGRASAFDAVILATGYRSNVPSWLKERNFFSDKDGLPRKPFPNGWKGDCGLYAVGFTKRGLLGASMDAKRIAQDIERCCKPRLSNSKPPSGWHK

>GSMUA_Achr9G05490_001|Macuminata_v1/1-417

MERWREAEGKTLHDPLDEAERSIWVPGSIIVGAGPSGLAVAACLEAKGVPSMILERSNCIASLWQLKTYDRLRLHLPKRFCQLPLAPFPACFPTYPTKQQFVAYLEAYARRFHIRPCFNQTVVSAEYDGRVKLWRVRAVRAGNDKAAEYVSRWLVVATGENAEAAVPDIDGMSIFKGPIIHTSSYKSGDEFQGKRVLVIGCGNSGMEVCLDLCNHSVRPRIVVRESVHILPREMLGRSTFGLCMWLLKWLPMRTVDRILLLVSRVMLGDTQRYGLRRPRLGPLELKSLSGKTPVLDVGALAKIKSGDIKVCPAVKRLTGHGAEFVDGRSEDFDAIILATGYKSNVASWLKEREFFSDKDGFPRKVFPDSWKGEQGLYAVGFTRQGLMGTSVDAKRIAHDIKQCWMAEPKQRMLPSQT

>Aco010742|Acomosus_v3/1-448

MDCWRESEGKRVHDPLFYHFPTTDNNNTDDDKDDDDKESESDSDCDCVAVEGPIIVGAGPSGLAAAACLKEKGIPSLILERSDCIASLWQRKTYDRLRLHLPKQFCQLPLMPFPCGLPAYPTKQQFVAYLESYAARFAIRPAFGVAVADASFDGQFWRVRAHCCRPAAGKKTREYVGRWLVVATGENAEPVVPEIAGIGEFSGEVLHTSAYRSGEAYRRKRVLVVGCGNSGMEVCLDLCNHNAQPSVVVRDTVHVLPREMLGSSTFGLSMWLLKWLPMRAVDRVLLLVARLMLGDTARLGLPRPRLGPLELKSLSGKTPVLDVGTLAKIRSGDIKVFPAIRRFTEHGAEFVDGRSEDFDAVVLATGYRSNVPSWLMEREMFSEKDGLPKKPFPNGWKGQHGLYAVGFTKRGLLGTSMDAARIADDIEQCWKVQKASQLVMSFSLPVIN

>XP_020574968.1|Phalaenopsis_equestris/1-405

MACFREIEGKRTHDPSGSPIPVSGPIIIGAGPSGLAVAACLKAKGIPSLVIERSHCIASLWQLKSYDRLRLHLPKSFCELPLMRFPPHFPTYPTKEQFLSHLKSYAANFNIHPLFGQRVISAEYDSRGYWRVRTEAMGRRREYMSRWLVAATGENAEEVLPDIAGMMTEFNGEVVHTSGYRSGEVYKGKKVLVVGCGNSGMEVSLDLCNCGARPGIVVRDSVHMLPRDMFGQSTFGLSMCLLKWLPIRIIDRILLFIARLKLGDTARLGLRRPKLGPLELKGFSGKTPVLDVGTLAKIKSGDIKVFPAVKRFTAKGAEFVDGRSEDFEAVILATGYRSNLPIWLKEKDFFSEEDGLPVRPFPNGWKGERGLYAVGFTKKGLLGASMDATRIALHIERCWKLEATQ

>XP_020585723.1|Phalaenopsis_equestris/1-408

MNFYNYSQEMEAKLAHDFIHVPGPIIVGAGPSGLAVAACLNSKGVPSLIIERSHCIASLWQLKSYDRLRLHLPKKFCELPLMPFPTEFPTYATKEQFVGYLENYAASFEVRPTFGESVVRAEFDERLGFWRVRTVREGSGWEKEYVSRWLVVATGENAEEVVPAIEGLSEFEGTVVHTSGYRSGEAFRGKKVVVVGCGNSGMEVCLDLCNYDAIPMIVVRDAVHILPREMLGWSTFGLCMWLLKWLPVQMVDRLLLFVTGLMVGDTARFGLRRPELGPLELKAITGKTPVLDVGTLDKIKSGDIKVYPGVKRFTSKGAEFIDGKSEDFDAVILATGYRSNVPSWLKESEFFSKVDGLPKRPFPNGWKGERGLYAVGFTKRGLLGASIDATKIAQDIALCWNLEHNQEI

>Aco004396|Acomosus_v3/1-403

MDCNDDQESNGESLRDHRCCVVADGAIIVGAGPSGLAVAACLKAKGVPCLVLERARCVASLWQLKTYDRLRLHLPRKFCELPFMPFPRGLPTYPTKKQFVSYLEAYAEAFGIRPEFDATVGSAEYDRGVWRVIAEGSEGERKEYVSRWLVVATGENAEAVAPRIEGMGEFKGSVVHTSMYKSGDVYRGKRILVVGCGNSGMEVCLDLFDHNARPSIVVRDTVHVLPREMLGISTFGLSMWLLKWFPIRAVDRLLLLLARLALGDTARLGLNRPWLGPLELKSLTGKTPVLDIGTLAKIRSGHIKVFPAIRRFTKNGAEFVDGRLEDFDAVVLATGYKSNVPAWLKEQGLFSENDGLPKRPFPNGWKGENGLYAVGFTKRGLLGASIDATRIANDIERIWKTQH

>evm.TU.AsparagusV1_01.1621|Aofficinalis_V1.1/1-427

MAMSMSMQWRETQGKQLHDPFFLKSAADTCRFCPGPIIVGAGPSGLAVAACLKEKGIPSVVIERSTCIASLWQLKTYDRLRLHLPKQFCELPLKAFPKDFPTYPTKQQFLAYLESYAKEFDIKPAFGESVESAEFDAGMGMWRVRTVLFGKEIHRTYLGRWLVVATGENAEAVVPEIEGMEEFEGTRIHTSVYRRGNEFEGKKVLVVGCGNSGMEVCLDLCNHNARPSIVVRDSVHVLPQEMLGRSTFGLSMSLMSWFPVQTVDRFLLFISWLMLGDTAKLGIKRPDLGPLALKSISGKTPVLDVGTLAKIKSGDIKVCPGIQRLTRKGAEFVDGISEDFDAIILATGYRSNVPSWLKEREFFSEKDGMPRRPFPNGWKGENGLYAVGFTKRGLLGASMDARRIADDIEQCFEVEANQFMVFSCSPN

>Asa5G01036.1|Allium_sativum/1-400

MQWSKESLVKQIHDPFFTKTPKPSCNNPIIVGAGPSGLAVAACLKQKGIQSTIIERSSCIAALWQHRTYDRLRLHLPKQFCELPLMPFPPNYPKYPSKQEFISYLEAYRKKFDIRPVFNQSVVSAEFDKGIGLWRVVTSKNVYVSRWLVVATGENAEVVVPDIEGMDEFEGRKIHVSAYRNGEEFEGKKVLVVGCGNSGMEVCLDLCNHHARPSIVVRDSVHVLPQEVLGLSTFGLSMSLLKWLPVDTVDKILLFMSWLMFGDIEKLGLRRPKLGPLQLKSMSGKTPVLDVGTLDKIKSGDIKVRPAIRRLVAHGVNFVDGKSENYDAIIFATGYKSNVPAWLKDRELFSNEDGYPKKPFPKGWKGEHGLYAVGFTKRGLLGASMDARRIADDVEKWFLN

>AUR62010996|Cquinoa_v1.0/1-154

MLGDTAQFGINRPAIGPLEYKSITGKTPVLDIGTLTKVKDGTVKVFPAIKRLTRQGAEFVDGRMENFDAIILATGFKSNVPSWLKDTTMFSEKDGYPVKPFPEGWKGENGLYAVGFTKRGLLGASHDAIMIAEDIDRDWKTKAKQLMQQRSIFE

>AUR62021506|Cquinoa_v1.0/1-420

MDPLKEMAAKLAQDPHPQSPSTRRVWVPGPVIVGAGPSGLAVAACLKERGIPRLILERAQCLGSLWKLHTYDRLRLHLPKQFCQLPHYPFPQSFPTYPTKEQFITYLDSYATHFNLRPMFGRSVVKAQYDTRHGLWRVLVSGPKKEVVEYVCQWLVVATGENAEPYMADIVGREEYKGVIIHSSEYKSGEEYSDKKVMVVGCGNSGMELCLDLCNHNAYPSLVVRDSVHILPQQMLGTSTFGLSMCLLKWFPLKLVDQFLLLISRLMLGDTAQFGINRPAIGPLEYKSVSGKTPVLDIGTLSKVKDGTVKVFPAIKRLTRQGAEFVDGRMENFDAIIFATGFKSNVPSWLKDTTMFSEKDGYPVKPFPEGWKGENGLYAVGFTKRGLLGASHDAIMIAEDIDRDWKTKAKQLMQQRSISE

>Sapof.08G055400|Sofficinalis_v1.1/1-421

MDPMKEMEAKLANDPAQKDSVRRVWVPGPVIVGAGPSGLAVAACLKEKGIIPGLILERSECVGSLWKYRTYDRLRLHLPKQFCQLPYLKFPSHFPTYPTKQQFITYLDSYAKEFNLRPAFGWAVVRAEYDPRHGLWRVRVQAQAGPGQKREEEVEYVCQWLVVATGENAEAYVPKIVGQEEFVGHMIHSSEYRSGEEYWGKKVLVVGCGNSGMEVCLDLCNHNAFPSLVVRDSVHVLPQQIFGLSIFGLSMFLLKWLPIKLVDKLLLLISRLILGETAKLGLIRPAIGPLKYKNISGKTPVLDIGTLAKIKAGFIKIFPAIRKLTRYGAQFVDGRIEHFDTIILATGYKSNVPSWLKGTTMFSEKDGYPVKSFSKGWKGENGLYSVGFTKRGLLGASQDAMRVAEDIARDWNNKTKPLMNQ

>evm.TU.Scaffold_671.932|Carabica_v0.5/1-403

MMSRSTRRRLWVPGSVIVGAGPSGLATAACLKEKGIPSLILERANCIASLWQHKTYDRLRLHLPKNFCELPLLPFPKDFPTYPTKQQFISYLESYAKHFDLEPAYNNTVVSAEFDEGCGFWRVRTGVGLKGDQETEYVCQWLIVATGENAEEIVPDFEGMDKFLGPVVHTSSYRRGDCFQGKSVLVVGCGNSGMEVSLDLCNHNACPSLVVRDSVHVLPQEMLGRSTFGLAMQLLEWFPVRLVDRFLLVISRIMLGNTAQLGLPRPKIGPLELKSITGKTPVLDVGTLAQIRTGKIKVVRGIKRLMRNAVEFVDGMVENFDAIILATGYRSTAPAWLKGKNLSSEEDGFPSKPFPDGWKGHNGLYAVGFTKRGLLGTSMDARRIAEDIEKQWKVRAVQFQGLC

>Eucgr.C01042|Egrandis_v2.0/1-425

MDYLREMEVKRVQDPLVANMKEPATCVNGPLIVGAGPSGLATAACLKERGVRSLILERTDCIGSLWQHKTYDRLRLHLPKQFCELPHMPFPKDFPTYPTKQQFLAYLYLYTKRFDLRPVFNQTVTSAEFDQSSRCWRVKSSGHNGEETEYLSRWLIVATGENAEELVPQFEGMSDFGGQILHTSSYKSGEIFCGKRVLVIGCGNSGMEVSLDLCNFNACPSLAVRDSVHVLPQEMLGRSTFGMSMWLLKWLPISLVDWFLLLMSYFILGDTTSYGLNRPKVGPLQLKSMSGKTPVLDVGALAKIKSGHIKVFRGIKRIRRHAVEFVDGKSETFDAIILATGYRSNVPSWLKEGDMFSEEDGFPKKPFPNGWKGKNGLYSVGFTKRGLLGASLDARSISQDIGQLWRLDVEGMHFRALSHATPLHP

>GWHPCBIR014844|Santalum_yasi/1-429

MEYSYTNPPKSELECKLLHDEMAPPRPQRVVIVPGPVIVGAGPSGLAVAACLKQRGVPSVVLERANCIASLWQLKAYDRLRLHLPKQFCELPILGFPPDFPTYPTKEQFVEYLEGYAGEFGIRPVFRETVARAEYDEGEGMWRVRTVGPEGAETEWVSRWVVAATGENAEAVVPPEMEKGGFMGPIVHTSGYRSGDAYEGKRVLVVGCGNSGMEVCLDLCHHKANPSIVVRDTVHVLPREMLGRSTFGLSMWLLKWLPVSVVDRLLLVVARFALGDTARLGLHRPKLGPLQLKNLHGKTPVLDVGTLANIKSGNIKVFPGIKGLNRHSVEFIDGQTEDFDAIILATGYKSNVPSWLKENEMFSKKDGFPRKPFPNGWKGECGLYAVGFTKRGILGASMDAKRIAEDVARSWEMPASPDGFSLSRKQRFD

>gene_prefix00720.t1_1|Ceratophyllum_demersum/1-436

MGSMIESRGKRLHDRLCYSEQNNKMIAGKCFWVPGAVIVGAGPSGLAVAACLKDRGVPSEIIERDNCIASLWQHKTYDRLRLHLPKQFCELPLMPFPSNFPTYPTKQQFISYLESYAQRFDIRPRFNERVDYAAYDQNLGLWRLRTLRTKDKNDNEVEETEYVCRWMVVATGENAEEVIPKIEGTDDFLGPVIHSSSYKTGESFYGKRVLVVGCGNSGMEVCLDLCNYNARPSLVVREALHILPREMLGRSTFGLSMWLLKWLPIRWVDRFLLFISRFMLGDTTPFGLVRPSIGPLELKNRTGKTPVLDVGTLTKIRNGDIKIYPAIKKVMRHGVEFVDERVEAFDAIVFATGYRSNVPTWLKEKEMFSEIDGLPKKPFPNGWKGRCGLYAVGFTKRGLLGASVDARRIALDIESSWKADAKHLSHSKPLFTSCHS

>Nycol.A02312|Ncolorata_v1.2b/1-433

MDCLREQQGKRLHDGFFEKRSKSRSRFVWVPGPLIVGAGPSGLATAACLKERGVPSLILERAHCIASLWQLKTYDRLRLHLPKNFCELPLFPFPPEFPTYPTKEQFIRYLESYAKHFGLRPVFNETVVSAEYDRSFGFWRVKGVSCEEGGCCKPQETEYVCRWLVVATGENAEAVIPDIPGAADFSGPLVHTSCYKSGAAFRRKRVLVVGCGNSGMEVCLDLCNHNAYPSLVVRKAVHVLPREMLGTSTFGLSMFLLKWFSVKVVDKFLLLMTWLIMGSTDTLGLGRPQMGPLELKNKSGKTPVLDVGAFAKIKSGDIKVRPEIKRLTANGAEFVDGKTEELDAIILATGYRSNVPSWLKEGDMFSEKDGLPRRPFPEGWKGENGLYAVGFTKRGILGACMDARRIAQEIDYSWKAESKPIFLATATPTSLPY

>g21250.t1_1|Euryale_ferox/1-432

MDRLREQQGKRLHDGLFDKRNKSRGRFVWVPGPLIVGAGPSGLATAACLKERGVPSLILERAHCIASLWQLKTYDRLCLHLPKNFCELPLFPFPPDFPTYPTKEQFIRYLESYAKRFGLRPVFNETVVSAEYDRSFGFWRVRGVRCTDGGGCKPEETEYVSRWLVVATGENAEVVIPDIPGAANFSGPVVHTSCYKSGVPFRRKRVLVVGCGNSGMEVCLDLCNHNAYPSLVVRKAVHVLPREMLGTSTFRLSMFLLKWFSVKAVDKLLLLMTWLIMGSTEKLGLGRPQMGPLELKNTHGKTPVLDVGAFAKIKSGNIKVRPAIKRLTANGAEFVDGRTEELDAIILATGYRSNVPSWLKEGDMFSEKDGLPKRPFPEGWKGENGLYAVGFTKRGLLGACMDARRIAEDIDNSWKAEPKQIILATATPSLPY

>g15548.t1_1|Euryale_ferox/1-432

MDRLREQQGKRLHDGLFEKRSKSRSRFVWVPGPLIIGSGPSGLATAACLKERGVPSLIIERAHCIASLWQLKTYDRLRLHLPKNFCELPLFPFPPDFPTYPTKEQFIRYLESYAKHFDLLPVFNQTVVSAVYDRRFGFWRVKGVRCAEGGGCKPEETEYVSRWLVVATGENAEVVIPDIPGAADFSGPVVHTSCYKSGAPFRRKRVLVVGCGNSGLEVCLDLCNHNAYPSLVVRKAVHVLPREMLGTSTFGLSMFLLKWFSVKVVDKFLLLLTWLIMGSTEKLGLGRPQMGPLELKNTHGKTPVLDVGAFAKIKSGDVKVRPAIKRLTANGAEFVDGRIEELDAIILATGYRSNVPSWLKEGNMFSEKDGLPKRPFPEGWKGENGLYAVGFTKRGLLGASMDARRIAQDIYHCWKAESKQIILATATPSLPY

>XP_020580150.1|Phalaenopsis_equestris/1-426

MSSRDSNDVGAQFSDQKTPTILKLKLNEEAMKSSNSKRIWVPGPLIIGAGPSGLAIAACLKEKGVPSLILEKENCIASSWRMRTYERLKLHLPKHLCELPLMPFPSDFPVYPTKQQFISYLDSYIKQFSIRPFFGMEVWKAEYDKTIEFWRINAGDDWEFICRWLIVATGENATAKVPDIPGMAEFKGRILHTSSFKQGDDFNGERVLVIGCGNSGMEVCLDLCNNHARASMVVRDELHVLPREVLGISTFGLSMWLLRWLPMSAADCLLLLCARLFLGDTEKHGLRRPKIGPLKLKNSTGKTPVLDIGALSKIKSGQIKVVPGINKFFEKGVEFVDGKQEEFDAIIFATGYKSNVSSWLKEEEFFCKKNGIPRTQFPNSWRGKNGLYATGFTRRGLLGASMDAHRISEDIACQWNSKTKHLPMEL

>Aco003772|Acomosus_v3/1-400

MTSKETSTTKRLWWVPGPLIIGAGPSGLATAACLKDRGVPYLVLEKDCCIAASWKLRTYERLKLHLPKHFCELPLMPFPQHFPTYPTKQQFISYLECYAKRFAVEPLFGARVDRAERDASLGLWRVRAGESEFISRWLVVATGENAEAVTPEIGGVAEFRGRVMHTSCYRKGDDFTGEKVLVVGCGNSGMEVSLDLCNNGAHPSMVVRDKLHVLPREIFGISTFGLSVWLLKWLPTRYVDWFLLLCARITLGDTEKYGLKRPKVGPLQLKSTTGKTPVLDVGTLAKIKSGHIKVVPNINRFTDKGVEFVDGRKEEFNSVILATGYKSNVPSWLKDEEFFNKNDGFPKTRFPNSWKGENGLYATGFTQRGLMGASIDARKIAEDIATQWNSKPKHLPCGLL

>Acora.06G229000|Aamericanus_v1.1/1-394

MKQVWVPGPVIVGAGPSGLATAACLKEHGVPSLILERETCIASLWKLKTYDRLKLHLPKQFCELPLMPFPSDFPTYPTRQQFINYLNAYAQRFSIEPVFGTEVRLAEYDSNIGFWRVRTFDSEFICQWLVVATGENAEAFVPDIKGVSEFRGRVLHSSEYKSGADFSGEKVLVVGSGNSGMEICLDLCNNCAAASMVVRDTLHVLPREILERSTFSLSLWLLKFLPIQLVDSLLLLFSRLILGDTGKFGLKRPKIGPLQLKNTTGRTPVLDIGTFSKIKSGQIKVVPGILRLTARGAEFSDGRQAEFDSVILATGYRSNVPSWLKEGEFFNHENGYPKKPFPHSWRGQNGLYATGFNKRGLLGASMDARKIAEDIACQWNSGAKHLPLQLLFTS

>Litul.17G003500|Ltulipifera_YP108A_v1.1/1-394

MTLKPKRVLVPGPVIIGAGPSGLATAACLKENGVPSLILERENCIGSLWKMKTYDRLKLHLPKKFCELPLMPFPPQFPTYPTKQQFISYLEAYAKHFSIKPMFGVDVKRAEYDSSIGFWRVLANGSVFVCRWLIVATGENAEAVLPNIDGILDFQGPVLHTSSYKNGEDFRAEKVLVVGCGNSGMEICLDLCNNGALASLVVRDKLHILPRGMLGTSTFGLSMWLLRWLPIHLVDAFILFCSWLILGDTGRFGLQRPEIGPLQLKNTTGKTPVLDVGTLAKIKTGNIKVVPGIQRFTTKGVEFVDGVAAEFDAVVLATGYRSNVPSWLKEGDFFSKQDGFPKKPFPNGWKGDNGLYAVGFTKRGLLGASVDAQRVAEDIACLWNSETKHLRLEM

>Aqcoe5G196400|Acoerulea_v3.1/1-394

MNKELKRVWVPGPVIIGAGPSGLAAGACLKEKGVPSLILERENCLASLWKLKTYDRLRLHLPKKFCELPYMDFPPEFPTYPTKQQFLNYLNDYANMFSIKPIFEEEVRLAEYDSNIGFWFVQTNNFEFVCQWLIVATGENSEPVTPELPQISNFRGRILHTSRYKNGYEFEGEKVLVVGCGNLGMEISLDLCNSGAQALISVRNMVHILPREILGLSTFGLSMWLLKWIPLKLVDLFLLLSSLFILGDTQRFGFKRPKIGPLDIKNTIGKTPVLDVGCLAKIRTGKIKVVPGVKNFTADGAQFIDGRVEEFDSIILATGYRSNVPSWLKEEEFFSKKDGYPITPFPNSWKGNNGLYSVGFTKRGLLGASLDAHKIAEDIAQQWNLNAKYMHLAL

>Pso01G34200.0|Papaver_somniferum/1-394

MTTEIKRVWVSQPVIIGGGPSGLAVAACLKKKGLPSLILERENCAASLWRNRTYDRLRLHLPKNFCQLPYMEFPPDFPSYPTKEQYIEYLDAYVKHFSIEPIYEEHVQIAEYDSAMGFWLVKTNNAEFVCRWLIVATGENADPVIPEIDGISDFHGQVLHTSLYKNGDEFEGDKVLVLGCGNSGMEISLDLCNCGAQVSIVVRDTLHILPRDILGRSTFGVSMCLLKWFPIWLVDQFLLFCSRLILQDVRQLGFKRPELGPLELKIRTGKTPVLDVGTLAKIRTGQIKVVPGVKRFTNRGVEFVDGRVDEFDSVILATGYRTNVPSWLKEDEFFSKDDGYPRKPFPNGWKGKNGLYSVGFTKKGLLGASIDAQRVAEDISQQWKSETKHFHLRV

>Pso06G23540.0|Papaver_somniferum/1-394

MTTEIKRVWVSQPVIIGGGPSGLAVAACLKKRGLPSLILERENCAASLWRNRTYDRLRLHLPKNFCQLPYMEFPPDFPSYPTKEQYIEYLDAYVKHFSIEPIYEEHVQIAEYDSAMGFWLVKTNNAEFVCRWLIVATGENADPVIPEIDGISDFQGQILHTSLYKNGDEFEGDKVLVLGCGNSGMEISLDLCNCGAQVSIVVRDTLHILPRDILGRSTFGISMCLLKWFPIWLVDQFLLFCSRLILQDVRQLGFKRPELGPLELKIRTGKTPVLDVGTLAKIRTGQIKVVPGVKRFTNKGVEFVDGRVDEFDSVILATGYRTNVPSWLKEDEFFSKEDGYPRKPFPNGWKGKNGLYSVGFTKKGLLGASIDAQRVAEDIAQQWKSETKHFHLRV

>Hyque.05G088700|Hquercifolia_v1.1/1-399

MFFKETKSELRRVWIPGPVIIGAGPSGLAAAACLKHRGVPSLILEKESCLASLWKLKTYDHLSLHLPKNFSELPYMALPRDFPTYPTKQQFISYLEAYAKNFSIQPIFGEEVQCAKYDAAMGFWWVRTNDSEFVCPWLIVATGENSEPLLPEISGIAEFGGKIIHTSKYKNGADFEGRKVLVVGCGNSGMEVSLDLCNSGAEVSLVVRDKLHILPREVLGISTFALSMLLLKWFPVRLVDWFILLCSWAILGDTQRIGIKRPEIGPLELKKSIGKTPVLDVGAMAKIKSGKIKVVRGIQRFTGKAVEFVDGKVDVFESVILATGYRSSVASWLTEGNFFSQEDGYSKNLFPNNWKGKNGIYSVGFTKQGLFGASIDAQSVAEDIARQWNSEMKHLSFQF

>VIT_204s0023g01480|Vvinifera_v2.1/1-465

MSVSSSVWLPYKFLFTFLYLSFWFCPQSTLQLSVSTLFLFFCTYSFLPHLNELNRSPQNQIPNYSQWDEAMMSKELKQVLIPGPLIIGAGPSGLAVAACLKKRGVPSLILEKENCMASLWNLKTYDRLRLHLPKKFCELPYMKFPSEFPAYPTKQQFISYLEDYAKSFSIEPMFGQEVRWTKYDRSMRLWQVEAKESKFLCRWLIVATGENAEPVVPEIAGISNFGGRLLHTSIYKNGADFKGSKVLVVGCGNSGMEVSLDLCNSGAQVSLVVRDKLHVLPREIFGISTFALSMWLLKWFPVSLVDGLILLCSRMILGDTGQIGIKRPEFGPLHLKNATGKTPVLDVGAVAKIRSSEVKVVCGIRRFTAKGVEFVNGEVQEFDSVILATGYRSNVASWLKEGNFFSQKDGYPKNPFPNNWKGEDGAYSVGFTRRGLYGASIDAQRVAEDIARQWKSQMKHLHLDL

>Rhdel08G0125200.1|Rhododendron_delavayi/1-435

MINLTRKKAKSCSDHNAVFTANGMIIEPMNSVLKRVWVPGAVIIGAGPSGLAAAACLKKMEVPFLIIEKENCLASLWKLKTYDRLKLHLPKEFCKLPHFPFPPEIPSYAEKQQFISYVEAYAEHFSIEPLFGKEVKRAKYDGTMGLWQVQTNDTEFVCRWLIVATGENAEPVLPQIDGLTEFRGNILHTSEYKNGADFKGSKVLVVGCGNSGMEIGLDLCNSGAQVSVVVRDKLHVLPREVLGQSTFALSMRLLKWFPVRLVDRFLLLCSWLILGDICQMGITRPEIGPLELKNTSGKTPVLDVGTIAKIKSGHIKIVDGVKRFTSRGVEFVDGRSDEYESIILATGYRSNVASWLMEGNFFNREDGYPKKPFPDNWKGKNGVYSVGFTKLGLLGVSFDAQRVAKDIASQWNSELKHLRLAQSVACASSQISHEF

>BUSI_005726-RA|Buxus_sinica/1-407

MCDLQFKLIRIFRKMNSESKHVCIPGPVIVGAGPSGLAIAACLKEKGVPFLILEKEHCIASLWKLRTYNRLRLHLPKKFCELPYMVFPPEFEEYPTKQQFISYLEAYSKCYSIEPMFGEEVQRAGYDPTMGFWQVKTNKSEYVCQWLIVATGENAEPVLPEISGISDFRGQLIHTSMYKNGSNFVGKKVLVVGCGNSGMEISLDLCNSGAHASLVVRDKLHILPREILGRSTFGLSVWLLKWFPLQLVDRFLLLSSRLILGDTEQFGIKRPEIGPLELKNTTGKTPALDVGTLSKIKSGQIKVVPGIQRLTTKGAEFLDGRVGQFDSIILATGYRSNIPSWLKGEFSGEQDGIAMKSCPSSWKGKTGLYSVGFSKQGILGSSMDAQRVAEEIAQQWNSKTKQFRFEF

>CKAN_01428800|Ckanehirae_v3/1-398

MKSKQECVMALGPLIIGAGPSGLATAACLKEKGIPSLIIERENCIASLWNLKTSDCMKLHLPKRFCELPLMPFPPEFPTYPTKWQFISYLEAYAQQFSIEPVFGEEVKQAEYDQRIGMWRVLTSKSEYICRWLIVATGENAEAVLPNIAGISEFRGRVLHSSSYENGASFKGEEVLVVGCGNSGMEISLDLSKHCAQPSMVVRNKMHILPKEMLGTSTFALSMWLLKWFPLKLVDALILFYSRLVLGDTAQFGLERPKIGPLQMKNDTGRTPVLDVGTFAKIMSGQIKVVPSIRQFTATGAEFVDGVSREFDSLILATGYRSSVPKWLKEEGFFNKDGIPRMPFPNGWKGKNGLYAVGFTKRGLLGASISAQRVAEDIACHWDCETKHLCLELQTILN

>gene_prefix01183.t1_1|Ceratophyllum_demersum/1-392

MKKKSDRVWFLGPVIIGAGPSGLAASACLKEKGVPSLILERENCIASLWKLKTYDRLKLHLPKHFCELPLMGFPENFPTYPSKDQFLAYLESYAENFSINPMFEQEVKCAKYDSSIKLWRIRTQKSEILCRWMIVATGENADRVLPKIKGSSEFKGRVLHSCEYKNGFDFKGEKVLVVGCGNSGMEVCLDLCNSGVETSISVRDKLHILPREILGRSTFGISTFLSKWLPLRIVDWFLIIFSRVILGDIAKFGLKMPKIGPLELKSTSGRTPVLDVGAVSKIKCGQIKVFPGVGRFTSDGIEFVDGTAEKFDSVIFATGYRSNVPSWLQEKAFFNKLDGYPQKPFPENWKGENGIYSVGFTRRGLLGTSMDAHRVAEDIASRWNSKTKKVHN

>Potri.007G028200|Ptrichocarpa_v4.1/1-413

MAMQTTAIQEHKGNGIFHKAMSSEKPVWIPGPVIIGAGPSGLAVAACLKERGVPFLILEKERCIGSLWTLKTYNRLQLHLPKETCKLPHMPFPPEVPAYPTKQQFISYLEAYAKHFAIEPMFRQEVQSAIYDARMGFWRVQSNESEFLCRWFIVATGENAEPVLPNIEGISDFKGSLIHTSRYKDGADFKGQKVLVVGCGNSGMEISLDLCNNDAQVSLAVRDKLHILPREVLGRSTFSLSMWLLNWFPVKLVDRFLLICSQLILGDTHKMGIRRPKMGPLEQKNSTGKTPVLDVGAFSKIKSGKIKVVCGVQRFTASGAEFVDGHVENFDSVILATGYRSNVTSWLKEDSFFNEKDGYPRNPFPDNWKGKNGLYSVGFTRRGLLGSSIDAQRVAEDIARQWNCETKHLRIES

>orange1.1g039923m.g|Csinensis_v1.1/1-395

MSSKAKRFWTPGPVIVGAGPSGLAAAACLKERGVPSLIIEKESCLASLWKLKIYDHLQLHLPKQFCQLPYVPFPREYPAYPSGQQFITYMEAYANHFEIEPLLGQEVQWAKYDAAMGHWRVKTHEYEFMCRWLIVATGENEVPVLPKIPGISEFRGRLLHTSTYKNGVEFRASKVLVVGCGNSGMEISFDLCKNGAQVSLVVRDKVHILPKKILGRSSFAISVWLLKWFPVDVVDRFLLFCSRLVLGDTKQIGIQRPKMGPLQWKNSVGKTPVLDDGAFAKIKSGEIKVVPGIQKFTAKGAEFVNRTVKEFDSIILATGYRSNVSSWLKEASLFNQKNNNNPQDSYPKNWKGKNGVYSVGFARQGLLGISMDAHKVADDIASQWNSETRHLWLDS

>Aqcoe6G114000|Acoerulea_v3.1/1-393

MDRELKRVWVPGPVIVGAGPAGLATAACLKKRGVPCLVIERETCIASLWKMKTYDRLRLHLPKKFCELPYMKFAPETPAYPRKQEYILYLDAYAKHFSIEPILGVEVRLAKYDSTMGFWHVQSNEFEFVCQWLIVAAGENAESVIPDIPGISYFGGRVFHSSLYGNGADYKGEDVLVVGCGNSGMEISLDLCNSGAQASIVVRNKLHVLPREMFGISTFGLSMWLLKRLPLRLVDRFLVLCSWLILGDTGWLGFKRPEIGPFELKDSTGKTPVLDVGTIAKVKAGQIKVVPGIQKFTTTGAEFSDGTKSKFDSIIFATGYRSNVASWLKDGELFDQEGHPKTPFPDSWKGKHGLYSVGFTGRGLLGISMDAQKVAEDILQQWSSETKHLRMEL

>Kalax.0052s0005|Klaxiflora_v1.1/1-439

MLIPATMESVKQVDSKRVLVHDHVVDLDSSMASTPPPASRCVYVPGPVIVGAGPSGLAAAACLKQRGIPSLILERASCIASLWKLKTYDRLRLHLPKQFCQLPLMPFPENFPTYPTKQQFIRYLESYATRFDIKPKFDVAVISAEHDARFGFWRVATVALAAEVIVEYECQWLIVAAGENAETVVPEIEGLGEFVGPVVHTSCYKSGEEFKEKRVLVVGCGNSGMEVCLDLCNFNAKPSLVVRDTVHVLPQEMLRQSTFGLSMFLLKWFHVRLVDRFLLMVSRLMIGDTSRFGLNRPKVGPLELKSISGKTPVLDVGTFAKIKSGHVKVRPGVKRITCDKAVFVDGRSEKFDAIVLATGYRSNVPSWLKEQNMFSEKDGMPREAFPGGWRGQHGLYAVGFTKRGLLGTSMDARRIAEDIEHEWKSDAKKYAKFLGHILT

>Kalax.0162s0077|Klaxiflora_v1.1/1-440

MLIPATMESVKQVDSKRVVHDHVVDLDSSMAATAPTASRCVYVPGPVIVGAGPSGLAAAACLKQRGIPSLILERASCIASLWKLKTYDRLRLHLPKQFCQLPLMPFPENFPTYPTKQQFIRYLESYATRFDIKPKFDVAVISAEHDARFGFWRVATVALAAEVIVEYECQWLIVAAGENAETVVPEIEGLGEFVGPVVHTSCYKSGEEFKEKRVLVVGCGNSGMEVCLDLCNFNAKPSLVVRDTVHVLPQEMLRQSTFGLSMFLLKWFHVRLVDRFLLMVSHLMIGDTSRFGLNRPKVGPLELKSISGKTPVLDVGTFAKIKSGHVKVRPGVKRIARDTAVFVDGRSEKFDAIVLATGYRSNVPSWLKEQSMFSEKDGMPREAFPGGWRGQNGLYAVGFTKRGLLGTSMDARRIAEDIEREWKSDAKKYAKFLGHGPLKF

>Acora.01G123400|Aamericanus_v1.1/1-436

MDCLREAECKRIHDCHSILINGVSKEDDEEEGTKNALKTVTMMTRQVWVPGPVIVGAGPSGLAAAACLKVKGVPSVILERSHCIASLWQLRTYDRLSLHLPKNFCELPMMPFPHHFPTYPTKEQFIDYIHAYARQFNLRPVFNETVESAEYDCTIGFWRVRTTTMTEYLCRWLVVATGENAEAVVPGIPGMEEFNKGTVVHTSAYGSGDAFRDKRVLVVGCGNSGMEVSLDLCNHGAHTTIVVRDAVHVLPREMLGRSTFGLSMWLLKWLPMRVVDRFLLFVSRLMLGDTSLIGLHRPSIGPLELKTLSSKTPVLDIGTLSKIKSGDIKVRPAIKRMMMNGVEFVDGRVEEFDAVILATGYRSNVPSWLKESELFSEKDGLPRKPFPDGWKGERGLYAVGFSKAGLLGSSKDALRIAQDIDRSWKSISLLDMISSS

>Acora.07G067600|Aamericanus_v1.1/1-426

MFMMDCFREAEYKAMHDCLPILLNRGGEEGIALKTKDMMKSRQVWVPGPVIVGAGPSGLAAAACLKVKGVPSMVIERSSCIASLWQLRTYDRLRLHLPKKFCELPLMPFPDSFPTYPTKQQFITYLEAYAKRFDLNPVFNETVEKASYALGFWRVHTRSAEGTMTEYVCRWLVVATGENAEAVVPEIEGMGEFKGPVEHTNWYRSGDAFREKKVLVVGCGNSGMEIALDLCNHHAHPTLVVRDAVHVLPREILGRSTFGLSMCLLKWFPVRVVDTFLLFMSWLMLGDTSFVGLNRPSIGPLELKAVSGKTPVLDIGTLSKIKSGHIKVRPAIKKMTMNGAEFIDGRVDDFDAVVLATGYRSNVPSWLKESDFFSDVDGLPRKPFPNGWKGEKGLYAVGFTKRGLMGSSMDALRIAQDIEHCWKAHA

>evm_27.TU.AmTr_v1.0_scaffold00218.8|Atrichopoda_v1.0/1-428

MGAEWCYLREQETKQLHDSYSSFSSSYFMDKSSSPSLPLRSRRRLLWVQGPVIVGAGPSGLATAACLKERRVPSLILERANCIASLWQLKTYDRLSLHLPKKFCELPLMPFPSHFPTYPTKQQFIQYLQDYAQHFGLEPAFNERVECADYDPSVGLWRVCTSSWTEYVCRWLVVATGENAEAVLPQIRGMDEFQGPIIHTSCYRSGAGLDGQRVLVVGCGNSGMEVCLDLCNHNASPTLVVRNPVHVLPREMLGKSTFGVSMWLLKWFPVRMVDRFLLLVSWLMLGSTARLGLARPRVGPLELKSKAGKTPVLDVGTLAKIRSGQIKVRPGVKRLRAQGAEFEDGKGEEFDAVILATGYKSNVPSWLKETDFFSVEDGLPRTPFPNGWKGKSGLYAVGFTKRGILGASMDAVRIAQDIERDGRTSQGI

>evm.TU.AsparagusV1_01.690|Aofficinalis_V1.1/1-409

MGYCREIQRKQLHDHHHQQHPIFIPYPVIIGAGPSGLATAACLKEKGIDSLVLERSNCIASLWTHSTYDRLHLHLPKKYCQLPLMPFPPKFPKYPSKQQFISYLESYANKFDIRPLFKETVVRAEFDRGVGLWRVRTRVREYACRWLVVSTGENAEAVVGPEINIEGIGRFKGSVMHTSLYKSGELFRGKRVLVVGCGNSGMEVCLDLCNFNACPTIVVRDRVHILPREMLGISTFGLSTWLLKWLPIRIVDRILLLLARFLLGDTALYGLQRPVLGPLEIKSVSGKTPVLDTGTLVKIKSGDIKVRPAIKRLTEDGAEFVDGRSESFDAVVLATGYKSNVSSWLKEREFFSEKDGLPRDPFPNGWKGENGLYAVGLTKRGLMGTSMDARRIANDIQQCWKNDAKRCML

>Asa2G02541.1|Allium_sativum/1-410

MDCLRELEGKQVHDPSFSSSLYKWSPGPIVVGAGPSGLAVSACLKQKGIVSLVLERSDCIASLWQYNTYDRLRLHLPRQYCELPLMSFPSDFPKYPSKLQFIAYLDSYARKFDIRPKFNETVLRAEYDSSINMWRVKTNRSGYVSKWLIISTGENAEIVMPNIKGMDEYKGIVMHTSSYKSGDSFHRKNVLVVGCGNSGMEVCFDLCSFNAYPTLVVRDAVHVLPIEVMGYSTFSLSMLLLKWLPIRIVDWILLFIARILLGDTSCLSLHRPLLGPLELKSKTGKTPVLDIGTLAKIKSGEIKVRPGIKRLTKNGAEFVDGETENFDAIILATGYKSNVSSWLKENKLISNKDGLPKYPFPDGWQGENGLYAVGLTRRGLMGASIDAKKIADDIKQCWDEMNTKILQKIG

>LOC_Os01g53200|Osativa_v7.0/1-442

MDPWSEIEGKRAHDPIFQNYFSQNCRQSVDGFCKKRSADAAVARAERCIRVLGPIIVGAGPSGLAVAACLKEKGVDSLVLERSNCIASLWQLKTYDRLSLHLPRQFCELPLMPFPAYYPIYPSKQQFVAYLESYAARFGICPTYNRTVVCAEYDEQLQLWRVRTRATGIMGEEVEYVSRWLVVATGENAEVVLPEIDGLDDFKGTVMHTSSYKSGGAFAGKRVLVVGSGNSGMEVCLDLCNHNANPHIVVRDAVHILPREMLGQSTFGLSMWLLKWLPVHVVDRILLLIAQTMLGDTAQLGLKRPTIGPLELKSLSGKTPVLDVGTFAKIKSGDIKVRPAIKQISGRQVEFMDTRLEEFDVIVLATGYKSNVPFWLKDRELFSEKDGLPRKAFPNGWKGENGLYSVGFTRRGLMGTSVDARRIAHDIEQQWKARGKHPGVLL

>HORVU.MOREX.r3.3HG0286870|Hvulgare_Morex_V3/1-437

MDHWSESEGKRVHDPIFQPRISHNCRQPVEEFCRERSTDAAVTRSELCTWVPGPIIVGAGPSGLAVAACLKEKGVDSLILERSNCIASLWQLKTYDRLSLHLPRQFCELPLMPFPADYPIYPSKEKFVAYLEKYAARFGICPRYNHAVVRAEYDEKLLLWRVRSQLTGRMEEEVEYLSRWLVVATGENAEVVQPEIDGLKEFKGTVVHTSAYKTGSAFAGKRVLVVGCGNSGMEVCLDLCDHNAQPHIVVRDTVHILPREMLGQSTFGLSMWLLKWFPVHVVDRILLLVARTMLGDTARLGLKRPTVGPLELKSLSGKTPVLDVGTFAKIRSGDIKVLPGIKKISGRQVEFLDTRSEDFDAIVLATGYKSNVPFWLKDRELFSDKDGLPRKAFPNGWKGERGLYSVGLTRRGLMGTSVDARRIAHDIEQQLSAEGKL

>Bradi2g48980|Bdistachyon_v3.2/1-438

MDHWSESEGKRSHDPIFQTKLNCRQHVEDFCKKRADAVATSTGRCIWVPGPIVVGAGPSGLAVAACLKEKGVDSLILERSNCIASLWQLKTYDRLSLHLPRQFCELPLMPFPTDYPIYPSKEEFVLYLEEYAARFGICPTYNHEVVCAEYDEKLLIWRVRTQATDRMGEVVYMSRWLVAATGENAEVVQPEIDGLKEFKGTVLHTSEYKSGLAFAGKRVLVVGCGNSGMEVCLDLCNSGAQPHIVVRDTVHILPREMLGKSTFGLSMWLLNWLPVHMVDLILLFVAWIKFGDTAQLGLKRPTIGPLELKSLSGKTPVLDVGTFAKIRSGNIKVRPDIKQISGRQVEFLDRQTEDFDAIVLATGYKSNVPFWLKDRELFSEKDGLPRKAFPNGWKGGRGLYSVGFTRRGLMGTSADARRIAHDIEQQLSAEGKHPDVLL

>Zm00001d011764|Zmays_RefGen_V4/1-454

MDPWSESEGKRAHDPIFKCFSQNQGHHHFMERSTDAAVALSERCRCIWVSGPIIVGAGPSGLAAAACLKEKGISSLILERSRCLASLWQLKTYDRLSLHLPRQFCELPLMPFPADYPIYPSKQQFVAYLESYAASFGICPTYNRTVVCAEYDEQLLLWRVRTQTSDTTGEEVEYVSRWLIVATGENAEAVQPDIGGLQEFPGTIMHTSAYKSGSAFTGKRVLVVGCGNSGMEVCLDLCNHSAEPHIVVRDAVHILPREMLGHSTFGLSMWLLKWLPVHVVDRVLLCIARAMLGDTARLGLKRPASGPLQLKSLSGKTPVLDVGTFAKIKSGDIKVRPAVRQVSGRAVEFADGELEEFDAIVLATGYKSNVPFWLKDRVLFSEKDGLPRKAFPNGWKGEKGLYSVGFTRRGLMGTSVDARRVAHDVEQQWRKAKGTHRDGVPLPLVAVVYHGDGL

>Sobic.003G286500|Sbicolor_v5.1/1-436

MDPWSESEGKRAHDPIFKCFSQNHHFKEMSTDAAVALSERCPCIWVSGPIIVGAGPSGLAVAACLKEKGISSLILERSSCIASLWQLKTYDRLSLHLPRKFCELPLLPFPANYPIYPSKQQFVAYLESYAARFGISPTYNRTVVCAEYDEQLLLWRVRTQTSGTTGQEVEYLSRWLIVATGENAEAVQPDIGGLQEFPGTIMHTSAYKSGSAFTGKRVLVVGCGNSGMEVCLDLCNHNAEPHIVVRDAVHILPREMLGHSTFGLSMWLLKWLPVHVVDRVLLCIAWAMLGDTAQLGLKRPAFGPLELKSLSGKTPVLDVGTFAKIKSGDIKVRPAVRQISGRVVEFADGGLAEFDAIVLATGYKSNVPFWLKDRELFSEKDGLPRKAFPNGWKGENGLYSVGFTRRGLMGTSVEARSIAHDIEQQWKAKGTHPDAG

>ELECO.r07.1AG0032120|Ecoracana_v1.1/1-445

MDPWSESEGKRAHDPIFQSFSRNQNHCQPVENCCKERSMDAVVARSERCTCIWVSGPIIVGAGPSGLAVAACLKEKGIGSLILERSNYIASLWQLKTYDRLSLHLPRQFCELPLMPFPSDYPIYPSKQQFIAYLESYAARFGIRPTYNRTVVHAEYDEQLLLWLVKTRASGKMGDEVEYVSRWLIAATGENAEAVQPAIDGLQEFEGTVLHTSAYKSGSAFAGKRVLVVGCGNSGMEVCLDLCNYNAKPHIVVRDTVHVLPREMLGLSTFGLSMLLLKWLPVHVVDRILLLIARAMLGDTARLGLKRPTFGPLELKSLSGKTPVLDIGTFAKIKSGDIKIRPAIRHISGREVKFLEGQSEEFDAIVLATGYRSNVPCWLKDRELFSDKDGLPRKAFPNGWKGEKGLYSVGFTRRGLMGTSVDARRIAHDIEQQLKSQGLHPDVFL

>ELECO.r07.1BG0082330|Ecoracana_v1.1/1-445

MDPWSESEGKRAHDPIFQSFNRNQNHCQPVENCCKERSMDAVVALSERCTCIWVSGPIIVGAGPSGLAVAACLKEKGIGSLILERSNCIASLWQLKTYDRLSLHLPRQFCELPLMPFPADYPIYPSKQQFIAYLESYAARFGICPTYNRTVVHAEYDEQLLLWRVKTRASGKMGEEVEYVSRWLIAATGENAEAVQPAIDGLQEFEGTVLHTSAYKSGSTFAGKRVLVVGCGNSGMEVCLDLCNHNAKPHIVVRDTVHVLPREMLGLSTFGLSMLLLKWLPVHVVDRILLLIARAMLGDTARLGLKRPTFGPLELKSLSGKTPVLDVGTFAKIKSGDIKIRPAIRHISGREVKFLEGQSEEFDAIVLATGYRSNVPCWLKDRELFSDQDGLPRKAFPNGWKGEKGLYSVGFTRRGLMGTSVDARRIAHDIEQQLKSQGLHPDVFL

>Eucgr.C02888|Egrandis_v2.0/1-430

MDFSREIEGKQLHDPLYANWRMSRRFGDCVQVSGPIIVGAGPSGLAVAACLNQRGIRSIILEKSDCIASLWQRKTYGRLRLHLPKQFCELPMMGFPPASPTYPDKREFVKYLEDYAKRFGIRPQFNETVEAAEYDGEIGQWRVRSMGPKEGEKTEYVCQWLVVATGENAEPVVPEIEGMEGFGGPIKHTSMYSSGEEFRGKRVLVVGCGNSGMEVCLDLCNHNAMPSLVVRDTVHVLPRDVLGKSTFGLSMWLLKWLPMRLVDQLLLLAARVMLGDTARLGLKRPKVGPLELKNLNGKTPVLDVGTLAKIKSGDIKVYPGIRRLNPHEVKFLDCRSEKFDAIVLATGYKSNVPTWLKEGSMFSKDDGFPRRAFPNGWKGERGLYAVGFTRRGLLGVSMDARRIAEDIERCWKEEEKHRIQVPNFLFPKSS

>Cucsa.001610|Csativus_v1.0/1-406

MDDYWKELQGKRLHDPLTKNQVSPIPQILVQGPLIVGAGPSGLAVAACLKQKGIQSLILERAECIASLWQFKTYDRLRLHLPKPFCQLPFMPFPSHFPTYPTKQQFLSYLKAYADYFKLKTVFNSTVIRAEWDERCGFWKVKSVKDQKVIVEYFCKWLIVATGENAEEIIPQIEGLEIFEGPVVHTSSYKTGEVFHGKKVLVIGCGNSGMEVCLDLCNFNASPHLVVHILPHEILGRSTFGLSMCLLRWLPMRIVDQFLLLVSRLMLGDTSKLGLHRPKLGPLQLKNLSGKTPVLDVGTFAKIKTGKIKVRRGVTRLTRHAAEFVDGSLENFDAVILATGYKSNVPSWLKERHMFDEKDGMPRKEFPNGWKGECGLYAVGFTKRGLLGASMDAKRIAEDIELCLEN

>BUSI_014110-RA|Buxus_sinica/1-427

MDHLKELEGKRLHHGLHEKMRKSSRCVWVPGPVIVGAGPSGLAVAACLKQRGVPSLILERYNCIASLWQLKSYDRLCLHLPKQFCQLPLMPFPSSFPTYPNKQQFVEYLESYAKRFNLQPAFNETVKSAEYDKTLGFWRVKTMGLKGETEYVCRWLIAATGENAEAVIPEIEGMSDFVGPIIHTSSYKSGEEFRGKRVLVVGCGNSGMEVCLDLLNYNASPSIVVHVLPREMLGKSTFVLSMLLLKWLPISLVDRFLLLLSRLKLGDTASFGLHRPQLGPIQLKNMSGKTPVLDVGTFAKIKTGDIKVCPGVKRLKRCNAEFVDGRVENFDAVILATGYRSNVPFWLKDKYLMSEEEGSVASKAFQNGWKAERGLYAVGFTKRGLFGASMDARRIAEDIEHCWKAEANNLERSLVHHCHNHKLSKIV

>AT4G13260|Athaliana_Araport11/1-415

MEFVTETLGKRIHDPYVEETRCLMIPGPIIVGSGPSGLATAACLKSRDIPSLILERSTCIASLWQHKTYDRLRLHLPKDFCELPLMPFPSSYPTYPTKQQFVQYLESYAEHFDLKPVFNQTVEEAKFDRRCGLWRVRTTGGKKDETMEYVSRWLVVATGENAEEVMPEIDGIPDFGGPILHTSSYKSGEIFSEKKILVVGCGNSGMEVCLDLCNFNALPSLVVRDSVHVLPQEMLGISTFGISTSLLKWFPVHVVDRFLLRMSRLVLGDTDRLGLVRPKLGPLERKIKCGKTPVLDVGTLAKIRSGHIKVYPELKRVMHYSAEFVDGRVDNFDAIILATGYKSNVPMWLKGVNMFSEKDGFPHKPFPNGWKGESGLYAVGFTKLGLLGAAIDAKKIAEDIEVQRHFLPLARPQHC

>DCAR_830616|Dcarota_subsp_sativus_v3.0/1-441

MDYNYLTELDGKRLHDPRLTRPKTMTTTSRIRVEGPVIVGAGPSGLAVAACLRQRGIPSLILERANCLASLWQLKTYDRLRLHLPKHLCQLPHMPFPQDFPTYPTKQQFVAYLMAYAKAFHIEPEYNTTVVSAEFDNNCDFWRMKTVKGELELESGEIEYVCKWLVVASGENAEEVKPEFEGMDEFRGPIIHTSHYKSGDPFRDKRVLVVGCGNSGMEICLDLCNYNARPSLVVRDTLHVLPQEMLGRSTFGVSMWLLKWLPIRFVDCFLLLVSRLTIGDTDQLGLYRPKLGPLELKKKTGKTPVLDVGTLAKIRNGSIKVFPGVKRFTCHAIEFVDGRTENFDAIILATGYKNNIPSWLKDNVLFSKKDGHSKKPFRPEGWKGENGLYSVGFAKRGLLGTSMDAERVAEDIELQWYLKDSKIITESVDQVPEIERVLCNL

>Kalax.0593s0023|Klaxiflora_v1.1/1-423

MNLQSESGSGSARLVSFVDGAVVVGAGPSGLAASACLKQKGVPSLVLERSDCVASLWQRRAYDRLRLHLPKSFCQLPLAPFPPHFPKYPSKRQFVTYLESYARKFDIKPRFNEAVTAAEYDKALGLWRVKTSDSNLGLGLGLGCGEREYLCRWVVVATGENAEVVVPEMEGAEEFGGAVVHTCGYKSGEMFRGKRVLVVGSGNSGMEVCWDLCSFGAKPSIVVRDTVHVLPQEMLGRSTFGLSMWLQKWLPIRLVDRFLLLLSWFVIGDTSNLGLTRPKLGPLELKNLTGKTPVLDVGTIDKIRSGHVQVRPGIRRLKRLAVEFVNGRVENYDAIILATGYKSNVPSWLKSGNMFCEKDGLPKSPFPNGWKGEHGLYAVGFTKRGLLGASMDARRVAEDIKQCRSSHDSSSKQQVHPVLEGGR

>Kalax.2037s0001|Klaxiflora_v1.1/1-190

MLGRSTFGLSMWLQKWLPIRLVDRFLLLLSWFVIGDTSNLGLTRPKLGPLELKNLTGKTPVLDVGTIDKIRSGHVQVRPGIRRLKRLAVEFVNGRVENYDAIILATGYRSNVPSWLKSGNMFCEKDGLPKSPFPNGWKGEHGLYAVGFTKRGLLGASMDARRVAEDIKQCRSRHDSSSKQQVHPVLEGGR

>Kalax.0015s0078|Klaxiflora_v1.1/1-443

MNHQHTEPTTSSSSSSSNHRNGGDDSNNVVVDGAVIVGAGPSGLAASACLKQKGIPSIILERCDCIASLWQHKTYDRLRLHLPKSFCQLPLHPFPSHYPKYPSKRQFISYLETYARKFGIKPHFNETVTAAEYDETVGLWRVKTTSCNDDVSEVGLGLGFSRCCKNKEYVCRWVVVATGENAEVVVPEMEGVEEFAGAVVHTCYYKSGEMFKGKRVLVVGSGNSGMEVCLDLCSFGARPSIVVRDTVHVLPQEMLGRSTFGLSMWLLKWLPIRLVDRFLLLLSWLIIGDISKLGLTRPKMGPLQLKNLTGKTPVLDVGTIAKIRSGHVQVRPGIRRLKRLAVEFVDGRVENYDAIILATGYKSNVPSWLRGGNMFCEKDGLPKSPFTNGWKGGNGLYAVGFTKHGLLGASIDARKIAEDIKQQWTSHDSSSKKQVHPILEQVF

>Brara.B03625|BrapaFPsc_v1.3/1-445

MDFCWKREMEGKLAHDYLSSTKRHQHGMMTSPHRVCVVTGPVIVGAGPSGLATAACLKERNITSVILERSNCIASLWQLKTYDRLHLHLPKQFCELPLVPFPDHFPTYPTKQQFIEYLEDYARRFDIRPEFGQTVESAEFDANLGMWRVVSVGEEGTTEYVCRWLVAATGENAEPVVPRFEGMEKFKATGVVKHTSQYKTGGDFVGKKVLVVGCGNSGMEVCLDLCNFGAQPSLVVRDAVHVLPREMLGTSTFGLSMLLLKWLPIQLVDSFLLVVSRFILGDTTLLGLNRPLIGPLKLKNLTGKTPVLDVGTLAKIKTGDIKVCSGIRRLKHHEVEFDNGRTERFDAIILATGYKSNVPSWLKENKMFSKKDGFPMQEFPEGWRGECGLYAVGFTKRGIFGASMDAKRIAQDIYESSRKSGQPHRHIQVFMARKSDQACSRVLDG

>Brara.F02755|BrapaFPsc_v1.3/1-432

MDWKKEMEGNLANDYLSSNHGMMTSQRRICVFTGPVIVGAGPSGLATAACLKKKGITSVLLERSNCIASLWQLKTYDRLSLHLPKQFCELPLMPFPADFPTYPTKQQFIEYLEDYARRFDIRPEFGQTVESAEFDEKLGMWRVRSVGKEGMTEYVCRWLVAATGENAEPVVPRFEGMEKFAATGVVKHTSHYKTGGDFAGKRVLVIGCGNSGMEVCLDLCNFGAQPSLVVRDAVHVLPREMLGTSTFGLSMLLLRWLPIRLVDRFLLVVSRFILGDTTLLGLNRPRLGPLELKNLTGKTPVLDVGTLAKIKTGDIKVCSGIRRLKRHEVEFDNGKKERFDAIILATGYKSNVPSWLKENKMFSKKDGFPIQEFPEGWRGECGLYAVGFTKRGISGASMDAKRIAQDIYECSRKSDQAHRHIQVFMSSKPDQA

>Brara.I00535|BrapaFPsc_v1.3/1-441

MDSCWRKEMEGKLAHDYLSHHGKMTSPHHRISVVTGPVIVGAGPSGLATAACLKEKGITSVLLERSNCIASLWQLKTYDRLHLHLPKQFCELPLIPFPDHFPTYPTKQQFIEYLEDYAKRFDIRPEFGQTVDSAEFDENLGMWRVKSVGEEGTTEYVCRWLVAATGENAEPVVPRFEGMDKFEATGVVKHTSHYKSGRDFAGKRVLVVGCGNSGMEVCLDLCNFDAQPSLVVRDAVHVLPREMLGTSTFGLSMLLLKWLPIRLVDRFLLAISRFILGDTTLLGLNRPRLGPLELKNRTGKTPVLDVGTLAKIKTGDIKVCSGIRRLKQHEVEFDNGITERFDAIILATGYKSNVPSWLKENKMFSKKDGFPIQEFPEGWRGESGLYAVGFTKRGIFGASMDANKIAQDIFEWSRKSYQAHRHIQVLCMSRRSGQPYSRLLE

>AT5G25620|Athaliana_Araport11/1-434

MDFCWKREMEGKLAHDHRGMTSPRRICVVTGPVIVGAGPSGLATAACLKERGITSVLLERSNCIASLWQLKTYDRLHLHLPKQFCELPIIPFPGDFPTYPTKQQFIEYLEDYARRFDIKPEFNQTVESAAFDENLGMWRVTSVGEEGTTEYVCRWLVAATGENAEPVVPRFEGMDKFAAAGVVKHTCHYKTGGDFAGKRVLVVGCGNSGMEVCLDLCNFGAQPSLVVRDAVHVLPREMLGTSTFGLSMFLLKWLPIRLVDRFLLVVSRFILGDTTLLGLNRPRLGPLELKNISGKTPVLDVGTLAKIKTGDIKVCSGIRRLKRHEVEFDNGKTERFDAIILATGYKSNVPSWLKENKMFSKKDGFPIQEFPEGWRGECGLYAVGFTKRGISGASMDAKRIAEDIHKCWKQDEQLQCKLGKRMKRKFSESDCGGN

>Zosma01g06240|Zmarina_v3.1/1-480

MIPATIRAKSMYLFAESCWSSHPKNPKHAISISNAMQSAVIDILMEWREEAECKMVHDPLYCIYFQLQAAQIGSETRCVYIDGPIIVGAGPSGLAVAACLKEKGIPSLIVERSDCIASLWQHKTYDRLRLHLPKNFCELPFMSFPSSFPDYPTKQQFLTYLTSYADQFDIKPVFNSTVEKAEYDKGAGLWRVKTVGVGKESGKTKEFLCRSLVVATGENAEAVIPKMNGMDEFKGAMVHTSEYRSGDVFTGKKVLVVGCGNSGMEVCLDLYNFDAKPTIVVREAVHVLPRDMLGKSTFGLSMWLLKYLPVRLVDNFLLLASHLILGDTTSVGLDRPQLGPLELKSLSGKTPVLDVGTLAKIKSGHIKVSPAVKRFTSTGAEFSDGRSENFDAVVFATGYRSNVPYWLKERDFFSEKDGLPRNPFPNGWKGENGLYAVGFTKRGILGASMDAKRIAHDMVSNWNSTRQQALLFQTEKDKRT

>TESI_027891-RA|Tetracentron_sinense/1-417

MDCSKEVEGKRIHDRFKENTKSVRCVWFPVIIVGAGPSGLSVAACLKERGVTSLILEKSNCIASLWQLKTYDRLRLHLPKQFCELPLMPFPANFPTYPTKKQFVDYLESYAKHFNLQPEFNQTVESAEYDHGFWRVRTVGLKNEEMENGCVLGPIVHTSSYKSGDAFRGMKVLVVGCGNSGMEVCLDLCNHNARPSIVVRDKVHVLPRDMLGRSTFGLSMLLLKWLPVRLVDRILLLVSHFMLGDTASLGLNRPPIGPLELKSMSGKTPILDVGTLAKIKSGDIKVRPGIKRLTYCTAEFVDGRVEKFDAVIFATGYKSNVPSWLKDRDLFCEEDGLPQRPFPNGWKGKCGLYAVGFTKRGLLGVSMDAKRIAQDIERCCKAEAKLEEEPYRLCSSLWPGCLMARIYCTVVKSIQPT

>Nycol.I00883|Ncolorata_v1.2b/1-408

MSGSEMKKAEAEPRRVYVPGPVIVGSGPSGLATAACLKGQGVPYLVLERENCIASLWNLKTYDRLRLHLPKFFCQLPHLPFPSDFPLYPTKEQFIHYLETYAQQFGIRPRFGECVTSAEFDAGTGFWKLKTAAGGEFICRWLVVATGENADPAVPSIPGIEEFQGRIVHTSQYKSGWEFQGQKVLVVGCGNSGMEVCLDLCDSGARATMVVRNTVHVLPRDIMGRSTFGLSAWLLRWIPLRVADRVLLFLSRFILGDTARLGMKRPEMGPLELKFTSGKTPVLDVGTLAKIKSGHIKVVPGLESLTPKGAKFVDGREEEYDSIILATGYRSNVPMWLMEGKFFSKKDGYPTTPFPNGWKGENGLYAAGFTKRGLVGASTDAVRVAQDIAQQWNQEAKYFTFPPSKKNI

>g12398.t1_1|Euryale_ferox/1-405

MGASVMTKAETRCVYVPGPVIVGCGPSGLATAACLKGQGVPYLVLERENCIASLWNLKTYDRLRLHLPKFFCQLPHLPFPSDFPLYPTKEQFVHYLETYARQFGIRPRFGECVTSAEFDAGTGFWKLKTAAGGEFICRWLVVATGENADPVVPSIPGMEKFQGRIVHTSQYKSGLEFQGQKVLVVGCGNSGMEVCLDLFDSGARATMVVRNTVHVLPRDVMGRSTFGLSSWLLRWIPLRVVDRVLLFLSRFILGDTTSLGMKRPEMGPLELKFTSGKTPVLDVGTLAKIKSGHIKVVPGLESLTSKGAKFVDGREEEYNSIILATGYTSNVPMWLMEGKFSKKDGSPTTPFPNGWKDENGFYAAGFTKQGLVGASMDAMRIAQDIAQQWNQEAKVFTFPSSKKNI

>g11075.t1_1|Euryale_ferox/1-401

MTKAEPRCVYVPGPVIVGCGPSGLATAACLKGQGVPYLVLERENCIASLWNLKSYDRLRLHLPKFFCQLPHLPFPSDFPLYPTKEQFVHYLESYAQHFGIRPRFGECVTSAEFDAGTGFWKLKTAAGGEFICRWLVVATGENADPVVPSIPGMEEFQGWIVHTSQYKSGLEFQGQKVLVVGCGNSGMEVCLDLCDSGACATMVVRNTVHVLPRDIMGRSTFGLSSWLLRWIPLRVVDKVLLFLSRFILGDTARLGMKRPEMGPLELKFTSGKTPVLDVGTLAKIKSGHIKVVPGLESLTSKGAKFVDGREEEYNSIILATGYRSNVPMWLMEGEFFSKKDGYPTTPFPNGWKGENGLYAAGFTKRGLVGASMDAMRIAQDIAQQWNQEAKIFTFPSRKKNI

>Pn26.302|Piper_nigrum/1-249

MDCLREVEGKCAHDPWSAGYGLPAAVTWVPGAVIVGSGPSGLAVAACLKDRGVPSQIIERSCCIASLWQLKTYDRLCLHLPKHFCELPLMAFPDHFPTYPSKQQFISYLEEYAQRFNLCPAFNEAVDRAEYDQKLGLWRVRARWVKKEVVERREYLCRWLVVATGENAEAVVPDIEGMGEFAGDIVHTSSYRSGEGFRGKRVLVVGCGNSGMEVSLDLCNHNALPSLVVRDSVHILPREMLGTSTFGLS

>DCAR_519722|Dcarota_subsp_sativus_v3.0/1-391

MVYPNLMKSKSNCIQIPGPLIIGAGPSGLAAAACLRRKGVPFFIVEKESCLASLWKLKTYDRLKLHLPKEFCQLPYLPFPQEFPAYPTKQQFITYLEAYANQFSITPRYGEGVQWAHYDPAKRLWHVRADKAVYLCRWLIIATGENAVPVLPNIAGLQDFGGRILHTSEYKNGDEYRGRKVLVVGCGNSGMEISLDLCNYDAQVSLVVRHELHVLPKEMLGKSTIALSMHLIKWLPLRFVDWLLILSSWLIHGDTSRRGIVRPKTGPLQLKEATGRTPVLDVGSMAKINTGEIKVVRGIKKFIPKGVEFEGGRTEGFDTVILATGYRSNVASWLKEEDFISEKDGNSQNTFPKGWKGENGIYSVGFSRQGLLGISIDAQRVAEDIVGQWKS

>GWHPCBIR010901|Santalum_yasi/1-450

MDPSTEMEGKKLHDPIIFQETKKTTAATPFNSPVRRMWVPGPVIVGAGPSGLAVAACLKHQPGSLRSLILEKENCVASLWQLKTYDRLRLHLPKQFCELPYLPFPGSFPTYPTRDQFVEYLQDYAAKFDLEVAFNRTVRSAEFDPKCGVWVVRASVAGVSNAAGAAAEEETEEYWCRWVVVATGENAAAVVPEIEGMAGFGGPIVHTCEYRSGEEYRGKRVLVVGCGNSGMEISLDLCHFSASPFLVVRDSVHVLPQEMVGRSTFAVAMWMLKWLPVGVVDLVLLAVSHLMLGDTGRLGLHRPKLGPLQLKNASGKTPVLDVGTLAMINSGHIQVRPGIKRLTCRRAEFTDGRVENFDAIVLATGYKSNVQSWLKESEMFSKEDGLPRKPFPNGWKGEHGLYAVGFTNRGLLGASMDARRIAEDIAQCLKIEPTLYFMPLPCTQPSHSIL

>evm.TU.AsparagusV1_08.1148|Aofficinalis_V1.1/1-437

MRSVSVLMTIVNGMALCKWEFKSTSENPCAALEFNLNEPLAGHVEPTPKQFWVPGPIIIGAGPSGLATAACLQDKGVPSLILEKENCIASSWKLRTYERLTLHLPKQFCELPLMSFPANFPTYPTREQFISYLESYVDHFSIKHLLGMEVKCAEYDSSIGFWRIRVNDLQFITRWLIVATGENSEAVVPEFKGIMDYKGQVLHTSYYKKGDDFRRKKVLVVGCGNSGMEVCLDLCNNQAQASIVVRDKMHVLPREILGISTFGLLMWLLKWFPISVVDSILLSLSWLILGNTERYGLRRPKVGPLQLKNKTGKTPVLDVGTLAKIKNGQIQVVPGINRFTENGVEYVDGRQENFDSIILATGYKSNVPSWLKEAEFFSKENGLPRNPFPGNWKGKNGLYATGFTRRGLLGSSTDAKRIAEDIASQWNLKPKHPPSDL

>Asa2G01532.1|Allium_sativum/1-439

MTIVQEKPLLQWEILPENRNLTQVLEFNLHERLATMDSSAKEYRIPGPIIIGAGPSGLATAACLQNKGVQSLILEKENCIASSWKLRTYERLTLHLPKHFCELPFMPFPKHFPTYPTREQFITYLDSYVDRFCINHLCGVEVKCAEFDKSIGYWRIRTNDLQFMSRWLIVATGENSEAFVPDFKGTSEFKGQIIHTSCYRRGDDFRSRKVLVVGCGNSGMEVCLDLCNNQAQASMVVKDKLHILPREIFGTSTFGLSMFLLKWLPITMVDRLLLFFSWLILGNTGTYGLSRPKIGPIQLKHKTGKTPVLDVGTLSKIKNGQIKVVPAVKRLTVKGVEYTDGKQESFDAIILATGYKSTVASWLKEGEFFNKEDGFPKIPFPDSWKGKNGLYATGFSKRGLLGTSIEAKRIAEDIKSQWNTKTEHLPLEMKTINGKFSVK

>LOC_Os12g32750|Osativa_v7.0/1-448

MPTSQQDGGGGGERLFHGGGAGDGDGGGGGGAAAAAAARQVWVPGPVIVGAGPSGLATAACLKARGVPSLVLDKDATVAASWRERTYERLRLHLPRGFCELPLAPPFPPGTPPYPTRDQFVAYLDAYARAFAVEPRLGSRVRAASYDAAIGFWRVAAVDEAGGGGAGGETEFLSRWLVVATGENAVAAWPAEGVGAYRGAVMHTSSYKRGDEFAGKKVLVVGCGNSGMEVSLDLCNNGAATSMVVRDKIHVLPREILGISTFGLSVFLLKWFPIKWVDALLLIFSRLILGNIEKYGLRRPKIGPLQIKCSTGKTPVLDIGALKKIKNGEIKVVPAIHCFTEDGVEFVNGCREDFDAVIFATGYKSNVPSWLKEEEFFSESDGFPRKAFPHSWRGKNGLYATGFTKRGLQGTSYDAAMIAADIARRWTKSLAGPTAAADADHHETYIAN

>Zm00001d035294|Zmays_RefGen_V4/1-444

MPTSREEGQAEPDEHHPTILHRGGGGDAAGGSGGRPRQVWVPGPVIVGAGPSGLATAACLKARGVPSLMLEKDSCVAASWRHRTYERLRLHLPRCFCELPLAPFPPGTPPYPTRDQFIAYLDDYARVFGIQPHLNARVHRAAYDAAIGFWRVTVKEDSGGDGATAANTEFLSRWLVVATGENAEPAWPEGVEGMDTYRGVAMHTSSYKKGDEFRGKNVLVVGCGNSGMEVSLDLCNNGAKASMVVRDKLHVLPREILGVSTFGLSVFLLRWLPIKLVDSLLLFFTRLILGDTEKHGLPRPKIGPLQIKASTGKTPVLDIGALPKIRSGEIKVVPAIQRFTENGVEFVDGHQEDFDAVIFATGYKSNVPSWLKEEELFSHVDGVPRKPFPHSWRGKNGVYAAGFTKKGLMGTSHDALRIAGDIADQWTETFASPAATSRSSDQCA

>Sobic.008G110700|Sbicolor_v5.1/1-448

MPTSREQAQAEHDHHHHHPTILHRGGLGDDGDGGGGSDSRQVWVPGPVIVGAGPSGLATAACLKARGVPSLVLEKDSCVAASWRHRTYERLRLHLPRCFCELPLAPFPPGTPPYPTRDQFIAYLDDYARAFGIEPRLGARVRRAAYDAAIGFWRVTVDEDAGGDGGATVASTEFLSRWLVVATGENAEPAWPEGVEGMDGYRGVAMHTSSYKKGDEFRGKNVLVVGCGNSGMEVSLDLCNNGAKASMVVRDKIHVLPREILGISTFGLSVFLLRWLPIRQVDSLLLFFTRLILGDTEKYGLPRPKIGPLQIKSSTGKTPVLDIGALRKIKTREIKVVPAIQRFTENGVEFIDGHREDFDAVIFATGYKSNVPSWLKEEELFSHFDGFPRKPFPHSWRGKNGLYAAGFTKKGLMGTSYDALRIAGDIADQWTETFASPTATNRSSDHGA

>ELECO.r07.5BG0439230|Ecoracana_v1.1/1-445

MPTSRGKEDDHHPCILHGGTEGAAGDDGMAPRQVWVPGPVIVGAGPSGLATAVCLKARGVPSLVLEKDVCVASSWRHRTYERLRLHLPRCFCELPLLPFPAGTPAYPTRDQFITYLDAYSRAFEIRPRFSSRVQSAAYDASIGFWRVVVDDEAVDGEVAAATTSTEFLSRWLVVATGENAEPALPEGVERMGMDVYRGVTMHTCSYKRGDEFRGKKVLVVGCGNSGMEVSLDLCNNGAFASMVVRDKLHVLPREILGISTFGLSMFLLRWLPTKWVDSFLLFFTRLILGETEKYGLPRPKTGPLQIKSMTGKTPVLDIGALRKIKDGKIKVVPTINRFMENGVEFIDSHREDFNAVIFATGYKSNVPLWLKDEALFSHVDGFPRKPFPHSWRGENGLYAAGFTKKGLMGTSYDALRIAGDIADQWTEALSSPTLLLAGASTVRDA

>ELECO.r07.5AG0392180|Ecoracana_v1.1/1-444

MPTSRGKEDDHHPSILHGGTEDAAGEDGMAPRQVWVPGPVIVGAGPSGLATAACLKARGVPSLVLEKDACVASSWRHRTYERLRLHLPRCFCELPMLPFPAGTPAYPTRDQFIAYLDSYASAFEIQPRYSSRVQSAAYDASIGFWRVVVDKAVDGVVAAVATSTEFLSRWLVVATGENAEPALPEGVERMGIDVYRGVAMHTCNYKRGDEFRGKKVLVVGCGNSGMEVSLDLCNNGAFPSMVVRDKLHVLPREILGMSTFGLSMFLLRWFPTKWVDSFLLFFTRLIIGETEKYGLPRPKTGPIQIKSMTGKTPVLDIGALRKIKDGKIKVVPTINRFMESGVEFIDSHREDFNAVIFATGYKSNVPSWLKDEALFSHVDGFPRKPFPHSWRGENGLYAAGFTRKGLMGTSYDALRIAGDIADQWTEALASPTLLLTGASTVRDA

>Bradi4g06427|Bdistachyon_v3.2/1-468

MLARTTSLTLSSGSERAMLDGHHQQQQPPSSSAAAAALQDEGGVDGPARRVVWVPGPVIVGAGPSGLATAACLKARGVPSLVLERGGSVAPSWRHRTYERMHLHLPLCFCELPMAPFPPGTPPYPSRDQFVAYLDAYARDFALEPLFRAQVRSAAYDAAIGFWRLQVDVDAGAGDGEEDGGDTTTTTTTTEFVSRWLVVATGENAEPVWPEGMMAAGDGVYRAGPVMHTSAYKRGDGFAGKKVLVVGCGNSGMEVSLDLCDNGAKASMVVRDKLHVLPRDILGISTFGLSVCLVKWFPIKWVDALLLFFSRLILGDTEKYGLQRPKIGPLQIKRSTGKTPVLDIGALRRIRDGEIKVVPAINRFTEGGVEFADGRREDFDAVILATGYKSNVPSWLKEDEFFSETDGFPRKEFPHSWRGKNGLYATGFTRRGLMGSSYDASRIAADIANQWTEALAASSVAAAAADTN

>HORVU.MOREX.r3.5HG0448170|Hvulgare_Morex_V3/1-430

MPVSHRGLQLDAQPSAALLDGGHGQDGHVPTRRVWVPGPVIVGAGPSGLATAACLKARGVPSLVLERDACVAGSWRHRTYERMRLHLPVRFCELPLAPFPPGTPPYPTRDQFIAYLDAYARAFAVEPLLGARVRAAAYDAAIGFWRVTVQDAVTRTATEFMSRWLVVATGENAEPVWPHGVEGMDAYRGTMMHTSTYKRGDEFAGKKVLVVGCGNSGMEVSLDLCDNGAKASMVVRDKLHVLPRDILGISTFGLSVFLLKWLPMKWVDALFLFLSRLILGETGKYGLQRPKIGPLQIKKSTGKTPVLDIGALRKIRDGEIKVVPAINRFTESGVEFADGRKEDFDAVILATGYKSNVPSWLKEEDFFSQSDGFPRMAFPHSWRGKNSLYATGFTRRGLMGSSYDASRIAADIANQWTEALARNITAHNNA

>TnS000936361t02|Gnetum_montanum/1-425

MEKTSNAAAEEQERPLKEAQEQQQQQQLECVWVEGPIIVGAGPSGLATAACLKREGVAFEMLERADCVASLWQHRTYDRLRLHLPKYFCELPFMEFPRHFPTYPTKQQFVEYVEAYVRAFKLRPRFNECVCSASFDGALGMWRVRAANAKTGAHTEFLSRWLVVATGENAEPVEPRFAGREAFQGSVRHVCHYKSGAEYRGKRVLVVGCGNSGMEVCLDLINHNASPAIVVRSSVHVLPREIACVPTFRIAMALMKRLPVKLVDRLLLLLARFIVGDTARYGLPRPSTGPMQLKGEAGKTPVLDIGALQHIKRGHIKVLPGVERLTSRGAKFADGRELEFDSVILATGYRSNVPLWLKDCGDLFTDKGVPSRPFPNGWKGKKGLYTVGFTKRGLLGASVDAQHIAQDIAAQFRLHKMQLVLCRLS

>Zosma06g02500|Zmarina_v3.1/1-399

MGSSKAKRVCIPGAVIVGAGPSGIATAACLRERGVPFLILEKESCVVSSWKMKMYDNLRLHLPKQFCQLPMVPFPEDFPVYPTRQQFVEYVDEYVQRFQIEPVFGVEVRLAEYDRSIGFWKVMAGENEIVCQWLVIATGENGEAVIPKFAGMDEFAGKVLHTMEYMSFHQFHGQRVLVVGCGNSAMEICLDLCNNSSVRTSMVVRNELHILPKEMLGRSVFELSMWLLKWLPIKIVDPLILFFSWIYLGDTSKYGLRRPKIGPLQLKNTTGKSPVLDVGALSKIKLGQIKIFPGIKRLTSDEVEFIDGRKEEFESIILATGYKTNIPSWLKNGDQFFNGKEGFLRNPFPNNWKGENGIYAAGFTKKGLLGISIDAHKIAQDIFSQWDSKTAISLLNYIN

>Aco004234|Acomosus_v3/1-467

MDSSRVTVIVSDDDVEVEKAAAAARSSITMPSPLQGGRKGDGDEKKVRVAVEGPIIVGAGPSGLAVAASLRVLSVPFVVVERSEGLAELWRTRTYDRLSLHLPKRFCHLPHLPFPRHFPTYPSKSYFLHYLHSYASRFRLAPHFRRAVTHARFDRALSLWRVTALERRGAADPSPSPSPSSSSNSTSEEYVEYVAPWVVVATGENAEAVVPETKGRELFTGRVVHSSEYRSGEEYSGGKVLVVGCGNSGMEICLDLCEHGAMPFMSVRSGVHLLPRDMFGTSTFGLVTKLLQWLPIKLVDRILLLAAKMVLGDTEKYGLKRPNLGPLETKHITGKTPVLDVGTLALIKAGKIQIVREVESLTSNGARFVDGSEKRFDAVIFATGYKSNVPLWLQDCDFFTEDGKPKSPFPNGWKGENGLYCVGFTGRGLHGAGQDAVKVAQDIAQKWTNLSHNNNNNNNNNNNNAVT

>XP_020586106.1|Phalaenopsis_equestris/1-417

MTTTAADDDGGLTASSATPSTSLTFIPGPIIVGAGPSGLATAASLHLLSIPSVIIERSDGIAHLWRHNSYDRLSLHLPKQFCELPHLHFPSHFPIYPSKSDFISYLLTYVDHFGLRPNFHCTATAARFNEEDSLWHVEAVRRSPEREEEEVVELVSPWLVVATGENAEPVVPEMKGKERFKGGVLHSSEYRNGVDCKGKRVLVVGCGNSGMEMCLDLCEFGAVPFLSVRSGVHVLPRDLLGISTFGLAMKLLKLLPVRLVDRILLLLARMAIGDTEKYGLKRPMKGPLELKNSTGKTPVLDVGAFKLIKKGRTKVVSEVESLTRDGAKFADGREMAFDSVIFATGYRSNVPLWLKDCDFFSADGKPKLPFPNGWKGENGLYCVGFTGRGLLGAGIDAQKVALDIADSWKKTSSIKNQ

>Asa6G02094.1|Allium_sativum/1-400

MEKISIVVPGPIIIGAGPSGLAVAASLHLKSIPFIILERSSNIADLWHNRTYDRLSLHLPKQFCNLPHLHFPPHFPSYPSKSQFLSYLHTYTNHFALTPLFNQQVVSTCYDESTSMWRVVSASKDKEDGVKDKEVEFVSKFVIVATGENAESVVPDMKGREIFKGGLMHSSEYKNGREYREKNVLVVGSGNSGMEMCLDLCEHGAFPYMSLRSGVHILPRELLGTPTFGLAMNLVKWFPVKFVDKILLLLAKICIGDTEKYGIKRPKIGPIELKNRTGKSPVLDVGALSLIKNGSIKVIVPEVESLSSNGAKFVDGREMKFDAIILATGYRSNVPFWLKGCDFFSKDGKPRTPYPNNWKGENGLYSVGFTSKGLFGASSDATKTANDIAEKWNMTKKLNL

>LOC_Os03g06654|Osativa_v7.0/1-421

MQGQQKQNAGGGGGDNASPCIVLDGPIIVGAGPSGLAVAATLRQHGAPFTVVERSGGVADLWTNRTYDRLRLHLPKVFCELPHVAFPPDFPTYPTKHDFLRYLHSYAARFAIAPLLRRTVTRAWYDHPASLWRVTTTTTSSSATSVITEYASPWLVVASGENAEVVVPKVKGRERFAGEALHSSEYRSGERFRGMRVLVVGCGNSGMEMCLDLCEHGAMPFMSVRSGVHVLPREMFGASTFGIAMKLLRWLPIKMVDRFLLLVARMVLGDTEKYGLKRPKLGPLEIKNITGKSPVLDVGAWSLIKSGNIKIVPEVESFSGNGARFVDGNEMAFDAVIFATGYRSNVPSWLQEDGELFTEEGKLRSSGSSSEWRWRGPNGLYCVGFSGRGLLGAGADALRAAADIAGRWQETQQAAANISSV

>Bradi1g74279|Bdistachyon_v3.2/1-411

MQEQANGTSPRQSSCLVNGPIIVGAGPSGLAVAATLRQHSVPFTILERSNGIADLWTNRTYGRLRLHLPKVFCELPHVRFPPDFPIYPSKHDFLRYLHSYAAHFSIAPLFGRAVTQARFDEAAALWRVTAVADGGEVTEYVSKWLVVASGENAEVVVPKVKGRERFAGEVLHSSEYKSGERFKGKRVLVVGCGNSGMEMCLDLCEHGAIPFMSVRSGVHVLPREMLWSSTFGIAMKLLRWLPVKLVDRLLLLAAKMALGDTEKYGLKRPKLGPLEIKDVTGKSPVLDVGAWSLIKSGNIKVVAEVESLGCNGARFVDGSEMAFDAVIFATGYRSNVPSWLQDAGGFFTEDGKPRARSPASSNWRGPNGLYCIGFSGKGLLGAGNDALRAAADIAGSWLQEQEQAAAGISPV

>HORVU.MOREX.r3.4HG0402190|Hvulgare_Morex_V3/1-421

MDTMQAEQGNNGGTSPRQSSCIVNGPIIVGAGPSGLAVAATLRRHSVPFTILERSDGIADLWTNRTYGRLRLHLPKVFCELPHVRFPPEFPIYPSKREFLRYLHSYTAHFSISPLFGRSVTRARFDEATSLWRVTAVSSADGGEVTEYVSKWLVVASGENAEVVVPRVKGRERFAGEVLHSSEYKSGERFKGKRVLVVGCGNSGMEMCLDLCEHGAIPFMSVRSGVHVLPREMLGSSTFGIAMKLLRWLPVKLVDRFLLLVAKLILGDTEKYGLKRPKLGPLEIKEVTGKSPVLDVGAWSLIKSGNIKVVAEVESLGCNGARFVDGNEMAFDALIFATGYRSNVPSWLQDDGFFTEDGKPKARCPSNWRGPNGLYCVGFSGRGLLGAGADAVRAAADIAGSWQEEMVAAGAGAGAATISPV

>ELECO.r07.3BG0293360|Ecoracana_v1.1/1-426

MQTTKQAAADISSNNTDTSSKNNKQSSTTSCCVVHGPIIVGAGPSGLAVAATLRRHGVPFTVLERSDGIADLWTNRTYDRLRLHLPKVFCELPHVAFPDDFPTYPTKHDFLRYLRSYAAGFAVSPMFGRAVTRARFDAAAALWRVTAVSDDGAETEFVSRWLVVASGENAEVVVPKVKGRERFEGEVLHSSEYRSGERFKGKRVLVVGCGNSGMEMCLDLCEHGAMPFMSVRSGVHVLPREMFGSSTFGIAMKLLKWLPVKLVDRFLLLVASMVLGDTEKYGLKRPKLGPLEIKNITGKSPVLDVGAWSLIKSGNIKIVPKVESFNSNGARFVDGNEMAFDAVIFATGYRSNVPSWLKDGDLFTEDGKPKAQHPSNWRGPNGLYCVGFSGRGLLGAGADALRAAADIAGRWQEMVAAAAGAKISSV

>ELECO.r07.3AG0234770|Ecoracana_v1.1/1-185

MFGGSTFGIAMKLLKWLPVKLVDRFLLLVASMVLGDTEKYGLKRPKLGPLEIKNITGKSPVLDVGAWSLIKSGNIKIVPEVESFNSNGARFVDGNEMAFDTVIFATGYRSNVPSWLKDGDLFTEDGKPKAQHPSNWRGPNGLYCVGFSGRGLLGVGADALRAAADIAGRWQEMVAAAAGAKISSV

>Sobic.001G495850|Sbicolor_v5.1/1-435

MQGQVAGDDAGGGSSRRRESLVRGPIIVGAGPSGLAVAATLSQHGVPFTVLERSDGIADLWTNRTYDRLRLHLPKVFCELPHARFPADFPTYPTKHDFLRYLRSYAARFGVSPLFGRTVTRARYDADASLWRVTAVSSSSSAADGGGVTTTTETEYASPWLVVASGENAEVVVPTVKGREMFAGEVLHSSAYRSGERFKGMRVLVVGCGNSGMEMCLDLCEHGAMPFMSVRSGVHVLPREMFGASTFGIAMKLLRWLPIKLVDRLLLLIARMVLGNTERHGLKRPKLGPLEIKNITGKSPVLDVGAWSFIKSGNIKIVPEVESFTGGNGVRFVDGNKMAFDAVIFATGYRSNVPFWLKDDGDLFTEDGTAKQAAGQPSGGDWWRGPNGLYRVGFSGRGLLGAGADALRAAADIAGRWQAEVAAAGGAKIVSSSSV

>Acora.04G203400|Aamericanus_v1.1/1-405

MAPTTNDESTPPRVAIPGPIIIGAGPSGLAVAASLQRLSVPSLLIERSDSIAHLWNHQTYNRLSLHLPKHLCHLPHLPMPKTFPTYPTKSQFMSYLLSYTQSFSLKPIFNSTAVSARFDPVGVVWRVVVARAGAGEVEYESRWLVVATGENAEPVVAAEEQAMGGFHGKKIHSAEYRTGEEFKGKRVLVMGCGNSGMELCLDLVEHGATPFMVVHVLPREMLGISTFELAMNMLKWLPMKMVDRFLVMVAKIMLGDTKKYGLKRPRLGPLELKKTTGKAPVLDIGTLSFIKTGKIKIVPEVECLTQKGAKFVDGREMQFDAVIFATNYRSNVPCWLKVTPHDCDFFTKEGKPKSQFLNTWKGENGLYCAGFTGKGLLGASTDAVKIASDVAGQWRTLLENKKVVS

>64527|Smoellendorffii_v1.0/1-386

WVEGAIIVGAGPSGMATAACLQQLGITSSILLEKSDCIASLWQERTYDRLRLHLPKKFCELPLAPFPSHFPIYPAKQQFLDYLHDYARRFHIRPRFGEIVQSARFDQRLQLWRVQTMKINNPDLGNISSEGNLSTMEYVGRWIVVATGENAEARIPVEIPGMDLFAGKIRHSSVYKSGARFAGQRVLVVGAGNSGMEIAMDLVQHDARPSIVVRSPIHILPREMLGKSTFGVSVAMLKFLPLWLTDRLLLLYALLALGDTSRYGIRRPKTGPLEMKEKMGKTPVLDVGTLAHIKQGNIKVEPAIECFTASGCKFVNGEQHCYDAIVLATGYKPNVPKWLKDPHLNFSSDGFPSCGWRGQRGLYVAGLSRKGILGVSKDARLIAQDI

>80431|Smoellendorffii_v1.0/1-404

MWVDGAIIVGAGPSGLATAACLSAAGIGSSVILEKNSCIGSLWQNRTYDRLRLHIPKQFCELPMSPFPDSFPIYPTRTQFVDYLENYAAHFQIRARFHECVQSAVFDPRLGLWRVRTIRESGEGDRERQAREYVGRWLVVASGENAEPLLPWDLPGLASFRGSVKHSSEFKNGCDYAGKSVLVVGSGNSGMEIALDLVQHNAKPAIVVRSPVHILPREMLGFSTYSVAMKLLKHLPVWLADRLLVSYAIAALGSTARHGIRRPDVGPMEMKVKTGRTPVLDVGTLSKIKAGKIKVRPSLESLSSCSARFSDGQQGDYDAIIFATGYKSNVPQWLKGEVGNSFSADGFPRCGWKGERGLYVAGLSRKGIFGGSKDAQMIAEDISKEYSLVHKLVSTNRAKQQQQQ

>Dicom.21G017200|Dcomplanatum_v3.1/1-408

MPPLVSEQWGKEKNDCMIAGKILSTWVDGAIIVGAGPSGLATAACLHEQGVQSLILEKSDCIASLWQHRTYDRLHLHTPKKLCELPLLSIPDSFPTYPTRQQFVDYLDDYARHFDIKPRFGEIVQSASFDEACKVWRVHSIDKSKSVASQKAEYAGNWLVVATGENAEVSIPELPGIQSYRGSLIHSSKYKSGADYEGGKVLVVGSGNSGMEIVLDLSNFNAKPSIVVRSPVHVLPREVFGRATYAVAMSLMKRVPIWLADLLLVLYSYLTIGSTAKYGIQRPKNGPLAMKEQLGRTPVLDVGTFAKIKSGGIKVMPAIESLTISGAKFVDGQCHSYNAIILATGYRCNIPRWLKGEDEVLSNDGFPNCGWKGRRGLYLAGLSRKGLVGTARDARLIAEDIRKACIVG

>EVM0002494.1|Lycopodium_clavatum/1-408

MPPLVSEQWGKETNNYMIPGRTLSTWVDGAIIVGAGPSGLATAACLHEQGVQSVILEKKDCIAPLWQHRTYDRLHLHTPKKLCELPLLPIPDSFPTYPTRQQFVDYLDNYARHFDIKPRFGEIVQSASFDETCQVWRVQSINKCKSVASLKAEYVGKWLVVATGENAEVSIPDLPGIKSYRGSLIHSSKYKNGADYEGDKVLVVGCGNSGMEIVLDLSNFNAEPSIVVRSPVHVLPREVFGRATYAVAIDLMKRVPIWLADRLLILYSYLTIGSTAKYGIKRPKNGPLAMKEQLGRTPILDVGTFAKIKSGGIKVMPAIESLTTSGAKFVDGQCHNYNAIILATGYRCNIPRWLKGEDEVLSNDGFPNCGWKGRRGLYLAGLSRKGLLGAARDARLIAEDIRRAYIHG

>Dicom.22G026200|Dcomplanatum_v3.1/1-404

MPPLVSEEINDYLISGKISSTWVDGVIIVGAGPSGLATAACLQEKGVKSVILEKSDCIASLWQNRTYDRLHLHTPKKFCGLPLLPIPDSVPTYPTRQQFVEYVKEYARHFDIKPRFGELVQSASFDETSKVWRVQSTNKSKSLASQKVEYVGKWLVVATGENADDRIPELPGLQSYRGRLIHSSRYKNGADYEGDKVLVVGSGNSGMEIALDLSNFNAKPSIVVRSPVHVLPREVFGRATYAVAMALMKRVPLWFADRLLVLYSYFIIGNTAKYGIKRPKNGPLAMKEHLGRTPVLDVGTFAKIKSGGIKVMPAIESLTTSGARFVDGRCQIYDAIILATGYKCNVPSWLKGEDEVFSDDGFPKCGWKGRRGLYVAGLSKKGLLGCAQDARLIAEDIKKAYISG

>Dicom.Y383100|Dcomplanatum_v3.1/1-404

MPPLVSEEINDYLISGKISSTWVDGVIIVGAGPSGLATAACLQEKGVKSVILEKSDCIASLWQNRTYDRLHLHTPKKFCGLPLLPIPDSVPTYPTRQQFVEYVKEYARHFDIKPRFGELVQSASFDETSKVWRVQSTNKSKSLASQKVEYVGKWLVVATGENADDRIPELPGLQSYRGRLIHSSRYKNGADYEGDKVLVVGSGNSGMEIALDLSNFNAKPSIVVRSPVHVLPREVFGRATYAVAMALMKRVPLWFADRLLVLYSYFIIGNTAKYGIKRPKNGPLAMKEHLGRTPVLDVGTFAKIKSGGIKVMPAIESLTTSGARFVDGRCQIYDAIILATGYKCNVPSWLKGEDEVFSDDGFPKCGWKGRRGLYVAGLSKKGLLGCAQDARLIAEDIKKAYISG

>EVM0010770.1|Lycopodium_clavatum/1-404

MPPLVSEEINDYLISGKISSTWVDGVVIVGAGPSGLATAACLQDKGVKSVILEKSDCIASLWQHRTYDRLHLHTPKNFCGLPLLPIPDSVPTYPTRQQFVEYVKEYARHFDIKPRFGEIVQSATFDETCKVWRVQSINKSRSIASQKVEYVGKWLVVATGENAEICIPDLPGLQSYRGGLIHSSMYKNGADYEGRKVLVVGSGNSGMEIALDLSNFNAKPSIVVRSPVHVLPREVFGRATYAVAMNLMKRVPLWIADRLLVLYSYLTIGNTAKYGIKRPKNGPLAMKEQLGRTPVLDVGTFAKIKTGGIKVMPAIESLTNCGARFVDGQCHSYDAIILATGYRCNVPSWLKGEDEVFSHDGFPKCGWKGSRGLYLAGLSKKGLLGSAQDAISIAEHIKKAYISG

>Dicom.14G066800|Dcomplanatum_v3.1/1-420

MPPFSSEQRSKESHDYIFEKSPILWFDGAIIVGAGPSGLATAACLKEQGVPSIILEKADCIASLWQHRTYDRLHLHLPKHFCELPLLPFPRSFPTYPNRRQFIQYLQDYADHFHIHPVYSENVLSASYDTQCKIWRVQSVNTSKGLSGQKREYVARWIIVASGENADVSIPNLPGIQAYRGSVIHSSKYKNGSEYARKKVLVVGSGNSGMEIALDLANHQAKPSLVVRSPVHVLPREMFGVPTFAIIMTLLKTLPLWLADRLLVLSAFLTYGDTAKYGIKRPSTGPLEIKQTTGRTPVLDVGTVSKIKSGQIRVMPAMECLISSGATFIDGHSEEYDAIILATGYRSNVPSWLKGEERNFSAEGISTCGWKGGRGLYVVGLSRRGIFGAGLDAKHIAEDIREAYLSEQQAYLKRFRVSSR

>Dicom.14G066500|Dcomplanatum_v3.1/1-407

MPPFSSEQRSKESHDYIFEKSPILWFDGAIIVGAGPSGLATAACLKEQGVPSIILEKADCIASLWQHRTYDRLHLHLPKHFCELPLLPFPRSFPTYPNRRQFIQYLQDYADHFHIHPVYSENVLSASYDTQCKIWRVQEYVARWIIVASGENADVSIPNLPGIQAYRGSVIHSSKYKNGSEYARKKVLVVGSGNSGMEIALDLANHQAKPSLVVRSPVHVLPREMFGVPTFAIIMTLLKTLPLWLADRLLVLSAFLTYGDTAKYGIKRPSTGPLEIKQTTGRTPVLDVGTVSKIKSGQIRVMPAMECLISSGATFIDGHSEEYDAIILATGYRSNVPSWLKGEERNFSAEGISTCGWKGGRGLYVVGLSRRGIFGAGLDAKHIAEDIREAYLSEQQAYLKRFRVSSR

>EVM0015133.1|Lycopodium_clavatum/1-420

MPPFYSEQRSKESHDYILEKSPTFWVDGAIIVGAGPSGLATAACLKEQGVPSIILEKADCIASLWQHRTYDRLHLHLPKHFCELPLLPFPRSFPTYPDRKQFIQYLQDYADHFHIHPVYSENVMSASYDAQCKIWRVQSVNTTKGLLDQKREYVARWIVVASGENADVSIPNLPGIQSYCGSVIHSSKYKNGSEYSRKKVLVVGSGNSGMEIALDLANHHAKPSLVVRSPVHVLPREMLGVPTFAIIMTLLKTLPLWLADRLLVLSAFLTYGDTAKYGIRRPSTGPLEIKQKTGRTPVLDVGTVSKIKSGQIRVMPAMERLISSGAKFIDGRSEDYDAIILATGYRSNVPNWLKGEDRNFSSEGISTCGWKGGRGLYVVGLSRRGIFGAGLDAKHIAEDIREAYLSEQQAYLKRFRVSSR

>Mapoly0063s0040|Mpolymorpha_v3.1/1-420

MPPYASESLGKESHDLFLNIMPRSVDGAIIVGAGPSGMATAACLKEKGVPSIVLEKAECIGSLWKHKTYDRLHLHIPKEFCELPLMPFPASYPTYPNRQQFLDYLEEYARHFDIQPHFNESVQSAHYDESSQLWRVNTQTTGKSSCNKIEEYTARWLIVASGENAEIVTPKLPGMKDYQGDLLHSSKYKNGSEYVGKKVLVVGCGNSGMEIALDLANFSAKPSLVVRGPVHVLPREIFGTSTFSVAVKLMRTFPVWLTDWLLVFYTWLTLGNVANYGFNRPKEGPMELKDKVGKTPILDVGTLNKIKSGHIKVVPRVVSLTSSGVRFENGLTDSYDAVILATGYRSNVPRWLKGEEKFFADDGFPKRPTTNGWKGEHGLYAVGLTRKGLLGASMDARNIAEDIRKAYDTRNLVSRASEIL

>Rn_10143.1_1|Ricciocarpos_natans/1-421

MPRYASESLGKESHDLYLNRMPIKVDGAIIVGAGPSGMATAACLKEKGVPSIVLEKADCIGSLWKHRTYDRLHLHIPKEFCELPLLPFPASYPTYPNRQQFLDYLESYARHFDIEPHFNESVQNAHYDEAAEIWRVNTVATGKSSSNKVEEYTARWLIVASGENAEIVMPKLPGMKDYQGDLMHSSMYKNGADYVGKKVLVVGCGNSGMEIALDLANFNAKPSLVVRGPVHVLPREIFGTSTFSVALKMMRTFPVWLTDWLLVFYTWLTLGDVANYGFNRPKDGPMERKDKLGKTPILDVGTVNKIKSGHIKVVPQVVSLTSDGVRFENGETDSYDAVILATGYRSNVPRWLRGEEKFFTDDGFPKRPSTNGWKGEHGLYAVGLARKGLLGASMDARNIAEDIRCAYENQKLSEICGSSRR

>Rn_10143.1_2|Ricciocarpos_natans/1-421

MPRYASESLGKESHDLYLNRMPIKVDGAIIVGAGPSGMATAACLKEKGVPSIVLEKADCIGSLWKHRTYDRLHLHIPKEFCELPLLPFPASYPTYPNRQQFLDYLESYARHFDIEPHFNESVQNAHYDEAAEIWRVNTVATGKSSSNKVEEYTARWLIVASGENAEIVMPKLPGMKDYQGDLMHSSMYKNGADYVGKKVLVVGCGNSGMEIALDLANFNAKPSLVVRGPVHVLPREIFGTSTFSVALKMMRTFPVWLTDWLLVFYTWLTLGDVANYGFNRPKDGPMERKDKLGKTPILDVGTVNKIKSGHIKVVPQVVSLTSDGVRFENGETDSYDAVILATGYRSNVPRWLRGEEKFFTDDGFPKRPSTNGWKGEHGLYAVGLARKGLLGASMDARNIAEDIRCAYENQKLSEICGSSRR

>Rn_10143.1_3|Ricciocarpos_natans/1-421

MPRYASESLGKESHDLYLNRMPIKVDGAIIVGAGPSGMATAACLKEKGVPSIVLEKADCIGSLWKHRTYDRLHLHIPKEFCELPLLPFPASYPTYPNRQQFLDYLESYARHFDIEPHFNESVQNAHYDEAAEIWRVNTVATGKSSSNKVEEYTARWLIVASGENAEIVMPKLPGMKDYQGDLMHSSMYKNGADYVGKKVLVVGCGNSGMEIALDLANFNAKPSLVVRGPVHVLPREIFGTSTFSVALKMMRTFPVWLTDWLLVFYTWLTLGDVANYGFNRPKDGPMERKDKLGKTPILDVGTVNKIKSGHIKVVPQVVSLTSDGVRFENGETDSYDAVILATGYRSNVPRWLRGEEKFFTDDGFPKRPSTNGWKGEHGLYAVGLARKGLLGASMDARNIAEDIRCAYENQKLSEICGSSRR

>AANG012409|Anthoceros_angustus/1-315

MPSFYAHGWGPESEEADPDPLVRLVEGPVIVGAGPAGLATAASLRAKAIPSLVIERADCIASLWKYKTYDRLHLHIPKHLCELPLMPFPEECPAYPTRQHFIAYLEEYAAKFQIKPQFNESVTSATFDSAINLWRVRTCRHGPQLSQAQEYVTRWLIVATGENAEAVTPRLPGMDLYAGPAFHSSLYRNGSDFRNKRVLVVGCGNSGMVIALDLANHGARPTIAVRSPVHVLPREVLGRSTLQVAMTLLRVFPMWLTDYIVVFYTWLLMGNTARYGICRPAKGPIELKDTLGKTPVLDVGTLAKIRSGHVKVCVR

>Sphfalx16G065000|Sfallax_v1.1/1-467

MPPYSSYADEAFSAKQEYMGKAANDLRFCCSSEPEAAAAALAPATTTTFWNGAIVVGAGPSGLATAACLELKGVPCLILEKSDGIASLWKHKTYDRLHLHIPKQFCELPHLPFPADYPTYPDRQQFVDYLENYATRFKLDFRFNEQVTAAAFDSAAGHWKITTRNTNNTLLGGGGLMLQQQGGTVSSEFRARWLVVASGENAEAVCPVIPGAADFKGPVLHSSQYKSGADYKGRNVLVVGAGNSGMEIALDLFNFNARPSLVVRSPVHILPREVFGKSTFAVAMRLLKTFPIWFTDSLLVWYTWALMGDTSSYGFHRPAVGPMALKSKSGKTPVLDVGTFARIKNRDIKVVPAIDHLTSNGAQFVDGQVQQFDAIILATGYRSNVPQWLNDKGNFFSEDGFPRNPSPRSWKGAETGLYVAGLGRKGILGAVFDAKNIADDISQLFFADELESHIRVQPVLQQQQQLR

>Sphfalx19G049700|Sfallax_v1.1/1-506

MPPYESHSTYAAAEAAAEAFYSKQEYMGKAANDDSNRRLCCRSAVTVAAVAAAAAPVVTFWNGAIVVGAGPSGMATAACLQLKGVPCLILEKSDGIASLWKHNTYDRLHLHIPKEFCELPHLPFPADFPTYPNRRQFIQYLDNYAAHFKLDFRFNQQVTSAAFDPAAACWKITTRSCNTSSAAGAGLAAAAAAGAVVSEFRARWLVVASGENAEAVCPVIPGAGDFKGRVLHSSRYKSGADYQGLRVLVVGAGNSGMEIALDLTNFNVTPSLVVRGPVHILPREVFGKSTFAVAMKLLKTFPLWFTDSFLVWYAWALMGDTNSYGLHRPAEGPMTIKTKLGKTPVLDVGTFAKIKNGDIKVVPAIDHLTSNGAQFADGQVQQFDAIILATGYRSNVPQWLNDNCNFFSEDGFPRNPSPKAWKGETGLYIAGLGRKGILGAAFDAKNIADDISQLFFAAELESHARVQPAPQQQQQQLLRKPQMLQVPSFQPNAPTTQPPQLHTPMN

>Sphfalx17G062100|Sfallax_v1.1/1-478

MPPYSSYVAEAYSSCQEQQEELLDKGRKAGDEHDTKLLFCSSSTRVSQAVPLDSSSSSSPTFWKGAIVVGAGPAGLATAACLELKGVPCLIVEKSDGIASLWKHNTYNRLHLHIPKQFCELPHLPFPEDFPTYPNRQQFVDYLDKYAARFDLHFRFNEKVIAAAFDTAAGYWKITTMHCNHNNTVLESSGGRGGVLSEFRARWLVVASGENAEAVCPVIPGARHFKGPVVHSSQYKSGADYKGRNVLVVGAGNSGMEIALDLSNFNARPSLVVRSPVHILPREVFGKSTFAVAMKLVKNFPLWFADSFLVWYTWALMGNTSSYGLHRPAQGPMTLKLKFGKTPVLDVGTFAKVKNGDIKVVPAIDHLTSDGAQFTDGQVQQFDAIILATGYRSNVPQWLHDKSNFFSEEGFPRNSSAEAWKGETGLYVAGLGRKGILGAAFDAQNIAQDISQLFFAAELESHNRVQPVPQQHQHQQFR

>Pp6c1_6340|Ppatens_v6.1/1-432

MPPYLASWNPSGVEYLGKGGNDSLLMSKPLPTVVEGAIIVGGGPSGLAAAACLEMKGVPSLIIEKSDGIASLWKYKAYDRLHLHIPKQFCELPYYSFPEDYPLYPNKKQFVDYLENYFQHFDMRARFNTEVVCASYDPSSSYWKVQTRPSGSGLDSEGMLEFRAQWLVVASGENSEAYTPKMEGLDSFQGPVLHSSNYRTGAHYQGQRVLVVGCGNSGMEIAMDLANFNAKPFLVVRSPVHILPREIFGTSTFAVAMRMMKTFSLWFTDLLLVGYTRAVLGDITSYGFKRPTDGPMTIKCKQGKTPILDVGTFAKIKSGTIKVCPGVDHLTPTGSKFENGQFEEFDAIVLATGYRSNVPRWLRDESGFFSADGLPKNLSQGTWKAERGLYVAGLGRKGILGATFDAKNIAEDISIVYRSDSHKRLISPLQSR

>Pp6c13_10900|Ppatens_v6.1/1-427

MPPYFAPGMECLGKAGNESFMTREPFPTFVEGAIIVGGGPSGLAAAACLKMKGVPSLIIEKSDGIGSLWKYKAYDRLHLHIPKQFCELPYYAFPEDYPLYPDRKQFVDYLENYFQHFDMWAKFNTKVSTASYDPYSSCWKVKTQPSEADSEGERREYRAKWLVVASGENAEPYTPEVEGLKDFRGSVVHSSNYKTGAGYARQRVLVVGCGNSGMEIALDLSNFNAEPSLVVRSPVHILPREIFGTSTFAVAMRMMKSFPLWFTDACLVWYTWAMLGDTTRYGFKRPSDGPMTIKCKQGKTPILDVGTFAKIKSGAIKVCPGLKYVTPDGALFENDQFVKFDAIVLATGYRSNVPQWLKDDSGFFTAEGLPKNHSKGTWKAERGLYIAGLGRKGILGATFDAKYIAEDLSRAYASESHKRLICPLQSR

>Pp6c3_10660|Ppatens_v6.1/1-430

MPPCFVSPYSAGAEYLGKAGNDSFMMGSFASTFVEGAIIVGGGPSGLAAAACLKMKGVPSLIIEKSDGIGSLWKYKAYDRLHLHIPKQFCELPYYPFPEEYPLYPNRKQFVDYLENYFQHFDMRAKFETKVKTASYDPRLSCWKVDTQPSGADSECVTEYRARWLIVASGENAEPYTPDLSGLRDFKGPVLHSSTYKTGADYEGQRVLVVGCGNSGMEIALDLANFRAKPSLVVRSAVHILPREIFGTSTFAVAMRMMKSFPLWFTDMLLVWYTWAMLGDTTSYGFKRPTDGPMTIKCKQGKTPILDVGTFAKIKSGAIKVCPGVEYLAPHGAHFANRQFAEFDAIVLATGYRSNVPQWLKDDSGFFSAEGLPKNPSHGTWKAERGLYIAGLGRKGILGATFDAKNIAEDISEAYSSESHRILVSPLQHR

>Pp6c2_15130|Ppatens_v6.1/1-436

MPPYFAFSHSPGLEEMTREAGNVSSVMANSAPTFVEGAIIVGGGPSGLAVAACLTMKGVPSLIIEKSDGIGSLWKYKAYDRLHLHIPKQFCELPHYPFPEDYPMYPNRKQFVDYLENYFRHFDMRAQFNTKINAAYYDPQLSCWKVESQPSSAGSGECVTEYCSRWLIVASGENAEAYTPDMQGLRDFRGPVLHSSNYKTGADYMGQRVLVVGCGNSGMEIALDLANFNAKPSLVVRSPVHILPREILGVSTFAVAMSMMKRFPLWFTDALLVVYARAVLGDTTSYGFKRPANGPMTIKCEQGKTPILDVGTFAKIKSGAIKVCPGVTHLTSQGAKFENDQFAEFDAVVLATGYRSNVPRWLSDDGGFFSPEGLPKNPSLGTWKAGRGLFVAGFGRKGILGATFDAKYIAEAISDAWSLESHKHLVSPLKSRYPPP

>Pp6c11_6670|Ppatens_v6.1/1-431

MPPHVTATGYWVKGATQDLSTRKKQPPSYCNGAIIVGAGPSGLATAACLKKLGVHVLILEKSSCIASLWQTKTYDRLHLHLPKQFCELPHSPFPVDFPTYPTRHQFVEYLQEYAARFKLQPLFNHSVDLANFDSRSGLWHVHVTCAGGKGDPDREFRARWLVVASGENAEPVIPSFKGSKEFRGGSKIFHSSRYRNGTEYEGKKVLVVGCGNTGMEIALDLANFGAYPSIVVRSPTHILPREMMFKSTFLVAMKLMEKLPVWLVDWLLLAYAYSALGNTAKFGIHRPSEGPMVLKEKHGKTPILDVGTLKLIKSGQVKVLPAVERLTPTGALFEDGRLEEFDAVILATGYKSNVPSWLKDDSKFFSDEGFPRQPFPHGWKGENGLYVAGLGRKGLLGASKDATRIAKDIYDGSLTEITSLRLVPCQLNQRI

>AANG012408|Anthoceros_angustus/1-356

MPFPEDYPAYPTRQQFIDYLEAYARRFQINPHFNESVVSAVFDAGIGMWRVRTSRGLGGGGGRPCVSEEYVSKWLIVATGENAESVTPQLPGMDVFRGQAFHSSLYRNGSDFRDKKVLVVGCGNSGMEIALDLANHGAKPSIAVRSPVHVLPREVLGRSTLQVAMALLRIFPLWLTDYILVLYTWLLMGNTARYGLCRPAKGPMELKDTLGKTPVLDVGTVAKIRSGHIKVTSAVKALSATGATFVDGSWEDFECVVWATGFRSNVIKWLKDDTFFGVNGLPKVPFPHGWKGQRGLYAVGMSRRGLKGTSFEARQIAQDISASFEKQICVCPMPPVPVSSRAVYRMSHNTHPSRSR

>MA_70477g0010|Picea_abies/1-378

MKGISSTVIEKEDCIASLWKNKTYDRLKLHIAKEYCQLPFMPFPDSYPTFVTRNQFLDYLELYAQQFQIRPQFCQVVESASFCENSQTWHVLCSVRYNYADQPNLVEYKEYIGRWLVVATGENAEVVRPHISGIESFKGTVIHSSEYKTGFEYEDKKVLVVGCGNSGMEIALDLVSYNAKPSLVVRSSIHVLPRQVLGKSTFGLAMKLMKVFPVRIVDSLLLLYSRLTLGNTASYGLRRPVEGPLELKSKTGKTPVLDVGTLTKIRTGDIKVYPSIEGITATGVQFQNGHAEEYDVIILATGYRSDVPRWLKDDGRLFSEVGLPTSPFPENWKAVGKKGLYAAGFSRRGLLGSSIDAQLIAGDIKNSYNNYINSPVKS

>PITA_50706|Pinus_taeda/1-436

MPQFSISLEQLAKQINDPQFIKCNAKGYYCNSKRLEEVEGAIIVGAGPSGMASAVCLKMEGIPSIVIEKEECIASLWKNKTYDRLKLHIAKEYCQLPFMPFPESYPTFVSRDQFLDYLDRYAQQFQIQPRFCQVVESARFCENSQRWHVLCSGRYDHTDLPNAVEFKEYIGKWLVVATGENAEVVRPHISGIESFKGTVIHSSEYKNGFEYEYKKVLVVGCGNSGMEIALDLVNYNARPSLVVRSSVHVLPRQMMGKSTFGLAMKLMKLFPVRIVDRLLLLYSKFTLGNTASYGLSRPVEGPLELKSKTGKTPVLDVGTVAKIRAGDIKVFPNIKGITATGVQFEDGHTEDYDVIILATGYRSDVPRWLKDDGGFFSEEGLPKAPFPNNWKAVGKKGLYAAGLSRRGLLGSSIDAQLIARDIKEGYNNCVNNSVRS

>PITA_32408|Pinus_taeda/1-479

MPSFSLSLEQQANGCPQFINYNGKEYYSHCMRSEEVEGAIIVGAGPSGMACAGCLKMEGIPSIVIEKEECIASLWKNKTYDRLKLHIAKEYCQLPFMPFPESYPTFVSRDQFLHYLERYAQQFQIQPHFCQVVESARFCENSQRWHVLCSVRYNYTDRPNAVEFKEYVGKWLVVATGENAEVVRPHIAGIESFKGTVIHSSEYKNGFEYEDKKVLVVGCGNSGMEIALDLLNYNAKPSLVVRSSIHVLPRQILGKSTFGLAQTLMKLFSVRIVDRLLLLYSRLTFGNTDSYGLRRPVEGPLELKRKTGKTPVLDTGTLTKIRTGDIKVYPSIDGITATGVKFEDGNAEDYDVIILATGYRSDVLRWLKDHGRFLPEGELPTAPFPNNWKPVGKKGLYAAGFSRKGLLGTSIDAQLIARDIKNDYNNYIKSSVYPSINGITATGVKFEDGNAEDYDVIILATGYRSDVLRWLKDDGKFLP

>CYCAS_030297|Cycas_panzhihuaensis/1-424

MPPFSAVLEQLAKETNDLQLPKSANNNCKLRLIEVGGAIIVGAGPSGMASAVCLKMRGISSVVIEKEECIASLWKNKTYDRLKLHIAKEYCELPFMPFPESYPTFVPRKEFLDYLEVYARCFQIQPCFRETVEKAIFCESSGRWHVRTISHCCVHHTSTVLRKEYVGRWLVVATGQNAEVVWPNLAGLEGFKGSVIHSSQYKTGLGYEDKKVMVVGCGNSGMEIALDLANCRAKPSIVVRSAVHVLPREILGRSTFSVAMQLMKYLPVRMVDRLLLLYSRLVLGNTASYGLARPEEGPLELKNKTGKTPVLDVGALAKIRSGDIKILPNIKCVNPAGAEFEDGRVEEFDAIIFATGYRSDVPRWLKDDDEFFSADGLPKAPFPNNWKGKKGLYAAGLSWRGLFGTAVDAQLIARDISDAFYESV

>Gb_15900|Ginkgo_biloba/1-412

MLKMERFSAFTEQLAKEGNDNAGRLKIHAEGAIIIGAGPSGMATAACLVNLGISSTIIEKEYCIAPLWQHGTYDRLRLHIGKNFCDLPLVSFPEDYPTFVTRKQFVEYLEAYAKKFNIQPRFRQAVESAEFCQETGNWHVFTRGCADCQEREYVGRFLVVATGENGEVFVPDVPGLESFKGSVVHSSAYKDGLAYKDKKVLVVGCGNSGMEIALDLANNNAKTSIVVRSSLHVLPREIFGRSIFSVAMQLTKLLPVGIVDYLLVLCSKLTLGDTVAYGIPRPIQGPLELKNKLGKTPVLDVGTVAKIRSGDIKVVPNLNRITATGVEFENGHVQEFDAIIFATGYRSTVPTWLKDADDFFSKEGFPRMPFPNNWKGKNGLYAAGLGRRGLSGSSLDAHRIAQDISKAYNRSP

>MA_31349g0010|Picea_abies/1-408

MANFSTRPEQVAKKEHDKSFNEFGEKEVIIVGAGPSGMAVAACLANHGISSVVLERELCLASLWRHRTYDRVQLHIDKKFCELPLMPFPDEYPMFVTRDQFIVYLEEYAKRFNIQPRFCQSVESASFFQETGKWHVLARCSSDNSEREYLGRFLVVATGENGEKIMPDVSGLENFAGTVIHSIDYKNGKDFSGKKVLVVGCGNSGMEIALDLANNEAKPSMVVRSPSHVLPRKVFGRSTFSVAMLLLKLLPLRMVDNLLVMYCHLTLGDTASHGIPRPVEGPFEMKLKTGKTPVLDNGTIAKIRQGKIKVVPNVNRATATGVEFEDNREEDFDAIIFATGYRSKVHRWLKNGGELFSDDGFPRKPSPDNWKGSDGLYAAGLGRTGLMGSSADARQIAQDIFQAVSTSP

>PSME_00047339-RA|Pseudotsuga_menziesii/1-165

MQLLKLLPVRIVDNLLIMYSNLTLGDSASHGILRPVRGPLEIKFKTGKTPVLDNGTVAKIRQGNIKVVPNFKRVTATGVEFEDNREEDFDAIIFATGYRSVVRRWLKDGGELFSDDGFPRKPSPDNWKGNDGLYAAGMGRAGLQGSSKDALLIAQDIFQAVSTSS

>PITA_26624|Pinus_taeda/1-406

MANFSTPSEQLAEKDHRKSFDDSGDAAVIIVGAGPSGMAVAVCLANLGIASVILERERCLASLWKYRTYDQIHLHIDKKFCELPFMQFPDEYPMFVSRRQFIDYLDEYAKRFNIHPRFCQSVESASFSEETGKWHVLARCSLDNSQKEYLGRFLVVATGENGEKFVPDVSGLEGFAGTVIHSIDYKSGKEFEGKKVLVVGCGNSAMEIALDLVNNNARPSVVVRSPSHVLPRKVLGRSTFSVAMLLLKLLPLRVVDNLFVMYSNLTLGNTNSLGIPRPVQGPFDMKFKTGKTPVLDDGTMAKIRQGKIKVVPNFRRATATGVEFEDNLEEDFDAIIFATGYRSAVLRWLKNSGEFFSDDGFPRKPSPYNWKGREGLYAAGLGRAGLLGSSMDARLIAQDIFQAVNT

>PITA_00074|Pinus_taeda/1-411

MPNFSQPSEQIGKNEYDKSSFDENRDEEVIIVGAGASGLAVAACLADLGISSVVLDRDVCVGSLWKNRTYDRVTLQGSKQHANLPLMPVPDEYPMFLTRDHFVAYLEKYARRFNIQPQFCQSVESASFSQETGRWHVIARCSSDNSEREYHGRFLVVATGEFNEKFMPDVPGLDSFRGTVIHSIDYKNEKDFEGKRVLVVGWGNSGMEIALELLNNEAGPRPSIVVRSPNHVLPREVFGRSTVSLALQLHKVLPLRMVDNLLLMYSNLALGNTASYGLPRPVEGPLEFKFKTGKTLVIDHGTVDKIRQRKIKVVPNFIRVTATGVVFEDGVEEDFDALIFATGYKSGVRRWLKNGGELFPEDGRPGNRPPSDWKGSDGLYVAGFGKRGLLGLSEDGQRIAQDIFQSLKNAS

>PITA_46603|Pinus_taeda/1-410

MPNFSQPSEQIGKKGHDKSFDENRDEEVIIVGAGASGLAVAACLADLGISSLVLERDFCVGSLWKNRTYDRVTLQGSKQHAHLPLMPVPDEYPMFLPKDHFAAYLEKYARNFDIQPQFCQSVESASFSQETGRWHVIARCSSDNSEREYHGRFLVVATGEFNEKFMPDVPGLDSFRGTVIHSIDYKNEKEFEGKRVLVVGWGNSGMEIALELLNNDAGPRPSIVVRSPNHVLPREVFGRSPISLALQLLKLLPLRIVDKLLLMYSNLVLGNTASYGLPRPVEGPLEFKFKTGKSLVIDNGTVDKIRQRKIKVVPNFIRVTATGVVFEDGVEEDFDALIFATGYKSEVRRWLKNGRELFPEDGRPGNRPPSDWKGSDGLYVAGFGKRGLLGVSQDAKRIAQDIFQSLKNAS

>PITA_02185|Pinus_taeda/1-410

MPNFSRPSEQIGKKEHDKSFDENRDEEVIIVGAGASGLAVAACLADLGISSLVLERDFCVGSLWKNRTYDRVTLQGSNQHSSLPLMPVPDEYPVFLTRDHFSAYLEKYARRFKIQPQFCQTVESASFSQETGRWHVIARCSSDNSEREYHGRFLVVATGEFSEKFIPDVPGLDSFRGTVIHSIDYKNEKDFEGKRVLVVGCGNSGMEIALELVKNDAGPRPSIVVRSPNHVLPREIFGRSTVSLAFQLHKLLPLRMVDNLLLMYCNLVLGNTASYGLPRPVEGPLEFKFKTGKTPVIDHGTVDKIRQRKIKVVPNFIRIMATGVVFEDGVEEDFDALIFATGYKSEVRRWLKNGRELFPEDGRPGNRPPSDWKGSDGLYVAGFGKRGLLGASEDGQRIAQDISQSLKNAS

>PITA_27422|Pinus_taeda/1-411

MPNFSRPSEQVGKKEYDKSSFDENRDEKVIIVGAGPSGLAVAACLADLGISSLVLERDSCVGSLWKNRTYDRVTMQGGKQLSNLPLMPVPAEYPMYLTRDHFVAYMEKYARRFNIQPQFCQSVESASFSQETGRWHVITKCSSDNSEREYHGRFLVVATGEFSEKFMPDIPGLDSFRGTVIHSIDYKNEKAFEGKRVLVVGCGNSGMEIALELVNNDAGPRPSMVVRSPNHILPREFFGIPTMPIALQLYKLLPLRMVDNLLRLCSNLALGNTASYGLPRPVEGPLEFKLKTGKTLVIDDGAVEKIRQHKIKVVPNFIRVTATGVVFEDGVEEDFDAVIFATGYKNEVRRWLKNGKELFPEHGRPGNRPPSDWKGSDGLYIAGFGKKGLLGTSEDARRISQDIFQSLKNAS

>PITA_18009|Pinus_taeda/1-411

MPNFSRPSEQVGKKEYDKSSFDENRDEKVIIVGAGPSGLAVAACLADLGISSLVLERDSCVGSLWKNRTYDRVTMQGGKQLSNLPLMPVPAEYPMYLTRDHFVAYMEKYARRFNIQPQFCQSVESASFSQETGRWHVITKCSSDNSEREYHGRFLVVATGEFSEKFMPDIPGLDSFRGTVIHSIDYKNEKAFEGKRVLVVGCGNSGMEIALELVNNDAGPRPSMVVRSPNHILPREFFGIPTMPIALQLYKLLPLRMVDNLLRLCSNLALGNTASYGLPRPVEGPLEFKLKTGKTLVIDDGAVEKIRQHKIKVVPNFIRVTATGVVFEDGVEEDFDAVIFATGYKNEVRRWLKNGKELFPEHGRPGNRPPSDWKGSDGLYIAGFGKKGLLGTSEDARRISQDIFQSLKNAS

>PITA_26118|Pinus_taeda/1-411

MPNFSRPSEQVGKKEYDKSSFDENRDEKVIIVGAGPSGLAVAACLADLGISSLVLERDSCVGSLWKNRTYDRVTMQGGKQLCNLPLMPVPDEYPMYLTRDNFVAYMEKYARRFNIQPQFCQSVESASFSQETGRWHVITKCSSDNSEREYHGRFLVVATGEFSEKFMPDIPGLDSFRGTVIHSIDYKNEKAFEGKRVLVVGCGNSGMEIALELVNNDAGPRPSMVVRSPNHILPREFFGIPIMPIALQLYKLLPLRMVDNLLLLCSNLALGNTASYGLPRPVEGPIEFKLKTGKTLVIDDGTVEKIRQHKIKVVPNFIRVTATGVVFEDGVEEDFDVLIFATGYKNEVRRWLKNGKELFPEHGRPGNRPPSDWKGSDGLYIAGFGKKGLLGTSEDARRISQDIFQSLKNAS

>Thupl.29379968s0014|Tplicata_v3.1/1-417

MAYRGDFLEQLAKIEQDINLSKIENGEAIIVGAGPSGMAVAACLSKLGVRSTVLERESCVASLWKQRTYDRLKLHIDKKFCELPFMSFPSQFPTFVSRNQFVEYLEQYARRFSIRPLFCQSVESANFCQETGKWRVVTTCSVERRKRIYTGRFLVVATGENSEKFVPDLPGIERFEGIVVHSSEYKSGEDFKGKKVLVVGCGNSGMEIALDLLQKDASPSIVVRSPLHVLPREIFGMSTFFVFTCLLRIFPLRIVDRLLLIYSRLILGDSTPYGIARPLDGPLELKRNLGKTPVLDVGTMAKIRSGEIKTFPKIKRVMTAFDVEFEDNRVESFNAIIFATGYRSGVLRWLKDEGDFFGEDGFPRETFSNNWKGKDGLYTVGLGRRGLIGTSIDARLIAEDIKACSTSSSPANYAHLV

>KAH9311081.1|Taxus_chinensis/1-407

RAKMEQDKSSCAFETDGAIIVGAGPSGMAVAACLKKVGVCSIVLERESCVASLWKRRTYDRLKLHISKNFCELPFMEFPSEFPTFVGRDQFVEYLEEYAKRFSIQPRFCQSVESATFSRETGKWRVITMCSLEKLEKIYSGRFLVVATGENNEKFVPDLPGIETFEGSVIHSSEYKSGEGFKDKKVLVVGCGNSGMEIALDLLHKDARPSIVVRSPLHILPQKILGLSTFSVLTCLMNYFSLRTVDRLLLLYSRLSLGDTSAFGIARPVQGPLELKRKIGKTPVLDVGTMAKIRTGDIKISPNIKRVMALNVEFEDNRVERFDAIILATGYRSGVMRWLKEEGDFFSDNGFPREAFPNNWKGKNGLYAVGMGRRGLLGTSIDAQLIAQDILQVYNSSCSLFDHCLTK

>Adc01409|Adiantum_capillus-veneris/1-463

MRDLIASLSYSSMQVFLSTVTRYFLSVLHLSIASRHASASTQTEEPPVEAASAPRPMVSSNPFDRVIIVGAGPSGLATAACLQRRGVPFVILERAGCMASLWKEHTYDRLKMHLSKESCRLPFMDFPEKYPTFVTRQQFIDYMEDYAKTFSIQPRYNETVQTASFDEASQTWCVQTTAVGPFAEGPRRKLTHQGRWLVVATGENAEMVLPDDMPGLNEFKGGYVHSSQYRNGKAYTGKKVLVVGSGNSGMEIALDLTNFGADASIVVRSPVHILTLEMFGMSTFSVAMRLLKRLPMWLVDYLLVAYSRLVLGDTAAVGIERPKVGPLELKCKMGKTPVLDVGALQRIQAGDIKVRPAVERVMETGVRFKDGSSAHFDALIFCTGYKSTVLHWLKQDKERFFGTDSLPIGRACGQSWKGKNGLYVAGMCNKGILGGTTDAERIAHHVYTTHFAKPTALPLPLSS

>Ceric.05G083300.1.v2.1|Ceratopteris_richardii/1-466

MPGVSMAISSPMCRSPMVQDNTKPREEDQLDSPSLMLNMQPEESFNIQSGRSERVIIVGAGPSGMATAACLRMKGIPYIIIERAGCIASLWKERTYDRLKMHLSKESCRLPFKPFPDSYPTFVSRQQFIDYLEEYASEFGIQPRFNETVQSASFDEASQTWCVQTIVVGPFAEGPRRKLTHMARWLVIATGENAEMIVPELPGLNEFRGGYIHSSQYRNGDFYGGKKVLVVGCGNSGMEIALDLANCGADASIVVRSPVHILTREIFGMSTFSVAMRLMKSLPMWLVDYMLVSYSRLALGDTAVCGIERPKVGPLELKCKMGKTPVLNVGTFERIQAGDIKVRPAVERLTETGARFVDGTLEDYDAVIFATGYKSTVLRWLKDKENFFGVDSLPRDRACGQSWKGRRGLYVAGMCNKGILGGSTDAERIANDVHCIMTAKTASIATFATPPCGLTPPYSSSTSLTA

>Ceric.07G071400.1.v2.1|Ceratopteris_richardii/1-449

MPASMMESKYVPRNSNEEAILEMIASMGRVEDHTAPRMAHAEDSNSAQNVWTDRVIIIGAGPSGMATAASLRRKGIPYVLLERASGIASLWKERTYDRLKMHLAKEACELPFKAFPERYPTFITRRQFIDYLEEYARDFCIQPRFNETVQSATFDEASQTWQVQTIAVGPFAEGPRRKLVHTGRWLVVATGENAEMVLPSMPGLNEFKGTYIHSSQYKNGEAYAGKRVLVVGSGNSGMEIALDLANCGANTTMVVRSPVHILTREMFGMSTFSVAMRLTKSLPLWLVDYILVSYSRLALGDTTSLGIQRPKVGPLELKCKMGKTPVLNVGTLEKIQSGDIKVKPAVEQLMEGGARFVDGTSLEFDAIIFATGYKSTVLRWLKDKESFFGTDSLPKERACGRSWKGRNGLYVAGMCNKGVLGGTTDAQRIANHIYSTYFTGTASSAEAEA

>Aspi01Gene32088|Alsophila_spinulosa/1-437

MPAVVASRCVKQTHDVPAESQQAPEDPADKVIIVGAGPSGMATAACLKDRGIPCVILERCECIASLWKEKTYDRLKMHIDKEYCKLPLLPFPESFPTFITRTQFIEYLEEYAAKFDLQPRFNSTVQSASYDESSETWHVQTAVVGSLAEGPRRTVKHVGRWLVVATGENAELVNPNIPGMSDFKGSKAHSSQYRNGAAYTGKKVLVIGSGNSGMEIAFDLANSGARTSVVVRSPVHILPLKVFGKSTFSVAVRLMKHFPVWLVDGFLVSYSRLVLGDTSALGIQRPKVGPLELKCKMGKTPVLDVGTLARIQSGDIKVKHAVERLTEQGVRYADGTSESFDAVIFATGYKSNVLDWLKDKGKFFGKDSLPRMPCPRHWKGKNGLYVAGLCNKGILGAKTDAELIAADLCEAYLLPRPHKSNASRSPSKATITTQIPL

>Aspi01Gene30495|Alsophila_spinulosa/1-434

MRPAFIPARWTKQTHNVPAESPGEDATNRVIIIGAGPSGMATAACLKKQGVPCVILERSGCIASLWKEKTYDRLKMHTAKECCKLPFLPFPESCPTFITRNQFIEYLDKYASKFDLQPRFNETVTSASYDESSQRWHVQTEVAGAKGLVEHIGRWLVVATGENAEVVMPNIPGKSDFKGSLIHSSQYRNGADYAGKKVLVIGSGNSGMEISLDLVNSGARPSIVVRSPVHILPREVLGISTFSLAIRLMKSFPMWLVDYFLVSYSRLVLGDTAALGIQRPKVGPLKLKSKMGKTPILDVGTLARIQSGDIQVTPAVKSITETGVQYADDRSESFDDVIFATGYKSSVLHWLKENEKEPFFGRDSLPRLPFPHNWKGKNGLYVAGLCNRGILGSNRDAKLIAADVGDAYFAHPSQTHYPNTWQQEQCRQINSPLT

>Azfi_s0014.g013580|Azolla_filiculoides/1-465

MKLPPLSFLAMHANVSARVVPDSQPAEHDATDIFSGFPAFARRTQAEEAVPGMKLLNTIHSSSSPPPPSQSVIIVGAGPSGMATAACFKSLGIPFIIIERADCIASLWKERTYDRLKLHIAKEYCKLPLMPLPDSLPPFVTRQQFVHYLDEYAAKFQLKPRFNETVQSASYDEASQTWQVYTTVVGPFAEGPRRHHVKHVGRWLVVATGENAEAVLPVIPGLKDFKGSSVHSSQYKNGSSYKGKKVLVIGSGNSGMEIALDLANNGADTSIVVRSPFHILPREVFGKSTFSVAMRLMKSFPVWMVDYFLVSYSRVVLGDTAAFGIQRPKIGPLELKAKMGKTPVLDVGTLDKIRSGDVKVKPAVECLTETGVTFVKGATESFDAIIFATGYKSNVLGWLKDKGKFFGSNSMPRQPCPRNWKGENGLYVAGLCNKGVLGATTDAMLISQDLYHSHFNVRSNSRINT

>Sacu_v1.1_s0160.g023872|Salvinia_cucullata/1-387

MATAACLKKLGIPFVILERAGCIASLWKERTYDRLNLHIAKEYCKLPLMPLPDSFPVFVTRQQFVSYLEQYAAKFELKPRFDETVQSATFDESSRTWQVYTTVVGPFAEGPRRQLKHVGRWLVVATGENAEAVMPDIPGFGDFKGSYLHSSQYKNGAIYKGKKVLVVGSGNSGMEIALDLVTSGADTSIVIRSPFHILPREVFGMSTFSVGMRLLKTLPVWMVDYFLVSYSRMVLGDTAACGINRPKIGPLELKAKMGKTPVLDVGTFDKIRSGDIKLKAAVECLTETGVKFANNSFEDFDAVIFATGYKSNVLRWLKDKEKFFGVNSMPKKPCPFNWKGKNGLYVAGLSNKGILGATTDAMLISEDLYYAYFNLTDRQTDMVSVHS

>Mvestita_S17g16247-RA|Marsilea_vestita/1-404

MAPMQSRNNNVNTVDKVIIVGSGPSGMATAACLKQQNIPYIIIERSGCIGSLWKERTYDRLKMHIAKEHCSLPLMPLPDSFPTFVTRQQFIDYMEDYAAKFEIKPRFNESVQSATYDDAAKLWHVHTTNVGPFAEGPRRQFRHVGRWLVVATGENAEAVMPSFLGMRDFRGSYVHSSQYRNGSAYKGKKVLVVGSGNSGMEIALDLANCGAKTSIVVRSPFHVLPREVFGKSTFSVAMAMMKTLPVWMVDSFLVNYSRVVLGDTDSCGIIRPKVGPLELKAKMGKTPVLDVGTLDKIRAGKIKIKPAVETLTETGVKYMNGNVEEFDAIVFATGYKSNVLRWLKDKESFFGNNSMPKPGCQFNWKGDHGLYVAGLCKKGVLGATTDAMLIAQDLSEQYHGAGHN

>Mvestita_S17g16248-RA|Marsilea_vestita/1-404

MAPMQSRNNNVNTVDKVIIVGSGPSGMATAACLKQQNIPYIIIERAGCIGSLWKERTYDRLKMHIAKEHCSLPLMPLPDSFPRFVTRQQFIDYMEDYAAKFEIKPRFNESVQSATYDDAAKLWHVHTTNVGPFAEGPRRQFRHVGRWLVVATGENAEAVMPSFPGMRDFRGSYVHSSQYRNGSAYKGKKVLVVGSGNSGMEIALDLANCGAKTSIVVRSPFHVLPREVFGKSTFSVAMAMMKTLPVWMVDSFLVNYSRVVLGDTDSCGIIRPKVGPLELKAKMGKTPVLDVGTLDKIRAGKIKIKPAVEALTETGVKYMNGNVEDFDVIVFATGYKSNVLRWLKDKESFFGNNSMPKPGCQFNWKGDQGLYVAGLCKKGVLGATTDAMLIAQDLSEQYHGAGHN

>Mvestita_S17g16250-RA|Marsilea_vestita/1-419

MDSRTDNPSHGAVPIPAATDTPNKVIIVGAGPSGMATAALLKQRNIPFIMLERAGCIASLWKERTYDCLQLHIAKEFCSLPLMPLPESFPQFVRRKDFLNYLEEYAANFDLQPRFNESVQSATFDEATKLWHVHTTVIGPFAEGPRRQFRHLGRWLVVATGENAEAVLPDFPGLGEFRGSHIHSSQYRNASPYVGKRVLVVGSGNSGMEIAYDLACKGADTSVVVRSPMHILPLEVFGKSTFSVGMRLMKSLPVWLVDRFLVSYSRVVLGDTSSCGIQRPKIGPLELKFKMGKTPVLDVGTLAKIRSGDVKVKPAVECITENGVRFVNGTAENFDALIFATGYRSNVLRWLKDKESFFGSNSMPKPHSEFNWKGSNGLYVAGLCKKGVLGSTTDATLIIEDLSKAYFDQQSSSQLTSHN

>Mvestita_S15g12988-RA|Marsilea_vestita/1-537

MPGFFTSRRTHAEQGMQYSASEDMENCNNNNLNSNFNSNWNNSSYTNNELVIIVGAGPAGLATGACLRARNIPFIIIERTATIASLWKQRTYDRLKMHIAKDYCQLPLAPFPESFPTFVSRRQFIEYIDQYVNHFDLKPHFNESVISAHFDENVQNWVVETLGVADGEKRVYIGKWLVVASGENADMAIPEIPGRSDFRGSVVHSSQYKNGASFKGKKVVVVGCGNSGMEIALDLHNNGAHTSVVVRSPFHVLPLEVLGKSTFSVAMRLMKSLPVWLVDCFLVSYARVVLGDTAAVGIHRPKIGPLELKTKMGKTPVLDVGTLARMKSGDIKVKPAIECIMEDSIKYVNGSIEKCDAIIFATGYRSNVLKWLKDKEQFFGANSMPRLPCPRNWKGKNGLYVAGLCNKGILGGANDAELIAADIYAIQNNITNGVSGGDAPEGGGGEDVQQIIPTASDMLAICDKIKEIANEAGQIASNIGVGTGQIVELGVCNNDSIASNAYTDHQVLNSQQLEAVAAVASLYSDPIDNKKKPHYIR

>Azfi_s0129.g048961|Azolla_filiculoides/1-483

MPLFSNRKQQTHAELPNLFFDEEEEEEEEKNHPRVRADSILETSSELLGHDPSQNHTADNHGDSINKVIIVGAGPSGLAAAACLRARGVPLVIVERSSSIASLWKERTYDRLKMHIAKEYCRLPLVPFPDSYPRFISKKQFLEYLDGYATRFKLRPRFREMVTSAWFDKAVGAWVVETTCSCSGNGIDGEDDGKEAGNTSPASSQKKKKKEKYVGKWLVVATGENAEMVLPDIAGSDVFRGRIVHSSEYRNGSAYCGQKVLVMGAGNSGMEISLDLSNFGAKPSIIVRSPMHILPLEILGRSTFSVAQRMLKARLSLWLIDHFLVSYSRITLGDTSSLGIHRPKIGPMALKLKQGKTPVLDVGTLARIRDGHIKVVDLEVERITEDGVLYKDGQAEKFDSIVFATGYRSNVLDWLKVVEDDEPFFKTDSTPRHGNPNNWKATKGLYVAGLSKRGLLGSATDAELIAEDISNAYFNHSAATYMK

>TnS000901889t02|Gnetum_montanum/1-442

MPHLFCEQTGKAANDPLFLKNRGAVTVAAAQKGEFQQQQQQPQQILAHGPIIVGAGPSGLAVAACLKEKGVPSIILERADCIASLWQRRSYDRLRLHLPKRFCELPLHPFPKEFPEYPDKNQFIAYLESYARRFHLRPCFNECVQSASYNRALRMWRVRTVATGPDASELVKEYLGKWLVVATGENAEAIEPGFPGADRFTGNIMHACSYRNGHRFRDQNVLVVGCGNSGMEMCLDLSLYNANPSVVVRDSVHVLPRSILGLSTFGIAMCLLRWLPVRFVDRMLLLAARLLLGDTSRHGLPRPAMGPLELKNRLGRTPVLDVGTWELIRAGSIKVFPAVECLTPHGARFVDGQERDFDTVILATGYRTNVLSWLQGCDEEFAGDGYPKKPFPNGWKGENGLYTVGFTRKGMLGAAMDASRIAEDVALQWKAEEARAERSHFS

>evm.model.Chr7.2163|Isoetes_sinensis/1-428

MPPHMADHWGKSVSDFFFQEKPAAVWEPGVIIVGAGPSGLATAACLKKKGVRSVILEKAECIASLWQHRTYDRLHLHIPKQFCELPFAPFPDSFPVYPNRQQFLDYVQDYARENNICPTFNHHVKSVTHDSRRNCWRLRVLDTTKPMRPLEIEYRSQFLVVASGENAEARMPEIEGLDSFVGDLRHSSQYKNGVPYEGKKVLVVGCGNSGMEIALDLVNHNASPSIVVRSPVHVLPREIFGHSVFSVSMALKNIFPLWLIDRFLVLYSVLLLGSTEKYGLKRPKLGPLATKAKLGRTPVLDVGTLAKIKSGHIKILPAVKGLNTRGATFVDGHSEEFDAVILATGYRNNVPKWLKEEQEYFCTEGLPKCGWKIGKGGLYVAGLSGQGILGVSQDAKLIAEDISAIIESETDSLLNGGLNSVMPKEARI

>evm.model.Chr8.1874|Isoetes_sinensis/1-428

MPPHMADHWGKGVSDFFFQEKPAAVWEPGVIIVGAGPSGLATAACLKKKGVRSVILEKAECIASLWQHRTYDRLHLHIPKQFCELPFAPFPDSFPVYPNRQQFLDYVQDYAREHNICPTFNHHVKSVTHDSRRNCWRLRVLDTTKPMRPLEIEYRSQFLVVASGENAEARMPEIEGLDSFVGDLRHSSQYKNGVPYEGKKVLVVGCGNSGMEIALDLVNHNASPSIVVRSPVHVLPREIFGHSVFSVSMALKNIFPLWLIDRFLVLYSVLLLGSTEKYGLKRPKLGPLATKAKLGRTPVLDVGTLAKIKSGHIKILPAVKGLNTRGATFVDGHSEEFDAVILATGYRNNVPKWLKEEQEYFCTEGLPKCGWKIGKGGLYVAGLSGQGILGVSQDAKLIAEDISAIIESETDSLLNGGLNSVMPKEARI

>75206|Smoellendorffii_v1.0/1-449

MISDTSDSSSDSDLGGRSGSSSSSPSPHAPSPPPPPPRANSPSIVDSSSWVEGAIIVGAGPAGLAVAACLKDRGVPSIVLDKANCIASLWQQRTYDRLHLHIAKQYCELPLLSFARDVPQYPTKNQFIDYLHDYARHFEIQPLFDRCVVAATREQSGDRSLWRVETVDKRRGVREEFRSRWLVVATGENGAERIPEDLRPGLDRFQGTVLHSSQYRNGKPFKGQRVLVVGCGNSGMEIALDLLNHGAQPSIVVRSPMHILPREMFGRSTFAVAMSLMKLLPLRVTDKLLVMYATLALGNTTKYGILRPSTGPLETKAKFSKTPILDMGTFRKIRSGSIKVMPCMEKIDREGVYFGDGRYESYDSIILATGYKSMVCSWFKDDGNYFSRDGFPKSGWNCDKGLYAAGMSRQGIFGVSKDAKHISDHIYDDFSFIERKRTNSIVNFKMMMK

>Aspi01Gene11003|Alsophila_spinulosa/1-402

MMMMMSPMSETMCKESNDSQPMVRIEGALIVGAGPAGLATAVCLKHKGISSIVLEKSDRIASLWRNRTYDRLCLHLPKQFCELPYMPFPASFPKFPTRQHFLAYLDDYASTCGVLPRFNETVSHASFDASTHVWHVLSQGPAGPRTYCAPWLVVASGENAEPVFPSGLPGHHRFHGKTLHSSQYKNGVEFSGKKVLVIGCGNSGMEVSLDLANFGAKPAVSVRSPVHVLPREIYGISTFSIAMTLTKYLPLRWTDRLLLLLSRIVLGDPTRYGFIKPTYGPLELKEIVGKTPLLDVGTLTKIKDGSIQVVPAVQKFTSSGCFFKDGRYSEFDAVILATGYQSNVPSWLKVEAKCLNQACTGSWKGKDGLFFAGFSRKGLLGISMDAHKVAADISKSRNDNIV

>Aspi01Gene36448|Alsophila_spinulosa/1-417

MMGMAHMAEVMCKESNDSNGGGAIVRVEDGVIIVGAGPAGLATAACLKQKGIASVVLEKSDCIASLWRKRTYDRLCLHLPKQFCELPCMRFSSSVPKFPTRQNFVAYLEEYASKCGERPRFNETVSCASFDTSTSMWHVLSHEGARGRRDVVYCAPWLVVASGENAEPLLPQGLPGLHNFNGKSLHTSHYKNGLEFSGQRVLVIGCGNSGMEVSLDLANFGAKPAVSVRSPVHVLPREIYGISTFSIAMTLTKLLPLRWTDRLLLLLSRLVLGDPSRHGFKKPTFGPLELKETMGKTPLLDIGTLTKIKDGSIRVVPAVQKLTHSGCFFKDGTYSEFDAVILATGYQSNMPSWLKVEPNCFNQACTGHWKGKDGVFFAGFSRKGILGISMDAQRIAADISKHYNSLKDAKRKLHLHV

>Aspi01Gene38026|Alsophila_spinulosa/1-420

MSPYAIESPGKGAQLQEYRRHSVEGVVIIGAGPSGLATAACLQQQGIPFIILERADCIASLWKKHTYDRLHLHTAKHCCQLPFMPLPVSYPTFLTRNQFVDYLEGYAHKFSIKPVFNEAVDLAMYSHQTGEWLVHTRGRGHAANTIKLDPEMDMVFTQYACKCLVVATGENAEAVLPHLPGVVDFEGTIIHSSQYKNGSHFQKMKVLVVGAGNSGMEIALDLINSGATTSIVVRSPLHVLPREMFGMSTFTVGMTLCKILPIRLVDYILVAYSQFKFGDTYLYGLRRPKVGPLELKNKMGKTPILDVGTLSKIQSGEIKVRPAIECLTASGVRFVDNTTDSYDAVILATGYKSTIRSWLHDEDQFFTEKGLPRSQLENWKGKRGLYAAGLSGKGLLGATMDALQIAKDIRETYFRSHDGR

>Aspi01Gene72802|Alsophila_spinulosa/1-467

MPPYAVEAWRKAQSLQDNKQHRVEGAIIIGAGPSGLATAACLQQHGVPFVIIERADCIASLWKKNTYDRLHLHTAKHCCELPFMPLPESYPTFLTRKHFVDYLEEYARKFSIEPMFNEMVVLAKYNHDTGDWLVHTRQCGGGEESKDMVYKRYACKWLVVATGENADAVLPHLPGARDFTGTIIHSSQYKNGSHFNTKKVLVVGAGNSGMEIALDLVNCGATTSIVVRSPLHVLPREMFFGLSTFSVAMMLCKILPVRLVDCILVAYSNLRFGKTFLYGLRRPKVGPLELKNKMGKTPILDVGTLSKIQSGDIKVRPAIECLNVSGVRFVDSTTDSYDAIILATGYKSSIPSWLHDEDEFFTEQGLSRSPLENWKGKRGLYAAGLSGKGLLQATMDARQIAKDISQAYSPQGKLNQFGSTTAIWLGFTKPAPCMLSAAVVIVNYIFLAHLFYCCLHMIRGARLSMRN

>Mvestita_S7g18505-RA|Marsilea_vestita/1-535

MPPHVEAMERSWEQRSWRDPHDDQHLSHHDEFKHEEKQSNGDHDRHKYEEEQSNSDHDRHKHEEKQSISDHIGDGHGLFLYTSTDFHLLYTEQHQQIEYHRVNGVLIIGAGPSGLATAACLERHGVEYVILERAECIASLWKKHTYDRLHLHTAKNSCQLPFFPMPESYPTFITRQQFVEYLESYAARLKHKPVFKQSVVMAKFSEEAGGWIVEAVSMDNNIDSETNVVYGNENGNEACVKDIANSESAESADEACDTNVNKIVKKKKKKVVYVCKWLVVATGENAEAVVPQLPGVEDFKGPVLHSREYKNGNHFDQKKVLVVGAGNSGMEIALDLADCGAKTSIVVRSPLHVLPREMFGYSTFSVAMALCKVFPVRVVDRFLVAYSQIKFGDTFTYGLKRPKIGPLELKNKMGKTPILDVGTLAKIQSGHIQVKPAIESLTSTEVRFVDNSIDKYDAIILATGYKSAIPTWLHDEDKFFSDQGLPRSPLQNWKGEKGLYAAGLAGKGVLGATMDAQQIAKHISEAYVSIQRSIL

>Azfi_s0084.g039040|Azolla_filiculoides/1-425

MTNVRLGEVICNKNTMRIDHEVEGAIIIGAGPSGLATALSLQQQGVPYVILERAECVAPLWKKNTYDRLRLHTPKQCCHLPFLPFPSSHPTFLTRLHFLQYLHRYSRLLLNPPLFNHTVVLTSYSTSDPLQPRWLVHTRSSIANNLDDINHSKYCNDSCVIFSCRWLVVASGENSEAFWPNDILSLGLLDRFQGRVMHSCHYKNGKCFINKKVLVIGAGNSGMEIALDLVDYGATSTTIVVRSPVHVLPRQMFGRSTFTMGMTLSKIFPLRFVDRLLIAYSHIKFGDTSKYGLRRPKIGPLELKNKMGKTPILDVGTFSKIQKGDIKVKPTIESISESRVYFVDNTVDNYDAIILATGYKSTIMKWLHLDENTFFNEQGLSRPENSWKGNKGLYAVGLSGKGLLGATSDALNIAQDIKGAYFSFK

>LOC_Os01g12490|Osativa_v7.0/1-439

MDCFAETEGKRAHDPLYQRRAAAAATPATGVPVDDVDKVVDVPGAVIVGAGPAGVAVGALLGLRGVAYVVLERCGCIASLWRHRTYDRLCLHLPKRFCELPLRPFPASFPEYPTRDQFLGYLDAYAREFGVEPVFRRAVISAEYDGESWWVYTREVVAAAAGGEQAVLGCTMTVYRSRWLVVATGENAEPVVPEMDGAGRFKGQMMHSSEYRNGDGYAGKKVLVVGCGNSGMEVSLDLCNHNARASMVVRDTVHVLPREILGFSTFGLSMWLLRWLSVQTVDWLVLLLSFLVFGDTARLGIPRPSLGPFELKSVSGKTPVLDVGTLAKIKSGDIKVTPAIQCFQEHGVEFVDGSTEEFDVVILATGYKSNVPYWLKEKEFFSEKDGFPRKGNAWKGQNGLYAVGFSRRGLSGVSMDANNIVQDIVQRLHDMGYERSENN

>Bradi2g07407|Bdistachyon_v3.2/1-446

MDCFAEAEGKRSHDPLYCNPPAALATGFPIEDVKFEKVAGPLIVGAGPAGLATAALLGQQNVPYVLLERCSCIGSLWRHRTYDRLCLHLPKNFCELPLMPFPSSFPTYPTREQFLDYLESYAKRFDVQPVFRQAVVSAEFNGEFWWVRTKQVISAAIGGQQAVLGSAITMYRCKWLVVATGENAEPRVPEIEGAKRFKGQIMHSSEYRSGEGYAGKRVLVVGCGNSGMEVSLDLSNHNAHAAMVVRDTVNVLPREILGHSTFGISAWLLKWLPIKTVDMILLTMSRFVMGDITRLGIARPSLGPMELKSVSGKTPVLDVGTIAKIWSGNIQVFPAIQCFKEHGVEFIDGRIKNFDVVILATGYKSNVPYWLKEKQFFSEKDGFPRNSKEWKGKNGLYAVGFSRRGLTGVAMDATQISDDITKNWRDMHTRDVREDPSSRSGTVIFN

>Zm00001d041402|Zmays_RefGen_V4/1-455

MDHHFTEIEGKMAHEPLAHSSTFSDETKDKMEHDSLVYSPRSIARATTTGIPVGDQGVAVLRGPLIVGAGPAGLACAALLTMLYIPYVLLERDVCVASMWHRRTYRRLCLHLPKRYCELPLMHFPRTYPTYPTRQQFLVYLNEYMRAFGIRPFFSLEVVAAEYNGEYWCVRTKDVIPTGTLNGQSGEESIREYRSKWLIVATGENAEPVVPDMPGIHNFKGQVMHSCDYRSGESFQGKKVLVVGCGNSGMEVSLDLANHNVHTSMAVRRSGHVLPREVMGISTFSLSVWLLKFLRVQIVDRILLLLAWFILGDTAQIGIPRPSMGPMELKQVSGKTPVLDVGTIAQIKSGNIKVFPAIRSFQEYGVEFTNGRIESFDVVILATGYKSNVPYWLKENEFFSQQSGFPNKPNEWKGKDGLYAAGFSRRGLLGVSMDATKIAEDIAQSYNNIYKLQRS

>Sobic.008G154300|Sbicolor_v5.1/1-451

MDRFTETEGKMAHDPLSYAPSFAEETEGKMEQDPLMYSPRSAARATATGIPVVGDEGVAVLPGPLIVGAGPAGLACAARLTMGSVPYALLERDVCVASMWHRRTYRRLCLHLPKRYCELPLMPFPRSYPTYPTRKQFLAYIDEYMRNFGIRPFFRQEVIAAEHDGEYWCVRTKDSITGPINGGGEESIVSSTREYRSKWLIVATGENAEPVVPEIEGMHSFKGQLMHSSHYRSGEAFQGKKVLVVGCGNSGMEVSLDLANHNVHTSMVVRDSGHVLPREIMGLSTFTLSVWLLMFLHVQIVDRILLLLAWFVLGNTARLGIPRPSRGPMELKQVCGKTPVLDVGTIAKIKSGDIKVFPAIQSFQEHGVEFIDGRIESFDVVILATGYKSNVPFWLKENEFFSQKDGFPRRRNEWKGKNGLYAVGFSRRGLLGVSMDATKIADDIVQSYNNI

>Zm00001d039323|Zmays_RefGen_V4/1-432

MRPAEIMDRRRFAETEGKRAHDPLYSPRRAARATATGFPVGEHGEVLAGPVIVGAGPAGLAVAACLTMRGVPYVLLERHGCVASLWRHRTYRRLRLHLPKRFCELPLMPFPPSYPAYPTREQFLAYLEDYIATFGIRPFFRQAVVSAEHDGDFWCVRTLDGGGTSREYRSKWLVVATGENAEPVVPDIDGIHAFRGLVIHSSDYRSGESYRGKKVLVIGCGNSGMEVSLDLSNHNVHTSMVVRDSVHVLPREIMGVSTFGLSLWLLTHLSIQKVDQILLVLTQLVLGDTARLGIPRPNIGPMELKRVSGKTPVLDVGTIAKIKSGDIKVYPAIQSFQEHGVQFIDGKSESFDVVILATGYKSNVPYWLKDKDLFSEKNGFPHNSNEWKGKNGLYAAGFSRRGLLGVSMDATNIADDILGCWNDFGYERHKRK

>Sobic.003G012400|Sbicolor_v5.1/1-426

MDRFAETEGKRAHDPLYSPRRAARATATGFPVGEHGEVLAGPVIVGAGPAGLAVAACLTMWGIPYVLLERHGGIASLWRHRTYRRLRLHLPKRYCELPLMPFPPSYPAYPTREQFLAYLEDYIATFGIRPFFCQAVVSAEHDGDFWCVRAVDGGSGGVTRVYRSKWLVVATGENAEPVVPDIDGINAFRGLVMHSSDYCSGEGYRGKKVLVVGCGNSGMEVSLDLSNHNVHTSMVVRDSVHVLPREIMGFSTFGLSMWLLMCLSVQTVDQVLLLLTQLVLGDTSRLGIPRPSIGPMEQKKVSGKTPVLDVGTIAKIKSGDIKVFPAIQSFQQHGVQFIDGKTESFDVVILATGYKSNVPYWLKEKDFFSEKNGFPRNSNEWKGKNGLYAAGFSRRGLFGVSMDATNIADDIVRCWNDFGYERHKRK

>ELECO.r07.1AG0001270|Ecoracana_v1.1/1-434

MDRFAETEGKRAHDPLYRPRRATTTGFPVGDEYVDIPGPVIVGAGPAGLAVAACLAVKGVKYALLERYDCIASLWRHRTYHRLKLHLPKRFCELPLMPFPESFPTYPTREQFLEYLEDYALAYNIHPMCRQNVVGAEHDGEFWIVRTKEVITAAIDDEEEAVLSSTTRVYRSRWLVVATGENAEPVLPEIDGIETFKGHVMHSSDYRNGEGYEGKKVLVVGCGNSGMEVSLDLSNHNVHTSMVVRDAVHVLPREIIGYSTFGLSAWLLRWLPIQVVDRIVLLLARLVLGDTSCLGIPRPSIGPMELKKVCGKTPVLDVGTIDKIKSGDIQVFPGIQCFHEHGVEFIDGRIVNFDAVILATGYKSNVPYWLKDKGFFSEKDGFPRKPNEWKGKNGLYAVGFSRRGLLGVSMDATKIADDIVQCWHDMGYGRQKSK

>ELECO.r07.1BG0049720|Ecoracana_v1.1/1-436

MDRFAETEGKRAHDPLYRPRRATTTGFPVGDDYVDIPGPVIVGAGPAGLAVAACLAVKGVKYALLERYDCIASLWRHRTYHRLKLHLPKRFCELPLMPFPESFPTYPTREQFLEYLEDYALAYNIHPMCRQNVVSAEHDGEFWIVRTKEVIVIDGGEEEEEAVLSSTASRVYRSRWLVVATGENAEPVLPEIDGIETFKGHVMHSSDYRNGEGYEGKKVLVVGCGNSGMEVSLDLSNHNVHTSMVVRDAVHVLPREIIGYSTFGLSAWLLRWLPIQVVDRIVLLLARLVLGDTSCLGIPRPSIGPMELKKVCGKTPVLDVGTIDKIKSGDIQVFPGIQCFHEHGVEFIDGRIVNFDAIILATGYKSNVPYWLKDKGFFSEKDGFPRKPNEWKGKNGLYAVGFSRRGLLGVSMDATKIADDIVQCWLDMGYGRQKSK

>Adc02843|Adiantum_capillus-veneris/1-448

MCRMREPRVSPHSCHPFVDATLRLHPQEGAEQAVPSHFGEMFGKASNDPQLNCSYTPACEPLSMHWVEGAIIIGAGPAGLATAACLRKLGVPSLVLERENCIGSLWRYRTYDRLRLHIAKQFCELPYMPFPLSFPTFPTRAQFLEYLDDYARAFDIQPQFNESVQEATYCAGAGLWKVESKGPQGERTFLARWLVAATGENAQPVWPDVQGLSSFNGKLCHSSDYKSGEEFSGKRVLVVGGGNSGLELALDLANHEADVYMSVRSQVHILPREIFGFSTFAVAMTLLKFFSLQWTDRLLLLMSRMILGETSELGFPRPKVGPMEMKCKTGKTPSLDAGTLSKIREGCIEVLPEVLNFDANGCSFMESLSHMDFDAIILATGFKSNVPSWLKENELFSSEGFPKAGSPRGKNGLYSAGFARKGLLGVSMDAQSVAEDISKLYYRAARML

>Aspi01Gene11463|Alsophila_spinulosa/1-433

MSESRALLAPHVRPRTLDEEEESVPSNFVQLFGKASIDQCELSYRSINWVEGAIIVGAGPAGLATAACLQRRGVPSQVLEREDCIGSLWRYRTYDRLRLHIAKQFCELPYLPFPSSFPTFPTRAQFLEYLDEYARTFNIKPQFSECVVEATYSAGTGLWQVESRGHKGLRTFVGRWLIAATGENAQPVWPNSPGMDSFRGKVCHTSEYKSGEEFRGKKVLVIGGGNSGLEVALDLTDHQAQAFLSVRSEVHVLPREIFGFSTFSVAMTLLKFFSLRWTDRLLLLMSRLILGETSELGLPRPRLGPMELKSKTGKTPSLDSGTMSKIREGCIKVLAGVQEFTSNGCTFTDLNYQDFDAVVLATGFKSNVPSWLKENELFSSEGFPNPSLPCSWKGKNGLYSAGFAKKGLLGVSMDAQRIAEDISNFCISPRLLC

>Aspi01Gene19346|Alsophila_spinulosa/1-440

MCVISEPRALIAPPVRARTLEGEEDAVPSNFVELFGKASNDHFELSRPSINWVEGPIIVGAGPAGLATAACLQRRGIPSLVLEREDCIGSLWRYRTYDRLRLHIAKQFCELPYLPFPSSFPTFPTRAQFLEYLDEYARTFSIKPQFSECVVEATYCAGTGLWQVESRGPKGLRTFVGRWLVAATGENAQPVWPNMPGMDSFRGKVCHSSEYKCGEEFRGKKVLVIGGGNSGLEVALDLVNHQADAFLSVRSEVHVLPREILGFSTFSVAMTLLKFFSLRWTDRLLLLMSRLIMGETSNLGIQRPKLGPMEMKSKTGKTPSLDAGTMSKIREGCIEVLAGVQEFNSNGCTFTDLNHQDFDAVVLATGFKSNVPSWLKDNEDFSSEGFPNSNLPCSWKGKNGLYSAGFARKGLLGVSKDALSIAEDISKFHSSPRLQYRFDT

>Aspi01Gene53362|Alsophila_spinulosa/1-439

MCKTSDPRVPLAPHACARALEGVDEAVPSSPFVDLFGKASNDHDSHLSNLPYINWVEGAIIVGAGPAGLATAACLQRRGVPSLVLEREDCIASLWRYRTYNRLRLHIAKQFCELPYLPFPSSFPTFPTRAQFLDYLDEYVRTFDIRPQFNEYVVESTYCAGTGLWQLESRGPKGLRTFVGRWLIAATGENAQPVWPNVPGLDTFRGKVCHTRDFKSGEEFNGKKVLVVGGGNSGLEVALDLVNHQAQVFLSVRSQVHVLPREILGFSTFSVAMTLMKIFSLRWTDRLLLLMSRLILGDTSQLGFPRPKVGPMEMKSKTGKTPSLDAGTLSKIREGRIEVLSDVQEFNSNGCTFTDLTYQNVDAVILATGFKSNVPSWLKDNELFSSEGFPNPSSPSNWQGKNGLYSAGFARKGLLGVSMDAQRIAEDISKFYNPPRLRC

>Ceric.04G052300.2.v2.1|Ceratopteris_richardii/1-168

MTLLKYLSLQWTDRVLLLLSRIILGETSQLGFRRPNLGPMEMKSKEGKTPCLDAGTLSKIREGCIEVVSEVESFDSTGCYYMDGTPRMEFDAVILATGFKSNVPKWLKEGDLFSSEGFPKPGAPCNWKGRNGLYSAGFARKGLLGVSMDALNIAKDIASLYIRQNTML

>Ceric.04G052300.1.v2.1|Ceratopteris_richardii/1-452

MCRMPEPRNTATRSQPRVDDAEVRIRKHEWEEAVSTHFVELFGKSSNDMLLRSDHQSLPREPFSINWVEGVIIVGAGPAGLATAACLSKQGVPSLILEREDCIGSLWRYRTYDRLRLHIAKNFCELPFKPFPASFPTFPTRAQFLEYLDEYAREFDIRPQFNECVKEATYCPGTGLWRVESRGPLGSRTFIGRWLVVATGENAEPSWPDVPGMETFNGSLLHSCDYKSGAEFAGKRVLVVGGGNSGLEVSLDLANHQAAVFLSVRGEVHVLPKEMLGFSTFSIAMTLLKYLSLQWTDRVLLLLSRIILGETSQLGFRRPNLGPMEMKSKEGKTPCLDAGTLSKIREGCIEVVSEVESFDSTGCYYMDGTPRMEFDAVILATGFKSNVPKWLKEGDLFSSEGFPKPGAPCNWKGRNGLYSAGFARKGLLGVSMDALNIAKDIASLYIRQNTML

>Azfi_s0487.g073448|Azolla_filiculoides/1-177

MLGFSTFSVAMALLKFFSLRWTDRLLLGMSRVLIGETTKLGFPRPRLGPMEIKSTTGKTPSLDAGTLSKIRQGHIKVLCGIQEFSSNGCTFNDSCHEDIDAVIFATGFKSNVPSWLKDNELFSKDGLPHPNLQCNWKGKNGLYSAGFGRKGLVGTSMDAQKIAMDINKIYRSGPKAS

>Mvestita_S8g04883-RA|Marsilea_vestita/1-474

MCEMSYPPVGTSDQEAETLKPNSINTAKIISMAASPANYKISEFVHLFPKLQPFNNENITANVTRPILMMKQPTSPICWVEGAIIVGAGPAGLATAVCLQKKGIPCLILEKEDCIGSLWKYRTYDRLRLHIAKQFCELPYMSFPAHYPKFPTKTQFIEYLDNYAKTFNIQPHFKETVLEASYTSCGLWQVESQGPKGHRTFLARWLIAATGENALPAWPKFPGLDTFQGKICHSIQYKSGDEFQGQNVLVVGGGNSGLEVALDLVDHQANPVLSVRSPVHILPREMLGISTYSVATNLLKLFSLKWTDRLLLFMSNLILGSTADLGFKRPKNLGPMEMKARTGKTPSLDAGTLSKIREGHIKVFDNIKQFSSNGCTFEDNSYQSFDAVIFATGFKSNVPTWLKDSELFSSNGFPHPNSEFNWKGKNGLYSAGFAKKGLLGMSMDAHRIADDISKLYCPRNLVVAAAKESTMTAF

>Mvestita_S1g07006-RA|Marsilea_vestita/1-419

MCKMDTNRTEPQPGHKLHLQLNNLQNPHNPSIHWVDGSIIVGAGPAGLATSACLKLRGIPHVLLEKEDCIASLWRYRTYNRLQLHLPKHYCELPHMSFPPSVRTFPTRDQFLQYIDDYATTFNIKPLFKESVMDASYCPVTRLWQVECHGPRGITTYVSRWLVVATGENAQPIWPNVAGLTSFKGSVCHSSEYKSGEYFCGKRVLVVGSGNSGFEVARDLVNHHAEVYLSIRDRVHVLPKQMLGFSTFSVAMTLMKLFSLSWTDRLLLLLSRFFLGDTRRLGFERPQDGPMALKFKTGRTPSLDAGTISKIRKGCIKIVPEVKEFSSTGCLFKDDKQQSLDALILATGFKSNVPYWLRDNELFTATGMPNTKLACTWKGKNGLYSAGFGMKGLLGISQDAEMIAEDISKSYMSATLLMK

>MA_46423g0010|Picea_abies/1-415

MFPFSSDNWEAGDTNVECGPIIVGGGPSGLATAACMKQKGIPSLILEKSDCIASLWRYRTYNRIHLHLPKRFCELPLLPMGPNYPDSLTSAQYIDYLESYARQFQLCPRFNEHVMSASHDDSTGLWKVRTVNRERGNFRVIETFSTRWLVVASGENAEPQVPRIQDVRKFKGIVMHSCSYKNGATFKDSNVLVVGCGNSGMEIALDLAYFGANPSIVLRGYTHILPAKTLSVHTYTLISMFMKVFSVWMVDMLLLLGAWWELGSTSKYGLNRYDEGPVSIKNRLRKTPVLDPGTYRLIKNGDIKVLPAVARFTEKGAEFTDGHSEDFDAVVFATGYRSNVRDWLCSEVDFFTPEGIPKKSWKGRKGLYTVGLSGAGIPGVWVESQRIAQDIEQHLRSDHNFVTEDARSSIKNCSQ

>PITA_24868|Pinus_taeda/1-423

MFPFSNDNWEVGDINVVECGPIIVGGGPSGLATAACLKQKGIPSLILEKSDCIGSLWRYRTYDRIHLHLPKRFCELPLLPVDRNYPDSLTSLHYVHYLETYARRFQLCPRFNEHVISASHDESTGWWKVCTVNRERGNFRKIQTYSARWLVVASGENAEPQVPCIEDVRKFKGIVLHSCSYKNGATFKDANVLIVGCGNSGMEIALDLAYFGAKPSIVLRGHKHILPAKMLCVHTYTIISTLLKVFSVWMVDMLLLLGAWWVLGNTDKYGLKRHTEGPLSIKNRLRKTPVLDTGAYRLIKNGDIKVLPGVARFTEKGAEFTDGCSEDFDAVVFATGYRINVREWLFSEVDLFTPEGIPKSPSPESWKGRKGLYMVGLSGAGIPGVWIESQRIAHDIEQHLRSEHTFDSSFMKDTHSSKKNCSH

>Adc07144|Adiantum_capillus-veneris/1-405

MPPFSRLELWDEEPRNSLCFASGPIIIGAGPSGLAVAACLRMFKIPSLILEREDCIASLWKYNTYDRIHLHIPKQYCDLPFRPIPKHYPTYLSGAQFLDYLNDYASWFNIKPRFNERVMSAKFDERSQLWRVLTMGTSKANREEVREYVGHWLIVASGENAEEVKPNIENIHNFKGKICHSRSYKNGRDYAGKKVCVVGCGNSGMEIALDLLKSNAIPSIVVRNKVHVLPQEILGKATFAVIQGLLRIAPLWLADRILLFCAWCKWKNTAKWGLPRPKEGPIAYKIHTRRTPVLDIGTLCLVKDGSIEVHPAIESFTSHGANFCDGHYKEYDAIILATGYRSNVKDWLIGEDDQFDSHGLPKSLPVDGLKGKNGIYVVGMSGRGLLGSKLEAQQIAQHIKTQFGY

>Ceric.18G084200.1.v2.1|Ceratopteris_richardii/1-405

MPPFVRLELWEEEPRNSLCFASGPIIIGAGPSGLAVAACLRKHRIPCLILEREDCIAPLWQHNTYDRIHLHLPKQFCELPFMPIPDKYPTYLSGAQFLEYLHQYAKTFNIKPRFNERVTSAKFEEKTQLWRIFTRGTSKGNNDEVREYVGHWLVIASGENSEEIKPAIKNLESFKGKICHSKNYKNGKEYEGKKVCVIGCGNSGMEIALDLHKSNATPSIVVRNKVHVLPQEIFGKSTFGVIQLFLKFLPLWIIDCILLFYAWCKWRSTERYGLARPKRGPLAYKMQTRRTPVLDIGTIGLIKSGTIQVRPSIDFVTPHGVQFSDGQYKEFDAIILATGYRSNVKDWLLAEDDQFTSGGLPQSARQQTWKGRRGLYIVGMSGRGLLGTKIEAEQIGQHIKNEFGY

>Aspi01Gene10607|Alsophila_spinulosa/1-446

MGMQELWDEEPRNSLCFASGPIIIGAGPSGLAVAACLRRFKIPSLILERSDCIASLWKHNTYDRIHLHLPKNFCELPFSPIPHHYPTYLSRAQFLEYLDNYAKEFNIKPRFNERVTSASFEDKSGLWRILTLGTSKDTKNQVREYVGHWLIVASGENAEAVKPEINNLQKFRGSICHSCMYKNASEYEGKKVLVVGCGNSGMEIALDLIKSNAKPSIVVRNSVHVLPQEILGESTFAIILALLKVFPVWFADHLLLFFAWCKWKNTANCGLPRPKEGPIAYKNRMRRTPVLDIGTLCLVKSGRIKVYPAVETFTTNGAQFCNGQTEQFDAVILATGYRSNVNDWMLGEGDQFTRDGLPNALCIGGWKGKKGLYVVGMSGRGIVGTKSDAQLIAKDIQLEFHHWARMINSHVTNFHIPFALDGDDENDDDENDDDDNENDDNDDDDD

>Aspi01Gene36218|Alsophila_spinulosa/1-404

MSPLMRYELWDEEPLNSLCFASGPIIVGAGPSGLAVAACLRKFKIPSLILERSDCIASLWRHNTYERIHLHLPKKYCELPFLPIPQHYPTYLSGAQFLEYLENYGKEFNIKPRFNERVTSARFDERSGVWRVVTSGTSKDRKDEVREYAGHWLIVASGENAEAVKPKINNLQKFRGSICHSYMYKNAVEYEGKKVLVIGCGNSGMDIALDLFKSNAKPSIVVRNSVHVLPQEILGASTFAIILTLLKVFPVWFADLLLLFCAWCKWKNTATCGLPRPKEGPIAYKNRMRRTPVLDIGTLRLVKSGRIKVYPTIKTFTPKGAQFFNGHTEQFDAVIFATGYRSNVKDWLIGEDNQFTIDGLPNGPCTGGWKGKKGLYVVGMSGRGLLGTKSDAQLIAQDIQLEFI

>Mvestita_S6g00794-RA|Marsilea_vestita/1-402

MAKDALLWDEEPKNSLFIACGPIIIGAGPSGLAVAACLRHLGISSLILERADCIASLWKHKTYDRIHLHLPKKFCSLPYLDIPSEMPNYLSGVQFLEYLDAYATAFHLKPRFNEEVQSAHFDERHGLWRVLTMGNGPNNLNVVREYASHWLIIASGENAEAVVPNFPNLHKFHGPVIHSRDYKNATPFQNKKVLVVGCGNSGMEIALDLIKSDAAPSLVLRNSVHVLPQEIMGESTFAIILRLLKIFPVWIADQILLFSAWCKWKNTAKCGLPRPKEGPIAYKNKKRRTPVLDIGTLSFIKSGQIKVYPTVKSFTPIGAEFTNGEKGQFDVVILATGYRSNVKDWLTGEDDQFTTDGLPSTPCLGGWKGKKGLYVVGMSGKGLVGTKSDALSIAQDIQLEFK

>Nycol.A01223|Ncolorata_v1.2b/1-453

MARCIYYFGESHSEKISDFIVFTFFGTFLFLILNSCQLSTSATTLRQRLRVYVNGPIIVGAGPSGLATAACLQLLHLPFLLLEKESCIASLWHQRTYDRLKLHLPKAFCSLPFMPFPAHFPDYPTRDQFLAYLDAYCRHFHLRPRFGQCVESATFDHVTGLWHVSIMGAEFEYLCRWVVVATGENAVPDLPELPGREAFKGNVVHSVCYKNGEGLRGKEVLVVGCGNSGMEVCLDLCEHGAKPFMVVRNGVHVLPRDMFGVSTFRVAMALSKWLPLKMVDMILVFLAERMFGCTQECGLRRPEIGPLELKRKTGKTPVLDVGAIPMIKSRKIKIMAAIDTLTTNGAKFVDGKELEFHSIILATGYKSDFSSWLKDCNSFSEDEDEKIQTIPCNWKGQNGLYFVGFAGKGLQGIRLDAERTTDNIRRQWTDLPENKHFMVQVRSNHNYKKCVIL

>Tle1c07373.1|Takakia_lepidozioides/1-414

MEVPLEDTVVGSLPTFSQVWLPGALIVGAGPSGLATAACLHSHGVPCTILEREDCVASLWKKRTYDRLHLHLPKKCCELPLYAFPEDYPTYPTRDQFVHYLDQYASRFHINPIFNKVVESAEFLDHCSMWSVSTSDAVASGDVGSVYMARWLVVASGENAEIAMPTMKGQDDFVGEIIHGSSYRNGEGYQNLNVLVVGCGNTGMELALDLANYKARVSLVVRHPHHVLPRDLFGLGISTFEVISFLKKRFPLWFVDFLIVLASWLTIGNTAKFGIKRPNEGPLELKLKTGKTPVLDVGTVQKVKSGQVKVVPGIDRVTKEGVHFVDGTYKNFDAIIMATGYTSNVHLWLKDDNVFSTNGFLKHPPSNGWKGDRGLYAAGFGGGGLPGCSHDAVHVAEDISKAFKLLTVQKLEET

>evm.TU.supercontig_26.77|Cpapaya_ASGPBv0.4/1-385

MADEMVRDIEETVVLIVGAGPAGLATSACLNKLHIPNVLLDREDCYASLWKKRAYDRLKLHLAKEFCHLPHMHFPYCTPTYVPKDDFVRYLDKYVKHFGVSARLLRSLEYADYDEKRGLWNVLVNNMVSNVQEVYKAQFLVVATGENDEPLIPEVAGLESFGGEYMHSSRYVNGERFAGKHVLVVGSGNSGMEIAYDLSNWGAVTSISIRSPVHILSKDIVHMGMVLLKFLPCKLVDKICLASSKVKYGSLSPYGLRRPGKGPFYIRAAGGRSPTIDVGCLQKIKTGEIDVLPSIKRVEGNRVEFMNEENLLFFDAIVFATGYKSAVKSWLKNDGDLFGENGMPKRMFPNHWKGENGLYCAGFSRRGLSGISRDARDCQRYFKPE

>Brara.F01537|BrapaFPsc_v1.3/1-378

MENEMIKTQALIIGAGPAGLATSACLNRLNIPNIVVEREECSASLWKRRSYDCLKLHLAKHYCQLPHMPFPPNTPTFVSKSGFITYLDEYATRFNVSPRYNRNVESAYLNDGRWIVEVGKGAEREVYSAEYLVVATGENSEGVIPEIPGLVESFQGEYLHSSEYKNGEKFAGKRVLVVGSGNSGMEIAYDVSKWDADVSLVVHSPVHVLTREIVRIGMWLLRFFPVKLVDRWCLLLAKLRFGDTSRYGLIRPTKGPFMNKIVTGRSPTIDVGCVDEIKAGKIEVVPSIKRIEGKRVVFVDGNTKRVDSIVFATGYKSSVSKWLQVDDGDLFKENGMPKRELPDHWKGENGLYSVGFGRQGLAGITRDAQNVARDIASL

>AT1G21430|Athaliana_Araport11/1-391

MEKEIKILVLIIGAGPAGLATSACLNRLNIPNIVVERDVCSASLWKRRSYDRLKLHLAKQFCQLPHMPFPSNTPTFVSKLGFINYLDEYATRFNVNPRYNRNVKSAYFKDGQWIVKVVNKTTALIEVYSAKFMVAATGENGEGVIPEIPGLVESFQGKYLHSSEYKNGEKFAGKDVLVVGCGNSGMEIAYDLSKCNANVSIVVRSQVHVLTRCIVRIGMSLLRFFPVKLVDRLCLLLAELRFRNTSRYGLVRPNNGPFLNKLITGRSATIDVGCVGEIKSGKIQVVTSIKRIEGKTVEFIDGNTKNVDSIVFATGYKSSVSKWLEVDDGDLFNENGMPKREFPDHWKGKNGLYSAGFGKQGLAGISRDARNIARDIDSLVCGRSSKNKLSK

>evm.TU.supercontig_9.164|Cpapaya_ASGPBv0.4/1-373

MVIIVGAGPSGLAMAACLTLQTVPYVILEREDCFASLWQKYSYDRVHLHLPKSFCQLPHMEFPATYPTYLPKSLFLQYLKDYVSHFKIQPAYRRSVEEAGYDESMQKWVVKARNLGTGEVEEYFCRFLVVASGETTNAHTPEVEGLTSFFGEVIHSTQFKNGKGYRDQNVLVVGSGNSGMEIALELAEQGAKTSIAIRSPVHILSRGMAYVGLKMLKYISANMVDLIMVVMSKMVYGDLSKYGIKRPKEGPFFMKVAYGKYPVLDVGTIEKIKSRHIQVLPGMESIRDKEVVFENGESHYFDTIVFCTGFKRSTHMWLKGDDYMLDEDGVTKQSYPNHWKGKNGLYCVGLARKGLYGAAVDAERIAIDIKSLL

>Prupe.8G252500|Ppersica_v2.1/1-384

MEQEKAVIIVGAGPSGLAMAGCLSRLAIPYIVLEREDCFASMWKKYSYDRLHLHLQKQFCELPHMPFPPSCPTYVPKNQFIQYLDDYVSRFKISPILYHRNVESANYDEASERWVVKAVTDSNTGGSGEMEEYFGRFLVVATGEATNPYTPEVQGLNTFAGDVLHSTQFKSGKGFRNKNVLVVGSGNSGMEIALDLANHGAKTSIIVRSPVHVLSRGMVYLALVLLKHFSLNMVDSLMVLLSKLVFGDLTKYGIERPTEGPFYMKVKYGKYPAIDVGTFKKIKSSEIQVLPAEISSIRGSDVELKNGKSYQFDAIVFCTGFKRSTNLWLKGDDYLLKDDGLPRQSFPNHWKGKNGLYCVGLSRRGIYGSSEDAQNIANDIKSFL

>FvH4_2g24750|Fvesca_v4.0.a2/1-381

MPEAAVIIVGAGPSGLAVAGCLSRLEIPYLLLEREDCFASLWKKYSYDRLHLHLQKQFCELPHMSFPSSCPTYVPKKQFIQYLDDYVAHFKISPMYQRNVESATYDEGSERWVVKAMNNDEGCGGEVEVFLGRFLVVATGEATNPYVPEIEGLSSFDGEVLHSTRFKSGVEFKNKNVLVVGSGNSGMEIALDLANHGARTSIIVRSPVHFLSKRMVYLALVLLRYLSLSKVDTLMVLLSKLVYGDLAKYGIARPKEGPFFMKIKYGKYPAIDVGTCSKIKSGEIQVLPTEIGSIRGNDVKLKNGKSYQFDSIVFCTGFKRSTHLWLKGDDYLLKEDGLPRPSFPNHWKGKNGLFCVGLSRRGLYGSSEDAQNIANDIQSSL

>Aqcoe2G315400|Acoerulea_v3.1/1-377

MKEEMVIIVGAGPSGIATSACLNKLSIPNIVIEREDCFASLWKEKSYDRLHLHLAKQFCQLPHMSFPNESPTYVSRKQFIQYLDDYVSQFNVKPLYNRSVESATYDEVDGKWYVKAKNIHSNEIEEYMAKFLVVASGETSDVYIPEVEGLDSFKGDVFHSTKYKTGESFSNKNVLVVGCGNSGMEIALDLANFGAKTSIVVRNPLHILNREMVYMALVLVKYLPLHMVDGAVVLLSKFIYGDISKYGLNRPKEGPFFLKVKYGKYPVIDVGTFEKIKSGEIQVLPSIISIRGDSVEFANGKTHHFDTIIFATGFKRSTSKWLKDDNCLLDEDGIPKPSFPNHWKGQNGLYCVGLSRRGLYGAAMDAENIANDIKALL

>BUSI_019352-RA|Buxus_sinica/1-376

MEEMVIVIGAGPSGLATSACLNLLSIPNIPLEREDCFASLWKKKSYDRLHLHLAKQFCELPHMSFPSGSPTYVSKKQFIQYLDDYASHFKVSPLYQRSVESTSYDEGTKKWSVMVRNTNSNKIEEYIARFLIVATGETSDPFVPEIEGLDTFRGEVLHSTQYKNGEKYKNKSVLVVGGGNSGMEIALDLSNFGAKTSIVVRNPVHTLSREMVYLGLVLLKYLPPQMVDSMLAMLSKLMYGDMTKFGLSRPEEGPFYMKVKYGKYPVIDVGTIKKIKSGEIQVLPSITSIRGDEVLFTNGAVHHFDVIVFATGFKRSTSKWLKDDGHLLGEDGIPKSSFPNHWKGNNGLYCAGLARRGLYGAAMDAQNIADDIKSLL

>TESI_026094-RA|Tetracentron_sinense/1-358

MKEEVVIIVGSGPSGLATSACLNLLSIPNIVLEREDCFASLWKKKSYDRLHLHLPKQFCELPHMSLPIASPTYVPKKQFIQYLDDYASQFKVSPMYCRSVESATYDEVTKKWCVKARNTTSDEVEEYMARFLVVASGETSDAFVPKVEGLDTFSGEVLHSTQYISGEKFKSKNVLVVGCGNSGMEIALDLSNFGAKTSIVVRGPLHMLSREMVYMGLVLLKYLPYYMVDSLLIMLSKLVYGDMAKYGIPRPQEGPFSLKVRCGKYPVVDVGTYKKIKSGEIQVLPSIISIRGDDVLFANGRSDPFDVIVFATGFKRSSNKWLKDDDYLLNEDGIPKQSFPNHWKGRKGLYCAGLARRG

>Hyque.16G098300|Hquercifolia_v1.1/1-379

MEKRDRVTIIVGAGTSGLATAACLNRLSIPNILLEREDCFASLWQKKSYDRLHLHLAKQFCELPYMSFPSTFPTYVPRKEFIQYLDDYVNHFNISPMYQRSVEYANYDEVGKKWNVKVRNLTSNEIEEYSGRFLVVATGETSDVFVPEVDGLSSFMGEIIHSTQYKSGEIYRDKNVLVVGSGNSGMEISLDLSNYGAKTSIAIRSPLHIVSRGMIYMGVVLVKYIPFKLVDSLMVVLSKLWYRGLSKYGIQRPQEGPFAMNIKYRKYPVIDVGTYKKIKSGEIQVLPAITSIKGNNVLFKDGKSHPFDVIIFATGFKRSTNKWLKGDDYLLSDDGFAKQSSPNHWKGKKGLYCAGLARRGFYGAAMDAQNIANDIKLLM

>Hyque.16G098400|Hquercifolia_v1.1/1-377

MRDQVAIIVGAGPSGLATAACLNRLSIPNILLEREDCFASLWKKKSYDRLHLHLAKQFCELPYMSFPSTCPTYVPKKEFIQYLDDYVNHFNISPMYQRSVDFANYDEIGKKWNVKARNLTSNEIEEYSGRFLVVATGETSDVFIPEVDDLSSFMGETIHSTQYKSGENYRDKNVLVVGSGNSGMEISLDLSNYGAKTSIAIRSPLHIVSRGMVYMGLVLLKYIPYNLVDSLMVMLSKLRYGDLSKYGIQRPQEGPFALKVKYGKYPVIDVGTCKKVKSGEIQVLPAITSIKGNNVLFKDGKSHPFDVIIFATGFKRSTNKWLKGDDYLLSDDGLPKPSFPNHWKGQKGLYCAGLARRGLYGAAMDAQNIANDIKLLM

>Potri.002G207400|Ptrichocarpa_v4.1/1-377

MQDPVAIIVGAGPSGLATSACLNQHSIPHILLEREDCYASLWKKYSYDRLRLHLRKQFCELPRMSFPDSYPTYVPKDQFLQYLDDYVSHFKISPMYQRSVEFASFDEEAKKWNVKARNVSSGEIEEYSARFLVVASGETSNPFIPEFEGLNTFTGEVLHSTEFKNGKTYCDKNVLVVGSGNSGMEIALDLANHGARTSIAIRSPIHILSREMVYLGLNMLKYFSCGMVDKVMVMLSKLVYGDLSKHGIKRPKEGPFFMKVAYGKYPVFDVGTCNKIKSGEIQVLPALESIRGNEVVFENGKSHPFDTIVFCTGFERSTNKWLKGDDYLLNEDGIPKPGYPNHWKGKDGLYCIGLSRRGLYGASADAQNVVNDIKALI

>Qrob_P0019190.2|Quercus_robur/1-378

MEEQLTIIVGSGPSGLATAACLSQQSIPYIILEREYCIASIWKKYSYDRLHLHLAKQFCELPHMSFPASYPTFVPKKMFIQYLDDYVSHFSVSPMFQRTVESAEYNEVSKRWIVKARNASSGEVEIYSAKFLVVASGETTNPYTPEVAGLNTFPGEVLHSTQYKSGKEFKNKNVLVVGSGNSGVEIALDLANHGAKTTIIFRSPAHFLSREMVYLGLTMLKYFPVSLVDFLMVMLSKLVYGDLTKYGIGRPTEGPFYMKVKYGKYPLFDVGTYKKIKSGEIQVLPAEIIKVQGNDILFKNDKLHPFDTIIFGTGFKRSTNLWLKGDEYLLNEDGLAKPAYPNHWKGKNGLYCVGLARRGLYGASADAQNIANDIKSIT

>Qrob_P0339830.2|Quercus_robur/1-378

MQEQVAIIVGSGPSGLATAACLSQQSIPYIILEREECIASIWKKYSYDRLHLHLAKQFCELPHMSFPASYPTFVPKKMFIQYLDDYVSHFNISPMFQRTVESAEYNEVSKRWIVKARNASSGEVEIYSAKFLVVATGETTNPYTPEVAGLNTFPGEVLHSTQYKSGKEFKNKNVLVVGSGNSGVEIALDLANHGAKTSIIFRSPAHFLSREMVYLGLTMLKYFPVSLVDFLMVMLSKLVYGDLTKYGIGRPTEGPFYMKVKYGKYPLFDVGTYKKIKSGEIQVLPAEIIKVQGNDILFKNDKLHPFDTIIFCTGFKRSTNLWLKGDEYLLNEDGLAKPAYPNHWKGKNDLYCVGLSRRGLYGASADAQNIANDIKSIT

>Qrob_P0339890.2|Quercus_robur/1-378

MQEQIAIIVGSGPSGLATAACLSQQSIPYIILEREECIASIWKKYSYDRLHLHLAKQFCELPHMSFPASYPTFVPKKMFIQYLDDYVSHFNISPMFQRTVESAEYNEVSKRWIVKARNASSGEVEIYSAKFLVVATGETTNPYTPEVAGLNTFPGEVLHSTQYKSGKEFKNKNVLVVGSGNSGVEIALDLANHGAKTSIIFRSPAHFLSREMVYLGLTMLKYFPVSLVDFLMVMLSKLVYGDLTKYGIGRPTEGPFYMKVKNGKYPLFDVGTYKKIKSGEIQVLPAEIIKVQGNDILFKNDKLHPFDTIIFCTGFKRSTNLWLKGDEYLLNEDGLAKPAYPNHWKGKNGLYCVGLSRRGLYGASADAQNIANDIKSIT

>Qrob_P0491570.2|Quercus_robur/1-301

MFIQYLDDYVSHFNISPMFQRTVESAEYNEVSKRWIVKARNASSGEVEKYSAKFLVVATGETTNPYIPEVEGLNTFPGEVLHSTQYKSGKEFKNKNVLVVGSGNSGVEIALDLANHGAKTSIIFRRPAHFLTREMVYLGLTMLKYFPVSLVDFLMVMLSKLVYGDLTKYGIGRPTEGPISMKLKYGKYPLFDVGTYKKIKSGEIQVLPAEIIKVQGNDILFKNDKLHPFDTIIFCTGFKRSTNLWLKGDEYLLNEDGLPKPTYPNHWMGKNCLYCVGLSRRGFYGASIDAQNIANDIKSTV

>Qrob_P0019220.2|Quercus_robur/1-378

MQEQVAIIVGAGTSGLAIAACLSQQSIPYIILEREDCIASIWKKYSYDRLHLHLAKEICELPYMSFPASYPKYVPKNMFTQYLDDYVSHFKISPMYQRNVESAEYNEVSKRWFVKARNASSGEVEIYCAKFLVVATGETTNPYIPEVEGLNTFTGEVLHSTQFKSGKDFKNKNVLVVGSGNSGMEIALDLTNHGAKTSIIIRSPIHILSREMMDFARILVKYLKPSLLDSLVVMLSKLVYGDLTKYGIRRPTEGPFYMKRMFGKYPLIDVGTCKKIKSGEIQVLPAEILNIRGNDIIFKNGKLHPFDTIVFCTGFKRSTNLWLKGDDYLLNEDGLAKPNNPNHWKGKNGLYCVGLLQRGFYGASTEAQNIANDIKSIM

>Qrob_P0339870.2|Quercus_robur/1-378

MQEQVAIIVGAGTSGLAIAACLSQQSIPYIILEREDCIASIWKKYSYDRLHLHLAKEICELPYMSFPASYPKYVPKNMFTQYLDDYVSHFKISPMYQRNVESAEYNEVSKRWFVKARNASSGEVEIYCAKFLVVATGETTNPYIPEVEGLNTFTGEVLHSTQFKSGKDFKNKNVLVVGSGNSGMEIALDLANHGAKTSIIIRSPIHILSREMMDFAKILVKYLKPSLLDSLVVMLSKLVYGDLTKYGIRRPTEGPFYMKRMYGKYPLIDVGTCKKIKSGEIQVLPAEILNIRGNDIIFKNGKSHPFDTIVFCTGFKRSTNLWLKGDDYLLNEDGLAKPNNPNHWKGKNGLYCVGLLQRGFYGASTEAQNIANDINSIM

>Gohir.D08G080400|Ghirsutum_v3.1/1-378

MQEQPVIIIGAGPSGLATAASLNLHSIPYIILEREDCFASLWKKYAYDRLHLHLHKQFCQLPHLPFPHSYPRFISKQQFVSYLDDYVSHFKITPLYCRCVELAVFDEIIKEWTVKVRNLGSGEVEEFRGRFLVVASGEATDPYTPEIEGLKSFPGDVLHSTQFKNGKAFRDKNVLVVGSGNSGMEIAMDLANHGAKTSIVVRSPVHILSREMVYLGLNLLKYIPLNMVDWLMVMLSKLVYRELSKYGLSRPKEGPFFMKVADGKYPVLDVGTYSKIRSREIQVLPAILSVKGNEVVFENAESHAFDTIVFCTGFKRSTHIWLKGDDYLLNDDGIPKPSFPNHWKGKNGLYCVGLSRRGLYGAGFDARNIADDIKSLLK

>Gohir.A08G072200|Ghirsutum_v3.1/1-378

MQEQPVIIIGAGPSGLATAASLNLHSIPYILLEREDCFASLWKKNAYDRLHLHLHKQFCQLPHLPFPHSYPRFISKQQFVSYLDDYVSHFKITPLYCRCVELALFDEITKEWIVKVRKLSSGEVEEFRGRFLVVASGEATDPYTPEIEGLKSFPGDVFHSTQFKNGKAFRDKNVLVVGSGNSGMEIAMDLANHGAKTSIVVRSPVHILSREMVYLGLNLLKYIPLNMVDWLMVMLSKLVYRELSKYGLSRPKEGPFFMKVANGKYPVLDVGTYSKIKSREIQVLPAISSVKGNEVVFENAESHAFDTIVFCTGFKRSTHIWLKGDDYLLKDDGIPKPSFPNHWKGKNGLYCVGLSRRGLYGAGFDAQNIADDIKSLLK

>Gohir.D08G137000|Ghirsutum_v3.1/1-377

MQKEQVIIVGAGPSGLAMAACLNHHSIPYIILEREDCIASLWKKYAYDRLHLHLDKQFCVLPHMPFPDSYPRFVSRQQFVSYLDDYVSRFKISPLYRRTVESAEFDGETGKWIVKARNLGSGEVEELEGRFLVVASGETSNPFTPVIEGLNTFPGDVLHSTRFRNGKAFQNQKVLVVGSGNSGMEIAFDLANHGAQTSLVVRSPVHILSRDMIYLGLILVKYIRVNLVDSLMVMLSKLVYGDLSKYGINRPKEGPFFMKAVYGKYPITDIGTCKKIKSQEIQVLPSICSIRGNEVVFENGTTHPFDTIVFCTGFKRSTNVWLKGDDYLLNDDGISKLSFPNHWKGKNGLYCVGLSRRGLYGAASDALNIADDINSLQ

>Gohir.A08G116100|Ghirsutum_v3.1/1-377

MQKEQVIIVGAGPSGLAMAACLNHHSIPYIILEREDCIASLWKKYAYDRLHLHLDKQFCVLPHMPFPDSYPRFVSRQQFVSYLDDYVSRFKISPLYRRTVESAEFDGETGKWIVKARNLGSGEVEELEGRFLVVASGETSDPFTPVIEGLNTFPGDVLHSTRFRNGKAFQNQKVLVVGSGNSGMEIAFDLANHGAQTSLVVRSPVHVLSRDMIYLGLILVKYIRVNLVDSLMVMLSKLVYGDLSKYGIYRPKEGPFFMKAAYGKYPITDIGTCKKIKSQEIRVLPSICSIRGNEVVFENGTTHPFDTIVFCTGFKRSTNVWLKGDDYLLNDDGISKLSFPNHWKGKNGLYCVGLSRRGLYGAASDALNIADDINSLQ

>evm.TU.Scaffold_2016.135|Carabica_v0.5/1-385

MATKEQVVIVVGAGPSGLATAACLTSLSIPNLVLEREDCFASLWKKYSYDRLHLHLAKQFCQLPLKPFPTTYPTYVSRDQFLRYLDDYVSHFNICPLYQRSVESAQYDEAAEAWIVKARNLGSSDPEEMEEYSSKCLVVATGETTDAFIPQLEGLNTYLGEVLHSTRYKNGKSYENKNVLVVGSGNSGMEIAFDLSNYGAKTSIAVRSPLHIMSRGMANMGLVLLKYVSLNAVDSLLLMLSKLWYGGDLSRYGIKRPEEGPFAMKVKYGKYPVIDIGTYQKIKSGEIQVLPAVASLGGNDVVFEDGKSYPFDAIIFATGFKRSTNKWLQGADHLLTDDGLAKPAFPNNWKGTKGLYCAGLARRGLYGAALDAQSIANDIKRALQM

>evm.TU.Scaffold_557.187|Carabica_v0.5/1-385

MATKEQVVIVVGAGPSGLATAACLNSLSIPNIVLEREDCFASLWKKYSYDRLHLHLAKQFCQLPLKPFPTTYPTYVSRDQFLRYLDDYVSHFNICPLYQRSVESAQYDEAAEEWIVKARNLGSSDPEQMEEYSSKCLVVATGETTDAFIPQLEGLNTYLGEVIHSTRYKNGKSYENKNVLVVGSGNSGMEIAFDLSNYGAKTSIAVRSPLHILSRGMANMGLVLLKYVSLNAVDSLLLMLSKLWYGGDLSRYGIKRPEEGPFTMKVKYGKYPVIDIGTYQKIKSGEIQVVPAVASLGGNDVVFEDGKSYPFDAIIFATGFKRSTNKWLQGADYLLSDDGLAKPAFPNNWKGTKGLYCAGLARRGLYGAALDAQSIANDIKRALQM

>evm.TU.Scaffold_557.190|Carabica_v0.5/1-385

MATKEQVVIVVGAGPSGLATAACLNNLSIPNIVLEREDCFASLWKKYSYDRLHLHLAKQFCQLPLKPFPTTYPTYVPRDQFLRYLDDYVSHFNICPLYQRSVESARYDEAAEAWIVKARNLGSSDSEEMEEYSSKCLVVATGETSDAFIPQLEGLNTYLGEVIHSTRYKNGKSYENKNVLVVGSGNSGMEIAFDLSNYGAKTSIAVRSPLHILSRGMVYIGPVLLKYFSLNTVDWLVLMLSKLWYGGDLSRYGIKRPEEGPFTMKVKYGKYPVIDVGTCQKIKSGEIQVLPAVASLGGNDVVFEDGKSYPFDAVIFATGFKRSTNKWLQGADDLLTDDGFAKPAFPNNWKGTKGLYCAGLAGRGLYGAALDAQNIANDIKRVLQL

>DCAR_521004|Dcarota_subsp_sativus_v3.0/1-379

MDVKETVVIIVGAGPSGLATAACLKAQSIPYMILERENCFASLWKHKSYDRLHLHLAKQFCQLPFMSFPKSSPTFVPKKEFLQYLDDYVTHFAINPLYQRLVVSAFYNESLGKWNVKATNGVSGELEEYISRFLVVATGVTCDAYIPKVEGLSKFAGDVIHSTQYKSGGIYENQHVLVVGSGNSGMEIAFDLANHGAKASIVVRSPVHVLSRGMVYWGLKLLNYFPYYIVDSLMVLLSKIKYGDLSKYGICRPQEGPFSLKVKYGKFPVIDVGTCSKIKSGEIQVLPGLRSIGETEVIFEDGRSYPFDSIIFATGFTRSTKAWLKGDEYLLNDDGLPKPEFPNHWKGEKGLYCVGLARRGLYGAAMDAQNVANDINNLV

>DCAR_314079|Dcarota_subsp_sativus_v3.0/1-378

MEFKEAVIIVGAGPSGLATAACLKTHSIPYIILEREDCFASLWKQKAYDRLHLHLAKQFCQLPHMPFPKSCPTFVPRKQFLQYLDDYVTRFDIKPLYKRLVVSAFYDESSMKWTVEVTNGVSGEAEKYYGRFLVVATGETCDPYVPEFEGLSKFKGDAIHSTQYKCGEAYENKHVLVVGCGNSGMEIALDLANYGAKVSIVVRRPAHVLSRGMVYWGLILLKYFPYYMVDSLMLLLSKIKYGDLSKFGIHRPEKGPFALKVKDGKYPIIDVGTCKKIKSGEIQVLPGLRSIAESEIFFEDGRSDPFDSIIFATGFTRSTKMWLKGDDFLLNEEGFPKPGFPNHWKGEKGLYCVGLARRGLYGAAMDAENIANEIKKLF

>VIT_207s0104g01260|Vvinifera_v2.1/1-389

MPEAAVIIVGAGPSGLAMAGCLCQLSIPYLILEREDCCASLWKKKAYDRLHLHLPKQYCTLPHMEMPADWPKYPSRQQFVQYLDDYADHFNIRPMYRRSVESGSFDESRGKWNVGVRNGESGELEEYSGLFLVVASGETSDAFVPDIDGLSTFIGKVIHSTQYKNGKEFADMKVLVVGSGNSGMEIALDLSNCGAKTSIVVRSPLHMLSREMVNLGLALLKYIPYNMVDSLMVILSKLVYGDLNKYGITRPEEGPFFLKVKYGKYPVVNTGTFGKIKSGEIQVLPKLIGIRGDEVVFEGGKSHPFDAIVFATGFKRSTSKWLKGDDYLLNEDGLPKPSFPNHWKGKNGLYCAGLARRGLYGSALDAQNIANDIKTQLRSNIFKFFLKFL

>Glyma.03G208900|Gmax_Wm82.a6.v1/1-384

MEEKQRVIIVGAGPSGISAAACFTKQSIPYIILEREDCSASLWKKYSYERLHLHLRKQYCQLPHKPFPASFPPYVPKKQFLQYLDDYVSHFGITPLYRRTVELAEYDQGCHNWRVMALNGDSGQLEEYRGRFLVVATGETTDPFVPELQGLSGFPGKLIHSTGFRSGKDFKDQHVLVVGSGNSGMEIALDLVNHGAKTSILVRSPVHFLSREMVSLGLFLLKYLSLSTVDSLMVMLSTMIYGDVTKYGVARPNEGPFYMKVKYGKYPVIDVGTYKKIKSGELKVLPSEIESLRGKDVLFKNGESHPFDSIVFCTGFKRSTNKWLKGDDYLLNDEGLPKPSYPIHWKGNNGLYCVGLSRRGFYGAAADAENIANDVSSFTQHPCP

>KAI5425124.1|Pisum_sativum/1-384

MKKENQTVIIVGAGPSGLSVAASLTNQSIPYIILERENCFASLWKKYAYDRLHLHLKKQFCELPHKPFPPSFPSYIPKEKFLQYLEDYVSHFKINPVYHRTVEYAEFDEGCEKWKVKAVNKDSGEVEEYDGRFLVVASGETSDPFVPEIEGLKSFGGKVIHSTRFKNGKEFKDEHVLVVGSGNSGMEIALDLINHGAKTSILVRSPVHILSKGMLNLGLFMMKYLSMKIVDSLMVMLSKLVYGDMTNYGVGRPNEGPFYMKVKYGKYPVVNVGTYQKIKSKELKVLPTEIECLSGNNVLFKNGELHTFDSIIFCTGFKRSTHKWLKGDDYLLSDDGIPKQSYPFHWKGKNGLYCVGLSRRGLYGAAQDAENVANDVRSIMQEIV

>KAI5425125.1|Pisum_sativum/1-383

MKKENQTVIIVGAGPSGLSVAASLTNQSIPYIILERENCFASLWKKYAYDRLHLHLKKQFCELPHKPFPPSFPSYIPKEKFLQYLEDYVSHFKINPVYHRTVEYAEFDEGCEKWKVKAVNKDSGEVEEYDGRFLVVASGETSDPFVPEIEGLKSFGGKVIHSTRFKNGKEFKDEHVLVVGSGNSGMEIALDLINHGAKTSILVRSPVHILSKGMLNLGLFMMKYLSMKIVDSLMVMLSKLVYGDMTNYGVGRPNEGPFYMKVKYGKYPVVNVGTYQKIKSKELKVLPTEIECLSGNNVLFKNGELHTFDSIIFCTGFKRSTHKWLKGDDYLLSDDGIPKQSYPFHWKGKNGLYCVGLSRRGLYGAAQDAENVANDVRSIMQEI

>Eucgr.K03435|Egrandis_v2.0/1-379

MEQEAAAAVVVGAGPSGLAASACLKTLSIPHVVLEREDCFASMWKKYAYDRLHLHIRKRYCELPHMHFPPDYPEYVPKDQLLRYFDDYVARFGIEPAYGRSVESAVYDEGDGMWRVGARDVGSGEAVEYRCRFLVVATGESSDAFIPEVEGIEGFGGKVIHSTRYKNGKEFENSRVLVVGSGNSGMEIALDLANHGARTSIVVRSPVHVLSKGIANLGLALLKYFSPGTVDTLLVLLSKLVYGDMSKYGLRRPSEGPLLTKTKYGKYPFIDLGTCQKIKSGEIQVLPALKRIEGDVAEFEDGKSHPFDAIVFCTGFKRSTHKWLKGDDYLLNEDGIAKPSFPNHWKGKKGLYCVGLSRRGFSGAASDAQNVADDIKSQL

>Eucgr.G00316|Egrandis_v2.0/1-380

MEQEAAAAVVVVGAGPSGLAASACLKTFSIPHVVLEREDCFASLWKKYAYDRLHLHLRKRFCELPHMHFPPDYPEYVPKDQLLRYFDDYVARFGIEPAYGRSVESAVYDEGDGRWRVGARDVGSGEAVEYRCRFLVVATGESSDAFIPEVEGIEGFGGKVIHSTRYKNGKEFENSRVLVVGSGNSGMEIALDLANHGARTSIVVRSPVHVLSRGIANLGLALLKYFSLGTVDSLLVLLSKLVYGDMSKYGLRRPSEGPFLMKTKYGKYPFIDLGTCQKIKSGEIQVLPALKRIEGDVAEFEDGKSYPFDAIVFCTGFKRSTHKWLKGDDYLLNEDGIAKPSFPNHWKGEKGLYCVGLSRRGFYGAASDAQNVADDIKSQL

>Eucgr.K03436|Egrandis_v2.0/1-379

MEQEAAAAVVVGAGPSGLAASACLKTLSIPHIILEREDCFASLWKKYAYDRLHLHVRKRYCELPHMHFPPDYPEYVPKDQLLRYFDDYVARFGIEPAYRRSVESAVYDEGDERWRVGARDVGSGEVMEYRCRFLVVATGESSDAFIPEVEGIEGFGGKVIHSTRYRNGKEFENSRVLVVGSGNSGMEIALDLANHGARTSIVVRSPVHVLSRGIANLGMALLKYFSPGTVDSLLVLLSKLVYGDMSKYGLRRPSEGPLLMKTKYGKFPFIDLGTCQKIKSGEIQVLPALKRIEGDVAEFEDGKSHPFDAIVFCTGFKRSTHKWLKGDDYLLNEDGIAKPSFPNHWKGEKGLYCVGLSRGGFPGATSDAQNVADDIKSQL

>VIT_207s0104g01250|Vvinifera_v2.1/1-378

MQETVVIVVGAGPSGLATAASLNLLSIPNIVLEREDCFAPLWQKKSYDRLHLHLPKQACELAHMPMPTSYPTYPSRLQFIQYLRDYVSHFGISPVYHRLVESASFDEVTEKWKVKVRVINGGSDEIEEYSCRFLVVASGETSDAFIPEVEGLSSFKGEVLHSTQYKCGKEYAEKTVLVVGSGNSGMEIALDLSNYGAKTSIVVRSPVHILSKEIMHLGLFLARYLPFNMVEYLTVMLSKIMYGDLTKYGIIRHEEGPFTVKAKYGKYPIIDLGTYKKIKSGEIQVLPALTSIRGSEVVFKNGESHPFDVIVFATGFKRSTNKWLKDDDLLDDNGFARLMPPNNWKGKKGLYCAGLAGRGLTGARVDAEKIANDIKTLL

>GWHPBAUK022513.1|Macadamia_integrifolia/1-384

MEMSQKMSDDELVIVAGAGPSGIATSACLNLLSIPNIVLEREDCCAPLWQKKSYDRLHLHLPKQSCELPHMPFPSVYPKYVPKQQFIEYLEDYVNHFKVNIKYNRSIESATYDELTKTWCVKAMNTTLYEMEEYKTRFLIIATGETCDPFIPEIEGLDSFKGEVLHSTQYKSGDKYRNKSVLVVGCGNSGMEIAFDLSDFGAKTSIVVRNPVHILSKEILQVGLFLFKFLPFKLREFIVVMLCKLWFGDMTKYGITRPVEGPFTRKMKYGKFPVIDVGTAKKIKSGEIQVLPGITTIKAKEVTFKNGYSYPFDAIVFATGFQRSTNKWLKDDGYLLDEDGFSKQSFPNHWKGRNGLYCAGLARRGLYGSGMDAQNIANHIKTLL

>GWHPBAUK005613.1|Macadamia_integrifolia/1-378

MKKETVVIVAGAGPSGISTSACLNLHSIPHIVLEREDIYCPIWTKKSYDRLHLHLAKQFCELPHLPFPDSYPTYVPRKLFVQYLEDYVTQLKVNPVYNRSIESAAFDKATGKWLVKAKNRKTDDMGEYSSRFLVVATGETTDPFIPEIEGIESFSGEFLHSTKYKTGAPFANKSVLVVGCGNSGMELAYDLSNFGAKTSIVIRNPLHVITRWMMYVELELLKHLPYDMVDGFVLLLSKFWYGDLSKYGLTRPQEGPFAMKDKYGKYPIVDVGTIGKIKSGEIQVLPGISSVKDDEVIFIDGKSYQFDVILFATGFQRTTQKWLKDDEDLLDKDGIPKQSYPNHWKGKNGLYCAGLARKGLYGTAMDAQNIANDIKKLL

>GWHPBAUK005614.1|Macadamia_integrifolia/1-378

MKKETVVIVVGAGPSGISTSACLNLQSIPHILLEREDIYCPIWTKKSYDRLHLHLAKQFCELPHLPFPDSYPTYVPRKLFVQYLEDYVTQLKVNPVYNRSIESAAFDKATGKWLVKANNIKTDEMEEYSSRFLVVATGETTDPFIPEIEGIGSFSGELLHSTKYKTGAPFANKSVLVVGCGNSGMEIAYDLSNFGAKVSIVIRNPIHVVTRWMASMGLTLFKYLSHELVDELVLSLSKLWYGDLSKYGIRRPQEGPFAMKDKYGKYPVVDVGTIGKIKSGEIQVLPGISSVEGGQVIFTDGKFYHFDVILLATGFHRITKKWLKDDDDLLNEDGIAKKRYPNHWKGKNGLYCVGLARKGLYGAAVDAQNIANDIKKLL

>GWHPBAUK037266.1|Macadamia_integrifolia/1-378

MKETTVIVAGAGPCGITTSASLNLQSIPHILLEREDIFCPIWTKYSYDRLHLHLAKEFCELAHMPFPDSYPTYVPRNLFIQYLEEYMTRFNVNPVYNRSIESATYDKLAGKWIVKTRNTKTDEIEEYCSRFLVLATGETTDPFVPKIEGMETFTGQILHSTEYKTGKPFTNKSVLVVGCGNSGMELAYDLTLFNAKTSIVIRHPIYIITRWMGYMSLVLFQRLPFGLVDRFIALVSRLWYGDMSKYGIEKPTEGPYERKEKYGKYPVVDVGTAGKIKSGEIQVLPGISCINGDEVLFTNGKSYHFDVILSATGFHRSIRNWLKDDYGFLGEDGLAKERDPKPYWKGKNGLYCAGLARKGLIGAGPEGILIAHDIKKLL

>GWHPBAUK037265.1|Macadamia_integrifolia/1-377

MKETVAIVVGAGPAGISTSACLNLQSIPHILIEREDCYCPIWKKKAYDRLHLHLPKQYCELPHMSFPESYPKFVPRKQFVQYLDEYVSTFKVNPACNRTIELATYDEVAKKWSVKVRHNKSDEIEEYKASFLIVATGENCEPFIPEMEGLETFKGEVLHSTQYKSGSKYNNKNVLVVGCGDSGMEIACDLANYGAKTSVVIRSPLHVLTRWMVQFTLFLWKYLPLHWVDAIIVILSMLWFGDMSKYGITRPKEGPYYMKDKYGKYPNIDAGAIKKIKAGEIQVLPGISSIDGNEVSFTNGKSYYFDVVLFATGFRRTTKKWLKGDDYLLGEDGIAKQSFPNHWKGKKGLYCAGLGRAGLRGLGMDAQSIANDIKTLL

>Soltu.DM.09G022810|Stuberosum_v6.1/1-410

MYIDIPHLFFFSQHHQIFFSILVMTTFDNHEEAMVIIVGAGPGGLATSACLNKLCIPNLILEKEDCYSPMWKKYSYDRVHLHLAKQFCQLPYIPFPSSSPTYIPKKLFIQYLDNYVTHFNITPFYKRKVEFAKFDKFTKKWNVKVRNGNSDEMEEYFCKFLVVATGEASYPFTPDVPGLASFTGDAFHSTQYKNGEKYKGKNVLVVGCGNSGMEIALDLANNGVNTSIIVRSPMHLISREMGYLGLMLLKYKVAYTVVDTIMVMLSKLMYGDISKYYGVKRPEEGPFACKIKYGKYPVFDVGTYQKIKSGEIQVLPVMKGIRGNDVVLENGKIHQFDGIVFSTGFKRTIHKWLQGDDYLLNEDGLPKPEFPQHWKGKNGLYCVGLSRRGLYGIAFDAQNIATHINSLLSK

>Soltu.DM.09G022830|Stuberosum_v6.1/1-410

MYIYIPHLFFFSQHHQIFFSILVMTTFDNHEEPMVIIVGAGPGGLATSACLNKLCIPNLILEKEDCYSPMWKKYSYDRVHLHLAKQFCQLPYIPFPSSSPTYIPKKLFIQYLDDYVTHFNITPFYKRNVEFAKFDEFTKKWNLKVRNGNSDEMEEYFCKFLVVATGEASYPFTPDVPGLASFTGDAFHSTQYKNGEKYKGKNVLVVGCGNSGMEIALDLANNGANTSIIVRSPMHLISREMGYLGLVLLKYKVAYTVVDTIMVMLSKLMYGDISKYYGVKRPEEGPFACKIKYGKYPVFDVGTYRKIKSGEIQVLPAMKSIRGNDVVLENGKFHQFDVIVFATGFKRTTHNWLQGDDYLLNKDGLPKQEFPQHWKGKNGLYCVGLSRRGLYGIAFDAQNIATHINSLLSE

>Solyc09G002190|Slycopersicum_ITAG5.0/1-388

MATFDNHVEQPMVIIVGGGPGGLATSACLNKLCIPNLILEKEDCYSPMWKKYSYDRVHLHLAKQFCQLPLFPFPSSSPTYVPKKQFIQYLDDYVTHFNITPFYNRNVEFAEFDVITEKWNVKVRNGNSGEMEEYFCKFLVVATGEASYPFIPDVPGLTSFTGEAIHSTQYKNAEKYKGKNVLVVGCGNSGMEIALDLANNGANTSIIVRSPMHLISREMGYLALMLLKYKVALRVVDTIMVMLSKLMYGDISKYYGVKRPEEGPFACKVKYGKYPVFDVGTYRKIKSGEIQVLPAMRSIRGNDVVVENGKIHQFDGIVFATGFKRTTHKWLQGDDYLLNEDGLPKPEFPQHWKGKNGLYCVGLSRRGLYGIAFDAQNIATHINSLLSE

>Rhdel01G0085900.1|Rhododendron_delavayi/1-379

MSLLDPVVIIIGAGASGLATAACLTHLSIPYIILEREDCSASLWKKKSYDRLHLHLAKQYCQLPHFPFPTNFPKYVPKSQFIQYLDSYATHFHITPQFNRLVDSARFDESIEKWSVVARNVGSGEEEEYSGRFLVVATGRESEAFVPVVEGLGGFTGEVIHSTGYKSGEKYRERRVLVVGCGNSGMEIAFDLAVHGAKTSIVVRIPIHILSKELMNWAIELLKYLPFSTVDSLVLLFSKLWYGGLAKYGIQRPNEGPLTLKKKYGKSPVVDVGTYNKIKSGEIQVLPAIARIKGDEVEFENGKSHQFDTIVFATGFKRSTHKWLKGDNYLLDEDGNPKPSFPNHWKGQNGLYCDGLAGRALHGAGLDAQNIANDIKSSL

>Kalax.0339s0022|Klaxiflora_v1.1/1-383

MQQQHQQVIVVGAGPSGLAVAACLGLNSIPYVLLERDDCYAPIWKKKSYDRLNLHLAKQFCELPHMSFSPKSPTYLSKNEFVQYLDEYVTRFHISPVYRRSVELAEFDEESKLWHVKVRKVDDGVFEEFTTTYLVVASGETANAFIPEVEGLADFQGEVIHSTQYKNGKEFEKKSVLVVGSGNSGMEIALDLANFGAKTSIVVRSPTHVLSRGMAYIGLVLFGYLPFHLVDSFVAVLSKLRYGDLSKYGIKRPEETPFLMKEKYSKYPVIDVGTIKKIKSGEIQVLPAMKRVRGGQAEFENGKSHPFDVIIFATGFKRSTCIWLKGDDYLLNEDGLPKPHFPDHWKGEKGLYCCGLSRRGLYGICLDAKSIADDINKSRLNTK

>Kalax.1105s0008|Klaxiflora_v1.1/1-383

MQQQHQQVIVVGAGPSGLAVAACLGLNSIPYVLLERDDCYAPIWKKKSYDRLNLHLAKQFCELPHMSFSPKSPTYVSKNEFVQYLDEYVTRFHISPVYRRSVELAEFDEESKLWHVKVRKVDDGVFEEFTSTYLVVASGETANAFIPEVEGLADFQGEVIHSTQYKNGKEFEKKSVLVVGSGNSGMEIALDLANFGAKTSIVVRSPTHVLSRGMAYIGLVLFGYLPFHLVDSFVAVLSKLRYGDLSKYGIKRPEETPFLMKEKYSKYPVIDVGTIKKIKSGEIQVLPAMKSVRGGLAEFENGKSHPFDVIIFATGFKRSTCIWLKGDDYLLNEEGLPKPHFPDHWKGEKGLYCCGLSRRGLYGICLDAKSIADDINKSRLNTK

>Pso09G03390.0|Papaver_somniferum/1-385

MATAADQVVIIVGAGPSGIATSACLNHLSIPNIVLERDDCLASIWTKKSYDRLHLHLSKQFTDLPHMPLPSDYPMFISRQQFVDYLENYATHFNVSPLYNRCVELATFDENTKKWNLKVKNTGSAGDSEDYEEYSTKFLVVATGETTDAYIPEIEGLSGFKGDILHSTEFKNGEKFAKKNVLVVGSGNSGMEISLDLADFGANTSIVVRSPVHIINKETAYLGLRLLKHLPLNIVDGLLVFLSKLVYGDTSKYGIVRPKEEGPFLMKGKYGKYPILDVGTFNKIKSGHIQVLPAIKSITGDCVEFVNGKSYQFDVLLFATGFTRATNKWLKDDDYLIGENGISKQSFPNNWKGKNGLYCAGLARKGIYGTAIDAQKIAADINTLL

>Glyma.19G206200|Gmax_Wm82.a6.v1/1-390

MQEATKVIIIGAGTSGIATAGCLTKQSIPYIMLEREDCFASLWQKYTYDRLHLHLRKQVCELPHLPFPKSYPHYVPRKQFIDYLGNYVNHFEIKPLYQRAVELVEYDGWKGIWRVKAQNRRSGELEEYAGKYLVVASGETAEPRLPQIQGLESFNGKVIHSTAYKNGNEFKNKHVLVVGSGNSGMEIALDLSNFGAKPSIIVRSPVHFLSRDMMYYASLMLNYLSLSTVEKVLVMVSKVVYGDLSEYGIPYPSEGPFTMKMKYAKFPIIDVGTVKKIKSREIQVLPAEIKSIRGNEVLFQDGKSYTFDSIVFCTGFKRSTQKWLKGGDDLLNEDGFPKNSFPNHWKGRNGLYCVGLSRRGFFGANMDAQLVANDIASLIPQEEREGICVM

>KAI5425121.1|Pisum_sativum/1-396

TKLIKPKLRNSIMQVAPVIIIGAGTSGIATAASLTKQSIPFIILERENCFASLWQNYTYDRLHLHLRKQICELPYFPFPSSYPNYVPKKQFIQYLDSYVKNFNINPLYNRKVVLAEYFEDDEKWRVEAENRSSGEVEEYSGRFLVVASGETAEPRVPEVLGLESFNGKVMHSTGYKNGREFKDEHVLVVGSGNSGMEISLDLANFGAKPSIIVRSPVHFLSRDMMYYAGVLMSYLSLSTVEKLVVIVSRIVYGDLSKYGIPFPSEGPFTMKNKYGKFPIIDLGTVKKIKSGEIQVLGAEIESIRGNQVLFRDGKSYPFDSIIFCTGFSRSTQKWLKGSDDLLNEDGFPKPGLPYNWKGKNGLYCVGLSRRGFYGANLDAQNVANDIALLVPQDERK

>CKAN_01929600|Ckanehirae_v3/1-386

MEIAVVIVGAGPSGIATSACLNLHSISNIVLEREDCSASLWRKRAYNRLNLHLAKEFCQLPHMPHPPNAPTFLSKKDFINYIDDYVTHFNVNPIYRQSVESARYDATIGRWRVEAKDTCSGEVIEYIARFLVVATGENSEGSIPEIPGLDTFSGDVIHSSQFKSGKSYSGKAVLVVGSGNSGMEIAYDLSNYGAVASIVIRNPSHIVTKEIVHLGMVLLKFLPLSLVDSLTLIQSRWKFGDLSKYGLRRPKEGPFFIKATKGHSPVLDVGTIGKIKTGEIKVLPALKGLKGNEATFANGEAHCFDAIVFATGYISTAKRWLQDGDDLLNKDGMPRSRFPNHWKGKMGLYCAGFSSRGLDGVATDARMIAEDIKKICMEEELEPPLK

>Litul.04G157500|Ltulipifera_YP108A_v1.1/1-381

MESVVVIVGAGPSGLATSACLNHLSIPNIILEREDCCASLWKKRAYDRLNLHLAKQFCYLPHMPHPNSSPTYIPKKDFIQYIDQYVSHFKLAPLYRRSVESALYNQDVGQWVVMARNMVTDEVEEYRGRFLVIATGENSQGFIPDVPGLETFPGEVVHSKLYKSGSDYSGKAVLVVGSGNSGMEIAFDLSNFGAKTSVVVRSPVHVLTRDMVHLGMVLQKYLPCSLVDTLTLLLSKLKFGDLSKYGICRPAKGPFYLKAIAGRSPIIDVGTIGKIKSGEIQVLPALASIRHGLADFADGTSHPFDAIVFATGYRSTAKCWLKGDEYLLDKEGMPLPSFPNHWKGKEGLYCAGLSRRGLAGVSMDAWNIANDIKRVLDEHFN

>Litul.04G157400|Ltulipifera_YP108A_v1.1/1-384

MESVVVIVGAGPSGLATSASLNHLSIPNIILEREDCYASLWKKRAYDRLNLHLAKQFCYLPHMPHPPSSPTYIPKKDFIQYIDQYVSHFKLAPLYHRSVESALYNQDVGRWVVMARNMVTDEVEEYRGWFLVIATGENSQGFIPDVPGLETFPGEVVHSSLYKSGSDYSGKAVLVVGSGNSGMEIAFDLSNFGAKTSVVVRSPVHVLTRDIVHLGMILLKYLPYSLVDTLTLFLSKLKFGDLSKYGVRRPAKGPFYLKAIAGRSPVIDVGTIGKIKSGEIQVLPALASIRDGLADFADGTSHPFDAIVFATGYRSTAKCWLKGDEYLLDKEGMPLLSFPNHWKGKEGLYCAGLSRRGLAGVSMDAQNIANDIKRVLDEHFKHFN

>Litul.04G157700|Ltulipifera_YP108A_v1.1/1-378

MDNMVVIVGAGPSGLATSACLNLLSIPNIILEREDCCASLWKKRAYDRLNLHLAKQFCYLPHMPHPPSSPTYISKNDFIQYIDQYVSHFKLAPLYHRSVTSALYDQDVGRWVVMAHNTTSDAVEEYRGRFLVVATGENSEGFIPDVPGLETFHGEVVHSCLYKSGRDYSGKAVLVVGCGNSGMEIAFDLSNFGAKTSIVVRNPVHVLSRDMVHLGMVLLKYLPCSLVDALTLMLSKLKFGDTSNYGIRRPTKGPFHLKATTGRSAVIDVGTLRKIKSGEIKVLPALASIKDGEAYFANGTLYPFDAIVFATGYRSTAKCWLKGDEDLLDKDGMPLPSFPNHWRGKKGLYCVGLSRSGLAGVSMDAQNIANDIMKALGE

>Acora.05G103700|Aamericanus_v1.1/1-397

MKKEVVIVGGGPSGLATSACLNLLSIPNVILEKEDCYNSLWKKRSYDRLKLHLAKEFCNLPHFPHPKSSPTYIPKDAFIKYVDDYVSHFNLNPLCCKYVESASYDEETKTWRVMSKDTNTGEVDEYIAKFLVVATGENAEGYIPDVPGLETFHGSVLHSSQYKSGSKYKGDAALVVGCGNSGMEIAFDLANFGAKTSISIRSAFHVVPNEVIHMGMVLLKYLPLKFVDTVVNLFRGLLYGDLGKYGIYRPKDGPFFLKATTGRSPVIDVGTISKIKSGEIQVLPEIKEIRGSKVVFSDGKSHPFDAIIFSTGYKSTANYWLKDGEHLLNKEGFPKEKFPNHWKGKNGLYFAGLARRGLAGVSMDAQNIANDIKKIFQCCCNLRHHQAIPIQEDPCMY

>Acora.05G088300|Aamericanus_v1.1/1-386

MKKQVVIVGAGPSGLATSVCLNLLSIPNVILEKEDCYNSLWKKRSYDRLKLHLAKEFCNLPHFPHPKSAPTYIPKDAFIKYVDDYVSHFNLNPLCCKYVESASYEEETETWCVVSKDTNTGEVDEYIAKFLVVATGENGEGYIPDIPGLESFHGSVLHSSQYKSGSKYKGDSVLVVGCGNSGMEIAFDLANFGAKTSISVRSTFHVVKKEIVHMGMVLLNYLPLKFVDTLVNMLCRLLYRDLSKYGIHRPKDGPFFLKVTTGRSPVIDVGTVSKIKSGEIQVLPEIKDIRGSEVVFSDGKSHPFDAIVFSTGYKSTANYWLKDDENLLNKEGFPKERFPNHWKGKNGLYFAGLARRGLEGVSMDAQNIANDIKNKFSAVVAKASPS

>gene_prefix05956.t1_1|Ceratophyllum_demersum/1-383

MEEVVLIVGAGPSGLATAACLNKHSIPYIILEREDCCGSLWKKRTYDRLKLHLAKRFCHLPHQPHSPSAPNYIPKNDFIKYMDDYANDFNIVPRYCRSVESATYDEITEKWRVMARNLVTNELEEYVSRFLVVATGENSEAIIPSIPGLSSFNGTVLHSNDYKSGTEFVGKSVLVVGSGNSGMEISYDLATFGVQTSIVVRSKFHVLTKEMVHLGMFLLGYLPLSLVDSFISLVGDLKYGDLSKYGIERPRQGPFYLKASTGTSSVIDVGTIGKIKKGEIQVYPGLTSINGSVASFEDGSRHSFDVIIFATGYRSTVHYWLKDNKSLMGENGMPKNRYPRHWKGRNGLYCTGFASRGLDGVSEDAQNVANDIKYSYTSKVFTI

>gene_prefix15828.t1_1|Ceratophyllum_demersum/1-387

MEEVVLIVGAGPSGLATAACLNKHLIPYIILEREDCCGSLWKKRTYDRLKLHLAKGFCHLPHQPHPPSAPNYIPKKDFIRYMDDYVSNFNIAPRYRRSVESATYDEITEKWRVMARNLVTNEMEEYLSRFLVVATGENSEAVIPNIPGLNSFNGTVLHSNDYKSGIEFVGKSVLVVGSGNSGMEISYDLATFGVQASIVVRSKFHVLTKEMVHLGMFLLRYLPLSLVDSFISVVGNLKYGDLSKYGIQRPRQGPFFIKATTGTSAVIDVGTIGMIKKGEIQVLPGLTSINGSVASFEDGSRHSFDTIIFATGYRSTVHYWLKDNKSLMGENGMPKNRFPHHWKGRNGLYCTGFASRGLDGVSADAQNVANDIKESYTSKAFTLYNHK

>BUSI_006822-RA|Buxus_sinica/1-384

MKVVVAIVGAGPSGLATSACLNKLSVPNIILEREECCASLWKNRTYDRLKLHLAKSFCELPYMPFPPSAPTYISRNGFIEYIDNYVSQFNISPRYSRLVQSATYDVATKKWCIKAKNSVTNQMEVYISDFLVVATGENSEGFIPEIPGLESFKGKVIHSSQYKSGWEYSGKEVLVVGSGNSGMEIAYDLCNSGANTSLVVRSPFHVVTKEMVHLGMILLKFIPLGLVDSFIVMLEKAVFGDLAKYGIRRPAEGPFHLKAVAGRSPVIDVGTVNKIKMGEIHVVPKISSIKENYVVFDNKRAGQFDLIIFATGYKSTTNYWLKDYGYVLNDSGMPKYNFPKHWKGENGLYCAGFSQRGLAGVSMDAKAIALDISAARLVGKEKIY

>Aqcoe6G037100|Acoerulea_v3.1/1-382

MEGKEVQVVIVGAGPSGIATSACLKNFSISNIILEREDCCASLWKKKVYDRLKLHLAKEFCHLPHMSLPSDAPVYVPKDQFVSYIDSYVSYFDINPKFHRFVESASYDATSKKWRISAKNSNTSKVEVYVADFLVVATGENSERFIPNFPGQESFVGEIIHSSQYKSGSVYRNKNVLVVGSGNSGMEIAYDLFYHGACTSIVVKSPFHVLTKEMVRLGMQLLKCIPIGWVDSLITKIANLWYGDLSEYGIPRPAQGPFLLKVTTGKSPVLDVGTVHAITKGGIKVLPAVSSIKEKSVVFANGEAHQFDAIISATGYRSTANYWLKDYDTLLNNESMPKHKFPDHWKGSNGLYCAGLGKRGLAGVNMDARAIADDIKSLLTCM

>Pso06G33940.0|Papaver_somniferum/1-380

MEEVVVIVGAGPSGLATSACLKLLNIPNIILEKENVYASLWKDRSYDRLKLHLAKQFCHLPHMPYPPNAPTFVPRQEFIRYIDDYVSTFNIKPKYHRSVVSAKYDALHQKWRIEVTNTMTGEVELYFARFLVIATGENSKGYIPRVQGLECFEGKVIHSSQYKSGAEYNGKKVLVVGCGNSGMEIAYDLSNYGASTSIVVRSRFHVLTKEMVYLGMVLLKYLPVSCVDPLINMLSYLSYGDLSKYGIHRPAAGAFEIKAKAGRSPVIDVGTVERIKNGVITVMPGISRMEKNNIVFDNDTKKHHFDAIIFATGYRSTVNNWLTDDHDSLLGKSGMPKNPFPDHWKGANGLYCAGLSRRGLAGVSMDAGTIAKDIKKSFFS

>Pso06G33910.0|Papaver_somniferum/1-411

MVSTDKVSILSLYRVLHLLILHFILGFSMEEVVVIVGAGPSGLATFACLKQLNIPSIILEREDCHASLWKKRAYDRLKLHLAKQFCSLPHMPYPPNTPTFVPRAKFVEYIDSYVEKFDINPKCNRSVESAVYDDHEAKWKIEVKNLVTGEPEHYVATFLVVATGENSKGFIPKIPGIESFAGDILHSSEFKSGKEYNGKKVLVVGCGNSGMEIAYDLSNHGASTSIVIRSPFHIVTKEMIWLGMNLLNYLPLWLVDSITLALARLAYGNLDKYGIHRPSGGPFELKVKLGRSPVIDVGTVAKIKQGIIKVFPEIMKIEKDSIQFNNGDTEQFDALVFATGYQSTANNWLKDFNYILGKDGMPKNKFPSHWKGTNAVYCTGLSRRGLAGVSADAQKVANDIAMIFKAEKRHN

>Aqcoe5G234600|Acoerulea_v3.1/1-386

MLELPVVAIVGAGPSGLATAVSLQQLSIPSIIFEKENCCASLWKNRSYDRLKLHLAKEFCSLPYFRFPTSTPKYIPKQTFIKYMDAYVAKFKLKPLYCRFVQSATFNTSLKKWAVEVKNTTTNKIDVYNAQFLIIASGENSEGFIPDLHGLDSFPGEIIHSSHYKSGDHFHGKRALVVGCGNSGMEIAYDLNNNGVDSSIVVKNPVHILNEELVYVGMKLLKFFPFGLVDRLVMFLARIKYGDLSKYGLRRPEKGPFILKARTGRSPVIDVGTIDKIKKGEIKVFPGLAKIQGNHVTFENGEEKDFDTMVFATGYRSTANEWLKDFYYVLNEEGMPKAKFPNHWKGENGLYCAGFSSRGLVGISMDADAIADDIFNNYMVENVKEL

>Hyque.05G023500|Hquercifolia_v1.1/1-397

MEETKVVIVGAGPSGLATAACLNLQSIPNMVLEREDCCASLWRKRAYDRLHLHLAKEFCSLPFLPHSPSSPTFMSKNEFIEYLDDYVSLFNINPLYYHSVESAFYHEEKKKWEIQFKNIVSGKIRVCFTEFLVVASGENSEGYIPMLHGLERFGGEVIHSNKYKSGLRYKGKDVLVVGCGNSGMEIAYDLSNFGAHTSIVVRSPFHVMSKEIVQIGMLLLKYLPIIEVDTLIILLANAKFGDLSKFGVHRPKEGPFYLKEVKGRSPVIDVGTVGKIRSREIKVVPAISSIKDNNVKFNNGIEQHFDTIIFATGYKSSAKSWLKDYNYVLTSEGQPKNSFPTHWKGENGLYCAGFSRRGLIGVSNDAIAIAADIKKSYPQCFHVLDPKLQGDNLQRES

>Eucgr.L00638|Egrandis_v2.0/1-387

MEEVVVLIVGAGPAGLAVSNCLSHLSIPNIVLEREDCSASLWRKRSYDRLTLHLAKEFCSLPYMPHASSSSTFIPRKCFIEYIDSYISRFGIEPRYCRSVEAASYLENEGKWKVEARNTSTQEKEVYGARFLVVATGENSEGFIPELPGLDGFEGKIIHSSEYKSGKDYENKEVLVVGCGNSGMEISYDLSNFGARTCIVIRNPFHVLNKEMIYIGMLLSKYVAMTIADVVTTFIGKLWYGDLTKYGIRRPTKGPFQLKVATGKTPVIDVGTVKKIQSGDIKVLPEIVRINGNDVMFENSVVKRFDGIIFATGYMSTANYWLKDYKYIMNKDGMPKYPWPHHWKGENNIYCAGFSKEGLAGIARDSVAIANDINEILDGIEGKIKVN

>Gohir.A11G056900|Ghirsutum_v3.1/1-383

MEEIVVLIVGAGPSGLATSACLSVHSIPHIILEKEDIYASLWKKRAYDRLKLHLAKEFCSLPFKPHSPDSPTYIPKDMFVDYLDDYVKTFNIQPKYQRHVESASYDEADGKWRIEAKNVLTGGVEVYVAEFLVVASGENSGKYIPELPGLDSFSGETLHSSEYKSGAKYENKEVLVVGCGNSGMEIAYDLSNYGVQTAIVIRNPVHVVTKETVRVGMIFSKYLPIFIVDIMAVLMSKILYGDLSNYGIRRPTKGPFYLKATAGRAPVIDVGTVAKIKSKEIKVVPAISSIDGKKVLFEDGAEREFDVIVFATGYRSVANNWLKDFKHVLNETGMPKNDFPHHWKGEKNLYCCGLSRRGLFGVSMDASAIADDIKKVVTEKMNK

>Gohir.D11G061000|Ghirsutum_v3.1/1-383

MEEIVVLIVGAGPSGLATSACLSVHSIPHIILEKEDIYASLWKKRAYDRLKLHLAKEFCSLPFKPHSPDSPTYIPKDMFVDYLDDYVKTFNIQPKYQRHVESASYDEADGKWRIEAKNVLTGGVEVYVAEFLVVASGENSGKYIPELPGLDSFSGETLHSSEYKSGAKYENKEVLVVGCGNSGMEIAYDLSNYGVQTAIVIRNPVHVVSKEIVRVGMIFSKYLPIFIVDIMAVLMSKILYGDLSKYGIRRPTKGPFYLKATAGRAPVIDVGTVAKIKSKEIKVVPAISSIDGKKVLFEDGAEREFDVIVFATGYRSVANNWLKDFKHVLNETGMPKNDFPHHWKGEKNLYCCGLSRRGLFGVSMDASAIADDIKKVVTEKLNK

>Glyma.06G152700|Gmax_Wm82.a6.v1/1-381

MEEVSVIIAGAGPSGLAISACLSQNSISHIILEKDDCSASLWRKNAYDRLKLHLASEFCALPLMPHSPSSPTFLSKDEFVQYIDSYIARFNINPLYCRFIEFAAYDEVENKWRVEAKNTLEGTREIYVAKFLVIATGENSEGYIPDVHGLESFEGEIMHSKYYKSGSKYESKEVLVVGCGNSGMEIAYDLNDWGANTSILIRNPVHVFTKELINEGMRMMKHLPIHVVDTIITSLANMEYGDLSKYGIYQPKKGPFQLKFITGRAPVIDVGTIRRIKEGAIKVIPSHIVRIENKKVIFGNDVEKKFDVIVFATGYISVANKWLKDYKYILNDEGMPKNDFPNHWKGDRGLYCAGLSNRGLFGVKMDAEAIADDINQTLKLH

>Glyma.04G213600|Gmax_Wm82.a6.v1/1-381

MEEVSVIIVGAGPSGLAISACLSQNFISHIILEKEDCSASLWRKNAYDRLKLHLASEFCVLPLMPHPPSAPTYLSKDEFVQYIDSYIACFNINPLYCRMVEFAAYDEVENKWRVEAKKTLEGTSETYVAKFLVIATGENSEGYIPDVPGLESFEGEIVHSKYYKSGSKYETKEVLVVGCGNSGMEIAYDLNDWGANTSILIRNPVHVFTKELINEGMRMLKHLPVHVVDNIITSLANMEYGDLSKYGIYQPKKGPFHLKFITGRAPVIDVGTIEKIKEGAIKVIPSHIVRIENKKVIFENDAEKEFDVIVFATGYRSVANKWLKDYKYVLNDEGMPNNDFPNHWKGDRGLYCAGLSNRGLFGVKMDVEAIADDINQTLKLH

>KAI5381574.1|Pisum_sativum/1-380

MQEFTVVIVGGGPSGLAISALLTQNSILHVILEKEECNASLWRKNAYDRLNLHLASEFCSLPLMPHPSSGPTYLTKHQFLQYIDKYVDHFDIKPRYYCVVESAKYDEVRNRWVVEATNTLEGTLEVYETKFLVIASGENSEGFIPNVPGLGKFEGDVVHSKYYKSGSKYKSKNVLVVGCGNSGMEIAYDLHNGGANTSIVIRNPLHVFTKDMIRTGMCLVQYFPVYIVDTIITLQAKFKYGDMSKYGIYRPKDGPLYLKNTTGKSAVIDVGTIEKIKEGAIKVVPSGIKKIEKKNIIFENNMEKEFDAIVFATGYKSVANEWLKDYKYGLNEKGFPKNPFPKHWKGDHGLYCAGLARKGLFGVKKDAEAIAEDINQILKL

>KAI5410872.1|Pisum_sativum/1-382

MQEFTVVIVGGGPSGLAISALLTQNSILHVILEKEECNASLWRKNTYDRLNLHLASEFCSLPLMPHPPSGPTYLTKDQFLQYIDKYVNHFDIKPRYCRAVESAKYDEVMNKWVVEATNTLEGTLEVYGAKFLVIASGENSESFIPNVYGLGKFEGDVVHSKYYKSGSKYKSKNVLVVGCGNSGMEIAYDLHNGGANTSIVIRNPFHVFNRDMIRTGMWLVQYFPVYIVDIIITLQAKFKYGDMSKYGIYRPKDGPLYLKNTIGKSAVIDVGTIEKIKEGAIKVVPSGIKEIEKKNVIFENNVEKEFDAIIFATGYKSVASEWVKDYKYALNEKGFPKNPFPKHWKGDRGLYCAGLARKGLFGVKKDAEAIAEDINQILMLKN

>KAI5405034.1|Pisum_sativum/1-382

MKEFTVVIVGGGPSGLAISALLTQNSISHTILEKEDCNASLWRKNAYDRLNLHLASEFCSLPLMPHPPSGPTYLTKDQFLQYIDKYVNHFDLKPRYYCVVESAKYNEVKNKWVVEVKNTLEGILEVYGAKFLVIASGENSEGFIPNVHGLGKFEGDVVHSKYYKSGSKYKSKDVLVVGCGNSGMEIAYDLQNWGANTSIVIRNPLHVFTRNMIRIGMRLVQHFPVYIADTIITILAKLKYGDLSKYGIYRPKDGPLYLKNITGKSAVIDVGTIEKIKEGSIKVVPSGIKKIEKKNVIFENNMEKEFDAIVFATDYKSVANEWLKDYKYALNEKGFPKNPFPKHWKGDHGLYCAGLARKGLFGVKKDAEAIAEDINQTLKLIK

>KAI5405041.1|Pisum_sativum/1-381

MQEFTVVIVGGGPSGLAISALLTQNSISHTILEKEDCNASLWRKNAYDRLNLHLASEFCSLPLMPHPPSGPTYLTKDQFLQYIDKYVNHFDIKPRYYCVVESAKYDEVKNKWVVEAKNTLEGTLEIYGAKFLVIASGENSEGFIPNFHGLGKFEGDVVHSKYYKSGSKYKSKDVLVVGCGNSGMEIAYDLQNWGANTSIVIRNPLHVFTRNMIRIGMRLVHYFPVYIADTIITILAKLKYGDLSKYGIYRPKDGPLYLKNITGKSAVIDVGTIEKIKEGSIKVVPSGIKKIEKKNVIFENNMEKEFDAIVFATGYKSVANEWLKDYTYALNEKGFPKNPFPKHWKGDHGLYCAGLARKGLFGVKKDAEAIAEDIKQTLKFN

>KAI5405035.1|Pisum_sativum/1-367

MQEFTVVIVGGGPSGLAISALLIQNSISHTILEKEDCNASLWRKNAYDRLNLHLASEFCSLPLVPHPPSGPTYLTKDQFLQYIDEYVDHFDIKPRYYRVVESANYDNVQNKWVVEAKNTLEGTLEVYGAKFLVIASGENSEGFIPNVHGLGKFEGEVVHSKYYKSGSKYKSKDVLVVGCGNSGMEIAYDLHNWGANTSIVIRNPLHVFTRDMIRIGMRLVQYFPVYIADTIITILAKLKYGDLSKYGIHRPKDGPLYLKNSTGKSAVIDVGTIEKIMEGSIKVVPSGIKKIEKKNVIFENNMEKEFDAIVFATGYKSVANEWLKDYKYALNEKGFPKNPFPKHWKGDHGLYCAGLARKGFVWSKKGC

>KAI5408364.1|Pisum_sativum/1-382

MEESTVVIVGGGPSGLAISALLTQNSISHILLEKEDCNASLWRKNAYDRLNLHLASEFCSLPLMSHPPSGPTYLTKDQFLQYIDKYVDHFVIKPRYYRVVESAMYDEVRNKWIIEAKNTFEGTLEVYGAKFLVIASGENSEDFIPNVPGLRKFKGEVVHSKNYKSGLKYKSKDVLVVGCGNSGMEIAYDLHNWGANTSIVIRNPVHVFTRDMIRLGMHLVQYFPVYIADTIITFLAKLKYGDLSKYGIHRPKDGPLYLKNIIGKSAVIDVGTVEKIKEGAIKVVPSGIKEIKKKNVMFENNMEKEFDAIVFATGYKSVANGWLKDYQYALNEKGFPKNPFPKHWKGDNGLYCAGLARKGLFGVKKDAEAIAEDINRTLKLEN

>KAI5408365.1|Pisum_sativum/1-192

MEIAYDLHNCGANTSIVIRNPVHVFTRDMIRLGMYIVQYFTVYIADTIITFLAKLKYDVLSKYGIHRPKYGPLYLKNIIGKSAVIDVGTVEKIKEGAIKVVPSGIKEIKKKNVMFENNMEKEFDAIVFATGYKSVANGWLKDYQYTLNEKGFPKNPFLKHWKGDNGLYCAGLARKGLFGVKKDAEAIAEDIN

>Qrob_P0171700.2|Quercus_robur/1-402

METKVLIVGAGPSGLATSACLTRHSIPHLILEREDCYASLWKKRTYDRLGLHLAKEFCFLPHKPLPPDAPTYMPKDFFLKYIDDYVSQFNIKPLYHRTVESASLIEGGKKWRIEAKNTLEGNVEVCHAEFLVVASGENNEGYIPDIKGLSSFPGEILHSNHFKSGSRHKSKDVLVIGCGNSGMEIAYDLEDHGARTSIVIRSPFHVLTKELVHRGMSLLNYLPTYMVDTLITMLARLEYGDLTKFGIYRPPRGPFSNKNVTGRSPVIDVGTIGKIQNEEIKVVPEILNIEKNNVKFKNGTEKNFDAIVLATGYKSAANKWLKDYKYALNGDGMPKNRVPEHWRGEKGLYCAGLSRRGLFGVSMDAEAIANDINKVITSQKKSLIHPEDTTNDFFSLTSPISI

>Qrob_P0171710.2|Quercus_robur/1-380

METKVLIVGAGPSGLATSACLTRHSIPHLILEREDCYASLWKKRTYDRLGLHLAKEFCFLPHKPLPPDAPTFMPKDFFLKYVDDYVSQFNINPLYHRIVESASLVEAGKKWRIEAKNTLEGNVEVYHADFLVVASGENNEGYIPDIKGLSSFPGEIVHSNHFKSGSRYKSKDVLVIGCGNSGMEIAYDLKDHGARTSIVIRSPFHVLTKELVHRGMSLLNYLPVYMVDTLITMLARFKYGDLTKYGIYRPPRGPFAHKNVTGRSPVIDVGTIGKIQNGEIEVVPEILNIEKNNVKFKNGTEKNFDAIVLATGYKSAANKWLKDYKYALNGDGMPKNRMPEHWRGEKGLYCAGLSRRGLFGVSMDAMAIANDIDKVITSKK

>Qrob_P0171720.2|Quercus_robur/1-380

METKVLIVGAGPSGLATSACLTCHSIPHLILEREDCYASLWKKRTYDRLGLHLAKEFCFLPHKPLPPDAPTFMPKDFFLKYVDDYVSQFNINPLYHRTVESASLVEAGKKWRIEAKNKLEGNVEVYHAEFLVVASGENNEGYIPDIKGLSSFPGEIVHSNHFKSGSRYKSKDVLVIGCGNSGMEIAYDLKDHGARTSIVIRSPFHVLTKELVHRGMSLLNYLPVYMVDTLITMLARLKYGDLTKYGIYRPPRGPFAHKNVTGRSPVIDVGTIGKIQNGEIEVVSEILNIEKNNVKFKNGTEKNFDAIVLATGYKSAANKWLKDYKYALNGDGMPKNRMPNHWKGEKGLYCAGLSRRGLFGVSMDAEAIANDINKVITGQK

>Cucsa.249910|Csativus_v1.0/1-386

MEEVRVLIVGAGPSGLATSAYLNHLSIPNIVLEKEDCYASLWKKRAYDRLCLHLAKEFCSLPLMPHSSSTPTFMSRATFLKYLDEYVSKFNIKPRYSRNVERAWLEDEEDGEMKKWRVEARHIETGEMEAYKAEFLVVASGENSVGHVPEVTGLDTFEGEIVHSSKYKSGKAFEGKDVLVVGCGNSGMEIALDLSNYGAHPSIIIRNPLHVLKREVVYVGMVLMKYLPVSVVDGILVGLSKLKFGDMSAYGICRPKLGPMQLKYATGKTPVIDVGTISKIQDGQIKVVPQISNIDGETIEFENGVRKKFDAIVFATGYRSSANNWLQDYELVLNEKGMPKSGIPNHWKGKKNVYCVGLSRQGLAGVSFDAKAVAQDISNNISNKFT

>AUR62022873|Cquinoa_v1.0/1-383

MEETLVLVVGAGPAGLGIAACLTKKSIPYIVLEKEDCYASLWKKRAYDRCHLHLAKEFCSLPYKPHAPHAKKYMSKNDFIEYIDNYVSDFDIRPQYFHSVEFASFDEVKGKWLVEAKDTLMKVTKVFVASFLVVATGENGKGYIPNIPGLKDFKGGIMHSSEYKNGREFQDKNVLVVGCSNSGMEISYDLCNSGANTSIVIRNPVHVVTREMIFVGMHLLKYFSLSIVDALVTFASRLTYENLSKYGICRPNEGPFLLKATKGRSPVLDVGTIAEIQSRKIKVLPGIKRINESKIIFEDKVELNFDAIIFATGYKSTSCEWLKDYDYILNKDGFPKNRFPTHWKGNTNVYCAGLSRMGLQGISKDVIAIANDIEMVLRAKLCS

>AUR62012227|Cquinoa_v1.0/1-383

MEETFVLIVGAGPAGLGIAACLTKKFIPYIVLEKEDCCASLWKKRAYDRCHLHLAKEFCSLPHKPHAPQAKKYMSKNDFIEYIDDYVLDFDIRPRYFHSVESASFDEAKEKWLVEAKDIFANASKFFVASFLVVATGENGKGYIPNIPGSNDFKGDIIHSSEYKSGREFQDKNVLVVGCGNSGMEISYDLCNSGANTSIIIRNPVHVVAREMIFAGMHLLKYFSLPIVDALVTLASTLKYGNLSKYGIYRPNEGPFLLKATKGRSPVIDVGTIAKIQSGEIKVLTGIERINKSKVIFEDKVELSFDAIIFATGFKSMACEWLKDYDFILKNDGFPKNHFPNHWKGENKLYCAGLSRMGLQGVSKDAIVIANDIEMVLRTMLRN

>AUR62006006|Cquinoa_v1.0/1-383

MEETLVLVVGAGPAGLGIAACLTKKSIPYILLEKEDCCASLWKKRAYDRCHLHLAKEFCSLPFKPHAPQANKYMGKDDFIEYIDDYVLDFDIRPRYYHSVESASFDEATGKWLVKARDILVKATKVFVASFLVVATGENGKSYIPDISGLKDYKGDVMHSSEYKCGREFQDKDVLVVGCGNSGMEISYDLCNSGAKTSIVIRNPVHVVAREMIFVGMHLLKYIPVSTVDAIVTFASKLKYGNLSNYEIHRPNEGPFFLKATKGRSPVLDVGTVAKIQSGDIKVLQGISRINKTKVIFEDDVELAFDAIIFATGFKSTACDWLKDYDYILNNNGFPKNRFPNHWKGKNKLYCAGLSRMGIQGASKDAIVITNDIEMVVRAMIAP

>AUR62035621|Cquinoa_v1.0/1-383

MEETLVLVVGAGPAGLGIAACLTKKSIPYILLEKEDCCASLWKKRAYNRCHLHLAKEFCSLPFKPHAPQANKYMSKDDFIEYIDDYILDFDIRPRYYHSVESASFDEATGKWLVEARDILVKATKVFVASFLVVATGENGKSYIPDIPGLKYFKGDVMHSSEYKSGREFQGKDVLVVGCGNSGMEISYDLCNLGANTSIVVRNPVHVVAREMIFVGMHLLKYIPLSMVDAIVTFASKLKYGNLSKYGIHRPNEGPFFLKATKGRSPVLDVGTIAKIQSADIKVLPGISRINKTKVIFEDDVELAFDAIIFATGFKSTACDWLKDYDYILKNDGFPKNRFPNHWKGKNKLYCAGLSRMGIQGASKDAIAITNDIEMVVRAMIAS

>Sapof.03G166600|Sofficinalis_v1.1/1-383

MEDVLVMIVGAGPAGLALAACLTVMTIPFILLEKEDCHNSLWKKRAYDRCHLHLAKEFCSLPHRPHPRQAKKYMPKNDFINYVDDYVTHFGIKPRYNHVVEHAAFDEIQGKWEIRALDMSCNVTQVFISSFLVVATGENGKSVIPDIPGVKDFEGDLVHSSQYKSGLEYQDKDVLVVGCGNSGMEIGYDLANYGARTSIVVRNSFHVVSKEMIWVGMHLLKYLSIWVVDILVLMAVKIKYGDLSKYGIHRPEEGPFFIKATKGRSPVIDVGTVAKIRSGKIKVCPGISRINKNSVVFENEVESHFDAIVFATGFRSTVCEWLKDYEYLMKDDGFPKNRFPNHWKGSKRLYCAGLARMGLQGLSRDAQAIAADIKSTLQSTEFQ

>Cucsa.172940|Csativus_v1.0/1-386

MEETQVIVVGAGPAGLATSACLNRLSIQNIVLERDDCSASLWRKRAYDRLKLHLAKNYCNLPYMPFPDNAPTYISRVDFIKYLDEYMSSFGIQPRCCRTVEEAWYEKEEERWKVVVENTSSGEQERYVCKFLVAATGENCEGFLPNIPGLESFNGEVLHSSGYDNGQRFRGKDVLVVGCGNSGMEIAYDLSNHAANTSIVVRSPVHVLTKDIVRLGMFLLKYFPCNVVDSISINLAKLKYGDYSKYGIQRPRAGGPFLIKSKTGRSPTIDVGCMKRIRTGEVKVFPSITCIKRDQVRFAYGIVNCFDAIIFATGYKSTVINWLQDEKNHFNENGMPRERFPNHWKGENGLYCAGFGQQGLFGISNDAKKIATDISLALGLNNTTVE

>orange1.1g035902m.g|Csinensis_v1.1/1-381

MEEVPVVIVGAGPAGLATSACLNNLSVPNIILEREDCSASLWKKRAYDRMKLHLAKQFCELPHMPFPSRTPTFVPRISFINYVDNYVSQMGINPRYHRSVESASYDENAKAWIIVAKNTALDAYEEYVARYLVVATGENGLIPEVPGLGSFEGEYMHSSKYENGGKFIGKNVLVVGCGNSGMEIAYDLSSCGACTSIVVRGPVHVLTREIVFAGMLLLKFLPCKLVDFIVVMLSKMKFGNLFKYGLERPKKGPFYFKAITGQTPTIDVGAMDKIRKGEIQVFPSITSINRNEVEFENGKIEEFEAIIFATGYKSTVRNWLKRADKDFFDEYGMPKRNCPNHWKGENGLYCAGFSRTGLHGISIDAKNIANDINLALTDHQV

>Potri.005G186100|Ptrichocarpa_v4.1/1-381

MEEVVVIIGAGPAGLASSACLNRLDIPNIILEREGCYASLWRKKAYDRVKLHLAKQFCELPYMSYPPKLPMFVPKNDFISYLDNYASHFGINPRFHCYVESVYYDKNASKWCIVVRNNKLNTTEVYIAKFLVVATGENSEGLIPKVPGLDGFEGMYMHSSQYANGKDFNGKDVLVVGCGNSGMEIAYDLLYWGAHTSIVARSPVHVISKEIVFLGMCFLKYLPCRLVDFIATTLSKIKFGDTSKYGIQRPTEGPFYIKAKTGRSPTIDVGAVQKIKTGEIQVFPSIANIEGTEITFENGKSKQYDAIIFATGYRSTVLDWLKDGKDLFNENGMPKLRFPNHWKGGNGLYCAGFSRSGLMGISLDAQQIATDIGLAWKGASK

>Prupe.8G014100|Ppersica_v2.1/1-383

MEQVDVVIVGAGPAGIATSACLNRLNISNVVLEREDCYASLWKKRSYDRLKLHLAKQFCELPHMPFPPNAPKYVPKDQFVEYLDSYVSHFKIQPLYHRVVETAFYDADVEKWHVIVKNTSLDAQEIYLGKFLVVASGENSEGYIPQVQGLDSFGGEFMHSSKYENGKKYSGKNVLVVGCGNSGMEIAYDLSNSGANTSIVVRSPIHVLTKEIVFLGMVMAKYIPINLVDNVMVILSKLRFGDLSKYGIRRPKSGPFFLKEKGQAPIIDVGSVNKIRTGELKVVPSITSIQGDNIEFENGCKDRFDAIVFATGYKSTVLNWLKDGHNLFNDDGMPKQSFPNNWKGKNGLYCAGFSRRGLFGISHDARMIISDISFALDQNKKKS

>VIT_218s0001g15100|Vvinifera_v2.1/1-379

MEEVVIIVGAGPSGLATSACLNVLSIPNIILEREDCFASLWKKRSYDRLKLHLGKQFCQLPHMPYPPGTPTFIPKAGFLQYLEDYVSHFQINPRYHRFVESASYDKVVGKWHIVAKNTLSDELEVYLGKFLVVATGENSEGLIPKIPGLDSFGGEFMHCSDYKNGKRFTNKEVLVVGCGNSGMEIAYDLWDHGAITSIVVRNPVHVVTKEMVLLGMLLLKYIPCKVVDYVTVSLSKLIYGDLSSYGLPRPSEGPFYLKDVTRSSPVIDVGTIGKIKEGEIQVVPTITKIEGDNVYFSNGKMNRFDAIIFATGYKSTVLKWLKESEDLFNEDGMPKKSFPNHWNGENGLYCVGFASRGLFGIARDAEHIANHIRGVMSRK

>VIT_218s0001g15190|Vvinifera_v2.1/1-379

MEEVVIIVGAGPSGLATSACLNVLSIPNIILEREDFFASLWKKRSYDRLKLHLGKQFCQLPHMPSPPGTPTFIPKARFLRYLEDYVSHFQINPRYHRLVESASYDKVAAKWHIVAKNTLSDESEVYLGKFLVVATGENSEGLIPKIPGLDSFGGEFMHCSKYKNGKRFADKEVLVVGCGNSGMEIAYDLWDRGAKTCIVVRSPKHVVTKEMVLLGMFLLKYVPRKVVDYVIVSLAKLNYGDLSNYGLPRPKEGPFYLKDVTRSPPIVDVGTIGKIKEGEIQVVPAVTKIEGQYVYFSNGKMNQFDAIIFATGYKSTVLKWLQDDENLFNEDGMPKKNFPNHWNGENGLYCVGFASRGLFGIARDAEHIANHINGAVRGK

>Potri.005G111800|Ptrichocarpa_v4.1/1-393

MEAVDVVIVGAGPAGLATSACLNRLSIPNIVLEREDCYASLWQKRAYDRLKLHLAKEYCELPFMPFPSDAPTFVPRRGFIDYLHSYVSHFRINPRCNTAVESAYHDEESGKWHIKAKKADLNVHEEYVAKFLVVATGENSKGFIPEVSGLDSFGGEFIHSSKYENSQKYKGKAVLVVGCGNSGMEIAYDLSNWGVKTSIVARSPVHVLTTNIVYIGMRLLSYGVPCNIVDFIVVLLSKLQHGDISNYGFPRPTRGPFYIKQRVGRTPTIDVGAVEKIRRKEVQVFPSIRGIQGSKIEFANGEVKQFDAIIFATGYKSTVRHWLKGGQDLFDGSGMPKLCFPNIWKGKNGLYCSGFARRGLFGISVDSQNIAKDIDLALRSERVLIGIPDARVC

>evm.TU.Scaffold_622.354|Carabica_v0.5/1-171

MVRFGMKLLKFIPCDLVDSTVVMLSKFLHGNLAKYGLERPDKGPFLLKNMTGQSPVIDVGTLKMIKSGKIQVLPAVKRVDGDYVEFSNGIKKNFDAKVFATGYKSTVRTWLKDETSLFNEDGMPQKSRPNHWKGDDGLYCAGFSSSGLFGISKDAMNIAEDINFMPGKEKN

>evm.TU.Scaffold_628.378|Carabica_v0.5/1-171

MVRFGMKLLKFIPCDLVDSTAVMLSKFLHGNLAKYGLERPDKGPFLLKNMTGQSPVIDVGTLKMIKSGQIQVLPAVKRVDGDYVEFSNGIKKNFDAIVFATGYKSTVRTWLKDETSLFNEDGMPQKSRPNHWKGDDGLYCAGFSSSGLFGISKDAMNIAEDINFMPGKEKN

>evm.TU.Scaffold_628.384|Carabica_v0.5/1-234

MHSSHYDNGQNFCDRDVLVVGSGNSGMEIAYDLANWDAKTSIVIRSPVHVLTKEMVRFGMKLLKFIPCDLVDSTVVMLSKFLHGNLAKYGLERPDKGPFLLKNMTGQSPVIDVGTLKMIKSGKIQVLPAVKRVDGDYVEFSNGIKKNFDAIVFATGYKSTVRTWLKDGTSLFNEDGMPQKSRPNHWKGDDGLYCAGFSSSGLFGISKDAMNIAEDINFMPGKEKNLKLKYIEEI

>evm.TU.Scaffold_622.358|Carabica_v0.5/1-383

MNQETVVIIVGGGPAGLATSACLNVRGIPNIVLEREDCSASLWKKRTYGRLKLHLAKQFCQLPHMSFPSDAPTFVPKDGFIQYLDDYTSHFNINPIYCRLVESASFGVTDEKWLVLAKNALSGAIENYVAKFLVVATGENGQGLIPEIPGLDSFGGVAMHSSHYDNGQNFCDKDVLVVGSGNSGMEIAYDLADWDAKTSIVIRSPVHVLTKEMVRFGMQLLKFIPCDLVDRTVVMLNKFLHGNLAKYGLVRPDKGPFLLKNMTGQSPVIDVGTLKMIKSGKIQVLPAVKRVDGDYVEFSNGIKKNFDAIVFATGYKSTVRTWLKDETSLFNEDGMPQKSRPNHWKGDDGLYCAGFSSSGLFGISKDAMNIAEDINFMPGKEKN

>Gohir.D10G119500|Ghirsutum_v3.1/1-380

MVVIVGAGPAGLATSACLNRLSIPNIVLEREDCYASLWKKRSYDRLKLHLAKQFCHLPYMDFPPNAPTYVPKNGFIDYLDNYVSHFGITPRYLRSVESVIYDSDAEKWQIVVKNMTTTDNVIETEVYTSRFLVVASGENSQGTIPDIDGLDSYVGEYIHSNQYENGRKFRGKDVLVVGCGNSGMEIAYDLWNWGANTSIVVRNPVHVLTKEMVKMAMIMSQYLPCKAVDIITVAISRLKYGKLSKYGIRMPTKGPFHLKETTGRSPVIDVGTISKIKSGEIKVLPGMECIKDNEVVFTNGATVQFDAIVFATGYKSTVRNWLKGSYESFDEKGMPKRSFPYHWKGGNGIYNAGFSRRGLRGISSDAQNIANDIYINITAD

>Gohir.A10G147700|Ghirsutum_v3.1/1-389

MEETVVIIVGAGPAGLATSACLNRLSIPNIVLEREDCYASLWKKRSYDRLKLHLPKQFCHLPYMDFPPNAPTYVPRKGFIYYLDNYVSQFGITPRYLRSVESAIYDLDAEKWQIVVKNMATTDNVIETEVYTSRFLVVASGENSQGTIPDIDGLDSYGGEYIHSNQYENGRKFRGKDVLVVGCGNSGMEIAYDLWNWGANTSIVVRNPVHVLTKEMVKMAMIMLQYLPCKAVDKITVAISKLKYWKLSKYGIQRPKKGPFHLKETSGRSPVIDVGTISKIKSEEIKVLPGMKCIRDKDIVFTNGETVQFDAIIFATGYKSAVRNWLKGSYETFDEEGMLRKSFPYHWKGRNGIYNVGFSRRGLQGFSSDAQNIANDIYSNTTVALGFNN

>Rhdel07G0229200.1|Rhododendron_delavayi/1-384

MIQETEVIIIGAGPAGIATSACLNLLSLPNIVLERESCTASLWKNKSYDRLKLHLAKQYCQLPHMPFPSRFPTFLSKSLFINYLDTYVSWFGVNPMLGRSVESASHEDGENCWRVVARNAGSGETEVFEGRFLVVATGENSEGFVPEVVGLEDFGGEVVHSSEYGNGKRYEGKDVLVVGCGNSGMEIAYDLVNCGARAAISVRSPVHVLSKEMVQLGMCMLKLLPCDVVDKMVQSLAKLKYGDFSHYGLQTPSKGPFYLKRTTGRSPVIDVGTMHKIKTRDIQVLPSITSIKGDFIEFENGKTNRFQAIVFATGYKSTVRKWLKDDGELFNEDGMPKKRWPDHWKGENGLYCVGFARAGLLGISSDAQNIANDICLILSQQKNR

>Potri.016G003300|Ptrichocarpa_v4.1/1-383

MEEVAVIIVGAGPSGIATSACLNRLEIPNIVLEREGCCGSLWKKRAYDRLTLHIPKQYCELPYMSYPSNAPTFVPRNGFIAYLDEYLSHFGVTPRFNRSVGLAFYDVDAGKWRLEVTNVCSHVKEVYVAQFLVVATGENAEGVIPDIPGLGGFAGECMHASQFSNGRKYRGKDVLVVGCGNSGMEISYDLCQSNARTSIVNRSPVHVVTKEMVSLAMFLLKFLSVTSVDKILAKLCKLRFDDLSEYGIQRPKEGPFYLKTTQGRSPTIDVGCVDRIKQGKIKVFPSMANIEGKKIEFMNGESNQFDVIIFATGYRSTVGRWLKGGEELFDDNGFPKQDLVNKWKGSNGLYCVGFARNGLLAISSDAKNVSQDISTLILNCQIC

>AT1G48910|Athaliana_Araport11/1-383

METVVVIVGAGPAGLATSVCLNQHSIPNVILEKEDIYASLWKKRAYDRLKLHLAKEFCQLPFMPHGREVPTFMSKELFVNYLDAYVARFDINPRYNRTVKSSTFDESNNKWRVVAENTVTGETEVYWSEFLVVATGENGDGNIPMVEGIDTFGGEIMHSSEYKSGRDFKDKNVLVVGGGNSGMEISFDLCNFGANTTILIRTPRHVVTKEVIHLGMTLLKYAPVAMVDTLVTTMAKILYGDLSKYGLFRPKQGPFATKLFTGKAPVIDVGTVEKIRDGEIQVINGGIGSINGKTLTFENGHKQDFDAIVFATGYKSSVCNWLEDYEYVMKKDGFPKAPMPKHWKGEKNLYCAGFSRKGIAGGAEDAMSVADDIRSILATLKNN

>GWHPCBIR003446|Santalum_yasi/1-386

MAVEVSAVIVGAGPSGLATSACLNRHGISNLVLEREDCYASLWRNRTYDRLGLHLAREFCTLPYMEYPSGTPTYVPKKDFIAYLDAYVRVSHVSPKCSRSVESASYDKIERKWTVRALNSKTGAREVYSSEFLVVATGENGEGFVPRTPGLESFRGDVLHSSQYKSGLKYRNKKGLVVGCGNSGMEISYDLASYGCSTSIVVRSPFHFLSKGMVHLGMVLLNYLPVSVVDALVTFLSKIKFGDLSSHGVCRPKEGPFILKVKKGRSPVIDVGTIEKIRSGEIQVVPVISKIDENTAEFEDGTKEEFDVIVFATGYRSTATKWLKNYDYCLNKEGMPKNGFPLHWKGENGLYCAGLSRMGLAGISDDAKAIADHIKLEMDGSDLQIR

>CKAN_02337900|Ckanehirae_v3/1-379

MRIEVVIVGAGASGLATSACLNLHSIPNIVLEREDCSASLWKKKSYDCLKLHLPKQSCQLPHMPHPSTTPTYIPKDLFIQYLDNYESHFKVTPRCCRLVESASYDGGSWRVVAKNTVSGEIEEYDASFLVVASGETCDASVPKVPGLDGFKGSVLHSTEYKSGAEYGGKDVLVVGSGNSGMEIALDLCNHGARTSIVVRSPIHVLSGWMVTLGVLLSKLLPFHLLDSLVVMLSKLRYGDLTKYGIRRPEEGPFYLKQKQGKFPLIDVGTTNKIKSGEIQVLPALTAIRDRELEFANGELHYFDTIIFATGFRTSTDRWLKDRDNILDKDGFPKPSYPKHWKGNRGLYCVGLVRKGFYGLSMDAQCIADDIKRLSEGSTV

>evm_27.TU.AmTr_v1.0_scaffold01333.1|Atrichopoda_v1.0/1-391

MGMAMREQVVIIVGAGPSGLATSACLKNLCIPNVVIERDDCSASLWKKRCYDRLNLHLGKEFCELPHFPFPPDAPTFISKIDFIKYIDDYSTRFGVEPLFSREVIEATYDREVQRWILHVTNTCTKNVECYSGRFLVVATGENSEGIIPEIPGLPDFEGEVLHSSQFKSGRTHSGQNVLVVGCGNSGMEIALDLSIHGADASIVVRNPIHVMNKELIHMGMRLLRYFPLGLVDRLMVACSQWLYGNTSQFGIRRPEEGPFLLKRKTGRSPILDQGTFNKIKSGDIKVLPAIAHIRGKEVDFSNGEKKVFDAIILATGYKSAIKAWLKDEEEIFNEDGMPRAKYPNHWRGPNGLYCAGLSRRGLFGVSEDAMNIANDIYSLFRDGLNSERGL

>evm_27.TU.AmTr_v1.0_scaffold00209.9|Atrichopoda_v1.0/1-391

MGMAMREQVVIIVGAGPSGLATSACLKNLCIPNVVIERDDCSASLWKKRCYDRLNLHLGKEFCELPHFPFPPDAPTFISKIDFIKYIDDYSARFEVKPLFSREVTDATYDREVQRWIVHVNNTCTNNVECYSGRFLVVATGENSEGIVPEIPGLPEFEGEVLHSSLFKSGKDYSGKNVLVVGCGNSGMEIALDLSNHGAETSIVVRNSIHVMNKELIHMGMRLLRYFPMALVDRLMVSCSIWLYGDTSKFGIRRPEEGPFLLKCKTGRSPILDLGTIKKIKTGDIKVMNAIAYIRGNEVRFSNNEKQVFDAIILATGYKSTIKAWLKDEDGFFIEDGMPRAKYPNHWSGPNGLYCAGLSKRGLFGVSEDAMNIATDIYSLFKDGLKDKILL

>evm_27.TU.AmTr_v1.0_scaffold00017.294|Atrichopoda_v1.0/1-391

MGMAMREQVVIIVGAGPSGLATSACLKNLRIPNVVIERDDCSASLWKKRCYDRLNLHLGKEFCELPHFPFPPDAPTFISKIDFIKYIDDYSARFEVKPLFSREVTDATYDREVQRWIVHVNNTCTNNVECYSGRFLVVATGENSEGIVPEIPGLPEFEGEVLHSSLFKSGKDYSGKNVLVVRCGNSGMEIALDLSNHGAETSIVVRNSIHVMNKELIHMGMRLLRYFPMALVDRLMVFCSIWLYGDTSKFGIRRPEEGPFLLKCKTGRSPILDLGTIKKIKTGDIKVMNAIAYIRGNEVRFSNNEKPVFDAIILATGYKSTIKAWLKDEDGFFIQDGMPRAKYPNHWSGPNGLYCAGLSKRGLFGVSEDAMNIATDIYSLFKDGLKDKIVL

>Asa6G05468.1|Allium_sativum/1-381

MTIKEVMIVGAGPGGMATLACLNMLNILNILVEKKECYAHMWKKTAYDRLKLHLAKEFCFLPHMPHPYSAPTYIPKGQFVQYLDNYAKKFNINPQCNTSVESAAFDEVKEAWVVKTKNLETTKEEEVLAKFLVVASGENAQGIIPNIEGLKGFSGKVIHSSEFRTGKDFVGKRVLVVGCGNSGMEIAYDLCNHGALTSISIRSPLHVSTKELIHLAMFLLKYMPMSLVDKLTAILSYIYYGDLSKHGIVTPRKGPFALKFGGGRSPVIDVGTIQKIKSGEIQVVKEPVKINGDEVLFSDGTSHFFDAIVFATGYSSSVMNWLKDEEGKWLNKDGYPKNEFPNQWKGKNGLYFVGFAKRGLAAIASDSDNVANDIAKALGQA

>Asa6G05469.1|Allium_sativum/1-366

MATSACLNVLNIPNILVEKKECYAHMWKKTAYDRLKLHLAKEFCFLPHMPHPYSAPTYIPKNQFVQYLDNYAKKFNINPQCNTSVESAAFDEVKDAWVVKTKNLETSKEEEVFAKILVVASGENAQRIIPNIEGVKGFSGKVMHSSEFRTGKDFVGKRVLVVGCGNSGMEIAYDLCNHGALTSIAIRSPLHVSTKELIHLAMFLLKYMPMSLVDKLTAILSYIYYGDLSKHGIVTPRKGPFALKFGGGRSPVIDVGTIQKIKSGEIQVVKEPVKINGDEVLFSDGTSHFFDAIVFATGYSSSVMNWLKDKEGKWLNKDGYPKNEFPNQWKGKNGLYFVGFAKRGLAAIASDSDNVANDIAKAFGQA

>Asa8G05008.1|Allium_sativum/1-366

MATSACLNVLNIPNILVEKKECYAHMWKRTAYDRLKLHLAKEFCFLPHMPHPYSAPTYIPKGQFVQYLDNYAKKFNINPQCNTSVESATFDEVKEAWVVKTKNLETNKEEEVLAKFLVVASGENAQRIIPNIEGLKGFSGKVLHSSEFRSGKDFVGKRVLVVGCGNSGMEIAYDLCNHGALTSIAIRSPFHVSTKELIHLAMFLLKYLPMSLVDKLTAILSYIYYGDLSKYGIVTPRKGPFSLKFGGGRSPVIDVGTIQKIKSGEIQVVKEPIKINGDEVLFSDGTSHFFDAIVFATGYSSSVMNWLKDEEGKWLNKDGYPKNEFPNQWKGKNGLYFVGFAKRGLAAIASDSENIANDIAKALGQA

>Asa8G05276.1|Allium_sativum/1-366

MATSACLNVLNIPNILVEKKECYAHMWKKTAYDRLKLHLAKEFCFLPHMPHPYSAPTYIPKGQFVQYLDNYAKKFNINPQCNTIVESAAFDEVKEAWVVKTKNLETTKEEEVLAKFLVVASGENAQGIIPNVEGLKGFGGKVLHSSEFRTGKDFVGKRVLVVGCGNSGMEIAYDLCNHGASTSIAIRSPLHVSTKELIHLAMFLLKYLPMSLVDKLTAILSYICYGDLSKYGIITPRKGPFALKFDSGRSPVIDVGTVQKIKSGEIQVVKEPIKINGDEVLFSDGTSHFFDAIVFATGYSSSVMNWLKDDEGKWLNKDGYPKNEFPNQWKGKNGLYFVGFAKRGLAAIASDSENAANDIAKALGQA

>Asa8G05270.1|Allium_sativum/1-375

MIVRAGPGGMATLACLNMLNILNILVEKKECYAHMWKKTAYDRLKLHLAKEFCFLPHMPHLYSAPTYIPKGQFVQYLDNYAKKFNINPQCNTIVESAAFDEVKEAWVVKTKNLETTKEEEVLAKFLVVASGENAQGIIPNIEGLKGFSGKVLHSSEFRTGKDFFDKRVLVVGCGNSGMEIAYDLCNHGALTSIAIRSPLHVSTKELIHLAMFLLKYLPMSLVDKLTAILSYICYGDLSKYGIITPRKGPFALKFGGGRSPVIDVGTVQKIKSGEIQVVKEPIKINGDEVLFSDGTSHFFDAIVFATGYSSSVMNWLKDEEGKWLNKDGYPKNEFPNQWKRKNGLYFIGFAKRGLAAIASDSENVSNDIAKSLGQA

>Asa6G06224.1|Allium_sativum/1-363

MATSACLNVLNIPNILVEKKECYAHMWKKTAYDRLKLHLAKEFCFLPHMPHPYSAPTYIPKDQFVQYLDNYAKTFNINPQCNTSVESAAFDEVKGGWVVKTKNLETGKEEEVLAKFLVVASGENAQGIIPYIEGLKGFSGKVIHSSEFRTGKEFVGKRVLVVGCGNSGMEIAYDLCNHGALTSIAIRSPLHVSTKELIHLAMFLLKYMPMSLVDKLTAMLSYIYYGDLSKYGIVTPRKGPFSLKFGGGRSPVIDVGTIQKIKSGEIQVVKEPVKINGDEVLFSDGTSHFFDAIVFATGYSSSVMNWLKDDEGKWLNKDGYPKNEFPNQWKGKNGLYFVGFAKRGLAAIASDSENAANDIAKAL

>Asa1G03432.1|Allium_sativum/1-381

MTIKEVMIVGAGPGGLATSACLNVLHIPNILVEKKECYAHMWKKTAYDRLKLHLAKEFCFLPHMPHPYSTPTYIPKDQFVQYLDNYVKKFNIKPQCNTNVEYAAFDEENKVWVVKTKNLETNEEEEVLAKFLVVATGENAQGIIPNIEGLKGFSGEVLHSSQYRTGKDFVGKRVLVVGCGNSGMEIAYDLCNHGALTSIAIRNPLHVSTKELIHLAMFLLQYFPMSFVDKLTTTLSYIYYGDLSKYGIVTPSKGPFALKFDGGRPPVIDVGTVQKIKSGEIQVVKEPIKINGNEVLFSDGTSHFFDAIILATGYTSSVMNWLKDEEGRWLDKDGYPKNEFPNQWKGKNGLYFVGFAKRGLAAIASDSVNTANDIASALGQA

>Soltu.DM.09G028920|Stuberosum_v6.1/1-220

MPHTTSTPKYLSKKEFIQYLDKYVEHFNIKPKYQTCVELAFFNSEEGKWNVKSRDLASGDLEVYACNFLILATGENNEGYIPKVVGIENFEGEIIHSSDYKSGQKYEGKNVLVVGSGNSGMEIAFDLSNYGSHVSVVVRSPIHVLTREMVYTAMLMLKYLPVSLVDTVIAKYAKFKFGNLAELGIPQPEEGPFSVKISKGRSPVIDVGAIDKIKLGQIKV

>Solyc09G002716|Slycopersicum_ITAG5.0/1-381

MVIFSKQEIEVVVVGAGPSGIAVSACLNKFGIKNVVLEKEDCCAYLWKKKTYDRLHLHLAKGFCSLPFMSYTTSTPKYLSKNEFIQYLDKYVEYFDVKPKYHTCVEWAYFNNEESKWNVKSRDLSSGDMKVYVCDFLILATGENNEGYIPKVVGMENFKGEIVHSSDYKFGRKYEGKNVLVIGSGNSGMEIAFDLSNYGCHASIVVRSPIHILTREMVYTAMLMLKYLPVSWVDAVIAKYAKFKFGNLAKLGISQPKEGPFSVKISKGRSPVIDVGAIDKIKLGQIKVLPGISKIKEQTVVFDNGDEHQFDTIIFATGYKNVATKWLKDYSSIFLEDGTLKNWKGQNGLYAAGFSKRGIAGISMDAIAIADSIKSVRGDKI

>Soltu.DM.09G013380|Stuberosum_v6.1/1-137

MVGIENFKGEIIHSSDYRSGEKYKDKKVLVVGSGNSGMEIAFDLSSYEGLKSIVVRSPIHVLTREMVYTATLLLIYLPVSSVDTVIPKYTKFKFGNLEELGIPQPEEGPFSVKISNGRSPVIDVGAIDKIKLGQIKV

>Soltu.DM.09G028780|Stuberosum_v6.1/1-288

MANFTKEEIDIVVVGAGPSGIAVSACLNKVGINNVVLEKQDCCAYLWKKKTYDRLHLHLSKDFCSLPFMPHASSSPKYMPKKEFIQYLDEYVEHFNIKPKFQTCVESAFYNNGEKKWNVKSRNLTSGEIELYASDFLILATGENNEGYIPKMVGIENFKGKIIHSSDYRSGEKYKDKKVLIIGSGNSGMEIAFDLSNYESHTSIVVRSPIHVLTREMVYTAMLLLKYLPISLVDTVIAKYANFKFGNLAELGIPQPEEGPFSIKISKGRSPVIDVGAIDKIKLGLIKV

>Soltu.DM.07G009360|Stuberosum_v6.1/1-158

MYASDFLILAAGENNESYIPKMIVIENFKGETIHSSDYRYGEKYKDKKVLVVGSGNSGMEIAFDLSNCESHRSIVVGSPIHVLTREIVYTAMLVLKYLPISLVDTVIAKYAKFMFGNLAEFGIPQTEEGPFFKKISKGRSPVIDVGAIDKIKLGQIKV

>Soltu.DM.07G009370|Stuberosum_v6.1/1-220

MPHATSSTKYMPKKEFIQYLDEYVEHLNIKPKFQTCVESAFYYSGEMKWTVKSRNLTSGKIEIYASDFLILATVENNEGYIPNMIGIENFKGEIIHSSDYRSGEKYKDKKVLVVGSGNSGMEIAFDLSNYESQTSFFVRSRIHVLTKDMVYTAMLLPKYLPISLVDTVTTKCTKFKFGNFEELGIPQPEEGPFSVKRSKGRSTVIDVGVIDNIKLGQIKV

>Glyma.06G120700|Gmax_Wm82.a6.v1/1-400

MEIEVPVVIVGAGPAGLATAACLNKYSIPNVVLERDDCHASLWRKRAYDRLKLHLGKDFCNLPHMPFPPDFPTFVPRVDFLRYLDNYVTRFKISIRYNRNVESASMDEQNNGKWRVVVKDTTTNADEVYVANYLVVATGENSEGYVPQIEGLEGFEGKHMHCSEYLNGRDLYGKHVLVVGCGNSGMEIAYDLSNWGANTSIVVRGPVHYFTKEMVYVGMSLLKYFKIEKVDKLMLLMSKLKYGDMSNYGLIRPKDGPFFLKKKGGTTPTIDVGCVSRIKKGEVKVFPAISSIKEDKLIEFEDGQNGQFDVIIFATGYNSTVLKWLKDYRGLFNENGMPKPDFPNHWKGENGIYCAGFSRRGLDGIAFDAKRIAADIKKTVNIKNLPSEEDNFASFKLLEK

>Glyma.04G242500|Gmax_Wm82.a6.v1/1-434

MNSQAYKSSTQQREENHQSHCITNLKPKHQNQTMEIEVPVVIVGAGPAGLATAACLNKYSIPNVVLERHDCHASLWRKRTYDRLKLHLGKDFCNLPHMPFPLDFPTFVPRVDFLRYLDNYVTRFKISIRYTRNVESASVDEENNGKWRVVVKDTTTNADEVYVADYLVVATGENDEGYVPQIEGLEGFEGEHMHCSQYLNGRHLYGKNVLVVGSGNSGMEIAYDLSTWGANTSIVIRGPVHYFTKEMVFVGMSLLKYFKMEKVDKLMLLMSKLKYGDMSEYGLVRPKDGPFFLKIKGGTTPTIDVGCVSRIKKGEVKVFPAISSIKKDKMVEFEDGQNGQFDVIIFATGYNSTVLKWLKDYRGLFNENGMPKPSFPNHWKGNNGIYCAGFSRRGLDGIAFDAQRIADDIKKTLNARNLPGDEEDNFASLKLLGK

>KAI5405794.1|Pisum_sativum/1-400

MEIQMPVVIVGAGPAGLATSACLNKLSIQNIVLERDDCHSSLWRKRTYDRLKLHLGKGFCNLPHMPFSSDLPVFIPRVDFLRYLDDYVSTFKIFIRYNRYVHEASFDVKTGKWRVCVMDSALNVGEVYVADYLVVASGESCDAYIPKITGLDKFEGEFFHCTKYQNGRPFYDKNVLVVGSGNSGMEIGYDLSTWGANTSMVIRSPVHFLIKEMVYIGMSLLKYVSVENVDKLMLVMSKLLYGDLSKYGLIRPKEGPFALKLKGGRTPTVDVGTIKHIKEGKIKVHSEISSIKNGKTIEFVDGKTGQFDVIIFATGYRTNVHKWLKDYKDLFNENGMPKPAYPNHWKGENGIYCVGFSKRGLQGINYDAQKVARDISVTINARKKILTADEANDAQIKLLD

>Eucgr.F02758|Egrandis_v2.0/1-387

MEKANEKGVVVAIVGAGPAGLAAAACLNRHGVANVVLEREDCSASLWRNRAYDRLQLHLGKQFCELPHMPFPSNSPTFIPRNQFIEYLDNYKSQFEVKLYCRRSVESASYDDQTGKWRIVARNTGSGEREVYVAEFLVVATGENSEGVMPEVDGLESFGGEVMHSNKYRNGSGFRGKNVLVVGCGNSGMEIAYDLWNHGANTSIVARSPVEVFTKEIVFIGMVLIKFLPRRIVDILMRLLSQLKYGDFAKYGLRRPKHGAFRVKAATGRTPTIDVGAMERIKDGEIKVFPSIKSIHGELVTFVNNTRSQFEAIIFATGYRSNVRKWLQGGDDLFNFQGMTKVRDQWKGENGLYSVGFSQWGLFGIANDARNVANDINFELQRRRKSE

>Eucgr.B02006|Egrandis_v2.0/1-390

MEKANEKEVVVAIVGAGPAGLAAAACLNRQGVANVVLEREDCSASLWRNRAYDRLKLHLGKQFCELPHMPFPSNSPTFIPRAQFIEYLDNYKSQFEVKLYCQRSVESASYDDQTGKWRIVARNTGSGEHEVYVAEFLVVATGENSEGMTPEVDGLESFGGEVMHSSKYRNGRGFRGKNVLVVGCGNSGMEIAYDLWNHGANTSIVARSPVEVFTKEIVFIGMVLIKFLPRRIVDILMRLLSKLKYGDFTKYGLQRPKNGALHLKAATGRSPTIDVGAMERIKDGEIKVFPSIKSIHGKLVTFVNDTTSQFEAIIFATGYKSNVRKWLQGGDDLFNVQGMTKVRDQWKGENGLYSVGFSQWGLFGIGSDARNVANDINFELQHQRKSKWEK

>Eucgr.B02005|Egrandis_v2.0/1-387

MEKGNEKEVVVAIVGAGPAGLAAAACLNRQGVANVVLEREDCSASLWRNRAYDRLKLHLGKQFCELPHMSFPSNSSTFVPRAQFIEYLDNYKSQFEVKLYCQRSVERASYDDQTGKWRIVARNTGSGEHEVYVAEFLVVATGENSEGMIPEVDGLESFGREVMHSSKYRNGRGFRGKNVLVVGCGNSGMEIAYDLWNHGANTSIVARSTVDVFTKEMVFIGMVLIKFLPRRIVDILMRLLSKLKYGDFTKYGLRRPKNGAFHLKATTGRSPTIDVGAMERIKDGEIKVFPSIKRIHGKLVTFVNDTTSQFEAIIFATGYKSNVRKWLQGGDDLFNVQGMTKVRDQWKGENGLYSVGFSQRGLLGIASDAQNVANDINFELRHRRKSK

>Nycol.A00015|Ncolorata_v1.2b/1-383

MKGEQAVVIVGAGPAGLAVSACLTRLSISNLILERETCSVSLWRNRAYDRLCLHLGKIFCQLPHMPYPEPTPTFPSKAQFISYVDDYMARFGLQPSYGRWVRRAAYDATAGTWRLEVSNNQSGEAEEYTARFLVVATGENGEGFVPEIGGLDGFPGDVLHSSEYKSGGAYWGREVLVVGCGNSGMEIAFDLCEHGARTSVVVRNPVHVLTKEIVYMGMQLLNCLPVSSVDPLAIALGKNMFGNLEKYGISEPQEGPFFLKAATGRSPVLDVGTIDKIKSGEIKVRHSIKHIRGDNVEFEDGKVHQFDAIVFATGYKSTVRTWLQEGDGLFNKDGFPEAGYPDHWKGKNGLYCAGFSRRGLFGISEDARKIADDISNHLLNRHK

>g26891.t1_1|Euryale_ferox/1-384

MKRKETVVIVGAGPAGLAVSACLTKLDIPNLILERETCSISLWRNRAYDRLCLHLAKSFCHLPHMPFPEPTPTFPTKAHFIAYVDAYISRFGLQPCYGRCVRRAAYDASTGTWRLEVSSSGSGEAEEYEARFLVIATGENAKGFIPEIVGLDDFPGDVLHSNEYKSGGVYSGREVLVVGCGNSGMEIAFDLCEHGARPSVVVRNPVHVLTKEIVHMGMQLLNCLPMSSVDPMAIALGKIMFGNLEKYGITEPQEGPFFLKATTGRSPVLDVGTIDKIKSGEIKVRRGIKHIKGNDVEFEDGQVHRFDAIVFATGYQSTVRTWLQEGDGLFNKQGYPETEYPHHWEGKNGLYCAGFSRRGLFGIAEDAQKIADAISSFLLNERIV

>LOC_Os02g17230|Osativa_v7.0/1-384

MEEVVVLIIGAGPAGLATAACLTLQHVAYAIIERESCTASLWRHRTYDRLKLHLAKEFCELPHMAYPSGTPTYVPRESFVEYLDSYTDRFGIQPRYDTSVESATYDQGKKHWAVLAQDTDTGVVARLTARFLIMATGEKSAASIPLVPGLAGFEGEAIHSSAYKSGNGYTGKSVLVVGAGNSGMEIAYDLATHGAHTSIVVRSPVHIMTKELIRFGMTMVQNLGLSVTIVDPLLVMAAKLIFWDLSKHGIMRPKMGPLLLKSQTGKSAVIDVGTAKLITRGVIDVLEGILKINANNVEFHCGRQIPFDAIVFATGYKSTVNTWLKNGESMFRNDGFPKKKFPNHWRGENGLYCAGFARRGLVSIAMDAKNIVDDIRATMYQVSC

>LOC_Os11g10170|Osativa_v7.0/1-387

MNAMEDVVVLIVGAGPAGLATAACLAQRHVPYIIVERESSTASLWRHRAYDRLKLHLAKEFCELPHMAYPAGTPTYVPRDMFVEYLDSYANQFGIRPRYHTAVESAIHDKGKNQWVVLVRDMDTSVVARLATQFLVVAAGENSAANIPPIPGLSRFEGEAIHSSAYKSGRAYTGKSVLVVGAGNSGMEIAYDLATHGAHTSIVVRSPVHIMTKELIWYGMTMVQNLGLNVTAVDSLLVMAANFYFGDLSKHGIMRPKMGPLLLKSQTGRSAVIDVGTARLIKGGVIKVFQGISKINTNSVEFHGGRQNSFDAIVFATGYKSTVNAWLKNGESMFKDDGFPKNYFPNHWRGENGLYCAGFARRGLAGIAMDAKNIANDIVAAMDKMSC

>Bradi2g04247|Bdistachyon_v3.2/1-384

MEEVVVLIVGAGPAGLGTAACLSQLSVPYIIVERENCSASLWRNRAYDRLKLHLAKEFCELPHMSYPVDAPTYIPKRLFVKYLDDYIERFNIQPKYLTVVQSSTYDIDGKYWSIMVRDITSDTIINYMAKFLVVASGENSAPNIPMFSGQETFPGVAVHSSSYKSGSVYSGRNVLVIGSGNSGMEIAYDLVTHGANTSVVIRSPIHIMTKELIRLGMTLVNHLSPELVDNLLVMMSNFIFGDLSKHGIMRPKNGPLVLKSETGRSAVIDVGTVGLIKKGIIKVQGRVIKIKGKTIEFEGGDEASFDAVVFATGYKSTTNMWLKNGESMLNNEGLPNKEFPDHWKGENGLYCAGLARRGLAGIAIDAKNIANDIKCNLDSLFMSG

>Bradi2g04240|Bdistachyon_v3.2/1-384

MEEVVVLIVGAGPAGLATAACLSQLSIPYVIVERESCSASLWRNRAYDRLKLHLAKEFCELPHMSYPLDAPTYIPKNQFVKYLDDYIERFNIQPKYLTVVESSTYDNDGKFWSVMVRDMTRCVVVNYMAKFLVVASGENSAVNIPMFRGQETFPGVAIHSSSYKSGGSYSGRNVLVIGSGNSGMEIAYDLATHGANTSLVIRSPIHVMTKELIWLGMTLAHHLPLNIVDHLLVMMADFVFGNLSKHGIMRPKKGPLVLKLETGRSAVIDVGTVGLIKKGTIKVQGRVTKIKGKTIEFQGGNEASFDAIVFATGYKSTATMWLKNCESMLNSDGLPNKKFPNHWKGENGLYCAGLARMGLACIAMDAKNIANDIKSNLDSMSMSG

>Bradi2g04257|Bdistachyon_v3.2/1-383

MEEVVVFIVGAGPAGLATAACLNQFSIPYVIVECENCSASLWRNRAYDRLKLHLAKEFCELPHMSYPVDAPTYIPKDQFVKYLDDYIERFNIQPKYLTVVESSTYDVDGNFWSVMARDMASCTVVNYVAKFLVVASGENSATNIPMFPGQQTFPGVTIHSSNYKSGSIYSGRNVLVVGSGNSGMEIAYDLATHGANSSLVIRSPIHVMTKELIRLGMRLLRHLPPKLVDHLLVMMADFVFGDLSKHGITRPKKGPLVLKSETGQSAVIDVGTVGLIKKGTIKVQGGVTKIKGKTIEFQGGNEASFDAIVFATGYKSTANMWLKNGESMLNNDGLPNKEFPNHWKGENGLYCAGLARRGLAGIAIDAKNIANDIKSKIDVMRSI

>HORVU.MOREX.r3.1HG0078920|Hvulgare_Morex_V3/1-379

MEEVEVLIVGAGPAGLATAACLTQFCIPFVILERENCSASLWRNRTYDRLKIHLAKEFCELPHMSYPVDAPTYIPKNTFIKYLDDYIECFDIQPRYLTVVESATYDIDGKCWSVIARDKVKCRIVNYMAKFLVVASGENSAEHIPTVPGLETFPGVVIHSSSYKSGITYCGRNVLVIGSGNSGMEIAYDLASHDANTSIVVRSPIHVMTKELIRLGMTLVRYLPMKMVDDLLVMVSNFKFGDLSRHGITRPKKGPFVLKSEIGRSAVIDVGTVGLIKERKIKVQGRITKIKGKTIEFQGGNEGSFDAIVFATGYKSTSNMWLKNGEDMLNSEGLPKKKFPNHWKGGNGVYCVGLARMGLLGIAIDAKNIASDIRDSMSD

>HORVU.MOREX.r3.1HG0069150|Hvulgare_Morex_V3/1-382

MEDVVVLIVGAGPAGLATAACLNQFSIPYVIVERENCSASLWRNRTYDRLKLHLAREFCELPHMPYSVDTPTYIPKKTFIKYLDDYIECFNIRPRYFTVVESSTYDIGGKCWSIMARDMAKCATVNYRAKFLVVASGENSVENIPVIPGLENFPGEAIHSSGYKSGINYSGRNVLVIGSGNSGMEIAYDLASHGANTSIVIRSPIHVMTKELIRLGMTLIHYLPPKMVDHIVLMMANFIFGDLSRHGITRPNKGPFVLKMGSGRSAVIDVGTVGLIKEGKIKVHGRITKIKGETIEFQGGKEDSFDDIVFATGYKSTANMWLKSGEDMLNSEGLPTKEFPNHWKGENGLYCAGLARRGLAGITIDAKNIANDINCNIDSMSG

>HORVU.MOREX.r3.6HG0556600|Hvulgare_Morex_V3/1-382

MENVTMLIVGAGPAGLATAACLSQFSISHVIVERENCSGSLWRHRAYDRLKLHLGKEFCELLHMSYPADATTYIPKNMFVKYLDDYIEHFNIQPKYLTSVESCIYDNDKKCWSIKALDMAKCMTTDFTTQFLVMATGENSAENIPMVSGLQSFPGETIHSRNYKSGERFYGKKVLVIGSGNSGMEIAYDLASHGANSSIVIRSPIHVMTKELIRLGMTLARHLPLHLVDNLLVMGANYIFGDLSRYGITRPKLGPMSLKSRTGRSAVIDVGTVGLIKKGIIKVQREVTNIKGRTVEFQCGNKVSFDAIVFATGYKSTTNIWIKNNESMLNENGMPIKEYPKHWKGENGLYCTGLARTGLAGIAMDVKNIANDINDMIGSFSN

>HORVU.MOREX.r3.2HG0209110|Hvulgare_Morex_V3/1-382

MESVTVFIVGAGPAGLATAACLSQFSIPYAIIERESCSASLWRNRAYDRLKLHLAKEFCELPHMSYPVDAPTYIPKTLFVKYLDDYVERFNIQPKYLTTVESSTYDNDEKCWSIMANDMSKCTTIKFTAKFLVVASGENSAQNIPMFPGLENFPGDVIHSSSYKSGKSYSGKNVLVVGSGNSGMEIAYDLATHGANTSIVIRSPIHIMTKELIRLGMTLAHHLPLNLVDNLLVMLANLIFGDISRHGITRPKMGPMTLKSETGRSAVIDVGTLGLIKKGIIKVQGGISDIRGNIVEFQCSKKISFDAIVFATGYKSTANKWLKHGESMLNGNGLPIQKYPNHWKGENGLYCAGLARRGLAGIAAEAKNIANDIRSVIEYMSS

>HORVU.MOREX.r3.2HG0209140|Hvulgare_Morex_V3/1-382

MESVTVLIVGVGPAGLATAACLSQFSIPYIIVERESCSASLWRNRSYDRLKLHLAKEFCELPHMSYPVDAPTYIPKTLFVKYLDDYVERFNIQPKYLTSVESSTYDNDEKCWSIVANDMSKCTTVKFTAKFLVVASGENSAENIPMFIGLENFPGDVIHSSSYKSGNKYSGKNVLVVGSGNSGMEIAYDLATHGANTSIVIRSPIHVMTKELIRLGMSLAHRLPLNLVDNLLVMSANLIFGDLSRHGITRPKMGPMTLKSETGRSAVIDVGTVGLIKKGIIKVQGSINEIRGNTVEFQCSKKISFDAIVFATGYKSTTNVWLKNGESILNGNGLPIQKYPNHWKGENGIYCAGLARRGLAGIATDAKNIANDIKSVINSMSS

>HORVU.MOREX.r3.7HG0647260|Hvulgare_Morex_V3/1-382

MDNVVVLIVGAGPAGLATAACLSQFSISYVIVERESCSASLWRNRAYDRLKLHLAKEFCELPHMSYPVDAPTYIPKTMFVKYLDDYVERFNIQPKYLTSVESSTYDNDEKCWSIVIHDMAKSTIVKFTAKFLVVASGENSAENIPMIPGLQSFPGDVMHSSSYKSGKSYSGMNVLVVGSGNSGMEIAYDLATHGCNTSIVIRSPIHVMTKELIRLGMTLVHRLPLNLVDNLLLMAAKLIFGDLSRHGITTPKMGPMILKSKTGRSAVIDVGTVGLIKKGIIKVHGSISKIMGDMVEFQCRKKISFDVIVFATGYKSTANIWLKNGESMLNGNGLPIKEYPNHWKGENGLYCAGLARRGLAGIAADAKNIANDIKSVIGAMSG

>HORVU.MOREX.r3.7HG0647220|Hvulgare_Morex_V3/1-382

MDNVVVLIVGAGPAGLATAACLSQFSIPYVIVERESCSASLWRNRAYDRLKLHLAKEFCELPHMSYPVDAPTYIPKTMLVMYLDDYVEHFNIQPKYLTSVESSTYDNEKKCWSIVTHDMAKSTIVKFTAKFLVVASGENSAENIPMIPGLQSFSGDVLHSSSYKSGKSYSRMNVLVVGSGNSGMEIAYDLATHGCNTSIVIRSPIHVMTKELIRLGMTLAHRLPLNLVDNLLLMAAKLIFGDLSRHGITTSTMGPMILKSKTGRYAVIDVGTVGLIKKGIIKVHGSISKIMGDMVEFQCSKKISFDMIVFATGYKSTANIWLKNGESMLNGNGLPIKEYPNHWKGENGLYCAGLARRGLAGIAADAKNIANDIKSVIGAMSG

>HORVU.MOREX.r3.7HG0647280|Hvulgare_Morex_V3/1-382

MENVVVLIVGAGPAGLATAACLSQFSIPYVLVERESCSASLWRNRAYDRLKLHLAKEFCELPHMSYPVDAPTYIPKTLFVNYLDDYIERFNIQPKYLTSVESSTYDNDKKCWCIVAHDMAKSKIVKFTAKFLVVASGENSAENIPMIPGLQSFSGDVIHSSSYKSGKSYSGMNVLVVGSGNSGMEIAYDLAAHGANTSIVIRSPIHVMTKELIRLGMTIAHHLPLNLVDNILVMVANLIFGDLSRHGITRPKMGPMILKSKTGRSAVIDVGTVGLIKKGIIKVYGSISKIMGNIVEFQCSKNISFDAIVFATGYKSTTNTWLKDGESMLNGNGLPIKEYPSHWKGEKGLYCAGLARRGLAGIAADAKNIANDIKSVIGTMSS

>HORVU.MOREX.r3.7HG0652990|Hvulgare_Morex_V3/1-377

MENVVVLIVGAGPAGLSIGACLSQLSIPYVILERKSCSASLWRNRAYDRLKLHLAKEFCELPHMSYPVDAPTYIPKALFVKYLDDYVERFNIRPKYYTSVESSTYDNDKKCWSIVAKDMAKCTTIKFTAKFLVVASGENSAENIPTIPGLESFSGEIIHSSSYKSGKSYSGKNVLVVGSGNSGMEIAYDLATHGANTSIVVRSEIHVMTKELIRLGMTLAHRLPLNLVDNILVMAANFIFGDLSRHGITRPKMGPMALKSKTGRSAVIDVGTVGLIKKGIIEVQGCTNKIFGNIVEFKCGKKISFDMIVFATGYKSTKNIWLKNNENGLPIKEYPNHWKGENGLYCAGLAMRGLAGIAVDAKNIANDIKSMIGSMSS

>HORVU.MOREX.r3.7HG0653020|Hvulgare_Morex_V3/1-293

MENVVVLIVGAGPSGLSIAACLSQLSIPYVKLERESCSASLSRNRAYDRLKLDLAKEFCELPHMSYPVDAPTYIPKALFVKYLDDYVERFNICPKYHTSVESSTYDNDKKCWSIVIHVMTKELIRLGMTLAHRLPLNLVDNILVMAANFIFGDLSRHGITRPKMGPMALKSKTGRSAVIDVGTVGLIKKGKIEVQGCTNKIFGNIVEFKCGKKISFDMIVFETGYKSTTNIWLKNGESMLNDNGLPIKEYQNHWKGENGLYCAGLARRGFAGIAADAKNTANDIKSMIGSMSS

>HORVU.MOREX.r3.7HG0653050|Hvulgare_Morex_V3/1-382

MENVVVLIVGAGPSGLSIAACLSQLSIPYVKLERESCSASLSRNRAYDRLKLDLAKEFCELPHMSYPVDAPTYIPKALFVKYLDDYVERFNICPKYHTSVESSTYDNDKKCWSIVAKDMAKCTTIKFTAKFLVVASGENSAENIPTIPGLESFSGEIIHSSSYKSGKSYFGKNVLVVGSGNSGMEIAYDLATHGANTSIIIRSQIHVMTKELIRLGMTLAHRLPLNLVDNILVMAANFIFGDLSRHGITRPKMGPMALKSKTGRSAVIDVGTVGLIKKGKIEVQGCTNKIFGNIVEFKCGKKISFDMIVFETGYKSTTNIWLKNGESMLNDNGLPIKEYQNHWKGENGLYCAGLARRGFAGIAADAKNTANDIKSMIGSMSS

>HORVU.MOREX.r3.1HG0023920|Hvulgare_Morex_V3/1-382

MENVVVLIVGARPAGLATAACLSKFSIPYVIVERQDCSASPWCKRAYDRLKLHLAKEFCELPHMSYLVDAPTYIPKNTFVKYLDDYIERFNIQPKYLTNVESSTYDNDKKRWSIVAHDMTTCTIVKFRAKFLVVASGENSAENIPMILGLQSFPGDVIHFSSYKSGKSYSGKNVLVVGSGNSGMEISYDLATHGANTSIVIRSPIHVMTKELIRLGMTLAQRLPLNLVDNLLVMSAKFIFGDLSRQGITRPKMGPMTLKSATGKSAVIDVGTVGLIKKGIIKVQGGISKIMGNIVEFQCSKEISFDAIVFATRYKSTANIWLKDGESMLNGNGLPIKEYPNHWKGENGLYCAGLARRGLASIAVDAKNIANDIIYVMGSGSS

>LOC_Os12g08780|Osativa_v7.0/1-384

MEKALVLIVGAGPSGLATAACLGQLSIPYVIIEREDCTASLWRKHTYDRLKLHLAKEFCEMPHMPYPEDTPTYIPKIQFLRYMDDYVEHFNICPKFNSSVESCLYDDVQKYWVVTTHDQVNGMVSKYAARFLVVASGENSAGNIPSIPGLEDFSGHVIHSSSFRSADSYAAQRVLVVGCGNSGMEIAYDLSSHGANTSIVIRSPLHVMTKELIHMGMKLASWSLPVKFVDFILVVLAYLWFGNLSKYGIVRPNKGPLLLKANTGRSAVIDVGTVELIKKGDIKVFGTISCIKGNVVEFDDGKESYFDAIVFATGYTSTANNWLKNGEDMMNKEGMPKKDFPNHWKGSNGLYCVGFARRGLSGIAHDAKNVANDVKAFLDLMAPF

>Sobic.008G064100|Sbicolor_v5.1/1-384

MEKVLVLIVGAGPSGLAAATCLSEDGIPYRIIEREDCSASLWRKRTYDRLKLHLAKEFCELPHMSLPSDSPKYITKEQFVRYVDDYVERFNIFPKYRTSVESCEYDEVSNCWDVIARDLVNGQVNEYTARFLVVATGENSEGVIPNIPGLHDFPGDVIHSSNYKSWNNYTGKGVLVVGCGNSGMEIAYDLASNGVETSLVIRSPVHVMTKGLINLGMKLLKWHLPVKFVDFIILTLANIRFGDLSKYGIVRPDMGPLLLKAKTGRSAVIDVGTTQLIKTGVIKVLGPISCIRGNTVEFEDGKKSDFDSLVFATGYRSTANTWLKNGESLLNENSMPKRELTNPWKGENGLYCVGLGMAGLAGISRDAKSVAADIKSAMNSMGPF

>ELECO.r07.5BG0431000|Ecoracana_v1.1/1-168

MKLSKWNIPIKFLDFILVTLAYFFFGDLSKHGIIRPNMGPLFLKAQTGRSAVIDVGTIELIKKDVIKVFGPIYCIRGNAVEFEDGKTDYFDSLVLATGYKSTANTWLKNGESLLNEEGMPKKGFPNHWKGENGLYCVGLAMRGLAGISYDAKMVAEDIKSIIDSMEPF

>ELECO.r07.5AG0384130|Ecoracana_v1.1/1-408

MSSSRLKQDHHSLDFASSPSTEFQMEKVLVFIVGAGPSGLAVAACLQHASIPYVIVEREDCSASLWRKRTYDRLKLHLPKEFCELPHMPYPSDTPTYIPKEQFVRYVDAYIEHFNICPKYRTLVQSSKYDEASNCWVIMAHDQVSGLVIEYAARFLVVATGENSEGNIPKIPGIHDFHGEVIHSSSYKSWNHYGGKRVLVIGCGNSGMEIAYDLAGNGVDTSIVIRSPLHIMTKGLIRLGMKLSKWNIPIKFVDFILVTLAYFFFGDLSKHGIIRPNMGPLFLKAKTGRSAVIDVGTIELIKKDVIKVFGPICCVRENAVEFEDGKRDYFDSLVLATGYKSTANTWLKNGESLLNEEGMPKRDFPNHWKGENGLYCVGLAMRGLAGISYDAKRVAGDIKSIIDSMEPF

>Bradi4g40770|Bdistachyon_v3.2/1-384

MDMVEVLIVGAGPSGLSTAACLSKFSIPYVIVEREDCIASLWHKHTYDFLKLHIAKEFCELPHMSYPTDAPTYIPKKDFLRYVDDYVEHFNIIPKFNTSVESCIYDEARKRWVILARDKVNGTILDYASRFLVVATGENSVSNIPEIIGLQSFPGETIHSSSYKSGNDYVGKSVLVVGSGNSGFEIAYDLAVHGAKTSITIRSPMHVMRKELIHLGMVLAKWHIPLKFVDFILMVLAYLLFGDLSKYGIVRPRMGPLLLKAKTGRSAVIDVGTTELIKKGDIKVVGPISHIRGDLVEFEDANERCYDTIVFATGYKSNVNMWLKNDMGMLNNDGMPKNDFPNHWKGAKGLYCVGLGRRGLAGVAKDANMVANDIHDTIEMMSFN

>Bradi4g40750|Bdistachyon_v3.2/1-389

MEVVEVFIVGAGPSGLATAACLNKFSIPYVIVEREDCIASLWHKHTYDRLKLHIAKEFCELPHMSYPDDAPTYIPKDQFMRYVDDYVEHFNIVPTFNASTESCMYDEEKKYWAISAHDKVNNKMLEYAAKFLVVATGENSASNIPEIIGLPSFPGETMHSSSYKSGNDYAGKSVLVVGSGNSGFEIAYDLAVHGAKTSIIIRNPMHVMKKEMIHLGMVLAKWHIPLKFVDFVLIVLAYFLFGDLSKYGIVRPFLGPLLLKAKTGRSAVIDVGTTELIKKGDIKVLDSISRIRGKLIEFKDGKKRYYDTIVFATGYKSTVNMWLKSDVSMINSDGMPKNDFPNHWKGANGLYCVGLARRGLAGIANDAGVVANDIHDVIEMVRDEVKIQL

>HORVU.MOREX.r3.7HG0647150|Hvulgare_Morex_V3/1-383

MEKVQVFIIGAGPSGLATAACLSKHSIPYIIAEREDCIVSLWHKRTYDRLKLHIAKDFCELPHMAYPTDSPTYLPKDQFLRYMEDYVKHFNICPKFNALVESCMYDEVMKCWVITTHNKVDGPIIYASKFLVVATGENSAGYVPEIVGLQSFRGEAIHSSSYKSGSNYVGKSVLVVGCGNSGFEISYDLAVHGANTSIIIRSPLHVMTKELIHLGMVLSMWHLPLKLVDFILMILAYSLFGDLSKYGIVRPDIGPLTLKAKTGRSAVIDTGTTELIKKGDTKVFGPISCIRGNLIEFADGNERYYDAIVFATGYKSTANMWLKNDKSMLNSDGIPKNDFPNHWKGSNGLYCVGFGRRGLVGIAHDATIVASDIHANIEMADFN

>Zm00001d000445|Zmays_RefGen_V4/263-639

MELVVLIVGAGPAGLATAACLSQRSIPYLIVEREDCSASLWRYRTYDRVKLHLSKEFSCLPYMPHEEDTPTYIPKAEFLKYLDCYREHFGIKPRYCTSVVSAAYDEGTGRWVVAARDTVEGTEIRYAARFLVVATGENGAGRIPEIQGLESFCGEAIHSSTYKSGRSYAGRRVLVVGAGNSGMEIAYDLANHGADTSIVVRSPVHIMPKELIRLGMTFVQYMPVTIVDLFLVKLADFIFGDLSNYGIVRPGVGPLQLKSKTGRSSVIDVGTAGLIKKGVVKVFKRISKITGNKVQFECGKDCEFDAIVFATGYKSSANLWLKADDKCMVNSDGRPNTCRPNIWKGENGLYFSGFRRMGLAGICMDAYNIANEIVSVY

>Zm00001d049077|Zmays_RefGen_V4/413-789

MELVVLIVGAGPAGLATAACLSQRSIPYLIVEREDCSASLWRYRTYDRVKLHLSKEFSCLPYMPHEEDTPTYIPKAEFLKYLDCYREHFGIKPRYCTCVVSAAYDEGTGRWVVAARDTVEGTEIRYAARFLVVATGENGAGRIPEIQGLESFCGEAIHSSTYKSGKSYAGRRVLVVGAGNSGMEIAYDLANHGADTSIVVRSPVHIMPKELIRLGMTFVQYMPVTIVDLFLVKLADFIFGDLSNYGIVRPGVGPLQLKSKTGRSSVIDVGTAGLIKKGVVKVFKRISKITGNKVQFECGKDCEFDAIVFATGYKSSANLWLKADDKCMVNSDGRPNTCRPNIWKGENGLYFSGFRRMGLAGICMDAYNIANEIVSVY

>Sobic.005G104600|Sbicolor_v5.1/1-376

MELAVLIIGAGPAGLATAACLSQRSIPYLIVEREDCSASLWRYRTYNRVKLHLSKEFSSLPYMPHPDGTPTYIPKEEFLKYLDCYAQHFDIKPRYCTCVVSAAYDEGTRRWIVAARDTAAGTEILYAAKFLVVATGENGEGRIPEILGLESFHGEAIHSSTYKSGSSYAGKRVLVVGAGNSGMEIAYDLASHGADTSIVARSPVHIMTKELIRLGMTFIQYIPITIVDLFIMNIADVIFGDLSKYGIVRPRIGPLLLKSKTGRSCVIDVGTAGLIKNGIVKVFKGISKITGNKVQFECGNGSEFDAIVFATGYKSTANLWLKDDKCMLNSDGHPNKGYPNIWKGENGLYFSGFARMGLAGISKDAYNIANDVASVY

>Nycol.A00013|Ncolorata_v1.2b/1-386

MWKEVIIVGAGAAGLSASACLNKLSIDNLILEKDDCSVPLWRKYSYDRLHLHLAKTFCELPHLPYPKTAPTYLSKDEFVKYIDAYVQQFDLRPVYDRCVETAVYDRAAGAWTVRAKHTRTGEAEEYRARFLVVATGENSEGYVPDVPGLDGFPGEVMHSSQYKNGVPYKDQDVLVVGSGNSGMEIALDLCNHHARASLVVRAPVHIITREVANLGLHLFKYLPYSTVDSLQVLHSKLKYGDTAKYGIVRPTEGPNHLKDTTGKYPVVDIGTFDKIKSGDIQVYPGVKCIRSSDLEFENGSTRRFDAIIFATGYKSTVKAWLKDDDGFFDESGFPAEGWPSQWKGNNGLYCVGFSRKGFYGIAEDAKNVAQDISVVLSSQSVSKPKP

>g36161.t1_1|Euryale_ferox/1-386

MWREVIIVGGGAAGLSASACLNKLSIDNLILEKDDCSVPLWRKYAYDRLHLHLAKNFCELPLLPYPNTTPTYPSKDEFVQYIDAYIAEFGLQPLCHRSVESAVCDRSAGTWTVQAKNTLTGELEEYRSRFLVVATGENSEGHVPDIPGLDGFPGKVLHSCQYKNGVPFKDQDVLVVGSGNSGMEIALDLSTHHAKASIVVRTPVHIITREVANLGLHLLKYLPYSTVDSLQVFHSKLKYGDTAKYGIRRPIEGPNFLKDNFGRYPVVDIGTFDKIKSGEIQVYPEVKCIRGNDVEFENGSIHRFDTIVFATGYKSRVKTWLKDDDGFFDETGFPAAGWSSKWKGNNGLYCIGFSRLGFYGIAADANNVAQDVSLVLSSQNESKPKP

>Cucsa.108650|Csativus_v1.0/1-385

MADTTVIIIGAGPSGLATAASLTLSSISYIILEREDCSIPLWRKHSYDRLRLHLPNRFCHLPAMPFPSSAPNYLPKVNFLDYLDRYADNFRIRPLYRRNVEAAEFDHPEGKWKVRARNLDKGEVEEFRSRFLVVATGETAEAYTPAVPGMEGFGGDLMHSTKFKSGKGFEGKNVLVVGSGNSGMEIALDLCLHAANTSVLVRSPVHFMSKGMMTLGLDMLKYNLPIWFVDSFIVMLSKLIYGDLTKYGIKRPLEGPLYMKVKYGKYPIIDGGALHKIKCGQIQVLGEEISSIKGNNNVVFNNGKCYQFDSIIFCTGFKRSTNLWLKGDEYLLNDDGLPKPCYPNHWKGKNGLYCVGLSRRGLYGASLDAQNVAKDISTQINKFGY

>Eucgr.H01211|Egrandis_v2.0/1-383

MSKEAEVIIVGGGPSGVAAAGALSSLSIPYLLLERANCFAPLWQRGAYDRLHIHLPKHTCQLPHVPVPADWPKYPPKSLFVQYLQEYVSHFGINPVYSRSVESASYDEASGRWRVVARDVSKGGAGVVEEYSTRFLVVATGESCDAFVPEVEGMESFPGKAMHSTEYKNGKEFAKKKVLVVGSGNSGMEIALDLADHGAITSIVVRSPVNFVTREMLNWAIVLVKYLSFSWAERLTMIASRLWFGDMSKYGIPKPKEGPFTMKMKYGKYPLVDVGTCAKIRSGEIQVLPAVKCIRGNEVVFENGHSDSFDAIVFATGFKRPTKQWLKVKEEDCILNDDGIVKPSYPDKWKGKNGLYCVGLSRRGFFGASLDAQSVANDIKGLL

>GWHPCBIR007413|Santalum_yasi/1-393

MTQSTESRSQPQEMDRDTTTAIIVGAGTSGIAVAACLNRLSIPNIVVEREDCIASLWQKKSYDRLHLHLSKRICQLPHLPFPDSYPNFVPRELFVQYLNDYSSHFGVNPRFRRRVDSAEYDGNSRRWRVRVANLGTGGEAEELSGRFLVVATGVSCDPFVPEVEGLEGFAGDWIHSTEFRSGKGYEGKSVLVVGSGNSGMEIAFDLATHGVKTSIVARSGMHILSKEMMDWAGYLLKYMSGDKVEKLMVLLSRIWYGDLTKYGITRPQEGPFTMKRKYNKFPIIDVGTCQLIKSGQIQVLPAVKSIRGNEVAFVNGRSYPFDVIIFATGFSRPTKSWLKGDDGLLNDDGYANPTNPNHWKGKNGLYCVGLASRGFYGAGLEAQSIADDIKKHL

>Asa0G03804.1|Allium_sativum/1-403

MQIEVVIVGAGPAGLSTAACLNNLTIPNIIIEKENCNAHLWRNKTYDRLKLHLAKEFCTLPLMPHEESAETYISKEDFVKYLDKYVQKFNLNIVYNTPVTSACYDESVEKWIVKAIDEVTGDEHVYEATFLVVATGENAKGFIPEIPGLATFKGDMMHSCQYKSGKNYEGKRVLVVGSGNSGMEIAYDLCNHGAKTSIAIKSPFHVLKKEFIHMAMIFAKHRPIKEVDLFLVYLSWLHYGNLSKYGIYRPNKGPMQLKHECSISPVVDVGTVDKIKSGEIQVVKELIRVGEDEAEFKDGKVYQYDAIVFATGYKSTANYWLKDEGRLMDGEGFPKESFPNHWYGKDGIYCAGFSRRGLDGIALDAKKIAKHINVMLRMKEQKSQGEKEEEEKKKNEADVPLSY

>Aco006326|Acomosus_v3/1-404

MHDSDTTPQTPKMSGASEEAVVVIVGAGPSGLAVAACLAARSVPSLILERDDCVAPLWRHRAYDRLHLHLAKRFCALPLLPFPRDAPTFVPARDFLRYLELYAARFAARVRLRRRVAAALFDPPSSTWRVRARALDTGAEEEYAARFVVVAAGENCAPALPALPGLDGFPGPVRHSSEYRSGSGYAGMRVLVVGSGNSGMEIALDLAEAGAKTSIVVRSPLHILSREMWLLGMILTKFLPVRLVDAFLLFLCYLKYGDTSKYGIRRPAIGPFDMKINTPLYPVLDVGTYDKIRSGEIQVLPAVSSVKGNNVEFSDGNSYPFDAIIFATGYKSTIKEWLQSDEYLIGDDGMAKQKYPNQWKGKNGLYCVGLARRGLYGTSEEAQLIAEDISKEYNNKYNNNDKGN

>Aco002405|Acomosus_v3/1-197

MEIALDLAESGAKTSIVVRSPLHLVTREIWLLGMHLRKFLPLYLVDALALFLCYLKFGDTSKYEIHRPTKGPFYLKANSPVFPVMDVGTFDKIKAREIQVLPSITRINSNSVTFANGKVEYFDAIILATGYRATTKEWLKGDDYLIDDDSMAKQKYPNNWKGKNGLYCAGLGRRGIPGTSEDALKIAKDIYESSCCE

>Acora.03G132600|Aamericanus_v1.1/1-394

MMKKKTTTIEKEVVIVGGGPSGLATAACLKVLSITNIAILEREDCLCSLWKKRTYDRLHLHLHKNLCSLPHMPYPSNSPSYVPRSGVVDYLDSYAKRFDVRPTFNSDVEKVEFDEEEGRWRVVVGMGEEVVEYLARFVVVASGENVEGVVPEVKGLEGFGGEVLHSSQYRSGKKYEGKNVLVVGCGNSGMEIACDLAESGAKTSIVVRSPVHVVTREIWHLGIFLSKFFPYHWVDSIVLFLCYLKFGNTSEFGLRRPSKGPFYLKDNTPFYPVVDVGTVDKIKSGQIQVLPSLASVKGSEVRFVDGKTLQFDAIVFATGYKRTVKNWLKDDGYLIDDDGMAKQEFPNHWKGMNGLYCAGLKRGGFYGSSEDALNIANDINNLLKLRETVSSEQK

>Asa7G00370.1|Allium_sativum/1-183

MHIVSRYIWLFCMRLMKYFSLETIDKIALLLCYLKYGDTSKFGIRRPAQGPFYLKANTPVYPIIDCGTFQKIKTGEIQVLPSLERVKGYNATFSDGKAQQFDAIILATGYRSKINSWLKDDDYLIDDMGMARQAFPNHWKGKNGLYCAGFTRRGFYGISEDAISIANDISKKLKLTKSVKKMI

>Asa7G00375.1|Allium_sativum/1-375

MKQEAQVIIIGAGPAGLATAACLSNLSIPSLILEREDCITPLWRNRTYDRLHLHLSKDFCQLPLLCYPASYPTFISKHQFLLYMENYSHKFILQPLFNHEVKSLSYDNVSTKWNILTKDDKYEAEYVVIATGENDVPHVPHIDGIDKFIGKVMHSSEYKSGLEYEGKNVLVVGSGNSGMEIAYDLCSFGAKTSIVIRSPMHILSRYIWLFYLRLMKYFSLKTIDKIVLFLCYLKYGDTSKFGIRRPAHGLFHFKENNPVYPVMDPGTFQKIKTGEIQVLPSLKSIEGNGATFSDGKFHCFDAIVLATGYRSKINNWLKNGDFLINDIGMAKQAFPNHWKGKNGLYCAGLARRGLNGISGDAVNIANDIRKNSDLA

>LOC_Os01g16714|Osativa_v7.0/1-398

MAAVEQDQEEEVIIVGAGPSGLAAAACLSVRGVTGCLVLERDDCVASLWRHRTYDRVRLHLAKRYCALPHAPHGEASPTYLPRDDFLRYLDAYASRFGVRARLRREVRSARYDAARARWLVDAVDLATGRAERYAARHLVAAAGENDERVVPEVPGMETFPGKVVHAADYRSAEGFKGKSVLVVGGGNSGMEIAYDLAVGGAATSIVIRSELHLVSKEIWNLAMTLYRYLPVWVIDKVVLLMCAAVFGDTARYGLRRPAVGPFTMKATTTMYPVVDVGTFAKIRSGEIRVLPAAIKGVRGRDVEFADGQRHAFDAVVFATGYRSTTKHWLKSDDGLIGDDGMAGRSYPDHWKGENGLYCAGMVRRGIYGSYEDAEHIADDISKQLRSSSKPTHNNGSA

>LOC_Os01g16750|Osativa_v7.0/1-411

MAVAAEGEEMTPEHEEEVIIVGAGQSGLAAAACLSVRGVASCLVLERDDCVASLWRHRAYDRLRLHLPKRHCALPRAPHAAAAPDYLPRDDFAAYLDAYASRFGVRTRLRREVRSARHDAARARWLVEAVDLATGKAERYAARHLVAAAGENDERVVPEVPGMDTFPGKVVHSADYRSAGAFKGRSVLVVGCGNSGFEIAYDLAAGGAAAVSIAVRGEVHLVSREVWSVGMALQRYLPTWAVDKVVLLMCAVVFGGDTARYGLRRPAVGPFSMKMTTPAYPVFDVGTFAKIRSGEIRVVPAGIKSVRGGDVEFADGRRHAFDAIVFATGYRSTTKQWLKSDDGLIGDDGMAGRSYPDHWKGENGLYCAGMVRRGIYGSGEDAELIADDISKQMKRWSSEPVYNGHISNGSP

>Zm00001d008255|Zmays_RefGen_V4/1-391

MAAVAEEEVIVVGAGQSGLAVAACLSLRGVRALVLERDDCVGSLWRKRAYDRLHLHLTKKYCALPHAPHPAEAPAYLHRDDFARYLDGYAARFAVRPRLRREVRSARYDPASARWEVEAVDLGTGQAERYAARFLVVASGENAEKFLPEVPGLEAFPGQVMHAVEYRSAEGMRGKAVLVVGSGNSGMEIAYDLAAAGAVTSIVVRGELHLVTKEIWNVAMTLYPYLPVWVIDKLVLLMCAVVFGDTSRHGLRRPAIGPFTMKLTTPGYPVVDVGTYAKIRSGEIRVLPAAVKSVRGNVVEFGDGSRHPFDAIVFATGYRSTVRRWLKSEDGLVGDDGMAARSYPEHWKGDHGLYCAGMVRRGIYGSCEDAELIAADISKLLHPKQEQGNGV

>Sobic.003G128700|Sbicolor_v5.1/1-406

MAAAAAVTTEEDVIIVGAGQSGLAVAACLSLRGVRALVLERDDCVGSLWRKRAYDRLHLHLAKKYSALPHAPHPDSAPTYLHRDEYAAYLDGYAARFGVRTRLRREVRCARYDPGAARWEVEAAAAAAGGAGEVVVERYAARFLVVASGENAEKFVPEVPGLEAFPGKVMHAAEYRSAEGMQGKAVLVVGSGNSGMEIAYDLAAAGAITSIVVRSELHLVTKEIWNVAMALSAYLPVWVIDKLVLLMCAVVFGDTSRHGLRRPAVGPFTMKLTTPAYPVVDVGTYAKIRTGEIRVLPAAVKSVRGNVVEFGDGKRHPFDAIVFATGYRSTVTHWLKSEDGLIGDDGMAARSYPEHWKGDNGLYCAGMVRRGIYGSCEDAELIAGDISKLLLLRPQQEQEQEQGNGF

>ELECO.r07.1AG0004030|Ecoracana_v1.1/1-401

MAVVVEEDVIIVGAGQSGLAAAACLSQRGVRSLVLERDDCVGSLWRKRSYERVRLHLAKHYCALPHAPHPDAAPTYLPRDDFVRYLDAYAARFAVRTRLRREVRAARYDKATARWEVEAMDLAAGGKVELYAARFLVVASGENDEKVVPEVPGIEGFPGTVMHACEYKSPEGMKGKAVLVVGGGNSGMEIAYDLADAGAVTSIVVRGELHVVNKQIWNVAMALYPYLPLWLIDKLVLLMCAVVFGDTAKHGLRRPAIGPFTMKCTTPAYPVVDVGAYAKIKSGEIRVLPAAVKSVHGNTVEFNEGKQYPFDAIVFATGYRSTVRRWLKSDDGLIGDDGMAARSYPEHWKGEKGLYVAGMVRRGIYGSYEDAEFIAEDISKQLHPEAHGNGKNGEQEQLLAT

>ELECO.r07.1BG0052450|Ecoracana_v1.1/1-404

MAAAAVVEEEEVIIVGAGQSGLAAAACLSLRGVRSLVLERDDCVGSLWRKRSYDRVRLHLAKHFCALPHAPHPDAAPTYLPRDDFARYLDAYAARFAVRTRLRREVRRASYDVAAARWEVEAVDLAAGGKVELYAARFLVVASGENDEKVVPEVPGIEGFPGKVLHACEYKSPEGMKGKSVLVVGGGNSGMEIAYDLAGAGAVTSIVVRGEVHVVTKEIWNVAMALYPYLPLWVIDKLVLLMCAVVFGDTAKHGLRRPAIGPFTMKCTTPAYPVVDVGTYAKIKSGEIRVLPAAVKSVHGNLVEFNDGKQYPFDAIVFATGYRSTVRRWLKSDDGLIGDDGMATRSYPEHWKGENGLYVAGMVRRGIYGSYEDAEFIAEDISKQLHPEAHGNGNNGKQEQLLAS

>Bradi2g10302|Bdistachyon_v3.2/1-396

MAEEQQEYSEEVIIVGAGPSGLAAAASLSVRGVPSLILERDNCVASLWRNRTYDRVQLHLAKHYCALPHFPHPPSAPTYLPRADFIRYLDGYAARFGVRAALGREVRAARFDAARGLWAVDAVDAATGKSERYVARRLVAAAGENDRMVLPEGLPGMETFPGTVMHAGEYRNGKGFEGKRVLVVGSGNSGMEIAYDLAVAGAAASVVVRSELHLVTKEIWNVAMTLYRYLPVWLIDRIVLLLCAVVLGDTSRYGLRRPAIGPFSMKLQTPAYPVVDVGTYAKIKTGEIQVLPAMKSVDRDVVEFADGKRHPFDAIVFATGYRSTTKQWLKDDGLIGEDGMAKRSYPGHWKGENGLYCAGMVRRGIYGSYEDAELIAEDISNNKKRQSKPDDHEKSN

>HORVU.MOREX.r3.5HG0481610|Hvulgare_Morex_V3/1-393

MAQAAEAERPREEEEEEVIIVGAGPSGLAAAACLSLRGVRSLVLERDGCVGSLWRNRTYDRIRLHLAKQYSALPHAPHGPAAPTYLPRDDFVRYLEDYAARFGVRVRLRREVREARFDGARGAWLVDAVDHATGLVERYAARHLVAAAGENDEKVLPEVPGLDGFPGKVMHACEYKAGKGMEGKAVLVVGSGNSGMEIAYDLAEAGAATSIIVRSELHLVTKEIWNVAMTLYRYLPLWLIDRIVLFMCSVVFGDTSRYGLRRPAIGPFSMKILTPAYPVVDVGTYAKIKTGEIQVLPAMKTVCGNVVEFADGKRHPFDAIVFATGYRSTTKKWLKSDDGLIGEDGMARRSYPEHWKGEKGLYCAGMVRRGLYGSCEDAESIAEDISKKKHHQA

>Kalax.0112s0062|Klaxiflora_v1.1/1-395

MENVAVLIVGAGPSGLATSACLSSLSIPHIILEKEDCDTSLWRKYTYDRVGLHLPKDMCSLPFAPHSPDAPTFLSKDIFLEYVDNYVARFNIRPRYNRNVTDARFDEEVQRWRVEAVNKEGDVEVYGARFLVVATGENDIANLPEIDGLEGFKGEIVHSKYYKSGAKFEGKNVLVIGSGNSGMEIAFDLSNFGAHTSIVVRSPIHVISKEMMRWGFKSLQVFPVSMVDRVLTFASKMEMGDLSKYGVQRPSKGPLYIKSTTGRTPVLDVGTVAKIHSGDIKVFPAINKFDENIVKFSNGSKQEFDAIVLATRYKSGASGWLKDYHYMFNADRTPIRKFPDQWKGENGIYCGGMAKMGLFGISKDAKFIANNIHTILAKEEFFQFDRTELELELEF

>Kalax.0828s0002|Klaxiflora_v1.1/1-394

MENVVAVLIVGAGPSGLATSACLSSLSIPHIILEKEDCDTSLWRKYTYDRVGLHLPKDMCSLPFAPHSPDAPTFLSKDIFLEYVDNYVARFNIRPRYNQNVTDARFDEEVQRWRVEAVNKEGDVEVYGARFLVVATGENDIANLPEIDGLEGFKGEIVHSKYYKSGAKFEGKNVLVIGSGNSGMEIAFDLSNFGAHTSIVVRSPIHVISKKMMRWGFKSLQVLPVSMVDRVLTFASKMEMGDLSKYGVQRPSKGPLYIKSTTGRTPVLDVGTVAKIHSGDIKVFPAINKFDENIVKFSNGSKQEFDAIVLATGYKSGASGWLKDYHYMFNADGTPIRKFPDQWKGENGIYCGGMAKMGLFGISKDAKLIANDIHTILAKEEFFQSDQTELELEL

>Kalax.0601s0010|Klaxiflora_v1.1/1-383

MVVEDVAVLIVGGGPSGLATAACLTNHSIPYVILEREDCNASLWRKHTYDSPKLHTPKEASALPLHPISDESPTYIPKAAFIDYIDAYVARFDIRPRCNRNVTKAEFDRALGKWRVEAAVGEGGVEVYVARFLVVASGENDVENVPEIEGLARFKGRVVHSRQYKNGVEYEGKRVLVVGAGNSGMEIAYDLSVSGARASVVVRSPIHILTKEMLMWGLKSYLTGVYTRPVVDIALMFMSRLRMGDLSRYGIRRPPKGPIQNRLATGRGSVLDVGTVAKIHSGDIKVFPAIKKICHNTVTFSDGSEQEFDAIILATGYKSGAPGWLKDYHSILNGDGTLKKKSPEQHRKGEDGVYCAGLVKQALYGCANDAQLIAGDIHMTLNK

>Kalax.0601s0009|Klaxiflora_v1.1/1-381

MRDVVVLIVGGGPSGLATAACLTYHSIPYVILEREDCSASLWRKHSYDSLKLHTPKEMSALPLLPISDESPTFIPKAAFVDYIDAYAARFDIRPRCNRNVTKAEFDRELGKWRVEAAVGEGGVEVYAARLLVVASGENDLENVPEIEGLARFRGRVVHSRRYKSGAEYEGKRVLVVGSGNSGMEIVYDLSVSGARASVVIKSPIHILSKEMLSWGFKSVAEADYPLPVVDAILMFLSRLRMGDLSRYGIIRPPKGPIQTRFTTGRAPVLDVGTFGKIHSGDIKVVPAIEKINENSVTFSDGSEQEFDAIILATGYKSGAPGWLKDHHFIMNSDGTLKKKSPEQHWKGENGAYCAGLAKMALIGVANDAQLIANDIHMTLKK

>Kalax.0312s0011|Klaxiflora_v1.1/1-382

MVEDVAVLIVGGGPSGLATAACLTYHSIPYVILEREDCSASLWRKHSYDSLKLHTPKEMSALPLLPISDESPTFIPKAAFVDYIDAYAARFDIRPRCNRNVTKAEFDRELGKWRVEAAVGEGGVEVYAARLLVVASGENDLENVPVIEGLARFRGRVVHSRRYKSGAEYEGKRVLVVGSGNSGMEIVYDLSVSGARASVVIRSPIHILSKEMLSWGFKSVAEADYPLPVVDAILMFLSRLRMGDLSRYGIIRPPKGPIQTRFTTGRAPVLDVGTVGKIHSGDIKVVPAIEKINENSVTFSDGSEQEFDAIILATGYKSGAPGWLKDHHFIMNSDGTLKKKSPELHWKGENGAYCAGLAKMALIGVANDAQLIANDIHMTLKK

>FvH4_4g17980|Fvesca_v4.0.a2/1-397

MENNVFGNYLVVIVGAGPSGIATSALLNSMSIPNIVFEREDCCASLWKKRSYDRLCLHLAKNFCSLPMMPHSFRTATFMSKDKFADYVDKYVTRFNVNPRYCHNVESALYEEANQKWKIEVKNTEVTDGVGSLQVYYADFLVIATGENSRPVTPELPGIETFKGNVMHAQDYKCGASFKDQNVLVVGCGNSGMEISNDLAESGAHASIVVRSQVHVLSRELVRLGMVLLDYLPMNIVDRFILYLAKFSYGDLPSYGISPPVEGPFFFKALTGKTPVIDRGTVKKIRSGKIKVFSGVETIRHNIVEFKNGSIQRVDAIVMATGYRSVAHKWLKDYKVILDENDKPKNKYPGHWKGEKGVYCVGFSGKGIPGISFDSRAVANDIHQILLVKTGNAAREV

>Cucsa.084650|Csativus_v1.0/1-391

METTVIIVGAGPSGLSTAACLSKASIPYKLLEREDCSASLWRKYAYDRLCLHLPKKSSELAFMEIPDPFPNYLTKKMFVEYIDSYISKFGIEPMFWRNVEGAELDRELKKWKVRVRVRNNNKNKSINGEEGEMEEYVGRYLVVATGETAEAYMPEVEGMEKFGGGVMHSKMYKSGKGYEGKKVLVVGSGNSGMEIAYDLVNHSAATSLLVRSPIHILTRRLINLQVFLGKYLPLGFLDSLMVFLSKMVFGDLTKYGMKRPNKGPIYMKRHHGKFPIIDAGTCNKIKSGEIQVISSEIAKVESKKNVIFKDGKMVSFDDIIFCTGFTSSANSWLKDDGSLLNDDGLSKVNQPNHWKGSNGLYCVGLSKRGLFGSKFEAQEVAKDIAAQLQCI

>gene_prefix28865.t1_1|Ceratophyllum_demersum/1-379

MKKELVIIVGAGTSGLSTSACLKTLSIPHIILEREDCFASLWKRRVYDRLHLHLAKRFCELPHKPFPKCTPLYVPKENFVQYLEDYVLEFDIKPLYSRSVELAEYKSISGEWYLQTRNRNTGETEEYSCRFLVIASGENSEAFVPKIRGLDEFKGMIIHSTHYKSGKDYKDKSVLVVGCGNSGMEIADDLVNFGANTSIVIRNPFHVQSRFVTNFALVLFSFLPFNMVDTMVVMLSKLQYGDLSKYGIIRPDLGPFHLKHFYGKYPVIDAGTISKMKSGKLKVLPSIESITSTKVSFINGESHPFDAIIFATGFKRSTWLKDDNEFLNEDGIPKQTYPDYFKGKNGIYCVGLSRKGFYGASADAISVANDIKKKLLNDG

>gene_prefix25970.t1_1|Ceratophyllum_demersum/1-155

MMDTMVVMLSKLQYGDLSKYGIIRPDLGPFHLKHFYGKYPVIDAGTISKMKSGKLKVLPSIESITSTKVSFINGESHPFDAIIFATGFKRSTHKWLKDDNEFLNEDGIPKQTYPDYFKGKNGIYCVGLSRKGFYGASADAISVANDIKKKLLNDG

>Zosma02g06810|Zmarina_v3.1/1-388

MMMDDRSTEVIIAGAGPAGLATSACLTRYSIPHVVLEREQCHSSLWKMKAYDRCNLHLAKEFCQLPHMPHMSHLPTYIPRKSFIEYLDSYVDFFNIRPIYHRCVESAFYEQKAKIWSVLTKNTEEKDDRSRDLFTGKFLVVATGENSEGNIPDIKGLESFTGKMMHSSEYKTGFKFNGKKVLVVGCGNSGMEIAYDLSNHGAKTSIVVRSPFHVVTKEMIRVGMSLMRYLNCPIWLVDFLILVWTKFKFGNLSKYKIFKPAEGPMLGKAVRGRSPVIDVGTIKHIKTDKIKVTSGISNIEGSKVTFVNGEQEDFHAIIFATGYKCTTKNWFKENYFLDETGMPLQKFPHHWKGKNGVYCAGFSQKGLAGVSFDAQLIADDIANKLNDT

>TnS000915671t02|Gnetum_montanum/1-428

MAEEVETREEQGAKQSNDKKEKMKVVVKVEGAIIVGAGPSGLACAACLEREKVPFVILDRDHCIASLWHNHTYDRLRLHLPKKYCELPHAPFPESFPQLLARAQFLSYLHYYAHAHANVSLRLRLGHRVDAAHWNPASAAWHVLSTILPPPSSKATSEYVGRWLVVASGENAEKVMPNIPGMENFRGMLLHSSQYKSGRPFQGLKVLVVGYGNSGMEIALDLCHHYAKPSISVRGPVHVLPREMLGMSTFGVAMGLMKVVPVRVVDWVLLLLSRMAFGDTAVYGLRRPKDGPLLLKATTGRTPVLDVGTFHLIRTGQISVVPGIKRVTATGVVFEDDRTADFDVIVCATGYRSNVNAWLKDDGGHFSKEGLPRQEWVGKGQHWKADGNRGLYVAGLSGRGLLGSATDAHLIAHHISTKYHALHNPNSC

>XP_020595074.1|Phalaenopsis_equestris/1-119

VHLVTREIWRIALVAMKILPFSWVDLIVLTLCYMWMGNTAKYGLHRPSKGPLYLKMNTLFYPVVDAGTFSKIKSGEIEVFPEITAIEGKNVDFVNGKQRRFDAIILATGYRSAVKKWLK

>Pp6c3_9570|Ppatens_v6.1/1-434

MYPRKDSWKSGDLPVCPSVQLGSIAGESLRNRSISSKVNSKEAPQTVLIIGAGPAGLATAACLRSKYWIPSIILERANCSAPLWRYMTYDRLRMHLPKQFCQLPLRPFPAVYPKYPTKNQFIAYLEDYQRHFGISPVYNATVTSAEFSTALGLWVVIAEQKLEDNCETVTYTTRSLVVATGENAEPYMPDLFGSHKFHGVISHGSTYRNGVKYKDMKVLVVGAGNTGMEISLDLAKFGAKPTLVARSKFHVMPRDLFGLNISAFQVMLMLLKVLPVSFVDKLLVIFSRLTLGDTDHLNLVRPKEGPLKMKARTGHTPVLDVGTVAEVRNGFIKVAPAIDQLTKSGARFVNGVEEEFDAVIMATGYTSNVYEWLKIDGMSGINGFPKRPFRNGWKGGRGLYAVGFGRKGLMGCAHDAELVADDIGAHHRDNEKTY

>410626|Smoellendorffii_v1.0/1-529

MATSLLILLVFNAWVAIVASDGHTKTDSVLIVGAGISGIMAAKTLSQNGINDFVILEATERIGGRMREEAFAGGIVEIGANWVEGVHGSKVNPIWTLANKYNLTSFYTDFSNQSSNIYTKNGYVDPSTVTNETKMAEAEKEYVTNLAISKSKNGEQDISILTGQRLFGSVPQTPIEMCLEYQNYDFEFAEPPRVTSLENTHPNPTFRDFGDDEYFVADPRGYSHIVHQLAGDFLQTRNGKITDPRLLLNKVVRKIKYSKDGVKLLTEDGSTYFGKFAIVTASLGVLQSSLIKFQPVLPDWKVEALFQFDMAIYTKIFLRFPYTFWPIYPGAQFLIYCDERRGYYSTWQSLVSFQHLAKEFPGKNMIFVTVTDEESRRIEQLPDKEIKAEIMSVLRKMFGPNIPEIEEMLVPRWGSMKYFKGSYSNWPIGVSDSEFEAIQAPVETLYFAGEHTSQKYSGYVHGAYLTGIEAGKDLVACIKHKKCRKFSQEKHKDLKNSTCKEEIAKEREATRQAWTKKMDAVLASHKNGM

>GSMUA_Achr5G08640_001|Macuminata_v1/1-452

MDSASVIIIGAGMSGISAAKTLSDAGVKEILILEATNRIGGRIRNTYFADLSVETGANWIEGVHGEKQNPIWEMAQQLGLRTFRSDYSNLSSNTYKQDGGRYEKAAVEAAIDESEKIHSLGEEYSKTLPPSGRNDISISTFQRLENKIPSTPLEMIVDYYSYDYEFAEPPRVTSLQNTVPLPTFDDFGDNVYFVADERGYEILVYNLASMFLETNSHGVIVDPRLQLGKVATEIQYSGDGATVSTDDRSSYRADFVLVSVSIGVLQSDLIKFNPVLPKWKILALYEFDMAVYTKIFLKFPFKFWPDGDDTEFFLYASKRRGYYPLWQHFEKQYPGANILMVTVTDEESRRIEQQDESTTKKEAMEVLRSMFGKDIPEATDILVPRWWSNKFFRGSFSNWPLGVNRHEFDSIKAPIKRLYFTGEHTSEHYNGYVHGAYLAGLHHLHDQSHHHS

>Kalax.0005s0287|Klaxiflora_v1.1/1-471

MASTRPSWHRACTIIAYVALCCSSLLALGSAKNPSVIIVGAGMSGISAAKTLSDAGIKDFLILEATNRVGGRIHKENFAGLSIEIGANWVEGVGGKQLNPVWTMAKNLQLKTFFSNYENLSANTYQQTGLVDQAAAQKTFDLADEVSEGVTRVSTSLSAHRQDDISILAAQRLKHQVPSTPLEMAYDYYTYDYEFAEPPRVTSLQNTEPLATFANFGEDLYFVADPRGYEAVVHEVVVEIRHSPSGVILKTEDGSVYRSEYAMVSVSVGVLQTKLIDFVPDLPPWKLLAIYQFDMAVYTKIFIKFPTVFWPTGNGTEFFLYAHEKRGYYPIWQQFEAQYPGSNVLLVTVTDDESRRIEQQPDSETQEEIMGVLRDMFGKNIPQPTDILVPKWWSDRFYKGTFSNWPIGVSRYEYDQLRAPVGRVYFTGEHTSEHYNGYVHGAYLAGIDSTNILLNCLKKKMCHYRVAAKGT

>Aqcoe3G119000|Acoerulea_v3.1/1-510

MGKKWGFVSVCCSMMIMGGTNIGATLVMVLVALFSLLSFEPMVVEARPPTVIIVGAGMSGISAAKTLSDAGIKDILILEATDRIGGRMHKTQFAGLSVEIGANWVEGVNGAEVNPIWPMVNKLKLKTFLSDYDNLTSNTYKQAGGLYDGATSKAAFEASDELTEFTTKTSISLTAQKKEDISILAAQRLKHQVPSTPIEMAIDYYNYDYEFAEPPRVTSLQNTEPLATFANFGEDLYFVADPKGYESVVYDIAGQFLTVDKTGKITDPRLKLNKVVIEIQHSASGVIVKTEDGSLYRSAYVMVSASVGVLQTNLIDFQPDLPQWKILALYQFDMSVYTKIFLKFPYKFWPSGNGTEFFLYAHEKRGYYPIWQQLEREYPGANVLLVTVTDDESRRVEQQPDSETKAECMKVLQDMFGKNIPEATDILVPRWWSNKFFKGTFSNWPIGVTRYEYDQIRAPVGRVYFTGEHTSEHYNGYVHGAYLAGIDSANILINCAKRRMCKYHIMPKGT

>VIT_201s0127g00750|Vvinifera_v2.1/1-477

MGIRRTRLMLASLIVIMGTLLGCPGAATAKRAPTVIIVGAGMSGISAAKTLSDAGIKRILILEATNRIGGRMYKANFSGVSVELGANWVSGVGGPQVNPVWIMANKLRLKSFLSNFLNLSSNTYKPEGGVYEESVARKAFEVAEQVVEFGTKVSKDLAARKQPDISILTSQRLKNYFPKTPLEMVIDYYLCDFESAEPPRATSLLNSEPSSTYSNFGEDSYFVSDPRGYESVVHYVAQQFLTTNAAGQITDPRLQLKKVVTEISRSPRGVAVKTEDGLVHRADYVIVSVSLGVLQNDLIKFHPSLPQWKILALDQFNMAIYTKIFLKFPYKFWPSGNGTEFFLYAHEKRGYYPFWQHLEREFPGENVLLVTVTDDESRRLEQQSDSETKAEIMAVLRNMFGKQIPEATDILVPRWLSNRFFKGSYSNWPIGVSHHQFNQIKAPVGKVYFTGEHTSAAYYGYVHGAYFAGFLLFVIGL

>Bradi3g20677|Bdistachyon_v3.2/1-497

MMKPSTFSALAAVILLVVTQHASVAAGRGPRVIIVGAGMSGISAGKRLADAGVRDVLILEATGRVGGRMHKHNFGGINVEIGANWVEGVEGKKVNPIWPMVNATLNLRNFLSDFDSVVSNVYKENTCRREWDRADEVEELGGKLASQMDPSGRDDISILAMQRLFNHQPNGPTTPVDMALDYFRYDYEFAEPPRATSLQNTEPLPTAADFGEDNHFVADQRGFEAIIYHIARQYLSSDRKSGNIVDPRLKLNKVVREISYNRKGVVVRTEDNSAYSGDYVIVSTSLGVLQSDLIQFKPQLPAWKIIAIYRFDMAVYTKIFLKFPTKFWPVGEGKQFFVYASSRRGYYGMWQSFEKEYPGANVLMVTVTDQESRRIEQQPDNQTKAEAVAVLRKMFPDRHVPDATDIYVPRWWSDRFFKGSYSNWPIGVNRYEYDQLRAPVGRVFFTGEHTSEHYNGYVHGAYLAGMDSADILMNSIFNKVGFKVRPKDDHQIIAEAK

>Sobic.001G472000|Sbicolor_v5.1/1-510

MKTSSLVVVALAAVVLFAAHDASLAGASSSSSPRVIIVGAGMSGISAGKQLWESGVRDLLILEATERVGGRMHNHNFGGINVEIGANWVEGLGGDNLNPIWPIVNDTLKLRTFYSDFDSVVGCVYKENGGGLYDEEYVQKKMDRAYEVQELGANLTKKMRPNGQDDISLLAMQRLFNHQPNGPATPVDMALDYFIYDYEFAEPPRVTSLQNTQPTPTNADFGEDNYFVADQRGFESIIHAIGSSYLSTDANGKLSDRRILLNKVVRQIAYNKEGVVVKTEDGSSYSADYVVVSTSLGVLQTDLIKFTPQLPFWKIAAIYRFDMGAYTKIFLKFPSKFWPVGEGKQFFVYASSRRGYYGMWQSFEREYPGANVLLVTVTDDESRRIEQQSDNHTKAEVAAVLRDMFPDADVPAPEEIDIYVPRWWSNRFFKGSYSNWPIGVSRYEYDQLRAPVGRVYFTGEHTSERYNGYVHGAYLAGIDSANILINHIFKNEEYKVRGKYEPDDQIAKAK

>HORVU.MOREX.r3.7HG0747970|Hvulgare_Morex_V3/1-495

MKPSTAIVLVLGVVAQYVALATAAGPKVIIVGAGMSGISAGKRLSEAGITDLVILEATDRIGGRIHKEKFAGVNVEMGANWVEGVNGDEMNPIWTMANGTGGLNLRTFRSDFDHLAGNTYKQDGGLYDEKVVEKIIERMEEVEESGSKLSGTLHASGQQDMSVMAMQRLNDHMPSGPATPVDMVIDYYQHDFEFAEPPRVTSLQNTQPLPTFNNFGDDVYFVADQRGYESVVYHVAGQYLKTDRKSGAITDPRLKLNTVVREITYFPSGVAVRTEDNKVYRADYVVVSASLGVLQTDLIRFKPQLPSWKIVSIYQFDMAVYTKIFLKFPKRFWPEGPGKEFFLYASGRRGYFPVWQQFEKQYPGSNVLLVTVTDDESRRIEQQSDNQTMAEAVAVLRKMFPGKEVPDATEILVPRWWSNRFFKGTFSNWPIGVNRYEYDLIRAPVGRVYFTGEHTSEKYNGYVHGAYLAGIDSADILINCAKKKMCKYNVKGKHD

>Potri.015G074600|Ptrichocarpa_v4.1/1-483

MKKFLGMVLVLLFHSFLSLASASQTPTVIIIGAGMSGISAAKTLQDAGIRDILILEATDRIGGRVMKTQFSGYAVEMGANWLFGGGPVHNPVLEMAKKVKLKTSLNDYDNLTSNTYKQDGGLYPKKLVEAVDKVAVARDDFCAEFSTLLTKKVKNDVDISILAGQRLFKQEPKTPLEMVIDYYHNDYEDGEPPKVTSLKHTYPRNEFVDHGEDPYFVADPRGFEIVVQYLAKQFLSSLKSDPRLKLNKVVREIIYSKNGVAVKTEDGSIYKAKYAIVSVSVGVLQTDLIDFRPKLPLWKRLAISDFSMTIYTKIFLKFPYKFWPSGPGTEFFLYTHVRRGYYPLWQHLENEYPGSNILFVTVTAEESRRVEQLSDQEVEAEVMVVLKTLFGNNIPKPEDILVPRWGLNRFYKGSYSNWPDKYNQNRHDQLGDPVGPVYFTGEHNSNKYIGYVTGAYFTGIDTANDLLGCIKNQTCRGYNSQHM

>Potri.012G079500|Ptrichocarpa_v4.1/1-483

MTSASPSPTVIIIGAGMSGILAAKTLHDSGIQDILILEANSKIGGRIHSVQFRGHTVELGANWVIGGGPRSNHLYEIASKLNLKTYLSDYGNISANIYKQEGGLYPKHIVSAALEVAETRDQFCTSFSTRLSAPGHDRDDVSILVSQRLFKEVPTTPLDMVIDYFYNDYEDAEPPRVTSLKNTIPRYEFLDFGDQTYFLADSRGFESILIYIAKQFLSHKHEVIRDQRLKLNKVVREINYSKSGVQVKTEDGSVYQAKYVIVSVSVGVLQSDLIVFKPHLPQWKTQAIYEFDMAVYTKIFLRFPYKFWPSGPETEFFLYAHEKRGYYPIWQHLETEMPGSNILFVTVTDEEAKRIEQQQDIKIQEEIMDVLKKMFGNDIPEPDEILIPRWWSNRFFKGSFSNWPIGYSQRRHMQLKEPVGRIYFSGEHTYSRYLGYADAAYFAGIETANHLIRCIKHEKYCNGYDRHSTIDRLHNEDPYDPDA

>Adc06547|Adiantum_capillus-veneris/1-515

MECLVWLLMSCTSLLSLSASSSENPTVIIIGAGMSGIMAAKTLSERGINDFLILEASDRIGGRMLHTNFSGYVIEVGANWVEGVGGQQENPIWTLAKKYDLRVSYSDYNNISANTYNEEGELLQLSKTKPHFDLADASFNYSNALSFSLNAYSEEDVSIATAQRLFGVIPTSPLDMAIDYFYYDFEIAEPPRITSLKNVLPNPTFDYYGDSEYFVCDKRGYSYLPEQLAREFLKHDNEKFIDSRLKLQKVVRQIKYSPHGVHISTEDNSTYHASYAILSVSLGVLKSRLITFKPDLPYWKIKTFYQFDMSIYTKIFLKFPYKFWPTGPGTEFFLYADDKRGYYPFWQHLENEYPGANILFVTVTDDEARRIEQQSNKVTKEEIMEILKKLFGEGIPEAEDVLVPRWWKNRFYGGSYTNWPIGVSGADFDRMKVPVGPLYFTGEHTSEKFNGYVHGAYLAGIATAETLLDCINHGKCREVTSDGLPWAHQNNSQADIQANKTCTGVCCKDVQQVRL

>Mvestita_S9g05486-RA|Marsilea_vestita/1-549

MKQSNYNWRFIPLLLLLLPLTLSSIPTTQQASTHEVIIIGAGMSGIMAARTLSKHGITDFLILEATNRIGGRMHQTTFAGHVIEIGANWVEGVGGEKENPIWTLAKEYKLNGTFSNFDNFSANTYNEEGELLPLSVTTPHYDLTEASYNYSNSLSVSLSDHNEEDISIATAQRLFGFIPTSPLDMVYDWAFYDFEIAEPPRVTSLKNVQPNPTFIYYGENEYFVHDPRGYVYILEQLAKGFLKHNENGDIIDERLQLNKVVRKVKYTSKGATITTEDNKTYNAKYVIISVSLGVLKSQLIQYTPDLPYWKIKTFYQFDMSIYTKIFLKFPYTFWPIGSGTEFFLYADPKRGYYPFWQHLESEYPGSNILFVTVTDDESRRIEQQSDNVTKAEIMVVLRKMFGDNIPEAEDILVPRWWTDRFHGGSYTNWPIGVTEADFDRMKDPVGPLYFTGEHTSAQFNGYVHGAYMAGIATANTLLDCIKHGKCKEITSDDVPWEQNEPTNVDKNLTRTCNGTCCQEIQQERETLREYYVNSIQSLQHALDLKCGKH

>AUR62030978|Cquinoa_v1.0/1-486

MRVLARANIIMLKHFFLFPFLLASAYALSSSPSVIIVGAGMSGISAAKRLQDAGIKDFLILEATDRIGGRIKKTQFAGHTVEMGANWLHGVGGPKRNPLYEMSKKIHLKKFYSDFNNVSLNTYKQEGGKYKENEVDAAFSKAEKTLDYGEKLSKVLNSKNNGDDMSILSLERLQKTDPRSTLEKMVDFFTFDGEQAEAPRVTSLRHVLPMTESHDFGENAYFVADSRGFESLVHLIAKQFLSYKNGAINDPRVKFNQVVTEIDQSKGGVTVRTEDGKQYHAKLVILSPSLGVLQSELISYKPELPLWKRRAIAEFSIGVYTKIFLKFPRTFWPVGNSTEFFFYVHERRGYYAIWQHLDNEYPGSNVMFVTLTDDEAIRVEQQSDEETKAEAIEVLRKMFGKNIPDATHIMIPRWKSDRFYRGCFTNWPVGYPQERHDQLRAPFGKIYFTGEHTHPKLFGYADGAYYSGADTAKDVIKCLRNKSCSG

>evm_27.TU.AmTr_v1.0_scaffold00025.360|Atrichopoda_v1.0/1-509

MDIPSLSSASVIIIGAGISGLGAAKVLSEKGIKDFVILEASDRIGGRIHKEDFCSLKVETGAGWIQGVGGKELNPVWELAQKLGLRTCFSDYSNARFNIYDQSGNVFPRSLASEAYERAVHLANERLRCEEAEVGQCATEEAPLAPSTPMELAIDYILHDFEMAEVEPIATYTEFGEREVLVADARGYDHLLYKMAQSFLTTREGHILDPRLKLNKVVREMEYGRQGVSVRTEDGYIYRGRYAILTVSLGVLQSDLITFRPPLPKWKQDAISKCEIIIYTKIFLKFPFKFWPCGPGTEFFLFAHSKRGYYTFWQHMENAYPGSNMLVVTVTNDESKRIENQNDEETLMEIMEVLRSMFGPKVPMAESILVPRWWNNRFQRGSYSNYPILSSPSDFRDIKAPVGPIFFSGEHTSEKFNGYVHGGYLSGTDTANSLLETMKTKSMALDSQRASRSSEDMAWGVAKRHVVHPWLFITDNLALNHSASSCLHKWDFAQQLFPSGKLAVDEAIK

>Eucgr.G03359|Egrandis_v2.0/1-496

MDPASRSSVIIVGAGISGICAAKVLAENGVEDILILEATDRIGGRIRKEAFGGVSVELGAGWIVGVGGKVPNPVWELAAKSSLRTCFSDYSNARYNIYDRSGKIFPSGLAADSYKKAVDSAIENLRNQQETDRSGDFSRDAEPPSTPKTPIDLAIDFILHDFEMPEVQPISTFLDYGEREFLVADERGYEDLLYKMAQDFLFTSEGKILDSRLQLNKVVREIQHSRSGVTITTEDGCVYDANYAILSVSIGVLQSDLISFRPSMPRWKMEAIEKLDVMVYTKIFLKFPYKFWPCGPEKEFFIYAHERRGYYTFWQHMENAYPGSNILVVTLTNGESKRVENQPDEETLKEAMEVLRDMFGPDIPNATDILVPRWWNNRFQRGSYSNYPAIINHQVVQSIKAPVGRIFFTGEHTSERFNGYVHGGYLAGLDTSKALLEEMRKEKERNSQNQSFLLEPLLALTLTQTDSSVSGSVQHKSDIPNHLHLSGKLSIPGPIS

>Glyma.15G276500|Gmax_Wm82.a6.v1/1-501

MDSPSRPSVIIVGAGISGIAAAKVLAENGIEDLVILEASDRVGGRIRKESFGGVSVELGAGWIAGVGGPQPNPVWELGVQFGLRTCFSDYSNARYNIYDRSGNIIPSGIAADSYKKAVDSAIEKLRKLEEEEATAYGDDHSNNIKNNETKPPSTPETPIELAIDFILHDFEMAEVEPISTYVDFGEREYFVADERGYDYLLYKMAEEFLFTSKGRILDNRLKLNKVVRELQYSKSGVTVKTEDGCVYETNYVILSVSIGVLQSDLLAFNPPLPGWKVQAIDKCDVMVYTKIFLKFPYKFWPSGPEKEFFIYAHERRGYYTFWQHMENAYPGSNILVVTLTNGESKRVEAQLDEETLREAMAALRDMFGSNIPDAIDILVPRWWNNRFQRGSYSNYPIISNHKVFHDIKAPVGRIFFTGEHTSERFNGYVHGGYLAGIDTSKALLEEMRKEKESQTLLLEPLLALTGPLTMSKPETVSNIHKCDIPTQLYLSGKLGIPEAIL

>GWHPCBIR017637|Santalum_yasi/1-487

MDSPTRRSVIVVGAGISGIAAAKVLSENGSQDVVILEASDRVGGRIRKERFGGVSVELGAGWIAGVGGPESNPVWELAHDSGLRTCFSDYSNAPYNIYDRSGRIFPSGVAADSYKKAVDSAIRRLSDSPELESPFSPETPIELAIDFILHDFEMAEVEPISTYTDFGEKEFLVADERGYDHLLFKMAETFLCTSEGKISDDRLQLNKVVRELQQSRNGVTVITEDGCIYEANYVILSVSIGVLQSDLISFKPPLPRWKTEAIERCDVMVYTKIFLKFPYKFWPSGAEKEFFIYAHERRGYYTFWQHMENAYPGSNILVVTLTNGESKRVEAQSDEQTLKEAMDVLKDMFGPNIPNATDILVPRWWNDRFQRGSYSNYPILCNYQAVQEIKAPVGRVFFTGEHTSERFNGYVHGGYLSGIDTSKALLEEMKEERERKNESQTFLLEPLLALTGSITLTQTETVSPLHKCDIPRHPFLGSKLGIPEAIL

>Sapof.08G230100|Sofficinalis_v1.1/1-492

MDSSKRSSVIIVGAGVSGISAAKHLAENGLEDIVILEASDRIGGRMRKQAFGGVWVEVGAGWIAGVGGSHINPVWELANHTSLRTCFSDYTNARFNIYDRRGDIYPSGIASELYKKAVESAMQRINNHAHNPSHCPKTPIELAIDFILHDFEMPEVEPISTYVDFGDREYLVADERGYEYLLHNMAHTFLLTCDGHILDNRLKLNTVVREIQHSRNGVTVYTEDGSTYEANYLILSVSIGVLQSRLISFQPPLPRWKTEAIDKCDVMVYTKIFLKFPYMFWPSGQEKEFFIYAHERRGYYTFWQHMENAYPGSNILVVTLTNDESKRVEAQPDEETLREATEVLRDMFGPNIPDATDILVPRWWNNRFQRGSYSNYPIFSNNQLVRDIKVPVGRIFFSGEHTSERFSGYVHGGYFAGIDTSKALLEEMKSESEGRKKKKKKKQTFMLEPLLALRESLTLSQTETVSNLHKYELPLPRQIFLGSNLGMPKPIV

>FvH4_7g01450|Fvesca_v4.0.a2/1-491

MDSPHRSSVIIIGAGVSGLSAAKRLIENGVEDVVILEASDRIGGRIRKQEFGGVSVELGAGWIVGVGGEQLNPVWELAEKTNLRTCLSDYSNARFNIYDRSGNLIPSGVAADSYKKAVDSAIEELRKLEADGGDVSKVTEPPSTQKTPIELAIDFTLHDFEMAEVEPIATYKDFGGQEFLVADERGYEHLLYKMADDFLFTCEEKILDSRLSLNKVVRELKHSRDGVTVMTEDGCFYQANYVILSASIGVLQSDLISFTPPLPKWKTEAIEKLDVMVYTKIFLKFPRKFWPCGPGKEFFIYAHERRGYYTFWQHMENAYPGSNILVVTLTNGESKRVEAQCDEETVKEAMGVLRDIFGPNIPEATDILVPRWWNNRFQRGSYSNYPIISNHRVVRDIKAPVGCIFFTGEHTSEKYSGYVHGAYLTGIDTANALLEEMGRQEERSSENKTSLSKPELALTSSLNLTQKDAVSNLKCDVPTQLYLGSKQPKLY

>GWHPBFSI011823|Chloranthus_spicatus/1-522

MGLSSNPSVIIIGAGISGISAAKVLAENGVDDLLILEASDRIGGRIRKQDFGGLSVETGAGWIQGVGGKESNPVWELAAQSALRTCFSDYSNARYNIYDRSGKIFPSGLAADSYDRAVSSAIEELKNQEANLRGISGALVSEPICVPKTPIELAIDFILHDFEMAEVEPISTYTDFGEREFLVADERGYEFLLYKMAATFLKTVDGNIVDGRLKLNKVVRELRYSRNGVTVKAEDGFCYKARYVILSASIGVLQSDLIKFTPPLPKWKMEAISKCDVIVYTKIFLKFPYKFWPCGTGSEFFLYAHEKRGYYTFWQHMENAYPGSNILVVTLTNGESKRVESQRDEETMKEAMGVLRDMFGSDVPDADEILVPRWWNNRFQRGSYSNYPIFATQQDFTHIKAPVGSIFFTGEHTNERFNGYVHGGYLSGIDTSNSLLEVMKKEKEKEKNRSHHRVLKSPFLQPLIALTESLTLTPHDAVSHLQKWDIARRLFLSGKLVIEESQQIKPRQWTSPTSNFNGPQGW

>evm_27.TU.AmTr_v1.0_scaffold00068.6|Atrichopoda_v1.0/1-573

MASACFPASSGSFCGEMVGIPAPRYPETRVGVKVWQLGARCSLEEKVSDMRVNAPKGLFPPEPEHYRGPKLKVAVIGAGLAGMSTAVELLDQGHEVDIYESRQFIGGKVGSFQDRRGNHIEMGLHVFFGCYNNLYRLMKKVGADKNLLVKDHTHTFINKGGQVGELDFRFPIGAPVHGIRAFLSTNQLKTIDKARNAFALARSPVVRALIDPDGAMQDIRNLDNVSFSDWFLSKGGTRMSIQRMWDPVAYALGFIDCDNISARCMLTIFGLFATKTEASLLRMLKGSPDVYLSGPIRKYITDKGGRFHLRWGCREILYDYLEDGGAYVTGLSISKATEKQIVKADAYVAACDVPGIKRLVPSKWRENEFFDNIYKLVGVPVITVQLRYNGWVTELRDLQLSRQLRRAAGMDNLLYSPDADFSCFADLALSSPEDYYIEGQGSLIQAVITPGDPYMPLTNDKIIERIQKQVLSLFPSAQGLEVIWSSVVKIGQSLYREGPGMDPFRPDQRTPVQNFFLAGSYTKQDYIDSMEGATLSGRQAASYICSSGEELASLRKKIAQGTAVGAKIEPCIA

>evm.TU.AsparagusV1_03.1606|Aofficinalis_V1.1/1-587

MASASCSFACVPAPSFGLGSKETVMVSMKPSRGFSIRSALDTKVSDMGVNAPKGLFPPEPEHYRGPKLKVAIIGAGLAGMSTAVELLDQGHEVNIYESRQFIGGKVGSFVDKRGNHIEMGLHVFFGCYNNLFRLMKKVGADENLLVKDHTHTFVNRGGEIGELDFRFPVGAPIHGIRAFLSTNQLKPYDKARNAVALALSPVVRALVDPDGAMQDIRNLDDISFSDWFMSKGGTRMSIQRMWDPVAYALGFIDCDNISARCMLTIFSLFATKTEASLLRMLKGSPDVFLSGPIRKYITDKGGRFHLRWGCREILYEKSPNGDTYVTGIAMSKATNKKIVKADVYVAACDVPGIKRLIPSQWRKWDLFENLYKLVGVPVVTVQLRYDGWVTELQDLEKSRQLRRAVGLDNLLYTPDADFSCFADLALTSPEDYYIEGQGSLIQAVLTPGDPYMPLPNKEIIERVQKQVLDLFPSSQGLEVLWSSVVKIGQSLYREGPGKDPFRPDQKTPVKNFFLAGSYTKQDYIDSMEGATLSGRQAAAYICSAGEELAALRKQIAALHPEQEPNTKDSNTSDEIRSGIWHNGNGNN

>Asa4G01247.1|Allium_sativum/1-571

MASSTFTSIYAPFTLARSEKKRVFLKDFSIRAALDTKVSDMSVNAPKGLFPPEPEHYRGPKLKVAIIGSGLAGMSTAVELLDQGHEVDIYESRPFIGGKVGSFVDKRGNHIEMGLHVFFGCYNNLFRLLKKVGAEENLLVKDHTHTFVNRGGEIGELDFRFKFGAPLHGIRAFLATNQLEPYDKARNALALALSPVVRALIDPDGAMQDIRNLDSISFSDWFMSKGGTRKSIQRMWDPVAYALGFIDCDNISARCMLTIFSLFATKTEASLLRMLKGSPDVYLSGPIRKYITDKGGRFHLRWGCREILYDKSSNGDTYVKGIAMSKATNKKIVKADVYVAACDVPGIKRLIPSQWREWELFENIYKLVGVPVVTVQLRYNGWVTELQDIEKSRQLARAVGLNNLLYTPDADFSCFADLALSSPADYYIEGQGSLIQAVLTPGDPYMPLPNEEIIERVQKQVLDLFPSSQGLEVIWSSVVKIGQSLYREGPGMDPFRPDQKTPVKNFFLAGSYTKQDYIDSMEGATLSGRQAAAYVCSSGEELAALRKVIAAEQTEHQSIEGSNVADELSLV

>FvH4_6g03420|Fvesca_v4.0.a2/1-569

MASWVLLPVAPLTSRCLVAPVRSQRSSSISVRSSLDTNVSDMSVNAPKGLFPPEPEHYRGPKLKVAIIGAGLAGMSTAVELLDQGHEVDIYESRPFIGGKVGSFVDKRGNHIEMGLHVFFGCYSNLFRLMKKVGAEENLLVKDHTHTFVNKGGQIGELDFRFPIGAPIHGILAFLSTNQIKTYDKARNALALALSPVVKALVDPDGALSDVRDLDSISFSDWFMSKGGTRTSIQRMWDPVAYALGFIDCDNISARCMLTIFTLFATKTEASLLRMLKGSPDVYLSGPIRKYIIDKGGRFHLRWGCREILYDKSADGETYVAGLAMSKATNKQTVKADAYVAACDVPGIKRLLPSQWREWEFFNNIYELVGVPVVTVQLRYDGWVTEMQDLERSRQLKQALGLDNLLYTPDADFSCFADLALTSPEDYYIEGQGSLLQCVLTPGDPYMPLPNEEIIAKVTKQVLTLFPSSQGLEVTWSSVVKIGQSLYREGPGKDPFRPDQKTPVKNFFLAGSYTKQDYIDSMEGATLSGRRASAYICNAGEDLVELRKKLASQEGYTKAANTTDELSLV

>GWHPBFSI007895|Chloranthus_spicatus/1-576

MASPTGFPATSSCFRSEILGCSVRHRPILLSAGRIQLRSEVRASLDTNVSDMSVNAPKGLFPPEPEHYRGPKLKVAIIGAGLAGMSTAVELLDQGHEVDIYESRTFIGGKVGSFVDRRGNHIEMGLHVFFGCYNNLFRLMKKVGANENLLVKDHTHTFINKGGQVGELDFRFPLGAPLHGIQAFLTTNQLKTYDKARNAIALALSPVVRALIDPDGAMKDIRNLDNISFSDWFLSKGGTRMSIQRMWDPVAYALGFIDCDNISARCMLTIFSLFATKTEASLLRMLKGSPDVYLSGPIRKYITDRGGRFHLRWGCREILYDKSADGDTYVTGIAMSKATSKKIVKADAYVAACDVPGIKRLVPQQWREWDLFDNIYTLVGVPVVTVQLRYSGWVTELQDLELSRQLRQAVGLDNLLYTPDADFSCFADLALTSPEDYYIEGQGSLIQAVLTPGDPYMPLPNDEIIRRVQKQVLALFPSSQGLDLLWSSVVKIGQSLYREAPGKDPFRPDQKTPVKNFFLAGSYTKQDYIDSMEGATLSGRQAAAYICNAGEDLAVLRKKLAANSEDAIVVDQLSLV

>TESI_002751-RA|Tetracentron_sinense/1-584

MASPSYFPATSIPGSRSGFHGEITGLFFSGRRPMVRLKPQRSEIRSSLDTNVSDMSFNAPKGLFPPEPEHYRGPKLKVAIIGAGLAGMSTAVELLDQGHEVDIYESRPFIGGKVGSFVDRRGNHVEMGLHVFFGCYNNLFRLMKKVGAEKNLLVKDHTHTFINKGGQVGELDFRFPVGAPLHGISAFLSTNQLKTYDKARNALALALSPVVRALIDPDGAMRDIRNLDNISFSDWFLSKGGTRMSIQRMWDPVAYALGFIDCDNISARCMLTIFSLFATKTEASLLRMLKGSPDVYLSGPIRKYITDKGGRFHLRWGCREILYDKSVNGDTYITGIAMSKATNKKTVKADAYVAACDVPGIKRLVPPQWREWEFFDNIYKLVGVPVVTVQLRYNGWVTELQDLERSRQSRQAVGMDNLLYTPDADFSCFADLALTSPEDYYIEGQGSLLQCVLTPGDPYMPLPNDEIIKRVAKQVLALFPSSQGLEVTWSSVVKIGQSLYREAPGKDPFRPDQKTPVKNFFLAGSYTKQDYIDSMEGATLSGRQAATYICSAGEELIALRKKLAANEAQQVLKTAGITDKLSFV

>KAI5398377.1|Pisum_sativum/1-576

MASLFHCPATRLGDSSGFFLPTRSTRFLKIHTRKPHLRCSLDSNVSDMSINAPKGLFPPEPEHYRGPKLKVAIIGAGLAGMSTAVELLDQGHEVDIYESRSFIGGKVGSFVDKRGNHIEMGLHVFFGCYNNLFRLMKKVGADNNLLVKDHTHTFVNKGGQIGELDFRFPVGAPLHGIRAFLTTNQLNTYDKARNAVALALSPVVRALVDPDGALRDIRNLDSISFSDWFMSKGGTRTSIQKMWDPVAYALGFIDCDNISARCMLTIFALFATKTEASLLRMLKGSPDVYLSGPIQKYITDRGGRFHLRWGCREILYDKSADGSTYVTGLSMSKATDKKIVEADAYVAACDVPGIKRLIPPEWREKELFNNIYELVGVPVVTVQLRYNGWVTELQDLELSRQLRKATGLDNLLYTPDADFSCFADLALASPEDYYIEGQGSLLQCVLTPGDPYMPLPNEEIISRVAKQVISLFPSSQGLEVTWSSVVKIGQSLYREGPGKDPFRPDQKTPVKNFFLSGSYTKQDYIDSMEGATLSGRQTSAYICDAGEELVALRKELVAQSKDDIKYTNTKDELSLV

>GWHPBAUK037001.1|Macadamia_integrifolia/1-582

MASSTCFPATFTNFRYEAMGLTVRPSLILHRGKVQLKTERLEVRSSLDTNVSDMGVNAPKGLFPPEPELYRGPKLKVAIVGAGLAGMSTAVELLDQGHEVDIYESRSFIGGKVGSFLDKRGNHIEMGLHVFFGCYNNLFRLMKKVGADKNLLVKDHTHTFINKDGQIGELDFRFPFGAPIHGIRAFLSTNQIKTYDKARNALALALSPVVRALVDPDGAMRDIRNLDNISFSDWFMSKGGTRMSIQRMWDPVAYALGFIDCDNISARCMLTIFSLFATKTEASLLRMLKGSPDVYLSGPIKQYIIDKGGRFHLRWGCREVLYNKTANGDTYITGLAMSKATNKKTVKADVYVAACDIPGIKRLLPSQWREWELFDNIYKLAGVPVVTVQLRYNGWVTELQDIEKSRQLRQAIGLDNLLYTPDADFSCFADLALTSPEDYYIEGQGSLLQCVLTPGDPYMPLPNDEIIRRVEKQVLDLFPSSQGLEVTWSSVVKIGQSLYREAPGKDPFRPDQKTPVKNFFLAGSYTKQDYIDSMEGATLSGRQAASYICDAGEELAALRKLLDSKEAQVREAGNIQDELSLV

>GWHPCBIR000309|Santalum_yasi/1-575

MASLICFPTCGSGSRFSGDTAGALLWGMRPIPRLKNRRLGVWSSLDTSVSDMRVNAPKGLFPPEPGHYRGPKLKVAIIGAGLAGMSTAVELLDQGHEVDIYESRPFVGGKVGSFVDKRGNHIEMGLHVFFGCYNNLFRLMKKVGADKNLLAKDHTHTFVNKGGEIGELDFRFPIGAPLHGIFAFLSTNQLKTYDKARNAVALALSPVVKALIDPDGAMRDVRNLDSISFSDWFLSKGGTRMSIQRMWDPVAYALGFIDCDNISARCMLTIFSLFATKTEASLLRMLKGSPDVYLSGPIRKHITDKGGRFHLRWGCRKILYDRSASGDTVVTGIAMSKATSKKIVKADAYVAACDVPGIKRLLPPQWRELEYFDDIYKLVGVPVVTVQLRYNGWVTELQDLEQQRQSREAVGLDNLLYTPDADFSCFADLALTSPEDYYVEGQGSLLQCVLTPGDPYIPLPNDEIIKRVAKQVLVLFPSSRGLEVTWSSVVKIGQSLYREAPGRDPFRPDQKTPVKNFFLAGSYTKQDYIDSMEGATLSGRQASAYICDAGEELLALRKMLASDGTQVPKEMTLVP

>XP_020578623.1|Phalaenopsis_equestris/1-566

MASAASCSSAYVSAASFESSKGSRLSSRIRASLDSKVSDMGVNAPRGLFPSEPEHYRGPKLRVAIIGSGLAGMSTAVELLDQGHQVDIYDSRPFIGGKVGSFVDKHGNHIEMGLHVFFGCYNNLFRLMKKVGADGNLLVKDHTHTFVNKGGDIGELDFRFPVGAPIHGIRAFLATNQLKPYDKARNALALALSPVVRALVDPDGALQAIRNLDHVSFSDWFLSKGGTRASIQRMWDPVAYALGFIDCDNISARCMLTIFALFATKTEASLLRMLKGSPDVYLSGPIKKYITNRGGRFHLRWGCRQILFEKSDGGDTYVTGLAMSKATEKNIVKADVYVAACDVPGIKKLIPAQWREWDMFNNLFELVGVPVITVQLRYNGWVTELQELELSRQLRQAVGLDNLLYTPDADFSCFSDLALSSPEDYYREGQGSLIQAVLTPGDPYMPLPNAEIIRRVQKQVLDLFPSSQGLEVLWSSVVKIGQSLYREAPGKDPFRPDQKTPVKNFFLAGSYTKQDYIDSMEGATLSGRRAAAYICGSGEELLLLRKKLVADKASGFAEAPNTLSLV

>gene_prefix08383.t1_1|Ceratophyllum_demersum/1-594

MAASPSSSSSTFFYSNWFGRRSEMRNDVLGFSPPGINTFTERKLHLNKRREKIRSSLDTNVSDMATNAPKGLFPPEPKPYRGPKLKVAIIGSGLAGMSTAVELLDQGHEVDIYESRPFIGGKVGSFVDKHGNHIEMGLHVFFGCYSNLFRLMKKVGADENLLVKDHTHTFINRGGIVGELDFRFPIGAPIHGIQAFLSTNQLKTYDKARNALALALSPVVKALIDPDGALRDIRNLDNISFSDWFLSKGGTRASIQRMWDPVAYALGFIDCDNISARCMLTIFSLFATKTEASLLRMLKGSPNVYLSGPISKYIIDRGGRFHLRWGCRQILYSKSSSGETCVTGIAMSKATEKKIVKADVYVAACDVPGIKRLIPQQWKEWDFFNNIFKLVGVPVVTVQLRYDGWVTELRNLDHSRQLKKAVGLDNLLYTPDADFSCFADLALTSPEDYYREGQGSLLQAVLTPGDPYMPLPNEEIIDKVQKQVLELFPSSRGLKVLWSSVVKIGQSLYRESPGSDPFRPDQRTPVKNFFIAGSYTKQDYIDSMEGATLSGRQAAAYICDSGEELAALGLKLSASEYQHVNKDTATVSDNLSLV

>Pn3.733|Piper_nigrum/1-575

MASCSSACSLAAAAFPVTGRTSFGWSFRRPALPSRPGVRSALDEKVSDMRVNAPKGLFPPDPEPYKGPKLKVAIIGAGLAGMSTAIELLDQGHEVDIYESRPFIGGKVGSFVDRKGNHIEMGLHVFFGCYSNLFRLMKKVGAEANLLVKDHTHTFVNKGGKIGELDFRFPIGAPLHGIRAFLSTNQLETYDKARNAVALALSPVVKALVDPDGAMRDIRNLDSVSFSDWFISKGGTRASIQRMWDPVAYALGFIDCDNISARCMLTIFALFATKTEASLLRMLKGSPDVYLSGPIRKYITDRGGRFHLRWGCRRILYDNYANGDTCVTGIAISKATSKKIVKADAYVAACDVPGIKRLLPRDWRKFKFFDDIYRLVGVPVVTVQLRYNGWVTELQDLERSRQLRRAAGLDNLLYTPDADFSCFADLALSSPEDYYIEGQGSLIQAVLTPGDPYMPLLNEEIIRRVEKQVLALFPSSQGLEVLWSSVVKIGQSLYREAPGVDPFRPDQKTPIQNFFLAGSYTKQDYIDSMEGATLSGRQAASYICSSGDELAALRKFITVKEAEKDVATADEPRLV

>g36437.t1_1|Euryale_ferox/1-576

MACLSIPTAYTARRSEFCGESLGLPALFLRGGKSGVRCDLGTKISDMSVNAPKGLFPPEPERYGGPKLKVAIIGAGLAGMSTAVELLDQGHEVDIYESRSFIGGKVGSFMDKRGNHIEMGLHVFFGCYNNLFRLLKKVGADQNLLVKEHVHTFVNKGGQIGDLDFRFPIGAPIHGIRAFLTTNQLQVYDKARNAFVLATSPIVKALFDPEGAMKDIRDLDSISFSEWFLSKGGTRLSIQRMWDPVAYALGFIDCDHISARCMLTVFGFFATKTEASLLRMLKGSPDNFLSGPIQKYIISKGGRFHLRWGCRDIYFDKFADGETYVTGLALSKATSKQTVKADAYVAACDVPGIKKLLPSKWREWEAFDSIYKLVGVPVITVQLRYNGWVTELQNVEKSRQLRKAAGLDNLLYTPDADFSCFADLALTSPEDYYIEGQGSLIQAVLTPGDPYMPFPNEEIIDRVQKQVLSLFPSAQGLQVTWSSVVKIGQSLYREAPGMDPFRPDQRTPVKNFFLAGSYTKQDYIDSMEGATLSGRQAAAYICDSGEELAAIRKKLAGSEVRSIPKSADIVDELSMV

>Sphfalx10G050300|Sfallax_v1.1/1-593

MAGLLPAAKCCSGSLLGSNYNNSQASALESSRSSSLVTSSKRRRTRRDSRRLLLVSSSLQSMVSDMSKKAPKGLFPPDPEPYNGPKLKVAIIGAGLAGMSTAVELLEQGHEVDIYESRKFIGGKVGSFQDKAGNHIEMGLHVFFGCYNNLFRLLTKVGAADNLLVKDHIHTFINAGGDVGELDFRFPVGAPLHGMKAFLTTNQLNPLDKAANALALATSPVVRALVDPEGAMRDIRDLDKISFSDWFTSHGGTRMSIKRMWDPIAYALGFIDCDNISARCMLTIFAFFATKTEASLLRMLSGSPDMRLSGPISKYITDRGGRFHQRWGCREVLYDYTTEGETYVSGLVMTKGAQRQIVKADAYVAACDVPGIKKLLPQPWREWEFFDNIYKLVGVPVVTVQLRFNGWVTEMKDIELSRQISRAAGLDNLLYSADADFSCFADLALTSPEDYYKPGEGSLLQVVLTPGDPYMRLPNEKIIAKVLDQVMRLFPSAQGLQMTWSSVVKIEQSLYREAPGMDLYRPDQKTPVSNFFLSGSYTKQDYIDSMEGATLSGRQTSARICEMGQYLAELRQRLSDNDNFLNQSKLMDELTLV

>Adc06011|Adiantum_capillus-veneris/1-591

MSATSLLQSTPCQKHSSSLLSGTFWGESSQLKLSPPASAFNSNNGSKKQPLQWQSSLKTKVSDMSKKAPKGLFPPEPEKYAGPKLKVAIIGAGLAGMSTAVELLDQGHEVDIYDSRSFIGGKVGSFLDKEGNHIEMGLHVFFGCYNNLFRLMAKVGADNNLLVKEHIHTFVNKGGNIGELDFRFFTGAPLHGIKAFLTTNQLSTVDKAFNAVALATSPVVRALVDPDGAMGDVRDLDKVSFSKWFMSHGGTRMSIKRLWDPVAYALGFIDCDNISARCMLTIFTLFAIKTDASLLRMLNGSPDLRLNGPISKYITDRGGRFHLKTGCREILFKYTSDGEPYVSGLMMSKATEKEVVKADVYVAACDVPGIKRLLPEQWRQWAIFDNIFKLVGVPVITVQLRYNGWVTEMQDIEKSRQLSKATGMDNLLYTADADFSCFADLALTSPEDYYKPGEGSLLQVVLTPGDPYMPLPNEEIVSKVKKQVVALFPSAEGLEVTWSSVVKLGQSLYREAPGLDPFRPNQKTPISNFFLAGSYTKQDYIDSMEGATLSGRQAAAYICEAGKPLGELRSKLQALEARDQVESEQENLAVV

>Aspi01Gene68728|Alsophila_spinulosa/1-608

MEGLPYCLNSPSSSSLSKMASPSLPSSLCQGNGCLPSSFWGDSSPLKHATVASVQSFNGGTRRQLPQWQNALKTKVSDMSKKAPKGLFPPEPEKYTGPKLKVAIIGAGLAGMSTAVELLDQGHEVDIYDSRSFIGGKVGSFLDKDGNHIEMGLHVFFGCYNNLFRLMTKVGADDNLLVKEHIHTFVNKGGDIGELDFRFFTGAPLHGIKAFLTTNQLSTVDKAFNAVALATSPVVRALIDPDGAMRDIRNLDKVSFSEWFMSHGGTRMSIKRMWDPVAYALGFIDCDNISARCMLTIFTLFAIKTDASLLRMLNGSPDVRLNGPISKYITDRGGRFHLRWGCREILYKYTSDGEPYVAGLLMSKATEKEVVKADVYVAACDVPGIKRLLPEQWRQWEMFDKIYKLVGVPVITVQLRYNGWVTELQDIEKSRQLKQALGMDNLLYTADADFSCFADLALTSPEDYYRPGEGSLLQVVLTPGDPYMPLPNEEIVSKVKKQVVSLFPSAEGLEVTWSSVVKLGQSLYREAPGLDPYRPDQKTPISNFFLAGSYTKQDYIDSMEGATLSGRQAASYICEAGSRLGELRSRLQALDALDQVKAVKEVDSLSLV

>Ceric.37G066700.1.v2.1|Ceratopteris_richardii/1-604

MAGLAQCMLVSPSSCPPRANGSPCTGSEECDSLPTSFFGDRLILSSSSAASDKRSRGFVRVRHLPRWTGSLKTKVSDMSKKAPTGLFPPEPEKYTGPKLKVAIIGAGLAGMSTAVELLDQGHEVDIYDSRSFIGGKVGSFLDKHGNHIEMGLHVFFGCYNNLFRLMTKVGADNNLLIKEHIHTFVNKGGDIGELDFRFFTGAPLHGIKAFLTTNQLSVVDKAFNAVALAVSPVVRALIDPDGAMRDIRDLDKVSFSDWFMSHGGTRMSIKRMWDPVAYALGFIDCDNISARCMLTIFALFAIKTDASLLRMLNGSPDLRLNGPIRKYITDKGGRFHLKWGCREVLHSCTEDGEPYVTGLLMSKASEKKIINADVYVAACDVPGIKRLIPKEWRNFEIFDNIYKLVGVPVITVQLRYNGWVTELRDIEKSRQLQQAVGMDNLLYTADADFSCFADLALTSPEDYYKPGEGSLLQVVLTPGDPYMPLPNEEIVRKVKQQVIDLFPSAEGLEVTWSSVVKLGQSLYREAPGLDPFRPDQRTPISNFFLAGSYTKQDYIDSMEGATLSGRQAAAYICESGHLLGDLKSRLQHLNTQNEVPEVQTLSVA

>Azfi_s0010.g012159|Azolla_filiculoides/1-598

MAMAMAPPISSSSSSAAAASPFLYSTPCLPSSRTCSLWSHHTSHLLFSAHPHHQEEGKCQERRRNGVLPTWGSALKTRVSDMSKKAPKGLFPPEPEQYNGPKLKVAIIGAGLAGMSTAVELLDQGHEVDIYDSRSFIGGKVGSFLDKDGNHIEMGLHVFFGCYNNLFRLMSKVGADNNLLIKDHVHTFVNKGGDIGELDFRFFTGAPLHGIKAFLTTNQLSTVDKAFNAVALATSPVVRALVDPDGAMDVIRDLDKVSFSEWFISHGGTRMSIKRMWDPVAYALGFIDCDNISARCMLTIFTLFAIKTDASLLRMLNGSPDVRLSGPISKYITDRGGRFHLRWGCREILHDRLSDGVPYVSGLVMSKANKKEVIKADVYVAACDVPGIKKLLPEEWKEYDTFNNIYKLVGVPVITVQLRYNGWVTEMQDIERSRQLKQASGMDNLLYTGDADFSCFADLAITSPEDYYKPGEGSLLQVVLTPGEPYMPLQNDEIVRKVKQQVVSLFPSAEGLEVTWSSVVKLGQSLYREAPGLDPYRPDQKTPISNFFLAGSYTKQDYIDSMEGATLSGRQAAGFICEAGSRLGEVRSKILALDLSVV

>Mvestita_S4g08744-RA|Marsilea_vestita/1-627

MIATRSLSYLLLWSEHLLAESKLGTTCCPKNIWQKLNTPQTQTAMEGLASSISSYQSHRSCLNSAFLGQSIDLLRCHGIPSCSSSGGRKVQTTKWRASLQTKVSDMSKKAPKGLFPPEPENYTGPKLRVAIIGAGLAGMSTAVELLDQGHEVDIYDSRSFIGGKVGSFVDKDGNHIEMGLHVFFGCYNNLFRLMTKVGADNNLLVKDHVHTFVNKGGDIGELDFRFFTGAPLHGIKAFLTTNQLSTVDKAFNAVALATSPVVRALVDPDGAMRDIRDLDKISFSEWFMSHGGTRMSIKKMWDPVAYALGFIDCDNISARCMLTIFTLFAIKTDASLLRMLNGSPDLRLSGPIKKYITDRGGRFHLRWGCREVMYNYTPDGEPYVSGLLMSKATEKQIIKADAYVAACDVPGIKRLLPQEWRKYELFDNIYKLVGVPVITVQLRYNGWVTEMKDIERSRQLKQAVGMDNLLYTGDADFSCFADLALTSPEDYYKPGEGSLLQVVLTPGDPYMPLTNEQIVNKVKEQVLDLFPSARGLEVTWTSVVKIGQSLYREAPGLDPFRPDQKTPVPNFFLAGSYTKQDYIDSMEGATLSGRQAAAYVCEAGPNLSKLVADIEGLGSVAKVPAGV

>evm.model.Chr6.1590|Isoetes_sinensis/1-588

MSLPLHISPVSFLINERNLGTPNFAIGSPFASSLEGLDLRTKKWPRTMALGSLSAKVDDMGKQAPKGLFPPEPEKYTGPKLKIAIIGAGLAGMSTAIELLDQGHEVDIYDSRSFLGGKVGSFRDKDGNHIEMGLHVFFGCYNNVYRLMHKVGADDNFLVKEHVHTFVNKGGNIGELDFRFPIGAPLHGIKAFLSTNQLPLFDKAANAVALATSPVVQALIDPDGAMKTIRDLDKVSFSNWFMSHGGTRESIKRMWDPVAYALGFINCDNMSARCMLTIFALFATKTNASLLRMLNGSPELRLHGPISKYISDRGGRFHLNWGCREILYDYTPEGETYVTGLAMSKATQKQIVKADAYVAACDIPGIKKILPERWREWQFFDNIYKLVGVPVVTVQLRFNGWVTEMRDIELSRQLKQAVGLDNLLYSADADFSCFADLALASPQDYYKPGEGSLLQVVLTPGEPYMPLPNDIIAKKVLEQVHSLFPSSRGLEMTWSSVVKIAQSLYREAPGMDPFRPDQKTPISNFFLAGSYTKQDYIDSMEGATLSGRQAAARVCDAGPYLSELQKRVSKDAPSNFAPLESIDEFTIV

>evm.model.Chr5.1710|Isoetes_sinensis/1-588

MSLPLHISPVSFLINERNLGTPNFSIGSPFASSLEGLNLRTKKWPRTMALGSLSAKVDDMGKQAPQGLFPPEPEKYNGPKLKIAIIGAGLAGMSTAIELLDQGHEVDIYDSRSFLGGKVGSFRDKDGNHIEMGLHVFFGCYNNVYRLMHKVGADDNFLVKEHVHTFVNKGGNIGELDFRFPIGAPLHGIKAFLSTNQLPLFDKAANAVALATSPVVQALIDPDGAMKTIRDLDKVSFSNWFMSHGGTRESIKRMWDPVAYALGFINCDNMSARCMLTIFALFATKTNASLLRMLNGSPELRLHGPISKYISDRGGRFHLNWGCREILYDYTPEGETYVTGLAMSKATQKQIVKADAYVAACDIPGIKKILPERWREWQFFDNIYKLVGVPVVTVQLRFNGWVTEMRDIELSRQLKQAVGLDNLLYSADADFSCFADLALASPQDYYKPGEGSLLQVVLTPGEPYMPLPNDIIAKKVLEQVHSLFPSSRGLEMTWSSVVKIAQSLYREAPGMDPFRPDQKTPISNFFLAGSYTKQDYIDSMEGATLSGRQAAARVCDAGPYLSELQKRVSKDAPSNFAPLESIDEFTIV

>CYCAS_011903|Cycas_panzhihuaensis/1-589

MLSALVLERRQKRASLLQIRFSNQMLGNCFCFGHEVQSSKSRTNKFQVYCSLKSKVSDMGKQAPKGLFPPEPEEYKGPKLKVAIIGAGLAGMSTAVELLDQGHEVDIYESRPFIGGKVGSFTDKAGNHIEMGLHVFFGCYNNLFRLMKKVGADENLLVKEHTHTFINKGGQIGELDFRFLIGAPLHGIKAFLTTNQLLNFDKAANAVALATSPVVRALIDPDGALQDIRNLDNVSFSKWFMSHGGTRTSIQRMWDPVAYALGFIDCDNISARCMLTIFVLFAIKTEASLLRMLKGSPNLYLNGPIRNYITEKGGRFHLRWGCREILYDRTSECDTCVSGLLMSKNLSYGMILMSFFSMAACDVPGIKRLIPLEWRKWEFFDNIFQLLGVPVVTVQLRFNGWVTELRDLKRSRQLNQVAGLDNLLYTPDADFSCFADLAITSPEDYYRPDQGSLLQVVLTPGDPYMPLANEEIVDRVHKQVLSLFPSAHGLEVTWSSVVKIGQSLYREAPGMDPFRPNQRTPVQNFFLAGSYTKQDYIDSMEGATLSGRQAAAYICEVGTRLVELRSKLQVNDVLGHVKSAEGMNKMMFV

>Lc_composite_24479.1_1|Lunularia_cruciata/1-1065

NNPLYLQSFTNGDDALKFHYIVHCSLDVIDEKVSNPKKLGVSLNETFLGLLYPTEDYKVFGYLSNTKIKFILVTTDQDLRDADVRNDILVVLAWITWSRRIKKICFFGELKGQIIPVGVHMALGTSAGKDRRFHAAYVDAASNPFHVPGKRITSPAFAERGVVEDLKLKCSSVTVQAVECLSTECKLGATLRIQGLGFILLRDSDGYRDHTCRLENLPSLRCFKTRMTTRTPQMRKPPMRSNDAGVIDLHTIGWSTRHSWLQDTRTWQTREFSPSKRQKRAQEDRVLTAASLARSLTSLLLPLPRAGSGLLLVCRSISINLGGHSPSFLKREKMSAVLSATLATPVLAMASSPSALLHSKGTKLGQLASSQFLSALPVSLRNSCRHGVRSTRRVQLRVSSSLQSMVSDMGRAGTVSVFAWFQCEDSSAWEWEHLVEKGNELNFGVVVAVSLIRVDVSNTRRNQKFEHIQQIHRGSNQPMSVFHDQIYAVVPPWEEAESGTKAVHRISVRCWLNQEWKEICGWFLTLACELLLVPAPKGLFPDEPEKYTGPKLKVAIIGCGLAGMSTAVELLEQGHEVDIYEARPFIGGKVGSYVDKGGNHIEMGLHVFFGCYNNLFRLLTKVGGDDNLLLKEHTHTFINEGGNVGELDFRFLVGAPFHGLKAFATTNQLGVYDKIRNAMALGTSPVVRALVDPEGAMRDIRELDKISFSDWFIGHGGNRSSIQRMWDPIAYALGFIDCDNISARCMLTIFAFFATKTEASVLRMLNGSPDVRLSGPISQYIKDKGGRIHTRWGCREVLYDKTEDGTPFVSGFVMSKATEKKIVKADAYVAALDIPGIKRLLPPKWREYESFDNIYKLIGVPVVTVQLRYNGWVTEMQDVEKRRQLTRAEGMDNLLYSADADFSCFADLALTSPLDYYKPGEGSLMQLVITPGDPYMPLTNDKIVEKINEQVKNLFPSARELEMTWSSVVKIGQSLYRESPGMDPFRPDQKTPIPNFFLAGSYTKQDYIDSMEGATLSGRKCSAAICDVGSSLSELNKKLPSMDPSVAGAALNFEPAPVETEFSTV

>Klenit1_1_1441_kfl00012_0080_v1.1|Klebsormidium_nitens/1-599

MMATSAAQQALCSAPLSQMGGQVASRSRHLQTPVASGSSCSSFVGNALACQRRIKAPRRHLKAGARADLQTMVSDMSRAAPKGLFPPEPPKYTGPKLKVAIVGAGLAGMSTAVELLDQGHEVDLYESRPFIGGKVASFKDKDGNHIEMGLHVFFGCYNNLFRLMRKVGATDNLLLKEHTHQFINKGGNLGELDFRFITGAPFHGLKAFFTTNQLSTMDKVQNAIALATSPVVRALVDPEGAMRDIRNLDKVSFSDWFISHGGSRASITRMWDPIAYALGFIDCDNISARCMLTIFQFFATKTEASVLRMLAGSPQTGLHDPLIKYIEDKGGRFHVRWGCREVQYEEAADGSVRVTGLHMTKANQSEIVKADAYVAACDVPGIKRLLPTAWRRWPLFDNIYHLEGVPVITVQLRYNGWVTELQDQAKINQLARAEGLDNLLYSADADFSCFADLALTSPQDYYKPGEGSLLQCVLTPADPYMALPNEEIVAKVNEQVFNLFPSARNLSMTWHSVVKIGQSLYREAPGMDVFRPDQRTPIDNFFLAGSYTKQDYIDSMEGATLSGRQAAAKILESGADLAALREQLQQSLASSPAKDLATV

>Chabra1_342456_rna-CBR_g37129|Chara_Braunii/1-749

MAMAASMSVAGTAVPAAYVEAGTWGKSSTTLDTCSLLSCRRQSVPGACMDAAGRGSRERGLAAWMGSRRCPDCFYWSMSSSVVGRRGSRSRLGARLGSAAAGAAAEQFLSGQRLVLDPKKEEEEGGFVPTGSDVGCAPRQQRRRSRMMVRRVVRAGLREVQRQREEQQEEDSAAAGENGSWGGEIFQSTSSSSIDRASLATMVSNMRRKAPKGLFPPEPAKYNGPKLRVAIVGSGLAGMSTAVELLDQGHEVDIFEGRPFIGGKVASYKDKNGNHVEMGLHVFFGCYNNLFRLMNKVGGDRNLLLKDHTHTFINRGGGVGELDFRFFTGAPLHGIKAFLSTNQLSFGDKVQNSLALATSPIVRALVDPEGAMQDIRDLDKVSFTEWFLGQGGSRGSIDRMWNPVAYALGFIDCDNISARCMLTIFAFFATKTEASVLRMLKGSPDNGLSGPIRKYIEDRGGRFHVRWGCREILYEEQLDGSAVVTGLVVGKSTEKKVVKADCYVAACDIPGIQRLLPKRWREWEFFDNIYKLSGVPVVTVQLRYDGWVTEMQDIQRSRNSKSAVGIDNLLYSADADFSCFADLALTSPEDYYKEGEGSLLQCVITPGDPYMPLSNEEIVRRVNEQVFKLFPSSRGLNLTWSSVVKIAQSLYREAPGMDLFRPDQSTPVPNFFLSGSYTKQDYIDSMEGATLSGRQASTKICQAGQLLYDLSQSLTAKAAQQQEQQISGESDQQLQHQKEEDEEELLTFV

>EVM0003928.1|Lycopodium_clavatum/1-639

MSDLIAGDQGLGTVTQENLVAVETTVASLDDNSPQGLGIAAQEHHVVVEVSVEGSGVGELSHGRQVDVPIEGSENQDVCCCPQRETFLLSRIAVLFSSLQQLPVTSCSASFQRICSPMAAASLSASAFLSSDCQSLKTPFLASSTTTPTNAVFFSGGKCRILQCQTSRMHRLQMASSLQELVSDMSRNAPKGLFPPEPEKYVGPKLKVAIIGAGLAGMSTAIELLDQGHEVDIYDSRSFLGGKVGSFIDKDGNHIEMGLHVFFGCYNNLYRLMSKLDFRFPIGAPLHGIKAFLSTNQLPVLDKAANSLALARSPVVQALFDPDGAMKTIRELDKISFSDWFLSHGGTRASLKRMWDPVAYALGFLDCDNISARCMLTIFALFATKTNASLLRMLNGSPDLRLHGPISKYITDRGGRFHLRWGCREILFDNTLKGEEFVTGLAMTKATQKKIVKADAYVAACDVPGIKKILPEQWRGWEFFDNIYRLVGVPVVTVQLRFNGWVTEMQDIELSRQLKQAIGLDNLLYTADADFSCFADLALTSPADYYKPGLGSLLQVVLTPGDPYMPLPNETVVKKVHEQVLALFPSAEGLEVTWSSVVKIGQSLYREAPGMDRFRPDQKTPISNFFLAGSYTKQVISKS

>MA_516g0010|Picea_abies/1-225

MASASIHLQLPNQMLGGTSSHSHSLKEMLHTSKSSRNRRVNVPSVSCNLKAKVSDMSSKAPKGLFPPEPEKYEGPKLKVAIIGSGLAGLSTAVELLDQGHEVDIYESRSFIGGKVGSFVDKRGNHIEMGLHVFFGCYNNLFRLMKKGRGLSRPSSIFEVCKGRVVNSSTPLSTHDELVESGTPLSTHIGVASPPSSIDARPGPSAFGSRPGKQPMIVSCKRGRGH

>PSME_00008058-RA|Pseudotsuga_menziesii/1-146

MASASIHLQLCNQMLGGTSSLSHSLNELLHTSKSGSNRRANALSVSCNLKSKVSDMSRKAPKGLFPPEPDKYEGSKLKVAIIGSGLAGLSTAVELLDQGHEVDIYESRSFIGGKVGSFVDKKGNHIEMGLHVFFGCYNNLFRLMKK

>PITA_49723|Pinus_taeda/1-173

MQLDYIMASASIHLQLPNQMLGGTSSLSHSCKELLHSSKSSNNRRANAPAVSCNLKSKVSDMSRKAPKGLFPPEPEKYEGPKLKVAIIGSGLAGLSTAVELLDQGHEVDIYESRSFIGGKVGSFVDKRGNHIEMGLHVFFGCYNNLFRLMKKVKSVENHCQKNCCYLEMRMNC

>TnS000982373t01|Gnetum_montanum/1-667

MADRSKSNGDLWEKQATASSMEQLNSNNSDRTPDIEKKFVHNVYDAIAPHFSSTRFAKWPKVAEFLGSLSHGSVVLDVGCGNGKYLGFNQNCYFIGCDISAGLVNICANKGHEVLVADAVKIPYRTGFGDAAISIAVLHHLSTEERRKRAIEELVRVVRKGGKVLITVWAVEQEDRSLVGKWTPLMQKYMEEWVGPGSHIGSNSFSSLESIHEVGFDDKCNTETEEESIQENENSCSSTGNCSENSQSKERKDQEFFVPWHLPYHRAEVSKSCVSDIDSGFARRDDKKGSVVYNRYYHVFAQGELERLASGISSAVIVDNFYDKSNWCLFLRSLGAAALRALQRSASCTLSLLLRSASCALPLSLCRCAAEPSSALPPDAEQKLTVGCSFLPLGAVALQSPPAPPHEIGLVDVKLFCFDFGVGEIDFAFIVVPSQIVLVEKGAFFGTRWIQTSAALKNNEAEDCHELTTPPPPPPATKSRSDRKPDMISVIEFGHDTPKGLFPPEPERYSGPKLKVAIIGAGLAGMSTAVELLDQGHEVDIYESRSFIGGKVGSFVDKRGNHIEMGLHVFFGCYNNLFRLMQKVGADENLLVKEHIHTFINNGGQIGELNFKFFTGAPLHGIKAFLTTNQLVNFDKAANTVALATSPVVRALIDPNGAMQDIRNLDN

>evm.TU.supercontig_130.4|Cpapaya_ASGPBv0.4/1-508

MASTTKEDKPGSGKKVAVIGAGVSGLAAAYKLKSHGLNVTVFEADGRAGGKLRSVSHNGLIWDEGANTMTESEMEVRNLLDDLGLREKQQYPIAQNKRYVVRSGVPVLLPSNPIAFIQSSILSSRSKLQIILEPFLWKKGNSSKVSDSCAHESVGGFFERHFGKEVVDYLIDPFVAGTSAADPESLSMCHSFPELWDLERRFGSVIAGAIKSKLSSKRDKIGETKISSDKRKHQRGSFSFEGGMQTLTDTLCKNLLKDEIKLNSKVLSLSYNQDGKRASDNWSLCCASNDEKHSQGLSFDAVIMTAPMCSVKEMTIKKGRDLFSLDFLPEVNYMPLSVIITTFKKENVKRPLEGFGVLVPSKEQQNGLKTLGTLFSSVMFPDRAPSDLHLYTTFVGGSRNNQLARASTDELKEIVKSDLQQLLGAEGEPIFVNHFYWSKAFPLYGRNYGSVLEAIGKMEKNLPGFFYAGNHRGGLSVGKAVASGCKAADHVISYLESYSDGKILKDGS

>Prupe.3G303000|Ppersica_v2.1/1-505

MTSLTTKEGEQSSVKRVAVVGAGVSGLAAAYKLKSHGFDVTVFEAEGRAGGKLRSVSHDGLIWDEGANTMTESEKEVQTLLDDLGIREKQQFPISQNKRYIVRNGSPVLIPTNPIALIKSNFLSAQSKLQIILEPYLWKDKRVSDDHTEESVGGFFQRHFGEEVVDYLIDPFVAGTSAGDPESLSMRHSFPDIWNIEKRFGSVISGAIKSKLSASKGKSGETKGSVEKGKRQRGSFSFHGGMQTLTDILCNQLEKDELKLNSKVLSLSYRQGGNSASENWSVSRVADDDKHSQSLSVDALIMTAPLCNVKEMKITKRGTRFPLDFIPEVVYMPLSVIITTFKKENVKRPLEGFGVLVPSKEQKNGLKTLGTLFSSMMFPDRAPSDLYLYTTFVGGSRNKELAKASTDELKQIVTSDIRHLLGAEGEPTFVNHFYWSNAFPLYGRDYDSVIEAIENMEKNLPGFFYAGNHRGGLSVGKSIASGCKAAELVISYLESPSDEKTRHKG

>FvH4_6g53090|Fvesca_v4.0.a2/1-506

MASPSQPHNHRSVKKVAVVGAGVSGLAAAYKLKSHGFDVTVLEAEGRAGGKLRSVSYNGLIWDEGANTMTEAETEVQTLLDSLGLRDKQQFPISQNKRYVARNGMPVLLPTNPIELIKSNFLSTKSKFQILLEPYLWKKKKVSDDHTQESVAGFFQRHFGKEVVDYLIDPFVAGTSAGDPESLSMPHSFPELWNIEKRYGSVITGTIRSKVSSRKEKRGDTKGSVEKGKRQRGSFSFQGGMQTLTDTLCKQLGKHELKLNSKVLSLSYSHDGSSTSENWSLSCVANDDKHSQSSSVDAIIMTAPLCSIKEMKITRRGTIFPLDFLPEVNYMPLSVLITSFKKENIKRPLEGFGVLVPSKEQENGLKTLGTLFSSMMFPDRAPSDQYLYTTFVGGSRNKELAKASKDELKQIVTSDIRQLLGAEGEPTFVNHYYWSKAFPLYGHNYDSVIEAIEKMEKNLPGLFYAGNHRGGLSVGKAIASGCKAADLVISYLESSSDGKILQQGSS

>VIT_214s0171g00190|Vvinifera_v2.1/1-508

MAEKSDAQSHYNGSGKRVAVVGAGVSGLAAAYKLKLHGVNITLYEAEERAGGKLRSVSQHGLVWDEGANTMTESEIEVGSLLDNLRLREKQQFPISQNKRYIVRNGMPVLLPSNPIALIKSNILSAKSKFQIILEPFLWKKSDLSKVSDDHMKESVGGFFQRHFGKEVVDYLIDPFVAGTSGGDPESLSMHHTFPELWNLEKRFGSIIAGAVLSKLSAKREKRGETKGSSEKKKRQRGSFSFQGGMQTLTDTLCKELGKDKLRLKSKVLSLSYSHGEKSALENWSVAYASNPGKQSKDLSFDAVIMTAPLCNVREMKIMKKGNPFLLDFLPEVSYLPLSVIITTFKKENVKRPLEGFGVLVPSKEQQNGLKTLGTLFSSMMFPDRAPNDLYLYTTFIGGSRNRELAKASTDELKQIVTSDLRQLLGAEGEPTFVNHFYWSKAFPLFGHNYDSVLEAIDKMEKDLPGFFYAGNHKGGLSVGKAIASGCKAADLVISYLNSSSDGKMFKE

>Hyque.05G178900|Hquercifolia_v1.1/1-506

MASSSQQDKPSSGKRVAVVGAGVSGLAAAYKLKAHGLNVTIFEAEGRAGGKLRSVSQDGLIWDEGANTMTESDAEVRCLLDDLGLREKQQFPISQNKRYIARNGTPVLIPSNPIALIKSNFLSAQSKYQIIMEPFLWKKNDLSHVSNAEESVGGFFQRHFGKEVVEYLIDPFVSGTSAGDTESLSMCHSFPELWNLEKRFGSIIVGAIRSKLSARGEKSGDTKGSSAVKKRQRGSFSFLGGMQTLTDALCKHLGKDKLKLQSKVEELSYSCDMNSQLENWSVSFSPNHNHHSPKQAFDAIIMTAPLCNVKEMKITKKGSPFLLDFLPEVSYLPLSVIITTFKKENVKRPLEGFGVLIPSKEQQNGLKSIGTLFSSMMFPDRAPSDAYLYTTFVGGSRNKELAKASTDELKQIVTSDLRQLLGAEGEPTFVNHFYWSKAFPLYGRNYDSVIKAIEKMEENLPGFFYAGNHKGGLSVGKALASGCKAADLVISYLNSSSDDMMLKEKP

>Gohir.A05G340200|Ghirsutum_v3.1/1-523

MLNIAPFCVLASGISKPGTKMASTENKDDHSSAKRVAVIGAGVSGLAAAYKLKSQGLHVTVFESEGRAGGKLRSVSRDGLIWDEGANTMTESEIEVRSLFDDLGIQDKQQVPIAQNKRYIMRNGVPVLIPSNPLSLFTSSILSAKSKFQIILEPFLWRNSEASKVSDAYNQESVGGFFQRHFGQEVVDYLVDPFVAGTSAGDPESLSMCHSFPGLWDLEQRFGSIIVGAVKSKFSAKRTNREETKNSVKRKALRGSFSFQGGMQTLADMLCKDLSKDELKLKSKVLSLSYSHEGKSTSENWSLSYASDRDKRSQGSSFDAVIMTAPVCNVKEMKITKGGNVFPLNFIPEVSYMPLSVIITAFKKENVKKPLEGFGVLIPSKEQQNGLKTLGTLFSSVMFPDRAPNNLYLYTTFVGGNRNKELAKVSTDELKHIVTSDLQQLLGVEGEPTFFNHFYWSKAFPLYGRNYASVLEAIEKMERDLPGFFYAGNHKGGLSVGKSIASGCKAADNVITYLESSHDKLLK

>Gohir.D04G078700|Ghirsutum_v3.1/1-523

MLNIAPFCVLASGISKPVTKMASTENKDDHSSAKRVAVIGAGVSGLAAAYKLKSQGLHVTVFESEGRAGGKLRSVSREGLIWDEGANTMTESEIEVRSLFDDLGIQDKEQVPIAQNKRYIVRNGVPVLIPSNPLALFTSSILSAKSKFQIILEPFLWRKSEASKISDAYNQESVGGFFQRHFGQEVVDYLVDPFVAGTSAGDPESLSMCHSFPELWDLEQRFGSIIVGAVKSKFSAKRTNREETKNSVKRKALRGSFSFKGGMQTLADMLCKDLSKDELKLKSKVLSLSYSHEGKSTSENWSLSYASDRDKRSQGSSFDAVIMTAPVCNVKEMKITKGGNVFPLNFIPEVSYMPLSVIITAFKKENVKKPLEGFGVLIPSKEQQNGLKTLGTLFSSVMFPDRAPNNLYLYTTFVGGNRNKELAKASTDELKHIVTSDLQQLLGVEGEPTFFNHFYWSKAFPLYGRNYASVLEGIEKMERDLPGFFYAGNHKGGLSVGKSIASGCKAADNVITYLESSHDKLLK

>BUSI_017364-RA|Buxus_sinica/1-545

MYLENGRCTWSGSAQAPMPTEAASFVTAIGSSLSIVSPSPLEAPQFGSDISSGKSVAVVGAGVSGLAAAYRLKSHGCKVTVFESEERAGGKLRSVSRAGIIWDEGANTMTESEPEVRKLLDELGLREKQQFPISQNKRYIVRNGTPKLIPTNPIALIKSNFLSAQSKLQMILEPLSLGIFVKKKNAAKVSNESLEESVAEFFQRHFGKEVVDYLIDPFIAGTSAGDPESLSMRHAFPEIWNLEKRFGSVIVGAVRSKLSAKKDKNGERETSSGKLKRPRGSFSFEGGMQTLTDMLCKELNKDDLKLMSKVLSLSYSHGGNSLLGNWSVSFAANHNKTSQNESFDAVVMTAPLCNVKEMKISKRGSPFLLNFLPEVNYLPLSVIITTFKKENVKRPLEGFGVLIPSKEQHNGLRTLGTLFSSMMFPDRAPNDLYLYTTFVGGSRNRDLAKASTDELKQVVTSDLRKLLGAEGDPTFLNHFYWSKAFPLYGLDYGSVLEAIGKMEESLPGFFYAGNHRSGLSVGKAIASGCKAADLVISYLNSSSDN

>TESI_001491-RA|Tetracentron_sinense/1-501

MAATIKEDKKSGRSVKTVAIIGAGVSGLAAAHKLKSHGLNVTLFEAEERAGGKLRSVSQDGLIWDEGANTMTETEMEVGRLLDNLELREKQQFPIAQNKRYIVRNGTPELIPTDPIALIKSNFLSARSKFQIILEPLLWKAFTKKSNSPKVSNEDMQESVGEFFQRHFGKEVVDYLIDPFVAGTSAGDPESMSMCHSFPDLWNLEQRFGSIIVGAIRSKLSAKKEENGERGTSSGKKKRQRGSFSFQGGMQTLTDMLCKELGKDDLKLKSKVLSLSYNPDGNFPLENWSVSYAARHNEPSQDQSFDAIIMTITNRGNPFLLDFLPEVSYLPLSVLITSFKKDNVKRPLEGFGVLIPSKEQHNGFKTLGTLFSSMMFPDRAPSDLYLYTTFVGGSRNRDLAQASTEELKQVVTSDLRKLLGAEGDPTFVNHFYWSKAFPLYGRDYNSVLEAIGKMEKSLPGFFYAGNHRGGLSVGKTIASGIKAADLVVSYFNSSDDDKDAY

>Glyma.19G087600|Gmax_Wm82.a6.v1/1-502

MASSATDDNPRSVKRVAVVGAGVSGLAAAYKLKSHGLDVTVFEAEGRAGGRLRSVSQDGLIWDEGANTMTESEIEVKGLIDALGLQEKQQFPISQHKRYIVKNGAPLLVPTNPAALLKSKLLSAQSKIHLIFEPFMWKRSDPSNVCDENSVESVGRFFERHFGKEVVDYLIDPFVGGTSAADPESLSMRHSFPELWNLEKRFGSIIAGALQSKLFAKREKTGENRTALRKNKHKRGSFSFQGGMQTLTDTLCKELGKDDLKLNEKVLTLAYGHDGSSSSQNWSITSASNQSTQDVDAVIMTAPLYNVKDIKITKRGTPFPLNFLPEVSYVPISVMITTFKKENVKRPLEGFGVLVPSKEQKNGLKTLGTLFSSMMFPDRAPSDLYLYTTFIGGTQNRELAQASTDELRKIVTSDLRKLLGAEGEPTFVNHFYWSKGFPLYGRNYGSVLQAIDKIEKDLPGFFFAGNYKGGLSVGKAIASGCKAADLVISYLNSASDNTVPDK

>KAI5431115.1|Pisum_sativum/1-502

MKMTSSVKDDKHRSVKRVAVIGAGVSGLAAAYKLKSHGLDVTVFEAEGRAGGRLRTVSRDGLVWDEGANTMTENETEVKGLISALGLQEKQQYPLSQHKRYIVKNGTPLLVPANPAALLKSNLLSAQSKIHVIFEPFLWKRNGSSTVCDDDSEESVSRFFERHFGKEVVDYLIDPFVGGTSAADPESLSMRHSFPELWNLEKRFGSIISGALQSSLFGKRNKTGETKDASRKNKHQRGSFSFHGGMQTLTDTLCKEIGKDDLTLNAKVLTLAYSHDGSSSSENWSITCTSNKKTQDVDAVIMTAPLGNVKDIQITKKGTPFSLNFLPQVTYLPLSVLITTFKKENVKRPLEGFGVLVPSKEQQNGFRTLGTLFSSMMFPDRAPSDVHLYTTFIGGTRNRELAQASTDELKKIVTSDLRKLLGVEGEPTFVNHFYWRKGFPLYGHNYGSVLEAIDRMEKDLPGFFYAGNHRGGLSVGRAIASGCKAADLVISYLNNASDNSVS

>Rhdel10G0008800.1|Rhododendron_delavayi/1-507

MASANNEDKQSSAKRVAVVGAGVSGLAAAYKLKLHGLNVTVFEAEGRAGGKLRSISQDGLIWDEGANTMTESETEVRFLLDNLGLHDKQQFPIAQNKRYIVRNGAPALIPSNPIALIRSNFLSAQSKLLIILEPFLWKKSNMEKVSDSDESVGDFFQRHFGKEVVDYMIDPFVAGTSAGDPGSLSMRHTFPELWNLEKKFGSIISGAIQSKLFTKKGKNSKLNSSSISKKHQRGSFSFLGGMQVKYSWLLSRIWYGMLSMGSIVILMLLSLDSKPVFKNWSVSSAPMHNKQSEKQSFDAVIMTAPLCNVKEMKFTMKGSPFLLNFLPEVSYLPLSVIITTFKKDSVRRPLQGFGVLVPSKEQQNGFKTLGTLFSSMMFPDRAPSDLHLYTTFVGGSRNKELAKASTDELKQIVTSDLRQLLGAEGEPTFVNHFYWSKAFPLYGHNYDAVLGAIEKMEANLPGFFYAGNHRGGLSVGKAISSGCKAADLVISYLEYSSHDKMPNEKPC

>DCAR_624437|Dcarota_subsp_sativus_v3.0/1-507

MASAPAQDNNSAAKRVAVVGAGVSGLAAAYKLSQRGLNVTIFEAEERAGGKLKSVSHDGLIWDEGANTMTESEGNVSYLLDNLGLRDKQQFPISQHKRYIAKNGTPVLLPSNPIALIKSNILSTQSKIQLLLEPFLWKKNSNSKMSDKHESVGGFFRRHFGQEVVDHLIDPFVAGLNAGDPESLSMYHAFPDLWNLEKKFGSIIVGAVRSKISFKKGEGQGMNASSKSKRRKRGSFSFLGGMQTLTDALCEEVGKDKVNLQTRVLELSYPCSQESQLSNWSISYALDFDKHLKEQCFDAVIMTAPLCDVKQMKITKRGKPFVLDFIPEVSYMPLSVVITSFKKENVRRPLEGFGVLVPSKEQQNGLKSLGTLFSSMMFPDRTPSDLYLYTTFIGGSRNQELARASRDELKKIVAADLRQLLGAEGEPTFVNHLYWSKAFPLYGHDYNSVIGAIDKMEKDLPGFFYAGNHRGGLSVGKAISSGCKAADLVISYLDSSLENNLSRENCT

>GWHPBAUK009902.1|Macadamia_integrifolia/1-517

MAPRAPTIKEDKTIERPVKSVAVVGAGVSGLAAAFRLKSHGLDVKVFEAEERAGGKLRSVFKNGLIWDEGANTMTESEIDVKRLLDDLGLGVKQQFPISQNKRYIVRNGRPELIPSDPISLIKTNFLSTQSKLQIILEPFLRGDSLKKSNISKVSDENLHESVGEFFQRHFGKEVVDYLVDPFVAGSSGGDPESLSIRHAFPELWNLERKYGSIIVGAIRSKFSGKREENEARKNSSGKKKGQRGSFSFHGGMQTLTDTLCNVLGEDNLKLKSKVLSLSYNHDVNSASNNWSISYATKHSRTSQHQSFDAIIVTAPLSDVKEMKIARMGNPFLLDFLPMVNYLPISVVITTFKKEKVQRPLEGFGVLVPSKEQQNGLKTLGTLFSSMMFPDRAPGDLYLYTTFVGGSRNRDLAKASLDELKHVVTSDLRKLLGAEGEPTFVNCFHWSKAFPLYGHGYDSVLKAIDKMEESLPGFFYAGNHRGGLSVGKSIASGCKAADLVISYLNSLDGSRPTEESS

>Soltu.DM.03G000120|Stuberosum_v6.1/1-507

MVPMAPSAGEDKQNCPKRVAVIGAGVSGLAAAYKLKIHGLDVTVFEAEGRAGGKLRSLSQDGLIWDEGANTMTESEGDVTFLLDSLGLREKQQFPLSQNKRYIARNGTPTLIPSNPIDLIKSNFLSTGSKLQMLFEPLLWKNKKLTKVADEHESVSGFFQRHFGKEVVDYLIDPFVAGTCGGDPDSLSMHLSFPELWNLEKRFGSVIVGAIRSKLSPIKEKKQGPPKTSVNKKRQRGSFSFLGGMQTLTDAICNDLKEDELRLNSRVLELSCSCSGDSATDSWSIFSASPHKRQAEEESFDAVIMTAPLCDVKGMKIAKRGNPFLLNFIPEVDYVPLSVVITTFKKESVKHPLEGFGVLVPSEEQKHGLKTLGTLFSSMMFPDRAPNNVYLYTTFVGGSRNRELAKASRTELKEIVTSDLKQLLGAEGEPTYVNHVCWSKAFPLYGHNYDSVLDAIDKMEKNLPGLFYAGNHKGGLSVGKALSSGCNAADLVISYLEAVSTDTKNHS

>Brara.J02031|BrapaFPsc_v1.3/1-508

MASNAVADHDKLSGKRVAVVGAGVSGLAAAYKLKSKGVNVTVFEADGRVGGKLRSVMHNGLIWDEGANTMTEAEPEVGSLLDDLGLREKQQFPLSQKKRYIVRNGLPVMIPTNPIALVTSSVLSTQSKFQILLEPFLWKKNDSSSKVSDASVVESVSGFFQRHFGQEVVDYLIDPFMGGTSAADPESLSMKHSFPDLWNIEKSFGSIIVGAIRSKFAAKGSKNGETKSSTGTKKGSRGSFSFKGGMQILPDMLCKDLSREDLNLDSKVLSLSYNTGPREENWSLSCVSHNETQRQNLHYDAVVMTAPLCNVKEMKVMKGGEPFKLNFLPEIKYMPLSVIITTFTKEKVKRPLEGFGVLIPSIEQKHGFKTLGTLFSSMMFPDRCPSDLHLYTTFIGGSRNQELAKASTDELKQVVTSDLQRLLGIEGEPVFVNHVYWNKAFPLYDRSYDSVMEAIDKMEKDLPGFFYAGNHRGGLSVGKSIASGCKAADLVISYLESCSNDNKPEDSL

>AT5G14220|Athaliana_Araport11/1-508

MASGAVADHQIEAVSGKRVAVVGAGVSGLAAAYKLKSRGLNVTVFEADGRVGGKLRSVMQNGLIWDEGANTMTEAEPEVGSLLDDLGLREKQQFPISQKKRYIVRNGVPVMLPTNPIELVTSSVLSTQSKFQILLEPFLWKKKSSKVSDASAEESVSEFFQRHFGQEVVDYLIDPFVGGTSAADPDSLSMKHSFPDLWNVEKSFGSIIVGAIRTKFAAKGGKSRDTKSSPGTKKGSRGSFSFKGGMQILPDTLCKSLSHDEINLDSKVLSLSYNSGSRQENWSLSCVSHNETQRQNPHYDAVIMTAPLCNVKEMKVMKGGQPFQLNFLPEINYMPLSVLITTFTKEKVKRPLEGFGVLIPSKEQKHGFKTLGTLFSSMMFPDRSPSDVHLYTTFIGGSRNQELAKASTDELKQVVTSDLQRLLGVEGEPVSVNHYYWRKAFPLYDSSYDSVMEAIDKMENDLPGFFYAGNHRGGLSVGKSIASGCKAADLVISYLESCSNDKKPNDSL

>evm.TU.Scaffold_1810.104|Carabica_v0.5/1-584

MNNNNNVPVVGRWQQESWQSIHKSLWALGMQSKRYGVHTSGIIICIMYASPFMKGGAVVRREDWLNESWEMQNDTLLAFVFCPTMKAKGTCTKKSVAVVGAGVSGLAAAYKLKLRGLNVTVFEADERAGGKLKSVSRDGLIWDEGANTMTESDAEVGFLLDNLGLRDKQQYPISQTKRYIARNATPILIPSNPLALIGSSFLSAQSKLQILFEPFLWKKNPSKFSEEQESVGGFFERHFGKEVVEYLIDPFVAGTSGGDPDSLSIRYSFPDLWSLEKRFGSVIYGAIQSKLSAKRENKGGTRSSPANKRRQRGSFSFLGGMQTLTNALCKELGEKDLKLRHEVLELSCSSRSKSSLLDSWSVSYASQHKKMSEEKSFDAVIVTAPLNNLKDMKITKGGKPFRLDFIPEVTYLPVSVVITTFNKKNVRRPLEGFGVLVPSKEQQNGLKTLGTLFSSMMFPDRAPNDTYLYTTFVGGSRNKELAKATRDELKDIVTSDLRQLLGAEGQPTFMNHFYWSAAFPLYGRNYKTVIEAIDKLEKDLPGFFYAGNHKEGLAVGRAISSGCKAADLVISYLNSSLHVKESSD

>Aco008083|Acomosus_v3/1-550

MSSYGVTSQIPNSHRCGYPARDFVCGHRPHHLRAPPLKPLSPNPATIAASSSSMEGGKSQSPIKSVAIVGAGVSGLAAAYKLKSNGVRVTVFEAEERAGGKIRTNSENGFIWDEGANTMTESEMEVGRLIDDLGLRDKQQLPIAQNKRYIVRNGMPQLIPSNPIALITSSILSPKSKLKLLMEPFLWKQSGTMSSAEVFDESSHESVRKFFQRHFGKEVVDYLVDPFVAGTSAGDPESLSMRHAFPELWDLEKRYGSVIVGAIRAKKSAKSNDKTAGSSSPGKKRNQRASFSFHGGMQTLTDTLYKEIGGDSLKLSSKVLSLSCKYDESSPSSSWAISLGAKDANSKDLASQSFDAVIMTAPLSNVQEMKFTNRGNPFVLDFLPKVNYLPLSVLITAFKKENVRRPLEGFGVLVPSKEQENGLKTLGTLFSSMMFPDRAPSDQYLYTTFLGGSRNRELARAPLDVLKQIVTDDLKKLLGVEGQPTFVKHVYWKSAFPLYGQNYDLVLEAIEKMEKNLPGFFYAGKGGLSVGKAMTSGLKAADLVIAYLGS

>evm.TU.AsparagusV1_Unassigned.847|Aofficinalis_V1.1/1-508

MDLSDAASVSGSAKSVAVVGSGVSGLAAAYRLKSNGVRVTVFEAEGRAGGKIRSNSLDGLIWDEGANTMTESETEVGRLIDDLGLREKQQFPISQTKRYVVKNGEPQLIPSNPVALIKSNILSAQSKLKIILEPFTCRQYRRKDSKVSGEDVKESVGEFFGRHFGREVVDYLIDPFVAGSSAGDPESLSMRYAFPDLWNLEKKFGSIIIGAIRSRTSAKSKDTAPGKTPASLQKKKQRGSFSFQGGMQALTNMLCAKLDKDSMKLDSKVLSLSYDLDGRYPSKSWSISYSGKGGSELDLDQSFDAIIMTAPLRNVQEMKFTNRGNPFVLDFFPKINYLPLSVLITSYKKEDVKRPLEGFGVLVPTKEQENGLKTLGTLFSSMMFPDRAPKDQYLYTTFVGGSRNRELASASLDKLEEVVTSDLRKLLGAEGPPTFVKHIYWRNAFPLYGRDYGLVMEAIDKMEKNLPGFYYAGNHRDGLSVGKAILSGLRAADQVMYYLNSEEKDDAS

>GWHPCBIR018679|Santalum_yasi/1-516

MASDAQRDKHRSAKRVAVIGAGVSGLAAAHKLKLQGLNVMVFEAEGRAGGKLRSVSRDGLTWDEGANTMMDSEVEVGNLLDILRLREKQQFPISQEKRYIVKNGVPELIPTNPIALIKSNFLSGRSKLEIILEPFFWKKGSKVAEVSCDQEESVGGFFQRHFGKEVVDYLLDPLLAGTCAGDPDSISMRHVFPELWNLEKSRPTSLFGSIIVGAIKSKLSASKEKSGEASPSSRQKKHGRGSFSFHGGMQTLTNTICKELGEDNLKLESKVLSLSYSLDGKSAPDNWYVSYGHGKHSQDMPFDAVIMTAPLNSVKEMKISKEGNLFPLDFVPEVNYLPLSVIITAFRKENVKRPLEGFGVLVPSNEQQGGLKSLGTLFSSMMFPDRTPNDTYLYTTFIGGSRNKELARASKDELKQVVSSDLKQLLGAEGEPEFVNHFYWSKAFPLYDHNYGSVLEAIKKMEENLPGFFYAALTVEMRLKRASWNSFQERRRTVPHSLIMEEKEAAPDVDVNILID

>gene_prefix19181.t1_1|Ceratophyllum_demersum/1-486

MASPVNDKTERSIKSVAVVGAGVSGLAAAYKLKSHGLEVTLFEAEGRTGGKIRSMSQNGFVWDEGANTMTETEFEVGRLINNLGLREKQQSIPPNPIALIKGNFLSLQSKFRIIFEPFLWRSSGIRSGPSKISPEYSQESVGEFFCRHFGKEVVDYLIDPFVAGTSGGDPESLSMRHAFPEIWQLEKNFGSIILGSIRSKFSQNKVSETCTSLEKRDKKILHGSFSFVGGMQTLTDALSKEIGLDNIQLNSKVRSLSYNHDEDPLSRDWFVSYDTNTATNNGSTLERSFDAVVMTAPLSNVKEMKITRNGNPFILDFLPVVNYLPLSILVTAFRKEDVKRSLEGFGVLVPSKEQENGLKTLGTLFSSMMFPDRAAGDCYLYTTFIGGSRNRDLASASLDQLKAIVTSDLRKLLGVEGQPTFLKHIYWSDAFPLYSRDYDLVLKAIERLENGFPGLFYAGNHRDGLSVGKAISSGCKAADLVVSYLK

>Aqcoe5G367800|Acoerulea_v3.1/1-221

MAENQMESSSSSSSVSTITEEEKKKKNPRLVSKSVAIVGGGISGLAAAYKLKSYGLNVTLFETEERAGGKLRSVSQDGLIWDEGANTMMESEMEVTTLLDNLRLREKQQFPISQNKRYIVRNGKPEMIPTNPIALIKSSFLSTKSRGFQLMLEPFLWRSSAKNNISDENSKQSVGEFFERHFGKEVVDYLIDPFVAGTSAADVDSLSMPHAFPELWNLEKM

>Nycol.C00145|Ncolorata_v1.2b/1-519

MAPSSQPSDGTQSSAKSVAVVGAGISGLVAAYQLKLHGLRVTLFESNQHPGGKIRTNSENGIVWEEGANTMTESDDAVGRLLDGLCLREKQQFPISQNKRYIVKNGKPELIPSNPLALIQSKFLSSKSKLQILMEPFLMGRSDRKTNPSEMPGQDLSESVGMFFKRHFGQEVVDYLIDPFVAGTSGGDPESLSMHLAFPEIFQLEKMYGSIILGFIKSKISKRGVEKGEQSASRGKSKHSKGSFSFQGGLQVLTDKLCTELTNCSLKLHSEVLSLSYNIEEGSPTNSWLVSYTSQPANDKNPLQDQTFDAVVMTAPLCSVQQMQIEKMGRPFLLDFLPKVDYVPLSVIVTTFKKENVKFPLEGFGVLVPTKEQENGLKTLGTIFSSMMFPDRAPSDQYLYTTFIGGTRNRDLAGASLDELQHVVSSDLQKLLGVEGQPASIRHVYWKDAFPLYGHDYDRVIEAIKKMEEGLPGFFYAGNHRGGLSVGNAISSGFKAADLVIAYLNSQHGKTPDHFESEK

>Zm00001d003214|Zmays_RefGen_V4/1-549

MLALTASASSASSHPYRHASAHTRRPRLRAVLAMAGSDDPRAAPARSVAVVGAGVSGLAAAYRLRQSGVNVTVFEAADRAGGKIRTNSEGGFVWDEGANTMTEGEWEASRLIDDLGLQDKQQYPNSQHKRYIVKDGAPALIPSDPISLMKSSVLSTKSKIALFFEPFLYKKANTRNSGKVSEEHLSESVGSFCERHFGREVVDYFVDPFVAGTSAGDPESLSIRHAFPALWNLERKYGSVIVGAILSKLAAKGDPVKTRHDSSGKRRNRRVSFSFHGGMQVFSSISLINALHNEVGDDNVKLGTEVLSLACTFDGVPALGRWSISVDSKDSGDKDLASNQTFDAVIMTAPLSNVRRMKFTKGGAPVVLDFLPKMDYLPLSLMVTAFKKDDVKKPLEGFGVLIPYKEQQKHGLKTLGTLFSSMMFPDRAPDDQYLYTTFVGGSHNRDLAGAPTSILKQLVTSDLKKLLGVEGQPTFVKHVYWGNAFPLYGHDYSSVLEAIEKMEKNLPGFFYAGNNKDGLAVGSVIASGSKAADLAISYLESHTKHNNSH

>Sobic.006G129700|Sbicolor_v5.1/1-544

MLARTATVSSTSSHSHPYRPTSARSLRLRPVLAMAGSDDSRAAPARSVAVVGAGVSGLVAAYRLRKSGVNVTVFEAADRAGGKIRTNSEGGFLWDEGANTMTEGELEASRLIDDLGLQDKQQYPNSQHKRYIVKDGAPALIPSDPISLMKSSVLSTKSKIALFFEPFLYKKANTRNPGKVSDEHLSESVGSFFERHFGREVVDYLIDPFVAGTSAGDPESLSICHAFPALWNLERKYGSVVVGAILSKLTAKGDPVKTRRDSSAKRRNRRVSFSFHGGMQSLINALHNEVGDDNVKLGTEVLSLACTLDGAPAPGGWSISDDSKDASGKDLAKNQTFDAVIMTAPLSNVQRMKFTKGGAPFVLDFLPKVDYLPLSLMVTAFKKEDVKKPLEGFGVLIPYKEQQKHGLKTLGTLFSSMMFPDRAPDDQYLYTTFVGGSHNRDLAGAPTSILKQLVTSDLKKLLGVQGQPTFVKHIYWGNAFPLYGHDYNSVLEAIEKMEKNLPGFFYAGNNKDGLAVGSVIASGSKAADLAISYLESHTKHNNLH

>ELECO.r07.4AG0312740|Ecoracana_v1.1/1-534

MLSSTSTASSASAHPYRPASTRAMAGSDDTRAAPARSVAVVGAGVSGLAAAYRLRKSGVNVTVFEAGDRAGGKIRSNSEGGFLWDEGANTMTESELEVSRLIDDLGLQDRQQYPNSQHKRYIVKDGAPVLIPSDPISLIKSSVLSTKSKFALFLEPFIYKKTSTRNSGIVSDEHLSESVGSFFERHFGQEVVDYLIDPFVAGTSGGDPESLSIRHAFPALWNLEKKYGSVIAGAILSKLTAKRDPVKKTSDSSGKRRNRRVSFSFLGGMQSLIDALHNEVGDGNVKLSTEVLSLACSVDGVPASGGWSISIDSKNTGSKEFGKKQTFDAVIMTAPLSNVQKMKFMKGGAPFVLDFLPKVDYLPLSLMVTAYKKEDVKRPLEGFGVLIPYKEQQKHGLKTLGTLFSSMMFPDRAPSDQFLYTTFIGGSHNRDLAGAPTTILKQLVTSDLRKLLGVEGQPTFVKHVYWRNAFPLYGRDYNSVLEAIEKMEKNLPGFFYAGNNKDGLAVGSVIASGSKAADLAISYLESRTKHDSSH

>Bradi5g14120|Bdistachyon_v3.2/1-544

MLTSATASPSASTRFSSTCRPCRSDSVPARRPRPVLAMAASDDPRAAPARSVAVVGAGVSGLVAAHRLRKSGVRVTVFEADDRAGGKIRTNSDSGFLWDEGANTMTESALEASRLIDDLGLQDKQQYPNSQHKRYTVKDGAPTLIPSDPIALMKSTVLSTKSKFKLFLEPFLYEKSHTRNSQKVSDNHLSESVGSFFERHFGKEVVDYLIDPFVAGTSAGDPESLSIRHAFPGLWDLEKKYGSIIVGAILSKLTAKGDSTKKADTSSGKGRNKQASFSFHGGMQTLVEGLHKDVGDGNVKLGTQVLSLACSCDRLSASDGWSISVNSKDASSKLAAKNQLFDAVIMTAPLSNVQRMKFTKGGVPFVLDFLPKVDYLPLSLMVTAFRKEDVKRPLEGFGVLIPYKEQQKYGLKTLGTLFSSMMFPDRAPNDQHLFTTFVGGSHNRDLAAAPTAILKQLVTSDLRKLLGVEGQPTFVKHVHWKNAFPLYGHDYDLALEAIGKMENELPGFFYAGNNKDGLAVGNVIASGSKTADLVISYLESHQAR

>HORVU.MOREX.r3.2HG0177420|Hvulgare_Morex_V3/1-554

MLTSATAPPSSSSCSSHAPARFASPSRPRRSASASARGRGRRVRPVLAMAASDDPRARSVAVVGAGVSGLVAAYMLRKSGVRVTVFEAEDRAGGKIRTNSDGGFLWDEGANTMTESALEASRLIDDLGLQDRLQYPNSQHKRYTVKDGAPALIPSDPIALMKSTVLSTKSKFKLFLEPFLYEKSSTRNSKKVSDEHLRESVGSFFERHFGKEVVDYLIDPFVAGTSAGDPESLSIRHAFPGLWNLEKKYGSLIVGAILSKLTAKGGSAKKGGASSGKGRNKRASFSFHGGMQTLVDALHKEVGDTNVKLGTQVLSLACNCDGLSASDGWSIFVDSKDASSKELARNQSFDAVIMTAPLSNVQRMKFTKGGAPFVLDFLPKVDYLPLSLMVTAFKKEDVKRPLEGFGVLIPFKEQQKHGLKTLGTLFSSMMFPDRAPNDQHLFTTFIGGSHNRDLAGAPTAILKQFVTSDLTKLLGVEGQPTFVKHIHWRNAFPLYGHDYDLALEAIGKMEGDLPGFFYAGNNKDGLAVGNVIASGSNTADLVISYLESGIKHVN

>Asa6G04321.1|Allium_sativum/1-439

MKCVSSKPPYLARCLYCAAIDVCKYHVKSVAVVGGGVSGLAAAYKLKSNGVKVTVFEAEGRAGGKIRSNSVDGFIWDEGANTMTESETEVGNLIDELGVRKKQQYPISQSKRYIAKNGVPQLIPSNPFSLITSNFFSAQSKVSIVINFNSVPFSDYSILYYLFQRRSYYFIVVDYLIDPFVAGSSGSDPESLSMRHAFPELWSLEKNYGSVIVGAIRAKASQNPKAGKTATSSGKKKHPRGSFSFQGGMQVLTDMLCTKIGDDSMKLRSKVLSLAYDLDKTTMKKCWSVSYATNGENHSKLQKPFDAIIMTAPLGNVEEMKITKKGKPIIIDPFPKVDYLPLSVLITSFKKGNVKKPLEGFGVLVPSKEQKHGLQTLGTLFSSMMFPDRAPDDQYMYTTFVGGSRNRELASAPVDKLKEIVTSDLRKLLGAEGQPSFVK

>AANG009548|Anthoceros_angustus/1-209

MASPSSESSRLLTPTQGPVLGSVAVIGAGFSGLAAAYRLRAKGVAVKVFEASEVVGGKVNSVSQDGIIWEKGPNTMAETDPLIGRLIDELQLRLKQQFPVSQHKRYIVRDGKPHMLPSSPLSLVGSKLLSTKAKLNIVLEPFFWKRRKDVQGEPQEPDDESVGHFMARHFGSEVVEYLVNPFVAGTSAADPDRISMRHTFPDIVMLEEK

>Lc_composite_05663.1_1|Lunularia_cruciata/1-932

MGSQRLEVSTYSSMKVKQKGVEVVGSRERSKSVWKEEGDVNQEERETKRNGEEDSSVSSAPFLQVELVGFQAAAVLPLCLPLERDNFVALSFSGSRVYLQAEGGKERRRRRSKRSLRRERRGGHGLPSGARIVKEQRDLDIGVVAERLTVIGTEVGSGLRGEEVRVELRERGTVAGIEMAQAASDSWLKEFEEAMRLADDILARINERNMMLASGNDPTRLVSATRRKITMLGTKLDRLESLLQNPPIKPSISEKEMYRRQDMLLGIRFRTKQMAAALSTSQPSNRATLMGNDSRSAPPEETSRTTGLDNYGIINLQKQVMKDQDDDLAGLEATVTSTKHIALAVNEELDLQNHLLNDMDKDADDTNNRLKAAQRKIGVLSKNSNQGCSFMCMCLMLLAIVLLDGIYSVKDKVGSSQFPVFKGKMQLNTNVHLGVIVGEVLLELTSQLGSLSRQVSGATFCMSTAELVLCEGIRSWTVLRQGIMLFIDGVWVRRRTSYGEAHNTCPRSRAKDHSANTAPGMFLPEGDGYSQDSHLRIKKCNKLEVQEIDVRTVMMHLKVQVKEKKESNSHSKLELTRISLRYTETINESRYYQDSRGDFEGIGIRDTGKNLLTHLIGNCGTGVGKTLTRVKMVFHATREGGPLRGDLTLQYCCDQSSVPCGIVNIQESHGFHRTSAGKTGVGLEDSIQSVADNFVLILVLLSIGSGSGAVVLETMAPTQTDVLIEEVDYTHSLLQRTSNPFSFANTESNRSGSVAVIGAGVSGLAAAYRLRKEGVNVTVFEAESTVGGKLRSLSQDGLIYDFGPNSMVENDSDVSYLIDDLQLRGKQQYPVMQNKKYILRDGNPVLLPSNPIGLIGSKILSTKAKLDLFLEPLRWKNKSAVPEDGTKDESVGDFMGRHFGTEMVDYLVDPFIAGTTGSDPASLSVSSIYASD

>Lc_composite_05663.2_1|Lunularia_cruciata/1-966

MGSQRLEVSTYSSMKVKQKGVEVVGSRERSKSVWKEEGDVNQEERETKRNGEEDSSVSSAPFLQVELVGFQAAAVLPLCLPLERDNFVALSFSGSRVYLQAEGGKERRRRRSKRSLRRERRGGHGLPSGARIVKEQRDLDIGVVAERLTVIGTEVGSGLRGEEVRVELRERGTVAGIEMAQAASDSWLKEFEEAMRLADDILARINERNMMLASGNDPTRLVSATRRKITMLGTKLDRLESLLQNPPIKPSISEKEMYRRQDMLLGIRFRTKQMAAALSTSQPSNRATLMGNDSRSAPPEETSRTTGLDNYGIINLQKQVMKDQDDDLAGLEATVTSTKHIALAVNEELDLQNHLLNDMDKDADDTNNRLKAAQRKIGVLSKNSNQGCSFMCMCLMLLAIVLLDGIYSVKDKVGSSQFPVFKGKMQLNTNVHLGVIVGEVLLELTSQLGSLSRQVSGATFCMSTAELVLCEGIRSWTVLRQGIMLFIDGVWVRRRTSYGEAHNTCPRSRAKDHSANTAPGMFLPEGDGYSQDSHLRIKKCNKLEVQEIDVRTVMMHLKVQVKEKKESNSHSKLELTRISLRYTETINESRYYQDSRGDFEGIGIRDTGKNLLTHLIGNCGTGVGKTLTRVKMVFHATREGGPLRGDLTLQYCCDQSSVPCGIVNIQESHGFHRTSAGKTGVGLEDSIQSVADNFVLILVLLSIGSGSGAVVLETMAPTQTDVLIEEVDYTHSLLQRTSNPFSFANTESNRSGSVAVIGAGVSGLAAAYRLRKEGVNVTVFEAESTVGGKLRSLSQDGLIYDFGPNSMVENDSDVSYLIDDLQLRGKQQYPVMQNKKYILRDGNPVLLPSNPIGLIGSKILSTKAKLDLFLEPLRWKNKSAVPEDGTKDESVGDFMGRHFGTEMVDYLVDPFIAGTTGSDPASLSVRHTFPDLWALEQRSVVMMELSTMPRCENLCLCFDSLELEDP

>Klenit1_1_2348_kfl00136_0130_v1.1|Klebsormidium_nitens/1-526

MTKPHYDPVGSVAVIGAGVSGLAAAYRLNKEGADVTIFEAGDHAGGAIQSFSKDGFVWERAANTMAENDPLIGQLIRDLNIEDKRIWPANANKRYIVKNGKAVQAPANPLALLTNNLLSPGAKLRALAEPLIWKRKKHAKQQKGEEAHGEAQEEDESIGDFLERHVGREPVEYLLDPFLAGTSGSDPQSTAVKYAYPNLYDWEESKGSLLIGAIAAQLSKKKPQSKAHGSSKVAPAGSQSDGHVADKGKKGKRHRGSFSFLDGMQTLPNALADHVGRHRLKTQTPVTRLDLTAAGPTRRGDWTVEYAARPGKSESRHFDAVLVTAPLEDVKNRMKISKGNRPFSLGFIPKITYQPMTALIQGFRAEDVKQPLEGFGVLVPSKETEFKTLGTLFNSYMFPNRAPSGFVTFTTFIGGSRHPELPGRPIDELKEVAARDLQRLLGVEGKPAFSQHIYWERAFPQYGKGYGEVLEAIRKLETDLPGLYVAGNHRDGLAVGKALVSGYDAAGRILTDLRGMVAAIPAVKLS

>Chabra1_344735_rna-CBR_g44281|Chara_Braunii/1-592

MVTVTRASTRALQRHRPLLPDAPPGRMSSLKIERTRLLTKERTLKLDSLPHVCLLIAMDKKGGGAAIGSVAVIGAGVSGLAAAFRFRKEGVAVTVFEAEVPGGVIQTEGKDGFLWEKGPNTMAENHPVVGQVISELQLQQKIAWPADQSKRYTVRNGKAVMLPASPLGFLTTEALSPQAKLRVLMEPFLWRRRGNSVGKSGGAAIDEDESVGDFLERHVGKEPVDYLVDPFLAGTAGSDPQSIAVKYGLRGLWELEQRHGSLFVGALAGALSKLAKKASARTKGVAAAPDSSKLLTSTIKGRTGSSFSFLGGMQTLPNALMERVGRDRVRTGCAVVGLDGRVLTGAKPERWEWKVSYTQRANKNKKRHEMFDAVLVTAPLHKVRNFEVVKAGKKFPLDWLPQVKYAALSVIVQAFRLEDIKRPLPGFGVLVPSKEQDFRTLGTIFSSSMFSDRVPPGYALFSSFMGGTRDQQLAGRPAAELQKLAFQDLKRLIGVEGNPVLTRHICWPQAFPVYGKGYGAVLKAMETMERDLPGVYFAGNHRNALSVGKALQSGYEAAGNIVEDLKSRRSGNPVTVASALPPPGTCQETVIC

>KAH9315263.1|Taxus_chinensis/1-841

MRVAVIGAGVSGLAAAYTLARAGVDVVLYEKEDYFGGHARTLCVDGIDVDLGFMICNRVTYSNLMAFFEELGVDLELSDMSFSVSLDNGKGCEWSSSNGLSGLFAQKSNALNPCFWQMIREIVKFKQDVLEYLKNHENNPSINVNKTLEEFIESNGYSKKFRDSYLIPICASIWSCSSEMVKGFSAASVLSFCRNHYLLQIFGRPQWLTVKGRSHTYVNKVVEELERRGCKTQRNCCVQSISATEQGILVNANGSKEIYDRCIVGAHAPDALKMLGNNATFDECRVLGAFQYVYSDIYVHRDRKFMPKRSAAWSSWNFLGSSGNHVCVTYWLNKLQNLGDTGLPFLVTLNPPCPPKQSLATWHTGHPVPSPAAAQASKELDVIQGKRGIWFCGAYQGYGFHEDGLKAGIEAAYCLLGERYNTLQDTRQMSPSWCETGARLVVNSFLQRYICAGCLCLMEDGGTIFQFEGKDERCDKKSVLRIHHPSFYWKIATRADLGLADAYIDGDFSFADGEEGLLNMFLILIANRDLGQSVTKKNHRRGWWTPMLITAVYWSATSFLQHVSKRNTLTQARRNISRHYDISNDFFSLFLDDTMTYSSAIFKGPEEPLKDAQLRKLHLLIDKARIDTSHEVLEIGCGWGSFAIEVVKRTGCKYTGITLSEEQLKLAQEKVKVACLQDKITLLLYDYRELPGFHKFNRIISCEMLESVGHEFIEEFFSRCEYLLAEDGLLVLQFISIPDKRYDEYRRSSDFIKEYIFPGGCLPSLSRTTSAMVAASNLCVEHVENIGIHYYQTLLCWRKNFHAHRSDILKLGFNDHFIRTWDYYFVYCAAGFKSLTLGDYQ

>Gb_22960|Ginkgo_biloba/1-374

MEVVRAQEKRVSGTKEVGTGEKHCGYRTGEGGRSIRTMRVAVVGAGVSGLVAAYTLARAGVHVVLYEKEDYLGGHAHTLCVDGIDMDLGFMVWNRVTYSNTMAFFEELGVDLELSDMSFSVSLDNGKGCEWGSSNGLAGLFAQKSNALNPFFWRMIREIVQFKRHVIEYLEILENNNNIHANETLGHFIESHRYSQQFRDYYLVPICASIWSCSSEKVMEFSAASVLSFCRNHHLLQIFGRPQWLTVKGRSHTYVNKVVEELEGRGCKIMTKSLVCSISTTEQGVQVIDANGVEEIYDRCIVGVHAPDALKLLGNSATFEEHRVLGAFQYAYRKNWAASLHRKVGIVLGDALMQRALPLELLVACYHAIGDTLR

>PITA_08788|Pinus_taeda/1-297

MRVAVVGAGVSGLVAAYTLARAGVHVAVYEKENYLGGHARTFSVDGIDMDLGFMVCNRVTYSNTMRFFEELGVDLELSDMSFSVSLDNGKGYEWASSNGLAGLFAQKTNVLNPFFWQMLYEILKFKQHVVDYLEKVENNPKIDTNQTLGQFIQSHGYSQQFQSCYLIPMCASIWSCSSNKVMDFSAISVLSFCLNHYLLQIFNRPQWLTVKGRSHTYVKKVIEELGKRGCKIKTNCSVQRISTTEEGILVDANGIEEIYDRCIIGAHAPDALNLLGPSATFDERRVLGAFQYFYRYL

>Prupe.1G045800|Ppersica_v2.1/1-866

MRVAVIGAGISGLVSAYVLAKEGAEVVLFEKDDYLGGHARTVTFDGVDLDLGFMVFNRVTYPNMMELFERLGVDMETSDMSFSASLDKGQGCEWGSRNGLSSLFAQKRNLFNPYFWQMLREITKFKHDAINYLEELENNPDIDRNETLGQFIKSRGYSELFQKAYLVPVCGSIWSCPSEGVMSFSAFSVLSFCRNHHLLQLFGRPQWLTVRWRSHCYVKKVRQVLESKGCQIRTSSEVHRVSTTDEGCSVLSGDGLEEIYDRCVMAVHAPDAVRILGDQATSDELRVLGAFQYVYSDIFLHRDKTLMPQNPAAWSAWNFLGSNGNKVCLTYWLNVLQNIDEKGLPFLVTLNPDHTPEHTLLKWSTSHPVPSVAASKASVELHRIQGKRGIWFCGAYQGYGFHEDGLKAGMAAAHGMLGKGCSLLSNPKHMVPSLTETGARLFVTRFLRHYISTGCLILLEEGGTIFNFEGTRKGCSLKCVLRVHTPQFYWKVMTQADLGLADAYINRDFSFIDKDKGLLNLFMILIANRDSNSSDSKLNKKRGWWTPLLFTASIASAKYFFQHVSRQNTLTQARRNISRHYDLSNDLFSLFLDETMTYSSAVFKTEDEDLKTAQLRKISLFIEKSRIEKNHEVLEIGCGWGSLAIEVVKQTGCKYTGITLSEEQLKYAQKKVKDAGLQDRIRFLLCDYRQLPNYKYDRIISCEMLESVGHEFMDEFFACCESVLADNGLLVLQFISIPDERYDEYRRSSDFIKEYIFPGGCLPSLSRVTSAMAASSRLCVEHLENIGIHYYQTLRCWRKNFLERHSEILALGFNENFIRTWEYYFDYCAAGFKTYTLGNYQIVFSRPGNTPAFADPYKGFPSASAC

>FvH4_4g04310|Fvesca_v4.0.a2/1-865

MRVAVVGSGISGLVAAYVLAKDGVEVDLYEKEDYLGGHARTVTFDGVDLDLGFMVFNRVTYPNMMEFFESLGVEMEISDMSFSASLDKGKGYEWGSRNGLSSLFAQKSNVLNPYFWQMLREITKFKHDALSYLEVRENNPDIDRNETLGKFIESRHYSELFQKAYLVPICGSIWSCPAEGVMSFSAFSVLSFCRNHHLLQLFGRPQWLTVRWRSHCYVKKVREVLESKGCRIRTSCEVHKVSTNDEGSTVVSGDEFEEIYNGCIMAVHAPDAVRILGDQATSDELRVLGAFQYVYSDIFLHRDKDLMPQNPAAWSAWNFLGSTDNKVCLTYWLNVLQNLGETSLPFLVTLNPDHTPKHTLLKWSTTHPIPSVAASKAALELPHIQGKRGIWFCGAYQGYGFHEDGLKAGMAAAHGILGNNCALLSNPKHMVPSLTETGARLFVTRFIRHYISTGCLILLEEGGTMFTFEGTRKGCSLKCVLKVHNPQFYWKVMTQADLGLADAYINGDFSFVDKDRGLLNLFMILIANRDNDSSDSKLIKKRGWWTPMLFTASIASAKYFFQHVSRQNTLTQARRNISRHYDLSNELFSLFLDETMTYSSAVFKTEDEELKIAQLRKISILIEKAKISKNHEVLEIGCGWGSLAIEVVRQTGCRYTGITLSQEQLKYAQQKVKDADLQDRIRFLLCDYRQMPANYKCDRIISCEMLESVGHEFMNDFFASCESVLAENGLLVLQFISIPDERYDEYRRSSDFIKEYIFPGGCLPSLSRVTSAMVNSSRLCVEHIENIGIHYYQTLRYWRKNFLERQSEILALGFNEKFIRTWEYYFDYCAAGFKTYTLGNYQIVFSRPGNVPAFSNPYKGFPSAY

>GWHPCBIR017199|Santalum_yasi/1-920

MALLSFAFYPLCPHQNNVKLPSNMSIEMIQEFEVKAGEGSSTLNPVMPKKEMRVAVIGGGVSGLVSAWVLAEAGAEVVLYEREECLGGHARTVTVDGVDIDLGFMVFNRVTYPNMMEFFENLGVDMEASDMSFAVSLENGQGCEWGSREGLSSLFAQKRNVFNPYFWQMVREIVKFKDDVVMYLEELQNNPDIDRNETLGHFVKSRGYSQLFQKAYIIPICASIWSCPSEGVMSFSAFSVLSFCHNHHLLQLFGRPQWLTLRWRSQHYINKVREALERRDSQIRTGCEVYSVSAFGDGCTVICQDGSQEAYNGCIVAVHAPDALKILGNQATHDEKRVLGAFQYVYSDIFLHHDKNLMPQNPAAWSAWNFLGNTDNKVCVTYWLNVLQNLGETSLPFLVTLNPSNTPEHTLLKWSTGHPIPSVAASKASVELEHIQGKRGIWFCGAYQGYGFHEDGLKAGMVAAHSMLGRSYAVLNNPKHMVPSVMETGARLFVTRFLRSYISAGCLVLLEEGGTVFNFEGSRRKCHLKTILRVHSPQFYWKVATEADLGLADAFINGDFSFVDKDEGLLNLIMILIANRDSNSSSSTFNKKRGWWTPLFFTAGIASAKYFLRHVLRQNTLTQARRNISRHYDLSNDFFALFLDETMTYSCAIFETQEEDLKVAQHRKTSLLIEKARIEKKHNVLEIGFGWGSLAIEVVKRTGCKYTGITLSKEQLKFAQMKVKEAGLQDHIRFLLSDYRQLPKTYKYDRIISCEMLESVGHEFIEDFFGSCESILAEDGLLVLQFISIPDERYDEYRRSSDFIKEYIFPGGCLPSLSRVTSAMVAASRLCIEHLDNIGIHYHQTLRIWRKNFLEKKSKILGLGFDEKFIRTWEYYFDYCAAGFKSRTLGDYQIVFSRPGNISAFSDPYEGIPSAYRLSG

>GWHPBFSI002541|Chloranthus_spicatus/1-864

MRVAVIGAGVSGLVSAYVLAKAGVEVVLYEKEDYLGGHARTVTVDGVGLDLGFMVFNRVTYPNMMEFFESLGIDMETSDMSFSVSLENGSGCEWGSRNGLSSLFAQKTNAFNPYFWQMIREITKFKKDTIKYLEELENNPDIDRNQTLGHFIESQGYSQLFQKAYLVPICASIWSCSSEGVMNFSAFSVLSFCRNHHLLQLFGRPQWLTVKSRSCIYINKIKEELESRCCQIRTGCTVKHVSIVDGGYCVSGVDGSHEVYDRCIMAVHAPDALKILGDHTTYDEARVLGAFQYVYSDIFLHRDKNLMPQNPTAWSAWNFLGTTGNKVCLTYWLNVLQNLDDTSLPFLVTLNPPHTPQHNLLKWSTSHPVPSVAASRALLDLNEIQGKRGIWYCGAYQGYGFHEDGLKAGMVAAHGVLGENFVLLGNKQHMVPSLMETGARLIVTQFLSRFISTGCLTLLEEGGTVFVFEGTSKKSCPKSVLKVHHPMFYWKVATEADLGLADAYINGDISVINEEGLLNLFMIFILSRDTNNSAKKHKSLRGWWTPLILTAGVASAKYFFQHISRQNSLTQARRNISRHYDLSNDLFALFLDETMTYSSAIFKMENEDLKVAQLRKISLLIEKARIDGKHEILEIGFGWGTLAIEVVKRTGCKYTGITLSEEQLKYAELKVKEAGLQDRITFLLCDYRQLPSTHKYNRIISCEMIEGVGHEYMEEFFSCCESVLAEDGVLVLQFISIPDERYDEYRRSSDFIKEYIFPGGCLPSLSRITSAMATASRLCVDHLENIGIHYYQTLRYWRKNFMAKRSKILALGFDEKFIRTWEYYFIYCAAGFKTRTLGNYQVVFSRPGNLDAFSDPYRGVPSAY

>PsoUNG04690.0|Papaver_somniferum/1-863

MRIAVIGAGISGLVSAYVLAKEGVDVVLYEKEDYLGGHARTVTFEGVDLDLGFMVFNRVTYPNMMEFFESLGVDMETSDMSFSVSLDKGKGCEWGSRNGFSSLFSQKRNIVNPYFWQMIREIIKFKEDVIQYIEHLETNPDIDRNETLGHFIKSKGYSQLFQKAYLIPFCGSIWSCSSEGVMSFSAFSVLSFCRNHHLLQLFGRPQWLTVRWRSQSYIDKVTEELDRRGCQIKTGYAVNSVSTYNGGCTVTCLDGSSESYDACIMGVHAPDALSMLGEEVTYEEKRILGAFQYASSDIFLHHDKNLMPRNQAAWSSWNFLGNTGNRICLTYWLNVLQNLDETGQPFLVTLNPPHTPDNTLLKWTTSHPVPSVAASKASLELDQIQGKRGIWFCGAYQGYGFHEDGLKAGMIAAHSVLGRNCNLLTNKKHMVPSLTETGARLSVTRFLGNFISTGSLILLEDGGTVYTFEGTKGKCHLKSVLRVHNPQFYWKIATQADLGLADAYINGDFSFVDKEEGLLDLFMIFIANRDMNNSVNNSKRGWWTPMFFTAGIASAKYFLKHVSRQNTLTQARRNISRHYDLSNDLFALFLDDSMTYSCAIFKKDGEDLKVAQMRKILLLIEKAKVESKHEVLEIGCGWGTLAIELVRRTGCRYTGITLSEEQLKYAEIKVKEAGLQDRIRFLLCDYRQVPDNIKYDRIISCEMLEAVGHEFMESFMNSCDSVLAENGLLVLQFISIPDERYNEYRQSSDFIKEYIFPGGCLPSLSRLTTAMAAASRLCVEHIENIGIHYYETLIRWRNNFMARQSEIVALGFDEKFIRTWEYYFIYCAAGFKTRTLGVYQIVFSRPGNLSAFKDPYKSIPSDF

>evm.TU.Scaffold_315.713|Carabica_v0.5/1-865

MRVAVVGAGISGLVAAHVLAKEGVDVVIYEKEDYLGGHAKTVTMDGIDLDLGFMVFNRVTYPNMMELFENLGVDMELSDMSFSVSLDKGKGCEWGSRNGLSSLFAQKKNVLNPKFWQMIREIVKFKADVISYVEELEKNPDLDRNETLGHFIRSRGYSELFQKAYLVPICASIWSCSSEGVLNFSAYSILSFCRNHHLLQLFGRPQWLTVRWRSQTYISKVKDDLERRGCQIRNGCEVRAVSTKDEGCVVTCEDGSLEVYNSCIVAAHAPDALKMLGEQATYDESRILGAFQYAYSDIFLHRDKNFMPQNPAAWSAWNFLGTVDNKVGVTYWLNILQNLGETGLPYLVTLNPPHTPENTLLRWSTGHPVPSVAASKASTRLNDIQGKRGIWFCGAYQGYGFHEDGLKAGTVAAQSLLRKSCTILSNPKHMVPSWTETGARLLVTKFLNGFIATGSLMLLEEGGTMFTFEGTTKKSFLKVSIKVHSPQFYWKVATQADLGLADAYINGDISFVDKNEGLLNLFMVYVANRDLKASATSSSYQRGWWTPLLLTAGLASAKYFFQHVSRQNTLTQARRNISRHYDLSNELFSLFLDETMTYSCAIFKSEDEDLKIAQLRKISLLIERARISKDHHVLEIGCGWGSLAIEVVKRTGCQYTGITLSEKQLTYAEQKVREVGMQDHIKFLLCDYRQLPETYKYDRIISCEMLEAVGHEYMEKYFSSCNSVLAEDGILVLQFISIPDERYEEYRQSSDFIREYIFPGGCLPSLSRVTSAMAAASGLCVEHLENFGIHYYHTLRRWRTNFLSKQSEILELGFDEKFIRTWEYYFDYCAAGFKTCTLGNYQVVFSRPGNVVSFGNLQKGVPSAY

>XP_020581327.1|Phalaenopsis_equestris/1-864

MRVAVVGAGISGLAAAFELCNAGAEVVIYEKEDHLGGHSRTVAVDGINVDLGFMVFNRVTYPNMMEFFEKLGVEMEVSDMSFSVSLEGGKGCEWGSRNGLASLFAQKANFFNPHFWLMLREIIKFKDDTLKYLTELEENPDLDRNETLEAFIKSHGYSELFQKAYLVPMCASIWSCPSEGVMNFSAYSVLSFCRNHHLLQLFNRPQWLTTKHRSHDYVNKLREELEARACQIKLGCAVESVSSTDNGCSVLGSNLSEEYDECIICTHAPDALKLLGGQITYEELRILGSFQYAYSDIYLHRDKSLMPQNPLAWSSWNLLGTTEEGVCLTYWLNVIQNLGSTSLPFLVTLNPSFTPKHKILKWTTSHPIPSVAASKATLELHKIQGKRRIWFCGAYQGYGFHEDGLKAGSTVAKNMLGKEVKLLKNPKQMVLSLMEVGARLVVVKFLERFVSTGALTLLEQGGTMFVFEGNNKSTHVKSVLKVHNPLFYWKIATEADIGLADAYISGYFSFVDEKEGLLNLLLVLIANRDLWNSFSHKQNKRGWWTPLLLTAGIASAWYFLQHISRRNSLSQARRNISRHYDLSNEFFSLFLDETMTYSCAIFKAGNEELQIAQQRKLSLLIEKAKIESKHEVLEIGFGWGSFAIEAVRRTGCKYTGITLSIEQLKFAKRRVEEAGLQDRVTLLLCDYRQLSTSQKYDRIISCEMIEGVGHEYLGQFFGCCEGLLAEHGLVVLQFISIPDQRYQEYRRSSDFIKEFIFPGGCLPSLSRVTSAMTDSSKLCIEHVENLGIHYYQTLICWRNNFMANRRKIMALGFDDKFMRTWDYYFIYCAAGFKSRTLGNYQIVFSRPGNLKAFNNSFEGIPSAC

>Nycol.L01043|Ncolorata_v1.2b/1-866

MRVAVIGAGVSGLVAAYTLAKAGVHVVLYEKEDYLGGHAWTVHVNGVDVDLGFMVFNGVTYPNMLQLFESLGVDLQRSEMSFSVSLDGGRGCEWGSRNGLSSLFAQKRNAFSPSFYRMLGEILTFKNDVMSYVEEHENNPDLDRNETLGQFIKSRGYSNAFRDHYLIPMCASIWSCSSEGMLSCSAYCVLSFCRNHHLLQVFGRPQWLTVKDRSHSYVNKIRAELEKRGCQIRTGCAVKSVSTSSRGCSVVTETAMSDTYDGCIMAVHAPDALKILGDGATFDEARVLGAFQYAYSDIYLHHDHSLMPKNPAAWSAWNFLGAKGDRICVTYWLNVLQSLETSLHFFVTLNPPHLPDNIVLKWSTSHPLPTVASVKASLELSKIQGERGIWFCGAYQGYGFHEDGLKGGMVAAQDLLGSRCVLLDNRKCMTPSLMESGARLIVTRFLKSYITTGYFSFLEEGGTLFEFEGTNDKDDLKSVMKIHSPSFYWKVATEADLGLADAYINGDISFVDKEEGLLNLFLILIDGRDKHNHYANCMNKGGWWKPIMLTAGLASAKFFFQHLSRQNTITQARRNISRHYDLSNDLFSLFLDDTMTYSCAIFKEENEDLRLAQMRKVSLLIEKARITNKHEVLEIGCGWGTLALEVVRQTGCRYTGITLSEEQLKYAQQRVKEACLEDKIKFLLCDYRQLPKLQKYDRIISCEMLEAVGHEYMEEFFACCDSVLAEDGIFVLQFISIPDQRYNEYRRSSDFIKEYIFPGGLLPSLNRVTSAMTTASRLCIEHVENIGIHYYQTLLQWRRNFMANKSKILILGFDEKFIRTWDYYFVYCAAGFRSRTLGNYQIVLSRPGNLSAFNNPYEGIPSALAT

>ELECO.r07.8BG0666140|Ecoracana_v1.1/1-950

MRVAVVGAGVSGLAAAHELARSGDVRVTVYEKEDYLGGHARTVAVDAGGGGAVHLDLGFMVFNRPARERESRMRVAVVGAGVSGLAAAHELARSGGDVNVTVYEREEHLGGHARTVAVDAGDGTAVHLDLGFMVFNRVTYPNMLEWFEGLGVEMEISDMSFSVSTHLGANNSRCEWGSRNGIAGLFAQKSNALSPSFWHMIREIIKFKDDTIKYLEDHENNPDMDRNETLGQFIQSHGYSQLFQDAYLIPICACIWSCPSQGVLGFSAFFVLSFCHNHHLLQLFGRPQWLTVKGRSHSYVSKVREELEGMGCQIKTGCEIKSVSSFEGGYRVIAFDGSEEIYDRVVFGVHAPDALKLLGAEATHEELRILGSFQYVYSDIYLHSDKSMMPRSSSAWSAWNFLGTTSKGVCVTYWLNLLQNIESMRPFLVTLNPPHVPDHVLLKWYTSHPVPSVAAAKASHELHHIQGNRGIWFCGAYQGYGFHEDGLKAGKAAAQCLLGKESSLLVNPKQMVPSWTESGARILVARFLNQYVSVGNLVLFEEGGTMFSFGEAGKRCHAKSVLRVHDPLFYWKVATEADLGLADAYINGYFSFVDKREGLLNLFLILIANRDTHKSSSSAGSKRGWWTPLLLTAGVASAKYFLRHISRKNTVTQTRQNISQHYDLSNEFFSLFLDPTMTYSCAIFKTDDESLETAQLRKVCLLIDKAKVERDHHVLEIGSGWGSLAIQVVKQTGCKYTGITLSVEQQKYAQRKVKEAGLEDHITFLLCDYRQIPSSRKYDRIISCEMIEGVGHEYMDDFFGCCESLLAQDGLFVLQFISIPEERYEEYRRSSDFIKEYIFPGGCLPSLARITSAMSAASRLCIEHLENIGYHYYQTLIRWRDNFMANKDEILALGFDDKFIRIWEYYFIYCAAGFKSRTLGNYQIVFSRPGNDKLGSDNPYASFPAANQAA

>HORVU.MOREX.r3.2HG0106860|Hvulgare_Morex_V3/1-868

MGKMRVAVVGAGVSGLAAAHELARSGGASVTLYEQEEHLGGSACRTVAVDGVHLDLGFLLFNRVTCPNMVDWLEGLGVEMERSDISMSASTQLASGGRCEWGGGGVSGLLAQRSNALSPSFWRMVREVFRLKTDALRYLEDHEGNLDLERSETLRQFVKSHGYSQFFQEAYLIPVCACLWSCPSQDILGFPAFFVLSCLRDHHLLELFGRPQWLTVKDGSQSFVNKVTGELESMGCRIKTSCQVKSVSCFDGDCRVLQADDSEEAYDRIVLCVAAPGALKLLGAEATQQELRILGAFHYVCSDIYLHRDESFMPLNLSAWSALNYLKTSSGGTSATYWLNLLQIIESAGTPYLVTLNPSNVPDHVLLKWNASHLVPSIAAAKASLELHCVQGNRGIWFCGAYQGYGLHEDGVKAGKATAQGLLGKQSILLSNPKQMVPSWTEAGARILVARFLNQFITLGNLILLEEGGTVFNFGKAGPKCHVKCVLQIHDPLFYWKVATEADLGFADAYINGYFSFVDKRDGLLNLFLIFISNRDAHKRCVSVSSKRGWWTPLLVTAGVASAKYFLRHVSRKNTVAQTRQNISQHYDLSNDFFSLFLDPTMTYSCAIFKVNNESFEEAQLHKVGLLIDKANVERNHHVLEIGSGWGTLAIEVVKQTGCKYTGITLSVEQLKYAKRKVKEAGLEDHITFFLCDYRQVPTDVKYDRIISCEMIEAVGHEYMDEFFGCCEFLLAQDGILVLQFTSIPEERYDQYRRSSDFIKEYIFPGCCIPSLARVTSAMAATSRLSIEWVENIGDHYYPTLMHWKGNFMENKDKILALGFDDKFIRIWEYYFVYCMAGFKTRTLGNYLIVFSRPGNDKLERTHAMKEA

>Acora.05G004500|Aamericanus_v1.1/1-871

MRVAVIGAGISGLAAAHVLLKAGGADVDVVLYEEERRLGGGEARIVATIDGVDLDLAFMSFNHVIDSQVMDFMEGLEVNTKTSDSSFSVSLPSDRGVRREWGTRHGLSGLFTRKRNVVDSYYWMVVREIMKFKDDVIRYFNEYENCFEVNLNETLGQFITSHGYSLSFQKAYLIPICVSIWSCCSLEGIMDFSAYHVLSFFRDHHLFQLFPWQKCHPVRWSSLHYVSKIQEELEKSRHCQIRTCCHVQSVASIDGVCRVTDSQGLQEDYDRCIVSVNAPDALKLLGEQATYEELRVLGAYQYVHSDTYLHRDRNFMPENPEAWSARNILGTTDNSVCLTYWLDALQNTENTSLPFLVTLNPPHAPQQNLLKWTTSHPIPSIAAAKASFDLNEIQGKRGIWYCGSYQGYGFCEDGLKAGIVAAHGLIGQNHANLSKSLAVFSFLESGTRLIVTRFLQHYISIGSLILLDGGSIFRFEGAKGKTSPKCILKVNNPQFYWKVVTQADLGFADAYINGDISFIDKSEGLLNFFMIFIANRDARLNNNRHKSTKGWWTPLLSTAVVGSAKYFFQHILRHNSPTQARRNISRHYDLSNDLFSLFMDETMMYSAAIFKSENEPLIVAQQRKISLLIEKARIDSKHEVLEIGCGWGALAIEVVKQTGCKYTGITLSEEPLKFAEMKVKEAGLQDRITFILCDYRQLPTPKKYNRIISCEMIEAVGHEYMEEFFGSCEALLAEDGLLVLQFTSIPDERYDHYRRSSDFIKEYIFPGGCIPCVSRVTSAMANASRLCVEHLENIGIHLYQTFECWRKQFMENQSKIQELGFDEKFIRTWEYYFVYCAAGFKSRTLKNYQIVFSRPGNHDAFSDPYKSFPAA

>166747|Smoellendorffii_v1.0/1-864

MKRIAVIGAGVSGLVAAYTLCKAGCHEVVLYEKENYFGGHARTEDANGIAVDLGFMVFNQVTYPNMVSLFDELGVDMETSDMSFSVSLDGGKGCEWGSTGLAAVFAQKSNLVNPYFLRMIREIVKFQDDVLNYLNKLEQGDDAVDPNQTLGSFLQLHGYSEKFRDCYLIPVCASIWSCSSEQVLGFSAASILTFCRNHHLLQIFGRPQWLTVKDRSIKYVEKIVAELTKMGCELRLGCAVSRISSTEGGVKLILESGEKVVYDTCLVAAHAPDALTLLGETATFDERRLLGAFQYSYSDIYLHRDRALMPKSTAAWSAWNFLGSVQGRVCVTYWLNLLQNLGDTEGKPYLVTLNPPRPPKNLISKWRTGHPVPSPGAATALKELDTIQGARKIWFAGAYQGYGFHEDGLKAGMAAANSLLGAKYQALSNVKQLNFTWCQSQARREVTKFLQKYIVFGKLQFIEAGGTILDFVGKDCDLTSKLRIHHPDFYWKIATRSDLGLAEAYMDGDFTCIDSQNGLLNLLLIIIKNRDWMLKSLKDEKRGWWTPPILTAAFGSAVAYLKHKLRNNSIVNARKNISEHYDLSNEMFSLFLDETMTYSCAIFKGPEEPLKDAQLRKIHHLIDKARIDNSHEVLEIGFGWGSMAVEVVRRTGCKYTGISLSEEQLAFAKQLVTQERLEDKITLKLCDYRCLEGCKKYNRIISCEMMEAVGPEYYDEFFAKCDYLLAEDGLAVIQVITVPEERYDELRRSSEFIKEYIFPGGSLPCLAALTAAMFRASSLCVEHLENIGLHYYQTLMCWRRNFNSKTSEIAKLGFSESFMRKWDYYFIYCAAGFLTCTLGNLQIVFSRSGNAAALGNPYRSFPTA

>Mapoly0062s0039|Mpolymorpha_v3.1/1-889

MGSARAGKMRVAVVGSGVAGLTAARTLAQSGANVVLYEKDSHIGGHANTLHVDGVALDIGFMVFNQVTYPNMVAFFDEIGVEMEKSDMSFAVSLDGGKGCEWGSKSVGGLFAQRSNAVNPFFWNMIREMLKFKADVLAFLARIESGDPGIDEATTLGDFLTSHGYSQKFKECYLIPVCASIWSCSSLTVLGFSAVSILTFCRNHHLLQLFGRPQWITVKGRSETYVSKVVDELQERGAEIRAATAVTRIESLDDGRVRVEDERGGNEIFDECIVAAHAPDALEMRGEYATSNEKKILGAFQYAYSDIYVHRDENFMPRNKAAWAAWNFLGDINNRCCVTYWLNLLQNLGDTGLPFLVTLNPAKKPERVVSLWRTSHPIPSPEAADASKRLGSIQGHRGVWYCGAYQGYGFHEDGFKAGLVAAKQLLGQKAEVLRNVKQMVPSWTEYSAQQAVVAVLHKFIRTGQLQILEAGGSILDFIGSEKGCSLKVSIRVNSPQFYWKIATRADIGLADAYVDGDFSIVGDKNGLLHFLQLIIANRDINRSEVVKQTRGWWNPVFVTAAVGGAVSFLRHKLRGNSLTNARRNISRHYDLSNELFALFLDDTMTYSSAIFKGPNDTLKDAQLRKLHLMIQKAKIEPHHEVLEIGFGWGSMAIELVRRTGCNYTGITLSKEQLAFATDRVKQEGLEDKITFKLCDYRSLEGHHKFNRIISCEMLEAVGHEYYKEFFSRCDYLLAKDGLIVLQVITIPDERYDEYLKSSDFIKEYIFPGGSLPCISALTSAMAAGSTLSLEHLENIGLHYFQTLMCWRENFLAKASECRKLGFSDKFIRMWDYYFIYCAAGFQTCTLGNLQLVFSRPGNVAAVGNPYVSFPMGLASDSSLEAFLECRKKL

>Tle2c02828.1|Takakia_lepidozioides/1-916

MINLCLRRNCTWTYLIISFLLQHHEASEERAEILRSEDKMRVAVVGAGVAGLTAARTLALAGADVVIYEKENYVGGHARTVHADSVALDTGFMVFNRVTYPNMIELFEETGVEMETSDMSFSISLDEGRACEWGSKSLAGVFAQKRNLLNPYFYHMIFEMVKFEGDTLRYLERLEGNDVTVNSNETLQEFLDAHGYSKKFCECYLVPVCASIWSCPSETVMGFSAASILTFCRNHHLLQLFGRPEWLTVKGRSETYVSKIVAELEGAGSKIRTSCTVTHIFTSSYGVCVEDEKGGVETFDKCIVGAHAPDALEMLGKEATYEERRILGAFQYRYSDIYVHRDTRLMPKNPAAWAAWNFLGDISGHVCVTYWLNILQNLGDTGRPYLVTLNPPKPPEHIVNEWRTSHPVPSPAAAQAARHLESIQGQRNIWFCGAYQGYGFHEDGLKAGLEAANAMLGRKFQPLPLVKQMVPTWLESGSRLAVVGFLRKFIRTGRLQLLESGGTVLDFIGEEKGCPLHSSLRILRPSFYWKVATRQDLGLADAYIDGDFSFVDKKDGLLNFLLILISNRDIGRMSSSQSVQNRGWWSPVMLTSAVGFALSYFQHALRGNSLTNSRRNISQHYDLSNDLFASFLDETMTYSCAIFKGPEEPLVCAQMRKINHLIDKARVESTHKVLEIGCGWGSLALQVVRRTGCRYTGITLSEEQLKFAQALAKKACLEDRITFKLCDYRTLSGYHKYNRIISCEMLEAVGHEYYKDFFSRCDYLLAKDGLLVVQVISMPEERYNEYRRSSDFIKEYIFPGGCVPAFSTLTAAMTAGSSFCVEHMENIGIHYCETLLRWREKFHMNRSLSSLERFWFWCSEILRLGFSEKFINMWDYYFLYCAAGFKTCTLGNLQIVFSRPGNVTLFGNPYISFPMA

>evm.model.Chr11.1585|Isoetes_sinensis/1-865

MKVAIVGAGVAGLIAAHTLAKSGFKVVIYEKESYVGGHARTLHFEGTALDLGFMVFNRVTYPNMVAFFEENGVDMEVSDMSFSVSLDDGKGCEWGSRGLSGLFAQKRNLLNPYFLQMVREIVKFNYDVHGYLQKVEEKDPEIDVNETLGNFLQRHGYSKKFRDCYLIPVCGSIWSCSSEQVMNFSALCILTFCQNHHLLQLFGRPQWLTVKGRSQTYVEKIVAQLKEMGAELRLNCAVDNIISRTEGIHVVDVKGTDTIYDKCIIGAHAPDALQMLGDEATYEERCILGAFQYSYSDIYLHRDTQFLPKHPASWSAWNFLGSQEGRVCVTYWLNLLQNLGNTGKPFLVTLNPTKKPLSVISKWQTGHPVPSPAAVRALQQLDQIQGKRGIWFSGAYQGYGFHEDGLKAGLRAAKNLVLEGYNPLQNYKQMVFSWSETGARYFVISFLRSFVSLGRIQLFEDGGTIVDIDGKDKGSNLNCRLRIHSPSFYWKVATRIDLGLAEAYMDGDFTFIDEKHGLLDFLMLLIRNRDLKRFSTKNRGWWTPVLFTATVSSGVYYFKHALRKNTINSARRNISKHYDLSNDLFALFLDETMTYSCGIFKGPDESLKDAQLRKIHRLIDKAKITSTHEVLEIGFGWGSMALEVVRHTGCKYTGISLSQAQLEFAQERVKKAGLGDKITLKLCDYRTINEHHKYDRIISCEMLEAVGHEYYKEFFSKCDYLLAPNGLMVVQVISIPDERYDEYRRSSDFVKEYIFPGGCLPSLSILTKAMVDGSSLCIEHLENIGIHYFQTLLCWRDKFLSSKSIVKELGFDERFIKMWDYYLVYCAAGFKTCTLGTLQIVFSRSGNFETLGNPYMTFPSILTIV

>evm.model.Chr12.1517|Isoetes_sinensis/1-865

MKVAIVGAGVAGLIAAHTLAKSGFKVVIYEKESYVGGHARTLHFEGTALDLGFMVFNRVTYPNMVAFFEENGVDMEVSDMSFSVSLDDGKGCEWGSRGLSGLFAQKRNLLNPYFLQMVREIVKFNYDVHGYLQKVEEKDPEIDVNETLGNFLQRHGYSKKFRDCYLIPVCGSIWSCSSEQVMNFSALCILTFCQNHHLLQLFGRPQWLTVKGRSQTYVEKIVAQLKEMGAELRLNCAVDNIISRTEGIHVVDVKGTDTIYDKCIIGAHAPDALQMLGDEATYEERCILGAFQYSYSDIYLHRDTQFLPKHPASWSAWNFLGSQEGRVCVTYWLNLLQNLGNTGKPFLVTLNPTKKPLSIISKWQTGHPVPSPAAVRALQQLDQIQGKRGIWFCGAYQGYGFHEDGLKAGLRAAKNLVLEGYNPLQNYKQMVFSWSETGARYFVISFLRSFVSLGRIQLFEDGGTIVDIDGKDKGNNLNCRLRIHSPSFYWKVATRIDLGLAEAYMDGDFTFIDEKHGLLDFLMLLIRNRDLKRFSTKNRGWWTPVLFTATVSSGVYYFKHALRKNTINSARRNISKHYDLSNDLFALFLDETMTYSCGIFKGPDESLKDAQLRKIHRLIDKAKITSTHEVLEIGFGWGSMALEVVRHTGCKYTGISLSQAQLEFAQERVKKAGLGDKITLKLCDYRTINEHHKYDRIISCEMLEAVGHEYYKEFFSKCDYLLAPNGLMVVQVISIPDERYDEYRRSSDFVKEYIFPGGCLPSLSILTKAMVDGSSLCIEHLENIGIHYFQTLLCWRDKFLSSKSIVKELGFDERFIKMWDYYLVYCAAGFKTCTLGTLQIVFSRSGNFETLGNPYMTFPSILTIV

>Dicom.21G068400|Dcomplanatum_v3.1/1-854

MRIAVVGAGVSGLIAARVLARAGMKVVVYEQEDYVGGHARTIQIDGIPIDVGFMVFNQVTYPNMVSFFEEIGVDMESSDMSFSVSLNEGKGCEWGSDSLAGLFSQKRNLFNPYFIQMIREIFRFQHDVLCYLEQLERQDDSIDWNETLGQFLTTHGYSQKFRNCYLVPVCGSIWSCSTEQVLNFSARTILTFFRNHHLLQVLGRPQWMVVKGRSETYVNKVVEELESNGCKIRTSCKVHQISTSDKGVLVTDSHGGDALYDKCIIGAHAPDALAMLGDASYEERAILGAVQYSYSDIYVHRDKSFMPKNVSAWSSWNFLGDTGGRVSLTYWINKLQNLGDTGLPILVTLNPLKLPNGKITKWCTSHPVPSPSAVSAMSKIGTIQGKRGVWFCGAYRGYGFHEDGLKAGLTVAKGILGEQFSMLENVRPFVLTWSEIAGRLAAVTFLRSFIRRGQLKIIEDGGTVLDFVGKDAGLRMRCTLKIHRASFYWKVATRADLGLADAYVDGDFSCADATNGLLMLFMILIVNRDFKDTSSKSSFRGWWTPALPTAIVSSSVSYLTHILQNNSLTNARRNISRHYDLSNDLFSLFLDETMTYSCAIFEGPKEDLKDAQLRKLHRLIDKARVQSYHEVLEIGCGWGSLAIELVQRTGCKYTGITLSEEQLKWSERRVKEAGLQERITLKLCDYRKVHGCHQYDRIISCEMIEAVGHEYYMEFFRTCDKLLAKDGLLVVQVITIPEARYDEYRRSSDFIKEYIFPGGCTPSLSTLTAAMSRASSLCVEHLENIGIHYYQTLIHWQNNFRAKHSELYRLGFDTKFLRTWDYYFAYCAAGFKTCTLSNLQIVFSRPGNITNFGN

>EVM0010766.1|Lycopodium_clavatum/1-864

MRIAVVGAGVSGLIAARVLARAGLKVVVYEQEDYVGGHARTIQVDGTSIDVGFMVFNQVTYPNMVSFFEEIGVDMECSHMSFSISLNEGKGCEWGSDSLAGLFSQKRNLFNPYFIQMIREIFRFQHDVLCYLKKQELQDDNIDWNETLGQFLTTHGYSEKFRDCYLVPVCGSIWSCSTEQVLDFSARTILTFFRNHHLLQVLGRPQWMGVKGRSETYVNKVVEELKSYGCEIRTSCKVHQISTSDKGILVMDTYGGNALYDKCIIGAHAPDALAMLGDDASYEERLILGAVQYSYSDIYVHRDKSFMPKNAAAWSSWNFLGDTGGRVSLTYWINKLQNLGDTGLPILVTLNPSKLPNGKIKKWCTSHPVPSPSAVSAMSKIGSIQGKRGIWFCGAYTGYGFHEDGLKAGLAVAKGILGEQFSLLENVRPFVLTWSEIAGRMTAVTFLRSFIRRGQLKIIEDGGTVMDFVGKDAGLKMRCTLKIHRASFYWKVATRADLGLADAYVDGTSHVLMQQMGCWCFSWYWLCSDMNPILIMNRDSKDTSSKSIYRGWWTPLLPTAIVSSSVSYLTHIMQNNSLTNARRNISRHYDLSNDLFSLFLDETMTYSCAIFEGPKETLKDAQLRKLHRLIDKARVQSNHEVLEIGCGWGSLAIELVRRTGCKYTGITLSEEQLKWSERRVKEAGLQERITLKLCDYRKVPVCHQYDRIISCEMIEAVGHEYYMEFFRTCDGLLAKDGLLVVQVICIPEERYDGYRRSSDFIKEYIFPGGCTPSLSTLTAAMSRASSLCVEHLDNIGIHYYQTLIYWRDNFRAKHSELYRLGFDTKFLRTWDYYFAYCAAGFKTCTLSNLQIVFSRPGNTATFGN

>Adc30893|Adiantum_capillus-veneris/1-911

MRCRPNADTISCLSGINLLIAVKLTIPLPSTPLPGASLLVDLCLRMTRVAVIGAGIAGLVAARTLALAGLEVVVYEKEDNVGGHARTVQADGVSLDLGFMVFNRVTYPNMLKLFDDLGVEIEESDMSFSISLDEGKGWEWSSNGLLGLFAQKKNLLNPYFLRMIKEIMKFQHDVVRYLDSIETDDDDAYVKDTLENFVKTRGYSNEFLQFYLVPMCASIWSCSSQHVLQSSAVSILTFCRNHHLLEVFGRPQWLTVKGRSQSYVTKVIEELRAHGCDLRLNCTATKVSSLHQGIQVEDAEGHTDVYDRCIIGAHAPDALTLLGSDATTEEHTILGAFQYSYSDIYLHRDRKYMPKNTAAWSAWNFLGCETSGVCLTYWLNKLQNLGDTGLPFLVTLNPSTPPQDSTLKWTTSHPIPSPAAAVASRHLDSIQGRRGIWFCGAYQGYGFHEDGLKSGLSAANDLLGCTFKPIKNVRHRSSTWPEYSAQLIVTSFLRQYIKIGSLQLEEAGGSILRFGEQSNPSSVKSVLRIHHSSFYWKIATRADLGLADAYIDGDFTCVDAERGLLSLLLILINNRDESNQTTQHSAKRSWWNPVLLTAVIGSASSYLRHLSNRNTLTNARRNIAAHYDLSNALFALFLDETMTYSCAFFQSPTESLEDAQLNKIRRMIDKAQIEQSHEVLEIGFGWGSMALEIVRRTGCKYTGITLSEEQLKFAQERVKEAGLEDKITFLLCDYRTLLGHKKFNRIVSCEMLEAVGHEHFQVFFRRCDHLLAKDGIVVVQVITIPEERYDAYRLSSDFIKEYIFPGGCLPSFSTLTAAMTAASSLCVEHMENIGIHYFHTLLEWRQRFNANQSAILQLGFSEKFLRMWEYYFVYCAAGFQSCTLGVAQIVFSRPGNVPAFGDAWRRRCFYE

>Adc08418|Adiantum_capillus-veneris/1-866

MTRVAVIGAGIAGLVAARTLALAGLEVVVYEKEDNVGGHARTVQADGVSLDLGFMVFNRVTYPNMLKLFDDLGVEIEESDMSFSISLDEGKGWEWSSNGLLGLFAQKKNLLNPYFLRMIKEIMKFQHDVVRYLDSIETDDDDAYVKDTLENFVKTRGYSNEFLQFYLVPMCASIWSCSSQHVLQSSAVSILTFCRNHHLLEVFGRPQWLTVKGRSQSYVTKVIEELRAHGCDLRLNCTATKVSSLHQGIQVEDAEGHTDVYDRCIIGAHAPDALTLLGSDATTEEHTILGAFQYSYSDIYLHRDRKYMPKNTAAWSAWNFLGCETSGVCLTYWLNKLQNLGDTGLPFLVTLNPSTPPQDSTLKWTTSHPIPSPAAAVASRHLDSIQGRRGIWFCGAYQGYGFHEDGLKSGLSAANDLLGCTFKPIKNVRHRSSTWPEYSAQLIVTSFLRQYIKIGSLQLEEAGGSILRFGEQSNPSSVKSVLRIHHSSFYWKIATRADLGLADAYIDGDFTCVDAERGLLSLLLILINNRDESNQTTQHSAKRSWWNPVLLTAVIGSASSYLRHLSNRNTLTNARRNIAAHYDLSNALFALFLDETMTYSCAFFQSPTESLEDAQLNKIRRMIDKAQIEQSHEVLEIGFGWGSMALEIVRRTGCKYTGITLSEEQLKFAQERVKEAGLEDKITFLLCDYRTLLGHKKFNRIVSCEMLEAVGHEHFQVFFRRCDHLLAKDGIVVVQVITIPEERYDAYRLSSDFIKEYIFPGGCLPSFSTLTAAMTAASSLCVEHMENIGIHYFHTLLEWRQRFNANQSAILQLGFSEKFLRMWEYYFVYCAAGFQSCTLGVAQIVFSRPGNVPAFGDAWRRRCFYE

>Aspi01Gene11063|Alsophila_spinulosa/1-860

MRVAVIGVGVAGLVAARTLALAGVEVVVYEKEKYVGGHARTVEVDGIAVDLGFMVFNRVTYPNMITLFEETGVELEGSDMSFSVSMKEGRGWEWSSNGLSGLFAQKKNLLNPSFLRMIREIIKFQREVLEYLESLETEDDDLSSNETLGHFTKKRGYSEEFLSCYLVPMCASIWSCSSRDVLQSSAAAVLKFCRNHHLLQIFGRPQWLTVKGRSQSYVGKLVAELKAKGCQFRMNCAVSQVLTSEQGIQVGDAEGNVDTFNHCIIGAHAPDALKMLGNNATDDEHQILGAFQYSYSDIYLHRDVNFMPKSSAAWCAWNFLGCKDDRVCVTYWLNKLQNLGDTGRPFLVTLNPSNPPQDVILTWYTSHPIPSPAAVVASKQLNSIQGKRGIWFCGAYQGYGFHEDGLKSGLSAANDLLGSTFDPLKNIKYTSPTWPEAAARLVVIAFLRQYIKTGCLQFQEAGGPVISFGEHKSGISHKCILRINHSSFYWKIATRADLGLADAYIDGDFTCVDSEKGLLNLLLVMIDNRDQHQQSRQHMSRRGWWRPVLFTAIVGSASSYIRHFLRGNSLTNARRNIAAHYDLSNDLFGLFLDETMTYSCALFKNPTERLKDAQLRKIIHMINKAQIQQSHEVLEIGFGWGSMALEIVKRIGCKYTGITLSEEQLKFAQKQVKEANLEDKITLLLCDYRTLPGFQKYDRILSCEMLEAVGHEHFKEFFGRCDHLLAKDGIVVVQVITIPEERYDEYRASSDFIKEYIFPGGCLPAFSTLTAAMTAGSSLCVEHMENIGIHYYHTLLAWRKKFHSNHSEILKLGFSETFLRMWDYYFIYCAAGFHSCTLGVLQIVFSRPGNVAAFGDPWKT

>Azfi_s0215.g058389|Azolla_filiculoides/1-861

MRVAVVGAGVSGLIAARTLALAGVHVVLYEKEAHIGGHARTHRIESDNVAVDLGFMVFNRVTYPNMIDLFEEIDVEIEDSDMSFSVSLDEGRGWEWGSNGLAGLFAQKKNVLNPSFLRMVKEILKFKDDVFVYLNDIEKQNNESSVNDTLGHFVKSRGYSQQFLSCYLVPMCASIWSCSADNILQSSAVAILKFCKNHHLLQIFGRPQWLTVKGRSQNYVQKVSVCLWQLVAELNAKGSEIRENTAVTQISTADQGIQVIDAQGNIENFDQCIIGAHAPDALQMLGTDATDDEQRILSAFKYSHSDVFLHRDTKFMPKNSAAWCAWNFLGCSNDNVCVTYWLNKLQNLGHSGKPYLVTLNPQDPPQDPILIWHTSHPIPSLASVTASRQLSTIQGRRGIWFCGAYEGYGFHEDGLKSGLSAAKGLLGCTFNALNTINYRSTTLLEAGARLVVISFLNQFIKSGSVEFHEAGGPVITFGDGTNKAHRSILRIHHSSFYWKIATRGDLGLADAYIDGDFTCADTGAGLFNLFMVLIDNRDQQKQASNYVSRRGWWSPVVFTSVFGSASSYIRHLLRGNSLTNARRNIAAHYDLSNDLFSIFLDETMTYSCAIFKNPTDSLKDAQLRKIHHIIKKAQIQQKHEVLEIGFGWGSMALEVVKLTGCKYTGITLSEEQLKFAQQRVKKAGLEDNITFELCDYRTLPGYHNEMLEAVGHEHFQEFFRRCDHLLAKDGVLVVQVITIPEERYDEYRTSSDFIKEYIFPGGCLPAFSTLTAAMSSGTSLCVEHMENIGIHYYHTLLAWRKNFHANHSEILKLGFSEKFLRMWDYYFIYCAAGFQSCTLGVLQIVFSRPGNVAAFGKAWLD

>Klenit1_1_8691_kfl00345_0140_v1.1|Klebsormidium_nitens/1-871

MSAQQRIAVVGSGVAGLAAAWALVDAGHRVVLYEKDSHVGGHAWTQTVDGHAVDVGFMVFNRVTYPNLVRWFEEAGVETEVSDMSFAVSLDGGKGCEWASTGLAGLFAQKRNLANPHFILMLREMLLFHTDVLAYLARLEAADPALDPNETLGAFLAARRYSAKFRDCYLVPVCAAIWSCGAEGVLGCSASAILSFLRNHHMLQIFGRPPWLTVTNRSQTYVAKIVAKLQAAGSEIRLSTPVTSVERTTSGVVITDAAGATATFDRVVMAVHAPDALTLLGKSASSAEQHILGAHTYSQSDIYLHRDPAYMPRNPAAWSAWNFLGSPAGKVSVTYWLNKLQNLGDTGAPILVTLNPQTPPAHIVSQWTTSHPVPSPAAAAASRALPSVQGQDRIWFCGAYQGYGFHEDGFKSGLVAAKSLLGVPFAPLPYPRQMAPSWAESVAKAAVLAFLRQFVHLGALRLFEDGGSVYSFGQAAGDISAKQARDGRHFVVGLRIHAPAFYWKVATRADLGIADAYVDGDFTCLPDVMDFLMLLVVNRDRTRRPGQVRAKKRGWWSPALLTAAVGSGASYVRHLTRKNTLSNARRNIADHYDLSNALFSLFLDETMTYSCAIFASPHEPLKDAQLRKLHAMIDKARITSSHHVLEIGFGWGSMSMELVRRTGCHVTGITLSEQQLQLATARVKAAGLQAKISFMLCDYRALPGIGTFDRIVSCEMLEAVGHEFYPDFFHHCDRLLAPDGIMVVQVITTPEERYEEYRCSSDFIKEYIFPGACCPSLTALMQAMTTHSNLCVEGVENIGVHYVPTLLRWRDTFHEKQSEIEAMGFGPKFVRTWDYYFAYCAAGFQTRTIGDLQIIFSRPGNTSGLGVVTSF

>Chabra1_335979_rna-CBR_g31184|Chara_Braunii/1-965

MGTNAGPEIANLTLYWDEAQYVDNLQRVDPSAARRYAFTYRFIDDVLTFDCLPPSSAHYGLEWKETTATDGSCTFLGAKLQTRADGSLRLSVFDKTVAWDFPVIRYPSATSNIPSHQPAGVFTGQFTRFEQICDNWRDFKTAATMLVRRLLSRGHAPSTLACGWTCYIRARQRRHPIHVAIVGSGVSGLSAAWEIVKAGHEVVLYEKDNHVGGHAWTLNVDGIAVDVGFMVFNRVTYPNLVKFFEELPVEMEQSDMSFAVSLDGGRGCEWGSAGIGAFLAQKSNVVNPPFLFMIREMLRFERNVLEFLKREEGKEAGMAENSDTLGHFLNSHGYSQKFRQCYLIPVCASIWSCSVQTVLDFSAMEVFRFLRNHHMLQLLGRPQWLTLKGRSKTYVNKVVQMMEIHGSQVRTKCGIVSVKPNNSSSGVEITDVEGRKENLGETGRPVLVTLNPEKTPQHLVSSWKMEHPIPTAGAAKAGRELKAIQGSRGVWFCGAYLGYGFHEDGFKAGQSAAMDLLGKPFQSLPLVKQFVPSYLESFAKWVVLQFLKDFIKVGHFELLEAGGSVMSFGEPVTGINPDIKITPLRCSVRVIRPAFYWKVATRADLGIADAYVDGDFTCVGDMLDLLLVLIANRDRVRAPGKVERSTRPWWKPPLLTAWLGSAMAYFRHMTRNNTLTMARQNIVAHYDLSNDMFKLFLDETMTYSCAIFKDPSESLKTAQLRKLHALIDKARIKPHHRVLEIGFGWGSLSMEIVRRAGCHVTGITLSRRQLELAEKRVRAAGLQDKIDFKLVDYRNLPGIGCFDRILSCEMLEAVGHEYYGTFFQNCDRLLAPNGLLVVQVISTPEERYEEYRKSSDFIKEYIFPGSTCPSLTALVSAMEASSSFSLEHVENIGTHYATTLLRWRENFMSHKREIKDLGFSDAFIRTWDYYFTYCAAGFQTRTLGDLQLVFSRPGNVEALGLPGVL

>Klenit1_1_5471_kfl00216_0110_v1.1|Klebsormidium_nitens/1-475

MSSRTPIVSMKVAIVGAGISGLSAASVLAKAGHRVTIFEKSAQLGGHARTVLVDGTPVDVGFQLLNRQVGDCVQLQFVNYATTYPTLVRFLGEYQVELEPSPLTFAFDFTDTPAQGPSSADLALFAEDALSFLEAAPQPEVTLGHFLEGGGYSAAFQRGYLVPVCAAVWFTSKEAILGASAYTVLSFLRDHKMLQASGRLDWLTVKGRTQTYISKVVSSLKEHGVQILTNSPVAAIERTAAGVTITANGVSATFDKLVLATHAPDALALLGEGATPAEKAVLGAFTYNKSTLVLHRDAALMPADRATWGAWNFVGSAAGASSVTYWLNKLQHLERAAPLFLTINPHVPPRHVVDGDYPWLHQGPHPDAAAANAHVAQLQGGNNTWFAGAYQGFGFHEDGCKAGMAAALSLLGDTTPAPVAVEALPLAFKGCKPLAPIEDRLQVPVATERRIVRASYDDVRHDVQVRNALPLRSFS

>Gb_22956|Ginkgo_biloba/1-299

MEKANPEQKAPKTKEGRSSENSIKPTEDNKPKYSRNEDQLWTSQRMVGQERKECLPGTRKHSEGPTLADGTPRRQDAEEPNTATKGTRNQRNLPAKLMPIVAIAQRRQRGHRRRTSPRRGQMLCRKATPEPLSSEKPNRVESLCRMPFLHQFLMLIRTMRVAVVGAGVSGLVAAYTLARAGVHVVLYEKEDYLGGHAHTLCVDGIDIDLGFMVCNRVTYSNTMAFFEELGVDLELSDMSFSVSLDNGKGCEWGSSNGLAGLFAQKSNALNPFFWRMITEIVQFKQHVIEYALFPLFSND

>GSMUA_AchrUn_randomG12680_001|Macuminata_v1/1-572

MKLSGTIQRRYWWHPNPDKKCSFHKCSGSNKLESFRNSEFMGFKMKAPIWLLKDKKPRHGASPLQVFCKDFPRPELENTVSFLEAAQLSSSFCNGPRPRKPLKVVIAGAGLAGLSTAKYLADAGHKPIVLEARDVLGGKVAAWKDNDGDWYETGLHIFFGAYPNMQNLFGELGINDRLQWKEHSMIFAMPNKPGEFSRFDFPETLPAPFNGIFAILRNSEMLTWPEKVRFALGLLPAMLGGQAYVEAQDGLTVTEWMRRQGVPDRVNDEVFIAMSKALNFINPDELSMQCVLIALNRFLQEKHGSKMAFLDGNPPERLCKPIVDHIESLGGEVWVNSRTQKIELNPDGTVKHFLLSSGNIISGDVYVIATPVDILKLLLPQEWKDILYFKKLEKLVGVPVINVHIWFDRKLKNTYDHLLFSRSPLLSVYADMSVTCKEYYDPDRSMLELVFAPAEQWISCSDQEIVDATMQELAKLFPDEIAADQSKAKILKYHVVKTPRSVYKTVPDCEPCRPLQRSPVKGFYLAGDYTKQKYLASMEGAVLSGKLCAQAITQDYDVLVAQAAQREVQVSI

>FvH4_4g12690|Fvesca_v4.0.a2/1-568

MSLGASVSATNLTHQAKVINTHHPHSPALSFHGSEIVGRNLGFVSSHASTRLRKGVQPLKVVCVDYPRPELDNTVNFLEAALLSSSFRSSPRPAKPLKVAIAGAGLAGLSTAKYLADAGHKPILLEARDVLGGKIAAWKDKDGDWYETGLHIFFGAYPNIQNLFGELGIDDRLQWKEHSMIFAMPNKPGEFSRFDFPEVLPAPLNGIWAILKNNEMLTWPEKVKFAIGLVPAILGGQAYVEAQDGLTVKEWMRKQGIPDRVTTEVFIAMSKALNFINPDELSMQCILIALNRFLQEKHGSKMAFLDGSPPERLCSPIVDHIQSLGGEVQLNSRLQKIELNNDGTVKSFVLNNNSVIEADAYVCAAPVDIFKLLVPENWKEIPYFKKLDKLVGVPVINVHIWFDRKLKNTYDHLLFSRSPLLSVYADMSVTCKEYYNPNQSMLELVFAPAEEWISCSDSEIIDATMEELAKLFPDEIAADQSKAKILKYHVVKTPRSVYKTIPDCEPCRPLQRSPLEGFYLTGDYTKQKYLASMEGAVLSGKLCAQAIVQDYELLVARGQKRLAEAAAR

>GWHPBFSI006238|Chloranthus_spicatus/1-534

MGHRLGNPDSWFVGTRQRHEAASLQVVCMDYPRPELENTVNFLDAAYLSSSFRSSSRPSKPLQVVIAGAGLAGLCTAKYIADAGHTPILLEARDVLGGKVAAWKDNDGHWYETGLHIFFGAYPNMQNLFGELGINDRLQWKEHSMIFAMPDKPGQFSRFDFPEVLPAPLNGIWAILKNSEMLTWPEKVRFAIGLLPAMIGGQAYVEAQDGLTVKEWMKRQGVPDRVSDEVFIAMSKALNFINPDELSMQCILIALNRFLQEKNGSKMAFLDGNPPERLCMPIVDHIQSLGGQVQLNSRIQEIELNPDGTVKHFLLTNGNVIEGDVYVIATPVDILKLLLPQQWKDISYFSRLEKLVGVPVINVHIWFDRKLKNTYDNLLFSRSPLLSVYADMSVTCKEYYDPSRSMLELVFAPAEEWIPRSDTEIIEATMKELSKLFPDEIASDQSKAKILKYHIVKTPRSVYKNVPNCEPCRPLQRSPIEGFYLAGDYTKQKYLASMEGAVLSGKLCAQAIVQDYDLLVARAQKNTKAEASIV

>GWHPCBIR021046|Santalum_yasi/1-530

MGFKLKMTDVHAVGTRPGNSLQVVCMDSPRPELENTVNFLDAAYLSSSFRASSQPNQPLQVVIAGAGLAGLSTAKYLADAGHKPIVLEARDVLGGKVAAWKDNDGDWYETGLHIFFGAYPNMQNLIGELGINDRLQWKEHSMIFAMPNRPGSFSRFDFPDLLPAPLNGIWAILKNNEMLNWPEKVKFAVGLLPAMLGGQAYVEAQDGLTVKDWMRKQGIPDRVTDEVFIAMSKALNFINPDELSMQCILIALNRFLQEKHGSKMAFLDGNPPERLCVPIVNHIKSRGGQVHLNSRVQKIELNDDGTVKSLLLSNGNVVVGDAFVIATPVDILKLILPEDWMGIPYFRRLEKLVGVPVINVHIWFDRKLKNTYDNLLFSRSPLLSVYADMSVTCKEYYNPNQSMLELVFAPAEEWISCSDSEIIDATMKELAKLFPDEISADQSKAKILKYHVVKTPRSVYKTVPDCEPCRPLQRSPIEGFYLAGDYTKQKYLASMEGAVLSGKLCAQAIVQDYELLAARRHKMLANASLL

>Pn38.178|Piper_nigrum/1-574

MDISGPTCFRNQPNPQCVWRFCRAPKYEFKYLTAFGCTKSMGFQLRIPNVFSVPRGSRSSVKPMKVVCIDYPRPELENTANFLEAAYMSAFFRSSPPPNKPLQIVIVGAGLAGLSTAKYLADAGHRPILLEARDVLGGKVAAWKDDDGDWYETGLHIFFGAYPNIQNLFRELGINDRLQWKEHSMIFALPNKPGEFSRFDFPELLPAPFNGIWAILKNNELLTWSEKVRFALGLLPAMVGGQAYVEAQDGLTVKEWMRKQGVPDRVNDEVFIAMSKALNFINPDELSMQCILIALNRFLQEKHGSKMAFLDGNPPERLCMPIVNHITSLGGEVRLKSRLQKIELNHDGTVKHFVLTNGSIIEGDAYVVATPVDILKLLLPNDWKALSYFQKLEKLVGVPVINVHIWFDRKLKNTYDHLLFSRSSLLSVYADMSVTCKEYYDPNRSMLELVLAPAEHWISQSDEEIIEATMKELARLFPDEIAADNSKAKILKYHVVKTPRSVYKTVPNCEPCRPLQRSPVKGFYLAGDYTKQKYLASMEGAVLSGKLCAQAVTQDYDQLIARTQSSEMTETSIA

>HORVU.MOREX.r3.4HG0405810|Hvulgare_Morex_V3/1-576

MDTGCLSSMNIAGAKQAGSFAGQLHTQRCFTSSSVQALKTSHRTTSLGLRNKGKGSRRGLRALQVVCQDFPRPPLENTVNYLEAGQLSSSFRGSERPSKPLQVVIAGAGLAGLSTAKYLADAGHKPIVLEARDVLGGKLAAWKDEDGDWYETGLHIFFGAYPNVQNLFAELGISDRLQWKEHSMIFAMPNKPGEYSRFDFPETLPAPLNGVWAILKNNEMLTWPEKVKFAIGLLPAMLGGQAYVEAQDGLTVSEWMEKQGVPDRVNDEVFIAMSKALNFINPDELSMQCILIALNRFLQETHGSKMAFLDGNPPERLCMPIVNHIQSLGGEVRLNSRIQKIELNPDGTVKHFALTDGTQITGDAYVCAAPVDIFKLLVPQEWREISYFKRLDKLVGVPVINVHIWFDRKLKNTYDHLLFSRSSLLSVYADMSLACKEYYDPNRSMLELVFAPAEEWIGRSDTEIIEATMLELAKLFPDEIAADQSKAKILKYHVVKTPRSVYKTVPNCEPCRPMQRSPIEGFYLAGDYTKQKYLASMEGAVLSGKLCAQSIVQDSKMLSRRSQESLQSETPVASQL

>Mapoly0108s0060|Mpolymorpha_v3.1/1-580

MQGSLCLQCGHARVVESSVLSSGPAAAASAQKNQPKANAVGFRGSEFCASQTSSFNGKLRAASRRRQYSPLKVEAVDYPRPDIDGTVNYLEAAALSAYLGTAKRPEKPLKVVIAGAGLAGLCTAKYLADAGHIPILLEARDVLGGKVAAWKDADGDWYETGLHIFFGAYPNMQNLFGELGINDRLQWKEHSMIFAMPDKPGEFSRFDFPDVLPAPLNGVWAILKNNEMLSWEEKIRFAIGLLPAIVGGQNYVEAMDKYTVKEWMAKQGVPQRVNDEVFIAMSKALNFIDPDDLSMQCVLIALNRFLQETHGSKMAFLDGAPPERLCKPIVDHFSALGGEVRLNSRLQKFVLNEDGTVKHFQLQDGTIVEGDVYVSSMPVDVFKLLIPDQWANIPYYRKLDKLVGVPVINVHIWFDRKLKNTYDHLLFSRSPLLSVYADMSTTCKEYYDPNKSMLELVFAPADEWIGRSDADIIDATMKELATLFPDEIAADQSKAKILKYHVVKTPRSVYKTIPNCEPCRPSQRSPIPRFYMSGDFTKQKYLASMEGAVLSGKLCAQAIVQDYEKLASPTPSLEKQKTLV

>AANG004907|Anthoceros_angustus/1-547

MEILRASLSVGASRSEYCAALRLGSSPVRGATRRRIQQLQIVCKDFPRPDIDTSPNYLEAAALSAFLRNAPRPARPLKVVIAGAGLAGLSTAKYLADAGHKPVLLEARDVLGGKVAAWKDEDGDWYETGLHIFFGAYPNVQNLFGELGINDRLQWKEHSMIFAMPNKPGEFSRFDFPEIPAPFNGVWAILKNNEMLTLQEKIQFALGLLPAIVGGQGYVEAQDGLTVREWMRKQGVPEKVNEEVFIAMSKALNFIDPDELSMQCVLIALNRFLQETHGSKMAFLDGAPPERLCQPIVDYFTQLGGELRFNSRLQNIELNADGTVKHFALIDGTIVEGDLYVSAMPVDILKLLLPAQWKPMPYFKRLDKLVGVPVINVHIWFDRKLKNTYDHLLFSRSPLLSVYADMSTTCKEYYDPDKSMLELVFAPADKWISRSEADIIQATMEELAKLFPDEIAADGSKAKIVKYHVVKTPRSVYKTVPDCEPCRPLQRSPIPGFYLAGDFTKQRYLASMEGAVFSGKLCAQAIAQDHNIASTARVDSAKQAALT

>Klenit1_1_401_kfl00103_0130_v1.1|Klebsormidium_nitens/1-581

MQAAALNRVLPSGALTAAGKDHVQGRAVQQHLEAHRSHVLCGKSFLGGRRLVCESKRAALQRRTFKRLQVVAADFPRPAIDGTPNFLDAAQLSARLREAPRPKRPLKIVIAGAGLAGLSTAKYLSDAGHHPILLESRDVLGGKVAAWKDKDGDWYETGLHIFFGAYPNMQNLFGELGINDRLQWKEHSMIFAMPNKPGEFSRFDFPDILPAPFNGVWAILKNNQMLSWPEKVQFALGLLPAMVGGQKYVEEQDSLTVSDWLKKQKIPKRIEVEILGAMSKALNFIDPDKLSMQCVLIALNRFLQETHGSKMAFLDGAPPERLCQPIVEYFSAKGGEVRLNSRLKEIVLNDDGTVKHFALANGQEVTGDLYVSAMPVDILKLLLPEKWKPMPYFKKLDKLVGVPVINVHIWFDRKLKNTYDHLLFSRSPLLSVYADMSTTCKEYEDPNKSMLELVFAPAKEWISRSDQDIIDATMGELAKLFPDEIAADGSKAKILKYHVVKTPRSVYETVPDCEPCRPLQKSPIERFYLAGDYTKQKYLASMEGAVFSGKLCSEAIVKDWNEKSLEELPEAEPDLVPASVA

>Mvestita_C94g20980-RA|Marsilea_vestita/1-574

MLNLGFASNSLVLCRGRSSSNSSYDINMASFRSTSISDSSFLGSTFCLAPPAVSETRNRYKPLKVSCRDYPRPNIDDTANYQEAAALSAYFKNNGKPARPLRVVIAGAGLAGLSAAKYLADAGHIPVLLEARDVLGGKVAAWKDKDGDWYETGLHIFFGAYPNIQNLFGELGINDRLQWKEHSMIFAMPNKPGQFSRFDFPETLPAPINGVWAILRNNEMLTWPEKIKFAIGLLPAILGGQSYVEAQDGLTVKEWMQKQGVPDRVNDEVFIAMSKALNFIDPDELSMQVILIALNRFLQETHGSKMAFLDGNPPERLCKPIVEYFSARGGELQMNSRLQKIDLNDDGTVKQFVLTDGRTVEGDVYVSAMPVDILKLLLPQKWKDIPYFEKLKKLEGVPVINVHIWFDRKLKNTYDHLLFSRSPLLSVYADMSLTCKEYNDPNRSMLELVFAPAEKWISCTDEQIIEATMEELAKLFPDEIAADGSKAKILKYHIVKTPRSVYKTVPNCEPCRPLQRSPIQGFYLAGDFTKQKYLASMEGAVLSGKFCAQAIVQDQDELRTRASSIDKVDNKMLV

>Sacu_v1.1_s0048.g013549|Salvinia_cucullata/1-593

MHCLCPPEHLFFPPRTKAGASPLRPSLGYQNSSFLARLSFPPLPFPAQSGLPKHSRPLQVLCRDYPRPNIEETTNYAEAAALSASFKNVPRPSKKLRVVIVGAGLAGLSTAKYLADAGHIPILLEARDVLGGKVAAWKDEDGDWYETGLHIFCKLKPFTIMKSLQYRLVIILVKCFLKEISIIIFPVGAYPNIQNLFSELGINDRLQWKEHSMIFAMPDKPGEFSRFDFPENLPAPLNGVWAILRNNEMLTWSEKVKFAIGLLPAILGGQSYVEAQDVLTVKEWMLKQGVPERVNDEVFIAMSKALNFIDPDELSMQVVLIALNRFLQEKHGSKMAFLDGNPPERLCQPIVDYFSTRGGELWTKSRLQKIELNNDGTVKHLLLNDGRVVEGDVYVSAMPVDILKLLLPSQWKEIPYFEKLKKLEGVPVINVHIWFDKKLKNTYDHLLFSRSSLLSVYADMSITCKEYNDPNRSMLELVFAPAEKWISCSDEEIIKATMAELAKLFPDEIAADGSKARILKYHVVKTPRSVYKTVPNCEPCRPIQRSPIPRFYLAGDYTKQKYLASMEGAVLSGKLCAQAIVQFGGYPGELCTV

>Pp6c18_10300|Ppatens_v6.1/1-540

MSNLMTEFDAVIVGAGIAGLYQLYRLRNLGLRTHVLEAEKEVGGTWYLNKYPGARCDIKSIMYSYSFSPELEQEWNWSENYSNQPEILRYINHVADRFDLRKDITFNTRVTGALYNESSKRWEVHTDQGKTIRTQFLIMATGCLSTPKQPEIPGIEKFQGSSYYTGRWPDKPVDFTGKRVAVIGTGSSAVQSIPIIAQQASQLTVFQRSAACCVPGLNYKLTADEIAASKASYSTLRKAQRESPGGFVDERGSKSALEDSEEDRQARYEMLYQRGTLDAIIVCAYNDLVINKEANDTFSEFIRQKIRSIVKDPVVADSLSPRGYAVGTKRICLDYGYYETFNLDHVHLVDLTKTPILEMTEKGIRTSEKDHVFDAIVYATGFDAITGTLTRIGIQGKNKLLLKDKWAKGPRTYLGIAIAEFPNLFTITGPFSPSVFTIMTVAIEQHVDWITDCLKYLREHGVEEIEATVEAEDAWVEHSLAVANMTLFPKTASYYTGGNVEGKPFMLYPYVGGLANYRQVCDGVAAKGYEGFKLKTKGIV

>GWHPBFSI000439|Chloranthus_spicatus/1-541

MGSVEQTPDFDVLIIGAGLSGIFSLHRMRGLGLTARVLESGSAEGGTWFWNRYPGCRFDSESYTYHFTFSQEVLDEWDWTEHFAPQPETLKYAQFLTDKFQLRDGMQFDTKVKTAHWQEESRTWLLTDAAGKQYSSRFLISAMGLLNDPTLPNIPGVETFEGEAFHTSRWPSEWSVDGKRVGIIGTGATAIQIIPELVKKPLQHLTVFQRTANWSAPLRNDKISPEEMVELRKQYPDIHRMCNESGGGFRYSLDPRKTMDVPEEERLAFWEYLYTQRGFSKWLGSFADHLTNHEANKAYSDFHANKIRQRVNDPVVAEKLVPKTHGFGTRRVPLETNYFDVYNRDNVRLIDFTADSPIEKINKKGVVLANGEEIELDLLIYATGFDAITGSLTRGIDLRGVNGISLGDKWEDGINTFLGLFVKDFPNMAMIMGPHQAFGNIPRSIEFAVESVTRFMKYCRDNEITRAEPKDKSVTEWTDHVLELSKGMLSMEVDSWLTGVNKNVKGKQKRTVARYFGTGPMYRKAVNDVADRGWSDMELKK

>SlFMO1_XP_069144694.1|Solanum_lycopersicum

MALISSILQHAKSHSSSKISTYIQRRLFSNDVRKENIVIVGAGIAGLASALSLQRFGIRSLVLEQAESLRTEGSSITLSKNGWKALDAIGVGDELRSQFLELQGVVIKSDDGKELNSFGFKDEDKSQELRVVERRVLLETLASKLPPNSISFSSKLSNIETSENGTNTLLQLQDGTRVSAEVVIACDGVWSPTAKWMGFRQPKYAGHIAFRGLGYFPEGQPYEPKVNYTYGSGLRSGFVPASKTKVYWFVLCNSSSPGPRITDPSILRQQAKELVRDWPVADILNLINLTPDDTLTRNPLYDRWLWPVISPPPFSGRTVLAGDAWHPMTPNMGQGACCALEDSIVLTEKLAEAMKSKHISVEDAFKAYGSERWRRVFPLTVMANRVGALLQSENKLICSVRNNLLVPKLKPRALTKHTNYEFEQAKRDGH

>FvH4_5g20080|Fvesca_v4.0.a2/1-452

MGIVSLSLVLHSRLVSPYHSQTRPIRLSQSNWFQAPIPARTKHTSLSIIRAQSGARKEEIVIVGAGIAGLATALSLHRLGIGSLVVEQAESLRTSGTSLTLFKNGWRVLDALGVGDDLRNQFLEVQGMVVTTAEGNELRSFKFKEEDESQEVRAVERRVLLETLANQLPQGAVRFSSKLAKIEKIEDGDTLLQLVDGTQLSAKIVIGCDGIRSPIAKWMGFPEPKYAGHCGLRGLAFFPGGHKFEPKLNQIYGRGQRAGIVPISPTKVYWFVCFNMPSPGQKITDPAVLKKLAKELVTNWPSDLLDIIDHTPDDTISRTPLVDRWLWPSISPPASAGRVVLVGDAWHPMTPNLGQGACCALEDSVILARKLAAAIESGPAAIEDALSSYGSERWPRIFPLTIRANLIGSLLQWENPVVCSVRNNVIIPKLVKLGPMLEHTNFDCQPLQNQVV

>TESI_007669-RA|Tetracentron_sinense/1-433

MARPSSALLLLHSSCSSSTPLHHHRTRRSPHGQSSFPIRTRTNPVRLSIVEAQADVRKEDVIVVGAGIAGLATAVSLHRLGVRSLVLEQAESLRTSGTSLTLFKNGWRVLDEIGVGDDLRSQFLEIEGMVIKSSDGRELRSFKFNDEAEGQEVRAVERRILLEALATQLPPDTICFSSSWQRLKELQQILIGCDGIRSPVAKWMGFSEPLYVGHCAFRGLAVYPDGQPFEAKVNYVYGRGLRAGYVPVSSTKVYWFICFNSPSPGPKISDPSVLKRQAQELVRSWPSELLDVIKLTPDETIIRTPLVDRWLWPVLSPSASSGGVVLAGDAWHPMTPNLGQGACCALEDAVVLARKLAGALESGPSSVQDALRSYGRERWTRIFPLTIRANIVGSLLQWENPVVCTVRNNVVIPKLVRLGPLLEHTNFNCEPLK

>PITA_14159|Pinus_taeda/1-119

MSHCIKGRKERISSKVCHSSHANNLQGSDRIREEEVVIVGAGIAGLATALSLHRLGVKPLVLEQADSLRTGGTSLTLSKNGWRALDVLGVAEDLRKEYKKILGCVSGKGKKIKSCLRVE

>PITA_20450|Pinus_taeda/1-125

MSHCIKGRKERISSKVCHSSHANNLQGSDRIREEEVVIVGAGIAGLATALSLHRLGVKPLVLEQADSLRTGGTSLTLSKNGWRALDVLGVAEDLRKEYKKILGLQIQTNDGAVLRYFSFQAEDNR

>Klenit1_1_8835_kfl00352_0070_v1.1|Klebsormidium_nitens/1-454

MGRRASSQVNRSTCTRAVNAELQTESKPQSRADPSVEEEVVVVGGGIAGLSTALALHRMGIKSLVLESAPGPRDSGAAIALWRNAWRALDVLGVGEKLRHDYILLDRVQLCTKKGKVVREFGLDEVKGGPHEYRGVHRSALLKVLASELPEGTVRYGTSVQQVLPLDGGQGTELILGGGERLSAKVVIGCDGTKSAVARHLALPQPNYAGYSAIRGVANFPNGHNQKSAVRQIWGAGVRAGTYPMNRTDVYWFTVFNSPEEEPCPSCGRHGTEAAGAGLGRGWGGDILNIIRATPAEATTRSDIRDRWTLPKPVGGQSTWGRGSVTVAGDAAHPMTPNLGQGGCAALEDAVVLARELAKAKVSNDTKTSQNGHRADKSTSLDPQAVEDVLRRYERERIPRAAKLTVQSWVYGTLLQIGFPPVTFARDNVVVPYLFQPDHWLDHTLYDCGELPTS

>evm.TU.supercontig_21.240|Cpapaya_ASGPBv0.4/1-373

MEEIEIVIAGGGICGLATALALHRKGIKSVILERAECVRATGSAIGVFPNGWRALDQLGVGSDLRPTAVPLLGVGEGRCLKRSDLVKALANHLPSGTIRFNSQIVSVELDPLSSYPILHLKDGTTIRAKVLIGCDGANSVIGDFLGLKPKKVFSSLAVRGLTEFANRHGLPIEGVRIRRGNTMSGGVPLNDHLLYWFLLHKWNPTDLAKVAHDPELIQKMAIETLIEHKFPAERIEIVKRCDLNSLSLTRLRYRSPWEVLLGNFRKGTVTVAGDAMHVMGPFLGQGGSAAMEDAVVLARCLAGKLGAKQKIEEALDEYIKERRMRLVWLSTQTYLVGSLLDTSSLIKKIFIMIFMALLFRNPTAHAKYDCGRL

>evm.TU.supercontig_21.242|Cpapaya_ASGPBv0.4/1-373

MEEIEIVIAGGGICGLATALALHRKGIKSVILERAECVRATGSAIGVFPNGWRALDQLGVGSDLRPTAVPLLGVGEGRCLKRSDLVKALANHLPSGTIRFNSQIVSVELDPLSSYPILHLKDGTTIRAKVLIGCDGANSVIGDFLGLKPKKVFSSLAVRGLTEFANRHGLPIEGVRIRRGNTMSGGVPLNDHLLYWFLLHKWNPTDLAKVAHDPELIQKMAIETLIEHKFPAERIEIVKRCDLNSLSLTHLRYRSPWEVLLGNFRKGTVTVAGDAMHVMGPFLGQGGSAAMEDAVVLARCLAGKLGSKQKIEEALDEYIKERRMRLVWLSTQTYLVGSLLDTSSLIKKIFIMIFMALLFRNPTAHAKYDCGRL

>evm.TU.AsparagusV1_09.401|Aofficinalis_V1.1/1-402

MRKQEEYEDIVIVGAGISGLATALALQRKGIRSIVLERSETLRATGAGISIYLNGWHALDHLGVGNQLRAKSTPINEIHGEWVYNDDTEVIPISKEGLRCVKRSDLIEALANSLPAESIRFGCQIVAIETDPVTSFPIVHTADGCIMRTKILIGCDGSNSIVASSLGLKPANLFQTREVRGYTSYPNGHGLSNHFLHLKGDKLFVGRIPVDENLVFWFVDQEFHPKDLETRRDPILIRDRTLEAVKDCPKEVIEMVKHCDLSSLSLTRIRYRAPWHLALKNLCKGTMTVAGDAMHQMDPSIGQGGCTALEDAVVLARSLSREMSVIQSERKISDQEIQNRAEVALRRYTSERKWRILILSTKSFLIGSLFGASWLKRFTFLTLFSTFFGGRFGHTQYNCGHL

>Gb_17956|Ginkgo_biloba/1-428

MKKRRFGEAKEDETLITEGFNEAKFEEFEGIVIAGGGIAGLATALALHRVGLKSLVLERADGLRATGTALTIWKNAWKALDALGIGDELRQHHLQLQAVQMVSTVVKEYSCEIDGKRCTELRCVKRSALLEAMAAALPPESIRLNSKIVRLQQKPKSKFSSTVELEDGTVIRAKAVIGCDGVNSVVANWLGISPPTFPGRCGIRGYATYPEGHHMEPKFYLHFGKGVNIGHMPVNEKEVYWFVTWGSNSQDSKTAHDPELIRQETLLQLKDFPETLTEMVQRSGSDTLNMASLRLRWPWRMLFGTRLWKGTVTAAGDAMHPMTPDLGQGGCAALEDAVVLGRCLAEALNRRVERDEEEAERIEEALNKYVEERRWRWAWLLGTAYFTGFVQQGSWAIVRLFRDKIFLRFRSSEMYFSPTQVDCGGLPL

>Rhdel06G0016000.1|Rhododendron_delavayi/1-389

MQEIMEIEEDIVVVGGGIAGLTTALGLHRLGLRSLVLESSESLRIAGFALTLWTNAWRALDALGIDNSLREHYPLIPSRCVRRKELLETLERELPHGTLRYSSKVVSIEETGYRKLLHLADGSTLKTKVLIGCDGVNSLVAKWLGLPKPVYSGRSAIRGFAEYPDGHGFHQSFHLYYEGGVRYGYFPCGANCFYWFCTYNSSLQYDEGMQENPTKMKQFVLSKISQLPKQAAEVVEMTDLDSISCSPLKLRLPWNLLLGNFSKGNVCVAGDALHPMTPDIGQGGCSALEDGVVLARCLAEALLKKPINEIQNEEEEYARIQRGLEKFAKERRWRSFSLISAAYVVGFIQQRDGKVMRFLREKFLLRFAASIGRKMADFHCGELNISGSK

>KAH9304316.1|Taxus_chinensis/1-415

SLYQNCVFCGISNPTMEEDIVVVGGGVAGLATALALHRVGLKSLVLERADSLRTAGAAFILWPNAWKALDSLGVAHALRPRYSLLDGIRGCSNCTGVSKEVQLKQGGSSARQECRCIERMAVLEALAKELPEGSVRFNSKIVHLYKNRVSPFTTLELADGTFLTAKIVIGCDGVYSVVAKWMGFPAAKSAGRVAIRGMATYPYTHHFQKLMIQVWGRGVRTGFVTCTDKQVYWFITRKSQPQDAEICSDGERVRGAALELVRDLPEHITEAIKSSPADTINLAELKVRWLWPWEWGRGKGRGSVTIAGDALHPLTPDLGQGACLALEDAVLLGKLLGEAMETVNMVDWGEPEVGKIESCFNKYLNARRRRVSAVISAAFVTGIVQEGSSRLMRFVRDWIFFPVFSMSYLLYFSES

>VIT_204s0044g00270|Vvinifera_v2.1/1-413

MERIAEEEVVIVGAGIAGLATAVALKRVGIRALVLERSDCLRATGAALTLFPNAWRALDALGVSHKLTPLYAVREKSYVTNVTTGAIQEVSLSRNNRGGPITVHRKALLESLAEELPSNSIRFSSKLISFEVEAQAEEGLYIIRLEDGTVITAKVLIGCDGVHSLVARKLGLAEPVNSGRSAVRGLAVFQEGHGLGDEVQQFLDVNIRAGMVPLNDKEIYWFLTFKSTLQGEAMARDPEQIQRQVIENFAKNFPPTYAEVVRHCDLSTLTWAPLLMRLPWHLIFGNVSKGTMTVAGDAMHPMTPDLGQGGCSALEDAVVLGRHIGNSFIDNGRLVPGAVAGAIEGYVKERRWRTTGLITGSYISGWAQLGGDGWLMKLFRDVIFYRFIFKRLVGGADYDCGKLPLLNEQNKPQ

>GWHPBAUK032979.1|Macadamia_integrifolia/1-424

MEKSEDIVIVGGGIAGLATALALKRVGIKALVLERSPELRKTGTALSLFPNAWLALEALGVAHKLTSIYSPLRRGFVTNVANGAIQQFTYTGISEKYSGPITVHRRALLETLAGELPTGTIRFSSKLTSIRTAAIDDEGSSGVILSLDDGTVIKAKVLIGCDGLHSVVASWLGLKEPVKANWSAIRGLAVFPEGHGYKKEEIYQFIDKGKRAGFVPLTDKELYWGMSYSSTIKGEETATDPKSLQKDVIENLAKDFPPSYLEVAQHADLATVSWAPLMFRYPWNLLFGQVYKGSITVAGDAMHPMTPDLGQGGCSALEDAVVLGRHLGNSFLLNGRRIVAIEAAKAIEGYVKERRWRAATLITGSYLSGWVQQGGSSSIMKFLRDTIFYKFLYRRVFDSIGYDCGKLPSVTSPSQSDDDQIKID

>FvH4_1g23770|Fvesca_v4.0.a2/1-407

MGTITVENVVIVGGGIAGLATGVALKRAGIEALVLERSKGLRVNGTALTLFPNAWFALDSLGVSHKLASYTPSLKGYITDIDTGEVRELSFTKANGDAVGPRSVHRKALLETLAGELPVHSIRFSSKISAIDTQQHEGSSIAIVHMENGTIIKAKVLIGCDGVHSVVSRWLGLREPVHSGRSAVRGLAVYPQGHGLKQIVQQYAGSGRRAGFVPLNDKEVYWFFTGTSPAKGTSLAEDPEEIQKEILENYANDLPPIYLDVVQHSDLSTLTWAPLMFRYPWNVVFGSLSKQNITVAGDAMHPMTPDLAQGGCSALEDAVILGRHIGTSFKHNGHVLVPKEMSEVLSKYVEERRWRIALLITGSYIAGWVQQGGSGWGSKFLRDTVYYKFLFMKLVRYVNYDSGKLDF

>FvH4_1g23750|Fvesca_v4.0.a2/1-407

MGTTVEDVVIVGAGIAGLATAVALKRAGIEALVLEKSEGLRTTGAALTLFPNAWIALDALGVSHKLASYTPSRKNHVTDLDTGEVQTVSLIKANGDAVGPRSVHRKALLEALADELPIHSIRFSSKISAIDTQQYEGSSIAIVHIENGTVIKAKVLIGCDGVHSVVSRLLGLSEPVHSGRSAVRGLAVYPQGHGLEQEVRQYVGLGRRAGFVPLTDKEIFWFFSGTTPAKGTSLAKDPEEIQKEILENYAKDLPPIYLDVVQHADVSTLTWAPLMFRYPWNVVFSSLSKQNITVAGDAMHPMTPDLAQGGCSALEDAAILGRHIGTSFKQNGHVLVPKEMSEVLSKYVEERRWRVVFLIAGSYISGWVQQGGSGWGMKFLRDAIFYKFIFPKMVSYVNYVDCGKLDF

>FvH4_1g23740|Fvesca_v4.0.a2/1-406

MGTTVEDVVIVGGGIAGLATAVALKRAGIEALVLERSEGLRATGAALTLFPNAWFALDALGVSQKLASYAHSRKLYITDLDTGEVQEVPFIKPNGDAVGPRSVHRKALLEALADELPVHSIRFSSKISAIDTQQHEGSSIAIVHMENGTVIKAKVLIGCDGVHSVVSRWLGLRDPVLSGRSAVRGLAVYPQGHGLEHNVRQYVGTGKRAGIVPLTDKEIYWFISYTSPAKETSLEDFPEDIQKEVLENYGKDIPTIFSDVVKQSDLSTLTRAPLKYRYPLNVVFGTLSKQNITVAGDAMHPMTPDLAQGGCSSLEDAVILGRHIGRYFVQNGQVLVAKEISEVLGKYVEERRWRVALLITGSYISGWVQQAGSGWGGKFLRDVIFYKFLYLKMVRYVNYDCGKLEF

>BUSI_006658-RA|Buxus_sinica/928-1359

MFLRDLIVYKILFLKETDSESLEAMETRSEDVVIVGAGVAGLATAIALKRVGVRALVLERSPELRASGAAVNLFPNAWVALHALGVAHKLTSVYPPLEMGHIFNVVTGATTDVSFSAMDRMGRGVRPVHRKLLVEALAEELPADAIRFSSKVATIETQQLDNSLTSMAVLHLEDGAIIKAKVLIGCDGVHSVVARWLGLKEPVYSGRYAVLGLSKFPEGHEFKHDVHQFLGEALRAGCVPLSDKEVYWFTGFRRSTGEMIREPEQIQNIVIEEAKGFPARYLDVVRRADLDTLYWVPLMLRVPWDIIVRKASKGPITVAGDAMHPMTPDLGQGAGSALEDAVVLGRHIGNSFLQKGGIVAGEIEGALESYVKERRWRAAGLITGSYISGLSQQGGSPWMKFLRDLIVYKILFLVMALVARYDCGKLPTASSN

>PSME_00015181-RA|Pseudotsuga_menziesii/1-2212

MAAAGLAKRGAISGTRIDERNAPLCLTPRFAASSSLVSFVRLEGSNRFSRSTRRNSLSVSGQRLRLGAADGEIMGRRMHLWRTEGPGRSPKLITVAVRCMALSEIPQERLGLYDPSLDKDACGVGFVAELSGEASRKTVMDAIEMLKRMSHRGACGCETNTGDGAGILVALPHDFLAEAVKDAGFDLPPLGEYAVGMFFLPTSETRREESKNVFNKVAESLGHVVLGWRRVKTDNAGLGNSALQTEPVVEQVFLTQSSRSSGEFEEQMYILRRTSMVAIRAALNLQYGGVRDFYICSLSSRTIVYKGQLKPDQLKSYYFADLGNEKFTSYMALIHSRFSTNTFPSWDRAQPMRVLGHNGEINTLRGNVNWMKAREGLLKCQSLALSKDEMQKLLPIVDASSSDSGAFDGVLELLVRAGRSLPEAVMMMIPEAWQNDKTMDPDRRSLYEYFSALMEPWDGPALVAFTDGRYLGATLDRNGLRPGRFYVTYSGRVIMASEVGVVEIPPEDVCQKGRLNPGMMLLVDFEKHVIVDDQALKKHYSTSRPYSEWIKRQKITLKDVVYSVPEAEREPEQIIGSVKVDGSDDTMENMGIYGLLSPLKAYGYTVESLEMLLLPMAKDGIESLGSMGNDAPLAVMSNRPKIIFEYFKQMFAQVTNPPIDPIREAIVTSMECMIGPEGDLTETSEKQCHRLSLKGPILSINQTEALKKMNYRGWRSKVLDITFPKEHGSRGLEETLDRICSEARLAIREGYKLLVLSDRATSSKRVPISSLLAVGAVHHHLVSKLERTRIGLIVESGEPREVHHFCTLVGFGADAICPYLAIEAIWRLQIDGKVPPKENGTLHAKEELVQKYLKASKSGMLKVIAKMGISTLASYKGAQIFEALGLSSEVVQRCFAGTPSRVEGATFEMLARDTLRLHDLAFPSRLLPKGSADAHALSNPGDYHWRKDGEVHLNDPLAIAKLQEAARTNSVAAYKQYSKIIQDLNKHCNLRGMLKFKEVPDRKIPLEEVEPASEIVKRFCTGAMSYGSISLEAHTTLAMAMNKIGGKSNTGEGGENPSRLEPLPDGSMNPKRSAIKQVASGRFGVSSYYLTNADELQIKMAQGAKPGEGGELPGHKVIGDIAVTRNSTSGVGLISPPPHHDIYSIEDLAQLIYDLKNANPGARISVKLVSEAGVGVIASGVVKGHADHVLIAGHDGGTGAARWTGIKNAGLPWELGLAETHQTLVANDLRGRTVLQTDGQLKTGRDVAIAALLGAEEFGFSTAPLISLGCIMMRKCHKNTCPVGIATQDPVLREKFAGEPEHVINFLFMVAQELRELMSEIGFRTVKEMVGRADMLEMDKEVINSNEKLENIDLSLLLRPAAEIRPEAAQYCVQAQDHGLDMALDQELIRLSKAALEKAIPVYMEMPVRNVNRAVGTMLSHEVTKRYNQMGLPTDTIHAKLTGSAGQSLGAFLCSGITLELEGDSNDYVGKGLSGGKIVVYPPRRSRFDPKENIVIGNVALYGATSGEAYLNGMAAERFCVRNSGAKAVVEGIGDHGCEYMTGGTVVILGKTGRNFAAGMSGGIAYVLDVDGKFDSRCNLELVDLEKVEEEDDIMTLKMMIQQHQRHSNSQLAREVLADFEAFLPKFVKVFPRDYKRVLQEMKAVNAAKEAEEADLMKKDAFEELKRMVEVDSSQHKKDDGAYKQNELKVVAQRPTKVDNAEKHRGFLKYERETLAYRDAAERINDWEEVVEENKPGPLLKTQSARCMDCGTPFCHQENSGCPLGNKIPEWNELVHQGRWRDALDRLLETNNFPEFTGRVCPAPCEGACVLGIIENPVSIKSIECAIIDKAFEEGWMIPRPPVKRTGKKVAIVGSGPAGLAAADQLNKAGHSVTIYERADRIGGLMMYGVPNMKTDKLDIVQRRVDLMAEEGVNFVVNANVGVDPVFSVEKLRDENDALLLACGATKPRDLPVPGRKLMGVHFAMDFLHANTKSLLDSGLQDGKFISAKGKRVVVIGGGDTGTDCIATSIRHGCSNVVNLELLSRPPNSRAPGNPWPQWPRVFRVDYGHQEAITKFGKDPRSYEVLTKRFIADDNGNVKGLELISVKWEKDESGKFQLKEMEGTEEIIEADLVFLAMGFLGPEQDIAEKLDMERDNRSNFKANFGDFATSVDGVFAAGDCRRGQSLVVWAIAEGRQAAAQIDKFVMKEDQLITTCEDRVEDKDKRPMFTLKT

>PITA_10092|Pinus_taeda/1-2211

MAAAGLAKGGAISGIKIDDRNVQLRLVPHSAASSSSVRFPSLEATNRFPRSKRRNSLSLSGKGLRLGAADGEIMGRKMHLWRTEGPGRSPKLITVTVRMALSEIPQERLGLYDPSLDKDACGVGFVAELSGKASRKTVTDAIEMLVRMSHRGACGCETNTGDGAGILVALPHEFLAEAVKDAGFDLPPQEEYAVGMFFLPTSETRREESKNVFNKVAESLGHVVLGWRHVITDNNGLGKSALQTEPVVEQVFLTKSSRSSAEFEEQMYILRRTSMVAIRAALNLQHGGVRDFYICSLSSRTIVYKGQLKPDQLKSYYFADLGDDKFTSYMALIHSRFSTNTFPSWDRAQPMRVLGHNGEINTLRGNVNWMKAREGLLKCQNLALSKDEMQKLLPIVDASSSDSGAFDGVLELLVRAGRSLPEAVMMMIPEAWQNDKNMDPDRRALYEYFSALMEPWDGPALVAFSDGRYLGATLDRNGLRPGRFYVTYSGRVIMASEVGVVDIPPEDVCQKGRLNPGMMLLVDFEKHGIVDDEALKKHYSMSRPYSEWIRRQKITLKDIVSSVPEAERVPEQVIGSVKVDGSDDTMENMGIYGLLSPLKVYGYTVEALEMLLLPMAKYGTESLGSMGNDTPLAVMSNRPKIIFEYFKQMFAQVTNPPIDPIREAIVTSMECMIGPEGDLTETSEKQCHRLSLKGPILSIEETEAIKKMNYRGWRSKVLDITFPKERGSRGLEETLDRICSEARLAIREGYKLIVLSDRATSSKRVPVSSLLAVGAVHQHLVSKLERTQIGLIVESGEPREVHHFCTLVGFGADAICPYIAFDAIWRLRIDGKIPPKKNGTLHTKEELVQKYIKASKSGMLKVLGKMGISTLASYKGAQIFEALGLSSEVVQRCFDGTPSRVEGATFEMLARDTLRLHNLAFPSRLLPKGSADAHALPNPGDYHWRKDGEVHLNDPQAIAKLQEAARTNSVAAYKQYSKIVQDLNKHCNLRGMLKFKEVPDRKIPLEEVEPASEIVKRFCTGAMSYGSISLEAHSTLAIAMNKIGGKSNTGEGGEDSSRLGPLPDGSLNPKRSAIKQVASGRFGVSSVYLTNADEIQIKMAQGAKPGEGGELPGHKVIGDIAVTRHSTAGVGLISPPPHHDIYSIEDLAQLIYDLKNSNPGARISVKLVSEAGVGVIASGVVKGHADHVLISGHDGGTGASRWTGIKNAGLPWELGLAETHQTLVANDLRGRTVLQTDGQLKTGRDVVIAALLGAEEFGFSTAPLISLGCIMMRKCHKNTCPVGIATQDPVLREKFAGEPEHVINFFFMLAEEVRELMSEMGFRTVKEMVGRADMLEMDKEVIDSNEKLENIDLSLLLRPAAEIRPEAAQYCVQAQDHGLDMALDQELIRISKAALEKGIPAYMEMPIRNVNRAVGTMLSHEVTKRYNMKGLPTDTIHIKLTGSAGQSLGAFLCSGVTLELEGDSNDYVGKGLSGGKIVVYPPRRSQFDPKENIVIGNVALYGATSGEAYFNGMAAERFCVRNSGAKAVVEGVGDHGCEYMTGGTVVILGKTGRNFAAGMSGGIAYVLNVDGKFDSRCNLELVDLENVEEQDDVMTLKMMIQQHQRHSNSQLAREVLADFEAFLPKFVKVFPRDYKRVLQETKARNTAKEAEEADLMKKDAFEELQRMANVASNQPKKNDGSYKQSEQKVVAQRPTKMDNAVKHRGFLMYERESLQYRDPVARINDWDEVVEENRPGPLLKTQSARCMDCGTPFCHQDNSGCPLGNKIPEWNELVHQGRWREALDRLLETNNFPEFTGRVCPAPCEGACVLGIIENPVSIKSIECAIIDKAFEEGWMVPRPPVKRTGKKVAIVGSGPAGLAAADQLNKAGHSVTVYERADRIGGLMMYGVPNMKTDKVGVVQRRVDLMAEEGVNFVVNANVGVDSSFSVEKLRAENDALLLACGATKPRDLPVPGRELMGIHFAMEFLHSNTKSLLDSGLHDGQFISAKGKRVVVIGGGDTGTDCIATAIRHGCNNVVNLELLPQPPNSRAPGNPWPQWPKVFRVDYGHQEAMTKFGKDPRSYEVLTKRFIADDNGNVKGLELIKVKWEKDESGKLQLKQMEGTEEIIEAELVFLAMGFLGPEQIIAEKLDMERDNRSNFKANFGDFATSVDGVFAAGDCRRGQSLVVWAIAEGRQAAAQIDKFVMKEDQLITTCEDRVEDKGKRPMFTFKT

>CYCAS_023642|Cycas_panzhihuaensis/1-2374

MKVKLELGRRTRVGKGRTRGSYRWRRIRPVGEKGVAGAPGASPWVGFQFRHPPRGGEQRRSRQEQNYREIRETREGRISILVPFPPSPLVFLAIGRKEPAKKTKINVTTSMAALPNGGAISARLGKNEASLSSSSRSLHSASLSSSSFSVSFANLQNESAKSCSQKRNARFSVGRRRFAVGESEFMGRRVQRMHLWSTEGPGRAPKLRVVVRSMALSEIPTGRLGLYDPSLDRDSCGVGFVAELSGKPSRKTVMDAVEMLVRMSHRGACGCETNTGDGAGILVALPHEFLAEAVKDAGFELPPLGEYAVGMFFLPTSEIRREESKNVFNKVAESLGHVVLGWRRVKTNNTGLGKSALQTEPVVEQVFLTPSSRCSAEFEQQMYILRRTSMVAIRAALNLQHGGVRDFYICSLSSRTIVYKGQLKPDQLKSYYIADLGDEKFTSYMALIHSRFSTNTFPSWDRAQPMRVLGHNGEINTLRGNVNWMRAREGLLKCKNLALSKDEMQKLLPIVDASSSDSGAFDGVLELLVRSGRSLPEAVMMMIPEAWQNDKTMDPDRRALYEYFSALMEPWDGPALVSFTDGRYLGATLDRNGLRPGRFYVTHSGRVIMASEVGVVDIPPEDVCKKGRLNPGMMLLVDFEKHVVVDEEALKKQYSVSRPYAEWLTRQKITLKDIVDSVPEPERAPASIIGSIKANHFDDTMENMGIHGLLSPLKAYGYTVEALEMLLLPMAKDGTEALGSMGNDTPLAVMSNRPKIIFEYFKQMFAQVTNPPIDPIREAIVTSMECMIGPEGDLTETSEKQCHRLSLKGPLLSIEEMEAIKKMNYRGWRSKVLDITFPKECGPKGLEETLDRICSEARVAIREGYQILVLSDRATSSKRVPVSSLLAVGAVHHHLVSKLERTRIGLIVESAEPREVHHFCTLIGFGADAICPYLAVEAIWRLQMDGKIPPKKNGVFHSKEELVQKYFKASNSGMLKVIAKMGISTLASYKGAQIFEALGLSSEVVQRCFVGTPSRVEGATFEMLAQDTLRLHDLAFPRRLLPKGSADAQALPNPGDYHWRKDGEVHLNDPLAIAKLQEAARTNSVAAYKQYSKIIQDLNKNCNLRGMLKFKEASGKRIPLEEVEPASEIVKRFCTGAMSYGSISLEAHTTLAIAMNKIGGKSNTGEGGENPSRLEPLPDGSMNPKRSAIKQVASGRFGVSSYYLTNANELQIKMAQGAKPGEGGELPGHKVIGDIAITRNSTAGVGLISPPPHHDIYSIEDLAQLIYDLKNSNPGARISVKLVSEAGVGVIASGVVKGHADHVLISGHDGGTGASRWTGIKNAGLPWELGLAETHQTLVANDLRGRTILQTDGQLKTGRDVVIAALLGAEEFGFSTAPLITLGCIMMRKCHKNTCPVGIATQDPILREKFAGEPEHVINFLFMVAEEARELMSEMGFRTVDEMVGRADMLEMDKEVIDSSEKLKNIDLSLLLQPAAEIRPEAAQHCVQRQDHGLDMALDQELIKLSKAALEKGLPVYMEMPIKNVNRAVGTMLSHEVTKRYKMEGLPTDTIHVKLSGSAGQSLGAFLCAGVTLELEGDSNDYVGKGLSGGKIVVYPPRGSQFDPKENIVIGNVALYGATSGEAYFNGMAAERFCVRNSGVKAVVEGVGDHGCEYMTGGIVVILGKTGRNFAAGMSGGIAYVLDVDGKFHSKCNLELVDLEKVEEEDDIMTLKMMIQQHQRHSDSQLAKAVLANFDALIPKFVKVFPRDYKRVLQEMKANKAAEEAEEAELMKKDAFEELKRLAKVASNEHPTENGASKRPTKIADAVKHRGFLMYERESLSYRDAAVRINDWKEVVEESKPEPLLKTQSARCMDCGTPFCHQEHSGCPLGNKIPEWNELVHQGRWREALDRLLETNNFPEFTGRVCPAPCEGACVLGIIENPVSIKTIECSIIDKAFEEGWMVPRPPVKRTGKKVAIVGSGPAGLAAADQLNKAGHSVTVYERADRIGGLMMYGVPNMKTDKVDVVQRRVNLMEQEGVNFVVNANVGIDPLFSADRLRAENDALLLACGATKPRDLPVPGRELTGVYFAMEFLHSNTKSLLDSALQDGKFISAKGKTVVVIGGGDTGTDCLATSIRHGCKNVVNLELLPQPPKSRAPGNPWPQWPRIFRVDYGHQEAATKFGKDPRSYEVLTKRFIGDDNGNVRGIEVVQVKWEKDASGKFQFKEIEDSEEIIEAQLVLLAMGFLGPEQNIAEKLGLERDNRSNFKADFGRFATNVEGIFAAGDCRRGQSLVVWAIAEGRQAAAQVDKFMMNKDAVSNKSFEDAIEEQDKHPVLTRAVLRPRDLQKKGSCSEAERLEPERRHIDGDGALSQLRLSSPVWLAGVAIRWFSHWIYG

>Gb_24796|Ginkgo_biloba/1-2211

MEALANGGAVSMRLGTKSNDSAFNLMQSGSSSFSSSSSRSLVSLHREIGRPFLRCCKKNGFKSVSGWRLGMEECELMGRRMHLWRSEGPGRAPKLRTVVRSMALSEIPTGRLGLYDPSLDRDSCGVGFVAELSGKPSRKTVMDAVEMLVRMSHRGACGCETNTGDGAGILVALPHDFLAEAVKDAGFDLPPLGEYAVGMFFLPTSEIRREESKNVFNKVAESLGHIVLGWRRVKTDNTGLGKSALQTEPVVEQVFLTTSSRSSADFEQQMYILRRTSMVAIRAALNLQHGGVRDFYICSLSSRTIVYKGQLKPDQLKSYYFADLGHEKFTSYMALIHSRFSTNTFPSWDRAQPMRVLGHNGEINTLRGNVNWMRAREGLLKCKNLALSKDEMQKLLPIVDASSSDSGAFDGVLELLVRTGRSLPEAIMMMIPEAWQNDKNMDPDRRALYEYFSALMEPWDGPALVSFTDGHYLGATLDRNGLRPGRFYVTHSGRVIMASEVGVVDIPPEDVCQKGRLNPGMMLLVDFEKHVVVDDEALKKQYSMSRPYAEWLRRQKITLKDIVDSVPEAERAPAPVIGSVKVNSFDDTMENMGIHGLLSPLKAYGYTVEALEMLLLPMAKDGTEALGSMGNDAPLAVMSNRPKIIFEYFKQMFAQVTNPPIDPIREAIVTSMECMIGPEGDLTETSEKQCHRLSLKGPLLSIEEMEAVKKMNYRGWRSKVLDITFPKDHGPKGVEETLDRICAEARVAIREGYKTLVLSDRATSSKRAPVSSLLAVGAVHQHLVSKLERTQIGLIVESGEPREVHHFCTLVGFGADAICPYLAIEAIWRLQLDGKIPPKKNGSFHSKEELVHKYFKASNSGMLKVIAKMGISTLASYKGAQIFEALGLSSEVVQKCFVGTPSRVEGATFEMLARDSLRLHDLAFPSRLLPKGSADAQALPNPGDYHWRKNGEVHLNDPLAIAKLQEAARTNSVAAYKQYSKIIQNLNKNCNLRGMLKFKEVLDKRIPLEEVEPASEIVKRFCTGAMSYGSISLEAHTTLAIAMNKIGGKSNTGEGGENPSRLEPLSDGSSNPKRSAIKQVASGRFGVSSYYLTNANELQIKMAQGAKPGEGGELPGHKVIGDIAVTRNSTAGVGLISPPPHHDIYSIEDLAQLIYDLKNSNPGARISVKLVSEAGVGVIAAGVVKGHADHVLISGHDGGTGASRWTGIKNAGLPWELGLAETHQTLVANDLRGRTVLQTDGQLKTGRDVVIAALLGAEEFGFSTAPLISLGCIMMRKCHKNTCPVGIATQDPVLREKFAGEPEHVINFLFMVAEEARELMSEMGFRTVEEMVGRADMLEMDKEVINSSEKLENIDLSLLLQPAAEIRPEAAQHCVQKQDHGLDMALDQELIKLSKPAIEKGLPVYMEMPIKNVNRAVGTMLSHEVTKRYKMEGLPTDTIHVKLAGSAGQSLGAFLCAGITLELEGDSNDYVGKGLSGGKIVVYPPRRSQFDPKENIVIGNVALYGATSGEAYFNGMAAERFCVRNSGVKAVVEGVGDHGCEYMTGGTVVILGKTGRNFAAGMSGGIAYVLDVDGEFRSRCNLELVDLENVEEEDDIMTLKMMIQQHQRHSDSELAKQVLADFDSLIPKFVKVFPRDYKRVLQEMKANKVAQEMEEAELMKKDAFEELKRLAKVTSSDQTEKEAIKQSEQTVLAQRPTKIEDAVKHHGFMMYERETIAYRDAAVRINDWKEVVEETKPGPLLKTQSARCMDCGTPFCHQEQSGCPLGNKIPEWNELVYQGQWREALDRLLETNNFPEFTGRVCPAPCEGACVLGIIENPVSIKNIECSIIDKAFEEGWMVPRPPAKRTGKKVAIVGSGPAGLAAADQLNKAGHSVTIYERADRIGGLMMYGVPNMKTDKVDVVQRRVDLMEKEGVDFVVSANVGADPLFSVERLHAENDALLLACGATKPRDLPVPGRELRGIHFAMEFLHANTKSLLDSGLQDGKFISAKGKTVVVIGGGDTGTDCIATSIRHGCNNVVNLELLPQPPKSRAPGNPWPQWPRIFRVDYGHQEATAKFGKDPRSFEVLSKRFLGDENGNVTGLEVVRVKWEKDESGKFQFKEIKGSEEIIEAQLVLLAMGFLGPEQNIAEKLGLERDNRSNFKADFGKFATSVEGIFAAGDCRRGQSLVVWAIAEGREAAAQVDKFMMEKDAQLKTSFEDALEEHDKHPVLT

>Thupl.29379458s0007|Tplicata_v3.1/1-2186

MEAVVLGKGGTILGKGRNEGRGVTVQPRSVGRVKWGGTARVTNPLVSASRKIQYVWRSEGPGRQPRLRHCRNVRSMALSEVPSGRLGLYDPSMDKDSCGVGFVADLSSRPTRKTVEDALEMLVRMAHRGACGCETNTGDGAGILVALPHRFFVQAVKDAGFDLPPAGEYAVGMFFLPTSETRREESKNVFNKVAESLGHVILGWRPVKTNNAELGKSALQTEPVVEQVFLTASSRSSADFEQQMYILRRTSMVAIRAALNLQHGGVRDFYICSLSSRTIVYKGQLKPDQLKNYYFADLGHEKFTSYMALIHSRFSTNTFPSWDRAQPMRILGHNGEINTLHGNVNWMRAREGLLKCQNLALSKDEMQKLLPIVDASSSDSGAFDGVLELLVRAGRSLPEAIMMMIPEAWQNDQNMDPDRRALYEYFSALMEPWDGPALVAFTDGRYLGATLDRNGLRPGRFYVTHSGRVIMASEVGVVDIPYEDVCQKGRLNPGMMLLVDFDKHVIVDDEALKKQFSCLRPYGEWLKRQKITLKEIVDSLPESQRVPEPITGSVKVEGSDDTMENMGIHGLLSPLKAYGYTIEALEMLLLPMAKDGTEALGSMGNDAPLAVMSTRPKIIFEYFKQMFAQVTNPPIDPIREAIVTSMECMIGPEGDLTETSEKQCHRISLKGPLLSIEEMEAVKKMNFRGWRSKVLDITFPKECGTKGLEEALDRICSEARSAIREGYKTLVLSDRATSSKRVPVSSLLAVGAVHHHLVSTLERTRIGLVVESGEPREVHHFCTLVGFGADAICPYLAIESIWRLQVDGKIPPKKNGIFHIKEELVRKYFKASNSGMLKVIAKMGISTLASYKGAQIFEALGLSSEVVQRCFVGTPSRVEGATFEMLARDMLQLHDLAFPSRLLPKGSADAQALPNPGDYHWRKDGEVHLNDPIAIAKLQEAARTNSVAAYKQYSKIIQDLNKHCNLRGMLKFKKSLDKKISLDEVEPASEIVKRFCTGAMSYGSISLEAHTTLAIAMNKIGGKSNTGEGGENPSRLQPLPDGSMNPKRSSIKQVASGRFGVSSYYLTNADELQIKMAQGAKPGEGGELPGHKVIGDIAVTRNSTPGVGLISPPPHHDIYSIEDLAQLIYDLKNSNPRARISVKLVSEAGVGVIASGVVKGHAEHLLISGHDGGTGASRWTGIKNAGLPWELGLAETHQTLVANDLRGRTVLQTDGQLKTGRDVVIAALLGAEEFGFSTAPLISLGCIMMRKCHKNTCPVGVATQDPVLREKFAGEPEHVINFLFMVAEEARELMADMGFRTVDDMVGHAEMLEMDEQVINSSEKLENIDLSLLLQPASEIRPDAAQHCIQKQDHGLDMALDQELIRLSKPALEKRLPVYAEMSIGNVNRAVGTMLSHEVTKRYNMEGLPTDTIHVKLRGSAGQSLGAFLCSGITLELEGDSNDYVGKGLSGGKIVVYPPTISQFDPKENIVIGNVALYGATGGEAYFNGMAAERFCVRNSGAKAVVEGVGDHGCEYMTGGTVVILGKTGRNFAAGMSGGIAYVLDVDGKFSSRCNAESVYLENVEEEEDLLTLKAMIQQHQRHTDSELAKNVLNDFGTLLPKFVKVFPKDYKRVLQEMKANNIIKDMENAELMKKDAFEELKKMSKVTLNAHKNDEKASKQSIVSAVAQRPTKIDDAVKNGGFMKYERESISYRDPVARVNDWKEVVDETRPDSLLKTQSARCMDCGTPFCHQDNSGCPLGNKIPEWNELVYQGRWREALDRLLETNNFPEFTGRVCPAPCEGACVLGIIENPVSIKSIECSIIDKAFDEGWMVPRPPVKRTGKKVAIVGSGPAGLAAADQLNKAGHFVTVYERADRIGGLMMYGVPNMKMDKIDVVQRRVDLMKEEGVNFFVNANVGVDPLFSVDRLRADNDALLLACGATKPRDLPVPGSEFKGIHFAMEFLHANTKSLLDSKLQDGKYISAKDKMVVVIGGGDTGTDCIGTAIRHGSTKIVNLELLPQPPKSRAPGNPWPQWPRIFRVDYGHQEVKAKFGEDPRKYEVLTKRFIGDDSGNVRGLEVVKVKWEKDESGKFQFKEIEGSEEIIKADLVLLALGFLGPEQNIAEKLGMERDNRSNFKADFGLFETNVDGIFAAGDCRRGQSLVVWAIAEGRAAACQIDKFVMKEDVQLPTTYQDAMEGKDKQPIFT

>KAH9322312.1|Taxus_chinensis/1-2189

MAGAAASAPAKVGVFWRSSVSKSEGKRVCCSSARCVKLGLASPLLSQGESSKRRRKMQYVWRTQGPGKQARLRLRVQAMALSEVPRGPLGLYDPSLDRDSCGVGFVADLSATPTRKTVEDALEMLVRMAHRGACGCETNTGDGAGIMVALPHHFFSQAVEDAGFDLPPLGEYAVGMFFLPTSDTRREESKIVFNKVAESLGHIILGWRPVKTNNTGLGKSALQTEPVVEQVFLTASSRSSADFEQQMYILRRTSMVAIRAALNLQHGGVRDFYICSLSSRTIVYKGQLKPDQLKSYYFADLGHDKFTSYMALIHSRFSTNTFPSWDRAQPMRILGHNGEINTLRGNANWMRAREGLLKCQNLALSKDEMQKLLPIVDASSSDSGAFDGVLELLVCTGRSLPEAVMMMIPEAWQNDQNMDPDRRALYEYFSALMEPWDGPALVSFTDGRYLGATLDRNGLRPGRFYVTHSGRVIMASEVGVVDIPAEDVRQKGRLNPGMMLLVDFDKHVVVDDEALKKQFSGARPYAEWLKRQKITLQEIVDSLPESERVPEPIIGSVKVERSDDAIENIGIHGLLSPLKTFGYTVEALEMLLLPMAKDGSEALGSMGNDAPLAVMSTRPKIIFEYFKQMFAQVTNPPIDPIREAIVTSMECMIGPEGDLTETSEKQCHRISLKGPLLSIEEMEAIKKMNFRGWNSKVLDITFSKECGTKGLEETLDRLCLEARLAIREGYKILVLSDRATSSKRVPVSSLLAVGAVHHHLVSTLERTRIGLVVESGEPREVHHFCTIVGFGADAICPYLAIESIWRLQIDGKIPPKKNDTFHSKQELIQKYFKASNSGMLKVIAKMGISTLASYKGAQIFEALGLSSEVVQRCFVGTPSRVEGATFEMLARDMLHLHSVAFPSRLLPEGSADAQALPNPGDYHWRKDGEVHLNDPLAIAKLQEAARTNSVVAYKQYSKIVQDLNRHCNLRGMLKFKKSLDKKISLEEVEPASEIVKRFCTGAMSYGSISLEAHTTLAIAMNKIGGKSNTGEGGENPSRLQPLPDGSMNPKRSAIKQVASGRFGVSSYYLTNADELQIKMAQGAKPGEGGELPGHKVIGDIAVTRNSTAGVGLISPPPHHDIYSIEDLAQLIYDLKNSNPRARISVKLVSEAGVGVIASGVVKGHADHLLISGHDGGTGASRWTGIKNAGLPWELGLAETHQTLVANDLRGRTVLQTDGQLKTGRDVVIAALLGAEEFGFSTAPLISLGCIMMRKCHKNTCPVGIATQDPVLREKFAGEPEHVINFLFMVAEEARELMSEMGFKTMEDMVGHAEMLEMDKEVINSSEKLENIDLSLLLRPAAEIRPEAAQHCIQKQDHELDMALDQELIRLSKPALEKRLPVYTEMPVRNVNRAVGTMLSHEVTKRYNMAGLPVDTIHVKLLGSAGQSLGAFLCAGITLELEGDSNDYVGKGLSGGKIIVYPPRASQFDPKENIVIGNVALYGATGGEAYFSGMAAERFCVRNSGAKAIVEGVGDHGCEYMTGGTVVILGKTGRNFAAGMSGGIAYVLDVDGKFSSRCNVESVYLEKVEEEEDVLTLKMMIQQHQRHTDSQLAKEVLADFGTVLPKFIKVFPQDYKRVLQEMKVNHAAKEAEEADLMKKDAFEELKKMRKVTANGHENNDKVSKQSEPTVVVSRPTNIGDAVKHRGFLMYERETISYRDAMTRVNDWKEVVEETRPEPLLNTQSARCMDCGTPFCHQENSGCPLGNKIPEWNELVYQGRWREALDRLLETNNFPEFTGRVCPAPCEGACVLGIIENPVSIKSIECSIIDKAFEEGWMVPRPPVERTGKKVAIVGSGPAGLAAADQLNKAGHSVTVYERDDRVGGLMMYGVPNMKTDKLDVVQRRVDLMKEEGVNFFVNANVGVDPLFSVDRLCADNDALLLACGATKPRDLPVPGRELGGIHFAMEFLHANTKSLLDSKLQDGKFISAKDKAVVVIGGGDTGTDCIGTAIRHGSNNIVNLELLPKPPKFRAPENPWPQWPRIFRVDYGHQEAKAKFGEDPRTYEVLTKRFIGDDNGNVKGLEVVQVKWEKDESGKFEFKEIEGSEVIIKAELVLLAMGFVGPEQNIAEKLGMQRDNRSNFKAKFGHFETSIEGIFAAGDCRRGQSLVVWAIAEGRAAASQIDKFVMKEEHQLITTREDAMEDKDKHPVFT

>Cucsa.040650|Csativus_v1.0/1-2202

MLAKPGSLLKLPAAPYTFNDNTSVKPQLNVNPKTRLGARAARCSASKGTSGLLNVSEKKFFGARLRAPGSGRVQFWHLDGPGRSPKLRLAVRSGLSSVPEKPLGLYDPSFDKDSCGVGFVAELSGETSRKTITDALEMLVRMSHRGACGCETNTGDGAGILLALPHEFFKQAARDNGFELPPAGQYAVGMFFLPTSDSRREESKKVFAQVAESLGHSVLGWRSVQTDNTGLGKSALLTEPVIEQVFLTPSTKSKVDLEKQMYILRRLSMVAIRAALNLEHGGARDFYICSLSSRTIVYKGQLKPVQLKDYYLDLGNERFTSYMALVHSRFSTNTFPSWDRAQPMRVLGHNGEINTLRGNVNWMKAREGLLKCKELGLSEDELKHLLPIVDASSSDSGAFDGVLELLIRAGRSLPEAVMMMIPEAWQNDKNMDPQRKALYEYFSCLMEPWDGPALISFTDGRYLGATLDRNGLRPGRFYVTHSGRVIMASEVGVVDIAPEDVSRKGRLNPGMMLLVDFENHVVVDDEALKQQYSLARPYGEWLKNQKIELKDVISSIDKSEMTSPTIAGALSAEDNMNNMGIHGLITPLKAFGYTTEALEMLLLPMAKDGVEALGSMGNDTPLAVMSNREKLTFEYFKQMFAQVTNPPIDPIREKIVTSMQCMIGPEGDLTETTEEQCHRLSLKGPLLSIGEMEAIKKMNYRGWRSKVLDITYPKYLGRRGLEETLDRICSEAQNAINEGFTTLVLSDRAFSSKRVSVSSLLAVGAVHQYLVKNLERTQVGLIVESAEPREVHHFCTLVGFGADAICPYLAIEAIWRLQIDGKIPAKSSGEFHTKEELVKKYFKASNYGMMKVLAKMGISTLASYKGAQIFEALGLSSEVVEKCFAGTPSRVEGATFEMLARDAHNLHEMAFPSRAFPPGSAEAVALPNPGDYHWRKGGEIHLNDPVVMAKLQEAARTNSVNAYKEYSKLVHELNKACNLRGLLKFKETGASIPLDEVEPASEIVKRFCTGAMSYGSISLEAHTTLAMAMNKIGGKSNTGEGGEQPSRMEPLPDGSMNPKRSSIKQVASGRFGVSIYYLTNADELQIKMAQGAKPGEGGELPGHKVVGEIAKTRNSTAGVGLISPPPHHDIYSIEDLAQLIHDLKNSNPAARISVKLVSEAGVGVIASGVVKGHADHVLISGHDGGTGASRWTGIKNAGLPWELGLAETHQTLVANDLRGRTVLQTDGQLKTGRDVAIAALLGAEEFGFSTAPLITMGCIMMRKCHKNTCPVGIATQDPVLREKFAGEPEHVINFFFMVAEEMREIMSQLGFRTVNQMVGRSDVLEVDKEVAWQNEKLENIDLSLLLRPAADLRPEAAQYCVQKQDHGLDMALDQKLIALSKSALEKSIPVYIETPIINVNRAVGTMLSHEVTKRYHMAGLPSETIHIKFTGSAGQSLGAFLCPGIMLELEGDSNDYVGKGLSGGKIVVYPPKGSLFDPKENIIIGNVALYGATSGEAYFNGMAAERFCVRNSGAKAVVEGVGDHGCEYMTGGTVVILGKTGRNFAAGMSGGIAYVLDMDGKFESRCNLELVDLDKVEEEDDILTLKMMIQQHQRHTSSNLAKEVLDNFENLLPRFIKVFPREYKRILANIKVQEAVKEASEPSAKDAEELDEAELVEKDAFEELKKMAAASLNGNSEQVEKTEPPKRPTEIPDAVKHRGFIAYEREGVKYRDPNVRMGDWNEVMEESKPGPLLKTQSARCMDCGTPFCHQENSGCPLGNKIPEFNELVYQNRWREALERLLETNNFPEFTGRVCPAPCEGSCVLGIIENPVSIKNIECAIIDKAFEEGWMIPRPPQARSGKQVAIVGSGPAGLAAADQLNKMGHKVTVYERADRIGGLMMYGVPNMKTDKVDVVQRRVNLMAEEGVNFVVNANVGTDPSYSLDQLRKENDALVLAVGATKPRDLPVPGRELAGVHFAMEFLHSNTKSLLDSNLQDGNYISAKDKKVVVIGGGDTGTDCIGTSIRHGCSRIVNLELLPQPPQTRAPGNPWPQWPRIFRVDYGHQEAAAKFGKDPRTYEVLTKRFIGDENGVVKGLEVIRVQWEKDADGRFQFKEVEGSEEIIEADLVLLAMGFLGPESTVAEKLGIEKDNRSNFKAEYGRFSTTVDGVFAAGDCRRGQSLVVWAISEGRQAAAQVDKYLAKEDKGGIKGKVKGGMVNDDVEIG

>Eucgr.A01471|Egrandis_v2.0/1-2218

MSSASSSSLLQPKTGPHALPTSLARPSITPQLSVSRYRDRAKVASRCSVSKQSTGVLQKNFFGPRLRETGSERLRALASDGSSKLRLLVRSALSAVPEKPLGLYDPRFDKDSCGVGFVAELSGESSRKTVTDAVEMLIRMSHRGACGCETNTGDGAGILVALPHEFYQEVVRENGFKLPPPGEYAVGMLFLPTSDTRREQSKKVFQKVAESLGHVVIGWRSVPTNNSGLGKSALETEPVIEQVFLTPSSRSKVDLERQMYILRRVSMVAIRAALNLQHGSARDFYICSLSSRTVVYKGQLKPVQLKDYYYADLGDERFTSYMALIHSRFSTNTFPSWDRAQPMRILGHNGEINTLRGNVNWMKAREGLLKCKELGLSKNELKKLLPIVDASSSDSGAFDGVLELLVRAGRSLPEAVMMMIPEAWQNDKNMDPQRKALYEYFSALMEPWDGPALISFTDGRYLGATLDRNGLRPGRFYVTHSGRVIMASEVGVVDIPPEDVSRKGRLNPGMMLLVDFEKHIVVDDEALKQQYSMARPYGEWLRRQKIELKDIVDSVPESKRKAPAVAGVVAASADDDSMENMGIHGLLAPLKAFGYTVEALEMLLLPMAKDGTEALGSMGNDTPLAVMSDREKLTFEYFKQMFAQVTNPPIDPIREKIVTSMECMIGPEGDLTETTEQQCHRLSLKGPLLSIEEMEAVKKMNYISWQSKIIDITYSKRRGRKGLEETLDRICAEASKAIKEGYTTLVLSDRAFSPERIAVSSLLAVGAVHHHLVRKLERTQVGLIIESAEPREVHHFCTLVGFGADAICPYLAIEAIWRLQVDGKIPSKANGEFFSKHELVKKYFKASNYGMMKVLAKMGISTLASYKGAQIFEALGLSSEVIEKCFAGTPSRVEGATFEMLAQDALDLHESAFPNRVFPPGSAEAVALPNPGSYHWRKGGEIHLNDPLAMAKLQEAARTNSVAAYKEYSRRIQELNKSCNLRGLLTFKEVAVKVPLDEVEPASEIVKRFCTGAMSYGSISLEAHTALAMAMNRIGGKSNTGEGGEQPSRMEPLPDGSVNPKRSAIKQIASGRFGVSSYYLTNADELQIKMAQGAKPGEGGELPGHKVIGDIAVTRNSTAGVGLISPPPHHDIYSIEDLAQLIHDLKNSNPAARISVKLVSEAGVGVIASGVVKGHADHVLISGHDGGTGAARWTGIKNAGLPWELGLAETHQTLVANDLRGRTVLQTDGQLKTGRDVAMAALLGAEEFGFSTAPLITLGCIMMRKCHKNTCPVGIATQDPVLREKFAGEPEHVINFFFMLAEEVREIMSQLGFRTMKEMVGRSDMLEVDREVTKTNEKLENIDLSLLLRPAAEIRPDAAQYCVQKQDHGLDMALDQKLISQSKAALEKSLPIYIEMPICNVNRAVGTMLSHEVTKRYHLAGLPTDTIHIKLSGSAGQSLGAFLCPGITLELEGDSNDYVGKGLSGGKIVVYPPKGSEFDPKENIVIGNVALYGATSGEAYFNGMAAERFCVRNSGARAVVEGVGDHGCEYMTGGTVVVLGKTGRNFAAGMSGGIAYVLDVDGKFQSCCNLELVDLDKVEEEEDIMTLRMMIQQHQRHTNSQLAKEVLVDFESLLPKFIKVFPRDYKRILADMKAGEATKNAQKNMEEEQEQDEGELMEKNAFEELKKMAAASTNAKASATVESVEAPKRPTQVHDAIKHGGFIAYEREGVKYRDPNVRMNDWKEVMEESKPGPLLKTQSARCMDCGTPFCHQENSGCPLGNKIPEFNELVYQNRWQEALDRLLETNNFPEFTGRVCPAPCEGSCVLGIIENPVSIKNIECSIIDKAFEEGWMVPRPPQKRTGKKVAIVGSGPAGLAAADQLNRMGHFVTVYERADRIGGLMMYGVPNMKTDKVDVVQRRVNLMAQEGVNFVVNASVGKDSSYSLDRLQKENDAIVLAVGATKPRDLPVPGRELSGVHFAMEFLHANTKSLLDSNLKDGNYISAKGKKVVVIGGGDTGTDCIGTSIRHGCSRIVNLELLSKPPEKRAPGNPWPQWPRVYRVDYGHQEAAAKFGKDPRSYEVLTKRFIGDENGVVKGLEVVRVGWEKDASGKFQLKEIEGSEEIIEADLVLLAMGFLGPESTIADKLGLERDGRSNFKADYGRFATNVEGVFAAGDCRRGQSLVVWAISEGRQAAAQVDRFLLPKEEDLTVDPENQEDLTSQQQDINKRQEGSGKFTVVT

>Prupe.2G311700|Ppersica_v2.1/1-2207

MLASSGSVVQLRTKPSLASQLNATPIARLGSRAAACSATRKSTKALANKFFGTRLRPAGSEKLHIWRSDGPGRSPKLRVVVRSLLSAVPEKPLGLYDPSFDKDSCGVGFVAELSGEGSRKTITDALEMLVRMAHRGACGCETNTGDGAGILVGLPHDFYKEVAKDVGFKLPPAGEYAVGMFFLPTSDSRREESKNVFTKVAESLGHTVLGWRSVPTDNSDLGKSALQTEPVIEQVFLTPTPRSKLDLERQMYILRRVSMVAIRAALNLEHGGAKDFYICSLSSRTVVYKGQLKPIQLKDYYFADLGNERFTSYMALVHSRFSTNTFPSWDRAQPMRVIGHNGEINTLKGNVNWMKAREGLLKCKELGLSRNELKKLLPIVDASSSDSGAFDGVLEFLVQAGRSLPEAMMMMIPEAWQNDKNMDPHRKALYEYFSSLMEPWDGPALISFTDGRYLGATLDRNGLRPGRFYVTHSGRVIMASEVGVVDIPPEDVSRKGRLNPGMMLLVDFENHIVVDDEALKQQYSLARPYGEWLERQKIELKDIVASVQESDRAPPSIAGVIPASTDDENMENMGIHGLLAPLKAFGYTLESLEMLLLPMAKDGVEALGSMGNDTPLAVMSNREKLTFEYFKQMFAQVTNPPIDPIREKVVTSMECMIGPEGDLTETTEEQCHRLSLKGSLLTIEEMEAIKKMNYRGWRCKVLDITYSKERGREGLEETLDRICAEAREAIKKGYTTLVLSDRAFSPKRVAVSSLLAVGAVHQHLVKNLERTRVGLIIESAEPREVHHFCTLVGFGADAICPYLAIEAIWRLQVDGKIPPKANGVIYSKDELVKKYFKASNYGMMKVLAKMGISTLASYKGAQIFEALGLSSEVIERCFAGTPSRVEGATFEMLAHDELHMHELAFPSRTFPPGSAEAVALPNPGDYHWRKGGEVHLNDPFAISKLQEAARTNSVAAYKEYSKFIHELNKACNLRGLLKFKSTEQKIHLDEVEPASEIVKRFCTGAMSYGSISLEAHTTLAMAMNKIGGKSNTGEGGEQPSRMEPLPDGSRNPKRSAIKQVASGRFGVSSYYLTNADELQIKMAQGAKPGEGGELPGHKVIGDIAVTRNSTAGVGLISPPPHHDIYSIEDLAQLIHDLKNANPTARISVKLVSEVGVGVVASGVVKGHADHVLISGHDGGTGASRWTGIKNAGLPWELGLAETHQTLVANDLRGRTTLQTDGQLKTGRDVAIAALLGAEEFGFSTAPLITLGCIMMRKCHKNTCPVGIATQDPVLREKFAGEPEHVINFFFMVAEELREIMSQLGFRTLNEMVGRSDMLEVDKDVTRNNEKLDNIDLSLLLRPAADLRPDAAQYCVQKQDHGLDMALDHKLISLSKAAIEKSLPVYFETTICNVNRAVGTMLSHEVTKLYNREGLPADTIHIKFNGSAGQSLGAFLCPGIMLELEGDSNDYVGKGLSGGKIVVYPPKKSKFDPKENIVIGNVALYGATSGEAYFNGMAAERFCVRNSGARAVVEGVGDHGCEYMTGGTVVVLGKTGRNFAAGMSGGIAYILDVDGQFRSRCNLELVDLDKLEEEDVMTLKMMIQQHQRHTNSLLASQVLADFGNLLPKFIKVIPREYKRVLANMKDEASKQDAADEAEQDEPELIEKDAFEELKKLAASSSLNGKSNQTVEDSEIFKRPSQVSDAVKHRGFISYEREGVQYRDPNVRMNDWKEVMEETQPGPLLKTQSARCMDCGTPFCHQENSGCPLGNKIPEFNELVYQNRWHEALERLLETNNFPEFTGRVCPAPCEGSCVLGIIENPVSIKSIECAIIDKAFEEGWMVPRPPLKRTGKRVAIVGSGPAGLAAADQLNRLGHTVTVYERADRIGGLMMYGVPNMKADKVDIVQRRVNLMAEEGVNFVVNASVGNDPLYSLDRLREENNAIILAVGATKPRDLPVPGRELSGVHFAMEFLHANTKSLLDSNLQDGNYISAKGKKVVVIGGGDTGTDCIGTSVRHGCSSIINLELLPQPPRTRAPGNPWPQWPRVFRVDYGHQEVAAKFGKDPRSYEVLTKRFVGDENGAVKGLELVSVKWEKDATGKFQFKEIEGSEEIIEVDLVLLAMGFLGPEATVAEKLGLERDNRSNYKAEYGRFSTNVDGVFAAGDCRRGQSLVVWAISEGRQAAAQVDKYLSIEEEDDHTISNGSHENILKRHQDLSKRNTGSSKHTVMT

>FvH4_7g31770|Fvesca_v4.0.a2/1-2226

MLAASSGSVLQLRTKPSVLASPQLNASPIARLSTGRAATSRSASKAIANKFFGTRLRAAAGSERLHLWRSEGPGRSPKLKVVVRSMLSAVPEKPQGLYDPKMDKDSCGVGFVAELSGESSRKTITDALEMLVRMTHRGACGCETNTGDGAGVLVAIPHDFYKEAAKDIGFELPALGEYAVGMLYLPTSESRREESKNVFTKVAESLGHTVLGWRSVPTDNSDLGNSALQTEPVIEQVFLTPTPRSKVDLERQMYILRRVSMVAIRAALNLQYGGAKDFYICSLSSRTVVYKGQLKPEQLKGYYYADLGNERFTSYMALVHSRFSTNTFPSWDRAQPMRVIGHNGEINTLRGNVNWMKAREGLLKCTELGLSKNELKKLLPIVDASSSDSGAFDGVLELLVRAGRSLPEAIMMMIPEAWQNDKNMDPDKRALYEYFSSLMEPWDGPALISFTDGRYLGATLDRNGLRPGRFYVTHSGRVIMASEVGVVDVPPEDVCRKGRLNPGMMLLVDFENHIVVDDEALKKQYSLARPYGEWLKRQKIELKDIVDSVNESDRVPPSIAGVAPASTDDEDMENMGVHGLLAPLKAFGYTVEALEMLLLPMAKDGVEALGSMGNDTPLAVMSNREKLTFEYFKQMFAQVTNPPIDPIREKVVTSMECMIGPEGDLTETTEEQCHRLSLKGPLLNIEEMEAIKKMNYRGWRCKVLDITYSKERGRKGLEETLDRICAEAREAIKKGYTTLVLSDRAFSPKRVAVSSLLAVGAVHQHLVKNLERTRVGLIIESAEPREVHHFCTLVGFGADAICPYLAVEAIWRLQVDGKIPPKSNGTIYSKAELVKKYFKASNYGMQKVLAKMGISTLASYKGAQIFEALGLSSEVIERCFVGTPSRVEGATFEMLARDGLHLHDLAFPSRAFPPGSAEAVALPNPGDYHWRKGGEVHLNDPFAISKLQEAARTNSVAAYKEYSKLIHQLNKACNLRGLLKFKNTEQQIHLDEVEPASEIVKRFCTGAMSYGSISLEAHTTLAIAMNRMGGKSNTGEGGEQPSRMEPLPDGSMNPKRSSIKQVASGRFGVSSYYLTNADELQIKMAQGAKPGEGGELPGHKVIGDIAVTRNSTAGVGLISPPPHHDIYSIEDLAQLIHDLKNANPGARISVKLVSEAGVGVVASGVVKGHADHVLIAGHDGGTGASRWTGIKNAGLPWELGLAETHQTLVANDLRGRTVLQTDGQLKTGRDVAIAALLGAEEFGFSTAPLITLGCIMMRKCHKNTCPVGIATQDPVLREKFAGEPEHVINFFFMVAEEVREIMAQLGFRTLNEMVGRSDMLEVDKEVTKDNEKLNNIDLSLLLRPAADIRPEAAQYCVQKQDHGLDMALDHKLISLSNSAIEKAVPVYFETPVCNVNRAVGTMLSHEVTKRYNRQGLPADTIHIKFNGSAGQSLGAFLCPGITLELEGDSNDYVGKGLSGGKIIVYPPKESKFDPKENIVIGNVALYGATSGEAYFNGMAAERFCVRNSGARAVVEGVGDHGCEYMTGGTVVVLGKTGRNFAAGMSGGIAYVFDVDGKFLSRCNPELVDLDKVEEEEDILTLRMMIQQHQRHTKSLLASEVLADFENLLPKFIKVIPREYKRALANLREEASKQAVEDADEEAEKQEEEELKLKEKDAFEELKKMASASLNELKNMASASLNGNSNQVKVQVEDAETLKRPKEVSKAVKHRGFISYEREGVQYRDPNVRMNDWDEVMEETKPGPLVNTQSARCMDCGTPFCHQENTGCPLGNKIPEFNELVYQNRWHDALERLLETNNFPEFTGRVCPAPCEGSCVLGIIENPVSIKSIECAIIDKAFEEGWMVPRPPVKRTGKKVAIVGSGPAGLAAADQLNRIGHTVTVYERADRIGGLMMYGVPNMKADKVDVVQRRVNLMAEEGVNFVVNANVGNDSSYSFDRLREENNAIILAVGATKPRDLPVPGRELSGVHFAMEFLHANTKSLLDSNLENGNYISAKGKKVVVIGGGDTGTDCIGTSVRHGCTDIVNLELLPQPPQTRAPGNPWPQWPRIFRVDYGHAEVAAKFGKDPRTYEVLTKRFVGDENGVVKGIEVVRVKWEKDATGKFQFKEIEGSEEIIEADLVLLAMGFLGPEAAIAEKLGLECDNRSNFKADYGRFSTNVDGVFAAGDCRRGQSLVVWAISEGRQAAAQVDNYLCKEEEDHTDSNSSPESDLLKRHQEITKSQKTVASTS

>VIT_216s0098g00290|Vvinifera_v2.1/1-2216

MSATSGSGSVVQIRNNSGSGLVPSRKRPTFAHQFNVTPLSRFNNKSKGSSCYVSKRTNVVENKFLGTRLRGCGSERLHFWQSDGPGRSPKLRVVVRSALSQVPEKPLGLYDPSFDKDSCGVGFVAELSGESSRKTVTDAVEMLVRMSHRGACGCETNTGDGAGILVGLPHDFFKEVAQDVGFELPPPGEYAVGMFFLPTSHTRREESKNVFTKVAESLGHTVLGWRSVPTNNSGLGNSALQTEPVVEQVFLTPTPRSKADFEQQMYILRRVSMVAIRAALNLQHGGVRDFYICSLSSRTVVYKGQLKPDQVKGYYYADLGNERFTSYMALIHSRFSTNTFPSWDRAQPMRVLGHNGEINTLRGNVNWMKAREGLLKCKELGLSKNEMKKLLPIVDASSSDSGAFDGVLELLVRAGRSLPEAVMMMIPEAWQNDKNMDPDRKALYEYFSALMEPWDGPALISFTDGRYLGATLDRNGLRPGRFYVTHSGRVIMASEVGVVDIAPEDVRRKGRLNPGMMLLVDFENHVVVDDEALKQQYSLARPYGEWLKRQKIELKDIVESVHESDKVSPTIAGVMPASNQDDSMENMGIYGLLAPLKTFGYTVEALEMLLLPMAKDGTEALGSMGNDAPLAVMSNREKLTFEYFKQMFAQVTNPPIDPIREKIVTSMECMIGPEGDLTETTEEQCHRLSLKGPLLSIQEMEAIKKMNYRGWRSKVLDITYSKNRGRKGLEETLDRLCSEAHHAIKQGYTVLVLSDRAFSSKRVAVSSLLAVGAVHQHLVQKLERTQVGLIVESAEPREVHHFCTLVGFGADAICPYLAIEAILRLQVDGKIPPKASGEFHSKDELVKKYFKASNYGMMKVLAKMGISTLASYKGAQIFEAVGLSSEVIQRCFTGTPSRVEGATFEMLAQDALELHEMAFPTRVFPPGSAEAVALPNPGDYHWRKGGEVHLNDPLAIAKLQDAARSNSVAAYKEYSKRIQELNKTCNLRGLLKFKEAEVKVPLDEVEPASEIVKRFCTGAMSYGSISLEAHTTLAIAMNRIGGKSNTGEGGENPSRLESLPDGSLNPKRSAIKQVASGRFGVSSYYLTNADELQIKMAQGAKPGEGGELPGHKVIGDIAVTRNSTAGVGLISPPPHHDIYSIEDLAQLIHDLKNANPSARVSVKLVSEAGVGVIASGVVKGHADHVLISGHDGGTGASRWTGIKNAGLPWELGLAETHQTLVANDLRGRTVLQTDGQLKTGRDVAIAALLGAEEFGFSTAPLITLGCIMMRKCHKNTCPVGIATQDPVLREKFAGEPEHVINFFFMLAEEVREIMSQLGFRTLSEMVGRADMLEVDKEVTKNNEKVQNIDLSLLLRPAADIRPEAAQYCVQKQDHGLDMALDQKLIALSKAALEKSLPVYIETPIRNVNRAVGTMLSHEVTKRYHSAGLPAETIHIKLSGSAGQSLGAFLCPGIMLELEGDSNDYVGKGLSGGKIVVYPPRQSKFDPKENIVIGNVALYGATSGEAYFNGMAAERFCVRNSGARAVVEGVGDHGCEYMTGGTVVVLGKTGRNFAAGMSGGIAYVFDVDEKFSSRCNPELVDLDKVEKEEDIMTLRMMIQQHQRHTNSQLAKEILADFDNLLPKFIKVFPRDYKRVIESMKQEEASKKALEQDTQEAEDQDEKELMEKDAFEELKKLAAASLNGKNSQKVEEAEPDKRPTRVANAVKHRGFIAYKREGISYRDPNSRMNDWKEVMVETKPGPLLKTQSARCMDCGTPFCHQENSGCPLGNKIPEFNELVHQNRWREALDRLLETNNFPEFTGRVCPAPCEGSCVLGIIENPVSIKSIECSIIDKAFEEGWMVPRPPPKRTGKRVAIVGSGPAGLAAADQLNRMGHFVTVFERADRIGGLMMYGVPNMKADKVDVVQRRVNLMAEEGVNFVVNASVGTDPSYSLDRLREENDAIVLAVGATKPRDLPVPGRELSGIHFAMKFLHANTKSLLDSNLEDGNYISAKGKKVVVIGGGDTGTDCIGTSIRHGCSSVVNLELLPQPPQTRAPGNPWPQWPRIFRVDYGHQEAAAKFGKDPRSYEVLTKRFIGDENGVLKGLEVIRVQWEKDASGKFQFKEVEGSQEVIEADLVLLAMGFLGPEVTVAEKLGLERDNRSNLKADYGRFATSVEGVFAAGDCRRGQSLVVWAISEGRQAASQVDKFLMREDEHLTNNWQDDNIKRQQKSIKHTVMT

>Hyque.14G104500|Hquercifolia_v1.1/1-2210

MSVASSSVLQTRNNSVVLPSISKPFVNNQLNVVPLSRLGLTRAARGSVAKRRSNVVENRFYGTRLSTGGAERLHQWQSDGPGRDPKLRVVVRSALSQVPEKPLGLYDPSFDKDSCGVGFVAELSGESSRKTITDAVEMLVRMSHRGACGCETNTGDGAGILVALPHEFFKEVAKDAGFELPPAGEYAMGMFFLPTSENRREQSKRVFTKVAESLGHTVLGWRHVPTDNSGLGQSALQTEPVIEQVFLTPTPRSKADFEQQMYILRRVSMVAIRAALNLQHGGVRDFYICSLSSRTVVYKGQLKPNQLNEYYYADLGNERFTSYMALVHSRFSTNTFPSWDRAQPMRVLGHNGEINTLRGNVNWMKAREGLLKCKKLGLSKDEMKKLLPIVDASSSDSGAFDGVLELLVRAGRSLPEAIMMMIPEAWQNDKNMEPQRKALYEYFSALMEPWDGPALISFTDGRYLGATLDRNGLRPGRFYVTHSGRVIMASEVGVVDIPPEDVYRKGRLNPGMMLLVDFEKHVVVDDDALKQQYSLARPYGEWLKRQKIELKDIVQSVRESDRVPPAIAGVVPASSEDDNMENMGIRGLLSPLKAFGYNVESLEMLLLPMAKDGIEALGSMGNDAPLAVMSNREKLTFEYFKQMFAQVTNPPIDPIREKIVTSMECMIGPEGDLTETTEEQCHRLSLKGPLLHTEEMEAIKKMNYRGWRSKVLDITYSKERGRKGLEETLDRICFEARDAIKQGFTTLVLSDRAFSSKRVAVSSLLAVGAVHHHLVTKLERTRVGLIVESAEPREVHHFCTLVGFGADAICPYLAVETIWRLQVDGKIPPKASGEFHSKNELVKKYFKASNYGMMKVLAKMGISTLASYKGAQIFEAVGLSSEVMERCFKGTPSRVEGATFEVLAQDALQLHELAFPTRVFPPGTAEAVALPNPGDYHWRKGGEVHLNDPLAIAKLQEAAKSNSVAAYKEYSKRIQELNKNCNLRGLLKFKEADVKIPLDEVESASEIVKRFCTGAMSYGSISLEAHSTLAIAMNAIGGKSNTGEGGENPSRMEPLPNGSRNPKRSAIKQVASGRFGVSSYYLTNADELQIKMAQGAKPGEGGELPGHKVIGDIAVTRNSTAGVGLISPPPHHDIYSIEDLAQLIHDLKNANPTARISVKLVSEAGVGVIASGVVKGHADHVLISGHDGGTGASRWTGIKSAGLPWELGLAETHQTLVANDLRGRTVLQTDGQLKTGRDVAIAALLGAEEFGFSTAPLITLGCIMMRKCHKNTCPVGIATQDPVLREKFAGEPEHVINFFFMLAEELREIMSQLGFRTITEMVGRSDMLELDKEVTKNNDKLKNIDLSLLLRPAADIRPDAAQYCVQKQDHGLDMALDQRLITLSEAALEKALPVYIETPIYNINRAVGTMLSHEVTKRYHMAGLPADTIHIKLGGSAGQSLGAFLCPGITLELEGDSNDYVGKGLSGGKIVVYPPKGSQFDPKENIVIGNVALYGATNGEAYFNGMAAERFSVRNSGAKAVVEGVGDHGCEYMTGGTVVVLGKTGRNFAAGMSGGIAYVLDVDGKFKSRCNLELVDLDRIEEEGDIMNLRMMIQQHQRHTNSQLAREVLADFENLLPNFIKVFPRDYKRVLASMKAVETTKEAEKKAAKEAEEQEEAELMERDAFEDLKKLATMSLNGKSNQKVEEAEPSKRPTRDADAVKHRGFISYDREGVSYRDPDVRMNDWKEVMEESKPGPLLKTQSARCMDCGTPFCHQENSGCPLGNKIPEFNELVYQNRWREALDRLLETNNFPEFTGRVCPAPCEGSCVLGIIENPVSIKSIECSIIDKAFEEGWMVPRPPLKRTGKKVAIIGSGPAGLAAADQLNRMGHLVTVFERADRIGGLMMYGVPNMKTDKIDVVQRRVDLMEKEGVKFVVNANVGKDPSFSVDRLKEDNDAIVLAVGATKPRDLPVPGRELSGVHFAMEFLHANTKSLLDSNLEDGNYISAKGKKVVVIGGGDTGTDCIGTSIRHGCTNIVNLELLPEPPKTRAPGNPWPQWPRIFRVDYGHQEAAAKFGKDPRSYEVLTKRFVGDENGVVKGLEVVRVKWEKDASGKFQFKEVEGSEEVIEADLVLLAMGFLGPESNVADKLGLEKDNRSNFKAEYGRFSTNVEGVFAAGDCRRGQSLVVWAISEGRQAASQVDKYLTTEDKDLRQEEDISKRHQDSSKHTVMT

>Gohir.D12G251500|Ghirsutum_v3.1/1-2209

MSSAPTSSSLVQLRKSSCSLPSINKSYLNPQLNVTLSNRRKTSNARCSVTKKSSAALEKKFLGTRLRGSEKLHFWQSEGPGRVPKLRVMVRSALSGVPEKPLGLYDPSFDKDSCGVGFVAELSGDSSRKTVTDALEMLIRMSHRGACGCETNTGDGAGILVALPHGFYKEVAKDVGFELPPPGEYAVGMFFLPTSESRREESKNVFTKVAESLGHRVLGWRSVPTDNSGLGNAALQTEPVIEQVFLTPTPRSKADLEQQMYILRRVSMVAIRAALNLQHGGVRDFYICSLSSRTVVYKGQLKPDQLQNYYYADLGNERFTSYMALIHSRFSTNTFPSWDRAQPMRVLGHNGEINTLRGNINWMKAREGLLKCKELGLSKNEMKKLLPIVDASSSDSGAFDGVLELLVRAGRSLPEAVMMMIPEAWQNDKNMDPQRKALYEYFSALMEPWDGPALISFTDGRYLGATLDRNGLRPGRFYVTHSGRVIMASEVGVVDIPPADVLRKGRLNPGMMLLVDFENHIVVDDEALKQQYSLARPYGEWLQRQKIELNDIVDSVQESERLPPSIAGSMPASNDDDNMDNLGIHGLLAPLKAFGYTVEALEMLLLPMAKDGTEALGSMGNDAPLAVMSNREKLTFEYFKQMFAQVTNPPIDPIREKIVTSMECMIGPEGDLTETTEEQCHRLSLKGPLLSIEETEAIKKMNFKGWRSKVLDITYSKDCGRKGLEETLDRICAEARDAIKEGYTLLVLSDRAFSSKRVAVSSLLAVGAVHHHLVKNLERTRVGLIVESAEPREVHHFCTLVGFGADAICPYLAIETIWRLQVDGKIPPKSSGEFHSKEELVKKYFKASNYGMMKVLAKMGISTLASYKGAQIFEALGLSSEVIEKCFAGTPSRVEGATFEMLARDALHLHELAFPSRAFAPGSAEAVALPNPGDYHWRKGGEVHLNDPLAIAKLQEAARSNSVAAYKEYAKRIHELNKTCNLRGMLKFKESEAKIPLDEVEPASEIVKRFCTGAMSYGSISLEAHATLAIAMNTLGGKSNTGEGGEQPSRMVPLPDGSRNPKRSAIKQVASGRFGVSSYYLTNADELQIKMAQGAKPGEGGELPGHKVIGDIAVTRNSTAGVGLISPPPHHDIYSIEDLAQLIHDLKNSNPSARISVKLVSEAGVGVIASGVVKGHADHVLISGHDGGTGASRWTGIKNAGLPWELGLAETHQTLVANDLRGRTVLQTDGQLKTGRDVAIAALLGAEEFGFSTAPLITLGCIMMRKCHKNTCPVGIATQDPVLREKFAGEPEHVINFFFMLAEEVREIMSQLGFRTVTEMVGRSDMLEVDKEVLSNNEKLQNIDLSLLLRPAADIRPEAAQYCIQKQDHGLDMALDQKLIKLSTAALEKGLPVYIETPICNVNRAVGTMLSHEVTKRYHLAGLPAGTIHIKLSGSAGQSLGAFLCPGIMLELEGDSNDYVGKGLSGGKIVVYPPKGSRFDPKENIVIGNVALYGATSGEAYFNGMAAERFCVRNSGAKAVVEGVGDHGCEYMTGGTVVVLGKTGRNFAAGMSGGIAYVLDVDGKFQSRCNPELVDLDKIEEEEDIVTLKMMIQQHQRHTNSQLAREVLANFESLLPKFIKVFPRDYKRVLAKMKDQEASERAAKEAEEQDEVELMEKDAFEELKKLAAASSNEKSSLTVEAEPVKRPTQVSDAVKHRGFVAYEREGVQYRDPNVRMNDWKEVMEESKPGPLLKTQSARCMDCGTPFCHQENSGCPLGNKIPEFNELVYQNRWREALDRLLETNNFPEFTGRVCPAPCEGSCVLGIIENPVSIKSIECAIIDKGFEEGWMVPRPPLKRTGKSIAIIGSGPSGLAAADQLNRMGHSVTVYERADRIGGLMMYGVPNMKTDKVDVVQRRVNLMVEEGVKFVVNANIGKDPSYSLDRLREENDAIVLAIGATKPRDLPVPGRDLSGVHFAMEFLHANTKSLLDSDLQDGNYISAKGKKVVVIGGGDTGTDCIGTSIRHGCSSIVNLELLPQPPQTRAPGNPWPQWPRIFRVDYGHQEAATKFGKDPRSYEVLTKRFIGDDNGTVKGLEVVRVRWEKDASGRFQFKEVEGSEEIIEADLVLLAMGFLGPESTLAEKLGVEQDNRSNLKAEYGRFTTNVDGVFAAGDCRRGQSLVVWAISEGRQAAAQVDKYLTKEDKDTSVEGENQDSVKRHQDLPQKQQTVMK

>Gohir.A12G250700|Ghirsutum_v3.1/1-2209

MSSAPTSSSLVQLWKSSCSLPSINKSYLNPQLNVTLSNRRKTSNARCFVTKKSSVALEKKFLGIRLRGSEKLHFWQSEGPGRVPKLRVMVRSALSGVPEKPLGLYDPSFDKDSCGVGFVAELSGDSSRKTVTDALEMLIRMSHRGACGCETNTGDGAGILVALPHGFYKEVAKDVGFELPPPGEYAVGMFFLPSSESRREESKNVFTKVAESLGHRVLGWRSVPTDNSGLGNAALQTEPVIEQVFLTPTPRSKADLEQQMYILRRVSMVAIRAALNLQHGGVRDFYICSLSSRTVVYKGQLKPDQLQNYYYADLGNERFTSYMALIHSRFSTNTFPSWDRAQPMRVLGHNGEINTLRGNINWMKAREGLLKCKELGLSKNEMKKLLPIVDASSSDSGAFDGVLELLVRAGRSLPEAVMMMIPEAWQNDKNMDPQRKALYEYFSALMEPWDGPALISFTDGRYLGATLDRNGLRPGRFYVTHSGRVIMASEVGVVDISPEDVLRKGRLNPGMMLLVDFENHIVVDDEALKQQYSLARPYGEWLQRQKIELNDIVDSVQESERLPPSIAGSMPASNDDDNMDNLGIHGLLAPLKAFGYTVEALEMLLLPMAKDGTEALGSMGNDAPLAVMSNREKLTFEYFKQMFAQVTNPPIDPIREKIVTSMECMIGPEGDLTETTEEQCHRLSLKGPLLSIEETEAIKKMKFKGWRSKVLDITYSKDCGRKGLEETLDRICAEARDAIKEGYTLLVLSDRAFSSKRVAVSSLLAVGAVHHHLVKNLERTRVGLIVESAEPREVHHFCTLVGFGADAICPYLAIETIWRLQVDGKIPPKSSGEFHSKEELVKKYFKASNYGMMKVLAKMGISTLASYKGAQIFEALGLSSEVIEKCFARTPSRVEGATFEMLARDALHLHELAFPSRAFAPGSAEAVALPNPGDYHWRKGGEVHLNDPLAIAKLQEAARSNSVAAYKEYAKRIHELNKTCNLRGMLKFKESEAKIPLDEVEPASEIVKRFCTGAMSYGSISLEAHTTLAIAMNTLGGKSNTGEGGEQPSRMVPLLDGSRNPKRSAIKQVASGRFGVSSYYLTNADELQIKMAQGAKPGEGGELPGHKVIGDIAVTRNSTAGVGLISPPPHHDIYSIEDLAQLIHDLKNSNPSARISVKLVSEAGVGVIASGVVKGHADHVLISGHDGGTGASRWTGIKNAGLPWELGLAETHQTLVANDLRGRTVLQTDGQLKTGRDVAIAALLGAEEFGFSTAPLITLGCIMMRKCHKNTCPVGIATQDPVLREKFAGEPEHVINFFFMLAEEVREIMSQLGFRTVTEMVGRSDMLEVDKEVLSNNEKLQNIDLSLLLRPAADIRPEAAQYCIQKQDHGLDMALDQRLIKLSTAALEKGLPVYVETPICNVNRAVGTMLSHEVTKRYHLAGLPAGTIHIKLSGSAGQSLGAFLCPGIMLELEGDSNDYVGKGLSGGKIVVYPPKGSRFDPKENIVIGNVALYGATSGEAYFNGMAAERFCVRNSGARAVVEGVGDHGCEYMTGGTVVVLGKTGRNFAAGMSGGIAYVLDVDGKFQSRCNPELVDLDKIEEEEDIVTLKMMIQQHQRHTNSQLASEVLADFESLLPKFIKVFPRDYKRVLAKMKDQKASERAAKEAEEQDEVELMEKDAFEELKKLAAASSNEKSSLTVEAEPVKRPTQVSDAVKHRGFVAYEREGVQYRDPNVRMHDWKEVMEESKPGPLLKTQSARCMDCGTPFCHQENSGCPLGNKIPEFNELVYQNRWREALDRLLETNNFPEFTGRVCPAPCEGSCVLGIIENPVSIKSIECAIIDKGFEEGWMVPRPPLKRTGKSIAVIGSGPSGLAAADQLNRMGHSVTVYERADRIGGLMMYGVPNMKTDKVDVVQRRVNLMAEEGVKFVVNANIGKDPSYSLDRLREENDAIVLAIGATKPRDLPVPGRDLSGVHFAMEFLHANTKSLLDSDLQDGNYISAKGKKVVVIGGGDTGTDCIGTSIRHGCSSIVNLELLPQPPQTRAPGNPWPQWPRIFRVDYGHQEAATKFGKDPRSYEVLTKRFIGDDNGNVKGLEVVRVRWEKDASGRFQFKEVEGSEEIIEADLVLLAMGFLGPESTLAEKLGVEQDNRSNLKAEYGRFTTNVDGVFAAGDCRRGQSLVVWAISEGRQAAAQVDKYLTKEDKDTSVEGENQDSVKRHQDLPQKQQTVMK

>Potri.015G017500|Ptrichocarpa_v4.1/1-2228

MSAAASSGALLQPKSVKAPFSSLSKSSSLSPSLNVATAASVSRRSARANRCASTRKSVVVERKSFLGSKVRGSAGSERLHFWQSDGPGREPKLRVVVRSALSGVPEKPLGLYDPSFDKDSCGVGFVAELSGDSSRKTVNDALEMLVRMTHRGACGCETNTGDGAGILVALPHDFYKEVAKDIGFELPPPGEYAVGMFFLPTSDNRKEESKNVFTKVAESLGHTVLGWRPVPTDNSGLGNSALQTEPVIEQVFLTATPRSKADFEQQMYILRRVSMVAIRAALNLQYGGVRDFYICSLSSRTVVYKGQLKPEQLKGYYYADLGNERFTSYMALVHSRFSTNTFPSWDRAQPMRVLGHNGEINTLRGNVNWMKAREGLIKCKELGLSKNEMKKLLPIVDASSSDSGAFDGVLELLIRAGRSLPEAVMMMIPEAWQNDKNMDPQRRALYEYFSALMEPWDGPALISFTDGHYLGATLDRNGLRPGRFYVTRSGRVIMASEVGVVDIPPEDVLRKGRLNPGMMLLVDFEKHIIVDDEALKQQYSLARPYGEWLKRQKIELSDIVDSVQESERVAPAISGVVPASDDDTSMQNMGTHGLLAPLKAFGYTVEALEMLMLPMAKDATEALGSMGNDAPLAVMSNREKLTFEYFKQMFAQVTNPPIDPIREKIVTSMECMIGPEGDLTETTEEQCHRLSLKGPLLSIEQMEAMKKMNFSGWRSKVLDITYSKERGRKGLEETLDRICAEAHEAIKEGYTVLVLSDRAFSSKRVAASSLLAVGAVHQYLVKKLERTQVGLIVESAEPREVHHFCTLVGFGADAICPYLAVEAIWRLQVDGKIPPKSTGEFHTKDELVKKYFKASNYGMMKVLAKMGISTLASYKGAQIFEGLGLSSEVIDKCFAGTPSRVEGATFEMLARDSLHLHELAFPSRVLPPGSAEAVALPNPGDYHWRKGGEIHLNDPLAIAKLQEAARGNSVAAYKEYSKRVQELNKACNLRGLLKFKEADVKVSLDEVEPASEIVKRFCTGAMSYGSISLEAHTTLAQAMNKIGGKSNTGEGGEQPSRMETLPDGSMNPKRSAIKQVASGRFGVSSYYLTNADELQIKMAQGAKPGEGGELPGHKVIGDIAITRNSTAGVGLISPPPHHDIYSIEDLAQLIHDLKNANPAARISVKLVSEAGVGVIASGVVKGHADHVLISGHDGGTGASRWTGIKNAGLPWELGLAETHQTLVANDLRGRTVLQTDGQLKTGRDVAIAALLGAEEFGFSTAPLITLGCIMMRKCHKNTCPVGIATQDPVLREKFAGEPEHVINFFFMLAEELREIMAQLGFRTMTEMVGRSDMLEVDKEVVKSNEKLENIDLSLLLRPAADIRPEAAQYCVQKQDHGLDMALDNKLIKLSEAALEKGLPVYIETPICNVNRAVGTMLSHEVTKRYHLAGLPADTIHIKLTGSAGQSLGAFLCPGIMLELEGDGNDYVGKGLSGGKIVVYPPKGSLFDPKENIVIGNVALYGATCGEAYFNGMAAERFCVRNSGARAVVEGVGDHGCEYMTGGTVVVLGKTGRNFAAGMSGGVAYVLDLDGKFRSRCNPELVDLDKVEEEEDITTLKMMIQQHQRHTNSLLAREVLADFDNLLPKFIKVFPRDYKRVLANMKEESATKEAADLAAKEVEEAEEQDEAELKEKDAFEELKKLAAASLNGNSIQVEDGPLKRPTRVNDAVKHRGFIAYEREGVQYRDPNIRMNDWKEVTEESKPGPLLKTQSARCMDCGTPFCHQENSGCPLGNKIPEFNELVHQNRWREALDRLLETNNFPEFTGRVCPAPCEGSCVLGIIDDPVSIKNIECSIIDKAFEEGWMVPRPPLKRTGRRVAIVGSGPSGLAAADQLNKMGHLVTVYERADRIGGLMMYGVPNMKTDKVDIVQRRVNLMSEEGINFVVNANVGIDPLYSLDRLRDENNAIVLAVGATKPRDLPVPGRELSGVHFAMQFLHANTKSLLDSNLQDGNYISAKGKKVVVIGGGDTGTDCIGTSIRHGCSSIVNLELLPEPPRTRGPGNPWPQWPRVFRVDYGHQEAAAKFGKDPRSYEVLTKRFIGDENGNVKGLELVRVHWEKDATGKFQFKEVEGSEEVIEADLVLLAMGFLGPELNVAEKLGLEQDNRSNFKAEYGRFSTNVEGIFAAGDCRRGQSLVVWAISEGRQAASQVDKYLMKEEDATINTDNTQDDLVKRHQDLTKRHQDSSKHTVMT

>Potri.012G011700|Ptrichocarpa_v4.1/1-2228

MSAAASSSSAILQPKSVKSSISSLNKSSYCVFSPSLNVATAASISRRRGRATRCVSARNSAVVERKSFLGSKVRGSPSERLHFWLSEGPGREPKLRVVVRSALSGVPEKPLGLYDPSFDKDSCGVGFVAELSGENSRKTVNDALEMSVRMAHRGACGCETNTGDGAGILVALPHDYYKEVAKDIGFELPPSGEYAVGMFFLPTSDNRREESKNVFTKVAESLGHTVLGWRPVPTDNSALGNAALQTEPVIEQVFLTATPRSKADFEKQMYILRRVSMVAITAALNLQYGGVKDFYICSLSSRTVVYKGQLKPDQLKAYYYADLGNETFTSYMAMVHSRFSTNTFPSWDRAQPMRVLGHNGEINTLRGNVNWMKAREGLLKCKELGLSKNEMKKILPIVDASSSDSGAFDGVLELLIRSGRTLPEAVMMMIPEAWQNDKNMDPQRRALYEYSSALMEPWDGPALISFTDGHYLGATLDRNGLRPGRFYVTRSGRVIMASEVGVVDIPPEDVLRKGRLNPGMMLLVDFEKHTVVDDEALKQQYSLARPYGEWLKRQKIELSDIVNSVQESDKVAPAISGVVAASDDDDSMVHMGIHGLLAPLKSFGYTVEALEMLMLPMAKDGTEPLGSMGNDAPLAVMSNREKLTFEYFKQMFAQVTNPPIDPIREKIVTSMECMIGPEGDLTETTEEQCRRLSLKGPLLSIGEMEAIKKMNYNGWRSKVLDITYSIKRGRKGLEETLDRICTEAHEAIKEGYTVLVLSDRAFSSKRVAVSSLLAVGAVHQYLVKKLERTQVGLIVESAEPREVHHFCTLVGFGADAICPYLAIDAIWRLQVDGKIPPKSTGELHSKDELVKKYFKASNYGMMKVLAKMGISTLASYKGAQIFEGLGLSSEVIDKCFAGTPSRVEGATFEMLAHDSLRLHELAFPSRALPPGSAEAVALPNPGDYHWRKGGEIHLNDPLAIAKLQEAARGNSVAAYKEYSKRIQELNKACNLRGLLKFKVADVKVSLDEVEPASEIVKRFCTGAMSYGSISLEAHTTLAQAMNKIGGKSNTGEGGEQPSRMEPLPDGSMNPKRSAIKQVASGRFGVSSYYLTNADELQIKMAQGAKPGEGGELPGHKVIGDIAVTRNSTAGVGLISPPPHHDIYSIEDLAQLIHDLKNANPSARISVKLVSEAGVGVIASGVVKGHADHVLISGHDGGTGASRWTGIKSAGLPWELGLAETHQTLVANDLRGRTVLQTDGQLKTGRDVAIAALLGAEEFGFSTAPLITLGCIMMRKCHKNTCPVGIATQDPVLRDKFAGEPEHVINFFFMLAEELREIMAQLGFRTMNEMVGRSDMLEVDKEVVKSNEKLENIDLSSLLRPAADIRPGAAQYCVQKQDHGLDMALDQKLIKLSEAALEKSLPVYIETPIRNVNRAVGTMLSHEVTKRYHLAGLPADTIHIKLKGSAGQSLGAFLCPGIMLELEGDGNDYVGKGLSGGKIVVYPPKGSLFDPKENIIIGNVALYGATGGEAYLNGMAAERFCVRNSGARAVVEGIGDHGCEYMTGGTIVVLGKTGRNFAAGMSGGVAYVLDLDGKFKSRCNLELVDLDKVEEEEDIMTLKMMIQQHQRHTNSLLAREVLADFDNLLPKFIKVFPRDYKRVLANMKEESASKEAAELAAKEAEEKNEAELREKDAFEELKKMAAASLNGKSNQVVEDEPLKRPTRVNNAVKHRGFIAYEREGVQYRDPNVRMNDWKEVMESSKPGPLLNTQSARCMDCGTPFCHQENSGCPLGNKIPEFNELVHQNRWREALDRLLETNNFPEFTGRVCPAPCEGSCVLGIIDDPVSIKNIECSIIDKAFEEGWMVPRPPLKRTGKRVAIVGSGPSGLAAADQLNKRGHLVTVYERADRIGGLMMYGVPNMKTDKVDIVQRRVNLMAKEGINFVVNANVGIDPLYSLDQLRQENDAIVLAVGATKPRDLPVPGREMSGVHFAMEFLHKNTKSLLDSNLQDGNYISAKGKKVVVIGGGDTGTDCMGTSIRHGCSGVVNLELLPEPPQTRAPGNPWPQWPKVFRVDYGHQEAASKFGKDPRSYEVLTKRFIGDEDGSVKGLEVVRVHWEKDASGKFQYKEVEGSEEIIEADLVLLAMGFLGPEPNVAKKLGLEQDNRSNFKAEYGRFSTNVEGIFAAGDCRRGQSLVVWAISEGRQAASQVDKYLMKEEDVTISTDNTQDELVKKHEDLTKRHQDSSKHTVMT

>Glyma.06G127400|Gmax_Wm82.a6.v1/1-2185

MSCSLSFSRLYARSERTLPSENEKPRLNVRCPVRVTKFLGTRLRSSGKSLGSERFHVWQSEGPGRSPKLRVAVRSAMSAVPNKPLGLYDPAMDKDSCGVGFVAELSGESSRKTVTDALEMLVRMTHRGACGCEANTGDGAGIMVALPHQFYKEVVDFELPPPGKYAVGMLFLPTSNSRREESKNVFQKVAESLGHSVIGWRSVPTDNTGLGKSAVLTEPVIEQVFLTPSTQSKVDLERQMYILRKLSMVAITSALNLDNDGITDFYICSLSSRTIVYKGQLTPAQLKDYYFADLGNERFTSYMALIHSRFSTNTFPSWDRAQPMRVLGHNGEINTLKGNVNWMKAREGLLKCKELGLSENELKKLLPIVDANSSDSGAFDGVLEFLIQSGKSLPEAVMMMIPEAWQNDKNMDPQRKAFYEYFSALMEPWDGPALISFTDGHYLGATLDRNGLRPGRFYVTHSGRVVMASEVGVVDIPVEDVSRKGRLNPGMMLLVDFEKHIVVNDDALKEQYSLARPYGEWLKKQKLELKDIVDSVHESERVPPSITGVVPASGDDVDMENMGINGLLVPLKAFGYTVESLEMLLLPMAKDGTEALGSMGNDAPLAVMSNREKLTFEYFKQMFAQVTNPPIDPIREKIVTSTECMVGPEGDLTEITEDQCHRLSLKGPLLSIEEMEAIKKMNYRGWRSKVIDITYSKGRGKKGLEEALDRICAEAHDAISDGYTTLVLSDRAFSRKRVAVSSLLAVGAVHQHLVKTLERTRVALVIESAEPREVHHFCTLVGFGADAICPYLAVEAIWRLQVDGKIPPKTNGEFYSKDELVKKYFKASNYGMMKVLAKMGISTLASYKGAQIFEALGLSSEVIERCFAGTPSRVEGATFEMLARDALKLHELAFPSRVFSAGSAEAKALPNPGDYHWRKGGEIHLNDPLAISKLQEAARTNSKDAYEQYSKLIHELNKACNLRGLLKFKEAAVKVSLDEVEPASEIVKRFCTGAMSYGSISLEAHTALATAMNKIGGKSNTGEGGEQPSRMEPLSDGSRNPKRSAIKQVASGRFGVTSYYLTNADELQIKMAQGAKPGEGGELPGHKVVGDIAVTRNSTPGVGLISPPPHHDIYSIEDLAQLIHDLKNANPAARVSVKLVSEAGVGVVASGVVKGHADHVLISGHDGGTGASRWTGIKNAGLPWELGLAETHQTLVANDLRGRTVLQTDGQIKTGRDVAIATLLGAEEFGFSTAPLITLGCIMMRKCHKNTCPVGIATQDPVLREKFAGEPEHVINFFFMIAEEMREIMSQLGFRTVNEMVGRSDMLEVDKEVIKSNEKLENIDLSFLLRPAAELRPEAAQYCVQKQDHGLDMALDNKLIGLSNAALEKGLPVYIESPIHNVNRAVGTMLSHAVTKKYHLNGLPTDTIHIRFNGSAGQSFGAFLCPGITLELEGDGNDYVGKGLSGGKIVVFPPKGSTFDPKKNIVIGNVALYGATSGEAYFNGMAAERFCVRNSGANAVVEGVGDHGCEYMTGGIVVVLGNTGRNFAAGMSGGIAYVLDMDGKFLSQCNHELVDLDKVEEEEDITTLRMLIQQHQRHTNSVLAKEVLPDFENLVPKFIKVFPKEYKRVLASTKSKEASKDAVESASNHGEEQDEIELVEEDAFEKLKKLATASINGKPSEAESSKRPSQVIDPVKHRGFVAYERESVQYRDPNARINDWNEVMKETKPGPLLKTQSARCMDCGTPFCHQENSGCPLGNKIPEFNELVYQNRWREALDRLLETNNFPEFTGRVCPAPCEGSCVLGIIENPVSIKSIECAIIDKAFEEGWMVPRPPARRTGKRVAVVGSGPSGLAAADQLNKMGHTVTVYERADRIGGLMMYGVPNMKTDKVDIVQRRVNLMAEEGINFVVNANIGHDPLYSLDRLREENDAIVLAVGATKPRDLPVPGRELSGVHFAMEFLHANTKSLLDSNLQDGNFISAKGKKVVVIGGGDTGTDCIGTSIRHGCSSIVNLELLPQPPQTRAPGNPWPQWPRIYRVDYGHQEGAAKFGKDPRSYEVLTKRFVGDENGVVKGLEVIRVLWEKDETGRFQFKEIEGSEEILEADLVLLAMGFLGPEPTIAEKLGIERDNRSNFKAEYGRFSTSLKGVFAAGDCRRGQSLVVWAISEGRQAAAQVDSFLTNEDIEHNVAGSPDDKLQHKFAVPNKQ

>Glyma.04G236900|Gmax_Wm82.a6.v1/1-2191

MSCSLSFSRLSARSARTLPRENEKPRLNARCPVRVTKFLGTRLRSSGRSLRSERFHVWQSEGPGRTPKLRVAVRSALSAVPNKPLGLYDPAMDKDSCGVGFVAELSGESSRQTVTDALEMLVRMTHRGACGCEANTGDGAGIMVALPHQFYKEVVDFELPPPGKYAVGMLFLPTSNSRREESKNVFQKVAESLGHSVLGWRSVPTDNTGLGKSAVLTEPVIEQVFLTPSTQSKVDLERQMYILRKLSMVAISSALNLDNDGIIDFYICSLSSRTVVYKGQLTPAQLKDYYFADLGNERFTSYMALIHSRFSTNTFPSWDRAQPMRVLGHNGEINTLRGNVNWMKAREGLLKCKELGLSENELKKLLPIVDANSSDSGAFDGVLEFLIQSGKSLPEAVMLMIPEAWQNDNNMDPQRKAFYEYFSALMEPWDGPALIAFTDGHYLGATLDRNGLRPGRFYVTHSGRVVMASEVGVVDIPLEDVSQKGRLNPGMMLLVDFEKHIVVNDDALKEQYSLARPYGEWLKKQKLELKDIVDSVHESERVPPSITGVMPASGDDVDMENMGIHGLLAPLKAFGYTVESLEMLLLPMAKDGTEALGSMGNDTPLAIMSNREKLTFEYFKQMFAQVTNPPIDPIREKIVTSTECMVGPEGDLTEITEEQCHRLSLKGPLLSIEEMEAIKKMNYRGWRSKVIDITYSKGRGKKGLEEALDRICAEAHDAISDGYTTLVLSDRAFSRKRVAVSSLLAVGAVHQHLVKTLERTRVALVIESAEPREVHHFCTLVGFGADAICPYLAVEAIWRLQVDGKIPPKANGEFYSKDELVKKYFKASNYGMMKVLAKMGISTLASYKGAQIFEALGLSSEVIERCFAGTPSRVEGATFKMLARDALQLHGLAFPSRVFSPGSAEAKALPNPGDYHWRKGGEIHLNDPLAISKLQEAARTNSIDAYKQYSKLIHELNKACNLRGLLKFKEAAVKVPLDEVEPASEIVKRFCTGAMSYGSISLEAHTALATAMNKIGGKSNTGEGGEQPSRMEPLSDGSKNPKRSAIKQVASGRFGVTSYYLTNADELQIKMAQGAKPGEGGELPGHKVVGDIAVTRNSTPGVGLISPPPHHDIYSIEDLAQLIHDLKNANPAARVSVKLVSEAGVGVVASGVVKGHADHVLISGHDGGTGASRWTGIKNAGLPWELGLAETHQTLVANDLRGRTVLQTDGQIKTGRDVAIATLLGAEEFGFSTAPLITLGCIMMRKCHKNTCPVGIATQDPVLREKFAGEPEHVINFFFMIAEEMREIMSQLGFRTVNEMVGRSDMLEVDKEVIKSNEKLENIDLSLLLRPAAELRPEAAQYCVQKQDHGLDMALDNKLIGLSNAALVKGLPVYIESPIHNVNRAVGTMLSHEVTKKYHLNGLPTDTIHIRFNGSAGQSFGAFLCPGITLELEGDGNDYVGKGLSGGKIVVFPPKGSTFDPKQNIVIGNVALYGATSGEAYFNGMAAERFCVRNSGAKAVVEGVGDHGCEYMTGGIVVVLGNTGRNFAAGMSGGIAYVLDMDGKFLSRCNHELVDLDKVEEEEDITTLRMLIQQHQRHTNSVLAKEVLADFENLVPKFIKVFPKEYKRVLASIKSKEASKDAAESASKHGEEQDEIELVEKDAFEELKKLATASVNGKPIEAESFKRPSQVIDPVKHRGFVAYEREGVQYRDPNARINDWNEVMKETKPGPLLKTQSARCMDCGTPFCHQENSGCPLGNKIPEFNELVYQNRWREALERLLETNNFPEFTGRVCPAPCEGSCVLGIIENPVSIKSIECAIIDKAFEEGWMVPRPPARRTGKRVAVVGSGPSGLAAADQLNKMGHTVTVYERADRIGGLMMYGVPNMKADKVDIVQRRVNLMAEEGINFVVNANIGHDPLHSLDRLREENNAIVLAVGATKPRDLPVPGRELSGVHFAMEFLHANTKSLLDSNLQDGNFISAKGKKVVVIGGGDTGTDCIGTSIRHGCSSIVNLELLPQPPQTRAPGNPWPQWPRIYRVDYGHQEGAAKFGKDPRSYEVLTKRFVGDENGVVKGLEVIRVRWEKDETGRFQFKEIEGSEEIIEADLVLLAMGFLGPESTIAEKLGIERDNRSNFKAEYGRFSTSLKGVFAAGDCRRGQSLVVWAISEGRQAAAQVDSFLTNEDLEHNVAGSPDELIKKKQQPRDTVAVANKQ

>Glyma.14G162300|Gmax_Wm82.a6.v1/1-2192

MSNALSFTLPALNNPQLNALNSPNKARLRHGLRIRVVASCIERKRFLGTRVRPSGSDRIWLLQSGRVPKLRVAVRSAFSAVPEKPLGLYDPAMDKDSCGVGFVAELSGESSRKTVTDALEMLVRMTHRGACGCEANTGDGAGILVALPHVFYQEVVDFELPPQGKYAVGMLFLPKSEKRREESKKMFSKVAESLGHTILGWRSVPTDNAGLGKSALQTEPVIEQVFLTPSAKSKVDLERQMYILRKLSMAAITSALNLQNDGITDFYICSLSSRTVVYKGQLTPAQLRDYYFADIGNERFTSYMALIHSRFSTNTFPSWDRAQPMRVLGHNGEINTLRGNVNWMKAREGLLKCKELGLSENELKKLLPIVDANSSDSGAFDGVLEFLLHSGKSLPEAVMMMIPEAWQNDKNMDSQRKAFYEYFSALMEPWDGPALISFTDGHYLGATLDRNGLRPGRFYVTHSGRVIMASEVGVVDIPPEDVRRKGRLNPGMMLLVDFEKHIVVNDDALKEQYSLARPYEDWLKRQKIELKDIVNSVDESERVPPPIAGVAPLSNDDADMENMGIHGLLAPLKAFGYTVESLEMLLLPMAKDGVEALGSMGNDTPLAVMSKREKLTFEYFKQMFAQVTNPPIDPIREKIVTSMQCMVGPEGDLTEITEEQCNRLSLKGPLLSTEEMEAIKKLNYKGWRSKVIDITYSKECGKRGLEEALDRICAEAHHGISEGYTTLVLSDRAFSKKRVAVSSLLAVGAVHQHLVKNLERTRVALIVESAEPREVHHFCTLVGFGADAICPYLAVDAIWRLQVDGKIPPKASGEFHSKDELVKKYFKASNYGMMKVLAKMGISTLASYKGAQIFEALGLSSEVIAKCFAGTPSRVEGATFEMLARDAFQLHELAFPSWVFSPGSAEAVALPNPGDYHWRKGGEVHLNDPLAMAKLQEAARTNSVDAYKQYSKLIHELNKACNLRGLLKFKETAVKIPIDEVEPASEIVKRFCTGAMSYGSISLEAHTALAMAMNKMGGKSNTGEGGEQPSRMEPLPNGSMNPKRSAIKQVASGRFGVSSYYLTNADELQIKMAQGAKPGEGGELPGHKVIGDIAVTRNSTAGVGLISPPPHHDIYSIEDLAQLIHDLKNANPAARISVKLVSEAGVGIIASGVVKGHADHVLISGHDGGTGASRWTGIKNAGLPWELGLAETHQTLVANDLRGRTVLQTDGQLKTGRDVAIATLLGAEEFGFSTAPLITLGCIMMRKCHKNTCPVGIATQDPVLREKFAGEPEHVINFFFMVAEEMREIMSQLGFRTVNEMVGHSDMLEFDKDVVKSNEKLENIDLSLLLRPAAELRPEAAQYCVQKQDHGLDMALDNKLISLSNAALEKGLPVYIETPIYNVNRAVGTMLSHEVTKRYHLAGLPTDTIHVRFTGSAGQSFGAFLCPGITLELEGDSNDYVGKGLSGGKIVVYPPKESNFDPKENIVIGNVALYGATCGEAYFNGMAAERFCVRNSGAKAVVEGVGDHGCEYMTGGTVVVLGKTGRNFAAGMSGGIAYVLDVDGKFQSRCNLELADLDKVEEEEDILALRMLIQQHQRHTNSLLAKEVLDDFENLLPKFIKVFPREYKRVLASMKSEETSKDALVHAAKDDQDDEAQAVEKDAFEELKKLVMASLNEEPSQAESPKRPSRVTGAIKHRGFVAYEREGVQYRDPNVRMTDWKEVMEETKPGPLLKTQSARCMDCGTPFCHQENSGCPLGNKIPEFNELVYQNRWREALERLLETNNFPEFTGRVCPAPCEGSCVLGIIENPVSIKSIECAIIDKAFEEGWMVPRPPVKRTGKRVAIVGSGPSGLAAADQLNKMGHTVTVYERADRIGGLMMYGVPNMKTDKVDIVQRRVNLMAEEGIDFVVNANVGHDPLYTLDRLRGENDAIVLTVGATKPRDLPVPGRELSGVHFAMEFLHANTKSLLDSNLQDGNYISAKGKKVVVIGGGDTGTDCIGTSIRHGCSSVVNLELLPQPPPTRAPSNPWPQWPRIFRVDYGHQEAAAKFGKDPRSYEVLTKRFIGDENGVVKGLEVIRVCWEKDETGKFQFKEIEGSEEIIEADLVLLAMGFLGPESTIAEKLGVERDNRSNFKADYGRFSTNVKGVFAAGDCRRGQSLVVWAISEGRQAAAQVENYLIKEDQDHRNQDGLVKRQQGLNMKQQGSNKHTVMT

>Glyma.19G065600|Gmax_Wm82.a6.v1/1-2197

MSNAVSFTLPSLNNPQLNALNSPNKARLRLGLRSRVVASCIERKRFLGTRFRPIGSDRIRLLQSCRLPKPRVAVRSAFSAVPEKPLGLYDPAMDKDSCGVGFVAELSGESSRKTVTDALEMLVRMTHRGACGCEANTGDGAGILVALPHAFYQEVVDFELPPQEKYAVGMFFLPKSEKRREESKRIFSKVAESLGHTVLGWRSVPIDNTGLGKSALQTEPVIEQVFLTPSAQSKIDLERQMYILRKLCMAAITSALNLQNDGIADFYICSLSSRTVVYKGQLTPAQLRDYYFADLGNERFTSYMALIHSRFSTNTFPSWDRAQPMRVLGHNGEINTLRGNVNWMKAREGLLKCKELGLSENELKKLLPIVDANSSDSGAFDGVLEFLLHSGKSLPEAVMMMIPEAWQNDKNMDSQRKAFYEYFSALMEPWDGPALISFTDGHYLGATLDRNGLRPGRFYVTHSGRVIMASEVGVVDIPPEDVCRKGRLNPGMMLLVDFVKHTVVNDDALKEQYSLARPYEDWLKRQKIELKDIVNSVHESERVPPPIAGVAPLSNDDADMENMGIHGLLVPLKAFGYTVESLEMLLLPMAKDGVEALGSMGNDTPLAVMSKREKLTFEYFKQMFAQVTNPPIDPIREKIVTSMQCMVGPEGDLTEITEEQCHRLSLKGPLLSTEEMEAIKKMNYRGWRSKVIDITYSKECGKRGLDEALDRMCAEAHDAINEGYTTLVLSDRAFSKKRISVSSLLAVGAVHQHLVKTLERTRVALIVESAEPRKVHHFCTLVGFGADAICPYLAIDAIWRLQVDGKIPPKASGEFHSKDELVKKYFKASNYGMMKVLAKMGISTLASYKGAQIFEALGLSSEVIEKCFAGTPSRVEGATFEMLACDAFQLHELAFPSWVFSPGSAEALALPNPGDYHWRKGGEVHLNDPLAMAKLQEAARTNSVDAYKQYSKLIHELNKACNLRGLLKFKETAVKIPIDEVEPASEIVKRFCTGAMSYGSISLEAHTALAMAMNKIGGKSNTGEGGEQPSRMEPLPDGSMNPKRSAIKQVASGRFGVSSYYLTNADELQIKMAQGAKPGEGGELPGHKVIGDIAVTRNSTSGVGLISPPPHHDIYSIEDLAQLIHDLKNANPAARISVKLVSEAGVGIIASGVVKGHADHVLISGHDGGTGASRWTGIKNAGLPWELGLAETHQTLVANDLRGRTVLQTDGQLKTGRDVAIATLLGAEEFGFSTAPLITLGCIMMRKCHKNTCPVGIATQDPVLREKFAGEPEHVINFFFMVAEEMREIMSQLGFRRVNEMVGRSDMLEVDKEVVKSNEKLENIDLSLLLRPAAELRPEASQYCVQKQDHGLDMALDNKLISLSSAALEKGLPVYIETPIYNVNRAVGTMLSHEVTKLYHLAGLPNDTIHIRFTGSAGQSFGAFLCPGITLELEGDSNDYVGKGLSGGKIVVYPPKESNFDPKENIVIGNVALYGATRGEAYFNGMAAERFCVRNSGAKAVVEGVGDHGCEYMTGGTVVVLGKTGRNFAAGMSGGIAYVLDVDGKFQSRCNLELVDLDKVEEEEDILTLRMLIQQHQRHTNSRLAKEVLDDFENLLPKFIKVFPREYKRVLASMKSEETSKDAVVHAAKHEQDDEAQAVEKDAFEELKKLATASLNEKPSQAESPKRPSQVTGAIKHRGFVSYEREGVQYRDPNVRMTDWKEVMEETKPGPLLKTQSARCMDCGTPFCHQENSGCPLGNKIPEFNELVYQNRWHEALERLLETNNFPEFTGRVCPAPCEGSCVLGIIENPVSIKSIECAIIDKAFEEGWMVPRPPVQRTGKRVAIVGSGPSGLAAADQLNKMGHTVTVYERADRIGGLMMYGVPNMKTDKVDIVQRRVNLMAEEGIDFVVSANVGHDPLYSLDRLREENDAIVLAVGATKPRDLPVPGRELSGVHFAMEFLHANTKSLLDSNLEDGNYISAKGKKVVVIGGGDTGTDCIGTSIRHGCSSVVNLELLPQPPPTRAPGNPWPQWPRIFRVDYGHQEAAAKFGKDPRSYEVLTKRFIGDENGVLKGLEVIRVCWEKDATDKFQFKEIEGSEEIIEADLVLLAMGFLGPESTIAEKLGVDRDNMSNFKAGYGHFSTNVKGVFAAGDCRRGQSLVVWAISEGRQAAAQVDNYLVKEDKDHRNQDGLVKRQQGLYKKQHGSSKHTVMTYRWLL

>KAI5394197.1|Pisum_sativum/1-2195

MSNSLSLTFTALNNPQINVISNPSARLRPFARVRCSAMCVERKRWLGTGLRRSGSERSRFLESVRPGRLPKLRVAVKASFSAVPEKPMGLYDPAFDKDACGVGFVAELSGQSNRNTVTDALEMLVRMTHRGGCGCEANTGDGAGILVALPHEFYQEVVDFQLPPQGNYAVGMFFLPRSDNRRKESKNIFRKVAESLGHTVLGWRSVPTDNTGLGKSALLTEPVIEQVFLTPSSSSKVDLEKQMYILRKLSMVAITAALNLQNDGITDFYICSLSSRTVIYKGQLTPTQLREYFADLGNERFTSYMALIHSRFSTNTFPSWDRAQPFRVLGHNGEINTLRGNVNWIKAREGLLKCEELGLSENDLKKFLPIVDANSSDSGCFDGVLEFLLHSGKSLPEAVMMMIPEAWQNDKNMDPQRKAFYEYYSALMEPWDGPALISFTDGHYLGATLDRNGLRPGRFYVTHSGRVIMASEVGVVDIPPEDVCRKGRLNPGMMLLVDFEKHIVVNDDALKEQYSLARPYGDWLKNQKIELKDIVDSVHESDTVPPAISGVAPLSNDDVDMESMGLHGLLAPLKAFGYSIESLEMLLLPMAKDGVEALGSMGNDTPLAVMSNREKLTFEYFKQMFAQVTNPPIDPIREKIVTSTQCMVGPEGDLTETTEEQCHRLSLKGPLLSTKEMEAIKKMNYRGWRSKVIDITYSKERGKKGLEEALDRICTEAHNAISEGYTTIVLSDRAFSHKRVAVSSLLAVGAVHQHLVKTLERTRVALMIESAEPREVHHFCTLVGFGADAICPYLAVEAIWRLQVDGKIPPKASGEFHSKDELVKKYFKASNYGMMKVLAKMGISTLASYKGAQIFEALGLSSEVIEKCFAGTPSRVEGATFEMLARDALQLHELAFPSRIFSPGSAEAVALPNPGDYHWRKGGEVHLNDPLAIAKLQEATRTNSVDAYKQYSKTIHELNKACNLRGLLKFKDTSSKIPIGEVEPASEIVKRFCTGAMSYGSISLEAHTALATAMNKIGGKSNTGEGGEQPSRMEPLADGSRNPKRSAIKQIASGRFGVSSYYLTNADELQIKMAQGAKPGEGGELPGHKVIGDIAVTRSSTAGVGLISPPPHHDIYSIEDLAQLIYDLKNANPAARISVKLVSEAGVGVIASGVVKAHAEHVLISGHDGGTGASRWTGIKNAGLPWELGLAETHQTLVANDLRGRTTLQTDGQLKTGRDVAIAALLGAEEYGFSTAPLITLGCIMMRKCHKNTCPVGIATQDPVLREKFAGEPEHVINFFFMVAEEMREIMAQLGFRTVNEMVGRSDMLEVDKEVVNGNAKLENIDLSLLLRPAAELRPEAAQYCVQKQDHDLDMALDNKLISLSNAALEKGLPVYIETPILNTNRAVGTMLSHEVTKRYNLAGLPADTIHIQFNGSAGQSFGAFLCPGITLELEGDSNDYIGKGLSGGKIVVYPPKGSNFDPKDNIIIGNVALYGATRGEAYFNGMAAERFCVRNSGAHAVVEGVGDHGCEYMTGGTVVILGKTGRNFAAGMSGGIAYVLDVDGTFQSRCNPELVDLDKVEEEEDIITLRMLIQQHQRHTNSLLAKEVLVDFDNLLPKFVKVFPREYKRVLASMKSDAASKEAVERAAADVDEKDDDEAQAVEKDAFEELKKLATASLNEKPSEAPKRPSQVTDAVKHRGFVAYEREGVQYRDPNVRLNDWNEVMMETKPGPLLKTQSARCMDCGTPFCHQENSGCPLGNKIPEFNELVYQNRWQEALERLLETNNFPEFTGRVCPAPCEGSCVLGIIENPVSIKNIECAIIDKAFEEGWMVPRPPVKRTGKRVAIVGSGPSGLAAADQLNKMGHTVTVFERADRIGGLMMYGVPNMKTDKVDIVQRRVNLMAEEGINFVVNANIGNDPRYSLEQLREENDAIVLAVGATKPRDLPVPGRELSGVHFAMKFLHANTKSLLDSNLQDGNFISAKGKKVVVIGGGDTGTDCIGTSIRHGCTAVVNLELLPQPPPTRAPGNPWPQWPRIFRVDYGHQEAETKFGKDPRTYEVLTKRFVGDENGNVKGLEVVHVRWEKDETGKFQFKEIEGSEEIIEADLVLLAMGFLGPESTIAEKLGVEQDNRSNFKADYGRFSTNVDGVFAAGDCRRGQSLVVWAISEGRQAASKVDSYLTKEDQSIDGNQDEFVQRQHELNNKHKGSSKHTVMT

>KAI5394198.1|Pisum_sativum/1-1796

MMMIPEAWQNDKNMDPQRKAFYEYYSALMEPWDGPALISFTDGHYLGATLDRNGLRPGRFYVTHSGRVIMASEVGVVDIPPEDVCRKGRLNPGMMLLVDFEKHIVVNDDALKEQYSLARPYGDWLKNQKIELKDIVDSVHESDTVPPAISGVAPLSNDDVDMESMGLHGLLAPLKAFGYSIESLEMLLLPMAKDGVEALGSMGNDTPLAVMSNREKLTFEYFKQMFAQVTNPPIDPIREKIVTSTQCMVGPEGDLTETTEEQCHRLSLKGPLLSTKEMEAIKKMNYRGWRSKVIDITYSKERGKKGLEEALDRICTEAHNAISEGYTTIVLSDRAFSHKRVAVSSLLAVGAVHQHLVKTLERTRVALMIESAEPREVHHFCTLVGFGADAICPYLAVEAIWRLQVDGKIPPKASGEFHSKDELVKKYFKASNYGMMKVLAKMGISTLASYKGAQIFEALGLSSEVIEKCFAGTPSRVEGATFEMLARDALQLHELAFPSRIFSPGSAEAVALPNPGDYHWRKGGEVHLNDPLAIAKLQEATRTNSVDAYKQYSKTIHELNKACNLRGLLKFKDTSSKIPIGEVEPASEIVKRFCTGAMSYGSISLEAHTALATAMNKIGGKSNTGEGGEQPSRMEPLADGSRNPKRSAIKQIASGRFGVSSYYLTNADELQIKMAQGAKPGEGGELPGHKVIGDIAVTRSSTAGVGLISPPPHHDIYSIEDLAQLIYDLKNANPAARISVKLVSEAGVGVIASGVVKAHAEHVLISGHDGGTGASRWTGIKNAGLPWELGLAETHQTLVANDLRGRTTLQTDGQLKTGRDVAIAALLGAEEYGFSTAPLITLGCIMMRKCHKNTCPVGIATQDPVLREKFAGEPEHVINFFFMVAEEMREIMAQLGFRTVNEMVGRSDMLEVDKEVVNGNAKLENIDLSLLLRPAAELRPEAAQYCVQKQDHDLDMALDNKLISLSNAALEKGLPVYIETPILNTNRAVGTMLSHEVTKRYNLAGLPADTIHIQFNGSAGQSFGAFLCPGITLELEGDSNDYIGKGLSGGKIVVYPPKGSNFDPKDNIIIGNVALYGATRGEAYFNGMAAERFCVRNSGAHAVVEGVGDHGCEYMTGGTVVILGKTGRNFAAGMSGGIAYVLDVDGTFQSRCNPELVDLDKVEEEEDIITLRMLIQQHQRHTNSLLAKEVLVDFDNLLPKFVKVFPREYKRVLASMKSDAASKEAVERAAADVDEKDDDEAQAVEKDAFEELKKLATASLNEKPSEAPKRPSQVTDAVKHRGFVAYEREGVQYRDPNVRLNDWNEVMMETKPGPLLKTQSARCMDCGTPFCHQENSGCPLGNKIPEFNELVYQNRWQEALERLLETNNFPEFTGRVCPAPCEGSCVLGIIENPVSIKNIECAIIDKAFEEGWMVPRPPVKRTGKRVAIVGSGPSGLAAADQLNKMGHTVTVFERADRIGGLMMYGVPNMKTDKVDIVQRRVNLMAEEGINFVVNANIGNDPRYSLEQLREENDAIVLAVGATKPRDLPVPGRELSGVHFAMKFLHANTKSLLDSNLQDGNFISAKGKKVVVIGGGDTGTDCIGTSIRHGCTAVVNLELLPQPPPTRAPGNPWPQWPRIFRVDYGHQEAETKFGKDPRTYEVLTKRFVGDENGNVKGLEVVHVRWEKDETGKFQFKEIEGSEEIIEADLVLLAMGFLGPESTIAEKLGVEQDNRSNFKADYGRFSTNVDGVFAAGDCRRGQSLVVWAISEGRQAASKVDSYLTKEDQSIDGNQDEFVQRQHELNNKHKGSSKHTVMT

>Soltu.DM.03G022630|Stuberosum_v6.1/1-2327

MSIASSSVLQTKNNGVVMPSPVKSLVGHQLIAMPLGRVGVGLGRTRVTRSSVVKRTTGFEKKFYGAKLRASGSERLHLWQSDGPGRAPKLRVVVRSALSQVPEKPLGLYDPSFDKDSCGVGFVAELSGESSRKTVTDAIEMLVRMSHRGACGCETNTGDGAGILVGLPHDFYKEVASEAGFELPPPGQYAVGMFFLPTSDSRREQSKIVFTKVAESLGHTVLGWRPVPTDNSGLGKSALQTEPIIEQVFLTPTPRSKVDFERQMYILRRVAMVAIRAALNLQHGGVKDFYICSLSSRTVVYKGQLKPNQLKEYYFADLGNERFTSYMALVHSRFSTNTFPSWDRAQPMRVLGHNGEINTLRGNVNWMRAREGLLKCKELGLSKTEMKKLLPIVDASSSDSGAFDGVLELLLRAGRSLPEAVMMMIPEAWQNDKNMDPSRKALYEYFSALMEPWDGPALMSFTDGRYLGATLDRNGLRPGRFYVTYSGRVIMASEVGVVDIPPEDVSRKGRLNPGMMLLVDFENHVVVDDDALKKQYSLARPYGQWLKKQKIELKDIVESVNYSYRVPPPIAGVLPAVSDEDSMENMGLHGLLAPLKAFGYTIEALEMLLLPMAKDGVEALGSMGNDAPLAVMSNREKLTFEYFKQMFAQVTNPPIDPIREKIVTSMQCMVGPEGDLTETTEEQCHRLSLKGPLLSIEEMEAVKKMNYRGWRSKVLDITYSRDRGTKGLEETLDRICSEAHDAIQEGYTAIVLSDRGFSPKRVAVSSLLAIGAVHHHLVKKLERTRVALIVESAEPREVHHFCTLVGFGADAICPYLAVEAIWRLQVDGKIPPKSTGEFHSKDELVKKYFKASHYGMMKVLAKMGISTLASYKGAQIFEAVGLSSEVMERCFNGTPSRVEGATFDALAKDALNLHGLAFPSRALAPGSAEAVALPNPGDYHWRKGGEIHLNDPFAIAKLQEAAQSNSVAAYKEYSKRVQELNRQCNLRGLLKFKEGEVKVPLEEVEPASEIVKRFCTGAMSYGSISLEAHATLAMAMNKIGGKSNTGEGGEQPSRMEPLPNGSKNPKRSAIKQVASGRFGVSSYYLTNADELQIKMAQGAKPGEGGELPGHKVIGDIAVTRNSTAGVGLISPPPHHDIYSIEDLAQLIHDLKNANPGARVSVKLVSEAGVGVIASGVVKGHADHVLISGHDGGTGASRWTGIKSAGLPWELGLAETHQTLVANDLRGRTVLQTDGQLKTGRDVAIAALLGAEEFGFSTAPLITLGCIMMRKCHKNTCPVGIATQDPILREKFAGEPEHVINFFFMLAEELREIMSQLGFRTLIEMVGRSDMLEMDNDLVKNNDKLKNIDLSLLLRPAADIRPEAAQYCIQKQDHGLDLALDNNLIALSKAALEKSLPVYIETPICNVNRAVGTMLSHEVTKRYHLAGLPADTIHIKLSGSAGQSLGAFLCPGITLELEGDSNDYVGKGLSGGKIVVYPPKGSKFDPKENIVIGNVALYGATSGEAYFNGMAAERFCVRNSGAKAVVEGVGDHGCEYMTGGTVVVLGKTGRNFAAGMSGGVAYVLDLHSTFHSRCNSELVDLDKVEEEEDVMTLKMMIQQHQRNTNSQLAKEVLADFDNLLPRFIKVFPRDYKRVLASMKKEEAYEAAKERAIKEAEEQEEEELKEKDAFEELKKLAAASKDESSQVEEENTLKRPTQVAEAVKHRGFVAYERQGVSYRDPNVRMKDWKEVMEESKPGPLLTTQSARCMDCGTPFCHQENSGCPLGNKIPEFNELVYQNRWREALDRLLETNNFPEFTGRVCPAPCEGSCVLGIIENPVSIKSIECAIIDKAFEEGWMVPRPPSERTGRRVAIVGSGPSGLAAADQLNRLGHTVTVFERADRIGGLMMYGVPNMKTDKIDVVQRRVDLMEKEGVKFVVNANIGNDPAYSLDSLREDHDAIILAVGATKPRDLPVPGRDLSGVHFAMEFLHANTKSLLDSNLQDGKYISAKGKKVVVIGGGDTGTDCIGTSIRHGCSSVVNLELLPQPPNTRAPGNPWPQWPRVFRVDYGHQEASAKFGKDPRSYEVLTKRFIGDENGNVKGLEVIRVQWEKDASGRFQFKEVEGSEEIIGADLVMLAMGFLGPESTIADKLGLEKDNRSNFKADYGRFSTSVEGVFAAGDCRRGQSLVVWAISEGRQAAAQVDKFLMKDDEDSSADAASQQESVKKQPTVGRTEKRGKIEGAADSKLNQSPNRFSVSLASLNSASRLICSSTQGGQIKHDHPGEHEVAHTLDEKEKVDNKNEGNLEVEDDSDEEDTSNVNKKTGEIGGPKGPEPTRYGDWERGGRCSDF

>Solyc03G001767|Slycopersicum_ITAG5.0/1-2231

MSIASSSVLQSKNNGVVMSSPVKSLVGHQLNAMPLGRVGVGLGRTRVTRSSVVKRTTGFEKKFYGAKLRASGPERLHLWQSDGPGRAPKLRVVVRSALSQVPEKPLGLYDPSFDKDSCGVGFVAELSGESSRKTVADAIEMLVRMSHRGACGCETNTGDGAGILVGLPHDFYKEVTSEAGFEIPPPGQYAVGMFFLPTSDSRREQSKIVFTKVAESLGHTVLGWRPVPTDNSGLGKSALQTEPIIEQVFLTPTPRSKVDFERQMYILRRVAMVAIRAALNLQHGGVKDFYVCSLSSRTVVYKGQLKPNQLKEYYFADLGNERFTSYMALVHSRFSTNTFPSWDRAQPMRVLGHNGEINTLRGNVNWMRAREGLLKCKELGLSKTEMKKLLPIVDASSSDSGAFDGVLELLLRAGRSLPEAVMMMIPEAWQNDKNMDPSRKALYEYFSALMEPWDGPALMSFTDGRYLGATLDRNGLRPGRFYVTYSGRVIMASEVGVVDIPPEDVSRKGRLNPGMMLLVDFENHVVVDDDALKKQYSLARPYGQWLKKQKIELKDIVESVNYSYRVPPPIAGVLPAVSDEDSMENMGLHGLLAPLKAFGYTTEALEMLLLPMAKDGVEALGSMGNDAPLAVMSNREKLTFEYFKQMFAQVTNPPIDPIREKIVTSMQCMVGPEGDLTETTEEQCHRLSLKGPLLSIEEMEAVKKMNYRGWRSKVLDITYSRDRGTKGLEETLDRICSEAHDAIQEGYTAIVLSDRGFSPKRVAVSSLLAIGAVHHHLVKKLERTRVALIVESAEPREVHHFCTLVGFGADAICPYLAVEAIWRLQVDGKIPPKSTGEFHSKDELVKKYFKASHYGMMKVLAKMGISTLASYKGAQIFEAVGLSSEVMERCFNGTPSRVEGATFEALAKDALNLHGLAFPSRALAPGSAEAVALPNPGDYHWRKGGEIHLNDPFAIAKLQEAAQSNSVAAYKEYSKRVQELNRQCNLRGLLKFKEGEVKVPLEEVEPASEIVKRFCTGAMSYGSISLEAHATLAIAMNKIGGKSNTGEGGEQPSRMEPLPNGTKNPKRSAIKQVASGRFGVSSYYLTNADELQIKMAQGAKPGEGGELPGHKVIGDIAVTRNSTAGVGLISPPPHHDIYSIEDLAQLIHDLKNANPGARVSVKLVSEAGVGVIASGVVKGHADHVLISGHDGGTGASRWTGIKSAGLPWELGLAETHQTLVANDLRGRTVLQTDGQLKTGRDVAIAALLGAEEFGFSTAPLITLGCIMMRKCHKNTCPVGIATQDPILREKFAGEPEHVINFFFMLAEEVREIMSQLGFRALTEMVGRSDMLEMDNDLVKNNDKLKNIDLSLLLRPAADIRPEAAQYCIQKQDHGLDMALDNNLIALSKAALERSLPVYIETPICNVNRAVGTMLSHEVTKRYHLAGLPTDTIHIKLSGSAGQSLGAFLCPGITLELEGDSNDYVGKGLSGGKIVVYPPKGSKFDPKENIVIGNVALYGATSGEAYFNGMAAERFCVRNSGAKAVVEGVGDHGCEYMTGGTVVVLGKTGRNFAAGMSGGVAYVLDLHSTFHSHCNPELVDLDKVEEEEDIMTLKMMIQQHQRNTNSQLAKEVLADFDNLLPRFIKVFPRDYKRVLASMKKEEAYEAAKERAIKEAEEQEEEELKEKDAFEELKKLAAASKDESSQVEEEQTLKRPIQVAEAVKHRGFVAYERQGVSYRDPNVRMEDWKEVMEESKPGPLLTTQSARCMDCGTPFCHQENSGCPLGNKIPEFNELVYQNRWREALDRLLETNNFPEFTGRVCPAPCEGSCVLGIIENPVSIKSIECAIIDKAFEEGWMVPRPPSERTGRRVAIVGSGPSGLAAADQLNRLGHTVTVFERADRIGGLMMYGVPNMKTDKIDVVQRRVDLMEKEGVKFVVNANIGNDPAYSLDSLREDHDAIILAVGATKPRDLPVPGRELSGVHFAMEFLHANTKSLLDSNLQDGKYISAKGKKVVVIGGGDTGTDCIGTSIRHGCTSVVNLELLPQPPNTRAPGNPWPQWPRIFRVDYGHQEAAVKFGKDPRSYEVLTKRFIGDENGNVKGLEVIRVQWEKDASGRFQFKEVEGSEEIIGADLVMLAMGFLGPESTIADKLGLEKDNRSNFKADYGRFSTSVEGVFAAGDCRRGQSLVVWAISEGRQAAAQVDKFLMKDDEDSSADAASQQESVKKQPTVGRTDKKRKDRRRSRINFTGFLSR

>evm.TU.Scaffold_637.1640|Carabica_v0.5/1-2206

MSVASGTVIPSKSGRGAVGLPSVTKPFANNQLNVVPLSRVLGIRSAAGRRSGCGLERSSKLFGARLPRALGSERSHLWQTDGPGRPPKLRVFQLRSALSQIPEKPLGLYDPSFDKDSCGVGFVAELSGESSRKTVTDAIEMLIRMSHRGACGCETNTGDGAGILVAKEVGFELPPPREYAVGMLFLPTSESRREQSKIVFTKVAESLGHTVLGWRTVPTDNSGLGKSALQTEPVVEQVFLTPTPRSKADFEQQMYILRRVSMVAIRAALNLQHGGVRDFYICSLSSRTVVYKGQLKPAQLKEYYYADLGNERFTSYMALIHSRFSTNTFPSWDRAQPMRVLGHNGEINTLRGNVNWMKAREGLLKCKELGLSKTEMKKLLPIVDASSSDSGAFDGVLELLVRAGRSLPEAIMMMIPEAWQNDKNMDPQRKALYEYFSALMEPWDGPALISFTDGRYLGATLDRNGLRPGRFYITHSGRVIMASEVGVVDIPPEDVSRKGRLNPGMMLLVDFEKHVVVDDEALKQQYSLARPYGEWLKRQKIELKDIVESVHESQRVSPAIAGVVPASKDDDSMENMGIHGLLAPLKAFGYTVEALEMLLLPMANDGIEALGSMGNDAPLAVMSNREKLTFEYFKQMFAQVTNPPIDPIREKIVTSMECMIGPEGDPTETTEEQCHRLSLKGPFLSVEEMEAIKKMDYRGWRSKVIDITYSVGHGRKGLEETLDRICSEAHDAIKEGYTALVLSDRAFSPKRVAVSSLLAVGAVHHHLVKRLERTRVALIVESAEPREVHHFCTLVGFGADATCPYLAIEAIWRLQVDGKIPPKPTGQFHSKDELVKKYFNASNYGMMKVLAKMGISTLASYKGAQIFEAVGLSSEVMERCFTGTPSRVEGATFEALANDALELHELAFPSRVLPAGSAEAVALPNPGDYHWRKGGEIHLNDPLAIAKLQEAARSNSVAAYKEYSRRIQELNKSCNLRGLLKFKDEVMGVPLEEVEPASEIVKRFCTGAMSYGSISLEAHSTLAIAMNKIGGKSNTGEGGEQPSRMDPLPDGSMNPKRSAIKQVASGRFGVSSYYLTNADELQIKMAQGAKPGEGGELPGHKVIGDIAVTRNSTAGVGLISPPPHHDIYSIEDLAQLIHDLKNANPGARISVKLVSEAGVGVIASGVVKGHADHVLISGHDGGTGASRWTGIKSAGLPWELGLAETHQTLVANDLRGRTVLQTDGQLKTGRDVAIAALLGAEEFGFSTAPLITLGCIMMRKCHKNTCPVGIATQDPVLREKFAGEPEHVINFFFMLAEEVREIMSQLGFRTLNEMVGRSDMLELNEDLVKNNEKLKNIDLSLLLRPAADIRPDAAQYCIQKQDHSLDMAMDNKLIDLSKAGIEKCVPVYVETGICNTNRAVGTMLSHEVTKRHSMVGLPADTIHIKFNGSAGQSFGAFVCPGITLELEGDSNDYVGKGLSGGKIIVYPPRESRFDAKENIVIGNVALYGATGGEAYFNGMAAERFCVRNSGAKAVVEGVGDHGCEYMTGGTVVVLGKTGRNFAAGMSGGIAFVLDMDSKFQSHCNSELVDLDKVENEDDILTLKMMIQQHQRHTNSKLAKEVLSNFDDLLPKFIKVFPRDYKRVLASMKEKETANIAAERTAREIEEQEEAELMGKDAFEELKKFSAGSLNKKANQVERVISVKRPTCVADAEKNGGFVRYEREGISYRDPNKRIYDWKEVMEELKPGPLLTTQSARCMDCGTPFCHQENSGCPLGNKIPEFNELVYQNRWREALDRLLETNNFPEFTGRVCPAPCEGSCVLGIIENPVSIKSIECTIIDKAFEEGWMVPRPPQMRTGKRVAVVGSGPAGLAAADQLNRMGHAVTVFERADRIGGLMMYGVPNMKADKVDVVQRRVDLMEKEGINFVVNANVGKDPSFSLDRLREEHDAIVLAVGATKPRDLPVPGRELSGVHFAMEFLHANTKSLLDSNLEDGNFISAKGKKVVVIGGGDTGTDCIGTSIRHGCSGLINLELLPQPPQSRAPNNPWPQWPRVFRIDYGHQEAAAKFGKDPRSYEVLTKRFVGDENGVVKGLEIVRVQWEKDPSGKFQFKEVEGSEEMIEADLVLLAMGFLGPESTIADKLGLEKDNRSNLKADYGRFSTSVEGVFAAGDCRRGQSLVVWAISEGRQAAAQVDKYLMEDTTGLPIDGRRQEDFVKREQDSNRQTVRT

>evm.TU.Scaffold_637.1259|Carabica_v0.5/1-2216

MSVASGTVIPSKSGRGAVGLPSVTKPFANNQLNVVPLSRVVGIRSAAGRRSGCGLERSSKLFGARLPRALGSERSHLWQTDGPGRPPKLRVFQLRSALSQIPEKPLGLYDPSFDKDSCGVGFVAELSGESSRKTVTDAIEMLVRMSHRGACGCETNTGDGAGILVALPHDFYKEVAKEMGFELPPPREYAVGMLFLPTSESRREQSKIVFTKVAESLGHTVLGWRTVPTDNSGLGKSALQTEPVVEQVFLTPTPRSKADFEQQMYILRRVSMVAIRAALNLQHGGVRDFYICSLSSRTVVYKGQLKPAQLKEYYYADLGNERFTSYMALIHSRFSTNTFPSWDRAQPMRVLGHNGEINTLRGNVNWMKAREGLLKCKELGLSKTEMKKLLPIVDASSSDSGAFDGVLELLVRAGRSLPEAIMMMIPEAWQNDKNMDPQRKALYEYFSALMEPWDGPALISFTDGRYLGATLDRNGLRPGRFYITHSGRVIMASEVGVVDIPPEDVSRKGRLNPGMMLLVDFEKHVVVDDEALKQQYSLARPYGEWLKRQKIELKDIVKSVHESQRVSPAIAGVVPASKDDDSMENMGIHGLLAPLKAFGYTVEALEMLLLPMAKDGIEALGSMGNDAPLAVMSNREKLTFEYFKQMFAQVTNPPIDPIREKIVTSMECMIGPEGDLTETTEEQCHRLSLKGPLLSIEEMEAIKKMDYRGWRSKVIDITYSVGHGRKGLEETLDRICSEAHDAIKEGYTALVLSDRAFSPKRVAVSSLLAVGAVHHHLVKRLERTRVALIVESAEPREVHHFCTLVGFGADAICPYLAIEAILRLQVDGKIPPKPTGQFHSKDELVKKYFNASNYGMMKVLAKMGISTLASYKGAQIFEAVGLSSEVMERCFTGTPSRVEGATFDALANDALELHELAFPSRVLPAGSAEAVALPNPGDYHWRKGGEIHLNDPLAIAKLQEAARSNSVAAYKEYSRRIQELNKSCNLRGLLKFKDEVMGVPLEEVEPASEIVKRFCTGAMSYGSISLEAHSTLAIAMNKIGGKSNTGEGGEQPSRMDPLPDGSMNPKRSAIKQVASGRFGVSSYYLTNADELQIKMAQGAKPGEGGELPGHKVIGDIAVTRNSTAGVGLISPPPHHDIYSIEDLAQLIHDLKNANPGARISVKLVSEAGVGVIASGVVKGHADHVLISGHDGGTGASRWTGIKSAGLPWELGLAETHQTLVANDLRGRTVLQTDGQLKTGRDVAIAALLGAEEFGFSTAPLITLGCIMMRKCHKNTCPVGIATQDPVLREKFAGEPEHVINFFFMLAEEVREIMSQLGFRTLIEMVGRSDMLELNEDLVKNNEKLKNIDLSLLLRPAADIRPDAAQYCIQKQDHSLDMAMDNKLIDLSKVGIEKCVPVYVETGICNTNRAVGTMLSHEVTKRHSMVGLPADTIHIKFNGSAGQSFGAFVCPGITLELEGDSNDYVGKGLSGGKIIVYPPRESRFDAKENIVIGNVALYGATGGEAYFNGMAAERFCVRNSGAKAVVEGVGDHGCEYMTGGTVVVLGKTGRNFAAGMSGGIAFVLDMDSKFQSHCNSELVDLDKVENEDDILTLKMMIQQHQRHTNSKLAKEVLSNFDDLLPKFIKVFPRDYKRVLASMKEKETANIAAERTAREIEEQEEAELMGKDAFEELKKFSAGSLNKKANQVERVISVKRPTCVADAEKNGGFVRYEREGISYRDPNKRIYDWKEVMEELKPGPLLTTQSARCMDCGTPFCHQENSGCPLGNKIPEFNELVYQNRWREALDRLLETNNFPEFTGRVCPAPCEGSCVLGIIENPVSIKSIECTIIDKAFEEGWMVPRPPQMRTGKRVAVVGSGPAGLAAADQLNRMGHAVTVFERADRIGGLMMYGVPNMKADKVDVVQRRVDLMEKEGINFVVNANVGKDPSFSLDRLREEHDAIVLAVGATKSRDLPVPGRELSGVHFAMEFLHANTKSLLDSNLEDGNFISAKGKKVVVIGGGDTGTDCIGTSIRHGCSGLINLELLPQPPQSRAPNNPWPQWPRVFRIDYGHQEAAAKFGKDPRSYEVLTKRFVGDENGVVKGLEIVRVQWEKDPSGKFQFKEVEGSEEMIEADLVLLAMGFLGPESTIADKLGLEKDNRSNFKADYGRFSTSVEGVFAAGDCRRGQSLVVWAISEGRQAAAQVDKYLMEDTTGLPIDGRRQEDIVKREQDSNRQTVRT

>CKAN_02252300|Ckanehirae_v3/1-2203

MASIPGSAFQLRNDPKMLPCINQPRNISAAFNRHRNQSMLCSSSRRTGAVSLEKRFLGARLRQSGSERLHLWRSDGPCRSPKLRLVVRSMGLSQVPEKPLGLYDPSFDKDSCGVGFIAELSGESSRKTVMDAVEMLTRMTHRGACGCETNTGDGAGILVALPHDFFSEVAKDVGFELPPSGEYAVGMIFLPTSEVRRHESKVVFTKVAESLGHVVLGWRSVPTDNSGLGQSAMQTEPVVEQVFLTPTARSNVDFEQQMYILRRVSMVAIRAALNLQHGGVKDFYICSLSSRTVVYKGQLKPEQLKDYYYADLGHDRFTSYMALVHSRFSTNTFPSWDRAQPMRILGHNGEINTLRGNVNWMKAREGLLKCKELGLSKNEMKKLLPIVDSSSSDSGAFDGVLEFLLRAGRSLPEAMMMMIPEAWQNDKNMDPDRKAFYEYFSALMEPWDGPALISFTDGRYLGATLDRNGLRPGRFYITHSGRVIMASEVGVVDIPPEDVSRKGRLNPGMMLLVDFENHIVVDDEALKKQYSLARPYSEWLGRQKIELKDILNSVPETERVPPAISGTVAAFSHDDNMENMGIHGLLAPLKAFGYTVEALDMLLLPMAKDGTEALGSMGNDAPLAVMSNREKLTFEYFKQMFAQVTNPPIDPIREKIVTSMECMIGPEGDLTETTEEQCHRLSLKGPLLSIEEMEAMKKMNYRGWRSKVLDITYSKDRGRKGLEETLDRICAEAHNAIREGYTTLVLSDRAFSSKRVAVSSLLAVGAVHHHLVSKLERTRIGLMVESAEPREVHHFCTLVGFGADAICPYLAVEAIWRLQIDGKVPPKANGEFHSKEDLVKKYFKASNYGMMKVLAKMGISTLASYKGAQIFEALGLSSDVIQRCFNGTPSRVEGATFEMLAQDALRLHELAFPSRDLPVGSAEAVALPNPGDYHWRKGGEVHLNDPLAIAKLQEAARGNSVAAYKEYSRRIQELNKTCNLRGMLKFKDTKAKIPLDEVEPASEIVKRFCTGAMSYGSISLEAHTTLALAMNKIGGKSNTGEGGENPSRMEPLPNGSMNPKRSAIKQVASGRFGVSSYYLTNADELQIKMAQGAKPGEGGELPGHKVIGDIAVTRNSTAGVGLISPPPHHDIYSIEDLAQLIYDLKNSNPGARISVKLVSEAGVGVIASGVVKGHADHVLISGHDGGTGASRWTGIKNAGLPWELGLAETHQTLVANDLRGRTTLQTDGQLKTGRDVAIAALLGAEEFGFSTAPLITLGCIMMRKCHKNTCPVGIATQDPVLREKFAGEPEHVINFFFMLAEEVREIMSQLGFRTINEMVGRSDMLEIDKEVVKNNEKLENIDLTLLLRPAADIRPDAAQYCIQKQDHGLDMALDRKMISLSKAALEKSLPVYIEMPIRNVNRAVGTMLSHEVTKRYHMNGLPKDTIHIRFTGSAGQSLGAFLCPGITLELEGDSNDYVGKGLSGGKIVVYPPRKSKFDPKENIVIGNVALYGATCGEAYFNGMAAERFCVRNSGARAVVEGVGDHGCEYMTGGIVVVLGKTGRNFAAGMSGGIAYVLDLEGKFPSRCNQELVDLDKVEEEEDITTLRVMIQQHQRHTNSVLASEVLANFETLLPKFLKVFPRDYKRVLKNMEAEKAALEAEAKEEAELMREDAFEELKKMAAASSANKVVELKQEDTQQLKRPTRVPDAIKHGGFIAYERESISYRDPNSRINDWKEVVEDTKPSQLLKTQSARCMDCGTPFCHQENSGCPLGNKIPEFNELVYQNRWREALDRLLETNNFPEFTGRVCPAPCEGSCVLGIIENPVSIKSIECSIIDKAFEEGWMVPRPPLARTGKRVAIVGSGPAGLAAADQLNKMGHLVTVYERADRIGGLMMYGVPNMKTDKVNIVQRRVDLMAEEGIKFVVNANVGVDPEYSLENLRVENDAFVLACGATKPRDLPVPGRELSGVHFAMEFLHANTKSLLDSDLQDGKYISAKGKKVVVIGGGDTGTDCIGTSIRHGCTQIVNLELLPEPPRTRAAGNPWPQWPRIFRVDYGHQEAAAKFGKDPRSYEVLTKRFVGDENGAVKGLEVVRVRWAKDASGKFQFKEVEGSEETIEADLVLLAMGFLGPESTVADKLGVERDNRSNFRAEYGRFLTNVDGVFAAGDCRRGQSLVVWAISEGRQAAAQVDKYLMEQQHQDVIINSREDVTDQQNVRRTVMTGK

>Litul.08G148700|Ltulipifera_YP108A_v1.1/1-2192

MAAIPGLAFQLNNSALLPSINHQRNLSPLSSHGSRATFCSVSRRSGAVSLENKFFGARLRCTGSERLHLWRSDGPGRSPKLRVVVRSMSLSQVPEKPLGLYDPSFDKDSCGVGFVAELSGEYSRKTVIDAIEMLVRMSHRGACGCETNTGDGAGILVALPHDFFAKVTKDVGFELPPPGEYAVGMFFLPTSEVRREESKTVFSKVAESLGHVVLGWRSVPTDNTGLGKSALQTEPVIEQVFLTPSPRSSADFEQQMYILRRVSMVAIRATLNLQHGGVRDFYICSLSSRTIVYKGQLKPDQLRDYYYADLGHERFTSYMALVHSRFSTNTFPSWDRAQPMRVLGHNGEINTLRGNVNWMTAREGLLKCKELGLSKNEMKKLLPIVDASSSDSGTFDGVLELLVRAGRSLPEAIMMMIPEAWQNDKNMDPSRKALYEYFSALMEPWDGPALISFTDGHYLGATLDRNGLRPGRFYITHSGRVIMASEVGVVDIPPEDVQRKGRLNPGMMLLVDFDKHIVVDDEALKKQYSLARPYGEWLSRQKIELKDIVNSVHGNDRVPPIISGTMPVHGHDDDMENMGIHGMLAPLKAFGYTVEALEMLLLPMAKDGTEALGSMGNDAPLAVMSNREKLTFEYFKQMFAQVTNPPIDPIREKIVTSMECMIGPEGDLTETTEEQCHRLSLKGSLLSIEEMEAIKKMNYRGWRSKVLDITYSKERGRKGLEETLDRICSEARDAIREGYTTLVLSDRAFSSKRVAVSSLLAIGAVHHHLVSKLERTRIGLIVESAEPREVHHFCTLVGFGADAICPYLAIEAIWRLQIDGKIPPKSDGEFHSREELVKKYFKASNYGMMKVLAKMGISTLASYKGAQIFEALGLSSEVIQRCFNGTPSRVEGATFEMLARDALRLHELAFPTRALPPGSAEAVALPNPGDYHWRKGGEVHLNDPLAIAKLQEAAKSNSVAAYKEYSKRIQELNKTCNLRGMLKFKDVEAKVPLDKVEPASEIVKRFCTGAMSYGSISMEAHSTLAIAMNKIGGKSNTGEGGENPSRMEPLPDGSMNPKRSAIKQVASGRFGVSSYYLTNADELQIKMAQGAKPGEGGELPGHKVIGDIAVTRNSTAGVGLISPPPHHDIYSIEDLAQLIHDLKNSNPGARISVKLVSEAGVGVIASGVVKGHADHVLISGHDGGTGASRWTGIKNAGLPWELGLAETHQTLVANDLRGRTVLQTDGQLKTGRDVAIAALLGAEEFGFSTAPLITLGCIMMRKCHKNTCPVGIATQDPVLREKFAGEPEHVINFFFMLAEEVREIMSQLGFRTVNEMVGRSDMLEMDEEVIKNNEKLENIDLTLLLRPAADIRPEAAQYCIQKQDHGLDMALDQKLTSLSETALEKGLPVYIEMPIRNVNRAVGTMLSHEVTKRYHMEGLPTDCIHVRLSGSAGQSLGAFLCSGITLELEGDSNDYVGKGLSGGKIVVYPPRKSQFDPKENIVIGNVALYGATLGEAYFNGMAAERFCVRNSGARAVVEGVGDHGCEYMTGGTVVVLGRTGRNFAAGMSGGIAYVLDVDGKFHSRCNLELVDLDKVEDEDDITTLRMMIQQHQRHTNSNLAREVLANFEDLLPKFVKVFPRDYKRVLQNLKVEQAASEAEAQEEVELMEKDAFEELKKLAAASMNGKEDMVEETEPLKRPTRVPDAVKHRGFLAYERESISYRDPNVRMNDWEEVAVESKPGQLLKTQSARCMDCGTPFCHQENSGCPLGNKIPEFNELVYQNRWREALDRLLETNNFPEFTGRVCPAPCEGSCVLGIIENPVSIKSIECSIIDKAFEEGWMVPRPPLKRTGKRVAIVGSGPAGLAAADQLNKMGHLVTVFERADRIGGLMMYGVPNMKADKVDVVQRRVDLMAKEGVNFVVNANVGTDPMFSVERLRAENDAIILACGATKPRDLPVPGREFSGIHFAMKFLHANTKSLLDSNLQDGKYISAKGKKVVVIGGGDTGTDCIGTSIRHGCTEIVNLELLPMPPQTRAPGNPWPQWPRVFRVDYGHQEAAAKFGKDPRFYEVLTKRFVGDENGAVKGLEVVRVHWAKDASGKFQFQEVEGSEEIIEADLVLLAMGFLGPELTVAAKLGLERDNRSNFKAEYGRFSTSVDGVFAAGDCRRGQSLVVWAISEGRQAASQVDKYLMEDHLNNAAAHQEAVENKYTVMT

>Litul.03G219100|Ltulipifera_YP108A_v1.1/1-2197

MAAVPGSVFQHHKDPAVLPSLNSQRNIAALSRCGNRASCCSVSRRSGAVTLENRFFGTRLRHSSAGRLHLWLSDGPGRAPKLRTVRSMALSQVPEKPLGLYDPSFDKDSCGVGFVAELSGEYSRKTIMDAIEMLVRMSHRGACGCETNTGDGAGILVALPHDFFNEVGKNVGFELPPPGEYAVGMFFLPTSEVRREESKIVFTKVAESLGHVVLGWRPVPTDNTGLGNSALQTEPVVEQVFLTPSPRSNADFEQQMYILRRVSMVAIRAALNLQYGGQKDFYICSLSSRTIVYKGQLKPDQLNGYYYADLGHEKFTSYMALVHSRFSTNTFPSWDRAQPMRILGHNGEINTLRGNVNWMKAREGLLKCKELGLSKNEMKKLLPIVDASSSDSGAFDGVLELLVRAGRSLPEAIMMMIPEAWQNDKNMDPDRKALYEYFSALMEPWDGPALISFTDGRYLGATLDRNGLRPGRFYITHSGRVIMASEVGVVDILPEDVARKGRLNPGMMLLVDFENHIVVDDEALKQQYSLARPYGEWLSRQKIELKDIVNSVPEADRVPPTISGTVPVHRHDDNNMENMGIHGLLAPLKAFGYTVEALEMLLLPMAKDGTEALGSMGNDAPLAVMSNREKLTFEYFKQMFAQVTNPPIDPIREKIVTSMECMIGPEGDLTETTEEQCHRLSLKGPLLSIEEVEAMKKMNYRGWRSKVLDITYSKNRGRKGLEETLDRICAEARNAIREGYTTLILSDRAFSSKRVAVSSLLAVGAVHHHLVSKLERTRVGLIVESAEPREVHHFCTLVGFGADAICPYLAAEAIWRLQIDGKIPPKANGDFYSREDLVKKYFKASNYGMMKVLAKMGISTLASYKGAQIFEALGLSSEVIERCFNGTPSRVEGATFEMLARDALRLHELAFPSRVLPLGSAEAVALPNPGDYHWRKGGEVHLNDPLAIAKLQEAARANSVAAYKEYSKRIQELNKTCNLRGMLKFKDIKEKVPLDEVEPASEIVKRFCTGAMSYGSISLEAHTTLAIAMNQIGGKSNTGEGGENPSRMESLPDGSRNPKRSAIKQVASGRFGVSSYYLTNADELQIKMAQGAKPGEGGELPGHKVIGDIAVTRNSTAGVGLISPPPHHDIYSIEDLAQLIHDLKNSNPGARISVKLVSEAGVGVIASGVVKGHADHVLISGHDGGTGASRWTGIKNAGLPWELGLAETHQTLVANDLRGRTTLQTDGQLKTGRDVAIAALLGAEEFGFSTAPLITLGCIMMRKCHKNTCPVGIATQDPVLREKFAGEPEHVINFFFMLAEEVREIMSQLGFRTIYEMIGRSDMLEIDNEVVKNNEKLENIDLTLLLRPAADIRPEAAQYCIQKQDHGLDMALDQELISLSKAALEKGLPVYIEMPIRNVNRAVGTMLSHEVTKRYHMNGLPTDTIHVRLTGSAGQSLGAFLCPGITLELEGDSNDYVGKGLSGGKIVVYPPRRSQFDPKENIVIGNVALYGATSGEAYFNGMAAERFCVRNSGARAVVEGVGDHGCEYMTGGTVVVLGKTGRNFAAGMSGGIAYVLDMDEKFHSCCNVELVDLDKVEDEDDITTLRMMIQQHQRHTNSEFAREVLADFENFLPKFVKVFPRDYKRVLEELKAEKAANEAEAQEEAELMKKDAFEELKKLAAASSNGKANVKVEQTQSSKRPTRVPDAVKHRGFLAYERESISYRDPNERINDWEEVAEESRPSQLLNTQSARCMDCGTPFCHQENSGCPLGNKIPEFNELVYQNRWREALDRLLETNNFPEFTGRVCPAPCEGSCVLGIIENPVSIKSIECAIIDKAFEEGWMVPRPPPKRTGKRVAIVGSGPAGLAAADQLNRMGHLVTVFERADRIGGLMMYGVPNMKADKVDVVQRRVNLMAEEGINFVVNANVGSDPLYSLDHLRAENDAIVLACGATKPRDLPVPGRELSGIHFAMEFLHGNTKSLLDSNLQDGKYISAKGKKVVVIGGGDTGTDCIGTSIRHGCTNIVNLELLPEPPRTRAPGNPWPQWPRVFRVDYGHQEAAAKFGKDPRDYEVLTKRFVGDENGTLKGLEVVRVRWAKDASGRFQFQEVEGSEEMIEADLVLLAMGFLGPEPTVADTLGLERDNRSNFKAEYGRFSTNVEGVFAAGDCRRGQSLVVWAISEGRQAATQVDKYLMDDVSITAPSQEDMVKKQGSKHTVMT

>GWHPBFSI014333|Chloranthus_spicatus/1-2198

MAAIPGSAFQLRTDSAFPSLNHQTRNLVAFSCRGRRGSACSLTTRRQQTSSLKLENSILGPQFRLHLWRSDGPGRSPNLRVVSASMGFSQVPEKPLGLYDPAFDKESCGVGFVAELSGECSRVTVTDAIEMLVRMAHRGACGCETNTGDGAGILVALPHDFLIEVTKDVGFELPPPGEYAVGMFFLPQSPSRREESKIVFNKVAESLGHVVLGWRSVPTDNTGLGNSAIQTEPIVEQVFLTPSPRSNADFEQQMYILRRVSMVAIRAALNLQYGGVKDFYICSLSSRTIVYKGQLKPDQLKGYYYADLGDERFTSYMALIHSRFSTNTFPSWDRAQPMRVLGHNGEINTLRGNVNWMKAREGLLKCKELGLSKNEMKKLLPIVDASSSDSGAFDGVLELLVRAGRSLPEAMMMMIPEAWQNDKNMDPAKKALYEYFSALMEPWDGPALISFTDGRYLGATLDRNGLRPGRFYVTHSGRVIMASEVGVVDIPPEDVCRKGRLNPGMMLLVDFEQHIVVDDEALKSRYSLARPYGEWLNRQRIELKDIVNSVHETDRIPPIIPGTMPAHSQDNSMENMGIHGLLAPLKAFGYTVEALEMLLLPMAKDGTEALGSMGNDAPLAVMSNREKLTFEYFKQMFAQVTNPPIDPIREKIVTSMECMIGPEGDLTETTEEQCHRLSLKGPLLSIEEMEAIKKMNYRGWRSKVLDITYSKSRGRKGLEETLDRICIEARTSIREGYTTLVLSDRAFSSKRVAVSSLLAVGAVHHHLVSKLERTRIGLIVESAEPREVHHFCTLVGFGADAICPYLAIETIWRLQVDGKIPPKANSKFHSKEDLVRKYFKASNYGMMKVLAKMGISTLASYKGAQIFEALGLSSEVIQKCFNGTPSRVEGATFEMLARDALCLHELAFPSRVLPLGSAEAMALPNPGDYHWRKGGEVHLNDPLAISKLQEAARTNSVGAYKEYSKRIQELNKSCNLRGMLKFKDGMEKIHLDEVEPASEIVKRFCTGAMSYGSISLEAHTTLAIAMNKIGGKSNTGEGGENPSRMEPLPNGSINPKRSAIKQVASGRFGVSSYYLTNADELQIKMAQGAKPGEGGELPGHKVIGDIAVTRNSTAGVGLISPPPHHDIYSIEDLAQLIHDLKNSNPGARISVKLVSEAGVGVIASGVVKGHADHVLIAGHDGGTGASRWTGIKNAGLPWELGLAETHQTLVANDLRGRTVLQTDGQLKTGRDVAIAALLGAEEFGFSTAPLITLGCIMMRKCHKNTCPVGIATQDPVLREKFAGEPEHVINFFFMLAEEVREIMSHLGFRTINEMVGRSDMLEVDKEVVKNNEKLENIDLTLLLRPAADIRPDAAQYCMQKQDHGLDMALDQQLISLSKSAIERSLPVYIEMPIRNVNRAVGTMLSHEVTKLYHMNGLPSDTVHIKLTGSAGQSLGAFLSPGITLELEGDSNDYVGKGLSGGKIVVYPPRKSLFDPKENIVIGNVALYGATSGEAYFNGMAAERFCVRNSGARAVVEGVGDHGCEYMTGGTVLVLGKTGRNFAAGMSGGVAYVLDVDEKFYSRCNLELVDLDKVEEEEDIATLRMMIQQHQRHTSSELAREVLADFENLLPKFVKVFPRDYKRILQNMKAEKAVKEAEEREEAELMKKDAFEELKKLAAASSNGKPDQHVQETQPMMRPTRVSDAVKHRGFLAYERESISYRDPKVRINDWEEVAEESKPGQLLTTQSARCMDCGTPFCHQENSGCPLGNKIPEFNELVYQNRWREALDRLLETNNFPEFTGRVCPAPCEGSCVLGIIENPVSIKSIECSIIDKAFEEGWMVPRPPLTRTGKRVAIVGSGPAGLAAADQLNRMGHLVTVLERADRIGGLMMYGVPNMKADKVNIVQRRVDLMAKEGINFVVNANVGTDPMYSFDRLRSENDAIVLACGATKPRDLPVPGRDLSGVHFAMEFLHANTKSLLDSDLEDEKYISAKGKKVVVIGGGDTGTDCIGTSIRHGCTNIVNLELLPEPPRTRAPGNPWPQWPRIFRVDYGHQEAATKFGKDPRSYEVLTKQFIGDDNGAVKGLEVIRVHWAKDASGKFQFEEIKGSEEIIEADLVLLAMGFLGPEPAVADRLGLERDNRSNFKAEYGRFSTNVEGVFAAGDCRRGQSLVVWAISEGRQAACQVDKYLMPEKDLDVTANQEESDLIKKDSKHTVMTAT

>GWHPBAUK002248.1|Macadamia_integrifolia/1-2208

MSALSGSALQLRIKSGTFPSIHGSPSNHQCNITPLGSHGKKISSCFATKRQNAMENTFFGTRLRSYTPERLHLWRSDGPGRQPKLKVVVRSALSQVPEKPLGLYDPSFDKDSCGVGFVAELSGDSSRRTVTDAIEMLVRMTHRGACGCETNTGDGAGILVDLPHSFFTEVAKDVGFELPPLGGYAVGMFFLPTSETRREESKIVFTKVAESLGHVVLGWRSVPTDNTGLGKAALQTEPVIEQVFITPSPRSKATFEQQMYILRRVSMVAIRAALNLQHGGVKDFYICSLSSRTIVYKGQLKPDQLKDYYYADLGNESFMSYMALIHSRFSTNTFPSWDRAQPMRVLGHNGEINTLRGNVNWMKAREGLLKCKELGLSKNEMKKLLPIVDASSSDSGAFDGVLELLVRAGRSLPEAVMMMIPEAWQNDKNMDPDRKALYEYFSALMEPWDGPALISFTDGRYLGATLDRNGLRPGRFYVTHSGRVIMASEVGVVDIPPEDVLRKGRLNPGMMLLVDFEKHIVVDDEALKKQYSLARPYGEWLSRQKIELKNIVDSVPESDRVPPAISGAVPVSGQDENMEKMGIHGLLAPLKAFGYTVEALEMLLLPMAKDGTEALGSMGNDTPLAVMSNREKLTFEYFKQMFAQVTNPPIDPIREKIVTSMECMIGPEGDLTETTEEQCCRLSLKGPLLSTEEMEAIIKMNYRGWRSKVLDITYSKSRGRRGLEETLDRICLEARNAIKEGYTTLVLSDRAFSSNRVAVSSLLAVGAVHHHLVSKLERTRIGLVVESAEPREVHHFCTLVGFGADAICPYLAIEAIWRLQVDGKIPPKANGEFHSKEDLVKKYFKASNYGMMKVLAKMGISTLASYKGAQIFEALGLSSEVIQKCFNGTPSRVEGATFEVLAQDALRLHEIAFPARVLPPGSSEAVALPNPGDYHWRKDGEVHLNDPLAMAKLQEAAKSNSVAAYKEYSKRIQELNKTCNLRGMLKFKESEFKVPLDEVEPASEIVKRFCTGAMSYGSISLEAHTTLAIAMNRIGGKSNTGEGGENPSRMQPLPEGSMNPKRSAIKQVASGRFGVSSYYLTNADELQIKMAQGAKPGEGGELPGHKVIGDIAVTRNSTAGVGLISPPPHHDIYSIEDLAQLIHDLKNSNPGARISVKLVSEAGVGVIASGVVKGHADHVLISGHDGGTGASRWTGIKNAGLPWELGLAETHQTLVANDLRGRTTLQTDGQLKTGRDVAIAALLGAEEFGFSTAPLITLGCIMMRKCHKNTCPVGIATQDPVLREKFAGEPEHVINFFFMLAEEVREIMSQLGFRNITEMVGRSDMLEVDKEVTRNNEKLENIDLSLLLRPAADIRPEAAQYCTQKQDHGLDMALDQKLISLSQSALDKGLPVYIEMPILNVNRAVGTMLSHEVTKRYHMAGLPTDTIHIKFSGSAGQSLGAFLSSGIMLELEGDSNDYVGKGLSGGKIVVYPPRESNFDPKENIVIGNVALYGATSGEAYFNGMAAERFCVRNSGARAVVEGVGDHGCEYMTGGIVVVLGKTGRNFAAGMSGGIAYVLDVDKKFQSRCNLELVDLDNVEDEEDIMTLRMLIQQHQRHTNSKLAREVLADFENLLPKFIKVFPRDYKWILANMKAEKANREAEERAATEAEEQKEAELMEKDAFEELKKLAAASSNGSASQAAQEEPVKRPTRVADAIKHRGFVAYERESVSYRDPNARISDWKEVMEESKPGELLKTQSARCMDCGTPFCHQENSGCPLGNKIPEFNELVYQNRWREALDRLLETNNFPEFTGRVCPAPCEGSCVLGIIENPVSIKSIECSIIDRAFEEGWMVPRPPPVRTGKRVAVVGSGPSGLAAADQLNKMGHLVTVFERSDRIGGLMMYGVPNMKADKMEIVQRRVNLMAEEGVNFIVNANVGTDPLYSIERLRAKNDAIVLALGATKPRDLPVPGRELTGVHFAMEFLHANTKSFLDSDLQDGNYISAKGKKVVVIGGGDTGTDCIGTSIRHGCSSIVNLELLPQPPQTRAPGNPWPQWPRVFRVDYGHQEAAAKFGKDPRSYEVLTKRFIGDENGAVTGLELVRVNWEKDATGRFQFKEVEGSEEIIEANLVLLAMGFLGPESTIADQIGLERDNRSNLKAEYGRFSTNAEGVFAAGDCRRGQSLVVWAIAEGRKAATEVDKYLMKGEDEDLSIATDNEDVVKKNSKFTVVT

>Asa0G00877.1|Allium_sativum/1-1497

MDYRGWRSKVVDITYSKIRGRKGLEEALDRICSEARTAISDGYTTLVLSDRGFSSERVPVSSLLAVGAVHQHLVSKLERTRIGLIIESAEPREVHHFCTLVGFGADAICPYLAIEAISRLQIDGKIPPKKDGTFHTQEELVKKYFKASYYGMMKVLAKMGISTLASYKGAQIFEALGLSSEVIQKCFNGTSSRVEGATFEMLAKDALRLHELAFPKRSLPPGSAEAEALPNPGDFHWRKGGEVHLNDPLAIAKLQEAARQNSVTAYKEYSRRIQELNKSCNLRGMLKFKDVKENISLDEVEPASEIVKRFCTGAMSYGSISLEAHTTLAIAMNRLGGKSNTGEGGEQPSRMEPLPDGSMNLKRSAIKQVASGRFGVSSYYLTNADELQIKMAQGAKPGEGGELPGHKVIGDIAVTRNSTAGVGLISPPPHHDIYSIEDLAQLIYDLKNSNPGARVSVKLVSEAGVGVVASGVVKGHADHVLISGHDGGTGASRWTGIKSAGLPWELGLAETHQTLVANDLRGRTTLQTDGQLKTGRDVVIAALLGAEEFGFSTAPLITLGCIMMRKCHKNTCPVGIATQDPVLREKFAGEPEHVINFFFMLAEEVREIMAQLGFRTLTEMVGRADVLEIDPDVVKNNEKLKNIDLSLLLKPAAEIRPDAAQYCVQKQDHDLDAALDQKLISLSKASLEKGLPVYIETPVRNVNRAVGTMLSHEVTKRYRMDGLPSDTIHIKLFGSAGQSLGAFLCPGITLELEGDSNDYVGKGLSGGKIVVYPPRESQFDPKENIVIGNVALYGAIKGEAYFNGMAAERFSVRNSGAKAVVEGVGDHGCEYMTGGIVVILGKTGRNFAAGMSGGLAYVYDVDGKFHTRCNAELADLDKVEEEEDITTLRMMIQQHQRHTSSVLAKEILSDFDNLVSKFVKVFPRDYKRVLKNLKADQAALEAKKKEEAELMKKDAFEELKTLAASNGKSSMVVKNLKVQDRPTEVPNAIKHRGFISYERQGVSYRDPNARISDWKEVAVESKPGPVLKTQSARCMDCGTPFCHQDNTGCPLGNKIPEFNELVHQNRWREALDRLLETNNFPEFTGRVCPAPCEGSCVLGIIENPVSIKSIECAIIDKAFEEGWMVPRPPAHRSGKRVAIVGSGPAGLAAADQLNKMGHSVTVYERADRIGGLMMYGVPNMKADKTDVVQRRVDLLAKEGIKFVVNASVGTDPAYSIEKLRSENNAIILACGATKPRDLPVPGRELSGVHFAMEFLHANTKSLLDSNLEDGKYISAKGKKVVVIGGGDTGTDCIGTSIRHGCTDIVNLELLPEPPRTRAPGNPWPQWPRIFRVDYGHQEATTKFGKDPRSYEVLTKRFIGDENGAVKGLEVVRVKWGKDNSGRFQFEEIKGSEEIIEAGLVLLAMGFLGPESTIAEKLGLEQDPRSNFKAEYGKFSTSIDGIFAAGDCRRGQSLVVWAISEGRQAAAQVDKYLMKEESENVGLKKDELVRSLESMHKA

>Asa2G00186.1|Allium_sativum/1-507

MRYERQGVSYRDPHTRINDWKEVAEESKPGPLLKTQAARCMDCGTPFCHQDNSGCPLGNKIPEFNDLVYQNRWREALDRLLETNNFPEFTGRVCPAPCEGSCVLGIIENPVSIKSIECSIIDKAFEEGWMVPRPPIQRSGKKVAIVGSGPAGLAAADQLNKMGHMVTVYERSDRIGGLMMYGVPNMKADKTDIVQRRVDLMAKEGIKFVVNANVGKDPAYSIGTLRMENDAIVLACGATKPRDLPVPGREHSGIHFAMEFLHANTKSLLDSNLQDGKYISAKGKKVVVIGGGDTGTDCIGTSIRHGCTDIVNLELLPEPPLTRAPGNPWPQWPRVFRVDYGHQEATAKFGKDPRSYEVLTKRFIGDENGAVKGLEVIRVKWGKDNSGKFQFEEIKGSEEIIEADLVLLAMGFLGPEPTIADKLGLERDPRSNFKAEYGRFSTNIDGVFAAGDCRRGQSLVVWAISEGRQAASQVDKFLMEEEDEVTEITINQERFLGNFESKHKIAT

>Acora.07G236000|Aamericanus_v1.1/1-2195

MAAISGAAVRFRSDPVGPPSVRRSGAVGSIRAARCPLPMRRPAAVSLESRFLFGRRIRASAPERRLQLWRSDGPGRSPRLRVVGPSMALSQVPEKPLGLYDPSFDKDSCGVGFVAELSGVCSRKTVTDGIEMLRRMAHRGACGCETNTGDGAGILTALPHDFFAEVTKDVGIELPPPGEYAVGMFFLPTAEGRREESKTVFTKVAESLGHIVLGWRSVPTDNTDLGKSAIQTEPVIEQVFLTPSPRSTADFEQQMYILRRVSMVAIRAALNLQHGGVKDFYICSLSSRTIVYKGQLKPDQLKGYYYGDLDNERFTSYMALVHSRFSTNTFPSWDRAQPMRVLGHNGEINTLRGNVNWMKAREGLLKCKELGLSKNEMKKLLPIVDASSSDSGAFDGVLELLVRAGRSLPEAVMMMIPEAWQNDENMDPERKALYEYFSALMEPWDGPALISFTDGRYLGATLDRNGLRPGRFYITHSGRVIMASEVGVVDIPPEDVSRKGRLNPGMMLLVDFEKHIVVDDEALKKQYSLARPYGEWLDRQKIELKDIMDSVPASDRIPPTVAGVVAVESHDDSMENMGIHGLLAPLKAFGYTVEALEMLLLPMAKDADEALGSMGNDTPLAVMSNREKLTFEYFKQMFAQVTNPPIDPIREKIVTSMECMIGPEGDLTETTEEQCNRLVLKGPLLSIEEMEAVKKMNYRGWRSKVLDITYDKKRGRKGLEETLDRICSEARAAIRDGYTTLVLSDRGFSPERVAVSSLLAVGAVHQHLVSKLERTRIGLVVESAEPREVHHFCTLVGFGADAICPYLAIETVWRLQVDGKIPPKADGELFSREDLVKRYFKASSYGMMKVLAKMGISTLASYKGAQIFEALGLSSEVIQKCFNGTPSRVEGATFEMLAGDALRLHELAFPNRTLPPGSAEAVALPNPGDYHWRKGGEVHLNDPLAMAKLQEAARSNSVAAYREYSKRVQELNQTCNLRGILKFKDVKEKIPLEEVEPASEIVKRFCTGAMSYGSISLEAHTTLAIAMNKIGGKSNTGEGGENPSRMEPLPDGSRNQKRSSIKQVASGRFGVSSYYLTNADELQIKMAQGAKPGEGGELPGHKVIGDIAVTRNSTAGVGLISPPPHHDIYSIEDLAQLIHDLKNSNPGARISVKLVSEAGVGVIASGVVKGHADHVLISGHDGGTGASRWTGIKNAGLPWELGLAETHQTLVANDLRGRTVLQTDGQLKTGRDVAVAALLGAEEFGFSTAPLITLGCIMMRKCHKNTCPVGIATQDPVLREKFAGEPEHVINFFFMLAEEVREIMSQLGFRTVIEMVGRSDMLEVDKNVVKNNEKLENIDLSLLLRPAADIRPDAAQYCIQKQDHGLDMALDQKLISLSKSALQKSLPVYVEMAVRNVNRAVGTMLSHEVTKRYKMNGLPGDTIHIKLNGNAGQSLGAFLCPGITLELEGDSNDYVGKGLSGGKIVVYPPRGSQFDPKENIVIGNVALYGATSGEAYFNGMAAERFAVRNSGAKAVVEGVGDHGCEYMTGGTVVVLGKTGRNFAAGMSGGIAYVLDVDDDFFSRCNHELVDLDKVEEEEDIMILRMMIQQHQRHTGSELASDILSNFDNLLHKFVKVFPRDYKRVLGEMKAEKAAEEAKEQEEQELMKKDAFEELKKLAAATLKNKVDLKVDSKPKRPTRISDAVKHRGFINYERESISYRDPNTRVNDWKEVAEESKPGPLLKTQSARCMDCGTPFCHQENSGCPLGNKIPEFNELVHQNRWREALDRLLETNNFPEFTGRVCPAPCEGSCVLGIIENPVSIKSIECSIIDKAFEEGWMVPRPPLKRTGKRVAIVGSGPAGLAAADQLNRMGHWVTVFERSDRIGGLMMYGVPNMKADKINIVQRRVNLMSEEGINFVVNANVGTDSLYSIDKLHAENDAIVLACGSTKPRDLPVPGREFAGIHFAMEFLHANTKSLLDSHLQDGNYISAKGKKVVVIGGGDTGTDCIGTSIRHGCTNIVNLELLPEPPKSRAPGNPWPQWPRVFRVDYGHQEATTKFGKDPRSYEVLTKRFIGDENGVVKGLEVVRVHWAKDASGRFQFTEVEGSEEIIEADLVLLAMGFLGPEPTIADKLGLEKDNRSNFKAEYGRFSTNVEGVFAAGDCRRGQSLVVWAISEGRQAASQVDKYLMKEEEAIDVTPNQDEMFEKKDNRYTVTA

>Acora.02G107600|Aamericanus_v1.1/1-2193

MAAVPGSVFRARNEPIVAVGSGRREIGTARCPLPMRRRSVAVSLENWFLSGRRLRASVPERRLQLWRSSGPGRLPRLRLVGPTMALSQLPEKPLGLYDPAFDKDSCGVGFVAELSGEYNRKTVTDAIEMSRRMSHRGACGCETNTGDGAGILTALPHDFFEEAAKDVGIELPPPGEYAVGMFFLPTSEGRREESKIVFTKVAESLGHVVLGWRPVPTDNTGLGKSAIQTEPVIEQVFLTPSPRSTADFEQQMYILRRVSMVAIRAALNLQYGGVKDFYICSLSSRTIVYKGQLKPDQLKDYYADLGNERFTSYMALVHSRFSTNTFPSWDRAQPMRILGHNGEINTLRGNVNWMKAREGLLKCKELGLSKNEMKKLLPIVDASSSDSGAFDGVLELLVRAGRSLPEAIMMMIPEAWQNDENMDPERKALYEYFSALMEPWDGPALISFTDGRYLGATLDRNGLRPGRFYITHSGRVIMASEVGVVDIPPEDVSRKGRLNPGMMLLVDFEKHIVVDDEALKKQYSLARPYGEWLDRQKIELKDIVDSVPVTDRIPPTVTGTVAVESHDNNMENMGIHGLLAPLKAFGYTVEALEMLLLPMAKDADEALGSMGNDAPLAVMSNREKLTFEYFKQMFAQVTNPPIDPIREKIVTSMECMIGPEGDLTETTEEQCHRLVLKGPLLSIEEMEAIKKMNYRGWRSKVLDITYDKKRGRKGLEETLDRICVEARTAIRDGYTTLVLSDRGFSRERVAVSSLLAVGAIHQHLVSKLERTRIGLVVESAEPREVHHFCTLVGFGADAICPYLAIETIWRLQIDGKIPPKVDGEFFSKQDLVKRYFKASNYGMMKVLAKMGISTLASYKGAQIFEALGLASEVIQKCFNGTPSRVEGATFEMLAGDALLLHELAFPRRTLPPGSAEAVALPNPGDYHWRKGGTVHLNDPLAIAKLQEAARSNSVAAYREYSKRIQELNKTCNLRGILKFKDVEEKIPLEEVEPASEIVKHFCTGAMSYGSISLEAHTTLAIAMNKIGGKSNTGEGGENPSRMEPLPDGSRNQKRSAIKQVASGRFGVSSYYLTNADELQIKMAQGAKPGEGGELPGHKVIGDIAVTRNSTAGVGLISPPPHHDIYSIEDLAQLIHDLKNSNPGARISVKLVSEAGVGVIASGVVKGHADHVLISGHDGGTGASRWTGIKNAGLPWELGLAETHQTLVANDLRGRTVLQTDGQLKTGRDVAIAALLGAEEFGFSTAPLITLGCIMMRKCHKNTCPVGIATQDPILREKFAGEPEHVINFFFMLAEEVREIMSQLGFRTIKEMVGRSDMLEVDKDVVKGNEKLDNIDLSLLLRPAADIRPDAAQYCIQKQDHGLDMALDQKLISLSRAALERSLPVYIEIGIRNVNRAVGTMLSHEVTKRYKMNGLPLDTIHIKLNGNAGQSLGAFLCPGITLELEGDSNDYVGKGLSGGKIVVYPPRGSQFDPKENIVIGNVALYGATNGEAYFNGMAAERFAVRNSGAKAVVEGVGDHGCEYMTGGTVVVLGKTGRNFAAGMSGGIAYVLDADDNFCSRCNHELVDLDKVEEEEDIMILQMMIQQHQRHTGSELASDILLNFDNLLPKFVKVFPRDYKWVLGNMKAEKAAEEAKEQEEHELTKKDAFEELKKLAAASIMKDKVDLEVDSKPKRPTKISDPIKHRGFINYERESISYRDPNIRVNDWEEVAEGSKPGPLLKTQSARCMDCGTPFCHQDNSGCPLGNKIPEFNELVHQNRWREALDRLLATNNFPEFTGRVCPAPCEGSCVLGIIENPVSIKSIECSIIDKAFEEGWMVPRPPLKRTGKRVAIVGSGPAGLAAADQLNRMGHWVTVFERADRIGGLMMYGVPNMKADKINIVQRRVNLMSEEGVDFVVNANIGTDPLYSLDKLRAENDAIVLACGSTKPRDLPVPGRDLSGIHFAMEFLHANTKSLLNSHLQDGNYISAKGKKVIVIGGGDTGTDCIGTSIRHGCTNIVNLELLPEPPKSRAPGNPWPQWPRVFRVDYGHQEAAAKFGKDPRSYEVLTKRFIGDANGEVKGLELVRVNWAKDASGRFQFTEVEGSQEIIEADLVLLAMGFLGPELTIADQLGLEKDNRSNFKAEFGRFSTNVEGVFAAGDCRRGQSLVVWAINEGRQAASQVDKYLMKEEEEVVDVIPIQGDIFGKKDDRYSVTA

>evm_27.TU.AmTr_v1.0_scaffold00021.277|Atrichopoda_v1.0/1-2201

MATVPGFVLPVNNSVILPSLKAQKGLVAPSSRRNSVFCRSVLKQNAREVRSIEKKFLGTRVRSGSERLHLWRSEGPGRTPKLRTVVKSMLSGVPTERLGLYDPSFDKDSCGVGFVAELSGEYSRKTVLDALEMLVRMSHRGACGCETNTGDGAGLLVGLPHQFFSEVAKESGFELPPPGEYAVGMFFLPTSEVRCEESKIVFAKVAESLGHVVLGWRRVPTDNTGLGKSALQTEPVIEQVFLTPSSRSNADFEQQMYILRRVSMVAIRAALNLQHGGVRDFYICSLSSRTVVYKGQLKPVQLKDYYYVDLGHEKFTSYMALIHSRFSTNTFPSWDRAQPMRVLGHNGEINTLRGNVNWMKAREGLLKCKKLGLSKNEMQKLLPIVDASSSDSGAFDGVLELLVRAGRSLPEAIMMMIPEAWQNDNNMDPERKALYEYFSALMEPWDGPALISFTDGRYLGATLDRNGLRPGRFYITHSGRVIMASEVGVVDIPPEDVRQKGRLNPGMMLLVDFENHTVVDDEALKKQYSLARPYAEWLSRQKIELKDIVESVSENDRVPPPINGVALADSHDDNMENMGIHGLLAPLKSFGYTVEALEMLLLPMAKDGTEALGSMGNDAALAVMSNREKLTFEYFKQMFAQVTNPPIDPIREKIVTSMECMIGPEGDLTETTEEQCHRLSLKGPLLSIDEMEAIKKMEYRGWCSKVLDITFSKDRGRKGLEETLDRICSEARAAIREGYTTLVLSDRAFSSKRVAVSSLLAVGAVHHHLVSKLERTQVGLIVESAEPREVHHFCTLVGFGADAICPYLAIEAICRLQIDGKIPPKSNGEFHSKEDLIKKYFKASNYGMMKVLAKMGISTLASYKGAQIFEALGLSSEVIQRCFAGTPSRVEGATFEILARDTLRLHEMAFPSRSLPQGSAEAVALPNPGSYHWRKGGEVHLNDPLAIAKLQEAARMNSVAAYKEYSRVVNELNKSCNLRGMLKFKKADSKIPLNEVEPASEIVKRFCTGAMSYGSISLEAHTALAIAMNKIGGKSNTGEGGEKPSRMEPLPDGSMNPMRSAIKQVASGRFGVSSYYLTNADELQIKMAQGAKPGEGGELPGHKVVGDIAVTRHSTAGVGLISPPPHHDIYSIEDLAQLIHDLKNSNPGARISVKLVSEAGVGVIASGVVKGHADHVLISGHDGGTGAARWTGIKNAGLPWELGLAETHQTLVANDLRGRTVLQTDGQLKTGRDVAIAALLGAEEFGFSTAPLITLGCIMMRKCHKNTCPVGIATQDPVLREKFAGEPEHVINFFFMLAEEVREIMSELGFRTVNEMVGQSDMLEVDQEVVKNNEKLENIDLSLLLRPAADIRPEAAQYCVQKQDHGLDMSLDQELIALAKPALEKAMPVYMEMPIRNVNRALGTMLSHEVTKRYQMDGLPSDTIHVKLTGSAGQSLGAFLCPGITLELEGDSNDYVGKGLSGGKIIVYPPKGSLFDPKDNIVIGNVALYGATKGEAYFNGMAAERFCVRNSGARAVVEGVGDHGCEYMTGGIVLVLGKTGRNFAAGMSGGIAYVLDLDGKFNSKCNPELVDLDKVEEEEDIMTLRMMIQQHQRHTNSEVAKEVLANFEALIPKFVKVFPRDYKRVLENMKAEQAAKEAEREAEEREEMELMEKDAFEDLKKMAAAAASNDKKVEEAVASNRPTRVDNAVKHRGFMAYERESISYRDPKARVNDWEEVAEEGKPGPKLKTQSARCMDCGTPFCHQENSGCPLGNKIPEFNELVYQNRWREALDRLLETNNFPEFTGRVCPAPCEGSCVLGIIENPVSIKSIECAIIDKAFEEGWMVPRPPHKRTGKRVAIVGSGPAGLAAADQLNKMGHLVTVYERADRIGGLMMYGVPNMKADKTHIVQRRVNLMGQEGVNFVVNANVGTDPTYSLERLRSENNALILACGATKPRDLPVPGRELSGVHFAMEFLHANTKSLLDSNLQDGQYISAKGKKVVVIGGGDTGTDCIGTSIRHGCTEMVNLELLPEPPQTRAPSNPWPQWPRIFRVDYGHQEAITKFGKDPRSYEVLTKRFIGDDNGNVKGLEVVRVKWAKDASGKFNFQEVEGSEEVIAADLVFLAMGFLGPESTVAEGLGVERDGRSNFKAEYGHFSTSVEGVFAAGDCRRGQSLVVWAINEGRQAAAQVDKFLVKKEEAQATSTRSSAKKLQQQQDKHTVMT

>DCAR_310084|Dcarota_subsp_sativus_v3.0/1-2208

MAIAPNSIVQPRSIHNLGCIAKIKPVLNPQLSVLKHAGVKTNVSPPACGLGLERTRFYGRRFFRGSGQERAHVWQTDGPGKDPKLKVVEVRSSMSQVPEKPLGLYDASFDKDSCGVGFIAELSGQSSRKMVTDAIEMLIRMTHRGACGCEENTGDGAGILVDLPHEFYKEVVKDIGIELPPPEKYAVGMFFLPTSESRREQSKTVFSKVAESLGHTILGWRRVPTDNSGLGPSAIQTEPVIEQVFLTPTPKSEAVFEQQLYTLRRVSMVAIRAALNLQNGSMKDFYICSLSSRTIVYKGQLKPDQLKEYYYADLGNQRFTSYMAMVHSRFSTNTFPSWDRAQPMRVLGHNGEINTLRGNVNWMKAREGFLKCEELQLSKNEMKKLLPVVDPSSSDSGSFDAVLELLIRAGRSLPEAIMMMIPEAWQNDENMDPERKALYEYFSALMEPWDGPALISFTDGHYLGATLDRNGLRPGRFYVTHSGRVIMASEVGVVDIPPEDVSRKGRLNPGMMLLVDFKKHVVVDDEALKKQYSLARPYGKWLERQKITLKDIVESVGESDRVSPTIEGVLQASTNDDNLENMGMHGLLAPLRAFGYTVESLEMLLLPMAKDGVEALGSMGNDTPLAVMSNREKLTFEYFKQMFAQVTNPPIDPIREKIVTSMECMIGPEGDLTETTEKQCHRLSLQGPLLSIEEMQAIKKMNYRGWRSKVVDITYSKEQGRNGLEETLDRICSEANDAIKRGYKILVLSDRGFSSKRVAVSSLLAVGAVHQHLVKKHERTRIALIVESAEPREVHHFCTLVGFGADAICPYLAVEAIWRLQVDGKIPPKSNGEFHSKEELVNKYYEASQYGMMKVLAKMGISTLASYKGAQIFEAVGLSSDVMERCFAGTPSRVEGATFEALAHDALHLHDLAFPSRALHPTSAEAVALPNPGDYHWRKGGEIHLNDPLAISKLQEAARVNSVAAYKEYSKRIQELNKSCNLRGLLKFKEAAVKIPLEEVEPASEIVKRFCTGAMSYGSISLEAHTTLAMAMNKIGGKSNTGEGGENPSRMEPLSDGSMNPRRSAIKQVASGRFGVSSYYLTNADELQIKMAQGAKPGEGGELPGHKVIGDIAVTRNSTAGVGLISPPPHHDIYSIEDLAQLIYDLKNANPAARVSVKLVSEAGVGVIASGVVKGHADHVLISGHDGGTGASRWTGIKSAGLPWELGLAETHQTLVANDLRGRTVLQTDGQLKTGRDVAIAALLGAEEFGFSTAPLITLGCIMMRKCHKNTCPVGIATQDPVLREKFAGEPEHVINFFFMLAEEVREVMSQLGFRTINEMVGRADMLEMDKELMKNNEKLKNIDLSLLLRPAADIRPEAAQYCVEKQDHGLDMVLDKKLISLSKPALSKGLPVYMETPICNRDRAVGTMLSHEVTKRYHNVGLPADTIHVKLNGSGGQSLGAFLCSGITLELEGDSNDYVGKGLSGGKIVVYPSKGSNFDPKANIVIGNVALYGATNGEAYFNGMAAERFCVRNSGAKAVVEGVGDHGCEYMTGGTVVVLGKTGRNFAAGMSGGIAYVFDGDTKFRSRCNAELVDLDKVEEEDDITTLRMMIQQHQRHTGSHLAKEVLANFDNLLPSFIKVFPRDYKRVLASLKKEEIAKNAAEKVSRETQQQEEAELAKKDAFGKLKKMAATFLNGEANEEPKAKVSDRPSQVADAVKHRGFVAYERAGISYRDPNVRMSDWKEVMEETKPGPLLNTQSARCMDCGTPFCHQENSGCPLGNKIPEFNELVYQNRWREALDRLLETNNFPEFTGRVCPAPCEGSCVLGIIENPVSIKSIECAIIDKAFEEGWMVPRPPVTRTGKRVAIVGSGPSGLAAADQLNRMGHSVTVFERSDRVGGLMMYGVPNMKTDKVEVVQRRVDLMEKEGVTFVVNATVGKDPSYSLARLREENDAIILAVGATKPRDLPVPGRELSGVHFAMEFLHANTKSLLDSNLEDGNYISAKDKKIVVIGGGDTGTDCIGTSIRHGCTSILNLELLPEPPRTRAPGNPWPQWPRIFRVDYGHQEAATKFGKDPRSYEVLTKRFIGNEDGVVKGLELVRVQWEKDASGRFQFKEVEGSEEIIEADLVLLAMGFLGPESTIAEKLELERDNRSNFKAEYGRFSTNIDGVFAAGDCRRGQSLVVWAISEGRQAASQVDKYLLKKKNDVSSDRQGNVKMDKETSKTTAR

>DCAR_626104|Dcarota_subsp_sativus_v3.0/1-2210

MSVAQNSIFQPKPASNLCSTTKPKAILNPQLNVLSRVAVRAKAKAKNCGFALEKNRFYGSRLRGSGQERHHLWQSDGPGRAPKLKVVVKSSMSQVPEKPLGLYDSAFDKDSCGVGFVAELSGESSRKTVRDAIEMLVRMAHRGACGCEANTGDGAGILVALPHDFYKEVAKDVGFELPPFGEYAVGMFFLPTSESRREQSKIVFTKVAESLGHTVLGWRSVPTDNSGLGPSTLQTEPVIEQVFLTPTPRSEVDFEQQLYILRRVSMVAIRAALNLQHGSVKDFYICSLSSRTIVYKGQLKPNQLKEYYYADLGNQRFTSYMALVHSRFSTNTFPSWDRAQPMRVLGHNGEINTLRGNVNWMKAREGLLKCRDLQLSKNEMKKLLPIVDASSSDSGSFDGVLELLIRAGRSLPEAIMMMIPEAWQNDKNMDPERKDLYEYFSALMEPWDGPALISFTDGRYLGATLDRNGLRPGRFYVTHSGRVIMASEVGVVDIPPEDVSRKGRLNPGMMLLVDFEKHVVVDDEALKQQYSKARPYGKWLERQKITLKNIVESVRESDRVCPPIAGVMQASNNDDNMENMGLRGLLAPLKAFGYTIESIEMLLLPMAKDGVEALGSMGNDAPLAVMSNREKLIFEYFKQMFAQVTNPPIDPIREKIVTSMECMVGPEGDLTETTEEQCHRLSLKGPLLSIDEMQAIKKMNYRGWRSKVLDITYSKERGGNGLEETLDRICLEAHNAIKEGYTTLVLSDRAFSPNRVAVSSLLAVGAVHQHLVKKLERTRVALIVESAEPREVHHFCTLVGFGADGICPYLAVEAIWRLQVDGKIPPKSSGEFHSKEELIKKYYRASQYGMMKVLAKMGISTLASYKGAQIFEAVGLSSDVMDRCFAGTPSRVEGATFEALAHDALHLHDIAFPSRALPPTSAEAVALPNPGDYHWRKGGEIHLNDPLAISKLQEAARGNSVAAYKEYSKRIQELNKSCNLRGLLKFKEAAVQIPLEEVEPASEIVKRFCTGAMSYGSISLEAHTTLAMAMNKIGGKSNTGEGGENPSRMEPLSDGSMNPKRSAIKQVASGRFGVSSYYLTNADELQIKMAQGAKPGEGGELPGHKVIGDIAITRNSTAGVGLISPPPHHDIYSIEDLAQLIYDLKNANPAARVSVKLVSEAGVGVIASGVVKGHADHILISGHDGGTGASRWTGIKSAGLPWELGLAETHQTLVANDLRGRTVLQTDGQLKTGRDVAMAALLGAEEFGFSTAPLITLGCIMMRKCHKNTCPVGIATQDPVLREKFAGEPEHVINFFFMIAEELREIMSQLGLRTINEMVGRSDLLEMDKDLIKDNEKLKTIDLSLLLKPAAEIRPEAAQYCVEKQDHGLDMALDQKLISLSAPALSKGLPVYMETPICNTNRAVGTMLSHEVTKRYHNIGLPADTIHVKLNGSAGQSLGAFLCSGIMLELEGDSNDYVGKGLSGGKIVVYPPKGSNFDPKENIIIGNVALYGATNGEAYFNGMAAERFCVRNSGAKAVVEGVGDHGCEYMTGGTVVVLGKTGRNFAAGMSGGIAYVFDEDSKFRSRCNAELVDLDNVEEEDDITTLRMMIQQHQRHTGSQLAKDVLSNFDKLLPSFVKVFPRDYKRILASLRKEEIAKRAAEKAAKEAEEQEEAELVEKDAFEELKKLAAANTMNEKASEEVKAKVSDRPSEVADAVKHRGFVAYERAGVSYRDPLVRMGDWKEVMEETKPGPLVKTQSARCMDCGTPFCHQENSGCPLGNKIPEFNELVYQNRWREALNRLLETNNFPEFTGRVCPAPCEGSCVLGIIENPVSIKSIECSIIDKAFEEGWMLPRPPLTRTGKKVAIVGSGPSGLAAADQLNRMGHSVTVFERSDRVGGLMMYGVPNMKTDKIDVVQRRVDLMEKEGVTFVVNASVGKDPSYSLDRLREENDAIILAVGATKPRDLPVPGRELSGVHFAMEFLHANTKSLLDSNLEDGNYISAKGKKVVVIGGGDTGTDCIGTSIRHGCNNIVNLELLPEPPRTRAPGNPWPQWPRIFRVDYGHQEAATKFGKDPRSYEVLTKRFIGDENGLVKGLEIVRVQWEKDASGRFQFKEVEGSEEIIGADLVLLAMGFLGPESTIADKLELERDNRSNFKAEYGRFSTNVDGVFAAGDCRRGQSLVVWAISEGRQAASEVDKYLLREENNVDSDRLDNTTNRQQDSNKQTVMTK

>GWHPCBIR008651|Santalum_yasi/1-2215

MSGSALQLRANPGLQIRSNSGARVQPLSALLPPRNGLSRFAQKRALRGLAAGRVEVIENRFLGAKLRAHGSERFHLWRSDGPRRSPKLRIAVRSALSQVPEKPLGLYDPSFDRDSCGVGFVAELSGESSRKTVTDAVEMLVRMTHRGACGCETNTGDGAGILVALPHPFYKEVTQGCGFELPAPGQYAVGMFFLPTSEDRRQEGKTVFAKVAESLGHTILGWRSVPTNNSGLGKSALQTEPVVEQVFLTPTPRSKANFEQQMYILRRLSMVSIRAALNLEHGGAKDFYICSLSSRTVVYKGQLKPDQLKDYYNADLGNRRFTSYMALVHSRFSTNTFPSWDRAQPMRILGHNGEINTLRGNVNWMKAREGLLKCEELGLSKDEMEKLLPIVDAGSSDSGAFDGALELLVRAGRSLPEAVMMMIPEAWQNDKNMDPHRKALYEYFSALMEPWDGPALISFTDGRYLGATLDRNGLRPGRFYVTYSGRVIMASEVGVVDIPPEDVCRKGRLNPGMMLLVDFEKHVVVDDDALKEQYSLARPYGEWLKRQKIELDDIVQTVHESERVPPAIAGVAPVSGLTDNMENVGIHGLLAPLKAFGYTVEALEMLLIPMAKDGTEALGSMGNDTPLAVMSNREKLTFEYFKQMFAQVTNPPIDPIREQIVTSMECMIGPEGDLTETTEEQCHRLSLKTPLLSIGEMEAIKKLNCRGWRSKILDITYSKDQGRKGLEETLDRICSEAHDAIKEGYTLLVLSDRAFSSKRVAVSSLLAVGAVHHHLVKNVERTRIGLIVESAEPREVHHFCTLVGFGADAICPYLAIEAIWRLQIDGKIPLKTNGGFLSKDDLVKKYFKASNYGMMKVLAKMGISTLASYKGAQIFEALGLSSEVIHRCFSGTPSRVEGATFEMLARDALHLHQMAFPARVFSPGSAESVSLPNPGDYHWRKGGEIHLNDPLAMAKLQEAARVNSVSAYKEYSKRIQELNKTCNLRGILKFKDAEVKIPLDQVEPASEIVKRFCTGAMSYGSISLEAHTTLAIAMNRIGGKSNTGEGGENPSRLEPLADGSMNPKRSAIKQVASGRFGVSSYYLTNADELQIKMAQGAKPGEGGELPGHKVIGDIAVTRHSTAGVGLISPPPHHDIYSIEDLAQLIHDLKNSNPSARVSVKLVSEAGVGVIASGVVKGHADHVLISGHDGGTGASRWTGIKNAGLPWELGLAETHQTLVANDLRGRTVLQTDGQLKTGRDVAIATLLGAEEFGFSTAPLITLGCIMMRKCHKNTCPVGIATQDPVLREKFAGEPEHVINFFFMLAEELREIMSQLGFRTINEMVGRSDMLEIDNEVTKNNEKLENIDLSLLLRPAADIRPEAAQYCVQKQDHGLDMALDQNLITLSEAALQKGLPVYIETPILNTNRAVGTMLSHEVTKRYHMTGLPADTIHIKLCGSAGQSLGAFLCPGVMLELEGDSNDYVGKGLSGGKIVVYPPKGSQFDPKENIVIGNVALYGATCGEAYFNGMAAERFCVRNSGAGAVVEGVGDHGCEYMTGGTVVVLGKTGRNFAAGMSGGVAFVLDVDEKFRSRCNPELVDLDKVEEREDIMTLRMMIQQHQRHTNSQLAKEVLANFENLLPKFIKVFPRDYKRVIARMKKEQASQDSVKQAAEKAEEPEEVDLQGKDAFEKLKKLAAATLKLKASEEVEKTEQLNRPTRVTDAIKHRGFIAYEREGVAYRDPNLRMDDWKEVMEESKPSPLLKTQSARCMDCGTPFCHQENSGCPLGNKIPEFNELVYQNRWREALDRLLETNNFPEFTGRVCPAPCEGSCVLGIIENPVSIKSIECSIIDKAFEEGWMVPRPPLKRSGKRVAVVGSGPAGLAAADQLNRMGHVVTVYERADRIGGLMMYGVPNMKADKVDIVQRRVNLMAREGVNFIVNAHIGKDPLYSFDRLREENDAIVLAVGATKPRDLPVPGRELKGVHYAMEFLHANTKSLLDSNLEDGKYISAKGKKVVVIGGGDTGTDCIGTSIRHGCSSITNLELLPQPPRTRAPGNPWPQWPRVFRVDYGHQEAATKFGSDPRSYEVLTKRFVGDENGALKGLEVVRVHWEKDSSGKFQFKEIEGSEEMIEADLVLLAMGFLGPEATVAEKLGLEQDNRSNIKADYGRFSTSVDGVFAAGDCRRGQSLVVWAISEGRQAASRVDEYLMKERETLDLSTECQGDIIKMHQRSNKCTVVT

>Brara.C01389|BrapaFPsc_v1.3/1-2199

MSAASSSSSLLQIRNNRLFPARSLRRDSTSVSQLAVASGVSRRGSCSVKNPFMGTRIKRSGSETLQLWRSDGPGRSPKLRTVVKSSFSGVPEKPMGLYDPSFDKDSCGVGFVAELSGETSRKTVTDSLEMLIRMTHRGACGCESNTGDGAGILVGMPHEFYAQAAKDLGFVLPPAGKYAVGMFFLPTAESRRDESKSVFTKVAESLGHSVIGWRSVPTDNSGLGKSALQTEPIIEQVFLTPTTKSQVDLEQQMYILRRLSMVAIRAALNLEHGAMKDFYICSLSSRTVVYKGQLKPDQLKEYYYADLGSERFTSYMALVHSRFSTNTFPSWDRAQPMRVLGHNGEINTLRGNVNWMRAREGLLKCKELGLSKQELKKLLPIVDVSSSDSGAFDGVLELLVRAGRSLPEAVMMMIPEAWQNDKNIDPSRKSFYEYLSALMEPWDGPALISFTDGRYLGATLDRNGLRPGRFYITHSGRVIMASEVGVVDVPPEDVMRKGRLNPGMMLLVDFEKHIVVDDDALKQQYSQARPYGEWLERQKIELKDIIKSVPETERIAPPISGAVTASSDDDSMESMGIHGLLSPLKAFGYTVEALEMLLLPMAKDGTEALGSMGNDAPLAVMSNREKLCFEYFKQMFAQVTNPPIDPIREKIVTSMECMIGPEGDLTETTEEQCHRLSLKGPLLTIEEMESVKKMNYRGWRTKVLDITYPKEEGTKGLEMTLDRICAEANEAIKEGYTLLVLSDRAFSSTRVAVSSLLAVGAVHHHLVKSLARTQVGLIVESAEPREVHHFCTLVGFGADAICPYLAVETVHRLQVDGKIPPKSNGEFHSKEELVKKYYKASNYGMMKVLAKMGISTLASYKGAQIFEALGLSSEVIEKCFAGTPSRVEGATFEMLARDALLLHEMAFPARAYAPGSAEASALSNPGNYHWRKNGEIHLNDPLAIAKLQEAARTNSVAAYKEYSKRINELNKQSNLRGLMKFKEADVKIPLDEVEPASEIVKRFCTGAMSYGSISLEAHQTLAMAMNKLGGKSNTGEGGELPSRMEPLPDGSRNPKRSSIKQIASGRFGVSSYYLTNADELQIKMAQGAKPGEGGELPGHKVIGDIAITRNSTAGVGLISPPPHHDIYSIEDLAQLIHDLKNANPGARISVKLVSEAGVGVIASGVVKGHADHVLIAGHDGGTGASRWTGIKNAGLPWELGLAETHQTLVANDLRGRTVLQTDGQLKTGRDVAVAALLGAEEFGFSTAPLITLGCIMMRKCHKNTCPVGIATQDPVLREKFAGEPEHVINFFFMLAEEVREIMSGLGFRTLTEMIGRADMLELDREVVKNNDKLANIDLSLLLRPAAEIRPGAAQYCVQKQDHGLDMALDQELIALSKSALEKSLPVYIETPICNVNRAVGTMLSHEVTKRYHLAGLPKDTIHIKFSGSAGQSLGAFLCPGITLELEGDSNDYVGKGLSGGKVVVYPPKGSSFDPKENIVIGNVALYGATSGEAYFNGMAAERFSVRNSGAQAVVEGVGDHGCEYMTGGTVVVLGKTGRNFAAGMSGGIAYVLDVDGKFHTRCNLELVDLDKVEDDEDKMTLKMMIQQHQRHTNSQLAQEVLGDFENLLPKFIKVFPRDYKRVLAAMKDEEVSKQAIDRAAEEAEEMEEKEVEEKDAFAELKNMAAAASSKEVVSGNGAATEAPKKPSLVDNAVKHRGFIAYEREGVKYRDPNVRMNDWNEVMEESKPGPLLKTQSARCMDCGTPFCHQENSGCPLGNKIPEFNELVYQNRWQEALNRLLETNNFPEFTGRVCPAPCEGSCVLGIIENPVSIKSIECSIIDKAFEEGWMVPRPPLTRTGKKVAIIGSGPAGLAAADQLNKMGHSVTVYERADRIGGLMMYGVPNMKTDKIDIVQRRVDLMTKEGINFVVNANIGKDPSYSLDGLKEENDAIVLAVGSTKPRDLPVPGRDLTGVHFAMEFLHANTKSLLDSNLEDGNYISAKGKKVVVIGGGDTGTDCIGTSIRHGCTNIVNLELLPQPPSTRAPGNPWPQWPRVFRIDYGHQEAATKFGKDPRTYEVLTKRFIGDDNGNVKGLEIVRVSWEKDESGRFNFKEIEGSEEIIEADLVFLAMGFLGPEPTLAEKLGLECDNRSNFKAEYGRFSTTVEGVFAAGDCRRGQSLVVWAISEGRQAASQVDKFLSKNEDELVDATVGQNLNEVKHQH

>AT5G53460|Athaliana_Araport11/1-2208

MSAASSSSVLHLRTNQQLLSLRSLKNSTSVASQLAVTSGVSRRRSCTARCSVKKPVIPESPFLGTRVRRSGSETLQFWRSDGPGRSAKLRTVVKSSFSAVPEKPLGLYDPSYDKDSCGVGFVAELSGETTRKTVTDSLEMLIRMTHRGACGCESNTGDGAGILVGLPHDFYAEAATELGFVLPSAGNYAVGMFFLPTVESRREESKNVFTKVAESLGHSVLGWRLVPTDNSGLGNSALQTEPIIAQVFLTPTTKSKADFEQQMYILRRVSMVAIRAALNLQHGAMKDFYICSLSSRTIVYKGQLKPDQLKDYYYADLGSERFTSYMALVHSRFSTNTFPSWDRAQPMRVLGHNGEINTLRGNVNWMRAREGLLKCNELGLSKKELKKLLPIVDVSSSDSGAFDGVLELLVRAGRSLPEAVMMMIPEAWQNDKNIDPSRKEFYEYLSALMEPWDGPALISFTDGRYLGATLDRNGLRPGRFYITHSGRVIMASEVGVVDVPPEDVMRKGRLNPGMMLLVDFEKHIVVDDDALKQQYSLARPYGEWLKRQKIELKDIIESVPEAERIAPSISGVVPASNDDDSMESMGIHGLLSPLKAFGYTVEALEMLLLPMAKDGSEALGSMGNDTPLAVMSNREKLCFEYFKQMFAQVTNPPIDPIREKIVTSMECMIGPEGDLTETTEEQCHRLSLKGPLLKIEEMEAIKKMNYRGWRTKVLDITYAKERGTKGLEETLDRICDEANEAIKEGYTLLVLSDRAFSATRVAVSSLMAVGAVHHHLVKTLARTQVGLVVESAEPREVHHFCTLVGFGADAICPYLAVEAVYRLQVDGKIPPKSNGEFHSKEELVKKYYKASNYGMMKVLAKMGISTLASYKGAQIFEALGLSSEVIQKCFAGTPSRVEGATFEMLARDGLQLHELAFPTRGYAPGSAEASALTNPGNYHWRKNGEIHLNDPLAIAKLQEAARTNSVAAYKEYSKRINELNKQSNLRGLMKFKDADVKIPLDEVEPASEIVKRFCTGAMSYGSISLEAHTTLAMAMNKLGGKSNTGEGGELPSRMEPLADGSRNPKRSSIKQIASGRFGVSSYYLTNADELQIKMAQGAKPGEGGELPGHKVIGDIAITRNSTAGVGLISPPPHHDIYSIEDLAQLIHDLKNANPGARISVKLVSEAGVGVIASGVVKGHADHVLIAGHDGGTGASRWTGIKNAGLPWELGLAETHQTLVANDLRGRTVLQTDGQLKTGRDVAVAALLGAEEFGFSTAPLITLGCIMMRKCHKNTCPVGIATQDPVLREKFAGEPEHVINFFFMLAEEVREIMSGLGFRTVTEMIGRADMLELDREVVKNNDKLENIDLSLLLRPAAEIRPGAAQYCVQKQDHGLDMALDQELIALSKSALEKSLPVYIETPICNVNRAVGTMLSHEVTKRYHLTGLPKDTIHIKFTGSAGQSLGAFLCPGIMLELEGDSNDYVGKGLSGGKVVVYPPKGSSFDPKENIVIGNVALYGATSGEAYFNGMAAERFSVRNSGAKAVVEGLGDHGCEYMTGGTVVVLGKTGRNFAAGMSGGIAYVLDVDGKFNTRCNLELVDLDKVEDEEDKMTLKMMIQQHQRHTNSQLAQEVLADFENLLPKFIKVFPRDYKRVLSAMKHEEVSKQAIERASEEADETEEKELEEKDAFAELKNMAAASSKEEMSGNGVAAEARPSKVDNAVKNGGFIAYEREGVKYRDPNVRLNDWNEVMEESKPGPLLTTQSARCMDCGTPFCHQENSGCPLGNKIPEFNELVYQNRWQEALNRLLETNNFPEFTGRVCPAPCEGSCVLGIIENPVSIKSIECAIIDKAFEEGWMVPRPPLKRTGKKVAIIGSGPAGLAAADQLNKMGHLVTVYERSDRIGGLMMYGVPNMKTDKIDVVQRRVDLMTKEGINFVVNANIGKDPSYSLDGLKEENDAIVLAVGSTKPRDLPVPGRDLSGVHFAMEFLHANTKSLLDSNHEDGNYISAKGKKVVVIGGGDTGTDCIGTSIRHGCTNIVNLELLPQPPSTRAPGNPWPQWPRVFRIDYGHQEATTKFGKDPRTYEVLTKRFIGDDNGNVKGLELVRVSWEKDETGRFQFKEIEGSEEIIEADLVFLAMGFLGPEPTLAEKLGLECDNRSNFKAEYGRFSTTVEGVFAAGDCRRGQSLVVWAISEGRQAADQVDKFLTKTDDDEDAKLQQDLNQMKHNTITN

>Aqcoe7G011100|Acoerulea_v3.1/1-2211

MSVISGSAFQLKSNSVVLNSINRSIITNHHGLSVVSPLNYRTKNRICSVKKSDNDNVIEHKFYGTRLRQQSSSERFHLWRTEGPGRSPKLRVAVRSMALSQVPEKPLGLYDPSFDKDACGVGFVAELSGESHRKTVMDAIEMLVRMTHRGACGCETNTGDGAGILVGLPHEFFMEVAKDMGFQLPPRGNYAVGMLFLPTSENRRQESKAVFTKVAESLGHTVLGWRPVPTDNTGLGKSALETEPVVEQVFLTPSPRSKADFEQQMYILRRFSMEAIRSALNLQHGGVKDFYICSLSSRTIVYKGQLKPDQLKDYYYADLGDERFKSYMALVHSRFSTNTFPSWDRAQPMRIVGHNGEINTLRGNVNWMRAREGLLKCKELGLTKNEMKTLLPIVDHSSSDSGAFDGVLELLVRAGRSLPEAVMMMIPEAWQNDKNMDPERKAFYEYFSTLMEPWDGPALVSFTDGRYLGATLDRNGLRPGRFYITHSGRVIMASEVGVVDVPPEDVCRKGRLNPGMMLLVDFENHVVVDDDALKKQYSFAKPYGEWLKRQKIELKDIVESVHKSDRAPPVISGALSAAVNGDTVDNKGIHGLLAPLRAFGYTVEALDMLLLPMAKDGTEALGSMGNDAPLAVMSDREKLTFEYFKQMFAQVTNPPIDPIREKIVTSMECMIGPEGDLTETTEEQCHRLSLKGPLLTIEEMEAIKKMNYRGWRSKVLDITYSKTRGRKGLEETLDRICYEACNAIKDGFTTLVLSDRAFSSKRVAVSSLLAVGAVHHHLVSKLERTRVGLIIESAEPREVHHFCTLVGFGADAICPYLAIETVWRLQIDGRIPPKSSGDFHSKEELVRKYFKASNYGMMKVLAKMGISTLASYKGAQIFEALGLSSEVIRKCFSGTPSRVEGATFEMLAQDALQLHELGFPSRFLPPGSAEEVALPNPGDYHWRKGGEVHLNDPLAIAKLQEAARTNSVAAYKEYSKRIQELNKSCNLRGMLKFKDAEVQVPLGEVEPASEIVKHFCTGAMSYGSISLEAHTTLALAMNKIGGKSNTGEGGENPSRMKPLPDGSMNPKRSSIKQVASGRFGVSINYLTNADELQIKMAQGAKPGEGGELPGHKVIGDIAVTRNSTAGVGLISPPPHHDIYSIEDLAQLIHDLKNSNPKARISVKLVSEAGVGVIASGVVKGHADHVLISGHDGGTGASRWTGIKSAGLPWELGLAETHQTLVANDLRGRTTLQTDGQLKTGRDVAIAALLGAEEFGFSTAPLITLGCIMMRKCHKNTCPVGIATQDPVLREKFAGEPEHVINFFFMLAEEVREIMSQLGFRTMKEMVGRSDMLEVDSEVVKNNEKLENIDLSLLLKPAAEIRPEAAQYCIQKQDHALDMALDQKLITLSNAALQRSLPVYIELPICNVNRAVGTMLSHEVTKRYHVDGLPADTIHIKLTGSAGQSLGAFLCPGIMLELEGDSNDYVGKGLSGGKIVVYPPRKSTFNPKENIVIGNVALYGATSGEAYFKGMAAERFCVRNSGAKAVVEGIGDHGCEYMTGGIVVILGKTGRNFAAGMSGGIAYVFDVDKTFQSRCNLELVDLDKVEDDEDIMTLRMMIQQHQRYTKSELAWEILDDFQNLLPKFIKVFPRDYKRVLQNMKVEKAVPQVDMQAENQEEAELMEKDAFEELKKLAVKSQHEKGSQVDEAQSLKRPTEVHDAVKHRGFVAYDRETISYRNPENRINDWKEVMEKSKPGPLLKTQSARCMDCGTPFCHQDNYGCPLGNKIPEFNELVYQNRWREALDRLLETNNFPEFTGRVCPAPCEGSCVLGIIENPVSIKSIECSIIDKAFDEGWMIPRPPLRRTGKKVAIVGSGPAGLAAADQLNRMGHSVTVFERADRIGGLMMYGVPNMKADKVNIVQRRVNLMAEEGINFVVNANVGSDPIYSIERLRSENDAIILSLGATKPRDLPVPGRELSGIHFAMEFLHANTKSLLDSNLQDGKYISAKGKKVVVIGGGDTGTDCIGTSLRHGCTNIVNLELLPEPPQKRAPGNPWPQWPRIFRVDYGHQEALTKFGKDPRSYEVLTKRFVGDKNGFVKGVEVVQVHWEKDSSGRFQFKEIEGSEMIIEADLVFLAMGFLGPEPIIADQLGLEQDNRSNYKAEYGRFLTSVEGVFAAGDCRRGQSLVVWAISEGRQAASQVDEYLMREMEDLSNTTNQETVNRQHNNSKYSVMR

>GSMUA_Achr7G24500_001|Macuminata_v1/1-2138

MAAVPGSAIKLQNQSVALPSVVNHKRNVCRGGRAARFAAYRRAQAVSLESQFLGAKLRASERVQRWRIDGPGRSPKLRVVSPSMALSQVPEKPLGLYDPSFDKDSCGVGFIAELSGEYSRKTVADSLEMLVRMAHRGACGCETNTGDGAGILVALPHGFFKEARILSFSIVTEDIGIQLPPPGQYAVGMFFLPTDDSRRKESKIVFTKVAESLGHVVLGWRPVPTDNRDLGESARQTEPLIEQVFLTPSPRSSADFEQQMYILRRVSMVAIRAALNLQHGGARDFYICSLSSRTVVYKGQLKPVQLKDYYYADLGDERFTSYMALVHSRFSTNTFPSWDRAQPMRVLGHNGEINTLRGNVNWMKAREGLLKCKELGLSKNEMKKLLPIVDASSSDSGAFDGVLELLVRAGRSLPEAVMMMIPEAWQNDKNMDPDRKALYEYFSALMEPWDGPALISFTDGRYLGATLDRNGLRPGRFYITHSGRVIMASEVGVVDIPPADVLRKGRLNPGMMLLVDFENRTVVDDEALKKQYSQARPYREWLKRQKICLDDIVNSIPKSDMIPPNIFGTVPSQNHDEDMENMGIRGLLAPLKAFGYTVEALDMLLLPMAKDATEALGSMGNDAPLAVMSNREKLSFEYFKQMFAQVTNPPIDPIREKIVTSMECMVGPEGDLTETTEEQCHRLSLKGPLLSIDEMESIKMMNYRGWRSKVLDITYPKKHGRKGLEETLNRICLEARAAIREGYTTLVLSDRGFSSERVAVSSLLAVGAVHQHLVSTLERTRIGLLVESAEPREVHHFCTLVGFGADAICPYLAMEAIWRYFKASNYGMMKVLAKMGISTLASYKGAQIFEALGLSSEVIEKCFKGTPSRVEGATFEMLAGDALCEVHLNDPLAIAKLQEAARANSVAAYKEYSRRIQELNKTCNLRGILKFKDVAEKVPLDEVESASEIVKRFCTGAMSYGSISLEAHSTLAIAMNKIGGKSNTGEGGEQPARMEPLSDGSRNPKRSAIKQVASGRFGVTSYYLTNADELQIKMAQGAKPGEGGELPGHKVIGDIAVTRNSTAGVGLISPPPHHDIYSIEDLAQLIHDLKNSNPGARISVKLVSEAGVGVIASGVVKGHADHVLISGHDGGTGASRWTGIKNAGLPWELGLAETHQTLVANDLRGRTVLQTDGQLKTGRDVAIAALLGAEEFGFSTAPLITLGCIMMRKCHKNTCPVGIATQDPVLREKFAGEPEHVINFFFMLAEEVREIMSQLGFRTINEMVGRADMLEIDKEVAKSNEKLGNIDLSLLLRPAADIRPGVAQYCIQKQDHGLEMALDQDLITSSKAALEKGLPVYVETPIRNVNRAVGTMLSHEVTKQYQLKGLPSDTIHIKLVGSAGQSLGAFLCPGITLELEGDSNDYVGKGLSGGKIVVYPPRESQFDPSENIVVGNVALYGATSGEAYFNGMAAERFCVRNSGARAVVEGVGDHGCEYMTGGTVVILGKTGRNFAAGMSGGIAYVFDVDGMFHTRCNPELVDLEKIEDGEDITTLRMMIQQHQRHTSSVLAREVLSNFDSLLPKFVKVFPRDYKRIVQNLKIEQAAKESEEQEEKELMEKDAFEELKKMAAASLNGKKAEGLAAAKRPTQVDNAVKHRGFIAYERQGISYRDPNDRIKDWKEVATESKPGPLMKTQSARCMDCGTPFCHQENSGCPLGNKIPEFNELVHQNRWREALDRLLETNNFPEFTGRVCPAPCEGSCVLGIIENPVSIKSIECAIIDKAFEEGWMVPRPPQKRTGKRVAIVGSGPAGLAAADQLNKMGHLVTVYERADRIGGLMMYGVPNMKADKVDVVQRRVDLMTREGVNFMVNANVGVDPMYSLNHLRAENDAIVLACGATKPRDLPVPGRELSGVHFAMEFLHANTKSLLDSNLQDGKYLSAKGKKVVVIGGGDTGTDCIATSIRHGCTNMINLELLPEPPQKRAPGNPWPQWPRIFRVDYGHQEASAKFGKDPRSYEVLTKRFVGDESGVVKGLEVVRVHWAKDSGGKFQFEEIKGSEEIIEADLVLLAMGFLGPELTIADQLGLERDNRSNFKAEYGHFSTSVDGVFAAGDCRRGQSLVVWAINEGRQAASQVDKYLMKDVNTCSKENPVSSEDLVHRVTA

>XP_020573934.1|Phalaenopsis_equestris/1-2021

MIPLLLSFQFFLGCLKLSSALQVAVSLGHEVIGWRRVPTDNSDLGQSALQTEPIIEQVFLKPRLGSNIDFEQQMYILRRFSMSAIRAALDLQHGGARDFYICSLSSRTVVYKGQLKPIQLKNYYYADLGDERFTSYMALVHSRFSTNTFPSWDRAQPMRVLGHNGEINTLRGNVNWMKAREGLMKCKNLGLSKDEMKKLLPIVDVSSSDSGAFDGVLELLLRAGRDLPEAVMMMIPEAWQNDHNMEPERKALYEYFSALMEPWDGPALVSFTDGRYLGATLDRNGLRPGRFYITYSGRVIMASEVGVVDIPPEDVLRKGRLNPGMMLLVDFENHVVVDDDALKKQYSLAQPFEEWLKKQKITLKDIVDSMPETDRVIPAVFGTIPANGVDDTIENMGIHGILAPLKAFGYTVECLEMLLLPMAKDGTEALGSMGNDAPLAVMSGREKLMFEYFKQMFAQVTNPPIDPIREKIVTSMECMIGPEGDLTETTEEQCHRLTLKGPFLSIEEMEAIKKMNYRGWRSKVLDLTYPKKLGRKGLEETLNRICSEARNAIYEGYTTIVLSDRGFSSDNVAVSSLLAVGAVHQHLVSALERTRVGLVVESAEPREVHHFCTLVGFGADAICPYLAIEAVWRLQIDGKIPPKGNGEFHSQEELVQKYFKACNDGVMKVLAKMGISTLASYKGAQIFEALGLSTEVIDKCFKGTPSRVEGATFEMLAGDALSLHDTAFPKRAFPPGSADVVALPNPGDYHWRKGGEVHLNDPLAIAKLQEAARQNSVAAYKEYSRRVQELNKSCNLRGLLKFKDNQEKISLNEVEPASEIVKRFCTGAMSYGSISLETHTTLAIAMNKLGGKSNTGEGGEQPSRMEPLPDGSKNPKRSAIKQVASGRFGVSSYYLTNADELQIKMAQGAKPGEGGELPGHKVVGDIAITRNSTSGVGLISPPPHHDIYSIEDLAQLIHDLKNANPGARISVKLVSEAGVGVIASGVVKGHADHVLISGHDGGTGASKWTGIKNAGLPWELGLAETHQTLVANDLRGRTILQTDGQLKTGKDVAIAALLGAEEFGFSTAPLITLGCIMMRKCHKNTCPVGIATQDPVLRAKFAGEPEHVINFFFMLAEELREIMAQLGFRTINEMVGRSDVLEIDPDVVKNNEKLTNIDLSLLLKPAAEIRPDAAQFFVQNQDHGLEMALDNKLISLSKPALEKAIPVYIEMPIRNVNRAVGTMLSHEVTKKYFMDGLPSDTIHIRLNGSAGQSFGAFLCPGITLELEGDSNDYVGKGLSGGKIIVYPPKQSQFDPKENIVIGNVALYGATKGEAYFSGMAAERFCVRNSGATAVVEGVGDHGCEYMTGGVVVVLGKTGRNFAAGMSGGIAYVYDVDGKFHNLCNLELVDLEKIEEEEDVMTLKMMIQQHQRRTSSQLAREILSDFDVLLPKFVKVFPRDYKRVLQNLKVEKAAEEAKKQEERELMKKDAFEELKQLATATRNVNGYLKSEDSKSRKRPTQVADAVKHRGFITYEREGITYRDPNLRINDWKEVAVESKPGPLLKTQSARCMDCGTPFCHQENSGCPLGNKIPEFNELVHQNRWREALDRLLETNNFPEFTGRVCPAPCEGSCVLGIIENPVSIKSIECSIIDKAFANGWMVPRPPLKRTGKMVAIVGSGPAGLAAADQLNKMGHWVTVYERSDRIGGLMMYGVPNMKADKIDIVQRRVDLMAKEGVKFVVNANVGTDPKYSLDEMRADSDAIILACGATRPRDLQVPGRELSGVHFAMEFLHSNTKSLLDSNLRDGQYISAEGKKVVVIGGGDTGTDCIGTSIRHGCSGIVNLELLSEPPRTRSPNNPWPQWPRIFRVDYGHQEAAAKFGKDPRSYEVLTKRFIGDETGAVKALEIVGVRWAKDSGGRLQPQEIEGSQTIIEADLVLLAMGFLGPDPTIATKLGLELDNRSNFQAEYGRFATSVDGVFAAGDCRRGQSLVVWAINEGRQAAAEVDKYLLREEEEDDSVNGASDSGRKYLLDNIDGRQKVAA

>XP_020573935.1|Phalaenopsis_equestris/1-1888

MFLIFQVHSRFSTNTFPSWDRAQPMRVLGHNGEINTLRGNVNWMKAREGLMKCKNLGLSKDEMKKLLPIVDVSSSDSGAFDGVLELLLRAGRDLPEAVMMMIPEAWQNDHNMEPERKALYEYFSALMEPWDGPALVSFTDGRYLGATLDRNGLRPGRFYITYSGRVIMASEVGVVDIPPEDVLRKGRLNPGMMLLVDFENHVVVDDDALKKQYSLAQPFEEWLKKQKITLKDIVDSMPETDRVIPAVFGTIPANGVDDTIENMGIHGILAPLKAFGYTVECLEMLLLPMAKDGTEALGSMGNDAPLAVMSGREKLMFEYFKQMFAQVTNPPIDPIREKIVTSMECMIGPEGDLTETTEEQCHRLTLKGPFLSIEEMEAIKKMNYRGWRSKVLDLTYPKKLGRKGLEETLNRICSEARNAIYEGYTTIVLSDRGFSSDNVAVSSLLAVGAVHQHLVSALERTRVGLVVESAEPREVHHFCTLVGFGADAICPYLAIEAVWRLQIDGKIPPKGNGEFHSQEELVQKYFKACNDGVMKVLAKMGISTLASYKGAQIFEALGLSTEVIDKCFKGTPSRVEGATFEMLAGDALSLHDTAFPKRAFPPGSADVVALPNPGDYHWRKGGEVHLNDPLAIAKLQEAARQNSVAAYKEYSRRVQELNKSCNLRGLLKFKDNQEKISLNEVEPASEIVKRFCTGAMSYGSISLETHTTLAIAMNKLGGKSNTGEGGEQPSRMEPLPDGSKNPKRSAIKQVASGRFGVSSYYLTNADELQIKMAQGAKPGEGGELPGHKVVGDIAITRNSTSGVGLISPPPHHDIYSIEDLAQLIHDLKNANPGARISVKLVSEAGVGVIASGVVKGHADHVLISGHDGGTGASKWTGIKNAGLPWELGLAETHQTLVANDLRGRTILQTDGQLKTGKDVAIAALLGAEEFGFSTAPLITLGCIMMRKCHKNTCPVGIATQDPVLRAKFAGEPEHVINFFFMLAEELREIMAQLGFRTINEMVGRSDVLEIDPDVVKNNEKLTNIDLSLLLKPAAEIRPDAAQFFVQNQDHGLEMALDNKLISLSKPALEKAIPVYIEMPIRNVNRAVGTMLSHEVTKKYFMDGLPSDTIHIRLNGSAGQSFGAFLCPGITLELEGDSNDYVGKGLSGGKIIVYPPKQSQFDPKENIVIGNVALYGATKGEAYFSGMAAERFCVRNSGATAVVEGVGDHGCEYMTGGVVVVLGKTGRNFAAGMSGGIAYVYDVDGKFHNLCNLELVDLEKIEEEEDVMTLKMMIQQHQRRTSSQLAREILSDFDVLLPKFVKVFPRDYKRVLQNLKVEKAAEEAKKQEERELMKKDAFEELKQLATATRNVNGYLKSEDSKSRKRPTQVADAVKHRGFITYEREGITYRDPNLRINDWKEVAVESKPGPLLKTQSARCMDCGTPFCHQENSGCPLGNKIPEFNELVHQNRWREALDRLLETNNFPEFTGRVCPAPCEGSCVLGIIENPVSIKSIECSIIDKAFANGWMVPRPPLKRTGKMVAIVGSGPAGLAAADQLNKMGHWVTVYERSDRIGGLMMYGVPNMKADKIDIVQRRVDLMAKEGVKFVVNANVGTDPKYSLDEMRADSDAIILACGATRPRDLQVPGRELSGVHFAMEFLHSNTKSLLDSNLRDGQYISAEGKKVVVIGGGDTGTDCIGTSIRHGCSGIVNLELLSEPPRTRSPNNPWPQWPRIFRVDYGHQEAAAKFGKDPRSYEVLTKRFIGDETGAVKALEIVGVRWAKDSGGRLQPQEIEGSQTIIEADLVLLAMGFLGPDPTIATKLGLELDNRSNFQAEYGRFATSVDGVFAAGDCRRGQSLVVWAINEGRQAAAEVDKYLLREEEEDDSVNGASDSGRKYLLDNIDGRQKVAA

>Kalax.0500s0005|Klaxiflora_v1.1/1-2202

MAATTSPATPLQLRNTQRAPLLNHQLNLITSVRRSARRNVCRRCNVASGPVRTFFGKALNSSSLSSRLCHSRNTRRVRFGHRASLSAVPDEPMGLYNPEYDKDSCGVGFVGELSGQPSRKTVTDALEMLVRMSHRGACGCETNTGDGAGILVALPHQFYAEIAKEVGFELPQPGKYAVGMFFLPTNDTRREQSKHLFSQVASSLGHSVLGWRTVPTDNSGLGKSALLTEPIIEQVFLTSSSTSKAEIEQQMYILRRTSMYTIREELDMQHGSSKDFYICSLSSRTVVYKGQLKPDQLMDYYYTDLGSERFTSYMALVHSRFSTNTFPSWDRAQPMRVLGHNGEINTLRGNVNWMKAREGLLKIKELNLSKEDRQKLLPIVDASSSDSGAFDGVLELLVRAGRSLPEAVMMMIPEAWQNDKNMDPQRKALYEYFSALMEPWDGPALISFTDGRYLGATLDRNGLRPGRFYITHSGRVIMASEVGVVDIPPADVARKGRLNPGMMLLVDFENHTVVDDSELKKKYSLSRPYGEWLIRQKIQLKDVVDSVNESERITPPINGVVSVSDNAENMENVGIHGLLAPLKAFGYTLEALEMLLLPMAKDGTEALGSMGNDAPLAVMSNREKLTFEYFKQMFAQVTNPPIDPIREKIVTSMECMIGPEGDLTETTEAQCHRLSLKGPLLTMKETEAIKKMNYRGWRSKVLDITYPKSRGRKGLEETLNRICYEAREAITEGYTLLVLSDRAFSPSRVAVSSLLAVSAVHHHLVKKLERTRVGLVIESAEPREVHHFCTLVGFGADAICPYLAIEAIWRLQIDGKIPPKPTGELLSKDELVKKYFKASNYGMMKVLAKMGISTLASYKGAQIFEALGLSSEVIERCFAGTPSRVEGATFEMLARDALHLHEMAFPGRVFPPGSAEAVALPNPGDYHWRKGGEVHLNDPLAIAKLQEAAKSNSVAAYKEYSKRIQELNKECNLRGILKFKEVESKVPVDEVEPASEIVKRFCTGAMSYGSISLEAHTTLAVAMNQLGGKSNTGEGGEQPSRMEPLSDGSMNPKRSAIKQVASGRFGVSSFYLTNADELQIKMAQGAKPGEGGELPGHKVIGDIAVTRNSTAGVGLISPPPHHDIYSIEDLAQLIHDLKNSNPSARISVKLVSEAGVGVIASGVVKGHADHVLISGHDGGTGASRWTGIKNAGLPWELGLAETHQTLVANDLRGRTTLQTDGQLKTGRDVAIAALLGAEEFGFSTAPLITLGCIMMRKCHKNTCPVGIATQDPVLREKFAGEPEHVINFFFMLAEELREIMSQLGFRSINEMIGRSDMLEVDKEVINSNEKLENIDLSLLLKPAAEIRPEAAQYCVQKQDHGLDMALDQRLISLSEPALEKRLPVYIETPICNVNRAVGTMLSHEVTKRYHVAGLPSGTIHVKLNGSAGQSLGAFLCSGIFLELEGDSNDYVGKGLSGGKIVVYPPRESTFDPKENIVIGNVALYGATSGEAYFNGMAAERFCVRNSGASAVVEGIGDHGCEYMTGGTVVVLGKTGRNFAAGMSGGIAYVLDIDGKFRSRCNPELVDLDKVEEEEDIMTLKMMIQQHQRHTNSQLAREVLSDFDSLLPKFIKIFPRDYKRILASLKTEHSLKDSLVINVKETEDQDEAELKEKDAFEELKKLAKSSMTEKNPLSTDEAEASKRPSRVPDAVKYRGFIAYEREGVQYREPSERLKDWKEVMMETKPGPLLKTQSARCMDCGTPFCHQENSGCPLGNKIPEFNELVYQNRWREALDRLLETNNFPEFTGRVCPAPCEGSCVLGIIENPVSIKNIECSIIDKAFEEGWMVPRPPVSRTGKRVAIVGSGPAGLAAADQLNRMGHWVTVYERADRIGGLMMYGVPNMKTDKVDIVQRRVNLMAEEGIDFVVNASVGKDPLYSLDRLREENDAIVLAVGATKPRDLPVPGRELSGVHFAMEFLHANTKSLLDSNLQDGNYISAKGKKVVVIGGGDTGTDCIGTSIRHGCSSIVNLELLPQPPKTRASGNPWPQWPRVFRVDYGHEEATTKFGKDPRSYEVLTKRFIGDENGVVKGIEVVSVKWEKDASGRFQFKEIEGSEEIIEADLVFLAMGFLGPEEAIAEKLGLERDNRSNFKAEYGRFATNVDGVFAAGDCRRGQSLVVWAISEGRQAASQVDNYLLKDDEEPMGNDEESTLNQQSDGKKYTVVT

>Kalax.0027s0017|Klaxiflora_v1.1/1-2201

MAATTSPATPLQLRNTQRAPLLNHQLNLIPSVRRSARRNVCRRCNVASGPVRTFFGKALNSSLSSRLCHSRNTRRVRFRHRASLSAVPDEPMGLYNPEYDKDSCGVGFVGELSGQPSRKTVTDALEMLVRMSHRGACGCETNTGDGAGILVALPHQFYAEIAKEVGFELPQPGKYAVGMFFLPTNDTRREQSKHLFSQVASSLGHSVLGWRTVPTDNSGLGKSALLTEPIIEQVFLTSSSTSKAEIEQQMYILRRTSMYTIREELDMQHGSSKDFYICSLSSRTVVYKGQLKPDQLMDYYYTDLGSERFTSYMALVHSRFSTNTFPSWDRAQPMRVLGHNGEINTLRGNVNWMKAREGLLKIKELNLSKEDRQKLLPIVDASSSDSGAFDGVLELLVRAGRSLPEAVMMMIPEAWQNDKNMDPQRKALYEYFSALMEPWDGPALISFTDGRYLGATLDRNGLRPGRFYITHSGRVIMASEVGVVDIPPADVARKGRLNPGMMLLVDFENHTVVDDSELKKKYSLSRPYGEWLIRQKIQLKDVVDSVNESERITPPINGVVSVSDNAENMENMGIHGLLAPLKAFGYTLEALEMLLLPMAKDGTEALGSMGNDAPLAVMSNREKLTFEYFKQMFAQVTNPPIDPIREKIVTSMECMIGPEGDLTETTEAQCHRLSLKGPLLTIKETEAIKKMNYRGWRSKVLDITYPKSRGRKGLEETLNRICYEAREAITEGYTLLVLSDRAFSPSRVAVSSLLAVGAVHHHLVKKLERTRVGLVIESAEPREVHHFCTLVGFGADAICPYLAIEAIWRLQIDGKIPPKPTGELLSKDELVKKYFKASNYGMMKVLAKMGISTLASYKGAQIFEALGLSSEVIERCFAGTPSRVEGATFEMLARDALHLHEMAFPSRVFPPGSAEAVALPNPGDYHWRKGGEVHLNDPLAIAKLQEAAKSNSVAAYKEYSKRIQELNKECNLRGILKFKEVESKVPVDEVEPASEIVKRFCTGAMSYGSISLEAHTTLAVAMNQLGGKSNTGEGGEQPSRMEALSDGSMNPKRSAIKQVASGRFGVSSFYLTNADELQIKMAQGAKPGEGGELPGHKVIGDIAVTRNSTAGVGLISPPPHHDIYSIEDLAQLIHDLKNSNPSARISVKLVSEAGVGVIASGVVKGHADHVLISGHDGGTGASRWTGIKNAGLPWELGLAETHQTLVANDLRGRTTLQTDGQLKTGRDVAIAALLGAEEFGFSTAPLITLGCIMMRKCHKNTCPVGIATQDPVLREKFAGEPEHVINFFFMLAEELREIMSQLGFRSINEMIGRSDMLEVDKEVINSNEKLENIDLSLLLKPAAEIRPEAAQYCVQKQDHGLDMALDQRLISLSEPALEKRLPVYIETPICNVNRAVGTMLSHEVTKRYHVAGLPSGTIHVKLNGSAGQSLGAFLCSGIFLELEGDSNDYVGKGLSGGKIVVYPPRESTFDPKENIVIGNVALYGATSGEAYFNGMAAERFCVRNSGASAVVEGIGDHGCEYMTGGTVVVLGKTGRNFAAGMSGGIAYVLDIDGKFRSRCNPELVDLDKVEEEEDIMTLKMMIQQHQRHTNSQLAREVLSDFDSLLPKFIKVFPRDYKRILASLKTEHSLKDSLVISVKETEDQDEAELKEKDAFEELKKLAMSSMTEKNPLSTDEAEASKRPSRVPDAVKHRGFIAYEREGVQYREPSERLKDWKEVMMETKPGPLLKTQSARCMDCGTPFCHQENSGCPLGNKIPEFNELVYQNRWREALDRLLETNNFPEFTGRVCPAPCEGSCVLGIIENPVSIKNIECSIIDKAFEEGWMVPRPPVSRTGKRVAIVGSGPAGLAAADQLNRMGHWVTVYERADRIGGLMMYGVPNMKTDKVDIVQRRVNLMAEEGIDFVVNASVGKDPLYSLDRLREENDAIVLAVGATKPRDLPVPGRELSGVHFAMEFLHANTKSLLDSNLQDGNYISAKGKKVVVIGGGDTGTDCIGTSIRHGCSSIVNLELLPQPPKTRASGNPWPQWPRVFRVDYGHEEATTKFGKDPRSYEVLTKRFIGDENGVVKGIEVVSVKWEKDASGRFQFKEIEGSEEIIEADLVFLAMGFLGPEEAIAEKLGLERDNRSNFKAEYGRFATNVDGVFAAGDCRRGQSLVVWAISEGRQAASQVDNYLLKDDEEAMGNDEESTLNQQSDGKKFTVVT

>Nycol.M00467|Ncolorata_v1.2b/1-2180

MAAVPGVGLQLRNESVLLPSVQGKRGLNAPNPSNKAVCRHVPRDNSMFGARVRPCSERLHHVWRSDGPGCTPELKVVRAMGMSGEPTEPLGLYNSAFDKDSCGVGFVAELSGEPSRKTVLDALEMLIRMAHRGACGCEVNTGDGAGILVALPHDFFKEVAKDSGFELPPPGEYAVGMFFLPTTDGRREESKIVFTKVAESLGHQVLGWRRVPTDNTGLGESAINTEPVIEQVFLTASSRSKVDIERQMYILRRVSMVAIRAALNMQHGAKKDFYICSLSSRTVVYKGQLKPIQLKDYYYADLTHEKFTSYMALIHSRFSTNTFPSWDRAQPMRVLGHNGEINTLRGNINWMRAREGLLKCKELNLSKDEMLKLLPIVDVGSSDSGAFDGVLELLVRAGRTLPEAIMMMIPEAWQNDKNMDPERKALYEYFSALMEPWDGPALISFTDGRYLGATLDRNGLRPGRFYITHSGRVIMASEVGVVDIPPEDVCKKGRLNPGMMLLVDFENHVVVDDDALKRQYSLARPYAEWLNNQKLELKDIVQSVPETERVPPAIAGALPVHKDDDSMENMGIHGLLAPLKAFGYTVEALEMLLLPMAKDSTEALGSMGNDTPLAVMSHRPKLAFEYFKQMFAQVTNPPIDPIREKIVTSMRCMIGPEGDLTETTEEQCHRLSLEGPLLSIDEMEAIKKINYKGWRSKVLDITFSKKHGRKGVEETLDRICNEARAAIREGYTLLVLSDRAFSQDRVAVSSLLAVGAVHHHLVSNLERTRIGLIVETAEPREVHHFCTLVGFGADAICPYLAVETIWRLQVDGKIPPKEDGSFHSKGELVDKYFYACNYGIMKVLAKMGISTLASYKGAQIFEALGLASEVINRCFAGTPSRIEGATFEMLARDTLRLHEMAFPTRALPPGSAETVALPNPGDYHWRKGGEVHLNDPQAIAKLQEAARSNSVAAYREYSKLVENLNKACNLRGMLKFKEIGSSIPLDEVEPASEIVKRFCTGAMSYGSISLEAHTALAVAMNRIGGKSNTGEGGENPSRMVPLPDGSRNPKRSAIKQVASGRFGVSSYYLTNADELQIKMAQGAKPGEGGELPGHKVIGDIAVTRNSTAGVGLISPPPHHDIYSIEDLAQLIHDLKNSNPSARISVKLVSEAGVGVVASGVVKGHADHVLISGHDGGTGASRWTGIKNAGLPWELGLAETHQTLVANDLRGRTVLQTDGQLKTGRDVAIACLLGAEEFGFSTAPLITLGCIMMRKCHKNTCPVGIATQDPVLRAKFAGEPEHVINFFFMLAEEVREIMSQLGLRTINEMVGRADMLEVDQEVVQNNEKLENIDLSALLRPASEIRPGAAQYCVQKQDHGLDMALDNELISLCKVAIEKAVPVYIERSVRNVNRAVGTMLSHEVTKRYHLEGLPSDTVHIKLTGSAGQSFGAFLCSGITLELEGDSNDYVGKGLSGGKVVVYPPRHSRFDPKENIVIGNVALYGAIKGEAYFNGMAAERFCVRNSGAKAVVEGVGDHGCEYMTGGTVVVLGKTGRNFAAGMSGGAAYVLDIENKFPSRCNLELVDLEKVEEEEDITTLRMMIQQHQRHTNSELAKQVLADFDVLLPKFVKVFPRDYKRVLQDMKAKKTAKELEEAELKKKNAFEELRKMSSSAQANGAIKENGPKVFRPTRIGNAIKNGGFVKYEREGIAYRDPAARVNDWKEVIEDARPAELLKTQSARCMDCGTPFCHQETSGCPLGNKIPEFNELVYQNRWREALDRLLETNNFPEFTGRVCPAPCEGSCVLGIIENPVSIKTIECSIIDKAFEEGWMVPRPPLTRTGKKVSIVGSGPAGLAAADQLNKMGHSVTVYERADRIGGLMMYGVPNMKADKVNIVQRRVDLMAKEGVEFVVNANVGKDPAYSLERLRAESDALILACGATRPRDLSVPGREFKGIHFAMEFLHANTKSLLDSNLDDGNYISAKGKKVVVIGGGDTGTDCIGTSIRHGCTQVINLELLPEPPKSRAPGNPWPQWPKIFRVDYGHQEATTKFGKDPRTFEVSTKRFLSDENGAVKGLEVVRVRWEKDANGRFNLKEVEGSEWIIEADLILLAMGFLGPESGVANQLGVDLDNRSNFKAEYGRFKTNVDGVFAAGDCRRGQSLVVWAIAEGRQTAAEVDKYLMKETLVIPQAEEAQENNKQAVAT

>g30432.t1_1|Euryale_ferox/1-2179

MAAVPGVGLQLRNESVLLPSVQGKRGLNAPNPSNKAVCRHVSRDNMFGARVRPSSERLHHVWRSDGPGCTPELKVVRAMGMSGEPTEPLGLYDSAFDKDSCGVGFVAELSGEPSRKTVLDALEMLVRMAHRGACGCEVNTGDGAGILVALPHDFFKEVAKDIGFKLPPPGEYAVGMFFLPTADGRREESKIVFNKVAESLGHQVLGWRRVPTDNTGLGESALNTEPVIEQVFLTASPRSKVDIERQMYILRRVSMVAIRAALNMQHGAQKDFYICSLSSRTVVYKGQLKPIQLKDYYYADLTHEKFTSYMALIHSRFSTNTFPSWDRAQPMRVLGHNGEINTLRGNINWMRAREGLLKCKKLNLSKDEMLKLLPIVDVGSSDSGAFDGVLELLVRAGRTLPEAIMMMIPEAWQNDKNMDPERKALYEYFSALMEPWDGPALISFTDGRYLGATLDRNGLRPGRFYVTHSGRVIMASEVGVVDIPPEDVCKKGRLNPGMMLLVDFENHVVVDDDALKRQYSLARPYAEWLKNQKLELKEIVQSVQETERVPPAIAGALPLHKDDDSMENMGIHGLLAPLKAFGYTIEALEMLLLPMANDATEALGSMGNDTPLAVMSHRPKLAFEYFKQMFAQVTNPPIDPIREKIVTSMRCMIGPEGDLTETTEEQCHRLSLQGPLLSIDEMEAIKKMNYKGWRSKLLDITFSKKRGRKGLEETLDRICNEARAAIREGYTLLVLSDRAFSQERVAVSSLLAVGAVHHHLVSNLERTRIGLIVESAEPREVHHFCTLVGFGADAICPYLAVETIWRLQIDGKIPPKEDGSFHSKGELVDKYFYACNYGIMKVLAKMGISTLASYKGAQIFEALGLASEVIGRCFAGTPSRIEGATFEMLARDTLRLHEMAFPTRVLPPGSAETLALPNPGDYHWRKGGEVHLNDPQAIAKLQEAARSNSVAAYKEYSKLIENLNKACNLRGMLKFKETGSSIPLDEVEPASEIVKRFCTGAMSYGSISLEAHTALAVAMNRIGGKSNTGEGGENPSRMVPLPDGSRNPKRSAIKQVASGRFGVSSYYLTNADELQIKMAQGAKPGEGGELPGHKVIGDIAVTRNSTAGVGLISPPPHHDIYSIEDLAQLIHDLKNSNPSARISVKLVSEAGVGVVASGVVKGHADHVLISGHDGGTGASRWTGIKNAGLPWELGLAETHQTLVANDLRGRTVLQTDGQLKTGRDVAIACLLGAEEFGFSTAPLITLGCIMMRKCHKNTCPVGVATQDPVLRAKFAGEPEHVINFFFMLAEEVREIMSHLGFRTINEMVGRADMLEVDREVVKNNEKLENIDLSALLLPASDIRPGAAQYCVQKQDHGLDMALDNELISLCKVAIDKAVPVYIKRSIRNVNRAVGTMLSHEITKRYHLEGLPSDTVHIKLTGSAGQSFGAFLCPGITLELEGDSNDYVGKGLSGGKIVVYPPRHSRFDPKENIIIGNVALYGAIKGEAYFNGMAAERFCVRNSGAKAVVEGVGDHGCEYMTGGTVVVLGKTGRNFAAGMSGGIAYILDIDNKFPSRCNMELVDLEKVEEEEDIMTLRMMIQQHQRHTNSELAKQVLADFDVLLPKFVKVIPRDYKRVLESMKAKRAAQELEEAELKNKNAFEELRKMSSSGRANGTTKENGPKVFRPTRIGNAIKNGGFVKYEREGIAYRDPAARVKDWKEVIEDARPADLLKTQSARCMDCGTPFCHQETSGCPLGNRIPEFNELVYQNRWKEALDRLLETNNFPEFTGRVCPAPCEGSCVLGIIENPVSIKTIECSIIDKAFEEGWMVPRPPLRRTGKKVSIVGSGPAGLAAADQLNKMGHSVTVYERADRIGGLMMYGVPNMKTDKVNIVQRRVDLMAKEGVEFVVNANVGKDPTYSLERLRTESDALVLACGATRPRDLSVPGREFKGIHFAMEFLHANTKSLLDSNLEDGNYISAKGKKVVVIGGGDTGTDCIGTSIRHGCTQVVNLELLPEPPKSRAPGNPWPQWPKIFRVDYGHQEATTKFGKDPRTYEVLTKRFVSDENGNVKGLEAVRVRWEKDANGRFNLKEVEGSEWIIEADLVLLAMGFLGPESGVANQLGLELDNRSNFKAEYGRFKTSVNGVFAAGDCRRGQSLVVWAIAEGRQAAAEVDKYLTKEALDKPQVEEAQQNNIQTVAT

>g34546.t1_1|Euryale_ferox/1-2101

MGMSGEPTEPLGLYDSAFDRDSCGVGFVAELSGEPSRKTVLDALEMLIRMAHRGACGCEVNTGDGAGILVALPHDFFKEVAKDIGFKLPPPGEYAVGMFFLPTADGRRQESKIVFNKVAESLGHQVLGWRRVPTDNTGLGESALNTEPVIEQVFLTASPRSKVDIERQMYILRRVSMVAIRAALNMQHGAKKDFYICSLSSRTVVYKGQLKPIQLKDYYYADLTHEKFTSYMALIHSRFSTNTFPSWDRAQPMRVLGHNGEINTLRGNINWMRAREGLLKCRELNLSKDEMLKLLPIVDVGSSDSGAFDGVLELLVRAGRTLPEAIMMMIPEAWQNDKNMDPERKALYEYFSALMEPWDGPALISFTDGRYLGATLDRNGLRPGRFYVTHSGRVIMASEVGVVDIPPEDVCKKGRLNPGMMLLVDFENHVVVDDDALKRRYSLARPYAEWLKNQKLELKDIVQSVQETERVPPAIAGALPVHKDDDSMENMGIHGLLAPLKAFGYTIEALEMLLLPMAKDATEALGSMGNDTPLAVMSHRPKLAFEYFKQMFAQVTNPPIDPIREKIVTSRRCMIGPEGDLTETTEEQCHRLSLEGPLLSIDEMESIKKMNYKGWRSKVLDITFSKKRGRKGLEETLDRICNEARTAIREGYTLLVLSDRAFSQDRVAVSSLLAVGAVHHHLVSNLERTRIGLIVESAEPREVHHFCTLAGFGADAICPYLAVETIWRLQVDGKIPPKEDGSFHSKGELVDKYFYACNYGIMKVLAKMGISTLASYKGAQIFEALGLASEVISRCFAGTPSRIEGATFEMLARDTLRLHEMAFPTRVLPAGSAETLALPNPGDYHWRKGGEVHLNDPQAIAKLQEAARSNSVAAYKEYSKLVENLNKACNLRGMLKFKETGSSIPLDEVEPASEIVKRFCTGAMSYGSISLEAHTALAVAMNRIGGKSNTGEGGENPSRMVPLPDGSRNPKRSAIKQVASGRFGVSSYYLTNADELQIKMAQGAKPGEGGELPGHKVIGDIAVTRNSTAGVGLISPPPHHDIYSIEDLAQLIHDLKNSNPSARISVKLVSEAGVGVVASGVVKGHADHVLISGHDGGTGASRWTGIKNAGLPWELGLAETHQTLVANDLRGRTVLQTDGQLKTGRDVAIACLLGAEEFGFSTAPLITLGCIMMRKCHKNTCPVGIATQDPVLRAKFAGKPEHVINFLFMLAEEVREIMSQLGFRTINEMVGRADMLEVDQEVVKNNEKLENIDLSALLRPASKIRPGAAQYCVQKQDHGLDMGLDNELISLCKVAIEKAVPVYIERSVRNVNRAVGTMLSHEITKRYHLEGLPSDTVHIKLTGSAGQSFGAFLCPGITLELEGDSNDYVGKGLSGGKVVVYPPRCSRFDPKENIVIGNVALYGAIKGEAYFNGMAAERFCVRNSGAKAVVEGIGDHGCEYMTGGTVVVLGKTGRNFAAGMSGGMAYILDIENKFPSRCNMELVDLEKVEEEEDIMTLRMMIQQHQRHTNSELAKKVLADFDVLLPKFVKVFPRDYKRVLENMKAKRAAQELEEAELKRKNAFEELRKMSSSARANGTTKENGPKVFRPTRNGNAIKNGGFVKYEREGIAYRDPAARVNDWKEVIEDARPADLLKTQSARCMDCGTPFCHQETSGCPLGNRIPEFNELVYQNRWKEALDRLLETNNFPEFTGRVCPAPCEGSCVLGIIENPVSIKTIECSIIDKAFEEGWMVPRPPLTRTGKKVSIVGSGPAGLAAADQLNKMGHSVTVYELADRIGGLMMYGVPNMKADKVNVVQRRVDLMAKEGIEFVVNANVGKDPTYSLERLRAESDALVLACGATKPRDLSVPGREFKGIHFAMEFLHANTKSLLDSNLEDGNYISAKGKKVVVIGGGDTGTDCIGTSIRHGCTQVINLELLPEPPKSRAPGNPWPQWPKIFRVDYGHQEATTKFGKDPRTYEVATKRFLSDENEAVKGLEVVRVRWEKDANGRFNLKEVEGSKWIIEADLVLLAMGFLGPEPGVANQLGLELDNRSNFKAEYGRFKTSVDGVFAAGDCRRGQSLVVWAIAEGRQAAAEVDKYLTKVAPVIPQIEKAQQNNIQTIAT

>g02873.t1_1|Euryale_ferox/1-2175

MAAVPRVGLQLRNKSVLLPSVQGKRGSSLPSPSNKAVCRHVPRDNNMFGARVRPCSERLHHVWRSDGPGCTPELKVVRAMGMSGEPTEPLGLYDSAFDKDSCGVGFVAELSGEPSRKTVLDALEMLVRMAHRGACGCEVNTGDGAGILVALPHDFFKEVAKDIGFKLPPPGEYAVGMFFLPTVDGRREESKIVFNKVAESLGHQVLGWRRVPTDNTSLGESALNTEPVIEQVFLTASPRSKVDIERQMYILRRVSMVAIRAALNMQHGAKKDFYICSLSSRTVVYKGQLKPIQLKDYYYADLTHEKFTSYMALIHSRFSTNTFPSWDRAQPMRVLGHNGEINTLRGNTNWMRAREGLLKCKELNLSKDEMLKLLPIVDVGSSDSGAFDGVLELLVRAGRTLPEAIMMMIPEAWQNDKNMDPERKALYEYFSALMEPWDGPALISFTDGRYLGATLDRNGLRPGRFYVTHSGRVIMASEVGVVDIPPEDVCKKGRLNPGMMLLVDFENHVVVDDDALKRQYSLARPYAEWLKSQKLEMKDIVQSVHETERVPPAIAGALPVHDDSMENMGIHGLLAPLKAFGYTIEALEMLLLPMAKDATEALGSMGNDTPLAVMSHRPKLAFEYFKQMFAQVTNPPIDPIREKIVTSMRCMIGPEGDLTETTEQQCHRLSLDGPLLSIDEMESIKKMNYKGWRSKVLDITFSKKRGRKGLELTLDRICNEARAAIREGYTLLVLSDRAFSQERVAVSSLLAVGSVHHHLVSNLERTRIGLIVESAEPREVHHFCTLVGFGADAICPYLAVETIWRLQVDGKILPKEDGSFHSKGELVDKYFYACNYGIMKVLAKMGISTLASYKGAQIFEALGLASEVISRCFAGTPSRIEGATFEMLARDTLRLHEMAFPTRVLPPGSAETLALPNPGDYHWRKGGEVHLNDPQAIAKLQEAARGNSVAAYKEYSKLIENLNKACNLRGMLKFKETGNSIPLDEVEPASEIVKRFCTGAMSYGSISLEAHTALAVAMNRIGGKSNTGEGGENPSRMVPLPDGSSNPKRSAIKQVASGRFGVSSYYLTNADELQIKMAQGAKPGEGGELPGHKVIGDIAVTRNSTAGVGLISPPPHHDIYSIEDLAQLIHDLKNSNPSARISVKLVSEAGVGVVASGVVKGHADHVLISGHDGGTGASRWTGIKNAGLPWELGLAETHQTLVANDLRGRTVLQTDGQLKTGRDVAIACLLGAEEFGFSTAPLITLGCIMMRKCHKNTCPVGIATQDPVLRAKFAGEPEHVINFFFMLADEVREIMSQLGFRTINEMVGRADMLEVDQEVVKNNEKLENIDLSALLRPASEIRPGAAQYCVQKQDHGLDMALDNELISLCKVAIEKAVPVYIERSVRNVNRAVGTMLSHEFTKRYHLEGLPSDTVHIKLTGSAGQSFGAFLCPGVTLELEGDSNDYVGKGLSGGKVVVYPPRHSRFDPKENIVIGNVALYGAIKGEAYFNGMAAERFCVRNSGAKAVVEGVGDHGCEYMTGGTVVVLGKTGRNFAAGMSGGVAYILDIDNKFPSRCNMELVDLEKVEGEEDIMTLRMMIQQHQRHTNSELAKQVLADFDVLLPKFVKVFPRDYKRILENMKAKRAAQELEEAELKKNNALEELRKMSARANGTTKENGPKVFRLTRTGNAIKNGGFMKYEREGIAYRDPAARVNDWKEVIEDARPDILLKTQSARCMDCGTPFCHQEASGCPLGNRIPEFNELVYQNRWKEALDRLLETNNFPEFTGRVCPAPCEGSCVLGIIENPVSIKTIECSIIDKAFEEGWMVPRPPLTRTGKKVSIVGSGPAGLAAADQLNKMGHSVTVYERADRIGGLMMYGVPNMKANKVNIIERRVDLMAKEGIEFVVNANVGKDPTYSLERLRAESDALVLACGATKPRDLSVPGREFKGIHFAMEFLHANTKSLLDSNLEDGNYISAKGKKVVVIGGGDTGTDCIGTSIRHGCTQVINLELLPEPPKSRAPGNPWPQWPKIFRVDYGHQEATSKFGKDPRTFEVSTKRFLSDENGAVKGLEVVRVRWEKDANGRFNLKEVEGSEWIIKADLVLLAMGFLGPESGVANQLGLELDNRSNFKAEYGRFKTNVDGVFAAGDCRRGQSLVVWAIAEGRQAAAEVDKYLTKEALVIPQVEKAQQNIQTVAT

>AUR62019375|Cquinoa_v1.0/1-2194

MSVAAGVGSVLNLRTKVGSDDHGGLVVVQPQLCRFGKRSSRSYAVRRSEAKICESKIFGNRLRSERLRPLLRDPNSRFVIQSALSLVPEQPLGLYDPKNDKDSCGVGFVAELSGECSLEMLVRMSHRGACGCETNTGDGAGILVGLPHDFFTEVTKDLGFQLPPLGEYAVGMFFLPTSESRREESKNVFTKVAESLGHTVLGWRPVPTDNSGLGKSALMTEPVVEQVFLTPTPRSKADLEQQMYILRRVSMVAIRAALNLQHGGVKDFYICSLSSRTIVYKGQLKPSQLKEYYYWDLGNERFTSYMALVHSRFSTNTFPSWDRAQPMRVLGHNGEINTLRGNVNWMRAREGLLKCKELGLSKNEMKKLLPIVDASSSDSGAFDGVLELLVRAGRSIPEAVMMMIPEAWQNDKNMDPHRKGFYEYLSALMEPWDGPALITFTDGRYLGATLDRNGLRPGRFYITHSGRVIMASEVGVVDISPEDVSKKGRLNPGMMLLVDFDKHIVVDDDALKKQYSMARPYGEWLKRQKLVLKDIVDSVAETERVAPAISGAVPVSNLDEDMENMGIHGLLAPLKAFGYSVEALEMLLIPMAKDATEALGSMGNDTPLAVMSNREKLTFQYFKQLFAQVTNPPIDPIREKIVTSMECMIGPEGDLTEMTELQCHRLSLKGPLLTIEEMEAVKKMDYRGWRSKVIDITYPKTLGRKGLEQTLDRISAEARDAIKEGYTVLVLSDRAFSPSRVAVSSLLAVGAVHQHLVKTLERTRVGLIIESAEPREVHHFCTLVGFGVDAICPYLAIETIWRLQVDGKIPPKSTGEFRSKDELIKRYFKASNYGMMKVLAKMGISTLASYKGAQIFEAVGLSSEVIERCFTGTASRVEGATFVMLASDALKLHEMAFPRRALPFGSAESVALPNPGDYHWRKDGELHLNDPLAMAKLQEAAKSNSVGAYKEYSKRINELNKSSNLRGLLKFKEMKDQVPLEEVEPASEIVKRFCTGAMSYGSISLEAHSALAIAMNKLGGKSNTGEGGENPSRLEPLPDGSMNPKRSAIKQVASGRFGVSSYYLTNADELQIKMAQGAKPGEGGELPGHKVIGDIAVTRNSTAGVGLISPPPHHDIYSIEDLAQLIHDLKNANPSARVSVKLVSEAGVGVIAAGVVKGHADHVLISGHDGGTGASRWTGIKNAGLPWELGLAETHQTLVANDLRGRTVLQTDGQLKTGRDVAIAALLGAEEFGFSTAPLITLGCIMMRKCHKNTCPVGIATQDPVLRAKFAGEPEHVINFFFMLAEEVREIMSQLGFRKLDEMVGRSDMLEVDEEVTKSNEKLKNIDLSLLLRPAATIRPGAAQRCVQKQDHGLDMALDQELIVSSKAALEKALPVYIETPIKNVNRAVGTMLSHEVTKRYHLEGLPTDTIHIKFNGSAGQSLGAFLCPGITLELEGDSNDYVGKGLSGGKIVVYPPRSSGFDPKENIVIGNVALYGATCGEAYFNGMAAERFCVRNSGAKAVVEGVGDHGCEYMTGGTVVVLGKTGRNFAAGMSGGIAYVLDLDGKFHSRCNSELVDLDKVEEEEDITSLRIMIQQHQRHTNSKLAQEVLASFDTLLPKFIKVFPRDYKRVLASMKTDGASQETGGKVAEEEKDEIEVTEKDAFEELKKMASSALQEQDSQEEDEVEEFKRPSRVPDAQKHRGFVAYEREGIQYRDPNERMKDYNEVMDEKRPAPLLKTQAARCMDCGTPFCHQDNTGCPLGNKIPEFNELVYQNRWREALDRLLETNNFPEFTGRVCPAPCEGSCVLGIIENPVSIKTIECSIIDKAFEEGWMVPRPPQKRTGKKVAVVGSGPSGMAAADQLNKMGHFVTVYERADRIGGLMMYGVPNMKTDKVNVVQRRVNLMAEEGVQFVVNANVGKDPQYSIDRLKEENDAIVLAVGATKPRDLPIEGRDLSGIHFAMEFLHANTKSLLDSNLQDGNYISAKGKKVVVIGGGDTGTDCIGTSIRHGCTNVVNLELLPQPPNSRAPGNPWPQWPRIFRVDYGHQEAAAKFGKDPRSYEVLTKRFVGDENGKLKGLELVHVVWEKDASGKFQFKEVEGSQEFIEADLVLLAMGFLGPEANVADKLGVEKDNRSNFKADYGRFSTNVEGVFAAGDCRRGQSLVVWAISEGRQAASQVDRYLMGEEKEVDVSTPVEEVAGKRQLESDKRTVMT

>AUR62006114|Cquinoa_v1.0/1-2202

MSVAAGVGSVLNLRSKVGSENHGGLVVVQPQLCRFGKRSSRSYAVRRSEAKICESKIFGNRLRSERLRPLLRDPNSRFVIRSALSQVPEQPLGLYDPKNDKDSCGVGFVAELSGECSRKTVTDAVEMLVRMAHRGACGCETNTGDGAGILVGLPHDFFTEVTKDLGFELPPLGEYAVGMFFLPTSESRREESKNVFTKVAESLGHTVLGWRPVPTDNSGLGKSALMTEPVVEQVFLTPTPRSKADLEQQMYILRRVSMVAIRAALNLQHGGVKDFYICSLSSRTIVYKGQLKPSQLKEYYYWDLGNERFTSYMALVHSRFSTNTFPSWDRAQPMRVLGHNGEINTLRGNVNWMRAREGLLKCKELGLSKNEMKKLLPIVDASSSDSGAFDGVLELLVRAGRSIPEAVMMMIPEAWQNDKNMDPQRKGFYEYLSALMEPWDGPALITFTDGRYLGATLDRNGLRPGRFYITHSGRVIMASEVGVVDISPEDVSKKGRLNPGMMLLVDFDKHIVVDDDALKKQYSTARPYGEWLKRQKLVLKDIVDSVAETERVAPAISGAVPVSNLDEDMENMGIHGLLAPLKAFGYSVEALEMLLIPMAKDATEALGSMGNDTPLAVMSNREKLTFQYIKQLFAQVTNPPIDPIREKIVTSMECMIGPEGDLTEMTELQCHRLSLKGPLLSIEEMEAVKKMDYRGWRSKVIDITYPKTLGRKGLEQTLDRISAEARDAIKEGYTVLVLSDRAFSPSRVAVSSLLAVGAVHQHLVKTLERTRVGLIIESAEPREVHHFCTLVGFGVDAICPYLAIETIWRLQVDGKIPPKSTGEFRSKDELIKRYFKASNYGMMKVLAKMGISTLASYKGAQIFEAVGLSSEVIERCFTGTASRVEGATFEMLASDALKLHEMAFPRRALPFGSAESVALPNPGDYHWRKDGELHLNDPLAMAKLQEAAKSNSVGAYKEYSNRINELNKSSNLRGLLKFKEMKDKVPLEEVEPVSEIVKRFCTGAMSYGSISLEAHSALAIAMNKLGGKSNTGEGGENPSRLEPLPDGSMNPKRSAIKQVASGRFGVSSYYLTNADELQIKMAQGAKPGEGGELPGHKVIGDIAVTRNSTAGVGLISPPPHHDIYSIEDLAQLIHDLKNANPSARVSVKLVSEAGVGVIAAGVVKGHADHVLISGHDGGTGASRWTGIKNAGLPWELGLAETHQTLVANDLRGRTVLQTDGQLKTGRDVAIAALLGAEEFGFSTAPLITLGCIMMRKCHKNTCPVGIATQDPVLRAKFAGEPEHVINFFFMLAEEVREIMSQLGFRKLDEMVGRSDMLEVDEEVTKSNEKLKNIDLSLLLRPAATIRPGAAQRCVQKQDHGLDMALDQELIVSSKAALEKALPVYIETPIRNVNRAVGTMLSHEVTKRYHLEGLPTDTIHIKFNGSAGQSLGAFLCPGITLELEGDSNDYVGKGLSGGKIVVYPPRSSGFDPKENIVIGNVALYGATCGEAYFNGMAAERFCVRNSGAKAVVEGVGDHGCEYMTGGTVVVLGKTGRNFAAGMSGGIAYVLDLDGKFHSRCNSELVDLDKVEEEEDITTLRIMIQQHQRHTNSKLAQEVLASFDTLLPKFIKVFPRDYKRVLASMKTDGASQETGGKVAEEEKDEIEVTEKDAFEELKKMATSALQEQDSQEEDEVEEFKRPSRVPDAQKHRGFVAYEREGIQYRDPNERMKDYNEVMDEKRPAPLLKTQAARCMDCGTPFCHQDNSGCPLGNKIPEFNELVYQNRWREALDRLLETNNFPEFTGRVCPAPCEGSCVLGIIENPVSIKTIECSIIDKAFEEGWMVPRPPQKRTGKKVAVVGSGPSGMAAADQLNKMGHFVTVYERADRIGGLMMYGVPNMKTDKVNVVQRRVNLMAEEGVQFVVNANVGKDPQYSIDRLKDENDAIVLAVGATKPRDLPIEGRDLSGIHFAMEFLHANTKSLLDSNLQDGNYISAEGKKVVVIGGGDTGTDCIGTSIRHGCTNVVNLELLPQPPNSRAPGNPWPQWPRIFRVDYGHQEAAAKFGKDPRSYEVLTKRFVGDENGKLKGLELVHVMWEKDASGKFQFKEVEGSQEFIEADLVLLAMGFLGPEANVADKLGVEKDNRSNFKADYGRFSTNVEGVFAAGDCRRGQSLVVWAISEGRQAASQVDRYLMGEEKEVVDVSSPVEEVAGKRQLESDKRTVMT

>TnS000029905t04|Gnetum_montanum/1-2206

MSVCGGSGGTGVLGSLTKSSRCGMISLSGGSASVGVAGGVRWWRRIGVGGGVGRRGCVSGRLECSNSNSNSYVRLHGCTHKQMLWRACARGRLSRLRPMAALSEVPSEALGLYDPAFDKDSCGVGFVAELSGRPSRKTVDDAIIMLERMAHRGACGCETNTGDGAGIMVALPHEFLAEAAKDAGFQLPEAGKYAVGMFFLPTSETRREESKNVFSKVAESLGHRVLGWRRVAVDNSGLGNSALQTEPIIEQVFLTASPRSRGDFEQQMYVLRRTSMVAIRAALNLQHGSVKDFYICSLSSRTIVYKGQLKPDQLRSYYYADLGHEKFTSYMALVHSRFSTNTFPSWDRAQPMRVLGHNGEINTLRGNVNWMKAREGLLTCKNLNLTKDEMQKLLPIVDVSSSDSGAFDGVLELLVRSGRSLPEAIMMMIPEAWQNDKNMDPDRRALYEYFSALMEPWDGPALIAFTDGRYLGATLDRNGLRPGRYYVTHSGRVIMASEVGVVDLPPEDVSKKGRLNPGMMLLVDFENHVVVDDDALKKQYSLARPYQEWLKRQKITLHDIVDSVPQTERQAPPISGMAKADMSDNNMDNMGIHGLLAPLKAYGYTVESLEMLLLPMAKDGTEALGSMGNDAALTVMSNRPKLIFEYFKQMFAQVTNPPIDPIREAIVTSTECMIGPEGDLTETSEGQCHRLSLKSPLLSIEEMECMKKMHFRGWRTKVLDITFPKERGPKGLEETLDRICAEARQAIREGFTTLVLSDRATSPERVPVTSLLAVGAVHQHLVSNLQRTRIGLIVETGDAREVHHFCTLVGFGADAICPYVAIEAIWRLQIDGKIPPRKNGSIHSKNDLVKKYFKASNSGMLKVLAKMGISTLASYKGAQIFEALGLSSDVVQRCFAGTPSRVEGATFEMLARDAIRLHELAFPVRGTSDSNVLPNPGDYHWRKGGEVHMNDPLAIAKLQEAARTNSVTAYKEYSKLIQNLNKRCTLRGMLKFKEMPERKIPLEEVEPASEIVKRFCTGAMSYGSISLEAHTSLAIAMNRIGGKSNTGEGGENPSRLVPLSDGSMNPKRSAIKQVASGRFGVSSYYLTNADELQIKMAQGAKPGEGGELPGHKVIGDIAVTRNSTAGVGLISPPPHHDIYSIEDLAQLIYDLKNSNPGARISVKLVSEAGVGVVASGVVKGHADHVLISGHDGGTGASRWTGIKNAGLPWELGLAETHQTLVANDLRGRTILQTDGQLKTGRDVAIAALLGAEEFGFSTAPLITLGCIMMRKCHKNTCPVGIATQDPILREKFAGEPEHVINFFFMLAEEMREIMSQLGFKTLNEMVGRSDMLEMDKEVINSNEKLTNIDLSLLLRPAADIRPNAAQYCVQTQNHGLDMALDQQLISLSQPALEKGFPVYIEMDIMNTNRAFGTMLSHEVTKRYHMQGLPTDTIHVKLRGSAGQSLGAFLCSGITLELEGDGNDYVGKGLSGGKIVVYPPRSSTFDPKENIVIGNVALYGATSGEAYLNGMAAERFCVRNSGAKAVVEGVGDHGCEYMTGGTVVILGKTGRNFAAGMSGGIAYVFDVDGKFNLRCNHELVDLEKVEDNDDIMTLKMLIQQHQRHTNSELAKEILSDFKSLLPNFVKVFPRDYKRILQDMKMEKAAKEAEEAKLMQTDAFEELKKMASAGSKQPHTNEKYLRDEHAIVSQRPTKVEKAVKDRGFVMYERQSISYRDPADRISDWKEVAQESKPGPLLKTQTARCMDCGTPFCHQENSGCPLGNKIPEFNELVYQGRWQEALDRLLETNNFPEFTGRVCPAPCEGSCVLGIIENPVSIKSIECAIIDKAFEEGWMVPRPPSNRTGKRISIVGSGPAGLAAADQLNKAGHFVTVYERSDRIGGLMMYGVPNMKTDKIDVVQRRVDLMEREGVSFVVNANVGFDPKYSVETLMNDSDAILLACGATKPRDLPIPGREFKGIHFAMEFLHSNTKSLLDSALQDGKYISAKGKKVVVIGGGDTGTDCVGTSIRHGSTNVVNLELLSKPPKSRAPGNPWPQWPRIFRVDYGHEEAITKYGKDPRTYEVLTKRFINDGNGNVKGLEVVKVNWEKDMTGKFQLKELVGTEEIIEADLVLLALGFVGPEQYVAEKLGLESDNRSNFKANYGEFATNIDGVFAAGDCRRGQSLVVWAIAEGRSAAAEIDKYVMKDLPQEQNDETLEDPEKESLFTLKA

>Zosma01g22390|Zmarina_v3.1/1-2188

MSAIPGGAVFRDPCFGTFRNYRGGSIINPKSACRIVRLHNAPKQSSMLTMDSRFWGKSLKLQQASSQGRCHIWRSDGLGLTPNLRVAGATMSLSLLPEKPVGLYDPSFEKDSCGVGFVAELSGESRRKTVTDALEMLIRMSHRGACGCEANTGDGAGILVALPHDFYKEVTMELGFELPGLGGYSVGMFFLPTSPERREQSKIVFSKVAESLGHVVLGWRLVPTNNTNLGKSVVDTEPVIEQVFLTPSPRSNADFEQQMYILRRVSMVAIRAALNLQYGEVKDFYICSLSSRTVVYKGQLKPDQMMEYYYADLGSEMFLSYMAMVHSRFSTNTFPSWDRAQPMRVLGHNGEINTLRGNVNWMKARQGLLKCKDLGLSDNEMKKLLPIVDTNSSDSGAFDGVLELLVRAGRSMPEAIMMMIPEAWQNDENMDEDKKALYEYFSSLMEPWDGPALISFTDGRYLGATLDRNGLRPGRYYITKSGRVVMASEVGVVDIPPYDVCKKGRLNPGMMLLVDFENGIVVDDEALKKQYSLARPYKHWIESQKISLKDIVDSVPESMRICPSINGVLCNNENTENIGIHGLLAPLKAFGYTVEALEMLLLPMARDASEALGSMGNDTPLAVMSNRDRLPFEYFKQMFAQVTNPPIDPIREKIVTSMECMIGPEGDLTETTEEQCHRISLKGPLLSIDEMEAIKKMDYKGWRSKILDITYTKIKGRNGLEETLDRICKEAHTAIQEGYTTLVLSDRGFSADKVAVSSLLAVGAVHQHLVSTFERTRIGLVVESAEPREVHHFCTLVGFGADAICPYLAVETIWRLQIDGKIPPKEDGLFHSQEELTQRYFTASNYGMMKVLAKMGISTLASYKGAQIFEALGLSSAVVDKCFKGTPTRVEGATFEMLAKDALHLHEMGFPSRMLPPGSADATALPNPGDYHWRKDGELHLNDPLAMSKLQEAARSNSVAAYKEYSKRIQQLNKACNLRGVLKFVDNNNKVSLDEVEPASEIVKRFATGAMSYGSISLEAHSALAVAMNKLGGKSNTGEGGENPSRMKPLSDGSMNPKRSAIKQVASGRFGVSSYYLTNADELQIKMAQGAKPGEGGELPGHKVIGDIAVTRNSTPGVGLISPPPHHDIYSIEDLSQLIHDLKNANPRARISVKLVSEAGVGVIASGVVKGHADHILISGHDGGTGASRWTGIKNAGLPWELGLAETHQTLVINNLRGRTTLQTDGQLKTGRDVVIAALLGAEEFGFSTAPLITLGCIMMRKCHKNTCPVGIATQDPVLREKFAGEPENVINFFFMLAEEVREIMAELGFRTINEMVGRSDMMEVDKEVIESNEKLKNINLGALLSPSFKQRPGSAQYCVEKQDHGLDMALDQKLISLCTDAIEQAMPVYVEVPICNVNRAVGTMLSHEVTKRYHMDGLPTDTIHIKFNGSAGQSLGAFLCSGITLELEGDSNDYVGKGLCGGKIVVYPPSESTFLPKENIVIGNVALYGATSGEAYFNGMAAERFCVRNSGARAVVEGIGDHGCEYMTGGTVVILGKTGRNFAAGMSGGIAYVLDEDETFHTRCNMELVDLDKVENYEDIATLRMMIQQHQRHANSSLAKEILSNFDELLPRFVKVFPRDYKRVLENLKAEKAAKEAEEEEEYELNQKDAFEELKKLAVTSLTESNNSLDRPTRVSGAIKHRGFINYERESISYRDSNARINDWKEVAVESKPGPLLKTQSARCMDCGTPFCHQDNSGCPLGNKIPEFNELVHQERWREALDRLLETNNFPEFTGRVCPAPCEGSCVLGIIENPVSIKSIECAIIDKAFEEGWMVPRPPLKRTGEKIAIIGSGPSGLAAADQLNKMGHSVTVYERSDRIGGLMMYGVPNMKTDKIDVVQRRVDLMAKEGIEFVVNANIGIDPLYSIERIRYENSAIVLACGATKPRDLPVPGREFSGVHFAMEFLHANTKSLLDSNLEDEKYISAKGKKVVVIGGGDTGTDCIGTSIRHGCTDIVNLELLPQPPQTRASGNPWPQWPRVFRVDYGHQEAATKFGKDPRTYEVLTKRFIGDENGAVKGLEVVSVQWVKDASGRFQFEEIAGSEEIIEADLVLLAMGFLGPEKNVAEKLGLELDSRSNFKAEYGEFSTNVNGVFAAGDCRRGQSLVVWAISEGRQAASAVDSFLMKKISNDGENTSNKSEFDGRQKIVA

>158084|Smoellendorffii_v1.0/1-2065

MYRCVVTMSSMSQVPEGPLGLYDPALDKDSCGVGFVAELSAKPSRQTVNEIGCELPPPSEYAVGMFFLPTSETRRMESKNVFNKVAESLGHTVLGWRSVVTDNSDLGKSALGTEPVVEQVFLTGSSRSSASLEQQMYILRKLSMVAIRAALNLQHGAVRDFYICSLSSRTVVYKGQLKPDQLKKYYASDLCHEKFTSYMALVHSRFSTNTFPSWDRAQPMRVLGHNGEINTLRGNVNWMRAREGLLKCRALGLSNDELQKLLPIVDASSSDSGSFDGVLEFLVRSGRSLPEAMMMMIPEAWQNDKNMDPERRALYEYFSALMEPWDGPALIAFSDGRYLGATLDRNGLRPGRYYITHSGRVIMGSEVGVVDVPPQDVSKKGRLNPGMMLLVDFENHAVVDDDALKKEYSTRQPYAEWLQHQKILLKDIVDSVSEDCTSPRIIGTLQPKKTDATRDNMGIHGLVPPLKAFGYTVEALEMLLLPMAKDGVEALGSMGNDAPLAVMTDRPKLSFEYFKQMFAQVTNPPIDPIREAIVTSTECMIGPEGDLTESTEEQCHRLSLKGPLISPLEMEALKKMNYQGWRSKIIDITFPREEGPTGLEKALDRICVEARKAISKGFKMLVLSDRGTSPERVPVSSLLAVGTVHHHLVSTLERTRIGLVVESGEPREVHHFCTLLGFGADAICPYLAVEAIWRLQVDGKIPSKENGVLSSKDELVQKYFKASNSGILKVLAKMGISTLASYKGAQIFEALGLSSDVVQRCFRGTPSRVEGATFEMLAKDSLRLHELAFPSRKLPPGSAEAHALPNPGDYHWRKDGEIHLNDPLAMAKLQEAARTNSVGAYKDYSKRIYELNKKCSLRGMLKFKASKTPVALEEVEPASEIVKRFCTGAMSYGSISLEAHTTLAMAMNQIGGKSNTGEGGENPSRLEALPNGQMNPKRSAIKQVASGRFGVTSYYLTNADELQIKMAQGAKPGEGGELPGHKVIGDIATTRNSTPGVGLISPPPHHDIYSIEDLAQLIYDLKNSNPGARISVKLVSEAGIGVVASGVVKGHADHVLISGHEGGTGASRWTGIKNAGLPWELGLAETHQTLVANDLRGRTTLQTDGQLKTGRDIVIAALLGAEEFGFSTAPLITLGCIMMRKCHKNTCPVGIATQDPVLREKFAGEPEDVINFFFMVAEEARELMAEMGFRTMDEMVGRADMLEVDKSVTASNEKLKNIDLSLLLRPAADIRPDAAQRCVQKQDHDLGMALDQQLIKGSKPALENGYPVYLESPVVNVNRAVGTMLSHEVTKKYKMEGLPADTIHVKLTGNAGQSLGAFLCRGITLELEGDSNDYVGKGLCGGRVVVYPPRDSGFDPKENIVIGNVALYGATGGEAYFNGMAAERFCVRNSGAKAVVEGVGDHGCEYMTGGTVVILGKTGKNFAAGMSGGIAYVYDVDGKFRLRCNPGQVDLEDVLEDEDVLTLRAMIQQHQRHTKSQHAKQILDEFDSALPKFVKVFPCDYKRALNEEKAKKRAAEEEEARLAAVDAFQELKKMAEAATNGGAAATPAKARPTKIPDAVKHRGFISYERETLPYRPAQERMQDWNEVLATARNDDLLKTQSARCMDCGTPFCNQDNSGCPLGNKIPEWNELVHQGRWREALDRLLETNNFPEFTGRVCPAPCEGACVLGIIENPVSIKTMECTIIDKAFEEGWMVPRPPAYRTGKRVAIVGSGPAGLAAADQLNKAGHSVTVFERADRIGGLMMYGVPNMKTDKVEVVQRRVNLMAAEGIEFIVNANVGVDKLFSVERLMSDYDSLLLACGATKPRDLKVEGREYEGIHFAMEFLHANTKSLLDSGLKDNNYISAKDKKVVVIGGGDTGTDCIGTAVRHGCKSVVNLELLPQPPATRAPGNPWPQWPRVFRVDYGHAEAKEKFGSDPRSYEVTTKKFITDGDNRVSGLVLKRVRWFKDASGRFQREEIEGSEEIIEADLVLLAMGFVGPEQDVAEKLKVATNDAATTFKAEYGKFATNVKGVFAAGDCRRGQSLVVWAIAEGREAAAQIDKFLMEDTSKVGEELKAARKKMGMLI

>evm.model.Chr20.3740|Isoetes_sinensis/1-2209

MLTISRSVLSNNCASYAQAYHTGLSSSSRLQCQTVSVAPSDASRKYCNPQIRSSLESASGSWAPEYTGLRCQKGPFQGARHRLDKQRLKWWLWEGKAKVWRSMRMALSEIPEGPLGLYDPSLDKDACGVGFVAELSAQPSRKTVDDALEMLVRMAHRGACGCEINTGDGAGILVALPHSFFAVVAKEELGVDLPPPSKYAVGMLFLPTSETRRMESKNVFTQVAESLGHTVLGWRRVKTDNSDLGNSARRTEPVIEQVFITASSRSTAGLEEQTYVLRKTSMVAIRAALNLQHGGVKDFYICSLSSRTIVYKGQLKPNQLRNYYFADLNHELFTSYMAMVHSRFSTNTFPSWDRAQPMRVLGHNGEINALRGNVNWMRAREGLLKCKALGLSNDEMQKLLPIIDASSSDSGSFDGVLELLVRCGRALPEAMMMMIPEAWQNDDHMDPDRRALYEYFSAIMEPWDGPALISFTDGRYLGATLDRNGLRPGRYYVTHSGRVVMASEVGVVDIAPEDISRKGRLNPGMMLLVDFENHKVVDDDELKREYSLRHPYAEWLKQQKLLLKDVVASVRESERKAPLINGSIQVKSSDVSMENMGVHGLVAPLKAFGYTVEALEMLLLPMARDGVEALGSMGNDTPLAVMAGRPKLIFEYFKQMFAQVTNPPIDPIREAIVTSTECMVGPEGDLTECTEKQCHRLSLKGPLLSVTEMEAIKKMNYKGWRSKIIDITFPREQGVEGVEKALDRICAEARAAITQGYKMLVLSDRATSSRNVAVSSLLAVGAVHHHLVSTLERTRIGLVLESGEPREVHHFCTILGFGADAICPYLAIEAIWRLQVDGKISPKSDGTLHTQDELVSKYFKASNSGILKVLAKMGISTLASYKGAQIFEALGLSSEVVQRCFKGTPSRVEGATFEMLARDSLALHDMAFPSRAMPEGSPEAHALPNPGDYHWRKDGEIHLNDPLAMAKLQEAARTNSVGAYKEYSKRIHELNKKSNLRGMLKFKNISEKEIPLEEVEPASEIVKRFCTGAMSYGSISLEAHTTLARAMNKIGGKSNTAGEGGENPARLEPLPDGSMNPKRSAIKQVASGRFGVTSYYLTNADELQIKMAQGAKPGEGGELPGHKVIGDIAVTRNSTPGVGLISPPPHHDIYSIEDLAQLIYDLKNSNPGARISVKLVSEAGVGVVASGVVKGHADHVLISGHDGGTGASRWTGIKNAGLPWELGLAETHQTLVANDLRGRTVLQTDGQLKTGRDIVIAALLGAEEFGFSTAPLITLGCIMMRKCHKNTCPVGIATQDPVLREKFAGEPEHVINFLFMVAEEARELMASMGFRNMDEMIGRADMLEVDKEVISGNEKLKNIDLSLLLQPAAEIRPDAAQRCIQKQDHGLDMALDNQLIASSQAAIEKAIPVYIEAGIVNVNRAVGTMLSHEVTKKYDLKGLPNDTIHVKLTGSAGQSFGAFVCSGITLELEGDSNDYVGKGLCGGKVVVYPPRNSGFDPKENIVIGNVALYGATSGEAYFNGMAAERFCVRNSGAKAVVEGVGDHGCEYMTGGIAVILGKTGRNFAAGMSGGIAFVLDVDGRFKERCNRGLVDLYAVESEADVMTLKMMIQQHQRHTKSQLATHVLENFDTMLPQFVKVFPRDYKRVLEETKAKEAAKKEEEELMKKDAFEELKKMAQQQAEVVPTVSVAKELKERPIDIKDAVKNGGFIKYERSTLKYRPAEERLNDWNEVLDDARPDGLLKTQSARCMDCGTPFCHQEISGCPLGNKIPEWNELVHQGRWREALDRLLETNNFPEFTGRVCPAPCEGACVLGIIENPVSIKTIECTIIDKAFEEGWMVPRPPLKRTGKRIAIVGSGPAGLAAADQLNKAGHLVTVFERADRVGGLMMYGVPNMKADKVEVVQRRVDLMAAEGVEFIVNAHVGQDPLYSIEKLRSDYNALLLACGATKPRDLVSVPGRDLGGIYFAMDFLHANTKSLLDSNLEDGHYISAKGKRVIVIGGGDTGTDCIGTSLRHGCKSLVNLELLPQPPQSRAPGNPWPQWPKVFRVDYGHEEAKTRFGKDPRTYEVLTKRFIGNDEGIVTGLEIKHVKWSKDAAGRLQMEEVEGSEEILEADLVLLAMGFLGPEQNIAEKLELERDNRSNFKADFGRFETSLQGVFAAGDCRRGQSLVVWAIAEGRQAAAQIDKFLMKSIEAEELEKCIREGLKVQQKM

>evm.model.Chr19.3944|Isoetes_sinensis/1-2208

MLTISRSVLSNNCASYAQAYHTGLSSSSRLQCQTVSVAPSDASRKYCNPQIRSSLESGSGSWAPEYTGLRCQKGPFQGARHRLDKQRLKWWLWEGKAKVWRSMRMALSEIPEGPLGLYDPSLDKDACGVGFVAELSAQPSRKTVDDALEMLVRMAHRGACGCETNTGDGAGILVALPHSFFAVVAKEELGVDLPPPSKYAVGMLFLPTSETRRMESKNVFTQVAESLGHTVLGWRRVKTDNSDLGNSARRTEPVIEQVFITASSRSTAGLEEQTYVLRKTSMVAIRAALNLQHGGVKDFYICSLSSRTIVYKGQLKPNQLRNYYFADLNHELFTSYMAMVHSRFSTNTFPSWDRAQPMRVLGHNGEINALRGNVNWMRAREGLLKCKALGLSNDEMQKLLPIIDASSSDSGSFDGVLELLVRCGRALPEAMMMMIPEAWQNDDHMDPDRRALYEYFSAIMEPWDGPALISFTDGRYLGATLDRNGLRPGRYYVTHSGRVVMASEVGVVDIAPEDISRKGRLNPGMMLLVDFENHKVVDDDELKREYSLRHPYAEWLKQQKLLLKDVVASVRESERKAPLINGSIQVKSSDVSMENMGVHGLVAPLKAFGYTVEALEMLLLPMARDGVEALGSMGNDTPLAVMAGRPKLIFEYFKQMFAQVTNPPIDPIREAIVTSTECMVGPEGDLTECTEKQCHRLSLKGPLLSVTEMEAIKKMNYKGWRSKIIDITFPREQGVEGVEKALDRICAEARAAITQGYKMLVLSDRATSSRNVAVSSLLAVGAVHHHLVSTLERTRIGLVLESGEPREVHHFCTILGFGADAICPYLAIEAIWRLQVDGKISPKSDGTLHTQDELVSKYFKASNSGILKVLAKMGISTLASYKGAQIFEALGLSSEVVQRCFKGTPSRVEGATFEMLARDSLALHDMAFPSRAMPEGSPEAHALPNPGDYHWRKDGEIHLNDPLAMAKLQEAARTNSVGAYKEYSKRIHELNKKSNLRGMLKFKNISEKKIPLEEVEPASEIVKRFCTGAMSYGSISLEAHTTLARAMNKIGGKSNTGEGGENPARLEPLPDGSMNPKRSAIKQVASGRFGVTSYYLTNADELQIKMAQGAKPGEGGELPGHKVIGDIAVTRNSTPGVGLISPPPHHDIYSIEDLAQLIYDLKNSNPGARISVKLVSEAGVGVVASGVVKGHADHVLISGHDGGTGASRWTGIKNAGLPWELGLAETHQTLVANDLRGRTVLQTDGQLKTGRDIVIAALLGAEEFGFSTAPLITLGCIMMRKCHKNTCPVGIATQDPVLREKFAGEPEHVINFLFMVAEEARELMASMGFRNMDEMIGRADMLEVDKEVISGNEKLKNIDLSLLLQPAAEIRPEAAQRCIQKQDHGLDMALDNQLIASSQAAIEKAIPVYIEAGIVNVNRAVGTMLSHEVTKKYDLKGLPNDTIHVKLTGSAGQSFGAFVCSGITLELEGDSNDYVGKGLCGGKVVVYPPRNSGFDPKENIVIGNVALYGATSGEAYFNGMAAERFCVRNSGAKAVVEGVGDHGCEYMTGGIAVILGKTGRNFAAGMSGGIAFVLDVDGRFKERCNRGLVDLYAVESEEDVMTLKMMIQQHQRHTKSQLATHVLENFDTMLPQFVKVFPRDYKRVLEETKAKEAAKKEEEELMKKDAFEELKKMAQQQAEVVPTVSVAKELKERPIDIKDAVKNGGFIKYERSTLKYRPAEERLNDWNEVLDDARPDGLLKTQSARCMDCGTPFCHQEISGCPLGNKIPEWNELVHQGRWREALDRLLETNNFPEFTGRVCPAPCEGACVLGIIENPVSIKTIECTIIDKAFEEGWMVPRPPLKRTGKRIAIVGSGPAGLAAADQLNKAGHLVTVFERADRVGGLMMYGVPNMKADKVEVVQRRVDLMAAEGVEFIVNAHVGQDPLYSIEKLRSDYNALLLACGATKPRDLLSVPGRDLGGIYFAMDFLHANTKSLLDSNLEDGHYISAKGKRVIVIGGGDTGTDCIGTSLRHGCKSLVNLELLPQPPQSRAPGNPWPQWPKVFRVDYGHEEAKTRFGKDPRTYEVLTKRFIGNDEGIVTGLEIKHVKWSKDAAGRFQMEEVEGSEEILEADLVLLAMGFLGPEQNIAEKLELERDNRSNFKADFGRFETSLQGVFAAGDCRRGQSLVVWAIAEGRQAAAQIDKFLMKSIEAEELEKCIREGLKVQQKM

>Adc04615|Adiantum_capillus-veneris/1-2206

MAASISAPGAWAVSDARPFSFKETSGLSFRSALSGGPAHGQFNGLWRCIAPRSSSSSSSKSKLLPHFHIARKTPWLQAVRSMALSDLPTGPLGLYDPSLDKDSCGVGFVAELSGVPSRQTVTDALEMLVRMAHRGACGCETNTGDGAGLTVALPHDYLVSVAKQDMNCELPPLNEYAVGMFFLPTSESRREKSKTIFNKVAESLGHRILGWRRVKTDNSDLGKSALKTEPVVEQMFLTPSSQSNLDLEQQLYILRKTAMIAIRAALNLAYGGARDFYICSLSSRTITYKGQLKPDQLKDYYYEDLGSEHFTSYMALVHSRFSTNTFPSWDRAQPMRVLGHNGEINTLRGNINWMQAREGLLKSRNLGLTPDELQKLLPIVDTSSSDSGAFDGVLELLVRSGRSLPEAVMMMIPEAWQNDVNMDPERRALYEYFSAVMEPWDGPALIAFTDGRYLGATLDRNGLRPGRYYETHSGRVIMASEVGVVDIAPEDVSKKGRLNPGMMLLVDFERHVVVDDDQLKKQYAARHPYADWLRRQKIVLKEVVESVPEAQRVAPHVIGSANVDSSDETMEKMGIHGLLPPLKAFGYTVEALEMLLLPMAKDGIEALGSMGNDTPLAVMSSRAKLGFEYFKQMFAQVTNPPIDPIREAIVTSTECMIGPEGDLTETTEGQCQRLSLKGPLLSIEEMEAIKKMDYQGWRSKVIDITFPRYDGTKGLEKALDRICLEARAAIREGYKILVLSDRETSPLRVPVGSLFAVGAVHQHLVSTLERTRIGLIVESGEPREVHHFCTLVGFGADAICPYLSIEAVFRLQVDGKIPPKKDGTFHSKDELVRKYFKASNSGILKVIAKMGISTLASYKGAQIFEALGLASDVVDKCFKGTPSRVEGATFEMLARDALRLHELAFPSRQLPEGSADALALPNPGDYYWRKDGEVHLNDPVAMAKLQEAARTNSVAAYKEYSKLIHSLNKQCNLRGMLKFKDLPERRIALEEVEPASEIVKRFCTGAMSYGSISLEAHSALALAMNKIGGKSNSGEGGENASRLEALSDGTPNPKRSAIKQVASGRFGVTSYYLTNAIEIQIKMAQGAKPGEGGELPGHKVIGDIAVTRNSTPGVGLISPPPHHDIYSIEDLAQLIYDLKNSNPGARISVKLVSEAGVGVIASGVVKGHADHVLISGHDGGTGASRWTGIKNAGLPWELGLAETHQTLVANDLRGRTVLQTDGQLKTGRDVAIAALLGAEEFGFSTAPLITLGCIMMRKCHKNTCPVGIATQDPVLREKFAGEPEHVINFFFMVAEEVRELMAEMGFRTVNEMIGRSDMLIVDHDVIKANEKLQGINLSLLLQPAADIRPDAVQYCVQKQDHSLDMALDQDLIKLSQPALQKKIPVYLEKPIVNVNRAVGTMLSHEVTKRYKMAGLPSDTIHVKFNGSAGQSLGAFLCSGITLELEGDSNDYVGKGLSGGKIIVYPPQGSGFDPKQNIVIGNVALYGATSGEAYFNGMAAERFCVRNSGAKAVVEGVGDHGCEYMTGGTVVILGKTGKNFAAGMSGGIAYVLDVDGKFKTRCNPGLVDLDKVEDAEDIMTLRMMVQQHQRNTQSQLAKEVIANFEELLPKFVKVFPRDYKQVLQKQKQKARKEEAELSKKDAFEELKKLANKSGVSNASEGKELKTVKANTAVAQRPTKVDNAVKHGGFVKYEREALPYREASDRIKDWKEVLEEKRSDTLLKTQSARCMDCGTPFCHQETSGCPLGNKIPEWNELVHQGRWREALDRLLETNNFPEFTGRVCPAPCEGACVLGIIENPVTIKTMECTIIDKAFENGWMVPRPPVKRTGKKIAVVGSGPAGLAAADQLNKAGHLVTVYERADRIGGLMMYGVPNMKTDKMEVVQRRVNLMAAEGIKFVVNANVGGDPSFSVEKLRQENDALLLACGATRPRDLSVEGRELQGIHYAMEFLHANTKSLLDSNLEDGKYISAAGKKVVVIGGGDTGTDCIGTSVRHGAESIVNLELLPQPPTTRAPGNPWPQWPRVLRTDYGHQEAIFKYGDDPRAYTVLTKRFIGDEQGKLKGLEIVKVQWEKDATGKFQMQEIEGSQEVIEADLVFLAMGFLGPEQDIAEKLGLERDARSNFKAAYGEYATNLEGVFAAGDCRRGQSLVVWAIAEGRQAAAKIDNFLSLDEVHPDERVTKEDLLHKVFSKKDSSRQEDQGTLLQRAR

>Ceric.24G023300.5.v2.1|Ceratopteris_richardii/1-1870

MRILGHNGEINTLRGNINWMQAREGLLKSRNLGLTPDELQKLLPIVDTSSSDSGAFDGVLELLVRSGRSLPEAVMMMIPEAWQNDVNMDPERRALYEYFSAVMEPWDGPALIAFTDGRYLGATLDRNGLRPGRYYETHSGRVIMASEVGVVDIPPEDVSKKGRLNPGMMLLVDFERHVVVDDDQLKKQYAARHPYADWLRRQKIILKEIVESVPETQRVPPHVIGAAYVDNSDETMEKMGIHGLVQPLKAFGYTVEALEMLLLPMAKDGVEALGSMGNDTPLAIISERPKLGFEYFKQMFAQVTNPPIDPIREAIVTSTECMIGPEGDLTETVESQCQRLSLKGPLLSIEEMEAIKKMSYRGWRSKVIDTTFDKHDGTHGFEKALDRICLEARAAIQEGYKILVLSDRETSPSRVPVGSLFAVGAVHQHLVSTLERTRVGLIVESGEPREVHHFCTLVGFGADAVCPYLAIEAVWRLQVDGKIPPKGDGNLHSKDELVRKYFKASNSGILKVLAKMGISTLASYKGAQIFEALGLSSDVVEKCFKGTPSRVEGATFEMLARDSLRLHDLAFPSRHLPDGSAESVALPNPGDYHWRKEGEAHLNDPVAIAKLQEAARTNSVAAFKEYSKLIHNLNKKCTLRGMLKFKEVPHREITLEEVEPASEIVKRFCTGAMSYGSISLEAHETLAQAMNRIGGKSNSGEGGEKASRLVAQPDGSPNPKRSAIKQVASGRFGVTSYYLTNAAEIQIKMAQGAKPGEGGELPGHKVIGDIAVTRNSTPGVGLISPPPHHDIYSIEDLAQLIYDLKNSNPGARISVKLVSEAGVGVIASGVVKGHADHVLISGHDGGTGASRWTGIKNAGLPWELGLAETHQTLVANDLRGRTVLQTDGQLKTGRDVAIAALLGAEEFGFSTAPLITMGCIMMRKCHKNTCPVGIATQDPVLREKFAGEPEHVINFFFMIAEEVRELMAGMGFRTMSEMIGRSDMLMVDQEVIKQNEKLQGIDLSLLLQPAADIRPDAVQYCVQKQDHGLDMALDQDLIKLSQPALTKKVPVYLEKPIVNVNRAVGTMLSHEVTKRYKMDGLPTDTIHVKFKGSAGQSLGAFLCSGITLELEGDSNDYVGKGLSGGKIIVYPPQGSGFDPKQNIVIGNVALYGATSGEAYFNGMAAERFCVRNSGAKAVVEGIGDHGCEYMTGGTVAILGKTGRNFAAGMSGGIAYVFDVDGKFRTRCNHGSVDLEKVESEEDIMTLRMMVQQHQRNTRSKLAKEVLANFDELLPKFVKVFPRDYKQVLLKQKEEAQKEEAELQKKNAFEELKKLAMKSGASAVPVLKEPKASKMKVAIPERPTKVNDAVKNGGFIKYEREALPYREAGERITDWKEVLEETRPTELLKTQSARCMDCGTPFCHQETSGCPLGNKIPEWNELVHQGRWREALDRLLETNNFPEFTGRVCPAPCEGSCVLGIIENPVTIKTMECSIIDKGFEEGWMVPRPPANRTGKKVAIVGSGPAGLAAADQLNKAGHSVTVFERADRIGGLMMYGVPNMKTDKMQIVQRRVDLMAAEGIKFVVNAHVGVDPQYSIDKLRKENDALLLACGATRPRDLNVQGKELKGIHFAMEFLHANTKSLLDSNLEDGQYISAAGKKVVVIGGGDTGTDCIGTSVRHGAESIVNLELLPQPPRTRAPGNPWPQWPRILRTDYGHQEAIFKYGEDPRAYEVLTKKFIGDEKGHVKGLEVVKVQWEKDATGKPVMQEVPGSQEIIEADLVFLAMGFIGPEQDIAAKLGLEVDVRSNFKASYGEYATNLEGVFAAGDCRRGQSLVVWAIAEGRQAAAKIDKFLIPEEEKQVDDIIEKDNLMYNPKSEHINKDHGTLLQGAR

>Ceric.24G023300.4.v2.1|Ceratopteris_richardii/1-1891

MALVHSRFSTNTFPSWDRAQPMRILGHNGEINTLRGNINWMQAREGLLKSRNLGLTPDELQKLLPIVDTSSSDSGAFDGVLELLVRSGRSLPEAVMMMIPEAWQNDVNMDPERRALYEYFSAVMEPWDGPALIAFTDGRYLGATLDRNGLRPGRYYETHSGRVIMASEVGVVDIPPEDVSKKGRLNPGMMLLVDFERHVVVDDDQLKKQYAARHPYADWLRRQKIILKEIVESVPETQRVPPHVIGAAYVDNSDETMEKMGIHGLVQPLKAFGYTVEALEMLLLPMAKDGVEALGSMGNDTPLAIISERPKLGFEYFKQMFAQVTNPPIDPIREAIVTSTECMIGPEGDLTETVESQCQRLSLKGPLLSIEEMEAIKKMSYRGWRSKVIDTTFDKHDGTHGFEKALDRICLEARAAIQEGYKILVLSDRETSPSRVPVGSLFAVGAVHQHLVSTLERTRVGLIVESGEPREVHHFCTLVGFGADAVCPYLAIEAVWRLQVDGKIPPKGDGNLHSKDELVRKYFKASNSGILKVLAKMGISTLASYKGAQIFEALGLSSDVVEKCFKGTPSRVEGATFEMLARDSLRLHDLAFPSRHLPDGSAESVALPNPGDYHWRKEGEAHLNDPVAIAKLQEAARTNSVAAFKEYSKLIHNLNKKCTLRGMLKFKEVPHREITLEEVEPASEIVKRFCTGAMSYGSISLEAHETLAQAMNRIGGKSNSGEGGEKASRLVAQPDGSPNPKRSAIKQVASGRFGVTSYYLTNAAEIQIKMAQGAKPGEGGELPGHKVIGDIAVTRNSTPGVGLISPPPHHDIYSIEDLAQLIYDLKNSNPGARISVKLVSEAGVGVIASGVVKGHADHVLISGHDGGTGASRWTGIKNAGLPWELGLAETHQTLVANDLRGRTVLQTDGQLKTGRDVAIAALLGAEEFGFSTAPLITMGCIMMRKCHKNTCPVGIATQDPVLREKFAGEPEHVINFFFMIAEEVRELMAGMGFRTMSEMIGRSDMLMVDQEVIKQNEKLQGIDLSLLLQPAADIRPDAVQYCVQKQDHGLDMALDQDLIKLSQPALTKKVPVYLEKPIVNVNRAVGTMLSHEVTKRYKMDGLPTDTIHVKFKGSAGQSLGAFLCSGITLELEGDSNDYVGKGLSGGKIIVYPPQGSGFDPKQNIVIGNVALYGATSGEAYFNGMAAERFCVRNSGAKAVVEGIGDHGCEYMTGGTVAILGKTGRNFAAGMSGGIAYVFDVDGKFRTRCNHGSVDLEKVESEEDIMTLRMMVQQHQRNTRSKLAKEVLANFDELLPKFVKVFPRDYKQVLLKQKEEAQKEEAELQKKNAFEELKKLAMKSGASAVPVLKEPKASKMKVAIPERPTKVNDAVKNGGFIKYEREALPYREAGERITDWKEVLEETRPTELLKTQSARCMDCGTPFCHQETSGCPLGNKIPEWNELVHQGRWREALDRLLETNNFPEFTGRVCPAPCEGSCVLGIIENPVTIKTMECSIIDKGFEEGWMVPRPPANRTGKKVAIVGSGPAGLAAADQLNKAGHSVTVFERADRIGGLMMYGVPNMKTDKMQIVQRRVDLMAAEGIKFVVNAHVGVDPQYSIDKLRKENDALLLACGATRPRDLNVQGKELKGIHFAMEFLHANTKSLLDSNLEDGQYISAAGKKVVVIGGGDTGTDCIGTSVRHGAESIVNLELLPQPPRTRAPGNPWPQWPRILRTDYGHQEAIFKYGEDPRAYEVLTKKFIGDEKGHVKGLEVVKVQWEKDATGKPVMQEVPGSQEIIEADLVFLAMGFIGPEQDIAAKLGLEVDVRSNFKASYGEYATNLEGVFAAGDCRRGQSLVVWAIAEGRQAAAKIDKFLIPEEEKQVDDIIEKDNLMYNPKSEHINKDHGTLLQGAR

>Ceric.24G023300.2.v2.1|Ceratopteris_richardii/1-2042

MRDMLCELPPPNEYAVGMFFLPTSEARREKSKTVFNKVAESLGHRILGWRKVKTDNSDLGKSALKTEPVVEQLFLTPSYHSNLSIECQMYILRKTAMIAVRAALNLQYGGARDFYICSLSSRTITYKGQLKPDQLKNYYFEDLGSEKFTSYMALVHSRFSTNTFPSWDRAQPMRILGHNGEINTLRGNINWMQAREGLLKSRNLGLTPDELQKLLPIVDTSSSDSGAFDGVLELLVRSGRSLPEAVMMMIPEAWQNDVNMDPERRALYEYFSAVMEPWDGPALIAFTDGRYLGATLDRNGLRPGRYYETHSGRVIMASEVGVVDIPPEDVSKKGRLNPGMMLLVDFERHVVVDDDQLKKQYAARHPYADWLRRQKIILKEIVESVPETQRVPPHVIGAAYVDNSDETMEKMGIHGLVQPLKAFGYTVEALEMLLLPMAKDGVEALGSMGNDTPLAIISERPKLGFEYFKQMFAQVTNPPIDPIREAIVTSTECMIGPEGDLTETVESQCQRLSLKGPLLSIEEMEAIKKMSYRGWRSKVIDTTFDKHDGTHGFEKALDRICLEARAAIQEGYKILVLSDRETSPSRVPVGSLFAVGAVHQHLVSTLERTRVGLIVESGEPREVHHFCTLVGFGADAVCPYLAIEAVWRLQVDGKIPPKGDGNLHSKDELVRKYFKASNSGILKVLAKMGISTLASYKGAQIFEALGLSSDVVEKCFKGTPSRVEGATFEMLARDSLRLHDLAFPSRHLPDGSAESVALPNPGDYHWRKEGEAHLNDPVAIAKLQEAARTNSVAAFKEYSKLIHNLNKKCTLRGMLKFKEVPHREITLEEVEPASEIVKRFCTGAMSYGSISLEAHETLAQAMNRIGGKSNSGEGGEKASRLVAQPDGSPNPKRSAIKQVASGRFGVTSYYLTNAAEIQIKMAQGAKPGEGGELPGHKVIGDIAVTRNSTPGVGLISPPPHHDIYSIEDLAQLIYDLKNSNPGARISVKLVSEAGVGVIASGVVKGHADHVLISGHDGGTGASRWTGIKNAGLPWELGLAETHQTLVANDLRGRTVLQTDGQLKTGRDVAIAALLGAEEFGFSTAPLITMGCIMMRKCHKNTCPVGIATQDPVLREKFAGEPEHVINFFFMIAEEVRELMAGMGFRTMSEMIGRSDMLMVDQEVIKQNEKLQGIDLSLLLQPAADIRPDAVQYCVQKQDHGLDMALDQDLIKLSQPALTKKVPVYLEKPIVNVNRAVGTMLSHEVTKRYKMDGLPTDTIHVKFKGSAGQSLGAFLCSGITLELEGDSNDYVGKGLSGGKIIVYPPQGSGFDPKQNIVIGNVALYGATSGEAYFNGMAAERFCVRNSGAKAVVEGIGDHGCEYMTGGTVAILGKTGRNFAAGMSGGIAYVFDVDGKFRTRCNHGSVDLEKVESEEDIMTLRMMVQQHQRNTRSKLAKEVLANFDELLPKFVKVFPRDYKQVLLKQKEEAQKEEAELQKKNAFEELKKLAMKSGASAVPVLKEPKASKMKVAIPERPTKVNDAVKNGGFIKYEREALPYREAGERITDWKEVLEETRPTELLKTQSARCMDCGTPFCHQETSGCPLGNKIPEWNELVHQGRWREALDRLLETNNFPEFTGRVCPAPCEGSCVLGIIENPVTIKTMECSIIDKGFEEGWMVPRPPANRTGKKVAIVGSGPAGLAAADQLNKAGHSVTVFERADRIGGLMMYGVPNMKTDKMQIVQRRVDLMAAEGIKFVVNAHVGVDPQYSIDKLRKENDALLLACGATRPRDLNVQGKELKGIHFAMEFLHANTKSLLDSNLEDGQYISAAGKKVVVIGGGDTGTDCIGTSVRHGAESIVNLELLPQPPRTRAPGNPWPQWPRILRTDYGHQEAIFKYGEDPRAYEVLTKKFIGDEKGHVKGLEVVKVQWEKDATGKPVMQEVPGSQEIIEADLVFLAMGFIGPEQDIAAKLGLEVDVRSNFKASYGEYATNLEGVFAAGDCRRGQSLVVWAIAEGRQAAAKIDKFLIPEEEKQVDDIIEKDNLMYNPKSEHINKDHGTLLQGAR

>Ceric.24G023300.1.v2.1|Ceratopteris_richardii/1-2207

MESSILSNSVGRISNAVFMPSSSSTRDVSGLSFRSSLNGGICSSQFCGLRRIGARSSFISVKNTKVSPYVQNVRRTPWLQAVRSMALSDIPTGPLGLYDPSRDKDSCGVGFVAELSGVPSRKTVTDALEMLVRMAHRGACGCETNTGDGAGLTVALPHEYLASVAMRDMLCELPPPNEYAVGMFFLPTSEARREKSKTVFNKVAESLGHRILGWRKVKTDNSDLGKSALKTEPVVEQLFLTPSYHSNLSIECQMYILRKTAMIAVRAALNLQYGGARDFYICSLSSRTITYKGQLKPDQLKNYYFEDLGSEKFTSYMALVHSRFSTNTFPSWDRAQPMRILGHNGEINTLRGNINWMQAREGLLKSRNLGLTPDELQKLLPIVDTSSSDSGAFDGVLELLVRSGRSLPEAVMMMIPEAWQNDVNMDPERRALYEYFSAVMEPWDGPALIAFTDGRYLGATLDRNGLRPGRYYETHSGRVIMASEVGVVDIPPEDVSKKGRLNPGMMLLVDFERHVVVDDDQLKKQYAARHPYADWLRRQKIILKEIVESVPETQRVPPHVIGAAYVDNSDETMEKMGIHGLVQPLKAFGYTVEALEMLLLPMAKDGVEALGSMGNDTPLAIISERPKLGFEYFKQMFAQVTNPPIDPIREAIVTSTECMIGPEGDLTETVESQCQRLSLKGPLLSIEEMEAIKKMSYRGWRSKVIDTTFDKHDGTHGFEKALDRICLEARAAIQEGYKILVLSDRETSPSRVPVGSLFAVGAVHQHLVSTLERTRVGLIVESGEPREVHHFCTLVGFGADAVCPYLAIEAVWRLQVDGKIPPKGDGNLHSKDELVRKYFKASNSGILKVLAKMGISTLASYKGAQIFEALGLSSDVVEKCFKGTPSRVEGATFEMLARDSLRLHDLAFPSRHLPDGSAESVALPNPGDYHWRKEGEAHLNDPVAIAKLQEAARTNSVAAFKEYSKLIHNLNKKCTLRGMLKFKEVPHREITLEEVEPASEIVKRFCTGAMSYGSISLEAHETLAQAMNRIGGKSNSGEGGEKASRLVAQPDGSPNPKRSAIKQVASGRFGVTSYYLTNAAEIQIKMAQGAKPGEGGELPGHKVIGDIAVTRNSTPGVGLISPPPHHDIYSIEDLAQLIYDLKNSNPGARISVKLVSEAGVGVIASGVVKGHADHVLISGHDGGTGASRWTGIKNAGLPWELGLAETHQTLVANDLRGRTVLQTDGQLKTGRDVAIAALLGAEEFGFSTAPLITMGCIMMRKCHKNTCPVGIATQDPVLREKFAGEPEHVINFFFMIAEEVRELMAGMGFRTMSEMIGRSDMLMVDQEVIKQNEKLQGIDLSLLLQPAADIRPDAVQYCVQKQDHGLDMALDQDLIKLSQPALTKKVPVYLEKPIVNVNRAVGTMLSHEVTKRYKMDGLPTDTIHVKFKGSAGQSLGAFLCSGITLELEGDSNDYVGKGLSGGKIIVYPPQGSGFDPKQNIVIGNVALYGATSGEAYFNGMAAERFCVRNSGAKAVVEGIGDHGCEYMTGGTVAILGKTGRNFAAGMSGGIAYVFDVDGKFRTRCNHGSVDLEKVESEEDIMTLRMMVQQHQRNTRSKLAKEVLANFDELLPKFVKVFPRDYKQVLLKQKEEAQKEEAELQKKNAFEELKKLAMKSGASAVPVLKEPKASKMKVAIPERPTKVNDAVKNGGFIKYEREALPYREAGERITDWKEVLEETRPTELLKTQSARCMDCGTPFCHQETSGCPLGNKIPEWNELVHQGRWREALDRLLETNNFPEFTGRVCPAPCEGSCVLGIIENPVTIKTMECSIIDKGFEEGWMVPRPPANRTGKKVAIVGSGPAGLAAADQLNKAGHSVTVFERADRIGGLMMYGVPNMKTDKMQIVQRRVDLMAAEGIKFVVNAHVGVDPQYSIDKLRKENDALLLACGATRPRDLNVQGKELKGIHFAMEFLHANTKSLLDSNLEDGQYISAAGKKVVVIGGGDTGTDCIGTSVRHGAESIVNLELLPQPPRTRAPGNPWPQWPRILRTDYGHQEAIFKYGEDPRAYEVLTKKFIGDEKGHVKGLEVVKVQWEKDATGKPVMQEVPGSQEIIEADLVFLAMGFIGPEQDIAAKLGLEVDVRSNFKASYGEYATNLEGVFAAGDCRRGQSLVVWAIAEGRQAAAKIDKFLIPEEEKQVDDIIEKDNLMYNPKSEHINKDHGTLLQGAR

>Ceric.24G023300.3.v2.1|Ceratopteris_richardii/1-1954

MYILRKTAMIAVRAALNLQYGGARDFYICSLSSRTITYKGQLKPDQLKNYYFEDLGSEKFTSYMALVHSRFSTNTFPSWDRAQPMRILGHNGEINTLRGNINWMQAREGLLKSRNLGLTPDELQKLLPIVDTSSSDSGAFDGVLELLVRSGRSLPEAVMMMIPEAWQNDVNMDPERRALYEYFSAVMEPWDGPALIAFTDGRYLGATLDRNGLRPGRYYETHSGRVIMASEVGVVDIPPEDVSKKGRLNPGMMLLVDFERHVVVDDDQLKKQYAARHPYADWLRRQKIILKEIVESVPETQRVPPHVIGAAYVDNSDETMEKMGIHGLVQPLKAFGYTVEALEMLLLPMAKDGVEALGSMGNDTPLAIISERPKLGFEYFKQMFAQVTNPPIDPIREAIVTSTECMIGPEGDLTETVESQCQRLSLKGPLLSIEEMEAIKKMSYRGWRSKVIDTTFDKHDGTHGFEKALDRICLEARAAIQEGYKILVLSDRETSPSRVPVGSLFAVGAVHQHLVSTLERTRVGLIVESGEPREVHHFCTLVGFGADAVCPYLAIEAVWRLQVDGKIPPKGDGNLHSKDELVRKYFKASNSGILKVLAKMGISTLASYKGAQIFEALGLSSDVVEKCFKGTPSRVEGATFEMLARDSLRLHDLAFPSRHLPDGSAESVALPNPGDYHWRKEGEAHLNDPVAIAKLQEAARTNSVAAFKEYSKLIHNLNKKCTLRGMLKFKEVPHREITLEEVEPASEIVKRFCTGAMSYGSISLEAHETLAQAMNRIGGKSNSGEGGEKASRLVAQPDGSPNPKRSAIKQVASGRFGVTSYYLTNAAEIQIKMAQGAKPGEGGELPGHKVIGDIAVTRNSTPGVGLISPPPHHDIYSIEDLAQLIYDLKNSNPGARISVKLVSEAGVGVIASGVVKGHADHVLISGHDGGTGASRWTGIKNAGLPWELGLAETHQTLVANDLRGRTVLQTDGQLKTGRDVAIAALLGAEEFGFSTAPLITMGCIMMRKCHKNTCPVGIATQDPVLREKFAGEPEHVINFFFMIAEEVRELMAGMGFRTMSEMIGRSDMLMVDQEVIKQNEKLQGIDLSLLLQPAADIRPDAVQYCVQKQDHGLDMALDQDLIKLSQPALTKKVPVYLEKPIVNVNRAVGTMLSHEVTKRYKMDGLPTDTIHVKFKGSAGQSLGAFLCSGITLELEGDSNDYVGKGLSGGKIIVYPPQGSGFDPKQNIVIGNVALYGATSGEAYFNGMAAERFCVRNSGAKAVVEGIGDHGCEYMTGGTVAILGKTGRNFAAGMSGGIAYVFDVDGKFRTRCNHGSVDLEKVESEEDIMTLRMMVQQHQRNTRSKLAKEVLANFDELLPKFVKVFPRDYKQVLLKQKEEAQKEEAELQKKNAFEELKKLAMKSGASAVPVLKEPKASKMKVAIPERPTKVNDAVKNGGFIKYEREALPYREAGERITDWKEVLEETRPTELLKTQSARCMDCGTPFCHQETSGCPLGNKIPEWNELVHQGRWREALDRLLETNNFPEFTGRVCPAPCEGSCVLGIIENPVTIKTMECSIIDKGFEEGWMVPRPPANRTGKKVAIVGSGPAGLAAADQLNKAGHSVTVFERADRIGGLMMYGVPNMKTDKMQIVQRRVDLMAAEGIKFVVNAHVGVDPQYSIDKLRKENDALLLACGATRPRDLNVQGKELKGIHFAMEFLHANTKSLLDSNLEDGQYISAAGKKVVVIGGGDTGTDCIGTSVRHGAESIVNLELLPQPPRTRAPGNPWPQWPRILRTDYGHQEAIFKYGEDPRAYEVLTKKFIGDEKGHVKGLEVVKVQWEKDATGKPVMQEVPGSQEIIEADLVFLAMGFIGPEQDIAAKLGLEVDVRSNFKASYGEYATNLEGVFAAGDCRRGQSLVVWAIAEGRQAAAKIDKFLIPEEEKQVDDIIEKDNLMYNPKSEHINKDHGTLLQGAR

>Aspi01Gene33520|Alsophila_spinulosa/1-2231

MAAISTTSAPLSIRSLTEQTTAAQPSSVSSLLFSKLHQEKTPLSFRSVFSGLPGPRQFCGLRRNGSLRSSIRGKKRMHPHWHIAQQTPWLQVVRSMALSDYPTGPLGLYDPALDKDSCGVGFVAELSGVPSRQTVTDALEMLVRMAHRGACGCETNTGDGAGITVALPHGYLAQVARQDIGCDLPPPNEYAVGMFFLPTSETRREESKNVFNKVAESLGHTILGWRRVRTDNSDLGKSALKTEPIVEQIFLTPSCRSNLDLEQQMYILRKTSMIAIRAALNLAYGGARDFYICSLSSRTITFKGQLKPDQLKKYYYEDLGNERFTSYMALVHSRFSTNTFPSWDRAQPMRVLGHNGEINTLRGNINWMQAREGLLKCKDLSLSTDELQKLLPIVDTSSSDSGAFDGVLEFLVRSGRSLPEAVMMMIPEAWQNDTNMDPDRKALYEYFSAVMEPWDGPALIAFTDGRYLGATLDRNGLRPGRYYETHSGRVIMASEVGVVDIAPEDVSKKGRLNPGMMLLVDFERHVVVDDDQLKKQYAASHPYADWLRRQKIVLKEIVDSVPEVDRVARHVIGCADVDSADETMEKMGIYGLLPPLKAFGYTVEALEMLLLPMAKDGVEALGSMGNDTPLAVMSSRPKLGFEYFKQMFAQVTNPPIDPIREAIVTSTECMIGPEGDLTETAEGQCQRLSLKGPLLSIEEMEAIKKMDYRGWRSKVIDTTFSKYDGTKGLEKALDRICLEARSAIQEGYKVLVLSDRETSALRVPVGSLLAAGAVHQHLVSSLERSRIGLIVESGEPREVHHFCTLVGFGADAICPYLAIEAVWRLQVDGKIPPKKEGVLHSKEELVRKYFKASNSGILKVIAKMGISTLASYKGAQIFEALGLASEVVEKCFKGTPSRVEGATFEMLAQDILRLHALAFPSRLLPEGSADALALPNPGDYHWRKDGEVHLNDPVAMAKLQEAARTNSVAAYKEYSKLIHSLNKKCNLRGMLKFKDMPEKRIPLEEVEPASEIVKRFCTGAMSYGSISLEAHSTLALAMNRIGGKSNSGEGGENASRLEALSDGSPNPKRSAIKQVASGRFGVTSYYLTNAIELQIKMAQGAKPGEGGELPGYKVVGDIAVTRNSTPGVGLISPPPHHDIYSIEDLAQLIYDLKNSNPGARISVKLVSEAGVGVIASGVVKGHADHVLISGHDGGTGASRWTGIKNAGLPWELGLAETHQTLVANDLRGRTVLQTDGQLKTGRDVAIAAFLGAEEFGFSTAPLITLGCIMMRKCHKNTCPVGIATQDPVLREKFAGEPEHVINFFFMVAEEVRELMAEMGFRTMNEMIGRSDILLVDNEVITENEKLQGIDLSLLLQPAAEIRPDAAQYCVQKQDHSLDMALDQELIKLSQPALMKKLPVYFEMPIVNVNRAVGTMLSHEVTKRYKMAGLPADTIHVKLTGSAGQSLGAFLCPGITLELEGDSNDYVGKGLSGGKIIVYPPQGSGFDPKKNIVIGNVALYGATGGEAYFNGMAAERFCVRNSGAKAVVEGIGDHGCEYMTGGTVVILGSTGKNFAAGMSGGIAYVLDVDGKFKSRCNLGLVDLDEVEDEEDIMTLCTMVQQHQRNTQSQLAKEVLARFDELLPKFVKVFPRDYKKVLLKEKQKALEEEAELFKKDAFEELKKLSNEGSVSGSSVLNGVGTLKSNGVVEQKPTKVEDAIKHRGFINYERESLPYRRTEDRINDWKEVLEETRPDVLLKTQSARCMDCGTPFCHQETSGCPLGNKIPEWNELVHQGRWREALDRLLETNNFPEFTGRVCPAPCEGSCVLGIIENPVTIKTMECTIIDKAFEEGWMVPRPPVKRTGKKVAIVGSGPAGLAAADQLNKAGHAVTIFERADRIGGLMMYGVPNMKTDKVEVVQRRVDLMAAEGIKFVVNANVGVDPLFSVEKLREENDALLLACGATSPRDLNVEGRELQGVHFAMEFLHSNTKSLLDSNLDDGKYISAAGKKIVVIGGGDTGTDCIGTSVRHGAESVVNLELLPQPPATRAPGNPWPQWPRTLRTDYGHQEAIFKFGKDPRSYRVLTKRFLGNEEGRVTGLEIVRVQWEKDANGKFQMQEIQGSEEVIEADLVLLAMGFLGPEQNIAEKLGLERDARSNFKAEYGQYATNLQGVFAAGDCRRGQSLVVWAIAEGRQAAAKIDKFLYADEEGTPADDRGAQEDKMYDLFGRKESSRQEGFSTESERGNQGSLLQRAR

>Mvestita_S12g15656-RA|Marsilea_vestita/1-2186

MAMLSASTSLGLGSVPRQRQREVQASLLSDLPRELSMRTGRAGFAGYGQFCGLRRTVNMRNSNKGKKRVPLQWHILNHTPWMHAIRAMALSDVPEGPLGLYDPALDKDSCGVGFVAELSAIPSRKTVTDALEMLVRMAHRGACGCEVNTGDGAGITVAKRDIGCELPPPNQYAVGMFFLPTDETRREESKNVFNKVAESLGHTILGWRRVKTDNSDLGQSALKTEPIVEQIFLTPSSRSKLDLEQQMYILRKTSMIAIRAALNLAYGGAKDFYICSLSSRTIIFKGQLKPDQLKKYYYEDLGSEKFTSYMALVHSRFSTNTFPSWDRAQPMRILGHNGEINTLRGNINWMRAREGLLKCKQLGLSADELQKLLPIVDTSSSDSGAFDGVLELLVRSGRSLPEAVMMMIPEAWQNDDNIDPERKALYEYFSAVMEPWDGPALIAFTDGRYLGATLDRNGLRPGRYYETHSGRVIMASEVGVVDIPPEDVSKKGRLNPGMMLLVDFERHVVVDDDQLKKQYSATRPYAEWLQRQKIILDEIVKSVPKSYLGSPKITGASYAESSDETMENVGVHGLLSPLKAFGYTMEALEMLLIPMAKDGYEALGSMGNDTPLAVMSNRPKLGFEYFKQMFAQVTNPPIDPIREAIVTSTECMIGPEGDLTETTEAQCQRLSLKGPLLSIEEMQAIKNMDYRGWKSKTIDMTFSIKEGTQGLEKALDRICAEARSAIKEGYKILVLSDRNTSAERVPVGSLLAAGAVHQHLVSSLERTRIGLVVESGEPREVHHFCTLVGFGADAICPYLAIETIWRLQVDGKIPPKAGGVFHSKEELVRKYFKAVNSGILKVIAKMGISTLASYKGAQIFEALGLSSEVVEKCFNGTPSRVEGATFEMLARDQLRLHELAFPSRLLPEGSAEAVALPNPGDYHWRKDGEIHLNDPVAMAKLQEAARTNSVAAYKEYSKLIQDLNKKCNLRGMLKFKSTPERRIPLEEVEPASEIVKRFCTGAMSYGSISLEAHTTLALAMNTLGGKSNSGEGGEQVERLESLSDGSPNPKRSAIKQVASGRFGVTTYYLTNAIELQIKMAQGAKPGEGGELPGHKVIGDIAVTRNSTPGVGLISPPPHHDIYSIEDLAQLIYDLKNSNPSARISVKLVSEAGVGVIASGVVKGHADHVLISGHDGGTGASRWTGIKNAGLPWELGLAETHQTLVANDLRGRTVLQTDGQLKTGRDVAIAALLGAEEFGFSTAPLITMGCIMMRKCHKNTCPVGIATQDPVLREKFAGEPEHVINFFFMVAEEMRELMSEMGFRTVNEMVGRSDMLEMDQEVISSNDKLQGIDLSLLLKPAAEIRPDAAQYCVQKQDHGLDMALDQKLIELSQPALQKKLPVYLEMPIVNVNRAVGTMLSHEVTKRYKMEGLPSDTIHVKLNGSAGQSVGAFLCPGITLELEGDSNDYVGKGLSGGKIIVYPPQGSGFDPKQNIVIGNVALYGATSGEAYFNGMAAERFCVRNSGAKAVVEGVGDHGCEYMTGGTVVILGKTGKNFAAGMSGGVAYVLDVDGNFKSRCNPGLVDLDKVEDEEDIMTLRTMIQQHQKYTQSQLAKEVLARFDELIPMFLKVFPRDYKQVLLQQKQKKLQEEAELSKKDAFEELKKLATSNVATSNGAPKQRPTRLETPVKNGGFKRYEREPLSYRKVDERVNDWKEVLDDNRPAELLKTQSARCMDCGTPFCHQETSGCPLGNKIPEWNELVYQGRWREALDRLLETNNFPEFTGRVCPAPCEGACVLGIIENPVTIKTMECSIIDKAYEEGWMVPRPPASRTGKRIAIVGSGPAGLAAADQLNKMGHTVTIFERSDRIGGLMMYGVPNMKTDKIDIVQRRVDLMAAEGVQFVVNAHVGVDPVYSVEKLRKEHDALLLACGATKPRDLNIEGRDLQGVHFAMEFLHANTKSLLDSNLQDGNYISAEGKKVVVIGGGDTGTDCIGTSVRHGAVNVVNLELLPQPPSSRAPGNPWPQWPRILRTDYGHQEAIFKYGKDPRSYQVLTKRFIGDEQGNLKGLEIVKIQWEKDANGKFQMHELEGSNEVIEADLAFLAMGFLGPEENVAEKLGLERDARSNFKADYGEYATNLQGVFAAGDCRRGQSLVVWAIHEGRQAAAKIDRFLTADEEAEEVIDISESIPSDKKKGSSSESPTLLRGAR

>Azfi_s0002.g001246|Azolla_filiculoides/1-2183

MASLSNIPTSLRQPSSVSCSVSSLLSSKPCSLHGSLLSSLSSIGSQGKYTHFPGGLRRSLNLQTSNIRTRKSESNKGRISPHANPPLRRNPCTHLIRSMALSEYPSGPLGLYDPAFDKDSCGVGFVAELSAIPSRKTVKDALEMLMRMAHRGACGCEENTGDGAGITVALPHEYLTEVVKRDIGCELPPPNEYAVGMFFLPTQDDKGNDSNTRRDVSKNVFNKVAESLGHTILGWRRVKTDNSDLGQSALVTEPIVEQIFLTPSSRSNLDLEQQMYILRKTSMIAIRAALNLAYGGPKDFYICSLSSRTITYKGQLKPNQLENYYFEDLGNENFTSYMALVHSRFSTNTFPSWDRAQPMRVLGHNGEINTLRGNINWMKAREGLLKCKELGLSADELQKLLPVIDTSSSDSGAFDGVLELLVRSGRSLPEAVMMMIPEAWQNDDNMDPERKALYEYFSAVMEPWDGPALIAFTDGRYLGATLDRNGLRPGRYYETHSGRVIMASEVGVVDVAPEDVSKKGRLNPGMMLLVDFERHVVVDDDQLKKQYASKHPYANWLRRQKIVLKEIVDSVPKAELAVPHIIGSMDVKIYDETMEKMGIHGLLPPLKAFGYTTEALEMLLLPMAKDGAEALGSMGNDTPLAVMSSRPKLGFEYFKQMFAQVTNPPIDPIREAIVTSTECMIGPEGDLTEINEGQCRRLSLKGPLLSIEEMEAIKKMDYRGWRSKVIDMTFSRQEGSKGLERALDRICLEARSAIQEGYKILVLSDRETSASRVPVGSLLAAGAVHQHLVSRMERTRIGLIVESGEPREVHHFCTLVGFGADAICPYLAIETVWRLQVDGKIPPKPDGKLQSKEELVRKFFKASNSGILKVIAKMGISTLASYKGAQIFEALGLSTEVVEKCFKGTPSRVEGATFEMLARDLLRLHEAAFPSRALPEGSADALALPNPGDYHWRKDGEVHLNDPLAIAKLQEAARSNSIEAYKEYSKLVQSLNKKCNLRGMLKFKDMPERKVPLEEVEPASEIVKRFCTGAMSYGSISLEAHTTLALAMNKIGGKSNSGEGGEEASRLELLPDGSINPRRSAIKQVASGRFGVTSHYLTNAIELQIKMAQGAKPGEGGELPGHKVIGDIAVTRNSTPGVGLISPPPHHDIYSIEDLAQLIYDLKNANPGARISVKLVSEAGVGVIASGVVKGHADHVLISGHDGGTGASRWTGIKNAGLPWELGLAETHQTLVANNLRGRTVLQTDGQLKTGRDVAIAALLGAEEFGFSTAPLITLGCIMMRKCHKNTCPVGVATQDPVLREKFAGEPENVINFFFMVAEEMRELMSEMGFRTVNEMIGRSDMLEVDNEVISANEKLQGIDLSLLLKPSAEIRPEAPQFCIQKQDHGLDMALDQKLIELSQPALAKKVPVYLETEIVNVNRAVGTMLSHEVTKRYKASGLPADTIHVKLSGSAGQSLGAFLCSGITLELEGDSNDYVGKGLSGGKIVVYPPRGSAFDPQTNIVIGNVALYGATGGEAYFNGMAAERFCVRNSGAKAVVEGIGDHGCEYMTGGTVVILGKTGKNFAAGMSGGIAYVLDVDGNFESRCNLGLVDLDKVEEEEDITTLRMMVQQHLRYTGSQLAKEVLASFDELVPRFVKVFPRDYKQVLLMEKQKALEEAASLEKKDAFEELKKLASKSSVQQSSPVKAVGPTSSTRTLEKPTKVDGAIKNGGFIKYERESLSYRKVEERVEDWKEVLEDARPDVLLKTQSARCMDCGTPFCHQETSGCPLGNKIPEWNELVHQGRWREALDRLLETNNFPEFTGRVCPAPCEGACVLGIIENPVTIKTMECTIIDRGFEEGWMVPRPPLSRTGKKVAIVGSGPAGLAAADQLNKMGHLVTVYERADRIGGLMMYGVPNMKTDKVDVVQRRVNLMAAEGIKFVFLHSNTKSLLDSNLEDGNYISAAGKKVIVIGGGDTGTDCIATSVRHGAENIVNLELLPQPPASRAPGNPWPQWPRVLRTDYGHQEAVFKYGKDPRSYQVLTKRFIGNEEGRVIGIEIVKVKWEKDADGKFQLQEVPDTVEVLEADLIFLAMGFVGPEQNIAEKLGLATDARSNFKAEYGHYATNLQGVFAAGDCRRGQSLVVWAIAEGRQAASQMDKFLCPDKEEVGEVEKEQVHDVYNEGARQKKKAGNERDNQGSLLQGTR

>Sacu_v1.1_s0021.g008313|Salvinia_cucullata/1-2121

MAAWPLCSSGSSSTQYAAARQPWTGGYSSISTLLCGRGRYRRPVSMLLQGEGSLRKGGIREKRRQEEGGGRRMWCGENGGMMTMMRRAVAAADRSNMGLYDPSMDKDSCGVGFVAELSAVPSRNTVEDALEMLVRMAHRGACGCEENTGDGAGITVVKEEIGVELPPPNEYAVGMLFLPTDDSRRETGKNVFSKVAESLGHTILGWRRLKTDNSDLGKSALDTEPIVEQIFLTPSTRSHLDLEQQMYILRRTSMIAIRAALKLAYGGAKDFYICSLSSRTVTYKGQLKPDQVKKYYFQDLGNEKFTSYMALVHSRFSTNTFPSWDRAQPMRILGHNGEINTLRGNINWMRAREGLLKCKELKLSDDELQKLLPIVDVSSSDSGAFDGVMEFLVRSGRSLPEAVMMMIPEAWQNDVNMDPERKALYEYFSAIMEPWDGPALIAFTDGRYLGATLDRNGLRPGRYYETHSGRVIMASEVGVVDVPPEDVSKKGRLNPGMMLLVDFERHIVVDDEELKKQYAARYPFADWLRRQKMTLEDIAYTAEALEMLLIPMAKDGAEALGSMGNDTPLAVMSSRPKLGFEYFKQMFAQVTNPPIDPIREAIVTSTECMIGPEGDLTETDESQCQRLSLKGPLLSIEEMEAIKKMDYRGWKSKVIDMTFPKAEGTKGLEKALERICAEARHAIQEGYKILVLSDREVSVSRVPVGSLLAAGAVHQHLVSSLERTRIGLIIESGEPREVHHFCTLVGFGADAVCPYLAIEAVWRLQVDGKISPKANGELHSKEELVKKYFKACNSGILKVMAKMGISTLASYKGAQIFEALGLSTEVVEKCFKGTPSRVEGATFEMLARDLLHLHETAFPYRLLPEGSADALALPNPGDYHWRKDGEVHLNDPVAIAKLQEAARTNSVAAYKEYSKLIQSLNEKCNLRGMLKFKETPERSIPLEEVEPASEIVKRFCTGAMSYGSISLEAHTTLALAMNEIGGKSNSGEGGEEASRLESLPDGSPNPRRSAIKQVASGRFGVTSYYLTNAVELQIKMAQGAKPGEGGELPGHKVVGDIAVTRNSTPGVGLISPPPHHDIYSIEDLAQLIYDLKNSNPGARISVKLVSEAGVGVIASGVVKGHADHVLISGHDGGTGASRWTGIKNAGLPWELGLAETHQTLVANNLRGRAVLQTDGQLKSGRDVAIAALLGAEEFGFSTAPLITMGCIMMRKCHKNTCPVGIATQDPVLREKFAGEPEHVINFFFMVAEEMRELMAKMGFKTVNEMVGRADMLEVNREVISRNEKLQGIDLSLLLKPAAEIRPEAAQYCVEKQDHGLDMALDQKLIELAQPALLKKIPVYLEEDVVNVNRAVGTMLSHEISKRYKAAGLPDDTIHVKLTGSAGQSLGAFLCPGITLELEGDSNDYVGKGLSGGKIIVYPPEGSAFDPKTNIVIGNVALYGATKGEAYFNGMAAERFCVRNSGAKAVVEGTGDHGCEYMTGGTVVILGKTGKNFAAGMSGGIAYVLDVDGKFKSRCNTGLVDLDRVEDEEDTLTLRMMIQQHLRYTGSRLAKEVLANFEDLIPRFVKVFPRDYKSVVLKEKQKASQERASLEKKDAFQELKKLAMKPSLNVSSPAASSSSNGAIKRPTKVEGAIKNGGFIKYEREPLSYRKPEERIKDWKEVLEENRSDVLLKTQSARCMDCGTPFCHQETSGCPLGNKIPEWNELVHQGRWREALDRLLETNNFPEFTGRVCPAPCEGACVLGIIENPVTIKTMECTIIDKGFAEGWVVPRPPASRTGKKVAIVGSGPAGLAAADQLNKMGHSVTVYERSDRIGGLMMYGVPNMKTDKVDIVQRRVDLMAAEGIEDLKIQGRDLQGVHFAMEFLHSNTKSLLDSNLEDGKYISTAGKKVIVIGGGDTGTDCIATSVRHGAENIVNLELLPKPPASRAGDNPWPQWPKVSRTDYGHQEAIFKFGKDPRSYQVLTKRFIGNKEGKLTGVEVVKVKWEKDTNGKFHMQEIPDSQEVLEADLVFLALGFIGPEQNVAEKLGLQTDDRSNFKAEYGHYATNVEGVFAAGDCRRGQSLVVWAIAEGRQAASNIDKFLCPDDGAGNNGAEDLHIKSELHYEPKVRGDRANQESQLQGAR

>Aco006097|Acomosus_v3/1-2196

MSAAQGLAFKLRNESGAHPPPVSLKRNIRHGGRATQYSVSRQQVSSVSLESEFLGAQVPISEEQRIKPWRADGSDPSQRPGAVSASMSFSQLPEKPVGLYDPSFEKDSCGVGFIAELSGEYKRKTVADAIKMLERMAHRGACGCEVNTGDGAGILVALPHGFYREACLFSVTKDAGFELPPPGQYAVGMFFLPTDDKRREQSKTVFTKVAESLGHTVLGWRLVPTDNTDLGESALQTEPVIEQVFLTKSSKSAADFEQQMYILRKLSLVSIRSALLIQTGGARDFYICSLSSRTVVYKGQLKPSQLKDYYYADLGDENFTSYMALVHSRFSTNTFPSWDRAQPMRILGHNGEINTLRGNKNWMKAREGLLKCKQLGLSKDEMTQILPIVDSTSSDSGAFDGVLELLVRAGRSLPEAVMMMIPEAWQNDENMDPDRKALYEYFSALMEPWDGPALISFTDGRYLGATLDRNGLRPGRFYITHSGRVIMASEVGVVDVSPEDVSRKGRLNPGMMLLVDFENHIVVDDEELKKIYSQARPYADWLKRQKISLDDIVGSVPETERVPPSIFGSIPAENRYQNMENLGIHGILTPLKAFGYTVEALDMLLLPMAKDGVEALGSMGNDAPLAVMSNREKLTFEYFKQMFAQVTNPPIDPIREKIVTSMECMIGPEGDLSETTEEQCHRLTLEGPLLSIDEIEAIKKMNYRGWRSKVLDITYPKKRGRKGLEETLDRICAEAHEAIREGYTIIVLSDRGFSSERVAASSLLAVGAVHHHLVSQLERTRIGLLVESAEPREVHHFCTLVGFGADAICPYLAIEAIWRLQIDGKIPPKDDGQFHSRKELVKKYFKASNYGMMKVLAKMGISTLASYKGAQIFEALGIASEVIHKCFEGTPSRIEGATFEMLGRDYLSLHDLAFPSRALPAGSAEAVALPNPGDYHWRKGGEIHLNDPLAIAKLQEAARVNSVAAYKEYSKRIQELNKTCNLRGMLKFKDVAEKIPLEEVEPAKEIVKRFCTGAMSYGSISLEAHTTLATAMNKIGGKSNTGEGGEQPSRMEPLPDGSRNPKRSSIKQVASGRFGVSSYYLTNADELQIKMAQGAKPGEGGELPGHKVIGDIAVTRHSTAGVGLISPPPHHDIYSIEDLAQLIHDLKNSNPGARISVKLVSEAGVGVVASGVVKGHADHVLISGHDGGTGASRWTGIKNAGLPWELGLAETHQTLVANGLRGRAVLQTDGQLKTGRDVAVAALLGAEEFGFSTAPLITLGCVMMRKCHTNTCPVGIATQDPVLREKFAGEPEHVINFFFMLAEELREIMAQLGFRTINEMVGRADMLEVDPEVVKSNPKLENIDLSLMLKPAAEIRPGAAQYCVEKQDHGLDMALDRVLISKSDAALEKGVKVFIETPVKNINRAMGTTLSHEVTKRYHMKGLPPNTIHVKLSGSAGQSFGAFLCSGITLELEGDSNDYVGKGLSGGKIVVYPPRESKFDPKDNIVIGNVALYGATAGEAYFNGMAAERFCVRNSGAQAVVEGVGDHGCEYMTGGTVVILGKTGRNFAAGMSGGIAFVFDVDGKFSSRCNHELIDLESVVEEEDVITLRSMIQQHRSHTGSVLAKEVLADFENLLPKFVKVFPRDYKRILQNMKAEKAAKEAEKKLKKLKGKNGYQETKISVTTSSSKKEVPARNKLQRPTRVDNAAKYRGFIEYEREGIGYRDPNERIGDWKEVASESIPGPLLKTQSARCMDCGTPFCHQENAGAGCPLGNKIPEFNELVHQNRWREALDRLLETNNFPEFTGRVCPAPCEGSCVLGIIENPVSIKSIECAIIDKGFEEKWIVPQPPLQRTGKKVAIVGSGPAGLAAADQLNKMGHFVTVYERADRIGGLMMYGVPNMKTDKLGVVQRRVDLMAEEGVTFVVNANVGKDPNYSINRIRADNDALLLACGSTVPRDLAVPGRELSGVHFAMEFLHANTKSLLDSNLEDGNYISAKGKKVIVIGGGDTGTDCIGTSIRHGCSSIINLELLTKPPNNRAPGNPWPQWPKVFRVDYGHQEAEAKFGKDPRTYEVLTKRFLGDENGNVKGLEVVRVKWERDTSGRFKFTEIDGSSETLEADLVLLAMGFLGPESTLAEKLGFDRDNRSNFKADFGRFSTSVEGVFAAGDCRRGQSLVVWAITEGRQAASEIDKYLMKDEAIITQDPRNLGEDLIQPIAA

>LOC_Os01g48960|Osativa_v7.0/1-2167

MSAAQGMAYKLRTDAAPTGAGRRARRSHSSVAAPYRAARLVQGGVSIEGGLVGGCQLTEERVAARPPRAAARDAEPVRPLSTLPESSIGLYDPSRERDSCGVGFVAELSGDYKRATVNDALEMLERMAHRGACGCEKNTGDGAGILVALPHNFFREVTKDAGFELPQPGEYAVGMVFLPIDEKRRERSKAEFQKVAESLGHVILGWRRVPTDNSDLGESALQTEPVIEQVFLTKSSSSEADFEQQLYILRRLSILSIRAALNLRRGGKRDFYMCSLSSRTIVYKGQLKPCQLKGYYYADLGHENFTSYMALVHSRFSTNTFPSWDRAQPMRVLGHNGEINTLKGNKNWMKAREGLLECEKLGLTKDQFSKILPIVDATSSDSGAFDGVLELLIRGGRSLPEAVMMMIPEAWQNDVNMEPEKKALYEFLSALMEPWDGPALISFTDGRYLGATLDRNGLRPGRFYVTHSGRVVMGSEVGVVDVPSKDVLRKGRLNPGMMLLVDFENHTVVDDEALKAQYSKAHPYGEWLKRQKIYLKDIVESVPETERVAPGISGSLTQKNEKKEHAGVNGIVTPLKAFGYTVEALEMLLLPMAKDGVEALGSMGNDTPLAVMSNREKLTFEYFKQMFAQVTNPPIDPIREKIVTSMECMIGPEGDLLETTEKQCNRLALEGPLVSIDEMEAIKKMNYRGWRSKVLDITYPKKSGRKGLEETLDRICTEARGAIKKGYTVLVLSDRGFSSDRVAVSSLLAVGAVHQHLVANLERTRVGLLVESAEPREVHHFCTLVGFGADAVCPYLAIEAIWCLQNDGKIPPNGDGKPYSKEELVKKYFYASNYGMMKVLAKMGISTLASYKGAQIFEALGLSSEVIRKCFDGTPSRIEGATFEMLARDALRLHELAFPSRAPPPGSADAKALPNPGDYHWRKNGEVHLNDPLAMAKLQEAARVNSRAAYKEYSRRIQELNKTCNLRGMLKFKDTADMISVDEVEPASEIVKRFVTGAMSYGSISLEAHTALAMAMNKLGGKSNTGEGGEQPSRMEPLANGSMNPKRSAIKQVASGRFGVSSYYLTNADELQIKMAQGAKPGEGGELPGHKVIGDIAVTRHSTAGVGLISPPPHHDIYSIEDLAQLIHDLKNSNPRARISVKLVSEAGVGVVASGVVKGHADHVLISGHDGGTGASRWTGIKNAGLPWELGLAETHQTLVANGLRGRAILQTDGQLKTGKDVAVACLLGAEEFGFSTAPLITLGCIMMRKCHTNTCPVGIATQDPVLREKFAGEPEHVINFFFMLAEELREIMSQLGFRTITEMVGRSDMLEVDPEVVKSNEKLENIDLSLILKPAAEIRPGAAQYCVEKQDHGLDMALDNKLIALSKAALEKEVRVFIETPIQNTNRAVGTMLSHEVTKRYHMKGLPAGTIHVKLTGSAGQSLGAFLCPGITLELEGDSNDYVGKGLSGGKIVVYPPRDSTFIPEDNIVIGNVALYGATIGEAYFNGMAAERFCVRNSGAQAVVEGIGDHGCEYMTGGTVVILGKTGRNFAAGMSGGIAYVYDIDGKFSVRCNHELVDLYHVEEEEDITTLKMMIEQHRLNTGSVVARDILSNFDTLLPKFVKVFPRDYKRVLDNMKAEKAAAKLAKEPKISNGVSVTTKKVQPEQSTNRPTRVSNAKKYRGFISYERESISYRDPNERVKDWKEVAIESVPGPLLNTQSARCMDCGTPFCHQESSGAGCPLGNKIPEFNELVHQNRWREALDRLLETNNFPEFTGRVCPAPCEGSCVLGIIENPVSIKSIECAIIDKGFEEGWMVPRPPLQRTGKKVAIIGSGPAGLAAADQLNKMGHFVTVFERADRIGGLMMYGVPNMKTDKIEIVQRRVNLMAEEGITFVVNANVGSDPLYSIERLRSENDAVILACGATKPRDLGIPGRELSGVHFAMEFLHANTKSLLDSNLEDGRYISAKGKKVVVIGGGDTGTDCIGTSIRHGCTSIVNLELLTKPPSKRAADNPWPQWPRIFRVDYGHQEASSKFGNDPRTYEVLTKRFIGDENGNVKALEVVRVKWEKVDGRFQFKEIEGSNETIEADLVLLAMGFLGPEATIAEKLGLEKDNRSNFKAQFGNFATSVDGIFAAGDCRRGQSLVVWAITEGRQAAAAVDKYLSRNEQDAAEDITPSGAGFVQPVAA

>Bradi2g46670|Bdistachyon_v3.2/1-2163

MPTAQGIGLKHAAPPGAGRRARRSQSASAPCRSTRQAHGGMSLEGGFVGGTQPTEGRVAPRPPRAAARDAEPIRPLSKLPESSIGLYNPAFERDSCGVGFIAELSGDENRETVNDAIQMLERMAHRGACGCEKNTGDGAGILVALPHKFFREVTKDAGFELPPPGEYAVGMVFLPTDEKRRDRSKAEFKKVAESLGHSILGWRQVPTDNSDLGESALDTEPAIEQVFLTKSSKSKADFEQQLFILRRLSIISIRAALNLRRGGERDFYMCSLSSRTVVYKGQLMPSQLKGYYYADIGHENFTSYMALVHSRFSTNTFPSWDRAQPMRVLGHNGEINTLRGNKNWMKAREGLLKCEKLGLSEDEMSKILPIVDATSSDSGAFDGVLELLIRGGRSLPEAVMMMIPEAWQNDANMQPEKKALYEFLSALMEPWDGPALISFTDGRYLGATLDRNGLRPGRFYVTHSGRVVMGSEVGVVDIPPQDVLRKGRLNPGMMLLVDFENHTVVDDEALKAQYSKAHPYGEWLKRQKIYLKDIVESVPETDRVAPSIAGSIPQMNDNKECKGINGIVTPLKAFGYTVEALEMLLLPMAKDGVEALGSMGNDAPLAVMSNREKLTFEYFKQMFAQVTNPPIDPIREKIVTSMECMIGPEGDLLEITEKQCNRLAIKGPLVSVDEMESIKKMDYRGWRSKVLDITYPKKSGRKGLEDTLDRICAEAREAIRNGYKILVLSDRGFSSDRVAVSSLLAVGAVHQHLVANLERTRVGLLVESAEPREVHHFCTLVGFGADAICPYLAIEAIWCLQTDGKIPPNGDGQPDSKEELVKKYFYASIYGMMKVLAKMGISTLASYKGAQIFEALGLSSEVIHKCFEGTPSRIEGATFEMLARDALHLHELAFPSRLPPAGSADAKALPNPGDYHWRKNGEIHLNDPLAMAKLQEAAKVNSRAAYKEYSKRIQELNKACNLRGMLKFKDISNKISLDEVEPASEIVKRFCTGAMSYGSISLEAHTALAVAMNKLGGKSNTGEGGEQPSRMEPLPDGSMNPKRSAIKQVASGRFGVSSYYLTNADELQIKMAQGAKPGEGGELPGHKVIGDIAVTRHSTAGVGLISPPPHHDIYSIEDLAQLIHDLKNSNPQARISVKLVSEAGVGVVASGVVKGHADHVLISGHDGGTGASRWTGIKNAGLPWELGLAETHQTLVANGLRGRAVLQTDGQLKTGRDVAVACLLGAEEFGFSTAPLITLGCIMMRKCHTNTCPVGIATQDPVLREKFAGEPEHVINFFFMLAEELREIMAQLGLRSINEMVGRSDMLEVDPEVVKSNEKLENIDLSLILKPAAEIRPGAAQYCVEKQDHGLDMALDNKLIALSKAALEKQVRVFIETPIKNTNRAVGTTLSHEVTKRYHMKGLDSGTIHVKLTGSAGQSFGAFICPGITLELEGDSNDYVGKGLSGGKIVVYPPRNSTFIPEDNIVIGNVALYGATKGEAYFNGMAAERFCVRNSGAITVVEGIGDHGCEYMTGGTVVILGKTGRNFAAGMSGGIAYVYDVDGTFSAHCNHELVDLYHVEEEDDITTLKMMIEQHRLHTGSVLAKDILSKFSSLLPKFVKVFPRDYKRVLEEMKAQKAAASHAKEPKVPNGVSVTTKKIQTEQSTSRPTRVANAKKYRGFISYEREGISYRDPNERVKDWKEVAIESVPGPLLNTQSARCMDCGTPFCHQESSGAGCPLGNKIPEFNELVHQNRWREALDRLLETNNFPEFTGRVCPAPCEGSCVLGIIENPVSIKSIECSIIDKGFEEGWMVPRPPLQRTGKKIAIVGSGPAGLAAADQLNKMGHFVTVFERSDRIGGLMMYGVPNMKTDKIGVVQRRVNLMAEEGITFVVNAHVGSDPLYSIERLRSENNAVILACGATKPRDLSIPGRELSGVHFAMEFLHANTKSLLDSNLEDGRYISAQGKKVVVIGGGDTGTDCIGTSIRHGCSSIVNLELLTKPPSKRAADNPWPQWPRVFRVDYGHQEASNKFGKDPRTYEVLTKRFIGDENGKLKALEVVRVKWEKVDGRFQFKEIEGSQEIIEADLVLLAMGFLGPEATIPEKLGLEKDNRSTFKAQFGHFATSVDGVFAAGDCRRGQSLVVWAITEGREAAAAVDKYLSRDEQNSTQGITPSGAGLVQPITA

>HORVU.MOREX.r3.3HG0280530|Hvulgare_Morex_V3/1-2158

MPTAQGIGLKHAAPPGAGRRARRSHSAAAPGRSARQAHGAMSLEGGFLGGPQRTEERVAPRPPRAAARDAESIRPLSLLPESSIGLYDPAFERDSCGVGFVAELSGVDNRATVVDAIQMLERMAHRGACGCEKNTGDGAGILVALPHNFFREVTKDAGFELPPPGEYAVGMVFLPTDEKRRERSKTEFTKVAESLGHSILGWRQVPTDNSDLGQSALDTEPAIEQVFLTKSSKSKADFEQQLFILRRLSIVSIRAALNLKRGGERDFYMCSLSSRTIVYKGQLMPSQLQGYYYADIGHENFSSYMALVHSRFSTNTFPSWDRAQPMRVLGHNGEINTLKGNKNWMKAREGLLKCEKLGLSQDEMSKILPIVDATSSDSGAFDGVLELLIRGGRSLPEAVMMMIPEAWQNDVNMDPEKKALYEFLSALMEPWDGPALISFTDGRYLGATLDRNGLRPGRFYVTHSGRVVMGSEVGVVDIPAQDVLRKGRLNPGMMLLVDFDNHTVVDDEALKAQYSKAHPYGEWLKRQKIYLKDIVESVPETDRVAPSISGSIPQTNENKECVGINGIVNPLKAFGYTVEALEMLLLPMAKDGVEALGSMGNDAPLAVMSNREKLTFEYFKQMFAQVTNPPIDPIREKIVTSMECMIGPEGDLLEITEKQCNRLALKGPLVSIDEIESIKKMDYRGWRSKVLDITYPKKSGRKGLEETLNRICAEAREAIRKGYKILVLSDRGFSSDRVAVSSLLAVGAVHQHLVANLERTRVGLLVESAEPREVHHFCTLVGFGADAICPYLAIEAIWCLQTDGKIAPTDSKEELVEKYFYASIYGMMKVLAKMGISTLASYKGAQIFEALGLSSEVIQKCFEGTPSRIEGATFEMLARDALRLHELAFPSRTPPPGSADAKALPNPGDYHWRKNGEVHLNDPLAMAKLQEAAKVNSRAAYKEYSKRIQELNKACNLRGMLKFIDSTSKISLDEVEPASEIVKRFCTGAMSYGSISLEAHTALAVAMNKLGGKSNTGEGGEQPSRMEPLSDGSMNPKRSAIKQVASGRFGVSSYYLTNADELQIKMAQGAKPGEGGELPGHKVIGDIAVTRHSTAGVGLISPPPHHDIYSIEDLAQLIHDLKNSNPRARISVKLVSEAGVGVVASGVVKGHADHVLISGHDGGTGASRWTGIKNAGLPWELGLAETHQTLVANGLRGRAVLQTDGQLKTGRDVAVACLLGAEEFGFSTAPLITLGCIMMRKCHTNTCPVGIATQDPVLREKFAGEPEHVINFFFMLAEELREIMAQLGLRTINEMVGRSDMLEVDPEVVKSNEKLENIDLSLILTPAAKIRPGAAQYCVEKQDHGLDLALDNKLIALSTAALEKEVRVFIETPIKNTNRAVGTTLSHEVTKRYHMKGLDPGTIHVKLTGSAGQSLGAFLCPGITLELEGDSNDYVGKGLSGGKIVVYPPRNSTFSPEDNIVIGNVALYGATKGEAYFNGMAAERFCVRNSGARTVVEGIGDHGCEYMTGGIVVILGKTGRNFAAGMSGGIAYVYDVDGTFSARCNPELVDLYHVEEEDDITTLKMMIEQHRLHTESVLAKDILSKFDSLLPKFVKVYPRDYKRVLEEMKAEKAAARSTKEPKVANGVSVTTKKIQTEKSSSRPTRVANAKKYRGFITYEREGVSYRDPNERVKDWNEVAIESVPGPLISTQSARCMDCGTPYCHQESSGAGCPLGNKIPEFNELVHQNRWREALDRLLETNNFPEFTGRVCPAPCEGSCVLGINQNPVSIKSIECSIIDKGFEEGWMVPRPPLQRTGKKIAIVGSGPAGLAAADQLNKMGHFVTVFERSDRIGGLMMYGVPNMKTDKIGVVQRRVNLMAEEGVTFVVNANVGSDPLYSIERLRSENNAVILACGATKPRDLSIPGRDLAGVHFAMEFLHANTKSLLDSNLEDGRYISAQGKKVVVIGGGDTGTDCIGTSVRHGCSRVVNLELLTKPPSKRAADNPWPQWPRVFRVDYGHQEASTKFGNDPRTYEVLTKRFIGDENGKLKALEVVRVKWEKVDGRFQFKEIEGSQEIIEADLVLLAMGFLGPEEKIADQLGLEKDNRSNFKAQFGHFATSVDGIFAAGDCRRGQSLVVWAITEGREAAAAVDKYLSRDEQNAAEGITALGAGLVQRIAA

>Zm00001d043845|Zmays_RefGen_V4/1-2165

MSTAQGTALRSAPLGAGRWARRGRPVAAPYRSGWQAYGGVSLEGSGFLGRVHRTEERVAPRAPHAAARDAEVVRPLSKLPNSSIGLYDSSFERDACGVGFVAELSGDYKRETVNDAIEMLERMAHRGACGCEKNTGDGAGIMVALPHDFFKEVAKDAGFELPPPGEYAVGMFFMPTDEKRREKGKAEFMKVAESLGHVILGWRLVPTDNSDLGESALETEPVIEQVFVTKSSRSEAEFEQQLYILRRLSIISVRASLNIKRGGERDFYMCSLSSRTIVYKGQLKPSQLKGYYYADLGHENFMSYMALVHSRFSTNTFPSWDRAQPMRVLGHNGEINTLRGNKNWMTAREGLLEAEKLGLSKEQLSRILPIVDATSSDSGAFDNVLELLVRGGRSLPEAVMMMIPEAWQNDVNMDPEKKALYEFLSALMEPWDGPALISFTDGRYLGATLDRNGLRPGRFYVTHSGRVIMGSEVGVVDVPPEDVLRKGRLNPGMMLLVDFENHTVVDDEALKAQYSKAHPYGEWLKRQKIYLKDIVESVPETDRVAPSISSSLSQKNENIDHVGISGILTQLKAFGYTVEALEMLLLPMAKDGVEALGSMGNDTPLAVMSNREKLTFEYFKQMFAQVTNPPIDPIREKIVTSMECMIGPEGDLLETTEKQCNRLALKGPLVSIDEMEAIKKMNYRGWRSKVLDITYPKKSGRKGLEETLNRICAEARKAIREGYTILVLSDRGFSSDRVAASSLLAVGAVHQHLVANLERTRIGLLVESAEPREVHHFCTLVGFGADAICPYLAIEAIWCLQNDGKITPNGDGQPYSKEELVAKYFYASNYGMMKVLAKMGISTLASYKGAQIFEALGLSSEVVGKCFEGTPSRIEGATFEMLARDALRLHELAFPSRALPAGSADAKALPNPGDYHWRKNGEIHLNDPLAMGKLQEAARVNSRAAYKEYSKRIQELNKACNLRGMLKFKHVSDKISLDEVEPASEIVKRFCTGAMSYGSISLEAHTALAIAMNKLGGKSNTGEGGEQPSRMEPLPDGSMNPRRSAIKQVASGRFGVSSYYLTNADELQIKMAQGAKPGEGGELPGHKVIGDIAVTRHSTAGVGLISPPPHHDIYSIEDLAQLIHDLKNSNPGARISVKLVSEAGVGVVASGVVKGHADHVLISGHDGGTGASRWTGIKNAGLPWELGLAETHQTLVANGLRGRAVLQTDGQLKTGRDVAVACLLGAEEFGFSTAPLITLGCIMMRKCHMNTCPVGIATQDPVLREKFAGEPEHVINFFFMLAEELREIMANLGFRTITEMVGRSDMLEVDPEVVKSNEKLENIDLSLILKPASEIRPGVAQYCVEKQDHGLDMALDNKLIDLSRTAIEKQVRVFIETPIQNTNRAVGTMLSHQVTKRYHMNGLPAGTIHVKFTGSAGQSFGAFLCPGITLELEGDSNDYVGKGLSGGKIVVYPPRNSSFSPEDNIVIGNVALYGATMGEAYFNGMAAERFCVRNSGAQAVVEGIGDHGCEYMTGGTVVILGKTGRNFAAGMSGGIAYVYDVDGKFSARCNNELVDLYHVEEEDDIITLKMMIEQHRRNTESVLARDILSDFDNLLPKFVKVYPRDYKRVLESMKVEKAVAKHAKEPKMANGISVTTKKVQPDQSASRPTHVANAKKYRGFITYERESISYRDPKERVRDWKEVAIESTPGPLLNTQSARCMDCGTPFCHQESSGAGCPLGNKIPEFNELVHQNRWREALDRLLETNNFPEFTGRVCPAPCEGSCVLGIIENPVSIKSIECAIIDKGFEEGWMIPRPPLQRTGRKVAIVGSGPSGLAAADQLNKMGHFVTVFERADRIGGLMMYGVPNMKTDKIGVVQRRVNLMAEEGVTFVVNANVGSDPLYSIERLRSENDAVILACGATKPRDLTIPGRELSGVHFAMEFLHANTKSLLDSNLEDGKYISARGKKVVVIGGGDTGTDCIGTSIRHGCTSLVNLELLSKPPSTRAADNPWPQWPRIFRVDYGHQEAATKFGKDPRTYGVLTKRFIGDGNGKVKALEVVRVKWEKVDGRFQLKEMEGSEEIIEADLVLLAMGFLGPEATIAEKLGLEKDNRSNFKAQFGDFATSVDGVFAAGDCRRGQSLVVWAITEGRQAAAAVDKYLSRYDQNAAATGDSTPSGTGLVQPVAA

>Zm00001d011610|Zmays_RefGen_V4/1-2163

MSTAQGTALKSAPIGAGHRARRGRPVVPPYRSGWQAYGGVSLEGSGFLGGLQRTEERVATLAPRAAARDAEVVRPLSKLPNSSIGLYDPSFERDACGVGFVAELSGDYKRETVNDAIEMLERMAHRGACGCEKNTGDGAGIMVALPHDFFKEVTKDAGFELPPPGDYAVGMFFMPTDEKRREKGKAEFKKVAESLGHVILGWRPVPTDNSDLGESALETEPVIEQVFVTKSSRSEAEFEQQLYILRRLSIISVRASLNIKRGGERDFYMCSLSSRTIVYKGQLKPCQLKGYYYADLGQENFMSYMALVHSRFSTNTFPSWDRAQPMRVLGHNGEINTLRGNKNWMTAREGLLEAEKLGLSKEQLARILPIVDATSSDSGAFDNVLELLVRGGRSLLEAVMMMIPEAWQNDGNMDPEKKALYEFLSALMEPWDGPALISFTDGRYLGATLDRNGLRPGRFYVTHSGRVIMGSEVGVVDVPPEDVLRKGRLNPGMMLLVDFENHTVVDDEALKAQYSKAHPYGEWLKRQKIYLKDIVESVPETDRVAPSISSSLPAKNENKDHVGINGILTPLKAFGYTVEALDMLLLPMAKDGVEALGSMGNDTPLAVMSNREKLTFEYFKQMFAQVTNPPIDPIREKIVTSMECMIGPEGDLLETTEKQCNRLSLKGPLVSIDEMEAIKKMNYRGWRSKVLDITYPKKSGRKGLEETLNRMCAEAREAIREGYTILVLSDRGFSSDRVAASSLLAVGAVHQHLVANLERTRIGLLVESAEPREVHHFCTLLGFGADAICPYLAIEAIWCLQNDGKIPPNGDGQPNSKEELVKKYFYASNYGMMKVLAKMGISTLASYKGAQIFEALGLSSEVIGKCFEGTPSRIEGATFEMLARDALRLHELAFPSRTPPAGSADAKALPNPGDYHWRKNGEVHLNDPLAMGKLQEAARVNSRAAYKEYSKRIQELNKACNLRGMLKFKDISDKISLDDVEPASEIVKRFCTGAMSYGSISLEAHTALAIAMNKLGGKSNTGEGGEQPSRMEPLPDGSMNPRRSAIKQVASGRFGVSSYYLTNADELQIKMAQGAKPGEGGELPGHKVIGDIAVTRHSTAGVGLISPPPHHDIYSIEDLAQLIHDLKNSNPGARISVKLVSEAGVGVVASGVVKGHADHVLISGHDGGTGASRWTGIKNAGLPWELGLAETHQTLVANGLRGRAVLQTDGQLKTGRDVAVACLLGAEEFGFSTAPLITLGCIMMRKCHMNTCPVGIATQDPVLREKFAGEPEHVINFFFMLAEELREIMANLGFRTITEMVGRSDMLEVDPEVVKTNEKLENIDLSLILKPASEIRPGVAQYCVEKQDHGLDMALDNKLIDLSRTAIEKQVRVFIETPIQNTNRAVGTMLSHEVTKRYHINGLPAGTIHVKFTGSAGQSFGAFLCPGVTLELEGDSNDYLGKGLSGGKIVVYPPRNSSFSPEDNIVIGNVALYGATMGEAYFNGMAAERFCVRNSGAQAVVEGIGDHGCEYMTGGTVVILGKTGRNFAAGMSGGIAYVYDVDGKFSARCNNELVDLYHVEEEDDIITLKMMIEQHRRNTESVLARDILSDFDNLLPKFVKVYPRDYKRVLENMKLEKSVANHAKEPKMVNGISVTTKKVQPDQSASRPTRVANAKKYRGFITYERESISYRDPKERVKDWKEVAIESTPGPLLNTQSARCMDCGTPFCHQESSGAGCPLGNKIPEFNELVHQNRWREALDRLLETNNFPEFTGRVCPAPCEGSCVLGIIENPVSIKSIECAIIDKGFEEGWMIPRPPLQRTGRKVAIVGSGPAGLAAADQLNKMGHFVTVFERADRIGGLMMYGVPNMKTDKIGIVQRRVNLMAEEGVTFVVNANVGSDPLYSIERLRSENDAVILACGATKPRDLTIPGRELSGVHFAMEFLHANTKSLLDSNLEDGKYISARGKKVVVIGGGDTGTDCIGTSIRHGCTSLVNLELLTKPPSNRAADNPWPQWPRIFRVDYGHQEAATKFGKDPRTYEVLTKRFIGDENGKVKALELVRVKWEKVDGRFQLKEIQGSEEIIEADLVLLAMGFLGPEASIAEKLGLEKDNRSNFKAQFGDFATSVDGVFAAGDCRRGQSLVVWAITEGRQAAAAVDKYISRHDQNVAGDITPFGAGLVQPVGA

>Sobic.003G258800|Sbicolor_v5.1/1-2163

MSTAQGTALKSAPMGPGRRARRGRPVAAPYRSGWQAYGGVSLEGGGFLGGVQRTEERVAPRAPRAAARDAEVVRPLSKLPNSSIGLYNPSFERDACGVGFVAELSGDYKRETVNDAIEMLERMAHRGACGCEKNTGDGAGIMVALPHDFFKEVTKDAGFELPPPGEYAVGMFFMPTDEKRREKGKAEFKKVAESLGHVILGWRPVPTDNSDLGESALETEPVIEQVFITKSSRSEAEFEQQLYILRRLSIISVRAALNIKRGGERDFYMCSLSSRTIVYKGQLKPCQLKGYYYADLGHENFTSYMALVHSRFSTNTFPSWDRAQPMRVLGHNGEINTLRGNKNWITAREGLLEAEKLGLSKEQLSRILPIVDATSSDSGAFDNVLELLVRGGRSLPEAVMMMIPEAWQNDGNMDPEKKALYEFLSALMEPWDGPALISFTDGRYLGATLDRNGLRPGRFYVTHSGRVIMGSEVGVVDVPPEDVLRKGRLNPGMMLLVDFENHTVVDDEALKTQYSKAHPYGEWLKRQKIHLKDIVESVPETDRVAPSISSSLPQKNENKDDVGINGILTPLKAFGYTVEALEMLLLPMAKDGVEALGSMGNDTPLAVMSNREKLTFEYFKQMFAQVTNPPIDPIREKIVTSMECMIGPEGDLLETTEKQCNRLALKGPLVSIDEMEAIKKMDYRGWRSKVLDITYPKKSGRKGLEEALDRICAEAREAISKGYTILVLSDRGFSSDRVATSSLLAVGAVHQHLVANHERTRIGLLVESAEPREVHHFCTLVGFGADAICPYLAIEAIWCLQNDGKIPPNGDGQLYSKEELVRKYFYASNYGMMKVLAKMGISTLASYKGAQIFEALGLSSEVIGKCFEGTPSRIEGATFEMLARDALRLHELAFPSRTPPAGSADAKALPNPGDYHWRKNGEIHLNDPLAMGKLQEAARVNSRAAYKEYSKRIQELNKACNLRGMLKFKDISDKISLDEVEPASEIVKRFCTGAMSYGSISLEAHTALAIAMNKLGGKSNTGEGGEQPSRMEPLPDGSMNPRRSAIKQVASGRFGVSSYYLTNADELQIKMAQGAKPGEGGELPGHKVIGDIAVTRHSTAGVGLISPPPHHDIYSIEDLAQLIHDLKNSNPGARISVKLVSEAGVGVVASGVVKGHADHVLISGHDGGTGASRWTGIKNAGLPWELGLAETHQTLVANGLRGRAVLQTDGQLKTGRDVAVACLLGAEEFGFSTAPLITLGCIMMRKCHMNTCPVGIATQDPVLREKFAGEPEHVINFFFMLAEELREIMANLGFRTITEMVGRSDMLEVDPEVVKSNEKLENIDLSLILKPASEIRPGVAQYCVEKQDHGLDMALDNKLIDLSRTAIEKQVRVFIETPIQNTNRAVGTMLSHEVTKRYHMSGLPAGTIHVKFTGSAGQSFGAFLCPGITLELEGDSNDYVGKGLSGGKIVVYPPRNSSFVPEDNIVIGNVALYGATKGEAYFNGMAAERFCVRNSGAQAVVEGIGDHGCEYMTGGAVVILGKTGRNFAAGMSGGIAYVYDVDGKFSARCNNELVDLYHVEEEDDIITLKMMIEQHRRNTESVLARDILSDFDNLLPKFVKVYPRDYKRVLENMKAEKAVAKHAKEPKVANGISVTTKKVQPDQSASRPTRVANAKKYRGFITYERESISYRDPKERVKDWKEVAIESTPGPLLNTQSARCMDCGTPFCHQESSGAGCPLGNKIPEFNELVHQNRWREALDRLLETNNFPEFTGRVCPAPCEGSCVLGIIENPVSIKSIECAIIDKGFEEGWMVPRPPLQRTGRKVAIVGSGPAGLAAADQLNKMGHFVTVFERADRIGGLMMYGVPNMKTDKIGIVQRRVNLMAEEGVTFVVNASVGSDPLYSIERLRSENDAVILACGATKPRDLTIPGRELSGVHFAMEFLHANTKSLLDSNLEDGKYISARGKKVVVIGGGDTGTDCIGTSIRHGCTNLVNLELLSKPPSNRAADNPWPQWPRIFRVDYGHQEAATKFGKDPRTYEVLTKRFIGDENGKVKALEVVRVKWEKVDGRFQLKEIEGSEEIIEADLVLLAMGFLGPEATIAEKLGLEKDNRSNFKAQFGDFATSVDGVFAAGDCRRGQSLVVWAITEGRQAAAAVDKYLSRYDQNAAGDITPSGAGLVQPVAA

>ELECO.r07.1AG0028780|Ecoracana_v1.1/1-2158

MSTAQGAALKSAPLGADRRSRRGRTVAAPSRSVWQAYGGVSLESGGFLGGLQRTEDRVTPRAPRATPRDAEVIRPLSKLPESIGLYNPSFERDSCGVGFVAELSGDYKRETVNDAIEMLERMAHRGACGCEKNTGDGAGILVALPHDFFREVSKDAGFELPPPGDYAVGMFFMPTDEKRREKGKAEFKKVAESLGHTILGWRLVPTDNSDLGESALETEPAIEQVFLSKSSRSNAEFEQQMYILRRLSIRSIRAALDIQRGSERDFYMCSLSSRTIVYKGQLMPSQLKGYYYADLGHENFTSYMALVHSRFSTNTFPSWDRAQPMRVLGHNGEINTVRGNKNWMTSREGLLECEKLGLSQEELSIILPIVDATSSDSGAFDNVLELLVRGGRSLPEAVMMMIPEAWQNDATMEPEKKALYEFFSALMEPWDGPALISFTDGRYLGATLDRNGLRPGRFYVTHSGRVIMGSEVGVVDVPPEDVLRKGRLNPGMMLLVDFENHTVVDDEALKAQYSKAHPYGQWLKRQKIHLKDIIESVPENDRVAPTLTLKDENNGREGINGILTPLKAFGYTVEALDMLLLPMAKDGVEPLGSMGNDTPLAVMSNREKLTFEYFKQMFAQVTNPPIDPIREKIVTSMECMIGPEGDLLETNEKQCNRLTLKGPLISVDEMEAIKKMDYRGWRSKVLDITYPKNSGRKGLEKTLNRICAEAREAVQMGYTVLVLSDRGFSSDRVAVSCLLAVGAVHQHLVVNHERTRTGLMVESAEPREVHHFCTLVGFGADAICPYLAIEAIWCLQNDGKIPPNSDGKPYRKEELVQKYFYASNYGMMKVLAKMGISTLASYKGAQIFEALGLSSEVIDKCFEGTPSRIEGATFEMLARDALRLHELAFPSRTLPPGSADAKALPNPGDYHWRKNGEVHLNDPLAMAKLQEAARVNSRAAYKEYSRRIQELNKTCNLRGMLKFKDISGKISLDEVEPASEIVKRFCTGAMSYGSISLEAHTALAMAMNKLGGKSNTGEGGEQPSRMEPLSDGSMNPRRSAIKQVASGRFGVSSYYLTNADELQIKMAQGAKPGEGGELPGHKVIGDIAVTRHSTAGVGLISPPPHHDIYSIEDLAQLIHDLKNSNPRARVSVKLVSEAGVGVVASGVVKGHADHVLISGHDGGTGASRWTGIKNAGLPWELGLAETHQTLVANGLRGRAVLQTDGQLKTGRDVAVACLLGAEEFGFSTAPLITLGCIMMRKCHTNTCPVGIATQDPVLRAKFAGEPEHVINFFFMLAEELREIMANLGFRTINEMVGRSDMLEVDPEVVKSNEKLENIDLSLILKPAAEIRPGVAQYCIEKQDHSLELALDNKLIVLSRTAIEKQVRVFIESPIRNINRSVGTMLSHEVTKRYHMNGLPADTIHVKFTGSAGQSFGAFLCPGITLELEGDSNDYVGKGLSGGKIIVYPPRNSSFDPEDNIVIGNVALYGATKGEAYFNGMAAERFCVRNSGAQAVVEGIGDHGCEYMTGGTVVILGKTGRNFAAGMSGGIAYVYDVDGKFSSHCNNELVDLYHVEEEDDIITLKMMIEQHRRNTESVVAKTILSDFDNLLPKFVKVFPRDYKRVLESMKAEKAAAKQAKDPKMTNGISVTTKKVQPDQATNRPTRVANAKKYRGFITYERESISYRDPNERVKDWKEVAIESTPGPLLNTQSARCMDCGTPFCHQESSGAGCPLGNKIPEFNELVHQNRWREALDRLLETNNFPEFTGRVCPAPCEGSCVLGIIENPVSIKSIECAIIDKGFEEGWMVPRPPPQRTGKKVAIVGSGPAGLAAADQLNKMGHFVTVFERADRIGGLMMYGVPNMKTDKIEIVQRRVNLMAEEGVTFVVNASVGSDPLYSIERLRSENDAVILACGATKPRDLSIPGRELSGVHFAMEFLHANTKSLLDSNLEDGKYISAKGKKVVVIGGGDTGTDCIGTSIRHGCSKIVNLELLSKPPSKRAADNPWPQWPRIFRVDYGHQEATTKFGNDPRTYEVLTKRFIGDENGKVKALEVVRVKWEKVDGRFQFKEVEGSEEIIEADLVLLAMGFLGPEATIAEKLGLEKDNRSNFKAQFGNFATSVDGVFAAGDCRRGQSLVVWAITEGRQAAAAIDKYLSRDDQNAAEGITPSGAGLVTPVAA

>ELECO.r07.1BG0079010|Ecoracana_v1.1/1-2158

MSTAQGAALKSAPLGADRRARRGRTVAAPSRSVWQAYGGVSLESGRFLGGLQRTEDRVTPRAPRATPRDAEVIRPLSKLPESIGLYDPSFERDSCGVGFVAELSGDYKRETVNDAIEMLERMAHRGACGCEKNTGDGAGILVALPHDFFREVSKDAGFELPPPGEYAVGMFFMPTDEKRREKGKAEFKKVAESLGHTILGWRLVPTDNSDLGESALETEPAIEQVFLSKSSRSNAEFEQQMYILRRLSIRSIRAALDIQRGSERDFYMCSLSSRTIVYKGQLMPSQLKGYYYADLGHENFTSYMALVHSRFSTNTFPSWDRAQPMRVLGHNGEINTLRGNKNWMTAREGLLECEKLGLSQEELSIILPIVDATSSDSGAFDNVLELLVRGGRSLPEAVMMMIPEAWQNDANMEPEKKALYEFFSALMEPWDGPALISFTDGRYLGATLDRNGLRPGRFYVTHSGRVIMGSEVGVVDVPPEDVLRKGRLNPGMMLLVDFENHTVVDDEALKAQYSKAHPYGQWLKRQKIHLKDIVESVPENDRVAPTLTQTDENNGREGITGILSPLKAFGYTVEALDMLLLPMAKDGVEPLGSMGNDTPLAVMSNREKLTFEYFKQMFAQVTNPPIDPIREKIVTSMECMIGPEGDLLETNEKQCNRLTLKGPLISVDEMEAIKKMDYRGWRSKVLDITYPKKSGRKGLEKTLDRICAEAHEAVQMGYTVLVLSDRGFSSDRVAVSSLLAVGAVHQHLVVNHERTRIGLMVESAEPREVHHFCTLVGFGADAICPYLAIDAIWCLQNDGKIPPNSDGKPYRKEELVQKYFYASNYGMMKVLAKMGISTLASYKGAQIFEALGLSSEVIDKCFEGTPSRIEGATFEMLARDALRLHELAFPSRTLPPGSADAKALPNPGDYHWRKNGEVHLNDPLAMAKLQEAARVNSQAAYKEYSRRIQELNKTCNLRGMLKFKDISGKISLDEVEPASEIVKRFCTGAMSYGSISLEAHTALAMAMNKLGGKSNTGEGGEQPSRMEPLPDGSMNPRRSAIKQVASGRFGVSSYYLTNADELQIKMAQGAKPGEGGELPGHKVIGDIAVTRHSTAGVGLISPPPHHDIYSIEDLAQLIHDLKNSNPRARVSVKLVSEAGVGVVASGVVKGHADHVLISGHDGGTGASRWTGIKNAGLPWELGLAETHQTLVANGLRGRAVLQTDGQLKTGRDVAVACLLGAEEFGFSTAPLITLGCIMMRKCHTNTCPVGIATQDPVLRAKFAGEPEHVINFFFMLAEELREIMANLGFRTITEMVGRSDMLEVDPEVVKSNEKLENIDLSLILKPAAEIRPGVAQYCVEKQDHSLELALDNKLIVLSRAAIEKQVRVFIESPIQNINRSVGTMLSHEVTKRYHMNGLPADTIHVKFTGSAGQSFGAFLCPGITIELEGDSNDYVGKGLSGGKIIVYPPRNSSFDPEDNIVIGNVALYGATKGEAYFNGMAAERFCVRNSGAQAVVEGIGDHGCEYMTGGTVVILGKTGRNFAAGMSGGIAYVYDVDGKFSSHCNNELVDLYDVEEEDDIITLKMMIEQHRRNTESVVAKTILSDFDNLLPKFVKVFPRDYKRVLESMKAEKAAAKQAKDPKMTNGISVTTKKVQPDQTTNRPTRVANAKKYRGFITYERESISYRDPNERVKDWKEVAIESTPGPLINTQSARCMDCGTPFCHQESSGAGCPLGNKIPEFNELVHQNRWREALDRLLETNNFPEFTGRVCPAPCEGSCVLGIIENPVSIKSIECAIIDKGFEEGWMVPRPPPQRTGKKVAIVGSGPAGLAAADQLNKMGHFVTVFERADRIGGLMMYGVPNMKTDKIEIVQRRVNLMAEEGVTFVVNASVGTDPLYSIERLRSENDAVILACGATKPRDLSIPGRELSGVHFAMEFLHANTKSLLDSNLEDGKYISAKGKKVVVIGGGDTGTDCIGTSIRHGCSKMVNLELLSKPPSKRAADNPWPQWPRIFRVDYGHQEAATKFGNDPRTYEVLTKRFIGDENGKVKALEVVRVKWEKVDGRFQFKEVEGSEEIIEADLVLLAMGFLGPEATIAEKLGLEKDNRSNFKAQFGNFATSVDGVFAAGDCRRGQSLVVWAITEGRQAAAAVDKYLSRDDQNAAEGITPSGAGLVTPVAA

>LOC_Os05g48200|Osativa_v7.0/1-2188

MSAAQGLALKLRAAPAAGGVRGEKRRRAASATAAAAARPRHGAMSLEGGFLGGALPAEDRVAPRASASRQAEAGAGAGAARPPPRSMSKIPESSIGLYDPSMERDSCGVGFIAELSGEYSRKTVDDAIEMLDRMAHRGACGCEKNTGDGAGILVALPHNFFREVTKDAGFELPPPGEYAVGMFFMPTDDKRREKSKLLFREKAELLGHTVLGWRRVPTDNSGLGQSAVDTEPVIEQVFVTKSASSKADFERQMYVLRRFSVMSIREVLGVKNGGTKDFYMCSLSSRTIVYKGQLKPSQLKGYFFADLGDESFTSYMALIHSRFSTNTFPSWDRAQPMRVLGHNGEINTLRGNKNWMKAREGLLKCEGLGLTRDEMLKLLPIVDATSSDSGAIDNVLELLIQSGRSAPEAVMMMIPEAWQNDVNMDPERKALYEFFSALMEPWDGPALISFTDGRYLGATLDRNGLRPGRFYVTYSGRVIMASEVGVVDVPPQDVSRKGRLNPGMMLLVDFENHCVVNDDELKKEYSKVRPYGEWLKRQRIQLTDIIESVNEAERIAPSISGALPITKENKADMGICGILTPLKAFGYTREALEMLMLPMAKDGQEALGSMGNDTPLAVMSNREKLTFEYFKQMFAQVTNPPIDPIREKIVTSMECMIGPEGDLSETTERQCHRLTLKSPLLNTNEMEAIKKMNYRGWRSKVLDITYPKKNGRMGLKQTLDKICAQAREAIHEGYTILVLSDRGFSSERVAVSSLLAVGAVHQHLVSHLERTRIGLLVESAEPREVHHFSTLIGFGADAICPYLAIEAIWRLQIDGRIPPNDGKPYTQEQLIEKYFYASNYGMMKVLAKMGISTLASYKGAQIFEALGLASEVVSKCFEGTPSRVEGATFEMLAQDALRLHEIAFPSRTLPPGSADANALPNPGDYHWRKNGEVHLNDPFSIAKLQEAARINSREAYKEYSRRIYELNKACTLRGMLKFREIPNQISLDEVEPAKEIVKRFCTGAMSYGSISLEAHTSLAEAMNTLGGKSNTGEGGEQPCRMVPLPDGSKNPRISAIKQVASGRFGVSIYYLTNAVEVQIKMAQGAKPGEGGELPGHKVIGDIAVTRNSTAGVGLISPPPHHDIYSIEDLAQLIHDLKNANPGARISVKLVSEAGVGIVASGVVKGHADHVLISGHDGGTGASRWTGIKNAGLPWELGLAETHQTLVANGLRGRAVLQTDGQMKTGRDVAVACLLGAEEFGFSTAPLITLGCIMMRKCHTNTCPAGIATQDPVLRAKFAGKPEHVINYFFMLAEEVREIMAQLGFRTVNEMVGRSDMLEIDPKVLEGNEKLENIDLSRLLKPAAEISPGAVQYCVEKQDHGLDMALDNKLIASSTAALRKGVRVFIETPVRNINRAVGTMLSHEVTKRYHIHGLPSDTIHIKLNGSAGQSFGAFLCPGITLELEGDSNDYVGKGLSGGKIVVYPPRNSRFNPQDNIVIGNVALYGATKGEAYFNGMAAERFCVRNSGAQAVVEGIGDHGCEYMTGGTAVILGKTGRNFAAGMSGGIAYVYDVDGKFSSRCNYELVDLYAVVEEDDITTLRMMIQQHRLHTQSDLARDILLNFDTLLPKFIKVYPRDYKRVLDKLKEEKAAKEAEQKAREVVDKKPVEVIQAPNGISVKTEKVMNEEPSSRPSRVSNAVKYRGFIKYEREGTSYRDPNERVKDWNEVAIELVPGPLLKTQSARCMDCGTPFCHQEGSGAGCPLGNKIPEFNELVHQNRWHEALDRLLETNNFPEFTGRVCPAPCEGSCVLGIIDNPVSIKSIECAIIDKGFEEGWMVPRPPLRRTGKRVAIVGSGPAGLAAADQLNKMGHFVTVFERADRIGGLMMYGVPNMKADKEGIVQRRVELMAKEGVQFIVNAHVGSDPLYSVEKLRSENDAIILACGATKPKDLPIPGRELAGIHFAMEFLHANTKSLLDSNLEDGNYISAQGRKVVVIGGGDTGTDCIGTSIRHGCTNLVNLELLPEPPRKRAPDNPWPQWPRIFRVDYGHQEATSKFGKDPRSYKVLTKRFIGDENGNVKALEVIRVEWGKVNGRFQFKEVEGSEEIIEADLVLLAMGFLGPEATVANKLGLEQDMRSNFKAQFGNFATNVEGVFAAGDCRRGQSLVVWAITEGRQAAAAVDNYLSKDDEGETNGTEDIAVSSEGLVQPVVA

>Sobic.009G225700|Sbicolor_v5.1/1-2200

MSAAQALALKLRAAPAPGRPRGCRTTVRAAAAAHVGALQHRRCVSLAQGGRSLRGAALLAAQQRPRPLPWAERASARRHDGVGVVASASSAAAGVRSMSRIPKGNTGLYDPSMDRDSCGVGFIAELSAQPSRKTIVDAIEMLERMSHRGACGCEKNTGDGAGILVALPDAFFREVTKDAGFELPPLGEYAVGMFFMPLDDERREKSKLVFHEIAKSLGHVVLGWRRVPTDNSDLGKAALETEPMIEQVFVSKSIHSKADIEQQMYILRGLSIKSIHEALGLEHGGPNDFYMCSLSSRTVVYKGQLKPSQLKGYFFADLGDQRFTSYMALVHSRFSTNTFPSWDRAQPMRVLGHNGEINTLRGNKNWMTAREGLLECKGLGLSRDEMSKLLPIVDPTSSDSGAFDNVLELLIRSGRSLPEAVMMMIPEAWHNDVNMDPERKALYEYFSALMEPWDGPALVSFTDGHYLGATLDRNGLRPGRFYVTYSGRVIMASEVGVVDVPNDDVMRKGRVKPGMILLVDFEKHCIVDDDELKKQYSRARPYGEWLERQKIQLADIIESVPETERGAPRIDMLPQKNENKEAFCIHGILAPLKAFGYTVETLEMLLLPMAKNGVEALGSMGNDTPLAVMSNREKMPFDYFKQMFAQVTNPPIDPIREKIVTSMECMIGPEGDLSETTEHQCHRLKLQGPLLHIDEMEAIKKMNFRGWRSKVLDITYPKVYGRQGLEQTLDKLCAQAREAIHQGYTILILSDRGFSPDRVPVSSLLAVGAVHQHLVLNLDRTRIGLLVDSAEPREVHHFCTLLGFGADAICPYLAIEAIWRLQIDEKIPPKDDGQLYPKELIDKYFYASNNGIMKVLAKMGISTLASYKGAQIFEALGLASEVVSKCFEGTPSRVEGSTFEMLAQDALHLLKLAFPSRTLPPGSAEANSLPNPGDHHWRKNGELHLNDPFSIAKLQEAARLDSREAYKEYSRHTEELSKSCTLRGMLKFREIPVRISLDEVEPASEIVKRFCTGAMSYGSISLEAHTTMARAQNIKRAKSNTGEGGEQPSRMEPLPDGSMNPLSSAIKQVASGRFGVSIYYLTNAIELQIKMAQGAKPGEGGELPGHKVIGDIAVTRHSTAGVGLISPPPHHDIYSIEDLAQLIYDLKSSNPGARISVKLVSEAGVGVVASGVVKGHADHILISGHDGGTGASRWTGIKNAGLPWELGLAETHQTLVANGLRARVVLQTDGQLKIGRDVVIACLLGAEEFGFSTAPLIALGCIMMRKCHTNTCPVGIATQDPVLREKFSGKPEHLINFFFMLAEEVREIMSQLGFRTINEMVGRSDMLEVDPDVLKGNEKLQNIDLSLILKPAAEISPEAVQYCVEKQDHGLDKALDNKLIASSRAALEKRFRVFIETLIKNTDRAVGTMLSHEVTKLFRMPGLPPDTIHVKLNGSAGQSFGAFLCPGVTLELEGDSNDYVGKGLSGGKIVVYPPRNSRFIPQDNIVIGNVALYGSTKGEAYFNGMAAERFCVRNSGAQAVVEGIGDHGCEYMTGGTVVILGKTGRNFAAGMSGGIAYVYDVDGMFSTRCNHELVDLYNVDEEDDITTLRVMIEQHRLNTESVLAKDILSNFEDLLPKFVKVFPRDYKRVLDNMKAEKVAKEAEQKMRKKGWDKKAGEMIKAPNGISVITKKVQNKKSSSRPTQVFNAERPRGFVEYEQQGISYRDENERVKDWGEVTNELVPGPLLNTQSARCMSCSTPFCHQENFGAGCPLGNKIPEFNELVYQNRWREALDRLLETNNFPEFTGRVCPAPCEGSCVLGIIENPVSIKSIECAIIDKGFKEGWMVPRPPLQSQRTGMTVAIVGSGPAGLAAADQLNKMGHYVVVFERADRIGGLMMYGVPNMKADKARIVKRRVDLMDKEGVKFIVNAHVGTDPRYSIERLRSENDAVILACGATRPRDLPIPGRELSDIHFAMDFLHSNTKSLLDTNLEDGNYISARGKKVVVIGGGDTGTDCIGTAIRHGCSNLVNLELLPEPPRERAPDNPWPQWPRIFRRDYGHQEAASKFGKDPRTYQILTKRFIGDENGKVKALEVVRVEWSKVDGRFQFKEIEGSQEIIEADLVLLAMGFLGPEAAIAKKLGLEQDNRSNFKAQFENFATNVEGVFAAGDCRRGQSLVVWAIAEGREAAAAVDKYLSREKTNVDENVVAPGPCECLVQPVAA

>ELECO.r07.5BG0411960|Ecoracana_v1.1/1-2195

MSASQGLALKLRAAPPASGRRRGAPRARPMVTAAHGGALQHRGCGGVSLGSGLFIHGAPLPAAPERARSAPPRAPCAARRHDGVAASAAAEVRSMSKVPESSIGLYNPSFERDSCGVGFIAELSAEPSRKTVDDAIEMLERMSHRGACGCEKNTGDGAGILVALPHAFFGEVTKDSGFEFPLPGEYAVGMFFMPTDEGRREKSRIAFHEIAKSLGHAVLGWRRVPTDNSDLGRSALETEPVIEQVFVSKSSRSSVDFEQQMYILRRLSIKSIREALDLQHGGPKDFYMCSLSSRTIVYKGQLKPTQLKGYFFADLGDENFTSYMALVHSRFSTNTFPSWDRAQPMRVVGHNGEINTLRGNKNWMKAREGLLKCKELGLSRDEMAKLLPIVDATSSDSGAFDNVLELLIQSGRSIPEAVMMMIPEAWQNDVNMDPKRKALYEYFSALMEPWDGQALVSFTDGRYLGATLDRNGFRPGRFYVTYSGRVIMASEVGVVDVPNEDVMRKGRLNPGMMLLVDFENHCVVDDDALKSQYSKAQPYGEWLEREKIQLADIIESVPETERAASLISGVHPQKTREAMGIHGILAPLKAFGYTTEALEMLLLPMAKSGIEALGSMGNDTPLAVMSNREKLTFEYFKQMFAQVTNPPIDPIREKIVTSMKCMIGPEGDLSETTEQQCHRLELKGPLLDIKEMEAIKKMNFRGWRSKVLDITYPKTRGRKGLEQTLDKICAQAREAIHEGYTILVLSDRGFSPDHVPVSSLLAVGAVHQHLVSTLERTRVGLLVDSAEPREVHHFCALIGFGADAICPYLAIEAIWRLQIDAKIPPNDDGQPYPQEELVKKYFNASNYGIMKVLAKMGISTLASYKGAQIFEALGLASEVVSKCFEGTPSRVEGATFEMLAQDALRLHELAFPSRTLPLGSADANALPNPGDYHWRKNGEVHLNDPVAIAKLQEAARINSREAYKEYSRTIQELNKACTLRGMLKFKEIPVKISLDEIEPASEIVKRFCTGAMSYGSISLEAHTSLAEAMNRMGAKSNTGEGGEQPSRMEPLPDGSRNPRISAIKQVASGRFGVTIYYLTNAVELQIKMAQGAKPGEGGELPGHKVIGDIAVTRNSTAGVGLISPPPHHDIYSIEDLAQLIHDLKNSNPGARISVKLVSEAGVGVVASGVVKGLADHVLISGHDGGTGASRWTGIKNAGLPWELGLAETHQTLVANGLRGRAVLQTDGQLKIGRDVVIACLLGAEEFGFSTAPLITLGCIMMRKCHTNTCPVGIATQDPVLREKFAGKPEHVINYFFMLAEEVREIMSQLGFRTIDEMVGRSDMLEVDPDVLRGNEKLENIDLSLILKPAAEINAGAIQYCVEKQDHGLDMALDNKLIASSRAALEKGFRIFTETPVQNTNRAVGTMLSHEVTKRYHMHGLPSDTIHVKLNGSAGQSFGAFVCPGITLELEGDSNDYVGKGLSGGKIVVYPPRNSRFVPHENIVIGNVALYGATKGEAYFNGMAAERFCVRNSGAQAVVEGIGDHGCEYMTGGTVVILGKTGKNFAAGMSGGIAYVYDVDGKFNTRCNHELVDLYGVVEEDDIITLRTLIEQHRRNTESILAKDILSNFDELLPKFIKVFPRDYKRVLDMQKAAKEAEEKERKIGVDKKLDVRVVGGGRGGGGALFEEKNDERKSSRPTQVANARNDRGFMTYEREGISYRDPNERIKDWNEVAIELVPGPLLNTQSARCMGCGTPFCHQESSGAGCPLGNKIPEFNELVHQNRWREALDRLLETNNFPEFTGRVCPAPCEGSCVLGIIENPVSIKSIECAIIDKGFQEGWMVPRPPLQRTGKNVAIVGSGPSGLAAADQLNKMGHLVTVFERDDRIGGLMMYGVPNMKTDKTEIVQRRVDLMAKEGVSFVVNANVGTDPLYSIDRLRSENDAVILACGATKPRDLPIPGRELSGIHFAMEFLRANTKSLLDTNLEGGNYISAQGKKVVVIGGGDTGTDCIATSVRHGCRNLVNLELLPEPPRGRAPDNPWPQWPRIFRIDYGHQEAVTKFGKDPRNYQILTKCFVGDENGKVKAVEVVRVEWEKVDGRFQFKEVQGSQETIEADLVLLAMGFLGPEAVIAKKLGLERDKRSNFKAQFGNFATNVEGVFVAGDCRRGQSLVVWAISEGREAAAAVDKYLSRDEMINTEENIAAPCPSDDLVQAVAA

>ELECO.r07.5AG0363860|Ecoracana_v1.1/1-2186

MSAAQGLALKLRAAPPASGRRRGAPRARTTVTAAHGGALQHRGCGGVSLGSGLFIHGAPLPAAQERARSAPRAPCAARRHDGVAAAEVRSMSKVPESSIGLYNPSFERDSCGVGFIAELSAEPSRKTVDDAIEMLERMSHRGACGCEKNTGDGAGILVALPHAFFGEVTKDSGFELPLPGEYAVGMFFMPTDEGRREKSRIVFHEIAKSLGHAVLGWRRVPTDNSDLGRSALETEPVIEQVFVSKSSCSSVDFEQQMYILRRLSIKSIREALDLQHGGPKDFYMCSLSSRTIVYKGQLKPTQLKGYFFADLGDENFTSYMALVHSRFSTNTFPSWDRAQPMRVVGHNGEINTLRGNKNWMKAREGLLKCKELGLSRDERAKLLPIVDATSSDSGAFDNVLELLIQSGRSIPEAVMMMIPEAWQNDVNMDPERKALYEYFSALMEPWDGPALVSFTDGRYLGATLDRNGLRPGRFYVTYSGRVIMASEVGVVDVPNEDVMRKGRLNPGMMLLVDFENHCVVDDDALKSQYSKAQPYGEWLERQKIQLADIIESVPETERVASLISGVRPQKTREAMGIHGILAPLKAFGYTTEALEMLLLPMAKSGVEALGSMGNDTPLAVMSNREKLTFEYFKQMFAQVTNPPIDPIREKIVTSMKCMIGPEGDLSETTQQQCHRLELKGPLLDIKEMEAIKKMNLRGWRSKVLDITYPKTRGRKGLEQTLDKICAQAREAIHEGYTILVLSDRGFSPDHVPVSSLLAVGAVHQHLVSTLERTRVGLLVDSAEPREVHHFCALIGFGADAICPYLAIEAIWRLQIDAKIPPNDDGQPYPQEELVKKYFNASNYGIMKVLAKMGISTLASYKGAQIFEALGLASEVVSKCFEGTPSRVEGATFEMLAQDVLRLHELAFPSRTLPLGSADANALPNPGDYHWRKNGEVHLNDPVAIAKLQEAARINSREAYKEYSRTIQELNKACTLRGMLKFKEIPVKISLDEIEPASVIVKRFCTGAMSYGSISLEAHTSLAEAMNRMGAKSNTGEGGEQPSRMEPLPDGSRNPRISAIKQVASGRFGVTIYYLTNAVELQIKMAQGAKPGEGGELPGHKVIGDIAVTRNSTAGVGLISPPPHHDIYSIEDLAQLIHDLKNSNPGARISVKLVSEAGVGVVASGVVKGHADHVLISGHDGGTGASRWTGIKNAGLPWELGLAETHQTLVANGLRSRAVLQTDGQLKIGRDVVIACLLGAEEFGFSTAPLITLGCIMMRKCHTNTCPVGIATQDPVLREKFAGKPEHVINFFFMLAEEVREIMSQLGFRTIDEMVGRSDMLEVDPDVLRGNEKLENIDLSLILKPAAEISAGAIQYCVEKQDHGLDMALDNKLIASSRAALEKGFRVFTETPVQNTNRAVGTMLSHEVTKRYHMHGLPSDTIHVKLNGSAGQSFGAFMCPGITLELEGDSNDYVGKGLSGGKIVVYPPRNSRFVPHENIVIGNVALYGATKGEAYFNGMAAERFCVRNSGAQAVVEGIGDHGCEYMTGGTVVILGKTGKNFAAGMSGGIAYVYDVDGKFNTRCNHELVDLYGVAEEDDIITLRTLIEQHRRNTESILAKDILSNFDELLPKFIKVFPRDYKRVLDMQKAAKEAEEKERKVGVDKKAIQVNKSLNDTSVFIKDGRKSSRPTQVANARNDRGFMTYEREGISYRDPNERVKDWNEVAIELVPGPLLNTQSARCMGCGTPFCHQESSGAGCPLGNKIPEFNELVHQNRWREALDRLLETNNFPEFTGRVCPAPCEGSCVLGIIENPVSIKSIECAIIDKGFQEGWMVPRPPLQRTGKNVAIVGSGPSGLAAADQLNKMGHLVTVYERDDRIGGLMMYGVPNMKTDKTGIVQRRVDLMVKEGVSFVVNANVGADPLYSIDRLRSENDAVILACGATKPRDLPIPGRELSGIHFAMEFLRANTKSLLDTNLEDGNYISAQGKKVVVIGGGDTGTDCIATSVRHGCRNLVNLELLSEPPRGRAPDNPWPQWPRIFRIDYGHQEAVSKFGKDPRNYQILTKRFIGDENGKVKAVEVVRVEWEKVDGRFQFKEVQGSQETIEADLVLLAMGFLGPEAVIAEKLGLERDKRSNFKAQFGNFATNVEGVFAAGDCRRGQSLVVWAISEGREAAAAVDKYLSRDEMINTEENIAAPCPSDGLVQAVAA

>Bradi2g17192|Bdistachyon_v3.2/1-2159

MSAAQGLALKLRAAPASGGRRAPRGRIFAACGPASRQRYGSGGVSLDGGFLRLPAAVARAAPRAPRASSSDFAAEEGAEAPLPPWSMSKIPESSIGLYDPSFERDSCGVGFIAELSAEYSRKTVVDAIEMLERMAHRGACGCEKNTGDGAGILVALPHEFFRKVVKDAGFELPSPGDYAVGMFFMPRDDQLREKSKLVFREIAEKLGHVVLGWRRVPTDNLDLGKSALDTEPVIEQVFVTKSQRSEAEFEQQMYILRRFSIVSIREALGAQKGQKNFYMCSLSSRTIVYKGQLMPSQLKGYFFADLGDESFTSYMALVHSRFCTNTFPSWDRAQPMRILGHNGEINTLRGNKNWMKAREGLLKCKGFGLSRDEMSKLLPIVDSTSSDSGAFDNVLELLVQSGRSVAEAVMMMIPEAWQNNVDVDPERKALYEYFSALMEPWDGPALVSFTDGRYLGATLDRNGLRPGRFYETYSGRVIMASEVGVVDVLPEDVMTKGRLNPGMMLLIDFEKHCVVDDDALKKQYAKAHPYGEWLKTQKIELRDIIESVSATERIAPMISGALREPVGVNGILAPLKAFGYTLETLDMLLLPMAKDGAEALGSMGNDTPLAVMSNREKLTAEYFKQMFAQVTNPPIDPIREKIVTSMECMVGPEGDLSETTERQCHRLTLKSPLLHIDEIEAIKNMNYRGWRSKVLDITYPKKYGKKGLEQTLDKVCAQAREAIQEGYKILIISDRGFSPDHVAVSSLLAVGAVHQNLVSNHERTHVGLLVESAEPHEVHHFCTLIGYGADAICPYLAIEVICRLQIDGRIPCTDGEQPYTQEQLAQKYFNASNYGMMKVLAKMGISTLASYKGAQIFEALGLASEVISKCFEGTPSKVEGAKFDMLANDALRLHDLAFPSRSWPHGSAEANALPNPGNYHWRKNGEVHLNDPLSIAKLQEAARINSKEAYKEYSRLIQDNNKACTLRGMLKFRETRDRISLDEVEPASEIMKRFCTGAMSYGSISLEAHTTLAEAMNILGGKSNTGEGGELPSRMELLADGSMNPRISAIKQVASGRFGVSIYYLTNAIEIQIKMAQGAKPGEGGELPGHKVIGDIAVTRHSTAGVGLISPPPHHDIYSIEDLSQLIHDLKNANPGARISVKLVSEAGVGVVASGVVKAHADHVLISGHDGGTGASRWTGIKNAGLPWELGLAETHQTLVANGLRGRAVLQTDGQLKTGRDVAIACLLGAEEFGFSTAPLITLGCIMMRKCHTNTCPVGIATQDPVLREKFSGKPEHVINYFFMVAEEVREIMSRLGFRTVNEMVGQADMLEVDPEVLKGNEKLENIDLSQILKPASKISPGAAQYCVEKQDHGLHRALDNKLIALSRVALEKGSRVFIETSVRNTNRTVGAMLSHEVTKRYHIHGLPSDTIHVKLNGSAGQSFGAFLCPGITLKLEGDSNDYVGKGLSGGKIVVYPPRNSRFNPQDNIVIGNVALYGSTKGEAYFNGMAAERFCVRNSGAEAVVEGIGDHGCEYMTGGIVVILGKTGRNFGAGMSGGIAYVYDVDGKFSSRCNHELVELYRVVEEEDIKTLRMMIEQHRLNTESHLAKYILSSFEDQLPKFVKVFPRDYKRVLDNLKVEKVAKAAEEKTRKMLMDKKAGEETKASNGSSVVTKQMNDRKPSGQPTQVSNAIKEQGFVIFGREEVSCRDPNERTKDWDELVKNELVPGPLLRTQSARCMGCGTPFCHQESSGAGCPLGNKIPEFNELVHDNRWREALDRLLETNNFPEFTGRVCPAPCEGSCVLGIIENPVSIKTIECAIIDKGFEEGWMVPRPPVHRTGKEVAIVGSGPAGMAAADQLNKMGHRVTVFERSDRIGGLMMYGVPNMKADKTRIVQRRVDLMTEEGITFVVNAHVGRDPSYSIDQLRSKNNAVILACGATKPRDLPIPGRQLSGIHVAMEFLHANTKSLLDSKLEDGNYISAQGKKVVVIGGGDTGTDCIGTSIRHGCSKLVNLELLPEPPTKRAPDNNWPKFPRIFRLDYGHQEAVSKFGKDPRTYQVMAKRFIGDEDGKVKAVEVVHVEWEKVDGRLQFKEVEGSEETIEADLVLLAMGFLGPEVTLADKLGLEQDERSNFKAQFGNFATNVEGVFAAGDCRRGQSLVVWAITEGREAAAAVDKYLSL

>Sphfalx11G060800|Sfallax_v1.1/1-2308

MAAAALRAPLLRLETRNNFVVATPRRQGGGGEATGSSSSSVVSPATTASFSTTTQNKGSRTSCRTLVASPAVYGSRNLLQGSTRYVGLRKEDDEVGCDACKRERRRRRRSSGSQFWWQHLEGPARAPLLRVVKAGLSAVPEGNLGLYDPAFDKDACGVGFVAELSAKPNRKTVTDAIEMLVRMAHRGACGCETNTGDGAGILVALPHDYFSAVAQDDIGCELPAAGEYAVGMIFLPTSETRREESKNVFNKVAESLGHTILGWRRVKTDNSDLGKSALQTEPIVEQVFLTPNSRSSAGFEQQMYILRKISMVAIRAALNLQHGASKDFYICSLSSRTIVYKGQLKPSQLRNYYYADLGDTRFTSYMGLVHSRFSTNTFPSWDRAQPMRVLGHNGEINTLRGNVNWMRAREGLLKCKTLGLSKDELHKLLPIVDAASSDSGAFDGVLEFLVRSGRSLPEAVMMMIPEAWQNDPNMDPERKALYEYFSCIIEPWDGPALISFTDGRFLGATLDRNGLRPGRYYVTHDQRVVMASEVGVVDIAPENVARKGRLNPGMMLLVDFDHHKVVDDNELKHQYSRSHPFGEWIERQKITLKDIVDSVTVKDRVPPPIIGTAKVDSMDDTMENMGIKGLLAPLKAFGYTVENLEMLLVPMATDGAEALGSMGNDTPLAVMSHRSKLSFDYFKQMFAQVTNPPIDPIREAIVTSTECMVGPEGDLTETVEQQCKRLSLKGPILSLEEMEAIKKMEHRDWRTKVIDITFPKSEGADGLEIALDRICAEAHLAVVDGYSMVVLSDRATSVDQVPVSSLLAVGAVHHHLVSSMERTRIGLVVESAEPREVHHFCTLVGFGADAICPYLAIEAIFRLQIDGKIVPKRDGKLHSRDEVLKKYFYATNSGMLKVLAKMGISTLASYKGAQIFEALGLSSEVVDRCFKGTRSRVEGATFAMLAQDMIHLHDMAFPSRLAPLGSADANALPNPGDYYWRKDGEVHMNDPAAIAKLQEAARTNSVQAYKEYSKMIHSLNQKCNLRGMLKFKDVLESQRVPLEEVEPASEIVKRFCTGAMSYGSISLESHTTLAIAMNQIGGKSNTGEGGENPTRLEPLEDGTRNPRRSAIKQVASGRFGVTSYYLTNADELQIKMAQGAKPGEGGELPGHKVIGDIAITRNSTEGVGLISPPPHHDIYSIEDLAQLIFDLKNSNPGARISVKLVSEAGVGVVASGVVKGHADHVLISGHDGGTGASRWTGIKNAGLPWELGLAETHQTLVANDLRGRTVLQTDGQLKTGRDIMVAALLGAEEFGFATAPLITLGCIMMRKCHKNTCPVGIATQDPVLRAKFAGQPEHVVNYLFMVAEEAREIMANLGIRKLDDLVGRADLLEVDKEVLAGNEKLANIDLSLLLRPAAEIRPDAAQHCIQKQDHALDVVLDQQLIRLSRPALELKLPVYLELPVVNVNRAVGTMLSHEVTKRYKLAGLPTDTIHVKLTGSAGQSLGAFMCPGITLELEGDSNDYVGKGLCGGKIVVYPPRASTFDPKENILIGNVALYGATSGEAYFNGMAAERFCVRNSGAKAVVEGVGDHGCEYMTGGIVVVLGQTGKNFAAGMSGGVAFVLDKPGTFRSKCNPGLVDLDPVEDEEDVMTLRAMIQQHHRATNSQLAKEVLAEFDDLLPKFVKVFPRDYKRVVKAMKAKEIAAREEADLSDTDAFEELKKLAAAARKANAPPQNGTPTTATTGVAKKQRPTKITDAVKNGGFVKYEREALPYRPAAERVKDWGEVLAHGNQDALLKTQSARCMDCGTPFCQQETSGCPLGNKIPEWNELVYQGRWREALDRLLETNNFPEFTGRVCPAPCEGSCVLGIIEKPVTIKTMECTIIDKAFEEGWMVPRPPVKRTGKKVAIVGSGPAGLAAADQLNKLGHWVTVYERSDRIGGLMMYGVPNMKTDKVEIVQRRVNLMTEEGIQFVVNAHVGVDPLYSVENLRLNNDSLLLACGATKPRDLPVEGRDLKGIHFAMEFLHKNTKSLLDSGLQDGNYISAKGKKVVVIGGGDTGTDCIGTALRHECTNLVNLELLPEPPATRANNNPWPQWPRIFRVDYGHEEAKTKFGSDPRSYEVLTKRFIGNEKGEVTGLELVHVWWEKDPSGKFVMQEIAGSEEVIEADLIFLAMGFLGPEQNIAEKLGVERDARSNFKADYGKFATNLQGVFAAGDCRRGQSLVVWAIAEGRQAAAQLDNFLMKDVVPEDISVVPLEEEIGRYAVSSSHQGHGRRQFPEGLNEAKEEARLACLAVAGEEDLGALKECIERSVEATALSDNTTVSEQSF

>Tle4c01921.1|Takakia_lepidozioides/1-2265

MAGTLQAPMILQRAVASPTSCQWVSHKHTSGIPFPTVPASAAFSSKISPKTTPPAIVARAASCLIESHSRSDPLLGGSGCVGLRRDVLKDGSVSLRRDRKRGSRRQFCWQYSEGPSKTPVLKVVKAGLSGVPEGNLGLYDPTLDKDSCGVGFVAELSAKPSRKTVTDAVEMLVRMAHRGACGCETNTGDGAGILVAIPHDYFSAVAKEEIGCDLPPLGKYAVGMVFLPTSETRRSESKHVFNKVAESLGHTILGWRQVKTDNSDLGKSALQTEPVVEQVFLSSSSRFSADFEQQMYILRKTSMVAIRAALNLQHGATKDFYICSLSARTIVYKGQLKPDQLRNYYFADLGDERFTSYMGLVHSRFSTNTFPSWDRAQPMRVLGHNGEINTLRGNVNWMQAREGLLKCNALGLSKDELQKLLPIVDASSSDSGAFDGVLELLVRSGRTLPEAVMMMIPEAWQNNLNMDADRRALYEYFSAIIEPWDGPALISFTDGQYLGATLDRNGLRPGRYYVTYDNRVIMASEVGVVDIEPENVARKGRLSPGMMLLVDFNKHKVVDDNELKQQYSSRYPYAEWLHRQKISLKEIVDSVPEQDRVPPCIIGTVKLDSTDETMGNMGTKGLLVPLKAFGYTVEIMEMLLLPMARDGTEALGSMGNDTPLAVMSNLPKLSFEYFKQMFAQVTNPPIDPIREAIVTSTECMVGPEGDLTETVEEQCHRLSLKGPLLSLEEMEAIKKMEHRGWCTKVIDMTSPKAEGPEGLEIALNRICAEARLAIADGYTMLVLSDRATSAQRVPVSSLLAVGAVHHHLVSTLERAQVGLVVESAEPREVHHFCTLVGFGADAICPYLAIEAIFSFQVDGKIPPKKDGKFHSREQLVEKYFKASNSGMLKVLAKMGISTLASYKGAQIFEALGLSSDVVQRCFRGTQSRVEGATFEMLARDTLRMHDIAFPSRLQPIGSADANALPNPGNYHWRKDGEVHLNDPVAIAKLQEAARTNSVAAYKEYSRMIQVMNKMCNLRGMLKFKAVPESERVPLEEVEPASEIVKRFCTGAMSYGSISLEAHTTLAMAMNTIGGKSNTGEGGENPLRLEPLKDGSMNPKRSAIKQVASGRFGVTSYYLTNADELQIKMAQGAKPGEGGELPGHKVIGDIAITRNSTEGVGLISPPPHHDIYSIEDLAQLIFDLKNANPGARISVKLVSEAGVGVVASGVVKGHADHVLIAGHDGGTGASRWTGIKNAGLPWELGLAETHQTLVANDLRGRTVLQTDGQLKNGRDILIAALLGAEEFGFSTAPLITLGCIMMRKCHKNTCPVGVATQDPVLREKFKGEPEHVVNFFFMVAEEARELMAELGVRRMDDLIGRADLLEVDKEVMADNEKLANIDLSLLLRPAAEIRPEAAQRCVQKQDHALEMVLDQQLIKLSRPALNRKLPVYLEMPVVNVNRAVGTMLSHEVTKRYKLEGLPADTIHVKLNGSAGQSLGAFMCPGITLELEGDGNDYVGKGLCGGKVVVYPPKTSTFDPKENILIGNVALYGATSGEAYFNGMAAERFCVRNSGAKAVVEGVGDHGCEYMTGGVVVVLGQTGRNFAAGMSGGMAFVLDKDGSFQSKCNLGLVDLDPVEEEEDVMTLRAMIQQHQRTTNSQLAKEVLANFEELLPKFVKVFPRDFKHVVKEMKAKEAAARAEEAELSKKDAFEELKKLAGAAALLDGSPTQNGASVIKEVEKKQRPTKTMHAVKNGGFVKYERETLPSRPVTERVKNWGEVVAHGNQDELLKTQSARCMDCGTPFCHQETSGCPLGNKIPEWNELVYQGRWREALDRLLETNNFPEFTGRVCPAPCEGSCVLAIIENPVTIKTMECTIIDKAFEEDWMVPRPPVKRTGKKVAIVGSGPAGLAAADQLNKAGHIVTIYERADRIGGLMMYGVPNMKTDKLEVVQRRVDLMVAEGIQFVVNAHVGVDPLFSVESLRLNNDALLLACGATKPRDLPVPGRELKGIHFAMEFLHLNTKSLLDSGLQDGKFISAKGKKVVVIGGGDTGTDCIGTSIRHGCTSLMNLELLPQPSASRAQGNPWPQWPRIFRVDYGHQEAKETFGKDPRSYEVLTKRFIGNENGEITGLELIHVQWGKDENGKFQMQEVAGTEEVIKADLVFLAMGFLGPDQNIAEKLGLERDAHSNFKANYGQYATNRQGVFAAGDCRRGQSLVVWAISEGRQAAAQLDKFLMKETSVPLEEEILRYVALGNGNGRSVSAGAQHIPESLNKAKEEAGLPSSER

>AANG010498|Anthoceros_angustus/1-2240

MAALLRTPFIGRAACQSLDSDSLSTARAEAVAQRLRLPCFVGLRPHHHASSKIAFRRSISSSSNSRKARPCSALRRLTTMALSQVPAERLGLYDPALDKDACGVGFVAQLSAVPSREIVADALEMLVRMAHRGACGCEANTGDGAGIMMAIPHQFYAEIALEQIGCELPSPGAYAVGVMFLPTNPTRRLESKSVFEKVAESLGHQVLGWRAVKTDNTDLGKSALQTEPVVEQVFLTPTPRSTADFEQQMYVLRKTSMVAIKAALNLQPGGVRDFYICSLSSRTVVYKGQLKPDQLKKYYYADLGDSRCTSYMALVHSRFSTNTFPSWDRSQPMRILGHNGEINTLRGNVNWMHAREGLLKCKALGLSPDEMQKLLPIIDASSSDSGAFDGVLEFLVRSGRSLPEAVMMMIPEAWQNDARMDPDRKGLYEYFSALMEPWDGPALIAFSDGRYLGATLDRNGLRPGRYYTTHSGRVIMASEVGVVDVAPEDVKRKGRLNPGMMLLVDFNKHVVVDDDELKKQYSASHPYSEWLQRQKIYLKDIVESVQQELRVPPPLVDAENGTLQTMGLQGILAPLKAFGYTVESLEMLLIPMAQNGTEALGSMGNDTPLAVMSNRPKLIFQYFKQMFAQVTNPPIDPIREAVVTSTECMIGPEGDLTETTEQQCGRLSLKTPFLTLEEMEAIKHSSHLGWRSQVVDITFPKVEGANGLEKALDRICSEARAAISEGYKLIVLSDRAISPTRVPVNSLLAVGAVHQHLVSKLERTRVGLIIESGEPSEVHHFCTLVGFGADAICPYVAVEAIWRLQVDGKIASKKDGSIQTREELVRKYYKASSSGMLKVMAKMGISTLASYKGAQIFEALGLSSEVVQKCFRGTASRVEGATFEMIGKDILRLHDAAFPARRLPAGSADANAIPNPGDYHWRKDGELHLNDPLAMAKLQEAARTNSTVAYKEYSRIICELNKQCNLRGVLKFKVVPERAVPLEDVEPASEIVKRFCTGAMSYGSISLEAHTSLAIAMNQIGGKSNTGEGGENPARLKPLPDGRMNPERSAIKQVASGRFGVTSYYLTNADELQIKMAQGAKPGEGGELPGHKVIGDIATTRNSTAGVGLISPPPHHDIYSIEDLAQLIYDLKNANPGARVSVKLVSEAGVGVVASGVVKGHADHVLISGHDGGTGASRWTGIKNAGLPWELGLAEAHQTLVANDLRGRAVLQTDGQLKTGRDIAVAALLGAEEFGFSTAPLITLGCIMMRKCHKNTCPVGIATQDPVLREKFAGEPEHVINFLFMVAEETRELMAEMGFTKMDDMIGRADMLEVDEEVIAGNEKLSNIDLSLLLHPAADIRPDAAQRCVEKQDHGLQYALDQHLIKLSNAALKNALPVYLEVPVVNVNRAVGTMLSHEVTKKYGQVGLPTDTIHIKLTGSAGQSLGAFLTSGITLELEGDSNDYVGKGLCGGKVVIYPPRNSTFDPQENIVIGNVALYGATSGEAFFNGMAAERFCVRNSGASAVVEGVGDHGCEYMTGGVVVVLGKTGKNFAAGMSGGIAYVLDVNADFHLRCNLGLVDLDPLEEEDAMKLRAMIQQHQRNTKSLRAQEILSNFDTFLPKFVKVFPRDYKRVLKEAKEEELVAKEEISVPQNAFEELKKLSNSARDVKKSSVKITKDPTQRPTKVSDADKRRGFVLYDRESLPYRPADERLHDWNEILAPANVDALLKTQSARCMDCGTPFCQQDKTGCPLGNKIPEWNELVHQGRWREALDRLLETNNFPEFTGRVCPAPCEGSCVLGIIEKPVSIKTMECTIIDKAFEKGWMIPRPPLRRTGKKVAIVGSGPAGLAAADQLNKAGHTVTVYERADRIGGLMMYGVPNMKTDKTGVVQRRVDLLADEGVNFIVNAHVGSDPRYSVENIRKEMDALILACGATKPRDLPVPGRDLQGIHFAMDFLTANTKSLLDSNLNDERFISAAGKKVIVIGGGDTGTDCIGTSIRHGCKEVVNLELLSQPPPTRASDNPWPQWPRVFRVDYGHEEASTKFGRDPRKYEVLTKRFIGDEDGNVKGLEVVRVQWGKDASGKFQMQEISGSEEEIEADLVLLAMGFLGPEQNIAEKLGLDRDARSNFKADYGRFATNLQGVFAAGDCRRGQSLVVWAIAEGRQAAAQVDRFLLRDDSSLMPPSEVDSLSGNGRGSPSGNGRTITGNMYPAGLNEAKEEARQACLAVKHGDDWGALKKCIDLSMETTALSDTSTVSEQSY

>Zosma01g06270|Zmarina_v3.1/1-2079

MAQAEKSIGSLIPRVRGMSLIPDKPIGLYNPSYDKDACGVGFIAELSGDYKRNIVEDGIEMLNRMAHRGACGCEVNTGDGAGILVAIPHDFFKEVAHELGFELPSPGEYAVGMFFLSPDKERREKSKKVFTEVAESLGHGVLGWRSVPTNNSDLGKAALDTEPIIEQVFLSPNVKSKVDFERQMYILRRFSMKSIRSALHLTVGSPKDFYVCSLSSTTIVYKGQLKPTQLNDYYYADIGDARFTSYMVLVHSRFSTNTFPSWDRAQPMRVLGHNGEINTLRGNKNWMKAREGLLKFNELGLSEDDKKNLLPIVDADSSDSGAFDGVLELLVRGGRSMPEAMMMMIPEAWQNDLNIDSERKALYEYFSALMEPWDGPALVSFTDGRYLGATLDRNGLRPGRYYITKSGRVIMASEVGVVDVDPKDVEKKGRLNPGMMLLVDFEHHMVVDDNALKHQYSKARPYADWLKNQKICLNDITDSVPQDQKFPPPIPGTMTKLVNSEANPMGINGLLAPLKTFGYTTEALEMLLLPMAKEAVESLGSMGNDTPLAVMSNRPKITFEYFKQMFAQVTNPPIDPIREKIVTSMECMIGPEGDLSETTESQCNRLSMNGLFLSLDEMEAIKKIDFRGWRTKVLDITFDKKSGSDGLEQTLHRICSEARAAIQQGYKMLVLSDRGFSEDRVPVSSLLAVGAVHQHLVSSLERTRIGLVVESGEPREVHHFSTLVGFGADAVCPYLAIESIWRLQVDGKIPTKNNEEIYSKDELVRRYFYASGYGMMKVLAKMGISTLASYKGAQIFEALGLSTEVVDRCFSGTPTRVEGATFPMLARDALRLHEMGFPSRPLPPGTAEAMALPNPGDYHWRKDGELHLNDPLAMAKLQESARTNSRDAYKDYSKRIHELNKATNLRGMLKFKGGEVAISLDQVEPASEIVKRFCTGAMSYGSISLEAHTTLAIAMNRLGGKSNTGEGGEEPSRMKPLPDGSMNPKRSSIKQVASGRFGVNSYYLTNADELQIKMAQGAKPGEGGELPGHKVVGDIALTRNSTEHVGLISPPPHHDIYSIEDLAQLIHDLKNSNPGARISVKLVSEAGVGVVASGVVKGHADHVLISGHDGGTGASRWTGIKNAGLPWELGLAETHQTLVANGLRGRTILQTDGQLKIGRDVAIAALLGAEEFGFSTAPLITLGCIMMRKCHKNTCPVGIATQDPALRKKFAGEPEHVINFFFLLAEEVREIMAELGLRSINEMVGRSDLLQLDDEVVRSNEKLGNINLDRLLHPSASIRPGASQYCTEIQDHGLDMALDKKLISKSKESLEHGLPVSIEENVRNTNRAVGTMLSHEVTKRYNMNGLPFNTIHIKLRGSAGQSFGAFLCNGITLELEGDSNDYVGKGLCGGQIRVYPFKESQFDPKDNIVIGNVALYGATKGDAYFNGMAAERFCVRNSGARAVVEGVGDHGCEYMTGGIAVILGKTGRNFAAGMSGGVAYVLDVDGKFESRCNKELVDLDRVEDSEDIEILKKMINQHKYATHSKLSKQVLSEFDALLPKFIKVFPRDYKKIVLAERTAAKSGKTLQQQDVPAPLQIKRPSRISKAVKHRGFINYDREALSYRDPAERMLDWEEVNDPSRPGDLLKTQSARCMDCGTPFCHQSVRQDIAGCPLGNKIPEFNELVYQDRWREALERLLETNNFPEFTGRVCPAPCEGSCVLGINENPVSIKTIECSIIDKAFEEGWITARPPLNRTGKKVAIIGSGPSGLAAADQLNKLGHSVTVFERSDRIGGLMMYGVPNMKTLKEAVVQRRVDLLAEEGIDFVVNANVGVDPAYSVQKLRDENDAVILACGASKPRDLPIPGRELSGVHFAMDFLSANTKSLLDSNLKDGNYISASGKKVVVIGGGDTGNDCIGTSIRHGCTNVTNLEIVSKPPSNRAPGNPWPQWPKTFRVDYGHQEAITKFGNDPRSYDVQTKRFVGDKNGVLTGLETVNVSWTKDDKGNFKMSEVSGSEKTIEADLVFISMGFLGPEKTVAQHLGVELDKRSNFKAEFGKFSTNVNGVFAAGDCRRGQSLVVWAITEGRQVASQVDNYLVNSVA

>Mapoly0002s0330|Mpolymorpha_v3.1/1-2331

MASSACSLTAGVRLGEQSGAAISKFSSTTARSSVNNVDLFVKRAGAGASLGLIPSAAIATQSSTQDASSSLRSAAGLACCSRFSGLRKLPARKSKKIQQWLQYTEGPAREAQLRVVQMALSTVPEGQQGLYDPSMDKDSCGVGFVAELSAEPTRSTVTDALEMLVRMAHRGACGCEENTGDGAGIMVALPHKFFSAISEKEIGCKLPAPGEYAVGMFYLPKDKARRVESKAEFKRVAESLGHTLLGWRKVRTNNSGLGQSALATEPVVEQVFLSASSKSKTSLDEQMYLLRKLAMIAIRVALNMPHGAGKDFYICSLSPQTIVYKGQLTPEQVLDYYRADLGDERFTSYMALVHSRFSTNTFPSWDRAQPMRVLGHNGEINTLRGNVNWMHAREGLLKCKNLGLSEAELEKLLPIVDASSSDSGAFDGVLEFLSRSGRSLPEAIMMMIPEAWQNDSNMDQERRALYEYFSAIMEPWDGPALIAFTDGRYLGATLDRNGLRPGRYYITHTGRVIMASEVGVVDIKPEEVSKKGRLNPGMMLLVDFEKHVVVNDDDLKRQVASSQPFGEWLQRQKITLKDIVDSVDAADRVPPSVVGSLQVGSSDTTMEKMGIKGLLAPLRAFGYSVEALEMLLIPMAKDASEALGSMGNDAPLAMMSERPKLLFEYFKQMFAQVTNPPIDPIREAVVTSTECMVGPEGDLTETIEEQCHRLSLKSPLLTLEEMEAIKNMDYRGWRSKVIDITFGKEEGVFGLEKALDRICAEARLAILDGYKMLVLSDRATSPSRVPVNSLLAVGAVHHHLVSTMERTQIGLVIESAEPREVHHFCTLVGFGVDAICPYLAIETIKRLNIDGKIPAKAQGALHSSDELVSKYFYASNSGMLKVLAKMGISTLASYKGAQIFEAVGLATEVVGRCFKGTPSRVEGATFEILARDTLKLHDLAFPVRMLPPGSADANAVPNSGDYHWRKGGEVHLNDPVAIAMLQEAARTNSPAAYKEYSKTIHELNKKINLRGMLRFKVDASKSIPLEEVEPASEIVKRFCTGAMSYGSISLEAHTTLAVAMNKIGGKSNTGEGGEAPERLEPLADGTQNPQRSSIKQVASGRFGVTSYYLTNADELQIKMAQGAKPGEGGELPGHKVIGNIATTRNSTPGVGLISPPPHHDIYSIEDLAQLIFDLKNSNPEARISVKLVSEAGVGVVASGVVKGHADHVLISGHDGGTGASRWTGIKNAGLPWELGLAETHQTLVANDLRGRTTLQTDGQLKTGRDVAIAALLGAEEFGFSTAPLITLGCIMMRKCHLNTCPVGIATQDPVLRRKFAGDPEYVVNFLFMVAEECREIMASLGYRHMDDLVGHAEHLEVDPDVMDNNSKLENVDLSLLLRPAATLRPDAAQRCVQKQDHGLADVLDRTLIRLALPALSRRLPVYIDLPVCNTDRAVGTMLSHELTKRYRAEGLPEGTIHVKLSGSAGQSLGAFLAPGITLELEGDSNDYVGKGLSGGKVVVYPPRSSTFDPKENIIIGNVALYGATSGEAFFNGMAAERFCVRNSGAKAVVEGVGDHGCEYMTGGTVVVLGKTGKNFAAGMSGGVAYVLDVDGKFPSRCNTGLVDLDRIGSDDVEDVMTLQTMIQQHLRHTGSKLAREVLADFDSLLPKFVKVFPRDYKRVLAELKAKKDAAELEEKSLAGKDAFEELKKLSATSNGSGKGNGTANGTVAAAPTKGSTNGASAASLTKEEKPVAVTRPSKIEEAVKHRGFVNYEREALPYRKPEERLTDWKEVTAQGSQDALLKTQSARCMDCGTPFCQQEKSGCPLGNKIPEWNELVHQGRWREALDRLLETNNFPEFTGRVCPAPCEGSCVLGIIEKPVTIKTMECTIIDKAFEEGWMVPRPPMKRTGKRVAIVGSGPAGMAAADQLNKAGHWVTVYERADRVGGLMMYGVPNMKTDKEGVVQRRVDLMAAEGVEFVVNAHVGSDSRYSVDSLRAANDAIILACGATKPRDLVVPGREFAGVHFAMDFLHANTKSLLDSGLQDGKYISAKDKRVVVIGGGDTGTDCIGTSLRHGCKQLINLELLPQPPYTRAQGNPWPEWPRIFRIDYGHEEAKTRFGKDPRTYEVLTKRFIGNDAGEVTGLELVHVKWEKDASGRFQMQEIPGSEEVIEADLVLLAMGFLGPEQNIAEKLGLECDARSNFKADFGRFATNLQGVFAAGDCRRGQSLVVWAIAEGRQAAAQVDEFIMKDFNEEDLVVDDRYMSLSGMGNVAGNGFLSGNGSGNGNGSGNGSSLGKDRQFPEGVVDAAEEARQACVAVAGDAEDYNSMRQCIELSVEAAALSDNTIVTEQSF

>Rn_15020.1_1|Ricciocarpos_natans/1-2696

MDGLVIQTPKQQIRGTFNRVRTTHYLSQDSSILSGQKKKRKTRSQVETRTTVSGLHSHGTGLCLITELGVFGDRSTLPVSTVAEMGVEGKLTGGSTVGRTFLLGEGESHNAAGTLRGRLLAALRTHFEHTPLPGDQTPVNGKDIVGRGFQCYYRHERTATTVVCDRASLVCQAYSSAPVGLFSREATPALEEKRRGEAIRQQRRRIGRFACRVVRRTRERREPTVDRRWEVRSLACCCSTEAHEKREREISTRKRGNSRRRKGMASSACSFTAGSRLAEQTGAATSKISSSSSRSTLNNVDIHVKRASSFPVTVNAGNAITLGVVPSTIAPQSIEQQTSSLRSAASWACSSRFSGLRKLPARKAKKIQQWLQYTEGPGRDALLRVVQMALSQVPEGPQGLYDPSMDKDSCGVGFVAELSAEPTRNTVSCLRHMPQLLMSDARPVSDEVLLVCFHPLREISVTVEVIVSRAHVYACGMTENFVDGILPVELQCRRRLVSGSDAENTTVLRHWLESVFDFHIKDSGGDGLGSDQSRVTVTDALEMLVRMAHRGACGCEENTGDGAGIMVALPHNFFAAIAEKEIGCKLPPPGQYAVGMFYLPTDKGRRVESKAVFKRVAESLGHKLLGWRKVKTDNSGLGKSALETEPVVEQVFLTPSPKSKHGLDEQMYVLRKLAMIQIRVALNMPHGSGKDFYICSLSPQTIVYKGQLTPEQVFQYYRADLGDERFTSYMALVHSRFSTNTFPSWDRAQPMRVLGHNGEINTLRGNVNWMHAREGLLKCKNLGLSEAELEKLLPIVDASSSDSGAFDGVLEFLSRSGRSLPEAVMMMIPEAWQNDQNMDPQRRALYEYFSAIMEPWDGPALIAFTDGRYLGATLDRNGLRPGRYYVTHSGRVVMASEVGVVDIKPEDVSKKGRLNPGMMLLVDFQNHVVVNDDDLKRQVAASQPFGEWLQRQKLTLKDIVDSVDENDRVPPPVLGSLQVSDSDLDMEKMGVKGLLNPLRAFGYNVETLEMLLIPMAKDASEALGSMGNDTPLAMMSDRPKLLFEYFKQMFAQVTNPPIDPIREAVVTSTECMVGPEGDLTETIEEQCHRLSLKSPLLTLEEMEAIKNMDHRGWRSKVIDITFGKEEGVDGLEKALDRICAEARLAILDGYRMLVLSDRATSPERVPVQSLLAVGAVHHHLVSTMERTQIGLVLESAEPREVHHFCTLVGFGVDAICPYLAIETIKRLNIDGKIPPKAGVALHTPEELIKKYFYASNSGMLKVLAKMGISTLASYKGAQIFEAVGLATEVVGRCFKGTPSRVEGATFEILARDTLKLHELAFPTRVAPPGSADANAVPNAGDYHWRKGGEVHLNDPVAIAKLQEAARTGSKDAYKEYSKTIHELNKKINLRGMLRFKVDSSNSIPLEEVEPAREIVKRFCTGAMSYGSISLEAHTTLAVAMNKLGGKSNTGEGGEAPERLEPLPDGSQNPQRSAIKQVASGRFGVTSYYLTNADELQIKMAQGAKPGEGGELPGHKVIGNIARTRNSTPGVGLISPPPHHDIYSIEDLAQLIFDLKNSNPSARISVKLVSEAGVGVVASGVVKGHADHVLISGHDGGTGASRWTGIKNAGLPWELGLAETHQTLVANDLRGRTVLQTDGQLKTGRDVAIAALLGAEEFGFSTAPLITLGCIMMRKCHLNTCPVGIATQDPLLRKKFAGDPEYVVNFLFMVAEECREIMASLGYRKMDDMIGHAEHLDVDPDVMNSNDKLENVDLSLLLRPAATLRPDAAQRCVQMQDHGIDDVLDRRLIKLSEPALTRRLPVYVDLPVCNTDRAVGTMLSHELTKRYRAEGLSEGSIHVKLTGSAGQSLGAFLAPGITLELEGDSNDYVGKGLSGGKIVVYPPRASTFDPKENIIIGNVALYGATAGEAYFNGMAAERFCVRNSGAKAVVEGLGDHGCEYMTGGTVVVLGKTGKNFAAGMSGGVAYVLDVDGGFTSRCNTGLVDLDKIGKDDVEDVMLLQTMIQQHLRRTQSQLAREVLANFESLLPKFIKVFPRDYKRVLGELKAKEEAAKLEEQALASKNAFEELKKLAGNVTNGNVTNGSAATITEASTYGSAPVTKEEKPTGETRPTKIPDAVKMRGFVNYEREPLPYRQPEERLNDWKEVTVQSRQDALLKTQSARCMDCGTPFCQQEKSGCPLGNKIPEWNELVHQGRWREALDRLLETNNFPEFTGRVCPAPCEGSCVLGIIEKPVTIKTMECTIIDKAFEEGWMVPRPPLKRTGKRVAIVGSGPAGLAAADQLNKAGHFVTVYERADRVGGLMMYGVPNMKTDKEEVVQRRVDLMAKEGVEFVVNAHVGVDSRYTVESLRASNDALILACGATKPRDLVVPGRELKGVHFAMEFLTANTKSLLDSGLKDGKYISAKDKRVVVIGGGDTGTDCIGTSVRHGCKQVINLELLPQPPYTRAPGNPWPEWPRVFRVDYGHEEAKTRFGKDPRTYEVLTKRFVGNDAGEVTGLELVHVRWDKDATGRFQMQEIPGSEEVIEADLVLLAMGFLGPEQNIAEKLGLQTDARSNFKADYGRFATNLQGVFAAGDCRRGQSLVVWAIAEGRQAAAQVDEYVMKDFNEADLVVDERFMSISGIGNVTGNGYSNGNGSSNGAGKDLQFPEGVKDAAEEARQACVAVVGDAEDYNKLKECIDLSVEAAALSDTNTVTEQSY

>Rn_15020.1_2|Ricciocarpos_natans/1-2696

MDGLVIQTPKQQIRGTFNRVRTTHYLSQDSSILSGQKKKRKTRSQVETRTTVSGLHSHGTGLCLITELGVFGDRSTLPVSTVAEMGVEGKLTGGSTVGRTFLLGEGESHNAAGTLRGRLLAALRTHFEHTPLPGDQTPVNGKDIVGRGFQCYYRHERTATTVVCDRASLVCQAYSSAPVGLFSREATPALEEKRRGEAIRQQRRRIGRFACRVVRRTRERREPTVDRRWEVRSLACCCSTEAHEKREREISTRKRGNSRRRKGMASSACSFTAGSRLAEQTGAATSKISSSSSRSTLNNVDIHVKRASSFPVTVNAGNAITLGVVPSTIAPQSIEQQTSSLRSAASWACSSRFSGLRKLPARKAKKIQQWLQYTEGPGRDALLRVVQMALSQVPEGPQGLYDPSMDKDSCGVGFVAELSAEPTRNTVSCLRHMPQLLMSDARPVSDEVLLVCFHPLREISVTVEVIVSRAHVYACGMTENFVDGILPVELQCRRRLVSGSDAENTTVLRHWLESVFDFHIKDSGGDGLGSDQSRVTVTDALEMLVRMAHRGACGCEENTGDGAGIMVALPHNFFAAIAEKEIGCKLPPPGQYAVGMFYLPTDKGRRVESKAVFKRVAESLGHKLLGWRKVKTDNSGLGKSALETEPVVEQVFLTPSPKSKHGLDEQMYVLRKLAMIQIRVALNMPHGSGKDFYICSLSPQTIVYKGQLTPEQVFQYYRADLGDERFTSYMALVHSRFSTNTFPSWDRAQPMRVLGHNGEINTLRGNVNWMHAREGLLKCKNLGLSEAELEKLLPIVDASSSDSGAFDGVLEFLSRSGRSLPEAVMMMIPEAWQNDQNMDPQRRALYEYFSAIMEPWDGPALIAFTDGRYLGATLDRNGLRPGRYYVTHSGRVVMASEVGVVDIKPEDVSKKGRLNPGMMLLVDFQNHVVVNDDDLKRQVAASQPFGEWLQRQKLTLKDIVDSVDENDRVPPPVLGSLQVSDSDLDMEKMGVKGLLNPLRAFGYNVETLEMLLIPMAKDASEALGSMGNDTPLAMMSDRPKLLFEYFKQMFAQVTNPPIDPIREAVVTSTECMVGPEGDLTETIEEQCHRLSLKSPLLTLEEMEAIKNMDHRGWRSKVIDITFGKEEGVDGLEKALDRICAEARLAILDGYRMLVLSDRATSPERVPVQSLLAVGAVHHHLVSTMERTQIGLVLESAEPREVHHFCTLVGFGVDAICPYLAIETIKRLNIDGKIPPKAGVALHTPEELIKKYFYASNSGMLKVLAKMGISTLASYKGAQIFEAVGLATEVVGRCFKGTPSRVEGATFEILARDTLKLHELAFPTRVAPPGSADANAVPNAGDYHWRKGGEVHLNDPVAIAKLQEAARTGSKDAYKEYSKTIHELNKKINLRGMLRFKVDSSNSIPLEEVEPAREIVKRFCTGAMSYGSISLEAHTTLAVAMNKLGGKSNTGEGGEAPERLEPLPDGSQNPQRSAIKQVASGRFGVTSYYLTNADELQIKMAQGAKPGEGGELPGHKVIGNIARTRNSTPGVGLISPPPHHDIYSIEDLAQLIFDLKNSNPSARISVKLVSEAGVGVVASGVVKGHADHVLISGHDGGTGASRWTGIKNAGLPWELGLAETHQTLVANDLRGRTVLQTDGQLKTGRDVAIAALLGAEEFGFSTAPLITLGCIMMRKCHLNTCPVGIATQDPLLRKKFAGDPEYVVNFLFMVAEECREIMASLGYRKMDDMIGHAEHLDVDPDVMNSNDKLENVDLSLLLRPAATLRPDAAQRCVQMQDHGIDDVLDRRLIKLSEPALTRRLPVYVDLPVCNTDRAVGTMLSHELTKRYRAEGLSEGSIHVKLTGSAGQSLGAFLAPGITLELEGDSNDYVGKGLSGGKIVVYPPRASTFDPKENIIIGNVALYGATAGEAYFNGMAAERFCVRNSGAKAVVEGLGDHGCEYMTGGTVVVLGKTGKNFAAGMSGGVAYVLDVDGGFTSRCNTGLVDLDKIGKDDVEDVMLLQTMIQQHLRRTQSQLAREVLANFESLLPKFIKVFPRDYKRVLGELKAKEEAAKLEEQALASKNAFEELKKLAGNVTNGNVTNGSAATITEASTYGSAPVTKEEKPTGETRPTKIPDAVKMRGFVNYEREPLPYRQPEERLNDWKEVTVQSRQDALLKTQSARCMDCGTPFCQQEKSGCPLGNKIPEWNELVHQGRWREALDRLLETNNFPEFTGRVCPAPCEGSCVLGIIEKPVTIKTMECTIIDKAFEEGWMVPRPPLKRTGKRVAIVGSGPAGLAAADQLNKAGHFVTVYERADRVGGLMMYGVPNMKTDKEEVVQRRVDLMAKEGVEFVVNAHVGVDSRYTVESLRASNDALILACGATKPRDLVVPGRELKGVHFAMEFLTANTKSLLDSGLKDGKYISAKDKRVVVIGGGDTGTDCIGTSVRHGCKQVINLELLPQPPYTRAPGNPWPEWPRVFRVDYGHEEAKTRFGKDPRTYEVLTKRFVGNDAGEVTGLELVHVRWDKDATGRFQMQEIPGSEEVIEADLVLLAMGFLGPEQNIAEKLGLQTDARSNFKADYGRFATNLQGVFAAGDCRRGQSLVVWAIAEGRQAAAQVDEYVMKDFNEADLVVDERFMSISGIGNVTGNGYSNGNGSSNGAGKDLQFPEGVKDAAEEARQACVAVVGDAEDYNKLKECIDLSVEAAALSDTNTVTEQSY

>Rn_15020.1_3|Ricciocarpos_natans/1-2696

MDGLVIQTPKQQIRGTFNRVRTTHYLSQDSSILSGQKKKRKTRSQVETRTTVSGLHSHGTGLCLITELGVFGDRSTLPVSTVAEMGVEGKLTGGSTVGRTFLLGEGESHNAAGTLRGRLLAALRTHFEHTPLPGDQTPVNGKDIVGRGFQCYYRHERTATTVVCDRASLVCQAYSSAPVGLFSREATPALEEKRRGEAIRQQRRRIGRFACRVVRRTRERREPTVDRRWEVRSLACCCSTEAHEKREREISTRKRGNSRRRKGMASSACSFTAGSRLAEQTGAATSKISSSSSRSTLNNVDIHVKRASSFPVTVNAGNAITLGVVPSTIAPQSIEQQTSSLRSAASWACSSRFSGLRKLPARKAKKIQQWLQYTEGPGRDALLRVVQMALSQVPEGPQGLYDPSMDKDSCGVGFVAELSAEPTRNTVSCLRHMPQLLMSDARPVSDEVLLVCFHPLREISVTVEVIVSRAHVYACGMTENFVDGILPVELQCRRRLVSGSDAENTTVLRHWLESVFDFHIKDSGGDGLGSDQSRVTVTDALEMLVRMAHRGACGCEENTGDGAGIMVALPHNFFAAIAEKEIGCKLPPPGQYAVGMFYLPTDKGRRVESKAVFKRVAESLGHKLLGWRKVKTDNSGLGKSALETEPVVEQVFLTPSPKSKHGLDEQMYVLRKLAMIQIRVALNMPHGSGKDFYICSLSPQTIVYKGQLTPEQVFQYYRADLGDERFTSYMALVHSRFSTNTFPSWDRAQPMRVLGHNGEINTLRGNVNWMHAREGLLKCKNLGLSEAELEKLLPIVDASSSDSGAFDGVLEFLSRSGRSLPEAVMMMIPEAWQNDQNMDPQRRALYEYFSAIMEPWDGPALIAFTDGRYLGATLDRNGLRPGRYYVTHSGRVVMASEVGVVDIKPEDVSKKGRLNPGMMLLVDFQNHVVVNDDDLKRQVAASQPFGEWLQRQKLTLKDIVDSVDENDRVPPPVLGSLQVSDSDLDMEKMGVKGLLNPLRAFGYNVETLEMLLIPMAKDASEALGSMGNDTPLAMMSDRPKLLFEYFKQMFAQVTNPPIDPIREAVVTSTECMVGPEGDLTETIEEQCHRLSLKSPLLTLEEMEAIKNMDHRGWRSKVIDITFGKEEGVDGLEKALDRICAEARLAILDGYRMLVLSDRATSPERVPVQSLLAVGAVHHHLVSTMERTQIGLVLESAEPREVHHFCTLVGFGVDAICPYLAIETIKRLNIDGKIPPKAGVALHTPEELIKKYFYASNSGMLKVLAKMGISTLASYKGAQIFEAVGLATEVVGRCFKGTPSRVEGATFEILARDTLKLHELAFPTRVAPPGSADANAVPNAGDYHWRKGGEVHLNDPVAIAKLQEAARTGSKDAYKEYSKTIHELNKKINLRGMLRFKVDSSNSIPLEEVEPAREIVKRFCTGAMSYGSISLEAHTTLAVAMNKLGGKSNTGEGGEAPERLEPLPDGSQNPQRSAIKQVASGRFGVTSYYLTNADELQIKMAQGAKPGEGGELPGHKVIGNIARTRNSTPGVGLISPPPHHDIYSIEDLAQLIFDLKNSNPSARISVKLVSEAGVGVVASGVVKGHADHVLISGHDGGTGASRWTGIKNAGLPWELGLAETHQTLVANDLRGRTVLQTDGQLKTGRDVAIAALLGAEEFGFSTAPLITLGCIMMRKCHLNTCPVGIATQDPLLRKKFAGDPEYVVNFLFMVAEECREIMASLGYRKMDDMIGHAEHLDVDPDVMNSNDKLENVDLSLLLRPAATLRPDAAQRCVQMQDHGIDDVLDRRLIKLSEPALTRRLPVYVDLPVCNTDRAVGTMLSHELTKRYRAEGLSEGSIHVKLTGSAGQSLGAFLAPGITLELEGDSNDYVGKGLSGGKIVVYPPRASTFDPKENIIIGNVALYGATAGEAYFNGMAAERFCVRNSGAKAVVEGLGDHGCEYMTGGTVVVLGKTGKNFAAGMSGGVAYVLDVDGGFTSRCNTGLVDLDKIGKDDVEDVMLLQTMIQQHLRRTQSQLAREVLANFESLLPKFIKVFPRDYKRVLGELKAKEEAAKLEEQALASKNAFEELKKLAGNVTNGNVTNGSAATITEASTYGSAPVTKEEKPTGETRPTKIPDAVKMRGFVNYEREPLPYRQPEERLNDWKEVTVQSRQDALLKTQSARCMDCGTPFCQQEKSGCPLGNKIPEWNELVHQGRWREALDRLLETNNFPEFTGRVCPAPCEGSCVLGIIEKPVTIKTMECTIIDKAFEEGWMVPRPPLKRTGKRVAIVGSGPAGLAAADQLNKAGHFVTVYERADRVGGLMMYGVPNMKTDKEEVVQRRVDLMAKEGVEFVVNAHVGVDSRYTVESLRASNDALILACGATKPRDLVVPGRELKGVHFAMEFLTANTKSLLDSGLKDGKYISAKDKRVVVIGGGDTGTDCIGTSVRHGCKQVINLELLPQPPYTRAPGNPWPEWPRVFRVDYGHEEAKTRFGKDPRTYEVLTKRFVGNDAGEVTGLELVHVRWDKDATGRFQMQEIPGSEEVIEADLVLLAMGFLGPEQNIAEKLGLQTDARSNFKADYGRFATNLQGVFAAGDCRRGQSLVVWAIAEGRQAAAQVDEYVMKDFNEADLVVDERFMSISGIGNVTGNGYSNGNGSSNGAGKDLQFPEGVKDAAEEARQACVAVVGDAEDYNKLKECIDLSVEAAALSDTNTVTEQSY

>Pp6c16_2470|Ppatens_v6.1/1-2200

MTTCVPALVQFNSRFTLVHANRSRVPQTCSHKLQLLEVTRFTGLRREVPPRLKPEERTRQWKGDWWEFTERFCDKGSGLKPVLVALSGFPDGNLGLYDPALDMDSCGVGFVAELSAQPNRKIVTDAIGMLLRMAHRGACGCEVNTGDGAGILVATPHDYFSQVLMEELDLSLPPLGQYAVGMVFLPQNDQRREESKNVFKKVAESLGHRILGWRVVKTDNTGLGKGAIETEPIVAQVFLSPSSHSTVDIERQMYVLRKTSMIAIRAALNLQYGAAKDFYICSLSSRTIVYKGQLKADQLEKYFHNDLGDKRFTSYMGLVHSRFSTNTFPSWDRAQPMRLLGHNGEINTLRGNVNWMHAREGLLQCDALKLSKDELQKLLPIVDAGSSDSGAFDGVLEILMRSGRDLPEAVMMMIPEAWQNDNNMDPERRALYEYFSCILEPWDGPALISFTDGRFLGATLDRNGLRPSRYYITHDQRVIMASEVGVVDVDPRNVFKKGRLHPGMMLLVDFDKHKVVDDTELKKKYASRYPYAEWLRRQKIPLQEIVDSVSKGERTPPPIIGAAEVGTSDETMDHMGVKGLIAPLRAFGYTTESLELLLLPMAGDGIEALGSMGNDTPLAVMSDRPKLSFEYFKQMFAQVTNPPIDPIREAVITSTECMVGPEGDLTEVAESQCNRLSLKSPLLTLEEMEAVKKTQHRGWRTKIIDITFPRSGGSEGLEKALDRICAEARLAISDGCTVLVLSDRATSAQRVPVSSLVATGAVHHDLVASLERTRVALMLETGEPREVHHFCTLIGFGADAICPYLAIESIYRLQVDGKIAPKKEGTLYNREEIIAKYFKGSNAGMLKVLAKMGISTLASYKGAQIFEAIGLSTAVVQRCFKGTSSRIEGATFEMLAHDMLRMHKLAFPARLLPDGSAEALALPNPGDYHWRLKGEVHLNDPDAIAKLQEAARENSPAAYKEYSKITQNMNRKCNLRGLLRFKDLPESEQIPLEEVEPASDIVKRFVTGAMSYGSISLETHTTLAVAMNRLGGKSNTGEGGEQPERLEPLEDGSTNPQRSAIKQVASGRFGVTSYYLTNADEIQIKMAQGAKPGEGGELPGHKVIGDIAITRNSTEGVGLISPPPHHDIYSIEDLAQLIYDLKNANPSARISVKLVSEAGVGVVASGVVKGHADHILISGHDGGTGASRWTSIKHAGLPWELGLAETQQTLVANGLRGRTILQTDGQLKNGHDVIIAALLGAEEFGFSTAPLITLGCIMMRKCHKNTCPVGIATQDPVLRTKFSGLPDHVTNFFFMVAEEAREIMASLGVRRMDDLIGRADLLEMDKTAMTENEKVSNIDLSLLLHPASKIKPDAAQRCVESQDHGLENALDRKLIALSQPALNRNMPVYVETSVFNVNRAVGTMLSHEVTKRYHRTGLPTDTIHVKLNGSAGQSLGAFLCSGITLELEGDSNDYVGKGLCGGKIIVYPHKNSSFDPKENIVIGNVALYGATGGEAYFNGMAAERFAVRNSGANAVVEGVGDHGCEYMTGGTVVVLGETGRNFAAGMSGGVAYVLDRERLFLRKCNTALVDLDSVEEEDDIILLRSLIQQHQRATHSRLARYVLNHFEALLPKFIKVYPRDFKRVVMEKKTKEAAERSARTGEDLSNKDTFAALKDLSTRSEATSPSANGASKVERPTIVYEPTKHGGFVRYGRESLPLRPIGERVQDWDEVIDHEINEPLLKTQSARCMDCGTPFCHQDHSGCPLGNKIPEWNELVYQGRWREALDRLLETNNFPEFTGRVCPAPCEGACVLGIIENPVTIKTMECTIIDKGWEMGWMVPRSPMKRTGKKIAIVGSGPAGLAAADQLNKAGHTVTVYERSDRVGGIMMYGVPNMKTDKVDIVQRRVDLMAAEGVQFAVNAHVGVDPMYSVESLRQDNNALLLACGATKSRDLPIPGRDLKGIHFAMEFLHANTKSLLDSGLQDGKYISAKGKKVVVIGGGDTGTDCIGTSVRHGCSNLVNLELLPQPTSTRAPGNPWPQWPRVFRIDYGHEEAHAKFGKDPRTYEVLTKKFIGSKKGELIGLEVVHVKWEKDSDKFQMQEIPGSEEVIETDLAFLALGFQGPEQNIAEKLGLERDARSNFKADYGRFATNLQGVFAAGDCRRGQSLVVWAIAEGRQVAEQMDNYLMKDEAVEKGREQLHAANGNRESKKGQRYPEGLCKA

>Pp6c5_10390|Ppatens_v6.1/1-2251

MAACQQTLSQFGSQYSLTYASLPRAPVSTCTHKAHWLKALRFTGLRRASLNLSPRDRLHQRQQSCLHFSDVSPVKGLGLKPVLAGLSGVPEGNLGLYDPSFDKDSCGVGFVAELSAKPNRKIVADAIEMLVRMKHRGACGCEVNTGDGAGILVSLPHDYFAKVLREDLDLDLPPIGKYAVGMVFLPTIEHRREESKKVFNKVAESLGHKILGWRPVKTNNSGLGKGAIDTEPVVAQVFLTPSSRSTNDFEQQMYILRKTSMIAIRAALNLAYGASKDFYICSLSSRTIVYKGQLKPDQLKNYYYNDLGDERFTSYMGLVHSRFSTNTFPSWDRAQPMRVLGHNGEINTLRGNVNWMHAREGLLQCNALNLTKDELQKLLPIVDAGSSDSGAYDGVLELLMRSGRDLPEAVMMMIPEAWQNDKLMDPERRAMYEYFSCLLEPWDGPALISFTDGRYLGAKLDRNGLRPSRYYITHDQRVIMASEVGVVDVDSKNVFKKGRLFPGMMLLVDFEKHKVVDDEELKKKYQSRYPYAEWLQRQKLTLEEIVESVPEEKRVTPPIIGALEVKTSDETMDHMGTRGIIAPLRAFGYTSESLELLLLPMADDGTEALGSMGNDTPLAVMSDRPRLSFEYFKQMFAQVTNPPIDPIREAVVTSTECMVGPEGDLTEVAESQCHRLSLKSPLLTLKEMEALKKTNYRGWRSKVIDITFPKSDGIDGLEMALDRICAEARLAVSDGYTVLALSDRATSAQRVPVSSLIAVGAVHHHLVASLERTRVALVIETGEPREVHHFCTLVGFGADAICPYLAIESIYRLQVDGKVAPKKDGTLWSREEIVAKYFKASNSGMLKVLAKMGISTLASYKGAQIFEALGLSTEVVQRCFKGTPCRIEGATFEMLAQDMLRMHELAFPSRHLPDGCADAVALPNPGDYHWRLRGEIHMNDPDAIAKLQEAARTGSTAAYREYSQIIQKLNRKCNLRGLLKFKDLPESEQIPLDEVEPASEIVKRFVTGAMSYGSISLETHTTLAVAMNRLGGKSNTGEGGEQPERMEPLEDGSMNPMRSAIKQVASGRFGVTSYYLTNSDEIQIKMAQGAKPGEGGELPGHKVIGDIAITRNSTEGVGLISPPPHHDIYSIEDLAQLIYDLKNSNPGARISVKLVSESGVGVVASGVVKGHADHVLIAGHDGGTGASRWTGIKNAGLPWELGLAETHQTLVANGLRGRTTVQTDGQLKNGRDIMVAALLGAEEFGFSTAPLITLGCIMMRKCHKNTCPVGIATQDPVLREKFAGQPEHVINFFFMVAEEAREIMASLGIRKMDDLIGRADLLEMDREAMGENEKVGNIDLSLLLRPAAEIRPGAAQRCVEAQDHNLEEALDQKLIALAQPALTKKVPVYVETPVVNVNRAVGTMLSHEVTKRFNRTGLPTDTIHVKLNGSAGQSLGAFLCSGITLELEGDSNDYVGKGLCGGKIIVYPPADSTFDPKENIVIGNVALYGATSGEAYFNGMAAERFAVRNSGANAVVEGVGDHGCEYMTGGKVVVLGETGKNFAAGMSGGIAYVLDKEGLFPKKCNTELVDLDPVEEEDDIMTLRSLIQQHQRATNSRLARYVLNHFEALLPNFIKVFPRDYKRVVNEKKAKEAAEIAAKEEEIELSKKDAFAELQKLAAQSAPAIAGINAASNGASKVERPTKISDPQKHGGFVKYERESLPLRPTEERLQDWDEVVEHGINESLLKTQSARCMDCGTPFCHQDHSGCPLGNKIPEWNELVYQGRWREALDRLLETNNFPEFTGRVCPAPCEGACVLGIIENPVTIKTMECTIIDKGWEMGWMVPRPPEERTGKKVAVVGSGPAGLAAADQLNKAGHTVTVYERSDRVGGLMMYGVPNMKTDKIDVVQRRVDLMAAEGIKFVTNAHVGMDPLYSIDTLRADNDALLLACGATKPRDLPIPGRDLSGVHFAMEFLHANTKSLLDSGLQDGNYISAKGKKVVVIGGGDTGTDCIGTSVRHGCSAMTNLELLPQPPPTRAPGNPWPQWPRIFRVDYGHEESAAKFGTDPRTYEVLTKRFIGNDEGKLTGLEIIQVKWEKDSTGKFQMQEIPGSEKIIEADLVFLALGFLGPEQNIAEKLGLECDPRSNFKADYGQFTTNLEGVFAAGDCRRGQSLVVWAIAEGRQAADKVDNYLMKDVPVEENAKSHVEGNGYGVGKPKERRYPEGLDKAKEEAVEACIAVHGSEDSDALKKCVELSVETAALGDNSVVSEQSY

>Pp6c14_4690|Ppatens_v6.1/1-2213

MEALLRAPLTLHSQPQAAVNVHGGNSSKSRKLLKQGNCAQAVARDVAGTGLQTDSSFGSRQLQAGCSFSGLRKTGGRSLAESFKSDKSRLVKRFWGQSSRQRHQKFLQLSVRAGLSSVPESNLGLYDPSFDKDGCGVGFIAKLSQESSRDIVKDALEMLERMAHRGACGCEENTGDGAGILVGIPHSYFSQVVPEEIGCELPPLGDYAVGMMFLPTSEIRREHAKKVFTEVAEALGHTVLGWRRVQTDNSDLGKSAIQTEPLIEQVFLTASTLSKVDFETQMYILRKTSMLAIRAVLNLKRGAAKDFYICSLSSKTVVYKGQLKPNQLTKYYYADLGDERFTSFMAIVHSRFSTNTFPSWDRAQPMRMLGHNGEINTLRGNVNWMHAREGLFKCKNLGLSPEELKKLLPIVDAGSSDSGVFDGVLELLVRAGRSLPEAVMMMIPEAWQNDPNMDPERRALYEYFSCIIEPWDGPALVTFTDGKYLGATLDRNGLRPGRFYVTHDGRVIMASEVGVVDVEPENVAQKGRLHPGTMLLVDFENHKVVDGDDLKKQQSSRYPYAEWLERQKFTLEDVVNSVPEKLRAPPAIIGDKNMASLNFSADNMGVKGLLTPLKAFGYTMENLEMLLLPMAKDGSEALGSMGNDAPLAMMSNRPKLLFEYFKQMFAQVTNPPIDPIREAVVTSTRCMVGPEHDLTEKVEEQCNRLSLSGPLLTVEEMESVKKMEYRGWKTMVVDITFDKSEGPAGLENALTRICAEARNAVAEGYSMLVLSDRATSAQRVPVSSLLATGAVHHHLVQSMERTRIGLVVESAEPREVHHFCTLVGFGADAICPYLAIETIFRVQVDGKIPMKVSKDDLVKKYFYASNSGMLKVLAKMGISTLASYKGAQIFEALGLSSEVVSRCFKGTPSRVEGATFEMLAKDSLRLHEMAFPAREAPEGSADANALPNPGDYSWRKGGEVHLNDPMAMAKLQEAARTNSVDAYKEFSKLTHELNKKINVRGMLKFKEQPESARIPLEEVEPAAEIVKRFCTGAMSYGSISLEAHSTLAIAMNKLGGKSNTGEGGENPKRLEPLQDGSQNPQRSAIKQIASGRFGVTSYYLTNADELQIKMAQGAKPGEGGELPGHKVIGDIAITRNSTPGVGLISPPPHHDIYSIEDLSQLIHDLKNANPDARVSVKLVSEAGVGVVASGVVKGHADHVLISGHDGGTGASRWTGIKNAGLPWELGLAETHQTLVANDLRGRTILQTDGQLKTGRDIMVAALLGAEEFGFATAPLITMGCIMMRKCHKNTCPVGIATQDPVLRAKFAGQPEHVINYFFMVAEEAREYMANLGIRKMDDLVGRADLLEVDEEVIAQNEKLTNIDLSLLLRPAADIRPGAAQRCVMKQDHELELALDQKLINLSRAALESKLPVYIETPVVNVNRAVGTMLSHEVTKRYKLEGLPIDTIYVKLNGSAGQSLGAFMCKGITMELEGDSNDYVGKGLCGGKIIVYPPRSSTFDPKENILIGNVALYGATSGEAYFNGMAAERFAVRNSGAKAVVEGVGDHGCEYMTGGTVVVLGKTGKNFAAGMSGGVAYVLDKEGTFKSTCNMGLVDLDPVVENEDVMTLKALIQQHQRHTKSQLAGEILANFDALLPSFIKVFPRDYKRVLQEIKAKEAAEQLKETPAVTVEVVTTVANGAPSGKASVTEEAAPSTIRPTQVLSPVKHGGFVKYERKPLPYRPADERMKDWGEVLASDNQDGLVKTQAARCMDCGTPFCNQDKTGCPLGNKIPEWNELVYQNRWREALDRLLETNNFPEFTGRVCPAPCEGACVLGIIENPVSIKTMECTIIDKGFENGWMVPRPPLKRTGKKVAIVGSGPAGLAAADELNKKGHSVTVFERADRIGGLMMYGVPNMKTDKVQVVQRRVDLMEAEGVQFVTNAHVGVDPVYSVEKLRSDYDALLLTTGATKPRDLPVEGRDLAGVHFAMEFLHKNTKSLLDSNLEDGQYISAKGKKVVVIGGGDTGTDCIGTSVRHGCTRVVNLELLPQPPQTRAANNPWPQWPRVFRVDYGHEEAATKFGKDPRSYEVLTKRFIGNDKGEVTGMETVRVKWDKSADGRFQMSEIPGSNEIIETDLVLLAMGFLGPEQLISEKLGLETDARSNFKADYGKHATNLQGVFAAGDCRRGQSLVVWAIAEGREAAVQVDNYLMVEESSEEQLPSGEEIREFTTTTSVQ

>Pp6c10_4470|Ppatens_v6.1/1-2210

MEALHFTPLTLHRQPQAALSIHDRGISTKSHESSDQGKRALGVVRGGVATGSQADSSFGSRQLQAGCNFSGLRKTREFVECSKSRKCRSVNRYWWESLRQRQSKFYFLPVRAGLSSAPVTNQGLYDPSFDKDGCGVGFIAKLSQEPSRDIVKDALEMLERMAHRGACGCEENTGDGAGILVGIPHTYFSQVVPEDLGCDLPPPGDYAVGMMFLPTSEIRREHAKKVFTEVAEALGHTVLGWRRIKTDNSDLGKSAIQTEPVIEQVFLTSSTLSKIDFESQMYILRKTSMLAIRAVLNLKRGAAKDFYICSLSSKTVVYKGQLKPNQLTNYYYADVGDERFTSFMSIVHSRFSTNTFPSWDRAQPMRMLGHNGEINTLRGNVNWMHAREGLFKCKNLGLSPEELKKLLPIVDAGSSDSGVFDGVLELLVRAGRSLPEAVMMMIPEAWQNDPNMDPERRNLYEYFSCIIEPWDGPALVTFTDGKYLGATLDRNGLRPGRFYVTHDGRVIMASEVGVVDVEPENVAQKGRLHPGTMLLVDFENHKVVDGDELKKQQSSRYPYGEWLERQSFTLQDVVNSVSENLRVPPAILGDKNVASPNFSASNMGVKGLLTPLKAFGYTMENLEMLLLPMAKDGSEALGSMGNDAPLAMMSNRPKLVFEYFKQMFAQVTNPPIDPIREAIVTSTRCMVGPEHDLTETVEEQCNRLSLSGPLLDLGEMEAVKKMEYRGWKTMVVDTTFDKSEGPAGLESALTRICAEARNAVAEGYSMLVLSDRATSAQRVPVSSLLATGAVHHHLVQSMERTRIGLVVESAEPREVHHFCTLVGFGADAICPYLAIETISRLQVDGKIPMEWSREELVKKYFYASNSGMLKVLAKMGISTLASYKGAQIFEALGLSSEVVSRCFKGTPSRVEGATFEMLAKDCLRLHELAFPARESPEGSADANALPNPGDYNWRKDGEVHLNDPMAMAKLQEAARTNSVEAYNEFSKLTHELNKKVNVRGMLKFKEQPESTRIPLDNVESAAEIVKRFCTGAMSYGSISLEAHSTLAIAMNKLGGKSNTGEGGENPRRLEPLSDGSQNPQRSAIKQIASGRFGVTSYYLTNADELQIKMAQGAKPGEGGELPGHKVIGDIAVTRNSTPGVGLISPPPHHDIYSIEDLSQLIHDLKNANPAARVSVKLVSEAGVGVVASGVVKGHADHVLISGHDGGTGASRWTGIKNAGLPWELGLAETHQTLVANDLRGRTILQTDGQLKTGRDIMIAALLGAEEFGFATAPLITMGCIMMRKCHKNTCPVGIATQDPVLRAKFAGQPEHVINYFFMVAEEAREIMANLGISKMDDLVGRADLLQVDEEVMAENEKLANIDLSLLLRPAAEIRPGAAQRCVQKQDHELELALDQKLISLSHAALESKVPVYIESPVVNVNRAVGTMLSHEVTKQYKLEELPTDTIYVKLNGSAGQSLGAFTCKGVTLELEGDSNDYVGKGLCGGKIIVYPPRSSTFDPKENILIGNVALYGATSGEAYFNGMAAERFAVRNSGAKTVVEGVGDHGCEYMTGGVVVVLGETGKNFAAGMSGGVAYVLDKEGTFSSTCNTGLVDLDPVEEEEDVMTLRAMIQQHQRHTKSQLAGEILANFDSLLPSFIKVFPRDYKRVLKEIKAKEEAEKMKVAAAAVADAGLAASVANSAPKVEEKAAPSTIRPTEVSNPIKHGGFVKYDRKPLPYRPAEERMKDWGEVLASDNQDGLVKTQSARCMDCGTPFCNQDKTGCPLGNKVPEWNELVYQNRWREALDRLLETNNFPEFTGRVCPAPCEGACVLGIIENPVSIKTMECTIIDKGFENGWMVPRPPLKRTGKKVAIVGSGPAGLAAADELNKKGHSVTVFERADRIGGLMMYGVPNMKTDKENVVQRRVNLMEAEGVQFVTNAHVGVDPVYSVENLRSDYDALLLATGATKPRDLPVEGRDLAGVHFAMEFLHKNTKSLLDSNLEDGQYISAKGKKVVVIGGGDTGTDCIGTSVRHSCASVVNLELLPQPPQTRAANNPWPQWPRIFRVDYGHEEAATKFGKDPRSYEVLTKRFIGNEKGEVTGMELVRVEWSKGADGRFQMTEVVGSTQIIEADLVLLAMGFLGPEQNIAEKLGLQTDARSNFQADFGKHATNSQGVFAAGDCRRGQSLVVWAIAEGRQAAAQVDNYLMVKEVVLEQLPSEEHIREFIATSSVQ

>Dicom.08G013200|Dcomplanatum_v3.1/1-2257

MRSLLNSCVMQNSGRKGTTFDSTYVEATASAPSAFRLRHRTKLAPEYVNVQPQKVCNPQVNAYLSNSFGNHDDWASKFSGLRGQVLPLRSKSDLKWSKKRIQWWLWEGKAAVRGGTRMSFSQIPEFPLGLYDPALDKDSCGVGFLADLSAKPTRETVVDGLEMLVRMSHRGACGCETNTGDGAGILVAIPHDYFSLVCTEEEGFELPPVNEYGVGMFFLPSLDDRRRDSKNVFNEVAQSLGHTVLGWRRVRTDNSDLGAAALSTEPVIEQVFVTASTISNSDLESQMYVLRKTSVAAIKDALDLERENSRDFYICSLSSRTIVYKGQLKPDQLKKYYFADLGHEKFTSYMALIHSRFSTNTFPSWDRAQPMRLLGHNGEINTLQGNVNWMRAREGLLKCKAIGITSEELQKLLPIVDPRSSDSGAFDGVLEFLIRCGRSLPEAVMMLIPEAWQNDSTMDPGRKALYQYFSSIMEPWDGPALISFTDGRYLGATLDRNGLRPGRYYITHSGRVIMASEVGVVDVLPEDVCHKGRLSPGKMLLVDFAKHVVVDDDELKSEYSARQPYAQWLQRQSITLKDILNSLDNEERVPPPTRTVNGSAEVTKHVGIRGMLPFLKVFGYTVETLEMLLLPMAKDGSEALGSMGNDTPLAVISGHPKLIFDYFKQMFAQVTNPPIDPIREANVTSLECMVGPEGDLTESVETQCHRLSLKGPLLSLEEMEAIKKMQYRGWRSKLIDITFPVEQGISGLEKTLDKICLEATIAIVEGYKLLVLSDRATSSERVPVSSLLAVGAVHHHLISTLERTRIGLLVESGDAREVHHFCTILGFGADAICPYVAIDSIWRMRLDGKVPPKMDRTLYSRDELVKQYFKASNYGMLKVMAKMGISTLASYKGAQIFEALGLSSEVVRKCFKGTPSRVEGATFEMLASDALQLHEIAFPSRLLPDGSADANALPNPGDYHWRKDGEVHLNDPLAMAKLQEAARTNSTQAYKDYSKLINELNKKCNLRGMLKFKFRQGKSIPLEDVEPASQIVKRFCTGAMSYGSISLEAHSTLAVAMNQIGGKSNTGEGGENSSRLEPLKNGLANPKRSAIKQVASGRFGVTSYYLTNADELQIKMAQGAKPGEGGELPGYKVIGDIALTRMSTPGVGLISPPPHHDIYSIEDLAQLIFDLKNSNPVARISVKLVSEAGVGVIASGVVKGHADHVLISGHDGGTGASRWTGIKNAGLPWELGLAETHQTLVANNLRGRAILQTDGQLKTGRDVVIAALLGAEEFGFSTAPLITLGCIMMRKCHKNTCPVGVATQDPILRAKFAGKPEHVINFLFMVAEEARELMAEMGFRNIDEMVGRADMLEVDEQVISSNEKLKNIDLSLLLQPAAKLRPGAAQTCIEKQDHGLELALDQILIQSALPALEKSLPVFLKLPIQNVNRAVGTMLSHEVTKRYNARGLPNDTIHFKFIGSAGQSFGAFLCAGITLELEGDSNDYVGKGLCGGKLIVYPNRLSKFDPENNIVIGNVALYGATSGEAYFNGMAAERFCVRNSGAKTVVEGVGDHGCEYMTGGIVVVLGKTGKNFAAGMSGGIAFVLDVDGSFKSRCNLGLVDLEEVEDQEDIVNLKSMIQQHHRNTKSKVAKEVLENFDSLLSKFVKVFPRDYKRILTELKDSGADGVQRQLSTRHPSTNLHGTKEHVISQNGASHLQDDKIPESAKRPTRVNDAQKNRGFITYERESLSYRPPGERVNDWKEVLSNGRESELLSTQSARCMDCGTPFCHQETTGCPLGNKIPEWNDLVHQGRWREALDRLLETNNFPEFTGRVCPAPCEGACVLGIIENPVSIKAIECSIIDKAFEEGWMVPRPPVIRTEKKVAIVGSGPAGLAAADQLNKAGHTVTVFERADRIGGLMMYGVPNMKTDKVEIVQRRVDLLVAEGVTFVTNANVGGDRLYSVERLRNEHDALLLACGATKPRDFSIPGRDLNGIHFAMDFLHANTKSLLDSNLQDGNFISAKDKRVVVIGGGDTGTDCIGTAVRHGCKNIVNLELLSRPPPTRDPSNPWPQWPKIFRVDYGHEEATEAFGKDPRSYEALTKRFIGDKNGVVTALEIKRVRWEKDAAGKFQLKEIEGSEEIIEADLVFLAMGFLGPEQTVVEALGLVTDQRSNIKAEFGRFSTNLEGVYAAGDCRRGQSLVVWAIAEGRQAAAEVDKFLMKNDSVANLSSQNQKDELLNGRDIARRNGNALARPGRHLNSDASTERAQEQESEVSVSGS

>Dicom.08G012100|Dcomplanatum_v3.1/1-2257

MRSLLNSCVMQNSGRKGTTFDSTYVEATASAPSAFRLRHRTKLAPEYVNVQPQKVCNPQVNAYLSNSFGNHDDWASKFSGLRGQVLPLRSKSDLKWSKKRIQWWLWEGKAAVRGGTRMSFSQIPEFPLGLYDPALDKDSCGVGFLADLSAKPTRETVVDGLEMLVRMSHRGACGCETNTGDGAGILVAIPHDYFSLVCTEEEGFELPPVNEYGVGMFFLPSLDDRRRDSKNVFNEVAQSLGHTVLGWRRVRTDNSDLGAAALSTEPVIEQVFVTASTISNSDLESQMYVLRKTSVAAIKDALDLERENSRDFYICSLSSRTIVYKGQLKPDQLKKYYFADLGHEKFTSYMALIHSRFSTNTFPSWDRAQPMRLLGHNGEINTLQGNVNWMRAREGLLKCKAIGITSEELQKLLPIVDPRSSDSGAFDGVLEFLIRCGRSLPEAVMMLIPEAWQNDSTMDPGRKALYQYFSSIMEPWDGPALISFTDGRYLGATLDRNGLRPGRYYITHSGRVIMASEVGVVDVLPEDVCHKGRLSPGKMLLVDFAKHVVVDDDELKSEYSARQPYAQWLQRQSITLKDILNSLDNEERVPPPTRTVNGSAEVTKHVGIRGMLPFLKVFGYTVETLEMLLLPMAKDGSEALGSMGNDTPLAVISGHPKLIFDYFKQMFAQVTNPPIDPIREANVTSLECMVGPEGDLTESVETQCHRLSLKGPLLSLEEMEAIKKMQYRGWRSKLIDITFPVEQGISGLEKTLDKICLEATIAIVEGYKLLVLSDRATSSERVPVSSLLAVGAVHHHLISTLERTRIGLLVESGDAREVHHFCTILGFGADAICPYVAIDSIWRMRLDGKVPPKMDRTLYSRDELVKQYFKASNYGMLKVMAKMGISTLASYKGAQIFEALGLSSEVVRKCFKGTPSRVEGATFEMLASDALQLHEIAFPSRLLPDGSADANALPNPGDYHWRKDGEVHLNDPLAMAKLQEAARTNSTQAYKDYSKLINELNKKCNLRGMLKFKFRQGKSIPLEDVEPASQIVKRFCTGAMSYGSISLEAHSTLAVAMNQIGGKSNTGEGGENSSRLEPLKNGLANPKRSAIKQVASGRFGVTSYYLTNADELQIKMAQGAKPGEGGELPGYKVIGDIALTRMSTPGVGLISPPPHHDIYSIEDLAQLIFDLKNSNPVARISVKLVSEAGVGVIASGVVKGHADHVLISGHDGGTGASRWTSIKNAGLPWELGLAETHQTLVANNLRGRAILQTDGQLKTGRDVVIAALLGAEEFGFSTAPLITLGCIMMRKCHKNTCPVGVATQDPILRAKFAGKPEHVINFLFMVAEEARELMAEMGFRNIDEMVGRADMLEVDEQVISSNEKLKNIDLSLLLQPAAKLRPGAAQTCIEKQDHGLELALDQILIQSALPALEKSLPVFLKLPIQNVNRAVGTMLSHEVTKRYNARGLPNDTIHFKFIGSAGQSFGAFLCAGITLELEGDSNDYVGKGLCGGKLIVYPNRLSKFDPENNIVIGNVALYGATSGEAYFNGMAAERFCVRNSGAKTVVEGVGDHGCEYMTGGIVVVLGKTGKNFAAGMSGGIAFVLDVDGSFKSRCNLGLVDLEEVEDQEDIVNLKSMIQQHHRNTKSKVAKEVLENFDSLLSKFVKVFPRDYKRILTELKDSGADGVQRQLSTRHPSTNLHGTKEHVISQNGASHLQDDKIPESAKRPTRVNDAQKNRGFITYERESLSYRPPGERVNDWKEVLSNGRESELLSTQSARCMDCGTPFCHQETTGCPLGNKIPEWNDLVHQGRWREALDRLLETNNFPEFTGRVCPAPCEGACVLGIIENPVSIKAIECSIIDKAFEEGWMVPRPPVIRTEKKVAIVGSGPAGLAAADQLNKAGHTVTVFERADRIGGLMMYGVPNMKTDKVEIVQRRVDLLVAEGVTFVTNANVGGDRLYSVERLRNEHDALLLACGATKPRDFSIPGRDLNGIHFAMDFLHANTKSLLDSNLQDGNFISAKDKRVVVIGGGDTGTDCIGTAVRHGCKNIVNLELLSRPPPTRDPSNPWPQWPKIFRVDYGHEEATEAFGKDPRSYEALTKRFIGDKNGVVTALEIKRVRWEKDAAGKFQLKEIEGSEEIIEADLVFLAMGFLGPEQTVVEALGLVTDQRSNIKAEFGRFSTNLEGVYAAGDCRRGQSLVVWAIAEGRQAAAEVDKFLMKNDSVANLSSQNQKDELLNGRDIARRNGNALARPGRHLNSDASTERAQEQESEVSVSGS

>EVM0019597.1|Lycopodium_clavatum/1-2260

MRSLLNPCVMQNYGRKGPTFDSTHIEATASAPSAIRFRHRTKLASDYVNVQTPQKVCNPQVNAYLSNNSYTHNDWAPKFSGLRRQVSSLRSESDLKWSKKRIQWWLWEGKAAVRGGTRMSFSQIPEFPLGLYDPALDKDSCGVGFIADLSAKPSRETVVDGLEMLVRMSHRGACGCETNTGDGAGILVAIPHDYFSLVCREEGFELPQVNEYGVGMFFLPPSDTRRRDSKNVFNEVAQSLGHTVIGWRRVRTDNSDLGASALSTEPVIEQVFVTASTISKSDFECQMYVLRKTSVAAIKDALRLERGNNRDFYICSLSSRTIVYKGQLKPDQLKKYYFADLGHEKFTSYMALIHSRFSTNTFPSWDRAQPMRLLGHNGEINTLQGNVNWMRAREGLLKCEALGISSEEMQKLLPIVDPRSSDSGAFDGVLEFLVRCGRSLPEAVMMLIPEAWQNDSTMDAGHKALFQYFSSIMEPWDGPALISFTDGRYLGATLDRNGLRPGRYYITHSGRVIMASEVGVVDVLPEDVCHKGRLSPGKMLLVDFEKHVVVDDDELKSEYSARQPYAQWLQRQSITLKDIVNSVEKEERVPPPMANYAKVNGSAEMAENLGIHGMLPFLKVFGYTVETLEMLLLPMAKDGTEALGSMGNDTPLALISERPKLIFDYFKQMFAQVTNPPIDPIREANVTSLECMVGPEGDLTESVETQCHRLSLKGPLLSLEEMEAIKKMQYRGWRSKFIDITFPVEQGITGLEKTLDRICLEATAAIDEGYKLLVLSDRATSSERVPVSSLLAVGAVHHHLISTLERTRIGLLVESGDAREVHHFCTILGFGADAICPYIAIDSIWQMQIDGKIPLKMDKPLHSRDELVKQYFKASNYGMLKVMAKMGISTLASYKGAQIFEALGLSSEVVKKCFKGTPSRVEGATFEMLARDALQLHEIAYPTRLLPDGSADANALPNPGDYHWRKDGEVHLNDPLAMAKLQEAARTNSKRAYEDYSKLINELNKKCNLRGLLKFKVTQGKSIPLEDVEPASQIVKRFCTGAMSYGSISLEAHSTLAIAMNQIGGKSNTGEGGENPSRLEPLKNGSANPKRSAIKQVASGRFGVTSYYLTNADELQIKMAQGAKPGEGGELPGYKVVGDIAFTRMSTPGVGLISPPPHHDIYSIEDLAQLIFDLKNSNPVARISVKLVSEAGVGVIASGVVKGHADHVLISGHDGGTGASRWTGIKNAGLPWELGLAETHQTLVANNLRGRTILQTDGQLKTGRDVVIAALLGAEEFGFSTAPLITLGCIMMRKCHKNTCPVGIATQDPILRAKFAGKPEHVINFLFMVAEEARELMAEMGFRNIDEMVGRADMLEVDKEVISSNEKLKNIDLSLLLQPAAKLRPGAAQRCIEKQDHGLEHALDQILIQSALPALEKSLPVFMEIPVRNVNRAVGTMLSHEVTKRYNATGLPNDTIHFKFIGSAGQSFGAFLCAGITLELEGDSNDYVGKGLSGGKLIVYPNRRSTFDPEKNIVIGNVALYGATSGEAYFNGMAAERFCVRNSGARTVVEGVGDHGCEYMTGGIVVVLGKTGKNFAAGMSGGLAFVLDVDGQFKSRCNLGLVDLEEVEDQEDIINLKTMIQQHQRNTKSNLAKEVLENFTSLLSKFVKVIPRDYKRVLTERKDSGADNVELQLSTRHPSTNSDGTNEHAISQNGASHLQDDKKPKSAQRPTRVSEAQKNRGFITYERESLSYRPPGERVNDWKEVLANGRENELLNTQSARCMDCGTPFCHQETSGCPLGNKIPEWNDLVHQGRWREALDRLLETNNFPEFTGRVCPAPCEGACVLGIIENPVSIKAIECSIIDKAFEEGWMVPRPPVRRTGKKVAIVGSGPAGLAAADQLNKAGHTVTIFERADRIGGLMMYGVPNMKTDKVEIVQRRVDLLAAEGVTFVCNANVGVDSIYSVEKLRHEHDALLLACGATKPRDLSIPGRDLKGIHFAMDFLHANTKSLLDSDLQDGNFISAKDKRVVVIGGGDTGTDCIGTAIRHGCKNVVNLELLSRPPPTRDPANPWPQWPRIFRVDYGHEEATEVFGKDPRSYEALTKRFIGDKNGVVTALEIKRVRWEKDAAGKFQLKEIEGSEEIIEADLVFLAMGFLGPEQTVVEALGLVTDQRSNIKAEFGRFSTNLEGVYAAGDCRRGQSLVVWAIAEGRQAAAEVDKFLMKNDSVANLSSANQKDKLLNGRDIARGNGNALAQPGRHFNSDASKERTQEQQSELSVSGT

>Klenit1_1_6065_kfl00237_0040_v1.1|Klebsormidium_nitens/1-2212

MSFSHAICGSQKGLALSVVGKKLVARRRRGVVMNLSQIPEARGLYDPAMDKDACGVGFVAELTSKPTRKTVADALEMLVRMAHRGACGCEANTGDGAGILVGMPHDFFAKRAGPDLGLDKLPPKGEYGVGMLFLPTNEERRAKVKSIFEQCAAQRGHDILGWRSVKTDNSDLGKGALDTEPVIEQVFMSPSSKSEANFESQLYILRRLVSRENRAELERALGAGAKRDWYICSMSSQTVVYKGQLTPAQLKGYYYKDLGSSDFTSYMALVHSRFSTNTFPSWDRAQPMRVLGHNGEINTLRGNVNWVRAREGLIKCTHLGLNEEELNLLLPIVDADSSDSGAFDGVLELLIRSGRSLPEAMMMMIPEAWQNDSNMDPARRALYQYHSALMEPWDGPALISFTDGHFLGATLDRNGLRPGRYYVTHSGRVIMASEVGVVDVPDDDVERKGRLSPGMMLLVDFEKGILVDDSELKRTFSQARPYEEWLDRQLMTLDGVVQSVPEEERQAAPIIGTLPVDPEKDRSMENMGVTGIVSPLKAFGYTIEALEMLLLPMATTGAEALGSMGNDAPLACMSERPKLSYEYFKQMFAQVTNPPIDPIREAIVTSTECMIGPEGDVTDTTEEQCHRLSLPGPLLTPEEMEALKKMDHRGWRTQTVDITFPKSEGAAGLENALVRICGEVTQALADGYKMLVLSDRATSPSRVPVSALLAVGAVHHHLVSTRQRTKIGLAIETGEAREVHHFCTLVGYGADAICPYLAIEAIWRLQIDGKVPPTKDGKLRTKDELTRTFFKASNSGVLKVLAKMGISTLASYKGAQIFEALGLSGEVVERCFKGTPSRVQGSTFLNLGEDTLRLHDLAFPPRRLAPEGSAEAHALPNPGDFHYRKDGEVHLNDPTAIAKLQEASSTNSRVVYAEFAKMTHELNKKVNLRGMLRFKTAPDGPIPLEEVEPASEIVKRFCTGAMSYGSISLEAHTTLAEAMNKIGGKSNTGEGGENPGRLEPLADGSQNPQRSAIKQVASGRFGVTSYYLTNADELQIKMAQGAKPGEGGELPGHKVIGDIAVTRHSTPGVGLISPPPHHDIYSIEDLAQLIYDLKNSNPSARVSVKLVSEAGVGVIASGVVKGHADHVLISGHDGGTGASRWTGIKNAGLPWELGLAETHQTLVANDLRGRTVLQTDGQLKTGRDIMVAALLGAEEYGFSTAPLMTIGCIMMRKCHKNTCPVGIATQDPVLREKFAGEPENVVNYFFMVAEEAREYMASMGFRNIDEMVGRADMLEVDPDVVNARNKLSHLDLSPILLPASTLRPGAAQRCVQKQDHELNMALDNELIEQARPALDKGMRVYCEAEVVNVNRAVGTMLSHEVTKIYKREGLPADTIHVKLTGSAGQSLGAFMCAGITLELEGDSNDYVGKGLSGGKVIVYPPRKSTFKAEENIVIGNVALYGATSGECYINGMAAERFCVRNSGAKAVVEGVGDHCCEYMTGGVTVVLGGTGRNFGAGMSGGVAYVLDIEKGFKQKCNRGTVDLEKVTDEEDKLELKFLIQQHRRHTNSRLAKWVLANFEQLLPKFHKVMPRDYKAVLEANKAAKREAEEKAAEAERLQKADAFAELQKLAQDAAANGVSPQVEEKHVELLNGQPKRPTRLADAVKNRGFVMYEREALPYRPAEERVKDWKEVLAGERQDDLLKAQSARCMDCGTPFCHQETSGCPLGNKIPEWNELVHQGRWREALDRLLETNNFPEFTGRVCPAPCEGACVLGIIENPVTIKTMECTIIDKAFENGWMVPRPPAQRTGKKVAVVGSGPAGMAAADQLNKAGHSVTVYERADRIGGLMMYGVPNMKTDKVEVVERRVNLMREEGIEFVTSAHVGVDGNFPIEKLRAEHDSVVLALGATKPRDLPVPGRELAGVHYAMEFLTANTKSLLDSNLEDGNYISAKDKAVIVIGGGDTGTDCIGTSLRHGCKSLVNLELLPQPPETRAPGNPWPQWPRVFRVDYGHAEAAHKLGKDPRQYEVLTKEFIGDGEGNVKGIKTVQVKWAKDESGRFKMEEVAGSEEVLDADLVLLAMGFLGPEQNLAQKLGLETDPRSNVKAEYGRFNTNVEGVFAAGDCRRGQSLVVWAINEGRLAAAEVDKWLMKDVALPKPEELEALLPGGGNGASTNGRHVESFPAGLEKAKEEAADACLAVQDENAPASVLQKCIDLSVEAAALSDRTITTEQSF

>Chabra1_339260_rna-CBR_g31844|Chara_Braunii/1-2158

MAAVESLRLMEGGGAEVAVPRCPAVDVGEAAGSNQRSEEEEELAILRKREVTGHSALPRKPMGLYDPAFESDACGVGFIAELSAEPSRKLVAEALDMLMRMSHRGAVGCEENTGDGAGILVALPHGFFQAVLREELGVELPPAGGYAVGMLFLPVNEQRRRLGKQMFADRAEALGHKILAWRPLRTNNAGLGRGALETEPVIEQVFVSPSALRPDVDFEKQLFILRRYVEGEFREALGLERGSPRDLYICSLSSKTVVYKGQLKPEQLPRYYADLADERFAAYTALVHSRFSTNTFPSWERAQPMRVLGHNGEINTLRGNINWMRARQGLLKCEALGLPPQELRKLMPIVDEDSSDSGAFDGVLEFLIRTGRNPPESVMMMIPEAWQNDTHMEEKKKQLYQYHSALMEPWDGPALIAFTDGRYIGATLDRNGLRPGRYYITHSGRVIMGSEVGVVDVDPKDVKKKGRLNPGMMLLVDFDEHIVVEDAEMKRKYSNARPYGEWLSRQMMTLEDIVRQFADAPEQLRAPPVNGVLADADLTDDGGSTTENRGLTKILAPLKAFGYTGEALEMLLLPMARDGMEALGSMGNDTPLACMSGRPKLLFEYFKQMFAQVTNPPIDPIREAVVTSTECMIGPEGDQTETTEEQCHRLSLKGPLLSLEEMEAVKKMDYRGWRTKVVDITFPRQEGPAGLERTLARICAEATAAIADGFKMLVLSDRPTSRDKVPVSSLLAVGAVHHHLVKTLSRTRLGLIVESGEPREVHHFCTLVGFGADAICPYLAMEAIWSLNEDRKVPPKGDGSLRTKTELVAAYFKASNAGILKVLAKMGISTLASYKGAQIFEALGLSSEVVDLCFRRTPSRVQGCTFQTLAADALRLHEQAFPRTALPPPAGSAESRALPNPGDYHFRATPDSEVHLNDPAAIAKLREAVRSNSNLAYADFATIVNNLNKKCTLRGMLQFKECPSPGGPIPLEEVEPAQSIVRRFCTGAMSYGSISLEAHTTLAAAMNRLGGKSNTGEGGENPARLVPQADGSHNPHRSSIKQVASGRFGVTAYYLTNADELQIKMAQGAKPGEGGELPGYKVVGDIAVTRHSTPGVGLISPPPHHDIYSIEDLAQLIFDLKNSNPGARVSVKLVSVAGVGVIASGVVKGHADHVLISGHDGGTGASRWTGIKNAGLPWELGLAETHQTLVANDLRGRMVLQTDGQLKTGRDVCIAALLGAEEFGFSTAPLMAMGCVMMRKCHRNTCPVGIATQDPVLRAKFKGEPEHVINYFFMVAEEARKYMAAMGFRRMDEMIGRADMLQADAEVIRSNPKLHNIDLSAILKPAAELRPGAAQHCVQKQDHGLESVLDVQLIELSRPALERAEPVRIQLPIRNVNRAVGTTLSHEVTKRYRLAGLAEDTIRIKFSGHAGQSFGAFVCRGITLELEGDSNDYVGKGLSGGKIVVYPPKDSGFAPKDNIVIGNVALYGATSGEAYFCGMAAERFCVRNSGARAVAEGVGDHGCEYMTGGVVVILGVTGRNFAAGMSGGIAYVLDTAGDFPTKCNLGLVDLERVTEEEDAALLRSMIEKHLLYTGSQLAAHVLQHMDQLLPAFVKVFPRDYKRVLMETKAKQEQEQEKKGALDNLRDMAAVPATMEEEESSRPSRDANAVKHRGFILYEREAAPYRPPEERLLDWDEVMKHEPQPDLIKAQSARCMDCGTPFCHQGYSGCPLGNKIPEFNELVHQNRGREALDRLLETNNFPEFTGRVCPALCEGACTLGIIDNPVAIKTIECHIIDRAWEEGWMVPRPPPRRTGMRVAVVGSGPAGLAAADQLNKAGHHVTVYERADRVGGLIMYGVPNMKCDKRQVVDKRVDLMRQEGVKFVTKCNVGGGTVTPTSLRAENDALVLACGATNPRDLMKTDGRHLKGIHFAMDFLTANTKSLLDSQLQDGNYISAKDKKVIVIGGGDTGTDCIATSLRHGCAGLINLELLPQPPAARAGDNPWPLWPRIFRIDYGHVEAEKKLGQDPRTYEVMTKRFFGNDRGEVTGLEIIRVQWSKEPASGRWVMKELPGTEQVLDADLVLLAMGFVGPEQYVARDLGISTHPRTTNFIAEFGQFHTNVDGVFAAGDCRRGQSLVVWAIAEGRGAAAAVNKYLAARAAAQDQASLAALNAGAIEVTA

>Chabra1_330157_rna-CBR_g18717|Chara_Braunii/1-701

MMGGRICCWLQLRKFVDVANIWVVFIVLQRFCVRNSGAKAVVEGVGDHGCEYMTGGITVILGGTGRNFAAGMSGGIAFVLDKTGEFPKKCNMGLVDLEKMTEEEDIQILRTMIQKHYKYTQSALAEQILREFDDLLPYFVKVFPRDYKRVMLEMKAKKAQESLDKLKEIAANGSVEEKPRRPTRVEGAVKHRGFVVYEREAAPYRKPEERLKDWGEVMLHEPQPALLNTQSARCMDCGTPFCHQGNSGCPLGNKIPEFNELVHQGRWREALDRLLETNNFPEFTGRVCPAPCEGACTLGIIENPVAIKTIECNIIDKAWEEGWMVPRPPVRRTRMKVAVVGSGPAGLAAADELNKAGHFVTVYERADRIGGLIMYGVPNMKCDKQLVVERRVNLMREEGVKFVTNANIGVTISAKDLKAENDALILACGATKPRDLRAAGRELKGIYFAMEFLTANTKSLLDSNLEDGNYITAKGKKVVVIGGGDTGTDCIATSIRHGCTNLINLELLPQPPAGRAEDNPWPQWPRIFRVDYGHVEAETTFGKDPRTYEVMTKRFFGNDKGEVTGLEIVQVQWSKDASGRWAMKEVAGSEKILEADIVLLAMGFLGPEQYIAQELGVSTDPRSNFKADFERFATNVDGVFAAGDCRRGQSLVVWAITEGRRAAAAANKYLMNKAALAESQSVASALKVGVVSKGEAVAVVA

>Chabra1_330151_rna-CBR_g18711|Chara_Braunii/1-2139

MAAEVQRLMESDGAEMAVASGDLEQTSQGVTDEAAEILKCRERMGHSAVPRKPMGLYDPAFDSDACGVGFIAELSTVPSRKLVVDALDMLIRMSHRGACGCEENTGDGAGILVALPHGFFQKALREELDAELPPPGEYAVGMLFLPVNEERRKIGKDIFTKRCEALGHNILVWRPVKTNNAGLGKGALETEPVIEQVFISPSVKSVVDFEKQLFILRRMVEGELRDALGLEHGQPRDFYICSLSSKTVVYKGQLKPDQLPGYFTDLKDDKFAAYTALVHSRFSTNTFPSWERAQPMRVLGHNGEINTLRGNINWMRAREGLLKCEALGLSREELKKLMPIVDEDSSDSGAFDGVLEFLIRTGRNPPESVMMMIPEAWQNDRLMDVNKKNLYQYHSALMEPWDGPALIAFTDGRYIGATLDRNGLRPGRYYITHSGRVIMGSEVGVVDVDPKDVKKKGRLNPGMMLLVDFDKHIVVEDAEMKKQYSAARPYGEWLAKQMLTLEDITESVPKEKLLPPTINGVVADADLGSDIKENRGLTKLLAPLKVFGYTVEALEMLLLPMARDASEALGSMGNDTPLACISDRPKLLFEYFKQMFAQVTNPPIDPIREAVVTSTECMIGPEGDQTETTEQQCHRLSLKGPLLTLEEMEALKKMDYRGWRTKVVDITFAREEGPAGLEHTLERILKESTQAIADGFKILVLSDRATSWGRVPVSSLLAVGAVHHHLVKTLSRTRLAVVVESGEPREVHHFCTLVGFGADAICPYVAINAIWSLNEDGKIPPKADGSCRTKEELVAAYFKASNAGILKVLAKMGISTLASYKGAQIFEALGLSSEVVDRCFRRTPSRVQGCTFKTLAADAMRLHEQAFPVVEPPKGSAEAKALPNPGDYHYRSTPDSEAHLNDPTAIAKLREAATLNSKIAYEDFSRITNELNKKCTLRGMLKFKGVGSSIPLEEVEPVQNIVKRFCTGAMSYGSISLEAHTTLAMAMNRLGGRSNTGEGGENPARLVPQPDGSHNPQRSSIKQVASGRFGVTAYYLTNADELQIKMAQGAKPGEGGELPGHKVVGDIAVTRHSTPGVGLISPPPHHDIYSIEDLAQLIYDLKNSNPGARVSVKLVSVAGVGVIAAGVVKGHADHVLISGHDGGTGASRWTSIKNAGLPWELGLSETHQVLVANDLRGRMILQTDGQIKTGRDVVIAALLGAEEFGFSTAPLMAMGCVMMRKCHKNTCPVGIATQDPVLRAKFKGEPEHVINYFFMVAEEARKYMASMGFRKMDEMIGRADMLEVDNEVIKNNPKLENIDLSAVLKPAAELRPGAAQFCVQKQDHGLHMALDVKLIELCRPALEKCEPVRLNLEICNVNRAVGTMLSHEVTKKYRLAGLPEDCIHIKFEGHAGQSFGAFICPGVTLELEGDSNDYVGKGLSGGKVIVYPPKDSGFVPKENIVIGNVALYGATSGEAYFCGMAAERFCVRNSGAKAVVEGVGDHGCEYMTGGIVVVLGVTGRNFAAGMSGGVAYVLDTKGEFQNKCNTGLVDLDKMTEEEDISILKTMIQKHYKHTHSAVAKHVLMNFDDLLPFFLKVFPRDYKRVLLETRAKKAQQAAASLDSLKAIAANGAVLEKPRRPTRVEGAVKHRGFVVYEREGIPYRNTEERLKDWKEVMVHEPPPALLKTQAARCMDCGTPFCHQGNSGCPLGNKIPEFNDLVHQNRWREALDRLLETNNFPEFTGRVCPAPCEGACTLGIIENPVAIKTIECTIIDKAWEEGWMVPRPPVRRTGMKVAVVGSGPAGLASADQLNKAGHFVTVYERADRIGGLIMYGVPNMKCDKEEVVERRVDLMRKEGVRFQTNVDVGVTISAKDLRAENDAVILACGATKPRDLPVPGRELKGIHFAMEFLTANTKSLLDSKLMDGKYISAKGKKVVVIGGGDTGTDCIATSVRHGCTAMINLELLPQPPAGRASDNPWPQWPRIFRIDYGHTESEATFGKDPRTYEVMTKRFFGNDRGEVTGLEIVKVQWTKDASGRWKMEQLAGSEQILEADLVLLAMGFLGPEQYVAQELGVTTDPRSNFKAEYGRFATNVDGVFAAGDCRRGQSLVVWAITEGRRAAAQADKYLARKAADVAASQGSLSALRMGMVSNGSTVGVTA

>Cre13.g592200_4532.1|Creinhardtii_CC-4532_v6.1/1-2284

MPQPVGQLVLPSAPAQSTPLHAVTAFPRLAAGSMARVGVPRSSAAASRPRAAVRPRWLPLGPGLGNRLVLCDAHSAVPPAQGLFDPENDKDACGVGFVGELSKKPSRKCIKDALTMLNRMTHRGACGCEANTGDGAGILVGMPDGFFQAVLMEEQGLKLPPLGAYAAGQVFLPQDEEVRQKVKDICDRVATSLGHVSLTWRPVPTNNRSLGKSAVDTEPAIEQWFITSTGNRSSLEVEQQLYLLRKQIEYALRADGIKDDDAYFCSLSSKTIVYKGQLTPEQVRVYFKDLQREDFRSYMALVHSRFSTNTFPSWARAQPMRNLGHNGEINTLRGNANWMRAREGAMACLKLGLPKEVVEQMEPIVPASSSDSGAFDSVLELLTRAGGRDMPEAMMMLIPEAWQNDPLMSKEKKAFYRYHSAVMEPWDGPALVSFTDGRFLGATLDRNGLRPGRYYVTKSGRVIMASEVGVVDVDPEEVVRKGRLMPGNILLVDFDEHRLVEDKELKERYANRRSYSMWLDEHALTLQQLTASVPPEKRIPAPLPGSRPVSALLPAAASNGNGNGNGNGKTAASNIVSFTPASNGAAAAVASELDAGVQSLLKPLKVFGYTREALELLMGPMAKSGAEPLGSMGNDAALAALSRRNRLPFEYFKQLFAQVTNPAIDPFREAVVTSLRCFVGPEVDLTSEPGPQQCARLELPQPVLDIDEMEALKNIDHPGGWKTKVLDCTFPVSEGPAGLRKALDRLCQEAEAAVKAGQYAMICLSDRGYGPNRAPVPSLLATGAVHHHLVQLRLRSNVGLLVDSGEPREVHQFCLLVGYGADGVCPYLAFDALAAMQRDGKLPASTPLEELKSKFVKGLGVGILKTMAKMGISTLQSYKGAQIFEALGLAPEVVDVCFRGTPTRIGGSGWEQLGRDVLALHDAAFASERMPDGSADAKALPNAGDYHWRNAPDAERHMNDPEAIAKLQAATSGNDRELYKQYSALNTRLSRNVQLRGLLRFKTEAATPIPIDEVQPAKEIVKRFVTGAMSYGSISLETHTTLALAMNTLGGKSNSGEGGENPRRLEPLADGSKNPFRSAIKQVASGRFGVTAYYLTNADELQIKISQGAKPGEGGELPGDKVKGDIAVTRGSTAGVGLISPPPHHDIYSIEDLAQLIYDLKSSNPSARVSVKLVSENGVGVVASGVVKGHADHVLISGHDGGTGAAKWSSIKHAGLPWELGLAETHQTLVANDLRGRTTLQVDGQLRTGRDVAIACALGAEEFGFSTAPLITLGCIMMRKCHTNTCPVGVATQDPELRAKFAGEPEHVINYFFMVAEEVREHLASLGLRNLDELVGRADLMEMDREAIAEGGAKLAGIDLGRLLTPAASLRPGAAQRCVQKQDHGLDAGLDVHLVPLCKAALPDEPNAASEPVYIEMEVQNTHRAVGTTLSHEVTKRFGDVGLPDDSIHIKLTGHAGQSLGAWLCRGITLELEGDANDYVAKGLSGGVVAVYPPRISTFRAEDNVIVGNVALYGAVRGECFIRGIAAERFCVRNSGARAVVEGVGDHACEYMTGGVTVVLGPTGKNFGAGMSGGIAYVYDPNDKFKSLCNVDVANDLLPVESAEDVRALKSLIQRHLKFTGSDVARRILLSWDRSRVHFKKVFPHEYRRALGEAEALAKAEAAEAALLASSDAKGLSPKDAFEELKAMAAAAVKSGQAPKALTLNMIPTADAQQDKRRMQKLAREGLPVKGISPTWEAGRPTIVAATAPAADKVRGFVNYDRKPLPYRAEAERIKDWDEVHDHSGTAAHAALLHTQAARCMECGTPFCHTQSTGCPLGNKIPEFNDLVHKGRWREALDRLLETNNFPEFTGRVCPAPCEGSCTLGIIEPPVSIKSIEATIIDKAFEEGWMVPRPPKLRTGKQVAVVGSGPAGLAAADQLNKMGHEVTVYERADRAGGLMMYGVPNMKAGKLDVVQRRVDLMAAEGVKFVMNAHVGGNVTAQDLVGKHDAVVLAAGATKPRDLPVPGREASGVHFAMEFLTANTKSLLDSDLADGKYISAKGKKVVVIGGGDTGTDCIATSLRHGATSIVNLELLDKPPATRAKNNPWPQWPRIFRVDYGHAEASHVYGADPRTYNVMTKRFIDDGKGNLKGVEIVTVKMEKDAKSGQFRPVEVPGSEKVLEADIVLLAMGFTGPEERLATSLGIKTDERSNFKATFGDFATSIPGVFAAGDCRRGQSLVVWAIREGRDAAASVDKYLQQKAGNGLLGQALDVQGGIFDVSGAPISVPQAGPPRDTSPKRPPVAV

>Klenit1_1_12616_kfl00058_0150_v1.1|Klebsormidium_nitens/1-544

MLIFSTGCPRFVKVSVALQLLTSLLSIASKTLACPATGYCTMEAAPESRHVVIVGGGLAGLTAALEVVKNKGRVTIIEKCAKLGGNSAKASSGINGAETAPQNALGIPDSTALFKEDTLRSGKGRCAAELVDVLVNKSAGAIDALQSLGIDLSNIAQCGGHSRPRTHRAAATAKATNIGGVIMHTLIDHLHSLPPSAVQVLTNTRALELLQDSRTGRVKGVRCRVGDGSGEGDRAVEADAVVLATGGYSADREGLLQKHVPGVSRLATTNGAWATGDGVKLGLAIGAGSVDLDQVQLHPTGLVDPKDPTNPTKFLAPEALRAIGGILLNQKGERFVDELSTRDVVAQAILEHCQPLQLEGATKRAAEEATLAPVTAFLVLNDESVERFGRSVAEFYMKMGFMQKFATEEELCDHWRLPVAHVARTLDEYGRVEHDAFGKPSAPVLFRLDQSLYTCLVTPSLHYTMGGLKIDTGGRVLTQAGDVIPGLWAAGEVTGGVHGANRLAGNSLLECMVFGKLVGEGASDPSAEQQPSPVPSSERQTQFA

>Chabra1_323161_rna-CBR_g4013-2_CBR_g4013|Chara_Braunii/1-807

MASVVGIALLFFVLEMVSMYGGHRFGGGLVLGQILQGCREGEHSRCDPLVGGGLDQVVERMLGNGEHLKEDGVYGPVPAVRVAIVGGGLAGLSAAVEVVKRGGSVVLIEKTARLGGNSAKASSGMNAANSTPQLLMGIEDDLDDFKSDTIKSGKGKSLKELVDVLVEHSADAVNFLQGHGIDLSNIVQLGGHSHPRTHRSRSDGKAMNIGWRIMSTLIKYMEAQPSESVQILKNAQATSLIVNDAQHVVGVNVTVTQKGESGDNIREEMQLFVDAVVLATGGYSADREGLLKEYVPEIRNLATTNGPFATGDGVKIAKEIGAGLMHMDQVQLHPTGFVDPKDPANPTKFLAPESLRGCGGILLNQKGERFVNELTTRDAATKAIMSECDRLPLEIVKRAGGKINGVEVVEGNVENNDNLPISAYMLMTEEGIDKFDRTIAKFYIGKGFIQRFENATEFCKAFALPEDVVVDVLENYGKQREDPYGKTVFPVLFSPHAPLYALIVTPSLHYTMGGLKFSQSGQILRDNGEPIVGLFGAGEVTGMHVFVFTLSGLVPTEYTALPLIQRYSPSKSCAVLRFALPSKDHVLGLLCGQYLAVRAQPRTPSEQPIVQFYSPLTPADEFGFFEVVIKHHQAAPGSMPDRMMSLPIGGTLEFAGPLGGFTYEPNMYEKVGMIAGGTGIAPMMQIIRTVIRHPADNTHLSLLYGNAVEDEILCKEELMYIAETRENVRVHMFLEQPPQRWTMGRGYITEEAIKERMPAPGTNSRIVMCGPATMIKVMKETLHRLGYADHQLYIFNETAIEDATAQS

>Chabra1_323160_rna-CBR_g4013|Chara_Braunii/1-763

MPELQADSVAKASISLPTHLYRLLHNDGVYGPVPAVRVAIVGGGLAGLSAAVEVVKRGGSVVLIEKTARLGGNSAKASSGMNAANSTPQLLMGIEDDLDDFKSDTIKSGKGKSLKELVDVLVEHSADAVNFLQGHGIDLSNIVQLGGHSHPRTHRSRSDGKAMNIGWRIMSTLIKYMEAQPSESVQILKNAQATSLIVNDAQHVVGVNVTVTQKGESGDNIREEMQLFVDAVVLATGGYSADREGLLKEYVPEIRNLATTNGPFATGDGVKIAKEIGAGLMHMDQVQLHPTGFVDPKDPANPTKFLAPESLRGCGGILLNQKGERFVNELTTRDAATKAIMSECDRLPLEIVKRAGGKINGVEVVEGNVENNDNLPISAYMLMTEEGIDKFDRTIAKFYIGKGFIQRFENATEFCKAFALPEDVVVDVLENYGKQREDPYGKTVFPVLFSPHAPLYALIVTPSLHYTMGGLKFSQSGQILRDNGEPIVGLFGAGEVTGMHVFVFTLSGLVPTEYTALPLIQRYSPSKSCAVLRFALPSKDHVLGLLCGQYLAVRAQPRTPSEQPIVQFYSPLTPADEFGFFEVVIKHHQAAPGSMPDRMMSLPIGGTLEFAGPLGGFTYEPNMYEKVGMIAGGTGIAPMMQIIRTVIRHPADNTHLSLLYGNAVEDEILCKEELMYIAETRENVRVHMFLEQPPQRWTMGRGYITEEAIKERMPAPGTNSRIVMCGPATMIKVMKETLHRLGYADHQLYIFNETAIEDATAQS

>Chabra1_323159_rna-CBR_g4013-3_CBR_g4013|Chara_Braunii/1-785

MGNPVAATSALKPPTREHARAAPTSRCDTCYSCTHQQVRYLLQLHPPADGVYGPVPAVRVAIVGGGLAGLSAAVEVVKRGGSVVLIEKTARLGGNSAKASSGMNAANSTPQLLMGIEDDLDDFKSDTIKSGKGKSLKELVDVLVEHSADAVNFLQGHGIDLSNIVQLGGHSHPRTHRSRSDGKAMNIGWRIMSTLIKYMEAQPSESVQILKNAQATSLIVNDAQHVVGVNVTVTQKGESGDNIREEMQLFVDAVVLATGGYSADREGLLKEYVPEIRNLATTNGPFATGDGVKIAKEIGAGLMHMDQVQLHPTGFVDPKDPANPTKFLAPESLRGCGGILLNQKGERFVNELTTRDAATKAIMSECDRLPLEIVKRAGGKINGVEVVEGNVENNDNLPISAYMLMTEEGIDKFDRTIAKFYIGKGFIQRFENATEFCKAFALPEDVVVDVLENYGKQREDPYGKTVFPVLFSPHAPLYALIVTPSLHYTMGGLKFSQSGQILRDNGEPIVGLFGAGEVTGMHVFVFTLSGLVPTEYTALPLIQRYSPSKSCAVLRFALPSKDHVLGLLCGQYLAVRAQPRTPSEQPIVQFYSPLTPADEFGFFEVVIKHHQAAPGSMPDRMMSLPIGGTLEFAGPLGGFTYEPNMYEKVGMIAGGTGIAPMMQIIRTVIRHPADNTHLSLLYGNAVEDEILCKEELMYIAETRENVRVHMFLEQPPQRWTMGRGYITEEAIKERMPAPGTNSRIVMCGPATMIKVMKETLHRLGYADHQLYIFNETAIEDATAQS

>MA_10427536g0010|Picea_abies/1-364

MRIAVIGAGVSGVVCASSLAEKGIAVTVFESARGPGGRMSQRRERTEDGKELCFDHGAPCFTVKDPGVRDLVHKWEAAGLVAEWKGPFGTFDAASGQFISQLEDSAKRYVGVPGMNAICKAICSQPGVEAKFGVTVAKVEWRREGGIWDLRGLQGESIGNYDAVVASDKNLASSRFTARTGLPPPLDIAELPELVQQMQTVLASSCFALMLAFSQPLTTIPVTGYNVRGSKILSWASCDSRKPGRSNNGSECWVLHSTAEYANHIVSQTDLKKPSDDILNMVKNDLFNEFQKTAPHMPSPFFMKAHRWGSAFPTKIIAKNDNCLWLDNKRFAVCGDFCVAPDVEGAMLSGLAAASKLIQVRNPL

>KAH9288468.1|Taxus_chinensis/1-186

MRTAVIGAGVSGLVCALSLAKNGVAVTVFESARGPGGRMSQRRERTEDGKELCFDHGAPYFTVKDPNVSDLVDNWEAAGLVAEWKGPFGSFDVGSGKLMLQLGSSMKRYVGVPGMNSICKAICHQPGVEAKFGVTVAKLHWRKEDGIWDLRGLQDESIGQFDAVVASDKNLASPRFTVQTGLPPPL

>Thupl.29377118s0006|Tplicata_v3.1/1-365

MRVAVIGAGVSGLVCASALAEKGVAVTIFESARGPGGRMSQRREKTEDGEELYFDHGASYFTVNDHSVRDLVDKWEAAGLLAEWKGPFGSFDVSSGKLIPQLGNSLKRYVGVPGMNSICEYICSQPGVEAKFGVMVAKLQWGEKEGFWDLRDLNIASIGKFDAVVASDKYLAYPRFTAQNVFPPPLESAEVPDLVPKLQTVMASSCFAVMLAFSRPLTSIPVSGCTIVGSKVLSWAACDSRKPGRFKSGSECWVLHSTAEYANEIISKTGLEKPPSDDILTNVKNELFQEFQKTAPDIPSPFFMKVHRWGSAFPTSILANNDECLWIENKRLVVCGDFCVAPDVEGAILSGLAAASKLLQIHGPQ

>Aspi01Gene60616|Alsophila_spinulosa/1-379

MASSSPSSTLADKLVAVVGAGVAGLACASRLAEQGFPVTVFDSGRGPGGRMSQRRERTSDGKFHYFDHGAQYFTVKDAVVQRLVDKWEAAGVVAEWKGKFGTYDASTKSFIGDANLLKKRYVGVPGMNAICQAFSQQPGVEAKFSVTVAKLDWREEQEVWMLADKNGQELGKFWAVVATDKNMASPRFFVQNGLPPPLVGAGVPSLAEKVAGVTSSSSFAVMLTFPQYLTSVPFDGFTIIGSKVLAWAARDSSKPGRDKLSIGECWVLQSTPEYAQEVIEEAGLGKPSEELLSTVTHSLLEDFKDIGETPAPLFAKAHRWGSAFPKASIAMEEKCLVDEGRRIAVCGDFCLGPKVECAILSGLAAAEKIQGLINADAKL

>Sacu_v1.1_s0036.g011750|Salvinia_cucullata/1-375

MASSSLTQKTVAVVGAGVSGLACASRLAEQGFGVTVFDSGRGPGGRMSQRRERTADGESYYFDHGAQYFTIKDRIVQTLVDKWESTGLVAEWEGKFGSYDVNSKNFIEDSDSSKKRYVGVPGMNAICQALSQQKGIQSKFGVTVTNFNWEEDQKSWTLADRSGQRFGQFHAVVATDKNVASPRFSIQHKVPPPLAGAGVPQLMEKLAGVSSSSSISVMLAFSERVTSIPFDGFTIVGSEVLAWASRDTSKPGRGERVVGECWVLQSTPQYAQEVIDKYGLEKPSNELLTSIAYSCFDHFKLIGNMPSPIFAKAHRWGSAFPKSYIAVEEKCLVDENRCIAICGDFCLGPKVECAILTGLAAAEKIQACLAARNKL

>Azfi_s0068.g036266|Azolla_filiculoides/1-220

MASSTSVAAEKMIAIVGAGVSGLVCASRLAEKGVPVMVFDSGRGAGGRMSQRRERSSDGKMYYFDHGTQYITVKDTNVQRLVEKWEDMGIIAEWKGKFGTYDTSSKIFVEDSDVMKKRYVGIPGMNAICQALSRQPGVQSRFGVTVTNMEWEKEHRNWILSGKDGQELGKFKAVIATDKNMASSRFFIQNKVPPPLGIASVSSSSSLSLCCSWWETLDVR

>Aqcoe4G053300|Acoerulea_v3.1/1-370

MKTVVSKVAVVGSGISGAVCASSLARNGVSVTIFESARGPGGRMSTRREITEDGKELYFDHGAPYFTANHKDVMALVCEWEAKGLVAEWKESTGIFDCVATKFIGIEKEGSSKKYVGIPGMNAMCRAMCHEPGVEAKFGTTVGTLQWLEDKNLWSPKDLDGKTLGHFDAMVASDKNVVSPRVTAVTGRPPPLDMSLVQELAPKLEEVPVLPCFCVMLAFSEPLSSIPVRSFAFKNSTVLSWAFCNSSKPERSSSTSECWVLHSTAEYAKRVISQTGLQKLPSSVLSNVAEELFQEFQRNELNIPRPFFMRAHRWGSAFPAIAVAEDKKCLWDRSKRLAICGDFCVSPNVEGAILSGMGAAANIMAIISSI

>Hyque.06G105100|Hquercifolia_v1.1/1-369

MNNAISKVAVIGSGISGAVCASNLARNGVSVTIFESARGPGGRMSQRRERTLDGKELLFDHGAPYFTACNGDVLSLIHEWEARGLVAEWKERFGSFDCISKTFVDIEKEELSKKYVGVPGMNSICRELCNEPGVESKFGVGVGRLKWLEDDDVWSLTGLDGQNLGCFKGVVASDKIIVSPRFTDVTGRPPPIDLDLLPELAVKMGEIPVGPRFAVMVAFEEPLSLIPVKGLSFRNSEVLSWASSDSSKPGRSTTSERWVLHSTEAYAKGVIAQTGLQKPSDAMLTKVADELFQQFQSTGLSIPQPFFKKAHRWGSAFPAVSIAKEDKCLWDGMKRVAICGDFCVSPNVEGAILSGMAAASKFADVLSCL

>FvH4_3g16060|Fvesca_v4.0.a2/1-370

MNMKGTKVAVVGSGISGAVCASSLARNGISVTLFESARGPGGRMSQRRENVEDGKELLFDHGAPFFTANNTEVQSLVHDWESKGLAAIWKEKFGCFDRTSNKFVDLEQEGLISKRYVGIPGMNSVCKALCREPGVESKFGTSVNRLEWLEDQNMWSLIGLDGQNLGQFKGVVATDKNIVSPRFTSVTGRQPPLDLKLFPELAVKLNDIPVRPCFALMIAFAEPLTSIPFKGFSIKNSEVLSWAHCDSSKPGRSTSSERWVLHSTMEYAQRIIAETGLQKLSDATLTKVAEELFQELQSLGLIISQPFFKKAHRWGSAFPATTIAREEKCLWDGNKRLAICGDFCVSPDVEGAIVSGLAAASQLSELLCCL

>Klenit1_1_1755_kfl00125_0100_v1.1|Klebsormidium_nitens/1-379

MSAGGEHAQVAVIGAGVCGLSCASALAKAGVPVAVFEQARGPGGRMSTRRQTLETGEQLRFDHGAQYFSAKDLAVQALVQEWVAAGVAAPWTPRLGTWEAGKGFVPEEADSKVRYVGVPGMNAICQHLSSQPGVSARLGTAVTGLEWRDGRWLLTGKDGAALGTYAALVVTDKNLASPRFSALNAGLPPPLESAGVPELFARVAAVSSIPSFALMLAFKQPLTQLPFDGLDVRNHKTLVWAGRDSAKPGRLKEPGAPECWVLHSNGDYAKRRVEEGPAGKPSAEWLGEIAEDMLAGFREIVPEMPEPLFARAHRWGGGFPAVAIANPGEACLWDESKRVAACGDYCLGPRVESAILSGLAAANKIFATRELLTLSKAHL

>evm.TU.supercontig_26.224|Cpapaya_ASGPBv0.4/1-807

MEAATEAPQEPSDNPNDVLFGGTSTETDPVLSPIHPQNHIDSLQNSTENNSPPPNTTVEGGGPVSDSQEDDSSDPVPEDPLDEHPQNPDPTEPGPPARKRRKRKRLFTEINGNPSLSKNRRHRVSGVAKEIDTEAWIAISVGFPIDSLTEEEIEANVVSTIGGTEQTNYIVVRNHILARWRSNVSVWLTRDHVLESIRAEHKTLVDSAYNFLVEHGYINFGLAPTIKEAKLRSFDGVERANVVVVGAGLAGLVAARQLVSMGFKVVVLEGRSRPGGRVKTRKMKGDGVVAAADVGGSVLTGINGNPLGVLARQLGLPLHKVRDICPLYLPDGKSVDADTDSRVEALFNKLLDRVCKLRQAMIEEVKSVDIPLGIALEAFRNVHRVAEDPQERMLLDWHLANLEYANASLMSNLSMAYWDQDDPFEMGGDHCFIPGGNERFVRALAEDLPIFYGRTVESIRYGVDGVMVYAGGQEIRGDMVLCTVPLGVLKKGTIEFVPELPQRKKDAIQRLGFGLLNKVAMLFPHNFWGRELDTFGHLTEDSSTRGEFFLFYSYSSVSGGPLLVALVAGDAARKFELMSPVESVKKVLEILRGIYHPKGIVVPDPVQALCSRWGKDQFSYGSYSYVSVGSSGDDYDILAESVGDGRVFFAGEATNKQYPATMHGAFLSGMREAANMLRMAKRRSLDLANKLNNDNEENDDLNELFETPDLTFGSFSSLFDPRSNELDSLALLRVKYHGTSDSICLYGLISRKQAVELSMLDEDASRMKTLHLDFGVKLVGRKGLSEAGESLVASVRAAKLNQQLFEQ

>FvH4_2g37680|Fvesca_v4.0.a2/1-791

METADQPQNPSDNLNDVVSGVSSPETDSSSPQETNQNHSSAPPQEAPISDSQDDSSEPIAEDTPNEPPQNPTGPGPAETTGLPPKRRRRRKKFFTELIGTPSMAKSRRTDLNKEVDTEALIALSVGFPVDSLTEEEIEANVVPVIGGVEQANYIVVRNHILSRWRSNVSFWLTREMALESIRLEHKALVDKAYNFLVEHGYINFGLAPAIRDAKLRSFDGSERGNVVIVGAGLAGLVAARQLVFLGFKVVVLEGRSRPGGRVKTKKMRGEGLEAAADLGGSVLTGINGNPLGVLARQLELPLHKVRDLCPLYLPDGKSVNSEMDDRIEASFNKLLDRVCKLRHAMIEEVKSVDVPLGTALEAFRRVCSVAQNPQERMLLDWHLANLEYANATLMSNLSMAYWDQDDPYEMGGDHCFIPGGNETFVRALAEGLPIFYDRTVQSIRYGADGVLVYANGQEFRGDMVLCTVPLGVLKKGSIEFVPELPQRKKDAIQSLGFGLLNKVAILFPYNFWGGDLDTFGHLTEDPSMRGEFFLFYSYSSVSGGPLLVALVAGDAAIKFEQMSPLESVNRVLEILRGIFHPKGIAVPDPIQAVCTRWGKDDFAYGSYSYVAVGSSGDDYDILAESIGDGRVFFAGEATNKQYPATMHGALLSGMREAANILRVAKKRTIIPSQKFNISSKEDEDLDNLFDTPDLTFGSFSVLFDPRSSDPESTSLFRVKLGGEKLDMGSFSLYGLILKKHTFKLRDVDGDGNRMTMLNFEFGVRLVGRKGLSGVAESLIACIRLAKSGMKQ

>GWHPCBIR017826|Santalum_yasi/1-832

MDSPEPPRPPSPPPPDPSDTPDAPTSGDSPSSDNHSMEPASHEPEPLDPEPPNPEPAEPQPVEPGPQRKKRRRRKKQFPGMISSADAVTGLRVLRPPGKSSGYSEKLMDDLVSAQIDAGAAFSKNRRRRMADLAKEVDVEALIAMSVGFPVDSLTEEEIEANVVPRLGGVEQANYIVVRNHILAWWRSNVNVWMTKDHALESIRSEHRQLVDSAYAFLLEHGYINFGLAPAIKEGRVRAHLGVERKNVVVIGAGLAGLIAARQLVSMGFKVAILEGRTRPGGRVKTRKMNGKEGVVAAVDIGGSVLTGINGNPLGVLARQLGFPLHKVRDICPLYLPDGRNINSEIDSSVEASFNKLLDRVCKLRQAMMEELKSVDVSLGTALEAFRRVYNVAEDLQERMLLNWHLANLEYANASLMSELSMAFWDQDDPYEMGGDHCFIPGGNERFIRALAEDLPIFYDRTVDSIRYGVDGVLVYASGQEFRGDMVLCTVPLGVLKRGSIEFVPGLPQRKIDAIQRIGFGLLNKVAMLFPYDFWGGEIDTFGHLTEDPSTRGEFFLFYSYSSVAGGPLLVALVAGEAALKFEAMPPVEAVRRALDILRNIFNPKGIHVPEPLQAVCTRWGKDCFTYGSYSYVAIGSSGDDYDILAESIGDGRVFFAGEATNKQYPATMHGAFLSGMREAANILRVAKRRTPTVPAEKSDSIGDEENDLDELFQTCDLKFGSFSVLFDPSSSDDDESCSLLRVELRGEKLNSGCTYLYGLIPKKHVIELSEVDGDANRMRILNQKFQVGLVGRNGLSSTGETLVTSIKSARYNHNGVENDAGHENENSNFMQ

>GWHPBAUK033344.1|Macadamia_integrifolia/1-909

METQPQILPLERPCNATAEDPSPVNHLPSPPNPPGNHLPDPQTITDSHLHTPTPQVPPENLLPLPQTAPENHIPVEDGPPEPSSHVSDSQRAPINHLLLPSAQVSSSDHLADSLIHVNHTPKRPLEDLLEASEKKRRRRKKLFPEMVPSATAVNGLRVLRPHVKSSIYDEKLMDEIIQMQINDSPSPSKNRRRKIADIAKEIDIEALIAISVGFPVDSLTEEEIECSVVSTLGGVEQSNFIVVRNHILARWRSNVSVWMTRDHALESIRSEHRGLVNSAYSFLLEHGYINFGLAPAIKEVNFRPIVGPEKANVIIVGAGLAGLAAARHLISLGFKVLILEGRKRPGGRVRTRKMSGEGVVAAADLGGSVLTGINGNPLGVLARQLGYPLHKVRDICPLYLPDGMSVNDEMDSRVEVSFNQLLDKVCKLRQAVMDEVESADVSLGTALEAFRQVYKVNEDPQERMLLNWHLANLEYANASLLSDLSMAYWDQDDPYEMGGDHCFIPGGNGRFIRALAENLPIFYDRIVESIRYGVDGVIVCAGGQVFRGDMALCTVPLGVLKRGSIEFVPELPQQKKDAIQRLGFGLLNKVAMLFPYDFWGGGIDTFGHLTDDENMRGEFFLFYSYTSVSGGPLLVALVAGESAIKFEMMSPVDAVGRVLDILRRIFTRIGIDVPNPVQVVCTRWGKDQFTHGSYSYVAVGASGDDYDVLAESVGDGRVFFAGEATNRRYPATMHGAFLSGLREAANILRVTNKRSLGLPEKTNNGREDDEDLATSNSLVPSEKTNNAREEGGDLDELFATPDLSFGSFAVLFDPRSVDLDSSSLLRVGFGAEKLDSGSLFLYGVISRKQVLELSAVDGDGNRVRLLNRNFGVSLVGSKALGSVGESLITCIKAARANLNGGGANESLHK

>TESI_002388-RA|Tetracentron_sinense/1-909

METEPPNPNPNPPIEHRNNATTDVPSPENHLPDSSIKLENHRLDPQNWPENQLRAPQSTFDNHLADSLIEDLIKDSSQPLFENHLEESQKLSPEQNGPPAKKRRRRKKQFPEMIPSAVAVNGLGVLRPHAKSSIYSEKLMDEIIQMQINDNTSFSRNRRRKVADLAKEVDVEALIAISVGFPVDSLTEEEIDCNVVSSIGGIEQANYIAVRNHILARWRSNVSTWMTGDQAIESIRSEHRGSVSSAYSFLLDHGYINFGLAPAIKKAYLRPPEGSEKANVVIIGAGLAGLAAARHLISLGFKVRILEGRNRPGGRVRTRKMNGEGVVADLGGSVLTGMNGNPLGVLARQLGFPLHKVRDTCPLYLPNGEAVNSEMDSRIEVSFNQLLDEVCKLRKGMLEEVNSVDVSLGTALEAFRRTHKIAEDPQEQMLLNWHLANLEYANASLLSDLSMAFWDQDDPYEMGGDHCVIPGGNGRFIHALAENLPILYGRTVESIRYGVDGVMVYAGEQVFHGDMVLCTVPLGVLKRGSLEFVPELPQRKKDAIQRIGFGLLNKVAMLFPYDFWGGGIDTFGHLTEDSSMRGEFFLFYNYSSMSGGPLLVALVAGESAIKFETMSPVDAVGRILDILRGIFVPKGVDVPNPVQVVCTHWGSDRFTYGSYSYVAVGASGDDYDVLAESVGDGRVFFAGEATTRRYPATMHGAFLSGMREAANILRMAKRRSLVPVEKINNAREERGDLDEHETMHGALLRGMREPANILGVPKEGSLVPVENQNNATEESGDLYELFETPDLSFGSFSILFDPRSIDLDSASLLQVRFGGDKLVSGSLFLYGLIPRKHVIELSEVSGDGNRVRVLNRIYGVKLVGRKGLGSAGESFITCIKSARANLNGGVTNEPHAHRNFLLPFPCNRY

>gene_prefix23363.t1_1|Ceratophyllum_demersum/1-714

MEEEIEYNVVPVVGGVEQTNYIVVRNHILALWRSNVSSWLTKEHAMKSIRMEHRGLVNIAYDFLMEHGFINFGLAPAIKEVQCKPLEWSEKPNVLIIGAGLSGLAAARHLLSLGFKVMVLEGRNRPGGRVVTKKMEGGGVIAAADLGGSVLTGINGNPFGVLARQLDFPLHKVRDKCPLYLPNGNAVNDEVDSRVEALFNRLLDKVCKLRQTMMDGMDCVDVSLGTALETFRSVFKVAEVHEERMLLNWHLANLEYANASLLSDLSLAFWDQDDPYEMGGDHCFIPGGNGRFIRALAENVPIFYGKAVNCIQYGIDEVRVITNNGQVFSADMVLCTVSLGVLKRDLIQFVPKLPQAKQDAIQRLGFGLLNKVAMLFPYDFWGGEIDTFGHLTKETHKRGEFFLFYSYVSVSGGPLLIALVAGASAIEFETMSPIDSTNRVLDVLKGIFAPKGVEVPDPIQVVCTRWGADKFTYGSYSHVAIGASGDDYDILAESVGDGRVFFAGEATNRHYPATMHGAFLSGLREAANILRVAKKRFSSLTDKDIVYTVKEKTDLVNLFHLPDMSFGNFSVLFDPTSSDLDANALVQVSFKGNKLGSNTVFLYCLILRKQAMELSSVKDDQNRLKLLQQRSGTKLVGSNGLCSVYDEDYLFFWVSSKFLFCMWPRFYTDQVTINILDGTIFLIGLRQDFAAAAGKVSDTIIILSFQSLMGRITI

>gene_prefix20312.t1_1|Ceratophyllum_demersum/1-946

MEPEPEVKQPSQHHFLQISSAENLYSTRSQNRISSEDHGSNYDTPGILKITSAEAMVSTENSNNAAKTLVSLETCIPRHEIQEIPNARTITTQENSSLSDAPRDHSTSIGTLKSSENQISVSEVSEPPNEINESIRDPGISENHLDPDEQPTKKRRRRKKQFPEMIPSAVAVNGLRVLRPQSKSNIYNEKLMDNIIRMQIDDVDPPSSKNRRRKFQELSREIDVEALIALAVGFPVDSLMEEEIEYNVVPVVGGVEQANYIVVRNHILALWRSNVSSWLTKEHAMKSIRTEHRGLVNMAYDFLMEYGFINFGLAPAIKEVQWKPLEWSEKPNVLIIGAGLSGLAAARHLLSLGFKVMVLEGRNRPGGRVVTKKMEGGGVIAAADLGGSVLTGINGNPFGVLARQLGFPLHKVRDKCPLYLPNGNAVNDEVDSRVEALFNRLLDKVCKLRQTMMDEMDCVDVSLGTALEAFRSVFKVAEVHEERMLLNWHLANLEYANASLLSDLSLAFWDQDDPYEMGGDHCFIPGGNGRFIRALAENVPIFYGKVVNCIQYGIDGVRVITNNGQVFSADMVLCTVSLGVLKRGLIQFVPKLPQTKEEAIQRLGFGLLNKVAMLFPYDFWGGEIDTFGHLTKETHKRGEFFLFYSYVSVSGGPLLIALVAGASAIEFETMSPIESTNRVLDVLKGIFAPKGVEVPDPIQVVCTRWGVDKFTYGSYSHVAIGASGDDYDILAESVGDGRVFFAGEATNRRYPATMHGAFLSGLREAANILRVAKKRFSSFADKDIVSTVKDKTDLVNLFHLPDMSFGNFSVLFDPTSSDLDANALVQVGFKGNKLGSDTVFLYCLVLRKQAMELSSVKDDQNRLKLLQQRSGAKLVGSNGLCSVCESLIGRLKASRPRFYTDQVTINIRDGALFLICLRQDFAAAVDKVSDNIIILSFQSLVGRITI

>Brara.C03318|BrapaFPsc_v1.3/1-898

MNPPDNNQDNAPSTPHSQLPTPPPQSNSNPNPDPTSISISNPSFEQMVSFSAPKKRRRGRSQRSTSSFHTLPNPGGILPNIDAAPRSTNPGIGDEIITINKDATTEALLALTAGFPADSLTEEEIDFGVVPVVGGIEQVNYILIRNHIISKWRENISSWIAKESFLDSIPNHCISLLDSAYDYLVLHGYINFGISQAVKDKVPPTQQQQSKPSVVIVGAGLSGLAAARQLLRFGFKVTVLEGRKRPGGRVYTKRMEGNRVEAAADLGGSVLTGTLGNPLGIIARQLGCSLYKVRDKCPLYRVDGKPVDPDLDMKVEGAFNRLLDKASKLRQLMGDVSMDVSLGAALETFRQVYGDATEEMSLFNWHLANLEYANAGLVSKLSLAFWDQDDPYDMGGDHCFLPGGNGRLVHALAENVPILYEKTVQTIRYGADGVKVTAGHQVYEGDMVLCTVPLGVLKNGSIKFVPELPQRKLDCIKRLGFGLLNKVAMLFPFVFWGTDLDTFGHLTEDPANRGEFFLFYSYAQVAGGPLLIALVAGEAAHKFETMPPTDAVTRVLQILRGMYEPQGINVPDPIQTVCTRWGGDPFSFGSYSNVAVGASGDDYDILAESVGDGRLFFAGEATTRRYPATMHGAFVTGLREAANMAQSAKSRGIRKRIDRNPSKNPHACATLLADLFRDPDLEFGSFSIIFSRRNPDPKSPAILRVTLSEPCKRNDDPVADQYSNKILFQQLQSHFNQQQQIQVYTLLTRQQALDLREVRGGDEKRLYYLCETLGVKLVGRKGLGIGADSVIASIKAERTGRKPALSSSGTKSGMLKVKSGALKRKLIRRIKGPVPLNQNNNGDSETVKHESLGNGKASVQATIADTIRSSSTQEENKWLGTGVASSSGGSLHGETVKK

>GWHPCBIR012243|Santalum_yasi/1-1098

MNPSNQTPDQFSPIPPFLHNPLQFSIFLAQSNTNPNPYPEPSSTSNPNPNLNQFLTLSVPRKRRRGRPRSTTSSVPSQTQVFNIPSFPNGTGNGNKEAGSSSSSSISSFPKPNGENPNHSVPGVPDISDEIIVINREATSEALIALTAGFPADSLTDEEIDAGVVSVIGGIEQVNYILIRNHIITKWRENVLNWVTKEMFFEVIPPHCKTLLDTAYNFLVSHGYINFGVAPAIKEKIPGEPCKPKVIVIGAGLAGLAAARQLMAFGFKVTVLEGRKRAGGRVYTKKMEGGNRSAAADLGGSVLTGTLGNPLGIVARQLSYSLHKVRDKCPLYCLDGKLVDPDMDTKVETAFNRLLDKASRLRQLMGEVSVDVSLGAALETFRQVYGDAVNAEEMNLFNWHLANLEYANAGLLSKLSLAFWDQDDPYDMGGDHCFLPGGNGRLVQALAENVPILYERTVHTIRYGSDGVQVIAGSQVFQGDMALCTVPLGVLKSGSIKFIPELPQRKLDGIKRLGFGLLNKVAMLFPHVFWGTDLDTFGHLTEDPSRRGEFFLFYSYATVAGGPLLIALVAGEAAHKFETMPPTDAVTQVLHILRGIYEPQGIAVPEPIQTVCTRWGSDPFCLGSYSNVAVGASGDDYDILAESVGDGRLFFAGEATTRRYPATMHGAFVTGIREAANMARYANGRASKVKVERSPSKNAQSCASLLADLFREPDLEFGSYSVILGRKNSDPKSTAILRVAFGGPRKKSHDVSKPDQQHSNKLLVQQLQSHFNQQQELHVYTLLSRQQALELREVRGGDEMRLNYLCEKLGVKLIGRKGLGPAADSVIASIKAERGKRKPASTSLALKSGVSKPKTGSLKQKMVRRAKIVSNGNSMVARAKIAASGNISMSPCNTDMGPPCSPDMGPPWNLDMGPPDNPDMGSPPNMYTPNPEMGAPSNLDMGAPVNRDMGAPPNRDMGPPPNANVDPLTNTNVDPPPNADMDLRSNSFAAPAADLNLEPSLDENMVPFFNVNMSPLPKVNVVPPTDMSLAPPDMSLAPPIDMSIPPHPDVNMVTPSNANALPPAMLSIMQSNLDVPIMDKGPPSADVLPTIVDLTNED

>Zosma06g29180|Zmarina_v3.1/1-865

MKIYPSIYSSVNHIPKGKTAQPAAPMSSEPPPPSPSSSLPFPSTLPVQQQNPNHTTASWLNPNFPFVTPLFARKRERTRFRRRPRVSLLTPTPISISSIPTITTVSTASSSDHSVPDDIITISIPASDPTPHSDVSEEIIVINKDATSEAITALSSGFPADTLTEEEIDANVVSIVGGIEQVNYILIRNHILCKWRENVSTWLTKESFADVVPANYQSLLNSAYGFLMFYGYINFGVAPAIKEHTKVEPRRKSVVVIGAGLAGLAASRQLMAFGFKVIVLEARKRCGGRVYTKKVEGLGKSAAAELGGSVLTGTLGNPLAIIARQLGGSQLHKVRENCPIYSTDGSPVDPVLDSKVENAFNRLLDKASKLRQCMGDVAMDVSLGAALETFREVYGDAVSEEEMNLFNWHLANLEYANAGLLSKLSLAFWDQDDPYDMGGDHCFLPGGNGRLVQALAENMPIVYEKTVHAIRYGADGVQVVTGGQVYECDMALCTVPVGVLKSRLIQFVPELPQRKLDAIKRMGFGLLNKVAMLFPHVFWNTDLDTFGHLSDEPTRRGEFFLFYNYATVAGGPLLIALVAGEAAHAFETMSPTDAVVLVLQILKGIFDPQGIQVPDPIQTVCTRWGVDTYSLGSYSHVSVGSSGDDYDILAESIGDGRLFFAGEATTRRYPATMHGAFLSGLREAANMTNHANARALRQKLEKSSSKNIESCASLLTDLFREPDIEFGSFSAIFGPKTVADPRSTAILRVTFGSCGKKNNNVEGFKSEQKHTNKPLFQQLQSHYNQQQQFHVYTLLSRQQALELREVRGGDEMRLHYLTEKLNVKLVGRRGLGSAADTVIAAIKDERASKRTKPVSVLVKRRGMSN

>Pso11G28110.0|Papaver_somniferum/1-1217

MSEQHPLTEPSISNPSETIPSLTEQQNPNSTSVPTSNSVAIQNTIPILSTDSSSTPIPNSTSVPNQNSISNSNNNPNLNHHHHPPLTPPFPKKPRKGGRAPRSSPLSINDQVFPQFNYSNGRPNGNINGNASGNRQHIPSSSSSNFMKSNLETSHSLPPLPSTTASIGRTIPDISEEIIFINKEATAEALTALTSGFPADSLTEEEIEAGVVSVVGGIEQVNYTLIRNHILTKWRENINSWLTKESLVRSIPSHGHTLLNSAYGFLVSHGYVNFGVAPAIKEKIQVEATKPKVIIIGAGLAGLAAARQLMTFGFKVVILEGRKRAGGRVYTKKMEGSDKIAAADLGGSVLTGTFGNPLAIIGRQLSCSLHKVRDKCPLYKPDGKPVDPDMDMKVETAFNRLLDKASRLRQSMGEVSVDVSLGSSLETFREVYGDAVTSEEMNLFNWHLANLEYANAGLLSQLSLAFWDQDDPYDMGGDHCFLPGGNGRLVQALAESLPILYEKTVHTIRCGSDGVQVLAGNQIFEGDMALCSVPLGVLKSGSIKFVPELPQRKLDGIKRLGFGLLNKVAMLFPHVFWGTELDTFGHLSDESSRRGEFFLFYSYATVAGGPLLIALVAGEAAHKFESMPPTDAVSHVLQILKGIYEPQGIDVPEPIQTVCTRWGSDPFSLGSYSNVAVGASGDDYDILAESVGDGRLFFAGEATNRRYPATMHGAFLSGLREAANMAHHANARALRVKVERSPSKNSQSCAALLTDLFREPDLEFGSFSVILARKKSDPKSAAILRVTFSGPRKKNTEGSKPDQQHSNKLLFQQLQSHYNQQQQLHVYTLLSRQHALELSEVRGGDEMRLHYLSEKLGVKLVGRRGLGPAADSVIASIKAERVIRRSASTFLALKSGTSKQRSANSKQKLVRRAKVIRNGSSAAPRPTSSASPSASRIASTAASALRPVSADSPPAPRPVSSGPPSAHRLGSAGLPLAPRLCSGPPSAQRPASGSPSTAPCPGTAAHPVSRLVSSAPLAPHSVAVASTATPHPNDAAHSTVPCNGSAASSAPELISAASVAADFDGVASSVAPYPSDAAHHIAPCIGSAAFPEPVCTLSLAPHLDDVTSSVTPHLSDAARFAEPFLGSAAPSVPHPPSPTLEPPMDDVSCPVLATPLEALSLPAFSPPLDPAPLLAFVSPPVLSPPSDFLSSPSAVSSLVPAPLPNSATPPMDTGLGS

>Pso09G11370.0|Papaver_somniferum/1-1210

MEPSISNPSETIPSLTEQQNPNSTSVPTSNSVSIQNTIPILSTDSSSTPIPNSTSVPNQNSISNSNNNPNLNHHHHPPLTPPFPKKPRKGGRAPRSSPLSINDQVFPQFNYSNGRPNGNINGNAGGNRQQIPSSSSSNFMKSNLETSHSLPPLPSTTASIGRTIPDISEEIIFINKEATSEALTALTSGFPADSLTEEEIEAGVVSVVGGIEQVNYTLIRNHILTKWRENINSWLTKESLVRSIPSHGHALLNSVYSFLVSHGYVNFGVAPAIKEKIQVEATKPKVIIIGAGLAGLAAARQLMTFGFKVVILEGRKRAGGRVYTKKMEGSDKIAAADLGGSVLTGTFGNPLAIVGRQLSCSLHKVRDKCPLYKPDGKPVDPDMDMKVETAFNRLLDKASRLRQSMGEVSVDVSLGSSLETFREVYGDAVTSEEMNLFNWHLANLEYANAGLLSQLSLAFWDQDDPYDMGGDHCFLPGGNGRLVQALAENLPILYEKTVHTIRCGSDGVQVLAGNQIFEGDMALCSVPLGVLKSGSIKFVPELPQRKLDGIKRLGFGLLNKVAMLFPHVFWGTELDTFGHLSDESSRRGEFFLFYSYATVAGGPLLIALVAGEAAHKFESMPPTDAVSHVLQILKGIYEPQGIDVPEPIQTVCTRWGSDPFSLGSYSNVAVGASGDDYDILAESVGDGRLFFAGEATNRRYPATMHGAFLSGLREAANMAHHANARALRVKVERSPSKNSQSCAALLTDLFREPDLEFGSFSVILACKKSDPKSAAILRVTFSGPRKKNTEGSKPDQQHSNKLLFQQLQSHYNQQQQLHVYTLLSRQHALELTEVRGGDEMRLHYLSEKLGVKLVGRRGLGPAADSVIASIKAERVLRRSASTFLALKSGTSKQRSVNSKQKLVRRAKVIRNGSSAAPRPTSSASPSASRIASTAASALRPVSADSPPAPRPVSSGPPPAHRLGSAGFPLAPRLCSSPPSAQRPASGSPSTAPCSGTVAHTVSRLVSSAPLAPHSVAVASTATPHPNDAAHSTVPCNGSAAPSAPELISAASVAPNFDGVASSVTPHPSDAAHHTAPCMGSAALPEPVCTVSLAPHLDDLALSVTPHLSDASLFAEPFLGSAAPSVPHPPSPPLEPPMDDVSCPVLATPLEAPPLPAFSPPLDPAPLLAFVSPPVLSPPSDFVSSPSYVSSLVSAPLPNSVTPPMNTGLDS

>CYCAS_002960|Cycas_panzhihuaensis/1-815

MVLGALHLGIRGLGEQSMADTSLIGLLSSSPLESDAQQRQRKRKRREAYKRDIVASGSMRALRQQARSTSYNENLIDEIIQRQLGDDPSSSGCRGRRKKIIDMAKEVDTEAMIAHSVGFPADSLTEEEIEAGVVSSIGGVEQANYIIVRNHILARWRENVSVWLNRDHAMESIRSQHRSLVNSAYNFLLTHGYINFGVAPAVKGTIPEESSRANVVIIGAGLAGLAAARQLLSFGFKVVIVEGRKRAGGRVYTKKMEGNGMIAAADLGGSVITGIHGNPLGVLARQLSFPLHKVRDKCPLYQPDGQPVDPHIDSKVEDAFNKLLDKACQLRQAMDEVAVDISLGKALETFRHVYGVAERLEERRLLNWHLANLEYANAGLLSKLSLAFWDQDDPYEMGGDHCFLPGGNCRFIQALAENLPIFYDKTVQTIRYGTDGVQVLAGGQVFQGDMALCTIPLGALKNGSVRFIPELPERKVDAIQRLGFGLLNKVAMLFPHVFWGAEIDTFGHLCDDTNRRGEFFLFYSYASVSGGPLLIALVAGEAAIKFESMSPTDAVYRILEILKGIYGPKGVEVPDPIQTVCTRWGSDPFCFGSYSHVAVGASGDDYDILAESVGEGRVFFAGEATNRRYPATMHGAFLSGLREAANISKVANNRSMPLKMEKNLSKDNQSYAALLAELFEVPDLAFGSFSILFDPQSADPNSLSLLRVLVGGGRRKTGDNAKSDQEQTFRLILQHLQLPSNQPPQQLQLYSLISRHQALELSEVEGGDENRLRHLYEKFGAKLVGRRGLGTLGDSLVASIKLVRSSNRNGLTSVP

>evm.model.Chr17.2931|Isoetes_sinensis/1-940

MASSEMEVEEAPIPLNSASDAVAVADAAAAAAAASSAFAEIKRKRKKREALAAMEEAVGEAGRLSGRGKSMRALRQQVRNMSYDENLMDAMIEQRLGGNAASSSVKGRKKKTMDMEKEVDVEAMIAAAIGFPSEFLTEEEIEAGVVSNVGGEEQANYIVIRNCILARWRENVNVWLDEDSVMESIRSQHKNLVRSAFKFLLSYGYINFGVAPTVKAAIPPEATKSSVVIVGAGLAGLAAARQLLAFGHKVVIVEGRQRPGGRVYTKKMEGGGQIAAADLGGSVVTGIHGNPLGVLARQIGFPLHKIREKCPLYQPNGAVVSEDVDLKVETQFNKLLDMASKLREEMENVADSIALGTTLETLREQQGVALVPEERQLYDWHLANLEYANAGLLSNLSLAFWDQDDPYEMGGDHCFVPGGNVRLVSALAEDIPIFYGKTVHTIRYGSGGVQVITADQVFEGDMVLCTVPLGVLKRRAVTFEPELPQRKLEAINRLGFGLLNKVAMLFPYVFWGPELDTFGRLADSPPRRGEFFLFYSYAAVSGGPLLIALVAGEAAINFEKMPPLEAIQRVLSVLRAIYQPKGISVPDPIQTVCTRWGSDPLCYGSYSNVAVGASGDDYDILAESVGDNLFFAGEATSRRYPATMHGAFLSGLREAGNIAAQAATKVAPPKLPKDVHSYAAVLADIFREPDLEFGNFSILFDPQSQDPQSYALLRLLIGGNAKRKPAENNSTDQQKEQFQPKQQQLHLYTLITRQQANDLREVRGGDKIRLAHLCEKYGVKLVGRRGLGIQGDAVIAAVKWTRSLKRSAASAGSPVLGVKTTKAVSVSVSGSQNLLVTASETSSPTIDSMKDASRSTRPQHNEVSRETKESSEDKSSVSHGSKHRLISKEERLKHGCIGIDARGNANWSLMKKCNCQFCMHRKQELEKKVPDEKKVVEEAS

>evm.model.Chr18.2969|Isoetes_sinensis/1-940

MASSEMEVEEAPIPLNSASDAVAVADAAAAAAAASSAFAEIKRKRKKREALAAMEEAVGEAGRLSGRGKSMRALRQQVRNMSYDENLMDAMIEQRLGGNAASSSVKGRKKKTMDMEKEVDVEAMIAAAIGFPSEFLTEEEIEAGVVSNVGGEEQANYIVIRNCILARWRENVNVWLDEDSVMESIRSQHKNLVRSAFKFLLSYGYINFGVAPTVKAAIPPEATKSSVVIVGAGLAGLAAARQLLAFGHKVVIVEGRQRPGGRVYTKKMEGGGQIAAADLGGSVVTGIHGNPLGVLARQIGFPLHKIREKCPLYQPNGAVVSEDVDLKVETQFNKLLDMASKLREEMENVADSIALGTTLETLREQQGVALVPEERQLYDWHLANLEYANAGLLSNLSLAFWDQDDPYEMGGDHCFVPGGNVRLVSALAEDIPIFYGKTVHTIRYGSGGVQVITADQVFEGDMVLCTVPLGVLKRRAVTFEPELPQRKLEAINRLGFGLLNKVAMLFPYVFWGPELDTFGRLADSPPRRGEFFLFYSYAAVSGGPLLIALVAGEAAINFEKMPPLEAIQRVLSVLRAIYQPKGISVPDPIQTVCTRWGSDPLCYGSYSNVAVGASGDDYDILAESVGDNLFFAGEATSRRYPATMHGAFLSGLREAGNIAAQAATKVAPPKLPKDVHSYAAVLADIFREPDLEFGNFSILFDPQSQDPQSYALLRLLIGGNAKRKPAENNSTDQQKEQFQPKQQQLHLYTLITRQQANDLREVRGGDKIRLAHLCEKYGVKLVGRRGLGIQGDAVIAAVKWTRSLKRSAASAGSPVLGVKTTKAVSVSVSGSQNLLVTASETSSPTIDSMKDASRSTRPQHNEVSRETKESSEDKSSVSHGSKHRLISKEERLKHGCIGIDARGNANWSLMKKCNCQFCMHRKQELEKKVPDEKKVVEEAS

>EVM0016568.1|Lycopodium_clavatum/1-771

MDQEAQFLGRGKSNRALRQHARITSYDEALVDEMIEQHLGGNPSSVARGRKKKPLDMDKEVDVEAMIAVSIGFPRDALTEEEIEAGVVTTVLGEEQANYIVVRNHILARWRENVNVWLDQGAVMESIRSQFKNLVASSYKFLLSHGYINFGVAPAIKAAIPAESTKASVVIIGAGLSGLAAARQLLAFGHKIVVVEGRQRPGGRVYTKKMEGGGKMAAADLGGSVVTGIHGNPLGVLARQLGFPLHKIREKCPLYQPDGLPVEEDMDSKVETQFNKLLDLASTWREQMESVADSIALGTTLETLREQASVAVTTDERRLFDWHLANLEYANAGLLSKLSLAFWDQDDPYEMGGDHCFVPGGNVRLISALAEDIPIFYGKTVHTIRYGSSGVQVITADQVFEGDMALCTVPLGVLKRKAIRFEPELPQRKTEAIGRLGFGLLNKVAMLFPHVFWGAELDTFGHLADHSSRRGEFFLFYSYAAVSGGPLLIALVAGEAAINFERMPPLQAIQRVLTILRAIYEPRGISVPDPIQTVCTRWGSDHLCYGSYSNVAVGASGDDYDILAESVGGHLFFAGEATNRQYPATMHGAFLSGLREAGNIAAQAAASSAPPKIPKDIQSYAAILADIFREPDLEFGNFAIVYDPQSEDPKSFAILRLLIGGNAKKKYGDGQGMEPHKYQYQQQQQQLHLYTIITRQQALDLREVRGGDGVRLVYLCEKFGVKLVGRRGLGVNGDAIVAAVKWARAARKSASSVHIPNTVVETKVQTVNAAT

>Tle2c00317.1|Takakia_lepidozioides/1-1003

MADEGVGSQAEEDVSPSIVEDTGQKVNWEEGDLSREVAEDVAAASGSDLGFLFDVGLGAELAANFNEQAGDVGRLYEGNNGSRLADDLSREEHVKVEHNGNEGSRLIDDLSREAYVEVEHADRGTGHPSEWDSSPNVKVVDGGTGYPSVWDRSPNVQVENSLEADRGTGHPSVWVTSPKIKLEDSLEAAERDVNSQGAGQLGEVLTAAAPQREGLEGNVVAENAVKRKRKRRERVVVMQEVAEQGAGGKIIRALRQQARVSTYDESAGDAEIEEQLGSTPSAAKGRKRKQVDVEKEVNVEAMIAASIGFPRESLTEEEIEAGVISTVGSVEQANYIVVRNHILAKWRENVNSWLDQDMVMESIRSQHKSLVAAAYKFLLSHGYINFGVAPAIKAAIPAENNKASVVIIGAGLAGLGAARQLLAFGHRVLVVEGRQRPGGRVYTKKMQGDNLMAAADLGGSVVTGIHGNPLGVLARQLGLPLHKIREKCPLYQPDGHPVKEDVDAKVESQFNKLLDKTSKWREDMEQVAEGISLGATLEILQQAAGVGETASERQLYDWHLANLEYANAGLLTNLSLAFWDQDDPYEMGGDHCFLPGGNAQLVVALAEDIPIFYGKTVHRIRYGSGGVQVLAGERVFEGDMALCTVPLGVLKKGMVKFEPELPQRKLDAIMRLGFGLLNKVVMLFPEVFWGSELDTFGHLSEHPSKRGEYFLFYSYAGVSGGPLLLALVAGEAAIEFERTAPLTAVTRVMSVLRGIYEPKGIQVPNPLQTVCTRWGSDGLSCGSYSNVAVGSSGDDYDILAESVGDGRLFFAGEATNRRYPATMHGAFLSGLREAGNIAAATAARMAPPKGERGIPKDIQSYTATLTDLFKEPDLEFGCFSVIFDHQTNDPNSLALVKLVYGGPAKKKIGESATVDELLHLYTTITRQQAYELREVKGGDKGRFIYLCQKFGVKLVGRRGLGPEGDALVVAIQNRGARKFGSIPTSNVPKLPKSCAVSSAVPTK

>Tle4c03005.1|Takakia_lepidozioides/1-771

MEEKRKRRRGESLDPIEEVIELGGGGRGIRAVRQKVRATYDETALDAMIEQQLGPSPAKGRKRKPASEEKEVNVEAMIAASIGFPRESLMEEEMEAGVVSTVGGVEEANYIVVRNHILARWRENVNAWLDQETVMETIRSQYKSLVAAAYKFLLAHGYINFGVAPAIKSAIPAEPDKAVVIIIGAGLAGLAAARQLLAFGHKVVVVEGRQRPGGRVYTKKMEGENLTAAADLGGSVITGIHGNPLGVLARQLGLPLHKIREKCPLYEPDGQSVRDDVDTKVESQFNKLLDKASKLREGMDQVANDISLGTAMEELRQGHGVGGTPQERQLYDWHLANLEYANAGLLTNLSLAFWDQDDPYEMGGDHCFLPGGNVQLIGALAEDVPIFYGKTVQTIRYGGGSVQVTAGEQVFEGDVALCTVPLGVLKKRTVKFEPELPQRKLDAIDRLGFGLLNKVVMLFQEVFWGPELDTFGHLAEHPSKRGEYFLFYSYAAVSGGPLLLALVAGEAAIEFESKPPLEAVTRVMSILRGIYEPRGIQVPNPVQTVCTRWGSDYLCYGSYSNVAVGASGDDYDILAESVGDGHLFFAGEATSRRYPASMHGAFLSGLREASNIAAATAARIVAPKAERAIPKDVQSYAATLADLFKEPDLEFGSFSIIFDHQTNDPLSLALVRLVYGPTAKKNVGSATVDQRLHLYTTITRQQAFELREVKGGDQGRFYYLCQKFRVKLVGRRGLGPQGDALMVAIRWNRAARKSSSTPLPNSTELPNLARP

>AANG009954|Anthoceros_angustus/1-932

MSLELPALDDIAKPASQNGGPAAPRVESPAIGLPFWHPALASGAARDAASLEPANRDGAAPAPAPAPPTGVGDGSHGNGDGGEGISLAGRDGGDGGAPATGDGDGAVQAAAGDGAGSAMEGREGAGAAAEELEGGAGGAAGKKKRKRKPRELFMDMEENQVRGRGMRPVRQHARPQTYDEAALDSMIDEQIGDIAGPGKRKKKQVDTAKENNMEAMIGAAIGFSREFLSDEEVAANVLPSDTSAQERANYIMVRNHILTRWRENVSAWLSQDTVMETIQTKFRGLVSVAYKFLMQYGYINFGVAAAIKANIPKEPNKGTVVIIGSGFAGLAAARQLLAFGHRVVILEGRQRPGGRVYTKRMEGVTSGGTVVAAADMGGSVVTGVHGNPLAVLARQLGFPLHKIREPCPLFQPDGSPVRQVLDKKMEKHFNDILDEVSLYRQEVENAEELSLGGALDVLRAKLGVVETEEERQLFDWHVANLEYANAELLSKLSLSHWDQDDPYEMGGDHCWLAGGNVRLIAALAENIPIFYGSTVHSITYSDTGVQVETGDQVFVADMALCTVPLGVLKRGCIKFHPELPQRKLDAIRRLGFGLLNKVVMLFPQTFWGSELDHFGHLTDDRSTNGEFFMFYSYAAVAGGPLLLALVAGQSAINFEDMAPVEAVTRVLEVLRGIYQPLGITVPDPLQSVCTRWGNDSLCYGSYSNVAVGSSGEDYDILAESVGDGRLFFAGEATNRRYPATMHGAFLSGLREAGNIAVRAAARLASTTKLSRGIQKDPTACANFMEDIFSEPDLKFGCFEVIFDPRTPDPMSSVLLRVVLVDSTRRPSGFIGEGQNGEPLKEPLKLYSVISRIQALQLMGVGGGDEMRLRFLIESLRARLVGRRGLGAHGDALAHAAKWARTGARRDSARPVKSGSAGNRQAPSPHGGRLPPS

>evm.TU.supercontig_80.102|Cpapaya_ASGPBv0.4/1-701

METSVSNGLGLRRTSRKKACIRNYDENLMDELIENHLGGALRKRNRTREELEKETETEAMIAFSLGFPIDALLEEEIQAGVVRELGGKEQNDYIVVRNHILARWRSNVQVWLTKGQIRETVSSECEHLISSSYDFLLHNGYINFGVSPSFTSFSSEEATEASVIIVGAGLSGLAAARQLLSFGFKVIVLEGRNRPGGRVHTQKMGMKGKAAAVDLGGSVITGIHANPLGVLARQLSIPLHKVRDKCPLYTPGGAPVDKEIDSKVEFIFNKLLDKVSELRQIMGRCANNISLGSVLETLSQLYAVASSREERQLLDWHLANLEYANAGCLSDLSAAYWDQDDPYEMGGDHCFLAGGNWRLINALCEGVPILYGKTVTGIRYGNEGVDVIFDGQVFQADIVLCTVPLGVLKKRSLKFEPELPERKLAAIERLGFGLLNKVAMVFSHVFWGEDLDTFGCLSEDSNQRGEFFLFYSYHTVSGGPVLIALVAGEAAERFECTDPSTLLHRVLTKLRGIYGQKGIDVPDPTQTICTRWGSDPFSCGSYSHVMVRSSGDDYDILAESVGSRLFFAGEATTRQYPATMHGAYLSGLREASNILQATSDDPKSKGFMRVTLDFSGDDSRKVSKDSCESPVNLPLQLYTVLSREQAQELQQVTGGDKGKLSYLNKNLGLKLMGTIALVNIANSSITTIRRGRFRRSATSAQ

>Chabra1_331238_rna-CBR_g19719|Chara_Braunii/1-1317

MAGCENGSAAAAAAGSEPGLMGTLAAGGDARAVASTGAVVGSGSPQPSMEMVGGSTGPSVPTAAVEPVAFLPTDRVNLTINSYPETASVNSRGVPPLSLRSCSVAAPAAENAGCTTSGYVGTATGAERPAVVVPAAGSVGGDLPLGVFTTSMDVEPSSTAMAVDDSTLSTPLKAVAAAARLSTDPASSPAPTSVSAATAADTAGPGADGVDPAASPPASGPSLLDVVALAAPSLANVAVVPGPRSTGDLAGACTTPSHTRDAAGSVALISDPQAASAARSAAHALAASAPPPVADTVVAVASIVAAAAAAAAAVPMQADGDVAASAPPPVTDAEVKVDKHDESRAKRRKSNNGLPLTPENEIVYLDDSEDESQHDSDDEVVFVEACVRRKGHKETRRGVPGKRGGRYSPRANSPRNAANLEKENMERGGTEGGRSGGTEAGRSGAEQGGESEGGRSGLRTNSGKKSGEREEKHNVKDGKDTNGSVSSRSVSSCDERRGQRSRGKDVSGGGLDGGGKRKRSGGKGRQGQSRSSSGESHQDSEAVENVGYAGTGSGLTAQSKGGVKRRNGAVKQMGRDGVVAVSMGFPVDSLMEEEIEAGMISTLHDSEQLAYISVRNHILARWRDDVTRFLTEDEVLKTLPAQHHSLVPATYKFLRMKGYINFGVAQALNQRPVPPLGIKKSVVVLGAGLAGLAAARQLLSFGLQVVVVEARQRPGGRVYTKRVEGSGRVAMADLGGSVLTGIQGNPLGVLARQLGFDLHKIRDNCPLFQPDGRPVDEELDVKVENQFNRLLDVANRWRERMESMAAQCVSLGTTLELLRDDYKIGKTPEEKQLFEWHLANLEYANAGLLSELSLAFWDQDDPYEMSGDHCFVPGGNVRLVSALAESLPIFYNSVVQNVKYGKEGVKVTCRPVGGDEPRVFEGDMVLCTIPLGVLKKRSIKFDPELPQRKLEAINKLGFGLLNKVVMLFPYTFWDEDLDTFGHLVEDPDQRGEFFLFYSYAPVSGGSLLLALVAGAAAVRFEQMPAQESVTRLMEVLRKIFGPKGIKVPYPLQTVCTRWGSDPLCLGSYSHVAVGASGEDYDILAESVDDCLFFAGEATIRRYPATMHGAFLSGLREAGNIANTAFASSAPAEAEERVPSDVQSFASTLCDLFEQPDVEFGCFGVIFDPRSMAADSSALIRVVVRGGDPMKGVNGGGGAANAQEEQLLQLHLYSVILRWQAIELREVKGGDNVRLHILTERYGVKLVGRRGLGPTGDALVAAVRTARATRAARKAVIAATAGLGGGAGLASTGGSGKGSFSSRGPFDAPGSLSLVTAA

>Klenit1_1_6344_kfl00249_0010_v1.1|Klebsormidium_nitens/1-1383

MPSPMGYDIVQEARVQMQGTGSAAAGPPMKVENTQGMEAELESDAQAASDPSLDVKEPSRRRRVPKRAFGEEDQKAVEEFLMEEEGSTKKKRRRRSLQEDDHMEAIIAAGMGFPNNYLTEEEIRAGVVGSLGGIEQANYIVIRNHILAKWRQNVDKWLHESAILENIRNEHKPLVRAAYRFLSQAGYINFGVAPAIKERVRTDGSKATMIVIGAGLAGLAAARQLLAFGHKVIVLEGRARPGGRVHTRQMDAGGHSASADLGGSVITGTDGNPLGVLARQLKLPMHHIRSDPCPIYGLDGQRVDEELDKRVEADFNTLLDKAEETRKAMATVADSISLGSTLDTLRRSYRVAQSEEERQLLDWHVANLEYANAGLLHDLSLAFWDQDDPHEMAGDHCFLTGGNGRLVSALADGLPIFYGRTVRTVRYSDAGVRVHTADGQVFEGDMALSTIPLGCLKRGDVTFDPPLPKEKDAAIRRLGFGLLNKVALLFPHNFWGTDLDTFGRVAPDAHRRGEYFLFYSYAAVSGGALLLALVAGEAARRFEKVPGADVIAEVMAILRSIYEPQGILVPNPLSTLCTRWGSDPFAYGSYSHVAVGASGDDYDVMAESCGNGRLFFAGEATTRWHPATMHGAFLTGLREAGWMDATATARAQGQPVPVQIAPSSLSLQAVTLAEVFRKPDMEFGGFSVVLDPRSSSSSAPALLRLILRSPQSLTKPLKPPQDTEAEPKTTAKTTSSHPNGNGAAAPKAAVSANPAVSQTLGNLPNASRFRVPDGLAPGAAPGVSPELDQAAERLSEARQDTGRGAAVAQGTGPVVTPLPGTRETFPGSESLKEPDRGDGIRTGSSEIAGNVNGLSGANGFHAYGRMRAFAQAVGSGLGSANAESGLATSSPKPAGLGLGPLGLESEPFSQGNNGIGGEPNRTSFVASSVAPVENGAGFGDEERRPDNTMTKLFSFLKTEALKVSPRTPPPVGAFPVSGIPVSGIPANGIPAAPPGGTVRPASALRVEPTDARTESHSQASDTGRAAQTAVSSHGVNTLSMPRPEPRFLGAAVPVHISANSGMPGAGTGGENVPGACRLLPFASARPMPVQSPQLDVRNTPLESPASRILNPAIPSSSNGPQTMNGPALPVSTGAQTGTAAGLVQNRTSHIASAPVTRPAVENLADARPESTSVGAGEKGGTSPGSQPSDLVDDKSTVLKVPAANGDAKGGSKKKPAQVAGAAGGRKKRRRAAPTDAFTPPQKELAVYTVVTRGQAEQLREVKGGDRARLQTLVDTFGVKLVGRHGLGSAGEALLAALNGPHLVAGGLLPRGQADIMKRMNKAKEVTEGNGVRQQVRPRQSRPQAEPEREGIAKSEPRVLKAAVRTTVTASVSLSKPKPEPASS

>Cucsa.159720|Csativus_v1.0/1-1909

MDGDNKKSGFRKRTKPKDGGFDSDVDEPIGSLLKLKRSRNSKKTKLNVDDGGGRDKMVDKKGAKLPVQEDFGGMDDTLASFRKKLKRPKKVSGTGIAREQSSALSVTESLNPLFNTSKGHGDLDTRLRPENGELMDREDSDSSAKIDAEMRCEAPDLESKDMVMGISSRRSANCSLDKQLDDSLSAFVQKVQSGSTRKSVVSTTFRPDCKDEASEDKLSPFCRAVSEDHEAHSIVSINSSSKLAQVVKKPDSELTTSYLISCSHCTKENCNPGRGQCHQGIKHDQEEDPCSLNGHENPDMRPCISNEVADKDSKNFSQFRDNFRVLERKASCEIKNVLKHCSCGNTITNSHLAEMGSFQDGLGENQINENMCSSCRPLEKINENHDLCVGVSSRDFCDAVAQETTVNLSKTIPGVDCEGKERLLVKYHDELSKSTNFCESSSNPQLSAGFDSTKVDKTDSDSDNLNTGNDEPNDKGRSMQKENATISNRIDSTAVQSLTLQKLGPKYPDFCPGGNFSMISDSQPAKVPLEMDGPNNILTGKEVKVSSLGSFTPDDNDLEDVISAPESEKDLKLSALQRVARKTKKPRHEDMAYEGDIDWEVLISERAVDGDHSFRSRKDSTSTTFTEAETGGRAAVSAGLKAHAVGLLEKIKFKDVLKRKGGLQEYIACRNQILGLWCKDVTRILRLVDCGVTDTPSMDEPPRFSLVREIYAFLNLRGYINAGIASEKAKSESDIKYDYELGEKKVGDVSVASAADSEEGVSVMVKNSDASNAENDVSAGCEVILKDAEGRDPVIANNLDLPKPVELEQELVHDLEYCIPDPTQVKFVGDVPGKAASHLTNQSRNSWGPISSDECVGDDQQQQSNSEIKKKVIVIGAGPAGLTAAKHLLRQGFTVTVLEARNRLGGRVHTDRSSLSVPVDLGASIITGVEADVATERRPDPSSLICTQLGLELTVLNSDCPLYDIITCKKVPLDMDEALEAEYNSLLDDMVLLVAQRGEHAMAMSLEEGLEYALKRRRMARGMDVCSEEEVLSPFERRVMNWHFANLEYGCAAMLKKVSLPNWNQDDLYGGFGGAHCMIKGGYSTVVESLGGGLDVRLNHVVADISYSTSDIGFNGNQCAKVKVSTTNGCEFLGDAVLITVPLGCLKAETIKFSPPLPEWKRLSIQRLGFGVLNKIVIEFPEVFWDDSVDYFGATAEETKWRGQCFMFWNVRKTVGAPVLIALVVGQAAVERQYMSSSDNVSHALMVLRKLFGEAVVPDPVASVVTDWGRDPFSYGAYSYVAVGASGEDYDILAKPVGKCLFFAGEATCKEHPDTVGGAMMSGLREAVRMIDILSDGYDFTAEVEAMEAAQRQSECENDEVGDIITRLEAVKLSDALYKSSLDGARILTIEALLQDLFFSSKTTAGRLHVAKELLNLPAETLKSFAGTKEGLTVLNSWILDSMGKDGTQLLRQCVRILVVVSTDLLAVRLSGIGKTVKEKVCVHTSRDIRAIASQLVSIWLEVFRKEKAANGGLKLSKSVSAVELLKRKSNKDSSSGKPPLHANNSTLDSRGNLLTAASAAMPLQSDVNMKNDNSKQLKFELENSSKSDISSSRSRGSFGKQDAEMEDNIAMTEEEEAAFAAAEAARAAALAAAKAYASAEAKSAMQLPKIPSFHKFARREHYAQMDECEYKKKLSGSVLGRQDCISEIDSRNCRVRNWSVEFSAACVNLESSRMSADNLSQRSHSNEIVSQLNFREHSGESAPVDSSIYTKAWVDTAGSVGMKDYHAIERWRTQAAAAHVNDEEDSNTNWHKPMWNNDQVANESSISQVTINKEPMRNHHRGADRIKQAVVDYVASLLMPLYKARKIDKDGYKSIMKKSATKVMEQATDAEKGMTVSEFLDFKRRNKIRAFVDKLIERHMATKPVTKS

>Prupe.1G370000|Ppersica_v2.1/1-1883

MDGEEKKSGFKRRSKLIEANINSDDDEPIGSLLKLKRQRNPKKVKPRLEGVSERSRKVEDEEEDLGGLDDTLASLRKKLKGPKKDSGAGTIRGRDVVQSLDRSSNGPVEDGGLDEKSVSMVLEKGPVMVDDGSDVTIDMEVENKLKGKGKRPKVSESRGYGEGSNSSLDHHPEDSLSAIFRKAQSGFTKKSRTSSSPKENNGSQVLEDGLNPSSEGVTGNTMPVMNNEAIVDPYGSNFQEGPCNSDKVNDGDSKHLTHKTHTFEDGLKHCSMVDLSTLTKYDVERHNSIPCPKQMEDVHGVGDRDSKGGFTDAFCIESKDVLDMSEDKRLVSSSHLPQNSLTFHVKMEDELDSDRCQNFSEHTQHPLCSFASGTLKMEETHNICNGQISCTEEPGLASHSLPEEKAVIADRRLSSLDITSSRAQKLGYANQLNHQGESFETCVHSNKSTAPIQKGSSAIRQDLSSDEASKERNGPNHDYLIIDEEADGASPPLCTYENESCPEDTVSLPDVENKDTKLSAVQRVVRNVRKRRHGDMAYEGDADWEVLINDQGLDSDNSFRTRVKFDSSSSIGTEAESGEAAAVSAGLKAHAVGPVEKIKFKEILKRRGGIQDYLECRNQILALWSKDVSRILPLTDCGVTDTACAGEPPRASLIRDIYAFLDLSGYINVGIACEKDKAEPGSKHDYKILREKNFEEISGVSVADSEDGVSFIIGQVKSSKTSVDVKNGVLIENENVTRRATNDNGLITAVELALSNATNHVDCNSAYQENSSGDARLQNRLDNMDFSSSDPTGDALGGGAVPVATPEMKNVSHSIQSASHDHAVRNSNPQCGPEVRMEIIVIGAGPAGLTAARHLQRQGFSVTILEARSRIGGRVYTDRSSLSVPVDLGASIITGVEADWATERRPDPSSLVCAQLGLELTVLNSDCPLYDITTGAKVPADLDEALEAEFNSLLDDMVLLVAQEGEHAMRMSLEEGLEYALKRRRMAQTGTSVKEKELHEQELLSPLERRVMDWHFANLEYGCAALLKEVSLPNWNQDDVYGGFGGAHCMIKGGYSTVVESLGEGLCIHLNHVVTDISYGIKDAGLNTNQCNKVKVSTSNGNDFLGDAVLITVPLGCLKAETIKFSPPLPHWKHSSIQQLGFGVLNKVVLEFPDVFWDDSVDYFGATAEETDLRGQCFMFWNIRKTVGAPVLIALLVGKAAIDGQNMSSSDHVNHALVVLRKLFGEASVPDPVASVVTDWGRDPFSYGAYSYVAVGASGEDYDILGKPVENCLFFAGEATCKEHPDTVGGAMMSGLREAVRIIDILTTGNDHTAEVEAIEAIQRQSDSERDEVRDITRRLDAVELSNVLYKNREALLQDMFFNSKTTKGRLHLVKELLSLPVETLKSVAGTKEGLTTLNSWILDSMGKAGTQLLRHCVRLLVLVSTDLLAVRLSGIGKTVKEKVCVHTSRDIRAIASQLVSVWLEVFRKEKASNGGLKLSRQAAAVDSFKRKPIRDPSSGKPPLHTFHGALEHKGSLQDSASTANHLPLNAVKKVNGKAIKIEAVNSSKLEINSSRSRGSTGRPDTKLEVNNFVMTEAERAAIAAAEAARAAALAAAEAYASSEAKSSTLLHLPKIPSFHKFARRDQYPQIDEYDFRRKWSGGDLGRQDCISEIDSRNCKVRNWSVDFSAACVNLDSSRMSVDNLSQRSHPNETASQLNFREHSGESAAVDSSIYTKAWVDTAGSVGIKDYHAIEMWQSQAAAADPDFFHPAPYINDEEDSNTTSKKLSWKHEGIVNESSVSQVTVNKESLKNHHRGADHIKQAVVDYVASLLMPLYKAKKIDRDGYKSIMKKSATKVMEQATDAEKAMAVSGFLDFKRRNKIRAFVDKLIERHMAVKPTVKS

>FvH4_2g39110|Fvesca_v4.0.a2/1-1909

MEGDEKRLGAKKRSKLMKVNHNSDGDDEPIGSLLKKRQRNPKKVKPGLEGERGKKVEAGEEDLGGLDDTLASFRKKLKGPKKDSGARGRSSSLDVVQSSDQDGGSDVKSVSRSAEKGLVTGDDNGCDVIRDVEAENKLKGKGKRPKVSGLVSGEGSNSSLDHQLQDSLSAFFPKAQSSVNKMSHPSSSLREKSGSQDLEDGLSPSSEGVGGNSMPVAVQGHGSVSKLTHEKPRFDDSLLSDSGLDPFGSVIDQNKIEERNRVSQDSDCNRQNQERSQGLCSIPDEMMKLEDRKNEPTVDPSGLNVQEEPCSLDKSNVSDSQHLQKTQTVENRMRHCSVTNSSTLTSWELKGEMPGVGNSDSKGGFDDALSIQSTDVLARCTSGADCEISSSAGKQILMPHYNDDLLNKSNSSPNALKREETTRDCGTAYTEGHDLDSSHLQEENVIIADCQISSIQFIHQAKALQTASIQKASYCEDLSSDEASKERIIPKHDYITGNEEVDGASPPLYAMLDVNESFPEDSVSQPDIENKDSKLSAILRAPRNIRKRRHGDMAYEGDVDWEISTNDQGLDSDNSIRARVKLDSSSSIGTEAESGGAAAVSAGLKAHAVGPVEKIKFKEILKRRGGLQDYLECRNQILALWSKDVSRILPLTDCGVTESACVDEPGRASLIRDIYAFLDLSGYINVGIAAEKDKAEPGSKHDYKILREKPFEEISGVSVADSEDGVSFIIGQVKNSKASTDAKNGITFNSENLTEGAPKDNGHVGAVALELLDVKNPAECQTDYLENCSADVRFQSRLDNMDVSSSDPSGETLDGGVVPVVTPEIKHESQSIQSTPYDHLPSNNTLQCGPEVRKEIIVIGAGPAGLTAARHLKRQGFSVNVLEARSRIGGRVFTDRSSLSVAVDLGASIITGVEADWATERRPDPSSLVCAQLGLELTVLNSDCPLYDIETGQKVPAELDEALEAEFNSLLDDMVLLVAQKGERAARMSLEEGFEYALKRRRMAQSGSAKEKELHGSRDDGRTNIDGRVADKSCSKQELLSPLERRVMDWHFANLEYGCAAPLKEVSLPHWNQDDVYGGFGGAHCMIKGGYSTVVESLGEGLRIHLDHVVTDISYGAEDGELNNNQRNKVKVSTSNGSIFCGDAVLVTVPLGCLKAETIKFSPPLPQWKHSSITRLGFGVLNKVVLEFPDVFWDDSVDYFGATAEETDLRGQCFMFWNIKKTVGAPVLIALVVGKAAIEGQNMSSSDHVNHALVALRKLFGEASVPDPVASVVTDWGRDPFSYGAYSYVAVGASGKDYDILGRPVNNCLFFAGEATCKEHPDTVGGAMMSGLREAVRVIDILTTGHDYTAEAEAMESIQSESASEKDEVRDITRRLDAVELSSVLYKNREALLQDLFFNAKTTKGRLHLAKELLTLPAETLKSFAGTKEGLTTLNSWILDSMGKAGTQLLRHCVRLLVLVSTDLLAVRLSGIGKTVREKVCVHTSRDIRAIASQLVSVWLEVFRREKASNGGLKLSRQASGVDSLKRKTVRDSSSGKPPLHLYHGAFEHKGSLQDSASTGSQLPSNSNAKKMNGKTIRLETANSSRFGGSTGKPHDDEFAMTEEERAAIAAAEAARAAALAAAKAYASSEAKSSSLLQLPKIPSFHKFARREQYAQMDEYDFRRKWSGGVLGREDCISEIDSRNCKVRNWSVDFSAACVNLDSSRRSVDNLSERSHPNEITSQLNFREHSGESAAVDSSIYTKAWVDTAGSVGVKDYHAIEMWQSQAAAADPDFYHPDPYVKDEEDSNTTSKGLSWKHDGLVNESSVSQVTVNKGSSKNHRRGADQIKHAVVDYVASLLMPLYKAKKIDREGYKSIMKKSATKVMEQATDSEKAMAVSEFLDFKRRNKIRAFVDKLIEKHMAVKPGVKS

>VIT_218s0001g13640|Vvinifera_v2.1/1-2126

MDDEEKKSGSKRRLKHIEIGFDSDDDEPIGSIFKLRRQTNPKKVKLGLDSGGKTGEKLKSVEARAEKLVGEDEELGGMDDTLASFRKKLRGPKKDTGSGTAVVRGSNLNVVELKDVEEGRGIRDYGSDVTMDKGLEKKLKRKSKRSKIVSTKKKTGDSVCQRSEGSSLQDQKEMGLWLEKGSNHSSDENLEDSLSAFVRRAQSGLIRRSRTSCSKKKRGPQGLEDGLSHRCEGVSEDSHAVVVKIPRSSSGSRLMHENLTSKDSLHPVSDRGLVDLGPEKTKTVENLRPGDGSGEVFNHIKKILQSVDPIKGVSSVPGATDDISRSSDDRVDQSSESIMEDTNHITALQQPHSHLVAYSNRSIEHQYSESNRLTERVQEENTVVPCDSNQFCDGDSEEFIHKQMKENSSASIHKTKLDTQNLKDVLRHCSMGKTTDLVHGAVQKHVAVAKQGGEIHGSDEGQSSVGFNDALTQQHEGVATIYHSSADQKACSSLSEKGTVAHCFDDNLLKRPHETVSKGTHKQIPGNSLEVSLKSPSWNSLPGYVKIEEPSKSETGLDFDKSSQNAELHSAYSVLNSMKMGGTSSDSDGPNQIPFTSIEEPDCASVDLEKEEDALIPDAGLSSIAPTSAGVHESGFASQMDCPEKSVETDHLDESFPLIQKCDSDFHQNQPSHDASRGDHVPIHDYLSASEEANGASSPSITPDKNDAYPEDAGSMPDPEIQDNKSSSAQRTLRKPKKHRQRDMAYEGDADWEILIHEQSFPQSHLVEDTDQPLRTRGKFDSSLNMVSGTDNGGAAAVSVGLKARAVGPVEKIKFKEVLKRKGGLQEYLECRNLILGLWGKDMSRILPLADCGVADTPSKDEPPRASLIREIYVFLDHRGYINVGIASEKEKADPDSKHNYKLLKEKTFGEKSGIAIADSEDGVSFILGQGRSSETSTEAKSGLVFDDENKASDGAVVDLRTLEPSTLVEPKECLADDYQEHGCMDANEFNRKVNLDVSESSCRIDDSGTIPTIAPELMNESCGVESASMDSAKRDHNVQFDSDVRKKIIVVGAGPAGLTAARHLQRHGFSVIVLEARSRIGGRVYTDHSSLSVPVDLGASIITGVEADVDTERRPDPSSLVCAQLGLELTVLNSDCPLYDIVTGQKVPADLDEALEAEYNSLLDDMVLIVAQKGEHAMKMSLEEGLEYALKRRRMPRLGSDYTENELQNLDKPSLDSEKIIVDRKMLERNSSKEEVLSPIERRVMDWHFAHLEYGCAALLKEVSLPYWNQDDVYGGFGGAHCMIKGGYSSVIESLGEGLHILLNQVVTDVSYSSKDAGGTGSQCKKVKVSTSNGSEFSGDAVLITVPLGCLKAEAIKFLPPLPQWKHSSIQRLGFGVLNKVVLEFPEVFWDDSVDYFGATSEQRNWRGQCFMFWNVKKTVGAPVLIALVVGKAAIDHQDLSSSDHVNHALSVLRKLFGETSVPDPVASVVTNWGKDPFSYGAYSYVAVGASGEDYDILGRPVENCLFFAGEATCKEHPDTVGGAMMSGLREAVRIIDILTTGNDYTAEVEAMEAAQRHSEGERNEVRDILKRLEAVELSNVLYKSSLDGDLILTREALLQDMFSNAKTTAGRLHLAKELLTFPVEALKSFAGTKEGLCTLNSWILDSMGKDGTQLLRHCVRLLVLVSTDLIAVRLSGIGKTVKEKVCVHTSRDIRAIASQLVNVWIEVFRKEKASNGGLKLLKQTTASNSAKGKSFKDLASGKPPIRVHHGALDFKGSSQVSASARSHSPSSASIKKDNGKPVKLESMTNSKPDGNQSRSPGSVGRMDVEGEEGNNLMSEEEKVAFAAAEAARAAALAAAEAYASEAKSNTSLQLPKIPSFHKFARREQYAQMDESDLRRKWSGGVSGRQDCISEIDSRNCRVRNWSVDFPAACVNLESSRMSADNHSQRSHSNDIACPLNFREHSGESAAVDSSLFTKAWVDSAGSVGIKDYHAIERWQSQAAAADSDFYQSTRHIRDEEDSNTISQPPTWKHDRQANESSVSHVTVNKELVKNQPRGAENIKQAVVDYVGSLLMPLYKARKIDKEGYKSIMKKSATKVMEQATDVEKTMAVSEFLDFKRRNKIRSFVDKLIERHMAMNPVGNP

>Hyque.06G074300|Hquercifolia_v1.1/1-1908

MECDGLDSRSVHGGSGKKSGLGLDVESLSPKPRVKKMNGEERKSGYKKRSKPIEVGFDSDDDEPIGSLLKLKSKRNPKKVKVGLDGGAAKGKEVEVGMLKLPVEDEVLGGMEDTLASFRKKLKGPKKDVGSGTKAGRGLSVNVAEPSGQCLNGFDTSVDRKLESSDKGTTKRPDTSLKLRKDGDDTKSEHALGGQRFGDNSIKNHCLDDNVEDSLSTYIQKAQSGLTRKSRNSLRPKQRKETQVSNDEHSSNPDDIVDDALPVARKRSSSASLLAQKAVKSEDDMRQQSSRIPVNSVHDQNKNDSLSQVCDRNSEAAAEDSVPISTFQRSCSHLIACSSKILQDGEVNRQTTGIQEGYTLDLLGSDHLSSAKVKDTISVPIQESISEIQNIKYESEIRLKETASDLVLDAVEMPNSTTAPEQTVELASQHEDLLMENGKVSASHGKKIHGPCLDDLFNKPCGDMSPEILTLVPRKKLGLSAKPTSQNSSSCEKMELASQLEDMPDFDQYPKGLPKKEGNYRDDGGANRTRSTRIEESDIASVSSQQDKVSASDGRLLPMTPCEVHKSDHAIQIKHEVKSLETDDKCNHYPREPWLDEGKIASSLSFTPDRNEIFSADNKQSQVQRTVRKVKKRRDGDMAYEGDLDWEILIHDHGFLESYQVEDNDQFIKTREKFNPFSNMAEEAENGGAAAVSTGLKAREAGPVEKIKFKEVLKRKGGLQEYLDCRNRILGLWSKDVTRILPITDCGVTATLSAHEPPRASLVREVYAFLDQSGYINVGVASERERADPSTEHNFRLLKEQNFGEVSSGPVADSDDGLFSVHSQARSSEASPEVKNGMLFDVENGYTENCCTEAKAPNKIWSSDPSSEVLNGGTVSVHQTQCDSEVGKRIIVIGAGPAGLAASRHLQRQGFSVTVLEARSRIGGRVFTDRSSLSVPVDLGASIITGVEADVATERRPDPSSLVCAQLGLELTVLNSDCPLYDIVSGQKVPIDLDEALEAEYNSLLDDMVLLVAQKGEHAMRMSLEDGLEYALKRRRMARLGRNFEESALHKLEEKFRERIFSDKCSLKEEILSPLERRVMDWHFAHLEYGCAALLKEVSLPYWNQDDVYGGFGGAHCMIKGGYSSVVESLAEGLCVHLDHVVTNISYHTKDQCDKVKVSTSTGNEFSGDAVLVTVPLGCLKAKKIEFSPPLPEWKQLSIERLGFGVLNKIVLEFPDVFWDDSVDYFGATAEETNRRGHCFMFWNVKKTVGAPILIALVVGKAAIDGQHMSSSDHVNHAVMVLRKLFGESVPDPVASVVTDWGKDPFSYGAYSYVAIGASGEDYDILGRPVGNCLFFAGEATCKEHPDTVGGAMMSGLREAVRIIDILNTGNDCTAEVEAMEATQRHSDSERNELRDILKRLEAVEFSNVMYKSSLDGAHILTKEGLLLDMFFNAKTTAGRLQVAKELLNLPVEALKSFAGTKEGLITLNSWILDSMGKDGTQLLRHCVRLLVLVSTDLLAVRLSGIGKTVKEKVCVHTSRDIRAIASQLVSVWIEVFRREKASNGRLKLLRQSIPVDSSKSKSNLASGKAPLCPIYGTSVKKVIVKPVKLETGAEPQPEDPKLTMSEEEYAALAAAEAARAAALAAAEAYASSEVKTNTLLQLPKIPSFHKFARREQYALMDESDFRRKWPGGALGKQDCISEIDSRNCRVRDWSLDFSAACTNIDSSKMSVDNLSQRSHSNIREHSGESVAIDSSILTKAWVDASGSVGIKDYSAIERWQSQAAAAADSDFFDRTEYIRDEEDSNMSSELQTRKLEGLANESSVSQVTVNKEFVGNQPRGADHIKQAVVDYVASLLMPLYKARKIDKEGYKSIMKKTATKVMEQTSDVEKAMAVFEFLDFKRKSKIRAFVDKLIERHMAMKPVAKPS

>Potri.002G027100|Ptrichocarpa_v4.1/1-1932

MESDDLSNCNDLEVKLGLEKGLSQNLLKPRVSKEMEKEEKKFLGKKIEAGIDSDDNEPIGSLFRLKRPRNPKKAKVGLEKVEVREAKDEDLGGMDDTLASFKKKLKGPKKDLGSVSASHDDGLLDVNVEKKEQKCKERARKVRIDGKRVRTGGDVVGDDVLEGLQSQGALLENQGEESWLPGESSNRPLDGKLEDSISAFFQKKQSGLARKSRANSSFKQINRVQCLDDRLSPGSGVGSGGSKDVAARTIGSGSVSSVVCKDLEAENSFHTVADLSLLDSSSRQILHEKNQRLDNGFCETSYFTNENSDRIKGIPATKDETMKSDDKRHGKSSEVTAEVSAPVLPAFSSQDGVMEDEQMQDPCISNTQEEPMVEPCSSDRIWNESRSASGHNDGLETQTLKNGLRLCSVSKASSLNALEQQSKDVSAACISNAEPQISLSSDGREISASSSPNSQNELQDLDSVPKKENVEISDGRLSPVTVISGEVHKSSHTNHNGNSLDYLSINEEANGLSPRSVTPEENESYLEDAVLVPGSDIKDGHLAAVQRAVRKAKKRRLGDMAYEGDADWEILINEQQFLENDHALESDRSLRAREKSDSSSNSVEAENGGIAAVSAGLKARAAGPVEKIKFKEVLKRKGGLQEYLECRNRILCLWSKDISRILPLADCGVTETPSQDESPRASLIRQIYGFLDQSGYINAGIASEKERAEPSTNHNYKLVEKKTFEGNSGASVADLEDGVSFILGQVKSSENSLEPKNGVSVDNQDLASKALKSGELVTPMTPDLPNVMEYEELPAAGIQQNSASNSKLPNGLVSLDPLSTDPSCTMLDGRTVVTSITPELRDDLQSVKSNSCANIGESHKLLCDSEDRKKIIVIGAGPAGLSAARHLQRQGFSAIILEARSRIGGRVYTDRSSLSVPVDLGASIITGVEADVTTERRPDPSSLICAQLGLELTLLNSDCPLYDVVTREKVPTDLDEELESEYNSLLDDMVLVIAQKGQHAMKMSLEDGLNYALKTRRMAYPGPTIDETESGIAVDTLYDSKTCSVDGGAHERSSKEEILSPLERRVMDWHFAHLEYGCAASLKEVSLPYWNQDDVYGGFGGAHCMIKGGYSNVVESLGEGLHIHLNHVVTDISYGVKDAGANESHRSKVKVCTLNGSEFLGDAVLITVPLGCLKAETIKFSPPLPQWKRSSIQRLGFGVLNKVVLEFPVVFWDDSVDYFGATAEETDQRGHCFMFWNVKKTAGAPVLIALVVGKAAIDGQRMSSSDHVSHALMVLRKLFGESLVPDPVASVVTDWGRDPFSYGAYSYVAIGSSGEDYDILGRPVENSVFFAGEATCKEHPDTVGGAMMSGLREAVRIIDILSMGTDYTAEVEAMEGAQRHSEVERDEVRDITKRLEAVELSNVLYKNSLDRARLLTREALLRDMFFSAKTTAGRLHLAKMLLNLPVGTLKSFAGTRKGLTMLNSWILDSMGKDGTQLLRHCVRLLVLVSTDLLAVRLSGIGKTVKEKVCVHTSRDIRAIASQLVSVWLEVFRREKASNGGVKFSRHATLLDSSKRKSFSNSTTGKPPLRTHHGALEARGNSQVSAPTRGPLPSNPNMKKASSKPETLKDPSRQDTEFEEGNTAISEEEQAALAAAEAARAAARAAAQAYASSEAKCSTLVQLPKIPSFHKFARREQYAQMDEYDLRRKWSGGILGKQDCISEIDSRNCRVRDWSVDFSAACANFDSSRMSGDNLSQRSHSNEIACHMNFREQSGESSAVDSSLLTKAWVDTTGSAGIKDYHAIERWQCQAAAADSDFFHRAMRIKDEEDSNTSSRPPTRKHDRRANESSISQDTINKEPSKHRSRGPDRIKQAVVDFVSSLLMPVYKARKIDKEGYKSIMKKSATKVMEKATDAEKAMAVSEFLDFKRKNKIRAFVDKLIENHMAMKPAVEP

>Potri.005G235400|Ptrichocarpa_v4.1/1-1934

MESDDLSNCKDLEAKLGSQKGLSKNLLKPRVNKGMEEQKELLGKQIEFGIDSDDNEPIGSLFRLKRPRNPKKVKVVLEKIEVREDKLVTEDEDLGGMDDTLASFKKKLKAPKKGLGSVSAIQNEGDELLDGNVEKKVQNKRKERASKVGSGWKRVRTGGDAAVDDDSEGLGSQGALLENQEEESLLPGESSSQSLDKLEDSISAFYQKKQSGSVRKSCANSSSKQINRVQCLEARLSPETGFGSGGSKDVDLSTFRSSPVSSVVCKDLEGGDSSHIVANSSLLDSTSRQILNTKNQRLDNGFGETSYCIEENSDRIKGLSVSKDESMKSNDKRHGKSSEVTAEVAAPASPALGSQHGVIEDEETQDPCISDFKGEPMGKPCSPYRIWNESHSAPGNNDGLEAQTLKNGLKLCSVGKVSTRNTLEQQSKGVSAACISNAEPQISISSGGREVSASSSPHSQNDLQDLASVPKKENVEISDVRLSPFTVTSREVHKCTFSLCMNHNGNSLDYLSINEEANGPSPRSLTPEENESYPEDAVSVPDSDIKDGHLAAVHRAMRKPKKRRLGDMAYEGDADWETLINEQQFLENYQVVESDRSFRTREKSDSSSNSAEAENGGIAAVSAGLKARAAGPVEKIKFKEVLKRKGGLQEYLECRNRILGLWSKDVSRILPLADCGITETPSQNESPRASLIRQIYEFLDQSGYINAGIASEKESAEPSANHNYKLVEEKTFEGNPGASVADLEDGVSFILGQVKSSQNSLEPKDRVPMDNQDLALKALKSGKLVDLPNVKECEEWPAEDIKQNSVSNTKLSNGLASLDALSTDPSCTMLDSRTAPVINPELRNGLQSVKSNSCAEMGGSHKLLCDSQDRKKIIVIGAGPAGLTAARHLQRQGFSVTILEARSRIGGRVYTDHSSLSVPVDLGASIITGVEADVTTERRPDPSSLICAQLGLELTVLNSDCPLYDIVTGEKVPTDLDEELEAEYNSLLDDMVLVIAQKGQHAMKMSLEDGLNYALKTRRMVHPGAFFDETESGNAVDALYDSKTCSVDGGAPENSKEEILSPLERRVMDWHFAHLEYGCAASLKEVSLPYWNQDDVYGGFGGAHCMIKGGYSNVVESLGERLPIHLNHVVTDISYGIKDARASVSHRSKVKVCTSNGSEFLGDAVLITVPLGCLKAEAIKFSPPLPQWKRSSIQRLGFGVLNKVVLEFPDVFWDDSMDYFGATAEETDRRGHCFMFWNVKKTVGAPVLIALVAGKAAIDGQRMSSSDHVSHALMVLRKLFGEALVPDPVASVVTDWGRDPFSYGAYSYVAIGSSGEDYDILGRPVENCVFFAGEATCKEHPDTVGGAMMSGLREAVRIIDILSMGTDFTTEVEAMEGAQRHSEVERDEVRDITKRLEAVELSNVLYKNSLDRARLLTREALLRDMFFSAKTIAGRLHLAKKLLNLPVGTLKSFAGTRKGLAMLNSWILDSMGKDGTQLLRHCVRLLVLVSTDLLAVRLSGIGKTVKEKVCVHTSRDIRAIASQLVSVWLEVFRREKASNGGVKLSRHATALESSKRKSFNNSTSRKPPLHAHHGALENSGNLQVSTSTRGPLPSNSNMEKAKSKPETLKCSSRLGIEVEEGNTIAISEEEQAALAAEEAARAAAHVAAQAYASSEAKFSTLVQLPKIPSFHKFARREQYAQMDEYDLRRKWSGGVLGKQDCISETDSRNCRVRDWSVDFSAAYANFDSSRMSGDNLSQRSHSNEIASHMSFREQSGESTAVDSSLFTKAWVDTAGSAGIKGYHAIERWQCQAAAADSDFFHRAMHIKDEEDSNTSSRPPTWKHDGRANESSISQVTVNNEPSKHHSRGADRIKQAVVDFVSSLLMPVYKARKIDKEGYKSIMKKISTKVMEKATDIEKAMAVSEFLDSKRKNKIRAFVDKLIENHMAMKPAVEP

>Gohir.A05G373900|Ghirsutum_v3.1/1-1807

MESDDGSNSKAGPQLQFDLGNNVELGLEKVTLQNSLKLRNKKMDGGGGSKKRLKVTAVEVDVDSDDDEPILSLLKLRKPKNPKKDKAGLEGSAGKCKKVEVKAVKTEGKNEEDLGGMNDTLASFRKKLKDPKKDVDPGAKRERDYSLNKSVEGGVVLDGKSVSNTGVKGQDIGEDRSDAVTDTVFERKHTGKVRRAKFDSKSKLIEVDDESRAKLEEDQNEGGLSPEGGLNQHSHEAQSDSVRKSCPITSLKHNCKGSHHASASKNPSRNYGDRSHSDSSSSFSHSFSKECNTAENQGFGHSVCQQESILEPGDLNVQKGPSGDPCRSPKVCDKEKYGHSNIELRDNCSAVDQRNKPESGGSRQTKHNLLPSVVDSLKMEDTCTDVPNACAEENSLEDSVHPNEFVASIQRCNSALRQPSEDACHGACGPSHDTLFISKEANVDSPTSTPDENESFHEDAISLPSSEIKDSMSSAVQRGGRSIKKRRHGDMAYEGDADWENLLNEQGFFGNQQFADSDRSFRAKEKFDEAAVSSGLKARAVGPVEKIKFKEVLKGRGGLQEYLECRNHILGLWSKDVNRILPLAECGVTDTPSEGEPPRASLIREIYAFLDQGGYINFGIASKKEKAELRVKDNRKLLKERKNYGNSVASVADSEDGVAFILGQVKNSEASMDAKISVRVDDENQASEATIPEVLVDSITSELPCRKEQKEHPSDNCQQNGSISAKLNPLLISSQVPSADLSCDAIDMGIAPVVTPEERNDSHYVQSATYDKPDGNHQLQGDSEVRKNIIIVGAGPAGLTAARHLKRQGFSVVVLEARDRIGGRVYTDCSSLSVPVDLGASIITGVEADVSTNRRPDPSSLICAQLGLELTVLNSSCPLYDIVSGQKVPADLDDALEAEYNSLLDDMVFLVAQKGQKAMTISLEDGLEFALKRHRMEEIGADIEEIESHSSVDAVYDLKASNGKKCSEGEILSPLERRVMNWHYAHLEYGCAAPLKEVSLPNWNQDDVYGGFGGAHCMIKGGYSKVVESLGEGLLIHLSHVVTNISYGPKDPGIDNSHHRQVKVSTSNGSEFSGDAVLITVPLGCLKAGAIKFSPPLPQWKHSSIQQLGFGVLNKVVLEFPEVFWDDTVDYFGVTAEETDSRGHCFMFWNVRKTVGAPVLIALVAGKAAIDGQTMSSSDHVNHAVLILRKLFGEASVPDPVASVVTDWGRDPFSYGAYSYVAIGASGEDYDMLGRPVENCLFFAGEATCKEHPDTVGGAMLSGLREAVRLIDIFTTGNDYTAEVEAMEGAQRRSESGRDEVRDIIKRLEAVELSNVLYKNSLDRAWVLSREALLQDMFFNVKTTSGRLHLAKKLLGLPVESLKSFAGTKEGLSTLNSWMLDSMGKDGTQLLRHCVRLLVLVSTDLLAVRSSGIGKTVKEKICVHTSRDIRAIASQLVSVWLEVFRKAKASSKRKPLKDTASGKPPLHSQHCAFESKASLQDPFPAGKQYPFYAKENGKSVDMEVESVNQGMSEEEQAAFAAEAAARAAAKAAAEALASTEANCNKLLQLPKIPSFHKFARREQYAQMDEGKWPGGVLGRQDCISEIDSRNCRVRDWSVDFSAACVNLDSSRMSVDNLSQRSHLKLREHSGESLAVDSSIFTKAWVENAGSEGIKDCHAIERWQSQAAAADPDFFHPTNFKDEEDSNASSRQTTWKHDGRANESSISQVSVNKERFENHPHGTDRIKQAVVDYVASLLMPLYKARKIDKEGYKSIMKKTATKVMEQATDAEKNMAVSEFLDFKRKNKIRPFVDKLIERHMAMKPIMKP

>Gohir.D04G041600|Ghirsutum_v3.1/1-1807

MESDDGSNSKAGPQLQFGLGNNVELGLEKVTLQNSLKLRNKKMDGGGGSKKRLKVTAVEVDVDSDDDEPILSLLKLRKSKNPKKDKAGLEGSAGKCKKVEVKAVKTEGKNEEDLGGMNDTLASFRKKLKDPKKDVDLGAKRERDYSLNKSVEGGGVLDGKSVLNTGVKGQDIGEDRSDVVTDTVVERKHTGKVRRAKFDSKSKPIEVDDESRAKLEEDQNEGGLSPESGLNQHSHEAQSDSVRKSCPISSLKRNCKGSYHASASKNPSRNYGDRSHSDSSSSFSHSSSKECNTAENQGFDHSVCQQESILEPVDLNVEKGPTEDPCRSPKVCDKEKYGHSNIELRDNCSAVDQRNKPESEGSPQTKHNLLPSVVDSLKMEETCTDVPNACAEENSLENSVHPNEFVASIQRCNSALRQPSEDACHGACGPTHDTLFISKEANVDSPTSTPDENESFHEDAVSLPSSEIKDSMSSAVQRGGRSIKKRRHGDMAYEGDADWENLLNEQGFFGNQQFADSDRSFRAKEKFDEAAVSSGLKARAVGPVEKIKFKEVLKGRGGLQEYLECRNHILGLWSKDVNRILPLAECGVSDTPSEGEPPRASLIREIYAFLDQGGYINFGIASKKEKAELRVKDNHKLLKERKNYGNSVASVADSEDGVAFILGQVKNSEASLDAKVGVRVDDENQASEATIPEVLVDSITSELPCGKEQKEHPSDNCQQNGSISAKLNPLLISSQVPSADLSCDAIDMGIAPVITPEERNDSHYVQSATYDKPDGNHQLQGDAEVRKNIIIVGAGPAGLTAARHLKRQGFSVVVLEARDRIGGRVYTDCFSLSVPVDLGASIITGVEADVSTNRRPDPSSLICAQLGLELTVLNSSCPLYDIVSGQKVPADLDDALEAEYNSLLDDMVFLVAQKGQKAMTISLEDGLEYALKTHRMEEIGADIEEIESHSSVEAVYDLKASNGKKCSEGEILSPLERRVMNWHYAHLEYGCAAPLKEVSLPNWNQDDVYGGFGGAHCMIKGGYSKVVESLGEGLLIHLNHVVSNISYGPKDPGIDNSHHRQVKVSTSNGSEFSGDAVLITVPLGCLKAGAIKFSPPLPQWKHSSIQQLGFGVLNKVVLEFPEVFWDDTVDYFGVTAEETDSRGHCFMFWNVQKTVGAPVLIALVAGKAAIDGQTMSSSDHVNHAVLILRKLFGEASVPDPVASVVTDWGRDPFSYGAYSYVAIGASGEDYDMLGRPVENCLFFAGEATCKEHPDTVGGAMLSGLREAVRLIDIFTTGNDYTAEVEAMEAAQRRSESGRDEVRDIIKRLEAVELSNVLYKNSLDRAWVLSREALLRDMFFNVKTTSGRLHLAKKLLGLPVESLKSFAGTKEGLSTLNSWMLDSMGKDGTQLLRHCVRLLVLVSTDLLAVRSSGIGKTVKEKICVHTSRDIRAIASQLVSVWLEVFRKAKASSKRKSLKDTASGKPPLHSQHGAFESKASLQDPFPAGKQYPFYAKENGKSVDMEVEYVNQGMSEEEQAAFAAEAAARAAAKAAAEALASTEANCNKLLQLPKIPSFHKFARREQYAQMDEGKWPGGVLGRQDCISEIDSRNCRVRDWSVDFSAACVNLDSSRMSVDNLSQRSHLKLREHSGESLAVDSSIFTKAWVDNAGSEGIKDCHAIERWQSQAVAADPDFFHPTNFKDEEDSNASSRQTTWKHDGRANESSISQVSVNKERFENHPHGTDRIKQAVVDYVASLLMPLYKARKIDKEGYKSIMKKTATKVMEQATDAEKNMAVSEFLDFKRKNKIRPFVDKLIERHMAMKPIMKP

>Gohir.D02G097300|Ghirsutum_v3.1/1-1767

MDGGGGSKKRLKVTAVEVGVDSDDDEPILSLLKLRRPKNPKKDKAGLEGSAGKCQKVEVKAGKTVDKDEEDFGGMNDTLASFRKKLKDPKKDIDPGAMRVRSNSLNKSVEGGGILDGKSVSNTDVKGQDIGEDRSDVATDKVVGKKRTGKVRRTKSDSKAKPSEVDDESRAKLEEDQNEGGLSPGEGLYQCSHKAQSGSVGKSCPIFCLKHNCEAAHHASDSKNPSRNCGDSSHSVSSSSFSHSSSKECNTAENQGFDHSLYQQESILEPGDLTVQKDPTEYPCRSSKVCDKERYCHSNIELRDNFSAIDLRSKPGTESSQQNKLNLSLSVVDSLKMEETCTDVPNSCAEEYSLETSIHPNELVASIQRCNSALHQPSEDASPGACGPIHDTVFISKKANVDSPISTPDENESCHEDAVSLPSSELKNSKSSAFQRGGRNIKKRRHGDMAYEGDADWENLLTEQGFFGNQQFADSDHSFRAREKFDEAAISSGLKACAVGPVEKIKFKEVLKCRGGLQEYLECRNHILGLWSKDVNRILPLVDCGVSDTPSEGEPSRASLIREIYAFLDQGGYINFGISSKKEKAELSVKDNYKLLEGRKSDGNSVASVADSEDGVAFILGQVKNSKASMDAKTGLRVVDENQASEATIAIAEVLVDSITPELPYKCQQNGSFSAKLNTGLISSQVSSTDLSCDATDVGVAPVVTPEERNDSQYVQSATYDKPDGNHQLLNDSEVRKNIIVIGAGPAGLTAARHLKRQGFSVVVLEARDRIGGRVYTDCSSLSVPVDLGASIITGVEADVSTNRRPDPSSLICAQLGLELTVLNSSCPLYDIVSGQKVPADLDDALEAEYNSLLDDMVFLVAQKGKKAMTMSLEDGLEYALKRHRMEEIGADIEETESHSSVDAFYDSKASNIFGFPRKKCSEEEILSPLERRVMNWHYAHLEYGCAASLKEVSLPNWNQDDVYGGFGGAHCMIKGGYSTVVESLGEGLLIHLNHVVTNISYSPKGPGVDNSHHRQVKVSTSNGSEFSGDAVLITVPLGCLKAGAIKFSPPLPQWKHSSIQQLGFGVLNKVVLEFPEVFWDDTVDYFGVTAEETDSRGHCFMFWNVRKTVGAPVLIALVAGKAAIDGQNISASDHVNHAVIILRKLFGEASVPDPVASVVTDWGRDPFSYGAYSYVAIGASGEDYDMLARPVENCLFFAGEATCKEHPDTVGGAMLSGLREAVRLIDIFTTGNDYTAEVEAMEAAQRHSESGRDEVRDIIKRLEAVELSNVLYKNSLDRARILSREALLRDMFFNVKTTAGRLHLAKKLLGLPVESLKSFAGTKEGLSTLNSWMLDSMGKDGTQLLRHCVRLLVLVSTDLLAVRSSGIGKTVKEKICVHTSRDIRAIASQLVNVWLEVFRKAKASSKRKSLKDPASGKPPLHSQHGAFESKESLQDPFSAGKQYPLNIKENGKSLDIEVEAVNQGMSEEEQAAFAAEAAARAAAKAAAEALASTEANCNKLLQLPKIPSFHKFARREQYAQMDEGKWPGGVFGRQDCISEIDSRNCRVRDWSVDFSAACVNLDNSGMSVDNLSQRSHLKLREHSGESLAVDSSIFMKAWVDTAGNGGIKDYHAIERWQSQAAAADPDFFHPTNFKDEEDSNTSSRQPTWKNDGRANESSVSQVSVNKERFENHPHGADCIKQAVVDYVASLLMPLYKARKIDKEGYKSIMKKTATKVMEQATDAEKNMAVFEFLDFKRKNKIRPFVDKLIERHMAMKPTMNYDVVKD

>Gohir.A03G080700|Ghirsutum_v3.1/1-1762

MDGGGGSKKRLKVTAAEVGVDSDDDEPILSLLKLRKPKNPKKDKAGLEGSAGKCQKVEVKAGKTVDKDEEDFGGMNDTLASFRKKLKDPKKDIDPGAMRVRSYSLNKSVEGGGILDGKSVSNTDVKGQDIGEDRSDVANDKVVGKKRTGKVRRTKSDSKATPSEVDDESGAKLEEDQNEGGLLPGEGSYQCSHKAQSGSVGKSCAILSLKHNCEAAHHASDSKNPSRNYGDSSHSVSSSSFSHSSSKECNTAENQGFDHSLCQQESILEPGDVTVQKDPTEHPCRSSKFCDKERYCHSNIELRDNFSAIDQRSRPGSESSQQNKHNLSLSVVDSLNMEETCTDVPNSCAEEYSLETSIHPNELVASIQRCNSALDQPSEDASHGACGPSHDTVFISKEANVDSPISTPDENESFHEDAVSLPSSEIRNSKSSAVQRGGRNIKKRRHGDMAYEGDADWENLLTEQGFFGNQQFVDSDHSFRAREKFDEAAVSSGLKARAVGPVEKIKFKEVLKCRGGLQEYLECRNHILGLWSKDVNRILPLVDCGVSDTPSEGEPSRASLIRKIYAFLDQGGYINFGISSKKEKAELSVKDNYKLLEGRRSDGNSVASVADSEDGVAFILGQVKNSKASMDAKTGVRVVDENQASEATIAIAEVLVDSITPKLPYICQQNSSVSAKLNTGLISSQVSSTDLSCDAIDVGVAPVVTPEERNDSQYVQSATYDKPDGNHQLLNDSEVRKNIIVIGAGPAGLTAARHLKRQGFSVVVLEARDRIGGRVYTDCSSLSVPVDLGASIITGVEADVSTNRRPDPSSLICAQLGLELTVLNSSCPLYDIVSGQKVPADLDDALEAEYNSLLDDMVFLFAQKGKKAMTISLEDGLEYALKRHRMEEIGVDIEETESHSSVDAFYDSKESNIFGFPGKKRSEEEILSPLERRVMNWHYAHLEYGCAASLKEVSLPNWNQDDVYGGFGGAHCMIKGGYSTVVESLGEGLLIHLNHVVTNISYSPKGPGVDNSHHRQVKVSTSNGSEFSGDAVLITVPLGCLKAGAIKFSPLLPQWKHSSIQQLGFGVLNKVVLEFPEVFWDDTVDYFGVTAEETDSRGHCFMFWNVRKTVGAPVLIALVAGKAAIDGQNMSASDHVNHAVIILRKLFGEASVPDPVASVVTDWGRDPFSYGAYSYVAIGASGEDYDMLARPVENCLFFAGEATCKEHPDTVGGAMLSGLREAVRLIDIFTTGNDYTAEVEAMEAAQRHSESGRDEVRDIIKRLEAVELSNVLYKNSLDRARVLSREALLRDMFFNVKTTAGRLHLAKKLLGLPVESLKSFAGTKEGLSTLNSWMLDSMGKDGTQLLRHCVRLLVLVSTDLLAVRSSGIGKTVKEKICVHTSRDIRAIASQLVNVWLEVFRKAKVSSKRKSLKDPASGKPPLHSQHGAFESKESLQDPFSAGKQYPLNIKENGKSLDIEVEAVDQGMSEEEQAAFAAEAAARAAAKAAAEALASTEANCNKLLQLPKIPSFHKFARREQYAQMDEGKWPGSVFGRQDCISEIDSRNCRVRDWSVDFSAACVNLDNSGMSVDNLSQRSHLKLREHSGESLAVDSSIFTKAWIDTAGNGGIKDYHAIERWQSQAAAADPDFFHPTNFKDEEDSNTSSRQPTWKNDGRANESSVSQVSVNKERFENHPCGADRIKQAVVDYVASLLMPLYKARKIDKEGYKSIMKKTATKVMEQATDAEKNMAVFEFLDFKRKNKIRPFVDKLIERHMAMKPTMNL

>TESI_003507-RA|Tetracentron_sinense/1-2024

MAKKAQVHAKSDNGLDRPSVVQRNKQKEGLLGLVESSNNSSDVNLEDSLSAFVRKSRFGLVGNARSSSVSKQRVGTQASEDGLSPSSDSKDVRKSCSASVSKLARKIKKSENGLSLVSGRSPIDQTHVQKETVGKQRFDGGASRASKGIQESVLPATVQELDSLPVLTARNLGFSSNRYYRSSEGTLEDSVPLFQPPRSHSVSVTDPEQMYTALHDDEFDRQTRGIPEGPAVYRDGLNQVLDGASKDFLYVQVQEPCSTSVQKHAEETRKFKDGLKQCSKQKASDLVQDIVDLSSSISAPKQVDTNNGFDNGELNQLPILISHNLPSSILSTGEANPKPMYRVDENSDVPRTIKEAIGSTDALDQPSVCVPANLWLNRPCEETFKESIVDHGDSLNQTSDNTSKELPGHGEKLSAVYYKSQKKESIRNDELAVAFFGAIVEDSLTSPIRNSSMSLVKMEAAPKPEGGFNPFSDGSLEDPLLVQPSYCASGSTKKGEICSNHDDPDQTPNTYIEGPHLASMQMKETPKSDDRICPVILTLEVVKKSDFACPLNHQEKSSATGDASNRFSGLLLNELVNSIQKSHSALPQNQHSHDASNETYVPSQDPVSVKEEAKGACSASITPEGNEIYPEDAGMIPDLQDKDNKLSMAQRAARTIKKRRHGDMAYEGDTDWDILMHEQGFFENNCVVDEERPFITKDNSDLLSNLVAGAGNGGAAAVAAGLKARAVGPVERIKFKEILKRRGGLQEYLECRNLILGLWSKDVSRILPLVECGVTDALSKDELPRTTLIREIYAFLDRSGYINVGIASEKEKAEPFPKAHYKLSKEKKPEENCGARVADSEDGVAFILGQVKSSEMLEEAKNVVFFDDGKLIPEVKKGRMLVTSSTGSELSTLIESEECTADNGQENGSNDAKLPNRMVNMDVLSTDPSSEIVDGGTDSRDDYMHCDSKVRKRVIVIGAGPAGLTAARHLQRQGFSVTVLEARNRIGGRVYTDHSSLSVPVDLGASIITGVEADVATERRPDPSSLICAQLGLELTVLNSDCPLYDIVTGQKVPADLDEALEAEFNSLLDDMVVLVAQKGELAMRMSLEDGLEYALERRRMAQLITDLEEIELHTPVDASFGSGKNGVDNQIPDRNSSKDEILSPLERRVMDWHFANLEYGCAALLKAVSLPYWNQDDVYGGFGGAHCMIKGGYGTVIESLGRGLNIHLNHAVTEVSYCARDSGETDERWSKVKVSTSNGYEFVGDALLITLPLGCLKANAIKFSPALPEWKRSSIQRLGFGVLNKVVLEFPEVFWDDSVDYFGATAEETEQRGECFMFWNVKKTVGAPVLIALVVGKAAMDGQNISSSDHVNHAIMVLRKLFGDTLVPEPVASVVTNWGKDPFSLGAYSYVAVGASGEDYDILATCKEHPDTVGGAMMSGLREAVRITDILSTGNDYTAEVEAMEAAQRQSDSERNEVRDILKRLEAVELSNFLCKNSLDGAQMLTKEALLQDMFSNAKTTAGRLHLAKELLNLSVEVLKSFAGTKDGLSTLNSWILDSMGKDGTQLLRHCVRLLVLVSTDLLAVRVSGIGKTVKEKVCVHTSRDIRAVASQLVSMWIEVFRKEKAANGGLKLLRQTTALDSSKTKSSKDLSSRRPPLRTPQGTPDNKGTVQVPSSAGSHSSSNEDNKKVNCKLVKFEAVTVSKSEIYSSRTQDVGCIRESKVEENNVAMSEEEAAAFAAAEAARAAALAAAKAYASSEAECGQLRELPKIPSFHKFARREQYAQMDDSDLRRRKWPGGVLGRQDCISEIDSRISRIKNWSVDFSATCVNLDSSRMSGDNYTQRSYSNEVAYQLNLREHSGESGAVDDRFTKAWVDTAGSEDVKDYHAVDYHAIERWQSQAEAADSDFFHPIMHTRDEEDSNTISELPTCKPEGQAEDSSNSQAVENKELVENQPRGAEHIKQAVVDYVASLLMPLYKARKIDKEGYKSIMKKSATKVMEQTTDAEKTMVVSEFLDFKRRNKIRAFVDKLIERHMSMKPAVKL

>TESI_010201-RA|Tetracentron_sinense/1-2282

MNYELYLSGKREMESEGFGSKLVECLHVGSDINGEVGLGRDLLSSKPRAVSKPRADDAKMDEEENKSGSKRRSKPIEVRVDPDDDEPIGYLLKLKRPRNPKKAKSGLDGDEVEKVEIRAEEFIVEDQDSGEMDDTLASFRKKLRGPKKDGGPGVVKGWNSAMNAVEQWDQSSDRPLKDGVLVAKLHSKLVKEISTAGEDGSDLPMDGVLEDRPRRRGRRSKLALMTKKTRFHAKSDDGLDRPSVQRSRGTTTHKQKEEVLGLVESFNNSSDENIEDSLFAFVRKSRFGSVRKARSSSIFKQRVGTQASEDGLSATSDGVSKDVRRCRSSYASKLARQNPKSELGSSLVSGMNPIDPAHVRKEKVENQRSDGGLLSGPVQELASVPVLTEEILRSSDNRYDISSERIPGDSVLVSPPPRSHCGSVTDPEEKTITLHVDEFDRQTQGIPKGPAANCYGLNRVRDVAFEGFLYEQVQNPCSASIQKLAEETQMSNDGLKQCSKWKTSDLVHDVVEISSLISDPKQMEAIHGFDNGGLNRLSVMISDDLPSSVLSTGEANPKPTNGLDEISDVQKTTKEATRSIDALDQSSVAVPAIYPSIAVRKPCSDPSPMPIKEILVPCFDDDRLKRLCDETFKESTMDRWDSLNQRPNSMSKELSGHVGKLSAVPCKSQNRDSARYDELLHPSGENVEDSLTSPIQDSFLLPAKMEEALKPEGGLYLFSDGSPEDSGFIQLQSSGSVKKDETCSKNDGFIQTPGTYVEESHCASVSMQTKETSRSDDRMCPVILTSDVVKKSDFASHVNYQEKSSSTGDVTNQFSEGLLLNDLVNSVQKCHSACPQNQHSDDASKGAYVPSQDFLSVNEEAKGFCSPSIIPYHDEIYPEDAGIVPDPESKDNKLSATQWAVQKVKKRKHFDMAYEGNTDGEILMREQVFFENNHAVDGEWPSITNDKSCLPSNLVVGADNGGSAAVAAGLKARAAGPVEKIKFKEVLKRRGGLQEYLECRNLILGLWNKDISRILPLVDCGVTDAPFKDEPPRASLIREIYAFLDQSGYINVGIASEREKAELRSKANYKLTKEKKPEENCGVRVADSEDGVAFILGQVKSSENFPEANNDVFINDGKLILEATKGRMLVTSSTGSELSTLIEFEECTADNGQENDFVDAKLPNRLVNLDALSTDPSSETVSGGTDSILAVDPVERNDYMHCDSKVRKKIIVIGAGPAGLTAARHLQRQGFSVTVLEARNRIGGRVYTDRSSLSVPVDLGASIITGIEADVATERRPDPSSLVCAQLGLELTVLNSECPLYDIVTGQKISADLDETLEAEYNRLLDDMVLLVAQKGERAMRMSLEDGLEYALKTRRMAQSATTGEEIELHTLVDASFDFEKNVVHKKIPNRSACSKEEILSPLERRVMDWHFANLEYGCAALLKAVSLPYWNQDDVYGGFGGAHCMIKGGYSTVVESLGKGLSIHFNHVVTEVSYSTRDSGETDEWWSKVKVSMSNGSELVGDALLVTVPLGCLKADTIKFSPALPEWKRSSIQQLGFGVLNKVVLEFPEVFWDDSVDYFGATAEETERRGQCFMFWNVKKTVGAPVLIALVVGKAAIDCQNMSSSDHVDHAVMVLRKLFGEALVPEPVASVVTNWGMDPFSRGAYSYVAVGASGEDYDILSRPVENCIFFAGEATCKEHPDTVGGAMMSGLREAVRIIDILNTGNDYTAEVEAMEAAQRQSDSERNEVRDILKRLDAVELSNVLYKNSLDGTHMLTKEALLQDMFSNAKTTAGRLHLAKKLLNLPVEALKSFAGTKEGLTILNSWILDSMGKDGTQLLRHCVRLLVLVSTNLLAVRLSGIGKTVKEKVCVHTSRDIRAVASQLVGMWIEVFRKEKAANGGLKLLRQTAALDSSKTKSSKDLSSRKPPLPTPQVAPENKETLQVPSSAGIHLPSNANNKKVNCMPVNLETITDSKPINFSRSKGFILTQDFKAEDSNVVISEEETTAFAAAEAARASALSAAEAYASSEAECSTLRELPKIPSFHKFARREQYAQIEDSDLRRRKWTGGVLGREDCISEIDSRNCKVRNWSVDFSATCVNLDSSRMSGDNYSNEVGYQLNLREHSGESGAVDSTFSKAWVDTAGSGGVKDYHAIERWQSQAAAADSDFYHPTVHKRDEEVSNTISELFAWKHERQEEDSSASQAVENKELVGNQPRGAEHIKQAVVDYLALLLMPLYKARKIDKDGYKSIMKKSATKVMEQTTDAEKAMAVSEFLDFKRRNRIRAFVDKLIERHMPMKSVVK

>GWHPBAUK029043.1|Macadamia_integrifolia/1-2244

MNDEGYLSFREGISDEELGSKSSEGLHVASEFNNMLGLQGESLSSRARAGDAKMSEEGKNSSSKRRSKIVEVRVHSNDDDEPIGSLLTLKRGRNPKRAKPVLNSGDVEPVQVRAEYSMDGDEDLGEMDDTLASFRKKLKGPKMGKDGESGRVKGTDSALSAGVMKGRYSHKPLKDGAMEAKSHENPVEQSPAIGVDGLSPLLNGELESRPKGRGKSSRVASIPKKTEDFNGGLGQHHRLPRSRGTLIQGKKENEDSSHSSDENLEVSLSAFVRKSQPGSVRRARSSSVRKHREESAASGDALIRGSEVSEDVLSMITQRPRSNSSSKSARKIHKIEGRIDMVSGVSQIDPVVQKESLETQRSDADTIMPFKVMQGNSLACPSQELASAPEIDIVRFNDIEYNKTPDGIPKDQGCQSVAHIKEKASTGRGLECYRENLDNSNQLSCGVSGDFPCGNVQESYAASVQKLTEETWSLQGRLKQCSQGKSGASGHDVTEKSSIESDSKLMKKSGVLVADVSNVPEDLPSSIASSSAGVSLKFDGAFDGTSDVLKTAEESAGSDNEYDQSAVGVPKICHSSAIPEPCSLSLPTPVKEILVSCSTDDQLNRSLEETCKGISVDHSNCLNQPCGNNSNEPSNHVEKLSSAHCKTQNGETKRCDEPFWPLSEKKLEEILKPQDRLGQFPDGFSKGQLVQLHPSSSASGSTRKEGTYGHDDVPNQSLDTFADYHHDSSSTRTEETSVPDCGLFSVSLLSDIVRKSDFSLAVNQEEAPDNVNDPRLFSGGPMIGSDTCFQQHSQPSDDALKAINIPNYDPVYEKEEAKGTCSPSLPMHPEEICPEDAEVEPDPENEDKKLSMMQRALRKIKKRRHGDMAYEGDNDWKVLMHEHGFLENNYILDGDRPSRVKDKSFSPSFMVSEADKGLAAVAAGLKARIAGPVEKIKFREVLKRRGGLQEYLECRNLILGLWSKDTSRILPLVECGVTDVSSKDETPSASLIREIYSFLDQSGYINVGVASEKDKAEPHAKAHYRLSEEKIRNERVGAGVADSEDGVAFILGQLKCSENLREMKNNVLFDDAKLISEATEDGKQVLGPELSTLESDECLVDNGNADGCVDALSIDPSSDIVDGGSDSILAAEHVRDSSGVESAVIHPVEGNDSMQCESEARKRIIVVGAGPAGLTAARHLQRQGFSVTVIEARNRMGGRVYTDHSSLSVPVDLGASIITGVEADVATERRPDPSSLVCAQLGLELTVLNSDCPLYDFVTGQKVPADLDEALEAEFNSLLDDMLVLVAQKGELAMRMSLEDGLEYALKRRLMVRSVSDAEKFELHTSVNASSETGTYSLTNEDLDKTKSKDEILSPLERRVMDWHFANLEYGCAASLKAVSLPYWNQDDVYGGFGGAHCMIKGGYSTVVESLGKGLNVLLNHAVTEIFYGTGDSRENDENWSKVKVSTSNGGEFVGDAVLITVPLGCLKANTIKFSPALPDWKESSIQRLGFGVLNKVVLEFPEVFWDDTVDYFGATAEETDRRGQCFMFWNVKKTVGAPVLIALVVGKAALDGQNLSSSDHVNNAVMILRKLFGESSVPDPVATVVTNWGKDPFSRGAYSYVAVGSSGEDYDILGRPVENRLFFAGEATCKEHPDTVGGAMMSGLREAVRIIDILSTGNDYAAEVEAMEAALRQSDGERNEVRDLLRRLDAVELSGVLYKSSLDGEQMLTKEALLKDMFFNAKTTAGRLHLAKELLNLPVEVQKSIAGTKEGLSTLNSWILDSMGKDGTQLLRHCVRLLVLVSTDLLAVRLSGIGRTVKEKVCVHTSRDIRAVASQLVSMWIEVFRKEKASNGGLKLLRQATTMDSSKAKSLKDFSSGKPPLRTTHGTPDNKGAIQVPSSGGSHSPSNVNSKNNKKPSCKPSKLETITDSKSEVSSRSEDAVQGLDFKVDDSGAAMSEEEAAAFAAAESARAAAMAAAKAYASSEAECSTLPELPKIPSFHKFARREQYAQMDDSDIRRKKWPGDVLGRQDCISEIDSRNCKVRNWSVDFFASCANLDSMKMSVDDYTQRSFSNEVAYQLNLREHSGESVAVDGRFSKAWVDSAGSGGVKDYHAIERWRSQAAAADSNFYHSTEHLRDEEDSNTGMKLHSGTHERRAEESSASQLSESKLLGENQRRGVERIKQAVVDYVASLLMPLYKARKIDKEGYKSMMKKSATKVMEQTTDAEKAMTVPEFLDFKRRNKIRSFVDKLIEKHMAMNSVAKS

>GWHPBAUK007913.1|Macadamia_integrifolia/1-2223

MNDEGNLSSKEGMIGEGLCSESAEGLHVVSDFSGALRLEGESLSSIARAGDNNMDGEGSKSGSKRRSKSIEVRVDSDDDDEPIGSLFKQKRPRNPKRAKPGLNGGEVGQVEVRAEKSMVEGVDFGEMDDTLANFRKKLKGPKMGKNGGSVRVRGKDSVLRVGVKLGQSSLKPLKDESVEAKSLEKLVEQSLTTGFDDLDLPLNEGLENRSKSHGKRSRVVSIPKKDRVKNQDEGSTHSCDGNVEVSLSAFVRKSQSASVRKARSSSISKHREETKASGDDLIQGPELSEGVLSMITRRSRSNSSTKSTRKMAKDDSGIDMVSMSSQIDPVVQKETLEIQRSGACTSVSNESMQEDSSSCLAQELASAPEIDIVRCSDIKYYTSPPECQSVKHVEEKPLTVHYLEGDRETPGKHDKSKQLSCGASEECSNVQESCTASLQNLMEETQRLQGALKQCSEGKHGAWVHDIVGKSSIESNSKLMEKNSVFDAERSNIPEDLPSSVASRSAELSLKLDAALDGTSDVLKTTQKAAGSDDEYDQLAGGVPEICHSSAVQEACSLSLQTPVKETIVSCSTDDRLKRSLEETCKENTMEHSNCLNQLSGSTFNELSDRSEKLSSAPCKTQNGEMMRSTELLQPPSENLLSSLRKLGDIPKPQYRPEEFPDEFSKDQLVQLHPSSSASGSMRKEGTHRHYDAPNQALYTSVDCCHDSISMRTEETSRSDCGLGPFSLLLDVVTKSDSSLAVNHKEEMPATINPFSDGPMVGSDTCVEKKTQHLDDALKVINVPNKDSLCVKEEAKGTCSASMHPHEIYLDDAEVAPVPKNKDKKLTLQQRAIRKIKKRRHRDMTYEGDNDWQVLMCEQGFFENDRVLDGDQPSRIKDKSSSPSLNVSEADSGVAVVAAGLKARAAGPVEKIKFREVLRRRGGLQEYLECRNLILGLWSKDMSRILPLIDCGVTDVPSKDETPRASLIREIYSFLDQNGYINVGIASEKGKADPHAKARYKLSEEKIPDERLGAQVADSEDGVAFILGQLKCSENLSELKNNVLLDDAKLISEATDIGKPHVPTMGHELSNLESEEHLIDNDNANGCVDAVSFDTSCDIVDGGPDSILATELLRDSSGVKSAVIHPVEVNDGMQCDSESCKRIIVVGAGPAGLTAARHLQRQGFSVTVLEARNRMGGRVYTDRSSLSVPVDLGASIITGVEADVATERRPDPSSLICTQLGLELTVLNSDCPLYDIVTGQKVPADLDEALEAEFNSLLDDMLVLVDQKGELATQMSLEDGLEYALRRRRMARPVSDAENLELHTSVNASTENGRCCFTNDHPDKFKSKDEILSPLERRVMDWHFANLEYGCAALLKAVSLPHWNQDDVYGGFGGAHCMIKGGYSTVIESLGKGVDILLNHVVKEISYRTRDSREDSEKWSKVKVSTSNGREFVGDAVLITVPLGCLKADTIKFSPALPDWKKSSIQKLGFGVLNKVVLEFPKVFWDDSVDYFGATAEETDRRGQSFMFWNVKKTVGAPVLIVLVVGKAALDGRDVSSYDHVNHAVMVLRRLFGEAKVPDPVASVVTNWGKDPFSRGAYSYVAIGSSGEDYDVLGRPVGNCLFFAGEATCKEHPDTVGGAMLSGLREAVRIIDILSTGNDHTAEVAEMEASQRQSESKGNEVKDMLRRLDAVEPSGVVYKSSLDGAQMLTKESLLKDMFFNAKTNAGRLRLAKELLNLPVEVLKSFAGIKEGLSTLNSWILDSMGKDGTQLLRHCVRLLVLVSTDLLAVRLSGIGRTVKEKVCVHTSRDIRAVASQLVSLWIEVFRKEKAANGGLKLLRQATALDSSKAKSSKDSSSGKPPLCNTHGTADDRGAIEVPSSGGSHSLSNVNSKNNKKMNCKPSKLETVTDSKSEVSSRSEGVVQRLDPKVGESCVAMSEEEAAAFAAAEAARAAALEAAKAYASSEADCSALRELPKIPSFHKFARREQYAQMDDSDLRRKKWSGDVLGRQDCISEIDSRNCRVRNWSVDFSATCANLDSSKMSGDNYTQMSYSNEVAYQLNLREHSGESVAIDSSFTKAWVDTAGGGGVKDYLAIERWQSQAAAADLDFYHSTGQVRDEEDSNTATKLHTEKHERQAEESSVSQLSENKLLVENQRRGVERIKQAVADYVASLLMPLYKARKLDKEGYKSIMKKSATKVMEQTTDAEKAMTVSEFLDLKRRNKIRPFVDKLIERHMAMNKVAKS

>GSMUA_Achr2G09900_001|Macuminata_v1/1-1415

MRSCNSQLLPANISSDENPQPDDVIHQSLSANIQGVSLVNHILYDASKEPSSTKSRVPFTIEDPDVVNSETRFNQTETHQTEPSSVSECDVYNQSTISRVVRNTKIHRHGDMAYEGDADWEVLMHEQGPFANLSATNEDQSLRQRDKSCAHSLDEVSYDGSVAVATGLKAHAVSPIEKIKFRDILKRRGGLQEYLDCRNFILGCWSKDVKHILPLMNCGATDASSKDESARQSLIREIYMFLDQNGYINAGIASEQCVPKPSVNVQVKENANTELDLSPVVLICEREPPKTFIAGDPVCSDLVCNLRSGYDKFSPYDEGHIEAFSDMHNVTNMVKENAKIHSSFAQSLEVNKSTESGLNIHKRIIIVGAGPAGLTAARHLHRQGFSVTVLEARDRVGGRVYTDRSSLSVPVDLGASIITGVEADVATERRPDPSSLICNQLGLELTVLNSDCPLYDIVTGLKVPADLDEALEAEYNSLLDDMVLLVAQNGEGAMEMCLEDGLEYAIRKRRMSKATSDSVEFNKLNGADHDIETNILSPLERRVMDWHFAHLEYGCAASLKEVSLPHWNQDDVYGGFGGPHCMIKGGYSTVIESLGLGLDIQLNKNVTEIIYNTNEISGAGQDENKVKVITSSGMEYVGDAALITVPLGCLKAETIKFSPALPDWKQTSIKRLGFGVLNKVVLEFSKAFWDENVDYFGATAEETDKRGQCFMFWNVKKTVGTPVLIALVVGKAARQTFSKSDHVNHALMVLRKLFGEASVPDPIASVVTNWGMDPFSRGAYSYVAVGASGEDYDILGRTVANCLFFAGEATCKEHPDTVGGAMMSGLREAVRIIDILTTGKDYLAEVEAMESTLRQSDSERNEVRDMSKRLDACKLSSGLCKSSSDGKHILSTKESLLQDLFFSAKTTSGRLHLAKELLRLPVESLKSFTGTKEGLSTLNTWILDSLGKNSTQLLRHCVRLLVLVSTDLVAVRLSGIGRTIKDKVCVHTSRDIRSVASQLVGMWIEVFRKEKAVNGLKLLRQATCSESSKVRSKELILGKPHLRMTTETSENKCTIQVPSSSGSHSPSKVNIKKPEILPTKLESSVCIKSDVRSLRSQSIVHTKSKTKDNLVVSEEAATFAAVESARAAALKAVEAYATSEAEVPLRELPKIPSFHKFARREHCVQLDEFDVRRKWSDGNFGRQDCVSEIDSRNCRVRNWSIDVSAACLDNSKMSGDNYTQCSYSNEVPYTSSLREHSGESGAVDSRLTKAWIDTDAVGSGGVKDSLAIERWQLQAMDADADFYSSIHIRDEEDSNNLVLPALRNQCQTGADAALQVAENKSSLEHQQRGVNYIKQGVVDYVASLLMPLYRTRKIDREGYKSIMKRTATKVVEQCTEEEKRLAIYDFLDFRRKNKIRSFVDKLIERHMAMNQSVKS

>Litul.04G069300|Ltulipifera_YP108A_v1.1/1-2230

MGEEDNIRKKKGSSGKMKLKPVEMKVAGGDSDDDDEPIGSLFKFKKPRIGKRSKSGSDGVKVEAAGVEKSRVGDVDLGGMNDTLASFRKKLKGPRIVGESGSGLLDRSSVSALMDGAAVGLDSPSVEYSECVPEAKRKRRQRQKKTGVKPESRNGPDLLISMREQEDRLLNVEGSLGDSSDENMKDLLSGFVRKAQSGSVRKARSLSRRRAGDGSSKISAGVLKEPPVTVMPKSEGGFVLGSDGTFKDPVLVKGRRARRTSRFNGNLDQTSGASSDDEPLSVVVGKVSSASDRKETAEARRSDEGLRQTSSLIGRDGLSVSVPHTMEETAGSNDGQFDMSSERVPEDPLPAVPRRSNLGSIPCPREKSETPENDSLDGLIEGASEGRVPCDDGLDQPCDGAMEDRPSGRVPETCSVSSEVPTEEYVRSDDGLNQSSEIIPAVLLRNAAQNSCSMRTRETTASCFDYDLLNGAHEETQKVNHAGCDDCSDEISKNTEKEFPNHVAKFVSVSNLCQAEETTRCDELELPAFENVRGGLSCLDGKSSMTSMPMQMDQMPLSDDGLNKCSNRLLKDFQSQPKTSSSGSMKIDATPCCDDDLNQPSDESIGNPIFVAIQRPHLASVAMEKEGISTADGALDRLYDGMLSVLPSEALEKSDFVPSLNHKEELTDTGDVLNRFSDGLAIDSTTFIKKSCSDSPQTQPSDDASERPFDDVPVRDSFSVKEDKSAGGQSMVPDLKETYPDDAGRASDPDKLDKKLSEMQRVGRKVKKRRHGDMAYEGDIDWEILMHGQGPFENNPVIDGDRPIKTKDRPDTRSNMLAEANNGGAAAVAAGLKARAAGPVEKVKFKEVLKRRGGLQEYIECRNFILDLWSKDMCHILPLIDCGVTDAPSEDEQPRASLIREIYSFLDYSGYINVGIASEKEKVKSHSIPHVKLSKENELKETSGSQNADSQEEVGYILGQVKSYENLIVVKNKVSVADGRPASALDMEATKSDYLVPRKGPETASCKLHAGMQTACVSCAPVPVSGEKIDVSEKCSDGEVPPKTGSCMLVMNDQENDCMDAQVTNTAAKLDASSTDPSCKTVDGGLDSASALEQIKSSCIKHQPTDINREKAAHVGSVRIECAGRDPTEGNDILQCDQEVRKRVIIIGAGPAGLIAARHLQRQGFSVNVLEARDRIGGRVYTDRSSLSVPVDLGASIITGVEADVATERRPDPSSLICGQLGLELTVLNSDCPLYDIVTGQKVPADLDESLEAEYNSLLDDMVVLVAQNGEGAMKMSLEEGLEYALKRRRVVQSTAVAKESEQFKVPYDSEVLDTSANGSIETAIKNNADNRLMDISILNEELLSPLERRVMDWHFANLEYGCAALLKAVSLPYWNQDDVYGGFGGAHCMIKGGYSAVLESLCKGLSIHLNHVVTEVMYSTRDSGGTSGRRSEVKVSTSSGDEFVGDAVLITVPLGCLKTNMIKFSPVLPDWKQSSIQRLGFGVLNKVVLEFSEVFWDDTVDYFGATAEETDRRGQCFMFWNVKKTVGAPILIALVVGKAAIDGQSMSSSDHVNHALMVLRKLFGEASVPNPVASVVTNWGKDPFSRGAYSYVAVGASGEDYDILARPVENCLFFAGEATCKEHPDTVGGAMMSGLREAVRIIDIFNTGNDCTSEVEAMEAAQRQSDSERNEVRDMVKRLDAVELSNVLCKSTWDGTQRFLTKDALLRDMFSNTKTTSGRLHLAKELLRLPVEVLKSFAGTKEGLSTLNLWILDSMGKDGTQLLRHCVRLLVRVSTDLLAVRLSGIGRTIKEKVCVHTSRDIRAVASQLVSMWIEVFRKEKAANGGLKLLRQTVASDSSKIKSKDIGLGKPPLRMSNEVPDGRGNLQVPSSAGSHSPSNPNNKKVDGRPAKLETMTDTKAKINPRPNHVIQNRDSKADESNVLSEEEAAAFAAAEAARAAALAAAEAYASSEAECSTLRELPKIPSFHKFARREQYAQVDDFDVRRKWLGGMLGRQDCISEIDSRNCRVQNWSVDFSATCGNLDSSRTSGDNYTQRSNSNDLVGHLNVKEHSGESVAVDCRLTKAWVDSAGSGGVKDYRAIELWQSQAVDAYSDFFHPSVHERDEEDSNKMSKLSIGKTEREGEESSGSQAAENKGSVGNQPRGAEHIKQAVVDYVASLLMPLYKARKIDKEGYKSIMKKSVTKVMDHSTEAEKGMTVGEFLDFKRKNKIRSFVDKLIERHMAMNPNTKP

>Aco016593|Acomosus_v3/1-1958

MWLAISGSNPFALDPTPNPIRTESTYQRLGVSERLHPLSSSSSSSPFVSNAGKKKKIPLRPLATSTHASRGRALASDAASLAEIARSPSPIPRSGSCKMGGGDGDEGHVLGAEGNSDDDERPLGSLFKKKIRVPRKRREAAPEARAEGNPRINERGSGEVNDTLAAMRKKLKRRRRVGEADGAGSGVGQDSTTLDGDRVSEQKGALAEVDNCSDHSSDLVNDSLSAFLTSRSVRRSRQVSKKSKETAPRGINDAKRSLNKASDDGYGGKNGEMSTRADRCSTPNSDDELLANQHVVGKTQKGRAGNKTALKQPSRIPSRASRKPLSSSAQELLVPISGSEVKNVEKDFDSSYRKLDCNLDNSAPSLIQAKDSDSVLKAKDDKLSATQRENNSIQASDRALEDGSDHFISGNVTQLKVPTWTIDGSGLKPCSGELIEESALASSNETNQKTEGVSEENRRNIDRVDLSCDETPNQENIMNGDGVVLTFQKEVEKPSLREKEISFVLLSRKSEAIADCDKLLQHSPDNCEGSLPKSDGKSSQDDGLSSYVNISVTSHQVVEDSAVPFTNCNGVPSANACVDQSVRGISVVNKSAADAFKEPSPTMSMKEGGITKETNEYDVQLDPDNAENQSMVPRAMRNAKKRRHGDMAYEGDADWDVLMHEQGLFTNISIDCRDRSIKSKDKSNADSNLQVFDDATYGAAAAVAAGLKARAVSPIEKIKFKDFLKRKGGLQEYLDCRNLILSHWSKDVRRILPLADCGISDSPSEDESSRNSLIREIYQFLDRNGYINTGIALENGANKLLYTPHSEVASDFELNESYGLNGASHNNEIASSRGNDAAVDNTKTLNAPETEFDARAQAYTENEKEPSAYIRKASGITDSDSKGCKRIIVVGAGPAGLTAARHLQRQGFFVTVLEARDRIGGRVCTDSTSLSVPVDLGASIITGVEADIATERRPDPSSLICTQLGLELTVLNSHCPLYDLVTGDKVPADLDEALEAEYNSLLDEMVLVVAQNGEGAFKMSLEDGLERLLRKRRMTQSTSFIATDQYKMICDSQCVDASKFGSTDSGIVSNEGSDKTDVLSPLERRVMDWHFAHLEYGCAAMLKDVSLPHWNQDDIYGGFGGPHCMIKGGYSTVIESLGKGLDIRLKHIVTKVEYETKESDNVCQNQNKVKVCTSNGSEFFGDAVLITVPLGCLKVEAIKFSPCLPDWKQLAIQRLGFGVLNKVVLEFPEVFWDDTVDYFGTTAEETAHRGQCFMFWNVKKTVGAPVLIGLVVGKAAIDGQSMSSYDHVNYALMVLRRLFGQASVPDPVAFVVTNWGTDPFSRGAYSYVAKGASGQDYDILGRPVANCLFFAGEATCKEHPDTVGGAMLSGLREAVRIIDVLSSGKDYMAEVEAMETVQRQSESEKNEVRDLSKRLETCKFSSDAIHSLVTKEALLRDMFSSAKTTSGRLHLAKELLRLPVEVLKSFAGNKEGLGTLNSWILDSLGKNATQLLRHCVRLLMLVSTDLLAVRLSGIGRTVKEKVCVHTSRDIRAIASQLVSMWIEIFRKEKANNGGLKLFRNISASETTKIKTKDLQQGKLPLRPTNEVMDNKGSLKIPKSAGSYSPSKPYHKKSDSKTTNLETPMDTKSEANSLRFRKPAQGLESEVEHNTVISEEEAAAYAAAEAARASAIAAAKAYASSEAEATTSRELPKIPSFHKFARREHNLQMDETDFRKKWSAGSFGRQDSVTEIDTRNCKVRNWSVDFTATYANPEASRMSGDNYTQRSLSNEMACPLNLREHSGESVVDSRLTRAWVDTDNVGTGGVKDFSAIERWQSQAMDADADFYRNLRIPDEEDSNKIFRLHTVNHQKRTDGSSASQVAESKPILDGRSRVEHIKQGVVDYVASLLMPLYKTRKLDREGYKAIMKKAVTKVMEQCTEAEKAMAVYEFLDFKRKNKIRSFVDKLIEKHMQVNQTPNS

>Acora.08G090800|Aamericanus_v1.1/1-1879

MADGGDVMKGEGREEMGGGLAGTVGFGDSLSSFVQQARSSSKRKVGNAKPSTGRKSRSLSVRKGRMSNDDRLGRASNQDKGADSVWEVSSPSTDVLDGSSVKGSRRGRGKALKARRIEETQRLEDGLKQCSNERQGQLGHSVVKLSMPVSEAEGEEAHGVGDDAGLNGPTNEIGKNAENRSTKQTCGDILKTSRDLSGLEDPRGSPSKCEQPNVYREDESKLYGSVEDQCPDQPFMKTMNELLVHAVSSESFKMGDRLKSGEFNQFSGANLEDSQDVNLQKPISGSGRTELEEIHVDDNSKFDDKLTQPSNGALSVFKYDTIKGTDFLSIAYGGDQNPDVDESLNRLSLGVSSFSPLNQPIQNASNEFSSVAYTKNAKETGCSSVLTFQNNVRIDEHIGIADSNKTVKEPVDLVRAVRKIKKRRHGDMAYEGDNDWEVLVHEQGFLENIPTVYGGRSTRTGDKHNSSANALVNANDGGEAAIAAGLKAWAAGPIEKIKFKEVLKRKGGLQEYLECRNLILGLWSKDIGHILHLEDCCGVTEAPLEDESPRESLIREIFVFLDHSGYINVGIASEKGKSKLHIEPSTEFVKETKSTETRRRPVIGSGDEASAFFGQLHVTENIAAPVVDLFSEYEKSSSRLDTELNSGKELPVPIMSSKSFNEVDPGRHLAENMEQDLPLASVDGTPRLSINDNNLTAPCLGRETQLETGSCNSLLLDVDIPCRDAQLNALSTDLSNEAVDDIFAQNQINGLSCNHRFIENSRDKVNRFEVSRGESGGMHHLQPTDDLSSDMEVHKRIIIIGAGPSGLTAARHLQRQGFSVNVLEARDRIGGRVFTDHTSLSVPVDLGASIITGVEADVATERRADPSALICAQLGLQLTILNSDCPLYDIATSQKVPADLDEALEAEYNSLLDDMVVLVAHNGESAMRMSLEDGLEYALRRRRSNQLSGFPAEPEQLKIDPYSGISDADTMMVKSSDGSVLGDGALTENLLSPLERRVMDWHFANLEYGCAASLKEVSLPYWNQDDVYGGFGGPHCMIKGGYSTVVETLGKGLNIHLNQVVTEIAYNTISSGAGGKWKNEVKVITSNGSVFVGDAVLITVPLGCLKARTIKFSPDLPEWKNSSIQRLGFGVLNKVVLEFAEVFWDDSVDYFGATAEETNCRGQCFMFWNVKKTVGAPVLIALVVGKAAINGQNMNNSYHITHAVMVLRKIFGEASVPDPVASVVTNWGRDPFSRGAYSYVAIGASGEDYDILGRPVANCLFFAGEATCKEHPDTVGGAMMSGLREAVRIIDIFNTGNDHTAEVEAIEAAQRQSDSERNEVRDLAKRLDACKLSNVLCKSSLDGGSLTKEALLQDMFFYAKTNAGRLHLIKELLRLPVEVLKSFAGTKEGLCTLNSWILDSLGKNATQFLRHCVRLLVLVSTDLLAVRLSGIGRTVKEKVCVHTSRDIRAVASQLVSVWVEIFRKEKASNGLSKVLRQTSMTDSSRMKSKDTGSGKPPIRAIIDATDCRVNLHSLLSDGSHSPSGANNKKNHCRPPAKLETLLDTKSDISSSRSRSVPLGMDSKTEDNTVISEEEAAAIAAAEEARAAALAAAEAYASSGGHNSSRELPKIPSFHKYARREQYAQMDESELRRRRWLAGGLGRQDCISEIDSRNCKVRNWSVDFSATCGNLNGPRMLEDNPMHQSYSNELGCHLNLREHSGESGAVESRLTKAWVDSDTAGSGGVKDYLSIERWQAQAIDADADFYNARMYVKDEEDSTKYSNPSMEKHEGRAEESSVSQTARNKVPFEANQRGAEHIKQGVVDYVASLLMPLYKARRFDKDGYKSIMKKSATKVMEQCTDVEKMMTVPEFLDFKRKNKIRAFVDKLIERHMAMRPNTKS

>Aqcoe6G245800|Acoerulea_v3.1/1-1810

MDDTLASVWKKLKGPKTTTGKDDAGSGMKKGNCSGLNVGELRSEGVEESLTSSGDGADLSMDRVVDVKEKGRGKRSRLVKKMWSHSEFQTIDQESSDLKSQKKLRKKLKIEGLALGESCSHSSDENLELSTFILKSQSCLAGKSHSQKKKRGTPVSGDGFAQSPDAALEHASSQLIQRSHSASMVSCKNLKPECGLSLASTKTSKDPAVVQTNRTGVATEPCDAHNTTTVCCSDAAGEPYESFELQREETLVPSYGVDILSRQSEGKHDTNGLGGSSCLNQPSEDVFEDLAGKKSALLASKIEAEVTARHDEFAQISGDIAENSLLQITDTNIGETLVAPVLKQMEETSRLNEATRGFPAQSRDEDAAQSHGGDTICFHSVKLTPVETFPEDADKAFDPENKDEKLEAMQRAARKIKRRKHGDMAYEGDTDWDFLLHEQGLFGSRDIFNGDRPSREKNRSNFSCMAVEAENCGAPAVAAGLKARAASPFEKIKFKEILKRRGGLQEYLECRNLILGHWSKDTSRILPLSDCGATDASSNYDSPRASLVREIYDFLDRNGYINIGVASVKEKAELLTETHHKFFTEIKAEDNCGAQVTDSEGRSACLVVQGECSESLTETKKDVLLDNNISQATENIVLVASIGSENGLIKSEDHMINNEQKNEWIDVKQPNRAAELGSESLTLTKVSGVVVDGGPNAVAMGKQKHALAIQSIVDSTEGIDSIECNSKDKKRIIVIGAGPAGMTAARHLQRQGFSVTILEARDRIGGRVYTDHSSLSVPVDLGASIITGVEADVATERRPDPSSLICSQLGLELTVLNSDCPLYDIVTGQKVPSELDEALEAEFNALLDDVAVLVSKKGELADKMSLEGGLEYALKRRRVALAGFNAEELEFDALMNTSLDTETDCDANGISSRISSEKETLSPFERRVMNWHFANLEYGCAALLKAVSLPYWNQDDVYGGFGGAHCMIKGGYSSVIESLGKDLQIHLNHVVNEVSYSMKGYDEIDESNGKVTVSTSNGREFVGEAVLITVPLGCLKANTIKFSPSLPDWKQSSIRQLGFGVLNKVVLEFPEVFWDDSVDYFGATAEETDLRGQCFMFWNVKKTVGAPVLIALVVGKAAIDGESMSSTDHVNHALKTLRRLFGTASVPDPVASVVTNWGQDPFSRGAYSYVAVGASGEDYDILGRPVGNCLFFAGEATCKEHPDTVGGAMMSGLREAVRIMDILNTGKDYTAEAEALEAAQRHTESERNEVNNILKKLEAVKLSNVHYKSSFDDDHMLSKEGLLQEIFHNAKTTSGRLHLAKELLNLPVEALKSFAGTREGLSILNSWILDSMGKDGTQLLRHCVRLLVLVSTDLLAVRLSGVGRTVKEKVCVHTSRDIRAIASQLVSMWIEVFRKEKAANGGLKLLRQATVTESSKKKFSKASMSVKYTRMADVATYNRGNMHVPSPPGKKTLSNANNKKTNSKLVRLDTVTGSKSEGNSLHSREVGQDHHSKAADVPDNNVPMSEEEAAVFAAAEAARAAALAAAEAYTSSEAERLSIRELPKIPSFHKFARREQYAQMDESDFRKRWSGGVLGRQDCLSEIDSRNCKVRNWAVDFSAACANLDTSRMSGDNYTQQSQSNEVGYHMNLREHSGESAVTDSRFTKAWVDSAGSGGVKDYQDIERWQSQAAAVDPDFFHHTLNIRDEEDSSAASKPVSRRYDRRKEGSAVSQGAVNQVLADNQTKGGERIKKAVADYVGSLLMPLYKTRKIDREGYKSIMRKSVTKVMELVTDAEKAMNAFDFLDFKRKNKIRAFVDKLIDKHMLSNPVKS

>Aqcoe5G272100|Acoerulea_v3.1/1-1853

MNEEDEENKQHKSSNSDDDGCLLNEIATTSKKRIQQQVEEEDDDEQPLGSLLKLKKKKKNKKRNSRNPKKVKKSVDFINVEIRAHEEKEKEKEEDDFGEMDDTLASLWKKLKAGKDVTGSGKMMNKNKKGKSFCLNANMDDLSKQSNNNKKKLGSHSELLDDNMDDQESGGDLRSQKKLIRKKLKKKATLGETSSTVLEHDASPQFQFVQGSCSASQSECCKTTTNDSAIVQEGEEQFHVSEKPVVVVVLPKVGEEVNMTCYRSQTQRLINGLKLCSKVNNLNAVSTEPCDTYNSTAVCFSESAVGEPCESCVLPKEEEMLVSKSNYGDDILSRRSEEKCGTHGLDRRSSCLNTAENSLMQLTDINIGEISVSPILKQMEENSRFNDGSHSVFAPFDAKKDVHFCSSENHKEESRTTGDIIIQSSDSLTPVYAAHTEKKESYVSRGSSAQSQDGDATHSHDGDATCSHSIKLKPTETFLEDADKSFNPENKDKKLEAMQRAARNVKRRRHGDMAYEGDTDWDFLLHEQGLFLSKDIFNCHRPPREKNKSDYSIMAVESVNCGAAAVAAGLKARATCPFEKIKFKEILKRRGGVQEYLECRNSILGLWSKDTSHILPLSDCGAADALSDHDSPRASLIREIYDFLDHNGYINIGVASEKGKKEAHTETRHKISKESPHNDISEPAESIDLVPSIGSENGLIKSEDHRINNEQKNWIDVQLPNRTADSDTLTKFSGVVMDGGLNVVAPEKTKYSLAIGSTVDSVEGIDGMQCISKGKKRIIVIGAGPAGLTAARHLQRQGFSVTILEARNRLGGRVYTDQSSLSVPVDLGASIITGVEADVATERRPDPSSLICSQLGLELTVLNSDCPLYDIVTAQKVPSELDEALEAEFNALLDDVVVLVSKKGEHAEKMSLECGLEYALRKRRMARTGSDAEELEVDTLMDAALDTETDCNANKIPSRICCEKEPLSHFERRVMNWHFANLEYGCAALLKAVSLPYWNQDDVYGGFGGAHCMIKGGYTNVIESLGKGLQIHLDHVVTEVSYNMKGYDKINESNGKVKVYTSNGSEFVGDAILITVPLGCLKANTIKFSPSLPDWKQSSICQLGFGVLNKVALEFPEVFWDDSVDYFGATAEETDRRGQCFMFWNVKKTVGAPVLIALVVGKAAIDGESMSSTDHVNHALKVLRRLFGTASVPDPVASVVTNWGQDPFSRGAYSYVAVGASGEDYDILGKPVENCLFFAGEATCKEHPDTVGGAMMSGLREAVRIMDILNTGKDFTAEVEAMEAAQRHTESERSEVRDILKRLEAVTLSNVLDKNSFVGDHMLTKEALLQDIFHNAKTTAGRLHLAKELLTLPVEALKSFAGTREGLSTLNSWILDSMGKDGTQLLRHCVRLLVLVSTDLLAVRLSGIGRTIKEKVCVHTSRDIRAVASQLVSMWIEVFRKEKAANGGLKLLRQKTVSESSKNKFSKASIPGKYTLTAHGATYNRGNMQVPSFPGKNSLSNADNKKTYSKIVRLDTLTDSKSEGNSLHSQEVGEDLVSKAEDNNVAMSEEEAAVFAAAEAARVAALAAAEAYASSEAERFAIRELPKIPSFHKFARREQYAQMDESDLRKRWSGGVLGRQDCLSEIDSRNCKVRNWTVDFSAACANLDVSRMSGDNYTHESQSNEVAYHMNLREHSGESAAIDNRFTKAWVDSAGSGGVKDYQDIERWQSQAAALDPDFFHHTLNIRDEEDSSAVSKPVSRRYDKRTEGSTVSQGAENQVSVSNQTRGVERIKKAVVDYVGSLLMPLYKARKIDKEGYKSIMKKSATKVMEQTTDAEKAMNAFDFLDFKRKNKIRAFVDKLIERHMLMNPVKS

>GWHPBFSI001270|Chloranthus_spicatus/1-2325

MNDERGLFGKGGIGQKGGVLSDLDEGLNLGFSNTRSDYAKMGVENKVDDFDKKSGSRPLEMKADFGDDDEPIGSFFKLKKRPRSCGTKSVKSGENGGIMVAKSVISGDNGRFRVANSVTSGDNGGIRIGVEKPRVKEEDSGEMEDTLASFKKKLKAPKRVEGGGSGVVKGKDEARFSLKDGAGLASAILDESLKDSVQQLVRKPRRASDKVLDRKRLDESGASDQSSIVSLTDGAGPEGLKDSVKKRGRKSRHALTGKKMVHASGIQKYELTSLEEQKKGDLGFGEIVGHSSDDNLELSLSEFVRKSNSCSVRKARSMSIQKRKNDGISTDLAPMLGQRPHSASTLKLTKEIVKSEGKFIPLSDGTSKRKSRRASVAKLQEGNTKFDEQLDQASNENSEESIPAGVGKTFSDLVPKDTVENQRSDDGSSRNPNFIRTDSFDGSDSFPGVTEVPVKLTEEVHKCEDGLKKYFDEKSSDPLYDITKTSNFASDLKHEETSGIDDKRLNEHPNEISVDLSSSLVIMSGTAVLPHAEEHRETKNILNKFSDGTCIQVQNPCSVNAVKITEDTISSDDGLNRSSDCFPEVQSLSAILKSCSASNLVPRKEIPSSCFNDSPFRPCEKMDTESAPDSDNCSGYSKNEATEGTGYDELRAPVGGNLNYSLSSLVKKFPLNSMPMKTGDIPLPDDGLNKYSEGILKDPQFVQLQTSSSASGTVKKEETHSYSDGLPQTLDVAIGDSLIVTETKPHPAPVDMQIEQTRVTGDELKRFSDTTMAVAPSIDDEKSDFVTPLCPKEELTPTGDVFNQFSCGLKVDSPTLSQKRDSANLRISSTVDASKEPSSVPINKEFYLEDPGKASDPEIKEKTLLTMQREVRKIKKRRHGDMAYEGDIDWEVLMQQQGFFESNNPATDEDWSIRTKDKSDSPSNMLSEVDDGGAAAVAAGLKAHAVGPVEKIKFKELLKRRGGLQEYLECRNLILGLWNKDVSRILPLIDCGVTEAPLENESPRSSLIREIYAFLDHRGYINVGIASEKQVARPCLMSHLKLSKENMLVDSFRGRVSDSDDDTPFILANVRKSDNLIVAKNNGSSSVVKEDVRSKKLSVSNKQTELSTIGEPAECAVDNMDIDTSAKTVLINRNSDLEVHLGSESWTYEPSDQEDGCRDAQVPNVMTKWDNNDDPSFVALDAVDVLEQSKVAHSKLQSIDINKEKAMDAKITGIQSTTIDPLEVGNGTQYDSDVGKRIIVIGAGPAGLTAARHLQRQGFSVVILEARNRIGGRVYTDRTSLSVPVDLGASIITGVEADMTTERRPDPSSLLCAQLGLELTVLNSECPLYDTVTGEKVPSDLDEALEAEYNSLLDDMVVFMDQNCEVAMRMSLEDGLEYALRKRRTRVPRSMVAALESDEPKVSDSEVSHISANASFDTTLTNGDDNRVLGDSRSSENILSPLERRVMNWHFANLEYGCAALLKEVSLPYWNQDDVYGGFGGAHCMIKGGYSTVLEALGQGLRVQLNQIVTEVTYGKDSAGAHGQGSVVNIHTSSGSVFVGDAVLITVPLGCLKANAIKFSPVLPEWKQLSIQRLGFGVLNKVVLEFPEVFWDATVDYFGATAEETGCRGQCFMFWNVKKTVGAPVLIALVVGKAAIDGQYMSISEHVNHALMVLRSIFGEASVPDPIASVLTNWGRDPFSRGAYSYVAVGASGEDYDILGRPVENCLFFAGEATCKEHPDTVGGAMMSGLREAVRIIDILSTGNDYTAEVEAMEAAHRELDNERNEVSDLVKRLDAVELSNNLCKNSLDGVKRLTEEALLRDMFFNAMTAAGRLHLAKKLLRLPVEVLKSFAGTKEGLKTLNSWIRDSMGKDGTQLLRHCVRLLVLVSTDLLAVRLSGIGKTVKEKVCVHTSRDIRAVASQLVNMWIEVFRKEKAANGGLKIPGQTTVADSSKVKSKVLSSVKPPLRNEMSDGRGTIQVPSTAGSDSPSGTINKKMPCASVKLEALNNSKSGFNSSNSHNVIHGGMDSTVDGSIAISEEEAAAFAAAEAARNAALAAAEAYASSEADCNTLRELPKIPSFHKFARREQYAQMDDSDLRRRKWSGGALGRQDCISEIDSRNCRVRNWSVDFPSACGNLDSSRMSGDNYTQRSNSNDFACHLNLREHSGESGAVDSSLLTRAWVDSPGNWGIKDYHAIERWQSQAVAADPDFFDPTIHIRDEEESNKVSKLLVGMYGEQAEEISASQATENRGITENDKRGAEQIKQAVVDYVASLLMPLYKARKIDKEGYKSIMRKSATKVMDQSTEAEKVMSVSEFLDFKRRNKIRAFVDKLIERQMEINPNIKS

>Pso01G29050.0|Papaver_somniferum/1-1913

MVIEQIFTNDESYLVGKGSMGVETLHVNSDINGGLVLEGEELLSSNKIRVDEDDVKMDKEAKKKPIHVIVDSGDDEPIGSLFKKLKNPKKVKAGLGDVGKVGKGEARVRVSKHDDSGEMETTLASFRKKVKKPKVGQDDRSGTGKGNGSGFSDVGLGSKEVKESLVCSGDDALDSSLVEEILQDKPKGKRKRFRDHVMQKKKGGDNSSLDDGSDQGSGMQRLQETSAMEEKEGILCCDNASGDRLDSLSGLLKKSQTSLVKKPLSSNPQKQRDGIPSSGAGLSCSSAGEDYRTDIKFSAPKNIRSSDGLVSVLASVPEIKKDVDEFATESEVIPEDHTVKHEGEGFGGSCYDSMTQTEEAHDHKALLKQRLHGETIKTLEYTSKSTCLRNTNGDDDHSSECVLETCKGTILDSGDCLNQLYEDTIEQRHVEKLSSMHCRSPSELTAKYDHVIQPSGEVLEHSFSCATKSHFADSITEKMENTDKFGSRFDEHSDHDEDSGKALHSRKKDKKQLSRKIKKRRHGDMAYEGDTAWEFLLHEQGIFENNRMSNGDRPSKTKDEFSDPTNAVLQSDDGRAAAVSVGLKACAAGPVEKIKFREVLKRRGGLQEYLECRNLILGLWSKDMGRILPLVECGVPEVPSEEELPRMALIREIYAFLDHRGYINVGVASDKKERMSESRLLTEALKETVIEEESEAPLVDLDNQIALDVGPFQSLKNPIEAKNDAFIQHQSLENPMEAKNHVFIQQAEEVPKDNNLHVLSSGSELSALIEFEECAVDRDQENDFIDSNLPKKLVNIETLNIDDPSSEAVEFREPHIISPKQMKDSCGVNSVVIDPVEEITTQCDLEAQKRIIVIGAGPAGLTAARHLQRQGFSVTVLEARERLGGRVYTDRSSLSVPVDLGASIITGVEADVATERRPDPSSLICSQLGLELTVLNSDCPLYDIVLGEKVQADLDEALEAEYNSLLDDMVVLVAQKGDRAMKMSLEDGLEYALKRRRTARSISDAEDRILATSEGGFRTRVNGTSEDVSCRTDFNEEILSPLERRVMDWHFAHLEYGCAASLKEVSLPYWNQDDIYGGFGGAHCMIKGGYSNVVESLGDGIQIHLNNVVTEISYSTAETGGQRRSVKVSTSTGNEFVAEAVLITVPLGCLKAETIKFSPALPEWKSSSIQRLGFGVLNKVVLEFPEVFWDDSVDYFGATAEETGQRGQCFMFWNVKKTAGAPILIALVVGQAAIDGQSVSSSYHVGHALSVLRKLFGEASVPEPVASVVTNWGNDPFSRGAYSYVAVGSSGEDYDILGRPVENCLFFAGEATCKEHPDTVGGAMMSGLREAVRIIDILNTGSDYTAEVEAMESAQEQSDGERNEVRDILKRLEAVEFSNVLYKNSSDRRQMLSKKVLLRDMFNHAKTTAGRLHLAKELLNLPIESLKCFAGTKDGLGILNSWILDSMGKDGTQLLRHCVRLLVLVSTDLLAVRLSGIGKTIKEKVCVHTSRDIRAVASQLVSVWVEVFRKGKTSNGRLKLLRQTAASDSLKAKLSKDTPSGKLLRMTRGSTSPGDISPSDVNNKKANSKLVKLDAVTDSKSEVNSLRSQGTAVSLDSKVEDDIVLMSEEEQAAFAAAEAARAAALAAAQAYASSEAVCSSLRELPKIPSFHKFARREQYAQMDDVDFRKRWPGGALGRQDCISEIDSRNCRVRDWSVDFSAACVNPDSSRMSGDNYTQHGYSNEDPHQVDMKEHSGESAANDNRLTQAWVDSAGYGGVKDYHDIERWQFQAAAVSSDFYHPMMDARDEEDSNTTMNVHNQKHEKRVEESSVSQSAANNLMFDKHPRGAERIKKAVVDYVGSLLMPLYKARKIDKEGYKSIMKKSSTKVMEQTTDAEKSMSISVFLDYKRKNKIRSFVDKLIEKHMALNPVDKP

>Pso06G28760.0|Papaver_somniferum/1-1911

MVIEQIFTNDESYLVGKGSMGVETLRVNSDINGGLVLEGEELLSSNKTRVDEDDVKMDKEAKKKPIHVIVDSGDDEPIGSLFKKLKNPKKVKAGLGDVGKVGKGEARVRVSKHDDSGEMETTLASFRKKVKKPKVGQDDRSGTGKGNGCGFSDVGLGSKEVKESLLCSGDDALDSSLAEEILQDKPKGKRKRFRDDVMQKKKGGDNSSVDDGSDQGSGMRRLQETSAMEEKEGILCRDNASGDRLDSLPGLVKKSQTSLVKKPLSSNPQKQRDGIPSSGAGLSCSSEGEDYRTDIKFSAPKNIRSSDGLVSMLASVPEIKKDVDEFAIESEVIPEDHTVKHEDEGFGGPCYDSMTQTEEAHDHKALLKQRLHGETIKTLEYTSKSTCLRNTIGDDDHSSECVLEMCKATILGSGDCLNQPYEDTIEQRHVEKLSSTHCRSPSELTAKCDHAIQPSGEVLEHSFSCATKSHFADSITEKMDDTDKFGRGFDEHSDHDEDSGKALHSRKKDKKQLSRKIKKRRHGDMAYEGDTAWEFLLHEQGIFENNRMSNGDRPSKTKDEFSDPTNVVLQSDDGRAAAVSVGLKACAAGPVEKIKFREVLKRRGGLQEYLECRNLILGLWSKDMGRILPLVECGVPEVPSEEELPRMALIREIYAFLDHRGYINVGVASDKKEIMSESRLLTEALKETVIEEESEAPLVDLDNQISLDVGPFQSLKNPIEAKNDVFIQHQSLENPMEAKNDVFIQQAEEAPKDNNLHVLSAGSELSALIEFEECAVDHDQENDFIDSNLPKKLVNIETLNIDDPSSEAVEFRKPHILSPKQMKDSCGVNSVVIDPVEEITTQCDLEAQKRIIVIGAGSAGLTAARHLQRQGFSVTVLEARERLGGRVYTDRSSLSVPVDLGASIITGVEADVATERRPDPSSLICSQLGLELTVLNSDCPLYDIVLGEKVQADLDEALEAEYNSLLDDMVVLVAQKGDRAMKMSLEDGLEYALKRRRTARSISDTEDRILATSEGGFQTRVNGTSEDVLCRTDFNEEILSPLERRVMDWHFAHLEYGCAASLKEVSLPYWNQDDIYGGFGGAHCMIKGGYSNVVESLGDGIQIHLNNVVTEISYSTAESGGQRRSVKVSTSTGNEFVAEAVLITVPLGCLKAETIKFSPALPEWKSSSIQRLGFGVLNKVVLEFPEVFWDDSVDYFGATAEETGQRGQCFMFWNVKKTAGAPILIALVVGQAAIDGQSVSSSYHVGHALSVLRKLFGEALVPDPVASVVTNWGNDPFSRGAYSYVAVGSSGEDYDILGRPVENCLFFAGEATCKEHPDTVGGAMMSGLREAVRIIDILNTGSDYTAEVEAMESAQEQSDGERNEVRDILKRLEAVEFSNVLYKNSLDGRLSKKVLLRDMFCNAKTTAGRLHLAKELLNLPIESLKSFAGTKDGLGILNSWILDSMGKDGTQLLRHCVRLLVLVSTDLLAVRLSGIGKTIKEKVCVHTSRDIRAVASQLVSVWIEVFRKGKTANGRLKLLRQTAASDSLKAKLSKDTPSGKLLRMTRGSTSPGDISPSDVNNKKANSKLVKLDAVTDSKSEVNSLRSQGTAVSLDCKVEDDIVLMSEEEQAAFAAAEAARAAALAAAQAYASSEAVCSSLRELPKIPSFHKFARREQYAQMDDVDFRKRWPGGALGRQDCISEIDSRNCRVRDWSVDFSAACVNPDSSRMSGDNYTQHGYSNEDPHQVDMKEHSGESAANDNRLTQAWVDSAGYGGVKDYHDIERWQFQAAAVSSDFYHPMMDVRDEEDSNTTMHVHNQKHEKRIEESSVSQSAANNLTFDKHPRGPERIKKAVVDYVGSLLMPLYKARKIDKEGYKSIMKKSSTKVMEQTTDAEKSMSVSVFLDYKRKNKIRSFVDKLIEKHMALNPVDKP

>Glyma.14G216900|Gmax_Wm82.a6.v1/1-1894

MDGEDIRSGTKKKRSKKEIGFDDDDEPIGSIFKLKRSKKKGSGGSSDAAVVREKEDLGGMDDNDTLASFRKRLKGPKRDQGSGVTRGASPALHVSDEDLVALGPKGKDEKVVVPVPGDEDMQMQGCSDQQHMEDSLSAIFNKAQFSSTRKSRGRGSRQKRGIQNVDSEGFVETVDSVVGSRSGSAFGSKLVGGNVESADALPQASEPVVASVVDDQKCGDDCFQEEAVKGNRNLDIPDGPSQSSNVCHGYRQQLSCVQVGDISCHSDQKVGLQESVLSDGLNKLPTTSHDVSLSKVGEGKRGFTEIGESENRLTDEQAKVCNSASEPDVSTSAGEKNVLTSCHTEPLIKSTENILNENNNMVARKVFQESSCNGALKLSGCHMEVDGGGKSETEIVSDRNFCDYSSSDTKAEVQDFVLGFSPKTNNVTVSGSLSSMVSNEANKAELTAHSNHPEKPLEACNIPKDYTASILKCSSVLDPIQSDGSSLQSSIPDENGNSAEYCAPASDFADNEGKISSIPRAVRKAKMRKHGDMTYEGDADWEILIDDQALNESQVMTDGDRTLRARLKHDSSLNTGEDSENVAVVAVSAGLKARKVGPIEKIKFKEILKRKGGLKEYLDCRNQILSLWNRDVTRILPLAECGVSDTHSEHGSPRFSLIREVYAFLDQYGYINVGIASQKENVGSSARHCYRLVKEKGFEESLAASMADPEDGVSFLVGQTKMSDTSNEINNGLTKDCDDLTTEAAEGMRHANEMKTDLSNMTHQAERKKIDYQENDSSVPSSNFPDCRLTSQVAEEKINDSTSIKSALDALVGDHLQSDLDPRKRVIVIGAGPAGLTAARHLQRQGFAVTVLEARSRIGGRVFTDHSSLSVPVDLGASIITGVEADVATERRPDPSSLICAQLGLELTVLNSDCPLYDIVTGQKVPADMDEALEAEYNSLIDDMVLVVAQKGEQAMRMSLEDGLEYALKIRRMARSESSEETEQNNSADSPFDSKKDSTVEKKFGEEILSPQERRVMDWHFAHLEYGCAALLKDVSLPYWNQDDVYGGFGGAHCMIKGGYSSVAESLGEGLTIHLNHVVTNVSYGIKEPGQNNKVKVSTANGNEFFGDAVLVTVPLGCLKAETIQFSPPLPQWKCSSVQRLGYGVLNKVVLEFPSVFWDDAVDYFGATAEERSSRGHCFMFWNVRKTVGAPVLISLVVGKAAIDGQSLSSYDHVNHALKVLRKLFGEDSVPDPVAYVVTDWGRDPFSYGSYSYVAVGASGEDYDIIGRPVDNCLFFAGEATCKEHPDTVGGAMMSGLREAVRIIDILSSGNDYIAEVEALEAARGQLDTERDEVRDIIKRLDALELSNIMYKNSLDGAQILTREALLKEMFNNTKTTAGRLHVAKQLLTLPVGNLKSFAGSKEGLAILNSWILDSMGKDGTQLLRHCLRLLVRVSTDLLAVRLSGMGKTVKEKVCVHTSRDIRAIASQLVNVWLEVFRKEKASNGGLKISRQTTAVDLSKRKSVKDSASGKPPLSTYHGTIENKGGLLNPTSAGSNSASTAHVKKLHSKQGRQPAAYDSRHEVSSSRSKGSIDTVVAEKEDNLCTVSEEEQAAIAAAEAARAKALAAAEAYASAEARCNTLLQLPKIPSFHKFARREQTSQNDEYDSRKRWPGGVYGRQDCISEIDSRNCRVRDWSVDFSAACVNLDNSRMPVDNLSQRSHSNEIASHLNFREHSGESVAGDSSIYTKAWIDTAGGIAIKDHHAIERWQSQAAAADSYFSNPTIHLKDEEDSNACSKLPSWKHDGIANESSISQVTVSKEAQKGHSRGADHIKQAVVDYVASLLMPLYKARKLDKDGYKAIMKKSATKVMEQATDAEKAMAVREFLDFKRKNKIRSFVDVLIERHMTTKPDMKS

>Glyma.17G255500|Gmax_Wm82.a6.v1/1-1905

MEGENIRSGTKKKRSKKEIGFDSDDDEPIGSMFKLKRSKKKGSGGGSSDAAVVREKEDLGGMDDNDTLASFRKRLKGPKRDQGSGVTRGGAIPALHVSDEDLVALGPKGKDEKGVAPVPLVWGDEDMQMQDCTDQQHMEDLLPVIFNKAQSSSSRKSRSQGSRQKKGIQNVDSEGFVEAVDSGVESRSGSASGSKLVGGNVESVELLPQASERVVASVVDDQKCGDDCFQEEAVKGHCDLDIPDGPSQSNNVCHGDMQQLSCVQVEDISCHSDQKVGLQESALSDALKNLSTTSHDEIVDTISLSKVGEGERGFTEAGESENRLTVEPAKVCNSASEPDVSTFAGKENVLTSCHTEPLIKSAETILNENNNMVARKVFQESSWNGALDLSGCHMEVDGGGKSETEFVSDRNFCDYSSLDTKAEVQDFVSGFSPKRNNVTVSGSLSSMVSNEANEAELTAHSNHPEKPLEGCNIPKDSTASILKCSSVLDPIQSDGSSLQSSIPDENGNSAEYRAPVSDFADNEGKISSIPRAVRKTKMRKHGDMTYEGDADWEILIDDQALNESQVMTDGDRTLRSRLKHDSSLNTGEDSENVAVVAVSAGLKARKAGPIEKIKFKEVLKRKGGLKEYLDCRNQILSLWNRDVTRILPLAECGVSDTHSEDGSPRSSLIREVYAFLDQYGYINVGIASQKENVGSNARHCYKLVKEKGFEESLAASMADSEDGVSFLVGQTKMSDTSNEINNGLTKDGDDLTLEAAEGMRHANEMKTDLSNMTQQVERKKNDYQGNDSSVPSSNFPDCRLISLVAKEKSNDSTCIKSALDARVGYHLQSDLDPRKRVIVIGAGPAGLTAARHLERQGFSVFVLEARSRIGGRVFTDHLSLSVPVDLGASIITGVEADVATERRPDPSSLICAQLGLELTVLNSDCPLYDIVTGQKVPADMDEALEAEYNSLIDDMVLVVAQKGEQAMRMSLEDGLEYALKIRRMARSESSEETEQNNSADSPFDSKKDSTLEKKLGEEILSPQERRVMDWHFAHLEYGCAALLKDVSLPYWNQDDVYGGFGGAHCMIKGGYSSVVESLGEGLTVHLNHVVTNVSYGIKEPGQSNKVKVSTENGNEFFGDAVLVTVPLGCLKAETIQFSPPLPQWKCSSVQRLGYGVLNKVVLEFPSVFWDDAVDYFGATAEERSSRGHCFMFWNVRRTVGAPVLIALVVGKAAIDGQSLSSSDHVNHALKVLRKLFGEDSVPDPVAYVVTDWGRDPFSYGSYSYVAVGASGEDYDIIGRPVDNCLFFAGEATCKEHPDTVGGAMMSGLREAVRMIDILSSGNDYIAEVEALEAARGQLDTERDEVRDIIKRLDALELSNIMYKNSLDGAHILTREALLREMFFNTKTTAGRLHVAKQLLTLPVGNLKSFAGSKEGLAILNSWILDSMGKDGTQLLRHCLRLLVRVSTDLLAVRLSGMGKTVKEKVCVHTSRDIRAIASQLVNVWLEVFRKGKASNGGLKISRQTSAVDLSKRKSVKDSALGKPPLGTYHGTIENKGGLLNPTSAGSNSPSTAHVKKLHSKQGRQPAAYDSRHEVSSSRSKGSIDTVVAEKEDNLCTISEEEQAAIAAAEAARAKALAAAEAYASAEARCNTLLQLPKIPSFHKFARREQPSQNDECDSRKRWPGGVYGRQDCISEIDSRNCRVRDWSVDFSAACVNLDNSRMPVDNLSQRSHSNEIASHLNFREHSGESVAGDSSIYTKAWIDTAGGIAIKDHHAIERWQSQAAAADSYFSNPSIDLKDEEDSNACSKLPSWKRDGIANESSISQVTVNKEAQKGHSRGADHIKQAVVDYVASLLMPLYKARKLDKDGYKAIMKKSETKVMEQATDAEKAMTVREFLDFKRKNKIRSFVDVLIERHMTTKPDMKS

>KAI5392854.1|Pisum_sativum/1-1874

MENEDLRSGTKKKKRSKPIEIDFDSDNDKPIGSFLKLKRNKKKVSFASEGSCGGDSVKEKENSRVMDDNDTLATFRKRLKGPKRDQGFEAMPALNVSVEGNGGSGKCDDDMQLHHDSDQHMEESLSTIFHKAQSCSVRKSRAAMGSKQKKRNRNVDSGLKHGSRSLTENVDSVVEIRSGSASALKSVERNHESDTFCSVSVMENQKGGGDCLQEEKAKGICDSNIPDEPSVDHSNSIIACDGDGQQLTSVQVENVCGASDEMVTLQEKIIDNSLNQCSSMLQDVEIIDIGSLSKVGEGVCGLSETGELENNRSIDEIAEEQVCNGASEGGISSSAEKEVLLTCHTKHLIKLNENNPIVSGKTFQESSINGGKKLETESVSGRDCYDYSPLETNAEVQDVVVGCSPEKHEAIASGNLSSILPNDANESELIVQSNHPDKPLEMCNIPKYSNAPIMKCSYTLNPNQSDGSSIQSSIPDENGNTTEYHASVSDFADNGGKISGIPRAARKTKMNKHGDMTYEGDADWEILINDKALIGNQDAADGERTLRARVKQDSSLNAVEDSESVAVAAVSAGLKARAVGPIEKIKFKEILKRKGGLKEYLDCRNKILSLWSSDVTRILPLAECGVSDTCSENESPRSYLIREVYAFLDQYGYINVGVASQKENVESCARHCYKLVKEKGFEESSTASLAGSEDGVPFNVGQTKMSYASMDINNDLIKDFEDLTTKATEGMMHVDEVMTDSSNMAQHERKNYDEQENVGIQDGVGGIIHFNDNSAVPSFKFPDCRLTSLVATKHNSESKFVKHALGDQIGDTLQSDLEARKRVIIIGAGPAGLTAARHLNRQGFTVTVLEARNRIGGRVFTDHSSLSVPVDLGASIITGVEADVATERRPDPSSLVCAQLGLELSVLNSDCPLYDTVTGQKVPAEMDEALEAEYNSLLDDMVLVVARKGEQAMKMSLEDGLEYALKIRRMGLSEGSEETKQNFDEEILDPRERRVMDWHFAHLEYGCAALLKEVSLPYWNQDDVYGGFGGPHCMIKGGYSNVVESLGEGLAIHMNHTVTNVSYGIKEPGENNKVKVSTLNGNEFFGDAVLITVPLGCLKAGTIQFSPSLPEWKCSSIQRLGFGVLNKVVLEFPTVFWDDAVDYFGATAEDRSKRGHCFMFWNVKKTVGAPVLIALVVGKAAIDGQSLSSSDHVNHALKVLRKLFGETSVPDPVGYVVTDWGRDPYSFGAYSYVAVGASGEDYDILGRPVDNCLFFAGEATCKEHPDTVGGAMMSGLREAVRIIDILNTGNDNTAELEALEAAQGQLDTERNEVRDIMKRLDAVELSSILYKNSFEGAQIITREALLKEMFLNVKTNAGRLHVAKQLLSLPVGNLKSIAGSKEGLTVLNSWILDSMGKDGTQLLRHCLRLLVRVSTDLGAVRLSGMGKTVKEKVCVHTSRDIRAIASQLVNVWLEIFRKEKASNGGLKLSRQITTLELSKRKSLKDSASGKPPLSIHQNAVENKEGLLNPVSAGSNSPSTTHARKLHSKQGRQQSVCDSRHEVSSSRSQGSIDKIVTKEENKRYAMSEEEKAAIAAAEAARTKAIAAAEAYASAEARCSTLSQLPKIPSFHKFARREQYSQNDEIDSRKKLPGGFFGRQDCVSEIDSRNCRVREWSVDFSTACVNLDSSKIPVDNLSQRSHSNEIASHLNFGERSGESAAVDSNLYTKAWIDTAGDGVVKDHLAIERWQSQAAEADSYFSDQINHLKDEEDSNAYSRLPSWKHDGVINESSVSQVTVNKEGSFKGHSRGADHIKQAVVDYVGSLLMPLYKARKLDKDGYKAIMKKSATKVMEQATDAEKGMTVREFLDFKRRNKIRSFVDILIERHMATKPGTKS

>KAI5402868.1|Pisum_sativum/1-1937

MDGEDMISGGNNKRTKEMEDGTDSDDNEMIFKLRKPHGVKKKVCLAPEEMCGNGGGENADSIEELVDVAQTDLGSMEDTLACFRKRLKGPKRDRDSSDAGGNVSSLNVVAESSDGPLNVSCQYEGLDERSWLNKDNVYEGRGSDLDMDMKVERPNVDLALKGIEDGDVSEGDLAVQESRNTPKDEKGVCVLLNVGMQHSSEEIMGDSLPEMLRTAQSSFVSKSCTAVSSEQKCGSGGQNLNDGLSPDSRCASKTADGTRDLNIPDGPLADPCFPANVCYGDSQPLSCVRSEGICSPSDQKTALQEIICNDGLKKCSVVLHDVEEITDTMSLPKMGERVSPIIEGELKKRLTDDQVELFNIPSKCSDFTSKDKEKFSPCNYEPLAKSSENVLCESNHQVSEKVLQGSSRHGAMSFFGCQPELDVGVFSETKFVSGKSCSDQNIIDTKDEVQDFVSGNLLKKNSAVSGGCLHPTVSNETNNSELAVQPNHPEIPSKTQIIPKDSDAYIPKCSLVLHPTQPSKNTYENTSLANGDCFYAKKDSEGASPQSLIPEDNENSAENDVSVSEVANFDDKTSAVIRRKAKKRRHGDMAYEGDADWEVLINDQAFLESQGVDGERILKPRGKLESFLNVVEESESDAVAVSAGLKAHEAGPVEKIRFKEILKRKGGLQEYIDCRNQILALWGCDITRILLLADCGVHDTPSEDEPPRSSLIRDIYTFLDHCGYINVGIASLKDNVESSAKHNYTLVGEKGYEESYTASVADSEDGVSFIVGQTKKPDASGETKCDRIVGNADLATEVTKDKMNVNMVTMDISNMTQHEERKSDDCQEKHGHGDVSEQSNESTCVKSVLGDQMGDILNSDSEVRKRVIVIGAGPAGLTAARHLQRLGFPVTVLEARSRIGGRVFTDRSSLSVPVDLGASIITGVEADVATERRPDPSALVCAQLGLELTVLNSDCPLYDIATRQKVPLDMDEALEAEYNSLLDDMVLLVAQKGDQAMRMSLEDGLEYALKRRRLERSRRSNKHNNYLDGSFGLKRGIIHGKEMEEVLSPLERRVMDWHFANLEYGCAALLKEVSLPYWNQDDVYGGFGGAHCMIKGGYSTVVESLGDGLDIHLNHAVTNVAYGSTEPGPGNKVKVSTSNGNEFFGDAVLITVPLGCLKAESIKFSPPLPPWKYSSIERLGFGVLNKVVLEFPSVFWDDAVDYFGATAEETSRRGHCFMFWNVRKTVGAPVLIALVVGKAAIDGQNLSSSDHANHALMVLRKLFGEASVPDPVAYVVTDWGRDPFSYGAYSYVAIGASGEDYDILGRPVDNCLFFAGEATCKEHPDTVGGAMMSGLREAVRLIDLFSTGNDYTAEVEAVEAALKQSDTEREEVRDIIKRLDAAELSNILYKKSLDGARILSRETLLRDLFLNAKTNAGRLHVAKQLLRLPVENLKSFAGSKRGLSILNSWILDSMGKDGTQLLRHCVRLLVLVSTDLLAVRSSGIGKTVKEKVCVHTSRDIRAIASQLVSVWLEVFRKEKASSGGRKLSRQANTVDISKRKSINDPASGKPPLSMHHNTFEDKGSILTPATNSASIAHMKKSHGKQGSQQAANDLRRDVSSSKSQGSTGKIDTEMKDTHCAMSEEEKAAIAAAEAARAKALAAAEAYTSTEVRSNTQLQLPKIPSFHKFKSREQYSQNGESDIRKKRSGGILLGKQDCISEIDSRNCRVRDWSVDFSAACVNLDNSRMSADNLSQRSHSNEIASHLNFREHSGESVAVDSSLYTKAWIDSADGEGIKDYHAIERWQSQAAAAGSDFSNPAIHLKHEEDSNACSRLPSWKHDGVANDSSISQVTVNKENSKSHSHNSKSHSRGADRIKQAVADYVASLLMPLYNAKTLDRDAYKAIMKKSTAKVMEQSTDADKAMTVHEFLDFKRKNKIRPFVDKLIERHMEMKPDVKS

>Kalax.0141s0053|Klaxiflora_v1.1/1-1792

MGEEEKVSVPQEKSETPFGGADGSDDDGPIGLFLKMKKSRKAKEEKMGLGEGEVKVKNVAQKVKEPAADQKLGGMDDTLANLRKKLRGPKKDNRFVPSASIELSDEVSGKLVNAKSTSKALEIGFVAGDEKSDAVVDSFLAPKRKKKSKKPKTGSPRTVHTESAHDDTECDISGDRLLQIGETDIVCRKRVSKNPSGAAVMKIPSSAVSRKPQSTYIKRSKKNLPNDVTGETVLIEVNPLANGFAIPVSASYENSDGKNFETRLNKPISVSEHKACSATGTCATTNMKLDVAVEANGWVNETQAQTVEAISSSLVLDKDNETLSKSDTCADDDFLASTVNPMELEYNRVLNGNNLKETIKVNKQMDSPKVVPHECADEEADRGSSPSPNIDQSDSTDALSDLDLKRSSVQRERRNKKKRRHWDMAYEGDADWDTLISAYNYHDRNNGLKTRKREKIGIFFESENGEAAAIAAGLKARKVGPIEKITFKEVLKQKGGLQEYLECRNHILGLWSKDISRILLLSDCGVTDAPSKDEHLLAPLIREVYAFLDRSGFINAGIAIEREKKSPNVTDKNYFIKEKSFLKPLVTSVADMEDCVSFIVDKVEDRKAPMEVKVEYVADDQIHAPNSTRGTSLHTTAEEPVILQECREYLANDHRIQLTKDRKSPRRMLDMYPMTTNSHKETLDKHLDSGPEAIRCPSDVGPTSMNYDVLNHHGDCDSVVRKRVIIIGAGSAGLSAARHLQRQGVSVIILEARSRIGGRVYTDRSSLSVPVDIGASIVTGVEADVDSERRPDPSSLICSQLGLEFSILNSDCPLYDTVTGQKVPSDLDETLEDEYNSLLDDMVLLVAQKGQHVMKMSLEDGLEYVLRKRREAQSRTDIRLFLQKLRGSCFDSAGVGDDCESPANEKSDDDILNPLERRVMNWHFAHLEYGCAALLKDVSLPYWNQDDVYGGFGGAHCMIKGGYSSVVESLAEGLQIRLNQVVTDISYNIPDYPGETGHGPSRVKVFTANGDEFSADAVLVTVPLGCLKAETIKFQPPLPQWKRSSIQRLGYGVLEKVVLEFPKVFWDDSVDYFGATAEETSQRGQCFMFWNLKKTVGAPVLIALLVGEAALKGQSLNSIDHVNHAVGTLRKLFGEDTVPNPVASVVTDWGRDPFSFGAYSYVAIGASGEDYDLLGRPVENCLFFAGEATCKEHPDTVGGAMMSGNREAVRILDIFTTGDDHTAEVEALEAAQRHSDTERDEVRDIKKRLEAVDLSNALYESSFDGTQILTREALLRDLFSNVKTTAGRLHLAKVLLTLPAEKLRTFAGTQQGLTTLNSWMLDSMGKDGTQLLRHCVRILVLVSTNLLAVRLSGIGRTVKEKVCVHTSRDIRAIASQLVSVWLDIFRKEKASNGGLQLLKQTANASKSRSLKGVASGKPPLRTHQGTPESSLMVKSTGCRVHPNANNKKLNNKMVKMENESKSDILSSRSDSSTVRLETKDEEKTYVMSEEEQAVLAAAEAARAAARAAAEAYASSSVKSSVLLPKIPSFHKFARREQYAQMDESEFRKKWSGGVLGRQDCYAEIDSRNCRVRDWSADFSAACPNIDSSAISVNKADYHSQSNNNAYHMNFREHSGESAAPNRNLFTKAWVDGGDSEVLKDFHAIERWQSQAAAADAHFYQQAMHEKDEDGSNVTSRLVPKKNEGMANESSVSHLTTNKHLDNRAKGAEQMKQAVVDYVASLLMPLYKARKIDREGYKTIMKKTAAKVMEQTSDAEKAMSVHEFLDFKRKNKIRAFVDTLIERHMSVKSAGNH

>Kalax.0150s0034|Klaxiflora_v1.1/1-1792

MGEEEKVSVPQEKSETPFGGADGSDDDGPIGLFLKMKKSRKAKEEKMGLGEGEVKVKKVAQKVKEPAADQKLGGMDDTLANLRKKLRGPKKDNRFVPSASIELSDEVSGKLVNAKSTSKALEIGFVVGDEKSDAVVDSFLAPKRKKKSKKPKTGSPRTVHTESAHDDTECDISGDRLLQIGETDIVCRKRVSKNPSGAAVMKIPSSAVSRKPQSTYIKRSKKNLPNDVTGETVLIEVNPLANGFAIPVSASYENSDGKNFETRLNKPISVSEHKSCSATGTCATTNMKLDVAVEANGWVNETQAQTVEAISSSLVLDKDNETLSKSDTCADDDFLASTVNPMELEYNRVLNGNNLKETIKVNKQMDSPKVVPHECADEEADRGSSPSPNIDQSDSTDALSDLDLKRSSVQRERRNKKKRRHWDMAYEGDADWDTLISAYNYHDRNNGLKTRKREKIGIFFESENGEAAAIAAGLKARKVGPIEKITFKEVLKQKGGLQEYLECRNHILGLWSKDISRILLLSDCGVTDAPSKDEHLLAPLIREVYAFLDRSGFINAGIAIEREKKSPNVTDKNYFIKEKSFLKPLVTSVADMEDCVSFIVDKVEDRKAPMEVKVEYVADDQIHAPNSTRGTSLHTTAEEPVILQECREYLANDHRIQLTKDRKSPRRMLDMYPMTTNSHKETLDKHLDSGPEAIRCPSDVGPTSMNYDVLNHHGDCDSVVRKRVIIIGAGSAGLSAARHLQRQGVSVIILEARSRIGGRVYTDRSSLSVPVDIGASIVTGVEADVDSERRPDPSSLICSQLGLEFSILNSDCPLYDTVTGQKVPSDLDETLEDEYNSLLDDMVLLVAQKGQHVMKMSLEDGLEYVLRKRREAQSGTDIRLFLQKLRGSCFDSAGVGDDCESPANEKSNDDILNPLERRVMNWHFAHLEYGCAALLKDVSLPYWNQDDVYGGFGGAHCMIKGGYSSVVESLAEGLQIRLNQVVTDISYNIPDYPGETGHGPSRVKVFTANGDEFSADAVLVTVPLGCLKAETIKFQPPLPQWKRSSIQRLGYGVLEKVVLEFPKVFWDDSVDYFGATAEETSQRGQCFMFWNLKKTVGAPVLIALLVGEAALKGQSLNSIDHVNHAVGTLRKLFGEDTVPNPVASVVTDWGRDPFSFGAYSYVAIGASGEDYDLLGRPVENCLFFAGEATCKEHPDTVGGAMMSGIREAVRILDIFTTGDDHTAEVEALEAAQRHSDTERDEVRDIKKRLEAVDLSNALYESSFDGTQILTREALLRDLFSNVKTTAGRLHLAKVLLTLPAEKLRTFAGTQQGLTTLNSWMLDSMGKDGTQLLRHCVRILVLVSTNLLAVRLSGIGRTVKEKVCVHTSRDIRAIASQLVSVWLDIFRKEKASNGGLQLLKQTANASKSRSLKGVASGKPPLRTHQGTPESSLMVKSTGCRVHPNANNKKLNNKMVKMENESKSDILSSRSDSSTVRLETKDEEETYVMSEEEQAVLAAAEAARAAARAAAEAYASSSVKSSVLLPKIPSFHKFARREQYAQMDESEFRKKWSGGVLGRQDCYAEIDSRNCRVRDWSADFSAACPNIDSSAISVNKADYHSQSNNNAYHMNFREHSGESAAPNRNLFTKAWVDGGDSEGLKDFHAIERWQSQAAAADAHFYQQAMHEKDEDGSNVTSRLVPKKNEGMANESSVSHLTTNKHLDNRAKGAEQMKQAVVDYVASLLMPLYKARKIDREGYKTIMKKTAAKVMEQTSDGEKAMSVHEFLDFKRKNKIRAFVDTLIERHMSVKSAGNH

>Kalax.0054s0112|Klaxiflora_v1.1/1-1780

MGEEDKDSVPQGKSEPAGVADSDDDGPIGLFLKTKKSRKAKEEKLGLGEGEGRVKKVAQKIKDPSGDEKFGGMDDTLASLRKKLRGPKKDNKLSTNLKIAPLASVELSDGVSGGVVNAKSTSKALEIGLRVEDEKSDAVADSCLAPKRKKKSKNPMTGSPPALNTEITPDGSKFDISGDKLLQIGKKNVRRKRMSKNPSGAVVMKYSSSVAVQKPQSACNKISKNNMPNDCETMLVEVNPLAISDSHENTDGKNLEATLNEPLSVSGLQFCSATVACAMTNLKLDIAGEANGWVNETQVQTVEVITSSLVPDEDNERHSKSDIFADDNLLASTVNPMELEDNHALNGTNLKETVKLNKQIDSSKVLPCESADDEGDRGSSQSLNLDPSDSTDAVSNVGLKRSSVQRVGRNSKKRRHWDMAYEGDADWDTLVSAYNNHDHNDGIKTGKREKIGMFFESENGKAAAIAAGLKARKAGPIEKIKFKEVLKRKGGLQEYLECRNHILGLWSKDISRILLLSDCGVTDAPSKDEHLLAPLIREVYAFLDRSGFINAGIAIEKEKKVPNMTDKDCLIKEKNLKPLVTSVANMEDGVSFIVGKVEDQKAPIEVKVESSADDQIYAPNSAKGISLATAAEESISLKERSDELADDHRMEHTKDGKSPGRMLDLDPMTTYSHEETLDQNLESGPEAIQCPNIGPTFMNHVDTNHHVHCESDVNKRVIIIGAGSAGLSAARHLQRQGVSVIVLEARSRIGGRVYTDHTSLSVPVDIGASIITGVEADIDSERMPDPSSLICSQLGLELTILNSDCPLYDTVTGQKVPSDLDETLEDEYNSLLDDMVLLVAQKGQHVMKMSLEDGLEYVLSKRREAQSVTDSTLFIQNTRGTCFDSARGSDDSESLAEKRSIDDFLSPLERRIMNWHFAHLEYGCAALLKEVSLPYWNQDDVYGGFGGPHCMIKGGYSSVVESLAAGLQIHLNQVVSDILYDIQNSGETSDERSRVKVLTSNGDEFSADAVLVTVPLGCLKADTIKFQPPLPQWKRSSIQRLGYGVLDKVVLEFPEVFWDDSVDYFGATAEETRQRGQCFMFWNLKKTVGAPVLIALLVGEAALVGRSLNSTDHVNHAVGTLRKLFGEDTVPNPVASVVTDWGRDPFSYGAYSYVAIGASGEDYDLLGRPVENCLFFAGEATCKEHPDTVGGAMLSGLREAVRILDIFTTGDDHTAEVEAIEAAQRHSDTERDEVRDIKKRFEAVDLSNALYESSFDGTQILTREALLRDLFSNVKTTAGRLHLAKELLTLPVEKLRSLAGTNEGLATLNSWMLDSMGKDGTQLLRHCVRILLLVSTNLLAVRLSGIGRTVKEKVCVHTSRDIRAIASQLVSVWLDIFRKEKASNGGLKLLKQTANPSKSRYLKGVASGKPPLRTHQGAPDSSLMVSSTGSHMRPNANIKKLNSKMIKIENESKSDMLSSRSDSSTVRLENREDEENYVMSEEEQAALAAAEAARAAAEAYASLSVKTNVVLPKIPSFHKFARREQYAQMDESEFRKKWSSGVLGRQECYAEIDSRNCRVRDWSVDFSAACPDFDGSAMSVDKADYHSQSNDDAYHANFRELSGESAAPNRNLFTKAWVDGAGSEGLKDFHAIERWQSQAAAADANFYQQAMHEKDEEDSNATSRSAPANHGGRANEGSVSQVTVNKHLENMPKGVEHIKQAVVDYVASLLMPLYRARKIDRDGYKAIMKKTAAKVMEQTSDAEKAMPVHEFLDFKRKNKIRGFVDTRIERHMTMKSAGNH

>XP_020572869.1|Phalaenopsis_equestris/1-2045

MGDEVEGESKRKSLSAVESLAVPDEEDERPIGSLFNLRKSRGVKKGRPLLAQSRVENPSVKMLDSGGLDATLASFRKNIRGLKKVKDEAGAELCGDVVRGSPPELMGGIASPAKVVLTGKPQTWRSKRAISKGDGYVGDLDLLGIPDGSSDSSFEDSLSAYIQRAQSSAGSGVKSAKAPSKQIARNLGSVVLSKDSSDGEKKHPRGARLGRKCTAANPAEVTVVAGFGGISDQVSKEESILAGFDRETNPLLKLRKRGRSRKSDRAAKNCASGDLINRDENMITGFAGEANPLLALKRKARIGKSDRALKDSTYERMITSPLISTVEVKVDLDKIEKSGVDKVSQEILENSAVACFDLDINKLNTGGEEGRVSNLSGHENGCHPNSSGFVDELRCCQVKEEQQSLHDGLQHCSNGSTSRKNEIVELYPSGLCPPRTCRIAESNQASHTVDEVNQNLMMWTSEPSGFQKEAADTSLSFVQGSCSLQLTIIPEDFSGSTDCKSQALPPSILQENDATSFGDCMLNDTCIEAPSLKAGTMLQCTSVPFDETTGKPSAYSEKLLELSGETIVAPIIQKTLIESVDEGFTNQPIVVAPKEQFLVTSEVLSIANVVDASLQNAVLTNGIQHLEESKQPDSGHLEHEPTVPRALRNVKKRKHGDMAYEGDDGWEVLMHEQGLFTNISIDDYHSTTNLEKPSSSEILCDVSDGGASAVAAGLKANTLSPIEKIKFKDVIKRKGGLQEYLECRNLILRCWSKDVKHILTLEDCGLSESSLEDEHSRISLNRDIYKFLDQHGYINSGIAAERGPKNSASLVSEPLKESKSKRTCDLKNTSSEDEAAYLVGEVEGSENMNGMVNTVICQAQPKKRTSEEEANEDKDPFESSRVSKSSVQANIGENVWGQNEPVFASSDIQSFDCTCQERDYYCDKVTTSFCAVHPWKSEGHNMVELDASKTIRMVDPVKSHVSMKQKTSEGSGSVEFNSSLLRKIVVVGAGPAGLTAARHLQRQGYYVTVLEARGRIGGRVYTDRTSLSVPVDLGASIITGVQADVATERRPDPSSLICTQLGLELTILNSDCPLYDIVTGHKVPTDLDEALEAEYNSLLDETLLFISEHGENAMNMSLEDGLEYALKRCRMALSTSSVAKAEQVNVASAKNGTTCSGGVVQKTDILSPLERRVMDWHFANLEYGCAASLKEVSLPYWNQDDVYGGFGGAHCMIKGGYGAVLDCFAKGLDIHLNHVVTEIFYDINDPNGTDQHSNKVKVCTSAGQEFLADSVLITIPLGCLKANTIKFSPPLPDWKESSIQRLGFGVLNKVVLEFPSIFWDETVDYFGATAEETDSRGRCFMFWNVMKTVGAPVLIALVVGKAAVYGQNLSSADHVNHALSVLRKLFGEASVPDPVAYVVTNWGVDPFSRGAYSYVAVGASGEDYDFLGRPVANCLFFAGEATCKEHPDTVGGAMMSGLREAVRINDILSTGKDYVLEVEAMEDMQSRSDSERNEVRDLCKRLDACKLFHYACGSSSDGTRKLLAEEALLQDMFISAKTTSGRLHLAKAMLSLPVEVLKSFAGNKEGLGILNSWILDSLGKNATQLLRHCVRLLVLVSTDLLAVRLSGIGKTIKEKVCVHTSRDIRAIASQLVSMWIEVFRKEKAANGRLKLLRQTAVSEQSKFRSKDQKLVTTPLCRGNEAMDSRGILTPPSSAGNHSPSKTKSRKTDKAGKLELAERKLSDTRRKFQKLDSEMGNVTTPSVEAAALDAAEAARASAIAAAKAFASSEAEMSTFRELPKIPSFHKFARRDQYMQLDDSDIRKRWSGGVSGKQDCMSEIDSRNCRVRDWSLDFTSTCRNFESSRLSDDMNQSLSNEIECPLNFGEQSGESGTIDSRLTKAWVDTDSASNGAVKDSSAIERWQLQAMDADVEFYNRVHLRDEEDSNRRQKLQALKQHESSDRMSASHVAENKSSCEGHSRGVEHIRQGVVDYVTSLLMPLYKTRKIDKDGYKSIMKKTATKVVEQCTETEKVMNAYEFLDFRRKNKIRAFVDKLIEMHMATRPPANS

>Soltu.DM.04G036220|Stuberosum_v6.1/1-2079

MGEEENVKLGLERGTKKRIEMKFDSGDDEPIGSLLKLKSKKHSKKAKVDLGGSKDVIQKTVVKGEDLVGMDDTLASFRKKLRGPKKNSGSVSTIVKSSSSNASKLTGESPDGSVKVAAKIVEMSLSDVECLSEGIIDKGFEKGNKRKGKRPKVSSELKKVEISGDMSLQNDKECGKSSPNCMDGILEDSLSAFLKKAQSGFIKKSHSSLQLKRGKESEVLCDVLNSSPTATEIFPSISTNMCQKLGEEIPESNEDVHVSLDGGSVDMHLSENKEFVQFIQSQSDSRPELSLSALNNVELLKSSIAIDDASSIEGSQLDVPACFNKIAGVLDGEVKCHSKLSEEGTATTNNIVGGNCKDLHDEDVMKNCSTFHEQSASKDGFSDRPMATGRDVSSADTITPENVEMLEHPMIKTKFNADMDVNSNAILSSRETHVDDQMCSSNRADDSGSCRSVQLLNKLDHTSQGSIGNVFSRTLISSTSRLEGSTAAKENTDMEGSGYAQVRLMPDFIAAEKCSSDFDDQQRISVDSVYEQACAPVSLPKEDGQVFVGGSSPVSIGRSQQVNVSQMKQEDQIMENSNDLYGSSKQMTIDNAAISLRKCSSVFHQSELADENCEGSHHQSRVFVSGDDEADASSPSITPECDESVAEETESKLAAEEKEQRLFSGQRASRKTKKRRHGDMAYEGDVDWDVLVHGQDFFLSHQDGEGRHDFKTREKLDSSLIVMDTENGGVAAVSVGLKAREVGPVERIKFKEVLKRRVGLLEFLECRNQILSLWNKDISRVLPLSECGVSETPLADESPRASLIRQIYSFLDQCGYINFGIASEKDKAENGVEHNLKILKEEKTIEKSGASVADRDDGVSFILGRSKSSEIIMPEKNDVLSDEGKKTEKCGADCQLIDRRAIELPALAEPRECPVDDCRVNGYPDIQSPRQPFDLGLVAQVPSSEVKDSELQNIVDPDLLPPNNTEIDVRAADKHLLISEDSCGFTPDSLGSQRLNTCCDAKGKKEIIVVGAGPAGLTAARHLKRQGFHVTVLEARSRIGGRVFTDRLSLSVPVDLGASIITGIEADVATERRPDPSSLICAQLGLELTVLNSDCPLYDVATGQKVPADLDEALEAEFNSLLDDMVLLVAQKGEHAMRMSLEDGLEYALKKRQKARSARNHMGNEPQKSSVTAVESTALSDGGVPQNNNSKVEILSPPERRVMDWHFANLEYGCAALLKEVSLPYWNQDDAYGGFGGAHCMIKGGYSSVVEALGEELCVHLNHIVTDISYCKEDVPSKNDLFNKVKVSTTNGREFSGDAVLITVPLGCLKAEAIKFSPPLPHWKDLSIQRLGFGVLNKVVLEFPEVFWDDSIDYFGATAEDTDERGRCFMFWNVKKTVGAPVLIALVVGKAAIDGQEMSSDDHVKHSLLVLRKLYGEEKVPDPVASVVTNWGKDPYSYGAYSYVAVGSSGEDYDILGRPVENCLFFAGEATCKEHPDTVGGAMMSGLREAVRIIDILTTGTDYTAEVEAIEDAKRHSDVERSEIRDIMKRLEAVELSSVLCKESLDGVKIVTRENLLRDMFCKANTTAGRLHLAKELLKLPVEVLRSFAGTKEGLSTLNLWMLDSMGKDGTQLLRHCVRVLVLVSTDLLAVRLSGIGKTVKEKVCVHTSRDIRAVASQLVNVWIELFRKEKAANGGLKLLRQSTATDTLKSKHIAAPGKPPIRNHPSAVDSKRSSKVSSSAGNHLAVSVNNKKLNVRPATLGTIPVVEPSTSQASVGRQNDTTKERQNFPMSEEEKAAFAAAEAARLAALAAAEAYASSGAKCNMPLQLPKIPSFHKFARREQYANMDESDIRRNWPGGVGGRQDCLSEIDSRNCRVRDWSVDFSAAGVNLDSSKMSVDNRSQRSLSNDNACQFNFKEHSGESAPVDSSIFTKAWVDSSNSVGIKDYNAIEMWQCQAAAANSDFYDPVMHVTDEEDSNVSSKMDMRKHDVLVCESSASQITVNKEALDNQPRGAERIKLAVVDYVASLLMPLYKARKLDREGYKSIMKKTATKVMEHATDAEKAMLVYEFLDFKRKNKIRDFVDKLIERHIQMKPGAKS

>Solyc04G002869|Slycopersicum_ITAG5.0/1-2078

MGEEENVELRLERGTKKRIEMKFDSGDDEPIGSLLKLKSKKQSKKAKVDLGGSKDVVQKTAVKDEHLVGMDDTLASFRKKLRGPKKDSGSVSTIGKSSSSNASKLTVESPDGSVKAVAKIVENGLSDVECLSEGIIDKGFEKGNKRKGKRPKVSSELKKVEISEDMSLQNDKESGKSPPNCMDGILEDSLSAFLKKAQSGMFKKSHSSLQLKRGKESEVLCDVLNSCPTATEIFPSISKNMCQKLVEGMPESNENVHVALDRGSVDMHLSENKEFVQLIQLQSDSRPELLSSALNNVELLKSSIAIDDTSSIEGSQLDLPACFNKIAGVVDGEVKCHSKLSEEETATTYNIVRGNCKDLHDEDVLKNCSIYYEQSASKDGFSDRPMATGRDVLSAGIITPENVEMLEHPVIETKFNADMDVNAILSSRETHVDDQMCSSNRVDDSGSCRSVQLLNKLDHTSEGSTCNVFSRTLISSTFRLEGLTAAKEKTDMEGSGYAQVRLAPDFIAVEKCSSDFDDQQRISGDSVNEQACSPVSLPKEDGQVFAGGLSLVSIGRSQQVNASQMKQEDQIMENDDDLYDSSKQLTIDNAATSLRKCSLVFHQSELADENCEGAHHQSRVFVSGDDEADDTSSPSITPECDESVAEETEAKLAAEEKEQRIFSGQRASRKTKKRRHGDMAYEGDVDWDVLVHGQDLFSSHQDGEGRHAFKTREKLESSLTVMDTENGGIAAVSVGLKAREVGPVERIKFKELLKRRVGLLEFLECRNQILSLWNKDISRVLPLSECGVSETPLADESPRASLIRQIYSFLDQCGYINFGIASEKDKAENGAEHSLKILKEEKTIEKSGASVADRDDGVSFILGRSKSSEIIMPEKNDVLSDEGKKTEKCGADCQLIDRRAIELSTLAEPRECPIDDCRVNGYLDIQSPRQPFDLGLVAQVPSSEVKDSELQNIVDPGLLPPNNTEIDGRAADKHIVISEDSCGFTSDSLGCQSLNTCCDAKGKKEIIVVGAGPAGLTAARHLKRQGFHVTVLEARSRIGGRVFTDRSSLSVPVDLGASIITGIEADVATERRPDPSSLICAQLGLELTVLNSDCPLYDVATGQKVPVDLDEALEAEFNSLLDDMVLLVAQKGEHAMRMSLEDGLEYALKKRQKARFARNHMGNESQKLSVTAVESMALSDVGVPQNNNSKVEILSPPERRVMDWHFANLEYGCAALLKEVSLPYWNQDDTYGGFGGAHCMIKGGYSSVVEALGEELCVHLNHIVTDISYCKKDVLSNNDLFNKVKVSTTNGREFSGDAVLITVPLGCLKAETIKFSPPLPQWKDLSIQRLGFGVLNKVVLEFPEVFWDDSIDYFGATAEDTDERGRCFMFWNVKKTVGAPVLIALVVGKAAIDGQEMSSDDHVKHSLLVLRKLYGEKMVPDPVASVVTNWGKDPYSYGAYSYVAVGSSGEDYDILGRPVENCLFFAGEATCKEHPDTVGGAMMSGLREAVRIIDILTTGTDYTAEVEAMEDAMRHSDVERSEIRDIIKRLEAVELSSVLCKESLDGVKIVTRENLLRDMFCKANTTAGRLHLAKELLKLPVEVLRSFAGTKEGLSTLNLWMLDSMGKDGTQLLRHCVRLLVLVSTDLLAVRLSGIGKTVKEKVCVHTSRDIRAVASQLVNVWIELFRKEKAANGGLKLLRQSTATDTSKSKHIAAPGKPPIRSHPSAVDSKRSSKVSSSAGNHLAVSVNNKKLNVRPATIGAIPVVEPSTSQASVGRQNDTSEETQNFPMSEEEKAAFAAAEAARLAALAAAEAYASSGAKCNMPLQLPKIPSFHKFARREQYANMDESDIRKNWPGGVVGRQDCLSEIDSRNCRVRDWSVDFSAAGVNLDSSKMSVDNRSQRSLSNDNASQLNYKEHSAECAPVDSSIFTKAWVDSSNSVGIKDYNAIEMWQCQAAAANSDFYDPVMHVTDEEDSNVSSKMDMRKHDVLVCESSASQITVNKEALDNQPRGAKRIKQAVVDYVASLLMPLYKARKLDRDGYKSIMKKTATKVMEHATDAKKAMHVYEFLDFKRKNKIRDFVDKLVERHIQMNPGAKS

>evm.TU.Scaffold_522.1034|Carabica_v0.5/1-2030

MEGEEKNKVGLKKRVKRIEIGIDSDDDEPIGSFLKLKSKRNPKKNKVVSDGEDKGKGVDKLSVEDQDLVGMDDTLASFRKKLRAPKKEGGPALVVGGGLGSTVAEPSIQSLTKPGLGDLVSKVIEKVKVDDDGYTVDKGLGLGARMKDRRPKVAAVSKDVSTDSASNNISDCQPSGEGSCENEKDVDLGLGDCPSNCLEEGLEDPLSAFFRKAQSGFIRKSCNSLRLKSGNETQISRNGVSEDIMPESTSKSRSASKLANEIAESKQYSHLASGRGKIGLTVGADKDDEILQRKFSSRSSDDVDDTSSERISYNLVPMSKIQGSQSSLRGCSDTFMQVQGAGLTSHTQISCRSEHCSGERTSALLHAMEVPVAVSVSEGLENHHTDEGPLARVHNNEQNLRSDFTSSNDASNLEQRTCNSVGKQFLQVCLDHDSLNRSCEDKFPSSIRKVCGETLELSISQMGDETTFAGKMDTADDSNSGPPGQYSAQAHVFSTADSPNKISNHDMEICTAEPDTASVSSEKEDVMEGSLSPASACGITQYEFAPRVKQQDGPLRHVGEADQLSQCRTPNDSLILNDKCSSGFYQNKPFDGVSKDASFPSLDYFSAEEEVKGASSPSDVPDSNDSYAEDAGLFPDPDNKTNSTEVGGRRKPRKRRLGDMAYEGDADWEILIHGQDFLIDRQVGDDFQSSTAREKLSSLSNTSETENGGAAAISAGLRAHAVGPVEKLKFKEVLKRKGGLQEYLDCRNNILSLWNKDVSRILPLSECGVSDTALVDESPRASLVRDIYAFLDQWGYINFGVALEKAENGSAHNLKLLKEEKFVERSGAPVADANDGVCFILGRIRDPESSKMEKNDTALGDEKQVMAKSQLDEGHINLQAAEISAQTDHEGFPAVNYEENGVFDAKIPGETVSSDYLGSNPSSEDEKSRILPVENPDSFPTSEAQVGRLLSCGLSQLEKDSNRQPSSCDDQSHFGICDLDTRKRIIVVGAGPAGLTAARHLKRQGFHVTVLEARSRIGGRVYTDRSSLSVPVDLGASIITGVEADVATERRADPSSLVCAQLGLELTVLNSDCPLYDTATGQKVPADVDEALEAEYNSLLDDMILLIAQKGENAMRMSLEEGLEYALKRRCMARFGRGKNLMNTELPKSLDAVMAFEKFSTDDEVPQGDSGETEILAPLERRVMDWHFANLEYGCAALLKEVSLPHWNQDDDYGGFGGAHCMIKGGYGTVVDSLGEGLSINLNHVVTDIIYGQTDGMSNDDKSKKVKVCTSNGSEFSGDAILITVPLGCLKAETIKFSPPLPQWKYLSIQRLGFGVLNKVVMEFSEVFWDDSIDYFGATAEETSQRGWCFMFWNVKKTVGAPVLIALVVGKAAMDGQKMSSSDHVNHALFVLRKLFGEMAVPDPVASVVTDWGQDPYSYGAYSYVAVGSSGEDYDMLGRPVENCLFFAGEATCKEHPDTVGGAMMSGLREAVRIIDILNTGTDYTAEAEAMEAAKRHSDIERSEIRDIMNRLEAYSLDQTQILTKKSLLQDLFYSAKTTAGRLHVAKQLLKLPVQVLKSFAGNKDGLSMLNSWMLDSMGKDGTQLLRHCVRLLVLVSTDLLAVRLSGIGKTVKEKVCVHTSRDIRAIASQLVRVWIEIFRKKKASKLRQPTGVDSFKNRSSQALGKPPLRTNHIAPENRGSPKVSSSRNHLASSSNVIRPTVEAKPSSSEGSVERQNTTGEETKEKEEKAAFAAKEASLAAALAAAKAYASSGAKSGLSLHLPKIPSFHKFARREQYSRMDDADIRRNWSAGAFGKQDCLSEIDSRNCRVRDWSVDFSATGVNLDPSRMSVDNHSQHSQSNEIGCQLNFREHSGESVAVDNSLFTKAWVDSAGSVGTKDYNDIERWQCQAAAANSDFYHQTMHLTDEEDSTLKLPAKKPDGPANESSVSQVTVNKELVKNQLRGAERIKQAVVDYVASLLMPLYKARKLDKEGYKTIMKKTATKVMEQATDAEKAMAVSEFLDFKRKNKIRAFVDKLIERHMAMKPAVKS

>Sapof.06G058100|Sofficinalis_v1.1/1-1826

MDFEEKKAGLKKRSKPGEFKLDSDDDEPIGSLLKLKRRSSKKLKLGVDGVKDKKKDEDFGGMEDTLASFKKKLKGPKKDKGCATLAKTPSNLDANDAGLVANLDAEIAEARSDVNVSKSSDKICVVKVKRSKSAPHAKNQMKSNEVVDDKVADEQEEGKDDTCDASDVLVESLSSFLNKKLRAVSGLKMRKAVQVSQTDPCPSSNNLMVDFQEESLLDTNLCSDSYESKECVPGSCEERSIAPQNKSAGPNNVAVEYPKCTNSLSADGNTRPDDNRSANCFETISDHPIPDILTASSEKIEDFKSSEVCVEDKMIQPKPVMDSPDELCDTGLCKLSAVDVRDNRASNYQDVALHSSVKQFVSPDASSSGAPCSTPTSRLMGDLTEPEKLESVVRSDDGCMQHGLNTARVVDQDRKDDVPLQTGVNGKANGSSSPSIAPNENGDFLENSSHCTERAVRRVKKRKDGDMAYEGDTDWEVLMHDRYLPYSYPAVDINHNAPSKDKFYSSNADPDIEIAAAAAAVSVGLKARALIPVEKIKFKGVLKRKGGPQLYLECRNKILSLWSKDVTRILPLTDCGISSATSDKESFRVSLMRDIYSFLDRSGFINTGIADNKDKADPSSRGSYKLLGEKKFEGSSGASIADSADGVSFILGQVESSDTPPKAKGYIASANVCPFKEGLDTLPKAKDDFALADKNHFKEAVENIEEKVIDTSTDKQLDRQGVGEFGKQLVDKKPSEIVSEPDCSANLIGRRLEHGEGLSPSMMRGHLKGNCSTECDLINQKRVIVVGAGPAGLIAARHLQRQGYLVTVLEARSRIGGRVHTDHSSLSVPVDLGASIITGVEADVASERRPDPSSLICAQLGLELTILNSDCPLYDVVTGQKVPPEIDESLEAEFNSLLDDMMLVVAQKGDNATRMSLEDGLEFGLKIRRAVLHQDNVKSELLEPLAGSHSHGNDMESRLSADDNGVKGGTLSQIERRVMDWHFAHLEYGCAALLKDVSLPHWNQDDVYGGFGGAHCMIKGGYSAVVESIAQGLPIYLNQVVSDISYGVTDLGITDNCSYKVKVSTSNGDEFFGDAVLITVPLGCLKADSIKFSPPLPQWKHSAIQRLGFGVLNKVVLEFPGIFWDDTVDYFGATAEHTDLRGRCFMFWNVKKTVGAPVLIALVVGKAAVDGQDISPSDHVGHALTVLRKLFGETAVPNPVASVVTDWGRDPFSYGAYSYVAVGASGEDYDIIGRPVDKCLFFAGEATCKEHPDTVGGAMMSGLREAVRIIDILTTGDDYIAEAEAEAVDVGQGQSSNQRNEVKDIARRLDAIDISDTGFTRESLLHDLFFTAKTSAGKLRLAKELLSLPVESVKSFAGTKEGLTILNSWILDSLGKDGTQLLRHCVRILVLVSTDLVAVRLSGIGKTVKEKVCVHTSRDIRSIASQLVTMWIEVFRKEKATNGGLKLLRHMTNADSSKAKGKDPSSRKPPKRAQHCGAGNKVNLGFKKLTGKQVKPETPSISQSSKAGPDSKSDVDEDVPLSEEERAAIAAAEAAHAAALAAIEAYASTEARSSMMQLPKIPSFQKFARREQYALMDDEERKKWSGGVLGRQDCISEIDSRNCRVRDWSVDFSATCANLENSRLSADNLSQRSNSNDVPCQLNWREHSGESAAGDNTDFTKAWVESSGSDVLKDYHAIERWQCQAAAAEYEFQSSMHVQDEEDSSSMSRLVAQKHERLVNESSVSQVTVNNELAVNQRRGTEHIKQGVVDYVASLLMPLYKARKIDKEGYKSIMKKSATKVMEQTTDAEKAMSVSEFLDFKRKNKIRAFVDKLIERHMSTKQAPKL

>GWHPBFSI013188|Chloranthus_spicatus/1-2082

MNDERSVFGNEEMDKEGSHASLDFNGERKLEQGLASPNPRSDDVEENKKFGFVNKSKLIEMDRDSDGDDDDDDNEPIGFLFKFAERNDMKRRRNGSDEEGVGDMPENFRVGRKNSGEMDNTLASFTKKLKVPKMLKDGESDAMAADEDSGLNGIGHSNQSSNVSKCCADERLEKPKFRGKISLRSLMKKKVEDSSEIYVGLDRSPGARRSHSTYLKEPKLGIRKNYVCNNPSDKKLEDSLSAFLRKLNSGLFKKARSSTKRKIREGTFTCVDALNGESYGVSRDVSMRVQKSYSGLNQKSNGIQKELLSGSVQEYDFASTPKITEEILRSSNDGRSYVSPDRRPYTVSAPDPLVKFPKPNNDGLCKQTDGIPEGTTLYNDGLNQSCDGDSEDLVSTLIRESCLASFQKLPGEEQRHMDGLNQCSNRSSSNLLHGIVEEQIEEITPSCFGGDGLNLCSVIDNSRSGKPSSSWGETNILTSKQEVPLPDGGFNHLDDGYLKDSQIVQLQTSSFTSDSVEKLTNQIYVDGLNQSADISKEDTTSVAAQKASLAPVTKQITETSKTYVGLHQHSGGVQSVLPCSNVKKPDFASTLNDKEELSVTAELLNKYSDEFSMDSRTLIQNSRSACLQNQSSGDASKGPSSVPFQVSFVKEATVARSQSIVPNVKEIDPDDLGRALDSDEKEIRITAMQRAVRKVRKCQNVVMAYEGDIDWEVLMRGQNFSKNYSVIDGDSFARTKYKSESPSIMLEESGNGGAAAAVAAGLKACAAGPVEKIKFKDVLKRRGGLQEYLECRNFILGLWSKDVSRILPLTDCGVSPEDESRRASLIRDIYTFLDQRGYINIGIASEKESTKSYSTAYFKISKENKPREICGGQATDTEDEVAYILGRTQTSENLATAENDISFEDGKPTSVPVAEAMKSKKLLVPGMGLKSSNLVEFGESGVVNLELNHRACVACVSEPHGSEKIVAAETCSDRTVQEDGSVPNKMDKLDALSVDPLRGIVDGGSTTLFLEHVKRSCGKYEFTDINSKNVMHFKGSGAKSAGISPLEVNCSIQDDLEVSKRIIVIGAGPAGLTAARHLQRQGFSVTVLEARNRIGGRVYTDYSSLSVPVDLGASIITGVEADVATERRPDPSSLLCAQLGLELTVLNSDCPLYDIVTGQKVPAALDEALEAEYNSLLDDMVVLVAQRGKGAMRMSLEEGLEYALKSRRMARSNLASVESDSCNFDRGLKDDILSPFERRVMDWHFANLEYGCAALLKAVSLPYWNQDDVYGGFGGPHCMIKGGYSTIVESLGQGLHIHLNHVVTEVTYGMKDSGGSDGQRCEVKVSTFSGTEFVGDAVLITVPLGCLKANTIKFSPPLPEWKQSSIQRLGFGVLNKVVLEFPGVFWDDTVDYFGATAEETECRGQCFMFWNVKKTVGAPVLIALVVGKAAIDGEHMSTSDHVDHTLRILRKIFGEASVPVPVASVVTNWGRDPFSQGAYSYVAVGASGEDYDILGQPVENCLFFAGEATCKEHPDTVGGAMVSGLREAVRLIEILSTGSDYLSEVEATVAAQQSDGERNEVRDMTKRLDAVELSNVLSKNSYDDAQRLLTKEALLQDMFSSAKTTGGRLHLAKELLCLPVETLKSFAGTKEGLCTLNSWILDSMGKDGTQLLRHCVRLLVLVSMDLLAVRLSGVGRTVKEKVCVHTSRDIRAIASQLVSIWIEVFRKEKASNGGTKLLRQSTTSDSSKAKSKDSGSGKTPTGTISYTSDSRGKLQVTSSAGNRLPSNTRKVDFIKLETLTDAKSKDNSSLTHHVTQSLDSKVVDNVLLSEQEAAAVAAAEAARAAAEAYASSEAESSKLRKLPKIPSFQKFAALEQHVQIDDSDLWRRKLSGGGLGKQDCISEVDSSNCKVRNWAVDFSTTSNKFDKSKTSGDNCTEQSYSNSILCPLNLKEQSGESGAVKSWSTKAWVDREVSGRVKGYDSIERWNSQAITTETDFSHPSLVSQAAENNALVENQPRKTEHIKQAAVDYVASLLLPLYKARKIDKDGYKSIIKKSATKVMAYCTEAEKNMTVSEFLDFKRRNKIRSFVDKLIERHMAMNQM

>Brara.H00853|BrapaFPsc_v1.3/1-1590

MDGKGKRCGSKKESKVFDFEDDADDDEPIGSLLKVMKHKTATKSKVETETTGKQMKKLTHVRKDSEDMDDTLASFRKRLKGHKKRVRNDKDVPPVDTVTDSNLIPSPDMADDASELGREGTVNKKSLDDIYKTKVKSSTRSVILEKEADADDKSKRPLPNVDVYPPVDRKDVSVLSPGQHIHLGEPESESGHFREEKIVVCDCGIQFNFEDRSLESNSEVAICQKCKRSSHRDASNGGGIQVNALKDGTAEASPVSVTPYEDEDFRGDAVSLPNSGKPSTLQRPERIARKRKHENMVYGEDMNWENEQGFLDCQSDKSFKGSDKCDFGPFISKEIEIGRAAAVAAGLKAQSVSPVEKTILKEVLKRKGSHQEYLRCRNSILGLWSKNVSRILPVTECVVGLSESEFPSAYLIREVYKFLDQRGYINTGVSSVRGKGGPSTNHDDDLSQGKKLEESYMASVANSEGDTFIFGQVKAVESTSEGKNCALQNDKTEIVGCATSAMLGSTSKTCEASIIDDCKHSVSINGLPADADAPKVEECPETVSVPKTALSSTPSSANCNQISGRDCVQCEIEDEKKVIVIGAGPAGLTAARHLQRQGFSVTVLEARSRVGGRVFTDRSSLSVPVDLGASIITGIEADVPSERMPDPSALVCNQLGLELSVLHGFCPLYDTVTGEKVPPELDDALQGEFNSLIDDMDLLVEEIGKDRANKMSLEDGLEYGLQRLRMPHEKVNIERFGIGNSINGSFSRTGITGTFKHDGRLKEDFLNPLERRVMNWHFAHTEYGCAAVLKEVSLSNWNQDEFYGGFGGPHAMIKGGYSRVAESLAEGLDIRLNNVVSEVSYTSDVSAMHNNKHKVIVSTSNGGEYLGDAVLVTVPLGCLKAETIKFSPPLPDWKYSSIKQLGFGVLNKVVLEFSKVFWDDSLDYFGATAEETDQRGECFMFWNVKKTVGAPVLIALVVGKAAVDYKDKSKSEHVNHAMMVLRKLFGGDLVPDPVASVVTDWGADPYSYGAYSYVAIGASGEDYDVLGRPVQNCLFFAGEATCKEHPDTVGGAMMTGVREAVRIIDILRSGNDYTAEIETLEKAQRKSVSIRDEVSDLIKRLEVVELSNVLARHSLLRNMFFSAKTTVGRLHLAKELLNLPGETLKSFAGTKEGLTVLNSWILDSMGKNGTQLLRHCVHILVRVTSDLFAVRLSGIGKTVKEKVCAHTSRDIRAIASQLVNVWLELYRKEKANREMKSLRRTNPTNISRIRRKQNSEDADSKGNLSNGNDTTTDGEIEDNQLPMSEEEKAVFATAEAARVAAEAAAKAFSEAYQSTSLQLPKIPSFHKFARREQYAKMDESDFKKKFPGNVLGRQDCMSEIDSRNCKVRDWYDFPASCLDPDSSRVQVDNCSQRSHSNELGSHSKFRECSRESGAAETSVFTGAWVDTGGSSDGVKDYNAIDRWQSQAAAADPEFFNRALHMKDEEGSMACSTGPPSWKHDQPANEGSMSQVTVNKEPRKSHIRGADRLKQGVVDFVASLLMTPYRAKKIDRDVYKSIMKKTATKVMQQTTDAEKAMAVPQFLDSKRKNKIRDFVDKQVDKYIAMAKVAKP

>AT4G16310|Athaliana_Araport11/1-1628

MDGKEKKSGSKRGSKVFQFDDDADDDEPIGSLLEIMKHKSSKKDKVETESTGKQRQKQVVEKKLSALGKDSEDMDDTLASFRKRLKGNKKGVESGTSRVRNHEGVDTVTNSNLKPIEEANKNEVQSVLLRENGASNSIQKCASETGTLLHKFSGKDKAASPSHEKVETVSSEKEADVFHQITKEESEIPMSEKAVELSRVSVPMPDVHGEVNCTIAPDKHIHLGEPTSESGYYREKNLVMCDCGTQFNFEDRSFESNTQVTLCQKCKYSSHHNASNGGGIQVNTLEDGTAQASPVSIIPCEDENFRGDAISLPNSGKPSTLQRPERIAKKRKLGNMVYEGDVKWENEQGFLDCQSDKSFKGSDKCGFVPSISKEIEIGRAAAVTAGLKAQSVSPIEKIILKEVLKRKGSNQEYLVCRNSILGLWSKNVSRILPVTECGVTGGPSESELPSASLIREVYKFLDQRGYINAGISSVNGKAASSTNQDYDLLQGRQLEESSMASVADSEEGVAFILGQVKAVESTSEGKKCALQNDERDLVGCATSEMLESISKKCEASIIDDNKRSVSMNALQDSTASNVEKHPETFSVAKPALSSTLSSAHSNQMRGRDCVPCEVIDEKKVIVIGAGPAGLTAARHLQRQGFSVTVLEARSRVGGRVFTDRSSLSVPVDLGASIITGIEADVPSERMPDPSVLVCNQLGLELSVLHGFCPLYDTVTGKKVPAELDDALQAEFNSLIDDVDLLVEEIGKERANKMSLEDGLEYGLQRLRMPHDKVNIDKFGLLNSSSKTGIRGPFMQDESWKDDFLNPLERRVMNWHFAHTEYGCAAVLKEVSLPHWNQDEFYGGFGGPHAMIKGGYSRVVESLAEGLDIHLNKIVSDVSYVSDVSAMDNSKHKVRVSTSNGCEYLGDAVLVTVPLGCLKAETIKFSPPLPDWKYASIKQLGFGVLNKVVLEFPTVFWDDSVDYFGATAEETDLRGECFMFWNVKKTVGAPVLIALVVGKAAFEYTNKSKSEHVNHAMMVLRKLFGGDLVPDPVASVVTDWGTDPYSYGAYSYVAIGASGEDYDVLGRPVQNCLFFAGEATCKEHPDTVGGAMMTGVREAVRIIDILRSGNDYTAEIETLEKAQRKSVPVRDEVRDLIKRLEVVELSNVLARQSLLRNMFFSAKTTVGRLHLAKELLNLPGETLKSFAGTKEGLAVLNSWILDSMGKNGTQLLRHCVHILVRVTSDLFALRLSGIGKTVKEKVCAHTSRDIRAIASQLVNVWLDLYRKEKANSGKKSLRQANTTNTSRIRRKLNSPDTDSKGKLSNGNDVKTDEEFEDNQLPMSEEEKAVFAEAEAARAAAEAAAKAFSEAYHNTSLQLPKIPSFHKFARREQYAKMDESDFRKKFPGNVLGRQDCMSEIDSRNCKVRDWYDFPASCLDLDSARIPVDNYSQPSHSNELVSHSKFRECSGESVAADTSFLTGAWVDTGGSSDGFKDSQAIDRWQSQAAAADPEFFNRTLHIKDEEDSIACSTGPPSWKHDQRANECSVSQVTVNKEPHKNHIRSADRLKQGVVDFVASLLMAPYRAKKIDRDVYKSIMKKTATKVMQHTTDVEKAMAVTQFLDSKRKNKIRDFVDKQVDKYMVIPQVPKP

>LOC_Os10g38850|Osativa_v7.0/1-1832

MPDIRSAPGRGKSDEDERRPIGSLFKLKRKRRAPGSAEAKGDSNPSVESEAPDGVVPGEMDDTLAIIKRKLRKPKKGKEGGDAVVVGSGAEGELLVEEEDVQGGVNVGDGVAEDKSNLEGVKVEVDEVIGGELKDSGGLGLEDSLSTLFKRSGRKSRQVSVKEEEGVEVAGSHGEEILEKGSGLVSDRVAKGTKRRRRRTKEEMKNAAAKSESAMAHEGSPNRKVGTSLPRKAKAEAKVKISNSNRRSKKSDEKPKASDDVLCHRSLGETIEQDAETRTVLDDGSRNSSDGASHRIEVSACLSNQPCLKPCSGELAEEVSLSAANAATDGVSNEHTYSETLLKERNDDAGCSHGKPPTLAIKSIPGKKPIEMPKKPVRQKDQLLSTDVDNKCVVGSGDTKDVNIENQPAFGIPESHVTGKGLHPHKMATSVKELDVVDVVAPTDFEDMENASKSKRVTRSSRKRKHGDMAYEGDIDWETLMQEQGLFSNLSAALVDYPLKSKDKIKISEVLDNGDGSGVAAVRAGLKAKAVTPIEKIKFKDILKRRGGLQEYLECRNMILSRWCKDVKHILDLAECGVSDVCLDDESPRQTLIRDVYLFLDQNGYINAGIASDKVKTDHESPPEDVEVSKLNESHERKSVSIQDCIVTEAVQDKKAVVKQTDCVLTEASNEESSSAAIHCDAQDLLPPLKSEELIFKEKNQGVLTEGRDESALPSNSDIHSKSDLDGFILKVEGGSLHQAEAADIEHSENKHEASDRVESGGYGKKIIVVGAGPAGLTAARHLQRQGFSVTVLEARNRIGGRVYTDRVSLSVPVDLGASIITGVEADIATERRADPSSLICSQLGLELTVLNSACPLYDVVTGDKVPDDLDTDLESEYNGLLDEMAQLFAQNGESAVGLSLEDGLEYALRKNRVTRSEQDDQLRNVSSAGAVDISESASTEKEIAHCGKEDKTDVLSPLERRVMNWHFAHLEYGCAAMLKSVSLPYWNQDDVYGGFGGAHCMIKGGYDTVLESLAKGLDVQLNHVVTEVLYGSEELGASGNSRKFVKISTSNGNEFVGDAVLITVPLGCLKAQTIKFSPSLPDWKLSSIDRLGFGLLNKIVLEFPEVFWDDNVDYFGATAEQTDLRGQCFMFWNLKKTVGVPVLIALLVGKAAIDGQSISSDDHVKNAIVVLRKLFKDASVPDPVASVVTNWGLDPFSRGAYSYVAVGASGRDYDILGRPVSDCLFFAGEATCKEHPDTVGGAILSGLREAVRIIDLVHSGKDYVAEVEALQTYQMQSDSERNEVKDMSNKLDACELSTALCKTSSDASYPLFSKETLLQEMFFSAKTTSGRLHLAKELLKLPPDVLKSFAGSKDGLSTLNSWILDSLGKNATQLLRHCVRLLLLVSTDLLAVRLSGIGRTVKEKVCVHTSRDIRAIARQLVSVWVEVFRKEKASNGGLKLLRRMPSTESSKPRSKDLLSGKPIVRAPNQVSFNPKVASKNARSAGNHSPHTAIKKPENKAAKLEAMTATRSDGSSLRSQKQQHALEPKVDNGLAMSEEEAAAFAAAEAARAAAIAAAQAYASVEAEINAPRELPKIPSFHTFAMRDHYLDESDTRKKVLSDNLVRLECISEIDSRNDKAKNPSVDHANCADVDSSKMTGDNCTQRSYSNENACLINIRDHSTDSGAVDSRFTRAWVDTDTIFIDGVKDPLAIERWQQQAMEADKEFYSRIRIPDEEDSSSQKQTCRSSASQVAESKPASDGQSRGVEHLRQGLINFISTVLMPLYRNKKVDREGYKGIMRKAVTKIIETCTEGEKMMTVHEFLDSKRKNKIQTFVDKLVERHCHMNRPPNS

>Zm00001d029714|Zmays_RefGen_V4/1-1814

MFYTSSKVPREMDDTLATIRMKLKKRKKGKDSNDFDAVAECGPEVPALVQQEGERAVVDIGDGVADEKSNLNVVKLEGSDVSGNCAQGLDDLGLKDSLAVLFTRPGRKSRQVSEEAEGMEVSCPHDEDGLNKGSGLAPDTASKGIKRRRRRTKEEMKNASVQDQKASLPRKAKTNGSKSTKLYKVGAGPGQALGGLSSVSPQLEKSGKENAVDDGSFYQSLGEVLLQDVEASKVLKDGSITSFNGVTDHFEISAWASNHPGLESRSGNLDEKTPRTAEHTTNLGVSDAHTRSQTLGKESSDDVDCSQGKSPTSTIKRKIGLKPKQVPGKSVRRKEEVLSVDADNKPTDTTEIIEASTTPVIEENLDQLPVMAPKDSCSSHDIVGNSPMCLGADMAAPVKYVDIVDVATPLDYEDKENTSKVKRVTRGSKKRKHGDMAYEGDVDWETLMQEQGLFSNPSAGFPDQSIKTKNKIKTSEVYEGGGDTGVAAVRAGLKAKTITPIEKIKFKEVLKRKGGLQEYLECRNMILSRWSKDVKHLLDLADCGVSDVPLKDELPHQALTRAVFLFLDQYGYINAGIASDKVAKGHNDTPYEVIEVPKLNESHQMESVNIQNSVVSVSQKNEDFEHGTSTECCRTVSVKNTECALVEASNEKNRSTVHCGVLELLPHLMSEEHPMEKNILDVSTEVRNASLPSSNLDIQFTSYMNSSVGKVEAPHQQEALEIENSGNNCQSDRAEFAVHGKRIIIVGAGPAGLTAARHLQRQGFLVTVLEARERIGGRVYTDRTSLSVPVDLGASIITGVEADIATERRADPSSLICYQLGLELTTLNSACPLYDVVTGDKVPDSLDEDLEAEYNGLLDEMALLFAHNGDSAIGLSLEDGLEYALRKHRATQPMDSVDQDGHLRFMTNSRAVDISVSASIGKEIDHCGKNDKIDVLSPLERRVMNWHFAHLEYGCAATLKSVSLPYWNQDDVYGGFGGAHCMIKGGYDTVLRSLAKGLDIRLNHVVTEVLYGSEELGDSCKDGKYVKVSTSTGSEFTGDAVLITIPLGCLKADTINFSPSLPDWKVSSINRLGFGVLNKIVLEFPEVFWDDNVDYFGATAEETDLRGQCFMFWNLRKTAGAPVLIALLVGKAAIDGQSISSGDHVNNAMVVLRKLFKNASVPDPVASVVTNWGLDPFSRGAYSYVAVGASGRDYDILGRPVDNCLFFAGEATCKEHPDTVGGAILSGLREAVRIVDLLNTGSDYIAEVETLQTYQMQTDSERNEVKDMSNRLEACELSTALSKNSSDAMYPIVSKESLLQEMFFSAKTTSGRLHLAKELLKLPTDVLKSFAGCKEGLSTLNFWILDSLGKNATQLLRHCVRLLVLVSTDLVAVRLSGIGKTVKEKVCVHTSRDIRAIARQLVSVWIEVFRREKDSNGGLKLLRRMPSIESSKTKSKDLQSGKPTLRVPNGTLDNNKVVSQRQRTRFASSQSPPKINKKYDNKEMKLETVTAAMSDGKLLSQKQQHGIESKVEECGIPMSEEEAAAFAAAEAARAAAIAAAQAYASVEAEISVPRELPKIPSFQSFVMRDHHLDESDARKRTLKDDFGRLECISENDSKNGEVKDLPENTKYADGDSLKMSHDNFTQQSHSNETACLANAQDHSTDTVVVDGRFTRAWVDTDTICIDGVKDPLAIERWQAQAMEADKEFYSRIRIPDEDDSSSQKHTCRSSASQGADSKPASERQSRGVEHIKQGLVNFIASLLMPLYRGKKIDREGYKTIMRKSVNKIIDTCSEGEKSMTTLEFLDDKRKIKIESFVGKMVDKHLHVVSKSAKP

>Sobic.001G316700|Sbicolor_v5.1/1-1799

MEDTLASIRKKLKKRKKGKESNDFGAVAECGPEVPTLVQQEDVQGVVDIGDADADEKSNLDVVKLEGRDVSGNCAQGLDNLGLKDSLSVLFARSGRKSRQVSEEAEGMEVSCLHDGDGLNKGSGLAPDTASKGTKRRRRRTKEEMKNACVQDRKASLPRKAKAKANGSESTRHYKVGACPGQALGGLSSVFNELEENAADDGLFHRSSAEELLRDVEATKVLNDGSINSFNGVTDHFEISAWASNHPGLESYSGNLDEKTSCTAANATNVGVSDAHTCSQTLGKESSDDVDCSQGKSPTSTIKRKTALKPKQVPGKSVRRKEAVLSVDADNKPTETTEIIEANTTMVIEENLDQLPVMGPKDSCSSHDIVGNNPVCLGADMAAPVKDVDIVDVAAPLDYEDKENACASKVKLKRVTRGSKKRKHGDMAYEGDVDWETLMQEQGLFSNPSAGFPDQSIKTKDQIKTSEVYEGGGDTGVAAVRAGLKAKTITPIEKIKFKEVLKRKGGLQEYLECRNMILSRWSKDVKHLLDLADCGVSDVPLKDELPHQALTRDVFLFLDQYGYINAGIASDKVAKGHDDTLYEVVEVPKLNESHQMESVSIQNSIVSVSQKNEDFECGTSIECCRTVSVKNTECALVEASNEKNCPTVHCGALKLLPHLKSDEHLTEKNNLDMSTEVRNASLPSSNLDIQRTLYLDSSVGKVEVPHQQEAREIENSGNNCQSDRVEFAVRGKRIIIVGAGPAGLTAARHLQRQGFSVTVLEARERIGGRVYTDRTSLSVPVDLGASIITGVEADIATERRADPSSLICSQLGLELTTLNSACPLYDVVTGDKVPDSVDEDLEAEYNGLLEELALLFAQNGDSAIGLSLEDGLEYALRKHRATQPMDSVEQDGHLRFMTNSGAVDISVSASTGKDIDHCGKNDKIDVLSPIERRLMNWHFAHLEYGCAATLKSLSLPYWNQDDVYGGFGGAHCMIKGGYDTVLRNLAKGLDIRLNHVVTEVLYGPEELGASCKDGRYVKVSTSTGSEFTGDAVLITVPLGCLKAETIKFSPSLPDWKVSSINRLGFGLLNKIVLEFPEVFWDDNVDYFGATAEETDLRGQCFMFWNLRKTVGAPVLIALLVGKAAIDGQSISSGDHVNNAMVVLRKLFRNASVPDPVASVVTNWGLDPFSRGAYSYVAVGASGRDYDILGRPVENCLFFAGEATCKEHPDTVGGAILSGLREAVRIIDLLNTGNDYIAEVEALQTYQMQSDSERNEVRDMSNRLEACELSTALSKNSSDAMYPIVSKESLLQEMFFSAKTTSGRLHLAKELLKLPTDVLKSFAGSKEGLHTLNSWILDSLGKNATQLLRHCVRLLVLVSTDLVAVRLSGIGKTVKEKVCVHTSRDIRAIARQLVSVWIEVFRREKDRNGGLKLLRRIPSIELSKTKSKDLQSVKPALRVPNETLDNNKVVSQRQRTRFASSQSPPKTNKKYENKEMKLETVTAALSDGKLLSQKQQHGIESKVECGIPMSEEEAAAFAAAEAARVAAIAAAQAYASVEAEISVPRELPEIPSFESFVIRDHHLDEPNTRKRALKDNFGRLECISENDSKNVKTKDLPDNTNCADADSLKISHDNCTQQNHSNETACLANTDTGVVYGRFTRAWVDTDTICIDGVKDPLAIERWQAQAMEADKEFYSRIRIPDEDDSCSQKQTCRSSASQGADSKPASERQSRGVEHIKQGLVNFIASLLMPLYRGKKIDREGYKSIMRKSVNKIIDTCSEGEKSMTTLEFLDAKRKIKIESFVNKMVDKHLHVVSKSAKP

>ELECO.r07.1AG0032170|Ecoracana_v1.1/1-1809

MDDTLAIIKKKLKKRKKGSEGVEGPVAECGTEGEILAHQEDVQGVVDAGEDVADEKSSHDGVKVGDGLQSLNDLGLEDSLSVLFTRSARKSRQVLEKEAEGVDLAGPHDEEGLDKESALALDRTSKGTKRRRRRTKEEMKNAGAHDRKVSLPRKAKENANARDSNRFPKVAACQALGELQFVSHDLEKKSDWKEKAADDGSCHLSFGEAFLQDAEADIVLRDGSGNSFDGTTHHFEDSAWASSQPRLKPCSGVVAVKSSCTAADEIIGGVSDGHTCSQILGKDSSDDIDCSQGKSPTSTIKGKTGLKPKPAPRKPVRRKETQSSVDTKLAEKAKDGVLSKHQIKSEVELPQSIMMKSNSLATNVAGCTGDPKDGEIEPNAAVHTEETLGQLAPATDVGMVDVAAPLDFEDMENSSKLKRVLRSSRKRKLGDMAYEGDVDWETLMQEQGLFSSPSAGFVDQSVKSKDKIRISEVNDSGGDNGAAAVRAGLKAKAVTPIEKLKFKEVLKRKGGLQEYLECRNVILRLWSKDVKHLLDLAECGVSDVPSKDESPRHALIRDVYLFLDQNGYINAGVASGKVSKGRTTCSEVVKIPTSRLSASHEMEPDSLQDGIVTASLPNKGPQYDNGIECPSNESAKNIVCALTEASDEKNRPAVDCEALELLPHLKPDEQPIEEKNKDASTEGREALLLSSNFNIHCRSGVDSPAGKVEDPCQPEAASIESCRDNQSKSDKVVSGVYRKTVIVVGAGPAGLTAARHLQRQGFSVTVLEARDRIGGRVYTDRTSLSVPVDLGASIITGIEADIATERRADPSSLICSQLGLELTVLTSACPLYDVVSGDKVPDDLDDDLEAEYNGLLDEMSLLFAQNGESAMGLSLEDGLEYALRKHRAAQPMASVEQYNQLRTKANSGSIDIAKNASTEKEIVGEDDKIDVLSPLERRVMNWHFAHLEYGCAATLKSVSLPYWNQDDVYGGFGGAHCMIKGGYDTVLHSLAKGLDIRLNHVVSEVLYGSEESGASCADGKHVKVSTSNGNEFIGDAVLITVPLGCLKAQTIKFFPSLPDWKLSSINRLGFGVLNKIVLEFPEVFWDDNVDYFGATAEETDLRGRCFMFWNLRKTVGAPVLIALIVGKAAIDGQSISSDDHVNHAMVVLRKLFTDALVPDPIASVVTNWGLDPFSRGAYSYVAVGASGRDYDIIGRPVANCLFFAGEATCKEHPDTVGGAILSGLREAVRIIDLVNTGNDYIAEVEALQTYQMQSDSERNEVRDMSNRLEACELSTALSKNSSDIMYPVVSKESLLQEMFFSAKTTSGRLHLAKELLKLPADVLKSFAGSKEGLSTLNSWILDSLGKNATQLLRHCVRVLVIVSTDLVAVRLSGIGRTVKEKVCMHTSRDIRAIARQLVSVWIEVFRKEKASNGGLKLLRRIPSIESSKSKNKDLQAGKPTLHMLKETMDNHKVASQRQHGTSANSHSPPKTNKKFENKATKLEAVTTIRSNDSSLSEKQHHDIKPQVEHAVPISEEEVAAFAAAEVARAAAIAAAQAFASAEAVISVPRELPKIPSFHTFARRDHHLEQSDARKKVSKDNFGRLECISEVDSRNGKAKDSSADNTNCGDVDSSKMTGDNCTHRSHSNEKACLTNVRDHSTDSCAVDSRFTRAWVDTDTICIDGVKDPLAIERWQAQAMEADKEFYSRICIPDEEDSSSQKQPCRSSASQVAESKPASEGHSRGVELKQGLVNFIASLLMPLYRGKKIDREGYKTIMRKAVNKITESCTAGEKLMASHEYLDVKRKNKIEGFVNKMVDRHLHVASKHTKP

>HORVU.MOREX.r3.6HG0631680|Hvulgare_Morex_V3/1-1851

MDDTLANIRKKLKRLKKGKDEDDAADAPAAAEGGEAVAVAAAAGTEDLQEGGDVEDMGEGVADEGGDLEAPANVVPGEDGVKVPGGDLGLGDSLSVLFRKPGRKPRQQASKNEEADGVEAADSQAEEALNGGSGGLGSDEAAKGTKKRRRRRTKAEMEEMRAAAAAAAENYANAMAGDGSPRRSKAKPKAAYSDRCSRAAARRAVGELAGLPVGELAAPGPPPVSERNPNDKEEAADKEEAVDKEEAADDGLSLGETLLQDVETSRIVEDGSRNSSDAASHCVKLPARADNCRGLNPCSGGEPAEKASHSAANASADGVSDARTCSETLAEDAKADAVFSQDKPPAPTLKRKPGRKPKEAPKKPVRRKDLPKVSADDKPVEIAESDASSKQRQRKNKFLLARSTTVQSNSSLDNKAGSAGDIKYGNIKPNAAVPTEESLDQPAAYGVRESRAAKDSRASHNMAASAKEINIIDVAAPSNFEDMENASKVKRLTRNSRKRKHGDMAYEGDVDWETLMHEQGLFSNPAASFADQSVKPKDKIKALEVLENGGVAAVSAGLMAEAVSPMEKLKFKDVLKRKGGLKDYLECRNMILSCWNKDVKHLLDLADCDLSNIPMEDDSPRQILIRDVYRFLDQNGYINAGIASAKVTKDHGTLCSEVVEAAKLNELPKMEPVRVEGDILAVSLQNKDHDQVALGPLTEESKENNVPDAHCDVQELIPHLQSKEQAFEEKNLDVSTEAGGALLPSINISSVGNTEGPSLDKPEAAVIEHPGNNCEVNHRVESGGHCKKIIIVGAGPAGLTAARHLRRQGFAVTVLEARDRIGGRVYTDRTSLSVPVDLGASIITGVEADIATERRADPSSLICSQLGLELTVLKSACPLYDVVSGNKVPDDLDDELESEYNGLLDEMEQLFEQNGESALGLSLEDGLEYTLRKKRAAHVVSSVGHDDQLISMSNRGGVDISKNASTEKEIAHCGEDDKTDVLSPLERRVMNWHFAHLEYGCAAMLKSVSLPYWNQDDVYGGFGGPHCMIKGGYGAVLESLAEGLDVQLNHVVTEVMYRPDESDASRISGKTVKVCTSNGAEFVGDAVLITVPLGCLKAHAIKFSPSLPDWKTSSIDRLGFGVLNKIVMEFPEVFWDENVDYFGATAEQTDLRGQCFMFWNLKKTVGAPVIIALLVGKAAIDGQSISSDAHVSNAMVVLRKLFTDAAVRDPVASVVTNWGLDPFSRGAYSYVAVGASGQDYDIIGRPVANCLFFAGEATCKEHPDTVGGAILSGLREAVRIIDLVHSGKDYVAEVEALQTYQVQSDSERNEVRDMSNRLEARELSTALCKNSSDASYAVASKESVLQEMFFSAQTTPGRLHLAKELLKLPSDALKSFAGSKEGLTKLNSWILDSLGKNATQLLRHCVRLLLLVSTDLVAVRLSGIGRTVKEKVCVHTSRDIRAIARQLVSMWIEVFRKEKASNGALKLLRRLPSVESSKTRSKDLHSGMRVPHVPNEAGAQRVRSAGSHSPHRTSKKPDKIVKLSSIMATKSDGSSLRSPKQHHDPEPKANQGMVMSEEEAAAFAAAEAARAAAIAAAQAYASAEAVISKPRELPKIPSFGDFARRDHHLDESDTRKKMASDNHGRLECISEIDSRNGKTKNSSAEHANCPDVDSSKMTVDNCTQRSYSNEKACLINIRDNSAESGAVDSRFTRAWVDIDAVSIDGVKHPLAIERWQAQAMEADKEFYSRIRIPLEEDSGSQKQTCKSSASQVAESKPASEGQSRGVEHIKQGLISFISTLLMPLYKSKKIDREAYKTMMRKAVTKIIDACTEGEKLMTANEFLDFRRKTKIQTFVDKLVERHWHLAPAPKS

>Asa4G00226.1|Allium_sativum/1-1764

MNNNEEESADLRHGEAVIQLLQNPKIAIGFHLEELVTKAPSNPDMDESKVLGNPIKGSKRKINGGKGELDEDAPLIMLKKKKKRVSKIVKISDLEAREEARNPRSEVEEKFMMSDTLASLKKRVKKPPKDVEDLTVDGSVVDLESKRSKRKKNNSFCPVNVVEESISEFMQKTESNQLASGTEKNMDDLVSSDPGVHDSKVTNGRKLSANEDLGLVVTEDQNLSANEDLGLVVTEDEIRIESQDKVNGEDEVICISSLNDREVEVMEETQDLVDGLKPCSLEKPISFVYETHDVETTCDSIEMKHDPEICVGDLSIHNVGQDQCSTDDVLNQTCKTEGIINDSKSLGNELSFGLCELTPNTMEEDSLEKNVAKEEVKELYVHFENVAVSEINNAEETGEYQEAYKDVKEVGENPSMDKQLLDISNEGIAVGDIDSVGYQKPDAIPKFENQSKAARLLRNIRKPRYGDMAYEGDDDWDLLSNFRSVEKFDSSNFLEDEISGSAAAVAAGLKASAAGLIEQIKFKELLKRRGGLQQYLDCRNFILSLWSKDVKRILHLEECGVSSTPVKDESPRASLIREVFAFLDGNGYINAGIATDKSNSVCLLEPVFSEESKPLETCAENDKFKGEASVLINAYSSREVVETSPLLVPEDNNHDNPSASNVSSESSTQLIPGKHAIDNVQVGTWHVPTSEAQILNSVTDGNNTCMNEKYQVIGSSDRMVIQAENPCSNITISSDHVIDPTLKINRCEDVRKRVIVVGAGPAGLTAARHLERQGFSTVVLEARDRVGGRVHTDRSSLSVPVDLGASIITGVEADVATERRPDPSSLICKQMGIELTVLNSDCPLYDIITGEKVPHDLDGALEAEYNSLLDDVVPFIAQKGENANKLSLEDGLEHALRKRRLSQRMTAASDTVVANDIDTNLNGNMLRPLERRVFNWHFANLEYGCAAPLKQVSLPNWNQDDLYGGFGGAHCMIKGGYSTIVESIGAGLNIHLNHVVTEIEYGIDSENTSSPHKKKVKVRTSNGCDFVGDAVLITVPLGCLKANTIRFSPPLPIWKDSSIRKLGFGVLNKVVLEFPRVFWDDAIDYFGATAEETGNRGQCFMFWNVKKTVGAPVLIALVVGQAALDGQAMSKSEHVNHALKVLRKLFGEASVPNPVASVVTNWGLDPFSRGAYSYVAIGASGDDYDTLGKPVENCLFFAGEATCKEHPDTVGGAMMSGLREAVRIFDILTTGYDYAAEVEAMEAIRRESDNEIIELKDMIKRLEAYKYTNVTLVRDLFYKAKTTSARLHLIKEMLNLPVKSLKSFAGTKQGLSTLNSWILDSLGKNATQLLRHCVRLLVLVSTDLLAVRLSGIGKTVKEKVCVHTSRDIRSIASQLVNVWIDVFRKEKAANGRLKLLQESSKLRPKDLSSGKMGPNNEAAKSRHISKKTDSKNTRLENAMDTNSEANSSRSQSSFQCQDLKMDRDVIISEEEAAAIAAAEAAHAAALAAAEAFASSEANISSLHDLPKIPSFHKFVRRDQFTEPDDSEFRRRVSGSIPGRQDCISDIDSRNCKTQNWTGDFTETGANPDNSSQRSNSNDLAPLNIREHSEESTGLDRRHVKAWVDTETVGSGGVKDYLAIERWQSQAMEADAEFYQRLQLRDGEDSNGTFSTPPYKSEREGSSASQVVENKLSTEDPPRGADHIKQGVMDFVASLLRPLYKTRKIDRDGYKSIMKKTVTKVMEQCTEAEKRFTAPEFLDYKRKIKIRTFVDKLIERHMATARGLK

>Zosma05g27910|Zmarina_v3.1/1-1880

MGHGMDKEPLPKRKLGCRTEELEEEDQSIGSLFKLRKSRLGKNTKVGSSAKPLEVLEEKNGISRGEMEMGDTLATLRRKLKPSKKITKVSPNSAHGIDRSDVVRVLGAEVPLLDRIEEGHVKDGANENEVTPVVVDRDDMEGLLSDFVRNAQQSSSHGGSIVSSKRRKKATSDNQKVVSVTPMSFPLEDVISTKRKRIRKSKNDDLKNAVTTANILDKKQPRKQRLKREKNQLSRKHKEQTNTEAKHVLRKECARNNSNNIEGFCRTTKGCSGKDGQLDPCSVNSSQGLNSMPTRMDNLSKSEDGLTQQPETNVVCGNGDLTEVTEPVRIPCVDSANMQVEEVVNSDKGSIKSIHDFGQIRSASTVNCEDEDFSNHHTKGFSQSNEDQGNSSKGPLTVPNNEILEDKKVSRFQSVAFSSDLKGIPTIVESDTEIKKDERENLVTRTTRKVKRHRHRDMAYEGDADWEILMHNQEIFDYNKDKSDSKEALAAIASGLKAHVASPIEKIKFKDVLKRKGGLQEYLECRNFILGLWNKDVRHRLTIKDCVSTSTASWNGPGRSSLVEDIYKFLDGSGYINAGISLDRKTYLNNEYDRLLSYKSTHNEISANHTLDVKDLTSCVPLNDIVPISIPFTTNATELQNVKDIVPVVSVTNVIKLHDVKDIVTVTVASGINEMELQDVKTAGSEAGTDAVVNVNSGIQTMSPASRIEIHDNENAQKEMCHDRVVASTSAPSNNVWTNDVIIDCKGSFTKEERNTLDTKNDFSCEITNDIQSPVLIHEPLDFSSSKVKHAGDGMENIACANRLKVDSIDRNINYDVNDAHKKVIIIGAGPAGLTAARHLQHQGFSVILLEARDRIGGRVYTDHSSLSVPVDLGASIITGVEADIATQRRADPSSLICKQLGIELTVLNSDCPLYDIVTGQKVPSELDEALEAEYNNLLDNMDILISQNGDVIGMSLEEGLEFALKKCHMTKSTSLVPISSKMVDKTKSLEFPGNTSNIIQNDLHIEMEDKNGVVEDILSPIERRVMDWHFAHLEYGCAAPLKDVSLLHWNQDDVYGGFGGAHCMVKGGFSNITETLGKGLTVHLNHVVTEIMYNSKETSTGSKKNEVKVSTSGGSQFVGDMVLVTVPLGCLKANSIKFSPALPDWKQTSIQRLGFGILNKVVLEFSEVFWDDNVDYFGATAEETCHRGQYFMFWNVKKTVGAPVLIALVAGRAAVEGQDTSTSDHVSQALMILRKLFSEKYVPDPVASVVTNWGMDPFSKGAYSYVAVGASGEDYDILGRPVANCVFFAGEATCKEYPDTVGGAMLSGLREAVRIIDIFRTGNDYISEAEAMAAAQRQAESKRNEVRDFIKRLDTFKLSNGLVRNSFGGISPLFTKDILLQDMFCNAKTTAGRLHLAKELLHLPVESLKSFYGTKEGLAILNSWIRDSLGKDATHLLRHCVRLLVIVSTDLLAVRLSGIGKTVKDKVCVHTSRDIRAIASQLVNVWIEVFRKEKSGNSVLKLLRQTNASDSSKHKSQDHIAGKPPLSIDNEASDKKGSTSLSNIKKKADKSFLTSPGSKSNKYSLDNEANVMSAEENAAFAAAEAAHAAALAAAEAYASSEAGRALCELPKIPSFHKFARREHVHADDLDPRRKWSGEILGRQDCLSEIDSKNHRVRDWSIEFPAVRDNPDCSKNPTDSSYISWGDPSEVAYPTNLKERSAESGAVDGRLKKAWVDVDAADVGGIKDHTAIERWQSEAMDAGANFFHSMNLYDEEYSTGAVNSPKEKHQRPQGGSCASQAAESVSLSEGQPRGASRIKQGVVDYVASLLMPLYKTRKIDKEGYKSIMKKTANKVMEQCTVAEKVMTVLEFLDSKRKNKIRAFVDKLIERHLATHSNIQP

>Nycol.B01602|Ncolorata_v1.2b/1-2121

MDGVRMRGFGSTKRKVEVVGLNDDSEDDNEPIGSIFNLKKKKRRKKKRKELIDSKKVEATFEARAGAAVNEDANDSDDDEGLTLASFRKKLKDPKGSKAGGKDLVERPGLAKGSLHGGRTGVIQSCSSSKSMEEDDDGGVQDDDDDVGLDQVLIGSLKVSHPGVGEESQTTLPQTSEEGSLRGEEGLSQSAEESIEGSARMSAKRTRTSSRHRQNLEAPKSTNRPDGSPEDLLSEFVKAQRSRPPAMRKEEKRTVLPGQNWDQATERIRDGSSDSLVTKRHPVQKTHGESSTLGGVSPQLCDGLSGLTKEGSYKPNSSNIMDDLSSAQAVESIDAPTVEYTDEIHEDGNGQLNGNSKSEGDSSHKQVRSSPTLGAIHESINSDKPFDTKNAIEVSAASSGKLNRSYASREANMKPDGYSHNEASEDLLSSVFRRSRSGTKQRRVVKSSSAGAFTNQVSDCSCPKLSSVSNYLDGGNSLELSQPPHELMENLSSTQETDNMPSLPFESTVKGVVSNVVSKATQFVNELVTISQADNDKNSASVTEERHCSAVTTQQIDEVLDDKDDIVVMSKDSDHIADSLLPSKPVNTPCVASVECRRKLPCPSSSPVSNGIMKCSTLTQTISLTEIQNPPEETIRSDSRRHGLSGDVVDEKYCSESGNQAIQKVSGTVCSEEICSDRAGKGLPKMAKKVSTSQRVTRKVRKPRYGDMTYEGDSDWDSSAYQRGNSDSTLVMEDDVFVKTKTKADLLSTLLLEAHNGGAAAVAAGLKAKSLVPIEKIKFKEVLKRRGGLHEYLECRNMILSLWSKDVCRILPLSDCGVTSDPLYDELPRASLIRDIYAFLDSYGYINTGIASVKEIAKPDCMPQLKLSKRSDLTENSGGRAIDTEEEVAFILGQAKHFENVANVKYDTQMKDGKCITVGDVEAADSHKLQVADIELESSTSKEMEEDKAGQVAMDKCILGSSAPCTPEVPTHEQIVMAKDCAVLGFTSGEICKLEMDEKENGFSGYVSPRRTKVSPIDSVSIVDQNGEADTLNPEQAKTSDGTICDMDGHQRVIVIGAGPAGLTAARHLQRQGFMVNVLEARHRIGGRVHTDRSSLSVPVDLGASIITGVEADVATGRRPDPSSLLCTQLDLELTVLNSECPLYDIVTGVKVPSALDESLEAEYNSLLDDMEVLVSQNGEAALKLSLEDGLEHSLRSHRVARPTGDEIGLHHLKISDDLENSKISRNIQADGSKMAAAFETTGMETYKQDEDPLDPLERRVMNWHFAHLEYGCAASLDKVSLPYWNQDDVYGGFGGAHCMIRGGYSTIMESLAKGLNVHLNHVVSEVKYRVVDSKGSEARRNEVKVLTENGNEFVGDAVLVTVPLGCLKANAIKFSPSLPEWKQFSIQRLGFGVLNKVILEFPAVFWDDSVDYFGATAEKTDSRGQCFMFWNVRKTVGAPVLIALVVGKAAIDGQNETAAFHVKHAILVLRKLFGEAAVPDPVASVVTNWGQDPFSQGTYSYVAIGASGEDYDILARPVENCLFFAGEATCKEHPDTVGGAMLSGLREAVRIIDILRNSHDYAAEVEKMEAAQRQLDTERNEVRDMMKRLASSELSAPQIKILDGEQKLLTKESLLKDMFLTAKSTAGRLYLAKQLLQLPVKALKAFAGTKEGLSTLNSWILDSMGKDATQLLRHCVRLLLLVSTDLLAVRLSGIGKTVKEKVVLHSSRDIRAVAGQLVNMWIEVFRREKSAHGWPKFSKQTSASDQPKNKDLITKKSMVKAKTDAPEMQGNLVVPSSTGSYSPAHGSKKVESKLVKLESRAENSSTNMDSRDLQDHDAVMSEEEAAAFAAAEAARAAALAAAEAYASSEASMPRELPKIPSFHKFARREQYAQRDELDPNRKWWAGSDVGKQDFTAEIDSRNCKVRNWSVDFSATCGNLEGSRMSTENAQLGFSNDVHLNFREHSGESGGVDSRLARAWVHSNRSTGGMDRHAIDRWQSQSLGSDMDLYLSHEPVIDEGYLSKELMNTRPRSEVENSSASQAEESKGLENQQKGAEKIKQGLVDYIASLLMPLYKARKIDKEGYKSILKKSVTKVMEQSTEAEKLLTVSEFLEFRRKNKIRAFVDKLIERHMALVPRPKT

>g17805.t1_1|Euryale_ferox/1-2077

MDGGGMRGFDNTKRKVEVVGLNDDSDDENEPIGSIFNLKKKKRRKKRKELTDSKKVGATYEARARAEVDGLKGEVAVNEGANDSDDDEGLTLANFRKMLKDPKGSKTGGRDLVGRSGLAKGSLHRGRTGVIQSCRSSKATDENHDGSVQDDDDDEGLDQVLTGSLRGEEGLSQSAEESNEEWARMPAKRTRTSSRHRQSSEARKSTNRTDGSEEDLLSEFFKAQRSRPPAMRKEDKRTVIPGQSWDRNTEKIPDGSSDSPVTKKRPVQKRHGECSNIVGVLSGLSGLAKEGYYKQNSINIMDDLASAQAMESIDALTVEQTDEIHDGKGQLEGNSNDEGDSSHKQVQSSPRLSVHGSIDSDKPFDTKDEVSAASSGKLHRSCISRETEVNMKPDEYSHIEASEDLLLSVFRRSKPMTKQKKVLKISRTGDIANQVSETFSPKLSSVSNCLDGANGPKLSQPSHELMENSTQEIDNMPSPPLESTMKGVLPNVDELVKISQTDNDTNSAAVPEKTKRHGSAVTARQMDVVVDNKGDAVVMPKDFDHTVNSLKCILPSKSANIPCVASRKLPCSSSSQASNGITKCSTLTQDMSLTEIQNPPEETVRSDNRRHGLSVDVVDEKYCSESDNQETQKISKTVCLEEICSDDAEKGLPKMAKKASTTQRVTRKVRKPRYGDMTYEGDGDWDASVYERGNSDNTPVMDDNIFVRTKTKSDLLSTLLLEAHNGGVAAVAAGLKAKSLVPIEKIKFKEVLKRRGGLHEYLECRNMILSLWSKDVCRILPLSDCGVTSDPHDDEPPRASLIREIYTFLDSYGYINTGIASVKEIAKPNCQPQLKLSKRSDLTENSGGRSADTEEEVAFILGQAKHFENGMVKNEAQIKDEKCVIVDVVEAADSHKLRVADVELELPTLKEIDAPRTLGMPTVEHVIAKDCEDLEFTTGVTCKLAVDEKENGFSGCTLVELTKVSPLHSLSIVDQNGEANALIPVQTKTSDGTTCDMEGRQRVIIIGAGPAGLTAARHLQRQGFIVNVLEARNRIGGRVHTDRSSLSVPVDLGASIITGVEADVATGRRPDPSSLLCTQLDLELTVLNSECPLYDIVTGEKVQSELDESLEAEYNSLLDDMEILVSQNGEAAMKLSLEDGLEHALRSRRVAHPTVDGTGLHDLQVSGDLEKYKISRNIQADGSKMAAAFGTTGMERYKQDEDPLDPLERRVMNWHFAHLEYGCAASLDKVSLPYWNQDDVYGGFGGAHCMIRGGYSTIVESLAKGLNVHLNHVVSEVKYRVAYSKGSGTRRNGVKVLAENGNEFVGDAVLVTVPLGCLKANVIKFSPSLPEWKQSSIQRLGFGVLNKVILEFPTVFWDDSVDYFGATAEKTDNRGQCFMFWNVRKTVGAPVLIALVVGKAAIDGQNEIAAHHVNHAVLMLRKLFGEASVPDPVASVVTNWGQDPFSRGTYSYVAIGASGEDYDILARPVENCLFFAGEATCKEHPDTVGGAMLSGLREAVRIIDILRNSHDYAAEVEKMEAAQRQLDTERNEVRDMMKRLAFSELSAPQIKTLDGEQKILTKEFLLKDMFLNAKSTAGRLYLAKQLLQLPVKVLKSFAGTKEGLSTLNSWILDSMGKDATQLLRHCVRLLLLVSTDLLAVRLSGIGKTVKEKVVLHSSRDIRAVAGQLVSMWIEVFRREKAAHGGPRFSKQNSASDQPKNKDLITKKSTMKVRTDASEMQGNLVVPSSTGSYSPAHGSNNKKVESKLVKLEPRAGKSETSSTNIDSRDFQDYDAVMSEEEAAAFTAAEAARAAALAAAEAYASSEACMPRELPKIPSFHKFARRELYAQRDELDPNRKWWAASDVGKQDFTAEIDSRNCKVRNWSVDFSATCGNLEVSRMSAENAQLGFSNDIQLNFREHSGESGGVDSRMARAWVHSNRSTGGMDRHAIDRWQSQSLGSDMDMYFAHEPVLDEGCLSKELNASRKPRSEVENSSASQAEESKGLENQRRGAEKIKQGLVDYIASLLMPLYKARKIDKEGYKSILKKSVTKVMEQSTEAEKLLTVSEFLEFRRKNKIRAFVDKLIERHMTLDPRPKS

>g19266.t1_1|Euryale_ferox/1-2085

MDGGGMRGFGSTKRKVEVVGLNEDSEDDNEPIGSIFNLKKKKKKRKKKRKELTDSKKVGATFEARAGAEIDGLKGEAAVNEDADDGNDDEGLTLASIRKKLKDPKGIKTGGKDLVGRSGLAKGSLHGGRTGVIQSYRSSEAMEEDHDGGVQDDDDDDGGLDQVLIGSLKVSHRSLGQESQTTLPQTYEGSLRGEEGLSQSAEESNEEWARMPAKRTQTSSRHRQSLEARKLTNRPDGSEEDLLSEFVKAQRSNLPAMRKDDKGTAMPGKSWDRDTENIPDGSSDSLVIKRRPVQKRHGECSSICGVSSGLSGLTKEGSYKQNSSNIMDDLVSAQAVEFINAPTVEQTDEIHDGKAQLDGNSMYEGDSSHKQVQSSPRLSVHGSIDSDKPFDTKDEVSAASSGKSYRSYISRETEVNMKPDEYSHIEASEDLLLSVFRRSKPITKQRKVVKTSRTDDIANQVSESSSPKLSSVSNCLDGANSPELSQPSHELMENLSSTQETDNMPSLPLESIMKGVLPNVDELVKISEAVNDTNSAVVPEKRHCGAVTTRKMDVVLDDKGDTVVMSKDSDHTFDSLQHILPSKFVSTPSAASVECRRKLPCSSSSKASNSITKCSTLTQDISLTEIQNPPEEIVRSDNRRHGLSVDVVDEKYCSESDNQETQKVSGTVYLEEICSDDAGRGLPKVAKKASTSQRVTRKVRKPRYGDTTYEGDSDWDASVYGRGNSDNTPVMDDNVIVRTKTKSDLFLILLLEAHNGGVAAVAAGLKAKSLVPIEKIKFKEVLKRRGGIHEYLECRNMILSLWSKDVCRILPLSDCGVTSDPLVDESPRASLIREIYAFLDSYGYINTGIASVKEIAKPDCKPQLKLSKRSDLTENSRGRAADTEEEVAFILGQAKHFENGMVKNEVQIKNEKCIIVDVLEAADNHKLRVTDVELEPSNLKGMDAPCTLGMPTDEQVVIAKGCSDLEFTTGGTCKHAVDKKENGFTGCASVELTFAKTYDGTICDMEGRQRVIVIGAGPAGLTAARHLQRQGFIVNVIEARNRIGGRVHTDRLSLSVPVDLGASIITGVEADVATGRRPDPSSLLCTQLDLELTVLNSECPLYDIVTGEKVQSELDESLEAEYNSLLDDMDVLVSQNGEAAMKLSLEDGLEHALRSRRVAHPTVDGTGLHDLKVSNDLEKSKTSRNIKADGCKMASAFGTTGLERYKQDEDPLDPLERRVMNWHFAHLEYGCAASLDKVSLPYWNQDDVYGGFGGAHCMIRGGYNTIVESLAKGLNVHLNHVVSEVKYGVIDSKGSGTRRNEVKVLAENGNEFVGDAVLVTVPLGCLKANAIKFSPSLPEWKQSSIQRLGFGVLNKVILEFPAVFWDDSVDYFGATAVKTDNRGQCFMFWNVRKTVGAPVLIALVVGKAAINGQNECAAYHVKHAVLVLRKLFGEASVPDPVASVVTNWGQDPFSRGTYSYVAIGASGEDYDILARPVENCLFFAGEATCKEHPDTVGGAMLSGLREAVRIIDILRNSHDYAAEVEKMEAAQRQLDTERNEVREMMKRLASSELSVPQIKILDGEQKLLTKEFLLKDMFLNAKSTAGRLYLAKQLLQLPVKVLKSFAGTKEGLSTLNSWILDSMGKDATQLLRHCVRLLLLVSTDLLAVRLSGIGKTVKEKVVLHSSRDIRAVAGQLVSMWIEVFRREKTAHGGPRFSKQNSASDQPKNNDLMTKKSTVKVRTDAPELLVNPVVPSSTGSYSPAHGSNKKVESKLVKLEPRAGKSETSSTNIDSRDFQDYDAMMSEEEAAAFAAAEVARTAALAAAEAYASSEANMPRDLPKIPSFHKFARRELYAQRDELDPNRKWWAGSGDVGKQDFTAEIDSRNCKVRNWSVDFSATCGNLEESRMSAENAQLGFSNDIHLNVREHSGESGGVDSRMARAWVHSNRSTGGMDRYAIERWQSQSLGSDMDMYFSHEPVIDEGCLSKELNASRKPQSEVENSSASQAEESKELENQRRGAEKIKQGLVDYIASLLMPLYKARKIDKEGYKSILKKSVTKVMEQSTEAEKLLTVSEFLEFRRKNKIRAFVDKLIERHMALDPRPKS

>evm_27.TU.AmTr_v1.0_scaffold00182.5|Atrichopoda_v1.0/1-2095

MDSGEGGMDGLDNKKVGLKRNLKSIEFQGDSDDELPIGTLFKIKKLRNPKTKKSDNEEGSEIRAEDSKVSEEVSVNVNIVADMDDTLANFKKKLKVPKIVKESSESNCLVSKQSRVKLSKKFTRALKGQPSVVDESRSLLSTKSTRPRSSSIAKPVEETLKFDDGSDQSDVVKEDSLLSALVPKSRAALIRKKIGETLISDDVQQKVSDKSPKDFLISALVRKTRSASLRKQREENLRTEDPLDRPSVELLKDSLPAPVRRSRSVTYQKKGTETRRFNDGLNQSIDVNMEISIPVSLMKSHCAAIDEQMRETPSSDSGFEEVSVENLKDSQSALLQESGSGSMLKKKEETLMSVDELDLFQITGTTTGAGILKLTEDTQKSGDSFGQTRGVQEDGCSPLAQNHHFASLSLATDETRRSDGTSNDSSSGVIQEFSLVSLSKRLAQVPLNSGISDGGLRQQSYSELQNLQFTQVLESCNFSQAKMPKKIDRLDDGSSQAFDRILDDELKAFPSVEINRDSPRAQVQTSFSASLPEDRSGGLNHISNANQMQDLQMENEGLNKSVALSSVPVKDSSSPRLDGGLSRCYGFVSNDGFSPYSSTLDQALFPKPEVCVDQAPDGALGHKQFFDSNDGLNPLSDENQTFQKPNSAFVYEEETPISEDIPNQTSEDFSKDLPSTQVHESCSFATEKNKEEIPMSSDGSKGKTSTLEKKSQRLTAAQRVLRKVKRRRYGDMTYEGDSDWDDVLMHEERSFSLDDEDRLTRSKTRPDSFSSLFLDADSGAAAAVAAGLKARAPGPAEKIRFKEVLKRRGGLQEYLECRNMILGLWSKDVCRILPLSDCGITNVPLEDESPRAALIREIYSFLDHHGYINVGIAAEKENSRNHGTPQLKLARGNKTRSSYEGKVAADSEEEVAYILGQVKTSENVGLVQNDGPHEDGLPTIPTSSLDANYVEPNKGHLYPTVAEPLSLKNSGELGIDPHAGFVLNHNQALYKEDGFDEIDNQRALYVQSLESETIEKGVRLDPFVLNGVIETSMESGEKVIVIGAGPAGLTAARHLQRHGFRVCILEARNRIGGRVHTDRSSLSVPVDLGASIITGVEADVATERRPDPSSLVCTQLGLELTVLNSECPLYDIVSGVKVPGDLDEALEAEYNSLLDDMVVLVAQNGEAAMKMSLEDGLEYALRKRREAHIASVTPELDLLKVSDDFSSLNAAIAFDSEISTVAESRTPDRNTNRTEDDVLSPLERRVMDWHFANLEYGCAAQLDIVSLPYWNQDDVYGGFGGAHCMIKGGYSTVVESQGKGLDIRLNEVVQEVKYVVGQSKGECPKRSEVRVSTASGHEFVGDAVLVTVPLGCLKANTIKFSPSLPDWKLSSIQRLGFGVLNKVVLEFPFVFWDDNVDYFGATAEETNHRGRCFMFWNLKKTIGAPVLIALVGSSKAFWGASVPDPVASVVTNWGDDPFSRGAYSYVAVGASGEDYDILGRPVENCVFFAGEATCKEHPDTVGGAMMSGLREAVRIIDIMRNSNDYTAEVEAMEAAQRQSDSERNEVRDMMKRLDAGELSNVLCKGSLDGDQKLLTKEALLQDMFLNAKTTAGRLYLVKELLQLPIGVLKAFTGTKEGLSILNSWILDSMGKDGTQLLRHCVRLLVLVSTDLLSVRLSGIGKTVKEKVCVHTSRDIRAVASQLVNMWIEVFRKEKAANGGSRLFKQTSASLNTGDPLKMKPKDTSHAKPPTRAPNDPSESHTPFHMHAKKSDNKPLKSETGNDSKSEANSSRSQSLLQDSRVDDNVMTEEEAAALAAAETARAAALAAAEAYASCEANVLPELPKIPSFHKFARREHVASREDSDFKKKKWPGGVLGKQDCLSEIDSRNCRVRNWSVDFAATCVNLDDSRILGESHNTQRGYSNELLSQMNLREHSGESGAVESRFKKSWVHSTTGSVSGGKDYRATERWQPHPFGADDVVLHSGLHVTDEEDSTKALNHPIVKVEREGQSSCASEAAENKVMLDNQAGGMDQLKQGLVDYVGSLLMPLYKDKKIDKEGYKSILRKSATKVVENSTAAEKAMSISEFLDFKRKNKIRSFVDKLIERHMTSNQNKKS

>CYCAS_020788|Cycas_panzhihuaensis/1-1915

MNDGRWGKFELILDFTGPKQLQERLFQGQIWEFEGREICKRQSDSSVALKGFQEMQAKPMNGDRINNNDSCVVETKIMIEEDLSSHSAESMHGSEINGMGSVIKEDGVGLDGSAVSMVETNDRKEGRNAIGEIQGEQDGEESDWPLSKSKNLAAEMQVALDGEEREESSQSLPKLKNVVDEMQDDLDGEELVRSLAKIKKRRRSKNTEGKDVLEGDIGNSADASKGKKKGLSKVSGADDSEDTLADFKKKIKGPKPGKDMTKVTSVSRQASASEKDIKEAAKPDSVLKQQRTSIPSQSSPSVLVQKARAVKGAKASKASLESSLEGPQKGSAGKAPEISKGKKSSDSQRALRQVKKRRHCDMAYEGDSDWDVLMNEEEMLENKSSVDKERLGRGRARSGLMSSLLAQVVPGDAAAVAAGLKVRAPGPSEKIRFKEVLKRRGGLQEYLECRNLILWLWAKDVRRILPLTDCGVSLVPSADESPRASLMRDIYEFLDYHGYINVGVAVKKPKTKAGIEPKLKLSKESTQIEKAGDKVADAEEEVAFILGQIKGSEENASQVQNHIQVDEENQLLLLTAEDVKSSKLAGGSTGLAALDKKESEECGIALMEIDESPLDAACMAENAALGNASSPVVKTEAETGSTMLDKDNEELGTRNSSGSEGATNQDFLGKVPEVKIEAYPEAKVASALEQIKNSYGEDEFSDIDSAKVLHNLSKHDSCTDKQAVVNTASDNKTDANSDLKGRKKVIVVGAGPAGLAAARHLQRLNFHVIVLEARDRVGGRVYTDRTSLSVPVDLGASIITGVEADVATERRPDPSALLCTQLGLELTVLNSDCPLYDSVTGQKVPANLDQALETEYNSLLDDMVMLVAQNGDAAMRMSLEEGLEYALKKRRVARLSAVGSDLASDRTLLNDLDSTRPDLSLALQDFSVEGIEQLNTAVDANLTVISENNISVVAGMENGIDKESAQYKANLTGIETGEQQILTPLERRVMDWHFANLEYGCATELSRVSLPYWNQDDIYGGFGGPHCMIKGGYSTVMEALGEGLNIQLGHTVTEIAYTTKDSKSKGGIKREVKVCTENGCEFVGDGVLITVPLGCLKGNAIKFSPALPDWKLSSIQRLGFGVLNKVVMEFPEVFWDESVDYFGATAEKTELRGRCFMFWNVKRTVGAPVLIALVVGKAAVDGQNTESSDHVRHAVMVLRKLFGETAVPDPIASVATNWASEPFSRGSYSYVAVGASGEDYDILARPVENCLFFAGEATCKEHPDTVGGALMSGLREAVRIIDILENREDSTAEAEAMAAAQRHSDSERNEVRDMVKRLAAGELSNVLCKGSLDGEQKPLSRDALLQDMFGNAKTTAGRLLLAKEMLQLPVAVVKAFAGTKEGLGILNTWILDSMGKDGTQLLRHCVRLLLTVSTDMLSVRQSGVGRTVKEKVCVHASRDIRTVASQLVKMWIEVFRKEKATNGGFRLLKPATGGANVPSSADAVKAKSKEQGSAKPSPQAANDAKGNSLVPSSTGDHSPPSPNNKNLEVPSTQVEAGVEVKREVVCTGAQGFDQGHSSMPDDNAISELEAAALAAAEAARAAAVAAAEAYATSEAECSTLPELPKIPSFHKFARREHYAQKDDSEVRKRKWSGGVLGKQDCTSEIDSRSCRVRNWSVDFAATCGNLDSSRITGTDYGDGNLLPTVDSNKKSLIGENRVQQQRSQSNDVDSHSSLKERSAETGAVDSRFTKVRVQNAISHAGGRIHNAADNQHPYSVGLESELFEQSVQKLNDKHSSKIAQLPIEKPKTSGDNNCATEAGPNRGSPGSRNGHKDEARGSEHIKKGVSDYVATLLMPLYKTRKIDKDGYKSIMKKSTIKVMEHNTPAENAMVVSEFLDCKRRNKIRSLVDKFIERYMGTRLISEPALGEYQGAAAVS

>PITA_24616|Pinus_taeda/1-2591

MMMWMSMRVRMCIPFHSFILRTLTLFLQTLRFIDNGMGYSKAFGSANRKQANLMNDINSSGSSELGTKDTVGEVFLSHFPEPVDAPECNGMESVTKEDGAGLDGSAEIVEKIVEEDGENLMDDIQDTEEGEQSLPRSGSVDDIQVNQDVESSIPSPLSAKSTGDGIQDDPDGEETLAKFKRPHRSKKSVVKDDLEDNLGNEVEIPRDKGKSSSKGKAVRESDGENISDNSDADPDGKEGGHSSPRSRSVLDDMQVIQDAEESMWSLHKAKGIGDGMQDDPDGQETLAKFKRLRRSKKTAAKVVLEDDVGIDMEVPMYKDKSLLKDIQESDGQNLIDNIQADFDGEEGDHSSPKFRSVVDGRQAIQDAGESIPSLPKAKSIRDGMADSDGQENLAKSKRLPRSKKVAVKVALQDDVRNEMEVSIDKDKSLPKDKAVDESDGENLIDDIQADPDGQETPAKFKRLRRSKMAVDKVASQDDGGNEMEVPIDKDTILSKDKTVRESDGENLMDFIQADLDGEEGDYSSLKPRSIAYGMQVIEDTGESIPSLPKDKSIGDGMQDDPDGQETLPKFKRHRRSKKSKDKTVPESDDENLMDDIQAEPDGEEGDYSSRKPRSIADGRQVIEDTGESIPSLPKDKSIWDGMQDDPDGQETLAKFKRHRRSKKAKDKTVRESDGENLMDDIQADPDGEEGDYSSRKPRSVADVMQVVEDTGESIPFLSKDKSIGDGMQDDPDGQETLAKFKRRRRSKKAKVKVALQDDVGNEMEVPIDEDKSVLYDKAVQELDGGNLMGNIQAESGGDEVDRSLPKPVSVVDGIQDSEDVEKSISSLPKAKSIGDGMQDDADGEETLAKFKRPRRSKKAVVKDALEEVENEVVPTDKDKSLLKDKAVQETDDKPVKVKKLHRLKKREVKDSLEVDATKTTETYRDTNQNLKGNIADDSEDTLTHFKKKKKTPKSAKDLPKIASDSRQASASENDIKETANTGQVSKWQKTDGSSQGLPSVPAEKTRASKGAKLGKGSLESNPDGAEKGNVVKTSDKKKGKKQSDSQRVVRHVKKRRHGDMAYEGDSDWDVLMNGEEALESKSSVDKERLGRGRNKAGLISSMLGQVVMGEEAAVSAGLKFRQPGPVEKIKFKEVLKRRGGLQEYLECRNSILWLWSKDLRRILPLSDCGVSSVPLPDESPRDALIRDVYKFLDYHGYINVGVAVKKPKTKDDIEPKLKLSKESMQRENCGDKVVDAEEEVAFILGQIKDSEENLSPGQNQIQVEGENQTMLLNVEDAKRNKLAENCSGSTESDKKYSEECGIALMEIDETPVVVPHTTENLTAEAVRSSVVKTEVEIESAVTCKDEEELGTSDTSGSGGTENEIFLEKDHGVKIEVDPLVKVHSALDQIKNSYGEDEFSDIDSAKVLHNLRQHDLQVELQTEKQESKLNAVSEANDGNSVSESRKRIIVIGAGPSGLAAARHLQRLNCHVTVLEARDRVGGRVYTDRSALSVPVDLGASIITGVEGDVATERRPDPSALLCTQLGLELTVLNSDCPLYDSVTGQKVPATLDEALEAEYNSLLDDMLILVAQNGDVAMRMSLDEGLEYALKKRRIARSNSTGSEMASSKAHLNNSDCQSTTPDSSAGALETLSATVDSSLKAVSESNVSIVAGLENGINMESQPDKLNLSDNSSMEQQSLSPLERRVMDWHFANLEYGCAAELSRVSLPYWNQDDTYGGFGGAHCMIKGGYSTVMEALAEGLNIRLGHVVTEISYSTKDPESEGRIEREVKVCTENNQEFVGDAVLITVPLGCLKANTIKFSPALPDWKLASIKQLGFGVLNKVVMEFPKVFWDESVDYFGATAESTELRGRCFMFWNLKKTVGAPVLIALVVGKAAIDGQNLETSDHVKHAVMILRKLFGETAVPDPIASVATNWGGEQFSRGAYSYVAVGASGEDYDILGRPVENCLFFAGEATCKEHPDTVGGALMSGLREAVRIIDILENREDSMAEAEAMAAAQRQSDSERNEVRDMVKRLDAGELSSVLCKGSLDGEQKPLSKDALLQDMFGNAKTTAGRLCLAKKILQLPAAAVKAFAGTKEGLGILNKWILDSMGKDGTQLLRHCVRLLLIVSTDMIAVRQSGIGRTVKEKVCVHTSRDIRAVASQLVKMWIEVFRKEKANGGFKILKQLHGSGPNPSSTDAVKAKSKEQSSGKPSALAANDVKGNTDQSPSTPINKNPALHSLQVEDGSEQKKEANSVGAQTSDQYQHKKSEDCALSELEAAAIAAEEAARAAAAAAAEAYATSEAECSTFRELPKIPSFHKFARREQYVQKEDSDVRKRKWSGGVLGKQDCSSEIDSRSCRVRNWSVDFSATCGNLDSSKLAGSDFGSGNILPATDSNKKQRSHSNDVDSHSDLKERSAETGTVDSKSTKSRVQNSGSISGIKMPDSKAKQPYTLGSHSEVFDQSIQKLNEKCSSKIDQICIGKARHGNEGVAEAGSNRGSPGTKNGHKDEARGAEHIKKGITDYIASLLMPLYKTKKIDKDGYKSIMKKSTIKVMEHNTPAENAMTVSEFLDCKRRNKIRSLVDKLYLSVLQAGYSASFTAGLANFTASWW

>KAH9322201.1|Taxus_chinensis/1-2195

MHDNLMSDDSANVNSELGIGSTSSEGFSSQSAECMHVSECYGMESVTMKDVVGLDGSGEMVDKQERVDGKNLMIQVRADTEEESFDDVPLIASENDVYGMRAHQVGEESFQPNLKESFQPNLKPKDMMDGMQDDLDGEETLAKFKKPRRLRREEVKIVLEGDTANMMDISIDKNKNSSIENTVRESDEIPAKTKNPRRLKKGEVKESLEDDTGNMMEMSRYENQSSMKDKAVREADEIPAKIKKTRRLKKGEVKDILEDDTGNMADISRNENQRCVKENTVRGADETPAKIKKPGRLKKGKVKDMVEDDTGIMMEISKDENQCSINDTTVREADKIPDKIKKPCRLKKGEVKDSLEADMGNVAKTFAGTNQIFQGSNDEDLEDTFGATKMDDVPSKIKRRHKLKKLGTQDGLEADVENSVDTSTDINQSLKVNTAQEFEDNLGVKEAVEIPDKMKKPRRLKKREAKDDSEASVDNVAETATDMNRSLKGTIDQHLQDTLVFREANEVTAKFKKPRRLKKREAKDALELDVENAVESFRDMDQSLKCNISQNSEDNIEQFEKKIKSPKSSKKLTKVDSASRQTSISENDIKEAAGIDTGIKRQKTTASSQDSPSVPVEKTQPGKGSKAGKSFDSSLEGLQKDTAVKPSDMDKGKKQLDSQRTSRQVKKRKFGDMAYEGDSDWDILINGEETNENKTSDKDRLGRGRARGGLISSLLGQVVPGEETAVAAGLKMRQPAPAEKIRFKEVLKRRGGLQEYLECRNSILWLWGKDVCRILPLSECGISPVPLQDESPRASLIRDIYNFLDYRGYINVGVAVKKQKAEADSEPKLKLSNECTHIEKFGDKVVDAEEEVAFILGQIKGSEENGFNVQNLIQVGDESQILLLNMEESYKRAQDCSGLARSERKYAEECGVSLMEIDETPLVAQFKEDHAIMGTVRSSLMKNEIESESTVSNKDKEELGIGNISVSGRTEDQDSLGKDHHVKTEADLASKAPSALDQIKSSYDEDEFSDIDSAKVLHNLRKHELKNEPLFDKQEVILNSVAEDKGDTHSDLKDQKKIIVVGAGPAGLAAARHLQRLNFHVTVLEARDRVGGRVYTDRSSLSVPVDLGASIITGVEADVATERRPDPSALLCTQLGLELTVLNSECPLYDSVTAQKVPIDLDEALEMEYNSLLDDMVMLVAQNGDTAMRMSLEEGLEYALKKRQMARSSTSCLDLVSCRSPLNDVDGVGMVQDSSMTGLKELDGMADDSLTKAPDGNVLEVAGAENGTQMDSSCNNLKLSSNSAGEQKCLRELERRVMDWHFANLEYGCAAELRRVSLPYWNQDDAYGGFGGAHCMIKGGYSTVMEALAEGLNIHLGHNVTEIEYLVKDSNLKCETKKEVKVCTANGREFIGDAVLITVPLGCLKVNTIKFYPALPDWKLASIHRLGFGVLNKVVMEFPKVFWDESVDYFGATAESTELRGRCFMFWNLKKTVGSPVLIALVVGKAAVDGQNTETSDHVRHAVTILRKLFGETVVPDPIASVATNWGSDPFSRGAYSYVAVGASGEDYDILGRPVENCLFFAGEATCKEHPDTVGGAIMSGLREAVRIIDILENRVDSMAEAEAMAAAQRQSESERNEVRDIKKRLDAGELSSVLCKGSLDGEQKPLSKDALLQDMFGSAKTTSGRLHLAKEMLQVPAAAVKAFAGTKEGLSILNKWIQDSMGKDGTQLLRHCVRLLQIVSTDMMSVRQSGIGRTVKQKVINHTSRDIRAVANQLVNTWVELFRKEKANGGFKLLKQSDGLAKMSGLSDVVKTKYKEESSGRLSNLAANGYKGIAVEMTSAKDHSTSGLNIKISEMQFVSVDAISEQKKESNFVAAQNCDKGQVDKPDDGALSEYEAAAIAAAEAARAAAIAAAEAYATEAECSTFRELPKIPSFHKFARREQYVQKDDSEVRKKKWSGAVLGKQDCSSEIDSRSCRVRNWSVDFAATCGNLDGSRVGGSDYGGGNILPALDSNKKQRSQSNDIDSHSDLKERSAETGTVDSKSKKHRVHNIGNFSGGKIHNTGDKCKPYSLGSHSETFDQSIQKLNDKYSIKIGQLPIDQSRNGGDDKCIAEAGSNIGSPRTKNVNKDEARGSEHIKKGITDYVASLLMPLYKNKKIDRDGYKSIMKKSTIKVMEHNTLAENAMTLLEFLDIKRKNKIRSLVDKFIERYMANQQ

>TnS000858491t03|Gnetum_montanum/1-1805

MEADTKVDAQDLKRSVDIDESMAVQENGDLVAPHSRSRTGSIASEEGLPDIENSQIQFQGTMGGVQYESDSEQPIAKFKRPRRSKKAKLADTLEDDTDKGNAVSAGSPSDLLKENMINENGNKMKSSSFLGNTGDVMLVDTDGEEEVLDKFKRSYRSKKAVLKAALEDDVDNEREFNQEREGIFPHMNTHVKDGENEGKQPSPSGKLEHRVEANDDEEGNLVKIRRPQRLKRGSKESQNDKPIENMGMGAENESTGFGRIEDKPNEVLKEMKLNLGREESDQIMQKNNIDEFQSDPIEEETLSKLIKGKKLKNSAIDDTLSDAIEHGKQSSSSKLKSIPKNNDTEAESKKTKVKKAKTMGKGKEAPKLSGDKPEELKDIEVDNREEALSQLKRKRARKVVKGQAKPDTLSRQESATETDLSEAKVEVGSKHQKAGKPGKNSSDCNPVGAESGSTLNSGKERRQSDIQRTSRRTKKRRHGDMAYEGDSDWDVLMNGETMTENDSNDDKERFGYRSKGEGESTPGRKAAVAAGLKSRSVIPAEKIRFREVLKRRGGLLEYLECRNSILSLWSKDVLRILPLSDCGVSEHVQPDEAPRSALIRDIYKFLDSNGYINVGIAVDKPKAKEDFQPKLKISKESIPTEKVGDKVVDTEEEVAFILGQIKGSDENHSLEQRGTKAEGESQTSLFSAEEAKPKADAANCAESFALNRKDSDESGIAIMDTDENSVVAIKNESNLKNSEKQFLTVKHEDDPMVNEPISKNIKFYGEDEFSEIDSAKVLHDLGRQNLLSSKEMDERKRILVIGAGPSGLAAARHLKRQNFNVLVLEARDRVGGRVYTDRTSLSVPVDLGASIITGVEADMTTERRPDPSALLCTQLGLELTVLESACPMYDSVTGTKVPADIDEALETEFNALLDDMARDVDQYGDETMSKSLEEGFENALKKRRSSHKVNKHSECMVALHEEHLEGGLDEKFAAKENEPAKLDCNDLNLDCEILKPLERRVMDWHFAHLEYGCAAELKLVSLPNWNQDDVYGGFGGPHCMIKGGYSKVMEALAEGLNIHCGQVVTDIKYNIEDSDAKDRSKKIVKVQTDKGQEFIGDVVLITVPLGCLKSNAINFSPELPEWKQFSIKRLGFGVLNKVVMEFPSVFWDESLDYFGVTAESTDLRGRCFMFWNLKKTVGAPVLIALVVGKAAIDGKNIENSEHVKHALKVLRKVFGEDAVPEPIASVTTNWGADPFSLGAYSYVAVGSSGEDYDILGRPVENCLFFAGEATCKEHPDTVGGAIMSGVREAVRIMTILENGKDLFAEAEAMVAAQRQSDSERTEVSDMMKRLEASEYPSRDAVLQDMYGNAKTTVGRLFLAKKMLQLPESSVKAFAGTKEGLSILNRWILDSLGKDGTQLLRHCVRLLLVVSTDMLSVRQSGIGRTVKEKVCVHTSRDIRAIASQLVKMWIEIFRKEKANGGLKLLKQPHVTVSNSGSLDVLKVKSRDTKLATSTNIDTKGNSDLSNSGKEHKKENSTPAFYSADEGQQRIAEDAIIAELEAKAIAAEAAARAAAVQASSQAECSTIRELPKIPSFHKFAKREHIAPKDESEFKKRKWSGAVLGKQDCSSEIDSRSCRVKNWSVDFSATCGNLDGLAGSDYGGLDSNRRQRSHSNDIDNHSDLKEQSAETGMGDSKASKLQGQNSGIKIPSKHRHGSIDKGDDEACNNRGSLSSRNVNKDEPRGPEHIKKGIADYVASLLMPLYKTRKIDKDGYKSIMKKSTIKVLEHNTSVENAMSVAEFLDCKRRNKIRSLVDKFIERHLANHPS

>evm.model.Chr18.2572|Isoetes_sinensis/1-1559

MEDRAETTGPDKIEDADSDDDNITLQQLARKSFRKRKIPVLKKEAFSVDEEHGLEEPTKDLVPPVQESSRSEEDLRANGAKELSENQASDDVAVESRARKRARRQESQSICKPESMQKAQPERKKISSVRGKKSSETKKHLDSERTLRKTRKRRQEDMTYEGDMDWDILVGDADLSRDANGGSEKDRPLKGKYNGEAAVAVGLKVRTLITAEKVRFKDVLKRRGGLQEYLECRNMILGLWEKDVKHLLELTDCGVSCSPHPNEPPRLTLIRELHEFLECHGYINIGIVKEKKVHNPNVKVESDSSGIVDQHMDKVIQHRSKTSTMISKKPYEANGQKNLAAADENAAEEAALILGQIRDSEDASLENSPTMEDAKDLNGSDMNACNSEEEKIKDNENTFLGHGATEVIPVDMDIISNREDHVVEEAKTLKAKTASAISLVGQDTKTEETGSERQLDEINGAEVLHNFHKYELKSNSMASSPADGAHGRKAEDQARNRKKVIVIGAGPAGLMAARHMQRMKFSVTVLEARNRVGGRVCTDRSTFSVPVDMGASIITGVEADVATERRADPSALFCRQLGLELTTLRGDCPLYDSVTGCRVPDDLDEALEAEYNGLLDNTLYLVAQNGDAAMRMSLEQGLEQALERRRSNLVRKMGRTSVLTSPNKVSIIERCENDESIQNEFGESGVDGKERSDSLEDDDQQLNPLERRIMDWHFANLEYGCAAPLSHVSLPYWNQDDIYGGFGGAHCMIKGGYSTVMEALAEGLDIRLGHVVSEIVYGLQEGKAKGRMLKVRTNEGEEFIGDAVLVTIPLGCLKAQTINFIPSLPEWKLASIQRLGFGILNKVVLEFSTVFWDESVDYFGAAAESTDFRGQCFMFWNLKRTVGAPVLIALVAGNAAIEGEKQEKPNLVAHALKVLRKLYGDEAVPDPKASDVTKWGSDVYSRGSYSYVAVGASGEDYDILGRPVDNCLFFAGEGTCKEHPDTVGGALMSGLREAVRIVDILENRGDLTAEVEIMSAIQQQSDSERNEVRDMMKRISSAETAADLSNNNREMNGTWDGNEQAFSKTDVLKDMFANAKTTAGRLLLAKEMLQLPVKEVRSFAGTKAGLLVLNSWILDSMGKDGTQLLRHCVRLLLAVATDLLSIRQSGVGKTIKEKVCVHTSRDIRAIADQLVKMWIEVFRREKLVHGSVKSARRANVNSGLASNIQKHHRGLSISQDNSVSNLKGDTPSQQEITDEASEQRPDTVSGSESQLHEPEISEIEAAALAEAEAARAAAIAAAKAYEVSYSSLPDLPKIVSFHKFAKREKDINNDFRKKRPEQTQGASSISIVKPLSSIGSGKTLSSVSIGFKKVSNWSLDLTGSCSNVNSPGRLHLDMMGGCTYLAQDTLPSTVDDGNTGFNASLEHLAMKEEDSPFLTQDAIAEPETTEELGVSTGPGSNCQTATVVIPRLDLESSKVSDLTQKSGHQDKSASESSSYEQIKKAVSDYVAHLLAPLYKTKRLEKDGYKTIVKKATAKVLERDGVKDITVEVSEFLNCKRKNKIRALVDKLVEKHLACTIASQ

>Adc01587|Adiantum_capillus-veneris/1-1577

MHSTSEGKDTQSKVEAGDLRGKRACLRGMSDEIECRPGDPCTDRSDNGEAQGSDLRSKSMRKDDEMTISGVAFKLTQKPRSLRGNGVKKRTWGWIDVWKSFDSKASKNSVADRTTRQSRKRTRREVASDGDMEWEAVTGESGDANRKKPLRCKTWGGLSLSVEELNGEIAAVTVGLQADMPSPSERIRFKDVLKRRGGLQEYLECRNLILGLWERDVRHNLCVTDCGLSSVASLEETPRESLLRDIYDFLNYHGYINLGIISKKLKSETQTADCNFLPATANINSDYDFNEKNQKEPSGEGSLLHEKGTRIELPGDYMVLMNAVSATDPEPMDIDEKPLVNPAAFEEHIDEQKLSLPAHCADEESLLDIANRLKARNKKKRAGLLKLEKSVKADEEQSLRGGDATEEVSICKEKVEALQVEPAKCAAEVNDTHLSSKEEMALNEGIYARQKYCKEQTTESIHNQEELGRLIFPDNLIEKLPLPSVGKVETFPRVIIVGAGPAGLAAAKHLLNLNVSVTILEARKRVGGRVFTDRTVLSGPVDLGASIITGVEADVSSERRPDPTALLCRQLDLELTTLGGDCPLFDSVVGTKVPADIDEALEAEYNSLLDDTVMLVAQNGDVAMQMSLEEGLELALKRHKEQEKRASTLFRGTDSLSEKINQFEQDGHAVCMRESSESTKKSLGISPLERRVMDWHFANLEYGCAAELNRVSLPYWNQDDVYGGFAGPHCMIKGGYGTVMEALAEGLPIHFDQVVTRIEYSSKDEQIVDRGRVKVKTSSGNVFMGDAVLVTIPLGCLKLNTIDFVPSLPAWKTASIQRLGFGILNKVVMEFPVAFWDESIDFFGATAEHTESRGRCFMFWNLKRTVGAPVLSALVVGKAAIEEEKKESTESIEHALTVLRSIFGEASVPQPTGSVVTKWGSDPYSRGAYSYVAVGASGEDYDIIGRSVENCVFFAGEATCKEHPDTVGGAIMTGLREAVRIVDVLYNRVDSVAEAEAMAAAQRQSDTERNEVRDMTKRLTAAELSIAIRKDGGILDEDKEPFNKAILLQDMFGCAKTTSGRLLLAKEMLQLPASSLEAFAGTREGLTILNSWIQDSLGKDATQLLRHCVRLLLVVGKDLPSVRQSGIGRTVKEKVVVHTSRDIRAVASQLVKLWVDTFRKEKAAGKIARPLQQSPTGFRNTLTMSKVMHRNMKSGTSPMVSRDKVLHPAVVSDGSRQQVSEKESTYQSYSTELRDAEVEKEKSTAICSNSSWRELVYNPISDSEAAALAAAEAAVAAANAAAKAYETAEAASSQLELPKIVSFHKFANKREYMILPEATKRRSLERDALILHGKDQVMMGVDARNCKVRDWSIDFTDSCSYLRSPNVVLPVQDGKDGSKETELSGLSSACTDNSKVSFRIGKQLFSEQEEATVNMKEAASAGSPVKEAQEQTSGLVSELAPSCNISMGSSRNGRFFTESNIDDTARAITDARQCSREQLAVVRSTRDEARHLSRCSEHKKAVTDYVSNLLIPLYKTRKIDKDGFKSLVKKSAVKVIERQTELEAAMEVSQFLDTKRKIKIRTLVDKFVERYLENLVK

>Sacu_v1.1_s0017.g007295|Salvinia_cucullata/1-1889

MEIKRPPQFPIFAGSPTESAGPAVGDENYERLENAQYRRRGSPDDNLHSDCTRLLENPLRCTHVFLFHESDEDGKKEMHGNTSVSRSSVMRNASNRFVNEDGDKQVDVLEERYLPKASLIQEKGSTKEKPDIDGSSNVDERRSDQMNYQSKSTQKPIKSVLQDMEVEGKSDSSLQKEECKNVCNASLMTEPNGEKSLEMHIMEETQFEVDQISKYTETKKVFVTADVQDIYDAIIRDEKMEVNKKDPVGIILGNRDSGLIHGVHDPFEASNSTGHRNIENDLTDGIVGKLPHECEAALRTQLEGDGRKNVQEGGDKLLDTDIERNPYAGHLYEACRKSQSKTSLSRNIFYEQEKSILQNVDNETRSHRSVSPEINGYENLGEEEEKGEPNRDANSIGCEIMNEVYRKESCTTAKLIQKPRSTRNSNGTKQSWSVSDVFQEASQDLEIKPENISSEYKQDKRPRKRRQSETTLDGDMEWEALTGESEIAPGKLRKSRRTKSVFESGPLSVDLNGEVAAVTVGLRCSTLTPAERIRFKDFLKRRAGTQEYLECRNKILGLWERDVKHVLSLTDCGLSPVASEEETPRISLIREIYDFLNYHGYINLGITLNKFEVPSTLLIKLPEAVGGSNVLIDSQRLSTDCRAIDITASDRHSSSVVTEFMDIDEKLHETRENEFCKHPEEEQLSAELGKVISPMKSGEESLLDMLNKIKAKNRRKKAALQKSGKITNKHEKDKLSESLNNGTCKDSMDSVRMEVNFTTCRHEKNESIPEVEMCEDGLREKNMELDTPYVNTGGADTCISKVAEGLTISQECDAKKKQAIDSSVSVFGCAETTFTVNKVPEKNKRRVIIVGAGPAGLIAGRHLKNIGAQVTILEARQRVGGRVCTDRTIFSGPVDLGASIITGIEADIAAERRPDPTALLCKQLNLELTTLGGDCPLYDFVTGSRVPCQIDEGLEAEYNSLLDDTIMLVAQDGDVAMQMSLEEGLELVLRKRLEQGKKASHLPMEVNNFLDKSTTDDSYTEQTSCSNYSYNLLEKVADSSSLERRVMDWHFANLEYGCAAELKRVSLPYWNQDDVYGGFAGPHCMIKGGYSTVVEALGNGLTIKLGQVVTKIEYHCQEELGRVKVQTENGDVFIGDVVLVTIPLGCLKVGSIEFIPSLPDWKTASIQRLGFGVLNKVIMEFNSAFWDESVDFFGATAEEGEMRGRCFMFWNLKRTVGAPVLSALVVGKAAIEEEKKDDAESVKHALTVLRKIYGEDSVPQPIAFTVTRWGSDPFSRGAYSFVAVGASGEDYDIIGRSVDNCVFFAGEATCKEHPDTVGGAIMSGIREAVRIADVLYNRSDSIAEAEAIAASQVQSDTERIEVRDMRKRLAAAELSMAIRRDGGKLEEDEEPFSKATLLQDMFGNAKTMSGRLLVAKEMLLLSVSSLKSFLGTIEGLKILNVWIQDSLGKDGTTLLRHCVRLLLVAGKDLSSIRQSGIGRTVREKVIVHTSRDIRSIASQLVKLWIETFRREKAAGGIIKPLRHASVVKTPHMIAKMKMKAKSAEKANDKSSAFIVPTVALKHPDADIDCKDSVSACSDTSQNLLVPMSDSEAAALAAAEAAKAAADAAVKAYETAEAASAQFELPKILSFHKFAKREHIVTSDSTKRRPLEQEMLLLHGKDRVTTGVNARNCKVRDWSIDFSDSCNYLGSPSLLVMQNIPNTSKSGMVDIVAGVHDGLDGRHSLQKDESAHSMQEAIDETSERSDNHESKGNSVSGSVPPIKNLSNTLDTSTVSTAKQNIKASEPPANADNLADLIDSRRNSEHKKAVTEYVSSLLLPLYKAKKISKDGFKSIVKKSAVKVIERQTEEEVSMEVSQFLDTKRKLKIRALVDKFVERYLEGEKNSAWIHE

>Mvestita_C1619g21751-RA|Marsilea_vestita/1-1789

MVGVDNGSHALDSGAVQETDVVLLQTGNLPVAGEEIQLSMAVCEDSIQKADVSALEKAHSEITEGANIKGNQTHQHALEDLSIKRKRVTTEAKQESDVLFQSNGMKRLGQEEIEETTNHVLETPEVNVCERKADVMLSQSGICPDVAEFPKKMEVHREPEITDDRNNKVYCEKAFSETVLIKDDVIQNIGFLDGGERVQSADISPELEDAEQQIQTDCSLIERNSTQEDVNRKLMGRGLCCGGRRNVTIADAEDNDAKEKLNLLFSELKDDPAEQNVREYSVVKKESDSRFLKEGSADISEDSESVESEKTDKQIMDVSLPEEFDGACELGRNISQDSGGENRNTECSGEDSQSSKQKLIQKSRSSRTTSTTKKNKWSLVDVLQPHGKTNEKRLPKNSKKRRRNEMSSDGDMEWEALTGEKRPLTRKSTRTRNVVSLVPFAQALNGDVAAVTVGLQVSTPSPAERVRFKEFLKRRGGLQEYLEYRNMILGLWERDVCHVLPITDCGVISKASVEENPRASLIREIYDFLNYHAYINLGITSEKIPATTNVSGQLTESLISLEGSITSDPMDVDEKSLLSGSTIQAHDQINSELPDCLEDESLLDMVNRIKAKNKKKKGNLQTSAEPGVQRLVVCLNEQSNIVSLSNCKESENDETIKIEIEDCNSIKKESQGSCISSIGVKSQEMSDPADELSKPGSLLKEAQEQKSLDDGVLSITPKDDRIYGNESIDCKLEASECTEPEAKDDKVQVCEVDVNNVPNGTLEQGNKTDHRRVIIVGAGPSGLIAGRHLQNMGVNVTILEARHRVGGRVCTERSIFSGPVDLGASIITGVEADVATERRPDPSALICKQLKLDLTTLGGECPLYDVVTGSKVPAEIDETLEAEYNSLLDDTLLLVAQKGDAAMQMSLEEGLEYALKNRREQGKKSSSYSNRTQKSQTSDDSVSAGQSLLDKTIDNSSLERRVMDWHIANLEYGCAAELSRVSLPHWNQDDVYGGFAGPHCMIKRGYSTVMEGLAEGLTIKLGKIVTMVEYSRKDVQTKSGERVKIHTKDGDVLMGDAVLITVPLGCLKAGSINFLPPLPEWKVASIQRLGFGVLNKVIMEFPHVFWDESVDFFGATAEKPEIRGRCFMFWNLKRTVGAPVLSALVAGKAAIEEEKKEECDSIQHALTVLKRLFGEAAVPQPTAYTVTRWGSDPFSRGAYSFVAIGASGEDYDIIGRSLENSVFFAGEATCKEHPDTVGGAMMSGLREAVRITDALYSRSDSVAEAEAIVATERHSDSEINEVRDMSKRLAAIELSMGVRKEGGKVEEEDKPINKAMLLEEMFKSAKTISGRLLLAKEMLQLPTASLKSIAGNREGLTILNNWIKDSMGKDGTQLLRQCVRLLMVVGKDLASVRQSGIGRTVKEKVIVHTSRDIRAVASQLVNLWMQTFLRGKAANGVARTLRQPSLNADVKAKVTNKDSKVITDSKDKTPSDAILKHSSPEKENRPSYSSNVESSAGDTNKNGAAPESVGGNQTVDSSVPISDSEAAALAAAEAARAAAEAAVKAYQEAANSQVELPKILSFHKFAKREHITIPDTNKKRSLEYDMLMLHGKGKITTGVDARNCKVRDWSIDFSDSCSYLGSPSKLAVPETRTDNIMCIENVNDIRGTHIQTVIGIEKDTSSGEKRDETQVDRSNKSPLESKLCSITQQTVLTQPSSHDGSGHSGRSSEHKKAVTDYISTLLLPLYKTKKIDKDGYKSIVKKSAVKVIERQTEQEAAMNVAEFLDAKRKVKIRMLVDKFVERYLENGGK

>Mvestita_C347g21749-RA|Marsilea_vestita/1-1789

MVGVDNGSHALDSGAVQETDVVLLQTGNLPVAGEEIQLSMAVCEDSIQKADVSALEKAHSEITEGANIKGNQTHQHALEDLSIKRKRVTTEAKQESDVLFQSNGMKRLGQEEIEETTNHVLETPEVNVCERKADVMLSQSGICPDVAEFPKKMEVHREPEITDDRNNKVYCEKAFSETVLIKDDVIQNIGFLDGGERVQSADISPELEDAEQQIQTDCSLIERNSTQEDVNRKLMGRGLCCGGRRNVTIADAEDNDAKEKLNLLFSELKDDPAEQNVREYSVVKKESDSRFLKEGSADISEDSESVESEKTDKQIMDVSLPEEFDGACELGRNISQDSGGENRNTECSGEDSQSSKQKLIQKSRSSRTTSTTKKNKWSLVDVLQPHGKTNEKRLPKNSKKRRRNEMSSDGDMEWEALTGEKRPLTRKSTRTRNVVSLVPFAQALNGDVAAVTVGLQVSTPSPAERVRFKEFLKRRGGLQEYLEYRNMILGLWERDVCHVLPITDCGVISKASVEENPRASLIREIYDFLNYHAYINLGITSEKIPATTNVSGQLTESLISLEGSITSDPMDVDEKSLLSGSTIQAHDQINSELPDCLEDESLLDMVNRIKAKNKKKKGNLQTSAEPGVQRLVVCLNEQSNIVSLSNCKESENDETIKIEIEDCNSIKKESQGSCISSIGVKSQEMSDPADELSKPGSLLKEAQEQKSLDDGVLSITPKDDRIYGNESIDCKLEASECTEPEAKDDKVQVCEVDVNNVPNGTLEQGNKTDHRRVIIVGAGPSGLIAGRHLQNMGVNVTILEARHRVGGRVCTERSIFSGPVDLGASIITGVEADVATERRPDPSALICKQLKLDLTTLGGECPLYDVVTGSKVPAEIDETLEAEYNSLLDDTLLLVAQKGDAAMQMSLEEGLEYALKNRREQGKKSSSYSNRTQKSQTSDDSVSAGQSLLDKTIDNSSLERRVMDWHIANLEYGCAAELSRVSLPHWNQDDVYGGFAGPHCMIKRGYSTVMEGLAEGLTIKLGKIVTMVEYSRKDVQTKSGERVKIHTKDGDVLMGDAVLITVPLGCLKAGSINFLPPLPEWKVASIQRLGFGVLNKVIMEFPHVFWDESVDFFGATAEKPEIRGRCFMFWNLKRTVGAPVLSALVAGKAAIEEEKKEECDSIQHALTVLKRLFGEAAVPQPTAYTVTRWGSDPFSRGAYSFVAIGASGEDYDIIGRSLENSVFFAGEATCKEHPDTVGGAMMSGLREAVRITDALYSRSDSVAEAEAIVATERHSDSEINEVRDMSKRLAAIELSMGVRKEGGKVEEEDKPINKAMLLEEMFKSAKTISGRLLLAKEMLQLPTASLKSIAGNREGLTILNNWIKDSMGKDGTQLLRQCVRLLMVVGKDLASVRQSGIGRTVKEKVIVHTSRDIRAVASQLVNLWMQTFLRGKAANGVARTLRQPSLNADVKAKVTNKDSKVITDSKDKTPSDAILKHSSPEKENRPSYSSNVESSAGDTNKNGAAPESVGGNQTVDSSVPISDSEAAALAAAEAARAAAEAAVKAYQEAANSQVELPKILSFHKFAKREHITIPDTNKKRSLEYDMLMLHGKGKITTGVDARNCKVRDWSIDFSDSCSYLGSPSKLAVPETRTDNIMCIENVNDIRGTHIQTVIGIEKDTSSGEKRDETQVDRSNKSPLESKLCSITQQTVLTQPSSHDGSGHSGRSSEHKKAVTDYISTLLLPLYKTKKIDKDGYKSIVKKSAVKVIERQTEQEAAMNVAEFLDAKRKVKIRMLVDKFVERYLENGGK

>Ceric.04G013700.2.v2.1|Ceratopteris_richardii/1-1488

MHEESKKCMQMHEVSNQMVSDIKLSIEGLQSDLQVQPFFLGKAVATGMNQTTSKQSDEEFLLDIKNRIKAENRRKKQGPNDIPASLEFGCKRDSINSEGDGDLNINMEESNKQMSSHNGEALMDEMNDTGSCPKKVIIVGAGPAGLAAARHLQNMGIDVTILEARGRVGGRAFTDCRTFSGPVDLGVNVVTGIEVDLTTEVRPDPVALICKQLNLGLTEIEGQCPLLDVVNGAEVPFDLDMAVEADFSYLLEEASKLSIGKDDMKQFSLQKGLKVAYKRIETLLKDEHVRKKQRTDQRRQITGLFSSDSSNFPLSQRILNWHFGMLQLQCGAELNKISLSNFRQGIAHGGFAGPHCMLKGGFSTVMEAFGSGLTIHLEHEVTEIEYSCRNEKNNSCEVLVRTKEGNAFSAEAVLVTIPLGCLKAGSVKFSPPLPEEKILSIRRLGLGLVNRVVMEFPVSFWDENVDYFGSTIESPGLRGRYFVFWNLQRFVGFPILSTSIVGEAAWEDESKEVSEIVEEALSVLRRLFGVVAVPEPKAFTVTKWGSDPYSRGSFPYVAVGASGKDYDIIAQPVENKLFFAGEATCKEYSSTIGGAMLSGLREAVRILSVLHNRIELLMEAEGLHAVEKQLSSPSHEVEEIANNLSEGGISVSTKTGGGKVGRIMGPLKKVTLLQSLYRVPKTNAGRTLLAKEMLQLLDDSLINFLNTKDGLLVLNSWIQGALRRDGAHLLWLCLRLLLVIGSPPSLIQCSGILSTLKEKVLKHPSRGVRAFVRELLGMWLGGKMKGKLNHDIGRLAHRSEHSEKLAKKKFRDSSISLSQASREQKQNLLASDKDNLRVTSKDVRSLDSLIKKSPTDMKISSQEKSDCKHDLSPLREGMHSESIVKKRPRDAKSVLHQARAKQRSKPSISRDICQDFSEGAEDLERPVKKKIEEMNKSSITKKDMSFIDSEREKETLGPLAKKKVRSTKTLYQARGEHSSTLSLNNKPAIQGASESKTPALQPSARRKVRDEKISLCDANTSFGASHEVMQDSKTSIKKKSEDVKLPKKHEGAEIGCMSQEFSERQTQVSGCLSAEVVPSLVVEGARIGNSEQTWESHEPPIEPSLVTSGTTDVAAKNSDPEKLPNVKQPELPKIVSFQKFAKREHIDTNKRRPLLQETLLLYGRDLVTSGVDARQSKVRDWSIDFSDSCSYLGSPTLLAMLDNASSRDGSSAVIPSVPGISSTEIAGERHESGVTSNQDEAVLPSSKEITDDASAGSSLRDSQRRQSGICLASLNSDNTPLITAPSNEHCKHFELSSHQDCTADAKLETSISVHPSTDVDGKEGHSHSVEKRPDSAEGHCCSLNEANAASAGSSVRLGTEQHNETQNVVEGSPVHLLLDGGKSFVSTGALSKSAYVESQNHVLDTTEQVKKSVTDYVAQLLTPLYKTRRINKESFKSIMKKTIAKVMERRTDEELVMDPLEFMDTKRKIKIRSLIDKLVERYLEGVHR

>Ceric.04G013700.1.v2.1|Ceratopteris_richardii/1-1925

MEIFISDSVDNTSLIDEQTNECREPGSILKEVGFVRENLLNHFVECELGLSSKTADSSVSYREQNLLDVIVKKEQSVIDSSESIPRAEEIGFVDEMERESTMDEEHVETMKMQVRPSSNKNVKLPMPVHMYSRRKKGKNVPILTKDSINGLKDDTVLSGFEHVRISEIDNPDDTAKVQNLCHGKKMETAICLEEEHVHANTVCTNVSDDMLKLDSSKFVELDISLQVEKEQHNQNRVSEKKRGCNRIEVCVSPSHEVDIEKFLKSDHANEVEGVATLIDKGRYAAIRAGLRASEVIPVEKAWFKDFLKQTNGVPQYIDHRNFILGLWEKDVFHNLSITDCGLSASPSPDETFRVRLLRDIYFFLNYHGYINMGLVSKRPRLSSDTEAGGMVRTPPVSYSIMDVNASLALVEKTGRTGSLCLRSGREDVSDDWKFVVPMHEESKKCMQMHEVSNQMVSDIKLSIEGLQSDLQVQPFFLGKAVATGMNQTTSKQSDEEFLLDIKNRIKAENRRKKQGPNDIPASLEFGCKRDSINSEGDGDLNINMEESNKQMSSHNGEALMDEMNDTGSCPKKVIIVGAGPAGLAAARHLQNMGIDVTILEARGRVGGRAFTDCRTFSGPVDLGVNVVTGIEVDLTTEVRPDPVALICKQLNLGLTEIEGQCPLLDVVNGAEVPFDLDMAVEADFSYLLEEASKLSIGKDDMKQFSLQKGLKVAYKRIETLLKDEHVRKKQRTDQRRQITGLFSSDSSNFPLSQRILNWHFGMLQLQCGAELNKISLSNFRQGIAHGGFAGPHCMLKGGFSTVMEAFGSGLTIHLEHEVTEIEYSCRNEKNNSCEVLVRTKEGNAFSAEAVLVTIPLGCLKAGSVKFSPPLPEEKILSIRRLGLGLVNRVVMEFPVSFWDENVDYFGSTIESPGLRGRYFVFWNLQRFVGFPILSTSIVGEAAWEDESKEVSEIVEEALSVLRRLFGVVAVPEPKAFTVTKWGSDPYSRGSFPYVAVGASGKDYDIIAQPVENKLFFAGEATCKEYSSTIGGAMLSGLREAVRILSVLHNRIELLMEAEGLHAVEKQLSSPSHEVEEIANNLSEGGISVSTKTGGGKVGRIMGPLKKVTLLQSLYRVPKTNAGRTLLAKEMLQLLDDSLINFLNTKDGLLVLNSWIQGALRRDGAHLLWLCLRLLLVIGSPPSLIQCSGILSTLKEKVLKHPSRGVRAFVRELLGMWLGGKMKGKLNHDIGRLAHRSEHSEKLAKKKFRDSSISLSQASREQKQNLLASDKDNLRVTSKDVRSLDSLIKKSPTDMKISSQEKSDCKHDLSPLREGMHSESIVKKRPRDAKSVLHQARAKQRSKPSISRDICQDFSEGAEDLERPVKKKIEEMNKSSITKKDMSFIDSEREKETLGPLAKKKVRSTKTLYQARGEHSSTLSLNNKPAIQGASESKTPALQPSARRKVRDEKISLCDANTSFGASHEVMQDSKTSIKKKSEDVKLPKKHEGAEIGCMSQEFSERQTQVSGCLSAEVVPSLVVEGARIGNSEQTWESHEPPIEPSLVTSGTTDVAAKNSDPEKLPNVKQPELPKIVSFQKFAKREHIDTNKRRPLLQETLLLYGRDLVTSGVDARQSKVRDWSIDFSDSCSYLGSPTLLAMLDNASSRDGSSAVIPSVPGISSTEIAGERHESGVTSNQDEAVLPSSKEITDDASAGSSLRDSQRRQSGICLASLNSDNTPLITAPSNEHCKHFELSSHQDCTADAKLETSISVHPSTDVDGKEGHSHSVEKRPDSAEGHCCSLNEANAASAGSSVRLGTEQHNETQNVVEGSPVHLLLDGGKSFVSTGALSKSAYVESQNHVLDTTEQVKKSVTDYVAQLLTPLYKTRRINKESFKSIMKKTIAKVMERRTDEELVMDPLEFMDTKRKIKIRSLIDKLVERYLEGVHR

>Mvestita_C70g20412-RA|Marsilea_vestita/1-1659

MEGNPSSPSRCVGFDSESLLEAGMVNETSVGVAVGGSLDNVSSANNSSGSNSMRDEEEMMTKLMSSVRSQDQKSKEGTNLVQSPVMSASPKQTYSRRKSHQKESCFSATSLQASKSSGIAEFSQEKCVPQEPAVSFHGIATPNPVKELKHIENATTVSRFESQVITSLERTRFKEIIEKSGGEEQYLRCRDFLLGIWNRDVHNRLQADDSRITHSENNFDKHLLHGVYDFLNYHGFINFGFVKTKVLETIDEMNNEACNNVEEQLTSFGRKRSSANYDTTKEFKRRLRHGIHSDNSLTNLELKPLDCGHQDASVTSTNVSSLEVKSSMDCADEESLLDMKNRIKAYRKKKDTMMKTLTTESKPGPVFEPKARKLTTTQNQIGNDISNEKSSECQHDVKLPSMVKEEMQTVHNDKQARKKVIIIGAGSAGLAAGKHLKNMDIEVIILEARNRVGGRVFTDGSSFSGPVDLGASVIADMETNLYQSLSVLLSKQLVLELTSLQDECPLYDTFTGRKLPDDIIESSKKQYHLLLDEIESLVAQQHDVVSQMSLKDGFDIAFQKQNSQHRQKNLKQALEWHLCDIETEYGVDLSKVSLRHWKHGRIAQSVKQWILKKGLGTIMDALAANLDVRLNQVVCKIDYTCRDEANSSSPCQVQVWTENKDMFMADAVLITVPLGCLKSESIKFCPPLPDWKMTSIRRLGIGFLNKIVLEFPFVFWDETLDYFGTTGGRTEIGSCFKFCSLKRTLGLPILSAVCLGSPPSKDLQKEAEVLLEQVLLVLRRLFGETKVPQPTAFHVTRWESDPFSQGGYSYVAIGASPDDYDTLQFPIDDRIFFAGEATFRARPNTVDGALISGVLGAVRILDVLNKRASVMENEEICNANKQSNQATDEVQILKDRIAAVDNDNNGLPGGDEEIANRIECIAKLLTFPRTVSGRLLLARQMQKMPDAAVKKLLRPKDSLVVLNEWIQGCTSADGLELLRMCINLLLIVGRDDFSLQESGINETVRLKVLMHGDPSIASLARQLVQSWEIEHTSSKNITVLKSEERSSSHDFQKNTKVELTCTNDLVTDLPSSVSDLKDLESRKKREREQTNSVPNELLAGIITKKRSEIDPNIRVPSKQRDDEHESLCKANTGYEEPVNIGVVSKKATDENPLAEKSRSSPMSCPLVSSSNSQSNKPEKSTLASTNMPNEREAGHVSKEDNFPETNFEAISELEAAARAAAEAAEAAAKAYETAEAAKRKHPDLPKIVSFHKFAKRDEIGNSSRRRPLQRETLLLYGRDVVTSGIDARQSKVRDWAIDFSDTCSLLGSPSMSSFYDCGVSSKDGFSSAVPNLPGISSTENVVERHESCFTSNQEEGLPASKENTDDVSAESSLRGSHGRKHDLCLASLNSGNTPLCSTPSMEPHKVSKSISGLSPEKLGESVSPLPSLDPEKSADNIMTQGHADADKLRESIYLSPSAYRERLIKDVSPGLFSNKIRENLTQSFADPGSLYPNVYQKSPDVALKHESIECPYPDTNVPDDVSAGSSLRVASLQDEGLKKRESIYRQITVQQDKLQDPSPILSKHKVQECGGPFANGCDHIKKAVSDYVAQLLTPLYKTRRIDKETYKSILKKSTMKVMEKRTEQELAMELAEFLDTKRKLKIRSLIDKLVEKYVESAQK

>Cre13.g603176_4532.1|Creinhardtii_CC-4532_v6.1/1-894

MIAPKLLKPSPPRLHARSARVCVTAQMRKYELKPVAAAKSVAIIGAGLSGLVAAKSALEAGMLPTLFDRRGSIGGMWSTERSGMYDSLHTNVSRYSCVFSDFPWPEGAPDFPTPGELGAYLSAYRSTFLPPDKCPAQVGVEVLRVEPAGSPGGDGGGTGAKTGWRVQWRPLSPDADGSPSPSTCGHDTHDKQEQPPIQSAEFDAVIIASGYAAVPAVPDIPGLEAFAGKVVHSLHYKNPHEFAGLRVAVIGAGHSALDIASDLALASAACAPPSAAAEAEAAPAAAVGPAGVVHVFPRPMWVTPRYLPDSSAAAAAAASGTAAAAVAPPPAFLPLDMQFYRRSRRAGPEEVAVPNAAQRQKGNGFWASMCGDQGALISPLHAIAADTTEPPVIGICDSYVPLAATGAMHLRCARLLRITGPSTLELSDGGPPLSDIDALVLATGIKPDLSYLPHDVLEKLSYTPDDNYLPVAAHRGVTHPDVPGLACVGMYRGTYFGAVELQARLAAATLSGRAPPEPEEVVRAGVAAELAIREQTPRPVLPHGDYVGHTGGMAAALGVLPDAAWRAAHDQVVPGDFGLVPPSWEQQEQQAGTQAAMVKDAAAAAEAAMVAAVKEAPAGGEQGGGEPAGHVVVAGRPAAAYAAAAAAVAESHASLARFAGGRMVARAVFYALAGNWRLRRTLVSRMESSPSGVVTGAASFLPKRPDADAAANGSGSSNGSSGGSSSGGGSSRVRLGSMPFQYLYREQGQFAIQGSGSSSGSSSGSGGISSGGAVMRVSKEYVYRYDSPSDRLDVFFADAHGVPGGFFHSLRFLAPAEGAPGPQHFQREEEPAADDAADDSGCWRAVGEHLCVRDMYYSSYRFWFQGVHLKKFQIQFVVQGPNKDYTSTATYTRQ

>Cre13.g603176_4532.2|Creinhardtii_CC-4532_v6.1/1-595

MWVTPRYLPDSSAAAAAAASGTAAAAVAPPPAFLPLDMQFYRRSRRAGPEEVAVPNAAQRQKGNGFWASMCGDQGALISPLHAIAADTTEPPVIGICDSYVPLAATGAMHLRCARLLRITGPSTLELSDGGPPLSDIDALVLATGIKPDLSYLPHDVLEKLSYTPDDNYLPVAAHRGVTHPDVPGLACVGMYRGTYFGAVELQARLAAATLSGRAPPEPEEVVRAGVAAELAIREQTPRPVLPHGDYVGHTGGMAAALGVLPDAAWRAAHDQVVPGDFGLVPPSWEQQEQQAGTQAAMVKDAAAAAEAAMVAAVKEAPAGGEQGGGEPAGHVVVAGRPAAAYAAAAAAVAESHASLARFAGGRMVARAVFYALAGNWRLRRTLVSRMESSPSGVVTGAASFLPKRPDADAAANGSGSSNGSSGGSSSGGGSSRVRLGSMPFQYLYREQGQFAIQGSGSSSGSSSGSGGISSGGAVMRVSKEYVYRYDSPSDRLDVFFADAHGVPGGFFHSLRFLAPAEGAPGPQHFQREEEPAADDAADDSGCWRAVGEHLCVRDMYYSSYRFWFQGVHLKKFQIQFVVQGPNKDYTSTATYTRQ

>Cre16.g652750_4532.1|Creinhardtii_CC-4532_v6.1/1-1991

MQEFYADPGSLERPVLVVGAGVAGLQAARALLKRGLQVVVLEQSEDVGGVWIRNYAGYGLQVPWENYQFPEFPYPRKTELEQYPSGEQVREYVQSYARHFDLYRHILFSCKLISLRRKVPVPPAAGSGPGHGLRPGTGPGPVAEKRMSTEFHTSASAMSGANNDARRSGSASTNGFGPMAPRASAGSAATVAAGSGWGGHATSPPPHSTMGRRSFGTVFSSSAKRLFGQLVGGGGGAQGSGVHSSASAVTMPLQDGVAAGRPAVRGAGAGAIGGLNSRGGGSTTEQVARTFGGGAMQQPSPVTAGAAAAERMGGASNGGGAAPSGDGPDSAGARGPVSGSAGLSGSSSTSGEHADGPDGTSNTGAGSAQAKSSTAGATVNHSSKGGNLPTTRGAAPRVPPSGAKGAARGSSPSRSGSWADARRIRRQQQRVAAAERAAAAAADGAAARRAGLAGDGWLALYEDMTTGKQYMLEVSYVVLATGLYATPFIPSIPGQELYRGLQVHSKDFTDAAVAAGKEVLVVGAGKTAADIVTELTATSQASRVTLLYRRPHWPLPRAVGPVTIKSLAYTRLISSALLPYYYDAGPGGKAAAACLTPLRKAFWIRFMAKVNQTFGVKKTMGAPDRPICQDIWYSGQVLDAVKWPDVINNPKVQARQGEVEYFTPVSAVLRDGTELQPDIVLYATGYSNQYTFLEPDVRESINVQHDGVYLYRHVLVPGLPGLAFVGAEVSTFNNILTAALQAEWLASALAGEVELPLPHAMAADVEAQMEWRRRTMPPHKLRGSSVMLYMQAYHDQLVRDMGAATHRKQPSFRHPFAECFEPYTALDYSDVFMRDPLKVAASGAALAQAAAVPTAAAVAVGRATAAAALAANGTVDNGTDESNAANGTTVEAAAAAVAAAANATAGTATSPTVLGTTNNASCSAAASSGAYFTSLRASNSTGAQWAPCRTSTLASNGLGGFSNYTAVHSNSNGGPNSGAGPSPVRPGGAPGATATAAASNIYTNKTIAGLAAISARNGMGASNVRSPPWIYAPMAAGAIAAAAALQPATTQAAMPNAAARAAAGATMSGSRPPLVAPQRTASFTPASAFTSAATANAAAAAQTLPPNAAATAAAAGVLLGMSPRHGGHGTGTQQQHHNVPLDPLAPSEIDEDEPKARAVDEQDTLYSLDALPPALQAFRRTRVRSDAAARPSTGGGILDAMEEYADEYDEAAGDSQVYSDTPQQQQYRISGVVAPPPGGGAAAATGNGFAAPSGTLSRVTGSGPTPGTSQLRTSAAGSSYGPSSGGVLLQGAASSPVPTAAAAAVGAPAGSSPGSTYVILARHSGATADSAGGGGAVARVSGGGNSATAHAAAAGLVMGRVSTSSSGAASQSSTGAVSTATASSKASSHRPVQRGLSLATLLLRRRNAANAVYDVSSSRIEPTSAAVNELGYRVLPPRHVEEAMAAAAAAAAAGPAAAGKRQSGDGRAAAAVAAQAAARRTAGGGVVTSGAANVTLQRVTIGGATQSPAAVGTFTVPRQSSQEAANAAAIATPCRPVNAVAAPAPGDVTATPTTGAGPGRSTSRRRISADSVAPSTASGSASLSSAEGLLPQPPTQQVAAAAAADAGRIASAVNAAAAAAAAGRCQAGAAAMAAAPAAGAAAAGFAASEVSDYDGQSGLTRVPSISASSGISYMPSGSTAFIAPATHSAQQQQAQQQRQVPRQQQRQQPAVSYFNHITSHYGAADAAAAEAVAAAAAAADSASAVTGIEYQSDIVGAHAVHRSPPSISASSGISYMPSGSTAFVAPTQALAVHPQPRGTPAAAQSQRQGHAHVAHPQDPRREYLQNVVEHVQGRSVAAGARNTDSGGGGATESDAPGGGTRNCMSSGLSYVPAGSSFGPQPTLQLLPHYQVNIVDNTGSASTASAFGAYNTSGSTKLGYVSGSSGAGVLYANGNAVGGANHHGNGNGYTGGGVHMPVVHERRLSGASYASSGYSYMPSCSTVVPPQLAARS

>Cre17.g731700_4532.1|Creinhardtii_CC-4532_v6.1/1-1151

MGRKLALVIGAGVAGCQAARALLRAGYDVLVLEQSRGVGGVWRRNYHGFALQVPWDFFTFPEFPYELAPGDLAARAEAQDFPSGEIVQEYIEAYCKHFKLDRHIMFGVKVVHIAALPQFGNASAPTTTTAATAAAAAAGGGGLAAGASLHGGTANLPNPAGGPGTGPRWAVRFRQAVRPGQSPDVGELKTMECDVVVMATGMYFSPYVPFVQGMADYKGTVLHARDFNSLEPLKGKRVLVLGAGKSAHDCSVLAASEAAKAASVTTLFRQAHWPLPREIFGIPFHKIVYTRFTAAMLPAYYTAEGRLGKRVKHTLLSPLKRLFWGALERHVAGRMALRGHLRPERGFVQDLFFGGQIQDGSWNEAVNAGRIKAVRGSLAEVLPGGVRLADGTVLDCDVMLFATGYTKDYSLFDAATASRLAATPGSSTEEGLHLYRSIMSPRVPGLFFIGSEASTFNNVLTSGLQSLWLLHLLADRIRLPSPTAINKDILDQAKWRSRVMPAQRHSSSIIMLYQQRYHEQLLKDMGFPTRRKALAAKSKPTPGPGAPPGSNKPPQTGTSATDAAAAANGSKCSNGSSSASANRGSSSSSSKSGPVPRYGGSELLSPYSSHDYAELFDDRNLVGLSAAMVRIHGGAATAAGTRSAEEIVASAVTLMPAAAAIGTRLDLAVLPGAHGVAAVAARAGGGLVPLAQSDGCGGNGVPLMLAAGADAAAAAAALVGPSRLGPGPAAAAGQQQAAGGGAQLCVGNGLGNSASVSAFVGSFDSPTPTVSDVMPLGRAAQPPSALLSAAAVVAAARNARVAAAGLNIPIMGPGSRPMGGAFFAAGGMPWMQSALGAAAGGDGVAAAAAGPSQGQLPPLQEEGGLPASGGDLNGADAGASGIAASPAASPSAALPPPSQEEASDSLTSPANTNTGTAASGAAGPGGSTREMERSMGNNLLFVTTQSISGAGGPAAASGSPSPFATAATATLAAAAGSAGGASPAQPAAATSSAAAGPGRAGLSLPAASSHGGNATGGRVSLSRRVRLQVLHLMHRSIGGSLVPASAGDGEILELRVPSGTNTNTNNPLTSTNTNAMATTGTGTGAAAGATPADGHTGGTGGGGGAGAPPLGATSTATTATTAHVSSSLCFEAMRNAAPNVYGELVGTLEVH

>Cre06.g296550_4532.1|Creinhardtii_CC-4532_v6.1/1-1655

MPLSGASSFSAGRAGILPQPLGRLQKVLVIGAGVSGLQTARQLLKLGVQVLLLEANSEIGGVWCSNYVGYGLQVPWKLYEFAEFPWPDHPANDYPTGQEVHDYVLAYAQHFDLLRHVRLNSKLLRLRWDEESRTWGAVYMDTTTSKFYKLSADYVVVCTGIYSNPYIPAYPGAGDFVGTQLHAKDFTDYSVTRGRRVLIVGAGKTALDCMSGLVSANTAASVTMLYRKSHWPVPRSLLGVSIRRLMFNRTMANMLPPYYTAGKLERASAVVTKPLRRLFWKGLECLIARQFPAATKNQPAVPLPGDLFHGGQILDDRVDLQLEADGCRMLRGEVNRFVRNGVILQDNSFLPADVVLYCTGYEKTYDYFDGEMRSRLGLQKDGLYLYRNCLPPGVPQLAFVGSEVSTYNNILSSGLQALWLAHVLTGRVTLPPPHAMHEDVRAQQRWRREVMPPQRTRGAVVMLYMSQFHDQLMADMGHSPRRKGANVVAECFGVYSAADYAPVVEGCDGPLLAAMAEQCRAAEAFAAHGARSCAREQEQASQQRQQEQWQQQQREQEQAEAAAAATYASWQGGEGVQSPPSAASSRRHLLRQPSAAAAAAAARDAELLSPSRRGGGTRGNTPPRPVTTPAFGGPAVRPARASSARVRNVNSPAAGALYDPPHPSTVSGAATRGPVRSGGSHSAYAYPCLPSGHPNMHHHHPYAFTPSSSSGRGSFAHSRPPLLGLSPTSPYGHPHPDSASGRVSVGTSDMGTSGPSFADSASIRTRTSPWGASGGGGGGGSVLFGGGGGGGGAGTLPLGAGGDGGSSGYGSATSSCAQSCGGVPPGAAAAAALMSLPEAPRQQQSPQPVPLAAAVTPGQTWSGAGGAAAPRRQQSHNAQQAYLQHQHQQHAQCAEVRVSGGGAGDSLPTSPPEAWRAAPRLAPPPRAAAGAPDPLRPPLPGGVLCSQSASRGQAHGVAMRSCDDVMLARIAAAGASTGSSSLGAGGAGPFGKVPTSAGTATAAGVRSGPAPPSTASSAGAAMFPAVVAFAAAAAAAATASAMAAAAGAPAPWMQQLQQQQPQQQHPNQRHEPAAWPSALPEAAAHEPYRAAGSAGSEAALASIPEASHAAGLLSRAGHGCAGSAPAAAPASAAAFAAPASAAPYLAQAQCSPLLPEERRRAAATAAGGAATGGSAGAVDSASGSAPAPVAAQGPAEAQAQQSGAYAAGVVEGDGDTEGSAADTAAGGPAAGGTIAPATPQQAAATQRLPGPDDSAVHQQAQRQHHQSQMRQHQDLQQQQQQQQQQQALQQQQQQQQQQQQQPGSPHAAAAATPTAAGAGDGVEPSTASAPAPSTAASSHSEAPSPHSFEPPSAAPARLPRGPASASAPQTSHYSPADTSLLMSSLPPLAAPPPGSSARGRDRRGSRHALESMPGPDVATAASLSMLPLPPVLEREQPPQLQQQPRSRRGTPRRRRSTLAALPADLAAAAPPASAAPPMPPFTVSVGRVPLRPFGAMGPFPTANNNGNGSGVSSAGSSSMLYDRRRLSSSSLGGLMVLGPGPAATNSAGTPSYSPKRLAPAGWHGGGTGGSSVALPPSQHRWRRSAGRFSLDWGCLPPSTAAAAAAYLAALASGSAGGVSRAATVAGGALEVTGKAAGAGDATAAAEQAGQAPGAS

>Cre06.g296050_4532.1|Creinhardtii_CC-4532_v6.1/1-1246

MASPEASTPLLPEAGRLRRVLVVGAGIAGLQVARRFAQMGVECCVLEATDTIGGVWAANYAGFGLQVPWRLYQFADFPWPQSLQPPDEFPTGAQVQAYILAYAQHFGLLRLVRFNSRLLRLRWHSDQRQWEALYSNTETEKFYKVTADYVVMCNGIYSKPYIPEYQGAESFVGTQIHAKYFTDVLTVKGRRVVVVGAGKTALDCVSGLVSANTAASVTMLYRKSHWPVPRSLLGISIRRLVFNRAFAGMLPNYYTAGKLERARAAVTKPMRRLFWKSLECLISTKYPATTGRLKPAVSLPKDLFYGGQILDDRLDQMLEGKGVDLLQGEIKAFVRNGVILLDNSFLPADVVLYCTGYEKTYDYFDGEMRARLGLQKDGLYLYRNCIPPDVPHLAFVGSEVSTYNNILSGGLQALWLAHVLTGRVTLPPPHAMHEDVRAQQRWRRDVMPTQRCRGSVLMVYMGYYHDQLMADMGHSPRRKGANVVAECFGVYSAADYKQVMEASDRRLVQTLAEQWSSVAEQARLHGPPLTASVAASAAGTEPARRHTISNGMEAREAARPRALLRAATCSERPSAASTQQHASSLGRRTTPSPAAEAAWRSTTGGFPRQQRNTLYTRRSTGTPISDGISASGVAAGILMEGGGEGSLASTAVGGAGPAEARRTSRKSVMVPHPLPESSEVLYWQVPPGGAAEHAQQQQGGASGQQAFVEDLPALQNQQHPYPQHLLQHQFSQPHPLSHMHPQQQHPRHTVSGTQGGASAYSSFGGGATDGGLLHRQPPVARMYRHSSAASAHSWASRGGGGGGGATASPPMTAAMERGLVSMTADSSAMLSCSHAGPDGASVGWADAAGGSMHGGAGHIGTGSGMLPQGSGPPPGGCGDGEVAEACGIRVVPFAQASGAASVSGAQSMTGPAGGGGRIAPLLAAAVGNTEGGAAALAQQEACGDDAAFQPAVTVATLPSASSAMLSSPGTSSSGLGSSVVLQLARALASLQQLPMQQRQLLQRQQTTQELMAVPTGIYTSPLSIDAEQQQQHQHQQELSDASSAADIAAALTSMAAAAAAAAAAAAIAATTAMHRQQSQSSGPVCSTCGSPGAPASHSRQACNEPHHTQLIRHGSDLAATLVAAAIAEALRQSGTCGAAMVPQLQLPASAPVPLPLQESVQRREPPLPVSAPSSSVQAGSRAGGGPHGLASMSAGGMTTESELLPGASATAVAVEQGGKLLLHRECAQAEEEACWGGCASVAAARS

>Cre03.g148900_4532.1|Creinhardtii_CC-4532_v6.1/1-1863

MEFSGLIPSPDRLRKVLIVGAGVSGLQCARQFLKLGCNVLVLELNEDLGGVWLRNYSGFGLQVPWKLYQFPEFPWPKEMQPRQEYPTGEEVQEYARAYATHFDLRPHIRFNCKLLRLRWCPNIRSWEALYCDTSVEKFYKVVVDYAIICAGIYSQPYIPDYEGTDSYAGIQLHAKDFTDLSLARGRRVVIVGAGKTALDCVGSIVATSTAASVTLLYRQAHWPLPRRMLGTSVRRLMFNRAMTGMMAPYYTASAGKQLAARASAPLKKLFWRSVESLIGSKFRITEHLRPRVHLPEDLFYGGQILDNTMDKLIRAEALNTVKGEINRFVRNGVILQDGSFVAADLVLYCTGYLKTYDYLDGDMRARLDLQKDGLYLYRNCLPYAVPHLAFIGSEVSTYNNILTQGLQALWLAHVLTGRLQLPSPADMAEDVRAQQRWRRTIMPAQRSRGSVLMLYMMQYHDQLLEDMGARPRRKGLNVFAECFGTYSAHDYKDLLEADPTVSAAMEAEQRLMGPGMQPPFRGSHGNRQDHRHNDGGDYDGDGHGSGAYGHAANGHGSGAGPAPGRHGQPATPRGNAGSGSYAGTYSSMGQPGSSGNGYGDTYIGGNAAAVAAASAASASAAGSGNGAPPASAHGTMLAVYAQRAQQDGYEYHRALMGERVGSGANSGSGGVAGLRTMSMSVSRGAGSMTANKRPASGGMGLAGRQLSMSKGLLAPLAALMTGSPHNSFRRSIDNPHGRVPSAVALGGSGAPSASGSFGSRHPSTRPHTVEGFYGLATAPTAVAAGLAGARWGPGESPRPRHAALHSAVTAHEAAGLTAGGRLHLHASTTHGESAHSQGTPHADSHLSPRAVTVSGKSASNSEMFLIVQQQLRQYNQQHHPQQPQDRSRSRPIPASAHELHHAPPPAVAHSAPGRSRHTNGRALPSPQGQHAYRQHSQGQFSSMAVLAECSQLPSLEEVERRNRSAGSVPDSGALCGSQLVPLLGATDSGCAPVTGLHTNTTAPMYIGGAPMGSSPSNCAVSGGAAAFSSAGFGGGSASRLSLASSTSRNAQSPHASLGHPAPQSPVHSYSRLSRNTLATSPLLDVAGGHDGHGSGSGMHRPSQCVTEGGHLNSHSASLPYARSSSVGGMRTSQPLPSCEGMGSGAVDELMAEGDFVVVGPMEDDVDMQDELQVGEAPSKQQFSGTVIAVAGGPGAAPGVAGVAGSTRRTAGRLPPPSPSSGFSRFSRLGEGVPSRHTLHTGRGSTGGMAPYDSSNSAAAATGLGNSASNSRSSLHSPHQQHLQQQLPVQQTYANHAAIASHFDAGRVAGLAGQGSATPEHEHPYPPDSPFRSGTPISRRTTEADGGGDLSSGTMPSSCKNAVSACGFDSGTAHATAGPAAAASDMYESGASSTGTPVATMDRTLLQQAGPRAGAMAGLQQQPTLRQPPGGQVAMFDSALSTQACASEPSNRLSISSRTSFDSRPNSLDVTLPPSAASIPTGPGDGSGSKAAATAGRTRLAGTSDLVLRTGGDGAEQRQWQDQDGEPQLPVPAQPLVDELAGGAPVTPQQAQAAVVDIGGVVGAGGFFEGSAPTSSGYGEPTMAMSGMMMRAHSIPSTVSNGASSTTPTGSGMIPFGSAFPSESAALPMTGSSSVAGQRNGSDSGAPGARALLPPGSRASSGAALNTPFAQAGPPSAGRSSKRSGVLARHVSGGLGYGGGGALALAVVPPLVGGAGMVSASPSGGSGASSACVLASRSSGGVRLQPGSGSPSRLHGMPMLASAPREGMPMLASAPRFPTAQEAVAGAGEVAVTSCGADNNSNVLQLSNLTLGGWDVDVPAGAGGSSLMGLSGGICHAAGIMSLSRVESLPSRDEAHLGAEVLGQ

>Cre06.g298550_4532.1|Creinhardtii_CC-4532_v6.1/1-1604

MSAGYQMGQTPPYGLGHSPVSAPCSPAIQRVLVVGAGVAGLQTARQLLKIGLEVMVLEAHNDIAGVWSQNHYGCKLQTPWRQYQFPEFLWPLELQPEDEYPSAEQVRDYVRAYAWHFDLLPHIRLNCKLLRLRWTGSNHSWECLYADTAVEKFFKTRVDYVVVCTGIFSNPFIPSYPGASDFVGQQIHAKHFRDVSVASGRRVLIVGAGKTGTDCACNLLASRAAASVTMLYRQAHWPIPKRVLGLSTRTLFFSRMGAATVPPYYTAGRLRTAVAAATKPLRALFWKGMEVAISSRFHAARATPPSVSLPGDLFYGGQVLDDSLERLAKAGSLEAIRGEVNRFVRNGVILQDGSFHAADLVLYCTGYAKTYDYFDGPMRARLGLQKDGLYLYRNCIPPGVPQLAFVGSEVSTYSNIVTQGLQALWLAHVLAGRVEPPPAAAMEQDVREQQVWKREVMPVQRNRGSVLMTYQTHYHDQLLQDMQLHSRRKGANFLSECFGAYTAADYSPLLSKRDAELVTGLEQAAAACKAAQAAAVAGAAVARAQQQRQRQQQGPRTAAMAVGRSLYPAHLRAPAVAACSAAAREGTPARRLSGDLSNCDADDEESVRSVVAAALAATAAAMESGRHGGYGRSYRRASDTSGDGTCGGAGVGNWTTYPTAGMSFTSEAAFQVAKAGIVNKHTGVDSSDSVTAAAVEAVVAAALAATAAPSLVPTTRRSSDAMAAEQEMHTAWPYASGGDKWQEASSRCEEGASGRGGYVPVGRTVTPHGPSVLDGAAVSAAQQHPPQHSGASAMTEAAADCGSLSDGASSAPATKSQSMSLLRKLLRLRSSLSRSESATSVAGSNTPTTQQPGQTTQQEHSQQGASKLRIRIPSGCTSGDVLEGHGHAGHRSLESALRSGLSSEAAAAAAAAAMGAITLVIGSSASQSARMWEASSSPRAASAAATATGFLSAPSSKRGMSRSVVETRSMRGSVAAVAAAAAAALAAANTVANIQAAGYERGGGGGGGGSGGGSGVASARSSTQGIDTPGGSVHGANSYRSHRLCAQSSELVAAATATAFRRLSRRASVTTTAADEGSGAATEEPQSSLRFFAQLRPAASAAAACQPSQGDEPFVQCSINDCGVLHGTASGAAASASVRRGSHGLEDVHRCSEQLASGSYSLGPGLSVERNASADESAWDGLMAAETAGTPAAEPVPIPGLGHAASDTTCNHFDCSWRQHGASCGGASSSSSLRRAFTADVAASAVPPGGAGTGQQPHSSTAVSPPLPILGGGRGSNLSATTTGFWQGSPSTPPLSRSRSHVPRAAAAGYSSAGAAAGPGPAGIGAFAALPDGETPCSGASIGSAGLLSAFTGDSGTLSSAAGSITLNGGMCRLPPGGPPRQQQQQQQHHHATVPLSPPLPTLHHAHQLHHCSNGPLHHHHQHLQQQQQQVNMQYRQQQQQQHQQQHKHATARLAESLIGLGGASGAGAAGSGASPPPSLLLMLAHRRRHSALDPMPMSMGLETLSEEDVSVTTTATPSFCGGVVVASESRARSTGGVTGCEDDDSPRPLSRQESSGCSLTRGSSDCLKVGVAAVHAARLVEKQGAQSQLAASSAWLHTELSST

>Cre17.g802145_4532.1|Creinhardtii_CC-4532_v6.1/1-241

MVTANVLPAHPTTVVVGAGLSGLQTAAQFLALGHRVCVLERAEDVGGVWLRYTSFNIQVPRDFFSLPDFPCPPELEPEEYPTGRSLLRYIIAYAHKNQLYRHIIFRAHVTRLHRLAGAWQCFYDVEEPLPGGGGGGGGADGLGGGLGRSSMASAAGSPRAGGSGGGATGGEGAGAGGGGGGGGSMVVQHRMTADFVVVATGLHSTPAVPPIECPHLFRGQMLHAYDLPPDEELPEQVGGGC

>Aqcoe3G194100|Acoerulea_v3.1/1-324

MNLIFFPLSKPSLLFPPHQLHKHNHHYFKFKPHCLQTTISSPISNTPPETKTGVIIVGAGLTGLAAATHLNSKNIPFLLVEASDSVGGRVRTDEVDGFLLDRGFQIFITGYPEAKNVLDYESLDLQKHFTDAILSLTNPIGSVIDKLLIGLTRIRVLSKSDEEILSANEISIIDLLRNTGFSGLIIDRFFRLFLGGIFFDEDLETSSRLFDFIFKCLALGDNTLPAKVASIEEFESKSNLSSVKLQNGEILKSEFGVIIAVDEKVLPSPDPYITQPGRPKALRIKGDDEDQPSSNHRCKLCGEANHNYTTNVQRKKLVKRNPRR

>Sphfalx15G093800|Sfallax_v1.1/1-564

MDCMLRMLPMSSNSNVVALGWSTLPKIFVARKSLGVSCSDLQCGFGYGGSRLNSSSLAFANFLSSRTSRFAAAANSRASSSLQQNQAMLDQQESTSLLSTDRRRREEEVDEVVAETGVVIIGAGLSGLAAARHCAKEGVPFVLLESSDGVGGRVRTDVCQGFLLDRGFQIFISAYPEASEVLDYAALDLKEFYAGALVWLDGEFHRVADPFRHFTDGVASLGNPIGTAMDKVLVGIVRLRASLKPISAILLSSEETTILERLGKEGFSQQMVDRFFRPFFGGIFFDRELQTTSRLFEFVFKCLALGSNTLPAAGIGAIPQQLADQLPAGSILLNSRVAELIEDEDGILRSAALQNGKVFRAKYGMLIATEGPEAARLLGSRITDTSASSPSSSSAAAASSSSSSSITKPPRSTVCLYFAADSTPINEPVLLLNGSKEGIVNNMFFPSTVAPSYAPPGKTLVSISLVGQLYSNYSDPELEQQVRKELHSWFGAAVVDSWQHLRTYRIANAQPDQTPPTNLQKQPRVDQGVYVCGDHRDSSTFDGALVSGRRAVQALLADCRVIAA

>Tle4c04726.1|Takakia_lepidozioides/1-465

MAEQQQQVQERGSLSTQEGSNLGTEATERVEGKPTETGVVIVGGGLAGLAAARHLAEKEVPFLLVEASDGVGGRVRTDLYEGFLLDRGFQIFISAYPEASQILDYQALDLRSFYAGALVWFNGGFHRVADPFRHLTDGVASLGNPIGTALDKVLVGIVRLRAALKPVNAILSSDETTILERLGKEGFTQQMVNRFFRPFFGGIFFDRDLQTTSRLFEFVFKCLALGSNTLPANGIGAIPQQIASVLPKESILLNSKAAKLVEDENGMVSAVALEAGREIRAKYGVVIAAEGPEAARLLGERLPDTPSIRKPPRSTVCLYFSAEKSPINEPLLLLNGSNEGIVNNMFFPTTVSSSYAPAGKTLVSVSLVGQYADSSDAHLEQAVRTELHSWFGVGVVDAWQHLRTYRIALAQPDQTPPTNLQKDPRVGQGVYVCGDHQDSSTFDGALVSGRRAAQALLADCKLVRV

>Lc_composite_27985.1_1|Lunularia_cruciata/1-521

MPLLAAAAVGPCCSTFVGAATRISSICSRLNSVTSEFFTCNGRNLLPDSFGQKRCDMVSVKFHREVAKPLAKVSEGRPSAATVSNLGYPEETGVIVIGAGLAGLAAAKHLAQEGVPFTLLEASDGVGGRVRTDVLDGFLLDRGFQIHITAYPEAQRILNYSELDLRNFYAGAMVWFNGGFHRVADPFRHFTDGLGSLTNPIGSVLDKVLVGIVRFQAAVRTVDDILTTEETTIEERLSKEGFSTAMVDRFFRPFFGGIFFDRELQTTSRLFEFVFKCLALGSNTLPAAGIGSIPEQLASSLPSESLFLNSKVTGLVEGSSGVVSGVKLENGSQIMVKYGVIVAVEGPEAVRLLQSKLPEAPSVTKTPRSTICLYFSTENAPFTSEPVLLLNGSDQGIVNNMFFPSNVVPSYAPPGKTLVSVSLIGTHEGTSDADLEVTVRKELSEWFGKSFIDSWEHLRTYRIPFAQPNQSPPTDLLKKPRLSDGLYICGDHRNSSTFDGALVSGRVAVQALLAEKKVANI

>Rn_16247.1_1|Ricciocarpos_natans/1-878

MALLMNVQLNPVTKCDHKKQVDISRRRGRAIFFTSRSYSSGSSALFDKALREAGLCSSAESQVASSLLLGTTRCWFGGLFRGRKTDNVEQDDFPPRSNFLYHDSVPPFPKSLLAETFLAGKELRCCYKASKDGFSALQFHERCDFKGPCVIMGWTDKGLRFGAFNPEGYRSTDDYYDTFDAFLFYWPTDEREPTTPIVLRKVGGSGAALFDYARGGPQFGADGLLIGAPLAPVMGGFAGPDTNFGVGQLSQARSRLGLSYAVRPDGKASLFGDDKRASLVEIEVFCSPGIADLVDLQVIKILNCEAIRIHLQMSGTLTGNRKDEHSEGTSANNRQIGAVCSNRAIVCPKVEMVHLAVAVAGSGCGPLTTVSTRSFRESGSRYSRRSFTGNELFFSYGKDLANITLVHGKNLNVPSSRHRLEVVLGPRAKVSEGEVSVSVNNLQHPENTGVIVVGAGLAGLAAAKHLTREGVPFILLEASDGVGGRVRTDKFEGYLLDRGFQIHITAYPEAQRILDYDKLDLRNFYAGAMVWFNGGFHRVADPFRHFTDGLGSLTNPIGSVLDKVLVGIVRFRAALRAVEDILASEETTISERLTKDGFSQAMVDRFFRPFFGGIFFDRELQTTSRLFDFVFKCLALGSNTLPAKGIGAIPQQLASSLPPQTLFLNTKVTELVEEGDVVSGVKLGNGQEIKATYGVIVAVEGPEAHRLLKDKFTPTPSVTKPPRSTVCLYFSSDTAPFTNEPILLLNGSNKGIVNNMFFPSNVVRSYAPPGKTLVSVSLIGTHDEKSDSDLEGIVRDELSGWFGRSFVDSWKHLRTYRILFAQPNQIPPTELLKNPKVYDGLYVCGDHRSSSTFDGALVSGRVAVEALLAELKVTRPGN

>Rn_16247.1_2|Ricciocarpos_natans/1-878

MALLMNVQLNPVTKCDHKKQVDISRRRGRAIFFTSRSYSSGSSALFDKALREAGLCSSAESQVASSLLLGTTRCWFGGLFRGRKTDNVEQDDFPPRSNFLYHDSVPPFPKSLLAETFLAGKELRCCYKASKDGFSALQFHERCDFKGPCVIMGWTDKGLRFGAFNPEGYRSTDDYYDTFDAFLFYWPTDEREPTTPIVLRKVGGSGAALFDYARGGPQFGADGLLIGAPLAPVMGGFAGPDTNFGVGQLSQARSRLGLSYAVRPDGKASLFGDDKRASLVEIEVFCSPGIADLVDLQVIKILNCEAIRIHLQMSGTLTGNRKDEHSEGTSANNRQIGAVCSNRAIVCPKVEMVHLAVAVAGSGCGPLTTVSTRSFRESGSRYSRRSFTGNELFFSYGKDLANITLVHGKNLNVPSSRHRLEVVLGPRAKVSEGEVSVSVNNLQHPENTGVIVVGAGLAGLAAAKHLTREGVPFILLEASDGVGGRVRTDKFEGYLLDRGFQIHITAYPEAQRILDYDKLDLRNFYAGAMVWFNGGFHRVADPFRHFTDGLGSLTNPIGSVLDKVLVGIVRFRAALRAVEDILASEETTISERLTKDGFSQAMVDRFFRPFFGGIFFDRELQTTSRLFDFVFKCLALGSNTLPAKGIGAIPQQLASSLPPQTLFLNTKVTELVEEGDVVSGVKLGNGQEIKATYGVIVAVEGPEAHRLLKDKFTPTPSVTKPPRSTVCLYFSSDTAPFTNEPILLLNGSNKGIVNNMFFPSNVVRSYAPPGKTLVSVSLIGTHDEKSDSDLEGIVRDELSGWFGRSFVDSWKHLRTYRILFAQPNQIPPTELLKNPKVYDGLYVCGDHRSSSTFDGALVSGRVAVEALLAELKVTRPGN

>Rn_16247.1_3|Ricciocarpos_natans/1-878

MALLMNVQLNPVTKCDHKKQVDISRRRGRAIFFTSRSYSSGSSALFDKALREAGLCSSAESQVASSLLLGTTRCWFGGLFRGRKTDNVEQDDFPPRSNFLYHDSVPPFPKSLLAETFLAGKELRCCYKASKDGFSALQFHERCDFKGPCVIMGWTDKGLRFGAFNPEGYRSTDDYYDTFDAFLFYWPTDEREPTTPIVLRKVGGSGAALFDYARGGPQFGADGLLIGAPLAPVMGGFAGPDTNFGVGQLSQARSRLGLSYAVRPDGKASLFGDDKRASLVEIEVFCSPGIADLVDLQVIKILNCEAIRIHLQMSGTLTGNRKDEHSEGTSANNRQIGAVCSNRAIVCPKVEMVHLAVAVAGSGCGPLTTVSTRSFRESGSRYSRRSFTGNELFFSYGKDLANITLVHGKNLNVPSSRHRLEVVLGPRAKVSEGEVSVSVNNLQHPENTGVIVVGAGLAGLAAAKHLTREGVPFILLEASDGVGGRVRTDKFEGYLLDRGFQIHITAYPEAQRILDYDKLDLRNFYAGAMVWFNGGFHRVADPFRHFTDGLGSLTNPIGSVLDKVLVGIVRFRAALRAVEDILASEETTISERLTKDGFSQAMVDRFFRPFFGGIFFDRELQTTSRLFDFVFKCLALGSNTLPAKGIGAIPQQLASSLPPQTLFLNTKVTELVEEGDVVSGVKLGNGQEIKATYGVIVAVEGPEAHRLLKDKFTPTPSVTKPPRSTVCLYFSSDTAPFTNEPILLLNGSNKGIVNNMFFPSNVVRSYAPPGKTLVSVSLIGTHDEKSDSDLEGIVRDELSGWFGRSFVDSWKHLRTYRILFAQPNQIPPTELLKNPKVYDGLYVCGDHRSSSTFDGALVSGRVAVEALLAELKVTRPGN

>EVM0018363.1|Lycopodium_clavatum/1-561

MAYMQPAAAAIGAASSSSFFSFSFSTCPASSLLLLPHQSALISPSLHAAAASCHAYSTPKFLLPARKASSDGIFFRSCADSTNLPYCLVAAHKQCRPVLCRAAGSWEIDAPQQAASIVGTNAAAEGKEAAWETGVVVVGAGLAGLAAARRLQRQGVPFLVLEASDGAGGRVRTDIHHGFVLDRGFQIFISAYPEAQQVLQYDKLDLQSFYAGALVWYGGRFHRVADPFRHPVDGLLSLINPIGSIYDKILVGIVRLRAAIKPMDDILSADEVEIAEKLKREGFSSSMIDRFFRPFFGGIFFDTELRTTSRLFEFVFKCLALGSNTLPAEGIAAIPRQLADNLPADSLQFNSKVKQLLTEKEHGFPGVRLDSGRVVRGKYGVIVAVEAPEAVKLLGKRISEADSVSKPARSTVCLYFSAEKAPINDSILLLNGSGKGIVNNMFFPSNVSPSYAPQGKALVSVSLVGSHSEAKDEELEKTVRLELSQWFGAPEVAKWQHIRTYRIPFAQPNQTPSTNLRKEPRVGNKIYMCGDHQDSATFDGALVSGRRAAEALLLDSKLVTV

>Klenit1_1_11158_kfl00481_0070_v1.1|Klebsormidium_nitens/1-574

MAIAQLQQAPCARGQLVARHQTDHQTALKSGPRQCHSGQPHCHQNLGGQQQRGPCSARKALRHTHLSSVQFAAEHLNPQSGFPSWRHCRHVCAAFKWPWEQKDGNAASQEVDAVEAREQENRRNGVPAEETDVVVVGAGLAGLACARTLHAAGIPFRLLEASDGVGGRVRTDVTDDGFLLDRGFQIFLSAYPECKKVLDYEALDLRTFYSGAFVRFRGQFHRVADPFRHPLDGVASLSPTHPIGNVLDKLRVGLLRLKVLIKSDADLLSAPETSIVDKLRREGFSSAMIDRFFRPFLGGIFFDRDLTTTSRLLDFVIKVLALGDNTLPVQGIAAIPEQLASGLPSESVVLNAPVSSVVPARAGAPATVRVAGGRSYAARRGVVVAVDGPQAQRLLGGGLSSTPSDARDARGTVCLYYAAPSAPIEDPVLLLNGDGVGLINNMCFPSSVSPSYAPPGKALVSISLVGAYPDRSDADLQTAVEAELRDWFGKETATWRHLKTYRIPFAQPNQRPPSDPLREVRLGADLYVCGDHRDTATFDGAFVSGRRAAEALMADSQEKQGASTKAEAKVVVQV

>Potri.001G344800|Ptrichocarpa_v4.1/1-478

MTTLALAHSLPCSSSKKHLPFRFIAHASTQLSPASLKQQLSHQKTGVIVIGAGLAGLATATRLNSQNIPFLLLEASDAVGGRVRTDVVDGFLLDRGFQIFITAYPEAQRLLDYQALELQKFYSGARIYFDGRFHTVADPLCHFFDSVKSLTNPIGSVIDKLLIALTRIQVLSKSDDEIMRADEVPTIDLLKRIGFSDSIVERFFQPFFGGIFFDKELGTTSRLFDFIFKCLALGNNTLPAKGIGAIPNQLASKLPADSILLNTRVVSVGLDEDQNTPMPFVRLENGDILQSELGVIVAVEEPHVNELLAEINSIGPVQSKKPARSTVCLYFSADRDDIPVRDPVLFLNGSGEGIVNNMFFATNVAPSYGPPGKALVSVTLIGLFDDTSDHDLVDEVISELSGWFGGSMVGSWKPLRTYRVRYAQPNQCPPTNLMKNPQIGSGLYLCGDHLTSATFDGALASGRKAAEALLRDRAFIKV

>Rhdel05G0135100.1|Rhododendron_delavayi/1-485

MSLTLSLPFSNPSLSSLSSPTSHHHRRRLILKPPKSSSSQISIPTPPPDPFPPHLQKPGVIIIGAGLSGLAAATRLASQNIPFLLLDASDAVGGRVRTDTVDGFLLDRGFQIFITSYPEAQMLLDYQTLDLKKFYSGARIFYGGRFHTVSDPLRHFFDSLRTLTNPIGSVLDKLLIALTRIRVLTLSDEEIFSSDEVSTAELLSRIGFSDLAVDRFFRPFFGGIFFDTELKTTSRLFDFVFKCLALGNNTLPAKGISAIPEQLAAKLPTESIRLNSRAVSVDIESGWAIVGLESGERLRSEMGVIVAVEEPEAVKLLAGKWNRKPVQKPARSTVCLYFSAEPSQVPDRDPILFLNGSGKGIVNNMFFATNVAPSYGPPGKALVSVSLIGLFEDVGDEDLTAEVVQELSGWFGESMVRSWKHLRTYRIGFAQPNQSPPTDLMKNPRVRPGLYLCGDYQTSATFDGALVSGRKAVEALLRDRALIQV

>Chabra1_352321_rna-CBR_g49724|Chara_Braunii/1-651

MAGAASPSMLQRYPAVANAGSRAATQGTCCSEARGGAGDTWHSSATRRLSYASVSVAALPRQKSAAAGADHSAAKGRINRYGGGGAESASRTPNAIAPRRLPNRWGVGAVASEARFLRFSEGRFWRGTPAFATAMRISTWAKSKKTRLSGVAQASAGSAASAAEAPASGTQSAGVGGKEESSTSSSDILPPASAAADVVIVGGGLAGLCCALHLYTRGVDVVVLEASDDVGGRVRTDQLDGFLLDRGFQIFLTAYPEAQAVLEYDKLRLRPFYAGALVRFGGGFHRVADPFRHPLDALRTLAPDHPVGSLADKLRVGLLRLRSASKSDESILLSRTETTSADFFNQGFSFETDRFFKPFLGGIFFDRHLSTSSRLLEFIFKILLGDNALPAAGFGAIPKQIADRLPRGAIRLNARVLQIREEGAQSSDPTGSVAKDQDDGTTGQDQGGGGRMAGVKEKEVEVEGGAMVRARKGVVVIATEGPEARRLGESLVTSAGLKPRGTVCLYFALDHPPPSPGDEPIFLNGTSKGVINNMCFSRVAPEYAPSGKELVSVSLVGTYPDRTDVDLETEVRAELGDWFGVSEVATWRHLRTYRIPYAQPDQRPPWEAQRAVRLGDGLYVCGDHRNSASFHGAMLSGRQAAEAILQDLRIA

>Sphfalx02G048800|Sfallax_v1.1/1-665

MEPMIGRKMVAIIGAGPAGLVAAKSMLEVGLFPTLFDKLPDLGGVWNPQSSSCWFSLRTNDSRYSCCFSDFPWDSDSPIFPSQHDVYGYLQAYANKFLQPVHFCLGSEVTNVSRSNNDKWLVQWSSLEQKYQGSKAGSQIFDFVVFASGFFSIPYTPSIQGLDGFPGEVMHSSEYRSPERFRGKRVVILGASFSSAEVAADLAPLATEVINIVSRPFWVLPRYIALDPDSASTPFFPLDLVLYRRSTRQSQGEEVFANEEENRKANQYFRKLCGDQGPLNLQQDTDPPIIAISDSYLELVRSNHIMVRHGKVQEVQGQTVILEDQTTIPEVDCIIFCTGFQSSMPYLSSNVLDTLSFDPADRFVPFLLHRSTFHPSLPGSAFVGMYRGTFFGVMELQARWAAAIFSGDLPPIPESVQQEGIAFEKWIKSQTPRPQFPHGDYVGFADTFAADLNVLPSKDWYQKHDISIPAHFRRGSVAEEAVEVLEKACKEFLDGKMVPHAVFRSLAGSWTLHRHIENRIDFANSGEVKGTATFNVTELSHEYLYTEVGELSMQRGQHFRVHRKYVYVYDTHTNQIDVFFENQGSRGSPFHSLQFLSPKPSSATEKGGIEMSGGWRAAGQHLCGEDLYNASYRFSFSGNSIAQMEISYSVTGPKKDYTSTATYFR

>Sphfalx08G012900|Sfallax_v1.1/1-808

MTTMMGTGLSSFAQQQISSSSASVVGGRSLPLSSSSKLVQTRRRRQERSLSIFFFFCLRNVPSPPSSSKCESRLLGILKQKVRTAAIAAAVFVANPTCNQLGGEDIETMETTPTTAAAAATGDDSRRRGKTVAVIGAGPSGLVTAKCMLEVGLSPTIFDKAPELGGVWNPRVNPFWSSMRTNVSRYACCFSDFPWDSKAATYPTQHEMNDYLHAYAHKFLQPVHFSLSSEVTSVARSGGSDSDNGKWVVQWRSSSSSSSSLEEPKAIEGSPPAAALLSSSQEQMFDFVVFASGFFSIPHIPSIPGLDGFLGEVVHSSNYRSPERFRGKRVVVVGGAASSAEIAADIAVFASEVINTVSRPFWVVPRFIALNPDSDATPFFPLDLVFFRRSTRKSQSEDIFRSEEEVQKANEYFMKLCGDQGILNLEHDTDPPIVAISDTYMEFVRSNHITVRRSKVQEVQGRTLILEDHTTIADVDCIIFCTGFLCSMPYLSSELLDSISFDPANQFMPLLLHRSTFHPSLPISAFVGMYRAPFFGVIELQARWAAAIFSGVLPPVPESVQQEGIALEKQIRIQIPKPQFPHWDYVGLADGFAADLNVLPSKEWCQKHDIVIPAHYHTGDVAANEAVEILEKACEEFLNGKMVPYSVFRSLAGSWILHRCIENRMDSSQSGTVEGTATFAATDVSHQYLYSEIGELSMQRGHHFRVHRKYVYVYDKDTEQINVFFENEGTRGSFFHSLHFLSPKCSEKGELNEVSGGWKAEGEHLCSEDLYNVFYRFCFRGNQIEKMEISYHVKGPKKDYTSTATYSR

>178716|Smoellendorffii_v1.0/1-611

MATMNGSPKIDPDALVDAWLARLDSSLASGDASAAATLFEESTSFWRDLIAFTWNIVTVEGRSQIHAMLQSTLESVAPKGWVRNGTASYSADSGVIEAWIKFETRDAVCTGHLRLMATTSLCRTLLTAMEGLKDFPEKKGHTRPKGVTHGVIRHRASWLDGRKEEERTLGSTVQPYCVIVGGGQAGIGLAARLRQLGVPCIVVEKNPRPGDSWRSRYKSLCLHDPVWYDHLPYLPFPENWPIFTPKDKMGDWLEAYTKIMEINYWTSSECLGARFDPQSGEWEVKILRDRSKEVTLRPKQLILATGMSGFPNVPRIPGQEEFVGDLHHSSKHPGGKAYKGKRAVILGSNNSAHDIAADLWENGAAEVTMIQRSSTHVVRSESLLQFVMKNTYSESAVESGLTTDKADMIFASVPYKIMGDVNRPTYDAIRAHDKDFYAALTKTGFMLDFGDDDSGLFMKYLRRGSGYYIDVGASQLLIDGEIKLRSGVTIASLKPSSVVLSDGTEIAADVVILATGYGSMNQWAAKLISQEVADRVGKCWGLGSNTRKDPGPWEGELRNMWKPTAQEGLWFHGGNLHQARHYSLYLALQLKARMEGITTPVYGIPKVRHLS

>422043|Smoellendorffii_v1.0/1-611

MATMNGSPKIDPDALVDAWLTRLDSSLASGDPSAAANLFEESTSFWRDLIAFTWNIVTVEGRSQIHSMLESTLESVAPKGWTRHGTANYSADSGVIEAWIKFETRVAVCTGHLRLVATTSLCRTLLTAMEGLRDFPEKKGRTRPNGVTHGVIRHRASWLDGRKEDERTLGSTVQPYCVIVGGGQAGIGLAARLRQLGVPCIVVEKNPRPGDSWRNRYKSLCLHDPVWYDHLPYLPFPENWPIFAPKDKMGDWLEAYTKIMEINYWTSSECLGARLDPQSGEWEVKILRDGSKEVTLRPKQLILATGMSGFPNVPRVPGQEEFVGDLHHSSKHPGGEAYKGKRAVILGSNNSAHDIAADLWENGAAEVTMIQRSSSHVVRSESLFRFLTQEIYSESAVESGITTDKADMIFASLPYKIMGDAQRCNSDAIRAHDKDFYAALTKTGFMLDFGDDDSGLFMKYLRRGSGYYIDVGASQLLIDGEIKLRSGVTIASLKPSSVVLSDGTEIAADVVILATGYGSMNQWAAKLISQEVADRVGKCWGLGSNTRKDPGPWEGELRNMWKPTAQEGLWFHGGNLHQARHYSLYLALQLKARMEGIATPVYGVPKVHHLS

>113792|Smoellendorffii_v1.0/1-611

MATVNGSPKIDPDALVDAWLARLDSSLASGDASAAANLFEESTSFWRDLIAFTWNIVTVEGRSQIHAMLQSTLESVAPKGWVRNGTASYSADSGVIEAWIKFETRDAVCTGHLRLMATTSLCRTLLTAMEGLRDFPENKGRTRPNGVTHGVIRHRASWLDGRKEEERTLGSTVQPYCVIVGGGQAGIGLAARLRQLGVPCIVVEKNPRPGDSWRNRYKSLCLHDPVWYDHLPYLPFPENWPIFAPKDKMGDWLEAYTKIMEINYWTSSECLGARLDPQSGEWEVKILRDGSKEVTLRPKQLILATGMSGFPNVPRIPGQEEFVGGLHHSSKHPGGEAYKGKRAVILGSNNSAHDIAADLWENGAAEVTMIQRSSSHVVRSESLFRFLTQEIYSESAVESGITTDKADMIFASLPYKIMGDAQRCNSDAIRAHDKDFYAALTNTGFMLDFGDDDSGLFMKYLRRGSGYYIDVGASQLLIDGEIKLRSGVTIASLKPSSVVLSDGTEIAADVVVLATGYGSMNQWAAKLISQEVADRVGKCWGLGSNTRKDPGPWEGELRNMWKPTAQEGLWFHGGNLHQARHYSLYLALQLKARMEGIATPVYGMPKVHHLS

>evm.model.Chr1.494|Isoetes_sinensis/1-614

MAIDTCNGSFNSTADPSNAKVDDFLSNLDHYLQVRDVPKITDLFGDGGDAYWRDLLSFTWNIITVEGREEIARMLESTLEHTNPKGFIRDSEATAYGNDGLQAWFRFETDVGICKGHVRIKGDGKCRTLFTAIAELKGYPEQKGKNRPYGAEHGVIQGRKTWLDRRKEEEQTLGYSIQPYCVIVGGGQAGIGLGARLKQLQVPTLIVEKNGRAGDSWRNRYKSLCLHDPVWYDHLPYLQFPENWPVFSPKDKMGDWLEMYTKVMELNYWSSTECLSARFEEGNKEWSVLVRREGKEIELRPKQLVLATGMSGMPNVPKIPNAERFKGDIRHSSAHPGGEGYRGKRAIIVGSNNSAHDIAADFWENGADVTMIQRSTTHVSKCDSMLRYSIGELYSESAVESGITTDLADMTFASIPYKIMTEFQRPIVDKIRAQDADFYKELQKSGFMLDFGDDETGLFMKYLRRGSGYYIDVGCSQLIIEGKVKLKTKVDIMDVKEESVVLSDGTELGADVIILATGYGSMNGWAARLISKEVADKVGKCWGLGSGTTKDPGPWEGELRNMWKPTRQEALWFHGGNLHQARHYSLYVAFQLKARMEGIPTPVYGLPSTTYHLS

>evm.model.Chr2.397|Isoetes_sinensis/1-614

MAIDTCNGSFNSTADPSNAKVDDFLSNLDHYLQVRDVPKITDLFGDGGDAYWRDLLSFTWNIITVEGREEIARMLESTLEHTNPKGFIRDSEATAYGNDGLQAWFRFETDVGICKGHVRIKGDGKCRTLFTAIAELKGYPEQKGKNRPYGAEHGVIQGRKTWLDRRKEEEQTLGYSIQPYCVIVGGGQAGIGLGARLKQLQVPTLIVEKNGRAGDSWRNRYKSLCLHDPVWYDHLPYLQFPENWPVFSPKDKMGDWLEMYTKVMELNYWSSTECLSARFEEGNKEWSVLVRRDGKEIELRPKQLVLATGMSGMPNVPKIPNAERFKGDIRHSSAHPGGEGYRGKRAIIVGSNNSAHDIAADFWENGADVTMIQRSTTHVSKCDSMLRYSIGELYSESAVESGITTDLADMTFASIPYKIMTEFQRPIVDKIRAQDADFYKELQKSGFMLDFGDDETGLFMKYLRRGSGYYIDVGCSQLIIEGKVKLKTKVDITDVKEESVVLSDGTELGADVIILATGYGSMNGWAARLISKEVADKVGKCWGLGSGTTKDPGPWEGELRNMWKPTRQEALWFHGGNLHQARHYSLYVAFQLKARMEGIPTPVYGLPSTTYHLS

>evm.model.Chr1.476|Isoetes_sinensis/1-610

MAIDTSDHAADLSNPKVDHFLSSLDHYLQLRDVAKITGLFGDGGDAFWRDLLSFTWNVITVEGREEIARMLESTLEHTNPKGFVRDSEATTYGDDGLQAWFRFETDVGICKGHVRIKGDGKCRTLFTAIAELKDYPEQKGKNRPYGAEHGVIQGRKTWLDRRKEEEQTLGYSIQPYCVIVGGGQAGIGLGARLKQLQVPTLIVEKNGRAGDSWRNRYKSLCLHDPVWYDHLPYLQFPQNWPVFSPKDKMGDWLEMYTKVMELNYWSSTECLSARFEEGKKEWSVLVRKEGKEIELRPKQLILATGMSGMPNLPKIPNAERFKGDIYHSSAHSCGKGYRGKRAVIVGSNNSAHDIAEDLWENGADVTLVQRSTTHVCKTDSFVRYYLGDLYSESAIESGITTSLADMIFASIPYKMMTEFQRPIMDKIRAQDEDFYKDLQKSGFMLDFGDDETGLFMKYLRRGSGYYIDVGCSELIIEGKVKLKTKVNIVDVKEESVVLSDGTELGADVIVLATGYGSMNGWAAKLISKEVADKVGKCSGLGSGTTKDPGPWEGELRNMWKPTRQEALWFHGGNLHQARHYSLYVAFQLKARMEGIPTPVYGLPSTTYHLS

>evm.model.Chr2.391|Isoetes_sinensis/1-610

MAIDTSNHAADLSNPKVDYFLSSLDHYLQLRDVAKITGLFGDGGDAFWRDLLSFTWNIITVEGREEIARMLESTLEHTNPKGFVRDSEATAYGDDGLQAWFRFETDVGICKGHVRIKGDGKCRTLFTAIAELKDYPEQKGKNRPYGAEHGVIQGRKTWLDRRKEEEQTLGYSIQPYCVIVGGGQAGIGLGARLKQLQVPTLIVEKNGRAGDSWRNRYKSLCLHDPVWYDHLPYLQFPQNWPVFSPKDKMGDWLEMYTKVMELNYWSSTECLSARFEEGNKEWSVLVRREGKEIELRPKQLVLATGMSGMPNVPKIPNAERFKGDIYHSSAHSCGKGYRGKRTIIVGSNNSAHDIAADLWENGADVTMVQRSTTHVCKTDSFVLYYLGDLYSESAIKSGITTSLADMIFASIPYKMMTEFQRPIMDKIRAQDEDFYKDLQKSGFMLDFGDDETGLFMKYLRRGSGYYIDVGCSELIIEGKVKLKTKVNIVDVKEESVVLSDGTELGADVIVLATGYGSMNGWAAKLISKEVADKVGKCWGLGSGTTKDPGPWEGELRNMWKPTRQEALWFHGGNLHQARHYSLYVAFQLKARMEGIPTPVYGLPSTTYHLS

>Mapoly0052s0062|Mpolymorpha_v3.1/1-608

MASNGTLENGSSDNSKIVKAWLSYLDEALQKQDIAHLVTLFDEEECFWRDMLAFTWNLYTAESRDEIAAMMNSTLATVKPSGWELDGEVEEKGGALQVFLKFETSIARGRGHLRLKGGKCWTLLTAMTELKGYEEHLGSARPMGVKNENVPGRKTWLEERMQERQELGYKSQPYCVIVGGGQAGIALAARLRMLNVPALIIEKNARPGDSWRNRYKSLCLHDPVWYDHLPYLPFPSNWPVFSPKDKMGDWLEAYTNLMELNYWSSTECKSCTYNSADGVWEVSVVRSSGGSESETIQLRPQHLVLATGMSGMPNVPKFPGAQSFEGLQCHSSKFQTADDWKGKQCVVIGSNTSAHDICSSLWETGAAQVTMVQRSSTHVLRVNTCLNNLARDGYGEGGPPVDTVDLAFASVPYKIFHSFAIPAMQRIAKAEAPFYEGLSKAGFVLDWGADGSGLFTKYLRRGGGYYIDVGASELISNGSIKLKSGVSVQEIKPKSIVFSDGSELPADLIVYATGYGNMNQWAAKLISQEVADKVGMVWGLGSNTTNDPGPWEKELRNMWKPTQQPGLWFMGGNLHQCRHYSLVVALQLKARLEGIPTPVYALPKVNHA

>Rn_11391.1_1|Ricciocarpos_natans/1-609

MEAAPLQHPDQQQGGGEIVQAWLSEFDEALQNQDIAHLVTLFDEEECFWRDLLAFTWNLYTAESRDEIAAMMKSTLEGTKPSGWQVDGKVEEKDNVLSVFLKFETAVGRGRGHLRLKGGKCWTLLTAMTELKGYEEPLGMARPNGVKDGNVPGRKSWLEKKMEEKKNLGYNTQPYVVIIGGGQAGIILAARLRMLNVPTIIVEKNARPGDSWRKRYKSLCLHDPVWYDHLPYLPFPPNWPVFSPKDKMGDWLEAYTNLMELNYWSSTECKSASYDEKQGQWEVHVERSSGSSTETIQLRPKHLVLATGMSGMPKVPKFVGAENFQGKQYHSSKFSSGEDWVGKQCVVIGSNTSAHDICTNLWECGAASVTMVQRSSTHVLKVSTCTENLARDGYGEGGPPVDDSDLRFASVPYKIFHRFAIPVMQRIAKQHVQFYEQLSKAGFVLDWGDDGSGLFSKYLRRGGGYYIDVGASELVSNGSIKLKSRVNVVEIKPKSILLSDGSELAADLIVYATGYGNMNQWAAKLISQEVADKVGICWGLGSGTTNDPGPWERELRNMWKPTQQPGLWFMGGNLHQCRHYSLPLALQLKARMEGIPTRAYGIPPVNHVN

>Rn_11391.1_2|Ricciocarpos_natans/1-609

MEAAPLQHPDQQQGGGEIVQAWLSEFDEALQNQDIAHLVTLFDEEECFWRDLLAFTWNLYTAESRDEIAAMMKSTLEGTKPSGWQVDGKVEEKDNVLSVFLKFETAVGRGRGHLRLKGGKCWTLLTAMTELKGYEEPLGMARPNGVKDGNVPGRKSWLEKKMEEKKNLGYNTQPYVVIIGGGQAGIILAARLRMLNVPTIIVEKNARPGDSWRKRYKSLCLHDPVWYDHLPYLPFPPNWPVFSPKDKMGDWLEAYTNLMELNYWSSTECKSASYDEKQGQWEVHVERSSGSSTETIQLRPKHLVLATGMSGMPKVPKFVGAENFQGKQYHSSKFSSGEDWVGKQCVVIGSNTSAHDICTNLWECGAASVTMVQRSSTHVLKVSTCTENLARDGYGEGGPPVDDSDLRFASVPYKIFHRFAIPVMQRIAKQHVQFYEQLSKAGFVLDWGDDGSGLFSKYLRRGGGYYIDVGASELVSNGSIKLKSRVNVVEIKPKSILLSDGSELAADLIVYATGYGNMNQWAAKLISQEVADKVGICWGLGSGTTNDPGPWERELRNMWKPTQQPGLWFMGGNLHQCRHYSLPLALQLKARMEGIPTRAYGIPPVNHVN

>Rn_11391.1_3|Ricciocarpos_natans/1-609

MEAAPLQHPDQQQGGGEIVQAWLSEFDEALQNQDIAHLVTLFDEEECFWRDLLAFTWNLYTAESRDEIAAMMKSTLEGTKPSGWQVDGKVEEKDNVLSVFLKFETAVGRGRGHLRLKGGKCWTLLTAMTELKGYEEPLGMARPNGVKDGNVPGRKSWLEKKMEEKKNLGYNTQPYVVIIGGGQAGIILAARLRMLNVPTIIVEKNARPGDSWRKRYKSLCLHDPVWYDHLPYLPFPPNWPVFSPKDKMGDWLEAYTNLMELNYWSSTECKSASYDEKQGQWEVHVERSSGSSTETIQLRPKHLVLATGMSGMPKVPKFVGAENFQGKQYHSSKFSSGEDWVGKQCVVIGSNTSAHDICTNLWECGAASVTMVQRSSTHVLKVSTCTENLARDGYGEGGPPVDDSDLRFASVPYKIFHRFAIPVMQRIAKQHVQFYEQLSKAGFVLDWGDDGSGLFSKYLRRGGGYYIDVGASELVSNGSIKLKSRVNVVEIKPKSILLSDGSELAADLIVYATGYGNMNQWAAKLISQEVADKVGICWGLGSGTTNDPGPWERELRNMWKPTQQPGLWFMGGNLHQCRHYSLPLALQLKARMEGIPTRAYGIPPVNHVN

>Adc30932|Adiantum_capillus-veneris/1-631

MLCPNTPRAGCGRAACSLSLSLSLSLSLSRTLTLACSLMAERINAWLQNLELALQQRAISAATQLFQEEECFWRDFLSFTWNLYTAESREEIRAMLDATLGHTKPSKWAVDGEAEEAEDGALIQAFITFETERGRGRGHVKLRGDKCWCLFTSMVELKGFEEKVGFQRVMGVQHGAIKGRKTWLEGRIEEVEELGKSRQPYCLIVGGGQGGIALGARLRRLGVPTIIVEKNRRPGDSWRNRYKSLCLHDPVWYDHLPYLPFPSHWPVFSPKDKIGDWLEAYTKIMELVYWSSTECKSAKYDTTNGVWEVRLEKEKEEIVLYPKELVLATGLSGFPNVPAFLGAGTFEGVQCHSSEHKGGESWAGKHCVVIGSNNSAHDVSADLWENGAAEVTMVQRTSTHVLRSETMLQEELAFYREGSKYSTSDADILFAAIPFKLFHKFQIPVYQKIAEIDAEFYACLKESGFMLDFGEDGSGLFAKYLRRGSGYYIDVGASDLVIKGEIKLKSGVTVKEVRPKSVCFSDGTEAKADLIVYATGYGSMNEWAAKLISQEVADKVGKCWGLGSGTRKDPGPWERELRNMWKPTKQEGLWFHGGNLHQSRHYSLMLALQLKARMEGIPTPVYGLPEVHHIN

>Aspi01Gene36682|Alsophila_spinulosa/1-590

MASRISAWLESLDSALQKEDISAAADLFHEEECFWRDLVTFTWNLYTAESREEICAMLEATLEHTKPSLWAIDGEAEVAEGGAAVIQAFITFETQRARGRGHVKLRGDKCWCLFTSIVELKGYEERREFGVQHGAIKGRKSWLERRIEEQEELGRTKQPYCLIVGGGQGGIALGARLRQLQVPTIIVEKNKRPGDSWRNRYKSLCLHDPVWYDHLPYLPFPSHWPVFSPKDKMGDWLEAYTKLMELVYWSSTECKSAKFIASEGLWEVIVEREGEKAMLYPKELVLATGMSGYPNVPEYPGAETFEGVQCHSSQHKGGENWAGKHCVVIGSNNSAHDVSADLWESGAAKVTMVQRTSTTVVRSETMLQEGLVFYREGSKYSTSDADILLASRPYRIFHKFEAAVYRKIAEIDAEFYTRLEERGFLLDFGEDRSGHFTKYLRRGSGYYIDVGASELVISGDIQLKSGVTVKEVKPKSVVFSDETETEADLIVYATGYGSMNEWAAKLISKEVADKVGKCWGLGSGTRKDPGPWERELRNMWKPTQQEGLWFHGGNLHQSRHYSLMLALQLKARMEGIPTPVYGMPEVHHIK

>Aspi01Gene36686|Alsophila_spinulosa/1-590

MASRISAWLESLDSALQKEDISAAADLFHEEECFWRDLVTFTWNLYTAESREEICAMLEATLEHTKPSLWAIDGEAEVAEGGAAVIQAFITFETQRARGRGHVKLRGDKCWCLFTSIVELKGYEERREFGVQHGAIKGRKSWLERRIEEQEELGRTKQPYCLIVGGGQGGIALGARLRQLQVPTIIVEKNKRPGDSWRNRYKSLCLHDPVWYDHLPYLPFPSHWPVFSPKDKMGDWLEAYTKLMELVYWSSTECKSAKFIASEGLWEVIVEREGEKAMLYPKELVLATGMSGYPNVPEYPGAETFEGVQCHSSQHKGGENWAGKHCVVIGSNNSAHDVSADLWESGAAKVTMVQRTSTTVVRSETMLQEGLVFYREGSKYSTSDADILLASRPYRIFHKFEAAVYRKIAEIDAEFYTRLEERGFLLDFGEDRSGHFTKYLRRGSGYYIDVGASELVISGDIQLKSGVTVKEVKPKSVVFSDETETEADLIVYATGYGSMNEWAAKLISKEVADKVGKCWGLGSGTRKDPGPWERELRNMWKPTQQEGLWFHGGNLHQSRHYSLMLALQLKARMEGIPTPVYGMPEVHHIK

>Aspi01Gene20535|Alsophila_spinulosa/1-592

MAGQISAWLHSLDSALQKKDLTAAAELFQAEECFWRDLVAFTWNLYTAESREEIRAMLEATLDDTKPFGWEIEGEAEEGQDGVLQAFFRFETQTARGRGVVRLRGGKCWCLITSMLELKGHEENAGFRRQLGVQHGAVKGRKTWLEGRLEEIEELGRTRQPYCLIVGGGQGGIALGARLRRLGVPTIIVEKNKRPGDSWRNRYKSLCLHDPVWYDHMPYLPFPDHWPVFSPKDKIGDWLEAYTKIMELVYWNSTECKSARFDTSKGSWEVLVEREGEVVMLYPKELVLATGMSGVPNVPSFPGAEAFQGLQCHSSQHRGGESWANKHCIVIGSNNSAHDICADLWENGAAEVTMVQRTSTHVLRSETMMSVQLSLYREGSKYSTDDADLLVASTPYKILHKAQIPVYKRIADIDADFYGRLKKAGFMLDFGEDGSGLLLKYLRRGSGYYIDVGASQLVINGDIKLKSGVTVKEVRPRSVVFSDGTEADADLIVYATGYGSMNGWAAKLISQDVADKVGKCWGLGSDTRKDPGPWERELRNMWKPTQQCGLWFHGGSLQESRHYSLPLALQLKARMEGIPTPVYGLPKVHHSN

>Aspi01Gene20557|Alsophila_spinulosa/1-592

MAGQISAWLHSLDSALRKKDLTAAAELFQAEECFWRDLVAFTWNLYTAESREEIRAMLEATLDDTKPFDWEIEGEAEEGQDGALQAFFRFETQTARGRGVVRLRGGKCWCLITSMLELKGHEENAGFRRQLGVQHGAVKGRKTWLEGRVEEIEELGRTRQPYCLIVGGGQGGIALGARLRRLGVPTIIVEKNKRPGDSWRNRYKSLCLHDPVWYDHMPYLPFPDHWPVFSPKDKIGDWLEAYTKIMELVYWNSTECKSARFDTSKGSWEVLVEREGEVVMLYPKELVLATGMSGVPNVPSFPGAEAFQGLQCHSSQHRGGESWANKHCIVIGSNNSAHDICEDLWENGAAEVTMVQRTSTHVLRSETMMSVQLSLYREGSKYSTDDADLLVASTPYKILHKAQIPVFKRIADIDADFYGRPKKAGFMLDFGEDGSGLLLKYLRRGSGYYIDVGASQLVINGDIKLKSGVTVKEVRPRSVVFSDGTEADADLIVYATGYGSMNGWAAKLISQDVADKVGKCWGLGSDTRKDPGPWEHELRNMWKPTQQCGLWFHGGSLQESRHYSLPLALQLKARMEGIPTPVYGLPKVHHSN

>Aspi01Gene25861|Alsophila_spinulosa/1-592

MAGQICSWLRSLDSALQKQDLTAAAELFQAEECFWRDLVAFTWNLYTAESREEIRAMLEATLDDTKPFGWEIEGEAEEGQDGVLQAFFRFETQTARGRGVVRLRGGKCWCLITSMLELKGHEENAGFRRQLGVQHGAVKGRKTWLEGRVEEIEELGRTRQPYCLIVGGGQGGIALGARLRRLGVPTIIVEQNKRPGDSWRNRYKSLCLHDPVWYDHMPYLPFPDHWPVFSPKDKIGDWLEAYTKIMELVYWNSTECKSARFDTSKGSWEVLVEREGEVVMLYPKELVLATGMSGVPNVPSFPGAEAFQGLQCHSSQHRGGESWANKHCIVIGSNNSAHDICEDLWENGAAEVTMVQRTSTHVLRSETMMSDELSLYREGSKYSTDDADLLFASIPYKIFHKAQIPVYKRIADIDADFYGRLKEAGFMLDFGEDGSGLFLKYLRRGSGYYIDVGASQLLINGAIKLKSGVTVKEVRPRSVVFSDGTEADADLIVYATGYGSMNGWAAKLISQDVADKVGKCWGLGSATRKDPGPWERELRNMWKPTQQCGLWFHGGNLHQSRHYSLPLALQLKARMEGIPTPVYGLPKVHHSN

>Aspi01Gene47220|Alsophila_spinulosa/1-592

MAERVKSWLHSLEFALEKRDLIATAELFREDECFWRDLVAFTWNLYTAESREEIVAMLEATLKETMPSRWEIEGEVEEGEDGLLQAFISFETQRARGRGHVKIRGDKCWCLLTAMQELKGHEEKAGLRRELGVQHGVVKGRKTWLEGRVEELEELGRSRQPYCVIVGGGQGGIALGARLRRLGVPTIIIERNERAGDSWRNRYKPLCAHLPVWSNHMPYVPFPDHWPVFSSKDQLGDWLEAYTKMMELVYWTSTECKAARFDDGKKRWEVRVDREGEEVALYPKELVLAMGLSGLPNVPSILGAEHFQGVQCHSSQFKGGDEWAGKRCMVIGSNNSAHDIAADLWGSSAAKVTMVQRSPTHVLPSRAVVEILQPFYCERSKYSTADADLLLASCPYKLFPQLEIPFMNKIAETYGEFYHRLQKAGFIHTFGEDGSGAFSMYLRRGSGYYIDVGTSELLINGEIQLKSGVSVQEVRASSVTFSDGSEIEADLIVYATGYGPMNGWAAKFISCEVADKVGKCWGLGSDTAKDPGPWERELRNMWKPTHQPGLWFHGGNFQESRSYSHYLALQLKARMEGIPTPVYGLPEVHHTT

>Aspi01Gene63654|Alsophila_spinulosa/1-595

MAERVNAWLQSLEFALEKRDLIATAELFREDECFWRDLVAFTWNLYTAESREEILAMLEATLKETMPSRWAIEGEVEDGEDGQLLQAFISFETQRARGRGHVKIRGDKCWCLLTAMQELKGHEEKAGLRRELGVEHGAVKGRKTWLEGRGDELEELGRSRQPYCVIVGGGQGGIALGARLRRLGVPTIIIEKNERAGDSWRNRYKTLCAHSPVWCNHMPYVPFPDHWPVLSSKDQLGDWLEAYTKVMELVYWTSTECKAARFDEAKKRWEVRVDREGEEVVLYPNELVLATGVSGLPNVPSFSGAEHFQGVQCHSSQFRGGEEWAGKRCVVIGSNNSAHDIAADLWESGAAQVIMVQRSPTHVLPCEAWLEILQPFYRERSKHSTANADLLLAAWPYKLFPQLQIPFLNKIAETYGEFYHRLQNAGFMHTFGEDGSGALSMYLRRGSGFYIDVGTSELLIKGEIQLKSGTAVQEVRATSVTLSDGSEIEADLIVYATGYGPMNGWAAKFIAGEVADKVGKCWGLGSDTAKDPGPWELELRNMWKPTHQPSLWFHGGSFQESRAYSLYLALQIKARMEGIPTPVYGLPDVHHTTCC

>Aspi01Gene63657|Alsophila_spinulosa/1-595

MAERVNAWLQSLEFALEKRDLIATAELFREDECFWRDLVAFTWNLYTAESREEILAMLEATLKETMPSRWAIEGEVEDGEDGQLLQAFISFETQRARGRGHVKIRGDKCWCLLTAMQELKGHEEKAGLRRELGVEHGAVKGRKTWLEGRGDELEELGRSRQPYCVIVGGGQGGIALGARLRRLGVPTIIIEKNERAGDSWRNRYKTLCAHSPVWCNHMPYVPFPDHWPVLSSKDQLGDWLEAYTKVMELVYWTSTECKAARFDEAKKRWEVRVDREGEEVVLYPNELVLATGVSGLPNVPSFSGAEHFQGVQCHSSQFRGGEEWAGKRCVVIGSNNSAHDIAADLWESGAAQVIMVQRSPTHVLPCEAWLEILQPFYRERSKHSTANADLLLAAWPYKLFPQLQIPFLNKIAETYGEFYHRLQNAGFMHTFGEDGSGALSMYLRRGSGFYIDVGTSELLIKGEIQLKSGTAVQEVRATSVTLSDGSEIEADLIVYATGYGPMNGWAAKFIAGEVADKVGKCWGLGSDTAKDPGPWELELRNMWKPTHQPSLWFHGGSFQESRAYSLYLALQIKARMEGIPTPVYGLPDVHHTTCC

>Aspi01Gene63658|Alsophila_spinulosa/1-238

MAERVNAWLQSLEFALEKRDLIATAELFREDECFWRDLVAFTWNLYTAESREEILAMLEATLKETMPSRWAIEGEVEEGEDGQLLQAFISFETQRARGRGHVKIRGDKCWCLLTAMQELKGHEEKAGLRRELGVEHGAVKGRKTWLEGRVEELEELGRSRQPYCVIVGGGQGGIALGARLRRLGVPTIIIEKNERAGDSWRNRYKTLCAHSPVWYNHMPYVPFPDHWPVLSSKDQLGD

>Aspi01Gene63664|Alsophila_spinulosa/1-596

MLAMAERVNAWLQSLAFALEKRDLIATAELFREDECFWRDLVAFTWNLYTAESREEILAMLEATLKETMPSRWAIEGEVEEGEDGQLLQAFISFETQRARGRGHVKIRGDKCWCLLTAMQELKGHEEKAGLRRELGVEHGAVEGRKTWLEGRVEELEELGRSRQPYCVIVGGGQGGIALGARLRRLGVPTIIIEKNERAGDSWRNRYKTLCAHSPVWYNHMPYVPFPDHWPVFSSKDQLGDWLEAYTKVMELVYWTSTACKAARFDEAKKRWEVRVDREGEEVVLYPNELVLATGMSGLPNVPSFLGAAHFQGVQCHSSQFRGGEEWAGKRCIVIGSNNSAHDIAADLWGSGAAQVTMVQRSPTHVLPSGALLEILQPFYRERSKHSTADNDLLLAACPYKLFPQLQIPFLNKIAQTYGEFYHRLLNAGFMHTFGEDGSGAFSMYMRRGSGFYIDVGTSELLIKGEIQLKSGTAVQEVRATSVTLSDGSEIEADLIVYATGYGPMNGWAAKFIAGEVADKVGKCWGLGSDTAKDPGPWERELRNMWKPTHQPSLWFHGGSFQESRAYSLYLALQIKARMEGIPTPVYGLPEVHHTM

>Ceric.34G041100.1.v2.1|Ceratopteris_richardii/1-594

MAERINAWLREFDSVLQNEDIPAAVLLFQEEECFWRDLLSFTWNIYTAESRQEIRTMLEETLTNTKPSNWSIQGEAEELEDAEGLYIQAFVRFETEKARCRGLVKLRGDKCWCLFTTMVELKGFEEPAGRRREIGVQHGSVKGRKSWLQKRIEEEEALGKSKQPYVLIVGGGQGGIMLGARLRRLQVPTIIVDKRKRPGDSWRSRYKTLCLHDAVWYDHLPYLPFPDHWPVYSPKDKMGDWLEAYTKIMELVYWSSTECKSAAYDASTGTWEVHLDKEGKDVVLYPKQLVLATGMSGFPIVPTYPGSESFQGTQCHSSEYKGGEGWVGKHCVVVGSNNSAHDVCADLWENDAAEVTMVQRTSTLVVKAEIMLKVNELYQEGSKYSTEDADILFASVPYRIFHRPSILLYKKIKEWDAEFYSRLRESGFMLDFGEDESGLFPKYLRRGSGYYIDVGASELVINGEIKLKSGVTVKEIRPNSVVFSDGTETKADLIVYATGYGSMNEWVAKLISEEVADKVGKCWGLGSDTRKDPGPWERELRNMWKPTRQEALWFQGGNLHQSRHYSLMLALQLKARMEGIPTPVFGLAEVHHTR

>Ceric.34G041100.2.v2.1|Ceratopteris_richardii/1-538

MLEETLTNTKPSNWSIQGEAEELEDAEGLYIQAFVRFETEKARCRGLVKLRGDKCWCLFTTMVELKGFEEPAGRRREIGVQHGSVKGRKSWLQKRIEEEEALGKSKQPYVLIVGGGQGGIMLGARLRRLQVPTIIVDKRKRPGDSWRSRYKTLCLHDAVWYDHLPYLPFPDHWPVYSPKDKMGDWLEAYTKIMELVYWSSTECKSAAYDASTGTWEVHLDKEGKDVVLYPKQLVLATGMSGFPIVPTYPGSESFQGTQCHSSEYKGGEGWVGKHCVVVGSNNSAHDVCADLWENDAAEVTMVQRTSTLVVKAEIMLKVNELYQEGSKYSTEDADILFASVPYRIFHRPSILLYKKIKEWDAEFYSRLRESGFMLDFGEDESGLFPKYLRRGSGYYIDVGASELVINGEIKLKSGVTVKEIRPNSVVFSDGTETKADLIVYATGYGSMNEWVAKLISEEVADKVGKCWGLGSDTRKDPGPWERELRNMWKPTRQEALWFQGGNLHQSRHYSLMLALQLKARMEGIPTPVFGLAEVHHTR

>Mvestita_C233g22322-RA|Marsilea_vestita/1-300

MSGLPSMPSFPGSESFQGVQCHSSKFKGGEEWAGKRCVIVGSNSSAHDICADLWENGAAEVTMVQRSSTHVLRSEYMLSEGALGEYQEDAKLKTEDIDLKFAAIPYKILHRGAIALYNRIAEKDATFYSKLKEAGFMLDFGDDGSGLFIKYLRRGSGYYIDAGQSELIFKGDIKLKAAVTVKEIKTKSVVFSNGSEVEADLIVYATGYGSMNGWAPKLISQEVADKVGKCWGLGSNTRKDPGPWERELRNMWKPTHQHALWFHGGSLQQSRHYSLYLALQLKARMEAIATPVYGLGESMV

>Mvestita_S2g11366-RA|Marsilea_vestita/1-610

MSPSMSSNASTTMHNDNVAVANSWLCSFSSALSNRDIAAVVELFQAEECFWRDLLTFTWNIYTAESQHEIKAMLEATLDMTKPSNWEIQGDVEESKDGATLECFVTFETEQAWGKGILKLKNAKCWCLLTTMLELKGFKEKSGFGRERGVEHCATKGRVTWKETREKELSELGYTKQPYCLIIGGGQAGIMLGARLRRLGVPTLIVEKNARPGDSWRNRYKSLCLQDPVWIMHLPYLPFPQHWPLFTQKDKMADWLEAYTSIMELVYWASTECRSAHYDSNTQTWKVVLSKQDKEVVVYPKQLVLSTGVCGIPNIPSYPGCKRFRGVQSHCSSFKSAQGWVGKQCVVIGSNSSAHDICASLWENDAAKITMVQRSTTCVLRSKTFLSGGYYGEYEEDAKLKIEDIDLKTAATPYKLKDKFAKAITDRVKEEDSAFYSRLKGAGFLLDFGEDETGLYMKFLRRGSGYYIDVGASELIINGDIKLKTGVNVKEIKPNSVVFSDGSEVEADLIVYATGYQSMNGWVAKLISQEVADKVGICWGLGSNTCKDPGPWERELRNMWKPTQQSALWFHGGIIQQARHYSLYLALQLKARMEGIATPVYGMGEVYHAT

>Mvestita_C233g22319-RA|Marsilea_vestita/1-394

MTNLQDQDNVVANWLSCFGSALTNKDIAGAVQLFQVEECFWRDLLTFTWNIYTAESQDEMKAMLEATLAMAKPSNWQILGDVETSSDGALECFITFDTEKARGKGRLKLRNGKCWCLLTSMVELKRFEEKAGFRREYGVEHGVFKSRSTWKQAREKELVDLGFTEQPYCLIIGGGQAGIALGAWLRRLGVPTIIVEKYNRPGDSWRNRYTSLCSHTPVWYDHMPYLPFPDHWPVFSPKDKIGDWLEAYTSIMELVYWTSTECRSASYNSTTETWKVVLNKQGKEVVVYPKQLVLATGMSGVPNIPSFLGSESFQGVQCHSSKFKGGEEWAGKRCVIVGSNNSAHDICADLWENGAAEVTMVQRSSTHVLRSETMLSEGVHGEYQEDAKLKTEVC

>Mvestita_S16g10734-RA|Marsilea_vestita/1-600

MEATNHYIIVCNWLSSFGSALSKRDIAAAVELFQAEECFWRDLLTFTWNIYTAESQHEIEAMLEATLDIAKPCNWKIEGDVETSNNGAVLECFITFETEQARGKGRLSIKNGKCWCLLTSMLELKGFEEKAGVTRENGVQLGVSKNRATWKQARYEELAELGITKQPYCLIVGGGQAGLTLGARLRRLGVPTIIVEKNNRLGDTWRNRYNALCLHCPSWFQHFPYLPYPDHWPLYCPKDKMADWHEAYASIMELVCWTSTECKSACYDSNTEVWKIVLNKQGKEVVVYPKQLVLATGISGIPNMPNFPGYESFKGDQLHSSMFRSGANWAGKRCVVIGSCNSAFDICADLWENGASEITMVQRSSSHVIRTETTLSKQVFGPWHVEGSKYTTDEADLIFAAIPYKILHQAFIPLYDRVQEVDSAYYDKLKAAGFMLDFGDDGSGVLMNFLRSASGFAIDVGPAQLIIDGDVKLKSGVCVKEIRPSSVVFTDGSEIEADLIVYATGYGSMNALIAKLISQEIADKIGKCWGLGSKTRKDPGPWERELRNMWKPTHQPALWIHGGGLLHARHYSLYLALQIKARMEGIPTPVFGMPKVNHSS

>Adc32115|Adiantum_capillus-veneris/1-596

MAELQVKAWLERLEKALQERDVDTAVELFEEEECFWRDLLAFTWNLYTAESREEIRAMLAATLDASQPSAWHIEAHVEEDEATHVVQAFIRFETQQARCRGHLKLRAGKCWCLLTAMHELKGFEEKAGPRRDVGLQHVCAKGRKTWLELRTEEVQELERSKEIYCVIVGGGQAGVALAARLRRLGVPAIIIEKNKRVGDTWRNRYRSLCLHSPVWYAHFPYMPFPDDWPVYISKDRMADWIEAYTEAMGLMYWTDTECKAAKYENERQRWEVRVEREGEEMVLHCKQLVLATGMSGFPHVPTLKGAASFQGVQIHSSQYRSGEEWADKQCVVVGSNNSAHDICADLWECGAASVTMLQRSPTQVIRYPAFAEAHVPFYCADSKYSIPEADLLLASCPYKLFPRLQAPLYKKFAAMDKPFYQGLEKAGFMHTFGEDHSGIFSMYYRRGSGFYVDVGASELVINGEIKLKCRVEVEEVKASSVVVSDGSEVKADLIVYATGWSNMNAWAASFISQDVADKVGKCWGLGSATLKDPGPWEREIRNMWKPTQVRNLWFHGGGFQETRHYSLYLALQLKARLEGISTPVYGMPRVHHTSSV

>Adc32171|Adiantum_capillus-veneris/1-594

MAELQVKAWLHSFEKALQERDVNAASELFVEEECFWRDLLAFTWNLYTAESREEIRVMLAATLDAAQPRAWQIEGHVEEAEATLLIQAFIRFETQQASCRGHLKLRAGKCWCLLTAMHELKGFEEKAGLRRDVGLQHVPAKGRKTWQELRAEEIQEIQRSKETYCVIVGGGQAGVALAARLRRLGVVAIIIEKNKRAGDTWRNRYRSLCLHSPVWYAHFPYMPFPDDWPVYISKDRMGDWIEAYTEAMGLMYWTATECKGAKYAEEHQRWEVRVEREGEEMVLHCKQLVLATGMSGFPHVPIFKGAESFKGVQMHSSQYRGGQEWADKHCVVVGGNNSAHDICADLWECGAARVTMLQRSPTQVVRYPAFAEAHAPLYCADSKYSTTEADLMLASFPYKLLPRLQAPLYMKFAAMDKSFYQGLANAGFMHTFGEDHSGIFSVYYRRGSGFYVDVGASELVINGEIKVKSRVEVEEVKASSVVLSDGSEVEADLIVYATGWSNMNAWAASLISQEVADKVGKCWGLGSNTVKDPGPWEREIRNMWKPTQVKNLWFHGGSFQEARHYSLYLALQIKARLEGIPTRVYGMPLVHHTS

>Sacu_v1.1_s0140.g022671|Salvinia_cucullata/1-600

MASSAASPHAQIDSWLRLFESSLLSRDSSATAALFHPDECFWRDLAAFTWNIYTAESRDEIQAMLDATLASTDPKEWRVVGEAQVAGDVVEAFLAFETGVGRGNGYVKIREGKCWCLLTALKELKGYEERAGERRDIGAQYGNEKGKRTWAERREEEVEEAEKRPYCVIVGGGQAGITLAARLRRLGVPALILEKNERAGDSWRKRYKSLCLHDTVWYDHLPYLAFPDHWPIFTPKDKIADWLEAYVSVMELVYWTSSECVAARFDSAAERWEVVVKRRNYSDAEALVQLFPAHLVIATGMSGLPRIPSLPGSQLFRGVQIHSSGFPGGAAWRGKRCVVIGANNSAHDVCADLWENGAASVTMVQRSSTTILRQQSVLSARTDYKEGSRYTTEEADLRFAAIPYKIMHREQIQIYEGIQAVHREFYDGLRRCGFKLDFGEDGSGLFMKYVRRGSGYYIDTGASELVMKGEIRLKSGEVVSLKEGGVVYGDGSEEEAELVVYATGYGSMNGWAAELISEAAADSVGKCWGLGSGTTKDPGPWERELRNMWKPTRERNLWFHGGNLHQCRHYSLFLALQIKARMHSIPTPVYPLPAPPHHTS

>Adc16228|Adiantum_capillus-veneris/1-620

MDRQEDSAKIEVEDDTLQSRVRSWVRNFGEAMERGDVDGTVEMFEEGECFWRDLLAFTWNIDTAESRQDICCMLHATLQSTQPRNWELLGDAEGVPGSDVVQCFVTFETQFGRCKGVVKLRGVVDYQCWCLLTTLQELKGHEERAGPRREVGVQHGSIRGRKSWLERRTEELQALGSSRQPYCLIVGGGQNGIVLGARLRRLGVPAIIVEKNACAGDSWRQRYKSLCVHTPVWYDHLPYIPFPDHWPKFASKDQVGDWLEAYTLAMDLVYWASTECKAARFDDSKQRWEVKLLRKLDGTSPNKPIQVTLYPTHLVLATGMSGSPNVPDFPGARDVFQGTQYHSSQFRAGDQWAGKHCVIIGSNNSAHDICAHLWESAAASVTMVQRSPTHIFPSDNFVEVVSAAYLGKHSTAEGDLLLAALPYKLLPQAHIPVASKAGEEHVELYKELEKVGFMHTFGEDGSGMIPLYLRRGSGYYIDVGATELVRAGKVKLKSRVEVVGLSERGVQLSDGSEVEADLVVYATGYKPMNDWAARLISEEVAGKVGLCWGLGSNTVKDPGPWQRELRNMWKPTPQANLWFHGGGFHEARHYSLYLALQIKARMEHIPTPVFRFPPIYSSSL

>AANG004279|Anthoceros_angustus/1-605

MAATEQVKAWLARFQEALQEQQVEAAAELFQEDRCYWRDLLAFTWNIYTAESRADIVALLTATLPVVKPQQWEVVGEAVEADSDGLPVFQAFIRFETAVARGRGVVRIGKVSGKCQTLLTAVKELKGHEEQAGPRRPRGTAEGALRGRKTWLEVTAAERAELGYSTQPYCVIVGGGQAGIVLGARLRRLGVSCIILEKQARSGDSWRSRYKSLCLHDPVWFDHLPYLPFPDSWPIFSPKDKMADWLECYTKIMELDYWTSSECRSASYDEAGSRWEVKVVRKGGDGEKEDEIVTLRPAHLVLATGMSGIPNIPSIPGADSFQGVLCHSSGYGGPEKFAGKKCVVIGCNTSAHDISADLWEAGAEVTMVQRSETHIVRVSTIMQTIASLYGEESPFPVEDADLIFASTPYAIWMRTLAGVAEWVAQTDADLYARLKKAGFLVGPGDTRGGLFGGYLTRGSGYYIDVGASDLVADGEIKLKSGVGVKELTPEGVRLSDGDHLPAELVVYATGYGSMTGWAEKLVSKEVADKVGRTWGLGFGTARDPGPWEGELRNMWKPTRQPGLWFHGGNLQQDRFYSLVLALQIKARKEGISTPVYGMAKESYVR

>Ceric.1Z188500.1.v2.1|Ceratopteris_richardii/1-625

MRGIDMDEKLTNDESHHPDSEKTMEMEMSAWLQLFADALGRGDIDAAVDMFDEEECYWRDLLAFTWNIETAESRSEIGVMLHATLQTTMPGNWRVTSNVERIRRPGCNDGLIQCLVSFETKYGRCKGVVRLRGAKCWTLLTALQELTEESEESTPRLEIVPEHALRKTCKRSLERRRQQLDALDSSKQPYCLIVGGGHSGLGLGARLRMMGVPTIIVDKNTRAGDSWRKRYKSLCLHTLSCSDRMPYMPYPENWPQFPSKDKIADWLEVYAEAMELVYWNSTECATASFDETVQKWNVKVVMHDSGETVMLNPHHIVLATGISGQPNIPDLPGARDVFRGKQCHSSQFCDEEEWTGKHCVVIGSNNSAHDICSHLWKSRAATVTMVQRSPTHVLPSERSNFGTEALYMGKYNIRDADLIRASNPYKSLPTTQTHLTRETAELYADLYKELEEVGFKHTFGEDGTGAVLLYLRRASGYYIDVGTSQLIRNRQVKLKSGVEVVGLSERSVQLSDRTELDADLLVYATGYMPMQEQTARLISKEVADRVGRVWGLGSNTTRDPGPWVRELRNMWKPTPQSNLWFHGGGFQEARNYSLYLALQLKARMLNIPISIFQFPAGPCNKLQEN

>Mapoly0122s0026|Mpolymorpha_v3.1/1-622

MGSEELSAGQKITSWLSEFDTVLQDEDVDQILELFDDQECFWRDLLPFTWNIETFESKDEILKMLTENLARVKPRAFVIDGDVIDRPNGLMGSAFKFETSLAWCRGFVWLKGGKCWTITTSMWDLKGYEEKISRSRPFGPTLGQIKGRKTAYQEKQQEQHDMGRSKQPYCLVVGAGQGGVMLGARLRMLGVPAIIVEKNERLGDNWRNRYKSLALHTPCFSDHYPYLPFPSNWPVLAPKEKLADFIECYGKLMDLNVWCSTECKSASYDEKEGHWTVVLERKCGSEVSEVVVHPSHVVLACGLAGYPKMPSFPGKENFQGVQIHSSKFVQGEDYTGKKCIIIGAGTSAHDICLDLWEQGASEITLVQKSHTHIVTMKDFLESRLDPQLEKAELDTNFDTDAFDLKSASLPYKVLPRLNVPIYARIAQKEAELYDKLHKAGFKTFMGEDESGLILFLLRRAAGYYIDMGASELIADGEVRVKNGVTVQAMKENSVVFDDGSEVSADVIIYATGYGNMQEFVEKLISKEAAEKVGLCWGLGSGFKGDPGPWVGELKNMYRPLPVPALWILSGGLSHCRFYSRHVALQLKARMEGIPTPVYKLVRHKEPDSPVLASKMSSIQMQD

>Lc_composite_13448.1_1|Lunularia_cruciata/1-604

MGTRAEDQITAWLATFDTALQGGDVEHVLELFAPEECFWRDFVAFTWNIYTADTKDQIAALLRDNLTRVKPTGWEIDDAAFERPGGVVGAKIKFETSVGWARGFVWLKGEKCWIMMTAIWDLKGHEEKIGKRRPTGPTFGQVCGRKSWIEQRKEEEQELGHSKQPYCVIVGGGQTGLAVGARLKVLGVPTIIVEKNETAGDTWRNRYKSLSLHTPSFSDQFPYMSYPADWPILPSKDKLGDWFESYAKVMVLNYWCSTECKSASYDEKEGKWEVKVMRSSGTEVTEITLRPAHLIIACGLAGFPKVPTFPGAESFEGAQIHSVEYEKGADWKGKKCLVIGASTSAHDICLDLWEQGAAEVTMMQNSSTGVVRVQTFLDSRADADLPGTENDRAFDIDAFDLKSASLPFKVLTRMMIGTYTKIAERDAKFLEDLKKVGFQWTMGEDGSGLSLMLIRRGGGFYIDVGASGLLINGDVKLKSGVKVENINPKSVVFSDGSEVDSDVIIYATGFGNAKQFVEQLISQETADKVGLCWGLGSNTKGDPGPWEGELRNMYKPTAQPGLWIASGGFSHARFYSRYLAVQLKARMEGIPTPVYTPHPSIALK

>Lc_composite_20707.1_1|Lunularia_cruciata/1-611

MSTISSKTREEVSAWLSQFNEALLEHDVSGVLNLFHDEECFWRDFVAFTWNIHTAESKEQIAALLEATLDTTKPRGWEIDATVEQTNEPCDQAEEANALVEVPIKFETSVARCRGNVKLKRGKCWILFTAAWELKGYEEPAGRLRPMGKPYGVVRNRKTWLEKKEAEKEELESTNRPYCVIVGGGQAGIILGARLKVLGVPTIILEKNDKAGDSWRNRHNNLHTHTPAYMDHFPYMAFPTTWPVFPSKDQLAEFMECYTTVMGLNYWCSSECQRATFDEKEGRWHVLVKDKRTSSTKELNPVHLVLATGGSGYPFVPEFKGADSFEGVQFHSSEYRKGSDWTGKQCVVIGACTSAHDICEDLWEQGAAQVTMVQKSSTCVIGVNSMYPDPNPAAFVDAPLLAAENFDLLSSSNPYKVLTQQCVPMVKAIAEKDAKFYEDLNKAGFKTCFGEDNTGLPMLFHRRLGGYYLDVGASELIINGEISVKSEVNVKELRPKSVAFSDGSEIPADLIVYATGYSNMKEFVAKLMSQETADKLGRIWGLGSNTAHDPGPWVGEYRNVWKPTAQANFWTTGAAINQARQMSRYLAIQLKARMEGIQVSVYNDPASPQST

>Lc_composite_20712.1_1|Lunularia_cruciata/1-611

MSTTSSKTREEVSAWLSQFNEALLEHDVPRVLNLFHDEECFWRDFVAFTWNIHTAESKEEIAALLEATLDTTKPSGWEIDATVEQTDEPSDQAEEVNALVEVPIKFETSVARCRGNVKLKRGKCWILFTAAWELKGHEEPAGRFRPMGKPYGVVRNRKTWLEKKEAEKEELESTNRPYCVIVGGGQAGIMLGARLKVLGVPTIILEKNDKAGGSWRNRHNNLHTHTPAYADHFPYMAFPTTWPVFPSKDQLAEFMECYTTVMGLNYWCSSECQRATFDEKEGRWHVLVNDKRTSSTKELNPVHLILATGGSGYPFVPEFKGADSFEGVQFHSSEYRKGSDWTGKQCVVIGACTSAHDICEDLWEQGAAQVTMVQKSSTCVIGVNSMYPDPNPAAFVDAPHLAVENLDLVLSSNPYKVLTQQCVSMVKAIAEKDAKFYEDLNKAGFKTCFGEDNTGLFMLYHRRLGGYYIDLGASELIINGEISVKSGVNVEELRPKSVAFSDGSEIPADLIVYATGYSNMKEFIAKLISQETADKVGQIWGLGSNTAHDPGPWVGEYRNVWKPTAQANFWTTGAAIYQTRQMSRYLAIQLKARMEGIQISVYNDPVSPQST

>Lc_composite_20710.1_1|Lunularia_cruciata/1-609

MSRLTQSMNSTLSTGGTEELATWLSQFKEAIVKYDISGVLDLFADDECFWRDFVAFTWNIQTLESKDAIAAMLEATLEATSASGWELSGDAREANGLVEGNLTFETSVARCRGYVKLKGGKCWVLFTAAWELKGHEEEASKFRPIGLPYGSVRNRKTWLQKKEEEEKELECNKRPYCVIVGGGQAGIMLGARLKVLGVPTIILEKNDKAGDSWRNRYERLYTHTLICMDHFPFMPFPTSWPTFSSKEQLGDFHEAYTKIMGLNYWCSSECCSATFDQKEGRWNVLVRSTKSSVMKELHPVHLIFATGGSGFPFVPKFRGAESFEGTQCHSSQYRRGSDWTGKQCVVIGACTSGHDICQDLWEQGAAQVTMVQKSSTCVIGIHSQYEHPNPEVMQDAPLATLERFEPMLFATPYKVMHRITIPITKKIAENDAEFYSDLAKVGFRTDFGEDGSGLLMKLLRRQGGYYIDMGASQLIIDGEIKVKSGVNVQEVRPKSILFDDGSEVPADLIVYATGYANMKEFIAKIISQETADKLGLFWGLGSNTTNDPGPWVGEHRNIFKPLSQPNLWMTGGSFSPTRMMSRYLSIQLKARFEGIQTPVYNDPASHRSS

>Rn_04775.1_1|Ricciocarpos_natans/1-605

MQRTIMESSLSQELITAWLSKFNDALLRKDISGVLELFLDEECFWRDFLVFTWNLHTCESKHEISMMLEETLDRTEVSSWEIDGSVTEGALEAPIKFQTPLARCRGYLKLKDGKCWVLLTTLWELKGHEEQAGPFRPQGVLYGSKKGRRTWIQEKKQEELELGHSKQPYCVIVGGGQAGIALGARLKALGVPTIILEKNDRAGDIWRNRFNTLSTHTPVWADHMPYMNFPSTWPLFSAKDQMADFFECYVKLMRLNYWCSSECKSASFDEKEGRWDVLVENKKSSSAIILHPVHLILATGCTGFANVPRFKGAESFEGVQLHSSKYHAGEEWQGKQCVVIGAGVSAHDICQDLWEQDAAQVTMVQNSSTCIVGQMTLYTPPPNENEINSSTSAVEVSDMQWFSIPYKLLTRFSIPFYEQIAKTDSEFYNRLTKAGFKIDFGEDGSGVIMKLFRTHGGYYIDVGASELIANGDIKVKSGVSVVEVKPKSILFSDGSEVPADLIVYATGYKNMKEVAAKLISQEVADKVGLIWGLGSDTRNDPGPWVGELKNMWKPLPHPNLWIMGGSFTQCRQMSRFLAIQLKARMEHIPTPVFWPDYNTRINTGT

>Klenit1_1_804_kfl00109_0340_v1.1|Klebsormidium_nitens/1-587

MATQTLTEDVRAWLERFSAALEESDLEKVLALFGDQCFWRDMVSFTWNVKTAEGKDEIRAMLEATLPSAAPASWRVEGEAMEMFGAVTAFVAFETATGRGKGHLRLVDGKCFTLLTTLYELKGFEEKIGASRLPGRNLAPEPNRQSWAAKRELERGELGYGKQPFVVIVGAGHSGVMLAARLRALNVPTIVVDKGSPGDSWRNRYGSLHLHTPSYMDNFPYLDYPAGWPTYPSKDEMGDWIEAYVKIMNVNVWSHSSVESSTFDDESKEWAVAVTRPDGKTLTLRPKHLVLCTGMYGDPRVPDFSGAEIFGGSQHHSSRHGCGGADFAGKKCVVIGTGTSGHDVAQDLWEKGADVTMVQRSPVFVIRSETLAAVYVDPLFHDSSPMSQQHADLLMVSTPYRLVAPFQAPMVEEIKRRDGDLHERLRERGFRVHFAEDGSGLAGLLNRTATGYYYNMGASYLIASGEIKVRSGGVEKVTERTVVLTDGSQVPADVIVYATGSRPVSEVVARVTSPEVAAKVGPLWGYGSGLDGDPGPWEGELRNIYKPTAQEGLWFHIGGLVQTRFGSLLVALQLKARFEGLPTPVYR

>Chabra1_323739_rna-CBR_g4485|Chara_Braunii/1-825

MMRVVFSTDLVLAAVFLLVVGGGCCFLPVRAVVEHSDSGDTSENTEKYPTPHDLARPHPPPNIWDCKVGPSCCMAVPSSRRIKGTPFVANLSEPMRTRMSIQTAFQDPSYVRKLTEAYRLMKMLDKEDPDDPRGMEQQRRVHCAYCSLGRSYGQSYEIHGNWFFLPWHRMFLYFHERILGEMISYPSFAIPFWNWDDDAGRRFPRAYSRSPSSPLYNPARNTDPCSMKRLVADATTTRMLRSIVLEASTQDLFFGQPLTALRTRQAGLIETGSHNAVHSAVGRQAFPFNDMGNVDWAGRDPIFYSHHANIDRLWSVWLRLPPGPSGPRRNPTHEAWLRSRFFFYDENAQLRTISPADVQNVEDLRYRYEEVPIRWLLQGELDKAINSQLDMKRTKTDSGRTRSSTAALNVPGRRLGQQPTSPRRPPSSSSFGGSDGGSDGGSDGERGGGEGGASVGDSFNLWKTMRWIENNRTDAGTEEKKEKQKLEEKEEGEGEEEEEREEEEREDEGRHEFIVKEERKTQMQMEEDTAVRHSQRGRGYQKATAASNARKLEEEGPPREEKAMAALSDDDRGVAAQTSSSSSSSSSFPPPPPPPPPPSSSSFSSSSSSSSSSSSSSSSLRNHDDDIDDDDNCPPLNSTLSDAFFEKAIGFAASRNRNGVVIGPITMSNSPLIVSLSGKYGCTSGPATEDLPLITFQAMRCPNHALSFDVFLSSRNSTAAAGSSPRPSGSASTSADGGKEQATRGTRHYVSTFYSMAYGREAPVTTWKIPIQGVFGDMQLAADGREVQIAVTLVPGPHLRQGRGGVGAGITFGNIGLQVPTCRTS
