# Supplementary material for: A plant‐centric view of class B flavin‐dependent monooxygenase evolution and diversity
Source: Plant J. 2026 Sep 9;127(5):e71106. doi: 10.1111/tpj.71106 (PMC13557801; doi:10.1111/tpj.71106)
Supplement: Supplementary file 3 [file TPJ-127-0-s010.docx]

>Aspi01Gene36448|Alsophila_spinulosa/1-417

M--MG-------------------MAHM--------------------------------

------------------------------------------------------------

------------------------------------------------------------

------------------------------------------------------------

------------------------------------------------------------

------------------------------------------------------------

------------------------------------------------------------

------------------------------------------------------------

------------------------------------------------------------

------------------------------------------------------------

------------------------------------------------------------

------------------------------------------------------------

------------------------------------------------------------

------------------------------------------------------------

------------------------------------------------------------

------------------------------------------------------------

------------------------------------------------------------

------------------------------------------------------------

------------------------------------------------------------

------------------------------------------------------------

------------------------------------------------------------

-----------------------AEVM---------------------------------

------------------------------------------------------------

------------------------------------------------------------

-------------CKESNDSNGGGAIVRVEDGVIIVGAGPAGLATAACLKQKG------I

ASVVLEKSDCIASLWR--------------------------------------------

------KRTYDRLCLHLPKQFCELPCMR----FSSSV-----------------------

--------------------------------PKFPTRQNFVAYLEEYASKCGE----RP

RFNETVSCASFDTSTS--------------------------------------------

---------------------MWHVLSH----EGARGR----------------------

----------------------------------------------------RDVVYCAP

WLVVASGENAE-PLLPQ--GLP----GLHNF---NGKSLHTSHY-KNGL------EFS--

-------G---QRVLVIGCGNSGMEVSLDLANF---------------------------

------------------------GAKPAVSVRSPVH-----VLPREI--YG--------

---------------ISTFSIAMTLTKLL-------------------------------

-PLRWTDRLLLLLSRLVLG-------DPSRHGF-----------KKPT-FGPLELKET-M

GKTPLLDIGTLTKIKDGSIRVVPA-----VQKLTH-SGCF---FKDGTYS----------

--------------------E-FDAVILATGYQ---SNMPSWLKVEPNCFNQ--------

-------------------------ACTGHWK--GKDGVFFAGF-SRKG------ILGIS

MDAQRIAADISK------------------------------------------------

------------------------------------------------------------

----------------------------------------------HYNSLK--------

------------------------------------------------------------

-----DA-----------------------------------------------------

------------------------------------------------------------

------------------------------------------------------------

------------------------------------------------------------

----------------------------KRKL----------------------------

------------------------------------------------------------

------------------------------------------------------------

------------------------------------------------------------

------------------------------------------------------------

------------------------------------------------------------

-----------------------HLHV---------------------------------

------------------------------------------------------------

------------------------------------------------------------

------------------------------------------------------------

------------------------------------------------------------

------------------------------------------------------------

------------------------------------------------------------

------------------------------------------------------------

------------------------------------------------------------

------------------------------------------------------------

------------------------------------------------------------

------------------------------------------------------------

------------------------------------------------------------

------------------------------------------------------------

------------------------------------------------------------

------------------------------------------------------------

------------------------------------------------------------

------------------------------------------------------------

------------------------------------------------------------

------------------------------------------------------------

------------------------------------------------------------

------------------------------------------------------------

------------------------------------------------------------

------------------------------------------------------------

------------------------------------------------------------

-----------------------------

>Pp6c3_9570|Ppatens_v6.1/1-434

------------------------MYPRK-------------------------------

------------------------------------------------------------

------------------------------------------------------------

------------------------------------------------------------

------------------------------------------------------------

------------------------------------------------------------

------------------------------------------------------------

------------------------------------------------------------

---------------------------------------------DSWKSG---------

------------------------------------------------------------

----------------------------------------------DLP-----------

------------------------------------------------------------

----------------------------------------VC------------------

------------------------------------------------------------

------------------------------------------------------------

------------------------------------------------------------

--------PSV-------------------------------------------------

------------------------------------------------------------

------------------------------------------------------------

-----------------------------------------------------------Q

-------------------------------------------LG---------------

--------------------SIAGESL---------------------------------

------------------------------------------------------------

------------------------------------------------------------

--------RNRSISSKVNSKEAPQ-------TVLIIGAGPAGLATAACLRSKY-----WI

PSIILERANCSAPLWRY-------------------------------------------

-------MTYDRLRMHLPKQFCQLPLRP----FPAVY-----------------------

--------------------------------PKYPTKNQFIAYLEDYQRHFGI----SP

VYNATVTSAEFSTALG--------------------------------------------

---------------------LWVVIAE----QKLEDNC---------------------

----------------------------------------------------ETVTYTTR

SLVVATGENAE-PYMP---DLF----GSHKF---HGVISHGSTY-RNGV------KYK--

-------D---MKVLVVGAGNTGMEISLDLAKF---------------------------

------------------------GAKPTLVARSKFH-----VMPRDL--FGLN------

---------------ISAFQVMLMLLKVL-------------------------------

-PVSFVDKLLVIFSRLTLG-------DTDHLNL-----------VRPK-EGPLKMKAR-T

GHTPVLDVGTVAEVRNGFIKVAPA-----IDQLTK-SGAR---FVNGVEE----------

--------------------E-FDAVIMATGYT---SNVYEWLKIDG-------------

----MS----------GINGFPKR-PFRNGWK--GGRGLYAVGF-GRKG------LMGCA

HDAELVADDIGA------------------------------------------------

------------------------------------------------------------

----------------------------------------------HHR-----------

------------------------------------------------------------

-----DNEKT--------------------------------------------------

------------------------------------------------------------

------------------------------------------------------------

------------------------------------------------------------

------------------------------------------------------------

------------------------------------------------------------

------------------------------------------------------------

------------------------------------------------------------

------------------------------------------------------------

------------------------------------------------------------

------------------------------------------------------------

------------------------------------------------------------

------------------------------------------------------------

--------------------------Y---------------------------------

------------------------------------------------------------

------------------------------------------------------------

------------------------------------------------------------

------------------------------------------------------------

------------------------------------------------------------

------------------------------------------------------------

------------------------------------------------------------

------------------------------------------------------------

------------------------------------------------------------

------------------------------------------------------------

------------------------------------------------------------

------------------------------------------------------------

------------------------------------------------------------

------------------------------------------------------------

------------------------------------------------------------

------------------------------------------------------------

------------------------------------------------------------

------------------------------------------------------------

------------------------------------------------------------

------------------------------------------------------------

------------------------------------------------------------

-----------------------------

>AT4G32540|Athaliana_Araport11/1-414

------------------------ME----------------------------------

------------------------------------------------------------

------------------------------------------------------------

------------------------------------------------------------

------------------------------------------------------------

------------------------------------------------------------

------------------------------------------------------------

------------------------------------------------------------

------------------------------------------------------------

------------------------------------------------------------

------------------------------------------------------------

------------------------------------------------------------

------------------------------------------------------------

------------------------------------------------------------

------------------------------------------------------------

------------------------------------------------------------

------------------------------------------------------------

------------------------------------------------------------

------------------------------------------------------------

------------------------------------------------------------

------------------------------------------------------------

------------------------------------------------------------

------------------------------------------------------------

------------------------------------------------------------

-------------SHPHNKTDQTQHIILVH-GPIIIGAGPSGLATSACLSSRG------V

PSLILERSDSIASLWK--------------------------------------------

------SKTYDRLRLHLPKHFCRLPLLD----FPEYY-----------------------

--------------------------------PKYPSKNEFLAYLESYASHFRI----AP

RFNKNVQNAAYDSSSG--------------------------------------------

---------------------FWRVKTH----DNTE------------------------

--------------------------------------------------------YLSK

WLIVATGENAD-PYFP---EIP----GRKKF--SGGKIVHASEY-KSGE------EFR--

-------R---QKVLVVGCGNSGMEISLDLVRH---------------------------

------------------------NASPHLVVRNTVH-----VLPREI--LG--------

---------------VSTFGVGMTLLKCL-------------------------------

-PLRLVDKFLLLMANLSFG-------NTDRLGL-----------RRPK-TGPLELKNV-T

GKSPVLDVGAMSLIRSGMIQIMEG-----VKEITK-KGAK---FMDGQEK----------

--------------------D-FDSIIFATGYK---SNVPTWLQ----------------

-GGDFF----------TDDGMPKT-PFPNGWR--GGKGLYTVGF-TRRG------LLGTA

SDAVKIAGEIGD------------------------------------------------

------------------------------------------------------------

----------------------------------------------QWR-----------

------------------------------------------------------------

-----DEIKGST------------------------------------------------

------------------------------------------------------------

------------------------------------------------------------

------------------------------------------------------------

-----------------------------RNM----------------------------

------------------------------------------------------------

------------------------------------------------------------

------------------------------------------------------------

------------------------------------------------------------

------------------------------------------------------------

------------------------------------------------------------

------------------------------------------------------------

------------------------------------------------------------

--------------------CSSRFVFTSKS-----------------------------

------------------------------------------------------------

------------------------------------------------------------

------------------------------------------------------------

------------------------------------------------------------

------------------------------------------------------------

------------------------------------------------------------

------------------------------------------------------------

------------------------------------------------------------

------------------------------------------------------------

------------------------------------------------------------

------------------------------------------------------------

------------------------------------------------------------

------------------------------------------------------------

------------------------------------------------------------

------------------------------------------------------------

------------------------------------------------------------

------------------------------------------------------------

------------------------------------------------------------

------------------------------------------------------------

------------------------------------------------------------

------------------------------------------------------------

-----------------------------

>Mapoly0020s0006|Mpolymorpha_v3.1/1-460

------------------------MK----------------------------------

------------------------------------------------------------

------------------------------------------------------------

------------------------------------------------------------

------------------------------------------------------------

------------------------------------------------------------

------------------------------------------------------------

------------------------------------------------------------

------------------------------------------------------------

------------------------------------------------------------

------------------------------------------------------------

------------------------------------------------------------

------------------------------------------------------------

------------------------------------------------------------

------------------------------------------------------------

------------------------------------------------------------

------------------------------------------------------------

------------------------------------------------------------

------------------------------------------------------------

------------------------------------------------------------

------------------------------------------------------------

------------------------K-----------------------------------

------------------------------------------------------------

------------------------------------------------------------

-------------------------------RVAVIGAGPSGLSLLRAFDSAAEKGAEIP

EIVCFEKQGEVGGLWKFEEGKGV-----------DEHGEIVH------------------

------GSMYRDLYINGPKEVNEYVDYS----FEEHY-----------------------

----------------------------GKVIGSYPPRPVLLSYIRGRAEKYNLCAKFSV

RFNSSVSKISYSEDTA--------------------------------------------

---------------------KFTVTVK----DRSTARN-------GTEA----------

----------------------------------------------------KVYSEDFD

HVVVAVGHFST-PNVP---QFP----GLEAF---KGSVLHAHDV-RDLS------EFR--

-------G---MDVLLVGSGYTAEDIACQCFKL---------------------------

-----------------------GAASVTITFRSNPT-----------------------

------------------------------------------------------------

-----------------------------------------GCCNWPE------------

-----------------SIKEVPLL----ERVDSNGRTCH---FKDGSSK----------

--------------------D-VDAIILCTGYL---HDFPFMVG-DE-------------

-----------LRLVAGNRMWPLG-LYQGVVLE-THPKVFYLGM-QAQF---YS-FTMFD

AQAWYVRDVIMG-RLTLP-SSTEEMVAHSRKWREAELAALAKL-----------------

---------------DVKPFQGDYVKELI--RHTD-------CPITP-------------

-------EFVDKTQQMGVDWDKHKALDI-----------------MSYR-----------

------------------------------------------------------------

-----D------------------------------------------------------

----------------------------------------------HAFVSAVTGNLART

---HHT-----------------------P------------------------------

-----------W------------------------------------------------

------------------------------------------------------------

-----MQNFD--------------------------------------------------

------------------------------------------------------------

------------------------------------------------------------

------------------------------------------------------------

------------------------------------------------------------

------------------------------------------------------------

------------------------------------------------------------

------------------------------------------------------------

-------------------DSLESYVN---------------------------------

------------------------------------------------------------

------------------------------------------------------------

------------------------------------------------------------

------------------------------------------------------------

------------------------------------------------------------

------------------------------------------------------------

------------------------------------------------------------

------------------------------------------------------------

------------------------------------------------------------

------------------------------------------------------------

------------------------------------------------------------

------------------------------------------------------------

------------------------------------------------------------

------------------------------------------------------------

------------------------------------------------------------

------------------------------------------------------------

------------------------------------------------------------

------------------------------------------------------------

------------------------------------------------------------

------------------------------------------------------------

------------------------------------------------------------

-----------------------------

>413958|Smoellendorffii_v1.0/1-446

------------------------ME----------------------------------

------------------------------------------------------------

------------------------------------------------------------

------------------------------------------------------------

------------------------------------------------------------

------------------------------------------------------------

------------------------------------------------------------

------------------------------------------------------------

------------------------------------------------------------

------------------------------------------------------------

------------------------------------------------------------

------------------------------------------------------------

------------------------------------------------------------

------------------------------------------------------------

------------------------------------------------------------

------------------------------------------------------------

------------------------------------------------------------

------------------------------------------------------------

------------------------------------------------------------

------------------------------------------------------------

------------------------------------------------------------

------------------------------------------------------------

------------------------------------------------------------

------------------------------------------------------------

------------------------K------RVAVIGAGASGLVAARELLREG------H

SVVIFEQARRIGGTWVYDPPPQESHSRSSNV----ARNHSCH------------------

------SSIYQSLRTNLPREVMGFLDYP----FVPRR-----------------------

---------------------------SSRDARRFPGHEEVLDYLESFTVEFGL--HGYV

RFNSKVKYVGLVES----------------------------------------------

---------------------NWIVKSS----SSAGK-----------EE----------

----------------------------------------------------KESEEVFD

AVVVCNGHYFQ-PRVA---QIP----GIEKW---PGKQYHSHTY-RTPD------LFK--

-------D---QVVAVIGNGPSGQDLCSDIAAD---------------------------

------------------------CKKVHWCAKLW-------NSPVE-------------

------------------------------------------------------------

------------------------------------------------------------

------------SLQQGKIQRHLMI----SKADKD-GRLH---FMDGTSA----------

--------------------V-VDAILHCTGYL---YDFSFLDT----------------

--KN-------YVKVEDNRVGP---LFKHVFPPALAPSLSFIGL-PWKT---IP-FPLSE

LQAKWISAVLKG-RASLP-PK-GEMANDVVGFYRTLEAQRVPNRHTHRLDL---------

-------------------EMFDYAEWLA--KQCG-------GTCHG-------------

--------FEPWRKELFLSTRDNRKLNS-----------------DSYR-----------

------------------------------------------------------------

-----DEWSDND------------------------------------------------

------------------------------------------------LHEKVVGVLATS

FFMREQ------------------------------------------------------

------------------------------------------------------------

------------------------------------------------------------

------------------------------------------------------------

------------------------------------------------------------

------------------------------------------------------------

------------------------------------------------------------

------------------------------------------------------------

------------------------------------------------------------

------------------------------------------------------------

------------------------------------------------------------

------------------------------------------------------------

------------------------------------------------------------

------------------------------------------------------------

------------------------------------------------------------

------------------------------------------------------------

------------------------------------------------------------

------------------------------------------------------------

------------------------------------------------------------

------------------------------------------------------------

------------------------------------------------------------

------------------------------------------------------------

------------------------------------------------------------

------------------------------------------------------------

------------------------------------------------------------

------------------------------------------------------------

------------------------------------------------------------

------------------------------------------------------------

------------------------------------------------------------

------------------------------------------------------------

------------------------------------------------------------

------------------------------------------------------------

------------------------------------------------------------

-----------------------------

>jgi|Selmo1|175034|estExt_Genewise1Plus.C_280315

------------------------ME----------------------------------

------------------------------------------------------------

------------------------------------------------------------

------------------------------------------------------------

------------------------------------------------------------

------------------------------------------------------------

------------------------------------------------------------

------------------------------------------------------------

------------------------------------------------------------

------------------------------------------------------------

------------------------------------------------------------

------------------------------------------------------------

------------------------------------------------------------

------------------------------------------------------------

------------------------------------------------------------

------------------------------------------------------------

------------------------------------------------------------

------------------------------------------------------------

------------------------------------------------------------

------------------------------------------------------------

------------------------------------------------------------

------------------------------------------------------------

------------------------------------------------------------

------------------------------------------------------------

------------------------K------RVAVIGAGASGLVAARELLREG------H

SVVIFEQARRIGGTWVY----EENHSGSSNA----ARNYSCH------------------

------SSMYESLRTNLPREVMGFLDYP----FVPRR-----------------------

---------------------------SSRDARRFPGHEEVLDYRESFAVEFGL--HGYV

RFNSKVKYVGLVES----------------------------------------------

---------------------NWIVKSS----SSAGK-----------EE----------

----------------------------------------------------KESEEVFD

AVVVCNGHYFQ-PRVA---QIP----GIERW---PGKHYHSHTY-RTPD------PFK--

-------D---QVVAVIGNGPSGEDLCSDIAAA---------------------------

------------------------CKKVHWCAKSW-------NSLSE-------------

------------------------------------------------------------

------------------------------------------------------------

------------PLQQGKIQRHLMI----SRADED-GRLH---FMDGMSA----------

--------------------V-VDVILHCTGYL---YNFSFLDT----------------

--KS-------YIKVEDNRVGP---LFKHVFPPALAPSLSFVGL-PCKT---IA-FPLSE

LQAKWIAAVLKG-RASLP-SK-GEMANEVVDFYRTLEAQGVPKRYTNHLYL---------

-------------------ETFDYAEWIA--KQCG-------GTCHG-------------

--------FEPWRKELCLSTLDKKKLNP-----------------GIYR-----------

------------------------------------------------------------

-----DEWNDND------------------------------------------------

------------------------------------------------FHEKVVAILAKK

---QQQ------------------------------------------------------

------------------------------------------------------------

------------------------------------------------------------

------------------------------------------------------------

------------------------------------------------------------

------------------------------------------------------------

------------------------------------------------------------

------------------------------------------------------------

------------------------------------------------------------

------------------------------------------------------------

------------------------------------------------------------

------------------------------------------------------------

------------------------------------------------------------

------------------------------------------------------------

------------------------------------------------------------

------------------------------------------------------------

------------------------------------------------------------

------------------------------------------------------------

------------------------------------------------------------

------------------------------------------------------------

------------------------------------------------------------

------------------------------------------------------------

------------------------------------------------------------

------------------------------------------------------------

------------------------------------------------------------

------------------------------------------------------------

------------------------------------------------------------

------------------------------------------------------------

------------------------------------------------------------

------------------------------------------------------------

------------------------------------------------------------

------------------------------------------------------------

------------------------------------------------------------

-----------------------------

>AT1G12160|Athaliana_Araport11/1-468

------------------------MT----------------------------------

------------------------------------------------------------

------------------------------------------------------------

------------------------------------------------------------

------------------------------------------------------------

------------------------------------------------------------

------------------------------------------------------------

------------------------------------------------------------

------------------------------------------------------------

------------------------------------------------------------

------------------------------------------------------------

------------------------------------------------------------

------------------------------------------------------------

------------------------------------------------------------

------------------------------------------------------------

------------------------------------------------------------

------------------------------------------------------------

------------------------------------------------------------

------------------------------------------------------------

------------------------------------------------------------

------------------------------------------------------------

------------------------------------------------------------

------------------------------------------------------------

------------------------------------------------------------

----------------SVITSRAR-------HVAVIGLGAAGLVAVRELRREG------H

TVIGFEREKHVGGLWVYTDRVDS-DSVSVD-----PDRTIVH------------------

------SSIYQSLRTNLPRECMGYSDFP----FVTRS-----------------------

---------------------------SDGDPRRYPDHREVLMYLQDFAKEFKI--EDMI

RFETEVLCVEPSPENN--------------------------------------------

--------------------RKWRVQFK----SSNG------------------------

----------------------------------------------------VSGEEIFD

AVVVCNGHFTE-PRLA---HIP----GIESW---PGKQIHSHNY-RIPD------PFK--

-------D---EVVIVIGSQASGNDISTDIATI---------------------------

------------------------AKEVHISSKMV-------ASDSY-------------

------------------------------------------------------------

------------------------------------------------------------

-------------GCYDNLRIHPTI----YRARED-GSVV---FRNGKVV----------

--------------------F-ADAIVHCTGYK---YHFPFLKT----------------

--SG-------YVTVEDNRVGP---LYKHVFPPALAPGISFIGL-PFMG---LQ-FFMFE

IQSKWVASVLSG-RVKLP-AE-DKMMEEAVAFYSKLEDLGIPKRYTHFLTDPR-GNPMLG

TFKP---------EDAVVISQSDYFNWIA--KQCG---------CTS-------------

--------IERWRERLYNVAIKKVFFGG-----------------DSYR-----------

------------------------------------------------------------

-----DRWDDDQ------------------------------------------------

------------------------------------------------LIEEVYREFAKL

------------------------------------------------------------

------------------------------------------------------------

------------------------------------------------------------

------------------------------------------------------------

------------------------------------------------------------

------------------------------------------------------------

------------------------------------------------------------

------------------------------------------------------------

------------------------------------------------------------

------------------------------------------------------------

------------------------------------------------------------

------------------------------------------------------------

------------------------------------------------------------

------------------------------------------------------------

------------------------------------------------------------

------------------------------------------------------------

------------------------------------------------------------

-------------KPNQDCS----------------------------------------

------------------------------------------------------------

------------S-----------------------------------------------

------------------------------------------------------------

------------------------------------------------------------

------------------------------------------------------------

------------------------------------------------------------

------------------------------------------------------------

------------------------------------------------------------

------------------------------------------------------------

------------------------------------------------------------

------------------------------------------------------------

------------------------------------------------------------

------------------------------------------------------------

------------------------------------------------------------

------------------------------------------------------------

-----------------------------

>Poptr1_1|eugene3.00130182

------------------------MQ----------------------------------

------------------------------------------------------------

------------------------------------------------------------

------------------------------------------------------------

------------------------------------------------------------

------------------------------------------------------------

------------------------------------------------------------

------------------------------------------------------------

------------------------------------------------------------

------------------------------------------------------------

------------------------------------------------------------

------------------------------------------------------------

------------------------------------------------------------

------------------------------------------------------------

------------------------------------------------------------

------------------------------------------------------------

------------------------------------------------------------

------------------------------------------------------------

------------------------------------------------------------

------------------------------------------------------------

------------------------------------------------------------

------------------------------------------------------------

------------------------------------------------------------

------------------------------------------------------------

---------------ASPNLLSSH-------HVAVIGAGAAGLVAARELHREG------H

KVVVFEKDDQVGGLWMYDPRVEP-DPLGLD-----LTRPVVH------------------

------SSLYESLRTNLPRETMGFMDYP----FVTRE-----------------------

--------------------------GEGRDPRRFPGHREVLMYLQDYAREFGI--EEMV

RFGCEVVNVEMIDSG---------------------------------------------

---------------------KWKVKSK----RKRLDD--------NDRG----------

--------------------------------------------------DDFADHEDFD

AVVVCVGHYTQ-PRIA---EIP----GINLW---PGKQIHSHNY-RIPE------PFR--

-------D---QIIILIGASASAADISVEIAGL---------------------------

------------------------AKEVHIARRSA-------VDDDTY------------

------------------------------------------------------------

------------------------------------------------------------

----------EKKPGYDNIWLHSTI----ERACED-GTVI---FRDGSVI----------

--------------------L-ADVILHCTGYK---YGFPFLKT----------------

--DG-------IVTVDDNRVGP---LYKHVFPPILAPWLSFVGI-PYWT---FP-FPTFE

VQSKWIAGVLSG-RIALP-SQ-EDMVEDVKIYYSELEASGVPKHHTHNLAH---------

-------------------STNDYNMWLA--SQCQ---------CSC-------------

--------FEEWRIEMSHEILKNWRARP-----------------NMYR-----------

------------------------------------------------------------

-----DEWDDDH------------------------------------------------

------------------------------------------------LILQAHEDFNRR

------------------------------------------------------------

------------------------------------------------------------

------------------------------------------------------------

------------------------------------------------------------

------------------------------------------------------------

------------------------------------------------------------

------------------------------------------------------------

------------------------------------------------------------

------------------------------------------------------------

------------------------------------------------------------

------------------------------------------------------------

------------------------------------------------------------

------------------------------------------------------------

------------------------------------------------------------

------------------------------------------------------------

------------------------------------------------------------

------------------------------------------------------------

-------------ISNKASN----------------------------------------

------------------------------------------------------------

------------GHI---------------------------------------------

------------------------------------------------------------

------------------------------------------------------------

------------------------------------------------------------

------------------------------------------------------------

------------------------------------------------------------

------------------------------------------------------------

------------------------------------------------------------

------------------------------------------------------------

------------------------------------------------------------

------------------------------------------------------------

------------------------------------------------------------

------------------------------------------------------------

------------------------------------------------------------

-----------------------------

>jgi|Meskra658_1|1799451|fgenesh1_kg.Chr_24_#_1028_#_TRINITY_DN72_c2_g1_i3

------------------------MHSLST------------------------------

------------------------------------------------------------

----YL------------------------------------------------------

------------------------------------------------------------

------------------------------------------------------------

------------------------------------------------------------

------------------------------------------------------------

------------------------------------------------------------

------------------------------------------------------------

------------------------------------------------------------

------------------------------------------------------------

------------------------------------------------------------

--------------------------HSPSHLAF--------------------------

---------------------------------------TVSSSSKVK------------

------------------------------------------------------------

--------------GHLTKRS-----------KSGVKGS-------------------LP

HSQRGLRLPK--------------------------------------------------

------------------------------------------------------------

------------------------------------------------------------

------------------------------------------------------------

------------------------------------------------------------

--------------------FRPGQVFCK----------PY-------------------

------------------------VSKSKCRCRCGIGCVESSWSMEVSTEIST---RCQS

NF-----------------------VKVPGDFAEAAKVDKGGNSTCR----KIISTAAFN

HETSPVPSEGQQPMELEQNRKPGL-------RVAVIGAGAAGLAAARELLREG------H

QVEVFEQGSEVGGVWVYDDLTES-DPLGLS-----SSREAVH------------------

------SSMYASLRTNLPRECMSFLDFP----FTAPP-----------------------

---------------------------GSSDSSRYCTHQTVCLYLQHFAEVFGL--LPHV

RFRSRVEKVTPEHPVSRQFPAESRP--GEAQALSSSCNGVGLANQDAGKAAEDFIGRARD

SIEEG-ELLSASPTAKRNHWIQWRVETS----RVTSSESNIDDAN-GGENKG------LS

LN-------------------------SQACREGV----------TSSERGRMDKTEVYD

AVVICNGHYSQ-PKIA---SVP----GIETW---PGRQIHSHNY-RRPE------PFT--

-------G---LVVVILGAASSGEDIARETAGT---------------------------

------------------------AREVHLCSRGW-------TAEEVP------------

------------------------------------------------------------

------------------------------------------------------------

---------GSPEGKHRNLWRHPMV----VKAHSD-GRVD---FGDGSNV----------

--------------------H-ADVILHCTGYV---YHFPFLRG--V-------------

--EP-------WFGVEDARVGP---LYEHVFPLHLAPTLSLVGI-PWQV---AP-FPLME

IQAKWIAKVLAG-KITLP-SP-QEMEAEVQSFYGQMEAAGVPQRYSHAMGS---------

---------------TAVVGQFEYLDGIL--ERCG---------LPP-------------

--------LDRWRRGMYAAASLNKRSNP-----------------DNYR-----------

------------------------------------------------------------

-----ETWTDYD------------------------------------------------

------------------------------------------------ILAEAVASQAKI

------------------------------------------------------------

------------------------------------------------------------

------------------------------------------------------------

------------------------------------------------------------

------------------------------------------------------------

------------------------------------------------------------

------------------------------------------------------------

------------------------------------------------------------

------------------------------------------------------------

------------------------------------------------------------

------------------------------------------------------------

------------------------------------------------------------

------------------------------------------------------------

--------------------------------------------------------LEAN

PHW---------------------------------------------------------

------------------------------------------------------------

------------------------------------------------------------

------------------------------------------------------------

------------------------------------------------------------

------------------------------------------------------------

------------------------------------------------------------

------------------------------------------------------------

------------------------------------------------------------

------------------------------------------------------------

------------------------------------------------------------

------------------------------------------------------------

------------------------------------------------------------

------------------------------------------------------------

------------------------------------------------------------

------------------------------------------------------------

------------------------------------------------------------

------------------------------------------------------------

------------------------------------------------------------

-----------------------------

>jgi|Meskra657_3|3031339|fgenesh1_kg.23_#_1670_#_TRINITY_DN5806_c0_g1_i8

MC-------------------PCLMHSLST------------------------------

------------------------------------------------------------

----YL------------------------------------------------------

------------------------------------------------------------

------------------------------------------------------------

------------------------------------------------------------

------------------------------------------------------------

------------------------------------------------------------

------------------------------------------------------------

------------------------------------------------------------

------------------------------------------------------------

------------------------------------------------------------

--------------------------HSPSHLAF--------------------------

---------------------------------------TVSSSSKVK------------

------------------------------------------------------------

--------------GHLTKLS-----------KSGVKGS-------------------LP

HSQRGLRLPK--------------------------------------------------

------------------------------------------------------------

------------------------------------------------------------

------------------------------------------------------------

------------------------------------------------------------

--------------------FSPGQVFCK----------PY-------------------

------------------------VSKSKCRCRCGVGCVESSWSMEVSTEIST---RCQS

VF-----------------------VKVPGDFAEAAKVDKGGNSTCR----KIFSTAAFN

HETSPVPSEGQQPMELEQNRKPGL-------RVAVIGAGAAGLAAARELLREG------H

QVEVFEQGSEVGGVWVYDDLTES-DPLGLS-----SSREAVH------------------

------SSMYASLRTNLPRECMSFLDFP----FTAPP-----------------------

---------------------------GSSDSSRYCTHQTVCLYLQHFAEVFGL--LPHV

RFRSRVEKVTPEHPVSRQFPAESRP--GEAQALSSSCNGVGLANQDAGKAAQDFIGRARD

SIEEG-ELLSASPTPKRNHWIRWRVETS----RVTSSESNIDDAN-GGENKG------LS

LN-------------------------SQACREGV----------TSSERGRMDKTEVYD

AVVICNGHYSQ-PKIA---SIP----GIETW---PGRQIHSHNY-RRPE------PFT--

-------G---LVVVIVGAASSGEDIARETAGT---------------------------

------------------------AREVHLCSRGW-------TAEEVP------------

------------------------------------------------------------

------------------------------------------------------------

---------GGPEGKHRNLWRHPMV----VKAHSD-GRVD---FGDGSNV----------

--------------------H-ADVILHCTGYV---YHFPFLRG--V-------------

--EP-------WFGVEDARVGP---LYEHVFPLHLAPTLSLVGI-PWQV---AP-FPLME

IQAKWIAKVLAG-KVTLP-SP-QEMEAEVQSFYGQMEAAGVPQRYSHAMGS---------

---------------TAVVGQFEYLDGIL--ERCG---------LPP-------------

--------LDSWRRGMYAAASLNKRSNP-----------------DNYR-----------

------------------------------------------------------------

-----ETWTDYD------------------------------------------------

------------------------------------------------ILAEAVASQAKI

------------------------------------------------------------

------------------------------------------------------------

------------------------------------------------------------

------------------------------------------------------------

------------------------------------------------------------

------------------------------------------------------------

------------------------------------------------------------

------------------------------------------------------------

------------------------------------------------------------

------------------------------------------------------------

------------------------------------------------------------

------------------------------------------------------------

------------------------------------------------------------

--------------------------------------------------------LEAH

PHWL------------------------------------------------KEASLPL-

------------------------------------------------------------

------------------------------------------------------------

------------------------------------------------------------

------------------------------------------------------------

------------------------------------------------------------

------------------------------------------------------------

------------------------------------------------------------

------------------------------------------------------------

------------------------------------------------------------

------------------------------------------------------------

------------------------------------------------------------

------------------------------------------------------------

------------------------------------------------------------

------------------------------------------------------------

------------------------------------------------------------

------------------------------------------------------------

------------------------------------------------------------

------------------------------------------------------------

-----------------------------

>jgi|Zygcyl6981a_1|43428|Zci1a_00166.1

------------------------MS----------------------------------

------------------------------------------------------------

------------------------------------------------------------

------------------------------------------------------------

------------------------------------------------------------

------------------------------------------------------------

------------------------------------------------------------

------------------------------------------------------------

------------------------------------------------------------

------------------------------------------------------------

------------------------------------------------------------

------------------------------------------------------------

------------------------------------------------------------

---------------------------------------SVAIAPLL-------------

------------------------------------------------------------

------------------------------------------------------------

--VARVRSPL--------------------------------------------------

------------------------------------------------------------

------------------------------------------------------------

------------------------------------------------------------

------------------------------------------------------------

---------------------------------------PL-------------------

------------------------------------------------------------

---------------------------------------------------SSFQLSSSN

VRGAMAVSPHASVMTSAGSHGKTL-------KVAVVGAGAAGLVAARELLREG------H

HVAVFEQSSEVGGTWNYSEAIDS-DPISQD-----SSRTRVH------------------

------SSMYASLRTNLPRECMGFLDFP----LVPAL-----------------------

-------------------------TPGSEDSRRYCSHEAVALYLQHFARVFEI--VPHI

RFRTQVDFVEPLPMKS-------------------SHNSLQLRNATTDKNGEE-------

--------------ESKEEWLKWRVGSS----SASILDE----HD-SGLN----------

--------------------------------SSV----------DKDEVEEKSEEEIYD

AVFVCNGHYSE-PKVA---AIP----GLEEW---PGRQIHSHNY-RRPE------PFS--

-------G---LTVVVVGAGSSGEDLAREVAMV---------------------------

------------------------SREVHMCARSW-------SPSENG------------

------------------------------------------------------------

---------------------------------------------SPS------------

----------RPDGPRGNLWRHPMA----VRAFPD-GRIE---FADGFSV----------

--------------------V-ADVVLHCTGYT---FSFPFLRN--V-------------

--ES-------FLEVSDARIHP---LYEHVFPPSLAPSLSFVGI-PWQV---AP-FPLME

LQSTWIAKILSG-KVELP-SK-EAMDSDVQAFYERLEEAKIPKRYTHAMGK---------

---------------TAIIQQFEYLDGLL--SRCG---------LPP-------------

--------VENWRKEMYSLGGFNKQSNP-----------------DSYR-----------

------------------------------------------------------------

-----DTWQEAD------------------------------------------------

------------------------------------------------VALEAATAQEAQ

------------------------------------------------------------

------------------------------------------------------------

------------------------------------------------------------

------------------------------------------------------------

------------------------------------------------------------

------------------------------------------------------------

------------------------------------------------------------

------------------------------------------------------------

------------------------------------------------------------

------------------------------------------------------------

------------------------------------------------------------

------------------------------------------------------------

------------------------------------------------------------

--------------------------------------------------------LKAL

EKR---------------------------------------------------------

------------------------------------------------------------

------------------------------------------------------------

------------------------------------------------------------

------------------------------------------------------------

------------------------------------------------------------

------------------------------------------------------------

------------------------------------------------------------

------------------------------------------------------------

------------------------------------------------------------

------------------------------------------------------------

------------------------------------------------------------

------------------------------------------------------------

------------------------------------------------------------

------------------------------------------------------------

------------------------------------------------------------

------------------------------------------------------------

------------------------------------------------------------

------------------------------------------------------------

-----------------------------

>jgi|Zygcir6981b_2|15954|Zci_06976.1

------------------------------------------------------------

------------------------------------------------------------

------------------------------------------------------------

------------------------------------------------------------

------------------------------------------------------------

------------------------------------------------------------

------------------------------------------------------------

------------------------------------------------------------

------------------------------------------------------------

------------------------------------------------------------

------------------------------------------------------------

------------------------------------------------------------

------------------------------------------------------------

------------------------------------------------------------

------------------------------------------------------------

------------------------------------------------------------

------------------------------------------------------------

------------------------------------------------------------

------------------------------------------------------------

------------------------------------------------------------

------------------------------------------------------------

------------------------------------------------------------

------------------------------------------------------------

------------------------------------------------------------

------------------HGSHSL-------KVAVIGAGAAGLVAARQLLREG------H

HVAVLEQGSRVGGTWQYEERAES-DPLSRA-----PSRERVH------------------

------GSMYASLRTNLPREVMAFRDFP----LSVSV-----------------------

----------------------PVPVAVAVDGRRYCSHGTVARYLERFAEVFGV--LPHV

RFGRRVEWV---------------------------------------------------

---------------------KWRVASR----EVT-------------FG----------

----------------------------------------------------SAVEEVYD

AVVVCNGHFSE-PKIA---DIA----GLSEW---PGRQMHSHNY-RRPE------PFA--

-------G---LVVVVVGAGSSGEDIAREAAMA---------------------------

------------------------AREVHMCARSWPGAGGGGSPSPTP------------

------------------------------------------------------------

---------------------------------------------SPS------------

-------PSPSPGGKYKNLWRHPMA----VRAHAD-GKVE---FADGVSV----------

--------------------V-ADVILHCTGYM---YHFPFLRG--I-------------

--DH-------CVKVSDSRVGP---LFEHVFPPTLAPTLSFVGL-PWQV---AP-FPLME

IQSTWIAKVLSG-SVGLP-SR-EEMESEIETFYKRMEEAGLPQRFTHSMGK---------

---------------TAVVGQFEYLDGIC--ARCG---------ILP-------------

--------VEQWRREMYALGSLNKQNNP-----------------DEYR-----------

------------------------------------------------------------

-----DMWPESS------------------------------------------------

-----------------------------------------------LVEGEVAAAQAAQ

------------------------------------------------------------

------------------------------------------------------------

------------------------------------------------------------

------------------------------------------------------------

------------------------------------------------------------

------------------------------------------------------------

------------------------------------------------------------

------------------------------------------------------------

------------------------------------------------------------

------------------------------------------------------------

------------------------------------------------------------

------------------------------------------------------------

------------------------------------------------------------

--------------------------------------------------------LEAL

RAL-------------------------------------------------TDTVSIS-

------------------------------------------------------------

---------------------------------------------------------GL-

------------------------------------------------------------

------------------------------------------------------------

------------------------------------------------------------

------------------------------------------------------------

------------------------------------------------------------

------------------------------------------------------------

------------------------------------------------------------

------------------------------------------------------------

------------------------------------------------------------

------------------------------------------------------------

------------------------------------------------------------

------------------------------------------------------------

------------------------------------------------------------

------------------------------------------------------------

------------------------------------------------------------

------------------------------------------------------------

-----------------------------

>jgi|Myrbis2043_1|636|KAK9830413.1

------------------------MQ----------------------------------

------------------------------------------------------------

------------------------------------------------------------

------------------------------------------------------------

------------------------------------------------------------

------------------------------------------------------------

------------------------------------------------------------

------------------------------------------------------------

------------------------------------------------------------

------------------------------------------------------------

------------------------------------------------------------

------------------------------------------------------------

------------------------------------------------------------

------------------------------------------------------------

------------------------------------------------------------

--------------------------------RPG-------------------------

--VPALHKPRLL------------------------------------------------

------------------------------------------------------------

------------------------------------------------------------

------------------------------------------------------------

------------------------------------------------------------

------------------------------------------------------------

------------------------------------------------------------

-------------------------------------------------------VAWAR

VRRVMAPSAALQSASLAPQGPGQK-------SVAVIGGGAAGLVAARELLRES------H

CVTVFEQGPGLGGVWKYDPQTEQDDPLGLR-----PDRQRVH------------------

------SSMYARLRTNLPREVMAFLDFP----FDALT-----------------------

------------------------MQGRSSDPRRFCGHEEVQRYLEAFAEAFAL--EQHM

RFNMRVQQLTPLPHSQ-----------------LDSANGLDSTEQ---------------

GCPRG-------------QAAIWNGASN----ETSSNG--------ASTG----------

----------------------------------------------------REYSQEFD

AVVVCNGHYSQ-PRLP---DVA----GAAAF---PGRQLHSHNY-RENS------AFA--

-------G---QAVVVVGASASGEDICREVAEV---------------------------

------------------------ADKVYLCARSW-------QNPAWT------------

------------------------------------------------------------

------------------------------------------------------------

-------HETTPIGQRANIWRRPMV----QALGAN-GSVT---FTDGQTL----------

--------------------ERVDCILYCTGYQ---YAFPFLED----------------

--AG-------VVSIEDNRVSP---LFQHIFPP-TAPSLAFVGL-PWRV---VP-FPQME

LQSKWVARVLSG-RVSLP-AA-VDMQRWVDDRYRELDAKQVPVRYTHYQGD---------

-------------------AQWAYNDWLA--EACG-------PDVPK-------------

--------LPSWREAMYKATGNNKRDFP-----------------EKYR-----------

------------------------------------------------------------

-----DVWRDEG------------------------------------------------

------------------------------------------------LVEQAAVDARKS

------------------------------------------------------------

------------------------------------------------------------

------------------------------------------------------------

------------------------------------------------------------

------------------------------------------------------------

------------------------------------------------------------

------------------------------------------------------------

------------------------------------------------------------

------------------------------------------------------------

------------------------------------------------------------

------------------------------------------------------------

------------------------------------------------------------

------------------------------------------------------------

--------------------------------------------------------RSRP

QHAV------------------------------------------------VEGVAAA-

------------------------------------------------------------

------------------------------------------------------------

------------------------------------------------------------

------------------------------------------------------------

------------------------------------------------------------

------------------------------------------------------------

------------------------------------------------------------

------------------------------------------------------------

------------------------------------------------------------

------------------------------------------------------------

------------------------------------------------------------

------------------------------------------------------------

------------------------------------------------------------

------------------------------------------------------------

------------------------------------------------------------

------------------------------------------------------------

------------------------------------------------------------

------------------------------------------------------------

-----------------------------

>jgi|Cosub3|1196263|fgenesh1_kg.4_#_571_#_HTPTB_transcript/17940

------------------------MH----------------------------------

------------------------------------------------------------

------------------------------------------------------------

------------------------------------------------------------

------------------------------------------------------------

------------------------------------------------------------

------------------------------------------------------------

------------------------------------------------------------

------------------------------------------------------------

------------------------------------------------------------

------------------------------------------------------------

------------------------------------------------------------

------------------------------------------------------------

------------------------------------------------------------

------------------------------------------------------------

------------------------------------------------------------

--VAAQRSPR--------------------------------------------------

------------------------------------------------------------

------------------------------------------------------------

------------------------------------------------------------

------------------------------------------------------------

--------------------FFLSKVLG-----------PR-------------------

------------------------------------------------------------

--------------------------IVPTEY------------------------RSLV

IFGAQKLTTRAATMNGRDHTTESL-------KVAVIGAGAAGLVTARELRREG------H

EPVIFEQGSKVGGVWVYTDKVEEPHGASSRI---GAAEERVH------------------

------SSMYAGLRTNLPREVMSYTDFP----FTRSW-----------------------

-----------------------------GDTRRFCGHAEVEAYLEAFAAAFDL--EKYI

RFNTPVLSLTPCEAGS---PPRARL--GSDT---APTNGHQAGHQREAMPGTEEDGKGLV

GLPNGHAAVNGTSNSRELPWPRWRMTTA----PTGDQ---------GDVS----------

----------------------------------------------------TSGDEVFD

AVIVCNGHYSD-PRRP---DIP----GMAEF---PGRLLHSHSY-RRNE------PFE--

-------G---MTVVVIGASASGEDISREIAHV---------------------------

------------------------ADKVYLCARSW-------QNPAWA------------

------------------------------------------------------------

------------------------------------------------------------

------AETVEPFGARRNIWRRGVP----SRLHPD-GGVT---FQGGKRV----------

--------------------DAVDVVMFATGYC---YSFPFLADTRID------------

-GAE-------IVTVEDNRVSP---LYKHIFPPSTAPTLSFVGL-PWKV---VP-FAQYE

LQAKWIARVLSA-RVSLP-GQ-RHMLADISAFYADLDKEGVPKRHTHMLGD---------

-------------------KQWEYNDWLA--AACG-------PDVTP-------------

--------LPQWRPKMYKVAGDTKRAVP-----------------ETYR-----------

------------------------------------------------------------

-----DVWPEAE------------------------------------------------

------------------------------------------------LLAEAAAEASRL

---GAPV-----------------------------------------------------

------------------------------------------------------------

------------------------------------------------------------

------------------------------------------------------------

------------------------------------------------------------

------------------------------------------------------------

------------------------------------------------------------

------------------------------------------------------------

------------------------------------------------------------

------------------------------------------------------------

------------------------------------------------------------

------------------------------------------------------------

------------------------------------------------------------

------------------------------------------------------------

------------------------------------------------------------

------------------------------------------------------------

------------------------------------------------------------

------------EIQNQIAK----------------------------------------

------------------------------------------------------------

------------TTVV--------------------------------------------

------------------------------------------------------------

------------------------------------------------------------

------------------------------------------------------------

------------------------------------------------------------

------------------------------------------------------------

------------------------------------------------------------

------------------------------------------------------------

------------------------------------------------------------

------------------------------------------------------------

------------------------------------------------------------

------------------------------------------------------------

------------------------------------------------------------

------------------------------------------------------------

-----------------------------

>jgi|Klenit1_1|1302|kfl00117_0190_v1.1

------------------------MT----------------------------------

------------------------------------------------------------

------------------------------------------------------------

------------------------------------------------------------

------------------------------------------------------------

------------------------------------------------------------

------------------------------------------------------------

------------------------------------------------------------

------------------------------------------------------------

------------------------------------------------------------

------------------------------------------------------------

------------------------------------------------------------

------------------------------------------------------------

----------------------------------------VDVAA---------------

------------------------------------------------------------

------------------------------------------------------------

------------------------------------------------------------

------------------------------------------------------------

------------------------------------------------------------

------------------------------------------------------------

------------------------------------------------------------

--------------------------N---------------------------------

------------------------------------------------------------

------------------------------------------------------------

-------------GKEGQKTRVSR-------SVCVIGAGAAGLVALKELREEG------H

QVTAFEQNEDIGGVWKYDPDTDD-DPLGIR-----EDRKKVH------------------

------SSMYASLRTNLPREIMSFAAYP----FVPSG-----------------------

--------------------------EPGRDDRRFCSHQEVQAYLRDYARHFDL--HRHI

RFGTQVLRVSRLPEKT---------------------NQYGSVQQ---------------

--------------------PLWEVVTR----TASSS-----------ENW---------

----------------------------------------------------ACDTTEFD

AVVVCNGHYSQ-PKLA---TIE----GIASW---PGRQLHSHNY-RTPG------PFA--

-------G---QTVVLIGAFASGEDISRELAEH---------------------------

------------------------CSEVYLSARSW-------KDEPR-------------

------------------------------------------------------------

------------------------------------------------------------

-------------QTPPNLHRKLVV----TRAYPD-GSLE---FADGSRV----------

--------------------V-ADVIIHCTGYV---YEFSFLES----------------

---G-------LVSTEDQRVHP---VYEQVFHVGTAPTLSFIGL-PFKV---SP-LPMFE

LTTRWVAQCLSG-RAVLP-SE-AAMHKWIDGFYEGLKAGGVPVRYTHEMSR---------

-------------------TGFDYLARLC--EWTG---------TPM-------------

--------FESWREDLFKDFSYRRHNYP-----------------ANYR-----------

------------------------------------------------------------

-----DEWPEDLELK---------------------------------------------

---------------------------------------------RKVIWEDLRKEAEKL

---SL-------------------------------------------------------

------------------------------------------------------------

------------------------------------------------------------

------------------------------------------------------------

------------------------------------------------------------

------------------------------------------------------------

------------------------------------------------------------

------------------------------------------------------------

------------------------------------------------------------

------------------------------------------------------------

-------------------------------------------------------GG---

--------------------SEKSFGS---------------------------------

------------------------------------------------------------

------------------------------------------------------------

------------------------------------------------------------

------------------------------------------------------------

------------------------------------------------------------

------------------------------------------------------------

------------------------------------------------------------

------------------------------------------------------------

------------------------------------------------------------

------------------------------------------------------------

------------------------------------------------------------

------------------------------------------------------------

------------------------------------------------------------

------------------------------------------------------------

------------------------------------------------------------

------------------------------------------------------------

------------------------------------------------------------

------------------------------------------------------------

------------------------------------------------------------

------------------------------------------------------------

------------------------------------------------------------

-----------------------------

>jgi|Coprin2167_1|3429|KAK9903581.1

------------------------ME----------------------------------

------------------------------------------------------------

------------------------------------------------------------

------------------------------------------------------------

------------------------------------------------------------

------------------------------------------------------------

------------------------------------------------------------

------------------------------------------------------------

------------------------------------------------------------

------------------------------------------------------------

------------------------------------------------------------

------------------------------------------------------------

------------------------------------------------------------

---------------------------------------IVDVRPI--------------

------------------------------------------------------------

------------------------------------------------------------

------------------------------------------------------------

------------------------------------------------------------

------------------------------------------------------------

------------------------------------------------------------

------------------------------------------------------------

------------------------------------------------------------

------------------------------------------------------------

------------------------------------------------------------

-------------SSDSSRSLSAR-------KVAVIGAGSAGLVAARELSKEG------H

QVQVFEQGQSIGGIWCYQDEVED-DLLGKC-----PGRRKVH------------------

------SSLYSSLRVNLPREIMSFSDFP----FTPEV-----------------------

------------------------MGGRSQDRRRYPHHTEVLGYLEAFVQEFDL--RPLI

RFGTRVVDVSPVTGLE------------GRP---EAPNGLPSNGR---------------

------EICSAN---------HWIVKSE----LLPTAD--------LQVP----------

----------------------------------------------------EVHAEVFD

AVIVAVGNYHE-PNLP---DVE----GMEDF---PGLQMHCHNF-RHNE------VFT--

-------N---KTVVVVGASFSGEEIARQIADV---------------------------

------------------------ALHVYHSARTW-------SKQLT-------------

------------------------------------------------------------

------------------------------------------------------------

-----------EPKGRANLTRMPML----TRLGSN-GRAD---FFGGVSA----------

--------------------DSVDAVVYCTGYQ---YSYPFLKR----------------

--TG-------LICTDDNRVTP---LYRHIFVPSVAPTLAFIGL-LWKS---LR-NSQFE

LQAKWVAQVLSG-RVALP-TQ-QEMEADMEAFYELLKEKGIPVRYTHCQND---------

---------------AMPVSQWEYNNLLR--ESCS-------PVPPP-------------

--------AEAWRARLHDAISTKILESP-----------------DTFR-----------

------------------------------------------------------------

-----DEHSAEE------------------------------------------------

----------------------------------------------QALFDDAAQACAEM

---ARPFTGMPIMDRGLLQSGGLSFNTQDGSAVNDLLQLL--------------------

---LNNDPRAAQLIKQDAQ-------------------------------------Q--G

------------------RTG-----AIATAL------------------ATASATGNGQ

AAATAVSQAV--------------------------------------------------

---------------------------------AQGAPASAVAKAVAAATSNAP------

----------------------------------V-----------GAPSQALPPA----

----------------------------------SPGLEN--------------------

---------------------------------------------------AAASGNTQA

----------------------------IASAL---------------------------

------------------------------------------------------------

----A-----------------------------------------------VSTGG---

--------------------KVESVASA--------------------------------

------------------------------------------------------------

---------------------------------------------------NAQTFTQAE

NAISQA-------------------------V--------------------TTAVAQVK

----------------GGGNATAAALSAATAI------------------GNATAEAFAS

ASAKTTVQG---------------------------------------------KGLGA-

---------ADASATAAATS----------------------------------------

------------------------------------------------------------

------------TAV---------------------------------------------

------------------------------------------------------------

------------------------------------------------------------

------------------------------------------------------------

------------------------------------------------------------

----EHAIATAIAAATNNNAQA-------LS-----------------------------

------------------------------------------------------------

------------------------------------------------------------

-IARA-------------------------------------------------------

---------------------------E-------------------------DIQKAVA

RAIATATA---S--------LVSTG----G------------------------------

-----------------------------------TAQVDTQAVAR------------DV

QD----------------AVASTLAAA---------------------------------

----FAVVKGSQNGSSA-----------------PSSGRHKN------------------

-----------------------------

>jgi|Cosub3|1238953|fgenesh1_kg.8_#_2755_#_HTPTB_transcript/21239

------------------------ME----------------------------------

------------------------------------------------------------

------------------------------------------------------------

------------------------------------------------------------

------------------------------------------------------------

------------------------------------------------------------

------------------------------------------------------------

------------------------------------------------------------

------------------------------------------------------------

------------------------------------------------------------

------------------------------------------------------------

------------------------------------------------------------

------------------------------------------------------------

---------------------------------------TLDLKSG--------------

------------------------------------------------------------

------------------------------------------------------------

------------------------------------------------------------

------------------------------------------------------------

------------------------------------------------------------

------------------------------------------------------------

------------------------------------------------------------

------------------------------------------------------------

------------------------------------------------------------

------------------------------------------------------------

-------------GSDRCQSLSAS-------KVAVIGAGSAGLVAARELSREG------H

HVQVFEQGQTFGGIWNYQDEVED-DLLGRC-----PERRKVH------------------

------SSLYSSLRVNLPREIMSYSDFP----FTPDA-----------------------

------------------------MRGKSQDSRRYPHHTEVQHFLEAFVEEFGL--RDLI

QFSTRVVEVSPLDYTV------------GQQ---ACSNGLCSNGE---------------

------EPLSIS--------PQWVITTE----LLPGSD--------LQVP----------

----------------------------------------------------ERHTDVFD

AVVVAVGNYHE-PNLP---DVE----GLDDF---PGLQMHCHNF-RHAE------RFR--

-------D---QTVVVVGASFSGEEIARQIADV---------------------------

------------------------ALHVYHSARTW-------GKQLN-------------

------------------------------------------------------------

------------------------------------------------------------

-----------DSQTRPNLQRVPML----ARLGGN-GSAE---FSGGITA----------

--------------------EGVDAVVYCTGYR---YSYPFLER----------------

--TG-------LISTDDNRVTP---LYRHIFVPSVAPTLAFIGL-LWKS---LR-NSQFE

LQAKWVAQVLSG-RVALP-SR-EEMEADMEAFYRLLEKNSIPVRYTHCQND---------

---------------AMPVSQWEYNDMLR--RSCS-------PVPLP-------------

--------AEAWRVKLHDAISTKILQQP-----------------DTFR-----------

------------------------------------------------------------

-----DVHSAEE------------------------------------------------

----------------------------------------------QALFEDAAKGCAAT

---W--------------------------------------------------------

------------------------------------------------------------

------------------------------------------------------------

------------------------------------------------------------

------------------------------------------------------------

------------------------------------------------------------

------------------------------------------------------------

------------------------------------------------------------

------------------------------------------------------------

------------------------------------------------------------

------------------------------------------------------------

------------------------------------------------------------

------------------------------------------------------------

------------------------------------------------------AYLKER

HAA---------------------------------------------------------

------------------------------------------------------------

------------------------------------------------------------

------------------------------------------------------------

------------------------------------------------------------

------------------------------------------------------------

------------------------------------------------------------

------------------------------------------------------------

------------------------------------------------------------

------------------------------------------------------------

------------------------------------------------------------

------------------------------------------------------------

------------------------------------------------------------

------------------------------------------------------------

------------------------------------------------------------

------------------------------------------------------------

------------------------------------------------------------

------------------------------------------------------------

------------------------------------------------------------

-----------------------------

>jgi|Spipra1|3415|Sp_v1_0120200.1

------------------------MP----------------------------------

------------------------------------------------------------

------------------------------------------------------------

------------------------------------------------------------

------------------------------------------------------------

------------------------------------------------------------

------------------------------------------------------------

------------------------------------------------------------

------------------------------------------------------------

------------------------------------------------------------

------------------------------------------------------------

------------------------------------------------------------

------------------------------------------------------------

------------------------------------------------------------

------------------------------------------------------------

------------------------------------------------------------

------------------------------------------------------------

------------------------------------------------------------

------------------------------------------------------------

------------------------------------------------------------

------------------------------------------------------------

------------------------------------------------------------

------------------------------------------------------------

------------------------------------------------------------

------------------SSIEPL-------RVAVIGAGAAGLVSARELLREG------H

DVTIFEQSEQVGGVWVYDERTEE-DLTIHP-----NSRRKLHCETETKTKTKTEAEAETS

ASNGVFCSLYASLRTNLPRELMSFTDFP----FVTELSSESSLFPSLSNNRSSNHHPSSS

SSSSSSSSSSTLSSNSDINTSIQTLRVDSNDERRFPSHRTVLNYLRRFAEAYNL--LPRV

KFSTRVISIHPISDAS------------ASH---VTSN----------------------

-------------TSDTDTWIKWQVATC----QTVTKTK-------DEING-------LD

LN-------------------------SNLNRNSYK-------YEKKIEKENTNVIDTYD

AVIICNGHYSI-PKWT---DIE----GIDNW---NGKQMHSHNY-RSPN------MFK--

-------N---KVVGIIGFGNSGDDIAREIADV---------------------------

------------------------ADKVFVFSRSW-------NHENVP------------

------------------------------------------------------------

------------------------------------------------------------

---------IGSFGRRKNMFCYPMI----QKAHAN-GSVE---LEKGEKL----------

--------------------F-MDNLIHCTGYF---YSFPFLES--L-------------

--KS-------QFKVNEQRVEP---LYMHMFHPSFSPSLSFIGL-PWKV---VP-FPLFE

LQSRWVSKILSK-KVVLP-EK-SKMEKEIKKFYADLAKRNVPIRYTHMLGD---------

-------------------CQREYFDFIC--DELG---------DEK-------------

--------LEGWRNEMYESVSKQKKTKP-----------------DDYR-----------

------------------------------------------------------------

-----DRWQDVE------------------------------------------------

------------------------------------------------LHDMAMMCQCRQ

------------------------------------------------------------

------------------------------------------------------------

------------------------------------------------------------

------------------------------------------------------------

------------------------------------------------------------

------------------------------------------------------------

------------------------------------------------------------

------------------------------------------------------------

------------------------------------------------------------

------------------------------------------------------------

------------------------------------------------------------

------------------------------------------------------------

------------------------------------------------------------

--------------------------------------------------------YQTI

KERL------------------------------------------------DQGLFLI-

------------------------------------------------------------

------------------------------------------------------------

------------------------------------------------------------

------------------------------------------------------------

------------------------------------------------------------

------------------------------------------------------------

------------------------------------------------------------

------------------------------------------------------------

------------------------------------------------------------

------------------------------------------------------------

------------------------------------------------------------

------------------------------------------------------------

------------------------------------------------------------

------------------------------------------------------------

------------------------------------------------------------

------------------------------------------------------------

------------------------------------------------------------

------------------------------------------------------------

-----------------------------

>jgi|GsuMS1_1_1|5328|GsulpMS1_G1757.1

------------------------ME----------------------------------

------------------------------------------------------------

------------------------------------------------------------

------------------------------------------------------------

------------------------------------------------------------

------------------------------------------------------------

------------------------------------------------------------

------------------------------------------------------------

------------------------------------------------------------

------------------------------------------------------------

------------------------------------------------------------

------------------------------------------------------------

------------------------------------------------------------

------------------------------------------------------------

------------------------------------------------------------

------------------------------------------------------------

------------------------------------------------------------

------------------------------------------------------------

------------------------------------------------------------

------------------------------------------------------------

------------------------------------------------------------

------------------------------------------------------------

------------------------------------------------------------

------------------------------------------------------------

---------------QNLQLGNNA-------KIAIIGAGAAGLIAAKNLRDIG-----LF

QVEVFEETDTVGGTWVYQEEVAT--------------SYYFQ------------------

------SSMYRDLHTNLPKEIMGFLHFP----FDDTFGV---------------------

--------------------------------SSFPSHQVVLKYLVSFCKTFQL--YELI

RFHCSVKQISRFEYFG--------------------------------------------

-------------------GFHWDLVLY----NHGTQ-----------------------

----------------------------------------------------QVEQRRYD

AVVVCNGHYTK-PYIP---EIP----GADLF---LRPIIHSHFY-KSPE------PFC--

-------K---LRIAVLGAGNSGIDISYELSRM---------------------------

------------------------ASKVSLCHRKC-------QIRKTI------------

------------------------------------------------------------

------------------------------------------------------------

---------------GKNLEECPTI----ESLEAD-GKIL---LADKSSL----------

--------------------Q-VDILILCTGYE---YDFPFLDS----------------

--SC-------EVFVQDRVVLP---LYRHLIHA-KYPTMSFVGL-PLRV---LP-FPLFD

YQTRYLASIYSG-KCTLP-SC-ERMLVEQQEHLVDLDSKGCRKYYH--------------

---------------LFAEKQWNTAENLQ--I----------------------------

------------------------------------------------------------

------------------------------------------------------------

------------------------------------------------------------

------------------------------------------------------------

------------------------------------------------------------

------------------------------------------------------------

------------------------------------------------------------

------------------------------------------------------------

------------------------------------------------------------

------------------------------------------------------------

------------------------------------------------------------

------------------------------------------------------------

------------------------------------------------------------

------------------------------------------------------------

------------------------------------------------------------

------------------------------------------------------------

------------------------------------------------------------

------------------------------------------------------------

------------------------------------------------------------

------------------------------------------------------------

------------------------------------------------------------

------------------------------------------------------------

------------------------------------------------------------

------------------------------------------------------------

------------------------------------------------------------

------------------------------------------------------------

------------------------------------------------------------

------------------------------------------------------------

------------------------------------------------------------

------------------------------------------------------------

------------------------------------------------------------

------------------------------------------------------------

------------------------------------------------------------

------------------------------------------------------------

------------------------------------------------------------

------------------------------------------------------------

------------------------------------------------------------

-----------------------------

>jgi|GsuRT22_1_1|3935|GsulpRT22_G1455.1

------------------------ME----------------------------------

------------------------------------------------------------

------------------------------------------------------------

------------------------------------------------------------

------------------------------------------------------------

------------------------------------------------------------

------------------------------------------------------------

------------------------------------------------------------

------------------------------------------------------------

------------------------------------------------------------

------------------------------------------------------------

------------------------------------------------------------

------------------------------------------------------------

------------------------------------------------------------

------------------------------------------------------------

------------------------------------------------------------

------------------------------------------------------------

------------------------------------------------------------

------------------------------------------------------------

------------------------------------------------------------

------------------------------------------------------------

------------------------------------------------------------

------------------------------------------------------------

------------------------------------------------------------

---------------QNLQLGNNA-------KIAIIGAGAAGLIAAKNLRDIG-----LF

QVEVFEETDTVGGTWVYQEEVAT--------------SYYFQ------------------

------SSMYRDLHTNLPKEIMGFLHFP----FDDTFGV---------------------

--------------------------------SSFPSHQVVLKYLVSFCKTFQL--YELI

RFHCSVKQISRFEYFG--------------------------------------------

-------------------GFHWDLVLY----NHGTQ-----------------------

----------------------------------------------------QVEQRRYD

AVVVCNGHYTK-PYIP---EIP----GADLF---LRPIIHSHFY-KSPE------PFC--

-------K---LRIAVLGAGNSGIDISYELSRM---------------------------

------------------------ASKVSLCHRKC-------QIRKTI------------

------------------------------------------------------------

------------------------------------------------------------

---------------GKNLEECPTI----ESLEAD-GKIL---LADKSSL----------

--------------------Q-VDILILCTGYE---YDFPFLDS----------------

--SC-------EVFVQDRVVLP---LYRHLIHA-KYPTMSFVGL-PLRV---LP-FPLFD

YQTRYLASIYSG-KCTLP-SC-ERMLVEQQEHLVDLDSKGCRKYYH--------------

---------------LFAEKQWEYCRELA--DLAN---------GPR-------------

--------LSVAVQRVYEDVSQFRKKEP-----------------EKYR-----------

------------------------------------------------------------

------------------------------------------------------------

------------------------------------------------------------

------------------------------------------------------------

------------------------------------------------------------

-----------------------------RRK----------------------------

------------------------------------------------------------

------------------------------------------------------------

------------------------------------------------------------

------------------------------------------------------------

------------------------------------------------------------

-----------------------YII----------------------------------

------------------------------------------------------------

------------------------------------------------------------

------------------------------------------------------------

------------------------------------------------------------

------------------------------------------------------------

------------------------------------------------------------

------------------------------------------------------------

--------------------------------------------------------FG--

------------KGPND-------------------------------------------

------------------------------------------------------------

------------------------------------------------------------

------------------------------------------------------------

------------------------------------------------------------

------------------------------------------------------------

------------------------------------------------------------

------------------------------------------------------------

------------------------------------------------------------

------------------------------------------------------------

--WRV---------FE---------------------------NDS--------------

-----------------------------------------------------DV-----

------------------------------------------------------------

------------------------------------------------------------

------------------------------------------------------------

---------------------------------------------------TL-------

-----------------------------

>jgi|Caulen1|93398|g8005.t1

------------------------MA----------------------------------

------------------------------------------------------------

------------------------------------------------------------

------------------------------------------------------------

------------------------------------------------------------

------------------------------------------------------------

------------------------------------------------------------

------------------------------------------------------------

------------------------------------------------------------

------------------------------------------------------------

------------------------------------------------------------

------------------------------------------------------------

------------------------------------------------------------

------------------------------------------------------------

------------------------------------------------------------

------------------------------------------------------------

------------------------------------------------------------

------------------------------------------------------------

------------------------------------------------------------

------------------------------------------------------------

------------------------------------------------------------

------------------------------------------------------------

------------------------------------------------------------

------------------------------------------------------------

----------------------QK-------RVAIIGAGAAGLVCAKECIQEG------L

NVQMFEKYPYSGGLWVVDEVPLDLQELNVHEDGYTDMARKAY------------------

------SSVHDTLQTNLSRFLMGFSDFP----FDTNF-----------------------

--------------------------PGSKDPRTFPGNDEVLRYLDAFKKEFEL--DQFI

SYETEVINVDRTDDSE--------------------------------------------

--------------------FEWQVKIN----CKG-------------------------

----------------------------------------------------EIKTLFFD

SVAVCSGHHTV-PQLP---ELK----NIMGF---GRPCLHSHLY-RNPN------RYK--

-------D---KVVLILGSGGSGRDTALEISKV---------------------------

------------------------SKKVYFCAREW-------KAPYDM------------

------------------------------------------------------------

------------------------------------------------------------

---------STPFEVIENIECCDSI----SHCPAK-GTVQ---LEDGTIL----------

-------------------TD-VDEMLLCTGYI---YDYPFLKD----------------

--SG-------LISTAESFVYP---VYKHIFHT-KYPTLGFIGLIDFAN---VS-FLTFE

CQSKYFARVILG-RCQLP-DY-EIMMKDIDQHRQRLEENNQPIKHFHAL-----------

---------------SYIENPFIYKNEVL--SLCD--------DCSA-------------

--------LPSWRKELFDSNLYLLLTEP-----------------LTFRAIEI---P---

---------SDIQAIIGAGAAGLICAKECIQEGLNVQIFEKHPYSGGMWVVDEVPLDLQE

LNVHEDGYKDKAKKVH--------------------------------------------

----------------------------------------------SSIYDEMQTNLSRF

---VMGFSDFP-FDTEF----PGSKDPRTFPGTDEVLRYLDAFKKEFELDQFISYETEVI

DIDRTEDSECEWQVKINC------------------------------------------

-------------------KG-----KI-KTLFL---------------DSVAVCSGHYT

-----VPQLPELKNIV----------------------------GF---GRPCLHSHLYR

NPNRYKDKVVLI--------------------LGNGSSGRDIVIEIAEVAKKVY------

----------------------------FCARKW-------------KSTLDLSKA----

----------------------------------FEGIKNICC-----------------

---------------------------------------------CKSISHCPAEGTVQL

EDGTILTDVDEML----LCTGYSYDYPFL-------------------------------

------------------------------------------------------------

-----------------------------------------------------KNSGL--

------------------ISTAESFVYPIYKH-IFH----------------------TK

YPTLGFIG------------------QSSQLSVINRGIRLPPGIHLVTYFLQVAWQMLSL

IPHCFILITILSLGAIPC--IDAEYKDEGTD------IGT-------AVFISRLAFLHIV

QNLR----------------------------SHV---------------CFSKGEAAL-

--------------------------------------------------------RIGR

DETATSLQDRNPQF---------------------------------------VRPFG--

----------APSVPNAYRS----------------------------------------

------------------------------------------------------------

------------GP----------------------------------------------

------------------------------------------------------------

------------------------------------------------------------

------------------------------------------------------------

------------------------------------------------------------

----TRTLKYSGCSETHNT-----------------------------------------

------------------------------------------------------------

------------------------------------------------------------

--ISAIVECSNAVR-Q---------------------------NP---------------

----------------DLFVNDYPCSY------------------------------ASW

RNEVSGKSLGP----------LKTGPTIMR------------------------------

-----------------------------------LAQLHTDDQARMNTMSHQGSDGSDL

QTRADRVDLPWGTVGENVAVGFSSAKAVVMAWMCSPPHRKNLMSCDFSTIGVG-------

----AGVSSAGRNFYTQVFSCGVSEECSCGPQGGALKRRKEKKSSTEQFQLPLEKTHSGP

LCGWVDGEWKCDQHFGASTQ--PYNPFGY

>jgi|Cosub3|1167623|fgenesh1_kg.1_#_10111_#_TRINITY_DN7923_c0_g1_i1

------------------------MS--------------------------DEER----

------------------------------------------------------------

------------------------------------------------------------

------------------------------------------------------------

------------------------------------------------------------

------------------------------------------------------------

------------------------------------------------------------

------------------------------------------------------------

------------------------------------------------RNG---------

------------------------------------------------------------

-------------------P----------------------------------------

------------------------------------------------------------

------------------------------------------------------------

------------------------------------------------------------

------------------------------------------------------------

-----------------------------------------------------------T

PSCSDTKLPSK-------------------------------------------------

------------------------------------------------------------

------------------------------------------------------------

------------------DH----------------------------------------

------------------------------------------------------------

------------------------------------------------------------

------------------------------------------------------------

------------------------------------------------------------

-------KITIEITTRAGNDALKQ-------RACVIGAGKSGLIACKVLHERN------I

PFDCFEASSQVGGLWVLNSDSGL-------------------------------------

------SASYESLRINTSKQMTSFHDFP----MPKHY-----------------------

--------------------------------PTYPTRKEILEYLESYADHFGF--RSHI

TFRTEVKKVEQLPDG---------------------------------------------

---------------------SFRVFAE----DKATG-----------------------

----------------------------------------------------HTLARLYT

SVLVANGHHWH-AAWP---ELP----G--SF---TGTLMHSHEY-RTPK------VME--

-------G---KRVMVIGAGNSGMDIASEASQC---------------------------

-----------------------GAAAVFLSCRRRVH-----VVPRYI--FG--------

---------------APSDSILPAWLGVTA------------------------------

-PRRLMEKGVTCLIHISRG-------SQTSFKF-----------PPPD-FGLLRVHPT--

--VSPGTGDILQLIKDGKVTVRPG-----IERIED-RTVH---FTDGTKE----------

--------------------D-IDIIVCATGYN---VSCPFLPP----------------

-----------KVEVLKGDKPQ---LLLNVAHP-RAPGIFFLGF-VQAH---GPMIPCVE

GQMPWVADLIQG-ITELP-GE-AEMAAKIAEQHEFNRRNFTQVKRH--------------

---------------ALMVEFHRYLLQLRM-ARCPKG-----------------------

--------KGWLGRKLWKAKTFLVFCTG--------------------------------

------------------------------------------------------------

------------------------------------------------------------

------------------------------------------------------------

------------------------------------------------------------

------------------------------------------------------------

------------------------------------------------------------

------------------------------------------------------------

------------------------------------------------------------

------------------------------------------------------------

------------------------------------------------------------

------------------------------------------------------------

------------------------------------------------------------

------------------------------------------------------------

------------------------------------------------------------

------------------------------------------------------------

------------------------------------------------------------

------------------------------------------------------------

------------------------------------------------------------

------------------------------------------------------------

------------------------------------------------------------

---------KTC------------------------------------------------

------------------------------------------------------------

------------------------------------------------------------

------------------------------------------------------------

------------------------------------------------------------

------------------------------------------------------------

------------------------------------------------------------

------------------------------------------------------------

------------------------------------------------------------

------------------------------------------------------------

------------------------------------------------------------

------------------------------------------------------------

------------------------------------------------------------

------------------------------------------------------------

------------------------------------------------------------

------------------------------------------------------------

-----------------------------

>jgi|Coprin2167_1|5401|KAK9917333.1

------------------------MS--------------------------DEER----

------------------------------------------------------------

------------------------------------------------------------

------------------------------------------------------------

------------------------------------------------------------

------------------------------------------------------------

------------------------------------------------------------

------------------------------------------------------------

------------------------------------------------RNG---------

------------------------------------------------------------

-------------------P----------------------------------------

------------------------------------------------------------

------------------------------------------------------------

------------------------------------------------------------

------------------------------------------------------------

-----------------------------------------------------------I

PSCSDKKVPTK-------------------------------------------------

------------------------------------------------------------

------------------------------------------------------------

------------------GH----------------------------------------

------------------------------------------------------------

------------------------------------------------------------

------------------------------------------------------------

------------------------------------------------------------

-------TIAIQITTRAGNDAPEQ-------RVCVIGAGKSGLIACKVLHERR------V

PFDCFEASSQVGGLWVLNSDSGL-------------------------------------

------SASYESLRINTSKQMTSFHDFP----MPQHY-----------------------

--------------------------------PTYPTRKEILEYLEAYADHFGF--RGCI

TFRTEVTRVEPLPDS---------------------------------------------

---------------------SFRVFTE----DKATG-----------------------

----------------------------------------------------HTVARLYT

SVVVANGHHWN-AAWP---ELP----G--TF---TGTLMHSHEY-RTPK------SME--

-------G---KRLMVIGAGNSGMDIASEASQL---------------------------

------------------------------------------------------------

------------------------------------------------------------

----------------SRG-------SQASFKF-----------PPPK-FGLLRVHPT--

--VSPGTGDILQLIKDGKVAVRPG-----IERIED-HTVH---FPDGTKE----------

--------------------D-IDIIVCATGYN---VCCPFLPS----------------

-----------EVEVLKEDKPQ---LLLNVAHP-HAPGIFFLGF-VQAH---GPMIPCVE

GQMPWVADLIQG-IAELP-EK-AQMEAKIAKQHDFNRRNFTQVKRH--------------

---------------ALMVEFHRYLLQLRA-AKMQVK-----------------------

------------------------------------------------------------

------------------------------------------------------------

------------------------------------------------------------

------------------------------------------------------------

------------------------------------------------------------

------------------------------------------------------------

------------------------------------------------------------

------------------------------------------------------------

------------------------------------------------------------

------------------------------------------------------------

------------------------------------------------------------

------------------------------------------------------------

------------------------------------------------------------

------------------------------------------------------------

------------------------------------------------------------

------------------------------------------------------------

------------------------------------------------------------

------------------------------------------------------------

------------------------------------------------------------

------------------------------------------------------------

------------------------------------------------------------

---------AVC------------------------------------------------

------------------------------------------------------------

------------------------------------------------------------

------------------------------------------------------------

------------------------------------------------------------

------------------------------------------------------------

------------------------------------------------------------

------------------------------------------------------------

------------------------------------------------------------

------------------------------------------------------------

------------------------------------------------------------

------------------------------------------------------------

------------------------------------------------------------

------------------------------------------------------------

------------------------------------------------------------

------------------------------------------------------------

-----------------------------

>jgi|Botrbrau1_1|11255|Bobra.0173s0079.1

------------------------MAALP-----------------CVPQSLDEHEG---

-----------------SL----------------------------------------K

HAMQYG-------------------------LCG--------------------------

-------------MEHS---LR--------------------------------------

------------------------------------------------------------

------------------------------------------------------------

------------------------------------------------------------

------------------------------------------------------------

------------------------------------------NTKRQLRGH---------

------------------------------------------------------------

-------------------PA---------------------------------------

------------------------------------------------------------

----------------------------------------AC------------------

------------------------------------------------------------

------------------------------------------------------------

----------------------------------------------------------FA

AYRCPRIVP---------------------------------------------------

------------------------------------------------------------

------------------------------------------------------------

------------------------------------------------------------

------------------------------------------------------------

------------------------------------------------------------

------------------------------------------------------------

------------------------------------------------------------

-------------ESKSRTSSNHK-------TVCVVGAGCAGLSACNALRERG------I

PYVCYEKGSKIGGLWRIENDNGL-------------------------------------

------SGAYESLSINTSKSTMEFAGFP----FPEEW-----------------------

--------------------------------TPFLSHKQVLEYIEQYAAHFDL--KKNI

QFETTVTEVAPHNNG---------------------------------------------

---------------------GYTVSIQ----RKIES-----------------------

----------------------------------------------------KPKSKHFA

TVIVANGHHWD-PLFP---TLP----G--KF---AGQLLHAHQY-RNHS------ILD--

-------D---KRVVVLGMGNSGVDIASEASKY---------------------------

------------------------ARSVIISSRESAY-----VVPRWI--KG--------

---------------KPMDTINTSAIALL-------------------------------

-PWRWRQWLFSQQLKPFIQ-------EQIAAGW-----------GVPK-HGLMQAHFT--

-----IHKDIVEQCKAKKVTPKSG-----IKQISG-NLIT---FMDGTSV----------

--------------------E-VDVIVCATGYN---VTFPFFKG----------------

----------NRIRVVDEDGPR---LYKNMIHP-EHPNLFFVGL-LQPT---GPMIPLCE

QQTALIGDILQG-KVVPP-CP-EKQLRAIQLQKQRVKDRWISAQRH--------------

---------------LLEVDFHEYLMELK-CERCPSK-----------------------

--------ISFWERILYTIKVYLEALFPR----------TGCLGATRYE-----------

------------------------------------------------------------

------------------------------------------------------------

------------------------------------------------------------

------------------------------------------------------------

------------------------------------------------------------

------------------------------------------------------------

------------------------------------------------------------

------------------------------------------------------------

------------------------------------------------------------

------------------------------------------------------------

------------------------------------------------------------

------------------------------------------------------------

------------------------------------------------------------

------------------------------------------------------------

------------------------------------------------------------

------------------------------------------------------------

------------------------------------------------------------

------------------------------------------------------------

------------------------------------------------------------

------------------------------------------------------------

------------KSPEHHQS----------------------------------------

------------------------------------------------------------

------------------------------------------------------------

------------------------------------------------------------

------------------------------------------------------------

------------------------------------------------------------

------------------------------------------------------------

------------------------------------------------------------

------------------------------------------------------------

------------------------------------------------------------

------------------------------------------------------------

------------------------------------------------------------

---------------------------IEK------------------------------

------------------------------------------------------------

------------------------------------------------------------

------------------------------------------------------------

-----------------------------

>jgi|Botrbrau1_1|17708|Bobra.0352s0002.1

------------------------MD----------------------------------

------------------------------------------------------------

------------------------------------------------------------

------------------------------------------------------------

------------------------------------------------------------

------------------------------------------------------------

------------------------------------------------------------

------------------------------------------------------------

------------------------------------------------------------

------------------------------------------------------------

------------------------------------------------------------

------------------------------------------------------------

------------------------------------------------------------

------------------------------------------------------------

------------------------------------------------------------

------------------------------------------------------------

------------------------------------------------------------

------------------------------------------------------------

------------------------------------------------------------

------------------------------------------------------------

------------------------------------------------------------

------------------------------------------------------------

------------------------------------------------------------

------------------------------------------------------------

----------------------TN-------SICIIGGGAAGIALARVLNDRG------I

QFTWLEKGTRLGGLWVYNNDSGM-------------------------------------

------SAIYQSLTTNTSRTTMGFPGFP----LPEQL-----------------------

--------------------------------PDFPPHHEVLKYLDAYIERYNL--QPLA

TLGCEVEKLEEAPDG---------------------------------------------

---------------------AFLVSSL----VLATG-----------------------

----------------------------------------------------QRECRRYE

SVILATGMNWD-PALP---SLE----G--SF---SGIVMHSVQY-REPS------ILK--

-------G---QRVLVVGAGPSGADIATEAARE---------------------------

------------------------AGSATLSLRHGMY-----AFHRYG-KDG--------

---------------RPSDTKNAAWLGLL-------------------------------

-PWKLRQYIMTRAVQ-----------PKALPGF-----------PQPA-YQLLQ------

--------------RTGHLKTKPG-----IASLED-RTVQ---FVDGTRE----------

--------------------D-FDVIIFATGFR---ESTPFLSS-EL-------------

--EG-------RLRSADGQLS----LYLNIVPP-DVPGLYFTGF-VRQL---GPIFPSFH

EQALFLADLLQG-KCKLP-AP-ESMRQSLTIRHANGEKRYSKPYK---------------

---------------SLEVDLHIYWLQLRI-ARCLRG-----------------------

--------LPVWRRWIWTIQSFFS---------------------ILFR-----------

------------------------------------------------------------

------------------------------------------------------------

------------------------------------------------------------

------------------------------------------------------------

------------------------------------------------------------

------------------------------------------------------------

------------------------------------------------------------

------------------------------------------------------------

------------------------------------------------------------

------------------------------------------------------------

------------------------------------------------------------

------------------------------------------------------------

------------------------------------------------------------

------------------------------------------------------------

------------------------------------------------------------

------------------------------------------------------------

------------------------------------------------------------

------------------------------------------------------------

------------------------------------------------------------

------------------------------------------------------------

--------------PIEES-----------------------------------------

------------------------------------------------------------

------------------------------------------------------------

------------------------------------------------------------

------------------------------------------------------------

------------------------------------------------------------

------------------------------------------------------------

------------------------------------------------------------

------------------------------------------------------------

------------------------------------------------------------

------------------------------------------------------------

------------------------------------------------------------

------------------------------------------------------------

------------------------------------------------------------

------------------------------------------------------------

------------------------------------------------------------

-----------------------------

>jgi|Cyapar1_1|14550|g14034.t1

------------------------M-----------------------------------

------------------------------------------------------------

------------------------------------------------------------

------------------------------------------------------------

------------------------------------------------------------

------------------------------------------------------------

------------------------------------------------------------

------------------------------------------------------------

------------------------------------------------------------

------------------------------------------------------------

------------------------------------------------------------

------------------------------------------------------------

------------------------------------------------------------

------------------------------------------------------------

------------------------------------------------------------

------------------------------------------------------------

------------------------------------------------------------

------------------------------------------------------------

------------------------------------------------------------

------------------------------------------------------------

------------------------------------------------------------

------------------------------------------------------------

------------------------------------------------------------

------------------------------------------------------------

-------------------------------RIAVIGAGVSGLTAAKCCADEG------L

TPVVFEQSRQIGGLWRFTEEESH-------------------------------------

------SSIMRTTVINTSKEMMCFSDFP----MPESY-----------------------

--------------------------------PTFLPHRLVLQYLEDYAEHFKL--LQYV

RFGCRIVSVEPERVGE------------ERP---ESAGGPG-------------------

--------------------GRWRIRWESTHDESGTGAS---------------------

----------------------------------------------------AAAEELFD

AVMVCTGHHWK-PRTP---AFP----GADTF---KGVQVHSHAY-KDDL------PYR--

-------G---KRVVVVGIGNSGADIAVELSRA---------------------------

------------------------AAQVELSTRSGTW-----VFPRYL--LN--------

---------------TPFDHMLSRLVSTIC------------------------------

-PLWVRTLFMEKAISLTHG-------SVKDFGL------------RPK-HRIFQAHPT--

-----IHSELLGRIGTGTIAVRPD-----VAELHP-DAVS---FADGTRV----------

--------------------K-ADAIIYCTGYE---IEVPFVKN---RP-----------

-GGNPL-----SVGIGPSNSVR---LYKHMLPLEGPPTLAFIGL-VQPL---GAIMPISE

IQARWFARLLKG-LCRLP-PR-EEMARDVERREAAVRARYKPSPRH--------------

---------------TVQVDYLDYMEEIA--GLVG---------CRPRL-----------

-----SNPLVWLRSPRVALATLVGPASP-----------------VHFRLR---------

----------GPGAWAGAPDAVLRMAGW--------------------------------

-----DKGPSKAALGL--------------------------------------------

---------------------------------------------GALLLAAAAAALAFA

---LYAG-----------------------------------------------------

------------------------------------------------------------

------------------------------------------------------------

------------------------------------------------------------

------------------------------------------------------------

------------------------------------------------------------

------------------------------------------------------------

------------------------------------------------------------

------------------------------------------------------------

------------------------------------------------------------

-------------------------------------------------------AG---

------------------------------------------------------------

------------------------------------------------------------

---------------------------------------R-------A----SLSFL---

------------------------------------------------------------

------------------------------------------------------------

------------------------------------------------------------

--------------PVR-------------------------------------------

------------------------------------------------------------

------------------------------------------------------------

------------------------------------------------------------

------------------------------------------------------------

------------------------------------------------------------

------------------------------------------------------------

------------------------------------------------------------

------------------------------------------------------------

------------------------------------------------------------

------------------------------------------------------------

------------------------------------------------------------

------------------------------------------------------------

------------------------------------------------------------

------------------------------------------------------------

------------------------------------------------------------

-----------------------------

>Sphfalx14G087800|Sfallax_v1.1/1-543

------------------------ML----------------------------------

------------------------------------------------------------

------------------------------------------------------------

------------------------------------------------------------

------------------------------------------------------------

------------------------------------------------------------

------------------------------------------------------------

------------------------------------------------------------

------------------------------------------------------------

------------------------------------------------------------

------------------------------------------------------------

------------------------------------------------------------

------------------------------------------------------------

-------------------------KGQ-----------AVKETAIL-------------

------------------------------------------------------------

-----------------------------------------------------------F

SSTLDITLP---------------------------------------------------

------------------------------------------------------------

------------------------------------------------------------

------------------------------------------------------------

------------------------------------------------------------

------------------------------------------------------------

------------------------------------------------------------

------------------------------------------------------------

------------------PPPNSM-------KVAIVGAGLSGLVTAKVLAQFG------H

KVTVYEKCPDVGGTWSR-------------------------------------------

------TRWYPGLTTQNGKDSYCFSDFP----MPDSY-----------------------

--------------------------------PEWPSGEQVHQYLDEYVKHFGL--DALL

QLNTEVVSIELDEVAG--------------------------------------------

--------------------NIWTIGTR----QSDNNKD-------SSMPIL--------

----------------------------------------------------ATQSETFD

HVVIANGIFSD-PYVP---TFP----GLEEFQQAGGQVLHTTDV-HQLL-----GDAT--

-------G---KHFIIVGYGRSACDVACALSDI---------------------------

-----------------------SSIKVVVVARELIW-----KIPKWI------------

------------GGVLNFKYLLLTRMGEALF---------QYIHPHGFEQFL---HNGLG

KPLRDLMMSSLQAIITCQL-------KLRKLGL------------LPD-GP-LE-DIA-R

TTLSLVTDGFYEKVEMGAIEVHGGTHIVKLCVIDDKPSTL---LSDGTIV----------

--------------------P-TDFVICGTGFH---QSVPFFSM-NI-------------

--IK-------QLTD-DQGNFL---LYRHIHPI-NVPQLSFNGY-NSSL---FC-PLSSE

VAALWIGNLLAG-GMKLP-SP-NKQQLEVIEQLAWMEKRTKGKHA---CGG---------

---------------SIIPFTFHYIDELL--KDMN-------------------------

--------LTLNAHT--HIREWLLPINF-----------------GAYVGLI--------

------------------------------------------------------------

-----DELT---------------------------------------------------

------------------------------------------------------------

------------------------------------------------------------

------------------------------------------------------------

-----------------------------KRL----------------------------

------------------------------------------------------------

------------------------------------------------------------

------------------------------------------------------------

------------------------------------------------------------

------------------------------------------------------------

------------------------------------------------------------

------------------------------------------------------------

-----------------------------------------------------ASEGKL-

------------------------------------------------------------

------------------------------------------------------------

------------------------------------------------------------

------------------------------------------------------------

------------------------------------------------------------

------------------------------------------------------------

------------TFPLC-------------------------------------------

------------------------------------------------------------

------------------------------------------------------------

------------------------------------------------------------

------------------------------------------------------------

------------------------------------------------------------

------------------------------------------------------------

------------------------------------------------------------

------------------------------------------------------------

------------------------------------------------------------

------------------------------------------------------------

------------------------------------------------------------

------------------------------------------------------------

------------------------------------------------------------

------------------------------------------------------------

------------------------------------------------------------

-----------------------------

>jgi|Chrzof2|4671200|fgenesh1_kg.chr_08_#_4392_#_transcript/24020

------------------------M-----------------------------------

------------------------------------------------------------

------------------------------------------------------------

------------------------------------------------------------

------------------------------------------------------------

------------------------------------------------------------

------------------------------------------------------------

------------------------------------------------------------

------------------------------------------------------------

------------------------------------------------------------

------------------------------------------------------------

------------------------------------------------------------

------------------------------------------------------------

------------------------------------------------------------

------------------------------------------------------------

------------------------------------------------------------

------------------------------------------------------------

------------------------------------------------------------

------------------------------------------------------------

------------------------------------------------------------

------------------------------------------------------------

------------------------------------------------------------

------------------------------------------------------------

------------------------------------------------------------

-------------------------------RIAVIGSGVSGIVVAKTLLQAG------H

AVTVYEKSRNLGGVWNP-------------------------------------------

------DVVYPGAHTQNIGHMYRFTDFA----PPKTW-----------------------

--------------------------------DKYPTATQVYDYLRDYATHNEL--WGSI

RLSTNVIRIMALTTDD--------------------------------------------

------------------GRAGWFVTVH----SSG-------------------------

----------------------------------------------------QRLEEVFD

FVVISTGTYCQ-PSIP---SFT----GQEEFVEAGGKVFHTSQL-SDVD---ISSKIA--

-------G---KSVAVVGFGKSALDVATYCTNC---------------------------

----------------------ATAKSVTILYRQARW-----CVPKKL------------

------------AHLIPYEYVLSCRAGMLLM---------PSPYEGHLVQFI----RKLL

KPFTWLAFRSMEMLISMQQ-------GLRKIGM------------VPC-EQ-LEKALT-C

EGSHLETDGFYDAIHQGKLKPKQGE----IQSLSP-GQLT---LKDGSKL----------

--------------------S-ADVLILGTGYIW--HHLPFLDP-NT-------------

--RK-------QLIDEDKNTCQ---LYRRILPL-NIPNLAFIGY-QSSL---TS-VLTFE

MGAKWLTAYLEG-RIK-P-TH-DMMQDTLLKERSYALRNFTGKHVRTVSGP---------

---------------CIAPYAMPYVDSLL--SDMN-------------------------

--------QKPVKGLINKLKDFLLPWDA-----------------SLYK-----------

------------------------------------------------------------

-----D------------------------------------------------------

------------------------------------------------------------

------------------------------------------------------------

------------------------------------------------------------

------------------------------------------------------------

------------------------------------------------------------

------------------------------------------------------------

------------------------------------------------------------

------------------------------------------------------------

------------------------------------------------------------

------------------------------------------------------------

------------------------------------------------------------

------------------------------------------------------------

------------------------------------------------------------

------------------------------------------------------------

---CR-------------------------------------------------------

------------------------------------------------------------

------------------------------------------------------------

------------------------------------------------------------

------------------------------------------------------------

------------------------------------------------------------

------------------------------------------------------------

------------------------------------------------------------

------------------------------------------------------------

------------------------------------------------------------

------------------------------------------------------------

------------------------------------------------------------

------------------------------------------------------------

------------------------------------------------------------

------------------------------------------------------------

------------------------------------------------------------

------------------------------------------------------------

------------------------------------------------------------

------------------------------------------------------------

------------------------------------------------------------

-----------------------------

>jgi|Chrzof2|4671163|fgenesh1_kg.chr_08_#_4355_#_transcript/69809

------------------------M-----------------------------------

------------------------------------------------------------

------------------------------------------------------------

------------------------------------------------------------

------------------------------------------------------------

------------------------------------------------------------

------------------------------------------------------------

------------------------------------------------------------

------------------------------------------------------------

------------------------------------------------------------

------------------------------------------------------------

------------------------------------------------------------

------------------------------------------------------------

------------------------------------------------------------

------------------------------------------------------------

------------------------------------------------------------

------------------------------------------------------------

------------------------------------------------------------

------------------------------------------------------------

------------------------------------------------------------

------------------------------------------------------------

------------------------------------------------------------

------------------------------------------------------------

------------------------------------------------------------

-------------------------------QIAVIGGGISGLVVAKTLSKAG------N

VVTVFERSRNLGGVWNP-------------------------------------------

------DVIYPGAHTQNVGSMYRFTDFA----PPSTW-----------------------

--------------------------------ETFPTSEQVYDYVVSYVKHNDL--LGSI

QLNTTVTSIKPITGPT--------------------------------------------

------------------ACKGWSVTVE----HNG-------------------------

----------------------------------------------------QSSTEEFE

FVVVCTGTYRE-AKIP---IYP----GQDEFVKAGGKVVHTSQL-SDVD---ISGTIT--

-------G---KSVAVVGFGKSALDMATYCTNC---------------------------

----------------------TTAKSVTILYRHLRW-----CLPKVL------------

------------GGLLPYQYALLCRAGMLLL---------PSPYASPLVQFI----RMIL

LPVFWVAFRSLELLITLQQ-------GLHKLGI------------VPT-VQ-LNRSMT-C

EGAQIEPDGMFQLMHDGKLKAKQGE----IQSLSP-GQLT---LKDGSKV----------

--------------------S-ADVLILGTGYIW--HHLPFLDP-NT-------------

--RK-------QIIDEDKNTCQ---LYKKILPL-NVPNLAFIGY-QSSL---TS-VLTFE

MGAKWLTAYMEG-RIK-P-TH-EMMQDTMLKERSYAVEHFTGHNVNSLFGP---------

---------------CIAPYAMPYVDSLL--SDMK-------------------------

--------QEPVKGLINKLKDFLLPWDA-----------------SLYK-----------

------------------------------------------------------------

-----D------------------------------------------------------

------------------------------------------------------------

------------------------------------------------------------

------------------------------------------------------------

------------------------------------------------------------

------------------------------------------------------------

------------------------------------------------------------

------------------------------------------------------------

------------------------------------------------------------

------------------------------------------------------------

------------------------------------------------------------

------------------------------------------------------------

------------------------------------------------------------

------------------------------------------------------------

------------------------------------------------------------

---CR-------------------------------------------------------

------------------------------------------------------------

------------------------------------------------------------

------------------------------------------------------------

------------------------------------------------------------

------------------------------------------------------------

------------------------------------------------------------

------------------------------------------------------------

------------------------------------------------------------

------------------------------------------------------------

------------------------------------------------------------

------------------------------------------------------------

------------------------------------------------------------

------------------------------------------------------------

------------------------------------------------------------

------------------------------------------------------------

------------------------------------------------------------

------------------------------------------------------------

------------------------------------------------------------

------------------------------------------------------------

-----------------------------

>AT1G19250|Athaliana_Araport11/1-530

------------------------MA----------------------------------

------------------------------------------------------------

------------------------------------------------------------

------------------------------------------------------------

------------------------------------------------------------

------------------------------------------------------------

------------------------------------------------------------

------------------------------------------------------------

------------------------------------------------------------

------------------------------------------------------------

------------------------------------------------------------

------------------------------------------------------------

------------------------------------------------------------

------------------------------------------------------------

------------------------------------------------------------

------------------------------------------------------------

------------------------------------------------------------

------------------------------------------------------------

------------------------------------------------------------

------------------------------------------------------------

------------------------------------------------------------

------------------------------------------------------------

------------------------------------------------------------

------------------------------------------------------------

---------------SNYDKLTSS-------RVAIIGAGVSGLAAAKNLVH--------H

NPTVFEASDSVGGVWRS-------------------------------------------

-------CTYETTKLQSARVDYEFSDFP----WPNNR-----------------------

------------------------------DDTTFPPYLEILDYLESYAKHFDL--LKFM

KFGSKVIEVRFIGDGE-----------------TPQMVDLGAYGN---------------

---------------LLPGKPVWEVAVQ----IGDSG-----------------------

----------------------------------------------------DIQWHAFE

FVVVCTGKYGDVPRIP---AFP-AKKGPEMF---QGKVMHSMDY-CKLEKEEASTLLS--

-------G---KKVAVIGFKKSAIDLALESALANQ-------------------------

-----------G----------EGGKACTMVVRTTHW-----GIPHYW-VWG--------

---------------LPFFLFYSSRASQFLH---------DRPNQSFLRTLF----CLLF

SLLRAVVSKFIESYVLWKL-------PLEKYGL------------KPN-HS-FEEDYA-S

CQMAIIPENFFEEADKGMIRFKKSS----KWWFYE-EGIV---FEDGTTL----------

--------------------E-ADVVILATGYDGKKKLKAIVPE-PF-------------

--RT-------WLEF-PSGVMP---LYRGTIHP-LIPNMGFVGY-VQSS---SN-LHTSE

LRSMWLSRLVDE-KFRLP-SK-EKMLDQFLKEMEVTRNSSRFYKRH--------------

---------------CISTFSIQHADDMC--NDMG-------------------------

--------LNPWRKS-NFLLEAFSPYGS-----------------QDYRLGQ--------

------------------------------------------------------------

-----EEKEDMTA-----------------------------------------------

------------------------------------------------------------

------------------------------------------------------------

------------------------------------------------------------

------------------------------------------------------------

------------------------------------------------------------

------------------------------------------------------------

------------------------------------------------------------

------------------------------------------------------------

------------------------------------------------------------

------------------------------------------------------------

------------------------------------------------------------

------------------------------------------------------------

------------------------------------------------------------

------------------------------------------------------------

------------------------------------------------------------

------------------------------------------------------------

------------------------------------------------------------

------------------------------------------------------------

------------------------------------------------------------

------------------------------------------------------------

------------------------------------------------------------

------------------------------------------------------------

------------------------------------------------------------

------------------------------------------------------------

------------------------------------------------------------

------------------------------------------------------------

------------------------------------------------------------

------------------------------------------------------------

------------------------------------------------------------

------------------------------------------------------------

------------------------------------------------------------

------------------------------------------------------------

------------------------------------------------------------

------------------------------------------------------------

-----------------------------

>Mvestita_S18g14860-RA|Marsilea_vestita/1-572

------------------------MDHAT-------------------------------

------------------------------------------------------------

------------------------------------------------------------

------------------------------------------------------------

------------------------------------------------------------

------------------------------------------------------------

------------------------------------------------------------

------------------------------------------------------------

------------------------------------------------------------

------------------------------------------------------------

------------------------------------------------------------

------------------------------------------------------------

------------------------------------------------------------

------------------------------------------------------------

------------------------------------------------------------

------------------------------------------------------------

TKSSIAIHP---------------------------------------------------

------------------------------------------------------------

------------------------------------------------------------

------------------------------------------------------------

------------------------------------------------------------

------------------------------------------------------------

-------------------------------------YILRSQSSETYSERLSKEEEQGS

FVLLH---------ELMESSA---------------------------------------

-------------NLPVSQSPDAP-------SVCIVGAGMTGICASKYLLQHG------I

QPVIFEAQPGIGGVWRH-------------------------------------------

--------TFASTKLQTPRDAYQFSDYT----WPPGT-----------------------

--------------------------------PDYPSHDHVMKYLHSYASEFHV--LERI

RFNCKVVEARQVGCFD-----------------VAD--DNGNVDE---------------

---------------GARKGHQWAVCVQ----KISDASSA--------------------

----------------------------------------------------STEWFYFD

FLLLCIGRYGDVPNLP---IYP-PSRGPEIF---KGKVLHSMEY-AMLDDKTANEFLA--

-------N---KRVVVVGMMKSALDVAVEASNANL-------------------------

-----------G----------LHGHPCTVVFRSAHW-----MLPHHK-PFG--------

---------------LPLSYLYSTRFSELMI---------PKPDQGHFLSII----STLL

KPMRWTISKFVELYLLLKL-------PLRQFDL------------VPK-QT-FYQQFA-S

CQVPLLPDLFFQRVQAGHIQLKRTN----DWSFSE-NGLV---LDDGTNI----------

--------------------E-ADVVILATGYKGEMKLKSILQG-SC-------------

--AQ-------GLKS-SGDIIP---LYRGIIPP-RIPHLAVIGY-HENL---SN-LHSSE

MGARWLAHLLSS-KIHLP-SI-VKMEEDVQKWTDFMKNSTPFYQKS--------------

---------------CIGAVSIWHSDQMC--RDLG-------------------------

--------WNPKRKN-NCIQELFTPYSN-----------------MDYQ-----------

------------------------------------------------------------

-----DCST---------------------------------------------------

------------------------------------------------------------

------------------------------------------------------------

------------------------------------------------------------

------------------------------------------------------------

------------------------------------------------------------

------------------------------------------------------------

------------------------------------------------------------

------------------------------------------------------------

------------------------------------------------------------

------------------------------------------------------------

------------------------------------------------------------

------------------------------------------------------------

------------------------------------------------------------

------------------------------------------------------------

------------------------------------------------------------

------------------------------------------------------------

------------------------------------------------------------

------------------------------------------------------------

------------------------------------------------------------

------------------------------------------------------------

------------------------------------------------------------

------------------------------------------------------------

------------------------------------------------------------

------------------------------------------------------------

------------------------------------------------------------

------------------------------------------------------------

------------------------------------------------------------

------------------------------------------------------------

------------------------------------------------------------

------------------------------------------------------------

------------------------------------------------------------

------------------------------------------------------------

------------------------------------------------------------

------------------------------------------------------------

-----------------------------

>jgi|Spimu1_1|10495|SM000022S07132

------------------------------------------------------------

------------------------------------------------------------

------------------------------------------------------------

------------------------------------------------------------

------------------------------------------------------------

------------------------------------------------------------

------------------------------------------------------------

------------------------------------------------------------

------------------------------------------------------------

------------------------------------------------------------

------------------------------------------------------------

------------------------------------------------------------

------------------------------------------------------------

---------------------------------------------ML-------------

------------------------------------------------------------

--------------------------------GPGMVAAPT-------------DGDGCA

ATAAVLATPPG--------------------------------------SAW--------

------------------------------------------------------------

------------------------------------------------------------

------------------------------------------------------------

--------------------------------------------------------LDPR

G-----------------------------------------------------------

------------------------ARGSASRHA--AAAVCLSS-DERMASQAAHSGREPG

DVSLR---DERRWLLPASSAERNSVLFLPS-----EPTNERGGSLPRQQAGSDEAAQDAC

SRVSSDQWR----PPSADRTSSAK-------RVAVIGGGVSGIAACKVLSEDG------H

NVTVFEARGSIGGIWSH-------------------------------------------

--------TYPSTALQTPKEAYRFSDWP----WPESV-----------------------

--------------------------------PTFPSHEQVMEYLSTYVRHFNL--GARF

RLNTRVMKMTRLRVAP--------------------------------------------

-----------------GGQAGWSLILE----EQKP----------GSLQP---------

----------------------------------------------------QETVQEFD

FAVMCCGCFGDVPKLP---CFE-PGKGPEVF---QGTTLHSAQY-SELDKAGTEKLLH--

-------G---KKVVVVGYQKTSIDVAIEAAEVNR-------------------------

-----------D----------VAHASCTLLFRKPHW-----LLPRSQLLYG--------

---------------LPLSYLILSRFAEFWV---------PKPGQSRLETLK----QTLL

SPLRSLYFGLIEMYLLYNF-------PLRKYGL------------VPE-HS-FFQDLS-A

CILPQLPDSFFALIEQGSIQLQQST----QWHFTR-KGVV---LMGEKEI----------

--------------------E-ADVVLLCTGYDGASKLKSVLPC-EY-------------

--RD-------CLST-PGECLY---LYRGMLHP-EIPDVAFLGY-HEGL---SA-VQSAE

MGSRWLGKVISG-TLVLP-AG-EVMERERQHWRAYKQRQTPFFR----RVS---------

---------------CIAPVHIWHYSTLC--QDMG-------------------------

--------WNPRRKP-GFLANIFAPHSS-----------------MDYC-----------

------------------------------------------------------------

-----SPAVVADE-----------------------------------------------

---------------------------------------HVEVSFSSPACKEQALVQEPS

---ARQC-----------------------------------------------------

--------LHHWAAE---------------------------------------------

-----------------------------K----LLQVCGWWL-VL---------E----

------------------------------------------------------------

------------------------------------AVVTGALRGKGSRQA---------

------------------------------------------------------------

------------------------------------------------------------

------------------------------------------------------------

------------------------------------------------------------

------------------------------------------------------------

------------------------------------------------------------

------------------------------------------------------------

------------------------------------------------------------

------------------------------------------------------------

------------------------------------------------------------

------------------------------------------------------------

------------------------------------------------------------

------------GPPVCGAK----------------------------------------

------------------------------------------------------------

------------ED----------------------------------------------

------------------------------------------------------------

------------------------------------------------------------

------------------------------------------------------------

------------------------------------------------------------

------------------------------------------------------------

------------------------------------------------------------

------------------------------------------------------------

------------------------------------------------------------

------------------------------------------------------------

------------------------------------------------------------

------------------------------------------------------------

------------------------------------------------------------

------------------------------------------------------------

-----------------------------

>jgi|Spimu1_1|21718|SM000006S19534

T--RGAT-----EHRNRAWPDWHGLQ----------------------------------

------------------------------------------------------------

-------------AEEKQ------------------------------------------

-------------STDC---QT---------------------------DPTRHK-----

-----AWGRAETR---PGVQI---------------------------------------

-----CPPPPPPPPPPP------------SPPPTARAAAPARP-----------------

------DE-------------------------------AP-------------------

-------------------------------------------------------PSSSD

RRRCHGIA-----------------------------------VASLRRL----------

------------------------------------------------------------

-------------------PA---------------------------------------

------------------------------------------------------------

---------------------------WPLL-----RALRAC------------------

-------------------------RGA-----------LAGRGNKL-------------

------------------------------------------------------------

--------------------------------GLRMVAAPA-------------DGEGCA

AAAAVLATPAG--------------------------------------SVW--------

------------------------------------------------------------

------------------------------------------------------------

------------------------------------------------------------

--------------------------------------------------------PDSG

G-----------------------------------------------------------

------------------------AQGSASRHA--AAAVCSSSDDERMAFQTAHSGREPG

DVPLR---DEHRWILPASSAERNSELRLPS-----EPANERGGSLPCQQAVSDEAAQDAC

CRGSSEQWR----TPSADSTLSAK-------RVAVIGGGVSGIAACKVLTEDG------H

NVTVFEARGSIGGIWSH-------------------------------------------

--------TYPSTALQTPKEGYRFSDWP----WPDSV-----------------------

--------------------------------PTFPTHEQVMEYLSTYVRHFKL--GARF

RLNTRVMKMTRLPVAP--------------------------------------------

-----------------GGQAGWSLMLE----ERRP----------GSLQP---------

----------------------------------------------------QEIVQEFD

FVVMCCGCFGDVPKLP---YFE-PGKGPEVF---QGTTLHSAQY-SELDKVGTEKLLH--

-------G---KKVVVVGYQKTSIDVAIEAAEVNR-------------------------

-----------D----------VAHASCTLLFRKPHW-----LLPRSQLLYG--------

---------------LPLSYLILSRFAEFWV---------SKPDQSQLQTLK----QTLL

SPLRSLYFGLIEMYLLYNF-------PLRKYGL------------VPE-HS-FFQDLS-A

CILPQLPDSFFSLIEQGSIQLQQSR----LWHFTR-KGVA---LMGEKEI----------

--------------------E-ADVVLLCTGYDGASKLKSVLPC-VY-------------

--RD-------CLST-PGECLY---LYRGMLHP-EIPDVAFLGY-HEGL---SA-IQSAE

MGSRWLGKVTSG-TLVLP-AG-EVMERERQHWRAYKQRQTPFFR----RVS---------

---------------CIAPVHIWHYSTLC--QDMG-------------------------

--------WNPRRKP-GFLANIFVPHSS-----------------VDYC-----------

------------------------------------------------------------

-----SPAVVADDE----------------------------------------------

---------------------------------------HVAVSFNSPACKEQALVQEPS

---AVQC-----------------------------------------------------

--------LHHWATE---------------------------------------------

-----------------------------R----LLQVCGWWL-VL---------E----

------------------------------------------------------------

------------------------------------ATFTGALRGKGSRQA---------

------------------------------------------------------------

------------------------------------------------------------

------------------------------------------------------------

------------------------------------------------------------

------------------------------------------------------------

------------------------------------------------------------

------------------------------------------------------------

------------------------------------------------------------

------------------------------------------------------------

------------------------------------------------------------

------------------------------------------------------------

------------------------------------------------------------

------------GIPVCGAK----------------------------------------

------------------------------------------------------------

------------ED----------------------------------------------

------------------------------------------------------------

------------------------------------------------------------

------------------------------------------------------------

------------------------------------------------------------

------------------------------------------------------------

------------------------------------------------------------

------------------------------------------------------------

------------------------------------------------------------

------------------------------------------------------------

------------------------------------------------------------

------------------------------------------------------------

------------------------------------------------------------

------------------------------------------------------------

-----------------------------

>jgi|Spimu1_1|2272|SM000116S24242

------------------------------------------------------------

------------------------------------------------------------

------------------------------------------------------------

------------------------------------------------------------

------------------------------------------------------------

------------------------------------------------------------

------------------------------------------------------------

------------------------------------------------------------

------------------------------------------------------------

------------------------------------------------------------

------------------------------------------------------------

------------------------------------------------------------

------------------------------------------------------------

---------------------------------------------ML-------------

------------------------------------------------------------

--------------------------------GAGMIDAPA-------------DGDSCA

AAAAVLATPAG--------------------------------------SAW--------

------------------------------------------------------------

------------------------------------------------------------

------------------------------------------------------------

--------------------------------------------------------PDRG

G-----------------------------------------------------------

------------------------AGGSASRLA--AAAVCLSSDDGRMAFQTAHFGREPG

DVPLR---DEHRWLLSASSAERDSVLRLPS-----E---RRGGSLPCQQAVSDEAAQDAC

GRVSSDQWR----TPSADSTPPAK-------RVAVVGGGVSGIAACKVLSEDG------H

DVTVFEARGSIGGIWSH-------------------------------------------

--------TYPSTALQTPKEAYRFSDWP----WPESV-----------------------

--------------------------------PTFPSHEQVMDYLSTYVRHFNI--RARF

RLNTRVMKMTRLP-----------------------------------------------

---------------------GWSLMLE----ERIP----------VSLQP---------

----------------------------------------------------QETVQEFD

FVVMCCGCFGDVPKLP---SFE-PGKGPQVF---QGTTLHSAQY-SELDKAGTEKLLH--

-------G---KKVVVVGYQKTSIDVAIEAAEINR-------------------------

-----------D----------VAQASCTLLFRKPHW-----LLPRSQLLYG--------

---------------LPLSYLILSRFAEFWV---------PKPDQSGLETLK----QTVL

SPLRSLYFGLIEMYLLYNF-------PLRKYGL------------VPE-HS-FFQDLS-A

CILPQLPDSFFSLIEQGSIQLRQST----QWHFTR-KGVA---LMGEKEI----------

--------------------E-ADVVLLCTGYNGASKLKSVLPC-EY-------------

--RD-------CLST-PGECLY---LYRGMLHP-EIPDVAFLGY-HEGL---SA-VQSAE

MGSRWLGKVISG-TLVLP-AD-EVMERERQHWRAYKKRQTPFFR----RVS---------

---------------CIAPVHIWHYSTLC--QDMG-------------------------

--------WNPRRKP-GFLANIFVPHSS-----------------VDYC-----------

------------------------------------------------------------

-----SPAVVADDE----------------------------------------------

---------------------------------------HVGVSFSSPACKEQALVQEPS

---AMQC-----------------------------------------------------

--------LHHWATV---------------------------------------------

-----------------------------K----LLQVCGWWL-VL---------E----

------------------------------------------------------------

------------------------------------AIVAGALRGKGSRQA---------

------------------------------------------------------------

------------------------------------------------------------

------------------------------------------------------------

------------------------------------------------------------

------------------------------------------------------------

------------------------------------------------------------

------------------------------------------------------------

------------------------------------------------------------

------------------------------------------------------------

------------------------------------------------------------

------------------------------------------------------------

------------------------------------------------------------

------------GPPVCGAK----------------------------------------

------------------------------------------------------------

------------EH----------------------------------------------

------------------------------------------------------------

------------------------------------------------------------

------------------------------------------------------------

------------------------------------------------------------

------------------------------------------------------------

------------------------------------------------------------

------------------------------------------------------------

------------------------------------------------------------

------------------------------------------------------------

------------------------------------------------------------

------------------------------------------------------------

------------------------------------------------------------

------------------------------------------------------------

-----------------------------

>jgi|Meskra658_1|1272703|estExt_Genewise1.C_Chr_12_t20322

------------------------MALAN----------------LCMELATP-------

-----------------------------------------------------------K

ICTNF--------YDEA-------------------------------------------

-------------FSTC---RR---------------------------SP---------

-----HIAAAS-------------------------------------------------

----------------------------------LQAATLQFP-----------------

------------------------------QFGTGSEPLAP-------------------

-------------------------------------------------------SNSAL

QRNGR----------------------------------------SFKR-----------

------------------------------------------------------------

-------------------PH---------------------------------------

------------------------------------------------------------

------------------------------------------------------------

---------------------------------------RVFIHCTL-------------

------------------------------------------------------------

-------------AGDFTQD----------------YSSPANQQKIDNQEL---TGVNGI

ANAGHAAVPHF-------------------------------------------------

------------------------------------------------------------

------------------------------------------------------------

------------------------------------------------------------

------------------------------------------------------------

--------------------HSSGNKVATYADSAANGQ----------------------

-----------------------GIHHSAGRAGLDASSMNTGLSSGAVTRVSAT------

--------------SSIGLSE-----AWPGIGGLDERIDRRGHPLGRTEELNGVDSGR--

PRFGTPGDV----SAKATKLDPDS-------KVAIIGSGVSGIAACKALTEAG------I

DVTVFEARERLGGIWSR-------------------------------------------

--------TYPTTSLQTPRTAYRFSDWD----WPVDA-----------------------

--------------------------------PMNPRHDQVVEYLQGYIEHFQL--GDKF

KFNSRIAEMQRPEGGR--------------------------------------------

--------------------RGWRLRIQ----RKRTNED-------GSLGEV--------

----------------------------------------------------EEQWLQFD

FVVNCCGCFGDVPNIP---QFA-AGEGPDVF---GGTVMHSAQY-SGLGPDGCEALLR--

-------G---KRVVVVGFQKTAIDVAIEAAEANK-------------------------

-----------G----------SDGKPCTLLFRKPHW-----LVPRNQLIFG--------

---------------VPLKYLLLTRFSELFH---------RTPNQSPLLTAL----QTVL

-PLRALVGKLVEAYLCGKY-------PLQRHGL------------VPS-HS-LVDDVA-A

CILPQMPPAFFPLVEAGRVQLARAP----RWRFTP-AGVE---LDDGRQL----------

--------------------A-ADVVLLCTGYDALAKLRGLLPA-PY-------------

--AG-------QLSS-NGRSLY---LYRGVIHP-RVPNMAFLGF-HEGA---SS-VQTSE

MGARWLAKLLQG-AFALP-DE-GAMERDRREWWEFKRAQSPFA-G---RVS---------

---------------CVAPIHSWHFDLLC--RDMG-------------------------

--------WSPWRKK-GWVANLFSPHTS-----------------ADYV-----------

------------------------------------------------------------

-----EREADRRATAEEKR-----------------------------------------

----------------------AAH---GG--PS-SE-VKVV------------------

------------------------------------------------------------

------------------------------------------------------------

------------------------------------------------------------

------------------------------------------------------------

------------------------------------------------------------

------------------------------------------------------------

------------------------------------------------------------

------------------------------------------------------------

------------------------------------------------------------

------------------------------------------------------------

------------------------------------------------------------

------------------------------------------------------------

------------------------------------------------------------

------------------------------------------------------------

------------------------------------------------------------

------------------------------------------------------------

------------------------------------------------------------

------------------------------------------------------------

------------------------------------------------------------

------------------------------------------------------------

------------------------------------------------------------

------------------------------------------------------------

------------------------------------------------------------

------------------------------------------------------------

------------------------------------------------------------

------------------------------------------------------------

------------------------------------------------------------

------------------------------------------------------------

------------------------------------------------------------

------------------------------------------------------------

------------------------------------------------------------

------------------------------------------------------------

------------------------------------------------------------

-----------------------------

>jgi|Mesen1_1|1395|ME000013S00885

------------------------MP----------------------------------

------------------------------------------------------------

------------------------------------------------------------

------------------------------------------------------------

------------------------------------------------------------

------------------------------------------------------------

------------------------------------------------------------

------------------------------------------------------------

------------------------------------------------------------

------------------------------------------------------------

------------------------------------------------------------

------------------------------------------------------------

------------------------------------------------------------

------------------------------------------------------------

------------------------------------------------------------

------------------------------------------------------------

------------------------------------------------------------

------------------------------------------------------------

------------------------------------------------------------

------------------------------------------------------------

------------------------------------------------------------

------------------------------------------------------------

------------------------------------------------------------

------------------------------------------------------------

-------------------------------EVAIIGAGVSGLAVCKTLTEAG------V

PVVVYEARGSIGGIWSN-------------------------------------------

--------TYASTTLQTPRSSYCFSDWP----WPDDA-----------------------

--------------------------------PMHPSHDQVIHYLQGYIDHFRL--RSKI

SLSSKVLEMKRATAGD--------------------------------------------

---------------------SWLV-------------D-------G-------------

------------------------------------------------------------

---------GGYAPLV---VHN-TGKGTETR-----------------------------

-------G----------------------------------------------------

-----------G----------PGGVACTLLFRRAHW-----IVPRSGRVFG--------

---------------IPLPFFFLTRLSELGL---------PKPGQHPLVALL----QRLS

-PMRWVTSKLVELYMVRTY-------PLRKHGL------------VPS-HS-FSQDLA-A

CILPQLPDKFFPLIEQGRILLKRSA----SWHFTP-AGMR---LENGEEL----------

--------------------A-ADVVLLCTGYNGLAKVKAMLPE-PY-------------

--SA-------LLEE-EGESLH---LYRGLVHP-RIPDVAFLGF-HEGL---SS-MHVAE

MGARWLTALLRG-GFSLP-SA-AAMDAERAEWRRYKRAQTPFSQG---RVS---------

---------------CIAPLHVWHFDALC--RDMG-------------------------

--------WPLMRKR-GLLQNLLLPYGS-----------------VDYR-----------

------------------------------------------------------------

-----APAPPSASPAP--------------------------------------------

-----------------------------G--PALTP-VPVVVTLATGAADAAAAKADKE

---ARSV-----------------------------------------------------

--------HAHYQEQ---------------------------------------------

-----------------------------RTA----------------------------

------------------------------------------------------------

-----------------------------------RGAAAALASATASLTAAAA------

------------------------------------------------------------

------------------------------------------------------------

------------------------------------------------------------

------------------------------------------------------------

------------------------------------------------------------

------------------------------------------------------------

------------------------------------------------------------

------------------------------------------------------------

------------------------------------------------------------

------------------------------------------------------------

------------------------------------------------------------

------------------------------------------------------------

------------MTPATATE----------------------------------------

------------------------------------------------------------

------------------------------------------------------------

------------------------------------------------------------

------------------------------------------------------------

------------------------------------------------------------

------------------------------------------------------------

------------------------------------------------------------

------------------------------------------------------------

------------------------------------------------------------

------------------------------------------------------------

------------------------------------------------------------

------------------------------------------------------------

------------------------------------------------------------

------------------------------------------------------------

------------------------------------------------------------

-----------------------------

>jgi|Zygcir1559_1|6120|UTEX1559_15026.1

------------------------MHRRVRGS--PCRQAEGCVCAFCSDGALPASHVAT-

--------RSVSIAATAAAVGVGGGRGGEASCAEDGGRRKMAAVQREGGNSRGPPSLL-S

LCVAFLGCHLDELIDEAHHFVPAFPTPVRQALAGIARRRGLMCDELLRALIDDSWRVLDV

SGSAVTDDGIRCAADTC---SRLEAVDLSGCDDVTVEALQR----LVEANPQLHTIRFGG

TPASDAVARGSLRHIIPGISVMEAEREEGSWEDVDVEHVGRGAQALRWLVWPTIDEKSLR

RIESKCPRIRINPKKPEYWTS-----IVRTPPEALQQFDLDAPFVEGIDPITWSDGKDWR

RLNENCDENGDLGCDVSSFSDLSLAERFKLAYKLRDERLAPKRAKNLRQKLRRAERAWLD

ENGEARARVLAGTICKFQKVQKLGILTKVDDREETIVGLNIGDAKDSTLFTGGPLSSSSR

SQEERPIQQLADETRMKIFRFAKRSGTEHCNSAMNALFTLLHTLESLERGSRRESQQVMR

EAIDRAVAAEQEGANTPLNLQCTILLLGKSGAGKSTVVNIFLGGPIADASSNKGTKAVRE

YRQRVHGVDFTLIDTPGLQPASSDRKMNEKMLSSLSKYLQRKKKKIDLILYCERLDTKVE

EISELQILLTISSILGSRSW-LRTSILFTHALTPIGANFNEQDLHPEYAAERKSHIFRRL

ISEAAGPSLDMGLNPFALIDANDKMEESPLAGKWLPNIFSMCCALKLLNEVEEKATTHHS

YLAWDGEQREWESLFSGEGEGEGEGEGERE------DFWKGSLACLLDEELSSIRDSVVG

RKEATLRNGASEGFFPHTENLRRPNTSGEEGSKDRQLGFEEGDDLQSQQSPYSQPVKIQR

SFRILSDKIAVLLKGAFSANKTKGIEAEFAAHGAGMYGPRECQGSLQLQKLPWIDCYGCA

ARAAIRAKPT----------------------------------------AWNCVGVGAT

ALFADDGTGQTEKRERTAMGLKIEDSLQLSENIFISAAAGVVGARREIAKGAQIEADICN

GARSGRDNVPFKEREMLQRGRVGLGIMLLKAAGRVVTSGNLRTDI------TCEGGLRVQ

AGACMTSSGYGHIAASVLSHGLPEAVPLLLLPAGYGALWASRFFSGTGGERREEGGEREQ

EGVRGWLREASDRPERRVADLQPATLLACFKMGHGLSLRSDSLLG----FLERKGRVGPG

GDP-------------MW------PGVSPNGRVAGNGA----------------------

-----------------------QCSDSSSSP---ASHVPSAAISGPFTDSESTQLEQKS

FL------------KSLSSAE-----YWPS----------------HLKEFPGAAHAREA

ARRGKLKDAPSTHFSGRNALEKGS-------RVAVVGAGVSGMVSAKLLMEAG------Y

EVTVFEAREKIGGIWAR-------------------------------------------

--------TYASTSLQTPRIAYRFSDWE----WPDSA-----------------------

--------------------------------PMNPSHREVVEYLQGYANHFGV--YNKI

RFNTRIVEMQEAEGSE--------------------------------------------

--------------------RGWRLRVK----GGES----------GGRDGE--------

----------------------------------------------------TELWLHYD

FVVMCCGCFSGVPHIP---KFE-SGKGLEAF---KGTTMHSAQY-SELDEAASRSLLH--

-------G---KKVVVVGFQKTAIDVVLESAEINR-------------------------

-----------G----------SESAQSTLLFRKTHW-----LVPRDQMVCG--------

---------------VHLKYILLTRFVELFF---------PYPQQGILRTIL----QKLV

-PVRQVLNSMVERYLKQRY-------PLEKYNL------------IPS-HS-FHDDVS-A

CILPQLPPTFFPLVEEGSIRLQKSA----TWHFTS-AGIA---LEDGRQL----------

--------------------E-ADVVVLCTGYDGLGKLRGLLPP-HY-------------

--GS-------KLAS-PGQSLS---LYRGVLHP-RIPNMAFIGF-HEGL---SS-MHSAE

MGARWLSKLLQG-AFSLP-EA-VAMEAECSKWWTFKNSQTPFAG----RVS---------

---------------CVAPIHMWHFDALC--RDMG-------------------------

--------WDPMRKK-GFVANWFTPHGS-----------------VDYV-----------

------------------------------------------------------------

-----ETAPSVRDSPEEEK-----------------------------------------

----------------------------------LRE-REELLPLTAVPSSRDVAKPNFE

---GGSF-----------------------------------------------------

--------AEEFYNM---------------------------------------------

-----------------------------DKLAYTYSGQGEPA-GKQG------LQGPLP

LQPSGKARLAA-------------------------------GAG---------------

-----------------------------------AGAGAGAGAGAASAVAALCCILLLL

HL-SGAAADVYVANMTGLDVVG------YPAPVPETYEGTAVLRIQDTGRIDYVFTQTDL

DEIQE-----------------------VWLKEPVPG-----------------------

--------------------------DPINGQTI-FNFNLTRVQLAAFTENGSLSGFID-

--------------------VDPYLAIGVT------------------------------

------------------------------------------------------------

-----QRPYQFYISVKTSK----------------------Y--------SQGAIKGFLR

DTTPSPTTGLEPNWLIDTNMTAEEVVPKAGPEN---------------------------

--------GYTTG--------------------------------LFSVFIQS-------

---------------------------------------N-------GSLYVEFDFMDLN

----------------------------------------------------FTGLTSV-

------------------GLYT--------------------------------------

--------------GKKGAK-------------------------GTLVYD---------

-------MTPHVNLPAFASN----------------------------------------

------------------------------------------------------------

------------EFT---------------------------------------------

------------------------------------------------------------

------------------------------------------------------------

------------------------------------------------------------

------------------------------------------------------------

------------------------------------------------------------

------------------------------------------------------------

------------------------------------------------------------

------------------------------------------------------------

-------------------AGAFVTTKE------------------VLFPILSNFNQSYC

QIASSGYPSGASRG----------------QL----------------------------

----------------------------------HLVKVNLQS-----------------

----------------------------------PP------------------------

---SDAPVGYLQDSPPP---------------SGACRRREWEEWRLTL------SVLSSC

LLCLLGAPWL-----------------LG

>jgi|Zygcir1560_1|6463|UTEX1560_14885.1

------------------------MHRRVRGS--PCRQAEGCVCAFCSDGALPASHVAT-

--------RSVRIAATAAAVGVGGGRGGEASCAEDGGRRKMAAVQREGGNSRGPPSLL-S

LCVAFLGCHLDELIDEAHHFVPAFPTPVRQALAGIARRRGLMCDELLRALIDDSWRVLDV

SGSAVTDDGIRCAADTC---SRLEAVDLSGCDDVTVEALQR----LVEANPQLHTIRFGG

TPASDAVARGSLRQIIPGISVMEAEREEGSWEDVDVEHVGRGAQALRWLVWPTIDEKSLR

RIESKCPRIRINPKKPEYWTS-----IVRTPPEALQQFDLDAPFVEGIDPITWSDGKDWR

RLNENCDENGDLGCDVSSFSDLSLAERFKLAYKLRDERLAPKRAKNLRQKLRRAERAWLD

ENGEARARVLAGTICKFQKVQKLGILTKVDDREETIVGLNIGDAKDSTLFTGGPLSSSSR

SQEERPIQQLADETRMKIFRFAKRSGTEHCNSAMNALFTLLHTLESLERGSRRESQQVMR

EAIDRAVAAEQEGANTPLNLQCTILLLGKIGAGKSTVVNMFLGGPIADASSNKGTKAVRE

YRQRVHGVDFTLIDTPGLQPASSDRKMNEKMLSSLSKYLQRKKKKIDLILYCERLDTKVE

EISELQILLTISSILGSRSW-LRTSILFTHALTPIGANFNEQDLHPEYAAERKSHIFRRL

ISEAAGPSLDMGLNPFALIDANDKMEESPLAGKWLPNIFSMCCALKLLNEVEEKATTHHS

YLAWDGEQREWESLFSGEGEGEGEGEGERE------DFWKGSLACLLDEELSSIRDSVVG

RKEATLRNGASEGFFPHTENLRRPNTSGEEGSKDRQLGFEEGDDLQSQQSPYSQPVKIQR

SFRILSDKIAVLLKGAFSANKTKGIEAEFAAHGAGMYGPRECQGSLQLQKLPWIDCYGCA

ARAAIRAKPT----------------------------------------AWNCVGVGAT

ALFADDGTGQTEKRERTAMGLKIEDSLQLSENIFISAAAGVVGARREIAKGAQIEADICN

GARSGRDNVPFKEREMLQRGRVGLGIMLLKAAGRVVTSGNLRTDI------TCEGGLRVQ

AGACMTSSGYGHIAASVLSHGLPEAVPLLLLPAGYGAL----------------------

------------------------TLLACFKMGHGLSLRSDSLLG----FLERKGRVGPG

GDP-------------MWAHHFTGPGVSPNGRVAGNGA----------------------

-----------------------QCSDSSSSP---ASHVPSAAISGPFTDSESTQLEQKS

FL------------KSLSSAE-----YWPS----------------HLKEFPGAAHAREA

ARRGKLKDAPSTHFSGRNALEKGS-------RVAVVGAGVSGMVSAKLLMEAG------Y

EVTVFEAREKIGGIWAR-------------------------------------------

--------TYASTSLQTPRIAYRFSDWE----WPDSA-----------------------

--------------------------------PMNPSHREVVEYLQGYANHFGV--YNKI

RFNTRIVEMQEAEGSE--------------------------------------------

--------------------RGWRLRVK----GGES----------GGRDGE--------

----------------------------------------------------TELWLHYD

FVVMCCGCFSGVPHIP---KFE-SGKGLEAF---KGTTMHSAQY-SELDEAASRSLLH--

-------G---KKVVVVGFQKTAIDVVLESAEINR-------------------------

-----------G----------SESAQSTLLFRKTHW-----LVPRDQMVCG--------

---------------VHLKYILLTRFVELFF---------PYPQQGILRTIL----QKLV

-PVRQVLNSMVERYLKQRY-------PLEKYNL------------IPS-HS-FHDDVS-A

CILPQLPPTFFPLVEEGSIRLQKSA----TWHFTS-AGIA---LEDGRQL----------

--------------------E-ADVVVLCTGYDGLGKLRGLLPP-HY-------------

--GS-------KLAS-PGQSLS---LYRGVLHP-RIPNMAFIGF-HEGL---SS-MHSAE

MGARWLSKLLQG-AFSLP-EA-VAMEAECSKWWTFKNSQTPFAG----RVS---------

---------------CVAPIHMWHFDVLC--RDMG-------------------------

--------WDPMRKK-GFVANWFTPHGS-----------------VDYV-----------

------------------------------------------------------------

-----ETAPSVRDSPEEEK-----------------------------------------

----------------------------------LRE-REELLPLTAVPSSRDVAKPNFE

---GGSF-----------------------------------------------------

--------AEEFYNM---------------------------------------------

-----------------------------DKLAYTYSGQGEPA-GKQG------LQGPLP

LQPSGKARLA--------------------------------------------------

-------------------------------------AGAGAGGGAASAVAALCCILLLL

HL-SGAAADVYVANMTGLDVVG------YPAPVPETYEGTAVLRIQDTGRIDYVFTQTDL

DEIQE-----------------------VWLKEPVPG-----------------------

--------------------------DPINGQTI-FNFNLTRVQLAAFTENGSLSGFID-

--------------------VDPYLAIGVT------------------------------

------------------------------------------------------------

-----QRPYQFYISVKTSK----------------------Y--------SQGAIKGFLR

DTTPSPTTGLEPNWLIDTNMTAEEVVPKAGPEN---------------------------

--------GYTTG--------------------------------LFSVFIQS-------

---------------------------------------N-------GSLYVEFDFMDLN

----------------------------------------------------FTGLTSV-

------------------GLYT--------------------------------------

--------------GKKGAK-------------------------GTLVYD---------

-------MTPHVNLPAFASN----------------------------------------

------------------------------------------------------------

------------EFT---------------------------------------------

------------------------------------------------------------

------------------------------------------------------------

------------------------------------------------------------

------------------------------------------------------------

------------------------------------------------------------

------------------------------------------------------------

------------------------------------------------------------

------------------------------------------------------------

-------------------AGAFVTTKE------------------VLFPILSNFNQSYC

QIASSGYPSGASRG----------------QL----------------------------

----------------------------------HLVKVNLQS-----------------

----------------------------------PP------------------------

---SDAPVGYLQDSPPP---------------SGA-------------------------

-----------------------------

>jgi|Zygcir6981b_2|7494|Zci_13903.1

------------------------MAQAV----------------IWVNSALVAS-----

------------IASSASVY----------------------------------------

------------------------------------------------------------

-------------TSSC---NR---------------------------NPK--------

-----AVALAS-------------------------------------------------

--------------------------------DFLQPLHLLSP-----------------

------------------------------------------------------------

---EAR-------------------------------------------------SGSSE

RQRER------------------------------------------QRLRQREQQ----

------------------------------------------------------------

-RER--------------------------------------------------------

--------------------------------------------HRERQKQGGSH-----

---------------------------SP-------------------------------

--------------------------------------------CIL-------------

------------------------------------------------------------

------------------------------------------------------------

------------------------------------------------------------

------------------------------------------------------------

------------------------------AAAR---EGGASTD----------------

------------------------------------------------------------

---------------------------------------------------------GPG

G-----------------AHHFTGPGVSPNGRVAGNGA----------------------

-----------------------QCSDSSSSP---ASHVPSAAISGPFTDSESTQLEQKS

FL------------KSLSSAE-----YWPS----------------HLKEFPGAAHAREA

ARRGKLKDAPSTHFSGRNALEKGS-------RVAVVGAGVSGMVSAKLLMEAG------Y

EVTVFEAREKIGGIWAR-------------------------------------------

--------TYASTSLQTPRIAYRFSDWE----WPDSA-----------------------

--------------------------------PMNPSHREVVEYLQGYANHFGV--YNKI

RFNTRIVEMQEAEGSE--------------------------------------------

--------------------RGWRLRVK----GGES----------GGRDGE--------

----------------------------------------------------TELWLHYD

FVVMCCGCFSGVPHIP---KFE-SGKGLEAF---KGTTMHSAQY-SELDEAASRSLLH--

-------G---KKVVVVGFQKTAIDVVLESAEINR-------------------------

-----------G----------SESAQSTLLFRKTHW-----LVPRDQMVCG--------

---------------VHLKYILLTRFVELFF---------PYPQQGILRTIL----QKLV

-PVRQVLNSMVERYLKQRY-------PLEKYNL------------IPS-HS-FHDDVS-A

CILPQLPPTFFPLVEEGSIRLQKSA----TWHFTS-AGIA---LEDGRQL----------

--------------------E-ADVVVLCTGYDGLGKLRGLLPP-HY-------------

--GS-------KLAS-PGQSLS---LYRGVLHP-RIPNMAFIGF-HEGL---SS-MHSAE

MGARWLSKLLQG-AFSLP-EA-VAMEAECSKWWTFKNSQTPFAG----RVS---------

---------------CVAPIHMWHFDALC--RDMG-------------------------

--------WDPMRKK-GFVANWFTPHGS-----------------VDYV-----------

------------------------------------------------------------

-----ETAPSVRDSPEEEK-----------------------------------------

----------------------------------LRE-REELLPLTAVPSSRDVAKPNF-

------------------------------------------------------------

------------------------------------------------------------

------------------------------------------------------------

------------------------------------------------------------

------------------------------------------------------------

------------------------------------------------------------

------------------------------------------------------------

------------------------------------------------------------

------------------------------------------------------------

------------------------------------------------------------

------------------------------------------------------------

------------------------------------------------------------

------------------------------------------------------------

------------------------------------------------------------

------------------------------------------------------------

------------------------------------------------------------

------------------------------------------------------------

------------------------------------------------------------

------------------------------------------------------------

------------------------------------------------------------

------------------------------------------------------------

------------------------------------------------------------

------------------------------------------------------------

------------------------------------------------------------

------------------------------------------------------------

------------------------------------------------------------

------------------------------------------------------------

------------------------------------------------------------

--------------------GELVET----------------------------------

------------------------------------------------------------

------------------------------------------------------------

------------------------------------------------------------

------------------------------------------------------------

-----------------------------

>jgi|Rhomari1|33|NDN08_005046.1

------------------------MI----------------------------------

------------------------------------------------------------

------------------------------------------------------------

------------------------------------------------------------

------------------------------------------------------------

------------------------------------------------------------

------------------------------------------------------------

------------------------------------------------------------

------------------------------------------------------------

------------------------------------------------------------

------------------------------------------------------------

------------------------------------------------------------

------------------------------------------------------------

------------------------------------------------------------

------------------------------------------------------------

------------------------------------------------------------

------------------------------------------------------------

------------------------------------------------------------

------------------------------------------------------------

------------------------------------------------------------

------------------------------------------------------------

------------------------------------------------------------

------------------------------------------------------------

------------------------------------------------------------

----------------VREAKRGF-------RVAVIGAGTAGVATTRVLSEQG------F

DCVVFEKHSKSGGLWAD-------------------------------------------

--------NYYGASVQMPKSLYRFPDEV----HAAEA-----------------------

--------------------------------NEFLGKDEVCDYLERIIDKHEI--RKCF

RFNTLVTEMKQLEGG---------------------------------------------

---------------------IWRLTVK----DLKTD-----------------------

----------------------------------------------------VSNDETFD

YLVMCNGTFSSKPHRP---NFP----GQEKF---QGKIMHSSEW-RSPD------EFA--

-------G---KKIVIVGMGKSAVDVSMVASEV---------------------------

------------------------GESAVQLVRSMHW-----FTTSHV------------

-------------GFIPTDMVTFTRLFQMMLPP--------FYSSSSMYKNL----HKLA

APVRWLYFRTIEALMLTNY-------RLPR-NL------------WPK-SR-FEFDVL-H

PALFNGPAEYLADVRSGRINLKVTA----VNEIKE-KTVV---LSTGEEV----------

--------------------E-ADLIVLATGWD---KTIGLFND-EV-------------

--LN-------RLEV-EEDGIW---LYKNILPP-GIDRMAFIGS-NAFTA--IN-PMTHM

VQAKWLAALLTN-AMPRP-SH-KEMVLDIWDMRQSRRAITPMNAM---RAS---------

---------------CIQLHMQSYHDDLL--KDMN-------------------------

--------VNIRKRS----------------------GLMGWIEHYFYR-----------

------------------------------------------------------------

------------------------------------------------------------

------------------------------------------------------------

------------------------------------------------------------

------------------------------------------------------------

------------------------------------------------------------

------------------------------------------------------------

------------------------------------ITPAG-------------------

------------------------------------------------------------

------------------------------------------------------------

------------------------------------------------------------

------------------------------------------------------------

------------------------------------------------------------

------------------------------------------------------------

---------------------ENEVVK---------------------------------

-----------------------------------R------------------------

---CP-------------------------------------------------------

------------------------------------------------------------

------------------------------------------------------------

------------------------------------------------------------

--------------PTA-------------------------------------------

------------------------------------------------------------

------------------------------------------------------------

------------------------------------------------------------

------------------------------------------------------------

------------------------------------------------------------

------------------------------------------------------------

------------------------------------------------------------

------------------------------------------------------------

------------------------------------------------------------

------------------------------------------------------------

------------------------------------------------------------

------------------------------------------------------------

------------------------------------------------------------

------------------------------------------------------------

------------------------------------------------------------

-----------------------------

>jgi|Plesta4479_1|7928|GLC49954.1

------------------------MP----------------------------------

------------------------------------------------------------

------------------------------------------------------------

------------------------------------------------------------

------------------------------------------------------------

------------------------------------------------------------

------------------------------------------------------------

------------------------------------------------------------

------------------------------------------------------------

------------------------------------------------------------

------------------------------------------------------------

------------------------------------------------------------

------------------------------------------------------------

------------------------------------------------------------

------------------------------------------------------------

------------------------------------------------------------

------------------------------------------------------------

------------------------------------------------------------

------------------------------------------------------------

------------------------------------------------------------

------------------------------------------------------------

------------------------------------------------------------

------------------------------------------------------------

------------------------------------------------------------

-------------------------------TTVIIGAGISGLQCASAFLRLG------H

TVTILEREEDVGGVWL--------------------------------------------

--------RYTGFNAHVPREFFCFPDFA----PPPDP-----------------------

--------------------------DPDLDPEEYPTGRSLLRHVIAYAHRNGL--YRHI

IFRATVTRLHRLGG----------------------------------------------

---------------------QWQCFFDLA--TGGRGAGG---GAGGGGGGGGGGGA---

----------------------------RGGGGGLGEVQQCGVPQRHQQQQQQQQRISAD

YVVVATGLHSS-PNVP---VLD----GQHLF---RGRLLHVQDL-PPND-LELGQQFG--

-------G---RRVVVVGGTKTAVDTATRLVEA---------------------------

-----------------------GAEDTFVVFRQAHW-----LIPRFP-LRG--------

---------------MHFTQLLHSRLTAAML--------NPYYDAGLWGRVK----GGIS

RPLRRLFWRTVERSLVRQQ-------HLYG-DL------------LPS-MP-LDKDLL--

SGGVVHDGRWRQYLEEGAVHAVRGE----PLRLTP-GGLI---LRSGEFI----------

--------------------A-ADVVIMATGHL---KDYSFIDS-DI-------------

--LS-------KLGV-QRDGLY---LYRHVLPP-LVRNIAFVGA-ANST---NN-PLTSY

LQTKWLVACLQN-RVRLP-EP-HDMIRDLQRQTRWQRRKLPPQAD---RAS---------

---------------MIGIYAHHYHDRLL--KDMG-------------------------

--------LPIRRKK-NLVRDCFEPYTA-----------------ADFA-----------

------------------------------------------------------------

-----DIADVLAGDRRRQQSKDVAAAAAEVTAEVTATAGPAAAAGPPPPPLTTSMSMGLG

SSFKLSA-ASAATPSEVYDVTAAEHSVGFGLMPSMRRTAMAAPSRGSTAAETAAAAAAAV

---AAAA---------------GADGE---------------------------------

-----GEGDARWAYPPPQESSGPGSYPGGYVAAVGSLPGPGPGPGPGPAAGVGAAGQAAG

AGAGPATAPSMLYGA---RPGTAGAAYSNRLLGTLYEQEGGGAPGLHGRGALATRSGPGQ

----GPGRLGLAPAPALSSSLTPPTPPTPPDTPSQLMSPTSYGAGFRSVGRPAIYVPPSS

GRSCLMDAAALALMDGVVGGALLDRP----PKPGAAAAAAAEQQQQQQRATSTSGELPTA

AAGTGGAAD------GGGGPAGNRSET---AAVAVTREEVAAGAVYGTGRAHTAPAAAQM

TQVTSPAAAAAAAAAAAAAAAAAAAAAAVMSRSRRQGSGDTGGEPAASEAAAAAAGAGDG

RESSSSEALRRRRQGSLAAEAEAEAEEVMSSRN-----DAAAASTAASTA-GGGGGGGDG

GGGDARRPSRRPASARSVSGAPSFVTPALAAALAAGATAA-----AAGPPPWIESPEISE

ADGGGGDGGGGGGAACAARAEAAGASDTVRFGPSTEGGSGGGGAAAAAAPPLLRYASAGF

VANADDEAASCPSAASSESNNVVATCTRASSSSP---HSRSHSASRAGGGGGAAAAGVAG

KA------------------ASRNLVRLVTTR---HAAAAAASGADGA----------SG

PHSGGSVGAAASGSIAVRAGSSTAARSAGRV-SARR----------TSDFLDESEDFIGT

PPGCF----EGSLDAMMS--------DRGGREGGGGGGARSRIAAASSATASSRRRRRRL

TADRSTLDGDDVDEYDDEDAEYDEYDDDDDEYGYMPGGGKGREGQASDGGGGGDGGGAAG

ASGPDGKVAAVAGDHAGGGTTGAGRIGAATASAAGYKSPFALQPSASFGPGTTAAPALTR

SASSPRSNGSPPHRSGTGTRFAAAADGGGGGGARRTREVHPTRMSGDSVADAGNRPGGGG

GGGGGSQLRSYGTAPAAAPP--GGGGGGGGGGGGGGGSVNNASSALERYAARMTAAPTFR

MSLTNMADMKPANLNHAARASAAAAAMAAAMAAAGGASSGGGGGAAHHPAPLRPFFLQPS

AGGAGGAGGGGGSAAAAAAAAAAASGYVSHHHHTTSLHPHFAATSRLGAMRPMRVSEEEE

GDGCGELPPGVVVVGGSTDNGGGGGSGGEADDDDGGGGGGGRLSMSALMVVGGGDDDGGV

NQSCASNASFTAGGGGGGGGGGAAAAAPRRSNTTAGYGSSMQSEGYGRTPNVASVSPAFG

RGGGGAAAAAAGGGMLSSSPFGTHVSATNALPHTAGGGGGGGGFDGGGSWDMTLRQLSRA

VVAPPQRAAGVLRAALISRRNLASGGDRPSESGLSNPNVGGNAGGSFFNARRAGFADDGS

SGPVSRGSAAAAAAAAGGGGGGGIPDAQLMSVLLAPGGGGGGGGPPTGTGSGSGPVLLGA

GVQPSGPAMAPVREPRDASRFVAAMSTAAAAAAQCPPPPSDAAAPLAKSPRGSGDGGGGA

ATAASQNGAGGAADAADADAGGAFRRPATTDGGGSSSGGGGSGFVDDIGEIGGGGGGGAR

MHWSAAVAAAKELRLQSESARSAFAALAMAARARRSCTMYGAPNPSPAHESPMLAKLIAA

SQSHNGVASDGAAAAVPPHAAVHAAASSGGGGGAAAAAAAATVPRRATSP---NVVVAGG

RLSLLGNNSGGGGGGGDSRTSWDSGGGVAKSLKRLGGLLMAVPSSPPPSAVQLAAATTTG

NKSQTGSVADGGGGGGGSRLPSGSSEGGGVTPARLLRRMMSSSKRRSNEGAYAGGYIAMV

EEDNEAVALP------------------------PPPPPVAVTVAAFAGGGGGGGGSGGG

SCASTAVVSPLEAPPAEL--------------AAALFGRTTSRRSVVLYNPPV----AAP

L------------ELYASAPAAPAAPPTG

>jgi|Plesta1363_1|12791|GLC39407.1

------------------------MP----------------------------------

------------------------------------------------------------

------------------------------------------------------------

------------------------------------------------------------

------------------------------------------------------------

------------------------------------------------------------

------------------------------------------------------------

------------------------------------------------------------

------------------------------------------------------------

------------------------------------------------------------

------------------------------------------------------------

------------------------------------------------------------

------------------------------------------------------------

------------------------------------------------------------

------------------------------------------------------------

------------------------------------------------------------

------------------------------------------------------------

------------------------------------------------------------

------------------------------------------------------------

------------------------------------------------------------

------------------------------------------------------------

------------------------------------------------------------

------------------------------------------------------------

------------------------------------------------------------

-------------------------------TTVIIGAGISGLQCASAFLRLG------H

TVTILEREEDVGGVWL--------------------------------------------

--------RYTGFNAHVPREFFCFPDFA----PSPDP-----------------------

--------------------------DPDLDPEEYPTGRSLLRHVIAYAHRNGL--YRHI

IFRATVTRLHRLGG----------------------------------------------

---------------------QWQCFFDLA--TRGRGAGG---GAGGGGGGGGGGGGGA-

---------------------------RGGGGGGLGEVQQCGVPQR-QHQQQQQQRISAD

YVVVATGLHSS-PNVP---VLD----GQHLF---RGRLLHVQDL-PPND-LELGQQFG--

-------G---RRVVVVGGTKTAVDTATRLVEA---------------------------

-----------------------GAEDTFVVFRQAHW-----LIPRFP-LRG--------

---------------MHFTQLLHSRLTAAML--------NPYYDAGLWGRVK----GGIS

RPLRRLFWRTVERSLVRQQ-------HLYG-DL------------LPS-MP-LDKDLL--

SGGVVHDGRWRQYLEEGAVHAVRGE----PLRLTP-GGLI---LRSGEFI----------

--------------------A-ADVVIMATGHL---KDYSFIDS-DI-------------

--LS-------KLGV-QRDGLY---LYRHVLPP-LVRNIAFVGA-ANST---NN-PLTSY

LQTKWLVACLQN-RVRLP-EP-HDMIRDLQRQTRWQRRKLPPQAD---RAS---------

---------------MIGIYAHHYHDRLL--KDMG-------------------------

--------LPIRRKK-NLVRDCFEPYTA-----------------ADFA-----------

------------------------------------------------------------

-----DIADVLAGDRRRQQSKDVAAAAAEVTAEVTATAGPAAAAGPPPPPLTTSMSMGLG

SSFKLSA-ASAATPSEVYDVTAAEHSVGFGLMPSMRRTAMAAPSRGSTAAETAAAAAAAV

A-AAAAA---------------GADGE---------------------------------

-----GEGDARWAYPPPQESSGPGSYPGGYVAAVGSLPGPGPGPGPGPAAGVGAAGQAAG

AGAGPATAPSMLYGA---RPGTAGAAYGNRLLGTLYEQEGGGAPGLHGRGALAIRSGPGQ

----GPGRLGLAPAPALSSSLTPPTPPTPPDTPSQLMSPTSYGAGFRSVGRPAIYVPPSS

GRSCLMDAAALALMDGVVGGALLDRP----PKPG--AAAAAEQQQQQQRATSTSGELPTA

AAGTGGAAD------GGGGPAGNRSET---AAVAVTREEVAAGAVYGTGRAHTAPAAAQM

TQVTSP-----AAAAAAAAAAAAAAAAAVMSRSRRQGSGDTGGKPAASEAAAAAAGAGDG

RESSSSEALRRRRQGSLAAEAEAEAEEVMSSRN-----DAAAASTAASTAGGGGGGGGDG

GGGDARRPSRRPASARSVSGAPSFVTPALAAALAAGATAA-----AAGPPPWIESPEISE

ADGGGGDGGGGGGAACAARAEAAGASDTVRFGPSTEGGSGGGGAAAAAAPPLLRCASAGF

VANADDEAASCPSAASSESNNVVATCTRASSSSP---HSRSHSASRAGGGGGAAAAGVAG

KA------------------ASRNLVRLVTTR---HAAAAAASGADGA----------SE

PHSGGSVGAAASGSIAVRAGSSTAARSAGRV-SARR----------TSDFLDESEDFIGT

PPGCF----EGSLDAMMS--------DRGGREGGGGGGARSRIAAASSATASSRRRRRRL

TADRSTLDGDDVDEYDDEDAEYDEYDDDDDEYGYMPGGGKGREGQASDGGGGGDGGGAAG

ASGPDGKVAAVAGDHAGGGTTGAGRIGAATASAAGYKSPFALQPSASFGPGTTAAPALTR

SASSPRSNGSPPHRSGTGTRFAAAADGGGGGGARRTREVHPTRMSGDSVADAGNRPGGGG

GGGGGSQLRSYGTAPAAAPPGGGGGGGGGGGGGGGGGSVNNASSALERYAARMTAAPTFR

MSLTNMADMKPANLKHAARASAAAAAMAAAMAAAGGAGSGGGGGASIHSAPLRPFFLQPS

AGGAGGGGG-------SAAAAAAASGYVSHHHHTTSLHPHFAATSRLGAMRPMRVSEEEE

GDGCGELPPGVVVVGGSTDNGGGGGSGGEADDDDGGGGGGGRLSMSALMVVGGGDDDGGV

NQSCASNASFT--------------AAPRRSNTTAGYGSSMQSEGYGRTPNVASVSPAFG

RGGGGAAAAAAGGGMLSSSPFGTHVSATNALPHTAGGGGGGGGFDGGGSWDMTLRQLSRA

VVAPPQRAAGVLRAALISRRNLASGGDRPSESGLSNPNVGGNAGGSFFNARRAGFADDGS

SGPVSRGS-AAAAAAGGGGGGGGIPDAQLMSVLLAPGGGGGGGGPPTGTGSGSGPVLLGA

GVQPSGPAMAPVREPRDASRFVAAMSTAAAAAAQCPPPPSDAAAPLAKSPRGSGDGGGGA

ATAASQNGAGGAADAADADAGGAFRRPASTDGGGSSSGGGGSGFVDDIGEIGGGGGGGAR

MHWSAAVAAAKELRLQSESARSAFAALAMAARARRSCTMYGAPNPSPAHESPMLAKLIAA

SQSHNGVASDGAAAAVPPHAAVHAAASSGGGGGAAAAAAAATVPRRATSP---NVVVAGG

RLSLLGNNSGGGGGGGDSRTSWDSGGGVAKSLKRLGGLLMAVPSSPPPSAVQLAAATTTG

NKSQTGSVADGGGGGGGSRLPSGSSEGGGVTPARLLRRMMSSSKRRSNEGAYAGGYIAMV

EEDNEAVALP------------------------PPPPPGGGSVAAFA-GGGGGGGSGGG

SCASTAVVSPLEAPPAEL--------------AAALFGRTTSRRSVVLYNPPV----AAP

L------------ELYASAPAAPAAPPTG

>jgi|Plesta4481_1|6463|GLC75079.1

------------------------MP----------------------------------

------------------------------------------------------------

------------------------------------------------------------

------------------------------------------------------------

------------------------------------------------------------

------------------------------------------------------------

------------------------------------------------------------

------------------------------------------------------------

------------------------------------------------------------

------------------------------------------------------------

------------------------------------------------------------

------------------------------------------------------------

------------------------------------------------------------

------------------------------------------------------------

------------------------------------------------------------

------------------------------------------------------------

------------------------------------------------------------

------------------------------------------------------------

------------------------------------------------------------

------------------------------------------------------------

------------------------------------------------------------

------------------------------------------------------------

------------------------------------------------------------

------------------------------------------------------------

-------------------------------TTVIIGAGISGLQCASAFLRLG------H

TVTILEREEDVGGVWL--------------------------------------------

--------RYTGFNAHVPREFFCFPDFA----PPPDP-----------------------

--------------------------DPDLDPEEYPTGRSLLRHVIAYAHRNGL--YRHI

IFRATVTRLHRLGG----------------------------------------------

---------------------QWQCFFDLA--TGGRGAGG---GAGGGGGGGGGGGA---

----------------------------RGGGGGLGEVQQCGVPQRHQQQQQQQQRISAD

YVVVATGLHSS-PNVP---VLD----GQHLF---RGRLLHVQDL-PPND-LELGQQFG--

-------G---RRVVVVGGTKTAVDTATRLVEA---------------------------

-----------------------GAEDTFVVFRQAHW-----LIPRFP-LRG--------

---------------MHFTQLLHSRLTAAML--------NPYYDAGLWGRVK----GGIS

RPLRRLFWRTVERSLVRQQ-------HLYG-DL------------LPS-MP-LDKDLL--

SGGVVHDGRWRQYLEEGAVHAVRGE----PLRLTP-GGLI---LRSGEFI----------

--------------------A-ADVVIMATGHL---KDYSFIDS-DI-------------

--LS-------KLGV-QRDGLY---LYRHVLPP-LVRNIAFVGA-ANST---NN-PLTSY

LQTKWLVACLQN-RVRLP-EP-HDMIRDLQRQTRWQRRKLPPQAD---RAS---------

---------------MIGIYAHHYHDRLL--KDMG-------------------------

--------LPIRRKK-NLVRDCFEPYTA-----------------ADFA-----------

------------------------------------------------------------

-----DIADVLAGDRRRQQSKDVAAA----------------------------------

---------------------AAEHSVGFGLMPSMRRTAMAAPSRGSTAAETAAAAAAAV

---AAAA---------------GADGE---------------------------------

-----GEGDARWAYPPPQESSGPGSYPGGYVAAVGSLPGPGPGPGPGPAAGVGAAGQAAG

AGAGPATAPSMLYGA---RPGTAGAAYSNRLLGTLYEQEGGGAPGLHGRGALATRSGPGQ

----GPGRLGLAPAPALSSSLTPPTPPTPPDTPSQLMSPTSYGAGFRSVGRPAIYVPPSS

GRSCLMDAAALALMDGVVGGALLDRP----PKPGAAAAAAAEQQQQQQRATSTSGELPTA

AAGTGGAAD------GGGGPAGNRSET---AAVAVTREEVAAGAVYGTGRAHTAPAAAQM

TQVTSPAAAAAAAAAAAAAAAAAAAAAAVMSRSRRQGSGDTGGEPAASEAAAAAAGAGDG

RESSSSEALRRRRQGSLAAEAEAEAEEVMSSRN-----DAAAASTAASTA-GGGGGGGDG

GGGDARRPSRRPASARSVSGAPSFVTPALAAALAAGATAA-----AAGPPPWIESPEISE

ADGGGGDGGGGGGAACAARAEAAGASDTVRFGPSTEGGSGGGGAAAAAAPPLLRYASAGF

VANADDEAASCPSAASSESNNVVATCTRASSSSP---HSRSHSASRAGGGGGAAAAGVAG

KA------------------ASRNLVRLVTTR---HAAAAAASGADGA----------SG

PHSGGSVGAAASGSIAVRAGSSTAARSAGRV-SARR----------TSDFLDESEDFIGT

PPGCF----EGSLDAMMS--------DRGGREGGGGGGARSRIAAASSATASSRRRRRRL

TADRSTLDGDDVDEYDDEDAEYDEYDDDDDEYGYMPGGGKGREGQASDGGGGGDGGGAAG

ASGPDGKVAAVAGDHAGGGTTGAGRIGAATASAAGYKSPFALQPSASFGPGTTAAPALTR

SASSPRSNGSPPHRSGTGTRFAAAADGGGGGGARRTREVHPTRMSGDSVADAGNRPGGGG

GGGGGSQLRSYGTAPAAAPP--GGGGGGGGGGGGGGGSVNNASSALERYAARMTAAPTFR

PPTSNTQRAPPPPPPPWRRPWPPPVAPAAAAAAAP---------PSTRRRCVRSFCSRPP

AEPA--------EPAAPAAAAAAASGYVSHHHHTTSLHPHFAATSRLGAMRPMRVSEEEE

GDGCGELPPGVVVVGGSTDNGGGGGSGGEADDDDGGGGGGGRLSMSALMVVGGGDDDGGV

NQSCASNASFTAGGGGGGGGGGAAAAAPRRSNTTAGYGSSMQSEGYGRTPNVASVSPAFG

RGGGGAAAAAAGGGMLSSSPFGTHVSATNALPHTAGGGGGGGGFDGGGSWDMTLRQLSRA

VVAPPQRAAGVLRAALISRRNLASGGDRPSESGLSNPNVGGNAGGSFFNARRAGFADDGS

SGPVSRGSAAAAAAAAGGGGGGGIPDAQLMSVLLAPGGGGGGGGPPTGTGSGSGPVLLGA

GVQPSGPAMAPVREPRDASRFVAAMSTAAAAAAQCPPPPSDAAAPLAKSPRGSGDGGGGA

ATAASQNGAGGAADAADADAGGAFRRPATTDGGGSSSGGGGSGFVDDIGEIGGGGGGGAR

MHWSAAVAAAKELRLQSESARSAFAALAMAARARRSCTMYGAPNPSPAHESPMLAKLIAA

SQSHNGVASDGAAAAVPPHAAVHAAASSGGGGGAAAAAAAATVPRRATSP---NVVVAGG

RLSLLGNNSGGGGGGGDSRTSWDSGGGVAKSLKRLGGLLMAVPSSPPPSAVQLAAATTTG

NKSQTGSVADGGGGGGGSRLPSGSSEGGGVTPARLLRRMMSSSKRRSNEGAYAGGYIAMV

EEDNEAVALP------------------------PPPPPVAVTVAAFAGGGGGGGGSGGG

SCASTAVVSPLEAPPAEL--------------AAALFGRTTSRRSVVLYNPPV----AAP

L------------ELYASAPAAPAAPPTG

>jgi|Volre3785_1|12283|GIL87019.1

------------------------MP----------------------------------

------------------------------------------------------------

------------------------------------------------------------

------------------------------------------------------------

------------------------------------------------------------

------------------------------------------------------------

------------------------------------------------------------

------------------------------------------------------------

------------------------------------------------------------

------------------------------------------------------------

------------------------------------------------------------

------------------------------------------------------------

------------------------------------------------------------

------------------------------------------------------------

------------------------------------------------------------

------------------------------------------------------------

------------------------------------------------------------

------------------------------------------------------------

------------------------------------------------------------

------------------------------------------------------------

------------------------------------------------------------

------------------------------------------------------------

------------------------------------------------------------

------------------------------------------------------------

-------------------------------TTVIIGAGISGLQCASAFLRLG------H

TVTVLEREEDVGGVWL--------------------------------------------

--------RYTAFEVQVPREFFSFPDFA----CPPEL-----------------------

------------------------------EPEEYPNGRSLLRYIIAYAHKNQL--YRHI

VFRATVTRLHRLSG----------------------------------------------

---------------------QWQCFFDVR--TGGPGREA---GPGGSAGANGGGG----

------------------------------GGGGV---------------VVQQHRITAD

FVVVATGLHSS-PHMP---LFD----GHHLF---RGQVLHAQDL-PDSA-AGLSQLVS--

-------G---RRVLLVGASKTAVDTATRVSAA---------------------------

-----------------------GAEDTVVVYRQAHW-----PISRYP-FRG--------

---------------VHFTQLLHSRLAASML--------DPYYDAGLCARAK----GWLT

RPLRRLFWRTLERSLIRQQ-------HLSG-DL------------LPT-LP-LDKDLL--

TGGLVHDGKWLQCLQDGTVRAVRGE----PQRFTA-DGLV---LRSGDFI----------

--------------------A-ADVVVMATGHV---KDYSFIDA-DI-------------

--LS-------KLGV-QRDGLY---LYRHVLPP-LVRNIAFVGT-ANSL---NN-PLTSY

LQIKWLVACLQQ-RVRLP-EP-HEMLRDMQRQTRWQRRKLPPQAD---RAS---------

---------------MIGIYSHYYHDRLL--KDMG-------------------------

--------LRTHRKR-NLLRECFEPYTT-----------------ADYQ-----------

------------------------------------------------------------

-----DLAESAESEGQWQ---------------------PTPASAPMLAP---EMSMALG

SSFKLSAEPSAASPSELYDVTPAEHSVGFGFMPSMRR--GAAPSRGSTATDTAAAAAAAV

---AAAG---------------LTENEI--------------------------------

---GPAEAEARWVTM-----------------SGAAVPDHGP----------------AG

STSGPSTAPPILMPSLSYRSG-AGQVVSTRLL-TLYEQE-----ALNGRVASALRSGP--

----ALARVGVSSAAL-----------TPPETPMNLMSPVSYGAGFRSVGRPALYVPPSS

GRSCLMDAAALAMMDGVIGGALLDNPPNLSPQMAAAAAAAGTATATATVTVTAAGQHPKP

LVGSGSSGDVPASTTAPSTASGMRSSTAAAAAAAVVTERALGEMRFGVTRARTAPAATTM

SPTMMQGSGSGREPNAGGVTASGGGGG--------PGATSTSVVAVSAAAAAAAVEIDRG

ESESTSDEDPTTRSGPLVTEDESSSAEVIAARVLPFDFEASAA--------SGCNGGNAD

GGGVSHMGGPRGLSA-------PFMTPVLAAALAAAAGSANMVNHNSVREVSMSTLEVAN

GWGDGGSDGCGGGRAPHDETEAAATP-----------------AGLLAVPPNENVFLPCR

LGEEEEEEAPGPSSAGGE-----APASNAESNPEFQCRTRSNSRTNSTADGHGAQGGNVG

RSTP-------------PPLAPRSLVRLAMAH---HQQATTGAAATDAPSTLTADSTISR

PSSNGSVHAIAGG--AVRIGSS----SVGPLRASRR----------ISDFFDESDEFVGT

PPSCF----DGSMEALAVDRDRDRDRDRDGR------GSR-------SCTVMSLRQRR--

-SQRYPYNGDDTDDYDNED------DDFDEEYVYI---------------GGMDNVNA--

------------------------------------------------------------

------------------------------------------------------------

------------KLPVSAPQ----------------------------------------

------------------------------------------------------------

------------------------------------------------------------

----------------------------------------GR------------------

------------------------------------------------------------

------------------------------------------------------------

------------------------------------------------------------

------------------------------------------------------------

------------------------------------------------------------

------------------------------------------------------------

------------------------------------------------------------

------------------------------------------------------------

------------------------------------------------------------

------------------------------------------------------------

------------------------------------------------------------

------------------------------------------------------------

-----------------------------

>jgi|Gonpec1|3698|rna5993

------------------------ME----------------------------------

------------------------------------------------------------

------------------------------------------------------------

------------------------------------------------------------

------------------------------------------------------------

------------------------------------------------------------

------------------------------------------------------------

------------------------------------------------------------

------------------------------------------------R-----------

------------------------------------------------------------

------------------------------------------------------------

------------------------------------------------------------

------------------------------------------------------------

------------------------------------------------------------

------------------------------------------------------------

-----------------------------------------------------------A

AGTDAALIP---------------------------------------------------

------------------------------------------------------------

------------------------------------------------------------

------------------------------------------------------------

------------------------------------------------------------

------------------------------------------------------------

------------------------------------------------------------

------------------------------------------------------------

--------------DPNVQHGKPR-------TCLVVGAGVAGLQAARHLQRIG------Y

QVLILEASKDIGGVWAS-------------------------------------------

--------NYVGYGIQVPWRTYQFPDFL----WPEEL-----------------------

-----------------------------RPLSDYPTGQQVQQYIRAYAEHFGL--MRHI

RLNSKVLRLRWHPGSC--------------------------------------------

---------------------KWEAVYC----DSARE-----------------------

----------------------------------------------------KFYKVRVD

YAVICTGMYSS-PFIP---RYT----GADAF---AGKQLHAKDF-VDMS------IVR--

-------G---RRVLIVGAGKTALDCVCSLVAS---------------------------

----------------------RTAASVTMLYRQAHW-----PMPRSI--LG--------

---------------IGIRSLLFNRATPAML--------PPYYTAGRGSRAL----DVVA

QPLKRLFWKTMQGFVGCKF-------RITK-EM------------RPR-VG-LPADLF--

YGGQVLEDSYTRFLSSDGFTSLRGE----INSFVR-NGVI---LQDGNFL----------

--------------------P-ADVVLFCTGYE---KTYEYFDG-DI-------------

--RS-------RLGL-QKDGLY---LYRNCIPP-GVPHLAFVGS-EVSTY--SN-ILTHG

LQALWLSHVLSG-RVTLP-CP-AEMAADVRAQQHWRRQVMPAQRG---RGA---------

---------------VLMLYGMQYHDQLL--RDMG-------------------------

--------AQPRRKGVNLLAECFGSYSA-----------------VDYSPLMP--Q----

------------------------------------------------------------

-----D------------------------------------------------------

---------------------------------------------GPDAELEAAWTASQP

------------------------------------------------------------

-------SKSQWSHLA--------------------------------------------

-----------------------------RML----------------------------

------------------------------------------------------------

------------------------------------------------------------

-------AD---------------------------------------------------

------------------------------------------------------------

------------------------------------------------------------

------------------------------------------------------------

------------------------------------------------------------

------------------------------------------------------------

------------------------------------------------------------

------------------------------------------------------------

------------------------------------------------------------

------------------------------------------------------------

------------------------------------------------------------

------------------------------------------------------------

------------------------------------------------------------

------------------------------------------------------------

------------------------------------------------------------

------------------------------------------------------------

------------------------------------------------------------

------------------------------------------------------------

------------------------------------------------------------

------------------------------------------------------------

------------------------------------------------------------

------------------------------------------------------------

------------------------------------------------------------

------------------------------------------------------------

------------------------------------------------------------

------------------------------------------------------------

------------------------------------------------------------

------------------------------------------------------------

-----------------------------

>Mapoly0122s0026|Mpolymorpha_v3.1/1-622

---MGSE-ELSAGQKITSW-----LSEF--------------------DTVLQDED----

------------VDQILEL-----------------------------------------

----FD------------------------------------------------------

-------------DQEC-------------------------------------------

------------------------------------------------------------

------------------------------------------------------------

------------------------------------------------------------

------------------------------------------------------------

----------------------------------------------FWR-----------

------------------------------------------------------------

----------------------------------------------DLL-----------

----------------PFTWNIET------------------------------------

------------------------------------------------------------

----------------------FESKDE--------------ILKML-------------

------------------------------------------------------------

-----------------TE-----------------------------------------

--NLARVKP----------------------------------------RAF--------

------------------------------------------------------------

------------------------------------------------------------

------------------------------------------------------------

------------------------------------------------------------

--------------------VIDGDV----------------------------------

-------------------------IDRPN------------GLMGSAFKFETSLAWCRG

FVWL----KGGKCWTITTSMWD-------LKG-YEE---KISRSRPF-----GPTLGQIK

GRKTAYQEKQQEQHDMG--RSKQP-------YCLVVGAGQGGVMLGARLRMLG------V

PAIIVEKNERLGDNWRN-------------------------------------------

--------RYKSLALHTPCFSDHYPYLP----FPSNW-----------------------

--------------------------------PVLAPKEKLADFIECYGKLMDL----NV

WCSTECKSASYDEKEG--------------------------------------------

---------------------HWTVVLE----RKCGSEV---------------------

----------------------------------------------------SEVVVHPS

HVVLACGLAGY-PKMP---SFP----GKENF---QGVQIHSSKF-VQGE------DYT--

-------G---KKCIIIGAGTSAHDICLDLWEQ---------------------------

-----------------------GASEITLVQKSHTH-----IVTMKD-FLE--------

---------------SRLDPQLEKAELDTNF-------------DTDAFDLK----SASL

-PYKVLPRLNVPIYARIAQKEAELYDKLHKAGFKTF-------MGEDE-SGLILFLLR-R

AAGYYIDMGASELIADGEVRVKNGV---TVQAMKE-NSVV---FDDGSEV----------

--------------------S-ADVIIYATGYG---NMQEFVEK-LISKEAAEKVGLCWG

LGSGF-----------KGDPGPWVGELKNMYRPLPVPALWILS---------GG-LSHCR

FYSRHVALQLKARMEGIP-TP-VYKL----------------------------------

------------------------------------------------------------

---------------------------------------------VRHK-----------

------------------------------------------------------------

------------------------------------------------------------

------------------------------------------------------------

------------------------------------------------------------

------------------------------------------------------------

------------------------------------------------------------

------------------------------------------------------------

------------------------------------------------------------

------------------------------------------------------------

------------------------------------------------------------

------------------------------------------------------------

------------------------------------------------------------

------------------------------------------------------------

------------------------------------------------------------

------------------------------------------------------------

------------------------------------------------------------

------------------------------------------------------------

------------------------------------------------------------

------------------------------------------------------------

------------------------------------------------------------

----------EPDSPVLASK----------------------------------------

------------------------------------------------------------

------------------------------------------------------------

------------------------------------------------------------

------------------------------------------------------------

------------------------------------------------------------

------------------------------------------------------------

------------------------------------------------------------

------------------------------------------------------------

------------------------------------------------------------

------------------------------------------------------------

------------------------------------------------------------

------------------------------------------------------------

-----------------------------------MSSIQMQD-----------------

------------------------------------------------------------

------------------------------------------------------------

-----------------------------

>178716|Smoellendorffii_v1.0/1-611

MATMNGSPKIDPDALVDAW-----LARL--------------------DSSLASGD----

------------ASAAATL-----------------------------------------

----F-------------------------------------------------------

-------------EEST-------------------------------------------

------------------------------------------------------------

------------------------------------------------------------

------------------------------------------------------------

------------------------------------------------------------

---------------------------------------------SFWR-----------

------------------------------------------------------------

----------------------------------------------DLI-----------

----------------AFTWNIVT------------------------------------

------------------------------------------------------------

----------------------VEGRSQ--------------IHAML-------------

------------------------------------------------------------

-----------------QS-----------------------------------------

--TLESVAP----------------------------------------KGW--------

------------------------------------------------------------

------------------------------------------------------------

------------------------------------------------------------

------------------------------------------------------------

--------------------VRNGTA----------------------------------

-------------------------SYSAD-----------SGVIEAWIKFETRDAVCTG

HLRLM--ATTSLCRTLLTAMEG-------LKD-FPE---KKGHTRPK-----GVTHGVIR

HRASWLDGRKEEERTLG--STVQP-------YCVIVGGGQAGIGLAARLRQLG------V

PCIVVEKNPRPGDSWRS-------------------------------------------

--------RYKSLCLHDPVWYDHLPYLP----FPENW-----------------------

--------------------------------PIFTPKDKMGDWLEAYTKIMEI----NY

WTSSECLGARFDPQSG--------------------------------------------

---------------------EWEVKIL----RDRS------------------------

----------------------------------------------------KEVTLRPK

QLILATGMSGF-PNVP---RIP----GQEEF---VGDLHHSSKH-PGGK------AYK--

-------G---KRAVILGSNNSAHDIAADLWEN---------------------------

-----------------------GAAEVTMIQRSSTH-----VVRSES-LLQ--------

---------------FVMKNTYSESAVESGL-------------TTDKADMI----FASV

-PYKIMGDVNRPTYDAIRAHDKDFYAALTKTGFMLD-------FGDDD-SGLFMKYLR-R

GSGYYIDVGASQLLIDGEIKLRSGV---TIASLKP-SSVV---LSDGTEI----------

--------------------A-ADVVILATGYG---SMNQWAAK-LISQEVADRVGKCWG

LGSNT-----------RKDPGPWEGELRNMWKPTAQEGLWFHG---------GN-LHQAR

HYSLYLALQLKARMEGIT-TP-VYGIPKV-------------------------------

------------------------------------------------------------

----------------------------------------------RHLS----------

------------------------------------------------------------

------------------------------------------------------------

------------------------------------------------------------

------------------------------------------------------------

------------------------------------------------------------

------------------------------------------------------------

------------------------------------------------------------

------------------------------------------------------------

------------------------------------------------------------

------------------------------------------------------------

------------------------------------------------------------

------------------------------------------------------------

------------------------------------------------------------

------------------------------------------------------------

------------------------------------------------------------

------------------------------------------------------------

------------------------------------------------------------

------------------------------------------------------------

------------------------------------------------------------

------------------------------------------------------------

------------------------------------------------------------

------------------------------------------------------------

------------------------------------------------------------

------------------------------------------------------------

------------------------------------------------------------

------------------------------------------------------------

------------------------------------------------------------

------------------------------------------------------------

------------------------------------------------------------

------------------------------------------------------------

------------------------------------------------------------

------------------------------------------------------------

------------------------------------------------------------

------------------------------------------------------------

------------------------------------------------------------

------------------------------------------------------------

-----------------------------

>Poptr1_1|eugene3.32340002

------------------------------------------------------------

------------------------------------------------------------

------------------------------------------------------------

------------------------------------------------------------

------------------------------------------------------------

------------------------------------------------------------

------------------------------------------------------------

------------------------------------------------------------

------------------------------------------------------------

------------------------------------------------------------

------------------------------------------------------------

------------------------------------------------------------

------------------------------------------------------------

------------------------------------------------------------

------------------------------------------------------------

------------------------------------------------------------

------------------------------------------------------------

------------------------------------------------------------

------------------------------------------------------------

------------------------------------------------------------

------------------------------------------------------------

------------------------------------------------------------

------------------------------------------------------------

------------------------------------------------------------

------------------------------------------------------------

------------------------------------------------------------

--------------------------MP----FPDNW-----------------------

--------------------------------PVFAPKDKIGDWLEMYTKVMEV----NY

WPSTDCLNARFDEDRQ--------------------------------------------

---------------------EWEVTVE----RNG-------------------------

----------------------------------------------------ETQVLRPK

HVVFATGMSGK-ANVP---KIK----GQDVF---KGEQQHSSQH-HGPD------AYA--

-------G---KKVVVIGANNSAHDICAALWEH---------------------------

-----------------------GA-DVTMVQRSSTH-----IVRSDS-LMD--------

---------------IGLGDLYSERAVASGV-------------TTRKADLI----FASL

-PYRIMADFQVPVYDRIRERDADFYRQLEATGFMLD-------FGDDD-SGLFMKYLR-R

ASGYYIDVGACDLVIDGSIKLQAGK---QISHLSE-NAVV---LADGTEL----------

--------------------P-ADLVVYATGYG---SMNGWVAD-LISQEVADKVGKVWG

LGSDT-----------TKDPGPWEGEQRNMWKPTQQEALWFHG---------GN-LHQSR

HYSLYLALQLKARQEGLP-VR-VYDLQQV-------------------------------

------------------------------------------------------------

----------------------------------------------HHLXX---------

------------------------------------------------------------

------------------------------------------------------------

------------------------------------------------------------

------------------------------------------------------------

------------------------------------------------------------

------------------------------------------------------------

------------------------------------------------------------

------------------------------------------------------------

------------------------------------------------------------

------------------------------------------------------------

------------------------------------------------------------

------------------------------------------------------------

------------------------------------------------------------

------------------------------------------------------------

------------------------------------------------------------

------------------------------------------------------------

------------------------------------------------------------

------------------------------------------------------------

------------------------------------------------------------

------------------------------------------------------------

------------------------------------------------------------

------------------------------------------------------------

------------------------------------------------------------

------------------------------------------------------------

------------------------------------------------------------

------------------------------------------------------------

------------------------------------------------------------

------------------------------------------------------------

------------------------------------------------------------

------------------------------------------------------------

------------------------------------------------------------

------------------------------------------------------------

------------------------------------------------------------

------------------------------------------------------------

------------------------------------------------------------

------------------------------------------------------------

-----------------------------

>jgi|Mesvir1_1|21105|Mv12382-RA.1

--------------MAKEW-----IAKF--------------------GRALESGD----

------------TSAASSM-----------------------------------------

----F-------------------------------------------------------

-------------ADDC-------------------------------------------

------------------------------------------------------------

------------------------------------------------------------

------------------------------------------------------------

------------------------------------------------------------

----------------------------------------------YWR-----------

------------------------------------------------------------

----------------------------------------------DLV-----------

----------------CWTWNILT------------------------------------

------------------------------------------------------------

----------------------CEGKGE--------------IKAML-------------

------------------------------------------------------------

-----------------DA-----------------------------------------

--RLKATGP----------------------------------------SNW--------

------------------------------------------------------------

------------------------------------------------------------

------------------------------------------------------------

------------------------------------------------------------

--------------------EISSES----------------------------------

-------------------------TGAD-------------GVTEAWFTFETILARCNG

VLRL----KEGRCWIMLTTMQE-------LKG-YEE---KRGPTRPA-----GVVHGAIK

GRRSWLELKAEEESKLGHSDSSQP-------YVLIVGGGQCGIALAARLKMLN------V

PAIIIERNARPGDSWRR-------------------------------------------

--------RYRTLCLHDPIWFDHLPYLP----FPDHW-----------------------

--------------------------------PVFIPKDKMGDWLEAYTSIMEL----NY

WGSTACTHAAFDDSNK--------------------------------------------

---------------------EWVITVE----RHVGPA--------SSSNSGTG------

----------------------------HPGTGST--------P-PGDASPPAKLVLRPK

HLVIATGMSGF-PNIP---SFP----GKETF---LGEQCHSSQF-TGEG------EYA--

-------G---KKCVVLGANTSAHDICAALWEQ---------------------------

-----------------------GAASVTMVQRSPSL-----VAKSET-LFD--------

---------------VTMSPLYSEKALAAGI-------------SVDKADMI----LGSL

-PYRVMPFVHMPLYEEIQKRDAEFYKQLAGAGFLLD-------WGEDA-SGLFMKYIR-R

ASGYYIDVGASEMIIDGRIGLKSGV---NIAAVRE-KSVV---LTDGTEL----------

--------------------P-ADILIYATGYS---SMNDWVAQ-LISPQVADKVGKCWG

VGSGT-----------AKDPGPWEGELRNMWKPTAQEGLWFQG---------GN-LYQSR

QYSLYLALQLKARHEGLPDVE-VYGMKPV-------------------------------

------------------------------------------------------------

----------------------------------------------HHLR----------

------------------------------------------------------------

------------------------------------------------------------

------------------------------------------------------------

------------------------------------------------------------

------------------------------------------------------------

------------------------------------------------------------

------------------------------------------------------------

------------------------------------------------------------

------------------------------------------------------------

------------------------------------------------------------

------------------------------------------------------------

------------------------------------------------------------

------------------------------------------------------------

------------------------------------------------------------

------------------------------------------------------------

------------------------------------------------------------

------------------------------------------------------------

------------------------------------------------------------

------------------------------------------------------------

------------------------------------------------------------

------------------------------------------------------------

------------------------------------------------------------

------------------------------------------------------------

------------------------------------------------------------

------------------------------------------------------------

------------------------------------------------------------

------------------------------------------------------------

------------------------------------------------------------

------------------------------------------------------------

------------------------------------------------------------

------------------------------------------------------------

------------------------------------------------------------

------------------------------------------------------------

------------------------------------------------------------

------------------------------------------------------------

------------------------------------------------------------

-----------------------------

>jgi|Mesovir1_1|1813|Mesvi1423S02809

--------------MAKEW-----IAEF--------------------GRMLDSGD----

------------TSAASSM-----------------------------------------

----F-------------------------------------------------------

-------------ADDC-------------------------------------------

------------------------------------------------------------

------------------------------------------------------------

------------------------------------------------------------

------------------------------------------------------------

----------------------------------------------YWR-----------

------------------------------------------------------------

----------------------------------------------DLV-----------

----------------CWTWNILT------------------------------------

------------------------------------------------------------

----------------------CEGKGE--------------IQAML-------------

------------------------------------------------------------

-----------------DA-----------------------------------------

--RLKATAP----------------------------------------SKW--------

------------------------------------------------------------

------------------------------------------------------------

------------------------------------------------------------

------------------------------------------------------------

--------------------EITSES----------------------------------

-------------------------TGAD-------------AVTEAWFTFETGLARCNG

VLRL----KEGKCWTMLTTMQE-------LKG-YEE---KRGPTRPA-----GVVHGAIK

GRRSWLELKAEEESKLGHSDSSQP-------YVVIVGGGQCGIALAARLKMLD------V

PAIIIERNARPGDSWRK-------------------------------------------

--------RYRTLCLHDPIWFDHMPYIP----FPDHW-----------------------

--------------------------------PVFIPKDKMGDWLEAYTSIMEL----NF

WGGTRCIGAAFDEGKE--------------------------------------------

---------------------EWAVTVE----LDAKKAP-------STAHSITG------

----------------------------HVAAGGS--------HFAAPDSTLTTLVLRPK

HLVIATGMSGF-PEMP---LFP----GQESF---HGKQWHSSQF-NGEG------DFA--

-------G---KNCVVLGANTSAHDICAALWEQ---------------------------

-----------------------GADSVTMVQRSPSL-----VAKTET-LFD--------

---------------VTMSGLYSEKALAAGI-------------SVDKADMI----LASL

-PYRVMPFVHMPMYEEIQRRDAEFYKQLAGAGFLLD-------WGEDA-SGLFMKYIR-R

ASGYYIDVGASEMIIDGRIGLKSGA---NVATVKE-KSVV---LTDGTEL----------

--------------------P-ADILVYATGYS---SMNDWVAQ-LISKQVADKVGKCWG

VGSGT-----------AKDPGPWEGELRNMWKPTAQEGLWFQG---------GN-LYQSR

QYSLYLALQLKARFEGLPGVD-VYGMKPV-------------------------------

------------------------------------------------------------

----------------------------------------------HHLR----------

------------------------------------------------------------

------------------------------------------------------------

------------------------------------------------------------

------------------------------------------------------------

------------------------------------------------------------

------------------------------------------------------------

------------------------------------------------------------

------------------------------------------------------------

------------------------------------------------------------

------------------------------------------------------------

------------------------------------------------------------

------------------------------------------------------------

------------------------------------------------------------

------------------------------------------------------------

------------------------------------------------------------

------------------------------------------------------------

------------------------------------------------------------

------------------------------------------------------------

------------------------------------------------------------

------------------------------------------------------------

------------------------------------------------------------

------------------------------------------------------------

------------------------------------------------------------

------------------------------------------------------------

------------------------------------------------------------

------------------------------------------------------------

------------------------------------------------------------

------------------------------------------------------------

------------------------------------------------------------

------------------------------------------------------------

------------------------------------------------------------

------------------------------------------------------------

------------------------------------------------------------

------------------------------------------------------------

------------------------------------------------------------

------------------------------------------------------------

-----------------------------

>jgi|Auxpr25_1_1|888|APUTEX2500004442-RA

EGVLSGT---SPSDTVRAW-----LSSL--------------------DSALRARD----

------------AVTAASL-----------------------------------------

----F-------------------------------------------------------

-------------EEEG-------------------------------------------

------------------------------------------------------------

------------------------------------------------------------

------------------------------------------------------------

------------------------------------------------------------

----------------------------------------------LWR-----------

------------------------------------------------------------

----------------------------------------------DLV-----------

----------------CMTWNLKT------------------------------------

------------------------------------------------------------

----------------------CEGRGE--------------IAAML-------------

------------------------------------------------------------

-----------------GA-----------------------------------------

--CLDQASP----------------------------------------RDW--------

------------------------------------------------------------

------------------------------------------------------------

------------------------------------------------------------

------------------------------------------------------------

--------------------TLEGEA----------------------------------

-------------------------TAASSP--------VTGDTVEGWIRFRTAALECKG

YLRL----RAGRAWTLLTAAQG-------LVD-FPE---PSGANRPM-----GAEHGARR

NRTTWLDERQREARELG--RSVQP-------YAVIVGGGQGALALAARLRQAR------V

PTIVLERNARPGDSWRR-------------------------------------------

--------RYKPLCLHDPVWYDHLPYLP----FPDTW-----------------------

--------------------------------PVFTPKDKIGDWLEMYAKVMEI----NY

WASTTCKKAEWDEAAQ--------------------------------------------

---------------------EWAVHVT----RVGEDGA-------A-------------

----------------------------------------------------EDITLRPK

QLIMATGMSGR-PSMP---IIP----GAETF---QGDYHHSSQH-PGAE------PYC--

-------G---KDCVILGSNNSAHDIAADLWEN---------------------------

-----------------------GAKSVTIVQRSSTH-----VIRSQT-FTD--------

---------------LITGPLYSEEAMKAGI-------------TTEKADML----FASI

-PYKVLPAIHRAVNEVVKERDADFYAALRAKGFLLD-------FGDDD-SGLFLKYLR-R

GSGYYIDVGASGLVLSGEVSLRSGV---SIARVNP-RSVT---LTDGSEL----------

--------------------P-ADLVVLATGFG---SMNGWAEE-LISKDVADRVGRCWG

LGSGT-----------TKDPGPWEGELRNMWKPTAVPALWFHG---------GN-LHQSR

HYSKYLALQIKARYEGLP-TP-VYGQAKVPDWAHSKLERRANKAVLFLQQLTTIQQTGEG

LKVPTMPKSAVVSAEGIAIKYKSKYGALL--TRTTERGFVIRKLTGPGGEIRWSAPVFLQ

GSAMGAGLSGGYRKSRTCIALANDQAVQSALRSQSEFGPC-----VHFLLDMNG-T----

----------------------------------------------------R------I

KRAHFDSSDQASQTAVSQDRH---------------------------------------

----------------------------------------GGMTAKYYQVDAAMVDISVE

---GGST-----------------------------------------------------

---------------------------------------------------------YV-

----------------------------DKDF----------------------------

--------------------------------------------------NAAVY-----

---------------------------------GQGVTAEGIL-----------------

------------------------------------------------------------

------------------------------------------------------------

------------------------------------------------------------

------------------------------------------------------------

------------------------------------------------------------

-------------------------------------------------------SGEV-

-----------------PPPAEFQLLYTV-------------------------------

----------------------------------------------LQQFVDE-------

------------------------------------------------------------

------------------------------------------------------------

------------------------------------------------------------

------------------------------------------------------------

-----------TPSPVVKRE----------------------------------------

------------------------------------------------------------

------------------------------------------------------------

------------------------------------------------------------

----------------------------------------L-------------------

------------------------------------------------------------

------------------------------------------------------------

------------GASGHGARS---------------------------------------

------------------------------------------------------------

------------------------------------------------------------

--IRS-------------------------------------------------------

------------------------------------------------------------

------------------------------------------------------------

-----------------------------------VRSINMKSSPE--------------

----EGTALP--------------------------------------------------

--------------------------------GVALQRRTSATPSV--------------

------------------------AGIKE

>jgi|Ostque1|9343|CAD7697881.1

------------------------------------------------------------

------------------------------------------------------------

------------------------------------------------------------

------------------------------------------------------------

------------------------------------------------------------

------------------------------------------------------------

------------------------------------------------------------

------------------------------------------------------------

------------------------------------------------------------

------------------------------------------------------------

------------------------------------------------------------

------------------------------------------------------------

------------------------------------------------------------

------------------------------------------------------------

------------------------------------------------------------

------------------------------------------------------------

------------------------------------------------------------

------------------------------------------------------------

------------------------------------------------------------

------------------------------------------------------------

------------------------------------------------------------

------------------------------------------------------------

------------------------------------------------------------

------------------------------------------------------------

-------------------------------------------MIGARLARLG------V

PAIILERRARPGDSWRH-------------------------------------------

--------RYASLATHDPVWMNHLPYIP----FPSHW-----------------------

--------------------------------PTYFPKDKLADWLEMYVKVMEL----NY

WGGSACTGAAYDEGEG--------------------------------------------

---------------------VWRVTVD----KGG-------------------------

----------------------------------------------------AEVTLRPR

QLVLATGVHGA-PFVP---DFP----GAASF---KGPQVHSGNY-KGDE------EWE--

-------G---LRCVVIGSNNSAHDICHDLWER---------------------------

-----------------------GA-LPTMIQRTSTH-----VTRSAV-WQD--------

---------------MYLKPLYSEEALASGM-------------TTDKADMI----FASI

-PYRVHAVLDVPTYTAMAKRDAPFYKRLTDVGFLLD-------FGEDG-SGLFMKWLR-R

GAGFYIDTGASDLISSGDIGLKSSV---GVDSIGE-ASVK---LTDGTVL----------

--------------------P-ADVIVYATGWA---SLSDSLGK-MTSPEVAGKVGRCWG

LGSNT-----------TGDPGPWEGELRNMYKPTAQRGLWIFG---------GS-FMQCR

FYSQFLALQVKARMEGLH-TP-VYGGPEV-------------------------------

-------------------------------ATAN-------------------------

------------------------------------------------------------

------------------------------------------------------------

------------------------------------------------------------

------------------------------------------------------------

------------------------------------------------------------

------------------------------------------------------------

------------------------------------------------------------

------------------------------------------------------------

------------------------------------------------------------

------------------------------------------------------------

------------------------------------------------------------

------------------------------------------------------------

------------------------------------------------------------

------------------------------------------------------------

------------------------------------------------------------

------------------------------------------------------------

------------------------------------------------------------

------------------------------------------------------------

------------------------------------------------------------

------------------------------------------------------------

------------------------------------------------------------

------------------------------------------------------------

------------------------------------------------------------

------------------------------------------------------------

------------------------------------------------------------

------------------------------------------------------------

------------------------------------------------------------

------------------------------------------------------------

------------------------------------------------------------

------------------------------------------------------------

------------------------------------------------------------

------------------------------------------------------------

------------------------------------------------------------

------------------------------------------------------------

------------------------------------------------------------

------------------------------------------------------------

------------------------------------------------------------

-----------------------------

>jgi|Ostque1|10765|CAD7699277.1

---MSAP---THEEQRRAW-----LDAF--------------------ASALERRD----

------------VDAAASL-----------------------------------------

----F-------------------------------------------------------

-------------ADEC-------------------------------------------

------------------------------------------------------------

------------------------------------------------------------

------------------------------------------------------------

------------------------------------------------------------

----------------------------------------------YWR-----------

------------------------------------------------------------

----------------------------------------------DLV-----------

----------------AFTWNLVT------------------------------------

------------------------------------------------------------

----------------------LEGRDA--------------VAAML-------------

------------------------------------------------------------

-----------------TA-----------------------------------------

--TLPAASP----------------------------------------RGW--------

------------------------------------------------------------

------------------------------------------------------------

------------------------------------------------------------

------------------------------------------------------------

--------------------AAEGDA----------------------------------

-------------------------IESAE------------GVVEGRFNFETGAARCKG

HVRL----RGGRCWTLTTTMDE-------LIG-HEE---RRGRRRET-----GRCTAA--

--------GTKSVGEVG-DPGWQP-------YCLVVGGGQGGITVGARLGVLG------V

PVLVVDRLERPGDAWRR-------------------------------------------

--------RYACLVTHDPVHADHLPYMP----FPDHW-----------------------

--------------------------------PVFSTKDRMGDWLEAYATVMGL----NY

WGSTECRNAVYDEEGQ--------------------------------------------

---------------------RWTVTLN----REG-------------------------

----------------------------------------------------KEVKVYPT

HVVLATGLSGA-PKVP---EVP----GAATF---VGLQQHSSEF-SSGK------GWE--

-------G---KRCVVVGAGNSALDICGDLWAH---------------------------

-----------------------GA-QVTMVQRSATL-----VVQSQT-LTG--------

---------------RMLGKWYSEEAVEAGV-------------THEMADFA----QSTV

-PYRLMPARSKVLFDEIRAANAEFYDGLSKAGFLLT-------FGPDG-SGMPMRYLR-R

AAGSLIDTGNSELIASGKVRLKSGV---EISNAEA-DALV---LNDGTRL----------

--------------------P-ADLVVYATGYG---NMSEVVAK-LLGQGVADRIGRVWG

MGSGL-----------DGDPGPWEGELRNMWKPTRQPGLWIHA---------GM-LALSR

TYSRYLALQIKARFEGIP-TP-VYGLAEV-------------------------------

------------------------------------------------------------

----------------------------------------------HHKR----------

------------------------------------------------------------

------------------------------------------------------------

------------------------------------------------------------

------------------------------------------------------------

------------------------------------------------------------

------------------------------------------------------------

------------------------------------------------------------

------------------------------------------------------------

------------------------------------------------------------

------------------------------------------------------------

------------------------------------------------------------

------------------------------------------------------------

------------------------------------------------------------

------------------------------------------------------------

------------------------------------------------------------

------------------------------------------------------------

------------------------------------------------------------

------------------------------------------------------------

------------------------------------------------------------

------------------------------------------------------------

------------------------------------------------------------

------------------------------------------------------------

------------------------------------------------------------

------------------------------------------------------------

------------------------------------------------------------

------------------------------------------------------------

------------------------------------------------------------

------------------------------------------------------------

------------------------------------------------------------

------------------------------------------------------------

------------------------------------------------------------

------------------------------------------------------------

------------------------------------------------------------

------------------------------------------------------------

------------------------------------------------------------

------------------------------------------------------------

-----------------------------

>jgi|Klenit1_1|804|kfl00109_0340_v1.1

---MATQ---TLTEDVRAW-----LERF--------------------SAALEESD----

------------LEKVLAL-----------------------------------------

----F----------------------------G--------------------------

--------------DQC-------------------------------------------

------------------------------------------------------------

------------------------------------------------------------

------------------------------------------------------------

------------------------------------------------------------

----------------------------------------------FWR-----------

------------------------------------------------------------

----------------------------------------------DMV-----------

----------------SFTWNVKT------------------------------------

------------------------------------------------------------

----------------------AEGKDE--------------IRAML-------------

------------------------------------------------------------

-----------------EA-----------------------------------------

--TLPSAAP----------------------------------------ASW--------

------------------------------------------------------------

------------------------------------------------------------

------------------------------------------------------------

------------------------------------------------------------

--------------------RVEGEA----------------------------------

-------------------------MEMF-------------GAVTAFVAFETATGRGKG

HLRL----VDGKCFTLLTTLYE-------LKG-FEE---KIGASRLP-----GRNLAPEP

NRQSWAAKRELERGELG--YGKQP-------FVVIVGAGHSGVMLAARLRALN------V

PTIVVDK-GSPGDSWRN-------------------------------------------

--------RYGSLHLHTPSYMDNFPYLD----YPAGW-----------------------

--------------------------------PTYPSKDEMGDWIEAYVKIMNV----NV

WSHSSVESSTFDDESK--------------------------------------------

---------------------EWAVAVT----RPDG------------------------

----------------------------------------------------KTLTLRPK

HLVLCTGMYGD-PRVP---DFS----GAEIF---GGSQHHSSRHGCGGA------DFA--

-------G---KKCVVIGTGTSGHDVAQDLWEK---------------------------

-----------------------GA-DVTMVQRSPVF-----VIRSET-LAA--------

---------------VYVDPLFHD---SSPM-------------SQQHADLL----MVST

-PYRLVAPFQAPMVEEIKRRDGDLHERLRERGFRVH-------FAEDG-SGLAGLLNR-T

ATGYYYNMGASYLIASGEIKVRSGG----VEKVTE-RTVV---LTDGSQV----------

--------------------P-ADVIVYATGSR---PVSEVVAR-VTSPEVAAKVGPLWG

YGSGL-----------DGDPGPWEGELRNIYKPTAQEGLWFHI---------GG-LVQTR

FGSLLVALQLKARFEGLP-TP-VYR-----------------------------------

------------------------------------------------------------

------------------------------------------------------------

------------------------------------------------------------

------------------------------------------------------------

------------------------------------------------------------

------------------------------------------------------------

------------------------------------------------------------

------------------------------------------------------------

------------------------------------------------------------

------------------------------------------------------------

------------------------------------------------------------

------------------------------------------------------------

------------------------------------------------------------

------------------------------------------------------------

------------------------------------------------------------

------------------------------------------------------------

------------------------------------------------------------

------------------------------------------------------------

------------------------------------------------------------

------------------------------------------------------------

------------------------------------------------------------

------------------------------------------------------------

------------------------------------------------------------

------------------------------------------------------------

------------------------------------------------------------

------------------------------------------------------------

------------------------------------------------------------

------------------------------------------------------------

------------------------------------------------------------

------------------------------------------------------------

------------------------------------------------------------

------------------------------------------------------------

------------------------------------------------------------

------------------------------------------------------------

------------------------------------------------------------

------------------------------------------------------------

------------------------------------------------------------

------------------------------------------------------------

-----------------------------

>Sphfalx02G048800|Sfallax_v1.1/1-665

------------------------ME----------------------------------

------------------------------------------------------------

------------------------------------------------------------

------------------------------------------------------------

------------------------------------------------------------

------------------------------------------------------------

------------------------------------------------------------

------------------------------------------------------------

------------------------------------------------------------

------------------------------------------------------------

------------------------------------------------------------

------------------------------------------------------------

------------------------------------------------------------

------------------------------------------------------------

------------------------------------------------------------

------------------------------------------------------------

------------------------------------------------------------

------------------------------------------------------------

------------------------------------------------------------

------------------------------------------------------------

------------------------------------------------------------

------------------------------------------------------------

------------------------------------------------------------

------------------------------------------------------------

------------------PMIGRK-------MVAIIGAGPAGLVAAKSMLEVG------L

FPTLFDKLPDLGGVWNPQS-----------------------------------------

------SSCWFSLRTNDSRYSCCFSDFP----WDSDS-----------------------

--------------------------------PIFPSQHDVYGYLQAYANKFLQ--PVHF

CLGSEVTNVSRSNND---------------------------------------------

---------------------KWLVQWS----SLEQKYQ-------GS------------

----------------------------------------------------KAGSQIFD

FVVFASGFFSI-PYTP---SIQ----GLDGF---PGEVMHSSEY-RSPE------RFR--

-------G---KRVVILGASFSSAEVAADLAPL---------------------------

------------------------ATEVINIVSRPFW-----VLPRYI-ALDPDSA----

-----------STPFFPLDLVLYRRSTRQ----------------SQGEEVF--------

---------------------ANEEENRKANQYFRKLCGDQGP------LN-LQQDT--D

PPIIAISDSYLELVRSNHIMVRHGK----VQEVQG-QTVI---LEDQTTI----------

-------------------PE-VDCIIFCTGFQ---SSMPYLSS-NV-------------

--LDT------LSFDPADRFVPFL-LHRSTFHP-SLPGSAFVGM-YRGTF-----FGVME

LQARWAAAIFSG-DLPPI-PE--SVQQEGIAFEKWIKSQTPRPQFPH-------------

------------------GDYVGFADTFA--ADLN---------VLP-------------

--------SKDWYQK-------HDISIP-----------------AHFR-----------

------------------------------------------------------------

------------------------------------------------------------

---------------------------------------------RGSVAEEAVEVLEKA

---CKEF----------------LDGKMVPHAVFRSL-----------------------

--------AGSWTLH---------------------------------------------

-----------------------------RHI----------------------------

-----ENRID--------------------------------------------------

------------------------------------FANSGEVKGTATFNVTEL------

------------------------------------------------------------

------------------------------------------------------------

------------------------------------------------------------

--------------------SHEYLY----------------------------------

------------------------------------------------------------

-----TEVGELSMQ-----------------------------------------RGQH-

------------------FRVHRKYVYVYDTH----------------------------

---------------------------------TNQ----------IDVFFE--------

--------------------------NQGS---------R-------GSPFHSLQFL---

------------------------------------------------------------

------------------------------------------------------------

------------------------------------------------------------

-------------SPKPS------------------------------------------

------------------------------------------------------------

------------------------------------------------------------

------------------------------------------------------------

------------------------------------------------------------

------------------------------------------------------------

------------------------------------------------------------

------SATEKGGIEMSGG-----------------------------------------

------------------------------------------------------------

------------------------------------------------------------

--WRA-------------------------------------------------------

-------------------AGQHLCGE--------------------------DLYNASY

RFSFSGNS----------------------------------------------------

-----------------------------------IAQMEIS------------------

------------------------------------------------------------

----YSVTGPKKDYTST-----------------ATYFR---------------------

-----------------------------

>jgi|Gonpec1|8977|rna9192

------------------------------------------------------------

------------------------------------------------------------

------------------------------------------------------------

------------------------------------------------------------

------------------------------------------------------------

------------------------------------------------------------

------------------------------------------------------------

------------------------------------------------------------

------------------------------------------------------------

------------------------------------------------------------

------------------------------------------------------------

------------------------------------------------------------

------------------------------------------------------------

------------------------------------------------------------

------------------------------------------------------------

------------------------------------------------------------

------------------------------------------------------------

------------------------------------------------------------

------------------------------------------------------------

------------------------------------------------------------

------------------------------------------------------------

------------------------------------------------------------

------------------------------------------------------------

------------------------------------------------------------

------------------------------------------------------------

-------------MWGDP------------------------------------------

------SATYESLQTNLSRYTCSFSDFP----WPPTS-----------------------

--------------------------------PDFPSAVDVSNYLASYRDAFLAGDNCAM

ALGCEVLQVLRSPQGP--------------------------------------------

---------------------GWVVEWR----YAGPSSG-------GGEPA---------

----------------------------------------------------TPQSREFD

FVIVASGYAAA-PFSP---QIP----GLETF---PGLVIHSKDY-RGPS------SLT--

-------GR--RRVAVVGGGHSAVDIAADLAAG-----VSQGTAAP--------PSAQAG

DTAVHAAAAAA-------------STSVVHVLPRPFW-----VIPRYL-PSDPQSP----

-----------HPAFLPLDLQLYRRSRRQ----------------GSGERLF--------

---------------------PTAEHHRQANTYFHSVCGDQGARLSPC-LA-IPPDCS-E

PAIVAVSDTYGVLAASGRMQLRRAS----LQRIHG-ASLE---LSDGGP-----------

----------------PL-DG-VDAIVLCTGFR---PRMDFLPA-DV-------------

--LAT------LSYTPDDRYVPLA-LHRGVLHP-DVPGLAFVGM-FRGPY-----FGTAE

LQARLAAAALSG-ALPAE-AD--AAQREGVAVELRLRAQSPRPVLPH-------------

------------------GDYVGHADSLA--AALG---------VLP-------------

--------PPEWRAE-------HDVVVP-----------------AHFALV------PPP

RPAPPPHAQREQEQQSG-------------------------------------------

----------------------------EV---------PPGADQG--------------

----------------------------------------ADGAWEAEAARRAVANVESA

---LRAY----------------RGGRMVAGAVFRAL-----------------------

--------HGEWALH---------------------------------------------

-----------------------------RRI----------------------------

-----DSRMA--------------------------------------------------

------------------------------------SSPSGTVTGRASFVMTDA------

------------------------------------------------------------

------------------------------------------------------------

--------------------------------------------------PGGGADTAQ-

-----------------------YLY----------------------------------

------------------------------------------------------------

-----QESGQFTID-----------------------------------------GGGS-

------------------MRVRREYVYSYDKA----------------------------

---------------------------------VDR----------IDVHFA--------

--------------------------EGGS---------R-------GGFFHSLRFL---

------------------------------------------------------------

------------------------------------------------------------

------------------------------------------------------------

-------------PPASAGE----------------------------------------

------------------------------------------------------------

------------QPGGLQCEAAAA------------------------------------

-------PPG-------------------ADTDTSAGAGVG-------------------

---------------------------PCRTGGPAGCGTG--------------VCGAGS

TGCSPAIGALE-------------------------------------------------

------------------------------------------------------------

TAVVEQGEGGAGASISGDG-----------------------------------------

------------------------------------------------------------

------------------------------------------------------------

--WRA-------------------------------------------------------

-------------------VGDHLCIR--------------------------DMYRASY

RFAFQGIQ----------------------------------------------------

-----------------------------------LREFEIQ------------------

------------------------------------------------------------

----FRVSGPQKDYTAT-----------------ALYTRRK-------------------

-----------------------------

>jgi|Plesta1363_1|1829|GLC41090.1

------------------------------------------------------------

------------------------------------------------------------

------------------------------------------------------------

------------------------------------------------------------

------------------------------------------------------------

------------------------------------------------------------

------------------------------------------------------------

------------------------------------------------------------

------------------------------------------------------------

------------------------------------------------------------

------------------------------------------------------------

------------------------------------------------------------

------------------------------------------------------------

------------------------------------------------------------

------------------------------------------------------------

------------------------------------------------------------

-------MP---------------------------------------------------

------------------------------------------------------------

------------------------------------------------------------

------------------------------------------------------------

------------------------------------------------------------

--------------------HARRE-----------------------------------

------------------------------------------------------------

----------------------------P-------------------------------

----------------STTTPQPK-------SVAVIGGGLAGLSAAKAAVQAG------L

LPTIFDRRPSIGGMWGDAA-----------------------------------------

------GATYASLHTNVSRFTCSFSDFP----WPPGS-----------------------

--------------------------------PDFPTAAQVGDYLSAYQRAFLPPDKCRL

RLGCE------------------------------HAGDPPRTEQTRT------------

---------------EQQQQLPWVVEWH----EAQPSPG-------ASAP----------

------------------------------------------------THG-SSHTQHFD

FVVVASGFCAA-PRIP---SIP----GLDSF---PGLLLHSAAY-RGPH------SLRLA

AAAAAGAGRRCRRVAVVGAGHSAADIAADIAAAGGGTGAGGGTAA-----------AAAP

EPASDPDASVM-------------PMEVIHVTPRPFW-----VLPRYL-PLSLDAA----

-----------APPFAPLDVMCHRWSARPGC--------EGAENDKGRERLF--------

---------------------ASREERVKDNAFWATVCGDQGALLSPA-LA-ISPGSS-E

PMVGSITDCYGPLVASGRLKVLRGR----LHRVEG-STLE---LSGGPPGG----GGGGG

AAAADSAVAAAAPPPAPL-RD-VDAIVLCTGMR---PSLSFLPA-EV-------------

--LET------LSYTPYDDYVPLA-LHRQVLHP-DVPGLAFVGV-FRSPF-----FAVVE

LQARLAAAAAAG-TLPAA-EADPAAQRAGVDLELRVRQYEPRTPYPR-------------

------------------VDFVGHADGLA--AALG---------VRP-------------

--------PAEWRAA-------HDVIVA-----------------AHYGVF--------P

GPSAAANSTTTAETQGGEAAAAARTTAA----------------------VD--------

-DDDGDASAAAAAAMG-------DAV----------------------------------

------------------------------------------AA-APEGVRRALDDVAAA

---MRSY----------------RGGHRVASAVFHDL-----------------------

--------AGEWALH---------------------------------------------

-----------------------------RRI----------------------------

-----VSRLP--------------------------------------------------

------------------------------------SSPSGEVTGRATFTATAA------

-----------------------------------------------DTSGVYSP-----

------------------------------------------------------------

--------------------------------------------------AAGGAGGLS-

-----------------------YLY----------------------------------

------------------------------------------------------------

-----EEQGQFAML-----------------------------------------GGPV-

------------------MRVRREYVYAYDST----------------------------

---------------------------------ADR----------IDVFFA--------

--------------------------EGGSR--------R-------GAFFHSLRFL---

------------------------------------------------------------

------------------------------------------------------------

------------------------------------------------------------

-------------PPEEQSS----------------------------------------

------------------------------------------------------------

------------SA-----AAAAA------------------------------------

-------PAA-------------------AAAAQPAAA----------------------

------------------------------TSAEAAAATA--------------AADVAA

TDAGGASAATA-------------------------------------------------

------------------------------------------------------------

SSPASPAAGAAAAAAAAAG-----------------------------------------

------------------------------------------------------------

------------------------------------------------------------

--WRA-------------------------------------------------------

-------------------VGEHLCVR--------------------------DMYDASY

RFSFQGLR----------------------------------------------------

-----------------------------------LRQFEIE------------------

------------------------------------------------------------

----FRVKGPNKDYTAT-----------------ATYTRQPGGGRI-----PYEGC----

-----------------------------

>jgi|Plesta4481_1|2164|GLC71143.1

------------------------MLLSS-------------------------------

------------------------------------------------------------

------------------------------------------------------------

---------------------------------------------FQRPLHPQTVS----

------------------------------------------------------------

------------------------------------------------------------

------------------------------------------------------------

------------------------------------------------------------

TKNN--------------------------------------------------------

------------------------------------------------------------

------------------------------------------------------------

-------------------------------------NF-A-PIHFQHQGRQLSFILQR-

----------------------ARSSHS--------------------------------

------------------------------------------------------------

------------------------------------------------------------

------------------------------------------------------------

RATRASAMP---------------------------------------------------

------------------------------------------------------------

------------------------------------------------------------

------------------------------------------------------------

------------------------------------------------------------

--------------------HARRE-----------------------------------

------------------------------------------------------------

----------------------------P-------------------------------

----------------STTTPQPK-------SVAVIGGGLAGLSAAKAAVQAG------L

LPTIFDRRPSIGGMWGDAA-----------------------------------------

------GATYASLHTNVSRFTCSFSDFP----WPPGS-----------------------

--------------------------------PDFPTAAQVGDYLSAYQRAFLPPDKCRL

RLGCEVLRVLRPSPPPAPPPPPPPPLPPRTPE-TQHAGDPPRTEQTRT------------

---------------EQQQQLPWVVEWH----EAQPSPG-------ASAP----------

------------------------------------------------THG-SSHTQHFD

FVVVASGFCAA-PRIP---SIP----GLDSF---PGLLLHSAAY-RGPH------SLRLA

AAAAAGAGRRCRRVAVVGAGHSAADIAADIAAAGGGTGAGGGTAA-----------AAAP

EPASDPDASVM-------------PMEVIHVTPRPFW-----VLPRYL-PLSLDAA----

-----------APPFAPLDVMCHRWSARPGC--------EGAENDKGRERLF--------

---------------------ASREERVKDNAFWATVCGDQGALLSPA-LA-ISPGSS-E

PMVGSITDCYGPLVASGRLKVLRGR----LHRVEG-STLE---LSGGPPGG----GGGGG

AAAADSAVAAAAPPPAPL-RD-VDAIVLCTGMR---PSLSFLPA-EV-------------

--LET------LSYTPYDDYVPLA-LHRQVLHP-DVPGLAFVGV-FRSPF-----FAVVE

LQARLAAAAAAG-TLPAA-EADPAAQRAGVDLELRVRQYEPRTPYPR-------------

------------------VDFVGHADGLA--AALG---------VRP-------------

--------PAEWRAA-------HDVIVA-----------------AHYGVF--------P

GPSAAANSTTTAETQGGEAAAAARTTAA----------------------VD--------

-DDDGDASAAAAAAMG-------DAV----------------------------------

------------------------------------------AA-APEGVRRALDDVAAA

---MRSY----------------RGGHRVASAVFHDL-----------------------

--------AGEWALH---------------------------------------------

-----------------------------RRI----------------------------

-----VSRLP--------------------------------------------------

------------------------------------SSPSGEVTGRATFTATAA------

-----------------------------------------------DTSGVYSP-----

------------------------------------------------------------

--------------------------------------------------AAGGAGGLS-

-----------------------YLY----------------------------------

------------------------------------------------------------

-----EEQGQFAML-----------------------------------------GGPV-

------------------MRVRREYVYAYDST----------------------------

---------------------------------ADR----------IDVFFA--------

--------------------------EGGSR--------R-------GAFFHSLRFL---

------------------------------------------------------------

------------------------------------------------------------

------------------------------------------------------------

-------------PPEEQSS----------------------------------------

------------------------------------------------------------

------------SA-----AAAAA------------------------------------

-------PAA-------------------AAAAQPAAA----------------------

------------------------------TSAEAAAATA--------------AADVAA

TDAGGASAATA-------------------------------------------------

------------------------------------------------------------

SSPASPAAGAAAAAAAAAG-----------------------------------------

------------------------------------------------------------

------------------------------------------------------------

--WRA-------------------------------------------------------

-------------------VGEHLCVR--------------------------DMYDASY

RFSFQGLR----------------------------------------------------

-----------------------------------LRQFEIE------------------

------------------------------------------------------------

----FRVKGPNKDYTAT-----------------ATYTRQPGGGRI-----PYEGC----

-----------------------------

>jgi|Volca2_1|5804|Vocar.0002s0558.1

------------------------MP----------------------------------

------------------------------------------------------------

------------------------------------------------------------

------------------------------------------------------------

------------------------------------------------------------

------------------------------------------------------------

------------------------------------------------------------

------------------------------------------------------------

----------------------------------------------RARVD---------

------------------------------------------------------------

-------------------P----------------------------------------

------------------------------------------------------------

------------------------------------------------------------

------------------------------------------------------------

------------------------------------------------------------

------------------------------------------------------------

-------TP---------------------------------------------------

------------------------------------------------------------

------------------------------------------------------------

------------------------------------------------------------

------------------------------------------------------------

--------------------QAHAE-----------------------------------

------------------------------------------------------------

----------------------------P-------------------------------

------------------DPQHRK-------TVAIIGGGLSGIAAAKAAVQAG------L

FPTIFDRRPALGGMWTDG------------------------------------------

------AATYDSLHTNVSHFTCSFSDFP----WPSSS-----------------------

--------------------------------PDFPSAREMGQYLTAYQHAFLTQDNCLL

QLGCEVLRVEPNAPLLKCL-----------------------GSGGDG------------

---------------AGQAGPSWSVHWR----HVAHPRPE------AAVSP---------

------------------------------------------------SLG-AVATRRFD

FVVVATGFCAV-PYVP---DIP----GLATF---PGLVLHSADY-TGPS------CLP--

-------GS-CRRVAVIGSGHSAADIAADLAAA-----AAAGRSA---------------

-------AAAT----------VDSSLKVVHVTPRPFW-----VIPRFL-PLEPTAAAAAA

ADGGGG-----GSPFVPLDLFFHRRAARA------------AGGLGPEELVF--------

---------------------PRGHDNAGVNSFFASLCGDQAKLLSPV-LA-VSPSSA-E

PAVSSISDSYGPLLVSGRLQLRRAR----LHRVAG-STLE---FTAAAAAAGDEQPDSGG

GGGVAVAAEAAEAAPQPL-HD-VDAIVLCTGLR---PALPFLST-DL-------------

--LST------LSYTPNDNYVPLA-LHRRVLHP-DAPGLAFVGV-FRSPF-----FAVVE

LQARLAAAAFTG-TLSAA-EADPETQHAGVALELRVRQHRPRPPYPH-------------

------------------QDFVGHADGLA--EALG---------ALP-------------

--------PAAWRAE-------HEVVVS-----------------AHYAVHMFQRPTQPP

PPAPPPSSSRTASAFTAATAAAAVAAAA----------------------AA--------

-NGHGDMAAAATASEGLE----LDAIPTSA---------PPASADG--------------

----------------------------------------ETAA-AAAAVRQALTDVAAA

---MRAY----------------RSGRRVASAVFYAL-----------------------

--------AGEWALR---------------------------------------------

-----------------------------RSI----------------------------

-----ISRLD--------------------------------------------------

------------------------------------SSPSGEVTGRASFAAFAD------

--------------------------------------------------ADFTP-----

------------------------------------------------------------

--------------------------------------------------CAGGAGGLS-

-----------------------YLY----------------------------------

------------------------------------------------------------

-----DEQGQFAIH-----------------------------------------GGAV-

------------------MRVRREYVYAYDST----------------------------

---------------------------------ADR----------IDVYFAA-------

-------------------------EGSGSP--------R-------GNFFHSLKFL---

------------------------------------------------------------

------------------------------------------------------------

------------------------------------------------------------

-------------PPCDGAD----------------------------------------

------------------------------------------------------------

------------GGGGHHLAS---------------------------------------

------------------------------------------------------------

------------------------------------------------------------

------------------------------------------------------------

------------------------------------------------------------

------TAGIADTADGAGC-----------------------------------------

------------------------------------------------------------

------------------------------------------------------------

--WRA-------------------------------------------------------

-------------------VGQHLCGR--------------------------DMYDASY

KFYFQGIQ----------------------------------------------------

-----------------------------------LRQFEIV------------------

------------------------------------------------------------

----FRVTGPSKDYTAT-----------------AVYTRPYV------------------

-----------------------------

>jgi|Volre3785_1|1640|GIL77707.1

------------------------------------------------------------

------------------------------------------------------------

------------------------------------------------------------

------------------------------------------------------------

------------------------------------------------------------

------------------------------------------------------------

------------------------------------------------------------

------------------------------------------------------------

------------------------------------------------------------

------------------------------------------------------------

------------------------------------------------------------

------------------------------------------------------------

------------------------------------------------------------

------------------------------------------------------------

------------------------------------------------------------

------------------------------------------------------------

---MAVAMP---------------------------------------------------

------------------------------------------------------------

------------------------------------------------------------

------------------------------------------------------------

------------------------------------------------------------

--------------------FVPAE-----------------------------------

------------------------------------------------------------

----------------------------P-------------------------------

------------------TPQQRK-------TVAIIGCGLSGLAAAKAAIQAG------L

FPTIFDRRRAAGGMWTDG------------------------------------------

------SATYDSLHTNVSRFTCSFSDFP----WPASA-----------------------

--------------------------------PDFPSALEVGQYLSAYQRTFLTEENCRL

QLGYEVLRVAPSSPDPSLS-----------------------LSHPAA------------

---------------DSSQSPPWRVEWR----KVHEPTGL--REDCAASADADDSEVDLL

QNPESRPTQEQQTSATPASSHTLQAPSRDTAGGSM---------VSQASTASLGSSDQFD

YVVVATGFCAV-PHLP---DIP----GLDTF---PGLVLHSAAY-RGAH------LLR--

-------GR--RRVAVIGSGHSAADIAADLAAA-----ALRTAAVAVRSPG-----GSCS

SGDSTAAAAATDVPP--PQTEVNSGMEVIHATPRPFW-----LIPRYL-PLAAAAA--TD

ANGASGA----APPFVPLDVFFHRWSGRV------------ASGRGPEEQVF--------

---------------------PTSEDHIKSNAFFSSLCGDQGKLLSPV-LA-VPRVTS-E

PVGVAISDSYGPLVASGRLKLRRGR----ITRVSG-CSLE---LSDENAAY---VASASS

GSGSGGSDG-GNNLSQPL-HD-VDAIVLCTGLR---PALKFLPD-EL-------------

--LSK------LSYTPYDNYVPLA-LHRRVLHP-DAPGLAFVGV-FRSPF-----FAVVE

LQARLAAAAFTG-ALPAE-EADPATQYAGVAMELRVRNHTPRTPYPH-------------

------------------QDFVGHADGLA--AAMG---------LLP-------------

--------PPGWRTK-------HDVVMA-----------------AHYAGAVLG-PKPQP

AVAALPRGH-GPAANVPGGDAPTAAAAT----------------------VDG-------

-QQGGE-AKAAAITEE-------EEPRTEV---------PPA------------------

------------------------------------------VA-AVDAVQRALDDVEAA

---MGEY----------------RHGRRVASAVFYAL-----------------------

--------AGEWALR---------------------------------------------

-----------------------------RRI----------------------------

-----VSRFP--------------------------------------------------

------------------------------------SSPSGEVTGRARFTTIAD------

--------------------------------------------------ADFAP-----

------------------------------------------------------------

--------------------------------------------------AAGGAGGLS-

-----------------------YLY----------------------------------

------------------------------------------------------------

-----DEQGQFAVQ-----------------------------------------GGPV-

------------------MRVRREYVYTYDNT----------------------------

---------------------------------ADR----------IDVYFA--------

--------------------------DASPR--------R-------GGFFHSLRFL---

------------------------------------------------------------

------------------------------------------------------------

------------------------------------------------------------

-------------SPEGPQN----------------------------------------

------------------------------------------------------------

------------DEDGS-------------------------------------------

------------------------------------------------------------

------------------------------------------------------------

------------------------------------------------------------

------------------------------------------------------------

------SSSSSSGSGGDGC-----------------------------------------

------------------------------------------------------------

------------------------------------------------------------

--WRA-------------------------------------------------------

-------------------VGDHLCVR--------------------------DMYDASY

RFSFQGIQ----------------------------------------------------

-----------------------------------LREFEIE------------------

------------------------------------------------------------

----FRVKGPNKDYTAT-----------------ATYTRP--------------------

-----------------------------

>jgi|Volafr1|21367|GIL49855.1

------------------------MPCSN-------------------------------

------------------------------------------------------------

------------------------------------------------------------

------------------------------------------------------------

------------------------------------------------------------

------------------------------------------------------------

------------------------------------------------------------

------------------------------------------------------------

--------------------------------------------GVRQRPD---------

------------------------------------------------------------

-------------------PGRT-----------------------ILLG----------

---------------------------------NVSRNY----KHY------CSYIFLR-

----------------------SRSVHSPPLMK---------------------------

---W--------------------------------------------------------

------------------------------------------------------------

------------------------------------------------------------

AAPMAVAMP---------------------------------------------------

------------------------------------------------------------

------------------------------------------------------------

------------------------------------------------------------

------------------------------------------------------------

--------------------FVPAE-----------------------------------

------------------------------------------------------------

----------------------------P-------------------------------

------------------SPQQRK-------TVAIIGGGLSGLVAAKTAIQAG------L

FPTIFDRRRTVGGMWADG------------------------------------------

------STIYDSLHTNVSRFTCSFSDFP----WPSSS-----------------------

--------------------------------PDFPSALEVGRYLSAYQRAFLTEANCRL

QLGCEVLRVAPSYPDQQLP-----------------------VSQPAV------------

---------------AISQSPPWNVEWR----KAHEPAGI------AALADKDDPVSGLL

EDFELGPKNQRQAPNTPDSLHRLEAPYECTAGRAM---------GSQASAASRSCSDQFD

FVVVATGFCAV-PHLP---DIP----GLDTF---RGLVLHSAAY-RGAH------LLS--

-------GR--RRVAVIGGGHSAADIAADLAAA-----ALRAAAVEARSPGWGGPVAAAA

CTAAPAAAAMTDVASGGALAEIDPGMEVFHVTPGPFW-----VIPRYL-PIAAGNA----

----SCA----APPFVPLDVFFYRWSVRM------------ASGLGPEEQTF--------

---------------------PTGEDHAKSNAFFATLCGDQGKLLSPV-LT-VPRVTS-D

PIATAISDSYGPLVASGRLKLRRGR----LTRVSD-SSLE---LSDENAAS---VASAGN

GSGSGSGNGSGNSPSQSL-HD-IDAIVLCTGLR---PSLTFLSD-GL-------------

--LDK------LSYTPYDNYVPLA-LHHRVLHP-DAPGLAFVGL-NRSPF-----FAVVE

LQGRLAAAAFTG-ALPAA-EADLAAQRAGVAMELRVRQHTPRTPYPH-------------

------------------QDYVGHADGLA--TAMG---------LLP-------------

--------APGWRTE-------HDVVMA-----------------AHYAGAILA-PEPPP

PPAAALRGGLGPDADIAARAALTAAVAT----------------------VDG-------

-QQHGDVAKAAAIA--------------EV---------PTAA-----------------

---------------------------------------VVTVA-VADAIRQALDDVKAA

---MGEY----------------RLGRRVASAVFYAL-----------------------

--------AGEWALR---------------------------------------------

-----------------------------RRI----------------------------

-----VSRLP--------------------------------------------------

------------------------------------SSPSGEVTGRARFTTIAD------

--------------------------------------------------AHFAP-----

------------------------------------------------------------

--------------------------------------------------AVGGAGGLS-

-----------------------YLY----------------------------------

------------------------------------------------------------

-----DEQGQFTIQ-----------------------------------------GGAV-

------------------MRVRREYVYTYDST----------------------------

---------------------------------ADR----------IDVYFV--------

--------------------------DAGMR--------R-------GGFFHSLRFL---

------------------------------------------------------------

------------------------------------------------------------

------------------------------------------------------------

-------------PPEPPQN----------------------------------------

------------------------------------------------------------

------------DDD---------------------------------------------

------------------------------------------------------------

------------------------------------------------------------

------------------------------------------------------------

------------------------------------------------------------

---------DGGGGGGDGC-----------------------------------------

------------------------------------------------------------

------------------------------------------------------------

--WRA-------------------------------------------------------

-------------------VGDHLCVR--------------------------DTYDASY

RFSFEGIQ----------------------------------------------------

-----------------------------------LREFEIE------------------

------------------------------------------------------------

----FQVKGPEKDYTAT-----------------ATYTRSL-R-----------------

-----------------------------

>jgi|Chlre5_6|5911|Cre13.g603176.t1.1

------------------------MTLLSARTFGP---------AKQTGAELARHPAPQL

VHGPCARHRPWALGARLSLA-----RPAVAS----------------------PHAAADA

YIDEYK-------------------------LAGL-------------------------

-----------DLADEAQQQLRSWYL-----QELLRQEEQRQQQLLAAMTPPATAA----

--ATAAAAAAA-----EGVT----------------------------------------

-----GPRMKELPAAPVSSRTTNAKVTLLYDLEAIKPFTQQAP-----------------

-------------SAAPSLSD------------TASHADTP-------------------

--ASASA------------------------------------------------AAAAS

SRGGG-------------------AGSA--------------RGGGQSRLA---------

------------------------------------------------------------

--------------AAGLVPSSS----------------------PDLVA----------

----------------ALDQLVRTL-----------AEYGA--VHAVH-------LFLR-

---------------------ESTLAKAPALRK---QVQQLCT-----------------

---WV----EPEELLAGAADPPAAARGAASRRPPSTSPSSSSSSSSLR------------

------------------------------------------------------------

--------------TSTTSTS-----------SSGSTSSSS----------------GGA

AAAAAPATPHDCPLCRTCCATRLDLLSHFATTHQAPVLAATAAADPRARIMWT-------

-------------RGR-PLSLTLPQPMEAAEAGAGQAGAGQAGA----AAGQQLQV----

---SPQDFV--FAREALQRGRY-LDPLL--AAAP-PTTLSIAADQLQPPTQAQHGPLQPQ

HGPLQPSP----------QHRLQQHRP----------------------QQRPQQEQQQQ

------------------------------------AVSPDEALGGPGLFLH----VLPA

GQPGSALPGALMRVAQRLAQQAEAEAETLLPAAAAAGQAPRRGRGALLRGRGAGRRLAMA

EAAAAGAGPPRYLCVVSDGEELDQAVASLKRPGVGVIAVTRTTRVPSATATLRWWAVQGG

EYRLR--RKQLDNSSKAGKARQFYM-IAPK---------LLKPSPPRLHARSARVCVTAQ

MR-----------KYELKPVAAAK-------SVAIIGAGLSGLVAAKSALEAG------M

LPTLFDRRGSIGGMWSTER-----------------------------------------

------SGMYDSLHTNVSRYSCVFSDFP----WPEGA-----------------------

--------------------------------PDFPTPGELGAYLSAYRSTFLPPDKCPA

QVGVEVLRVEPAGSPG----------------------------GDGG------------

---------------GTGAKTGWRVQWR----PLSPDAD-------GSPSP---------

----------------------------STCGHDT---------HDKQEQP-PIQSAEFD

AVIIASGYAAV-PAVP---DIP----GLEAF---AGKVVHSLHY-KNPH------EFA--

-------G---LRVAVIGAGHSALDIASDLALASA-------ACAP--------P-SAAA

EAEAAPAAAVG-------------PAGVVHVFPRPMW-----VTPRYL-PDSSAAA-AAA

ASGTAAAAVAPPPAFLPLDMQFYRRSRRA----------------GPEEVAV--------

---------------------PNAAQRQKGNGFWASMCGDQGALISPL-HA-IAADTT-E

PPVIGICDSYVPLAATGAMHLRCAR----LLRITGPSTLE---LSDGGP-----------

----------------PL-SD-IDALVLATGIK---PDLSYLPH-DV-------------

--LEK------LSYTPDDNYLPVA-AHRGVTHP-DVPGLACVGM-YRGTY-----FGAVE

LQARLAAATLSG-RAPPE-PE--EVVRAGVAAELAIREQTPRPVLPH-------------

------------------GDYVGHTGGMA--AALG---------VLP-------------

--------DAAWRAA-------HDQVVP-----------------GDFGLV------PP-

-------SWEQQEQQAGTQAAMVK------------------------------------

-----DAAAAAEAAMV-------AAV-KEA---------PAGGEQGG------------G

E--------------------PAGHVVVAG----------RPAA-AYAAAAAAVAESHAS

---LARF----------------AGGRMVARAVFYAL-----------------------

--------AGNWRLR---------------------------------------------

-----------------------------RTL----------------------------

-----VSRME--------------------------------------------------

------------------------------------SSPSGVVTGAASFLPKRP------

-------------------------------------------------DADAAA-----

-----------------------------------NGSGS--------------------

--------------------------------------------------SNGSSGGSSS

GGGSSRVRLG--------SMPFQYLY----------------------------------

------------------------------------------------------------

-----REQGQFAIQGSGS------------------------SSGSSSGSGGISSGGAV-

------------------MRVSKEYVYRYDSP----------------------------

---------------------------------SDR----------LDVFFA--------

--------------------------DAHGV--------P-------GGFFHSLRFL---

------------------------------------------------------------

------------------------------------------------------------

------------------------------------------------------------

-------------APAEGAP----------------------------------------

------------------------------------------------------------

------------GPQHFQREE---------------------------------------

------------------------------------------------------------

------------------------------------------------------------

------------------------------------------------------------

------------------------------------------------------------

------EPAADDAADDSGC-----------------------------------------

------------------------------------------------------------

------------------------------------------------------------

--WRA-------------------------------------------------------

-------------------VGEHLCVR--------------------------DMYYSSY

RFWFQGVH----------------------------------------------------

-----------------------------------LKKFQIQ------------------

------------------------------------------------------------

----FVVQGPNKDYTST-----------------ATYTRQ--------------------

-----------------------------

>jgi|Mesvir1_1|12953|Mv17927-RA.1

------------------------ML----------------------------------

------------------------------------------------------------

------------------------------------------------------------

------------------------------------------------------------

------------------------------------------------------------

------------------------------------------------------------

------------------------------------------------------------

------------------------------------------------------------

------------------------------------------------------------

------------------------------------------------------------

------------------------------------------------------------

------------------------------------------------------------

------------------------------------------------------------

------------------------------------------STARL-------------

------------------------------------------------------------

----------------L-------------------------------------------

--SGSVSSP---------------------------------------------------

------------------------------------------------------------

------------------------------------------------------------

------------------------------------------------------------

------------------------------------------------------------

--------------------YATVQ-----------------------------------

-------------------------------------------LTPRQCPTALWESRQQG

KYHLHWQRQVGKNLCPAWASRKFG-----------------AQTSLHLQSRDGPLCSPAT

PRRIVC----RAEAADAVDMAPSK-------SIAVVGAGLSGLVAAKSLLERG------L

LPTVFDAAEKVGGMWNPEAHPG--------------------------------------

------CQRWPSMRTNVSRFTCSFSDLL----WPPGS-----------------------

--------------------------------PTFPSQPEVGRYLVRYAARFLSQPGVTF

SLGSRVTRVAPRPDG---------------------------------------------

---------------------LWDVHWQ----VTDASGN-------R-------------

----------------------------------------------------EQQRAVFA

GVVVASGFFAA-PRLP---PLPPPSRGAATF---QGTLLHANDY-RGATG-VHAAPFA--

-------G---KRVAVVGASLSAVEIAADIAVSGR-------------------------

------------------------AASVTNVVPHHAY-----VLPRLL-PDDTGSA----

-----------APAFLPLDLLLYQRKEPGKGGAQ---LGKGVAGQGGGEEGA--------

-----------------------EEGAEAAHAYFGRILPREEDVLAAL-KH-KEGDVP-P

PPYVGICDHYAGLVRGGAITLLPGR----MVGVAEDGRAL---LVDATPAP---------

-AAANSTTA-TSPPSPPVHLD-VDTVIFCTGFG---PSLDFLQP-APSH--ADKPGEPTA

SAADAVDPLVPLAYDPDDPLLPLLLDADSVFHP-TLHGLAFVGM-YRGVF-----PGVVE

LQARLVADVFAG-AIPHPYANDKGAAAASLSRAQALRDTHPRPQFPF-------------

-------------------KYVEHADVLA--ARIG---------ALP-------------

--------PASWPLA------TNYPVTP-----------------LQYRMAV--------

------------------------------------------------------------

----GDAGAQPRREMAFAGLG--EARTADV---------PFLATQ---------------

----------------------------------------PTRAPSPLSLGEAFRDLEDE

---LAAY----------------RAGKGVARAVFRGL-----------------------

--------GGKWCFR---------------------------------------------

-----------------------------RRL----------------------------

-----VSRLD--------------------------------------------------

------------------------------------ASPSGVVVGEASFKPQPWPLM---

------------------------------AEAWVV-----------EPPAETASG----

-----------------------------------QGDAPVAGQG---------------

------------RAWDVLKPSWSAHEDPVVARE-------QAAMPACAAT-GAGTGGVSG

RGATAANPV------------LDYLY----------------------------------

------------------------------------------------------------

-----QETGVFRTS-----------------------------------------SGLE-

------------------LNVKSSYVYTQEGE----------------------------

---------------------------------GDT----------LELFFAA-------

PPGAQ------ATNRDP----RIVQGDQGPI------MVR-------GRPFLSLR-----

------------------------------------------------------------

------------------------------------------------------------

------------------------------------------------------------

--------------PSR-------------------------------------------

------------------------------------------------------------

------------------------------------------------------------

------------------------------------------------------------

------------------------------------------------------------

------------------------------------------------------------

------------------------------------------------------------

-------------ADPGSG-----------------------------------------

------------------------------------------------------------

------------------------------------------------------------

--WEA-------------------------------------------------------

-------------------DATHLCGA--------------------------DTYVARF

RFAFEGAR----------------------------------------------------

-----------------------------------LREMEIH------------------

------------------------------------------------------------

----FHVSGPHKDYDSV-----------------TLMRRGPVSEL---------------

----------------AFQQGISFADALE

>jgi|Grachil1|4236|Gchil4728.t1

M-----------------------MS----------------------------------

------------------------------------------------------------

------------------------------------------------------------

------------------------------------------------------------

------------------------------------------------------------

------------------------------------------------------------

------------------------------------------------------------

------------------------------------------------------------

------------------------------------------------------------

------------------------------------------------------------

------------------------------------------------------------

------------------------------------------------------------

------------------------------------------------------------

------------------------------------------------------------

------------------------------------------------------------

------------------------------------------------------------

------------------------------------------------------------

------------------------------------------------------------

------------------------------------------------------------

------------------------------------------------------------

------------------------------------------------------------

------------------------------------------------------------

------------------------------------------------------------

------------------------------------------------------------

-------------SDGLLGKPPTT-------RICVVGGGISGICAARACLLSG------L

NVTLFEKRSKLGGIWSLEN-----------------------------------------

------SPVFDSLSSNTATFEAGLSDIPPLRVFPSSSL----------------------

----------------------------GKYDDLCCTSQEMADYIQLIVKKTPR-FFEST

KLKTNVLSVTQTPSK---------------------------------------------

---------------------TYCVSFL----SDGT------------------------

----------------------------------------------------RAQESEFD

KVIISTGYFNT-PFVPPASELP----GIDKY---RGKVLHVSMY-KNPS------QLK--

-------G---KHVLIVGGSVSGCEAAGDMLAFAS-------------------------

-----------S----------EGPTSVTLNTRSMRF-----LLLKQH--KG--------

-------------------EVLLSKLGTRFQ----------------------RFRDLAG

RFENEQYSQTMSSFIPQFLS--------------------NSDYPAPEPDGPIKIPYV--

PSWVPVSGKLYEACQKKKLQWKVSS----LECITE-NGVR---FRDGTEG----------

--------------------E-YDVIVFATGYR---LHLPFLEK-DV-------------

--QQT------ICAGNGVNLLD---LYDYTFHP-EVPNMAFVGM-FSPA---TSIIPIVD

NQSRWVARVFNG-DVQLP-SD-EELNHGVQLYRQTRSSPNS-------------------

---------------RFVVHGFDVLDVFA--KHGDFE-----------------------

--------VNLAKFPQLCRALLFGPMIP-----------------AQFRLFGS-------

------------------------------------------------------------

------------------------------------------------------------

-----------------------EH--------------------SEEALEETMKQFEAA

---GFKI-----------------GDNVVSSEIYEEL-----------------------

------------------------------------------------------------

-----------------------------RTI----------------------------

------------------------------------------------------------

------------------------------------------------------------

------------------------------------------------------------

------------------------------------------------------------

------------------------------------------------------------

------------------------------------------------------------

------------------------------------------------------------

------------------------------------------------------------

------------------------------------------------------------

------------------------------------------------------------

---CHIL-------------------DEKGI------C----------------------

------------------------------------------------------------

------------------------------------------------------------

------------------------------------------------------------

----------PSSLPVA-------------------------------------------

------------------------------------------------------------

------------------------------------------------------------

------------------------------------------------------------

------------------------------------------------------------

------------------------------------------------------------

------------------------------------------------------------

------------------------------------------------------------

------------------------------------------------------------

------------------------------------------------------------

------------------------------------------------------------

------------------------------------------------------------

------------------------------------------------------------

-----------------------------------LGHLVVSKT----------------

------------------------------------------------------------

------------------------------------------------------------

-----------------------------

>Pp6c18_10300|Ppatens_v6.1/1-540

------------------------MS----------------------------------

------------------------------------------------------------

------------------------------------------------------------

------------------------------------------------------------

------------------------------------------------------------

------------------------------------------------------------

------------------------------------------------------------

------------------------------------------------------------

------------------------------------------------------------

------------------------------------------------------------

------------------------------------------------------------

------------------------------------------------------------

------------------------------------------------------------

------------------------------------------------------------

------------------------------------------------------------

------------------------------------------------------------

------------------------------------------------------------

------------------------------------------------------------

------------------------------------------------------------

------------------------------------------------------------

------------------------------------------------------------

------------------------------------------------------------

------------------------------------------------------------

------------------------------------------------------------

------------------NLMTEF-------DAVIVGAGIAGLYQLYRLRNLG------L

RTHVLEAEKEVGGTWYLN------------------------------------------

--------KYPGARCDIKSIMYSYSFSPE---LEQEW-----------------------

-----------------------------NWSENYSNQPEILRYINHVADRFDL--RKDI

TFNTRVTGALYNESSK--------------------------------------------

---------------------RWEVHTD----QGKT------------------------

--------------------------------------------------------IRTQ

FLIMATGCLST-PKQP---EIP----GIEKF---QGSSYYTGRWPDKPV------DFT--

-------G---KRVAVIGTGSSAVQSIPIIAQQ---------------------------

------------------------ASQLTVFQRSAAC-----CVPGLNYKLTADEIAASK

ASYSTLRKAQ-RESPGGFVDERGSKSALEDSEEDRQARYEMLYQRGTLDAIIVCAYNDLV

--INKEANDTFSEFIRQKIR-----SIVKDPVV--------ADSLSPRGYA-------VG

TKRICLDYGYYETFNLDHVHLVDLTK-TPILEMTE-KGIR---TSEKDHV----------

----------------------FDAIVYATGFD---AITGTLTRIGIQ------------

-GKNKL-----LLKDKWAKGPR---TYLGIAIA-EFPNLFTITGPFSPS-VFTIMTVAIE

QHVDWITDCLKY-LREHG---VEEIEATVEAEDAWVEHSLAVANMT--------------

---------------LFPKTASYYTGGNV--EGKPF-------MLYP-----Y-------

-----VGGLANYRQV------------------------CDGVAAKGYEGFKL--K----

------------------------------------------------------------

------------------------------------------------------------

------------------------------------------------------------

------------------------------------------------------------

------------------------------------------------------------

------------------------------------------------------------

------------------------------------------------------------

------------------------------------------------------------

------------------------------------------------------------

------------------------------------------------------------

------------------------------------------------------------

------------------------------------------------------------

------------------------------------------------------------

------------------------------------------------------------

---------------------TKGIV----------------------------------

------------------------------------------------------------

------------------------------------------------------------

------------------------------------------------------------

------------------------------------------------------------

------------------------------------------------------------

------------------------------------------------------------

------------------------------------------------------------

------------------------------------------------------------

------------------------------------------------------------

------------------------------------------------------------

------------------------------------------------------------

------------------------------------------------------------

------------------------------------------------------------

------------------------------------------------------------

------------------------------------------------------------

------------------------------------------------------------

------------------------------------------------------------

------------------------------------------------------------

------------------------------------------------------------

------------------------------------------------------------

------------------------------------------------------------

-----------------------------

>jgi|Cyacal1|1570|CDCA_CDCA15G3951.1

------------------------MG----------------------------------

------------------------------------------------------------

------------------------------------------------------------

------------------------------------------------------------

------------------------------------------------------------

------------------------------------------------------------

------------------------------------------------------------

------------------------------------------------------------

------------------------------------------------------------

------------------------------------------------------------

------------------------------------------------------------

------------------------------------------------------------

------------------------------------------------------------

------------------------------------------------------------

------------------------------------------------------------

------------------------------------------------------------

--TQPAKEA---------------------------------------------------

------------------------------------------------------------

------------------------------------------------------------

------------------------------------------------------------

------------------------------------------------------------

------------------------------------------------------------

------------------------------------------------------------

------------------------------------------------------------

-------------HSASTNGAVEV-------DALIIGAGVAGLYQLYQLRNEG------L

RVLAVDTAADVGGTWYWN------------------------------------------

--------CYPGAKFDSESYVYQYLFDER---LYKDW-----------------------

-----------------------------TWSERYPSQPEIERWMHYVTERLEL--RRDI

RFGTTVTSAVYDEARG--------------------------------------------

---------------------RWMVSTD----RGGT------------------------

--------------------------------------------------------FDTR

FLISCGGMLSA-PLEK---RFP----GQERF---SGRIFHTARWPREPL------DLA--

-------G---KRVGVVGVGATGIQVIQTIASE---------------------------

------------------------VAHLKVFVRTPQY-----VVPMKNPKYGPREEKAYK

ARFDELRDLL-PRTHTGFEYEIKHRWADLTP-EQRRAVLEETYGDGSLRAWL-GGFLEIF

--TEPEVSEQVSEFVREKMR-----ARLKDPKL--------IEKLVPTDYG-------FG

CRRVPLETRFLEVFHRDNVEAVSVRD-NPIVEVVP-EGIR---LDDGTVH----------

--------------------P-LDVIVLATGFD---AGSGALTRIDIR------------

-GRDGR-----SLRDDWSRDIR---TALGMMVH-GYPNMFMTGAPLAPSAALCNMTTCLQ

QQIEWITDCIRY-MRGNG---YTVIEPTASFEEEWVRHHDEMANTT--------------

---------------LIAKTNSWYTGTNV--EGKPR-------RVLS-----Y-------

-----TAGVGTYRQK------------------------CTKVAEGGYQGFQM--V----

------------------------------------------------------------

------------------------------------------------------------

------------------------------------------------------------

------------------------------------------------------------

------------------------------------------------------------

------------------------------------------------------------

------------------------------------------------------------

------------------------------------------------------------

------------------------------------------------------------

------------------------------------------------------------

------------------------------------------------------------

------------------------------------------------------------

------------------------------------------------------------

------------------------------------------------------------

------------------------------------------------------------

------------------------------------------------------------

------------------------------------------------------------

------------------------------------------------------------

------------------------------------------------------------

------------------------------------------------------------

------------------------------------------------------------

------------------------------------------------------------

------------------------------------------------------------

------------------------------------------------------------

------------------------------------------------------------

------------------------------------------------------------

------------------------------------------------------------

------------------------------------------------------------

------------------------------------------------------------

------------------------------------------------------------

------------------------------------------------------------

------------------------------------------------------------

------------------------------------------------------------

------------------------------------------------------------

------------------------------------------------------------

------------------------------------------------------------

-----------------------------

>jgi|CyamerSoos_1_1|3320|CmerSOOS_G3306.1

------------------------MR----------------------------------

------------------------------------------------------------

------------------------------------------------------------

------------------------------------------------------------

------------------------------------------------------------

------------------------------------------------------------

------------------------------------------------------------

------------------------------------------------------------

------------------------------------------------------------

------------------------------------------------------------

------------------------------------------------------------

------------------------------------------------------------

------------------------------------------------------------

------------------------------------------------------------

------------------------------------------------------------

------------------------------------------------------------

--TELAQSP---------------------------------------------------

------------------------------------------------------------

------------------------------------------------------------

------------------------------------------------------------

------------------------------------------------------------

------------------------------------------------------------

------------------------------------------------------------

------------------------------------------------------------

-------------DSVTRNAVIEL-------DALIIGAGVAGLYQLHQLRSLG------L

RAIAVDTAGDIGGTWYWN------------------------------------------

--------CYPGAKFDSECYVYQYLFDKK---LYKDW-----------------------

-----------------------------TWGERFPSQPEIERWMHYVTDRLGL--WHDI

RLGTTITLAVYDDERD--------------------------------------------

---------------------RWMVETN----RGDS------------------------

--------------------------------------------------------FDTR

FLIGCCGMLSA-PLER---RFP----GQDRF---CGRIFHTSRWPREPL------SLA--

-------G---KRVGVVGVGATGIQVIQTIASE---------------------------

------------------------VGHLKVFIRTPQY-----VVPMKNTKYGPEEVHAYK

ERFDELRDLL-PRTFSGFEYEIKHRWAELTP-EQRLAVLEESYGDGSLKAWL-GGFNEIF

--FDPEVSEQVSEFVREKMR-----ARLKDPKL--------IEILIPKDYG-------FG

TRRVPLESGFLEVFHRDNVEAVSVRN-NPIVEVVP-EGIR---LADGTVH----------

--------------------E-LDVIILATGFD---ASSGALTRIDIR------------

-GRGGR-----SLREEWSRDIR---SSLGLMVH-GYPNLFLAGAPLAPSAALCNMTTCLQ

QQTEWITDCIRY-MQANG---FTVIEPTAAFEEAWVRHHDETANAT--------------

---------------LIAKTDSWYVGSNV--DGKPR-------RMLS-----Y-------

-----TGGVGTYRQK------------------------CAEIAKDNYRGFKM--A----

------------------------------------------------------------

------------------------------------------------------------

------------------------------------------------------------

------------------------------------------------------------

------------------------------------------------------------

------------------------------------------------------------

------------------------------------------------------------

------------------------------------------------------------

------------------------------------------------------------

------------------------------------------------------------

------------------------------------------------------------

------------------------------------------------------------

------------------------------------------------------------

------------------------------------------------------------

------------------------------------------------------------

------------------------------------------------------------

------------------------------------------------------------

------------------------------------------------------------

------------------------------------------------------------

------------------------------------------------------------

------------------------------------------------------------

------------------------------------------------------------

------------------------------------------------------------

------------------------------------------------------------

------------------------------------------------------------

------------------------------------------------------------

------------------------------------------------------------

------------------------------------------------------------

------------------------------------------------------------

------------------------------------------------------------

------------------------------------------------------------

------------------------------------------------------------

------------------------------------------------------------

------------------------------------------------------------

------------------------------------------------------------

------------------------------------------------------------

-----------------------------

>jgi|Cyamer1|57806|XM_005536881.1

------------------------MG----------------------------------

------------------------------------------------------------

------------------------------------------------------------

------------------------------------------------------------

------------------------------------------------------------

------------------------------------------------------------

------------------------------------------------------------

------------------------------------------------------------

------------------------------------------------------------

------------------------------------------------------------

------------------------------------------------------------

------------------------------------------------------------

------------------------------------------------------------

------------------------------------------------------------

------------------------------------------------------------

------------------------------------------------------------

--AELNQFT---------------------------------------------------

------------------------------------------------------------

------------------------------------------------------------

------------------------------------------------------------

------------------------------------------------------------

------------------------------------------------------------

------------------------------------------------------------

------------------------------------------------------------

-------------NHVQNKSVVEL-------DALIIGAGVAGLYQLHKLRSAG------L

NALAVDTAEDVGGTWYWN------------------------------------------

--------CYPGAKFDSECYVYQYLFDEK---LYKDW-----------------------

-----------------------------SWSERFPGQPEIERWLHYVTDRLGL--RPYL

RFCTTVTAAEYDEKRG--------------------------------------------

---------------------RWIIKTD----RGQT------------------------

--------------------------------------------------------YDAR

FLIGCCGMLSA-PLEN---RFP----GQERF---RGRIFHTARWPREPI------DLN--

-------G---KRVGVVGVGATGIQVIQTIADK---------------------------

------------------------VGQLKVFVRTPQY-----VVPMKNPKYNPDDVNSYK

KRFEELRELL-PTTASGFEYVIKHRWADLTP-EQRLAVLEETYNDGSLRAWL-GGFDEIF

--TDPQISEEVSEFVREKMR-----ARLKDPKL--------IEILVPTDYG-------FG

SRRVPLESGYLEVYHRDNVEAICVRN-NPIVEVVP-EGIR---LADGTVH----------

--------------------E-LDIIILATGFD---ASSGAFIRIDIR------------

-GRDGR-----SLKEEWSRDIC---SALGLMVH-GYPNMFLTGAPLSPSAALCNMTTCLQ

QQVEWITDCIQY-IQAKG---HNVIEPTASFQEAWVRHHDDLANST--------------

---------------LFSKTNSWYTGTNV--EGKPR-------RMLS-----Y-------

-----TGGVGKYRQK------------------------CAEVAAGGYEGFAV--A----

------------------------------------------------------------

------------------------------------------------------------

------------------------------------------------------------

------------------------------------------------------------

------------------------------------------------------------

------------------------------------------------------------

------------------------------------------------------------

------------------------------------------------------------

------------------------------------------------------------

------------------------------------------------------------

------------------------------------------------------------

------------------------------------------------------------

------------------------------------------------------------

------------------------------------------------------------

------------------------------------------------------------

------------------------------------------------------------

------------------------------------------------------------

------------------------------------------------------------

------------------------------------------------------------

------------------------------------------------------------

------------------------------------------------------------

------------------------------------------------------------

------------------------------------------------------------

------------------------------------------------------------

------------------------------------------------------------

------------------------------------------------------------

------------------------------------------------------------

------------------------------------------------------------

------------------------------------------------------------

------------------------------------------------------------

------------------------------------------------------------

------------------------------------------------------------

------------------------------------------------------------

------------------------------------------------------------

------------------------------------------------------------

------------------------------------------------------------

-----------------------------

>Mvestita_C1185g21950-RA|Marsilea_vestita/1-509

------------------------MHRV--------------------------------

------------------------------------------------------------

------------------------------------------------------------

------------------------------------------------------------

------------------------------------------------------------

------------------------------------------------------------

------------------------------------------------------------

------------------------------------------------------------

------------------------------------------------------------

------------------------------------------------------------

------------------------------------------------------------

------------------------------------------------------------

------------------------------------------------------------

------------------------------------------------------------

------------------------------------------------------------

------------------------------------------------------------

------------------------------------------------------------

------------------------------------------------------------

------------------------------------------------------------

------------------------------------------------------------

------------------------------------------------------------

------------------------------------------------------------

------------------------------------------------------------

------------------------------------------------------------

-------------------------------ETLIVGAGFSGICMGIKLQQAG-----RN

SFLIVEKLGDLGGTWTVN------------------------------------------

--------RYPGCECDVPSHLYSYSFYP----NPDWT-----------------------

--------------------------------TAFASRGEIHNYLQSCAIHYGI-LPDHI

RFHTRATTATWDNDAM--------------------------------------------

---------------------LWRVELV----SNNN------------------------

----------------------------------------------------EPYIVFSR

FLVSAVGMLSV-PTLP---SVP----GIQNF---RGVSVHSHEW-MPSV----DELLS--

-------G---KRVAVVGTGASAVQIVPEVARV---------------------------

------------------------AEKLYVVQRTPAW-----ILPKPG-FCVPKWCREAF

RC-------------LPLLMWLWRVLILCFM---------EFFGSGVYGGAV----FPLL

-----VIKKTIQKVAKHNME-----VHIKDVEL--------RKRLTPT-YH-------LG

CKRLLLSNHFYPALARPNVELISGS----LVEVRE-NNIVVKPLEDGSDE----------

----------------MVEIP-LDTIIYATGFD---VSQPKLHV----------------

VGSNPDSPL-------PGSSSP---SFLGVTKH-GFPNFFTIMGPHSYV-GHTSLLLIIE

AQARYILSCVDL-VLKQN---AQSLEVKLCSEDRFQEFLARRFSRTVWQNG--------G

C------------------ANSWYVDSMT--GQNS--------IIWPGSTIEYI------

-----------WHTS--------------------------SASLDDYT-----------

------------------------------------------------------------

------------------------------------------------------------

------------------------------------------------------------

------------------------------------------------------------

------------------------------------------------------------

------------------------------------------------------------

------------------------------------------------------------

------------------------------------------------------------

------------------------------------------------------------

------------------------------------------------------------

------------------------------------------------------------

------------------------------------------------------------

------------------------------------------------------------

------------------------------------------------------------

-----------------ICYAQENVLM---------------------------------

------------------------------------------------------------

------------------------------------------------------------

------------------------------------------------------------

------------------------------------------------------------

------------------------------------------------------------

------------------------------------------------------------

------------------------------------------------------------

------------------------------------------------------------

------------------------------------------------------------

------------------------------------------------------------

------------------------------------------------------------

------------------------------------------------------------

------------------------------------------------------------

------------------------------------------------------------

------------------------------------------------------------

------------------------------------------------------------

------------------------------------------------------------

------------------------------------------------------------

------------------------------------------------------------

------------------------------------------------------------

------------------------------------------------------------

-----------------------------

>jgi|Chrzof2|4539484|fgenesh1_kg.chr_02_#_14091_#_transcript/62828

------------------------MA----------------------------------

------------------------------------------------------------

------------------------------------------------------------

------------------------------------------------------------

------------------------------------------------------------

------------------------------------------------------------

------------------------------------------------------------

------------------------------------------------------------

------------------------------------------------------------

------------------------------------------------------------

------------------------------------------------------------

------------------------------------------------------------

------------------------------------------------------------

------------------------------------------------------------

------------------------------------------------------------

------------------------------------------------------------

------------------------------------------------------------

------------------------------------------------------------

------------------------------------------------------------

------------------------------------------------------------

------------------------------------------------------------

------------------------------------------------------------

------------------------------------------------------------

------------------------------------------------------------

------------------PNPIGL-------PIAIIGAGFSGLGMGIQLKKAG-----ID

SFTIFEAADDVGGTWRDN------------------------------------------

--------TYPGCACDIKSYLYCYSFEP----NPTWS-----------------------

--------------------------------KPYPSQPEILQYLHNCVDKHQL--KPHI

QLNAAIVEARFDETSG--------------------------------------------

---------------------IWHLSTH----DGRK------------------------

--------------------------------------------------------FQAR

VVIVAKGALTK-PKVP---ALP----GIDTF---QGTILHSARW-DPSI------DLS--

-------G---KRVAVVGTGASSVQMIPAIQPL---------------------------

------------------------VKQLVVFQRSAPW-----VFPRPN---KPFSAQQRM

LA-------------AVFPLFMKARRFLIYL---------QNELIGRAFYAK----PERL

--------RDAEGLAKAHME-----SQVSDPQL--------REKLTPQ-YR-------LG

CKRVCVSDDYYPALCQSNVNLVASP----VVEMRS-GSVV---AADGSEH----------

--------------------E-VDAIVFGTGFD----VARFLEGTAIH-----------G

LGGRELNQQW------REKGAQ---AYYGLAVS-GYPNMFMLLGPNSGL-GHNSVVVMIE

CQVKYVVNCIQQ-LLQRK---LKWLDVKPGCQQKFKDEVDAMMVGTVWQSG---------

-------------------CSSWYLTPEG--KSQT---------LWPRSTWEY-------

-----W-----WRLW--------------------------SVKLSDWQ-----------

------------------------------------------------------------

------------------------------------------------------------

------------------------------------------------------------

------------------------------------------------------------

--------LGRW------------------------------------------------

------------------------------------------------------------

------------------------------------------------------------

------------------------------------------------------------

------------------------------------------------------------

------------------------------------------------------------

------------------------------------------------------------

------------------------------------------------------------

------------------------------------------------------------

------------------------------------------------------------

------------------------------------------------------------

------------------------------------------------------------

------------------------------------------------------------

------------------------------------------------------------

------------------------------------------------------------

------------------------------------------------------------

------------------------------------------------------------

------------------------------------------------------------

------------------------------------------------------------

------------------------------------------------------------

------------------------------------------------------------

------------------------------------------------------------

------------------------------------------------------------

------------------------------------------------------------

------------------------------------------------------------

------------------------------------------------------------

------------------------------------------------------------

------------------------------------------------------------

------------------------------------------------------------

------------------------------------------------------------

------------------------------------------------------------

------------------------------------------------------------

-----------------------------

>jgi|Sceob152z_1|12062|Sobl_g21218.t1

------------------------MA----------------------------------

------------------------------------------------------------

------------------------------------------------------------

------------------------------------------------------------

------------------------------------------------------------

------------------------------------------------------------

------------------------------------------------------------

------------------------------------------------------------

------------------------------------------------------------

------------------------------------------------------------

------------------------------------------------------------

------------------------------------------------------------

------------------------------------------------------------

------------------------------------------------------------

------------------------------------------------------------

------------------------------------------------------------

------------------------------------------------------------

------------------------------------------------------------

------------------------------------------------------------

------------------------------------------------------------

------------------------------------------------------------

------------------------------------------------------------

------------------------------------------------------------

------------------------------------------------------------

-------------AAAAPAAGQEL-------SVAIVGAGFSGLCMAIQLKRAG-----IQ

DFTIFEAADAVGGTWRDN------------------------------------------

--------TFPGCACDIPSVLYCFSFEP----YP--------------------------

------------------------------FTRHYSGQPEIVRYQQHCVTKYGL--APHI

RLSTRVTAAAFDAGSG--------------------------------------------

---------------------TWAVTTA----AGDT------------------------

--------------------------------------------------------VTAR

VLVLARGGLAV-PSVP---SFP----GMDTF---PGAAFHTARW-DHSV------ELT--

-------G---KRVAVVGTGASAIQIVPAIQPH---------------------------

------------------------VASLTLFQRTPPW-----VMPRKA--RLPGWAAPAE

GC-------------SSWYNKVQRRRWFLGM---------ELLLGGALTRNK----QGIL

--------KRVDKVGLSHLQ-----AQVPDHQL--------RAALAPS-YS-------VG

CKRILASDEYYPALCQPNVQVVTSA----VKEAHG-SVII---AEDGTEA----------

--------------------E-VDVIIYATGFD---LTATHHGI-DIT-APAAGAG-VAA

AAAGSHAQQTLNFADVMGDKPR---AFYGLGVA-GFPNLFMINGPNTGL-GHNSMIYMAE

CGVNFIVKLLRR-MRRQR---LKSIQVTAAAQQQFNKELQARLRSSVWLAG--------G

-------------------CGSWYVDRDG--EGSS--------VLWPGLCLEY-------

-----W-----WRTC------------------------VAGPRAGDWAGAKM-------

------------------------------------------------------------

-----DKPVPAAPG----------------------------------------------

---------------------------------------------ASAAAAAAAA-VTAD

---VRR------------------------------------------------------

--------KESWGSE---------------------------------------------

-----------------------------R------------------------------

------------------------------------------------------------

------------------------------------------------------------

------------------------------------------------------------

------------------------------------------------------------

------------------------------------------------------------

------------------------------------------------------------

------------------------------------------------------------

------------------------------------------------------------

--------------------SDKKLL----------------------------------

------------------------------------------------------------

------------------------------------------------------------

------------------------------------------------------------

------------------------------------------------------------

------------------------------------------------------------

------------------------------------------------------------

------------------------------------------------------------

------------------------------------------------------------

------------------------------------------------------------

------------------------------------------------------------

------------------------------------------------------------

------------------------------------------------------------

------------------------------------------------------------

------------------------------------------------------------

------------------------------------------------------------

------------------------------------------------------------

------------------------------------------------------------

------------------------------------------------------------

------------------------------------------------------------

------------------------------------------------------------

------------------------------------------------------------

-----------------------------

>jgi|Sceobl393_2|1990655|estExt_Genemark1.C_8_t10164

------------------------MA----------------------------------

------------------------------------------------------------

------------------------------------------------------------

------------------------------------------------------------

------------------------------------------------------------

------------------------------------------------------------

------------------------------------------------------------

------------------------------------------------------------

------------------------------------------------------------

------------------------------------------------------------

------------------------------------------------------------

------------------------------------------------------------

------------------------------------------------------------

------------------------------------------------------------

------------------------------------------------------------

------------------------------------------------------------

------------------------------------------------------------

------------------------------------------------------------

------------------------------------------------------------

------------------------------------------------------------

------------------------------------------------------------

------------------------------------------------------------

------------------------------------------------------------

------------------------------------------------------------

-------------AAAAPAAGQEL-------SVAIVGAGFSGLCMAIQLKRAG-----IQ

DFTIFEAADAVGGTWRDN------------------------------------------

--------TFPGCACDIPSVLYCFSFEP----YP--------------------------

------------------------------FTRHYSGQPEILRYQQHCVTKYGL--APHI

RLSTRVTAAAFDAGSG--------------------------------------------

---------------------TWAVTTA----AGDT------------------------

--------------------------------------------------------VTAR

VLVLARGGLAV-PSVP---SFP----GMDTF---PGAAFHTARW-DHSV------ELT--

-------D---KRVAVVGTGASAIQIVPAIQPH---------------------------

------------------------VASLTLFQRTPPW-----VMPRKA--RLPGWAAPAE

GC-------------SSWYNKVQRRRWFLGM---------ELLLGGALTRNK----QGIL

--------KRVEKVGLAHLQ-----EQVPDHQL--------RAALAPS-YS-------VG

CKRILASDEYYPALCQPNVQVVTSA----VKEVHG-IVII---AEDGTEA----------

--------------------E-VDVIIYATGFD---LTATHHGI-DIT-APAAGAG-VAA

AAAGSHAQQTLNFADVMGDKPR---AFYGLGVA-GFPNLFMINGPNTGL-GHNSMIYMAE

CGVKFIVKLLRR-MRRQR---LKSIQVTAAAQQQFNKELQARLRSSVWLAG--------G

-------------------CGSWYVDRDG--EGSS--------VLWPGLCLEY-------

-----W-----WRTC------------------------VAGPRAGDWAGAKL-------

------------------------------------------------------------

-----DKPVPAAPG----------------------------------------------

---------------------------------------------ASAAAAAAAA-VTAD

---VRR------------------------------------------------------

--------KESWGSE---------------------------------------------

-----------------------------R------------------------------

------------------------------------------------------------

------------------------------------------------------------

------------------------------------------------------------

------------------------------------------------------------

------------------------------------------------------------

------------------------------------------------------------

------------------------------------------------------------

------------------------------------------------------------

--------------------SDKKLL----------------------------------

------------------------------------------------------------

------------------------------------------------------------

------------------------------------------------------------

------------------------------------------------------------

------------------------------------------------------------

------------------------------------------------------------

------------------------------------------------------------

------------------------------------------------------------

------------------------------------------------------------

------------------------------------------------------------

------------------------------------------------------------

------------------------------------------------------------

------------------------------------------------------------

------------------------------------------------------------

------------------------------------------------------------

------------------------------------------------------------

------------------------------------------------------------

------------------------------------------------------------

------------------------------------------------------------

------------------------------------------------------------

------------------------------------------------------------

-----------------------------

>jgi|Tetrobl72_1|508778|CE508777_788

------------------------M-----------------------------------

------------------------------------------------------------

------------------------------------------------------------

------------------------------------------------------------

------------------------------------------------------------

------------------------------------------------------------

------------------------------------------------------------

------------------------------------------------------------

------------------------------------------------------------

------------------------------------------------------------

------------------------------------------------------------

------------------------------------------------------------

------------------------------------------------------------

------------------------------------------------------------

------------------------------------------------------------

------------------------------------------------------------

------------------------------------------------------------

------------------------------------------------------------

------------------------------------------------------------

------------------------------------------------------------

------------------------------------------------------------

------------------------------------------------------------

------------------------------------------------------------

------------------------------------------------------------

-------------AAAAPAAGQEL-------SVAIVGAGFSGLCMALQLKRAG-----IQ

DFTIFEAADAVGGTWRDN------------------------------------------

--------TFPGCACDIPSVLYCFSFEP----YP--------------------------

------------------------------FTRHYSGQPEILRYQQHCVTKYGL--APHI

RLSTRVTAAAFDAGSG--------------------------------------------

---------------------TWAVTTA----AGDT------------------------

--------------------------------------------------------VTAR

VLVLARGGLAV-PSVP---SFP----GMDTF---PGAAFHTACW-DHSV------ELT--

-------G---KRVAVVGTGASAIQIVPAIQPH---------------------------

------------------------VASLTLFQRTPPW-----VMPRKA--RLPGWAAPAE

GC-------------SSWYNKVQRRRWFLGM---------ELLLGGALTRNK----QGIL

--------KRVEKVGLAHLQ-----AQVPDHQL--------RAALAPS-YS-------VG

CKRILASDEYYPALCQPNVQVVTSA----VKEVHG-IVII---AEDGTEA----------

--------------------E-VDVIIYATGFD---LTATHHGI-DIT-APAAGAG-VAA

AAAGSHAQQTLNFADVMGDKPR---AFYGLGVA-GFPNLFMINGPNTGL-GHNSMIYMAE

CGVNFIVKLLRR-MRRQR---LKSIQVTAAAQQQFNKELQARLRSSVWLAG--------G

-------------------CGSWYVDRDG--EGSS--------VLWPGLCLEY-------

-----W-----WRTC------------------------VAGPRAGDWEAAKM-------

------------------------------------------------------------

-----GESGPAAAG----------------------------------------------

---------------------------------------------AS-AAAAAAA-VTAD

---VRR------------------------------------------------------

--------KESWGSE---------------------------------------------

-----------------------------R------------------------------

------------------------------------------------------------

------------------------------------------------------------

------------------------------------------------------------

------------------------------------------------------------

------------------------------------------------------------

------------------------------------------------------------

------------------------------------------------------------

------------------------------------------------------------

--------------------SDKKLL----------------------------------

------------------------------------------------------------

------------------------------------------------------------

------------------------------------------------------------

------------------------------------------------------------

------------------------------------------------------------

------------------------------------------------------------

------------------------------------------------------------

------------------------------------------------------------

------------------------------------------------------------

------------------------------------------------------------

------------------------------------------------------------

------------------------------------------------------------

------------------------------------------------------------

------------------------------------------------------------

------------------------------------------------------------

------------------------------------------------------------

------------------------------------------------------------

------------------------------------------------------------

------------------------------------------------------------

------------------------------------------------------------

------------------------------------------------------------

-----------------------------

>jgi|Acuobl2326_1|2595238|estExt_Genewise1Plus.C_2_t10261

------------------------MGAA--------------------------------

------------------------------------------------------------

------------------------------------------------------------

------------------------------------------------------------

------------------------------------------------------------

------------------------------------------------------------

------------------------------------------------------------

------------------------------------------------------------

------------------------------------------------------------

------------------------------------------------------------

------------------------------------------------------------

------------------------------------------------------------

------------------------------------------------------------

------------------------------------------------------------

------------------------------------------------------------

------------------------------------------------------------

------------------------------------------------------------

------------------------------------------------------------

------------------------------------------------------------

------------------------------------------------------------

------------------------------------------------------------

------------------------------------------------------------

------------------------------------------------------------

------------------------------------------------------------

-------------APAAAAAPAEL-------SVAIVGAGFSGLCMAIQLKRAG-----IH

DFTIFEAADAVGGTWQQN------------------------------------------

--------TYPGCACDIPSVLYCFSFEP----YP--------------------------

------------------------------FTRHYSGQPEILRYQQHCVNKYGL--APHI

RLSTRVTAAAFDSASG--------------------------------------------

---------------------TWAVTTA----AGDI------------------------

--------------------------------------------------------VTAR

VLVLARGGLAV-PSVP---SFP----GMQAF---PGPSFHTARW-DHSV------DLK--

-------S---KRVAVVGTGASAIQLVPAIQPR---------------------------

------------------------VALLTLFQRTPPW-----VMPRKA--RLPGWAAPAE

EC-------------SSWYNKVQRRRWFLGM---------ELLLGGALTRNK----QGIL

--------KRVEKVGLSHLQ-----AQVPDPQL--------RAALAPS-YS-------VG

CKRILASDEYYPALCQPNVQVVTSG----VKEVRG-SVII---AHDGSAA----------

--------------------E-VDVIIYATGFD---LTATHHGI-DITVAPAAAA---AA

AGTSPDAPQTLSFSEVMGDKPR---AFYGIAVA-GFPNLLMVNGPNTGL-GHNSMIYMAE

CGVNSIVKLLRR-MRRQR---LKSIQVTAAAQEQFNKELQARLARSVWLAG--------G

-------------------CGSWYVNKDG--EGSS--------VLWPGLCLEY-------

-----W-----WRTC------------------------VAGPRAGDWVGVKI-------

------------------------------------------------------------

-----NKQAPAAASG---------------------------------------------

---------------------------------------------ATAAAAAAAAEVATD

---VRR------------------------------------------------------

--------KESWGSE---------------------------------------------

-----------------------------R------------------------------

------------------------------------------------------------

------------------------------------------------------------

------------------------------------------------------------

------------------------------------------------------------

------------------------------------------------------------

------------------------------------------------------------

------------------------------------------------------------

------------------------------------------------------------

--------------------SDKKLL----------------------------------

------------------------------------------------------------

------------------------------------------------------------

------------------------------------------------------------

------------------------------------------------------------

------------------------------------------------------------

------------------------------------------------------------

------------------------------------------------------------

------------------------------------------------------------

------------------------------------------------------------

------------------------------------------------------------

------------------------------------------------------------

------------------------------------------------------------

------------------------------------------------------------

------------------------------------------------------------

------------------------------------------------------------

------------------------------------------------------------

------------------------------------------------------------

------------------------------------------------------------

------------------------------------------------------------

------------------------------------------------------------

------------------------------------------------------------

-----------------------------
